# Dynamic Characterization of Structural, Molecular, and Electrophysiological Phenotypes of Human-Induced Pluripotent Stem Cell-Derived Cerebral Organoids, and Comparison with Fetal and Adult Gene Profiles

**DOI:** 10.3390/cells9051301

**Published:** 2020-05-23

**Authors:** Sarah Logan, Thiago Arzua, Yasheng Yan, Congshan Jiang, Xiaojie Liu, Lai-Kang Yu, Qing-Song Liu, Xiaowen Bai

**Affiliations:** 1 Department of Cell Biology, Neurobiology & Anatomy, Medical College of Wisconsin, Milwaukee, WI 53226, USA; sarahlogan@mcw.edu (S.L.); tarzua@mcw.edu (T.A.); yashengyan@mcw.edu (Y.Y.); 2 Department of Physiology, Medical College of Wisconsin, Milwaukee, WI 53226, USA; 3 Department of Anesthesiology, Medical College of Wisconsin, Milwaukee, WI 53226, USA; jiangcongshan@xjtu.edu.cn; 4 Department of Pharmacology and Toxicology, Medical College of Wisconsin, Milwaukee, WI 53226, USA; xiaojieliu@mcw.edu (X.L.); yulaikang@126.com (L.-K.Y.); qsliu@mcw.edu (Q.-S.L.)

**Keywords:** cerebral organoids, stem cells, neurodevelopment, differentiation

## Abstract

Background: The development of 3D cerebral organoid technology using human-induced pluripotent stem cells (iPSCs) provides a promising platform to study how brain diseases are appropriately modeled and treated. So far, understanding of the characteristics of organoids is still in its infancy. The current study profiled, for the first time, the electrophysiological properties of organoids at molecular and cellular levels and dissected the potential age equivalency of 2-month-old organoids to human ones by a comparison of gene expression profiles among cerebral organoids, human fetal and adult brains. Results: Cerebral organoids exhibit heterogeneous gene and protein markers of various brain cells, such as neurons, astrocytes, and vascular cells (endothelial cells and smooth muscle cells) at 2 months, and increases in neural, glial, vascular, and channel-related gene expression over a 2-month differentiation course. Two-month organoids exhibited action potentials, multiple channel activities, and functional electrophysiological responses to the anesthetic agent propofol. A bioinformatics analysis of 20,723 gene expression profiles showed the similar distance of gene profiles in cerebral organoids to fetal and adult brain tissues. The subsequent Ingenuity Pathway Analysis (IPA) of select canonical pathways related to neural development, network formation, and electrophysiological signaling, revealed that only calcium signaling, cyclic adenosine monophosphate (cAMP) response element-binding protein (CREB) signaling in neurons, glutamate receptor signaling, and synaptogenesis signaling were predicted to be downregulated in cerebral organoids relative to fetal samples. Nearly all cerebral organoid and fetal pathway phenotypes were predicted to be downregulated compared with adult tissue. Conclusions: This novel study highlights dynamic development, cellular heterogeneity and electrophysiological activity. In particular, for the first time, electrophysiological drug response recapitulates what occurs in vivo, and neural characteristics are predicted to be highly similar to the human brain, further supporting the promising application of the cerebral organoid system for the modeling of the human brain in health and disease. Additionally, the studies from these characterizations of cerebral organoids in multiple levels and the findings from gene comparisons between cerebral organoids and humans (fetuses and adults) help us better understand this cerebral organoid-based cutting-edge platform and its wide uses in modeling human brain in terms of health and disease, development, and testing drug efficacy and toxicity.

## 1. Introduction 

A host of diseases affect the human brain, including congenital, traumatic, and neurodegenerative conditions. Clinical approaches to garner information on such conditions mostly revolve around imaging, biomarkers, and cognitive assessment, which are often of poor resolution and only are possible after significant disease progression [1,2]. The complexity of the human brain has previously been difficult to study in vitro due to its relative inaccessibility. It has proved extremely challenging to translate whole-body and mechanistic cellular/molecular findings from in vivo and in vitro animal studies, respectively, to the human brain, highlighting a crucial need for a human model which can be utilized to investigate the brain. The advent of induced pluripotent stem cell (iPSC) technology to obtain human neural cells was pivotal in modelling human neurological disease conditions [3]. 

Two-dimensional (2D) monolayer iPSC-derived neural cell models have been widespread for studying neurodegenerative disorders (e.g., Alzheimer’s disease), pharmaceutical testing, and personalized medicine [4,5,6]. However, there are many weaknesses surrounding the use of 2D models to study complex diseases and their progression [7]. Homogenous neuron cultures isolated from other supporting cells cannot function in a manner that would resemble the in vivo brain [8,9,10]. In addition, neurons are highly metabolic and electrochemically active, which is a determinant of their elaborate network connection and interaction with other supporting cells (e.g., astrocytes). Homogenous populations of different neural subtypes cultured in a monolayer format greatly limit the ability to understand complex brain functions and interaction, and confound studies on signaling pathways and brain activity [11,12]. Electrophysiological function has also been a limitation in previous in vitro neuron models, due to reports that action potential generation and neurotransmission have been limited within these cultured neurons [9]. This greatly inhibits the ability of cell culture models to be employed in physiological studies.

To combat these limitations regarding homogeneity and functionality, Lancaster et al. were the first to publish a detailed protocol of how to use iPSCs to generate three-dimensional (3D) cerebral organoids [13]. These cerebral organoids display multiple brain region-like areas that are organized tissues, and have already been used to study various neurological conditions [7]. So far, the cerebral organoid model has most commonly been used to study developing brain disorders over time, such as microcephaly and early Zika virus infection [14,15], highlighting the dynamic properties of cerebral organoids relative to static 2D culture. However, the cerebral organoid field is currently in its infancy. Due to the novelty of the cerebral organoid field, the potential of cerebral organoids to be used in signaling and neurophysiological studies has also not yet been recognized. A gap remains in understanding its cellular components and electrophysiological activity prior to making decisions about the best manner in which to employ cerebral organoids in physiological and pathological studies. Specifically, without neurotransmitter signaling and electrical potential such as different ion channel activities and the response to ion channel-related drugs, it is impossible to accurately represent neuron function, drug action, and cellular injury. Additionally, the exact age at which cerebral organoids correlate with the in vivo human brain also remains unclear [14,16]. 

Thus, the purpose of the current study was to expand upon the previous development of the iPSC-derived cerebral organoid model by the characterization of electrophysiological properties at both molecular and cellular levels, and dynamic development throughout the 2-month differentiation process, dissecting the approximate age of cerebral organoids relative to the fetal and adult human brains by a comparison of genome-wide expression profiles among organoids, fetal and human brain tissues. This is the first time in the field that the dynamic emergence of spontaneous electrical activity, electrochemical responses, drug action, heterogeneous tissue components, and comparisons of gene profiles among organoids, fetal and human brains have been reported. In addition, pathway analyses of gene expression profiles shed light on the equivalency between cerebral organoids and the fetal brain versus the adult brain. 

## 2. Methods

### 2.1. iPSC Culture and 3D Cerebral Organoid Generation from iPSCs

All experiments utilizing human iPSCs were approved by the Medical College of Wisconsin Institutional Review Board. The detailed iPSC culture procedure and medium were described previously [17,18]. Briefly, iPSCs were maintained on matrigel (Corning Inc, Corning, NY, USA)-coated plates in mTESR1 medium (STEMCELL Technology) under hypoxic conditions (4% O_2_) at 37 °C. Upon reaching 80% confluency, iPSCs were passaged with Versene (Thermo Fisher Scientific, Waltham, MA, USA).

The protocol we used for generation of cerebral organoids was developed by Lancaster et al. [13]. During the iPSC passaging process, singularized cells were re-suspended in mTeSR1 medium. The singularized 12,000 iPSCs in mTeSR1 media were added (150 μL cell suspension) to each well of the 96-well ultra-low attachment plate. During the differentiation process, the media were changed every other day, and cells were maintained in normoxic conditions (21% O_2_) at 37 °C. Briefly, embryoid bodies formed in the ultra-low attachment plates for 6 days, and mTeSR1 was added every other day. On day 6, embryoid bodies were transferred to 24-well low attachment plates, and cells were cultured in neuroepithelial media Dulbecco’s Modified Eagle Medium (DMEM/F12, 1% N2 Supplement, 1% glutamine, 1% nonessential amino acids (Life Technologies, Carlsbad, CA, USA) and 1 μg/mL Heparin 50 (Sigma-Aldrich, St. Louis, MO, USA) for 5 days. On day 11, neuroepithelial tissues were embedded in matrigel droplets, and plated on 100 mm plates in 12 mL cerebral organoid differentiation media (DMEM/F12, Neurobasal media, 0.5% N2 Supplement, 1% Glutamine, 0.5% nonessential amino acids, 1% penicillin/streptomycin, 1% B27 without vitamin A (Thermo Fisher Scientific), 0.025% Insulin (Sigma), and 0.035% mercaptoethanol (Sigma) for 5 days. Plates were transferred to a spinner on day 16, and cultured long-term in 12 mL cerebral organoid differentiation media supplemented with vitamin A (DMEM/F12, Neurobasal media, 0.5% N2 Supplement, 1% glutamine, 0.5% nonessential amino acids, 1% penicillin/streptomycin, 1% B27 with vitamin A (Thermo Fisher Scientific) 0.025% Insulin, and 0.035% mercaptoethanol (Sigma). Cerebral organoids were cultured up until 2 months, with a subset of iPSCs and 1-month-old cerebral organoids collected for imaging and gene expression studies (see below). iPSC colonies, embryoid bodies, neuroepithelial tissues, and cerebral organoids were imaged under bright field by an EVOS FL Auto microscope (Thermo Fisher Scientific).All experiments below were from the 0-, 1-, or/and 2-month-old organoids. 

### 2.2. Immunostaining Analysis of iPSC, Neural Progenitor Cells, Brain Cell-Specific Marker Expression

For iPSC colony staining, iPSCs plated on glass coverslips were fixed in 4% paraformaldehyde, washed with phosphate buffered saline (PBS) (Life Technologies), 10% donkey serum was used as a blocker. Anti-rabbit octamer binding transcription factor 4 (OCT4; a pluripotent stem cell marker, Abcam, Cambrige, UK; ab18976) and anti-mouse microtubule-associated protein 2 (MAP2; a neuron-specific marker, Abcam, ab11267) primary antibodies were used, with the Alexa Fluor 594 (Thermo Fisher Scientific) secondary antibody. Hoechst 33,342 (Thermo Fisher Scientific) was used to stain cell nuclei. The coverslips were placed on glass slides with mounting medium (Thermo Fisher Scientific) and sealed with nail polish at the conclusion of immunostaining. Samples were imaged by laser scanning confocal microscopy (Nikon Eclipse TE2000-U, Nikon, Minato City, Tokyo, Japan).

Cerebral organoids were fixed in 10% zinc formalin (Sigma), embedded in paraffin, and slices of 4 µm thickness were attached to glass slides. Tissues were deparaffinized by sequential washing in xylenes, hydrated in graded ethanol, and incubated in antigen retrieval solution (Dako, Glostrow, Denmark) at 95 °C. Following PBS washing, 10% donkey serum was used as a blocker, and the following primary antibodies were used: Paired box 6 (PAX6; a neural stem cell marker; rabbit anti, Biolegend 901301), MAP2 (a neuron-specific marker; mouse anti, Abcam, ab11267), S100 calcium binding protein B (S100B; an astrocyte marker; rabbit anti, Abcam, ab52642), synapsin I (a synapse marker; rabbit anti, Cell Signaling Technology, Danvers, MA, USA; 5297), smooth muscle actin (SMA; a smooth muscle cell marker; rabbit anti, Innovexbio, Richmond, CA, USA; mab343c), and cluster of differentiation 31 (CD31; an endothelial cell marker; rabbit anti, Novus Biologicals, Littleton, CO, USA; Neb2284). The corresponding secondary antibody for all anti-rabbit primary antibodies was Alexa Fluor-488 nanometer conjugated immunoglobulin G (IgG), and Alexa Fluor–594 (Thermo Fisher Scientific) conjugated IgG for all anti-mouse antibodies. Hoechst 33,342 (Thermo Fisher Scientificwas used to stain cell nuclei. Coverslips and mounting medium (Thermo Fisher Scientific) were placed over the tissues and sealed with nail polish at the conclusion of immunostaining. Slides were imaged by laser scanning confocal microscopy (Nikon Eclipse TE2000-U, Nikon).

### 2.3. Electron Microscopy Analysis of Synapse Structure

Cerebral organoids were fixed with 2% glutaraldehyde at 4 °C in 0.1 M sodium cacodylate buffer, and post-fixed for 1 h with 1% osmium tetroxide on ice. Cerebral organoids were rinsed with water and dehydrated by acetonitrile and graded methanol. Cerebral organoids were then embedded in epoxy resin (Electron Microscopy Sciences) and polymerized overnight at 70 °C. Sections 60 nm in size were cut and stained with lead citrate and uranyl acetate. A H600 Electron Microscope (Hitachi) was used for imaging.

### 2.4. Ribonucleic Acid (RNA) Extraction

The isolation of total RNA was performed using the phenol–chloroform method described previously [7,19]. Briefly, cerebral organoids were collected in QIAzol lysis reagent (Qiagen, Hilden, Germany), chloroform (Sigma) was added to the samples. Following centrifugation, supernatants were transferred to a new tube, mixed with isopropanol, and centrifuged for pellet isolation. After washing with 75% ethanol, pellets were dissolved in RNase free water. The DNA-free kit DNAse treatment (Thermo Fisher Scientific) was used for DNA elimination based on the manufacturer’s instructions. The quantity and purity of RNA were validated by Nanodrop spectrophotometry (Thermo Fisher Scientific).

### 2.5. Complementary Deoxyribonucleic Acid (cDNA) Preparation

The RevertAid First Strand cDNA synthesis kit (Thermo Fisher Scientific) was used for cDNA synthesis following the manufacturer’s instructions. Briefly, 2 µg template RNA was mixed with water and the primer. Following incubation at 65 °C for 5 min, 5× Reaction Buffer, Ribolock RNase Inhibitor, deoxynucleoside triphosphate (dNTP) Mix, and Revertaid were added to the RNA mixture with the total volume of 20 µL following the manufacturer’s instructions. cDNA synthesis included 5 min at 25 °C, 60 min at 42 °C, and 5 min at 70 °C.

### 2.6. qRT-PCR

A total of 5 ng cDNA, Power up SYBR Green Master Mix (Thermo Fisher Scientific), primers, and water (Qiagen) were loaded in triplicate in 384-well plates (10 μL/well) and PCR was performed using QuantStudio™ 6 Real-Time PCR detection system (Thermo Fisher Scientific). Primers were designed for MAP2, OCT4, S100B, SCN1A, SCN2A, SCN3A, SCN8A, SCN9A (SCN gene family members encode for respective Nav 1 family of sodium channels) [20,21], and SMA, with glyceraldehyde 3-phosphate dehydrogenase (GAPDH) as the housekeeping gene (Table 1). The cycling conditions were 10 min at 95 °C, 40 cycles of denaturation (15 s at 95 °C) and a combined annealing/extension step (30 s at 60 °C). The mean cycle threshold (Ct) values of triplicates within each sample were detected and the expression data were normalized to the GAPDH housekeeping gene. Melting curves were monitored to validate the purity of the PCR product in each well. The 1-month and 2-month-old cerebral organoid gene abundance is expressed as a% change from iPSCs.

### 2.7. Electrophysiology Analysis of Action Potential and Different Channel Activities

Cerebral organoids were embedded in 3% low-melting-point agarose (Thermo Fisher Scientific) and slices were cut (300 μm-thick) using a vibrating slicer (Leica VT1200s, Wetzlar, Hesse, Germany). Slices were prepared in a choline-based solution containing (in mM): 110 choline chloride, 2.5 KCl, 1.25 NaH_2_PO4, 0.5 CaCl_2_, 7 MgSO_4_, 23 NaHCO_3_, 25 glucose, 11.6 sodium ascorbate, and 3.1 sodium pyruvate. Then, slices were allowed to recover for at least 1 h in the artificial cerebrospinal fluid (ACSF) containing (in mM): 119 NaCl, 2.5 KCl, 2 CaCl_2_, 1.3 MgCl_2_, 1.25 NaH_2_PO_4_, 23 NaHCO_3_, and 10 glucose. All solutions were saturated with 95% O_2_ and 5% CO_2_. 

Whole-cell patch clamp recordings were made using patch clamp amplifiers (Multiclamp 700B). Data were acquired with DigiData 1440A digitizers and analysis software pClamp 10.6 (Molecular Devices, San Jos, CA, USA). Cells were visualized under infrared differential interference contrast optics (Nikon Eclipse FN1 and Olympus BX51WI) and a 40× water immersion lens. Series resistance (10–20 MΩ) was monitored throughout the recordings and data were discarded if the resistance changed by more than 20%. Signals were filtered at 2 kHz and sampled at 10 kHz. For recording Na^+^ and K^+^ currents and action potentials, glass pipettes (3–5 MΩ) were filled with the internal solution containing the following (in mM): 140 K-gluconate, 5 KCl, 10 HEPES, 0.2 ethylene glycol-bis (β-aminoethyl ether)-*N*,*N*,*N*′,*N*′-tetraacetic acid (EGTA), 2 MgCl_2_, 4 Mg-ATP, 0.3 Na_2_GTP and 10 Na_2_-phosphocreatine (pH 7.3 with KOH). For recording Na^+^ and K^+^ currents, 500 ms-long steps (10 mV) from a voltage of −60 mV to +50 mV were given with the voltage clamp. For current clamp recordings, 500 ms-long steps (20 pA steps, from −60 pA to +120 pA) were applied.

For recordings of glutamate currents, cells were voltage-clamped at −70 mV; the internal solution contained K^+^-based solution as described above. For recordings of *N*-methyl-d-aspartate (NMDA) currents, the internal solution contained Cs+-based solution (in mM): 100 Cs-methanesulfonate, 10 CsCl, 10 HEPES, 1.1 EGTA, 2 MgCl_2_, 2.5 Mg-ATP, 0.3 Na_2_GTP and 10 Na_2_-phosphocreatine (pH 7.3 with CsOH) were used. Cells were voltage-clamped at +40 mV, and glycine (1 µM) was present in the ACSF. Patch pipettes were filled with ACSF containing glutamate (0.5 mM) or *N*-methyl-d-aspartate (NMDA; 0.5 mM) and were positioned near recorded cells (20 µm). Glutamate or NMDA was applied by a brief pressure (5 psi, 1 s). For recordings of gamma aminobutyric acid (GABA) or propofol currents, the internal solution contained (in mM): 100 K-gluconate, 50 KCl, 10 HEPES, 1 EGTA, 0.1 MgCl_2_, 4 Mg-ATP, 0.3 Na_2_GTP and 10 Na_2_-phosphocreatine (pH 7.3 with KOH) were used. Gamma-aminobutyric acid (GABA; 0.5 mM) or propofol (10 µM) was applied by a pressure ejection similar to the glutamate or NMDA pressure injection. For studies of the effects of propofol (10 μM) on the amplitude and the decay time of GABA currents, GABA currents were evoked and recorded before and after the bath and the application of propofol. 

### 2.8. mRNA Expression Profiling

Microarray and IPA analyses were performed in a similar manner to previous publications [7,19]. The microarray assay (Arraystar Human LncRNA Microarray V4.0) and data analysis services were provided by Arraystar Inc. (Rockville, MD) to assess and compare the gene expression of 20,723 mRNAs across iPSC-derived cerebral organoids following 2-month differentiation (designated as cerebral organoids 1, 2, and 3), fetal brain tissue, and adult brain tissue. Fetal brain tissue (*n* = 3) was obtained from Cell Applications (1F01-50; two separate lots from different human fetal brains aged 21 weeks: designated as fetal 1 and 2) and Takarabio (636526; pooled from 59 fetal/20–33 weeks: designated as Fetal 3). Adult human brain tissue (*n* = 3) was obtained from Biochain (R1234035-50; from a 29-year old donor: designated as Adult 1) and Takarabio (636530; two separate lots pooled from four donors/21–29 years old and five donors/21–66 years, respectively: designated as adults 2 and 3). Before performing the microarray assay, the RNA samples underwent quality control analysis for RNA integrity, quantity, purity, and genomic DNA contamination. The RNA was reverse transcribed to cDNA, from which the Cy-3 labeled cRNA was synthesized. The cRNA was hybridized to microarray probes for fluorescence intensity scanning. The *p*-values were calculated using an unpaired t-test. The fold change was calculated by comparing the normalized intensities between two conditions. Differentially expressed mRNAs were designated by expressing above ± 2.0 fold change and *p* < 0.05 between cerebral organoid, fetal, and adult brain samples, and were shown in volcano plots. Volcano plots are useful tools for visualizing differential expression between two different conditions. They are constructed using fold change values and *p*-values, and thus allow us to visualize the relationship between fold change (magnitude of change) and statistical significance. The volcano plot is constructed by plotting the negative log10 of the *p* value on the y axis. The x axis is the log2 of the fold change between the two conditions. The red data points denote significantly upregulated expression and the green points denote downregulated genes.

The heatmap shows the entire gene profile for all samples. The heatmap was generated in R software. The log2-transformed fragments per kilobase of exon model per million reads mapped (FPKM) gene expression values were hierarchically bi-clustered for the gene expression and the samples using the Euclidean distance metric and the average linkage method. The closeness of the samples was displayed on the top dendrogram. The samples were clustered together, unsupervised within the organoid, fetal brain, and adult brain groups. The color key on the top left represents the log2 (FPKM) value. 

Principal component analysis (PCA) was performed to determine the relative expressional distances between cerebral organoids, fetal, and adult brains in 3D coordinate space. The original log2-transformed normalized intensities were used for PCA in R. The data points on the PCA plot represent the samples, such that the expressional distances between them were maximized for visualization on the 3D plots. The Euclidean distance *d* between any two dots in 3D can be calculated using the following formula:

(1) $$d=\sqrt{\Delta \mathrm{PC}1^{2}+\;\Delta \mathrm{PC}2^{2}+\;\Delta \mathrm{PC}3^{2}}$$

where ΔPC1, ΔPC2, and ΔPC3, are the differences in the principal components 1, 2, 3 between the two data points. Heatmaps, volcano plots, and PCA analysis represent the expression of all 20,723 genes probed for by the microarray. The PCA elbow plot and PCA analysis are available in Supplementary Figure S1 and Supplementary Tables S1 and S2. The gene expression datasets generated during the current study are available on the NCBI data base, with the GEO Submission Number GSE134363.

### 2.9. Ingenuity Pathway Analysis (IPA) Bioinformatic Analysis of mRNA Expression Profiles

To propose and compare/contrast phenotypes and pathways relevant to neurodevelopment, maturation, and neuro-electrophysiology between cerebral organoids, fetal brain tissue, and adult brain tissue, mRNA abundance profiles from each group underwent bioinformatics assessment using IPA software (Qiagen Bioinformatics). IPA generates a causal network analysis based on previously reported upstream regulators, downstream effects, and a respective gene’s involvement in established pathways based on the literature and databases. The analysis provides gene clusters in disease mechanisms, toxicity functions, and canonical signaling pathways Using IPA predictions, a proposed phenotypic overlap between groups was reported. From the current dataset of raw gene abundance values for 20,723 mRNAs across the three groups, only mRNAs with a ± 2.0-fold change difference (*p* < 0.05) between groups were inputted into the IPA software. To more closely focus on signaling pathways related to functional neural networks, canonical pathways were screened based on statistically significant z scores (*p* < 0.05) generated by IPA, and phenotypic relevance was determined by literature searches. 

### 2.10. Statistics

All experiments were performed on samples from independent organoid differentiations. All data are presented as mean ± standard error of the mean (SEM). For qRT-PCR data comparing iPSC, 1-month-old cerebral organoid, and 2-month-old cerebral organoid groups, a one-way ANOVA was used to detect significant differences between groups, with a Holm–Sidak post-hoc test when applicable. Student’s unpaired *t*-test was used to compare GABAergic current with and without propofol treatment. *p* < 0.05 was considered statistically significant for all tests.

## 3. Results

### 3.1. iPSCs Are Pluripotent and Differentiate into Cerebral Organoids

**Figure 1:**
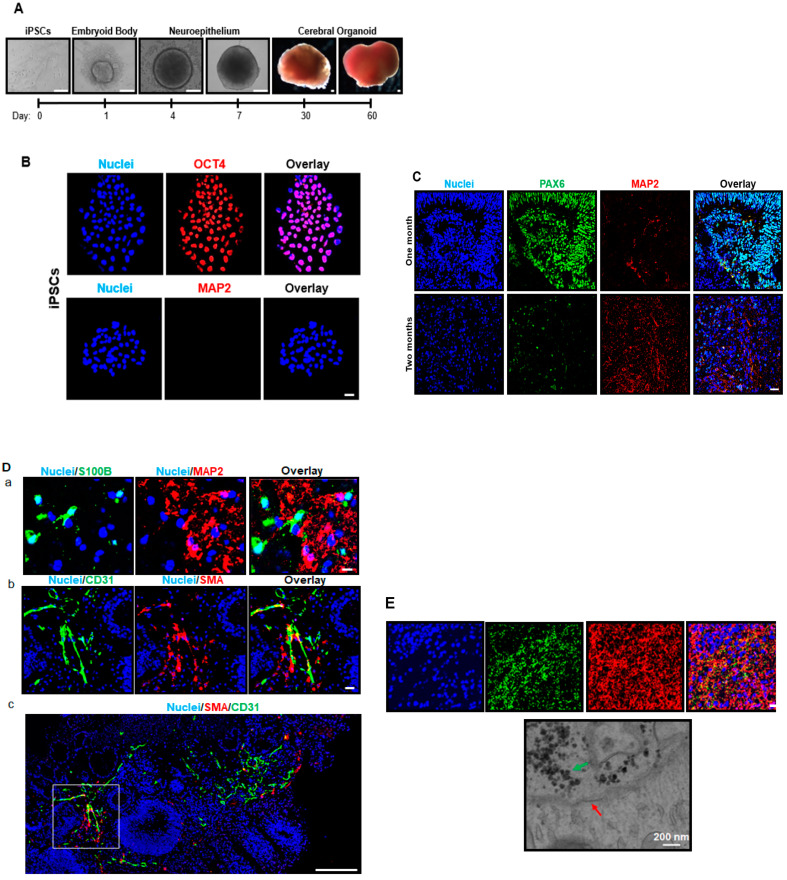
Human-induced pluripotent stem cells (iPSCs) retain pluripotency, but do not show markers of neural differentiation. iPSCs differentiate into cerebral organoids over time. (**A**) The schematic depicts the process of cerebral organoid generation from iPSCs. By use of chemically defined medium and plating strategies, iPSCs are differentiated into cerebral organoids. Singularized iPSCs aggregate into embryoid bodies in ultra-low attachment plates, begin to differentiate into neuroepithelial tissue, and result in formation of cerebral organoids following matrigel embedding. Scale bar = 200 μm. (**B**) Immunofluorescent staining marks the presence of octamer-binding transcription factor 4 (OCT4 (red)) as an indicator of pluripotency, but the microtubule-associated protein 2 (MAP2) (a neuron-specific marker) is not detected in iPSC colonies. Nuclei are stained in blue. Scale bar = 20 µm. (**C**) Immunofluorescent staining marks the expression of the paired box protein 6 (PAX6) in neural epithelial progenitor cells (green) in 1-month-old cerebral organoids, which is markedly reduced in 2-month-old cerebral organoids. MAP2-positive neurons are expressed in 1-month-old cerebral organoids, but more staining appears at 2 months. Nuclei are shown in blue. Scale bar = 10 µm. (**D**) Cerebral organoids exhibit protein markers of various brain cells, such as neurons, astrocytes, and vascular cells (endothelial cells and smooth muscle cells). Immunostaining shows S100 calcium binding protein B (S100B; an astrocyte marker) and MAP2 (a neuron marker)-positive cells (green and red, respectively) in 2-month-old organoids (**a**). Cerebral organoids stain positive for blood vessel-related endothelial and smooth muscle cell markers, CD31 (green) and smooth muscle actin (SMA) (red) (**b**), respectively. Nuclei are stained in blue. Image b shows a magnified view of the boxed area in image (**c**). Scale bar = 50 μm for image b and 200 μm for image (**c**). (**E**) Formation of organized synapse structure in cerebral organoids. In 2-month-old organoids, immunofluorescent staining for pre-synapse marker synapsin I (green; distributed in a punctuated pattern) shows a vast number of synapses between MAP2-positive cells. Nuclei are stained in blue. Scale bar = 50 μm. An individual synapse is shown by an image from electron microscopy. The green and red arrows point to pre-synaptic vesicles and post-synaptic density, respectively. Scale bar = 200 nm.

Over the 2-month differentiation process, singularized iPSCs transitioned to embryoid bodies, to neuroepithelial tissue, then to cerebral organoids. This transition was accompanied by an increase in tissue size (Figure 1A). Normal culture conditions allowed for the formation of iPSC colonies stained positive for OCT4 as an indicator of pluripotency. Prior to differentiation, iPSCs showed no presence of MAP2-positive neurons (Figure 1B). 

At 1 month, immunofluorescence staining images indicate that cerebral organoids were primarily composed of PAX6-positive neuroepithelial cells, relative to MAP2-positive neurons. There was a qualitative increase in neurons at two months, and a decrease in the neural stem cell signal (Figure 1C). Due to the study’s interest in profiling electrophysiologically functional characteristics of cerebral organoids, we chose to focus on cerebral organoids at the 2-month time point, where neurons were the most prominent neural subtype. Additionally, cerebral organoid tissue begins to degenerate after 2 months, so we chose to study the tissue at a time at which differentiated neurons were present in large numbers, before tissue quality was compromised.

### 3.2. Cerebral Organoids Exhibit Heterogeneous Gene and Protein Expression of the Markers for Different Neural Cell Types, Blood Vessel-Related Smooth Muscle Cells and Endothelial Cells, and Synapses

S100B (a marker of astrocytes) was expressed in 2-month-old cerebral organoids throughout the tissue. Like the developing brain, most cells were neurons and astrocytes and comprised roughly 8% of the heterogeneous neural tissue within the cerebral organoids. Neurons remained the most prominent cell type in the continuous cerebral organoid tissue. CD31- and SMA-positive blood vessel-like structures were located more sporadically throughout the tissue in 2-month-old cerebral organoids (Figure 1D). These vessel-like structures were most often located at the periphery of the cerebral organoid tissue, and occupied less than 3% of tissue. Immunostaining images show that in 2-month-old cerebral organoids, various synapses, indicated by punctuated synapsin I signals, are visible between the networks of MAP2-positive neurons. Synapsin I is only present in neurons, and highlights the elaborate network formation and tissue structure. Appropriately, not every cell was synapsin I-positive, highlighting the heterogeneous network within the cerebral organoids. Electron microscopy images of an individual synapse show a closer look at the pre-synaptic cell (green arrow), containing vesicles filled with neurotransmitters. The post-synaptic density (red arrow) is visible on the other side of the synaptic cleft (Figure 1E). The vesicles on the post-synaptic cell are indicative of a chemical synapse, and the space between adjacent cells allows for communication via neurotransmitter with the post-synaptic cell. The organelles of both the pre- and post-synaptic neurons are visible. 

### 3.3. Cerebral Organoids Dynamically Develop from iPSCs over Time

**Figure 2:**
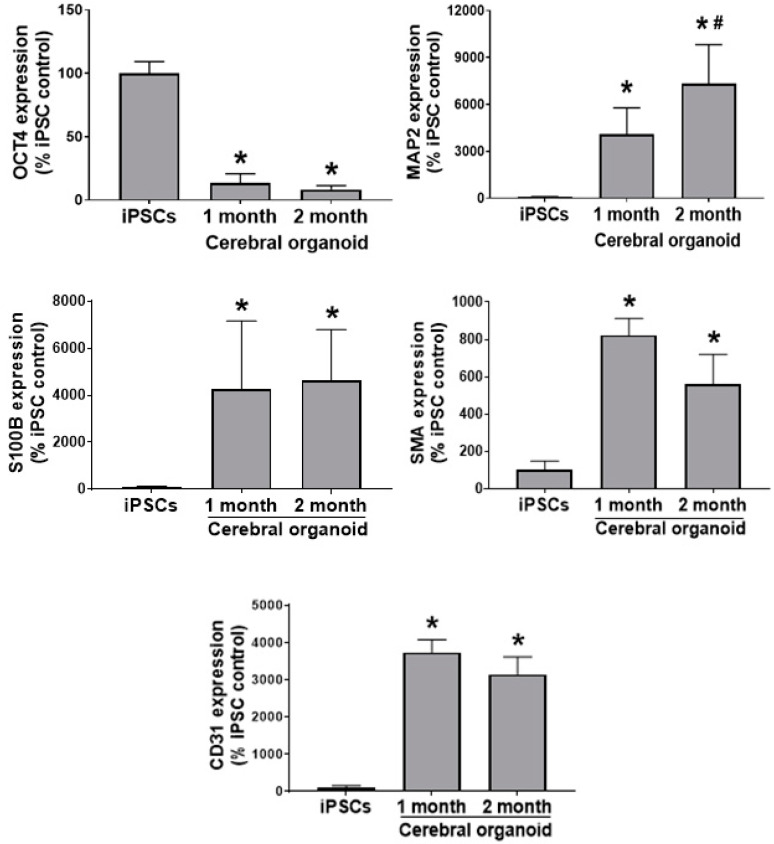
Reduction of the pluripotency marker gene expression and increases in the gene expression of neuron, astrocyte, endothelial cell, and smooth muscle cell markers over time as iPSCs differentiate into cerebral organoids. Quantitative real time polymerase chain reaction (qRT-PCR) reveals decreased OCT4 gene expression over the 2-month differentiation, while MAP2 increases over time. qRT-PCR shows an increase in S100B, SMA and CD31 gene expression over the two-month differentiation process from iPSCs to cerebral organoids. *n* = 4, data are expressed as mean ± standard error of the mean (SEM), and *p* < 0.05 was considered statistically significant. * Denotes a significant difference from iPSCs, and # denotes a significant difference in the 2-month relative to the 1-month stage.

Quantitative reverse transcription polymerase chain reaction (qRT-PCR) analysis further confirms that, as cerebral organoids were differentiated from iPSCs, there was a reduction in pluripotency, as indicated by a significant decrease in OCT4 gene abundance both one month and 2 months into the differentiation process (both *p* < 0.001)but an increase in MAP2 gene abundance after 1 month (*p* = 0.02). At 2 months after the initiation of differentiation, there was an even greater increase in MAP2 abundance relative to what was observed at the iPSC (*p* < 0.001) and 1-month stage. There was a significant, sustained increase in S100B (both *p* < 0.05) in cerebral organoids at 1 month and 2 months old, relative to respective gene abundance in iPSCs. There was a sustained increase in SMA and gene abundance in 1-month and 2-month-old cerebral organoids relative to iPSCs (both *p* < 0.05). Additionally, CD31 gene abundance was increased at 1 month (*p* = 0.016) and 2 months old (*p* = 0.018) relative to iPSCs (Figure 2).

### 3.4. Cerebral Organoids Display Electrical Activity

**Figure 3:**
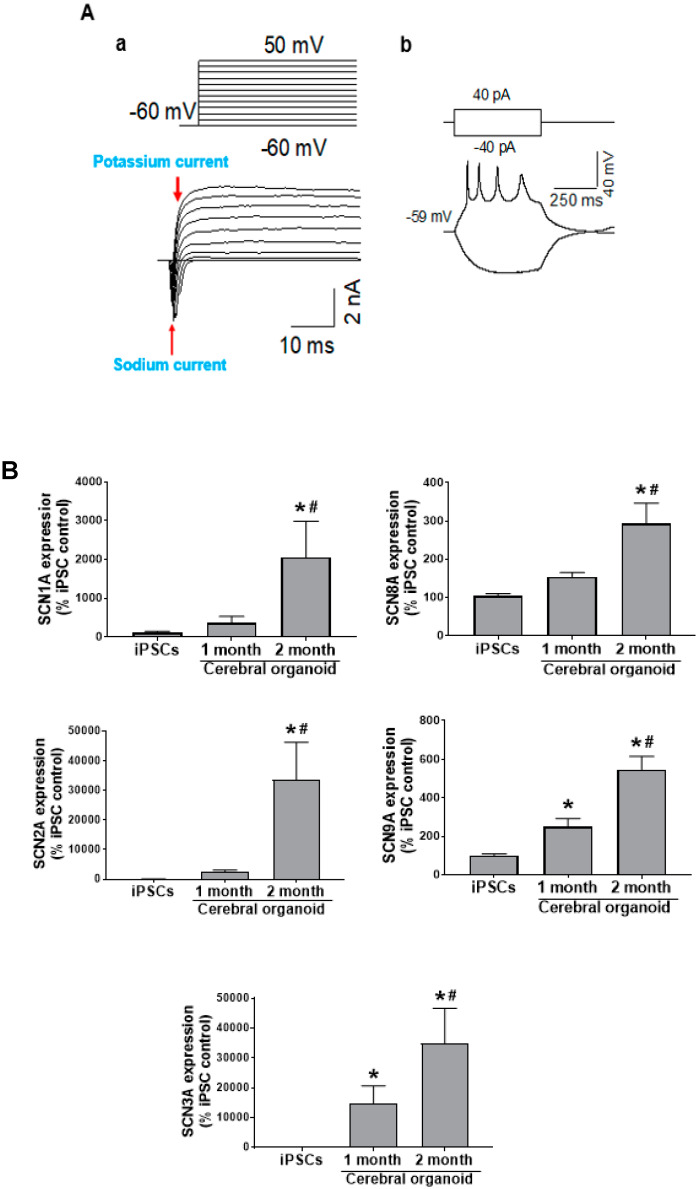
Cerebral organoids possess action potentials. (**A**) Over a range of depolarizing voltages, whole cell recordings from 2-month-old cerebral organoids show evidence of potassium and sodium current (**a**) and action potentials (**b**). (**B**) The genes encoding Nav1-3 and Nav6-7 voltage-gated sodium channels are SCN1-3A and SCN8-9A, respectively. qRT-PCR detected all genes to be upregulated throughout the differentiation process (*n* = 4). Data are expressed as mean ± SEM, and *p* < 0.05 was considered statistically significant. * denotes a significant difference from iPSCs, and # denotes a significant difference in the 2-month relative to the 1-month stage.

Over a range of depolarizing currents, cerebral organoids display action potentials. The sodium current is evidenced by the initial change in current amplitude, and the subsequent potassium current (Figure 3Aa). Spontaneous action potentials were also detected (Figure 3Ab). The voltage-gated sodium channel (SCN) subunit: SCN1A, SCN2A, and SCN3A encode Nav 1 family of sodium channel Nav 1.1, Nav 1.2, and Nav 1.3, respectively, SCN8A encodes Nav 1.6, and SCN9A encodes Nav 1.7. The Nav 1 channels are voltage-gated sodium channels responsible for action potential generation. All channels were significantly increased in 1-month and 2-month-old cerebral organoids during the differentiation from iPSCs to cerebral organoids. SCN2A and SCN8A were not significantly increased one month into the differentiation process, but were significantly upregulated at 2 months relative to earlier time points (all *p* < 0.05). A significant increase in iPSCs was observed at 1 month in SCB1A, SCN3A and SCN9A gene abundance relative to iPSCs, with a further increase at 2 months (all *p* < 0.05; Figure 3B).

### 3.5. Cerebral Organoids Display Various Functional Channel Currents

**Figure 4:**
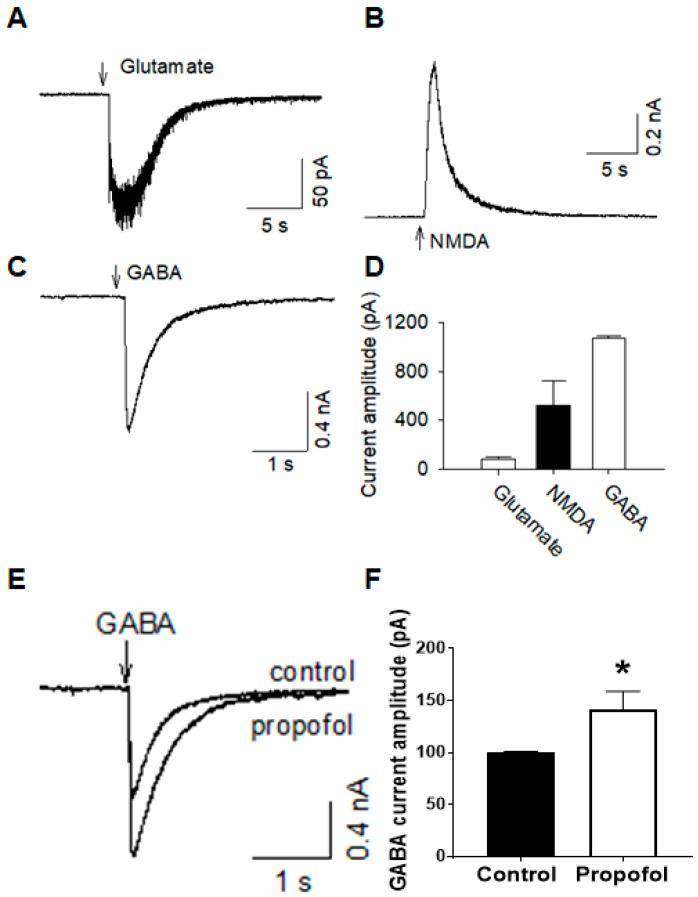
Cerebral organoids possess glutamatergic and GABAergic currents, and respond to propofol. The whole-cell channel current was assessed in 2-month-old cerebral organoid slices. (**A**) Glutamate (0.5 mM) and (**B**) *N*-methyl-d-aspartate (NMDA-0.5 mM), and (**C**) gamma-aminobutyric acid (GABA-0.5 mM) was applied by pressure injection. (**D**) Quantification of each current amplitude. (**E**,**F**) Propofol, a known GABA_A_ receptor agonist, potentiates the GABAergic current. *n* = 5–8, data are presented as mean ± SEM. * denotes *p* < 0.05.

Upon pressure ejection (5 psi, 1 s) of glutamate, *N*-methyl-d-aspartate (NMDA), and Gamma aminobutyric acid (GABA) from a patch pipette, their respective currents were detected in cerebral organoids (Figure 4A–C, respectively.) Exogenous glutamate and NMDA elicited current responses in cerebral organoids due, in part, to activation of α-amino-3-hydroxy-5-methyl-4-isoxazolepropionic acid (AMPA)/other metabotropic and ionotropic glutamate receptors, and NMDA receptors, respectively. Exogenous GABA resulted in current in cerebral organoids, due to activation of the GABA-type A (GABA_A_) receptors. All current values are quantified in Figure 4D. Propofol, a known GABA_A_ receptor agonist, potentiated the GABAergic current, quantitatively shown as an increased current amplitude and prolonged current decay time when propofol was perfused, relative to the control GABA response (Figure 4E). Pressure ejection of propofol (10 μM) itself did not induce any currents (*n* = 5). In developing neurons, the GABAergic current was potentiated by propofol. Current quantification is shown in Figure 4F (*p* < 0.05).

### 3.6. Cerebral Organoids Express Gene Profiles Common to Neurodevelopmental Electrophysiological and Signaling Pathways

**Figure 5:**
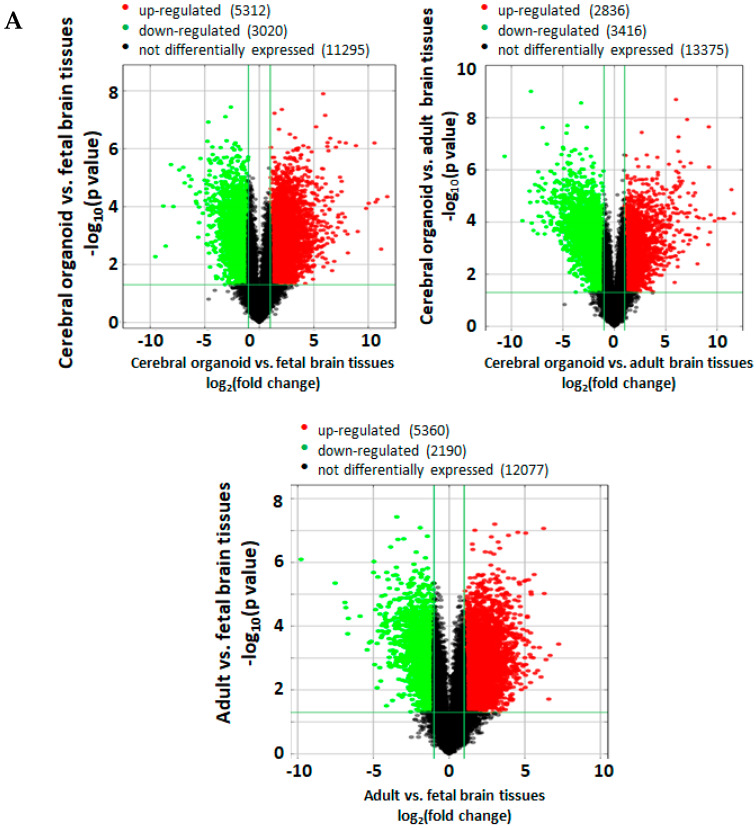

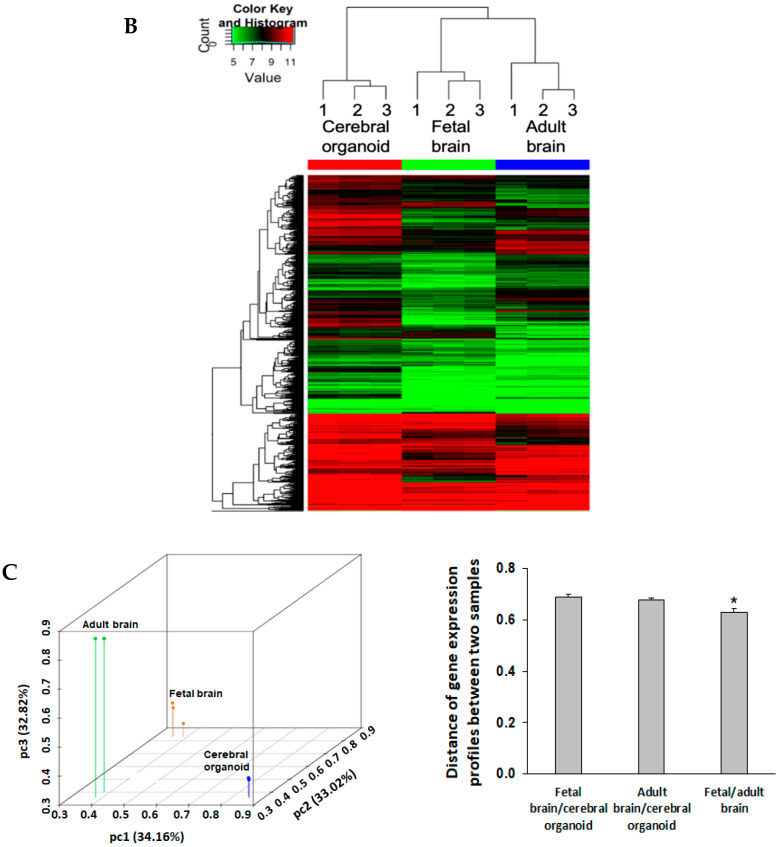
Cerebral organoid and fetal brain tissue shared similarities in mRNA profiles. A microarray analysis of 20,723 genes was performed, with *p* < 0.05 and |FC| ≥ 2 between groups considered statistically significant. (**A**) Volcano plots comparing differential expression of mRNAs between cerebral organoid vs. fetal brain, cerebral organoid vs. adult brain, and adult brain vs. fetal brain samples. The horizontal axis indicates a fold change in log2 scale. The vertical axis indicates *p*-values in -log10 scale. The significantly differentially expressed genes are defined as a ± 2.0 fold change difference (vertical divider lines) and *p* < 0.05 (horizontal divider line), where the red data points are for upregulated, the green for downregulated, and the black for unchanged genes. (**B**) A heatmap shows all significantly dysregulated genes, with high and low expression shown in red and green, respectively. Differentially expressed mRNAs were hierarchically clustered as shown on the top of the heatmap image, indicating the relative closeness in the expression of the samples. Individual samples within each group are designated 1, 2, and 3, and defined in the methods. (**C**) Principal Component Analysis (PCA) analysis of relative gene expression distances between cerebral organoids, fetal brain tissue, and adult brain tissue. The gene expression distances among the samples within the groups and between the groups are depicted in a 3D plot. Each dot represents one sample, as labeled. Principal component (PC) 1, 2, 3 axes were computationally determined by PCA analysis that can maximize the visual distances in the 3D plot. The percentage in each axis represents the proportional contribution of that PC among the 3 PCs in explaining the total gene expression distance. As shown in the PCA plot, the samples within the same experimental groups are very close, whereas the samples between the groups show significant distances, with the shortest distance between fetal and adult brain tissues. Data were expressed as mean ± SEM, and *p* < 0.05 was considered statistically significant. * denotes a significant difference with *p* < 0.05. Additional data related to PCA analysis are found in Supplementary Figure S1, and Supplementary Tables S1 and S2.

Separate comparisons of gene profiles between 1) cerebral organoid versus fetal brain tissue, 2) cerebral organoid versus adult brain tissue, and 3) adult brain tissue versus fetal brain tissue were performed following the microarray of 20,723 genes. The volcano plot illustrated the differentially abundant messenger ribonucleic acids (mRNAs) in the different tissues (Figure 5A). Among 20,723 mRNA transcripts analyzed, there were 8342 differentially expressed mRNAs (5312 upregulated and 3020 downregulated) between cerebral organoids and fetal brain tissues, 6252 mRNAs (2836 upregulated and 3416 downregulated) between cerebral organoids and adult brain tissues, and 7550 mRNAs (5360 upregulated and 2190 downregulated) between fetal and adult brain tissues (fold change above ±2, *p* < 0.05). In the heatmap of all genes assessed by the microarray, clustering/expression values showed gene profile similarity between adult and fetal samples (Figure 5B). PCA exhibited the shortest distance of all analyzed gene expression profiles, further confirming the most similarity between adult and fetal brain tissues compared with cerebral organoids (Figure 5C). The PCA elbow plot and output of the PCA analysis are included in the Supplementary (Supplementary Figure S1 and Table S1, respectively). A scree plot displays how much variation each principal component captures from the data. Supplementary Table S2 lists the contributions of the genes to PC3. The genes that contribute most to PC3 have the biggest absolute rotated component (RC) 3 values. RCs are PCA scores for principal components PC1, 2, and 3.

Ingenuity Pathway Analysis (IPA) analysis nominated various canonical pathways related to neural development, network formation, and electrophysiological signaling to be dysregulated between cerebral organoids, fetal brain tissue, and adult brain tissue. Based on the Z score, an indication of the degree to which a treatment group deviates from the mean, cerebral organoids and fetal tissue were predicted to show the most similar pathway activation. Of the selected canonical pathways, only calcium signaling, cyclic adenosine monophosphate (cAMP) response element-binding protein (CREB) signaling in neurons, glutamate receptor signaling, and the synaptogenesis signaling were predicted to be downregulated in cerebral organoids relative to fetal samples. Nearly all cerebral organoid and fetal pathway phenotypes (e.g., endocannabinoid developing neuron pathway, endocannabinoid neuronal synapse pathway, glial derived neurotrophic factor family ligand receptor interactions, neurotrophin/tyrosine kinase signaling, synaptic long-term depression, synaptic long-term potentiation, calcium signaling, CREB signaling neurons, glutamate receptor signaling, synaptogenesis signaling pathway) were predicted to be consistently downregulated compared with adult brain tissue (Table 2).

## 4. Discussion

In the current study, we characterized cerebral organoids as models of the human brain in the aspects of development, electrophysiological properties, gene profiles, and their potential to be utilized in physiological studies of human brain structure and function. We found that as cerebral organoids develop from iPSCs in the culture over time, they exhibit (1) a heterogeneous gene and protein markers of various brain cells, such as neuron, astrocytes, and vascular cells including endothelial cells and smooth muscle cells, (2) an increased gene expression of brain cell-specific markers over time, and (3) functional electrophysiological properties as evidenced by the neurons with action potential and synapse-like structure, functional response of ion channels to the drug stimulation. Additionally, a bioinformatics analysis of 20,723 gene expression profiles showed a similar distance between gene profiles in cerebral organoids compared to fetal and adult brain tissues. The subsequent IPA analysis of select canonical pathways related to neural development, network formation, and electrophysiological signaling, revealed that only calcium signaling, CREB signaling in neurons, glutamate receptor signaling, and synaptogenesis signaling were predicted to be downregulated in cerebral organoids relative to fetal brain samples. Nearly all cerebral organoid and fetal brain pathway phenotypes were predicted to be downregulated compared with adult brain tissues. These novel findings bring scientists one step closer to understanding how to better utilize cerebral organoids for their wide application in modeling human brains in health and disease, and testing drugs for their efficacy and toxicity. 

Moving forward from the iPSC stage, we noted a downregulation in the OCT4 pluripotency marker and Pax6 neural stem cell marker, and an upregulation in the markers of neural cell types (neurons and astrocytes; Figure 2). In a similar manner to the current study, others recently reported a similar upregulation in neurons and glia as cerebral organoid differentiation from stem cells progressed [22,23]. Interestingly, we also observed what appeared to be an attempt by the cerebral organoids to produce blood vessels (CD31 and SMA), (Figure 2), suggesting the presence of cell types not originating from the ectoderm [24]. This was perhaps surprising and not reported in previous studies, as the lack of vasculature within cerebral organoids, and other organoid applications, is one of their greatest limitations, and represents a problem to combat for more functional models. The intrinsic, spontaneous emergence of (at least) constituents of blood vessels is striking and further illustrates tissue architecture, and the supporting cell types possibly contributing to the greater maturity and complexity of the cerebral organoid model. It may be possible in the future to develop more targeted differentiation approaches that allow for their presence along with other cell types within the neural tissue.

With neuronal maturation at the 2-month time point in the current study, profound synapse formation was also evident (Figure 1). Allowing the cerebral organoids to self-assemble allows for the events encompassing synaptogenesis, such as neuronal migration, axonal pathfinding and dendritic contact with other neurons, and synaptic pruning and survival [25]. This likely allows for the intact tissue comprised of pre- and post-synaptic neurons and mature synaptic structures not present in 2D neuron models [26]. Previous reports show that the cerebral organoid model has remarkable differentiation efficacy in resembling various regions, cell types, and functions and developmental processes occurring in the in vivo human brain [14]. Past observations within cerebral organoids regarding their neurodevelopment, such as cortical layers and progenitor zone organization, have reinforced their utilization over animal models. Furthermore, the ability of various neural cell type differentiation from the neuroectoderm based on stem cell orientation in the early stages is unique to cerebral organoids, with the absence of any need for separation of cell types or protocols catered to a given cell population. In the perinatal period, accompanying the rapid increase in brain size, there are a greater number of neurons relative to glia [27,28]. By studying cerebral organoids at the 2-month time point, in which synapse-positive neurons are prominent relative to other cell types, it suggests the brain is at a more advanced stage of development. This informed our decision to use 2-month-old cerebral organoids to study functional characteristics of the model and the responsiveness of neurons to drugs in the current study. However, the dynamic nature of the model and shifting cellular population emphasizes its potential in being employed for studies at various stages of brain development in the future.

In order to connect this dynamic tissue architecture to functional neuronal activity, we measured electrical current, spontaneous action potentials, and neurotransmitter channel activity in 2-month-old cerebral organoids. Other studies have already reported calcium responses in cerebral organoids [14], sodium and potassium currents [29], pace-making and synaptic activity in cerebral organoids and midbrain organoids [30,31,32]. Also, neurons responsive to different neurotransmitters have previously been observed, such as GABAergic [33] and glutamatergic neurons [23,34]. However, functional channel current in these neuronal populations and channel responses to drugs have not been thoroughly investigated. Through whole-cell patch clamping of slices from cerebral organoids, we detected an initial rapid sodium current and, later, a prolonged potassium current over a range of depolarizing currents (Figure 3Aa), in addition to action potentials in this study (Figure 3Ab). There was no success in recording evoked action potentials from one-month-old cerebral organoids, and a significant increase in sodium channel genes over the course of differentiation, indicating greater electrochemical maturity at the 2-month time point (Figure 3B). 

The interaction between multiple cells during the developmental process might account for this greater neuronal maturity not observed in other in vitro models [7,35]. While recent studies discussed the observed gene expression of glutamate and GABA channels, observed excitatory and inhibitory neurons [36], and detected presence of GABAergic interneurons, we were the first to utilize patch clamping to detect channel current for these neurotransmitters. Upon pressure injection of GABA, AMPA, and NMDA, we detected that cerebral organoids showed a response. While we found that the concentrations of neurotransmitters exogenously added to elicit a rapid response were greater than what would be expected in vivo, cerebral organoids at 2 months show more maturity than two-dimensional applications [37]. Additionally, in response to propofol, a drug known to be the agonist of the GABA_A_ receptor [38], we observed its interaction with endogenous GABA receptors within the cerebral organoid, and its potentiation in terms of prolonging the GABA current and increasing the decay time (Figure 4). This study, for the first time, shows that cerebral organoids show compositional and electrochemical phenotypes that promote their use in physiological studies. Additionally, drug responses relevant to what occurs in the in vivo brain are promising for future drug testing and functional studies.

Researchers have previously estimated that cerebral organoids recapitulate the developing brain in the first trimester, largely based on histology [13]. Cerebral organoids have been proposed as an excellent neurodevelopmental model in capturing the dynamic process of developmental expansion of brain regions, cell identities, and neurogenesis [14]. More recently, others have reported that the transcriptomic profiles of cerebral organoids shift drastically throughout differentiation from the stem cell to embryoid body to cerebral organoid stage. Upon reaching the cerebral organoid stage, there are fewer changes in transcriptomic remodeling. Additionally, they found that similar methylation patterns were observed in both cerebral organoids and the early-to-middle fetal brain, indicative of the repression of transcription that occurs as the brain becomes more developed [39]. The cerebral organoid cortex and the fetal neocortex also shared similar genetic programming during development [16], and forebrain patterning and self-organizational events within organoids were similar to what is seen in vivo [40,41]. While the cerebral organoid has been classified as a fetal model based on comparing cerebral organoids to fetal histological samples [14], less is known about the age equivalency from a functional standpoint. We were able to address this, in part, in the current study, first through the detection of neurotransmitter current and action potential generation. Based on electrical activity, it has been previously reported that there was an increase in electrical activity over 10 months of differentiation in cerebral organoids [42]. While cerebral organoids remain less mature than the adult brain, researchers have hypothesized that this may be due to the fact that the developmental nature of the organoids yields a mixture of new neurons and more mature neurons with and without electrochemical activity, respectively [36]. Earlier studies have used high-throughput transcriptomic approaches to correlate genes involved in neuronal activity with electrophysiological characteristics and responses [43]. As it is related to electrophysiological maturity throughout fetal development, the inception of immature electrical activity is believed to occur largely in the second semester based on other studies [44,45], and the majority of synapse formation occurs in the third trimester [46]. Thus, early estimates about the cerebral organoids resembling the first trimester may not tell the entire story. Furthermore, continuing to understand functional components of the cerebral organoids sheds light on the applicability of the model for research on the developing brain, and will likely continue in the near future in the field.

To expound on these alternative approaches in understanding cerebral organoid maturity and functional human brain age equivalency, the current study profiled novel similarities/differences in genes and pathways between 2-month-old cerebral organoids, fetal human brain tissue (second to third trimester), and adult human brain tissue (21–66 years) (Figure 5A). It has been previously shown that cerebral organoids share similarities with the fetal brain tissues in several aspects. For instance, Camp et al. studied cell-specific gene expression profiles on neural progenitor cells and neurons by comparing organoids with the 12–13 weeks post-conception fetal neocortex using a single-cell RNA sequencing approach [16]. Luo et al. investigated epigenetic signatures of cerebral organoids [39]. Our current study is different from these two studies in terms of the aspects of fetal tissue compared, approaches used, and gene profiles covered. In addition, the comparison of organoids with human adult brains has not been conducted by any labs prior to our work. Thus, knowing the similarity and differences in the gene expression and related signaling pathways among organoids, fetal and adult tissues is valuable for future work studying neurodevelopment, neurological diseases, and drug testing. 

First, when looking at expression of all 20,723 genes obtained via the microarray, fetal and adult brain tissues showed the greatest similarities based on gene clustering (Figure 5B). A bioinformatics analysis of 20,723 gene expression profiles showed a similar quantitative distance between gene profiles in cerebral organoids compared to fetal and adult brain tissues. Fetal and adult brain gene expression profiles share more similarities than they share with cerebral organoids (Figure 5C, Supplementary Figure S1, and Supplementary Tables S1 and S2). This is likely due to the fact that both samples are taken from actual human brain tissue, versus the cerebral organoid in vitro model we employed in the current study. These RNA samples used in the fetal and adult samples also underwent normal development in the presence of other factors present in the developing human body, which may account for other differences from cerebral organoids. The vast array of genes tested should also be noted, as many are not related to neural phenotypes. However, when specific attention was given to neurodevelopmental and neural signaling pathways and their associated genes through IPA analysis of dysregulated expressed genes among cerebral organoids, fetal and adult brain tissues, cerebral organoids and fetal brain tissue showed the most overlap. Specifically, we report that signaling pathways predicted to show similarity between cerebral organoids and fetal brains were apoptosis, calcium transport, neurodevelopment, trophic factor signaling, and synaptic plasticity such as synaptic long-term depression and potential (Table 2). Compared with cerebral organoids and fetal brain samples, adults showed a greater predicted activation of many maturational and signaling pathways, including calcium signaling, the development of different neuron subpopulations and neurotrophic factor signaling; synaptic genes that were related to brain maturation signaling were differentially expressed among cerebral organoids, fetal and adult brains (Table 2). 

Cerebral organoids are currently used as models of the developing brain, while future approaches may be geared toward obtaining more of an adult-like maturational status by targeting transcriptomic signaling pathways. For instance, activating transcription factor 4 (ATF4) is associated with early synaptogenesis [47], and accordingly, is more highly expressed in cerebral organoids versus adult brain tissue. As an example of how cerebral organoids are functionally immature relative to the adult brain, calcium/calmodulin-dependent protein kinase II beta (CAMK2B) is involved in neuron activity related to synaptic plasticity [48], and is more highly expressed in adult brain tissue versus cerebral organoids. Similarly, gamma-aminobutyric acid type a receptor subunit alpha1 (GABRA1) is involved in neural maturation [49] and is more highly expressed in adult versus cerebral organoids, further indicating cerebral organoids as a developmental model Thus, our dataset provides a tremendous insight regarding neural-related transcriptomic differences across the human brain at different stages, and aids in the approximation of the maturational and functional characteristics of cerebral organoids. 

Overall, for the first time, this study reported neurotransmitter currents and the functional response of channel activities of cerebral organoids to the drug propofol. These observed characteristics of diverse cell components, functional channel activities, and progressive development in culture further suggest that this model has tremendous promise in being the more clinically relevant manner by which scientists can dissect detailed cellular, molecular, and functional mechanisms of brain development, physiology and disorders. Additionally, the findings from the comparisons of human iPSC-derived cerebral organoids to both human fetal brain samples and the adult human brain help us better understand this cerebral organoid-based cutting-edge platform and its wide uses in modeling the human brain in health and disease, development, and testing drug efficacy and toxicity.

## Figures and Tables

**Table 1:** Primer sequences for genes tested

Gene	Forward Sequence 5′ to 3′	Reverse Sequence 3′ to 5′	PCR Product Length (bp)
Cd31	AAGTGGAGTCCAGCCGCATATC	ATGGAGCAGGACAGGTTCAGTC	133
Gapdh	GTCTCCTCTGACTTCAACAGCG	ACCACCCTGTTGCTGTAGCCAA	131
Map2	AGGCTGTAGCAGTCCTGAAAGG	CTTCCTCCACTGTGACAGTCTG	153
S100b	GAAGAAATCCGAACTGAAGGAGC	TCCTGGAAGTCACATTCGCCGT	135
Scn1a	GGACTGTATGGAGGTTGCTGGT	GCAAGGTTGTCTGCACTAAATGAG	132
Scn2a	CTAGCCTCACTGTGACAGTACC	TCAACCGTGCTGCCTTCAGATG	145
Scn3a	CGTCACCTACTGGACAACTTCC	TCACGGCTCTTTGCCTTCCAGA	129
Scn8a	GGATTGAGACCATGTGGGACTG	ATCTGTGGCAGCCAGGTTGTCT	158
Scn9a	GTGGAAGGATTGTCAGTTCTGCG	GCCAACACTAAGGTGAGGTTACC	140
Sma	GATCTGGCACCACTCTTTCTAC	CAGGCAACTCGTAACTCTTCTC	479

Base pairs (bp); Cluster of differentiation 31 (CD31); glyceraldehyde 3-phosphate dehydrogenase (GAPDH); microtubule-associated protein 2 (MAP2); S100 calcium binding protein B (S100B); voltage-gated sodium channels (SCN); smooth muscle actin (SMA).

**Table 2:** Relevant developmental signaling pathways nominated by Ingenuity Pathway Analysis (IPA).

Canonical Pathway [Upregulation, Downregulation, or No Significant Difference (ns) Determined by Z Score Indicated below]	Cerebral Organoids vs. Adult Brains (Z Score)	Fetal Brains vs. Adult Brains (Z Score)	Cerebral Organoid vs. Fetal Brains (Z Score)	Relevance
Apoptosis	up (2.196)	ns	ns	Cell death, neurodegeneration
Calcium transport	down (−1.633)	ns	ns	Intracellular messaging, cell depolarization, synaptic transmission
Dendritic cell maturation	ns	Down (2.019)	ns	Neuron morphology
Endocannabinoid developing neuron pathway	down (−2.655)	Down (3.244)	ns	Neuron activity based, differentiation, neuron survival
Endocannabinoid neuronal synapse pathway	down (−5.588)	Down (2.142)	ns	Synaptic plasticity, hippocampus signaling
Glial derived neurotrophic factor family ligand receptor interactions	down (−2.66)	Down (3.182)	ns	Neurotrophic support, neuron growth, survival
Neurotrophin/tyrosine kinase signaling	down (−2.2)	Down (4.382)	ns	Neuron growth and survival
Synaptic long-term depression	down (−4.919)	Down (3.354)	ns	Activity-dependent reduction in synaptic signaling
Synaptic long-term potentiation	down (−4.608)	Down (2.728)	ns	Activity-dependent strengthening of neuron synapses
Calcium signaling	down (−5.661)	down (4.589)	down (−3.250)	Cellular signaling, synaptic transmission
CREB signaling neurons	down (−5.578)	down (6.306)	down (−0.911)	Neuron plasticity, neurodevelopment
Glutamate receptor signaling	down (−3.3)	Down (2.353)	down (−3.051)	Excitatory neurotransmitter pathway, neurodevelopment
Synaptogenesis signaling pathway	down (−7.952)	Down (8.06)	down (−1.741)	Neuron activity based, neuron network formation, pruning, neuron growth factors

Ingenuity Pathway Analysis (IPA); no significant difference (ns); upregulation (up); downregulation (down). cyclic adenosine monophosphate (cAMP) response element-binding protein (CREB); the abbreviations of the genes were defined in the file that is available on the National Center for Biotechnology Information (NCBI) data base, with Gene Expression Omnibus (GEO) Submission Number GSE134363.

## Supplementary Materials

The following are available online at https://www.mdpi.com/2073-4409/9/5/1301/s1, Figure S1: The principal component analysis elbow plot plots the eigenvalues for the first 9 principal components, Table S1: Output of principal component analysis, Table S2: mRNA-PCA RC3 score.

## Abbreviations

A	adult brain
AMPA	α-amino-3-hydroxy-5-methyl-4-isoxazolepropionic acid
cAMP	cyclic adenosine monophosphate
CD31	cluster of differentiation 31
CO	cerebral organoid
CREB	cAMP response element-binding protein
cDNA	complementary deoxyribonucleic acid
Ct	cycle threshold
dNTP	deoxynucleoside triphosphate
down	downregulation
EGTA	ethylene glycol-bis (β-aminoethyl ether)-N,N,N′,N′-tetraacetic acid
EDTA	Ethylenediaminetetraacetic acid
F	fetal brain
GABA	gamma-aminobutyric acid
IPA	Ingenuity Pathway Analysis
GABA_A_ receptor	GABA type A (GABA_A_) receptor
Gapdh	glyceraldehyde 3-phosphate dehydrogenase
HEPES	4-(2-hydroxyethyl)-1-piperazineethanesulfonic acid
iPSCs	induced pluripotent stem cells
MAP2	microtubule-associated protein 2
Mark1	MAP/microtubule affinity-regulating kinase 1
NMDA	*N*-methyl-d-aspartate
ns	no significant difference
OCT4	octamer binding transcription factor 4
PBS	phosphate buffered saline
qRT-PCR	quantitative reverse transcription polymerase chain reaction
S100B	S100 calcium binding protein B
SCN/Nav	voltage-gated sodium channels
SMA	smooth muscle cell marker
2D	two-dimensional
3D	three-dimensional
up	upregulation
