# Supplementary material for: Dynamic Characterization of Structural, Molecular, and Electrophysiological Phenotypes of Human-Induced Pluripotent Stem Cell-Derived Cerebral Organoids, and Comparison with Fetal and Adult Gene Profiles
Source: Cells. 2020 May 23;9(5):1301. doi: 10.3390/cells9051301 (PMC7291286; doi:10.3390/cells9051301)
Supplement: Supplementary file 1 [file cells-09-01301-s001.pdf]

Supplemental Table 1. Output of principal component analysis (PCA)

	PC1	PC2	PC3	PC_remainders
Proportion of Variance	34.9%	32.5%	31.5%	1.1%
Cumulative Variance	34.9%	67.4%	98.9%	
Proportion Explained	35.3%	32.9%	31.8%	
Cumulative Proportion	35.3%	68.2%	100%	

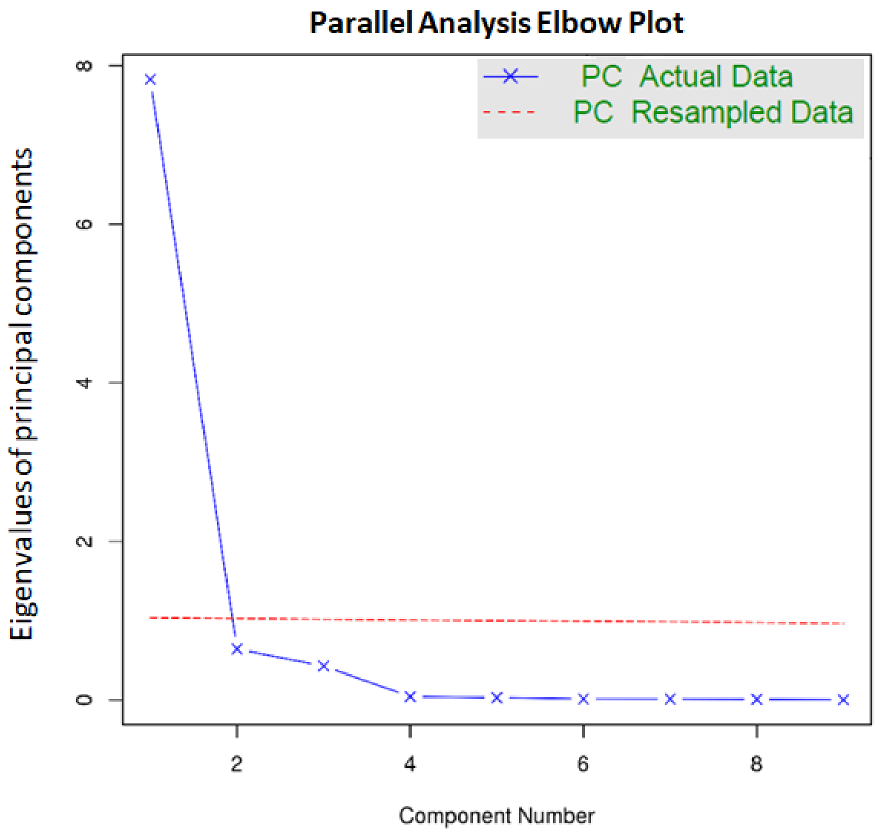

Supplemental Figure 1. The principal component analysis (PCA) elbow plot plots the eigenvalues for the first 9 principal components.

Supplemental Table 2 mRNA-PCA RC3 score

Scores	RC3	Abs(RC3)	type	seqname
ASHGV40055858	5.353505	5.353505465	protein_coding	NM_033207
ASHGV40054263	-4.74122	4.741220696	protein_coding	NM_021992
ASHGV40037745	4.552353	4.552352526	protein_coding	NM_004684
ASHGV40027601	4.502064	4.502064363	protein_coding	NM_020711
ASHGV40032419	4.445723	4.445722839	protein_coding	NM_006272
ASHGV40023551	4.350516	4.350516341	protein_coding	NM_032649
ASHGV40037863	4.341397	4.341397005	protein_coding	NM_031279
ASHGV40031307	4.297427	4.297426819	protein_coding	NM_130811
ASHGV40055240	4.2288	4.228799708	protein_coding	NM_000533
ASHGV40027129	4.092059	4.092059343	protein_coding	NM_207362
ASHGV40036548	4.040153	4.040152717	protein_coding	uc003epu.2
ASHGV40027731	4.019017	4.019016837	protein_coding	NM_001822
ASHGV40016877	-3.99746	3.997459614	protein_coding	NM_004701
ASHGV40042662	3.995197	3.9951969	protein_coding	NM_000806
ASHGV40006843	3.947358	3.947357941	protein_coding	NM_004171
ASHGV40039817	3.939329	3.939329017	protein_coding	NM_007030
ASHGV40040970	-3.93411	3.934113677	protein_coding	NM_003122
ASHGV40027992	-3.92913	3.929131668	protein_coding	NM_020989
ASHGV40012096	3.903573	3.90357303	protein_coding	NM_001033677
ASHGV40056804	-3.88966	3.889658359	protein_coding	NM_001643
ASHGV40034474	3.885575	3.885574839	protein_coding	NM_007177
ASHGV40033554	-3.86197	3.861970505	protein_coding	NM_001886
ASHGV40040455	3.844007	3.844006805	protein_coding	NM_001190470
ASHGV40016695	-3.82085	3.820854913	protein_coding	NM_016359
ASHGV40014871	3.81865	3.818649592	protein_coding	NM_198406
ASHGV40020728	-3.78703	3.787031044	protein_coding	NM_000042
ASHGV40053664	3.741794	3.741794053	protein_coding	NM_000954
ASHGV40054983	3.717272	3.717272053	protein_coding	NM_014767
ASHGV40028548	3.710798	3.710798421	protein_coding	NM_003385
ASHGV40028484	-3.70421	3.704211393	protein_coding	NM_001165931
ASHGV40028426	-3.69555	3.695551901	protein_coding	NM_003108
ASHGV40052821	-3.69122	3.691220206	protein_coding	NM_014791
ASHGV40034230	3.689254	3.68925443	protein_coding	NM_020707
ASHGV40037876	-3.67145	3.671446659	protein_coding	NM_153426
ASHGV40039044	-3.66123	3.661231673	protein_coding	NM_001134
ASHGV40049838	3.632142	3.632141562	protein_coding	NM_001040092
ASHGV40021518	-3.63046	3.630464563	protein_coding	NM_005208
ASHGV40019179	3.625233	3.625232861	protein_coding	NM_002996
ASHGV40043173	-3.62149	3.6214909	protein_coding	NM_003546
ASHGV40033128	3.612642	3.612642316	protein_coding	NM_174858
ASHGV40024698	-3.60997	3.609968463	protein_coding	NM_001161748
ASHGV40024680	3.596959	3.596959346	protein_coding	NM_002774
ASHGV40031031	3.563147	3.563146561	protein_coding	NM_003657
ASHGV40039535	-3.55847	3.558469841	protein_coding	NM_005141
ASHGV40029886	-3.55321	3.553213357	protein_coding	NM_000090

ASHGV40026359	3.505884	3.505883944	protein_coding	NM_012344
ASHGV40017291	-3.49146	3.491457236	protein_coding	NM_006011
ASHGV40010293	-3.47997	3.479966259	protein_coding	NM_203394
ASHGV40006569	-3.46442	3.464424585	protein_coding	NM_000559
ASHGV40041017	3.436599	3.436599174	protein_coding	NM_005613
ASHGV40052207	-3.41324	3.413235589	protein_coding	NM_001633
ASHGV40007623	3.412378	3.412378076	protein_coding	NM_001885
ASHGV40033147	3.378391	3.37839105	protein_coding	NM_006941
ASHGV40022978	3.362685	3.362685227	protein_coding	NM_001025101
ASHGV40020278	-3.35277	3.352765614	protein_coding	NM_001067
ASHGV40019958	3.349923	3.34992334	protein_coding	NM_016084
ASHGV40010276	3.345988	3.345987684	protein_coding	NM_139137
ASHGV40037393	3.334573	3.334572789	protein_coding	NM_015230
ASHGV40020545	-3.3251	3.325102021	protein_coding	NM_000088
ASHGV40046052	-3.30174	3.301741725	protein_coding	NM_001301056
ASHGV40039043	-3.28247	3.282466775	protein_coding	NM_000477
ASHGV40037462	3.280217	3.280216519	protein_coding	NM_173536
ASHGV40051831	3.275176	3.275176498	protein_coding	NM_001206
ASHGV40034109	-3.26103	3.261031216	protein_coding	NM_005442
ASHGV40029770	3.234167	3.234167401	protein_coding	NM_007023
ASHGV40049053	3.233867	3.233867411	protein_coding	NM_001831
ASHGV40032747	-3.22824	3.22824406	protein_coding	ENST00000596691
ASHGV40049531	3.202934	3.202933874	protein_coding	NM_002677
ASHGV40022612	3.202807	3.202807186	protein_coding	NM_001650
ASHGV40027255	-3.19929	3.199294734	protein_coding	NM_004336
ASHGV40053784	3.190058	3.190057614	protein_coding	NM_005278
ASHGV40011556	3.17903	3.179029783	protein_coding	uc001sos.4
ASHGV40011740	3.175261	3.175261134	protein_coding	NM_005639
ASHGV40053679	3.171326	3.171326039	protein_coding	NM_018245
ASHGV40003226	-3.16891	3.168912588	protein_coding	NM_001293171
ASHGV40036780	3.156252	3.156251616	protein_coding	NM_005025
ASHGV40046251	-3.15265	3.152647304	protein_coding	NM_006547
ASHGV40018332	-3.14842	3.14842422	protein_coding	NM_005558
ASHGV40006233	3.130306	3.130305942	protein_coding	NM_000684
ASHGV40043168	-3.12126	3.121257222	protein_coding	NM_021066
ASHGV40056647	-3.11951	3.119508936	protein_coding	NM_003960
ASHGV40033425	3.109704	3.109703938	protein_coding	NM_018584
ASHGV40007112	3.108948	3.108948431	protein_coding	NM_007069
ASHGV40057746	3.107604	3.107604048	protein_coding	NM_001190702
ASHGV40045928	-3.1045	3.104501273	protein_coding	NM_000301
ASHGV40016348	3.099575	3.099574782	protein_coding	ENST00000412799
ASHGV40046201	-3.09484	3.094844209	protein_coding	NM_006408
ASHGV40040484	3.094611	3.094611342	protein_coding	NM_005711
ASHGV40016204	3.086862	3.086862034	protein_coding	NM_002891
ASHGV40005147	3.08624	3.086239887	protein_coding	NM_177400
ASHGV40035359	-3.08161	3.081611959	protein_coding	NM_006548
ASHGV40020504	-3.07681	3.076812664	protein_coding	NM_018952

ASHGV40042398	3.067845	3.067844805	protein_coding	NM_001007189
ASHGV40036937	-3.06545	3.065449669	protein_coding	NM_001622
ASHGV40056634	-3.06508	3.065083332	protein_coding	NM_178435
ASHGV40038356	-3.0606	3.060596173	protein_coding	NM_024629
ASHGV40020423	3.060555	3.060554802	protein_coding	NM_002055
ASHGV40018015	-3.06014	3.060140111	protein_coding	NM_018136
ASHGV40025235	-3.05031	3.050309802	protein_coding	uc001ass.2
ASHGV40012011	3.036064	3.036064268	protein_coding	NM_001143854
ASHGV40031168	3.034916	3.034916053	protein_coding	NM_015894
ASHGV40020137	3.026679	3.026678787	protein_coding	NM_014210
ASHGV40000119	3.023359	3.023359312	protein_coding	ENST00000361227
ASHGV40046203	-3.01932	3.019318836	protein_coding	NM_176813
ASHGV40013592	3.013277	3.013277021	protein_coding	NM_001306070
ASHGV40007696	3.011443	3.011442983	protein_coding	NM_004588
ASHGV40060830	3.006982	3.006982454	protein_coding	uc003afp.3
ASHGV40040844	-2.99763	2.997627552	protein_coding	NM_002302
ASHGV40053698	2.99568	2.995680438	protein_coding	ENST00000361453
ASHGV40052342	2.994061	2.994061478	protein_coding	NM_001035254
ASHGV40046186	2.993438	2.993438132	protein_coding	NM_004080
ASHGV40006947	2.990243	2.990243248	protein_coding	NM_004476
ASHGV40037523	2.989948	2.989948492	protein_coding	NM_139211
ASHGV40013793	2.980363	2.98036324	protein_coding	NM_002933
ASHGV40018184	-2.9766	2.976604575	protein_coding	NM_014875
ASHGV40040999	2.975915	2.975915077	protein_coding	NM_171825
ASHGV40057581	2.972344	2.972344283	protein_coding	ENST00000361851
ASHGV40037603	-2.96497	2.964968903	protein_coding	NM_005420
ASHGV40054143	2.964203	2.964202947	protein_coding	NM_021963
ASHGV40020633	2.962725	2.962724761	protein_coding	NM_004574
ASHGV40013800	2.954086	2.954085774	protein_coding	NM_201535
ASHGV40009314	2.954059	2.954058995	protein_coding	NM_001037558
ASHGV40021155	-2.94972	2.949721564	protein_coding	NM_003963
ASHGV40049033	2.932942	2.932941539	protein_coding	NM_006158
ASHGV40027664	2.932528	2.932527951	protein_coding	NM_001165963
ASHGV40010384	-2.92916	2.929156972	protein_coding	NM_002345
ASHGV40044143	-2.92576	2.925755076	protein_coding	NM_175747
ASHGV40033634	2.924931	2.924930952	protein_coding	NM_003405
ASHGV40004953	2.921923	2.92192265	protein_coding	NM_025015
ASHGV40047387	2.918075	2.918075175	protein_coding	NM_002847
ASHGV40042313	2.917437	2.917436711	protein_coding	NM_175873
ASHGV40050400	2.907134	2.907133517	protein_coding	NM_005382
ASHGV40031460	-2.90449	2.904494284	protein_coding	ENST00000361350
ASHGV40044816	2.897039	2.897038587	protein_coding	NM_206809
ASHGV40021844	2.891882	2.891881918	protein_coding	NM_006178
ASHGV40008088	-2.89169	2.891689108	protein_coding	NM_003390
ASHGV40013845	2.883811	2.883810797	protein_coding	NM_020372
ASHGV40055571	-2.85799	2.857993334	protein_coding	NM_012253
ASHGV40050924	2.852563	2.852563356	protein_coding	NM_022351

ASHGV40055066	-2.85252	2.85252166	protein_coding	NM_012310
ASHGV40054214	2.851862	2.851861826	protein_coding	NM_004538
ASHGV40028919	2.851063	2.851063187	protein_coding	NM_005917
ASHGV40054537	2.849712	2.849712384	protein_coding	NM_031462
ASHGV40049777	2.849658	2.849658161	protein_coding	NM_014379
ASHGV40008662	2.84447	2.844469928	protein_coding	NM_006848
ASHGV40005692	-2.83282	2.832815448	protein_coding	NM_001786
ASHGV40040785	-2.8321	2.832098495	protein_coding	NM_001999
ASHGV40033996	2.830544	2.830543566	protein_coding	NM_001001331
ASHGV40023824	-2.82771	2.8277076	protein_coding	NM_032447
ASHGV40028139	2.822471	2.822471329	protein_coding	NM_014689
ASHGV40037754	2.819237	2.819236846	protein_coding	NM_153757
ASHGV40053211	-2.8191	2.819100589	protein_coding	NM_001855
ASHGV40037594	-2.80738	2.807379566	protein_coding	NM_024743
ASHGV40044764	-2.80637	2.806365041	protein_coding	NM_003495
ASHGV40017720	2.802912	2.802911627	protein_coding	NM_001888
ASHGV40018837	2.801782	2.801781955	protein_coding	NM_052944
ASHGV40009267	2.800995	2.800995187	protein_coding	NM_003105
ASHGV40005415	2.791652	2.791651591	protein_coding	NM_020752
ASHGV40012359	2.784482	2.78448229	protein_coding	NM_152704
ASHGV40010744	2.782488	2.782487885	protein_coding	NM_025140
ASHGV40019863	-2.77758	2.777578692	protein_coding	NM_002472
ASHGV40018891	-2.77647	2.776470984	protein_coding	NM_001199050
ASHGV40055540	2.775179	2.775178563	protein_coding	NM_032882
ASHGV40018183	-2.77486	2.77486214	protein_coding	NM_024939
ASHGV40053699	2.773354	2.773354384	protein_coding	ENST00000361739
ASHGV40054324	-2.76708	2.767081139	protein_coding	NM_000555
ASHGV40037050	-2.76651	2.766506942	protein_coding	NM_152672
ASHGV40055233	2.766349	2.766349356	protein_coding	NM_014380
ASHGV40031136	-2.76063	2.760625481	protein_coding	NM_080473
ASHGV40038234	2.759343	2.759342737	protein_coding	NM_016950
ASHGV40047281	-2.75751	2.757512377	protein_coding	NM_001203247
ASHGV40016661	-2.75675	2.756751977	protein_coding	NM_001211
ASHGV40043603	-2.75448	2.754482879	protein_coding	NM_145740
ASHGV40033285	-2.74136	2.741357733	protein_coding	NM_014246
ASHGV40054951	2.740211	2.740210987	protein_coding	NM_002778
ASHGV40010558	2.738658	2.738658005	protein_coding	NM_018711
ASHGV40057580	2.731306	2.731306398	protein_coding	ENST00000361624
ASHGV40021462	2.73047	2.73047017	protein_coding	NM_001190452
ASHGV40014877	2.727919	2.727918712	protein_coding	NM_004274
ASHGV40032812	-2.72569	2.725691135	protein_coding	NM_001136213
ASHGV40023377	-2.72396	2.723956461	protein_coding	NM_006033
ASHGV40020500	-2.72352	2.723516192	protein_coding	NM_002146
ASHGV40046299	-2.72225	2.72225166	protein_coding	NM_019102
ASHGV40006788	-2.71585	2.715846427	protein_coding	NM_031217
ASHGV40024114	-2.71261	2.712608023	protein_coding	NM_004720
ASHGV40033448	2.710346	2.71034625	protein_coding	NM_144704

ASHGV40041754	-2.70865	2.708653417	protein_coding	NM_001008397
ASHGV40048665	2.698445	2.698444901	protein_coding	NM_153236
ASHGV40011280	2.696517	2.69651691	protein_coding	NM_004980
ASHGV40020239	2.694199	2.694199002	protein_coding	NM_001130677
ASHGV40018018	-2.6924	2.692397128	protein_coding	NM_001308963
ASHGV40006076	-2.68994	2.689937495	protein_coding	ENST00000370449
ASHGV40014699	-2.68823	2.688234386	protein_coding	NM_001005356
ASHGV40006003	-2.68774	2.687735134	protein_coding	NM_001127182
ASHGV40039559	2.68556	2.685560427	protein_coding	NM_000826
ASHGV40046688	2.68484	2.684839872	protein_coding	NM_012479
ASHGV40045117	-2.67895	2.67894627	protein_coding	NM_005588
ASHGV40030460	-2.67878	2.678783113	protein_coding	NM_019609
ASHGV40037208	2.676167	2.676166652	protein_coding	NM_001130084
ASHGV40010176	2.671692	2.671692475	protein_coding	NM_007191
ASHGV40016115	2.664826	2.664825918	protein_coding	NM_012428
ASHGV40040867	-2.66371	2.663711909	protein_coding	NM_001790
ASHGV40042810	2.660476	2.66047604	protein_coding	ENST00000502515
ASHGV40050072	2.657332	2.657332436	protein_coding	NM_177457
ASHGV40046295	-2.65692	2.656916618	protein_coding	NM_006735
ASHGV40023959	-2.65053	2.650525431	protein_coding	NM_001105578
ASHGV40011896	2.647741	2.647741346	protein_coding	NM_206820
ASHGV40005084	-2.64241	2.642412537	protein_coding	NM_002417
ASHGV40020568	2.642244	2.642243988	protein_coding	NM_020178
ASHGV40006475	-2.63477	2.634767493	protein_coding	NM_031264
ASHGV40025572	2.632704	2.632703547	protein_coding	NM_002361
ASHGV40053700	2.63239	2.632390446	protein_coding	ENST00000361899
ASHGV40055230	2.629427	2.629427434	protein_coding	NM_001080425
ASHGV40057449	-2.62609	2.626086996	protein_coding	NM_152414
ASHGV40057659	-2.6229	2.622903348	protein_coding	NM_153488
ASHGV40046294	-2.62253	2.622526805	protein_coding	NM_005522
ASHGV40038155	-2.61945	2.619449868	protein_coding	NM_000508
ASHGV40027853	2.616608	2.616608237	protein_coding	NM_003151
ASHGV40017711	2.614011	2.614010568	protein_coding	NM_001428
ASHGV40003291	2.604595	2.604594845	protein_coding	NM_018476
ASHGV40034809	-2.60354	2.6035388	protein_coding	NM_199420
ASHGV40027422	-2.60251	2.602506227	protein_coding	NM_001277406
ASHGV40024589	-2.60167	2.601671106	protein_coding	NM_003167
ASHGV40018662	2.599471	2.599470548	protein_coding	NM_018723
ASHGV40049057	-2.59827	2.598265921	protein_coding	NM_018492
ASHGV40047669	2.596967	2.596967154	protein_coding	NM_152793
ASHGV40043604	-2.59678	2.596779192	protein_coding	NM_153699
ASHGV40006728	2.593846	2.593845975	protein_coding	NM_006906
ASHGV40016579	-2.59377	2.593765412	protein_coding	NM_001039841
ASHGV40047080	2.590605	2.59060481	protein_coding	NM_005302
ASHGV40027470	-2.59058	2.590582773	protein_coding	NM_002299
ASHGV40043605	-2.59046	2.590457399	protein_coding	NM_000847
ASHGV40040722	-2.58513	2.585131188	protein_coding	NM_002317

ASHGV40021733	2.582957	2.582957335	protein_coding	NM_033133
ASHGV40007795	2.581707	2.58170661	protein_coding	NM_005103
ASHGV40024608	2.577428	2.577427636	protein_coding	NM_001217
ASHGV40015483	-2.5763	2.576302015	protein_coding	NM_001362
ASHGV40007540	-2.57352	2.573515381	protein_coding	NM_002421
ASHGV40044986	2.572362	2.572361863	protein_coding	ENST00000423336
ASHGV40035479	2.572332	2.572331952	protein_coding	NM_005781
ASHGV40028215	2.571177	2.571177265	protein_coding	NM_019850
ASHGV40038156	-2.5702	2.570201441	protein_coding	NM_021870
ASHGV40014471	2.566982	2.566981674	protein_coding	NM_014216
ASHGV40029498	2.564346	2.564345688	protein_coding	NM_032144
ASHGV40046956	2.562192	2.562192425	protein_coding	NM_001037132
ASHGV40051059	2.561727	2.561727364	protein_coding	NM_001198532
ASHGV40054550	-2.56098	2.560984954	protein_coding	NM_005367
ASHGV40026953	2.558223	2.558223113	protein_coding	NM_001015055
ASHGV40045207	2.558167	2.558167402	protein_coding	NM_152731
ASHGV40009655	2.557409	2.557408879	protein_coding	NM_032918
ASHGV40041515	2.553582	2.553581676	protein_coding	NM_001677
ASHGV40031265	2.552507	2.552507287	protein_coding	NM_000311
ASHGV40040202	2.551833	2.551833468	protein_coding	NM_021072
ASHGV40037273	2.548845	2.548845192	protein_coding	NM_000320
ASHGV40034224	2.546042	2.546041767	protein_coding	NM_000729
ASHGV40048474	-2.54498	2.544975759	protein_coding	NM_001869
ASHGV40007211	2.543347	2.543346892	protein_coding	NM_003793
ASHGV40056153	-2.54313	2.54313186	protein_coding	uc021sfx.1
ASHGV40047719	2.5423	2.542300379	protein_coding	NM_018685
ASHGV40012893	2.542259	2.542258711	protein_coding	NM_052867
ASHGV40043996	2.541404	2.541404067	protein_coding	NM_003309
ASHGV40007446	-2.53934	2.539338223	protein_coding	NM_022337
ASHGV40046164	2.538692	2.538691654	protein_coding	NM_002489
ASHGV40051560	2.536427	2.536427025	protein_coding	NM_020344
ASHGV40005593	-2.53506	2.535056758	protein_coding	NM_014696
ASHGV40053701	2.533002	2.533001769	protein_coding	ENST00000362079
ASHGV40019483	-2.53187	2.531872192	protein_coding	NM_002153
ASHGV40015992	-2.5317	2.531700826	protein_coding	NM_014736
ASHGV40043411	2.530646	2.530646136	protein_coding	NM_020939
ASHGV40028075	-2.52667	2.52667333	protein_coding	NM_002181
ASHGV40016526	2.525687	2.525686711	protein_coding	uc010ayj.3
ASHGV40042368	-2.52394	2.523941216	protein_coding	NM_005733
ASHGV40037888	-2.52305	2.523047017	protein_coding	NM_024019
ASHGV40034987	-2.52248	2.522482835	protein_coding	NM_004164
ASHGV40015869	2.520432	2.520432299	protein_coding	NM_153374
ASHGV40046565	2.517466	2.517465513	protein_coding	NM_016139
ASHGV40015127	2.51671	2.516710364	protein_coding	NM_020715
ASHGV40032316	-2.51317	2.513168245	protein_coding	NM_003225
ASHGV40049021	-2.51197	2.511965133	protein_coding	NM_003155
ASHGV40000100	2.51133	2.511330003	protein_coding	NM_003097

ASHGV40055646	2.506496	2.506496058	protein_coding	NM_004653
ASHGV40053704	2.504845	2.504845084	protein_coding	ENST00000361567
ASHGV40043133	-2.50117	2.50117437	protein_coding	NM_003537
ASHGV40040837	-2.49806	2.498056044	protein_coding	NM_006161
ASHGV40034826	2.497703	2.497703035	protein_coding	NM_053025
ASHGV40044384	2.491995	2.491995019	protein_coding	NM_175922
ASHGV40031298	2.486899	2.486898586	protein_coding	NM_015192
ASHGV40029879	2.486606	2.486606228	protein_coding	NM_177454
ASHGV40035472	2.486093	2.486093092	protein_coding	NM_001647
ASHGV40024857	2.485778	2.485778077	protein_coding	NM_006210
ASHGV40039591	-2.48467	2.48466867	protein_coding	NM_001297550
ASHGV40022700	2.478991	2.478991105	protein_coding	NM_001004734
ASHGV40031662	2.475112	2.475111982	protein_coding	NM_003404
ASHGV40049885	-2.47444	2.474442833	protein_coding	NM_004306
ASHGV40009160	2.472679	2.472679485	protein_coding	NM_006006
ASHGV40040310	-2.47143	2.471431195	protein_coding	NM_022145
ASHGV40054598	2.468494	2.468493817	protein_coding	NM_000132
ASHGV40040286	-2.46799	2.467986341	protein_coding	NM_018369
ASHGV40056362	2.466685	2.46668458	protein_coding	NM_002544
ASHGV40043495	2.464624	2.464624382	protein_coding	NM_000322
ASHGV40057666	-2.46405	2.464052226	protein_coding	NM_001256577
ASHGV40031047	2.462804	2.462804376	protein_coding	NM_080617
ASHGV40030856	2.459631	2.459630595	protein_coding	NM_007050
ASHGV40020989	-2.45839	2.458387495	protein_coding	uc001htg.2
ASHGV40030874	2.451294	2.451294024	protein_coding	NM_006811
ASHGV40038348	2.44958	2.449579873	protein_coding	NM_153343
ASHGV40043551	2.449263	2.449263416	protein_coding	NM_005822
ASHGV40040608	-2.44251	2.442514711	protein_coding	NM_173488
ASHGV40018944	2.441881	2.441881376	protein_coding	NM_014712
ASHGV40057661	-2.43671	2.436709593	protein_coding	NM_004988
ASHGV40031639	-2.43613	2.436125395	protein_coding	NM_002466
ASHGV40008370	-2.43323	2.433233726	protein_coding	NM_002391
ASHGV40056864	-2.43225	2.432245347	protein_coding	ENST00000215956
ASHGV40043999	-2.42943	2.429431333	protein_coding	NM_205860
ASHGV40011461	-2.42629	2.42629161	protein_coding	NM_006897
ASHGV40041935	-2.42327	2.423271868	protein_coding	NM_001285460
ASHGV40048838	-2.42075	2.420749105	protein_coding	NM_005218
ASHGV40020199	-2.42035	2.420346096	protein_coding	NM_145654
ASHGV40053702	2.416903	2.416902969	protein_coding	ENST00000361335
ASHGV40032928	2.416748	2.416748438	protein_coding	NM_213720
ASHGV40045270	-2.41596	2.415956024	protein_coding	NM_000716
ASHGV40015816	2.412479	2.412479428	protein_coding	NM_001482
ASHGV40028858	2.410658	2.410657909	protein_coding	NM_138448
ASHGV40041744	2.409737	2.409736809	protein_coding	NM_006308
ASHGV40020579	2.409033	2.409032618	protein_coding	NM_013328
ASHGV40020326	-2.40781	2.407812149	protein_coding	NM_002275
ASHGV40026066	-2.4074	2.407404285	protein_coding	NM_133180

ASHGV40034233	2.404081	2.40408108	protein_coding	NM_014056
ASHGV40018387	2.403793	2.40379347	protein_coding	NM_004078
ASHGV40028920	2.402764	2.402764148	protein_coding	NM_006759
ASHGV40033592	2.402096	2.402095969	protein_coding	NM_021076
ASHGV40055231	2.40209	2.402089893	protein_coding	NM_152278
ASHGV40019915	2.401546	2.401546145	protein_coding	NM_000304
ASHGV40053697	2.399625	2.399625379	protein_coding	ENST00000361390
ASHGV40007752	2.399319	2.399319357	protein_coding	NM_006597
ASHGV40009084	2.397568	2.397567543	protein_coding	NM_001077244
ASHGV40010650	2.394848	2.394847595	protein_coding	NM_017899
ASHGV40043142	-2.39484	2.394839642	protein_coding	NM_003547
ASHGV40057181	-2.39415	2.394154877	protein_coding	NM_021065
ASHGV40013831	-2.39052	2.390519934	protein_coding	NM_032876
ASHGV40022442	2.389606	2.389605945	protein_coding	NM_012307
ASHGV40028265	-2.38932	2.389320258	protein_coding	NM_004369
ASHGV40011704	2.386835	2.38683454	protein_coding	NM_001136262
ASHGV40009133	2.385363	2.385362527	protein_coding	NM_024686
ASHGV40054306	-2.38339	2.383392111	protein_coding	NM_001847
ASHGV40051411	2.383311	2.383310687	protein_coding	NM_005028
ASHGV40044734	-2.38028	2.380284119	protein_coding	NM_003512
ASHGV40040794	2.379022	2.379021779	protein_coding	NM_015256
ASHGV40003271	-2.37691	2.376909057	protein_coding	NM_005634
ASHGV40007726	2.375493	2.375492865	protein_coding	NM_004205
ASHGV40021173	2.375045	2.375044575	protein_coding	NM_000029
ASHGV40027211	2.373584	2.37358393	protein_coding	NM_001450
ASHGV40027384	-2.37248	2.372477322	protein_coding	NM_001001665
ASHGV40034833	-2.37122	2.371223838	protein_coding	NM_033049
ASHGV40010186	-2.37058	2.370581712	protein_coding	ENST00000356215
ASHGV40043766	2.366862	2.366862345	protein_coding	NM_002395
ASHGV40030673	2.366153	2.36615283	protein_coding	NM_000099
ASHGV40039567	2.3651	2.36509982	protein_coding	NM_018342
ASHGV40012356	2.363066	2.363066442	protein_coding	NM_001123068
ASHGV40007677	-2.36304	2.36304018	protein_coding	NM_000482
ASHGV40024344	2.362262	2.362262226	protein_coding	NM_033256
ASHGV40015306	2.360582	2.360582184	protein_coding	NM_006888
ASHGV40049428	-2.35926	2.359259194	protein_coding	NM_020361
ASHGV40035145	-2.35899	2.358991513	protein_coding	NM_024621
ASHGV40008719	2.358804	2.358803539	protein_coding	NM_145200
ASHGV40038896	-2.35715	2.357152136	protein_coding	NM_133267
ASHGV40034730	-2.35537	2.355372367	protein_coding	NM_018189
ASHGV40056639	-2.35532	2.355323904	protein_coding	NM_178429
ASHGV40011391	2.35379	2.353790067	protein_coding	ENST00000599343
ASHGV40007225	-2.35304	2.353042591	protein_coding	NM_004153
ASHGV40012454	2.352162	2.352162024	protein_coding	NM_004734
ASHGV40054145	2.351502	2.351501868	protein_coding	NM_020987
ASHGV40018828	2.350533	2.350532639	protein_coding	NM_212535
ASHGV40049199	2.347191	2.347191183	protein_coding	NM_024645

ASHGV40045850	2.344612	2.344612325	protein_coding	NM_003381
ASHGV40053088	-2.34455	2.344551095	protein_coding	ENST00000314355
ASHGV40020256	2.342712	2.342712304	protein_coding	NM_000723
ASHGV40002018	2.339596	2.339596431	protein_coding	NM_012306
ASHGV40011536	2.339396	2.339396393	protein_coding	ENST00000322165
ASHGV40006731	-2.33856	2.338555813	protein_coding	NM_024680
ASHGV40045002	2.337357	2.337357347	protein_coding	NM_181644
ASHGV40014919	-2.33731	2.337308296	protein_coding	NM_054024
ASHGV40051842	2.33296	2.332959785	protein_coding	NM_000689
ASHGV40027321	-2.33061	2.330611997	protein_coding	NM_001426
ASHGV40057632	2.328554	2.328554078	protein_coding	NM_001012979
ASHGV40026736	2.325296	2.325296028	protein_coding	NM_004801
ASHGV40043174	-2.32114	2.321142992	protein_coding	NM_003535
ASHGV40022600	2.320148	2.320148142	protein_coding	NM_080597
ASHGV40054596	2.31972	2.319720453	protein_coding	NM_002436
ASHGV40019923	-2.31947	2.319466367	protein_coding	NM_001204477
ASHGV40017339	2.318856	2.318855913	protein_coding	NM_002065
ASHGV40010969	2.316966	2.316966021	protein_coding	NM_002046
ASHGV40033010	2.316518	2.316518469	protein_coding	NM_006477
ASHGV40051050	2.314087	2.314086908	protein_coding	NM_014677
ASHGV40002698	-2.31209	2.312086676	protein_coding	ENST00000602042
ASHGV40014284	2.311901	2.311900656	protein_coding	NM_006029
ASHGV40009594	2.311805	2.311804857	protein_coding	NM_018423
ASHGV40016019	2.309952	2.309951554	protein_coding	NM_003617
ASHGV40029799	-2.30957	2.30956726	protein_coding	NM_014213
ASHGV40029873	-2.30799	2.307991291	protein_coding	NM_173651
ASHGV40000128	2.307245	2.307245098	protein_coding	NM_014624
ASHGV40024598	2.30537	2.305370095	protein_coding	NM_003636
ASHGV40039570	2.304793	2.30479291	protein_coding	NM_021634
ASHGV40048143	-2.30157	2.301574943	protein_coding	NM_021955
ASHGV40038684	-2.3013	2.301299001	protein_coding	NM_020973
ASHGV40024542	-2.30022	2.300218781	protein_coding	NM_001029861
ASHGV40048698	2.299423	2.299422927	protein_coding	NM_145292
ASHGV40022631	-2.29872	2.298721897	protein_coding	NM_001941
ASHGV40009688	2.297781	2.297780724	protein_coding	NM_002300
ASHGV40050277	-2.29632	2.296318465	protein_coding	NM_001033017
ASHGV40032106	2.294532	2.294531659	protein_coding	NM_000484
ASHGV40011888	-2.29361	2.293612616	protein_coding	NM_174942
ASHGV40039255	-2.29277	2.292771339	protein_coding	NM_000995
ASHGV40047918	2.292573	2.292573397	protein_coding	NM_002245
ASHGV40033822	2.289531	2.289530929	protein_coding	NM_001103161
ASHGV40012503	2.289033	2.289033036	protein_coding	NM_015058
ASHGV40057195	-2.28765	2.287650136	protein_coding	NM_003509
ASHGV40016725	2.287364	2.287364479	protein_coding	NM_002373
ASHGV40035553	2.286709	2.286708932	protein_coding	NM_002222
ASHGV40046891	-2.2857	2.285696837	protein_coding	NM_138403
ASHGV40024389	2.2835	2.283499954	protein_coding	NM_152479

ASHGV40054027	-2.28237	2.282374334	protein_coding	NM_130776
ASHGV40049233	2.281615	2.281614723	protein_coding	NM_006749
ASHGV40028900	2.281459	2.281459215	protein_coding	NM_001143959
ASHGV40042960	2.28103	2.281029571	protein_coding	NM_016588
ASHGV40049644	-2.27938	2.279378171	protein_coding	NM_004063
ASHGV40052174	2.279142	2.27914212	protein_coding	NM_001401
ASHGV40054624	-2.27815	2.278153077	protein_coding	NM_175569
ASHGV40035392	2.277831	2.277830785	protein_coding	NM_001048
ASHGV40028327	2.277384	2.277384275	protein_coding	NM_004321
ASHGV40043144	-2.27531	2.275314291	protein_coding	NM_003534
ASHGV40045693	-2.27369	2.273693799	protein_coding	NM_003206
ASHGV40046446	2.272168	2.272168297	protein_coding	NM_001220
ASHGV40037146	2.271051	2.271051457	protein_coding	NM_020972
ASHGV40046305	-2.26931	2.269308683	protein_coding	NM_005523
ASHGV40024670	2.26646	2.266460335	protein_coding	NM_001080457
ASHGV40016118	2.266381	2.266381323	protein_coding	NM_001039614
ASHGV40005903	2.266274	2.266273624	protein_coding	NM_014394
ASHGV40020257	2.265395	2.265394836	protein_coding	NM_198993
ASHGV40009328	-2.26206	2.262057846	protein_coding	NM_001274
ASHGV40044041	2.262025	2.262025492	protein_coding	NM_020755
ASHGV40001397	2.261907	2.261906765	protein_coding	NM_014059
ASHGV40024558	2.26102	2.261020083	protein_coding	NM_020709
ASHGV40007362	2.260421	2.260421247	protein_coding	NM_033063
ASHGV40041217	2.259714	2.259714252	protein_coding	NM_003085
ASHGV40054132	-2.25712	2.257118166	protein_coding	NM_017669
ASHGV40021180	2.252814	2.252814117	protein_coding	NM_012168
ASHGV40012425	2.252579	2.252579252	protein_coding	NM_001143883
ASHGV40035987	2.252323	2.252323141	protein_coding	NM_080865
ASHGV40020243	2.251929	2.251929445	protein_coding	NM_003559
ASHGV40009830	2.25142	2.251420412	protein_coding	NM_052885
ASHGV40039052	-2.25091	2.250912077	protein_coding	NM_002620
ASHGV40046297	-2.24975	2.249745569	protein_coding	NM_030661
ASHGV40051042	2.249665	2.249665184	protein_coding	NM_024812
ASHGV40010370	2.247418	2.247418023	protein_coding	NM_001682
ASHGV40029708	2.247321	2.247320512	protein_coding	NM_001178015
ASHGV40044765	-2.24706	2.247064685	protein_coding	NM_080596
ASHGV40056662	2.246797	2.246796954	protein_coding	NM_006271
ASHGV40002226	2.246472	2.246472113	protein_coding	NM_006829
ASHGV40000228	-2.24623	2.246227843	protein_coding	ENST00000399979
ASHGV40015110	2.245658	2.245658354	protein_coding	NM_001308147
ASHGV40031861	2.245563	2.245563344	protein_coding	NM_000516
ASHGV40038355	-2.2438	2.243801785	protein_coding	uc003iwr.1
ASHGV40025887	2.242952	2.24295177	protein_coding	NM_000146
ASHGV40003255	-2.24278	2.242780032	protein_coding	NM_003539
ASHGV40024357	-2.24188	2.241881557	protein_coding	NM_006149
ASHGV40057547	2.241875	2.241874817	protein_coding	NM_017594
ASHGV40007044	-2.24057	2.240574112	protein_coding	NM_032110

ASHGV40046951	-2.24057	2.240569757	protein_coding	NM_000111
ASHGV40041266	-2.23911	2.239107889	protein_coding	NM_014244
ASHGV40039598	2.236639	2.236638743	protein_coding	NM_001873
ASHGV40044742	-2.23648	2.236480056	protein_coding	NM_003524
ASHGV40041224	-2.23608	2.236081576	protein_coding	NM_031300
ASHGV40026774	2.233694	2.233693542	protein_coding	NM_001039348
ASHGV40057177	-2.23218	2.232175588	protein_coding	NM_005325
ASHGV40019505	-2.23209	2.23209032	protein_coding	NM_002497
ASHGV40054516	2.230487	2.230486584	protein_coding	NM_013266
ASHGV40008374	-2.22913	2.229127986	protein_coding	NM_000506
ASHGV40050395	-2.22883	2.228833379	protein_coding	NM_014479
ASHGV40037191	2.228524	2.228523902	protein_coding	NM_020416
ASHGV40012441	2.228031	2.228030557	protein_coding	NM_178006
ASHGV40043365	2.227503	2.227502568	protein_coding	NM_054111
ASHGV40006583	-2.22657	2.226573198	protein_coding	NM_001001922
ASHGV40040312	2.226427	2.226427012	protein_coding	NM_019072
ASHGV40039977	2.226143	2.226142762	protein_coding	NM_001034850
ASHGV40032487	2.224845	2.224844508	protein_coding	NM_004540
ASHGV40030761	-2.22401	2.224010035	protein_coding	NM_005225
ASHGV40025220	2.222956	2.222955619	protein_coding	NM_002229
ASHGV40023054	-2.22283	2.222826198	protein_coding	NM_006101
ASHGV40055353	2.219689	2.219689459	protein_coding	NM_000828
ASHGV40015452	2.219355	2.219354617	protein_coding	uc001yif.2
ASHGV40044133	2.217179	2.217178646	protein_coding	NM_005923
ASHGV40054033	2.21658	2.216579821	protein_coding	NM_015075
ASHGV40040175	2.215543	2.215543247	protein_coding	NM_005410
ASHGV40027603	2.214511	2.214511238	protein_coding	NM_145259
ASHGV40039998	2.214113	2.214113226	protein_coding	NM_004934
ASHGV40017496	2.212849	2.21284917	protein_coding	NM_001089
ASHGV40026446	-2.21243	2.212429851	protein_coding	ENST00000233242
ASHGV40055743	2.212038	2.212037598	protein_coding	NM_001157
ASHGV40024639	2.211524	2.21152388	protein_coding	NM_020309
ASHGV40042010	2.210898	2.210897551	protein_coding	NM_001867
ASHGV40030970	2.207759	2.207759266	protein_coding	NM_004975
ASHGV40006014	-2.20654	2.206537639	protein_coding	NM_001289068
ASHGV40043395	2.205771	2.205770941	protein_coding	NM_003322
ASHGV40026675	2.202731	2.202730804	protein_coding	NM_006036
ASHGV40047631	2.20067	2.200669998	protein_coding	NM_013322
ASHGV40040963	2.200077	2.200076603	protein_coding	NM_181675
ASHGV40016838	2.199693	2.199693092	protein_coding	NM_001080534
ASHGV40053180	2.198533	2.198533334	protein_coding	NM_014282
ASHGV40046957	-2.19734	2.197336885	protein_coding	ENST00000366845
ASHGV40035536	2.197188	2.197187954	protein_coding	NM_001253387
ASHGV40023732	2.1969	2.196899687	protein_coding	NM_139355
ASHGV40005366	2.195214	2.195214013	protein_coding	NM_001145195
ASHGV40040729	-2.19362	2.193618942	protein_coding	ENST00000594609
ASHGV40016252	2.193444	2.193444247	protein_coding	NM_030594

ASHGV40054594	-2.19301	2.193008555	protein_coding	NM_172377
ASHGV40056635	-2.19227	2.192273978	protein_coding	NM_032563
ASHGV40028221	-2.191	2.191003053	protein_coding	NM_018410
ASHGV40024602	2.190681	2.190680957	protein_coding	NM_001080434
ASHGV40046856	-2.19045	2.190446974	protein_coding	ENST00000292377
ASHGV40028307	2.188998	2.188998176	protein_coding	NM_001163424
ASHGV40033459	2.188562	2.188561814	protein_coding	NM_182948
ASHGV40009536	-2.18316	2.183155545	protein_coding	NM_020634
ASHGV40038142	2.182532	2.182532397	protein_coding	NM_033632
ASHGV40015881	2.182289	2.182288898	protein_coding	NM_006628
ASHGV40014344	2.181901	2.181900763	protein_coding	NM_021257
ASHGV40040373	2.181224	2.18122411	protein_coding	NM_003768
ASHGV40006465	2.18046	2.180460392	protein_coding	NM_002939
ASHGV40046566	2.179724	2.179723676	protein_coding	NM_001145712
ASHGV40009425	2.17778	2.177780304	protein_coding	NM_016533
ASHGV40040806	2.176922	2.176921845	protein_coding	NM_007054
ASHGV40009503	2.176777	2.17677741	protein_coding	NM_014231
ASHGV40010392	-2.17437	2.174372441	protein_coding	NM_001731
ASHGV40050949	2.173954	2.173954248	protein_coding	NM_018444
ASHGV40040353	-2.1735	2.173497311	protein_coding	NM_001515
ASHGV40007786	2.172073	2.172073311	protein_coding	NM_003713
ASHGV40028008	2.172037	2.172037012	protein_coding	NM_006055
ASHGV40040434	2.168458	2.168458129	protein_coding	NM_004272
ASHGV40010227	2.16838	2.168380494	protein_coding	NM_001874
ASHGV40035513	2.168025	2.168025287	protein_coding	NM_004051
ASHGV40039860	-2.16802	2.168020345	protein_coding	NM_033267
ASHGV40041620	2.167308	2.16730825	protein_coding	NM_004172
ASHGV40014481	-2.1663	2.166299027	protein_coding	NM_001756
ASHGV40016197	-2.16319	2.163188259	protein_coding	NM_000743
ASHGV40014293	2.163177	2.163176756	protein_coding	NM_005589
ASHGV40055008	-2.16311	2.163105869	protein_coding	NM_001015038
ASHGV40035658	2.162644	2.162644234	protein_coding	NM_000060
ASHGV40017611	2.159822	2.15982155	protein_coding	NM_014015
ASHGV40055098	-2.15966	2.159656922	protein_coding	NM_032562
ASHGV40035190	-2.15844	2.158437868	protein_coding	NM_001041
ASHGV40028428	2.158296	2.158296488	protein_coding	ENST00000391666
ASHGV40036198	2.157983	2.157982935	protein_coding	NM_001167674
ASHGV40054602	2.157128	2.157128411	protein_coding	ENST00000369445
ASHGV40040829	-2.15703	2.157027188	protein_coding	NM_002653
ASHGV40050950	2.156986	2.15698581	protein_coding	NM_001010908
ASHGV40010939	-2.15519	2.155185785	protein_coding	NM_017417
ASHGV40010489	-2.15482	2.154819157	protein_coding	NM_002674
ASHGV40000691	2.15451	2.15450961	protein_coding	NM_001540
ASHGV40010764	2.152841	2.152841446	protein_coding	NM_021009
ASHGV40032636	2.149801	2.149800622	protein_coding	NM_001757
ASHGV40041052	-2.1489	2.148895612	protein_coding	NM_012206
ASHGV40055805	2.148675	2.148674509	protein_coding	NM_003956

ASHGV40042279	2.146859	2.146859177	protein_coding	NM_001046
ASHGV40054441	-2.14547	2.145465872	protein_coding	NM_004484
ASHGV40036903	2.145404	2.145403804	protein_coding	NM_004068
ASHGV40054487	-2.14419	2.144186215	protein_coding	NM_004065
ASHGV40050541	2.143925	2.143924962	protein_coding	NM_006283
ASHGV40035506	2.142021	2.142021291	protein_coding	NM_006621
ASHGV40043130	-2.14176	2.141764764	protein_coding	NM_005835
ASHGV40051252	2.141501	2.141501348	protein_coding	NM_015137
ASHGV40030697	2.139851	2.139851316	protein_coding	NM_020531
ASHGV40019429	2.139473	2.139472807	protein_coding	NM_172362
ASHGV40057655	-2.13916	2.139161054	protein_coding	NM_002362
ASHGV40012427	2.139042	2.139042041	protein_coding	NM_006644
ASHGV40012677	2.136659	2.136659493	protein_coding	NM_203487
ASHGV40006086	-2.1358	2.135804388	protein_coding	NM_003393
ASHGV40020840	2.135432	2.135431973	protein_coding	NM_000835
ASHGV40027050	-2.1354	2.13540276	protein_coding	NM_001443
ASHGV40016200	-2.13514	2.135136504	protein_coding	NM_014272
ASHGV40051038	2.133239	2.13323881	protein_coding	NM_001695
ASHGV40034729	-2.13135	2.131348311	protein_coding	NM_138815
ASHGV40048767	-2.13058	2.130581863	protein_coding	ENST00000444154
ASHGV40033115	2.129481	2.129480995	protein_coding	NM_006078
ASHGV40055393	2.128764	2.12876397	protein_coding	NM_001222
ASHGV40024362	2.127491	2.127491419	protein_coding	NM_012237
ASHGV40057212	-2.12621	2.126207222	protein_coding	ENST00000503322
ASHGV40026757	2.123697	2.123696577	protein_coding	NM_020532
ASHGV40049854	-2.12366	2.123662233	protein_coding	NM_005328
ASHGV40009285	-2.12338	2.123379017	protein_coding	NM_018298
ASHGV40035765	-2.12252	2.122518076	protein_coding	NM_001039111
ASHGV40027991	-2.12204	2.122040413	protein_coding	NM_006891
ASHGV40028830	-2.1208	2.120801469	protein_coding	NM_002354
ASHGV40016601	2.120656	2.120656457	protein_coding	NM_001144757
ASHGV40009985	2.119842	2.119841567	protein_coding	NM_000424
ASHGV40055775	2.117277	2.117276687	protein_coding	NM_017551
ASHGV40046838	-2.11641	2.116413441	protein_coding	NM_000777
ASHGV40023892	2.116404	2.116403658	protein_coding	NM_001800
ASHGV40007727	2.116259	2.116259046	protein_coding	NM_006288
ASHGV40055908	2.116057	2.116057233	protein_coding	NM_005004
ASHGV40050997	-2.11258	2.112577377	protein_coding	NM_001142462
ASHGV40022757	-2.11056	2.110560546	protein_coding	NM_001037802
ASHGV40032411	2.108745	2.108745073	protein_coding	NM_001142854
ASHGV40002664	-2.1083	2.108299173	protein_coding	ENST00000598764
ASHGV40017824	2.107368	2.107367821	protein_coding	NM_201575
ASHGV40011258	2.105543	2.105542626	protein_coding	NM_198578
ASHGV40032821	2.105495	2.105494797	protein_coding	NM_001696
ASHGV40040309	-2.10287	2.102866944	protein_coding	NM_197941
ASHGV40020933	-2.10179	2.101785282	protein_coding	NM_003258
ASHGV40033761	2.101733	2.101733374	protein_coding	NM_015705

ASHGV40042930	2.097702	2.097702135	protein_coding	NM_001069
ASHGV40040138	2.096513	2.096513196	protein_coding	NM_002310
ASHGV40020955	2.096506	2.096506235	protein_coding	NM_001082575
ASHGV40045197	-2.09586	2.095861935	protein_coding	NM_001010872
ASHGV40015888	-2.09556	2.095563351	protein_coding	NM_182758
ASHGV40042163	2.095043	2.095042688	protein_coding	NM_000038
ASHGV40009235	-2.0949	2.094899896	protein_coding	NM_024791
ASHGV40043302	2.094869	2.094869379	protein_coding	NM_030651
ASHGV40009829	2.094702	2.094701599	protein_coding	NM_005164
ASHGV40006867	2.09255	2.092549675	protein_coding	NM_020929
ASHGV40053041	2.091909	2.091909292	protein_coding	NM_001018064
ASHGV40040290	-2.0916	2.091603606	protein_coding	ENST00000594278
ASHGV40021202	2.089077	2.08907698	protein_coding	NM_174893
ASHGV40036427	2.088448	2.088447896	protein_coding	NM_014980
ASHGV40016123	2.085647	2.085646897	protein_coding	NM_004809
ASHGV40057191	-2.08484	2.084842028	protein_coding	NM_021064
ASHGV40041720	-2.0835	2.083502304	protein_coding	NM_002202
ASHGV40013715	-2.08349	2.083488786	protein_coding	NM_005986
ASHGV40035094	-2.08285	2.08285322	protein_coding	NM_023915
ASHGV40000108	2.082748	2.082748142	protein_coding	NM_018694
ASHGV40012544	-2.08124	2.081237068	protein_coding	NM_001872
ASHGV40056000	2.079643	2.079642698	protein_coding	NM_014365
ASHGV40043132	-2.07957	2.07956658	protein_coding	NM_003544
ASHGV40014681	2.07936	2.079360038	protein_coding	NM_181864
ASHGV40009828	2.079034	2.079034291	protein_coding	NM_001173464
ASHGV40016009	-2.07811	2.078107308	protein_coding	NM_003613
ASHGV40032687	2.076533	2.076532605	protein_coding	NM_006198
ASHGV40042795	-2.07435	2.074352633	protein_coding	NM_002011
ASHGV40057711	-2.07359	2.07359413	protein_coding	NM_024747
ASHGV40000185	-2.07185	2.071852397	protein_coding	NM_000612
ASHGV40012552	2.071636	2.071636412	protein_coding	NM_000621
ASHGV40030630	2.071457	2.071456707	protein_coding	NM_006004
ASHGV40051257	2.070868	2.070867844	protein_coding	NM_006393
ASHGV40040913	-2.06994	2.069942723	protein_coding	ENST00000502505
ASHGV40052557	2.068775	2.068774804	protein_coding	NM_001190489
ASHGV40011462	2.068279	2.068278583	protein_coding	NM_022658
ASHGV40018138	2.067889	2.067888748	protein_coding	NM_006141
ASHGV40022056	-2.06724	2.067235327	protein_coding	NM_005994
ASHGV40046304	-2.06641	2.066411357	protein_coding	NM_018951
ASHGV40030663	2.065613	2.065613109	protein_coding	NM_022080
ASHGV40024675	-2.06555	2.065547763	protein_coding	NM_002257
ASHGV40008388	2.064804	2.064803995	protein_coding	NM_004551
ASHGV40040877	-2.06379	2.06378979	protein_coding	NM_005847
ASHGV40036545	-2.06348	2.06348233	protein_coding	NM_003571
ASHGV40029625	2.063166	2.063166373	protein_coding	NM_004522
ASHGV40039064	-2.06277	2.062765136	protein_coding	NM_001657
ASHGV40036942	2.062736	2.062735836	protein_coding	NM_001967

ASHGV40016993	-2.06232	2.062324899	protein_coding	NM_138555
ASHGV40009685	2.062286	2.062285655	protein_coding	NM_021094
ASHGV40055225	2.061288	2.061287584	protein_coding	NM_001199818
ASHGV40053794	-2.06027	2.060272043	protein_coding	NM_004469
ASHGV40051961	2.05902	2.059019517	protein_coding	NM_016848
ASHGV40041735	-2.05723	2.057229925	protein_coding	NM_013409
ASHGV40011067	-2.05701	2.057012827	protein_coding	NM_138722
ASHGV40012604	2.05512	2.055119507	protein_coding	NM_002590
ASHGV40036684	-2.05442	2.054416212	protein_coding	NM_001086
ASHGV40009330	-2.05385	2.053849672	protein_coding	NM_001129883
ASHGV40019839	-2.05353	2.053525187	protein_coding	NM_152599
ASHGV40007308	-2.0534	2.053401665	protein_coding	ENST00000343767
ASHGV40053605	2.053192	2.053192141	protein_coding	NM_001282611
ASHGV40023850	-2.05293	2.052927099	protein_coding	NM_001005192
ASHGV40003120	2.052703	2.052702676	protein_coding	NM_001136002
ASHGV40014240	-2.05235	2.052351558	protein_coding	NM_003049
ASHGV40017573	2.052338	2.052337963	protein_coding	NM_016256
ASHGV40000731	-2.05015	2.050148751	protein_coding	NM_001164464
ASHGV40014617	2.048676	2.048676041	protein_coding	NM_001823
ASHGV40041209	2.047199	2.047198897	protein_coding	NM_004528
ASHGV40051488	2.047182	2.047181988	protein_coding	NM_001856
ASHGV40040807	2.045752	2.04575212	protein_coding	NM_001098811
ASHGV40044778	-2.04503	2.045034103	protein_coding	NM_003536
ASHGV40040106	-2.04389	2.043890365	protein_coding	NM_031900
ASHGV40032853	-2.0435	2.043503209	protein_coding	NM_000300
ASHGV40006570	-2.0434	2.043401769	protein_coding	NM_000184
ASHGV40037598	-2.04263	2.0426288	protein_coding	NM_021139
ASHGV40036361	2.041649	2.041649396	protein_coding	NM_013259
ASHGV40009308	2.033992	2.033991747	protein_coding	NM_006176
ASHGV40043143	-2.03151	2.031509051	protein_coding	NM_021018
ASHGV40040564	2.031326	2.031325946	protein_coding	NM_024717
ASHGV40038287	-2.03124	2.03123526	protein_coding	NM_000860
ASHGV40021800	2.030019	2.030018831	protein_coding	NM_001144825
ASHGV40026513	2.029683	2.029683032	protein_coding	NM_032409
ASHGV40037300	2.029303	2.029302774	protein_coding	NM_025221
ASHGV40045852	-2.02919	2.029191395	protein_coding	ENST00000392385
ASHGV40037790	-2.02791	2.027905189	protein_coding	NM_000670
ASHGV40041310	-2.02732	2.027315037	protein_coding	NM_052909
ASHGV40039546	2.026358	2.026358079	protein_coding	NM_000857
ASHGV40048552	2.026219	2.026219132	protein_coding	NM_004546
ASHGV40054753	2.026135	2.026135498	protein_coding	NM_014927
ASHGV40026470	-2.02497	2.024965996	protein_coding	uc021vep.1
ASHGV40027801	2.024669	2.024669314	protein_coding	NM_013436
ASHGV40015800	2.02332	2.023320414	protein_coding	NM_005099
ASHGV40010396	2.022884	2.022884065	protein_coding	NM_003566
ASHGV40039095	-2.02226	2.022262515	protein_coding	NM_006419
ASHGV40001898	-2.02177	2.021766081	protein_coding	ENST00000533341

ASHGV40003191	-2.02172	2.021719722	protein_coding	NM_001243237
ASHGV40054787	2.020018	2.020017517	protein_coding	NM_014271
ASHGV40023268	-2.01978	2.019780338	protein_coding	NM_001943
ASHGV40014976	2.019762	2.019761643	protein_coding	NM_015915
ASHGV40035508	2.019355	2.019354666	protein_coding	NM_001098424
ASHGV40027275	-2.01887	2.018868113	protein_coding	NM_152515
ASHGV40020900	2.017902	2.017901915	protein_coding	NM_134268
ASHGV40024099	2.017341	2.017341102	protein_coding	NM_198207
ASHGV40049512	2.016921	2.016920995	protein_coding	NM_001025252
ASHGV40039558	2.016861	2.01686127	protein_coding	NM_000824
ASHGV40006459	-2.01624	2.016238931	protein_coding	NM_001012302
ASHGV40053474	2.015929	2.015929095	protein_coding	NM_001032221
ASHGV40031123	-2.01443	2.014430564	protein_coding	ENST00000317652
ASHGV40032756	2.014297	2.014297207	protein_coding	NM_181688
ASHGV40036429	-2.01399	2.013991509	protein_coding	NM_001012659
ASHGV40027676	-2.01327	2.013269423	protein_coding	NM_020675
ASHGV40025734	-2.01314	2.013139675	protein_coding	uc002orj.1
ASHGV40008118	2.012539	2.012538759	protein_coding	NM_014632
ASHGV40011767	-2.01135	2.011346236	protein_coding	NM_001079910
ASHGV40020388	-2.01067	2.01066835	protein_coding	NM_004527
ASHGV40053390	2.00983	2.009829562	protein_coding	NM_000177
ASHGV40048503	-2.00842	2.008417802	protein_coding	NM_020299
ASHGV40021305	2.008088	2.008087513	protein_coding	NM_003010
ASHGV40021720	-2.00804	2.008040702	protein_coding	NM_033191
ASHGV40020936	2.006955	2.00695454	protein_coding	NM_173628
ASHGV40039609	-2.00644	2.006436755	protein_coding	NM_007193
ASHGV40036479	-2.00458	2.004580468	protein_coding	NM_182628
ASHGV40017563	-2.00456	2.004564034	protein_coding	uc002cwy.3
ASHGV40027861	2.004454	2.004454227	protein_coding	NM_004657
ASHGV40045095	2.002502	2.002501546	protein_coding	NM_007355
ASHGV40024131	2.000355	2.000354597	protein_coding	NM_002744
ASHGV40005704	2.000274	2.000273687	protein_coding	NM_014951
ASHGV40001950	-2.00011	2.000106632	protein_coding	NM_003483
ASHGV40030474	1.998559	1.998559349	protein_coding	NM_015417
ASHGV40017408	1.998135	1.998135445	protein_coding	NM_001077350
ASHGV40035096	1.997846	1.997845642	protein_coding	NM_022788
ASHGV40056036	1.997617	1.997617378	protein_coding	NM_198404
ASHGV40040248	-1.99761	1.997606519	protein_coding	NM_001170402
ASHGV40054992	1.997563	1.997563266	protein_coding	NM_022117
ASHGV40025833	1.997088	1.997088487	protein_coding	NM_005184
ASHGV40014734	-1.99671	1.996705958	protein_coding	NM_014579
ASHGV40041851	1.996066	1.996065844	protein_coding	NM_181523
ASHGV40018078	1.995972	1.995971602	protein_coding	NM_002080
ASHGV40019985	-1.99487	1.994867564	protein_coding	NM_001039999
ASHGV40013987	1.99459	1.994589965	protein_coding	NM_175060
ASHGV40010853	1.994536	1.994535633	protein_coding	NM_021808
ASHGV40053970	1.993772	1.993772484	protein_coding	NM_006950

ASHGV40054164	1.99326	1.993260464	protein_coding	NM_001029891
ASHGV40026217	-1.99301	1.993010854	protein_coding	NM_012293
ASHGV40027907	1.992725	1.992725166	protein_coding	uc021vup.1
ASHGV40046449	-1.99233	1.992331923	protein_coding	NM_013389
ASHGV40007202	1.992232	1.992231917	protein_coding	NM_006876
ASHGV40047997	-1.99146	1.99145884	protein_coding	NM_001305
ASHGV40025232	-1.98977	1.989765015	protein_coding	NM_198545
ASHGV40046786	-1.98887	1.988874981	protein_coding	NM_006528
ASHGV40002608	-1.98798	1.987976206	protein_coding	NM_130786
ASHGV40016812	1.987567	1.987566774	protein_coding	NM_013243
ASHGV40044330	-1.98693	1.986928801	protein_coding	NM_004433
ASHGV40015333	1.986909	1.986908877	protein_coding	NM_001275
ASHGV40040820	1.985509	1.985508683	protein_coding	NM_170679
ASHGV40039454	1.984397	1.984396685	protein_coding	NM_022475
ASHGV40001858	-1.98389	1.983890491	protein_coding	NM_006262
ASHGV40017680	1.983179	1.983178913	protein_coding	uc010vai.1
ASHGV40015837	1.982388	1.98238794	protein_coding	NM_001193489
ASHGV40034393	-1.98204	1.98204287	protein_coding	NM_152397
ASHGV40016502	1.980208	1.980208112	protein_coding	NM_014970
ASHGV40035332	-1.97903	1.97903306	protein_coding	NM_014398
ASHGV40055503	-1.9776	1.977599224	protein_coding	NM_005364
ASHGV40057688	1.976406	1.976405851	protein_coding	NM_001089591
ASHGV40051868	1.976029	1.976028977	protein_coding	NM_015225
ASHGV40035705	1.975433	1.975432679	protein_coding	NM_002948
ASHGV40007428	1.975168	1.975167941	protein_coding	NM_001364
ASHGV40056638	-1.97508	1.975084389	protein_coding	NM_178430
ASHGV40005043	1.974592	1.974592499	protein_coding	NM_000274
ASHGV40005399	-1.97384	1.973843857	protein_coding	NM_173081
ASHGV40032816	-1.97382	1.973817112	protein_coding	NM_001037814
ASHGV40025119	-1.97357	1.973570924	protein_coding	NM_005624
ASHGV40042385	1.973247	1.973247224	protein_coding	NM_018834
ASHGV40031476	-1.97225	1.972250971	protein_coding	NM_012112
ASHGV40019962	1.971823	1.971823013	protein_coding	NM_004176
ASHGV40040119	1.970877	1.970877385	protein_coding	NM_145000
ASHGV40052485	1.970417	1.970416669	protein_coding	NM_152833
ASHGV40055562	-1.97018	1.97018359	protein_coding	NM_000054
ASHGV40017360	1.970107	1.970106865	protein_coding	NM_015286
ASHGV40018825	1.968793	1.968792808	protein_coding	NM_005030
ASHGV40040940	1.968473	1.968472518	protein_coding	NM_000176
ASHGV40044614	1.968299	1.968298939	protein_coding	NM_030948
ASHGV40006775	-1.96667	1.966674944	protein_coding	NM_145650
ASHGV40011033	-1.96647	1.966466851	protein_coding	uc001qvo.2
ASHGV40043902	1.965701	1.96570115	protein_coding	NM_022361
ASHGV40026934	1.96507	1.965070053	protein_coding	NM_015470
ASHGV40045665	-1.96497	1.964969232	protein_coding	NM_006208
ASHGV40049112	-1.96361	1.963609434	protein_coding	ENST00000256257
ASHGV40042610	1.963166	1.963165578	protein_coding	NM_001291722

ASHGV40012731	1.963046	1.963046127	protein_coding	NM_015057
ASHGV40034865	1.962836	1.962836281	protein_coding	NM_001003794
ASHGV40006997	-1.96248	1.962483125	protein_coding	NM_001145101
ASHGV40051891	-1.96248	1.962481066	protein_coding	NM_021738
ASHGV40019633	-1.9616	1.961602402	protein_coding	uc002fqm.2
ASHGV40011465	-1.96126	1.961264251	protein_coding	NM_018953
ASHGV40022686	-1.96112	1.961116843	protein_coding	NM_001001824
ASHGV40037651	1.960229	1.960229262	protein_coding	NM_012297
ASHGV40039201	-1.95814	1.958143084	protein_coding	NM_000253
ASHGV40029836	-1.95814	1.958136676	protein_coding	NM_001101376
ASHGV40012094	-1.95776	1.957758251	protein_coding	NM_005518
ASHGV40021455	1.957275	1.957275269	protein_coding	ENST00000578713
ASHGV40046835	1.957116	1.957116358	protein_coding	NM_004889
ASHGV40045087	1.956758	1.956757536	protein_coding	NM_153246
ASHGV40031247	-1.95624	1.956244032	protein_coding	NM_001004339
ASHGV40021896	-1.95612	1.956118995	protein_coding	NM_006546
ASHGV40040395	1.955496	1.955495893	protein_coding	NM_003633
ASHGV40019615	-1.95528	1.955281477	protein_coding	NM_004413
ASHGV40044370	-1.95445	1.954447375	protein_coding	NM_144980
ASHGV40017045	-1.95372	1.953720593	protein_coding	NM_005576
ASHGV40014055	1.952999	1.952998961	protein_coding	NM_015163
ASHGV40015622	1.95295	1.952950402	protein_coding	NM_000814
ASHGV40014214	1.952941	1.952941302	protein_coding	NM_015994
ASHGV40011139	-1.95279	1.952785651	protein_coding	NM_006446
ASHGV40024782	-1.9517	1.951701642	protein_coding	NM_006865
ASHGV40003147	-1.95149	1.951487683	protein_coding	NM_001198593
ASHGV40056758	-1.95037	1.950365848	protein_coding	NM_001005279
ASHGV40015097	-1.95001	1.950013701	protein_coding	NM_006617
ASHGV40039542	-1.94937	1.949367674	protein_coding	NM_020387
ASHGV40047073	1.948577	1.948577278	protein_coding	NM_003941
ASHGV40012593	-1.94811	1.94810935	protein_coding	NM_000053
ASHGV40055064	-1.94482	1.944816152	protein_coding	NM_004312
ASHGV40017895	1.94435	1.944350229	protein_coding	NM_003783
ASHGV40022142	1.944299	1.944299467	protein_coding	NM_000891
ASHGV40034005	1.944158	1.94415773	protein_coding	NM_015237
ASHGV40031162	1.943587	1.943586659	protein_coding	NM_001958
ASHGV40057184	-1.94349	1.943493856	protein_coding	NM_003525
ASHGV40021855	-1.94148	1.941475562	protein_coding	NM_152347
ASHGV40043818	-1.94048	1.940483165	protein_coding	NM_021813
ASHGV40014569	1.939931	1.939931068	protein_coding	NM_020836
ASHGV40024289	-1.93941	1.939409714	protein_coding	NM_021232
ASHGV40009294	-1.93811	1.93810597	protein_coding	NM_001001965
ASHGV40034750	1.937895	1.937894665	protein_coding	NM_022488
ASHGV40024107	1.937532	1.937531617	protein_coding	NM_000815
ASHGV40052240	1.937439	1.937439493	protein_coding	NM_014618
ASHGV40056584	-1.93696	1.936955193	protein_coding	NM_001123375
ASHGV40012107	-1.93689	1.93689091	protein_coding	NM_032044

ASHGV40048087	1.935468	1.93546843	protein_coding	NM_000840
ASHGV40023364	1.935422	1.935422342	protein_coding	ENST00000600091
ASHGV40053798	-1.935	1.93500046	protein_coding	NM_021804
ASHGV40017015	-1.93428	1.934275728	protein_coding	NM_014249
ASHGV40043035	1.93374	1.93373986	protein_coding	uc003nar.3
ASHGV40027637	-1.93348	1.933477926	protein_coding	NM_002054
ASHGV40010703	1.933453	1.933453281	protein_coding	NM_006549
ASHGV40046238	1.931731	1.9317309	protein_coding	NM_012294
ASHGV40032151	-1.93165	1.931648142	protein_coding	NM_001077711
ASHGV40010080	1.930407	1.930407392	protein_coding	NM_001686
ASHGV40051839	1.929928	1.929927842	protein_coding	NM_006007
ASHGV40057771	1.929633	1.929632716	protein_coding	NM_002032
ASHGV40012874	-1.9292	1.929203304	protein_coding	NM_005292
ASHGV40051340	1.929046	1.929046196	protein_coding	uc001bte.1
ASHGV40055878	1.928924	1.928923903	protein_coding	NM_021732
ASHGV40038184	1.926727	1.926726951	protein_coding	NM_016613
ASHGV40012105	1.926483	1.926482574	protein_coding	NM_002562
ASHGV40024415	-1.92646	1.926464112	protein_coding	NM_000660
ASHGV40023759	1.925793	1.92579275	protein_coding	NM_139159
ASHGV40012457	1.924906	1.924906002	protein_coding	NM_015087
ASHGV40054547	-1.92465	1.924647305	protein_coding	NM_001129826
ASHGV40017195	-1.92329	1.923286722	protein_coding	NM_001243531
ASHGV40012002	1.922149	1.922149405	protein_coding	NM_000690
ASHGV40032905	-1.92151	1.921507527	protein_coding	NM_012400
ASHGV40037661	1.920773	1.920773009	protein_coding	NM_005506
ASHGV40003274	-1.91893	1.918925118	protein_coding	NM_006398
ASHGV40005683	1.918679	1.91867907	protein_coding	NM_032439
ASHGV40012557	1.917115	1.917114973	protein_coding	NM_003850
ASHGV40036599	1.917048	1.917047839	protein_coding	NM_001080412
ASHGV40050155	1.916493	1.916492916	protein_coding	NM_002189
ASHGV40021952	1.916367	1.916366747	protein_coding	NM_002924
ASHGV40030281	-1.9163	1.916296594	protein_coding	NM_021027
ASHGV40049321	1.916188	1.916188018	protein_coding	NM_138969
ASHGV40055016	-1.91591	1.915905944	protein_coding	ENST00000433279
ASHGV40031063	-1.91516	1.915162433	protein_coding	NM_080618
ASHGV40011674	-1.91506	1.915060847	protein_coding	NM_005725
ASHGV40057516	-1.9146	1.914602075	protein_coding	ENST00000377647
ASHGV40007433	-1.91406	1.914058756	protein_coding	ENST00000544076
ASHGV40037819	1.913425	1.913424974	protein_coding	NM_000944
ASHGV40008003	-1.91318	1.91318309	protein_coding	NM_001004751
ASHGV40037727	1.91213	1.912129502	protein_coding	NM_006168
ASHGV40027895	1.911981	1.911981372	protein_coding	NM_144629
ASHGV40043684	1.910001	1.910000644	protein_coding	NM_018368
ASHGV40029774	-1.90981	1.909814322	protein_coding	NM_145810
ASHGV40056513	-1.90887	1.908866135	protein_coding	NM_000980
ASHGV40015747	1.908843	1.908843208	protein_coding	NM_001304802
ASHGV40006314	1.908743	1.908743217	protein_coding	NM_002775

ASHGV40037205	-1.90808	1.908075342	protein_coding	NM_001085382
ASHGV40041280	1.908007	1.908007062	protein_coding	NM_018434
ASHGV40012010	1.907987	1.907987141	protein_coding	NM_002834
ASHGV40001303	1.907815	1.907814615	protein_coding	NM_024576
ASHGV40016254	1.907685	1.907685006	protein_coding	NM_004644
ASHGV40048613	-1.90719	1.907186728	protein_coding	NM_005435
ASHGV40053829	1.906621	1.906621378	protein_coding	NM_152780
ASHGV40040006	-1.90661	1.906611698	protein_coding	NM_018240
ASHGV40018884	-1.90581	1.905813079	protein_coding	NM_018203
ASHGV40041865	-1.90545	1.905445245	protein_coding	ENST00000503931
ASHGV40024520	1.904215	1.904214946	protein_coding	uc001alo.4
ASHGV40013312	1.90365	1.903649836	protein_coding	NM_021999
ASHGV40034358	-1.9035	1.903502459	protein_coding	NM_001007540
ASHGV40049177	-1.90342	1.903419042	protein_coding	NM_001001918
ASHGV40025598	1.90283	1.902829778	protein_coding	NM_005166
ASHGV40049263	-1.90272	1.902722267	protein_coding	NM_003068
ASHGV40054525	1.900884	1.900883843	protein_coding	NM_000202
ASHGV40028875	-1.90026	1.900255206	protein_coding	NM_014466
ASHGV40042386	1.898074	1.898073837	protein_coding	NM_016480
ASHGV40055113	1.897716	1.89771616	protein_coding	NM_000291
ASHGV40012733	1.897512	1.897512395	protein_coding	uc010aew.3
ASHGV40018912	1.894532	1.894532007	protein_coding	NM_000034
ASHGV40006533	1.894324	1.894324331	protein_coding	NM_002574
ASHGV40045705	-1.89405	1.894049789	protein_coding	NM_005375
ASHGV40043257	1.892853	1.892853061	protein_coding	NM_002117
ASHGV40001360	-1.89217	1.892165005	protein_coding	NM_001333
ASHGV40048326	1.892045	1.892044845	protein_coding	NM_001035
ASHGV40046129	1.891272	1.891272442	protein_coding	NM_020197
ASHGV40048529	-1.89125	1.891250298	protein_coding	NM_005989
ASHGV40026711	1.890774	1.890773501	protein_coding	NM_001305624
ASHGV40043442	-1.89052	1.89051672	protein_coding	NM_003740
ASHGV40032793	-1.89027	1.890268228	protein_coding	uc002zis.2
ASHGV40023845	-1.89021	1.890212849	protein_coding	NM_178525
ASHGV40028103	1.889576	1.889575876	protein_coding	NM_004438
ASHGV40032212	1.889274	1.889273945	protein_coding	NM_001697
ASHGV40054737	1.88906	1.889060157	protein_coding	NM_003159
ASHGV40057556	-1.88901	1.889007208	protein_coding	NM_005421
ASHGV40043232	1.888079	1.888079039	protein_coding	NM_005275
ASHGV40012309	1.885989	1.885989338	protein_coding	NM_006783
ASHGV40043879	1.885817	1.885817217	protein_coding	NM_032503
ASHGV40016013	1.885349	1.885348502	protein_coding	NM_197960
ASHGV40053943	1.884492	1.88449242	protein_coding	NM_000898
ASHGV40012092	1.883847	1.883847247	protein_coding	NM_003746
ASHGV40048348	-1.88363	1.883634004	protein_coding	NM_015641
ASHGV40022643	1.88341	1.883409619	protein_coding	NM_004775
ASHGV40024533	1.883023	1.88302296	protein_coding	NM_175875
ASHGV40015241	1.882286	1.882286427	protein_coding	NM_001272020

ASHGV40009559	1.882197	1.882196758	protein_coding	NM_000014
ASHGV40007840	1.881866	1.881865536	protein_coding	NM_007037
ASHGV40032314	-1.88153	1.88153037	protein_coding	NM_003226
ASHGV40007432	1.881094	1.881094362	protein_coding	NM_001162951
ASHGV40011693	1.880782	1.880781528	protein_coding	NM_013381
ASHGV40025758	1.880326	1.880326402	protein_coding	NM_001193621
ASHGV40039142	1.880256	1.880256166	protein_coding	NM_001040058
ASHGV40009518	-1.88016	1.880159515	protein_coding	NM_031299
ASHGV40012899	1.879425	1.879424981	protein_coding	NM_001010977
ASHGV40024390	-1.87875	1.878749218	protein_coding	NM_024877
ASHGV40011005	-1.87855	1.87855356	protein_coding	NM_001080454
ASHGV40009378	-1.8785	1.878496947	protein_coding	NM_001271983
ASHGV40021161	-1.87764	1.877642731	protein_coding	uc021tob.1
ASHGV40021806	1.877546	1.877546489	protein_coding	NM_001466
ASHGV40020309	-1.87663	1.876627673	protein_coding	NM_030976
ASHGV40035141	1.876411	1.876410748	protein_coding	NM_007269
ASHGV40037673	1.876108	1.87610756	protein_coding	NM_006835
ASHGV40007334	1.875838	1.875838422	protein_coding	NM_198896
ASHGV40054452	1.87551	1.875509543	protein_coding	NM_001078172
ASHGV40055405	1.873419	1.873418782	protein_coding	NM_000194
ASHGV40042757	1.873199	1.873199183	protein_coding	NM_015980
ASHGV40049875	1.872573	1.872573287	protein_coding	NM_007222
ASHGV40011140	-1.87206	1.872062936	protein_coding	NM_000415
ASHGV40025141	-1.87093	1.87093433	protein_coding	NM_001004456
ASHGV40003331	-1.8708	1.870803173	protein_coding	NM_130777
ASHGV40036425	1.870791	1.870790704	protein_coding	NM_004547
ASHGV40021493	-1.87058	1.870578556	protein_coding	NM_003593
ASHGV40008272	-1.87048	1.870481098	protein_coding	ENST00000330381
ASHGV40020296	-1.87023	1.870234631	protein_coding	NM_033185
ASHGV40012401	1.867847	1.867847301	protein_coding	NM_007106
ASHGV40038067	-1.8671	1.867095611	protein_coding	NM_001277353
ASHGV40021719	-1.86709	1.867089615	protein_coding	NM_031963
ASHGV40017528	-1.86639	1.866391643	protein_coding	NM_001282416
ASHGV40047386	1.865791	1.865791188	protein_coding	NM_000858
ASHGV40037529	1.864995	1.864995184	protein_coding	NM_001553
ASHGV40023798	1.864842	1.864842069	protein_coding	NM_000064
ASHGV40015858	1.863867	1.863866919	protein_coding	NM_207381
ASHGV40047307	1.862651	1.862650998	protein_coding	NM_004935
ASHGV40045813	1.860851	1.860850776	protein_coding	NM_005389
ASHGV40031688	-1.86028	1.860278013	protein_coding	NM_007019
ASHGV40056699	-1.8601	1.860100343	protein_coding	NM_001083538
ASHGV40048402	1.859832	1.85983172	protein_coding	NM_000740
ASHGV40006050	-1.85954	1.859544474	protein_coding	NM_138413
ASHGV40055825	1.85942	1.859419615	protein_coding	NM_005398
ASHGV40049682	1.858838	1.858837559	protein_coding	NM_005836
ASHGV40011147	1.858247	1.85824673	protein_coding	NM_018686
ASHGV40046520	1.857419	1.857419111	protein_coding	NM_015198

ASHGV40054296	-1.85737	1.857372636	protein_coding	NM_138382
ASHGV40020072	1.856657	1.856657475	protein_coding	NM_005165
ASHGV40017698	1.856589	1.856588959	protein_coding	NM_016641
ASHGV40023418	1.856273	1.856273424	protein_coding	NM_015879
ASHGV40022570	-1.85614	1.856141885	protein_coding	NM_001010890
ASHGV40047061	1.855007	1.855006826	protein_coding	NM_017954
ASHGV40030321	-1.85472	1.854720155	protein_coding	NM_006845
ASHGV40032580	1.854626	1.854626082	protein_coding	NM_000454
ASHGV40024268	-1.85441	1.854413219	protein_coding	NM_014638
ASHGV40013046	-1.85394	1.853944446	protein_coding	ENST00000601204
ASHGV40006446	1.851818	1.851817696	protein_coding	uc001lnh.1
ASHGV40011195	1.851814	1.851814	protein_coding	NM_018318
ASHGV40024453	-1.85165	1.851648509	protein_coding	NM_002782
ASHGV40027281	-1.85114	1.851142879	protein_coding	NM_014438
ASHGV40009547	-1.85109	1.851091601	protein_coding	NM_018088
ASHGV40005965	1.85044	1.85043982	protein_coding	NM_001548
ASHGV40057818	1.849596	1.849596389	protein_coding	NM_016090
ASHGV40018462	-1.84838	1.848379403	protein_coding	ENST00000408886
ASHGV40019136	-1.8474	1.847396457	protein_coding	NM_004530
ASHGV40050993	1.847198	1.84719838	protein_coding	NM_020697
ASHGV40018721	1.845659	1.845658563	protein_coding	NM_014048
ASHGV40056509	-1.84513	1.845132055	protein_coding	NM_024104
ASHGV40006322	-1.84481	1.844807766	protein_coding	NM_001029888
ASHGV40032315	-1.84462	1.844623767	protein_coding	NM_005423
ASHGV40038256	1.842828	1.842827511	protein_coding	NM_021647
ASHGV40054370	1.842324	1.842324216	protein_coding	NM_002294
ASHGV40013139	-1.84174	1.841738737	protein_coding	NM_000209
ASHGV40046563	1.841437	1.841436554	protein_coding	NM_004577
ASHGV40007470	1.840571	1.84057061	protein_coding	NM_001305043
ASHGV40041065	-1.84034	1.840338586	protein_coding	ENST00000599823
ASHGV40014452	1.840009	1.840008969	protein_coding	NM_005979
ASHGV40053999	1.839372	1.839372432	protein_coding	NM_006521
ASHGV40050396	-1.83893	1.838931	protein_coding	NM_003817
ASHGV40032129	-1.83845	1.838452865	protein_coding	NM_014495
ASHGV40001975	1.837391	1.837390731	protein_coding	NM_002567
ASHGV40034410	-1.83726	1.837261469	protein_coding	NM_003280
ASHGV40022522	1.837178	1.837178286	protein_coding	NM_006796
ASHGV40022792	1.83706	1.837060184	protein_coding	NM_031939
ASHGV40051287	1.836284	1.836283978	protein_coding	NM_006558
ASHGV40044189	-1.8353	1.835302422	protein_coding	NM_002511
ASHGV40025284	-1.83382	1.833817368	protein_coding	NM_013939
ASHGV40018645	1.83345	1.833449973	protein_coding	NM_002134
ASHGV40030737	1.833218	1.833218075	protein_coding	NM_138578
ASHGV40044080	1.83316	1.833159511	protein_coding	NM_001431
ASHGV40046904	1.832222	1.832221834	protein_coding	NM_198990
ASHGV40034516	1.832054	1.832053645	protein_coding	NM_014839
ASHGV40033389	1.83142	1.831419674	protein_coding	NM_031481

ASHGV40022022	-1.83011	1.830111282	protein_coding	NM_001004343
ASHGV40025951	-1.8298	1.829798791	protein_coding	NM_005551
ASHGV40014494	-1.8288	1.828796038	protein_coding	NM_173849
ASHGV40033246	1.82794	1.827939751	protein_coding	NM_001099294
ASHGV40031594	1.827593	1.827593327	protein_coding	NM_015568
ASHGV40043489	-1.82692	1.826917774	protein_coding	NM_002927
ASHGV40053304	1.826132	1.826131501	protein_coding	NM_133464
ASHGV40009645	-1.82599	1.825989778	protein_coding	NM_207398
ASHGV40031870	1.825554	1.825553957	protein_coding	NM_183244
ASHGV40006640	1.825174	1.82517362	protein_coding	NM_020645
ASHGV40055507	-1.82507	1.825070117	protein_coding	NM_016364
ASHGV40016948	1.824668	1.824668433	protein_coding	NM_016395
ASHGV40026584	1.824399	1.824399285	protein_coding	NM_015475
ASHGV40016036	-1.82393	1.823930838	protein_coding	NM_033429
ASHGV40022934	1.823829	1.823828766	protein_coding	NM_138999
ASHGV40012514	-1.82369	1.82368991	protein_coding	NM_144974
ASHGV40011997	1.823321	1.823320929	protein_coding	NM_005475
ASHGV40046663	-1.82329	1.823287086	protein_coding	NM_001306
ASHGV40042529	-1.82324	1.823244149	protein_coding	NM_001040129
ASHGV40054078	1.82307	1.823069859	protein_coding	NM_198215
ASHGV40006711	-1.82305	1.823045385	protein_coding	NM_004179
ASHGV40014971	1.822921	1.822920607	protein_coding	uc001fot.1
ASHGV40020420	-1.8206	1.820603997	protein_coding	NM_005497
ASHGV40016308	1.82055	1.820550344	protein_coding	NM_001012338
ASHGV40043001	-1.82046	1.820459661	protein_coding	NM_003220
ASHGV40019686	1.820367	1.820366578	protein_coding	NM_015229
ASHGV40006478	1.820045	1.820044639	protein_coding	NM_021008
ASHGV40029029	-1.81964	1.819642083	protein_coding	NM_001615
ASHGV40040501	-1.81952	1.819516311	protein_coding	NM_021181
ASHGV40025745	-1.81944	1.819435369	protein_coding	NM_133444
ASHGV40030814	-1.81935	1.819354003	protein_coding	NM_002895
ASHGV40039331	-1.81932	1.819321416	protein_coding	uc003idj.1
ASHGV40053040	1.819215	1.819215454	protein_coding	NM_080391
ASHGV40007539	-1.81842	1.818419272	protein_coding	NM_002425
ASHGV40051070	1.818102	1.818102497	protein_coding	NM_014673
ASHGV40051704	-1.81694	1.816939437	protein_coding	NM_032818
ASHGV40060871	-1.81656	1.81656254	protein_coding	uc010lzi.1
ASHGV40048591	-1.81646	1.816463199	protein_coding	NM_000083
ASHGV40035685	1.815662	1.815662068	protein_coding	NM_003884
ASHGV40037593	-1.81485	1.81484562	protein_coding	NM_001076
ASHGV40030187	1.814673	1.814673211	protein_coding	NM_004457
ASHGV40015108	1.81449	1.814489542	protein_coding	NM_021979
ASHGV40053985	-1.81434	1.814338444	protein_coding	ENST00000376775
ASHGV40044048	1.813127	1.813127045	protein_coding	NM_016063
ASHGV40017048	-1.8126	1.812598508	protein_coding	NM_020851
ASHGV40038275	1.812411	1.812410931	protein_coding	NM_007281
ASHGV40011428	-1.81232	1.812316783	protein_coding	NM_003578

ASHGV40027690	1.81221	1.81220984	protein_coding	NM_012290
ASHGV40011352	1.811818	1.811818489	protein_coding	NM_012284
ASHGV40017190	1.811024	1.811024048	protein_coding	NM_003027
ASHGV40005072	1.810823	1.810823434	protein_coding	NM_001004298
ASHGV40009670	1.810401	1.810401285	protein_coding	NM_024730
ASHGV40055440	-1.81017	1.810174934	protein_coding	NM_003413
ASHGV40056672	1.810121	1.810120802	protein_coding	NM_000993
ASHGV40035706	1.810069	1.810068691	protein_coding	NM_001145425
ASHGV40026718	1.809591	1.809591322	protein_coding	NM_022055
ASHGV40044922	-1.80887	1.808871221	protein_coding	NM_002263
ASHGV40038295	1.808171	1.808171235	protein_coding	NM_005277
ASHGV40011771	-1.80744	1.807440148	protein_coding	NM_006183
ASHGV40014285	-1.8056	1.805595004	protein_coding	NM_194278
ASHGV40046307	-1.80539	1.805393376	protein_coding	NM_000522
ASHGV40027477	-1.80533	1.80533285	protein_coding	NM_003467
ASHGV40011464	-1.80529	1.805288025	protein_coding	NM_004503
ASHGV40002658	-1.80509	1.805093463	protein_coding	ENST00000598428
ASHGV40052171	1.804989	1.804989465	protein_coding	NM_003329
ASHGV40037349	-1.80494	1.804935872	protein_coding	ENST00000507759
ASHGV40019147	1.80317	1.803170184	protein_coding	NM_020988
ASHGV40031050	-1.8024	1.802400101	protein_coding	NM_080615
ASHGV40043699	-1.80218	1.802176933	protein_coding	NM_001025290
ASHGV40045159	1.802135	1.802134787	protein_coding	NM_182663
ASHGV40044159	-1.80153	1.80153271	protein_coding	NM_021635
ASHGV40009491	1.801059	1.801059446	protein_coding	NM_000552
ASHGV40024457	-1.80051	1.800508748	protein_coding	NM_031246
ASHGV40053567	-1.79994	1.799935538	protein_coding	NM_001807
ASHGV40041179	-1.79936	1.799361178	protein_coding	NM_003714
ASHGV40029727	1.797549	1.797549051	protein_coding	NM_021007
ASHGV40043932	1.797224	1.797224414	protein_coding	NM_001199933
ASHGV40047302	1.796112	1.796111836	protein_coding	NM_014020
ASHGV40033028	-1.79556	1.795563272	protein_coding	NM_002309
ASHGV40020318	-1.79553	1.795534215	protein_coding	NM_021013
ASHGV40008566	1.795479	1.795479136	protein_coding	NM_001265589
ASHGV40055253	1.794894	1.794893704	protein_coding	NM_152423
ASHGV40018670	1.79459	1.794590154	protein_coding	NM_000663
ASHGV40042389	-1.79339	1.793391421	protein_coding	ENST00000593907
ASHGV40035788	-1.79219	1.792192415	protein_coding	NM_003149
ASHGV40018897	1.792111	1.792111295	protein_coding	NM_007317
ASHGV40034848	1.791653	1.791652585	protein_coding	NM_012190
ASHGV40052051	-1.79148	1.79148251	protein_coding	uc004awu.3
ASHGV40032066	-1.79125	1.791252246	protein_coding	NM_019079
ASHGV40055586	1.79113	1.791130416	protein_coding	NM_012151
ASHGV40009648	-1.79095	1.790945214	protein_coding	NM_001013698
ASHGV40007343	-1.79054	1.790540751	protein_coding	NM_182904
ASHGV40026353	1.790331	1.790330902	protein_coding	NM_004850
ASHGV40020636	-1.78929	1.789288403	protein_coding	NM_001201457

ASHGV40006537	-1.78903	1.789032044	protein_coding	NM_053017
ASHGV40056566	-1.78817	1.788170659	protein_coding	NM_033183
ASHGV40032276	-1.78739	1.787392035	protein_coding	NM_152505
ASHGV40018830	1.787355	1.787354658	protein_coding	NM_006539
ASHGV40009551	-1.7873	1.787296166	protein_coding	NM_020661
ASHGV40043601	-1.78718	1.787181036	protein_coding	NM_000846
ASHGV40054863	-1.78709	1.787088938	protein_coding	NM_080817
ASHGV40017592	-1.78665	1.786654882	protein_coding	NM_001146336
ASHGV40040765	1.786623	1.786622903	protein_coding	NM_003001
ASHGV40051136	1.786291	1.786291136	protein_coding	NM_052886
ASHGV40039022	1.785606	1.785606379	protein_coding	NM_014961
ASHGV40013735	-1.78452	1.784517883	protein_coding	NM_003891
ASHGV40011055	1.783785	1.7837851	protein_coding	NM_031412
ASHGV40032633	-1.78247	1.782467437	protein_coding	ENST00000600312
ASHGV40053930	-1.78237	1.782368147	protein_coding	NM_000242
ASHGV40042720	-1.78222	1.782222659	protein_coding	ENST00000524175
ASHGV40006245	1.780956	1.780955535	protein_coding	NM_207303
ASHGV40017202	-1.78047	1.780472399	protein_coding	NM_181877
ASHGV40045070	-1.78047	1.780467448	protein_coding	NM_032538
ASHGV40010980	1.780262	1.780261541	protein_coding	NM_002824
ASHGV40029797	-1.77987	1.779867035	protein_coding	NM_021192
ASHGV40029957	-1.7798	1.779797031	protein_coding	uc021vuo.1
ASHGV40014948	1.779754	1.779753812	protein_coding	NM_015091
ASHGV40050967	-1.7796	1.779602159	protein_coding	NM_001081
ASHGV40039526	1.779245	1.779245416	protein_coding	NM_001130067
ASHGV40009120	-1.77834	1.778344439	protein_coding	NM_001100388
ASHGV40016942	-1.77757	1.777574113	protein_coding	NM_178859
ASHGV40039480	1.777253	1.777253282	protein_coding	NM_152280
ASHGV40037180	-1.7764	1.776395241	protein_coding	NM_002614
ASHGV40030265	-1.77603	1.776027881	protein_coding	ENST00000389494
ASHGV40055459	-1.77512	1.775119071	protein_coding	ENST00000370530
ASHGV40006578	-1.77499	1.774993143	protein_coding	NM_017481
ASHGV40029163	1.774945	1.774945243	protein_coding	NM_004552
ASHGV40022652	-1.77456	1.774559303	protein_coding	NM_020805
ASHGV40054308	-1.77243	1.772434212	protein_coding	NM_003604
ASHGV40040614	1.772383	1.772383203	protein_coding	NM_031438
ASHGV40055891	1.772354	1.772354018	protein_coding	NM_002079
ASHGV40002426	-1.77167	1.771665014	protein_coding	ENST00000577797
ASHGV40021361	1.771576	1.771576257	protein_coding	NM_014801
ASHGV40050156	-1.77032	1.770323423	protein_coding	NM_000417
ASHGV40053489	-1.76994	1.769938635	protein_coding	ENST00000601297
ASHGV40014440	-1.76971	1.769712695	protein_coding	NM_020672
ASHGV40027824	-1.7693	1.769300415	protein_coding	NM_014474
ASHGV40002609	-1.76906	1.769064254	protein_coding	ENST00000595026
ASHGV40045312	-1.76841	1.768410047	protein_coding	NM_003318
ASHGV40021768	-1.76829	1.768290862	protein_coding	NM_002521
ASHGV40056102	-1.76807	1.768066645	protein_coding	NM_001101341

ASHGV40033741	-1.76805	1.768047765	protein_coding	NM_152426
ASHGV40044782	-1.76775	1.767746869	protein_coding	NM_012367
ASHGV40054120	-1.76774	1.76774006	protein_coding	NM_001025265
ASHGV40043488	-1.76752	1.767520669	protein_coding	NM_001164446
ASHGV40024619	-1.76744	1.767442275	protein_coding	NM_001278501
ASHGV40016027	-1.76666	1.766662751	protein_coding	NM_207338
ASHGV40033903	-1.76537	1.765372741	protein_coding	NM_014838
ASHGV40020503	-1.76466	1.76465954	protein_coding	NM_002147
ASHGV40024769	-1.76389	1.763893018	protein_coding	NM_130771
ASHGV40028798	-1.76382	1.76381748	protein_coding	NM_000341
ASHGV40044686	-1.76309	1.763090904	protein_coding	NM_003107
ASHGV40050423	1.762535	1.762534801	protein_coding	NM_004103
ASHGV40031164	-1.76246	1.762459329	protein_coding	ENST00000217188
ASHGV40020325	-1.76242	1.762417071	protein_coding	NM_002274
ASHGV40036799	1.762386	1.762385538	protein_coding	NM_003262
ASHGV40037794	-1.76161	1.761607498	protein_coding	NM_000668
ASHGV40035237	-1.76132	1.76131901	protein_coding	NM_000340
ASHGV40045027	-1.76009	1.760093969	protein_coding	NM_006789
ASHGV40027018	1.759459	1.759458678	protein_coding	NM_001747
ASHGV40015538	-1.75944	1.759437656	protein_coding	NM_001135050
ASHGV40046704	1.758751	1.758750834	protein_coding	NM_012301
ASHGV40039002	-1.75814	1.758141269	protein_coding	NM_001075
ASHGV40006249	-1.75742	1.757424072	protein_coding	NM_001011709
ASHGV40022685	1.757256	1.757256173	protein_coding	NM_020180
ASHGV40014635	-1.75558	1.755580432	protein_coding	uc001you.2
ASHGV40046226	-1.75519	1.755192329	protein_coding	NM_182762
ASHGV40016266	1.755187	1.755187291	protein_coding	NM_003851
ASHGV40035389	-1.7546	1.75460332	protein_coding	NM_001879
ASHGV40035418	-1.75405	1.754047153	protein_coding	NM_207316
ASHGV40012369	1.753618	1.753618376	protein_coding	NM_005977
ASHGV40006008	1.75285	1.75284987	protein_coding	NM_005097
ASHGV40007344	1.752823	1.752823108	protein_coding	NM_173582
ASHGV40015149	-1.75264	1.752636728	protein_coding	NM_014215
ASHGV40021232	1.752433	1.752432564	protein_coding	NM_001678
ASHGV40043817	-1.75161	1.751613513	protein_coding	NM_020204
ASHGV40028154	-1.75142	1.751422806	protein_coding	NM_024795
ASHGV40036260	-1.75133	1.751330582	protein_coding	ENST00000394191
ASHGV40029690	-1.75064	1.750636635	protein_coding	NM_001017920
ASHGV40040067	-1.75023	1.750225462	protein_coding	NM_030893
ASHGV40012453	-1.74982	1.749815485	protein_coding	NM_005584
ASHGV40010727	1.749383	1.749382727	protein_coding	NM_020845
ASHGV40018882	-1.74708	1.747080643	protein_coding	NM_173201
ASHGV40024754	-1.74696	1.746962147	protein_coding	NM_182609
ASHGV40018577	-1.7466	1.746599929	protein_coding	NM_001761
ASHGV40007941	1.74654	1.746539604	protein_coding	NM_000775
ASHGV40044280	1.746129	1.746128639	protein_coding	NM_015553
ASHGV40046898	-1.74557	1.745565939	protein_coding	NM_001277335

ASHGV40048579	1.745064	1.745063806	protein_coding	NM_004445
ASHGV40015749	-1.74498	1.744978771	protein_coding	NM_017726
ASHGV40024890	1.744933	1.744932735	protein_coding	NM_023926
ASHGV40011676	1.744154	1.744154245	protein_coding	NM_014505
ASHGV40018798	1.743898	1.743897823	protein_coding	NM_001164579
ASHGV40015884	-1.74301	1.743008967	protein_coding	NM_004498
ASHGV40051614	-1.743	1.743003702	protein_coding	NM_022901
ASHGV40023708	1.742872	1.7428716	protein_coding	NM_052847
ASHGV40049419	1.742732	1.742732375	protein_coding	NM_025054
ASHGV40037102	1.742674	1.742673716	protein_coding	NM_007100
ASHGV40008414	-1.74184	1.741836393	protein_coding	NM_001004124
ASHGV40008842	1.741138	1.741138418	protein_coding	NM_021200
ASHGV40010521	1.741128	1.741127778	protein_coding	NM_014840
ASHGV40006817	-1.74109	1.741093937	protein_coding	NM_001008391
ASHGV40031251	1.741062	1.741061655	protein_coding	NM_033453
ASHGV40048895	-1.74037	1.740368013	protein_coding	ENST00000400102
ASHGV40030482	-1.74006	1.740064	protein_coding	ENST00000379526
ASHGV40021647	-1.73944	1.739437729	protein_coding	NM_173625
ASHGV40057386	1.738766	1.738765832	protein_coding	uc022anf.1
ASHGV40046383	-1.73874	1.738744595	protein_coding	uc003tgl.2
ASHGV40010991	1.738487	1.738486878	protein_coding	NM_001975
ASHGV40028111	-1.73833	1.738332827	protein_coding	NM_181457
ASHGV40014864	-1.73752	1.737515166	protein_coding	NM_001093725
ASHGV40017407	-1.73661	1.736605803	protein_coding	NM_030933
ASHGV40019998	-1.7366	1.736602948	protein_coding	NM_152908
ASHGV40007975	-1.73606	1.736059577	protein_coding	ENST00000332881
ASHGV40045640	1.735913	1.735912536	protein_coding	NM_181782
ASHGV40017602	1.735455	1.735455267	protein_coding	NM_000833
ASHGV40007323	1.735157	1.735157129	protein_coding	NM_002599
ASHGV40052789	-1.73493	1.734929574	protein_coding	NM_203299
ASHGV40044117	1.733991	1.733991343	protein_coding	NM_006620
ASHGV40028501	1.732975	1.732974943	protein_coding	NM_002236
ASHGV40037305	-1.73297	1.732965983	protein_coding	ENST00000502482
ASHGV40044899	1.732142	1.732141643	protein_coding	NM_019111
ASHGV40024989	1.731236	1.731236264	protein_coding	NM_004152
ASHGV40008131	-1.73099	1.730985107	protein_coding	NM_001031853
ASHGV40013582	1.730964	1.730963896	protein_coding	NM_006984
ASHGV40036377	1.730605	1.730605191	protein_coding	NM_001690
ASHGV40033983	1.730591	1.730591391	protein_coding	NM_006354
ASHGV40043140	-1.73017	1.730170351	protein_coding	NM_003518
ASHGV40012700	1.729929	1.729928903	protein_coding	NM_001071775
ASHGV40016881	1.729295	1.72929529	protein_coding	NM_152450
ASHGV40034192	-1.7285	1.728497981	protein_coding	NM_194293
ASHGV40006753	1.728433	1.728433401	protein_coding	NM_148893
ASHGV40017112	1.728206	1.728205538	protein_coding	NM_005530
ASHGV40036723	1.728158	1.728158007	protein_coding	NM_172160
ASHGV40043207	-1.72811	1.728110348	protein_coding	NM_013937

ASHGV40056278	1.727606	1.727605989	protein_coding	ENST00000520915
ASHGV40024689	-1.72724	1.727241939	protein_coding	ENST00000156499
ASHGV40022594	1.726861	1.726861345	protein_coding	NM_173505
ASHGV40057595	-1.72681	1.726809585	protein_coding	NM_173523
ASHGV40023719	1.726709	1.726708813	protein_coding	NM_001130
ASHGV40034812	1.725889	1.725889454	protein_coding	NM_004487
ASHGV40052278	-1.72574	1.725735508	protein_coding	NM_001004454
ASHGV40019815	-1.7245	1.72449783	protein_coding	NM_021628
ASHGV40034800	-1.72413	1.724125577	protein_coding	NM_153002
ASHGV40033958	-1.72299	1.722990926	protein_coding	ENST00000600805
ASHGV40018781	-1.72275	1.722751716	protein_coding	NM_001308172
ASHGV40028516	1.722664	1.722664432	protein_coding	NM_002143
ASHGV40034785	1.722201	1.722201303	protein_coding	uc011biu.1
ASHGV40011217	-1.72161	1.721611268	protein_coding	ENST00000313737
ASHGV40057296	1.721293	1.721292852	protein_coding	NM_198956
ASHGV40028912	1.721161	1.721161035	protein_coding	NM_001286365
ASHGV40041948	1.720775	1.720774906	protein_coding	NM_003719
ASHGV40016169	1.720592	1.720591997	protein_coding	NM_005724
ASHGV40054304	-1.71932	1.7193246	protein_coding	NM_031273
ASHGV40055243	-1.71872	1.718720803	protein_coding	NM_194324
ASHGV40033839	-1.71746	1.717457138	protein_coding	ENST00000342894
ASHGV40008411	-1.71714	1.717142713	protein_coding	NM_001001920
ASHGV40050097	1.716985	1.716984627	protein_coding	ENST00000340490
ASHGV40040992	-1.7163	1.716297397	protein_coding	NM_000440
ASHGV40019530	1.716241	1.716240536	protein_coding	NM_001861
ASHGV40055806	1.716222	1.71622194	protein_coding	NM_000235
ASHGV40032321	1.716155	1.716154666	protein_coding	NM_080860
ASHGV40038459	1.715207	1.715206558	protein_coding	uc031sdb.1
ASHGV40008977	1.714979	1.714979276	protein_coding	NM_153696
ASHGV40030277	-1.71473	1.714729007	protein_coding	NM_000541
ASHGV40010277	1.714431	1.714430697	protein_coding	NM_032606
ASHGV40029755	1.714145	1.714145001	protein_coding	NM_000817
ASHGV40015756	-1.71341	1.713411656	protein_coding	NM_007280
ASHGV40015866	1.713397	1.713396854	protein_coding	NM_015263
ASHGV40032102	1.713322	1.713321598	protein_coding	NM_001685
ASHGV40052537	1.713287	1.713287441	protein_coding	NM_031297
ASHGV40056586	-1.71299	1.712993118	protein_coding	NM_003548
ASHGV40020506	-1.71272	1.712717767	protein_coding	NM_024016
ASHGV40029978	1.712088	1.712088495	protein_coding	NM_002491
ASHGV40043245	-1.71187	1.711872074	protein_coding	NM_205854
ASHGV40055065	-1.71182	1.711823157	protein_coding	NM_001032726
ASHGV40022742	1.71165	1.711650256	protein_coding	NM_004046
ASHGV40045212	-1.7113	1.711298801	protein_coding	NM_006850
ASHGV40053683	1.710859	1.710859156	protein_coding	NM_001256067
ASHGV40016814	1.710676	1.710675599	protein_coding	NM_014548
ASHGV40021922	-1.71033	1.710331387	protein_coding	NM_152463
ASHGV40049413	1.71027	1.710269882	protein_coding	NM_000756

ASHGV40015530	1.710098	1.710098163	protein_coding	NM_003564
ASHGV40031058	1.70978	1.70978008	protein_coding	NM_001190472
ASHGV40056186	-1.70945	1.709453768	protein_coding	uc031qsh.1
ASHGV40006115	1.709411	1.709410624	protein_coding	NM_006824
ASHGV40055422	-1.70915	1.709151913	protein_coding	NM_018666
ASHGV40009859	1.708136	1.708135654	protein_coding	NM_006159
ASHGV40035994	-1.70799	1.707994286	protein_coding	NM_145262
ASHGV40000140	-1.70768	1.707679252	protein_coding	NM_003600
ASHGV40032168	-1.7076	1.707595449	protein_coding	NM_181606
ASHGV40030680	-1.70759	1.707592152	protein_coding	NM_001900
ASHGV40051984	1.70678	1.706780397	protein_coding	NM_001698
ASHGV40008564	-1.70655	1.706552623	protein_coding	NM_001114120
ASHGV40040161	1.706459	1.706458847	protein_coding	NM_000997
ASHGV40021250	-1.70639	1.706385654	protein_coding	uc002gjx.1
ASHGV40045712	1.706211	1.706210936	protein_coding	NM_018945
ASHGV40054418	-1.70605	1.706051049	protein_coding	NM_001421
ASHGV40040171	1.705905	1.705904789	protein_coding	NM_000436
ASHGV40054002	1.705817	1.705816527	protein_coding	NM_015698
ASHGV40046800	1.705736	1.705735666	protein_coding	NM_002612
ASHGV40031987	-1.70516	1.705160912	protein_coding	NM_182484
ASHGV40034755	-1.70499	1.704994179	protein_coding	NM_001008784
ASHGV40053795	1.704749	1.70474853	protein_coding	NM_003662
ASHGV40036926	-1.70472	1.704724725	protein_coding	ENST00000296270
ASHGV40023382	-1.70459	1.704594356	protein_coding	NM_145060
ASHGV40031800	-1.70413	1.704130584	protein_coding	NM_019888
ASHGV40019796	1.704106	1.704106496	protein_coding	NM_020899
ASHGV40007545	-1.70404	1.704036383	protein_coding	NM_002427
ASHGV40049286	1.703741	1.703740621	protein_coding	NM_014781
ASHGV40057025	-1.70335	1.703350727	protein_coding	uc003hhq.1
ASHGV40035421	-1.70247	1.702468143	protein_coding	NM_001146686
ASHGV40038151	1.702249	1.702248902	protein_coding	NM_173662
ASHGV40040398	1.700875	1.700874504	protein_coding	NM_016591
ASHGV40024095	1.69999	1.699990398	protein_coding	NM_004750
ASHGV40053901	1.699696	1.699696304	protein_coding	NM_006520
ASHGV40011377	1.699473	1.699472583	protein_coding	NM_005167
ASHGV40035128	-1.69923	1.699232441	protein_coding	ENST00000399242
ASHGV40028677	1.699167	1.699167065	protein_coding	NM_024692
ASHGV40053409	-1.69801	1.698014544	protein_coding	NM_012364
ASHGV40009374	-1.6977	1.697700473	protein_coding	NM_139055
ASHGV40022122	1.697603	1.697603116	protein_coding	NM_002734
ASHGV40030239	1.697242	1.697242144	protein_coding	NM_030926
ASHGV40026488	-1.69683	1.696834508	protein_coding	NM_001039500
ASHGV40047616	1.696297	1.696296664	protein_coding	NM_000905
ASHGV40033214	-1.69625	1.696253917	protein_coding	NM_024053
ASHGV40000144	-1.69623	1.696225998	protein_coding	NM_017681
ASHGV40053919	1.696202	1.696202334	protein_coding	NM_023009
ASHGV40015652	1.695457	1.695457407	protein_coding	NM_017762

ASHGV40049097	1.695409	1.695409078	protein_coding	NM_001009552
ASHGV40041425	1.695137	1.695136921	protein_coding	NM_001017977
ASHGV40045977	-1.69509	1.695093908	protein_coding	NM_153606
ASHGV40020209	-1.69423	1.69422679	protein_coding	uc010cum.2
ASHGV40025009	1.693298	1.693298441	protein_coding	uc010xhe.3
ASHGV40007857	1.69291	1.692909639	protein_coding	NM_002545
ASHGV40039285	1.692872	1.69287165	protein_coding	NM_001148
ASHGV40027996	1.692236	1.692236473	protein_coding	NM_001099334
ASHGV40018610	-1.69207	1.692070229	protein_coding	NM_016639
ASHGV40043141	-1.69056	1.690561049	protein_coding	NM_005320
ASHGV40033844	1.690041	1.690040577	protein_coding	NM_013236
ASHGV40010981	-1.68986	1.689863151	protein_coding	NM_002286
ASHGV40054376	-1.68974	1.689739921	protein_coding	NM_001080146
ASHGV40037516	1.68972	1.689720424	protein_coding	NM_004898
ASHGV40023295	1.689398	1.689398393	protein_coding	NM_001390
ASHGV40054918	1.6892	1.689199877	protein_coding	NM_004651
ASHGV40016239	-1.68906	1.689058862	protein_coding	NM_032246
ASHGV40023303	-1.68864	1.688640791	protein_coding	NM_017947
ASHGV40032115	1.688308	1.68830759	protein_coding	NM_006988
ASHGV40015683	-1.68823	1.688226569	protein_coding	NM_001166663
ASHGV40014656	-1.68764	1.687637362	protein_coding	NM_001010979
ASHGV40051661	1.687601	1.687601064	protein_coding	NM_001172415
ASHGV40050599	-1.68737	1.687372826	protein_coding	NM_001005365
ASHGV40014100	-1.68733	1.687327244	protein_coding	NM_014750
ASHGV40005109	-1.68701	1.687012866	protein_coding	NM_001005463
ASHGV40042487	1.686988	1.686987823	protein_coding	NM_030571
ASHGV40010084	1.686573	1.686572892	protein_coding	NM_001113202
ASHGV40028007	-1.68642	1.686415599	protein_coding	NM_079420
ASHGV40019337	-1.68588	1.685879793	protein_coding	NM_006147
ASHGV40042267	1.685835	1.685834978	protein_coding	NM_023927
ASHGV40005328	1.685557	1.685556726	protein_coding	NM_021980
ASHGV40011138	-1.68527	1.68526628	protein_coding	NM_001009562
ASHGV40043581	-1.68525	1.685251283	protein_coding	NM_001037497
ASHGV40025218	-1.6852	1.685200654	protein_coding	NM_017682
ASHGV40056128	1.68442	1.684420438	protein_coding	NM_022151
ASHGV40013839	1.684414	1.684414418	protein_coding	NM_012244
ASHGV40056196	-1.68437	1.684374091	protein_coding	NM_017793
ASHGV40031076	-1.68423	1.684227319	protein_coding	uc002xyy.1
ASHGV40021249	-1.68365	1.683652966	protein_coding	NM_001141
ASHGV40042392	1.683604	1.683603947	protein_coding	NM_016463
ASHGV40060901	-1.68353	1.683525175	protein_coding	uc031pwv.1
ASHGV40006430	-1.68325	1.683252064	protein_coding	NM_173541
ASHGV40026364	1.682522	1.682522474	protein_coding	NM_001032363
ASHGV40023200	-1.68226	1.682264539	protein_coding	ENST00000579830
ASHGV40028811	-1.6814	1.681400345	protein_coding	NM_001145051
ASHGV40023646	1.68114	1.68113963	protein_coding	NM_003712
ASHGV40015236	-1.68091	1.680911081	protein_coding	NM_174943

ASHGV40039347	1.680408	1.680408461	protein_coding	NM_015312
ASHGV40036529	1.680182	1.680181537	protein_coding	NM_153264
ASHGV40009953	1.679971	1.67997083	protein_coding	NM_001290007
ASHGV40007437	1.679686	1.67968566	protein_coding	NM_006680
ASHGV40046860	1.679378	1.67937844	protein_coding	NM_030935
ASHGV40044489	1.678559	1.678558815	protein_coding	NM_000674
ASHGV40049221	1.676951	1.676951187	protein_coding	NM_020476
ASHGV40028861	1.676609	1.676608677	protein_coding	NM_003128
ASHGV40057625	-1.6764	1.676395471	protein_coding	NM_138960
ASHGV40046338	1.676026	1.676025521	protein_coding	NM_001191057
ASHGV40027180	-1.67565	1.675650417	protein_coding	NM_032718
ASHGV40057862	1.675613	1.675613217	protein_coding	NM_006009
ASHGV40025157	1.675507	1.675507171	protein_coding	NM_006221
ASHGV40013868	1.67544	1.675440281	protein_coding	NM_006156
ASHGV40028077	-1.67531	1.675305423	protein_coding	NM_001144889
ASHGV40013099	-1.67447	1.674471059	protein_coding	NM_018647
ASHGV40024448	-1.67444	1.674441968	protein_coding	NM_001816
ASHGV40016188	1.673502	1.673502256	protein_coding	NM_015162
ASHGV40035881	-1.67294	1.67294475	protein_coding	NM_020242
ASHGV40044737	-1.67294	1.672941279	protein_coding	NM_003522
ASHGV40020991	-1.67269	1.672691613	protein_coding	ENST00000425898
ASHGV40029032	-1.67191	1.671913875	protein_coding	NM_025079
ASHGV40049562	-1.67182	1.671817867	protein_coding	ENST00000546501
ASHGV40005367	1.671788	1.671788362	protein_coding	NM_201596
ASHGV40027733	1.67177	1.671770085	protein_coding	NM_001689
ASHGV40028656	-1.67173	1.671728407	protein_coding	NM_173853
ASHGV40056941	-1.67148	1.671480111	protein_coding	NM_000685
ASHGV40001261	1.671207	1.671206883	protein_coding	NM_003670
ASHGV40045822	-1.67085	1.670852557	protein_coding	NM_025218
ASHGV40042905	-1.67051	1.670506457	protein_coding	NM_005562
ASHGV40045066	-1.67	1.669997724	protein_coding	NM_002821
ASHGV40008942	1.66946	1.669459684	protein_coding	NM_182603
ASHGV40040862	1.669307	1.669307327	protein_coding	NM_003551
ASHGV40032638	1.668989	1.668988825	protein_coding	NM_001236
ASHGV40024649	-1.6687	1.668698186	protein_coding	NM_031475
ASHGV40000066	-1.66836	1.668359301	protein_coding	NM_004678
ASHGV40020364	-1.66794	1.667936343	protein_coding	ENST00000591022
ASHGV40022879	-1.6675	1.667496174	protein_coding	NM_001136561
ASHGV40057865	1.667408	1.667408256	protein_coding	NM_014033
ASHGV40040054	-1.66736	1.667363893	protein_coding	NM_001765
ASHGV40010462	1.667342	1.667341862	protein_coding	NM_015054
ASHGV40030028	1.667274	1.667274341	protein_coding	NM_003812
ASHGV40051852	1.666951	1.666951454	protein_coding	NM_017662
ASHGV40018609	1.666924	1.666923552	protein_coding	NM_020982
ASHGV40014643	-1.66661	1.666614678	protein_coding	NM_152263
ASHGV40007802	-1.66625	1.666248786	protein_coding	NM_001243597
ASHGV40015144	-1.66581	1.665813449	protein_coding	uc010aqz.3

ASHGV40020993	-1.66542	1.665422701	protein_coding	NM_025161
ASHGV40028498	-1.66523	1.665234384	protein_coding	NM_001039362
ASHGV40023072	-1.66505	1.66505422	protein_coding	NM_001099790
ASHGV40018498	1.664343	1.664343316	protein_coding	NM_177402
ASHGV40032391	-1.66378	1.663778455	protein_coding	ENST00000600921
ASHGV40015428	1.663592	1.663592061	protein_coding	NM_006668
ASHGV40032723	-1.66272	1.66272499	protein_coding	NM_000394
ASHGV40013949	1.662636	1.662635831	protein_coding	NM_021914
ASHGV40026877	1.662337	1.662337341	protein_coding	NM_000945
ASHGV40009504	1.662113	1.662113456	protein_coding	NM_016497
ASHGV40043276	-1.66208	1.662081099	protein_coding	NM_013974
ASHGV40033068	-1.6618	1.66180428	protein_coding	NM_174932
ASHGV40046113	-1.66093	1.660933218	protein_coding	NM_020144
ASHGV40010145	1.660434	1.660433763	protein_coding	NM_178539
ASHGV40021840	1.660305	1.660304506	protein_coding	NM_016835
ASHGV40015860	-1.66003	1.660028037	protein_coding	NM_000103
ASHGV40016467	1.659889	1.659888781	protein_coding	NM_078474
ASHGV40044720	-1.65919	1.659192302	protein_coding	NM_170610
ASHGV40010871	-1.65903	1.659025912	protein_coding	ENST00000545350
ASHGV40013485	-1.65878	1.658780141	protein_coding	NM_144777
ASHGV40000048	-1.65853	1.658530879	protein_coding	NM_006873
ASHGV40050543	-1.65835	1.658348162	protein_coding	NM_153692
ASHGV40044815	1.65773	1.657730105	protein_coding	NM_007160
ASHGV40016787	-1.65754	1.657541141	protein_coding	NM_002009
ASHGV40014560	1.657376	1.657375955	protein_coding	NM_001144995
ASHGV40051837	-1.65723	1.657232367	protein_coding	NM_001128618
ASHGV40023778	-1.65712	1.657118661	protein_coding	NM_000149
ASHGV40015019	1.657114	1.657113552	protein_coding	NM_001079521
ASHGV40036712	-1.657	1.656999378	protein_coding	NM_000902
ASHGV40033126	-1.65659	1.656593299	protein_coding	NM_031910
ASHGV40025814	-1.65633	1.656325628	protein_coding	NM_000164
ASHGV40048305	1.656296	1.656296202	protein_coding	NM_018844
ASHGV40018451	-1.65616	1.65616214	protein_coding	ENST00000567544
ASHGV40044104	1.65478	1.654779885	protein_coding	NM_005627
ASHGV40024753	1.653951	1.653950598	protein_coding	NM_015215
ASHGV40009553	-1.65342	1.653420686	protein_coding	NM_003480
ASHGV40036858	1.652637	1.652636928	protein_coding	NM_002492
ASHGV40003329	1.652612	1.652611893	protein_coding	NM_080723
ASHGV40006326	-1.6526	1.652601435	protein_coding	NM_005519
ASHGV40057411	-1.65188	1.651880877	protein_coding	ENST00000436041
ASHGV40007384	1.651743	1.651742577	protein_coding	NM_002576
ASHGV40048355	1.651557	1.651556943	protein_coding	NM_006136
ASHGV40035050	-1.65153	1.651527942	protein_coding	NM_001085420
ASHGV40017061	-1.65119	1.65119029	protein_coding	NM_021819
ASHGV40055859	1.650736	1.650735607	protein_coding	NM_012465
ASHGV40017114	1.650652	1.650652431	protein_coding	NM_001130182
ASHGV40037263	-1.65058	1.650582581	protein_coding	NM_006017

ASHGV40013007	-1.65056	1.650555659	protein_coding	NM_024719
ASHGV40050172	1.650433	1.650433379	protein_coding	NM_006257
ASHGV40043631	-1.65033	1.650326393	protein_coding	NM_021073
ASHGV40057725	-1.65032	1.650315835	protein_coding	ENST00000468317
ASHGV40057393	-1.65015	1.650151968	protein_coding	NM_198495
ASHGV40015995	1.649846	1.649845986	protein_coding	NM_002537
ASHGV40017369	-1.64957	1.649567219	protein_coding	ENST00000558188
ASHGV40029798	-1.64931	1.649313302	protein_coding	NM_002148
ASHGV40035018	1.648756	1.648756341	protein_coding	NM_173653
ASHGV40011905	-1.64797	1.647973702	protein_coding	NM_017915
ASHGV40016954	1.647164	1.647163621	protein_coding	NM_002755
ASHGV40013847	-1.64701	1.647013039	protein_coding	NM_002471
ASHGV40051738	1.646915	1.64691468	protein_coding	NM_003028
ASHGV40055641	-1.64669	1.646685606	protein_coding	NM_013230
ASHGV40027325	1.646592	1.646591556	protein_coding	NM_182528
ASHGV40049329	1.646326	1.646325671	protein_coding	NM_017813
ASHGV40048853	-1.64612	1.646122703	protein_coding	NM_152251
ASHGV40017081	-1.64607	1.646071893	protein_coding	ENST00000543887
ASHGV40051079	-1.64604	1.64604355	protein_coding	NM_177531
ASHGV40022936	-1.64559	1.645586791	protein_coding	ENST00000581862
ASHGV40052022	-1.64521	1.645212727	protein_coding	NM_000507
ASHGV40006141	-1.64497	1.644970052	protein_coding	NM_182517
ASHGV40044137	-1.64467	1.644671339	protein_coding	NM_052962
ASHGV40002618	-1.64449	1.64449276	protein_coding	NM_031310
ASHGV40022741	-1.64446	1.644456712	protein_coding	NM_024430
ASHGV40034664	1.643836	1.643835991	protein_coding	NM_014820
ASHGV40052163	1.643192	1.643192415	protein_coding	NM_014334
ASHGV40045455	-1.64317	1.643167656	protein_coding	NM_001004317
ASHGV40020701	-1.64303	1.643031979	protein_coding	NM_000334
ASHGV40031497	-1.64276	1.642760823	protein_coding	NM_006892
ASHGV40000732	-1.64233	1.642325719	protein_coding	ENST00000432515
ASHGV40057563	-1.64108	1.641084207	protein_coding	NM_000976
ASHGV40010683	-1.64103	1.641033168	protein_coding	NM_001002
ASHGV40044780	-1.64039	1.640390711	protein_coding	NM_021968
ASHGV40027049	-1.64005	1.640050101	protein_coding	NM_198174
ASHGV40027726	1.639507	1.639507346	protein_coding	NM_004882
ASHGV40025909	-1.6391	1.639096721	protein_coding	ENST00000602157
ASHGV40013977	1.638615	1.638615126	protein_coding	NM_003944
ASHGV40035424	-1.63845	1.638445844	protein_coding	NM_198152
ASHGV40010044	1.638323	1.63832336	protein_coding	NM_001098815
ASHGV40005737	1.638131	1.638130524	protein_coding	NM_152709
ASHGV40021156	1.637873	1.637872675	protein_coding	NM_002798
ASHGV40020306	-1.63715	1.637145655	protein_coding	NM_031960
ASHGV40053322	1.6371	1.637100445	protein_coding	NM_001860
ASHGV40028271	-1.63672	1.636718028	protein_coding	NM_001525
ASHGV40015949	1.636518	1.636517549	protein_coding	NM_134261
ASHGV40019275	-1.63612	1.636122418	protein_coding	NM_018296

ASHGV40057504	-1.63568	1.635684086	protein_coding	NM_020124
ASHGV40008472	-1.63532	1.635315938	protein_coding	NM_001004728
ASHGV40027924	1.635043	1.635043035	protein_coding	NM_015049
ASHGV40014533	-1.63495	1.634949017	protein_coding	ENST00000555187
ASHGV40040821	1.63487	1.634869626	protein_coding	NM_002715
ASHGV40038663	-1.63486	1.63485944	protein_coding	NM_022346
ASHGV40005643	-1.63446	1.634462895	protein_coding	ENST00000595931
ASHGV40051421	-1.63436	1.634357499	protein_coding	NM_138431
ASHGV40021574	-1.63246	1.632459599	protein_coding	NM_173847
ASHGV40030423	-1.63207	1.632067138	protein_coding	NM_153269
ASHGV40047070	1.631839	1.631839467	protein_coding	NM_005000
ASHGV40050831	1.631454	1.631454303	protein_coding	NM_001039844
ASHGV40047731	1.631424	1.63142383	protein_coding	NM_017549
ASHGV40033521	-1.63119	1.631189725	protein_coding	NM_145172
ASHGV40037468	1.630359	1.630358531	protein_coding	NM_000809
ASHGV40034749	-1.62994	1.629937083	protein_coding	NM_001085357
ASHGV40005349	-1.6295	1.629502473	protein_coding	NM_001039702
ASHGV40012957	-1.62926	1.62925806	protein_coding	NM_001303110
ASHGV40054825	-1.62825	1.628251242	protein_coding	NM_018055
ASHGV40015099	-1.62812	1.628123419	protein_coding	NM_001878
ASHGV40046220	-1.62719	1.627191384	protein_coding	NM_000474
ASHGV40010658	1.626715	1.626714637	protein_coding	NM_016281
ASHGV40052535	1.626309	1.626309434	protein_coding	NM_001128228
ASHGV40049764	1.625804	1.625803833	protein_coding	NM_178565
ASHGV40044097	-1.62541	1.625411868	protein_coding	NM_078488
ASHGV40024614	-1.62435	1.624349334	protein_coding	NM_000148
ASHGV40029954	1.624335	1.624334645	protein_coding	NM_006226
ASHGV40040272	-1.62379	1.623794431	protein_coding	NM_001017992
ASHGV40011136	1.623473	1.623473206	protein_coding	NM_017435
ASHGV40031129	1.622894	1.622893706	protein_coding	NM_002792
ASHGV40049425	1.622595	1.622594732	protein_coding	NM_006837
ASHGV40014138	1.622266	1.622266258	protein_coding	NM_021136
ASHGV40014157	-1.62211	1.622110883	protein_coding	NM_053055
ASHGV40037766	-1.6219	1.621903814	protein_coding	ENST00000504213
ASHGV40010673	-1.6211	1.621096221	protein_coding	uc021rep.1
ASHGV40041095	1.620826	1.620826393	protein_coding	NM_021911
ASHGV40024757	-1.62076	1.620761648	protein_coding	NM_033341
ASHGV40024098	1.620429	1.620429196	protein_coding	NM_001492
ASHGV40031595	-1.62012	1.620115099	protein_coding	NM_030919
ASHGV40011547	-1.61996	1.619961234	protein_coding	NM_005538
ASHGV40053822	1.619946	1.619945921	protein_coding	uc004cyw.3
ASHGV40043873	1.619939	1.619938935	protein_coding	NM_133494
ASHGV40024192	1.619636	1.619636107	protein_coding	NM_006003
ASHGV40029255	-1.61927	1.619269047	protein_coding	NM_003853
ASHGV40005555	1.619174	1.619174381	protein_coding	NM_020975
ASHGV40001774	1.618929	1.618928622	protein_coding	NM_022003
ASHGV40052995	1.61887	1.618870305	protein_coding	NM_033305

ASHGV40057034	1.61869	1.6186904	protein_coding	NM_181886
ASHGV40012828	-1.61829	1.618287969	protein_coding	NM_001922
ASHGV40056133	1.617936	1.617936156	protein_coding	ENST00000553811
ASHGV40041450	-1.61728	1.617277663	protein_coding	NM_005149
ASHGV40025561	-1.61714	1.617139123	protein_coding	NM_005971
ASHGV40045336	1.616879	1.616878661	protein_coding	NM_001009994
ASHGV40003299	-1.61676	1.616759653	protein_coding	NM_021182
ASHGV40020566	-1.61638	1.616376997	protein_coding	NM_017643
ASHGV40000259	-1.61631	1.616309473	protein_coding	NM_001145785
ASHGV40028809	1.615977	1.615976618	protein_coding	NM_014284
ASHGV40026420	-1.61584	1.615838607	protein_coding	NM_145260
ASHGV40043204	-1.61551	1.615512455	protein_coding	NM_001005226
ASHGV40035927	1.615511	1.615510567	protein_coding	NM_015933
ASHGV40027823	-1.61483	1.614828537	protein_coding	NM_006287
ASHGV40026059	-1.61481	1.61480684	protein_coding	NM_012314
ASHGV40044131	1.614438	1.61443792	protein_coding	NM_003980
ASHGV40039046	-1.61413	1.614129294	protein_coding	NM_001133
ASHGV40010187	-1.61389	1.613889806	protein_coding	NM_001292024
ASHGV40055244	-1.61327	1.613269464	protein_coding	NM_001164416
ASHGV40008458	-1.61325	1.61325333	protein_coding	NM_001005212
ASHGV40057674	-1.61318	1.613178437	protein_coding	NM_022573
ASHGV40028302	1.613026	1.613025523	protein_coding	NM_004544
ASHGV40027682	-1.61249	1.612493765	protein_coding	NM_001085447
ASHGV40040815	1.612482	1.61248153	protein_coding	NM_015082
ASHGV40011444	1.612057	1.61205749	protein_coding	NM_018457
ASHGV40019865	-1.61186	1.611858351	protein_coding	NM_017533
ASHGV40008193	1.611841	1.611840611	protein_coding	NM_005256
ASHGV40052210	1.611784	1.61178364	protein_coding	NM_015404
ASHGV40044128	-1.61122	1.611221147	protein_coding	NM_138419
ASHGV40048718	1.610899	1.610899062	protein_coding	NM_130797
ASHGV40024592	1.610677	1.610676527	protein_coding	NM_003706
ASHGV40049573	-1.60995	1.609954832	protein_coding	uc022axh.1
ASHGV40033943	-1.6098	1.609799991	protein_coding	NM_000564
ASHGV40003339	1.609519	1.609519457	protein_coding	NM_153456
ASHGV40022831	1.609291	1.609291343	protein_coding	NM_004539
ASHGV40043259	1.609115	1.609115233	protein_coding	NM_005514
ASHGV40056764	-1.60899	1.608988033	protein_coding	uc002wsw.2
ASHGV40042770	1.608648	1.608648468	protein_coding	NM_006650
ASHGV40055223	1.60839	1.608389615	protein_coding	NM_014710
ASHGV40045574	1.608389	1.608389293	protein_coding	NM_152729
ASHGV40015493	1.607852	1.60785203	protein_coding	NM_001376
ASHGV40000171	-1.60781	1.607814683	protein_coding	NM_001782
ASHGV40005219	1.60775	1.60775039	protein_coding	NM_002627
ASHGV40028159	-1.60761	1.607610921	protein_coding	NM_020161
ASHGV40013317	1.607569	1.607568858	protein_coding	NM_001079673
ASHGV40055558	-1.60737	1.607368	protein_coding	NM_014370
ASHGV40033815	1.607065	1.607064709	protein_coding	NM_015350

ASHGV40033208	-1.60703	1.607029893	protein_coding	NM_005008
ASHGV40027971	1.605992	1.605991669	protein_coding	NM_005006
ASHGV40034456	-1.60571	1.605705451	protein_coding	NM_130387
ASHGV40028736	1.605596	1.605596367	protein_coding	NM_012413
ASHGV40039751	1.605127	1.605127221	protein_coding	NM_001151
ASHGV40015286	-1.6051	1.605096244	protein_coding	NM_052938
ASHGV40031321	1.604948	1.604947507	protein_coding	NM_001282550
ASHGV40016740	1.60418	1.604180112	protein_coding	NM_004048
ASHGV40020166	-1.60358	1.603578116	protein_coding	NM_207454
ASHGV40004989	1.6034	1.603399712	protein_coding	NM_003750
ASHGV40036889	-1.60257	1.602569994	protein_coding	NM_032047
ASHGV40039367	-1.60249	1.602490127	protein_coding	NM_031291
ASHGV40008756	-1.60216	1.602156156	protein_coding	NM_001130058
ASHGV40029467	-1.60192	1.601919034	protein_coding	ENST00000436605
ASHGV40011143	-1.60189	1.601892409	protein_coding	NM_030572
ASHGV40034915	1.600384	1.600383557	protein_coding	NM_130808
ASHGV40037444	1.599872	1.599871773	protein_coding	NM_006095
ASHGV40011262	1.597284	1.597284217	protein_coding	NM_001843
ASHGV40044078	-1.59708	1.597083039	protein_coding	NM_001017373
ASHGV40024445	-1.59684	1.596843658	protein_coding	NM_198477
ASHGV40018083	-1.59591	1.59590858	protein_coding	ENST00000564533
ASHGV40044999	-1.59563	1.595629608	protein_coding	uc003oob.1
ASHGV40022682	-1.59563	1.595625052	protein_coding	NM_001001964
ASHGV40054969	-1.59479	1.594787931	protein_coding	NM_001477
ASHGV40021740	1.594457	1.594457474	protein_coding	NM_003152
ASHGV40037189	1.593116	1.593115686	protein_coding	NM_144720
ASHGV40050944	-1.59307	1.593072814	protein_coding	ENST00000391680
ASHGV40049284	1.592891	1.592890615	protein_coding	NM_014682
ASHGV40011473	-1.59225	1.592247018	protein_coding	NM_031157
ASHGV40029188	-1.59136	1.591364836	protein_coding	NM_138800
ASHGV40026528	-1.59133	1.591333627	protein_coding	ENST00000379677
ASHGV40001216	-1.59123	1.59122977	protein_coding	ENST00000456614
ASHGV40043170	-1.59052	1.590516045	protein_coding	NM_003541
ASHGV40014997	1.589892	1.589891985	protein_coding	NM_002806
ASHGV40048562	-1.58967	1.589667567	protein_coding	NM_006027
ASHGV40031077	1.58965	1.589650221	protein_coding	NM_014372
ASHGV40027759	1.58926	1.589260387	protein_coding	NM_006164
ASHGV40045744	1.588969	1.588968696	protein_coding	NM_015439
ASHGV40027896	-1.58886	1.588856165	protein_coding	NM_033030
ASHGV40011443	-1.58881	1.588806968	protein_coding	NM_020547
ASHGV40008946	1.587836	1.587835681	protein_coding	NM_018480
ASHGV40038635	-1.58642	1.586415719	protein_coding	uc003gny.1
ASHGV40025427	1.586356	1.586355881	protein_coding	NM_001242680
ASHGV40009932	-1.58578	1.585777947	protein_coding	NM_001008223
ASHGV40049323	1.58556	1.585560008	protein_coding	NM_001135690
ASHGV40027028	1.58547	1.585469501	protein_coding	NM_003896
ASHGV40046146	-1.58546	1.585464001	protein_coding	NM_016343

ASHGV40006252	-1.58536	1.585357734	protein_coding	NM_005396
ASHGV40048909	1.583595	1.583594563	protein_coding	NM_001908
ASHGV40025557	1.58281	1.582810156	protein_coding	NM_001037
ASHGV40044651	-1.58244	1.582438959	protein_coding	NM_001190766
ASHGV40051786	1.582214	1.582213941	protein_coding	NM_173496
ASHGV40042461	-1.58136	1.581362965	protein_coding	NM_032088
ASHGV40028246	-1.58106	1.581056905	protein_coding	NM_001485
ASHGV40021220	-1.58055	1.580549696	protein_coding	NM_001102614
ASHGV40057644	-1.58038	1.580382345	protein_coding	NM_016024
ASHGV40036216	-1.58013	1.58012539	protein_coding	NM_005233
ASHGV40008432	-1.57989	1.579885517	protein_coding	NM_001005205
ASHGV40017120	-1.5796	1.579602773	protein_coding	NM_002789
ASHGV40025376	-1.57953	1.579530409	protein_coding	NM_001066
ASHGV40006725	1.579131	1.579130691	protein_coding	NM_001243728
ASHGV40002143	-1.57859	1.578587839	protein_coding	ENST00000556949
ASHGV40003311	-1.57802	1.578017673	protein_coding	NM_025145
ASHGV40051577	-1.57794	1.577937771	protein_coding	NM_021002
ASHGV40040850	-1.5779	1.57790222	protein_coding	NM_020389
ASHGV40022421	1.577902	1.577901946	protein_coding	NM_014646
ASHGV40054293	-1.57743	1.577432061	protein_coding	NM_000354
ASHGV40016690	-1.57733	1.577333114	protein_coding	NM_019074
ASHGV40012301	1.577292	1.57729176	protein_coding	NM_001142684
ASHGV40053540	-1.57721	1.577206353	protein_coding	NM_021619
ASHGV40010605	1.577206	1.577205923	protein_coding	NM_000970
ASHGV40016699	1.577085	1.57708519	protein_coding	NM_006293
ASHGV40027696	1.576578	1.576577986	protein_coding	NM_003705
ASHGV40010493	1.576274	1.576273786	protein_coding	NM_000277
ASHGV40033756	-1.57624	1.576237872	protein_coding	NM_138435
ASHGV40013750	-1.57613	1.576129807	protein_coding	NM_004436
ASHGV40045989	-1.57591	1.57590766	protein_coding	ENST00000597278
ASHGV40035092	-1.57579	1.575785617	protein_coding	NM_174878
ASHGV40025302	-1.57551	1.575510167	protein_coding	NM_016270
ASHGV40034298	-1.57523	1.57522712	protein_coding	NM_001789
ASHGV40014172	-1.57422	1.574215596	protein_coding	NM_145171
ASHGV40028133	1.573729	1.573729261	protein_coding	NM_006216
ASHGV40007735	-1.57334	1.573337573	protein_coding	ENST00000595283
ASHGV40047171	1.572663	1.572662808	protein_coding	NM_001628
ASHGV40015055	-1.57256	1.572557217	protein_coding	NM_016651
ASHGV40040163	-1.57249	1.572491832	protein_coding	NM_173489
ASHGV40014231	-1.57241	1.572407124	protein_coding	NM_002016
ASHGV40028489	1.572359	1.572359149	protein_coding	NM_002149
ASHGV40046729	1.572055	1.572055375	protein_coding	NM_014510
ASHGV40048850	-1.57145	1.571452877	protein_coding	NM_016512
ASHGV40015757	1.571451	1.571451391	protein_coding	NM_016013
ASHGV40056226	1.571294	1.571293558	protein_coding	ENST00000326592
ASHGV40010923	1.57083	1.570829972	protein_coding	NM_019854
ASHGV40014981	1.57079	1.570789966	protein_coding	NM_001206673

ASHGV40042402	-1.57073	1.570732053	protein_coding	NM_144696
ASHGV40012518	-1.56991	1.569905045	protein_coding	NM_024058
ASHGV40010002	1.569457	1.569456528	protein_coding	NM_032840
ASHGV40014034	1.569446	1.569445721	protein_coding	uc001wwz.3
ASHGV40030971	-1.56938	1.569377419	protein_coding	NM_000961
ASHGV40006049	-1.56904	1.569040015	protein_coding	NM_001009997
ASHGV40015712	1.568964	1.568964061	protein_coding	NM_005739
ASHGV40039083	1.568617	1.56861652	protein_coding	NM_001130016
ASHGV40046925	1.567878	1.567878196	protein_coding	NM_182691
ASHGV40028318	1.566962	1.566962061	protein_coding	NM_016552
ASHGV40011211	-1.56685	1.566847986	protein_coding	NM_001080509
ASHGV40033415	-1.56639	1.566389897	protein_coding	NM_003504
ASHGV40053672	1.566199	1.56619903	protein_coding	NM_007327
ASHGV40030484	1.566006	1.566006069	protein_coding	NM_000678
ASHGV40040635	1.565823	1.565822739	protein_coding	NM_001163315
ASHGV40015351	-1.56567	1.56567038	protein_coding	ENST00000557646
ASHGV40050336	1.565604	1.565604281	protein_coding	NM_001693
ASHGV40022180	-1.56553	1.565527212	protein_coding	NM_001278587
ASHGV40043138	-1.56546	1.565458565	protein_coding	NM_003526
ASHGV40006707	1.565258	1.565257984	protein_coding	NM_000352
ASHGV40044827	-1.56507	1.565067751	protein_coding	NM_001286633
ASHGV40035897	-1.56473	1.564732466	protein_coding	NM_000579
ASHGV40036662	1.564215	1.564215304	protein_coding	NM_007282
ASHGV40056055	-1.56356	1.563564226	protein_coding	NM_032229
ASHGV40047628	-1.56355	1.563547766	protein_coding	NM_004289
ASHGV40012113	-1.56348	1.563478248	protein_coding	NM_001047980
ASHGV40001624	-1.56283	1.562829017	protein_coding	ENST00000514667
ASHGV40057062	-1.56281	1.562811777	protein_coding	NM_032136
ASHGV40023917	-1.56262	1.562619934	protein_coding	NM_001611
ASHGV40054481	1.562481	1.562481086	protein_coding	NM_005369
ASHGV40002028	-1.56213	1.562128853	protein_coding	NM_001195082
ASHGV40009201	-1.56164	1.561636557	protein_coding	NM_019894
ASHGV40043995	1.561436	1.561435914	protein_coding	NM_021648
ASHGV40026912	-1.56133	1.561328546	protein_coding	NM_173535
ASHGV40054173	1.561187	1.561186738	protein_coding	NM_004867
ASHGV40025902	-1.56084	1.560838928	protein_coding	NM_014419
ASHGV40044872	-1.56073	1.560729202	protein_coding	ENST00000375880
ASHGV40016729	1.560584	1.560584209	protein_coding	NM_001015001
ASHGV40057433	-1.56045	1.560451954	protein_coding	NM_001039361
ASHGV40038815	1.56023	1.560229817	protein_coding	NM_004181
ASHGV40025348	1.560132	1.560131849	protein_coding	NM_015016
ASHGV40048426	-1.55935	1.559349297	protein_coding	NM_020369
ASHGV40032222	1.559227	1.559226758	protein_coding	NM_004414
ASHGV40043516	1.559058	1.559057707	protein_coding	NM_001012974
ASHGV40040525	1.558899	1.558898603	protein_coding	NM_002397
ASHGV40052182	-1.5586	1.558601853	protein_coding	ENST00000318737
ASHGV40047331	-1.55858	1.558579148	protein_coding	NM_005431

ASHGV40008210	1.558114	1.558114103	protein_coding	NM_003986
ASHGV40003243	-1.55764	1.557637522	protein_coding	NM_002171
ASHGV40021604	-1.5576	1.557599305	protein_coding	NM_017559
ASHGV40026320	1.557122	1.557121563	protein_coding	NM_004763
ASHGV40009900	-1.55696	1.556961823	protein_coding	NM_001004134
ASHGV40056848	-1.55596	1.555961753	protein_coding	NM_001144931
ASHGV40051707	1.555086	1.555085949	protein_coding	NM_006289
ASHGV40042699	-1.55419	1.554191752	protein_coding	NM_001102609
ASHGV40034498	1.553936	1.553936147	protein_coding	NM_025075
ASHGV40020262	1.553854	1.553854498	protein_coding	NM_032339
ASHGV40023158	-1.5536	1.553603371	protein_coding	NM_032525
ASHGV40029773	-1.55358	1.553576502	protein_coding	NM_016653
ASHGV40012495	1.553511	1.553511212	protein_coding	uc001elk.2
ASHGV40050528	1.552835	1.552835217	protein_coding	NM_004874
ASHGV40026418	-1.55264	1.552640407	protein_coding	NM_001002006
ASHGV40047275	-1.55118	1.551178847	protein_coding	NM_183062
ASHGV40040257	1.548791	1.548790916	protein_coding	NM_002184
ASHGV40013343	1.548528	1.548528473	protein_coding	NM_001242312
ASHGV40018763	1.548152	1.548151705	protein_coding	NM_024847
ASHGV40014675	1.54814	1.548140081	protein_coding	NM_012405
ASHGV40006027	-1.54796	1.547956996	protein_coding	NM_019084
ASHGV40017705	-1.54774	1.547743032	protein_coding	NM_003361
ASHGV40041607	-1.5474	1.547401937	protein_coding	NM_001136107
ASHGV40034459	-1.54704	1.547035801	protein_coding	NM_198564
ASHGV40045902	-1.547	1.547000607	protein_coding	NM_001286229
ASHGV40056649	-1.54645	1.546446609	protein_coding	NM_016347
ASHGV40053886	-1.54644	1.546440002	protein_coding	NM_203408
ASHGV40055458	-1.54636	1.546359215	protein_coding	ENST00000449283
ASHGV40010562	-1.54628	1.546275332	protein_coding	NM_213596
ASHGV40012936	1.546147	1.546147448	protein_coding	NM_002312
ASHGV40055816	-1.54597	1.545970526	protein_coding	NM_014391
ASHGV40035247	-1.54591	1.54590884	protein_coding	NM_198407
ASHGV40041147	1.545745	1.545744865	protein_coding	NM_005565
ASHGV40017829	1.54531	1.545309737	protein_coding	NM_003586
ASHGV40030756	-1.54508	1.545083052	protein_coding	NM_080675
ASHGV40053475	-1.54491	1.544911907	protein_coding	NM_001012502
ASHGV40057861	1.544272	1.544272421	protein_coding	NM_001659
ASHGV40007768	-1.54403	1.544032646	protein_coding	NM_001005187
ASHGV40032254	1.543988	1.543987975	protein_coding	NM_002240
ASHGV40031331	-1.54394	1.54393753	protein_coding	NM_018327
ASHGV40007145	-1.54375	1.543752723	protein_coding	uc009ypx.3
ASHGV40022078	1.543737	1.543737127	protein_coding	NM_002805
ASHGV40037119	-1.54354	1.543544728	protein_coding	NM_001131034
ASHGV40025610	1.542918	1.542917883	protein_coding	NM_001749
ASHGV40055788	-1.54261	1.542614415	protein_coding	NM_001281956
ASHGV40057401	1.541567	1.541566657	protein_coding	NM_005614
ASHGV40054729	1.541344	1.541344101	protein_coding	NM_004726

ASHGV40032171	1.54125	1.541250007	protein_coding	NM_032852
ASHGV40012780	1.540917	1.540917478	protein_coding	NM_052910
ASHGV40047824	-1.54076	1.540755273	protein_coding	NM_001100159
ASHGV40042974	1.540752	1.540752378	protein_coding	NM_005563
ASHGV40013299	1.54054	1.540539979	protein_coding	NM_001282460
ASHGV40049975	-1.5403	1.540302958	protein_coding	NM_001045556
ASHGV40042663	1.540047	1.540046976	protein_coding	NM_000816
ASHGV40016011	-1.54001	1.54001385	protein_coding	NM_004884
ASHGV40053441	1.539093	1.539092932	protein_coding	NM_030978
ASHGV40006710	1.538841	1.538841392	protein_coding	NM_012139
ASHGV40024411	-1.53875	1.53875033	protein_coding	NM_000764
ASHGV40055786	1.538712	1.53871208	protein_coding	NM_005271
ASHGV40025445	1.538488	1.538487595	protein_coding	ENST00000354585
ASHGV40021727	1.538389	1.538388586	protein_coding	NM_005801
ASHGV40025997	-1.5379	1.537899617	protein_coding	NM_001099694
ASHGV40020772	1.537403	1.537402803	protein_coding	NM_018672
ASHGV40008426	-1.53713	1.537125105	protein_coding	NM_001003750
ASHGV40056464	-1.53711	1.537110015	protein_coding	ENST00000578092
ASHGV40016734	-1.53569	1.535693183	protein_coding	NM_024908
ASHGV40009226	1.53555	1.535550286	protein_coding	NM_014021
ASHGV40045040	-1.5351	1.535097085	protein_coding	NM_005586
ASHGV40039127	1.534803	1.534803076	protein_coding	NM_001263
ASHGV40055660	-1.53467	1.534667794	protein_coding	NM_001005375
ASHGV40054010	-1.53455	1.534552766	protein_coding	NM_001013742
ASHGV40042270	-1.53441	1.534411412	protein_coding	NM_005573
ASHGV40017146	1.534319	1.53431859	protein_coding	NM_014862
ASHGV40021680	1.534188	1.534188421	protein_coding	NM_032192
ASHGV40025767	-1.53375	1.533754269	protein_coding	NM_181845
ASHGV40044988	-1.53355	1.533546855	protein_coding	NM_001243186
ASHGV40015840	1.532735	1.532734814	protein_coding	NM_004236
ASHGV40027994	-1.53225	1.532249165	protein_coding	NM_001166005
ASHGV40030934	-1.53205	1.532052387	protein_coding	NM_001011547
ASHGV40008349	1.531788	1.531787854	protein_coding	uc021qgl.1
ASHGV40048669	-1.53177	1.531774623	protein_coding	NM_018384
ASHGV40041054	-1.53173	1.531729837	protein_coding	NM_031423
ASHGV40043172	-1.53131	1.5313125	protein_coding	NM_003533
ASHGV40004948	1.530818	1.530818047	protein_coding	NM_001001936
ASHGV40021861	1.530402	1.530401562	protein_coding	ENST00000604646
ASHGV40014931	1.530168	1.530168394	protein_coding	NM_005998
ASHGV40020015	-1.52987	1.529865539	protein_coding	NM_001004306
ASHGV40006284	1.5298	1.529799735	protein_coding	NM_201649
ASHGV40022584	-1.52973	1.529729417	protein_coding	NM_172241
ASHGV40024616	1.528686	1.528686221	protein_coding	NM_016246
ASHGV40028248	1.528455	1.528454603	protein_coding	NM_024726
ASHGV40044705	1.52844	1.528440093	protein_coding	NM_001080
ASHGV40057440	-1.52763	1.52762514	protein_coding	NM_001024661
ASHGV40025121	1.527449	1.527448985	protein_coding	NM_024552

ASHGV40006126	-1.5269	1.526897919	protein_coding	NM_001077494
ASHGV40012645	1.526605	1.526604806	protein_coding	NM_022843
ASHGV40060857	-1.52634	1.526344337	protein_coding	uc010aip.1
ASHGV40006529	-1.52606	1.526062446	protein_coding	ENST00000488405
ASHGV40018400	-1.52606	1.526057478	protein_coding	ENST00000538868
ASHGV40055108	1.525153	1.525153489	protein_coding	NM_001866
ASHGV40027862	1.525048	1.525047881	protein_coding	NM_016192
ASHGV40008484	-1.52486	1.524861374	protein_coding	NM_006138
ASHGV40011056	-1.52472	1.524720161	protein_coding	NM_007334
ASHGV40005671	1.524367	1.524367032	protein_coding	NM_018464
ASHGV40005617	1.523646	1.523645592	protein_coding	NM_016257
ASHGV40055059	-1.52347	1.523471788	protein_coding	NM_207320
ASHGV40008546	1.523032	1.52303161	protein_coding	NM_012202
ASHGV40043465	1.522677	1.522677152	protein_coding	NM_018965
ASHGV40054084	-1.52231	1.522314094	protein_coding	NM_152424
ASHGV40031849	1.521411	1.521410654	protein_coding	NM_004738
ASHGV40014480	-1.52132	1.521319612	protein_coding	NM_016186
ASHGV40042285	-1.52101	1.521013005	protein_coding	NM_001257308
ASHGV40028347	-1.52041	1.520408731	protein_coding	NM_005018
ASHGV40037912	-1.5204	1.520400504	protein_coding	NM_003619
ASHGV40017942	1.519979	1.519979493	protein_coding	NM_005880
ASHGV40014351	1.519809	1.519809243	protein_coding	NM_004863
ASHGV40018203	1.519569	1.51956852	protein_coding	NM_001605
ASHGV40024102	1.519345	1.519344596	protein_coding	NM_014884
ASHGV40018902	1.518825	1.518825489	protein_coding	NM_005115
ASHGV40055219	1.518472	1.518472441	protein_coding	NM_080390
ASHGV40055657	-1.51827	1.518271239	protein_coding	uc010nxc.1
ASHGV40041586	-1.51812	1.518122186	protein_coding	NM_018186
ASHGV40023266	-1.51795	1.517949882	protein_coding	NM_177986
ASHGV40015615	1.517946	1.51794562	protein_coding	NM_000462
ASHGV40048932	1.517941	1.517940731	protein_coding	NM_182643
ASHGV40040839	1.517657	1.517657247	protein_coding	NM_004887
ASHGV40026955	1.517213	1.517212522	protein_coding	NM_020362
ASHGV40048965	1.517056	1.517055829	protein_coding	NM_015310
ASHGV40012467	-1.51554	1.51554467	protein_coding	NM_006475
ASHGV40000037	-1.51546	1.515457108	protein_coding	NM_001258031
ASHGV40018956	-1.51471	1.51470554	protein_coding	ENST00000561916
ASHGV40046083	-1.51359	1.513594551	protein_coding	ENST00000382528
ASHGV40044002	1.513181	1.513181125	protein_coding	NM_001085480
ASHGV40023902	-1.51296	1.512959085	protein_coding	NM_182513
ASHGV40020614	1.512583	1.512582567	protein_coding	NM_006924
ASHGV40024875	-1.51166	1.511662632	protein_coding	ENST00000597520
ASHGV40003276	1.510943	1.51094266	protein_coding	NM_006570
ASHGV40032477	1.510593	1.510593083	protein_coding	NM_032291
ASHGV40030752	-1.50975	1.509750676	protein_coding	NM_182584
ASHGV40027146	1.509692	1.509691732	protein_coding	NM_198461
ASHGV40032303	-1.50943	1.509425407	protein_coding	NM_020639

ASHGV40026075	1.509366	1.509365509	protein_coding	NM_032701
ASHGV40018393	-1.50909	1.509090821	protein_coding	NM_001195125
ASHGV40005502	1.508932	1.508931632	protein_coding	NM_001304746
ASHGV40032599	-1.50813	1.50813149	protein_coding	NM_005806
ASHGV40054690	1.507873	1.507872936	protein_coding	NM_002765
ASHGV40005742	1.50766	1.507659615	protein_coding	NM_002727
ASHGV40025063	1.507434	1.507433791	protein_coding	NM_198533
ASHGV40049082	-1.50716	1.507158183	protein_coding	NM_001001914
ASHGV40019364	-1.50696	1.50695964	protein_coding	NM_020995
ASHGV40008858	1.506804	1.506803524	protein_coding	NM_015017
ASHGV40007660	-1.50626	1.506255295	protein_coding	NM_001077639
ASHGV40024569	-1.50499	1.504989854	protein_coding	NM_005628
ASHGV40009740	-1.50464	1.504644534	protein_coding	NM_001080406
ASHGV40007449	-1.5046	1.504596558	protein_coding	NM_148170
ASHGV40011390	1.504013	1.50401315	protein_coding	NM_014191
ASHGV40017473	1.503836	1.503836203	protein_coding	NM_005326
ASHGV40018337	-1.50366	1.503657996	protein_coding	NM_172347
ASHGV40033460	1.503507	1.503506629	protein_coding	NM_003347
ASHGV40008598	1.502547	1.502546575	protein_coding	NM_012094
ASHGV40045518	1.502169	1.50216944	protein_coding	NM_001634
ASHGV40017607	-1.50186	1.50186276	protein_coding	NM_144674
ASHGV40055533	-1.50182	1.501819075	protein_coding	ENST00000370287
ASHGV40017476	-1.50134	1.501344993	protein_coding	NM_001009606
ASHGV40038058	1.500381	1.500380683	protein_coding	NM_030648
ASHGV40019833	1.500119	1.500118871	protein_coding	NM_005964
ASHGV40043288	-1.49977	1.499766807	protein_coding	NM_181842
ASHGV40041825	1.498929	1.498928663	protein_coding	NM_001029875
ASHGV40042277	1.498888	1.498887612	protein_coding	NM_001048252
ASHGV40016079	1.498581	1.498580529	protein_coding	NM_018357
ASHGV40037828	-1.49851	1.498510973	protein_coding	NM_001813
ASHGV40030473	1.49835	1.498350463	protein_coding	NM_001258429
ASHGV40048443	1.498332	1.498331809	protein_coding	NM_001201372
ASHGV40008058	-1.49825	1.498245483	protein_coding	NM_198474
ASHGV40022249	-1.4974	1.497397745	protein_coding	NM_001088
ASHGV40048543	1.497224	1.497223657	protein_coding	NM_001080511
ASHGV40010289	1.497061	1.497060765	protein_coding	NM_020841
ASHGV40031502	-1.49664	1.496637477	protein_coding	NM_174897
ASHGV40010895	-1.49657	1.49657264	protein_coding	NM_030775
ASHGV40025675	-1.49622	1.496218454	protein_coding	NM_172138
ASHGV40022848	-1.49616	1.496161457	protein_coding	NM_133459
ASHGV40019355	-1.49602	1.496015487	protein_coding	NM_052858
ASHGV40005999	-1.49583	1.495833336	protein_coding	NM_148960
ASHGV40028989	1.495271	1.495270839	protein_coding	NM_006196
ASHGV40042418	-1.495	1.495003334	protein_coding	NM_018907
ASHGV40025135	-1.49471	1.494708173	protein_coding	NM_001004699
ASHGV40049184	1.494482	1.494482143	protein_coding	NM_001005471
ASHGV40039190	1.493804	1.493804321	protein_coding	NM_001100427

ASHGV40049241	1.493305	1.493305296	protein_coding	NM_030954
ASHGV40048164	1.493172	1.493171617	protein_coding	NM_004411
ASHGV40009300	-1.49292	1.492924594	protein_coding	uc021qrt.1
ASHGV40030852	1.492911	1.492911102	protein_coding	NM_015035
ASHGV40018056	1.492459	1.492458655	protein_coding	NM_015993
ASHGV40002113	-1.49207	1.492072788	protein_coding	ENST00000555074
ASHGV40043337	1.492027	1.492027213	protein_coding	NM_002118
ASHGV40006066	1.491877	1.491877338	protein_coding	NM_020348
ASHGV40000006	1.491724	1.491723563	protein_coding	NM_020439
ASHGV40034090	1.491636	1.491635754	protein_coding	NM_020345
ASHGV40010903	1.491178	1.49117777	protein_coding	NM_002959
ASHGV40027513	1.4911	1.491100262	protein_coding	NM_001006636
ASHGV40027456	-1.49015	1.490149338	protein_coding	NM_024674
ASHGV40034062	1.490131	1.490130632	protein_coding	NM_014744
ASHGV40000028	-1.49012	1.490123155	protein_coding	NM_001304382
ASHGV40034493	1.489724	1.489724347	protein_coding	NM_003716
ASHGV40009065	1.489525	1.489525076	protein_coding	NM_001165
ASHGV40009526	-1.48943	1.489429082	protein_coding	NM_031491
ASHGV40041860	-1.48908	1.489083356	protein_coding	NM_001038603
ASHGV40043688	1.488827	1.488827004	protein_coding	NM_000186
ASHGV40027011	1.488426	1.488426035	protein_coding	NM_001277053
ASHGV40014787	1.488343	1.488343301	protein_coding	NM_001288746
ASHGV40009896	-1.48832	1.488324792	protein_coding	NM_001844
ASHGV40012366	-1.48822	1.488222428	protein_coding	NM_001007538
ASHGV40054529	-1.48787	1.487865314	protein_coding	NM_001282302
ASHGV40045109	1.48762	1.487619793	protein_coding	NM_014936
ASHGV40030121	-1.48745	1.48745384	protein_coding	NM_198483
ASHGV40032318	-1.48702	1.487018413	protein_coding	NM_001293274
ASHGV40008506	1.486472	1.486471588	protein_coding	NM_024092
ASHGV40006608	-1.48625	1.486245666	protein_coding	NM_003737
ASHGV40055084	1.485582	1.485582271	protein_coding	NM_207422
ASHGV40016750	-1.48553	1.485528009	protein_coding	NM_004212
ASHGV40007454	-1.48481	1.484805737	protein_coding	NM_020358
ASHGV40013751	-1.48473	1.484734372	protein_coding	NM_002929
ASHGV40047772	-1.48459	1.48459391	protein_coding	NM_175064
ASHGV40038876	1.482745	1.482745413	protein_coding	NM_001030
ASHGV40030598	-1.48267	1.482666147	protein_coding	NM_001242671
ASHGV40024835	-1.48246	1.482461558	protein_coding	NM_176820
ASHGV40034194	1.48209	1.482089975	protein_coding	NM_001337
ASHGV40019767	-1.48185	1.48184702	protein_coding	NM_053285
ASHGV40040937	1.481666	1.481665857	protein_coding	NM_000800
ASHGV40024113	-1.48152	1.481524189	protein_coding	NM_025245
ASHGV40018771	1.481337	1.481336928	protein_coding	NM_014711
ASHGV40020691	1.480695	1.480695337	protein_coding	NM_020198
ASHGV40022563	1.480474	1.480473607	protein_coding	uc021pmb.1
ASHGV40042076	1.479557	1.479557077	protein_coding	NM_014412
ASHGV40029036	-1.47934	1.479338255	protein_coding	NM_006636

ASHGV40001601	1.479256	1.479255534	protein_coding	ENST00000511936
ASHGV40049310	-1.47869	1.478685194	protein_coding	ENST00000522186
ASHGV40023961	1.478385	1.47838455	protein_coding	NM_052850
ASHGV40006676	1.478342	1.478342011	protein_coding	NM_032320
ASHGV40028532	-1.47827	1.478269595	protein_coding	ENST00000436967
ASHGV40032800	1.478227	1.478226711	protein_coding	NM_004195
ASHGV40008016	-1.47804	1.478042134	protein_coding	NM_001004754
ASHGV40005375	-1.478	1.477999216	protein_coding	uc010qcq.1
ASHGV40020350	1.477864	1.477863867	protein_coding	NM_012448
ASHGV40047883	1.477696	1.477695729	protein_coding	NM_182546
ASHGV40029178	1.477158	1.477158387	protein_coding	NM_144705
ASHGV40024604	-1.4765	1.476498765	protein_coding	NM_017708
ASHGV40040311	1.476273	1.476272614	protein_coding	NM_001656
ASHGV40027564	1.475878	1.475878457	protein_coding	NM_012097
ASHGV40048445	1.475681	1.475680788	protein_coding	NM_004231
ASHGV40032166	-1.47556	1.47555724	protein_coding	NM_181617
ASHGV40026662	1.475139	1.475139439	protein_coding	NM_133329
ASHGV40038411	1.475042	1.475041643	protein_coding	NM_002796
ASHGV40027665	-1.4748	1.47479541	protein_coding	NM_002977
ASHGV40030882	1.47456	1.474560265	protein_coding	NM_002251
ASHGV40023755	-1.47435	1.474345554	protein_coding	NM_052972
ASHGV40017710	-1.47418	1.474176332	protein_coding	NM_052956
ASHGV40046269	-1.47415	1.474150381	protein_coding	NM_138796
ASHGV40012895	1.474141	1.474140987	protein_coding	NM_004115
ASHGV40005981	-1.47367	1.47367147	protein_coding	NM_006413
ASHGV40053889	1.473206	1.473206133	protein_coding	NM_031442
ASHGV40009499	-1.47319	1.473192652	protein_coding	NM_001038
ASHGV40044710	-1.47303	1.473033179	protein_coding	NM_005686
ASHGV40046907	-1.47275	1.472749532	protein_coding	NM_198999
ASHGV40052270	1.472729	1.472728569	protein_coding	NM_014222
ASHGV40052940	-1.47256	1.472561421	protein_coding	NM_001010940
ASHGV40027894	1.472467	1.472466689	protein_coding	NM_002156
ASHGV40003089	-1.47225	1.472249917	protein_coding	NM_001029886
ASHGV40013884	1.472057	1.47205658	protein_coding	NM_014178
ASHGV40029415	1.472035	1.472035365	protein_coding	NM_002830
ASHGV40033636	-1.47198	1.471977652	protein_coding	NM_000343
ASHGV40000092	-1.47192	1.471915021	protein_coding	ENST00000342888
ASHGV40029902	1.47178	1.471779633	protein_coding	NM_002194
ASHGV40028999	-1.47152	1.47152425	protein_coding	NM_001692
ASHGV40008457	-1.4714	1.471399398	protein_coding	NM_001005186
ASHGV40053212	-1.47128	1.471281283	protein_coding	NM_004612
ASHGV40006919	1.471204	1.471204186	protein_coding	NM_002334
ASHGV40010687	-1.47118	1.471180585	protein_coding	NM_002442
ASHGV40000223	-1.47104	1.471043341	protein_coding	ENST00000399077
ASHGV40009600	-1.47035	1.470353935	protein_coding	NM_007244
ASHGV40021240	1.469865	1.469864752	protein_coding	NM_144607
ASHGV40042817	1.469613	1.469613092	protein_coding	uc031smf.1

ASHGV40042066	-1.46955	1.469546783	protein_coding	NM_152548
ASHGV40003323	-1.46922	1.469215911	protein_coding	NM_033343
ASHGV40031826	-1.46892	1.468923275	protein_coding	NM_012444
ASHGV40006194	-1.46886	1.468863938	protein_coding	ENST00000369519
ASHGV40013091	-1.46862	1.468616703	protein_coding	NM_000231
ASHGV40018604	-1.46851	1.46851281	protein_coding	NM_172229
ASHGV40044728	-1.46829	1.468292533	protein_coding	NM_003538
ASHGV40006551	-1.46771	1.467710881	protein_coding	NM_001004752
ASHGV40020195	-1.46768	1.467683551	protein_coding	NM_152781
ASHGV40015617	1.467642	1.467642259	protein_coding	NM_024490
ASHGV40016263	1.467502	1.467502431	protein_coding	NM_016073
ASHGV40031873	-1.46748	1.467478164	protein_coding	NM_173644
ASHGV40028072	-1.46739	1.467390303	protein_coding	NM_057093
ASHGV40037795	-1.4673	1.467301142	protein_coding	NM_000669
ASHGV40017549	1.467215	1.467214683	protein_coding	NM_001116
ASHGV40026950	1.467112	1.467111588	protein_coding	NM_004082
ASHGV40007759	1.466919	1.466918567	protein_coding	NM_018400
ASHGV40016031	1.466899	1.466899312	protein_coding	NM_024666
ASHGV40030003	-1.46631	1.466306644	protein_coding	NM_012092
ASHGV40048303	1.465982	1.465982206	protein_coding	NM_005295
ASHGV40052583	1.46525	1.465249812	protein_coding	NM_003070
ASHGV40009981	-1.46521	1.465214555	protein_coding	NM_004693
ASHGV40042362	1.465051	1.465050626	protein_coding	NM_001135940
ASHGV40037583	-1.46485	1.46484828	protein_coding	NM_000406
ASHGV40006220	-1.46424	1.464240223	protein_coding	NM_004132
ASHGV40000055	-1.46419	1.464186248	protein_coding	NM_007057
ASHGV40024549	-1.46396	1.463959501	protein_coding	NM_207393
ASHGV40005678	-1.46387	1.463872323	protein_coding	NM_001033081
ASHGV40046793	1.463508	1.463507989	protein_coding	NM_003919
ASHGV40054694	1.463158	1.463158421	protein_coding	NM_021109
ASHGV40049881	1.463142	1.463141666	protein_coding	NM_058229
ASHGV40050675	-1.46313	1.46312577	protein_coding	NM_024522
ASHGV40023073	-1.46302	1.463019589	protein_coding	NM_170695
ASHGV40043346	-1.46273	1.462730813	protein_coding	NM_001163771
ASHGV40040856	1.462671	1.462671107	protein_coding	NM_004598
ASHGV40030491	1.462154	1.46215442	protein_coding	NM_170774
ASHGV40030880	1.46203	1.462030274	protein_coding	NM_006809
ASHGV40054830	1.461718	1.461718207	protein_coding	NM_004615
ASHGV40016720	1.461552	1.46155226	protein_coding	NM_012142
ASHGV40034360	-1.46123	1.46123372	protein_coding	NM_005879
ASHGV40031683	-1.4612	1.461201549	protein_coding	NM_080753
ASHGV40043664	-1.46114	1.46113651	protein_coding	NM_001292009
ASHGV40043767	1.460848	1.460847793	protein_coding	NM_014841
ASHGV40041013	-1.46065	1.460647425	protein_coding	NM_052860
ASHGV40024626	-1.46044	1.460439544	protein_coding	NM_033142
ASHGV40047539	1.460278	1.460278147	protein_coding	NM_006542
ASHGV40005263	-1.46004	1.460042214	protein_coding	NM_001038633

ASHGV40045648	-1.45996	1.459963724	protein_coding	NM_032784
ASHGV40054964	-1.45959	1.459592351	protein_coding	ENST00000407599
ASHGV40054283	-1.45957	1.459570835	protein_coding	ENST00000217926
ASHGV40045976	-1.4594	1.459404005	protein_coding	NM_031949
ASHGV40054259	1.458922	1.458922479	protein_coding	NM_001012978
ASHGV40053865	-1.45873	1.458726991	protein_coding	NM_001017930
ASHGV40038541	-1.45844	1.458435254	protein_coding	NM_005980
ASHGV40019784	1.457726	1.457725699	protein_coding	NM_001438
ASHGV40019829	-1.45771	1.457712876	protein_coding	ENST00000535173
ASHGV40050900	-1.4573	1.457301627	protein_coding	NM_152565
ASHGV40018505	1.457169	1.457168692	protein_coding	NM_024042
ASHGV40010313	1.45705	1.457050065	protein_coding	NM_002480
ASHGV40007189	-1.45668	1.456679698	protein_coding	NM_053054
ASHGV40024562	-1.45661	1.456613879	protein_coding	NM_033258
ASHGV40044741	-1.4564	1.456399233	protein_coding	NM_003532
ASHGV40049098	-1.45632	1.456316942	protein_coding	ENST00000283225
ASHGV40024763	-1.45599	1.455994787	protein_coding	NM_144687
ASHGV40054082	1.455708	1.455707759	protein_coding	NM_015185
ASHGV40045114	1.45567	1.455669872	protein_coding	NM_004277
ASHGV40001421	-1.45546	1.455456922	protein_coding	NM_181334
ASHGV40028885	1.455383	1.455382957	protein_coding	NM_003000
ASHGV40024088	-1.4553	1.455297368	protein_coding	NM_016368
ASHGV40052516	1.455256	1.455255591	protein_coding	NM_153200
ASHGV40046625	1.455017	1.455017074	protein_coding	NM_016038
ASHGV40003091	1.454634	1.454634157	protein_coding	NM_001033088
ASHGV40011500	-1.45461	1.454609698	protein_coding	NM_206899
ASHGV40015666	-1.4541	1.454097929	protein_coding	NM_003037
ASHGV40018353	-1.454	1.454004802	protein_coding	NM_198491
ASHGV40043240	1.45378	1.453780002	protein_coding	NM_005803
ASHGV40019582	-1.45374	1.453741808	protein_coding	NM_013278
ASHGV40024625	1.452502	1.452501661	protein_coding	NM_033377
ASHGV40052300	1.452472	1.452471999	protein_coding	NM_002799
ASHGV40011368	-1.45241	1.452406574	protein_coding	NM_003076
ASHGV40010073	-1.4523	1.45230401	protein_coding	NM_003920
ASHGV40054693	-1.4522	1.452198471	protein_coding	NM_138636
ASHGV40039946	1.451976	1.451976041	protein_coding	NM_001332
ASHGV40055706	1.451909	1.451908563	protein_coding	NM_004660
ASHGV40048008	-1.45142	1.451418296	protein_coding	NM_016328
ASHGV40014729	-1.45093	1.450927112	protein_coding	NM_001145
ASHGV40042355	-1.45033	1.450329591	protein_coding	NM_000358
ASHGV40032881	1.450043	1.450042744	protein_coding	NM_058004
ASHGV40019918	-1.44988	1.449883216	protein_coding	NM_031898
ASHGV40005815	-1.44967	1.44966502	protein_coding	NM_002658
ASHGV40000150	1.449327	1.449327151	protein_coding	uc010ogn.2
ASHGV40011176	1.449114	1.449114029	protein_coding	NM_015633
ASHGV40007396	1.44874	1.448739881	protein_coding	NM_004549
ASHGV40014179	-1.44873	1.448731367	protein_coding	NM_001437

ASHGV40034856	-1.44854	1.448537725	protein_coding	NM_001039783
ASHGV40047300	1.447953	1.447952634	protein_coding	NM_024711
ASHGV40016923	-1.44792	1.447915926	protein_coding	NM_006537
ASHGV40015911	1.447787	1.447787003	protein_coding	NM_017661
ASHGV40001328	1.447552	1.447552186	protein_coding	ENST00000472726
ASHGV40008706	-1.44703	1.447030733	protein_coding	NM_014578
ASHGV40046959	1.446908	1.446908018	protein_coding	NM_182529
ASHGV40035678	-1.44691	1.446906875	protein_coding	NM_001257177
ASHGV40057190	-1.44673	1.446729289	protein_coding	NM_021058
ASHGV40005440	1.446691	1.446690972	protein_coding	NM_021252
ASHGV40024808	1.446248	1.44624794	protein_coding	NM_003180
ASHGV40038866	1.446074	1.446073676	protein_coding	NM_017830
ASHGV40029372	1.44603	1.446030067	protein_coding	NM_020868
ASHGV40031708	-1.44589	1.445893983	protein_coding	NM_030777
ASHGV40013277	1.445485	1.4454846	protein_coding	NM_001010897
ASHGV40051489	1.445269	1.445269109	protein_coding	NM_012416
ASHGV40043811	-1.44483	1.444827341	protein_coding	NM_002043
ASHGV40006729	-1.44424	1.444239009	protein_coding	NM_054030
ASHGV40015614	-1.44415	1.444154604	protein_coding	uc001yyi.1
ASHGV40023907	1.443835	1.443834987	protein_coding	NM_033408
ASHGV40010102	-1.44382	1.443815705	protein_coding	NM_145064
ASHGV40021850	-1.44299	1.442988602	protein_coding	NM_002476
ASHGV40033601	-1.44259	1.442592064	protein_coding	uc003aha.3
ASHGV40053949	1.44237	1.442369639	protein_coding	NM_173794
ASHGV40044120	1.442093	1.442092804	protein_coding	NM_017651
ASHGV40018736	1.441831	1.44183053	protein_coding	NM_033201
ASHGV40023193	1.441345	1.441345412	protein_coding	NM_001010847
ASHGV40016187	-1.43998	1.439984845	protein_coding	NM_006383
ASHGV40042644	-1.43994	1.439943355	protein_coding	NM_004219
ASHGV40054718	1.43992	1.439919976	protein_coding	NM_152581
ASHGV40014514	1.439917	1.439917238	protein_coding	NM_018036
ASHGV40000179	-1.43964	1.439639795	protein_coding	ENST00000380530
ASHGV40008424	-1.43926	1.439262407	protein_coding	NM_001001921
ASHGV40050279	1.439256	1.439255855	protein_coding	uc003wuy.1
ASHGV40034810	1.438925	1.438925201	protein_coding	NM_001292041
ASHGV40052422	1.438266	1.438265649	protein_coding	NM_015046
ASHGV40017946	1.438006	1.438005864	protein_coding	NM_030790
ASHGV40032331	-1.43761	1.437605875	protein_coding	NM_001308491
ASHGV40022749	1.437277	1.437277341	protein_coding	NM_013305
ASHGV40023894	-1.43717	1.437172554	protein_coding	NM_152228
ASHGV40024624	-1.43661	1.436608714	protein_coding	NM_000737
ASHGV40003239	-1.4366	1.436596061	protein_coding	uc004doa.3
ASHGV40035786	1.436402	1.43640228	protein_coding	NM_016300
ASHGV40020139	1.436397	1.436397029	protein_coding	NM_018405
ASHGV40007777	-1.43567	1.435666924	protein_coding	NM_001005196
ASHGV40057392	-1.43534	1.43533735	protein_coding	NM_001001659
ASHGV40006155	1.43412	1.434120122	protein_coding	NM_004832

ASHGV40008811	-1.43379	1.43378998	protein_coding	NM_173042
ASHGV40056377	1.433202	1.433202048	protein_coding	NM_000981
ASHGV40043944	1.433043	1.43304328	protein_coding	NM_003649
ASHGV40053558	-1.43269	1.432694134	protein_coding	NM_207417
ASHGV40016376	-1.43255	1.432550701	protein_coding	NM_003175
ASHGV40044790	-1.43185	1.43184815	protein_coding	NM_006299
ASHGV40016727	1.431794	1.431793516	protein_coding	NM_020990
ASHGV40013585	-1.43161	1.431606838	protein_coding	NM_021059
ASHGV40015513	-1.4315	1.431497317	protein_coding	NM_012337
ASHGV40006599	1.430911	1.430911333	protein_coding	NM_001164
ASHGV40028763	1.430858	1.430858265	protein_coding	NM_152390
ASHGV40014695	-1.43083	1.430832767	protein_coding	NM_001013354
ASHGV40016346	1.430749	1.430749203	protein_coding	NM_002168
ASHGV40023869	-1.43063	1.430630551	protein_coding	NM_031917
ASHGV40023296	1.430263	1.430262875	protein_coding	NM_014268
ASHGV40038917	1.430253	1.430253365	protein_coding	NM_018475
ASHGV40022211	-1.43005	1.430049718	protein_coding	NM_004695
ASHGV40007185	1.429526	1.429525517	protein_coding	NM_031450
ASHGV40041867	1.429369	1.429368551	protein_coding	NM_017411
ASHGV40044332	-1.42908	1.429078541	protein_coding	NM_003058
ASHGV40018921	-1.42902	1.429021729	protein_coding	NM_013292
ASHGV40027908	-1.42896	1.428956785	protein_coding	NM_001269
ASHGV40017078	-1.42883	1.428825032	protein_coding	ENST00000435356
ASHGV40036495	1.428721	1.428720501	protein_coding	NM_172027
ASHGV40048908	-1.42812	1.428116589	protein_coding	ENST00000533405
ASHGV40045370	-1.42735	1.427347338	protein_coding	NM_000573
ASHGV40009506	1.427342	1.427342414	protein_coding	NM_080730
ASHGV40027111	1.427091	1.427090956	protein_coding	NM_016490
ASHGV40037749	1.426113	1.426113367	protein_coding	NM_004827
ASHGV40001178	-1.4259	1.425900756	protein_coding	NM_024534
ASHGV40011749	-1.42587	1.425873323	protein_coding	NM_005593
ASHGV40003165	-1.42514	1.425135483	protein_coding	NM_001199744
ASHGV40025748	-1.42429	1.42428907	protein_coding	NM_199285
ASHGV40024876	-1.42418	1.4241769	protein_coding	NM_001085384
ASHGV40030496	-1.424	1.423999693	protein_coding	NM_144773
ASHGV40025439	-1.42396	1.42395766	protein_coding	NM_001277403
ASHGV40042639	1.423844	1.423843889	protein_coding	NM_000679
ASHGV40053255	1.423615	1.423615349	protein_coding	NM_080546
ASHGV40040098	1.423397	1.423396569	protein_coding	NM_002432
ASHGV40036065	1.422659	1.422658929	protein_coding	NM_144642
ASHGV40019667	-1.42256	1.422558155	protein_coding	NM_001164407
ASHGV40034279	-1.42203	1.422027858	protein_coding	NM_001205271
ASHGV40053707	1.421634	1.421633942	protein_coding	uc004cpe.1
ASHGV40025155	1.421417	1.421417221	protein_coding	NM_024292
ASHGV40049436	-1.42119	1.421191514	protein_coding	NM_030958
ASHGV40049688	1.420453	1.420453162	protein_coding	NM_004374
ASHGV40042670	1.420451	1.420451249	protein_coding	NM_013283

ASHGV40055979	-1.42	1.420002651	protein_coding	NM_001037671
ASHGV40056832	-1.41968	1.419679446	protein_coding	NM_014406
ASHGV40024115	-1.41968	1.419676932	protein_coding	NM_016573
ASHGV40007092	1.419542	1.419541946	protein_coding	NM_001079559
ASHGV40037590	1.419266	1.419266429	protein_coding	NM_001031732
ASHGV40054499	1.41902	1.419020484	protein_coding	NM_173078
ASHGV40048238	-1.41879	1.418787695	protein_coding	NM_003386
ASHGV40020841	-1.41855	1.418546209	protein_coding	NM_001258015
ASHGV40028245	-1.41852	1.41851844	protein_coding	ENST00000409661
ASHGV40024636	-1.41845	1.418449274	protein_coding	NM_001024598
ASHGV40020620	-1.41821	1.418205414	protein_coding	NM_017777
ASHGV40039772	1.416805	1.416804574	protein_coding	NM_144772
ASHGV40009714	1.416705	1.41670484	protein_coding	NM_033360
ASHGV40016347	1.416273	1.416272763	protein_coding	NM_006384
ASHGV40049083	-1.41618	1.416177432	protein_coding	NM_001100916
ASHGV40016171	1.41599	1.415990493	protein_coding	NM_024776
ASHGV40007790	1.415636	1.415635851	protein_coding	NM_152722
ASHGV40008745	-1.41547	1.415467596	protein_coding	NM_015973
ASHGV40024450	-1.41542	1.415420054	protein_coding	NM_182707
ASHGV40019420	1.415418	1.415417952	protein_coding	NM_018975
ASHGV40036315	1.415346	1.415346337	protein_coding	NM_001627
ASHGV40024371	-1.41522	1.415220869	protein_coding	NM_172139
ASHGV40051752	-1.41521	1.415208701	protein_coding	NM_001034842
ASHGV40047027	1.415156	1.415155659	protein_coding	NM_033427
ASHGV40051353	-1.41511	1.415110875	protein_coding	NM_017527
ASHGV40048816	1.415056	1.415056066	protein_coding	NM_033225
ASHGV40032572	-1.41463	1.414632305	protein_coding	NM_181615
ASHGV40031651	-1.41417	1.414167614	protein_coding	NM_000457
ASHGV40039361	-1.41371	1.413710252	protein_coding	NM_024582
ASHGV40050350	1.413694	1.41369388	protein_coding	NM_182795
ASHGV40037806	1.413653	1.413653304	protein_coding	NM_001031723
ASHGV40023753	1.4136	1.413600401	protein_coding	NM_001080400
ASHGV40024820	-1.41335	1.413353386	protein_coding	NM_001190764
ASHGV40027382	1.412629	1.412629177	protein_coding	NM_139343
ASHGV40036008	-1.4125	1.412502746	protein_coding	NM_002217
ASHGV40052481	1.41204	1.412040307	protein_coding	NM_016172
ASHGV40048157	-1.41121	1.411206814	protein_coding	NM_016116
ASHGV40016010	-1.41096	1.410957454	protein_coding	NM_017851
ASHGV40012456	-1.41065	1.410651832	protein_coding	NM_001144981
ASHGV40048062	-1.4098	1.409799846	protein_coding	NM_000072
ASHGV40006016	-1.40979	1.409792643	protein_coding	uc009xus.1
ASHGV40001563	1.409703	1.409703018	protein_coding	NM_003009
ASHGV40022839	-1.40964	1.409642154	protein_coding	NM_052947
ASHGV40036280	1.409623	1.409622739	protein_coding	NM_000701
ASHGV40017681	1.409587	1.409587338	protein_coding	NM_000963
ASHGV40025882	1.40955	1.409549783	protein_coding	NM_006184
ASHGV40034266	1.409011	1.409011062	protein_coding	NM_020347

ASHGV40019311	-1.40809	1.408089721	protein_coding	NM_004360
ASHGV40013133	1.40776	1.407760311	protein_coding	NM_002097
ASHGV40021547	1.40775	1.407750186	protein_coding	NM_032932
ASHGV40050044	-1.40759	1.407591647	protein_coding	NM_005293
ASHGV40002592	-1.40718	1.407176988	protein_coding	ENST00000594218
ASHGV40049619	1.406883	1.406883226	protein_coding	NM_014889
ASHGV40044386	1.406432	1.406432033	protein_coding	NM_016098
ASHGV40020338	1.406298	1.406298409	protein_coding	NM_052935
ASHGV40019778	-1.40546	1.405458037	protein_coding	NM_001671
ASHGV40007753	-1.40544	1.405443746	protein_coding	NM_024769
ASHGV40008299	-1.40508	1.405078554	protein_coding	NM_000448
ASHGV40012399	1.404696	1.404696042	protein_coding	NM_003045
ASHGV40008886	-1.4041	1.4041044	protein_coding	NM_015516
ASHGV40047674	1.403438	1.403437932	protein_coding	NM_002047
ASHGV40057741	-1.40334	1.403338103	protein_coding	NM_001004750
ASHGV40022451	-1.40302	1.403015635	protein_coding	NM_005559
ASHGV40003131	-1.40214	1.402136323	protein_coding	NM_001146157
ASHGV40034401	-1.40205	1.402051659	protein_coding	ENST00000541313
ASHGV40033001	-1.4018	1.401804728	protein_coding	NM_007194
ASHGV40019488	1.401769	1.401768615	protein_coding	NM_001257
ASHGV40013740	1.40169	1.401690421	protein_coding	NM_005561
ASHGV40030503	-1.40168	1.401684854	protein_coding	NM_016486
ASHGV40010712	-1.40151	1.401512503	protein_coding	NM_002150
ASHGV40028166	1.40124	1.401239725	protein_coding	NM_001142644
ASHGV40057828	-1.40075	1.400748367	protein_coding	NM_001005468
ASHGV40026875	1.400358	1.400357776	protein_coding	NM_006333
ASHGV40008158	1.400179	1.400179372	protein_coding	NM_054032
ASHGV40057428	1.400005	1.400004519	protein_coding	ENST00000545648
ASHGV40056927	-1.39985	1.399847076	protein_coding	NM_024508
ASHGV40037761	1.398866	1.39886603	protein_coding	NM_000345
ASHGV40053630	1.398761	1.398761318	protein_coding	NM_020822
ASHGV40016143	1.398673	1.39867298	protein_coding	NM_004255
ASHGV40003358	-1.39859	1.398587545	protein_coding	NM_183058
ASHGV40032623	-1.3985	1.398499268	protein_coding	NM_053277
ASHGV40049968	1.39847	1.398469794	protein_coding	NM_004519
ASHGV40034654	1.39827	1.398269579	protein_coding	NM_000097
ASHGV40048217	-1.39799	1.397990359	protein_coding	NM_012447
ASHGV40010247	1.397911	1.397910632	protein_coding	NM_002849
ASHGV40050870	1.397479	1.397478898	protein_coding	NM_001010924
ASHGV40029355	-1.39734	1.397339646	protein_coding	NM_173842
ASHGV40026478	-1.39714	1.397140392	protein_coding	NM_022552
ASHGV40005401	-1.39702	1.397020075	protein_coding	NM_178161
ASHGV40037793	-1.39691	1.396910509	protein_coding	NM_000667
ASHGV40029859	1.396538	1.396537869	protein_coding	NM_001130445
ASHGV40042847	1.395951	1.395950648	protein_coding	NM_003900
ASHGV40007919	1.395947	1.395947108	protein_coding	NM_001004
ASHGV40035107	-1.39562	1.395622386	protein_coding	NM_001123228

ASHGV40026323	1.395191	1.395190883	protein_coding	NM_006826
ASHGV40029253	-1.3946	1.394597082	protein_coding	NM_016232
ASHGV40038816	1.394581	1.394581384	protein_coding	NM_001112717
ASHGV40020859	1.394404	1.394404305	protein_coding	NM_002086
ASHGV40015731	-1.39405	1.394052848	protein_coding	NM_033503
ASHGV40008868	-1.39403	1.39403412	protein_coding	NM_001235
ASHGV40022334	-1.39377	1.3937663	protein_coding	ENST00000321930
ASHGV40018881	-1.39316	1.393163611	protein_coding	uc009xbj.1
ASHGV40022845	-1.39291	1.392911573	protein_coding	NM_013435
ASHGV40033461	-1.39291	1.392905646	protein_coding	NM_152612
ASHGV40010837	1.391973	1.3919726	protein_coding	NM_144584
ASHGV40052000	-1.39168	1.391680379	protein_coding	NM_017680
ASHGV40016131	1.391641	1.391641283	protein_coding	NM_003612
ASHGV40025126	1.391637	1.391637093	protein_coding	NM_016496
ASHGV40024905	1.391108	1.391108299	protein_coding	NM_014453
ASHGV40009647	1.389859	1.389859012	protein_coding	NM_016312
ASHGV40021966	-1.38969	1.389690056	protein_coding	NM_032559
ASHGV40022633	-1.38956	1.389558666	protein_coding	NM_024422
ASHGV40007989	-1.38953	1.389527399	protein_coding	NM_004314
ASHGV40002246	-1.3895	1.38950023	protein_coding	NM_001001325
ASHGV40045650	-1.38948	1.389481582	protein_coding	NM_001010905
ASHGV40057178	-1.38866	1.388664341	protein_coding	NM_021063
ASHGV40032283	1.388308	1.388307686	protein_coding	NM_001389
ASHGV40057573	-1.38807	1.388068764	protein_coding	ENST00000277491
ASHGV40009917	1.387045	1.387044511	protein_coding	NM_015086
ASHGV40030183	1.386946	1.386946207	protein_coding	NM_152386
ASHGV40043784	1.386869	1.386869403	protein_coding	NM_153816
ASHGV40041021	-1.38682	1.386819794	protein_coding	NM_001447
ASHGV40042799	1.386744	1.386743999	protein_coding	NM_006480
ASHGV40021165	1.386742	1.38674191	protein_coding	NM_001976
ASHGV40029532	-1.38668	1.386684481	protein_coding	NM_001508
ASHGV40007247	-1.38606	1.386063365	protein_coding	NM_022338
ASHGV40001948	1.385173	1.385173283	protein_coding	ENST00000539422
ASHGV40014115	-1.38462	1.384616312	protein_coding	NM_172337
ASHGV40043742	1.384163	1.384163025	protein_coding	NM_022726
ASHGV40011071	1.383949	1.383949135	protein_coding	NM_001310
ASHGV40045260	1.382755	1.382755307	protein_coding	NM_019842
ASHGV40009662	1.382686	1.382685935	protein_coding	NM_018640
ASHGV40056452	-1.38214	1.382138783	protein_coding	NM_001242907
ASHGV40033440	1.38212	1.382120134	protein_coding	NM_024544
ASHGV40012273	-1.38208	1.382077707	protein_coding	uc001ukg.2
ASHGV40027495	1.38187	1.381870316	protein_coding	NM_018557
ASHGV40000130	-1.38187	1.381865706	protein_coding	NM_002966
ASHGV40020646	-1.38135	1.381348165	protein_coding	NM_173083
ASHGV40048990	-1.38135	1.381345357	protein_coding	NM_025232
ASHGV40036492	-1.38119	1.381191341	protein_coding	NM_004526
ASHGV40006491	1.381082	1.381081951	protein_coding	NM_019009

ASHGV40019666	1.380549	1.380548726	protein_coding	NM_006445
ASHGV40012231	-1.37969	1.379689954	protein_coding	NM_007197
ASHGV40028127	1.379655	1.379654565	protein_coding	NM_003469
ASHGV40033254	-1.37963	1.379629656	protein_coding	NM_138415
ASHGV40023800	-1.37954	1.379538408	protein_coding	NM_005490
ASHGV40014479	1.379408	1.379408369	protein_coding	NM_032036
ASHGV40020386	-1.37898	1.378975832	protein_coding	NM_001986
ASHGV40052931	1.378847	1.37884673	protein_coding	NM_004817
ASHGV40053553	1.378574	1.378573548	protein_coding	NM_021226
ASHGV40040570	1.378428	1.378428227	protein_coding	NM_012081
ASHGV40015598	1.3779	1.377900012	protein_coding	NM_144599
ASHGV40029756	1.377768	1.377768022	protein_coding	NM_015530
ASHGV40025721	-1.37739	1.377387848	protein_coding	ENST00000375910
ASHGV40011914	1.37706	1.377059924	protein_coding	NM_003299
ASHGV40055261	-1.37686	1.376856481	protein_coding	NM_194463
ASHGV40039802	1.376716	1.376716028	protein_coding	NM_021948
ASHGV40008421	-1.37619	1.376188656	protein_coding	NM_001004739
ASHGV40001130	-1.37591	1.375909354	protein_coding	NM_001114633
ASHGV40053584	1.375869	1.375869028	protein_coding	NM_000787
ASHGV40009790	1.375752	1.375751907	protein_coding	NM_144973
ASHGV40033979	1.37573	1.375729722	protein_coding	NM_014850
ASHGV40016116	-1.37554	1.375539883	protein_coding	NM_177398
ASHGV40030890	1.375376	1.375376251	protein_coding	NM_014477
ASHGV40020285	-1.37524	1.375237145	protein_coding	NM_019016
ASHGV40018922	1.374981	1.374981469	protein_coding	NM_033102
ASHGV40052531	1.374865	1.374864587	protein_coding	NM_013379
ASHGV40052417	1.374848	1.374847587	protein_coding	NM_005312
ASHGV40029911	-1.37478	1.374784551	protein_coding	NM_001255
ASHGV40016853	-1.37453	1.374527097	protein_coding	NM_198524
ASHGV40017039	-1.37441	1.374414896	protein_coding	NM_001024736
ASHGV40051186	1.373804	1.373803957	protein_coding	NM_005005
ASHGV40008180	1.372824	1.372823514	protein_coding	NM_006410
ASHGV40021699	1.372771	1.37277079	protein_coding	NM_000081
ASHGV40010909	1.372394	1.37239407	protein_coding	NM_000719
ASHGV40030467	1.372346	1.372345577	protein_coding	NM_023935
ASHGV40042193	1.371926	1.371926205	protein_coding	ENST00000361539
ASHGV40026035	1.371925	1.371924934	protein_coding	NM_004542
ASHGV40007146	1.371728	1.371728364	protein_coding	NM_015104
ASHGV40001621	-1.37171	1.371711592	protein_coding	NM_001164469
ASHGV40000114	1.371632	1.371632229	protein_coding	NM_000998
ASHGV40003145	-1.37163	1.37162675	protein_coding	NM_001195597
ASHGV40051535	1.371604	1.371603774	protein_coding	NM_152574
ASHGV40007217	1.371354	1.371353544	protein_coding	NM_000920
ASHGV40022131	1.37117	1.371170203	protein_coding	NM_005465
ASHGV40032813	-1.37088	1.370879433	protein_coding	NM_001005239
ASHGV40025021	-1.37047	1.370473033	protein_coding	NM_001267560
ASHGV40009770	-1.37009	1.370092569	protein_coding	NM_183378

ASHGV40017411	1.369563	1.369562528	protein_coding	NM_183337
ASHGV40040558	1.369401	1.369401462	protein_coding	NM_032042
ASHGV40034652	1.369367	1.369366886	protein_coding	NM_019895
ASHGV40050982	1.368841	1.36884129	protein_coding	NM_178812
ASHGV40049185	-1.36876	1.368759359	protein_coding	NM_030904
ASHGV40011608	1.368458	1.368458337	protein_coding	NM_013254
ASHGV40038077	1.367549	1.367548589	protein_coding	NM_003866
ASHGV40036636	-1.36738	1.367381501	protein_coding	ENST00000422482
ASHGV40054970	-1.36725	1.367250183	protein_coding	NM_001098411
ASHGV40014486	-1.36708	1.367081851	protein_coding	NM_001080451
ASHGV40023954	1.366456	1.366455759	protein_coding	NM_005809
ASHGV40016062	-1.366	1.366000084	protein_coding	NM_005078
ASHGV40043479	1.365525	1.365524833	protein_coding	NM_007162
ASHGV40046900	-1.36445	1.364454653	protein_coding	NM_006989
ASHGV40057607	-1.36376	1.363758255	protein_coding	uc011mnk.2
ASHGV40037441	-1.36374	1.363736082	protein_coding	NM_207406
ASHGV40010811	1.363674	1.363674331	protein_coding	NM_133448
ASHGV40054852	-1.3633	1.363296378	protein_coding	uc009xqj.1
ASHGV40002245	-1.36318	1.363183365	protein_coding	ENST00000562767
ASHGV40032874	-1.36304	1.363039915	protein_coding	NM_015672
ASHGV40055894	1.361884	1.361883912	protein_coding	NM_031212
ASHGV40020958	-1.36184	1.361836382	protein_coding	NM_020649
ASHGV40022976	-1.36161	1.361608998	protein_coding	ENST00000585258
ASHGV40037711	1.361229	1.361228856	protein_coding	NM_031372
ASHGV40037597	-1.36107	1.361073252	protein_coding	NM_001073
ASHGV40011553	1.360997	1.360997044	protein_coding	NM_004990
ASHGV40017800	1.360166	1.360166023	protein_coding	NM_003321
ASHGV40044956	-1.35991	1.359907452	protein_coding	NM_152753
ASHGV40002418	-1.35987	1.359871305	protein_coding	NM_001139
ASHGV40038745	-1.35953	1.359531463	protein_coding	NM_000906
ASHGV40023686	-1.35929	1.359285693	protein_coding	NM_003200
ASHGV40005374	-1.35928	1.359275039	protein_coding	NM_001142308
ASHGV40016636	1.358788	1.358788235	protein_coding	NM_152594
ASHGV40025608	1.358706	1.358705896	protein_coding	NM_001281
ASHGV40054059	1.357925	1.357924571	protein_coding	NM_001190708
ASHGV40021750	1.357719	1.357718736	protein_coding	NM_016437
ASHGV40046049	-1.35738	1.357376917	protein_coding	ENST00000514988
ASHGV40023909	1.357253	1.357252657	protein_coding	NM_000121
ASHGV40003280	-1.35724	1.357239522	protein_coding	NM_012390
ASHGV40020938	-1.35648	1.356477603	protein_coding	NM_033445
ASHGV40005566	1.356315	1.356315042	protein_coding	NM_014188
ASHGV40037093	-1.35617	1.356174892	protein_coding	NM_001145248
ASHGV40048119	1.355964	1.35596351	protein_coding	NM_001287135
ASHGV40035555	1.3559	1.355899887	protein_coding	NM_018184
ASHGV40050195	1.355553	1.35555332	protein_coding	NM_003970
ASHGV40018634	1.355171	1.355171358	protein_coding	NM_001009566
ASHGV40018984	-1.35507	1.35507324	protein_coding	NM_001007544

ASHGV40035394	1.354787	1.354787431	protein_coding	NM_001706
ASHGV40056254	1.354607	1.354607065	protein_coding	NM_001004067
ASHGV40022776	-1.35437	1.354365202	protein_coding	NM_001035005
ASHGV40009922	-1.35418	1.354182314	protein_coding	NM_021044
ASHGV40011584	1.354111	1.354111293	protein_coding	NM_004731
ASHGV40030278	-1.35391	1.353905366	protein_coding	NM_152879
ASHGV40028331	-1.35377	1.3537748	protein_coding	uc002wan.1
ASHGV40015045	1.353678	1.353678465	protein_coding	NM_018477
ASHGV40022105	-1.35353	1.353527909	protein_coding	NM_014405
ASHGV40042216	1.352983	1.352982726	protein_coding	NM_000414
ASHGV40014874	1.35262	1.352620447	protein_coding	NM_001030055
ASHGV40039147	1.352546	1.352545886	protein_coding	NM_017912
ASHGV40030448	-1.35229	1.352292103	protein_coding	NM_006065
ASHGV40020895	1.352244	1.352243598	protein_coding	NM_022066
ASHGV40026980	1.35137	1.351370457	protein_coding	NM_001134745
ASHGV40005872	-1.35127	1.35126509	protein_coding	NM_005411
ASHGV40032841	-1.35119	1.351193456	protein_coding	NM_173793
ASHGV40015518	-1.35091	1.350908726	protein_coding	uc001fuk.3
ASHGV40034506	-1.35085	1.350852944	protein_coding	NM_182920
ASHGV40028161	-1.35071	1.350710734	protein_coding	NM_025243
ASHGV40018865	1.350682	1.350682068	protein_coding	NM_021798
ASHGV40000056	1.350481	1.350480606	protein_coding	ENST00000321830
ASHGV40010474	-1.3502	1.350201687	protein_coding	NM_145913
ASHGV40008939	-1.34967	1.349670545	protein_coding	ENST00000430323
ASHGV40038476	-1.34959	1.349593146	protein_coding	NM_133330
ASHGV40055624	1.349306	1.349306161	protein_coding	NM_007125
ASHGV40024688	-1.34929	1.349286711	protein_coding	NM_015596
ASHGV40006494	1.348972	1.348972474	protein_coding	NM_053005
ASHGV40014478	1.348737	1.34873674	protein_coding	NM_020414
ASHGV40029907	1.348411	1.348411249	protein_coding	NM_014905
ASHGV40018038	1.348386	1.348386224	protein_coding	NM_031885
ASHGV40029800	-1.34824	1.348240291	protein_coding	NM_019558
ASHGV40017704	-1.34818	1.348181946	protein_coding	NM_001007240
ASHGV40045903	-1.34744	1.347443258	protein_coding	NM_032532
ASHGV40042640	1.347201	1.347201214	protein_coding	NM_003314
ASHGV40009842	-1.34703	1.347032916	protein_coding	uc001doc.1
ASHGV40028802	-1.34677	1.346770003	protein_coding	NM_005413
ASHGV40047361	-1.34664	1.346644292	protein_coding	NM_001103176
ASHGV40016599	-1.3465	1.346498084	protein_coding	NM_014783
ASHGV40026603	1.346215	1.346214514	protein_coding	NM_005102
ASHGV40034786	1.346137	1.34613723	protein_coding	NM_001015887
ASHGV40014821	-1.34608	1.346076822	protein_coding	NM_181657
ASHGV40032127	1.346059	1.3460586	protein_coding	NM_015565
ASHGV40047653	1.346057	1.3460566	protein_coding	NM_006024
ASHGV40037722	-1.34574	1.345742812	protein_coding	NM_139076
ASHGV40052972	1.345369	1.3453691	protein_coding	NM_012383
ASHGV40025054	-1.34517	1.345172698	protein_coding	NM_001048201

ASHGV40000169	-1.34497	1.344972755	protein_coding	ENST00000377726
ASHGV40017655	-1.34487	1.344867158	protein_coding	NM_003561
ASHGV40011769	-1.34473	1.344726213	protein_coding	NM_006982
ASHGV40052347	1.344518	1.344517987	protein_coding	NM_025072
ASHGV40017619	-1.34428	1.344276637	protein_coding	NM_002762
ASHGV40034254	-1.34375	1.343751861	protein_coding	NM_181489
ASHGV40055000	1.343665	1.343664687	protein_coding	NM_058163
ASHGV40055860	1.343632	1.343631877	protein_coding	NM_020123
ASHGV40055828	-1.34357	1.343567957	protein_coding	NM_152429
ASHGV40025856	-1.34356	1.343564032	protein_coding	NM_000554
ASHGV40054721	1.343517	1.343516985	protein_coding	NM_001721
ASHGV40002502	1.342943	1.342942667	protein_coding	NM_022006
ASHGV40023034	1.342461	1.34246125	protein_coding	NM_014410
ASHGV40036399	1.342275	1.342274502	protein_coding	NM_002045
ASHGV40043201	-1.34218	1.342181982	protein_coding	NM_001010877
ASHGV40043380	1.342102	1.342101889	protein_coding	NM_001014
ASHGV40011110	1.341308	1.341308203	protein_coding	NM_020300
ASHGV40002303	1.341157	1.341156917	protein_coding	NM_014287
ASHGV40000260	1.341103	1.341103339	protein_coding	NM_080678
ASHGV40034111	-1.34109	1.341094817	protein_coding	NM_001306076
ASHGV40050948	1.340534	1.340533746	protein_coding	NM_004102
ASHGV40019788	1.340342	1.340341957	protein_coding	NM_004489
ASHGV40023849	-1.33986	1.339856681	protein_coding	NM_001005193
ASHGV40015394	-1.33965	1.339651417	protein_coding	NM_032632
ASHGV40000010	-1.33941	1.339414803	protein_coding	NM_001164257
ASHGV40025624	-1.33936	1.339357367	protein_coding	NM_003419
ASHGV40044820	1.339349	1.339349334	protein_coding	NM_002116
ASHGV40009995	-1.33902	1.339015401	protein_coding	NM_015848
ASHGV40054446	-1.33891	1.338907672	protein_coding	NM_021796
ASHGV40002495	-1.33888	1.338880444	protein_coding	ENST00000585422
ASHGV40050833	-1.33858	1.338581887	protein_coding	NM_004133
ASHGV40045490	1.338436	1.338436104	protein_coding	NM_001455
ASHGV40056408	-1.3384	1.338398917	protein_coding	uc010wqd.1
ASHGV40000011	-1.33816	1.338163764	protein_coding	NM_000413
ASHGV40037497	1.337984	1.337984145	protein_coding	NM_001126328
ASHGV40036416	-1.33786	1.337857349	protein_coding	NM_001291949
ASHGV40053340	-1.33746	1.337463529	protein_coding	NM_000608
ASHGV40056047	-1.33688	1.336884585	protein_coding	ENST00000453638
ASHGV40020610	-1.33646	1.336456452	protein_coding	NM_001282544
ASHGV40015018	-1.33629	1.336285611	protein_coding	NM_178229
ASHGV40006806	-1.33588	1.335882471	protein_coding	NM_001304274
ASHGV40055107	-1.33553	1.335532622	protein_coding	NM_003868
ASHGV40025803	1.335244	1.335243631	protein_coding	NM_212550
ASHGV40026099	-1.33516	1.335162224	protein_coding	NM_001277397
ASHGV40047457	-1.33515	1.335152629	protein_coding	NM_001040167
ASHGV40040045	1.334886	1.334886026	protein_coding	NM_016279
ASHGV40032694	-1.33435	1.334345025	protein_coding	NM_058186

ASHGV40018905	1.334039	1.334038927	protein_coding	NM_181718
ASHGV40043031	-1.33396	1.333959434	protein_coding	NM_001242698
ASHGV40013806	1.333783	1.333782695	protein_coding	NM_031314
ASHGV40020965	-1.33361	1.33361215	protein_coding	NM_001291366
ASHGV40015356	-1.33323	1.333232219	protein_coding	NM_023112
ASHGV40016551	1.333006	1.333006067	protein_coding	NM_000810
ASHGV40006666	1.332947	1.332947453	protein_coding	NM_013253
ASHGV40033152	1.332827	1.332826933	protein_coding	NM_198549
ASHGV40038854	1.332562	1.332561609	protein_coding	NM_000812
ASHGV40054269	1.332119	1.332118899	protein_coding	NM_032621
ASHGV40006603	1.331964	1.331963985	protein_coding	NM_012402
ASHGV40033161	-1.33194	1.331935377	protein_coding	NM_001013647
ASHGV40018470	1.33139	1.331390248	protein_coding	NM_024571
ASHGV40019584	1.331149	1.331148671	protein_coding	NM_018252
ASHGV40001786	-1.33082	1.330816145	protein_coding	NM_020643
ASHGV40006641	1.330589	1.330588748	protein_coding	NM_020974
ASHGV40041445	1.330531	1.330530857	protein_coding	NM_020546
ASHGV40018621	-1.33046	1.330464687	protein_coding	NM_012360
ASHGV40008530	1.330402	1.330401956	protein_coding	NM_004183
ASHGV40057286	1.330087	1.330086724	protein_coding	NM_006908
ASHGV40013940	1.330071	1.330071427	protein_coding	NM_022073
ASHGV40027039	1.329777	1.329777139	protein_coding	NM_005667
ASHGV40010321	1.329728	1.32972796	protein_coding	NM_003625
ASHGV40053972	1.329523	1.32952339	protein_coding	NM_005229
ASHGV40030759	1.329391	1.3293907	protein_coding	NM_003098
ASHGV40045082	-1.32924	1.329238536	protein_coding	NM_152732
ASHGV40050573	-1.3283	1.328298485	protein_coding	NM_032336
ASHGV40017834	-1.32799	1.327991034	protein_coding	NM_024307
ASHGV40025010	-1.32795	1.327945585	protein_coding	NM_003775
ASHGV40035910	-1.32785	1.327851033	protein_coding	NM_015175
ASHGV40032336	1.327745	1.327745165	protein_coding	NM_006758
ASHGV40048622	-1.32772	1.327716017	protein_coding	NM_145304
ASHGV40054484	-1.32759	1.327586254	protein_coding	NM_173694
ASHGV40052217	-1.32722	1.327224984	protein_coding	NM_001244
ASHGV40034092	1.327078	1.327077952	protein_coding	NM_000461
ASHGV40054902	-1.32645	1.326447318	protein_coding	NM_001129898
ASHGV40055610	1.326126	1.326126194	protein_coding	NM_001161352
ASHGV40033229	1.326115	1.326115499	protein_coding	NM_001165877
ASHGV40010582	1.326082	1.326082294	protein_coding	NM_001278556
ASHGV40057854	-1.326	1.325996291	protein_coding	NM_021238
ASHGV40015664	1.32589	1.325890351	protein_coding	NM_001277313
ASHGV40054814	1.325782	1.325781904	protein_coding	NM_207119
ASHGV40051904	1.325705	1.32570473	protein_coding	NM_013438
ASHGV40035530	1.325686	1.325685821	protein_coding	NM_001010898
ASHGV40030429	1.325579	1.325579356	protein_coding	NM_001895
ASHGV40020344	1.325542	1.325542474	protein_coding	NM_024119
ASHGV40001305	-1.32539	1.325390396	protein_coding	NM_001010859

ASHGV40046846	1.324868	1.324868478	protein_coding	NM_001185
ASHGV40031387	1.324479	1.324479416	protein_coding	NM_018474
ASHGV40051354	1.324205	1.324204709	protein_coding	NM_016647
ASHGV40039113	1.323986	1.323985788	protein_coding	NM_021204
ASHGV40003295	-1.32392	1.323916484	protein_coding	NM_021049
ASHGV40024807	-1.32316	1.323159443	protein_coding	NM_178837
ASHGV40052079	1.322949	1.322949304	protein_coding	NM_005458
ASHGV40026060	-1.32279	1.322785414	protein_coding	NM_006737
ASHGV40053687	1.322586	1.322586	protein_coding	NM_032477
ASHGV40030960	1.322352	1.322351817	protein_coding	NM_020820
ASHGV40055122	-1.32183	1.321830157	protein_coding	NM_016954
ASHGV40009649	-1.32106	1.321060827	protein_coding	NM_021071
ASHGV40018566	1.320608	1.320608203	protein_coding	NM_014353
ASHGV40031528	1.32046	1.320460405	protein_coding	NM_014183
ASHGV40049966	-1.32035	1.320351731	protein_coding	NM_001080399
ASHGV40010110	1.319935	1.31993512	protein_coding	NM_014770
ASHGV40041328	-1.31981	1.319813744	protein_coding	NM_004237
ASHGV40054057	-1.31949	1.319489159	protein_coding	NM_000032
ASHGV40024232	1.319481	1.319480791	protein_coding	NM_019849
ASHGV40026087	-1.31848	1.318479631	protein_coding	NM_016535
ASHGV40006222	-1.31816	1.318158595	protein_coding	NM_024889
ASHGV40054014	1.318057	1.318057184	protein_coding	NM_020717
ASHGV40044527	-1.31787	1.317867864	protein_coding	NM_001085401
ASHGV40052720	-1.31765	1.317645184	protein_coding	NM_001004352
ASHGV40038245	1.317428	1.317427591	protein_coding	NM_032783
ASHGV40043176	-1.31733	1.317326137	protein_coding	NM_033057
ASHGV40010246	1.317281	1.317280587	protein_coding	NM_002837
ASHGV40035939	-1.31725	1.317245447	protein_coding	NM_173546
ASHGV40029901	-1.31694	1.316943942	protein_coding	NM_005373
ASHGV40057056	1.316856	1.316855844	protein_coding	NM_001006
ASHGV40003312	-1.3166	1.316599703	protein_coding	NM_030959
ASHGV40013138	-1.31628	1.316283445	protein_coding	NM_145657
ASHGV40025210	-1.31627	1.316266645	protein_coding	ENST00000397759
ASHGV40026606	1.316233	1.316233358	protein_coding	NM_003162
ASHGV40033038	-1.31601	1.316014678	protein_coding	NM_174977
ASHGV40055835	1.316011	1.316011478	protein_coding	NM_145246
ASHGV40031353	1.315902	1.315902392	protein_coding	NM_006870
ASHGV40050305	1.315543	1.315543252	protein_coding	NM_181723
ASHGV40029307	1.315284	1.315283552	protein_coding	NM_006267
ASHGV40040143	1.315265	1.315264572	protein_coding	NM_199335
ASHGV40043698	1.315035	1.315034803	protein_coding	NM_001251874
ASHGV40018446	-1.31467	1.314665626	protein_coding	uc002fna.1
ASHGV40034379	-1.31411	1.31410668	protein_coding	NM_015896
ASHGV40009974	1.314047	1.314046551	protein_coding	NM_002281
ASHGV40000067	1.313642	1.313641917	protein_coding	ENST00000331650
ASHGV40040403	1.313392	1.313392499	protein_coding	NM_005713
ASHGV40025546	-1.31337	1.313371579	protein_coding	NM_001080436

ASHGV40033219	1.31311	1.31311022	protein_coding	NM_002490
ASHGV40011090	1.312825	1.312825226	protein_coding	NM_004974
ASHGV40026061	-1.31265	1.312653702	protein_coding	NM_002000
ASHGV40025202	-1.31233	1.312329925	protein_coding	NM_021915
ASHGV40037160	1.31227	1.312269777	protein_coding	NM_002337
ASHGV40015778	1.311868	1.311867758	protein_coding	NM_012394
ASHGV40016782	1.311829	1.31182873	protein_coding	NM_014335
ASHGV40005594	1.311721	1.311720916	protein_coding	NM_001278795
ASHGV40008362	-1.31171	1.311709937	protein_coding	NM_001300722
ASHGV40035353	1.311046	1.311045543	protein_coding	NM_022149
ASHGV40003254	-1.31089	1.310894131	protein_coding	NM_003517
ASHGV40030442	-1.31084	1.310836187	protein_coding	ENST00000537552
ASHGV40021228	-1.31075	1.310752367	protein_coding	NM_001256615
ASHGV40011435	-1.31028	1.310280025	protein_coding	NM_012291
ASHGV40021920	-1.30999	1.309986516	protein_coding	NM_153229
ASHGV40031551	1.30963	1.309629634	protein_coding	NM_015511
ASHGV40041279	1.309613	1.309613032	protein_coding	NM_198868
ASHGV40009795	1.309485	1.309485356	protein_coding	NM_001013699
ASHGV40045307	1.309192	1.309191878	protein_coding	NM_031469
ASHGV40014672	-1.30915	1.309147149	protein_coding	NM_144622
ASHGV40024355	-1.30897	1.308974581	protein_coding	NM_144691
ASHGV40033189	1.30881	1.308809759	protein_coding	NM_020831
ASHGV40007569	1.308798	1.308798103	protein_coding	NM_032424
ASHGV40006468	-1.30867	1.30867115	protein_coding	NM_173573
ASHGV40006657	1.308584	1.308583894	protein_coding	NM_001418
ASHGV40012183	1.308071	1.308070959	protein_coding	NM_052907
ASHGV40032366	-1.30767	1.30766575	protein_coding	NM_144991
ASHGV40039684	-1.30764	1.307640298	protein_coding	NM_018248
ASHGV40056642	-1.3074	1.307402481	protein_coding	NM_178353
ASHGV40021990	1.307118	1.307118184	protein_coding	NM_014322
ASHGV40026244	1.307052	1.307051664	protein_coding	NM_018269
ASHGV40013019	1.306709	1.306708643	protein_coding	NM_000820
ASHGV40054003	-1.30643	1.30643035	protein_coding	NM_006150
ASHGV40014220	1.305621	1.305621106	protein_coding	NM_006370
ASHGV40032922	-1.30489	1.304888806	protein_coding	NM_016449
ASHGV40057194	-1.3047	1.304698733	protein_coding	NM_003519
ASHGV40052349	1.304691	1.304690549	protein_coding	NM_012127
ASHGV40006656	1.304579	1.304579342	protein_coding	NM_130385
ASHGV40003071	1.304295	1.304295058	protein_coding	NM_004038
ASHGV40024824	1.304187	1.304186519	protein_coding	NM_001136201
ASHGV40015715	-1.30406	1.304063457	protein_coding	NM_016946
ASHGV40047018	-1.304	1.30399942	protein_coding	ENST00000420664
ASHGV40043881	-1.30389	1.303886098	protein_coding	NM_005068
ASHGV40007570	1.303861	1.303860836	protein_coding	NM_152433
ASHGV40020067	-1.30357	1.303572605	protein_coding	ENST00000555059
ASHGV40025072	1.30356	1.303560071	protein_coding	NM_015074
ASHGV40002584	-1.30332	1.303316536	protein_coding	ENST00000593459

ASHGV40047455	-1.30266	1.302659303	protein_coding	NM_018641
ASHGV40029303	-1.30265	1.302649407	protein_coding	NM_001056
ASHGV40057245	1.302607	1.302606699	protein_coding	NM_005284
ASHGV40029748	1.302557	1.302556647	protein_coding	NM_003142
ASHGV40039343	1.302492	1.302491998	protein_coding	ENST00000424958
ASHGV40023740	1.302131	1.302130722	protein_coding	NM_015898
ASHGV40018003	1.301939	1.3019388	protein_coding	NM_022476
ASHGV40052100	1.301842	1.301841676	protein_coding	NM_000035
ASHGV40003160	-1.30182	1.301815941	protein_coding	NM_001199290
ASHGV40043264	1.301758	1.301758292	protein_coding	NM_130463
ASHGV40027035	1.30173	1.301729533	protein_coding	NM_006839
ASHGV40000578	-1.29981	1.29981184	protein_coding	NM_002441
ASHGV40008668	1.299565	1.299564509	protein_coding	NM_005146
ASHGV40007155	1.299545	1.299545185	protein_coding	NM_172230
ASHGV40019740	1.299358	1.299357986	protein_coding	NM_001167986
ASHGV40025149	-1.29909	1.299085735	protein_coding	NM_001172650
ASHGV40006251	-1.29881	1.298805488	protein_coding	NM_006229
ASHGV40035768	-1.29866	1.298664512	protein_coding	NM_005508
ASHGV40019200	-1.29865	1.298653934	protein_coding	NM_002428
ASHGV40060903	-1.29846	1.298460747	protein_coding	uc031skz.1
ASHGV40008002	-1.29837	1.298373175	protein_coding	NM_001005169
ASHGV40037257	1.297977	1.297976538	protein_coding	NM_012161
ASHGV40026765	1.296978	1.296977546	protein_coding	NM_018084
ASHGV40025864	-1.29697	1.296974207	protein_coding	NM_153608
ASHGV40046292	1.296716	1.29671592	protein_coding	NM_003930
ASHGV40040538	1.296634	1.296634212	protein_coding	ENST00000316610
ASHGV40015216	-1.29652	1.296520901	protein_coding	NM_017791
ASHGV40039085	1.296465	1.296465395	protein_coding	NM_001136570
ASHGV40015730	1.296392	1.296391525	protein_coding	NM_003134
ASHGV40020305	-1.29623	1.296225759	protein_coding	NM_033184
ASHGV40005706	1.295712	1.295711952	protein_coding	NM_032804
ASHGV40016469	-1.29542	1.295417587	protein_coding	ENST00000558592
ASHGV40025122	1.295024	1.295024187	protein_coding	NM_001031
ASHGV40014627	1.295024	1.295023548	protein_coding	NM_004894
ASHGV40049385	-1.29485	1.294853814	protein_coding	NM_000370
ASHGV40034039	1.29439	1.294389753	protein_coding	NM_022497
ASHGV40014513	-1.29429	1.294286939	protein_coding	ENST00000554299
ASHGV40003337	-1.29393	1.293934736	protein_coding	NM_152250
ASHGV40032845	1.293756	1.293755836	protein_coding	NM_003277
ASHGV40024530	1.293092	1.293091727	protein_coding	NM_012155
ASHGV40030470	-1.29301	1.293010512	protein_coding	NM_145762
ASHGV40024543	1.292848	1.292847587	protein_coding	NM_002516
ASHGV40001419	-1.29259	1.292591909	protein_coding	NM_032517
ASHGV40020711	1.29251	1.292509769	protein_coding	NM_004396
ASHGV40011550	-1.29245	1.292454882	protein_coding	NM_005269
ASHGV40005887	1.292363	1.292362538	protein_coding	NM_032333
ASHGV40007485	1.291894	1.291893745	protein_coding	NM_016403

ASHGV40049461	1.291782	1.291782181	protein_coding	NM_014393
ASHGV40008672	-1.29155	1.291550074	protein_coding	uc001ogs.1
ASHGV40034805	-1.29152	1.291521384	protein_coding	NM_000187
ASHGV40001365	-1.29151	1.291508188	protein_coding	NM_001204478
ASHGV40002004	1.290395	1.290395397	protein_coding	NM_019094
ASHGV40052179	1.290324	1.290323888	protein_coding	NM_001080398
ASHGV40041836	1.290285	1.290284501	protein_coding	NM_001253697
ASHGV40027515	1.290182	1.290181631	protein_coding	NM_014795
ASHGV40042275	-1.29008	1.290078136	protein_coding	NM_130809
ASHGV40024560	-1.29007	1.290066242	protein_coding	ENST00000593888
ASHGV40023765	-1.29005	1.290047135	protein_coding	uc002mbn.1
ASHGV40023734	-1.28997	1.289970636	protein_coding	NM_152486
ASHGV40009333	1.28984	1.289839759	protein_coding	NM_013264
ASHGV40046822	-1.2895	1.289502972	protein_coding	NM_018842
ASHGV40010608	1.289405	1.289404612	protein_coding	NM_004658
ASHGV40055333	1.289339	1.289339493	protein_coding	NM_004541
ASHGV40041582	-1.2891	1.289097443	protein_coding	NM_001204375
ASHGV40041213	1.289017	1.28901702	protein_coding	NM_007097
ASHGV40055902	1.288981	1.288981095	protein_coding	NM_173809
ASHGV40016605	1.288833	1.288833349	protein_coding	NM_003762
ASHGV40042684	1.288415	1.288415285	protein_coding	ENST00000338333
ASHGV40044061	1.28829	1.288289745	protein_coding	NM_001139510
ASHGV40025649	-1.28802	1.288019567	protein_coding	NM_004823
ASHGV40033062	-1.28794	1.287937844	protein_coding	NM_002440
ASHGV40041967	1.287829	1.287829076	protein_coding	NM_003248
ASHGV40035435	1.287583	1.287583377	protein_coding	NM_032279
ASHGV40047240	-1.28751	1.287505404	protein_coding	uc011ksa.2
ASHGV40018511	-1.2873	1.287299266	protein_coding	NM_001013638
ASHGV40028530	1.287272	1.287272388	protein_coding	NM_004939
ASHGV40032673	1.287029	1.287029257	protein_coding	NM_005239
ASHGV40037671	1.287021	1.287021122	protein_coding	NM_001029870
ASHGV40021978	1.286695	1.286695226	protein_coding	NM_002126
ASHGV40013826	1.286655	1.286654847	protein_coding	NM_022075
ASHGV40005376	-1.28631	1.286307409	protein_coding	uc010qcr.1
ASHGV40037482	1.286237	1.286237431	protein_coding	NM_015030
ASHGV40052025	-1.28615	1.286153606	protein_coding	NM_000136
ASHGV40007199	-1.28615	1.286151666	protein_coding	NM_020404
ASHGV40028277	1.286089	1.286089357	protein_coding	NM_022817
ASHGV40050897	1.285948	1.285947738	protein_coding	NM_000067
ASHGV40019519	-1.28591	1.285911832	protein_coding	ENST00000574293
ASHGV40005956	1.285848	1.285847525	protein_coding	NM_014947
ASHGV40039394	1.285738	1.285737614	protein_coding	NM_032961
ASHGV40031550	1.285731	1.285730879	protein_coding	NM_012156
ASHGV40032996	-1.2857	1.285703627	protein_coding	NM_002430
ASHGV40027723	1.285568	1.285567906	protein_coding	NM_013341
ASHGV40027066	-1.28555	1.285546634	protein_coding	NM_001010980
ASHGV40020961	1.285533	1.285533176	protein_coding	NM_003655

ASHGV40046694	1.285386	1.285386175	protein_coding	NM_006682
ASHGV40011164	1.285194	1.285193911	protein_coding	NM_001001660
ASHGV40010760	-1.28511	1.285111614	protein_coding	NM_001409
ASHGV40001431	1.284822	1.284822253	protein_coding	ENST00000498285
ASHGV40020522	1.284666	1.284666129	protein_coding	NM_014897
ASHGV40014575	1.284332	1.284331795	protein_coding	ENST00000596284
ASHGV40011991	-1.28382	1.283821246	protein_coding	NM_152591
ASHGV40055877	1.283806	1.283806231	protein_coding	NM_178832
ASHGV40057587	-1.28362	1.283621681	protein_coding	NM_001001888
ASHGV40008489	-1.28319	1.283191535	protein_coding	NM_139249
ASHGV40014176	1.283156	1.283155728	protein_coding	NM_030791
ASHGV40034981	-1.28296	1.282955894	protein_coding	NM_001013650
ASHGV40023443	1.282883	1.282883079	protein_coding	NM_033280
ASHGV40044074	-1.28243	1.282431758	protein_coding	NM_033515
ASHGV40039911	-1.28192	1.281924781	protein_coding	NM_144702
ASHGV40029764	1.281772	1.281772085	protein_coding	NM_178120
ASHGV40032400	-1.28174	1.281739672	protein_coding	NM_194255
ASHGV40008334	1.281411	1.281411396	protein_coding	NM_016142
ASHGV40035662	1.281291	1.281290573	protein_coding	NM_054110
ASHGV40021195	-1.28128	1.281280741	protein_coding	NM_153230
ASHGV40050977	1.281223	1.281223225	protein_coding	NM_016134
ASHGV40053691	1.281159	1.281159479	protein_coding	NM_000718
ASHGV40048309	1.280837	1.280836891	protein_coding	NM_000108
ASHGV40006631	-1.2806	1.280599658	protein_coding	NM_002315
ASHGV40012137	-1.28051	1.280511268	protein_coding	NM_201435
ASHGV40019252	-1.28048	1.280482361	protein_coding	NM_144601
ASHGV40029763	1.280283	1.28028339	protein_coding	NM_199227
ASHGV40053601	-1.28024	1.280243413	protein_coding	NM_000093
ASHGV40024804	1.280158	1.280158326	protein_coding	NM_001271618
ASHGV40014032	-1.28012	1.280121528	protein_coding	NM_002692
ASHGV40040925	1.280031	1.280030593	protein_coding	NM_175866
ASHGV40018314	-1.27989	1.27988733	protein_coding	NM_024980
ASHGV40043455	1.279726	1.279726287	protein_coding	NM_020737
ASHGV40032388	1.279486	1.279486332	protein_coding	NM_014787
ASHGV40029010	1.279223	1.27922327	protein_coding	NM_007365
ASHGV40057682	-1.27912	1.279124295	protein_coding	NM_015262
ASHGV40054815	-1.27903	1.279028351	protein_coding	uc011mkc.3
ASHGV40005156	-1.27876	1.278760112	protein_coding	NM_001109
ASHGV40045519	1.278359	1.278358753	protein_coding	NM_138408
ASHGV40025597	-1.27827	1.278274448	protein_coding	NM_199180
ASHGV40044440	1.278207	1.278207037	protein_coding	NM_018288
ASHGV40012862	-1.27817	1.278168275	protein_coding	NM_178861
ASHGV40049319	-1.2781	1.278099315	protein_coding	NM_005372
ASHGV40000214	-1.2779	1.27790205	protein_coding	ENST00000395528
ASHGV40044618	1.277745	1.277745425	protein_coding	NM_016167
ASHGV40042647	1.27746	1.277460161	protein_coding	NM_016545
ASHGV40023142	1.276716	1.276715835	protein_coding	NM_003826

ASHGV40048213	1.276378	1.276377942	protein_coding	NM_006833
ASHGV40007679	1.276255	1.276254536	protein_coding	NM_001281748
ASHGV40057489	-1.27624	1.276239505	protein_coding	NM_153244
ASHGV40018530	1.275994	1.275994441	protein_coding	NM_032520
ASHGV40039371	-1.27564	1.275641715	protein_coding	NM_014264
ASHGV40024660	-1.27546	1.275455236	protein_coding	NM_152358
ASHGV40030897	-1.27529	1.275288881	protein_coding	NM_020398
ASHGV40015304	1.275288	1.275287671	protein_coding	NM_002802
ASHGV40042416	-1.27508	1.275078805	protein_coding	NM_018905
ASHGV40011173	1.274756	1.274755881	protein_coding	NM_005086
ASHGV40042235	1.274751	1.274751101	protein_coding	NM_003100
ASHGV40048011	-1.27457	1.274568874	protein_coding	uc011kfh.1
ASHGV40049775	1.274561	1.274560603	protein_coding	NM_001099744
ASHGV40050113	-1.27455	1.274548381	protein_coding	NM_031308
ASHGV40045170	1.274219	1.274218657	protein_coding	NM_014051
ASHGV40057063	-1.27375	1.273753651	protein_coding	NM_001012414
ASHGV40023491	-1.27353	1.273534641	protein_coding	NM_005024
ASHGV40024908	-1.27316	1.273160594	protein_coding	NM_001215
ASHGV40053696	1.272249	1.272248756	protein_coding	uc001jij.4
ASHGV40055926	-1.27224	1.272238425	protein_coding	NM_001080418
ASHGV40019327	-1.27224	1.272235277	protein_coding	NM_182619
ASHGV40027883	1.272145	1.272144563	protein_coding	NM_020760
ASHGV40032879	1.272131	1.272130669	protein_coding	NM_032775
ASHGV40046243	1.271962	1.271961724	protein_coding	NM_019059
ASHGV40032722	-1.27188	1.271881563	protein_coding	NM_019062
ASHGV40033169	1.271305	1.27130531	protein_coding	NM_014293
ASHGV40054251	1.270816	1.270816072	protein_coding	NM_014782
ASHGV40023484	-1.27078	1.270776394	protein_coding	ENST00000269491
ASHGV40039573	1.27043	1.270430223	protein_coding	NM_014247
ASHGV40046266	-1.27018	1.270179069	protein_coding	NM_138811
ASHGV40045071	-1.27006	1.270057495	protein_coding	NM_153320
ASHGV40011875	1.26988	1.269880136	protein_coding	NM_181861
ASHGV40042949	-1.26929	1.269292432	protein_coding	NM_173156
ASHGV40018194	-1.26927	1.269267012	protein_coding	NM_144676
ASHGV40009615	1.269257	1.269256897	protein_coding	NM_006248
ASHGV40043714	1.269059	1.269058793	protein_coding	NM_018247
ASHGV40015636	1.269025	1.269025353	protein_coding	NM_138704
ASHGV40005488	1.268946	1.268945592	protein_coding	NM_030751
ASHGV40043549	1.268364	1.268364456	protein_coding	NM_021572
ASHGV40008540	1.26828	1.2682797	protein_coding	NM_025080
ASHGV40017523	1.26813	1.268130318	protein_coding	NM_017885
ASHGV40013455	-1.26797	1.267967026	protein_coding	NM_001730
ASHGV40040910	1.267589	1.267588991	protein_coding	NM_002109
ASHGV40054966	-1.2675	1.26749802	protein_coding	NM_012196
ASHGV40020560	1.267495	1.267494556	protein_coding	NM_052855
ASHGV40021695	1.267277	1.267276584	protein_coding	NM_003250
ASHGV40035954	-1.26727	1.267273358	protein_coding	ENST00000419183

ASHGV40009588	1.266841	1.26684101	protein_coding	ENST00000539033
ASHGV40006931	1.266675	1.266674725	protein_coding	NM_005055
ASHGV40032976	-1.26594	1.265939657	protein_coding	NM_001887
ASHGV40033729	-1.26589	1.265894867	protein_coding	NM_006855
ASHGV40040791	1.265776	1.265775698	protein_coding	NM_005340
ASHGV40021032	1.265691	1.265691167	protein_coding	NM_145257
ASHGV40028214	-1.26567	1.265674992	protein_coding	NM_002242
ASHGV40024889	-1.26538	1.26538227	protein_coding	NM_025027
ASHGV40016872	1.265371	1.265370673	protein_coding	NM_001040450
ASHGV40010699	-1.26533	1.265332012	protein_coding	NM_003733
ASHGV40015234	1.265243	1.265242904	protein_coding	NM_012111
ASHGV40042336	-1.26524	1.265238683	protein_coding	uc003kzl.2
ASHGV40021999	1.264701	1.26470112	protein_coding	NM_001821
ASHGV40017447	1.264352	1.264352221	protein_coding	NM_012467
ASHGV40012752	1.264171	1.264171447	protein_coding	NM_022118
ASHGV40034451	1.264168	1.264168056	protein_coding	NM_019555
ASHGV40025891	-1.26404	1.264040161	protein_coding	NM_033043
ASHGV40052181	1.26403	1.264029723	protein_coding	NM_004521
ASHGV40012889	1.263916	1.263916067	protein_coding	NM_001079669
ASHGV40042986	1.263912	1.263912197	protein_coding	NM_004280
ASHGV40052519	1.263782	1.263782368	protein_coding	NM_018998
ASHGV40040645	1.263638	1.263638251	protein_coding	NM_014819
ASHGV40026011	-1.2636	1.263603051	protein_coding	NM_173856
ASHGV40019152	1.263597	1.263597487	protein_coding	NM_005954
ASHGV40020236	-1.26341	1.263414229	protein_coding	NM_003268
ASHGV40021817	1.262832	1.262832112	protein_coding	NM_016438
ASHGV40054230	-1.26248	1.262482907	protein_coding	NM_003270
ASHGV40031916	-1.26246	1.262462136	protein_coding	NM_002531
ASHGV40040921	-1.26242	1.262424528	protein_coding	NM_033449
ASHGV40035796	-1.26205	1.262052618	protein_coding	NM_178339
ASHGV40044098	1.261888	1.261888434	protein_coding	NM_052831
ASHGV40035601	-1.2616	1.261603645	protein_coding	NM_001018115
ASHGV40020297	-1.26148	1.261480886	protein_coding	NM_031959
ASHGV40021937	-1.26147	1.261474909	protein_coding	uc002ism.3
ASHGV40006310	1.260905	1.260904736	protein_coding	NM_144587
ASHGV40036564	1.260544	1.26054379	protein_coding	NM_025246
ASHGV40011455	-1.26012	1.260124566	protein_coding	NM_017410
ASHGV40046183	1.260045	1.260045493	protein_coding	NM_004956
ASHGV40040004	1.259825	1.259825393	protein_coding	NM_004061
ASHGV40026928	1.259755	1.259754777	protein_coding	NM_015189
ASHGV40008615	1.259328	1.259328048	protein_coding	NM_001667
ASHGV40019565	1.259304	1.259304301	protein_coding	NM_020655
ASHGV40022870	-1.25915	1.259154249	protein_coding	ENST00000593319
ASHGV40040917	-1.25911	1.259105245	protein_coding	NM_031947
ASHGV40017786	1.258881	1.25888074	protein_coding	NM_015171
ASHGV40021312	-1.2585	1.258500337	protein_coding	NM_153604
ASHGV40011974	1.258495	1.258495142	protein_coding	NM_000431

ASHGV40010105	1.258451	1.258451304	protein_coding	NM_004083
ASHGV40048599	-1.25837	1.258369807	protein_coding	uc003wdi.2
ASHGV40019520	1.258159	1.25815865	protein_coding	NM_014873
ASHGV40004897	1.257838	1.257837699	protein_coding	NM_005066
ASHGV40039134	1.257577	1.257576895	protein_coding	NM_024047
ASHGV40046785	-1.25751	1.257512561	protein_coding	NM_001742
ASHGV40021227	-1.25714	1.257141282	protein_coding	NM_003808
ASHGV40055948	-1.257	1.256995523	protein_coding	NM_000102
ASHGV40032431	1.256775	1.256775475	protein_coding	NM_002600
ASHGV40037902	-1.25626	1.256261886	protein_coding	NM_022569
ASHGV40048539	-1.25564	1.255637183	protein_coding	NM_024926
ASHGV40006624	-1.25563	1.255628787	protein_coding	NM_001004461
ASHGV40053093	-1.25516	1.255161994	protein_coding	NM_006705
ASHGV40028649	-1.25512	1.255120754	protein_coding	NM_004341
ASHGV40006136	-1.25482	1.254818792	protein_coding	NM_020682
ASHGV40029945	-1.25479	1.25479408	protein_coding	NM_001080539
ASHGV40047349	1.254689	1.254689433	protein_coding	ENST00000543018
ASHGV40018394	1.254504	1.254503533	protein_coding	NM_024735
ASHGV40056316	1.254217	1.254216651	protein_coding	NM_000977
ASHGV40009639	-1.25411	1.254109433	protein_coding	ENST00000527705
ASHGV40055647	-1.25407	1.254065017	protein_coding	NM_004747
ASHGV40012388	-1.25402	1.254022238	protein_coding	NM_001265
ASHGV40034164	1.25386	1.253860087	protein_coding	NM_016176
ASHGV40052763	1.253841	1.253841379	protein_coding	NM_001244752
ASHGV40056895	-1.2537	1.253697753	protein_coding	NM_003973
ASHGV40019500	1.253639	1.253639205	protein_coding	NM_021197
ASHGV40029351	-1.25354	1.253540932	protein_coding	NM_019618
ASHGV40029060	1.253537	1.253537246	protein_coding	NM_014763
ASHGV40033017	1.253306	1.253306177	protein_coding	NM_000016
ASHGV40015999	-1.25329	1.253285162	protein_coding	NM_025049
ASHGV40015771	-1.25308	1.253080901	protein_coding	NM_213600
ASHGV40048961	1.252688	1.252687896	protein_coding	NM_177924
ASHGV40014896	1.252483	1.252482573	protein_coding	NM_032594
ASHGV40009879	-1.25243	1.252429002	protein_coding	NM_018018
ASHGV40020316	-1.25204	1.252037336	protein_coding	NM_004138
ASHGV40054234	-1.25203	1.252031895	protein_coding	NM_212559
ASHGV40034305	1.251937	1.251936612	protein_coding	NM_024661
ASHGV40008008	-1.25142	1.251421612	protein_coding	NM_021801
ASHGV40018767	-1.25126	1.251261171	protein_coding	NM_001256720
ASHGV40031245	1.251216	1.251216188	protein_coding	NM_001501
ASHGV40005584	1.251024	1.251023507	protein_coding	NM_032023
ASHGV40055026	-1.25102	1.2510218	protein_coding	NM_174912
ASHGV40039782	-1.251	1.250999145	protein_coding	NM_174900
ASHGV40015123	-1.25017	1.250166297	protein_coding	NM_173526
ASHGV40031950	-1.25004	1.250039991	protein_coding	NM_024059
ASHGV40008468	1.249853	1.249853124	protein_coding	NM_001018067
ASHGV40015877	1.249723	1.249723134	protein_coding	NM_016194

ASHGV40057419	-1.24958	1.249584842	protein_coding	NM_000015
ASHGV40040922	-1.24928	1.249281399	protein_coding	NM_022481
ASHGV40049905	1.249266	1.249265952	protein_coding	NM_014846
ASHGV40011107	1.249263	1.2492632	protein_coding	NM_007178
ASHGV40037510	1.248132	1.248131642	protein_coding	NM_033631
ASHGV40010507	1.246984	1.246984296	protein_coding	NM_006166
ASHGV40010094	-1.24673	1.24672971	protein_coding	NM_005379
ASHGV40016018	1.246468	1.246468169	protein_coding	NM_032445
ASHGV40034500	1.246163	1.246162946	protein_coding	NM_014814
ASHGV40025387	1.246069	1.24606904	protein_coding	NM_015965
ASHGV40007014	-1.24569	1.245685829	protein_coding	NM_145016
ASHGV40031270	1.245516	1.24551625	protein_coding	NM_002979
ASHGV40024496	-1.2455	1.24549748	protein_coding	ENST00000588931
ASHGV40027635	-1.24543	1.245426634	protein_coding	NM_001935
ASHGV40026340	-1.24516	1.24515756	protein_coding	NM_002539
ASHGV40009979	-1.24507	1.245066837	protein_coding	NM_033045
ASHGV40016431	-1.24498	1.244983404	protein_coding	NM_001288615
ASHGV40041093	1.244318	1.244317666	protein_coding	NM_025153
ASHGV40025717	-1.24405	1.244052693	protein_coding	NM_000766
ASHGV40002091	-1.24398	1.24397761	protein_coding	ENST00000553820
ASHGV40007830	1.243887	1.243886966	protein_coding	NM_001142685
ASHGV40015855	-1.24352	1.243520828	protein_coding	NM_001013625
ASHGV40045257	1.243331	1.243331231	protein_coding	NM_014989
ASHGV40017663	1.242948	1.242947599	protein_coding	NM_014647
ASHGV40024674	-1.24279	1.242791242	protein_coding	NM_032712
ASHGV40011227	1.24277	1.242770218	protein_coding	NM_012062
ASHGV40034400	1.242581	1.242580535	protein_coding	NM_001947
ASHGV40046827	1.242512	1.242512488	protein_coding	NM_001134450
ASHGV40005160	1.242384	1.242383758	protein_coding	NM_015722
ASHGV40057706	-1.24235	1.242351745	protein_coding	NM_001310138
ASHGV40030266	1.242294	1.24229411	protein_coding	NM_018150
ASHGV40020419	1.24184	1.241840045	protein_coding	NM_144609
ASHGV40016015	1.241693	1.24169256	protein_coding	NM_005848
ASHGV40008123	1.241626	1.241625905	protein_coding	NM_001297719
ASHGV40015329	1.241615	1.24161495	protein_coding	NM_153646
ASHGV40051684	1.241431	1.241430868	protein_coding	NM_001017363
ASHGV40013316	-1.2414	1.241395596	protein_coding	NM_020377
ASHGV40025544	-1.24131	1.241310008	protein_coding	ENST00000316412
ASHGV40036439	1.240981	1.240981433	protein_coding	NM_014367
ASHGV40041042	-1.24085	1.240849832	protein_coding	NM_004821
ASHGV40016652	-1.24054	1.240537621	protein_coding	NM_003246
ASHGV40050605	1.240493	1.240493416	protein_coding	NM_006214
ASHGV40019517	1.240064	1.240063791	protein_coding	NM_014732
ASHGV40028639	-1.23996	1.239956402	protein_coding	NM_017727
ASHGV40040280	1.239545	1.239545299	protein_coding	NM_006622
ASHGV40015734	-1.23953	1.239526943	protein_coding	NM_001190479
ASHGV40045190	1.239458	1.239457632	protein_coding	NM_138569

ASHGV40009059	1.239355	1.239354782	protein_coding	uc001pgn.3
ASHGV40042633	1.239135	1.239134785	protein_coding	NM_145049
ASHGV40020463	1.239076	1.239076201	protein_coding	NM_203400
ASHGV40048868	-1.23898	1.238977697	protein_coding	NM_024607
ASHGV40026609	-1.23885	1.238852387	protein_coding	NM_032264
ASHGV40044779	-1.23836	1.238358567	protein_coding	NM_003521
ASHGV40030796	1.238319	1.238319443	protein_coding	NM_021100
ASHGV40025738	-1.23795	1.23795489	protein_coding	NM_001022
ASHGV40052692	1.23755	1.237550136	protein_coding	NM_001282582
ASHGV40057285	1.237486	1.237485846	protein_coding	ENST00000403226
ASHGV40008436	-1.23731	1.237313724	protein_coding	NM_001005213
ASHGV40003192	-1.23689	1.236890591	protein_coding	NM_001243523
ASHGV40005927	-1.23685	1.236851995	protein_coding	NM_033282
ASHGV40013498	1.236834	1.236833811	protein_coding	NM_019080
ASHGV40056188	-1.23644	1.236436707	protein_coding	NM_004039
ASHGV40028083	1.236348	1.236347851	protein_coding	NM_006000
ASHGV40055951	1.235901	1.235900945	protein_coding	NM_032747
ASHGV40019476	-1.23508	1.23507726	protein_coding	NM_183059
ASHGV40016742	1.23473	1.234729966	protein_coding	NM_182985
ASHGV40024065	-1.23461	1.234605081	protein_coding	NM_001190844
ASHGV40017212	1.234377	1.234377446	protein_coding	NM_002605
ASHGV40052219	-1.23424	1.234241644	protein_coding	NM_002160
ASHGV40026286	1.234045	1.234045389	protein_coding	NM_207315
ASHGV40024309	-1.23394	1.233940408	protein_coding	NM_001012756
ASHGV40011323	1.233258	1.233258019	protein_coding	NM_000289
ASHGV40022066	-1.23324	1.233244489	protein_coding	NM_006039
ASHGV40056386	-1.23299	1.23298866	protein_coding	NM_000422
ASHGV40003177	1.232934	1.232934477	protein_coding	NM_001243598
ASHGV40038712	-1.23256	1.232558645	protein_coding	NM_006424
ASHGV40006887	-1.23228	1.232280937	protein_coding	NM_021926
ASHGV40039024	-1.23178	1.231783507	protein_coding	NM_000565
ASHGV40049160	-1.23172	1.231715951	protein_coding	NM_001004690
ASHGV40044793	-1.23167	1.23166625	protein_coding	NM_024493
ASHGV40048191	1.231456	1.231455531	protein_coding	NM_002523
ASHGV40007519	-1.23139	1.231390109	protein_coding	NM_001031672
ASHGV40000020	-1.23126	1.231256468	protein_coding	NM_017515
ASHGV40055092	1.231115	1.23111549	protein_coding	NM_001039840
ASHGV40018037	1.230753	1.230753225	protein_coding	NM_007006
ASHGV40017857	1.230556	1.230555764	protein_coding	NM_015984
ASHGV40010382	-1.2305	1.230495886	protein_coding	NM_004950
ASHGV40031440	-1.23049	1.230485103	protein_coding	NM_003650
ASHGV40007593	1.230052	1.230052492	protein_coding	ENST00000265843
ASHGV40020630	1.230009	1.230008542	protein_coding	NM_017763
ASHGV40029217	1.229883	1.229883261	protein_coding	NM_014044
ASHGV40033049	-1.22977	1.22977205	protein_coding	NM_015715
ASHGV40045199	-1.22966	1.229657277	protein_coding	NM_207410
ASHGV40009183	-1.22922	1.229223801	protein_coding	NM_000040

ASHGV40000999	-1.22895	1.228948135	protein_coding	ENST00000445351
ASHGV40056703	-1.22886	1.228863422	protein_coding	NM_173852
ASHGV40037948	1.228609	1.228608588	protein_coding	NM_176824
ASHGV40051218	-1.22839	1.228392299	protein_coding	NM_001159542
ASHGV40021131	1.228384	1.228384033	protein_coding	NM_001258311
ASHGV40034715	1.228262	1.228261825	protein_coding	NM_001777
ASHGV40011632	-1.22822	1.228221895	protein_coding	NM_033647
ASHGV40021209	-1.22816	1.228155393	protein_coding	NM_014716
ASHGV40011337	-1.22814	1.228144561	protein_coding	NM_033124
ASHGV40030390	-1.22789	1.227891993	protein_coding	NM_018226
ASHGV40031333	-1.22776	1.227761948	protein_coding	NM_080826
ASHGV40053711	-1.22764	1.227641983	protein_coding	NM_145177
ASHGV40001019	-1.2275	1.227501127	protein_coding	ENST00000446344
ASHGV40021755	-1.2273	1.227303534	protein_coding	NM_032387
ASHGV40056511	1.227233	1.227233146	protein_coding	NM_014173
ASHGV40042566	1.227211	1.227211411	protein_coding	NM_001166208
ASHGV40018766	1.227024	1.227023509	protein_coding	NM_016524
ASHGV40054196	1.226966	1.226966255	protein_coding	NM_000390
ASHGV40017673	-1.22692	1.226922098	protein_coding	uc021tdx.1
ASHGV40001292	1.22671	1.226710097	protein_coding	ENST00000465127
ASHGV40048561	-1.2267	1.226704925	protein_coding	NM_018980
ASHGV40028055	1.226515	1.226514768	protein_coding	NM_001087
ASHGV40051401	1.226333	1.226332754	protein_coding	NM_003801
ASHGV40045193	-1.22623	1.226226345	protein_coding	NM_013371
ASHGV40006926	-1.22621	1.226214127	protein_coding	ENST00000594280
ASHGV40029235	-1.2262	1.226198984	protein_coding	NM_001011717
ASHGV40044262	1.226025	1.226024754	protein_coding	NM_182961
ASHGV40016338	-1.22557	1.225569255	protein_coding	NM_001150
ASHGV40016612	-1.22554	1.225539757	protein_coding	NM_175741
ASHGV40019406	-1.22543	1.22542634	protein_coding	NM_001306094
ASHGV40012778	-1.22541	1.225409628	protein_coding	uc001eof.1
ASHGV40008957	-1.22491	1.22491136	protein_coding	NM_001156474
ASHGV40038070	1.224809	1.224808914	protein_coding	NM_015130
ASHGV40039547	-1.22477	1.224767175	protein_coding	NM_005651
ASHGV40051504	1.22452	1.22452031	protein_coding	NM_002839
ASHGV40057686	-1.22439	1.224393726	protein_coding	ENST00000374258
ASHGV40014150	-1.22435	1.224350454	protein_coding	NM_174978
ASHGV40005326	1.224074	1.224074452	protein_coding	NM_153498
ASHGV40022515	-1.22406	1.224063712	protein_coding	NM_001009611
ASHGV40051744	1.223717	1.223717211	protein_coding	NM_147195
ASHGV40041016	1.222979	1.222978814	protein_coding	NM_001155
ASHGV40035010	1.222657	1.222657112	protein_coding	NM_001184
ASHGV40019989	-1.22253	1.22253439	protein_coding	ENST00000399083
ASHGV40029943	1.222221	1.222221027	protein_coding	NM_020342
ASHGV40022017	1.222096	1.222095592	protein_coding	NM_080677
ASHGV40047323	1.222092	1.222092443	protein_coding	NM_016203
ASHGV40026947	1.222055	1.222055031	protein_coding	NM_212552

ASHGV40033944	1.222051	1.222051263	protein_coding	NM_016302
ASHGV40017187	1.221992	1.221992162	protein_coding	NM_023003
ASHGV40020455	-1.2219	1.221900012	protein_coding	NM_001113738
ASHGV40023672	1.221517	1.221517074	protein_coding	ENST00000382477
ASHGV40015183	-1.2215	1.221496767	protein_coding	NM_001004341
ASHGV40005700	1.221333	1.221332701	protein_coding	NM_173554
ASHGV40028797	1.221224	1.221224118	protein_coding	NM_002706
ASHGV40029409	1.221206	1.221206296	protein_coding	NM_022733
ASHGV40007956	-1.22109	1.221093709	protein_coding	NM_006757
ASHGV40008369	1.220981	1.220981413	protein_coding	NM_001105540
ASHGV40017855	1.220825	1.220825278	protein_coding	NM_145271
ASHGV40022291	-1.2208	1.220803434	protein_coding	NM_018996
ASHGV40020348	-1.22048	1.220478367	protein_coding	NM_001524
ASHGV40013331	-1.22044	1.220443511	protein_coding	NM_173605
ASHGV40011420	-1.2202	1.220196445	protein_coding	NM_000224
ASHGV40000043	-1.21977	1.219771111	protein_coding	NM_001338
ASHGV40017239	-1.21953	1.219532738	protein_coding	NM_001113378
ASHGV40024465	-1.21945	1.219445963	protein_coding	NM_014400
ASHGV40029952	1.219098	1.21909765	protein_coding	NM_002157
ASHGV40021125	-1.21897	1.218974733	protein_coding	NM_014565
ASHGV40049602	1.218932	1.218931736	protein_coding	NM_004929
ASHGV40003113	-1.21864	1.21863857	protein_coding	NM_001099850
ASHGV40039962	1.218435	1.218434677	protein_coding	NM_054027
ASHGV40014465	1.218383	1.218382961	protein_coding	NM_004545
ASHGV40043942	1.218185	1.218185256	protein_coding	NM_003931
ASHGV40012541	-1.21801	1.218013083	protein_coding	NM_198849
ASHGV40052817	1.217647	1.21764686	protein_coding	NM_007096
ASHGV40025563	-1.21744	1.217438978	protein_coding	NM_005031
ASHGV40043237	-1.21738	1.217381012	protein_coding	NM_007243
ASHGV40023996	1.21711	1.217110266	protein_coding	NM_004146
ASHGV40005163	1.217	1.21699961	protein_coding	NM_004092
ASHGV40020066	1.216742	1.216741811	protein_coding	NM_000638
ASHGV40011995	-1.21648	1.216476783	protein_coding	NM_015267
ASHGV40019589	-1.21603	1.216033305	protein_coding	NM_030928
ASHGV40007450	1.215864	1.215864401	protein_coding	NM_001143831
ASHGV40025704	1.215788	1.215787893	protein_coding	NM_020971
ASHGV40030825	-1.21561	1.215608556	protein_coding	uc002xhn.1
ASHGV40038473	-1.21548	1.215478466	protein_coding	NM_006342
ASHGV40024993	-1.21499	1.214985717	protein_coding	NM_182973
ASHGV40046882	1.214466	1.21446614	protein_coding	NM_001084
ASHGV40016779	1.214377	1.214376881	protein_coding	NM_001025248
ASHGV40033193	1.214317	1.21431687	protein_coding	NM_003932
ASHGV40027860	-1.21431	1.214314253	protein_coding	NM_017638
ASHGV40010292	-1.21431	1.214310098	protein_coding	NM_001321
ASHGV40011546	-1.21404	1.214044131	protein_coding	ENST00000598001
ASHGV40015514	1.214023	1.214022818	protein_coding	NM_001969
ASHGV40024597	1.213845	1.213845108	protein_coding	NM_018273

ASHGV40020575	1.213662	1.213662053	protein_coding	NM_004375
ASHGV40020996	1.213635	1.213635157	protein_coding	NM_001304994
ASHGV40014012	1.213557	1.213556687	protein_coding	NM_002013
ASHGV40009632	1.213284	1.213284336	protein_coding	NM_015987
ASHGV40046469	1.213144	1.213144279	protein_coding	NM_024709
ASHGV40055250	1.212969	1.212969221	protein_coding	NM_021132
ASHGV40018845	1.212825	1.212824859	protein_coding	NM_006040
ASHGV40050094	1.212623	1.212623182	protein_coding	NM_015117
ASHGV40020110	1.212209	1.212209084	protein_coding	NM_032854
ASHGV40024839	-1.21204	1.212043359	protein_coding	ENST00000270459
ASHGV40014142	1.21158	1.211580134	protein_coding	NM_016029
ASHGV40015377	1.211183	1.211183281	protein_coding	NM_016417
ASHGV40020623	1.211182	1.211181756	protein_coding	NM_004758
ASHGV40047771	1.210858	1.210857657	protein_coding	NM_015983
ASHGV40015163	1.210544	1.210543683	protein_coding	NM_015556
ASHGV40043114	1.210473	1.210472824	protein_coding	NM_016614
ASHGV40046322	1.21033	1.210330198	protein_coding	NM_014766
ASHGV40019725	-1.21022	1.210216032	protein_coding	NM_001122890
ASHGV40018477	1.210013	1.210012841	protein_coding	NM_000558
ASHGV40011604	-1.20958	1.20957841	protein_coding	NM_020762
ASHGV40042709	-1.20933	1.209328525	protein_coding	NM_014211
ASHGV40029688	1.209171	1.209170978	protein_coding	NM_003628
ASHGV40034801	1.207947	1.207947242	protein_coding	NM_001099678
ASHGV40025524	-1.20779	1.207786888	protein_coding	NM_152266
ASHGV40005955	1.207632	1.207631501	protein_coding	NM_020799
ASHGV40036823	-1.20755	1.20755396	protein_coding	NM_001258315
ASHGV40007251	-1.20753	1.20752677	protein_coding	NM_004923
ASHGV40006571	-1.20707	1.207074004	protein_coding	NM_005330
ASHGV40027437	1.20696	1.206960203	protein_coding	NM_001085365
ASHGV40002645	-1.20681	1.206809635	protein_coding	ENST00000597463
ASHGV40057111	-1.20679	1.206786308	protein_coding	NM_004472
ASHGV40047518	1.206533	1.206533468	protein_coding	NM_152745
ASHGV40056190	-1.2064	1.206401891	protein_coding	NM_207322
ASHGV40020283	-1.20611	1.206110773	protein_coding	NM_001143962
ASHGV40014606	1.205665	1.20566544	protein_coding	NM_006035
ASHGV40022721	1.205542	1.205541825	protein_coding	NM_002930
ASHGV40047259	1.205521	1.205520921	protein_coding	NM_014719
ASHGV40044558	-1.20535	1.205353301	protein_coding	NM_004415
ASHGV40013242	1.205147	1.205146995	protein_coding	NM_007187
ASHGV40018876	-1.2051	1.205095393	protein_coding	NM_001310136
ASHGV40014865	1.204946	1.204945937	protein_coding	NM_016106
ASHGV40054237	1.204817	1.204816924	protein_coding	NM_024885
ASHGV40045938	1.204588	1.204588026	protein_coding	NM_006775
ASHGV40036298	-1.2044	1.204404687	protein_coding	NM_175056
ASHGV40018292	-1.20437	1.204372123	protein_coding	ENST00000594986
ASHGV40007111	-1.20398	1.203984497	protein_coding	NM_017878
ASHGV40012158	1.203643	1.203643113	protein_coding	NM_152437

ASHGV40027794	1.203625	1.203625199	protein_coding	NM_001003683
ASHGV40053762	1.203277	1.203277494	protein_coding	NM_000273
ASHGV40025060	-1.20285	1.202851647	protein_coding	NM_181710
ASHGV40029302	-1.20256	1.202563583	protein_coding	NM_001008743
ASHGV40025522	-1.20234	1.20233906	protein_coding	NM_001300891
ASHGV40020697	-1.20225	1.202251655	protein_coding	NM_001317
ASHGV40042457	-1.20223	1.202226782	protein_coding	NM_032097
ASHGV40011518	1.200541	1.200540617	protein_coding	NM_001982
ASHGV40046445	-1.20012	1.200121828	protein_coding	NM_000162
ASHGV40040651	1.199462	1.199462034	protein_coding	NM_001039763
ASHGV40017634	1.199131	1.199130704	protein_coding	ENST00000399147
ASHGV40022445	1.199129	1.199129475	protein_coding	NM_173464
ASHGV40001882	1.199115	1.199115079	protein_coding	NM_017583
ASHGV40043249	-1.19902	1.199019977	protein_coding	NM_014070
ASHGV40046315	1.198999	1.198998678	protein_coding	NM_175061
ASHGV40003282	1.198898	1.198897561	protein_coding	NM_015234
ASHGV40025152	-1.19888	1.198881098	protein_coding	ENST00000592851
ASHGV40017875	-1.19874	1.198736403	protein_coding	NM_001136509
ASHGV40025167	-1.19862	1.198620563	protein_coding	NM_000201
ASHGV40027110	1.198546	1.198545805	protein_coding	NM_017789
ASHGV40049844	-1.19843	1.198434093	protein_coding	NM_024094
ASHGV40006527	1.198382	1.19838223	protein_coding	NM_001751
ASHGV40007166	1.198034	1.19803434	protein_coding	NM_002867
ASHGV40030290	-1.19801	1.198007067	protein_coding	NM_006944
ASHGV40020645	1.197936	1.197935791	protein_coding	NM_016077
ASHGV40046839	-1.1979	1.197900841	protein_coding	uc031syj.1
ASHGV40053694	1.197898	1.197898313	protein_coding	ENST00000361681
ASHGV40045663	-1.19734	1.197339027	protein_coding	NM_000045
ASHGV40026433	-1.19733	1.197332964	protein_coding	NM_022819
ASHGV40044818	1.197181	1.197180763	protein_coding	NM_018950
ASHGV40051712	1.197163	1.197162556	protein_coding	NM_032593
ASHGV40029899	1.196769	1.1967693	protein_coding	NM_032321
ASHGV40053705	1.196718	1.19671769	protein_coding	ENST00000361789
ASHGV40047952	-1.19668	1.19667703	protein_coding	uc022afe.1
ASHGV40037821	1.196623	1.196622818	protein_coding	NM_022154
ASHGV40035155	-1.19575	1.195752379	protein_coding	NM_020169
ASHGV40028560	1.195705	1.195704503	protein_coding	NM_022089
ASHGV40011898	1.195611	1.195611406	protein_coding	NM_020244
ASHGV40029749	1.195584	1.195584476	protein_coding	NM_172070
ASHGV40037752	1.19535	1.195350408	protein_coding	uc021xpz.1
ASHGV40046855	-1.19534	1.195336407	protein_coding	NM_024637
ASHGV40020805	-1.19513	1.195127809	protein_coding	NM_001144952
ASHGV40026020	-1.19509	1.195093052	protein_coding	NM_001012728
ASHGV40048096	1.1949	1.194900391	protein_coding	NM_021723
ASHGV40046751	1.194799	1.19479883	protein_coding	NM_003130
ASHGV40017951	1.194795	1.194795133	protein_coding	NM_033226
ASHGV40053494	1.194434	1.194433952	protein_coding	NM_016174

ASHGV40036997	1.194391	1.194391178	protein_coding	NM_015560
ASHGV40010115	1.193783	1.193782672	protein_coding	NM_001839
ASHGV40007091	1.193762	1.19376177	protein_coding	NM_001286077
ASHGV40047932	1.193745	1.193745237	protein_coding	NM_153363
ASHGV40049424	-1.19364	1.193644689	protein_coding	NM_033261
ASHGV40007796	-1.19347	1.193472553	protein_coding	NM_001004303
ASHGV40006448	1.193421	1.193421424	protein_coding	NM_024602
ASHGV40022605	-1.19328	1.193283399	protein_coding	NM_001001966
ASHGV40012692	-1.19286	1.192863377	protein_coding	NM_020866
ASHGV40006989	-1.1928	1.192799869	protein_coding	NM_003627
ASHGV40028385	-1.19269	1.192685867	protein_coding	NM_000547
ASHGV40018910	-1.19267	1.192672397	protein_coding	NM_001109660
ASHGV40034790	-1.19236	1.192358067	protein_coding	NM_018266
ASHGV40021101	1.192198	1.192198024	protein_coding	NM_001383
ASHGV40003284	-1.19204	1.192036632	protein_coding	NM_015545
ASHGV40018590	-1.19173	1.191734901	protein_coding	NM_004997
ASHGV40048937	1.190973	1.190972611	protein_coding	NM_139167
ASHGV40033785	-1.19095	1.190951529	protein_coding	NM_024821
ASHGV40008871	-1.19066	1.190664155	protein_coding	NM_025098
ASHGV40002150	-1.19037	1.190365953	protein_coding	ENST00000557574
ASHGV40054765	1.190354	1.190354112	protein_coding	NM_002970
ASHGV40012377	1.190132	1.190131989	protein_coding	NM_182488
ASHGV40037517	-1.18987	1.18987302	protein_coding	NM_152401
ASHGV40014897	1.189761	1.18976146	protein_coding	NM_032352
ASHGV40016323	1.189652	1.189651681	protein_coding	NM_000326
ASHGV40038300	-1.1891	1.189098173	protein_coding	NM_024041
ASHGV40000025	-1.18909	1.189091253	protein_coding	NM_001201380
ASHGV40005904	-1.189	1.188996962	protein_coding	NM_207373
ASHGV40005740	1.188816	1.188816497	protein_coding	NM_015634
ASHGV40054829	-1.18877	1.188765898	protein_coding	NM_000531
ASHGV40035974	1.188685	1.188684591	protein_coding	NM_004947
ASHGV40005609	1.188455	1.188454673	protein_coding	NM_001190810
ASHGV40013470	-1.18842	1.188418105	protein_coding	NM_001257995
ASHGV40040079	1.188403	1.188402766	protein_coding	NM_001040446
ASHGV40026827	-1.18837	1.188368406	protein_coding	NM_001309193
ASHGV40000233	-1.18837	1.188367038	protein_coding	ENST00000400553
ASHGV40009912	1.188355	1.188354686	protein_coding	NM_014470
ASHGV40010000	-1.18833	1.18833217	protein_coding	NM_002273
ASHGV40049951	1.188126	1.188125694	protein_coding	NM_016623
ASHGV40024373	-1.18812	1.188120845	protein_coding	uc031rkp.1
ASHGV40025053	1.187631	1.187631116	protein_coding	NM_018708
ASHGV40039298	-1.18755	1.187546584	protein_coding	NM_020897
ASHGV40050822	1.18701	1.18701043	protein_coding	NM_017866
ASHGV40030308	-1.18698	1.186977997	protein_coding	NM_014521
ASHGV40043928	1.186862	1.186861572	protein_coding	NM_003795
ASHGV40009371	1.186845	1.186844673	protein_coding	NM_001642
ASHGV40041337	-1.1864	1.18640308	protein_coding	NM_182632

ASHGV40005963	1.18635	1.186349597	protein_coding	NM_001549
ASHGV40009207	-1.18582	1.185824687	protein_coding	NM_005274
ASHGV40039003	-1.18578	1.185784885	protein_coding	NM_001074
ASHGV40035454	-1.18533	1.185329608	protein_coding	NM_001166305
ASHGV40003250	-1.1852	1.185202504	protein_coding	NM_002952
ASHGV40042056	1.185091	1.185090505	protein_coding	ENST00000431849
ASHGV40040723	-1.18498	1.184982116	protein_coding	NM_004106
ASHGV40045634	1.184717	1.184717072	protein_coding	NM_003287
ASHGV40026913	-1.18456	1.184564052	protein_coding	NM_015717
ASHGV40003349	-1.18442	1.184422825	protein_coding	NM_178568
ASHGV40010112	-1.18427	1.184267884	protein_coding	NM_000075
ASHGV40053449	-1.18358	1.18358053	protein_coding	NM_006195
ASHGV40019363	-1.18312	1.183119131	protein_coding	NM_005143
ASHGV40052756	1.182922	1.182921614	protein_coding	NM_016410
ASHGV40012720	1.182735	1.182735283	protein_coding	NM_014832
ASHGV40012090	-1.18272	1.182718153	protein_coding	NM_176818
ASHGV40025638	1.182572	1.182572011	protein_coding	NM_152606
ASHGV40044960	-1.18247	1.182467891	protein_coding	NM_021922
ASHGV40018587	1.182312	1.182311694	protein_coding	NM_002613
ASHGV40009009	1.182285	1.182285218	protein_coding	NM_004268
ASHGV40016560	1.182148	1.182147841	protein_coding	NM_005503
ASHGV40056841	-1.18203	1.182030259	protein_coding	NM_001243537
ASHGV40032209	-1.18178	1.181775975	protein_coding	NM_012183
ASHGV40019696	-1.18146	1.181459474	protein_coding	NM_003554
ASHGV40017886	1.180957	1.180956999	protein_coding	NM_197962
ASHGV40012320	1.180661	1.18066149	protein_coding	NM_152726
ASHGV40009779	1.180637	1.18063671	protein_coding	NM_001002259
ASHGV40007675	-1.18031	1.180310064	protein_coding	NM_052968
ASHGV40045241	-1.18024	1.180236718	protein_coding	NM_001858
ASHGV40018220	-1.18021	1.180207285	protein_coding	NM_001252100
ASHGV40055846	1.180071	1.180070643	protein_coding	NM_006434
ASHGV40033557	-1.17984	1.179844488	protein_coding	uc003adf.2
ASHGV40018858	-1.17979	1.179785408	protein_coding	NM_001145545
ASHGV40028807	1.179686	1.179686494	protein_coding	NM_005400
ASHGV40048149	1.17853	1.178530429	protein_coding	NM_022900
ASHGV40035371	-1.17822	1.178219091	protein_coding	NM_017541
ASHGV40003073	1.178137	1.178137285	protein_coding	NM_001008528
ASHGV40005449	1.178012	1.178012232	protein_coding	NM_016628
ASHGV40019905	-1.178	1.178004958	protein_coding	NM_001007530
ASHGV40041464	-1.17795	1.17794815	protein_coding	NM_002995
ASHGV40050890	1.177863	1.177863073	protein_coding	NM_173848
ASHGV40052928	1.177729	1.177729373	protein_coding	NM_003558
ASHGV40060893	-1.17772	1.177718514	protein_coding	uc021rpv.1
ASHGV40047019	1.17756	1.177559826	protein_coding	NM_003133
ASHGV40049523	1.177352	1.1773521	protein_coding	NM_018440
ASHGV40056281	-1.1773	1.177297821	protein_coding	NM_152458
ASHGV40002604	-1.17713	1.17713046	protein_coding	ENST00000594763

ASHGV40038870	-1.17624	1.176235976	protein_coding	NM_130898
ASHGV40057768	-1.17599	1.175987019	protein_coding	ENST00000398992
ASHGV40030054	1.175754	1.175754244	protein_coding	NM_002374
ASHGV40042290	1.175499	1.17549882	protein_coding	NM_020240
ASHGV40010735	1.175194	1.17519428	protein_coding	NM_178314
ASHGV40057775	-1.17505	1.175048469	protein_coding	NM_032667
ASHGV40011567	-1.17496	1.174957324	protein_coding	NM_015433
ASHGV40047715	-1.17465	1.174654531	protein_coding	ENST00000381493
ASHGV40037736	1.174612	1.174611734	protein_coding	NM_138982
ASHGV40005718	1.174425	1.174424855	protein_coding	NM_000310
ASHGV40027466	-1.17405	1.174050095	protein_coding	NM_025052
ASHGV40048971	1.173855	1.173854623	protein_coding	NM_018371
ASHGV40008020	-1.17375	1.173754977	protein_coding	NM_021616
ASHGV40008903	-1.17359	1.173589792	protein_coding	NM_003251
ASHGV40020314	-1.17356	1.173560417	protein_coding	NM_031964
ASHGV40038594	1.17333	1.173329622	protein_coding	NM_000798
ASHGV40029706	1.173278	1.173278367	protein_coding	NM_005805
ASHGV40024881	-1.17325	1.173245872	protein_coding	NM_001144989
ASHGV40012578	1.172658	1.172658327	protein_coding	NM_022359
ASHGV40015973	-1.17237	1.172370652	protein_coding	NM_001218
ASHGV40014094	-1.17234	1.172344207	protein_coding	uc001xbd.1
ASHGV40025718	-1.17206	1.172062879	protein_coding	NM_000774
ASHGV40027256	1.171936	1.171935866	protein_coding	NM_020379
ASHGV40006502	1.171863	1.171862959	protein_coding	NM_001909
ASHGV40024932	-1.17183	1.171831069	protein_coding	NM_173481
ASHGV40056073	-1.17182	1.171821785	protein_coding	NM_001145442
ASHGV40017510	1.171799	1.17179891	protein_coding	NM_007108
ASHGV40053144	1.171526	1.171526098	protein_coding	NM_177995
ASHGV40019963	1.170966	1.17096609	protein_coding	NM_001082968
ASHGV40019728	-1.17094	1.170937595	protein_coding	NM_001140
ASHGV40035270	1.1709	1.170900279	protein_coding	NM_024665
ASHGV40029248	1.170528	1.170527637	protein_coding	ENST00000414004
ASHGV40036381	-1.17046	1.170456324	protein_coding	NM_001014980
ASHGV40043394	-1.17021	1.170207539	protein_coding	NM_003214
ASHGV40022784	-1.16977	1.169769438	protein_coding	NM_145020
ASHGV40022756	-1.16958	1.169579985	protein_coding	NM_016097
ASHGV40029399	1.169088	1.169087708	protein_coding	NM_006770
ASHGV40007500	1.169075	1.169075066	protein_coding	NM_024725
ASHGV40007143	-1.16899	1.168989795	protein_coding	NM_017525
ASHGV40036582	1.16899	1.168989569	protein_coding	NM_018147
ASHGV40026961	-1.16891	1.168905032	protein_coding	NM_032603
ASHGV40033511	1.168812	1.168812491	protein_coding	NM_000854
ASHGV40017065	1.16866	1.168659706	protein_coding	NM_138967
ASHGV40047005	-1.16863	1.16862723	protein_coding	NM_012252
ASHGV40040253	1.168519	1.168518595	protein_coding	NM_003711
ASHGV40016878	-1.16838	1.168378584	protein_coding	ENST00000599727
ASHGV40034564	1.168307	1.168306799	protein_coding	NM_020872

ASHGV40043404	-1.16825	1.168254871	protein_coding	NM_001010903
ASHGV40037631	-1.16811	1.168113163	protein_coding	NM_002704
ASHGV40019265	-1.16805	1.168053128	protein_coding	uc002erg.1
ASHGV40010577	1.168004	1.168004391	protein_coding	NM_207435
ASHGV40045210	-1.16798	1.167980101	protein_coding	NM_000947
ASHGV40043308	-1.16767	1.167668576	protein_coding	NM_001136
ASHGV40043171	-1.16747	1.167468527	protein_coding	NM_003510
ASHGV40039903	1.167414	1.167413546	protein_coding	NM_032286
ASHGV40017560	1.167333	1.167332646	protein_coding	NM_020677
ASHGV40026030	1.167264	1.167263748	protein_coding	NM_002739
ASHGV40023694	1.167216	1.167216102	protein_coding	NM_199054
ASHGV40014968	1.167035	1.167034709	protein_coding	NM_014315
ASHGV40020168	-1.16689	1.166889485	protein_coding	NM_024746
ASHGV40020481	-1.16651	1.166510268	protein_coding	NM_199262
ASHGV40045893	-1.16629	1.166290106	protein_coding	NM_001242384
ASHGV40054253	-1.16574	1.165743768	protein_coding	NM_032946
ASHGV40051741	-1.16518	1.165175186	protein_coding	NM_001007563
ASHGV40019321	1.165024	1.165024318	protein_coding	NM_030579
ASHGV40039352	1.164996	1.164995995	protein_coding	NM_002006
ASHGV40041282	1.164785	1.164785036	protein_coding	NM_002752
ASHGV40033204	-1.16468	1.164681594	protein_coding	NM_032758
ASHGV40013453	-1.16449	1.164492086	protein_coding	NM_001286747
ASHGV40027639	1.16446	1.164460266	protein_coding	NM_006600
ASHGV40020639	1.164415	1.164414751	protein_coding	NM_015294
ASHGV40026878	1.163726	1.163725948	protein_coding	NM_015463
ASHGV40024541	1.163525	1.163524967	protein_coding	NM_001012643
ASHGV40047312	-1.16339	1.163394332	protein_coding	NM_001142459
ASHGV40053528	-1.16337	1.163367597	protein_coding	NM_014506
ASHGV40025105	-1.16337	1.163366077	protein_coding	NM_006949
ASHGV40049654	1.16323	1.163229692	protein_coding	NM_057749
ASHGV40052751	1.163164	1.163163503	protein_coding	NM_001539
ASHGV40020654	1.162791	1.162791273	protein_coding	NM_032582
ASHGV40000188	-1.16257	1.162566346	protein_coding	NM_020363
ASHGV40029558	-1.16239	1.162388841	protein_coding	NM_001080427
ASHGV40019926	-1.16225	1.162245378	protein_coding	NM_006382
ASHGV40023977	-1.16206	1.162064878	protein_coding	NM_024323
ASHGV40026605	1.16197	1.161970052	protein_coding	ENST00000593798
ASHGV40024429	-1.16171	1.16171052	protein_coding	NM_001288583
ASHGV40005289	1.161159	1.161158928	protein_coding	NM_001001973
ASHGV40031579	-1.16096	1.160955988	protein_coding	NM_004139
ASHGV40057774	-1.16074	1.160738341	protein_coding	NM_203422
ASHGV40028753	-1.16064	1.160641673	protein_coding	NM_138801
ASHGV40045128	-1.16058	1.160575279	protein_coding	NM_153838
ASHGV40010036	1.160534	1.160534297	protein_coding	NM_006741
ASHGV40050272	-1.16035	1.160346051	protein_coding	ENST00000525043
ASHGV40036415	1.160095	1.16009487	protein_coding	NM_016589
ASHGV40026608	1.15993	1.159929986	protein_coding	NM_002759

ASHGV40042467	-1.15976	1.159763313	protein_coding	NM_018914
ASHGV40031599	-1.15976	1.159755892	protein_coding	NM_174936
ASHGV40051398	1.159399	1.159398631	protein_coding	NM_000837
ASHGV40054934	-1.15919	1.159194163	protein_coding	NM_001079900
ASHGV40045356	-1.15908	1.159083713	protein_coding	NM_001010868
ASHGV40007039	-1.1587	1.158703411	protein_coding	NM_004778
ASHGV40043329	1.158533	1.158533441	protein_coding	NM_001290043
ASHGV40014017	1.158441	1.158441321	protein_coding	NM_001113498
ASHGV40033207	1.158363	1.158362602	protein_coding	NM_015704
ASHGV40000219	-1.1581	1.158103821	protein_coding	ENST00000397897
ASHGV40021614	1.157857	1.157856587	protein_coding	NM_033315
ASHGV40014350	-1.15734	1.157341199	protein_coding	NM_199296
ASHGV40051874	1.157182	1.157181635	protein_coding	NM_004297
ASHGV40047068	-1.15693	1.15693087	protein_coding	NM_022444
ASHGV40036177	-1.15685	1.156851841	protein_coding	NM_138959
ASHGV40055538	1.156602	1.156602036	protein_coding	uc022cho.1
ASHGV40047554	-1.15659	1.156589631	protein_coding	NM_001195280
ASHGV40015812	-1.15611	1.156107022	protein_coding	NM_001301168
ASHGV40027576	-1.15588	1.155877166	protein_coding	NM_001256368
ASHGV40048840	-1.15586	1.155860045	protein_coding	NM_001925
ASHGV40009153	-1.1558	1.155800461	protein_coding	NM_001101389
ASHGV40006124	-1.15535	1.155350418	protein_coding	NM_152310
ASHGV40020006	-1.15477	1.154771947	protein_coding	NM_001042685
ASHGV40001458	1.154754	1.154754442	protein_coding	NM_001008388
ASHGV40053769	1.154456	1.154455666	protein_coding	NM_006327
ASHGV40003149	-1.15436	1.154361314	protein_coding	NM_001198759
ASHGV40017745	1.153932	1.153932014	protein_coding	NM_005003
ASHGV40041024	1.153903	1.153902637	protein_coding	NM_003118
ASHGV40006157	1.153889	1.153889458	protein_coding	NM_022821
ASHGV40045797	1.153808	1.153807752	protein_coding	NM_001127715
ASHGV40010723	-1.15372	1.153716207	protein_coding	NM_006018
ASHGV40037868	-1.15353	1.15353037	protein_coding	NM_001226
ASHGV40007105	1.15346	1.153459994	protein_coding	NM_001981
ASHGV40022860	-1.15342	1.153421413	protein_coding	NM_173557
ASHGV40037729	1.153069	1.153069328	protein_coding	NM_014991
ASHGV40045072	-1.15295	1.152953756	protein_coding	NM_000707
ASHGV40025605	-1.15237	1.152370203	protein_coding	NM_001290056
ASHGV40048401	-1.1521	1.152096364	protein_coding	NM_012269
ASHGV40003183	-1.15195	1.151952425	protein_coding	NM_001204871
ASHGV40043036	1.151913	1.151913294	protein_coding	NM_018988
ASHGV40049161	-1.15173	1.151725123	protein_coding	NM_001102559
ASHGV40032754	-1.15142	1.151415836	protein_coding	NM_198695
ASHGV40022120	1.15139	1.151389785	protein_coding	NM_016627
ASHGV40030122	1.150979	1.150978796	protein_coding	NM_005731
ASHGV40040909	-1.15088	1.150878586	protein_coding	NM_194249
ASHGV40038431	-1.15087	1.150874662	protein_coding	uc001bhd.4
ASHGV40025564	-1.15077	1.15077421	protein_coding	NM_006474

ASHGV40015693	-1.15073	1.150734618	protein_coding	NM_017625
ASHGV40009902	-1.1507	1.150700206	protein_coding	NM_001287737
ASHGV40019034	1.150467	1.150466608	protein_coding	NM_031490
ASHGV40023403	1.150458	1.150458182	protein_coding	NM_004163
ASHGV40007141	-1.15033	1.150333835	protein_coding	uc010rnp.1
ASHGV40014645	-1.15022	1.150222703	protein_coding	NM_013345
ASHGV40031659	-1.14997	1.149973935	protein_coding	NM_003881
ASHGV40054706	-1.14995	1.149952126	protein_coding	NM_015507
ASHGV40052336	1.149745	1.149744645	protein_coding	NM_001114753
ASHGV40037318	1.149172	1.149172318	protein_coding	NM_173463
ASHGV40006617	1.149162	1.149162141	protein_coding	NM_016229
ASHGV40038485	-1.14896	1.148956502	protein_coding	NM_003023
ASHGV40025659	1.14886	1.148860053	protein_coding	NM_013234
ASHGV40042158	1.148546	1.148546476	protein_coding	ENST00000600409
ASHGV40053839	1.148502	1.148501974	protein_coding	NM_014332
ASHGV40009951	1.148385	1.148385088	protein_coding	NM_002702
ASHGV40035057	-1.14832	1.148322021	protein_coding	NM_032153
ASHGV40024673	1.147873	1.147873301	protein_coding	NM_016148
ASHGV40025592	-1.14778	1.147775398	protein_coding	uc021usu.2
ASHGV40024681	1.147723	1.147722656	protein_coding	NM_005046
ASHGV40029051	-1.14694	1.146939944	protein_coding	NM_000189
ASHGV40046797	1.146678	1.146677762	protein_coding	NM_000305
ASHGV40038516	-1.14592	1.145921424	protein_coding	NM_014357
ASHGV40018671	-1.14578	1.145777343	protein_coding	NM_000303
ASHGV40009098	-1.14567	1.145673894	protein_coding	NM_017516
ASHGV40022646	1.145478	1.145477552	protein_coding	NM_014939
ASHGV40005458	-1.14513	1.145127233	protein_coding	NM_001278522
ASHGV40033975	-1.14499	1.144989002	protein_coding	NM_015931
ASHGV40005756	-1.14497	1.144970318	protein_coding	NM_018649
ASHGV40016468	1.144848	1.144848251	protein_coding	NM_152334
ASHGV40053961	-1.14476	1.144756462	protein_coding	NM_001039891
ASHGV40023779	1.144742	1.14474195	protein_coding	NM_002034
ASHGV40005890	-1.14468	1.144682951	protein_coding	NM_207372
ASHGV40007150	-1.14449	1.14448975	protein_coding	NM_005468
ASHGV40022072	-1.14435	1.144351682	protein_coding	NM_005828
ASHGV40033050	1.143753	1.143753491	protein_coding	NM_004582
ASHGV40049681	1.143617	1.143617465	protein_coding	NM_000989
ASHGV40031272	-1.14354	1.143536209	protein_coding	uc002wly.1
ASHGV40025004	1.143534	1.143534162	protein_coding	NM_052960
ASHGV40011529	-1.14353	1.14352848	protein_coding	NM_016584
ASHGV40018209	1.143251	1.143251499	protein_coding	NM_138383
ASHGV40044929	-1.14296	1.142958178	protein_coding	NM_002224
ASHGV40000023	1.142466	1.1424661	protein_coding	NM_005054
ASHGV40039139	-1.14244	1.142440529	protein_coding	NM_004967
ASHGV40012585	1.142375	1.142375086	protein_coding	NM_001306135
ASHGV40039818	1.142326	1.142326311	protein_coding	ENST00000382776
ASHGV40034411	-1.14194	1.141939174	protein_coding	NM_022908

ASHGV40054045	1.141714	1.141714419	protein_coding	NM_018045
ASHGV40006642	1.141708	1.141708169	protein_coding	NM_015213
ASHGV40009400	-1.14161	1.141606028	protein_coding	NM_138342
ASHGV40000402	-1.14145	1.141450174	protein_coding	NM_013353
ASHGV40040261	-1.14134	1.141335252	protein_coding	NM_001287053
ASHGV40037431	-1.14063	1.140634235	protein_coding	NM_001098634
ASHGV40020299	-1.14058	1.140580027	protein_coding	NM_031957
ASHGV40009982	-1.14057	1.14057495	protein_coding	NM_005555
ASHGV40010734	1.140493	1.140493374	protein_coding	ENST00000280571
ASHGV40007913	-1.14046	1.140455224	protein_coding	NM_022772
ASHGV40055871	-1.14024	1.140243095	protein_coding	ENST00000439965
ASHGV40031969	1.139872	1.139872037	protein_coding	NM_003195
ASHGV40047184	1.139796	1.139795589	protein_coding	ENST00000589735
ASHGV40054137	-1.13974	1.139739918	protein_coding	ENST00000430888
ASHGV40014690	1.13961	1.139609978	protein_coding	NM_018489
ASHGV40000520	-1.13945	1.139453223	protein_coding	ENST00000421491
ASHGV40010082	1.139394	1.139394228	protein_coding	NM_006601
ASHGV40054407	-1.13902	1.139024982	protein_coding	NM_138289
ASHGV40011957	1.138696	1.138696347	protein_coding	NM_213595
ASHGV40039329	1.138675	1.138674571	protein_coding	NM_019050
ASHGV40022369	-1.13859	1.138591489	protein_coding	NM_004207
ASHGV40008463	-1.13826	1.138264081	protein_coding	NM_001220494
ASHGV40052522	1.138179	1.138178679	protein_coding	NM_001606
ASHGV40028859	-1.1381	1.138100325	protein_coding	NM_001100396
ASHGV40013208	1.137922	1.137921789	protein_coding	NM_015678
ASHGV40056093	1.137875	1.137875044	protein_coding	NM_002818
ASHGV40055328	1.13757	1.13756956	protein_coding	NM_001152
ASHGV40053809	1.137546	1.137546224	protein_coding	NM_002893
ASHGV40033150	1.137391	1.137391184	protein_coding	NM_013356
ASHGV40046387	1.136909	1.136909433	protein_coding	NM_014396
ASHGV40051875	1.136888	1.136887937	protein_coding	NM_002072
ASHGV40042737	1.136725	1.136724717	protein_coding	NM_153607
ASHGV40007040	1.13656	1.136560048	protein_coding	NM_014502
ASHGV40022674	-1.13645	1.136448188	protein_coding	NM_001001821
ASHGV40057263	1.136377	1.136377132	protein_coding	NM_031287
ASHGV40047257	-1.13628	1.136282219	protein_coding	NM_178561
ASHGV40016679	-1.13614	1.13613633	protein_coding	NM_170589
ASHGV40036292	1.136017	1.136017291	protein_coding	NM_145037
ASHGV40050416	1.135963	1.13596318	protein_coding	NM_001386
ASHGV40030707	1.135687	1.135686557	protein_coding	NM_001042472
ASHGV40029878	1.135672	1.135671881	protein_coding	NM_002210
ASHGV40049101	-1.13567	1.135668595	protein_coding	NM_015431
ASHGV40010288	1.135668	1.135668061	protein_coding	NM_024685
ASHGV40052253	1.135412	1.135411541	protein_coding	NM_005658
ASHGV40048886	-1.13475	1.134752028	protein_coding	NM_178857
ASHGV40006619	-1.1347	1.134703454	protein_coding	NM_198185
ASHGV40028470	1.134607	1.134607042	protein_coding	NM_001039613

ASHGV40046154	1.134587	1.13458684	protein_coding	NM_004968
ASHGV40057351	1.134395	1.134395459	protein_coding	NM_001146152
ASHGV40024752	-1.13431	1.134309711	protein_coding	NM_024733
ASHGV40034405	1.134091	1.13409108	protein_coding	NM_007284
ASHGV40016911	1.134053	1.134053387	protein_coding	NM_015059
ASHGV40022227	1.133947	1.133947281	protein_coding	NM_001126
ASHGV40042068	1.133933	1.13393322	protein_coding	NM_199243
ASHGV40038108	-1.1337	1.13370137	protein_coding	ENST00000538795
ASHGV40054675	-1.1335	1.133498773	protein_coding	ENST00000445307
ASHGV40051866	1.133214	1.133214288	protein_coding	NM_018339
ASHGV40030449	-1.13309	1.133090999	protein_coding	NM_018556
ASHGV40033745	-1.13275	1.132747502	protein_coding	NM_181773
ASHGV40019251	-1.13254	1.132536439	protein_coding	NM_144673
ASHGV40000008	-1.13253	1.132531082	protein_coding	ENST00000201961
ASHGV40024803	1.132244	1.132243904	protein_coding	NM_007262
ASHGV40029041	-1.13177	1.131767914	protein_coding	NM_012477
ASHGV40007601	1.131622	1.131622469	protein_coding	NM_002906
ASHGV40026854	1.131617	1.131616556	protein_coding	NM_181784
ASHGV40045557	-1.13135	1.131351533	protein_coding	NM_002356
ASHGV40032570	1.131245	1.131245297	protein_coding	NM_181605
ASHGV40021016	-1.13112	1.131122498	protein_coding	NM_178493
ASHGV40015671	1.131086	1.131086293	protein_coding	NM_020154
ASHGV40042149	1.131008	1.131007842	protein_coding	NM_001744
ASHGV40015990	-1.13086	1.130864882	protein_coding	NM_022048
ASHGV40033739	-1.13084	1.130838427	protein_coding	NM_004900
ASHGV40024906	1.130775	1.130774575	protein_coding	NM_003969
ASHGV40015146	1.130408	1.130407764	protein_coding	NM_006925
ASHGV40052611	-1.13029	1.130294825	protein_coding	NM_020829
ASHGV40031548	-1.13008	1.130081023	protein_coding	NM_001207076
ASHGV40024702	-1.12935	1.12935255	protein_coding	NM_014442
ASHGV40005108	1.129129	1.129129453	protein_coding	NM_001282547
ASHGV40048589	1.12896	1.128959793	protein_coding	NM_153345
ASHGV40027741	-1.12857	1.128566519	protein_coding	NM_001080458
ASHGV40014287	-1.12853	1.12852922	protein_coding	NM_178351
ASHGV40055872	-1.12815	1.128146565	protein_coding	NM_016046
ASHGV40018259	1.128016	1.128015926	protein_coding	NM_006324
ASHGV40019331	1.127943	1.12794324	protein_coding	uc002eyu.3
ASHGV40007991	-1.12763	1.127632343	protein_coding	NM_001256240
ASHGV40057813	-1.12762	1.127621696	protein_coding	NM_003002
ASHGV40048798	1.12707	1.127069821	protein_coding	NM_032328
ASHGV40013380	1.125944	1.125944059	protein_coding	NM_001040429
ASHGV40006635	-1.12586	1.125858261	protein_coding	uc001mgo.3
ASHGV40044045	1.125836	1.125835517	protein_coding	NM_001297707
ASHGV40042486	1.125617	1.125617454	protein_coding	NM_015602
ASHGV40012435	1.125599	1.125599425	protein_coding	NM_052818
ASHGV40000248	1.125136	1.125136154	protein_coding	uc011aic.1
ASHGV40025542	1.125011	1.125011021	protein_coding	NM_000175

ASHGV40043279	-1.1249	1.124903452	protein_coding	NM_025258
ASHGV40039752	1.124727	1.12472696	protein_coding	NM_031953
ASHGV40060852	-1.12469	1.12468858	protein_coding	uc004cnm.1
ASHGV40034878	-1.12467	1.12467201	protein_coding	uc021xdl.1
ASHGV40015836	1.12466	1.124660256	protein_coding	NM_203349
ASHGV40025323	-1.12447	1.124471774	protein_coding	NM_001278443
ASHGV40032652	-1.12444	1.124443547	protein_coding	NM_018962
ASHGV40052915	-1.12431	1.124312957	protein_coding	ENST00000322495
ASHGV40030466	1.124132	1.124132136	protein_coding	NM_001282533
ASHGV40046955	-1.1241	1.124098256	protein_coding	NM_001373
ASHGV40037464	1.124045	1.124044743	protein_coding	NM_000807
ASHGV40028727	-1.12399	1.123986778	protein_coding	NM_001177969
ASHGV40051916	1.123984	1.123983676	protein_coding	NM_015239
ASHGV40033159	1.123908	1.123907651	protein_coding	NM_006386
ASHGV40026526	1.123792	1.123791872	protein_coding	NM_014860
ASHGV40041063	-1.12378	1.123776424	protein_coding	NM_178424
ASHGV40005400	1.123762	1.123761826	protein_coding	NM_012228
ASHGV40012239	1.123698	1.123697884	protein_coding	NM_006325
ASHGV40049953	1.123564	1.123564339	protein_coding	NM_018482
ASHGV40005997	-1.12343	1.123431839	protein_coding	NM_004523
ASHGV40001401	-1.1233	1.12329758	protein_coding	ENST00000488788
ASHGV40030141	-1.12328	1.123283639	protein_coding	NM_006522
ASHGV40051921	1.122979	1.122978863	protein_coding	NM_030940
ASHGV40034286	1.122947	1.12294719	protein_coding	NM_003760
ASHGV40000349	-1.1228	1.12280038	protein_coding	NM_001044264
ASHGV40003074	-1.12258	1.122575971	protein_coding	NM_014984
ASHGV40024573	1.122486	1.122485994	protein_coding	NM_017854
ASHGV40028630	-1.12246	1.122456818	protein_coding	NM_145038
ASHGV40054767	1.122343	1.122343156	protein_coding	NM_001415
ASHGV40032368	-1.12228	1.12227528	protein_coding	NM_198693
ASHGV40057665	-1.12217	1.122165053	protein_coding	NM_000513
ASHGV40006438	1.121968	1.121968369	protein_coding	NM_152643
ASHGV40048839	-1.12191	1.121907246	protein_coding	NM_001926
ASHGV40019162	1.121901	1.121901003	protein_coding	NM_018566
ASHGV40042111	1.121756	1.121756033	protein_coding	NM_001177306
ASHGV40015738	1.121585	1.121584777	protein_coding	NM_207380
ASHGV40030965	1.121532	1.121532317	protein_coding	NM_021035
ASHGV40013953	1.120716	1.120716163	protein_coding	NM_017917
ASHGV40008449	-1.12064	1.120635239	protein_coding	NM_000062
ASHGV40047612	1.120462	1.120461586	protein_coding	NM_001127364
ASHGV40022651	1.120196	1.120196144	protein_coding	NM_001242409
ASHGV40044912	1.119992	1.119992248	protein_coding	NM_002121
ASHGV40029805	-1.11998	1.119983492	protein_coding	NM_024501
ASHGV40046061	1.119175	1.119175069	protein_coding	NM_001284309
ASHGV40052013	1.119056	1.119056253	protein_coding	NM_198841
ASHGV40034696	-1.11854	1.118538831	protein_coding	NM_170662
ASHGV40019729	1.118479	1.118478721	protein_coding	NM_014389

ASHGV40007190	-1.11839	1.118391717	protein_coding	uc001ogu.1
ASHGV40046696	1.118312	1.118311921	protein_coding	NM_017439
ASHGV40021728	-1.1182	1.118198522	protein_coding	NM_000805
ASHGV40010196	-1.11811	1.118114378	protein_coding	NM_021150
ASHGV40023733	1.117907	1.117907221	protein_coding	NM_015174
ASHGV40017687	1.117785	1.117784841	protein_coding	NM_015161
ASHGV40032556	1.117596	1.117595956	protein_coding	NM_006447
ASHGV40057472	-1.11752	1.117515948	protein_coding	NM_001204180
ASHGV40026163	1.117492	1.117491724	protein_coding	NM_014480
ASHGV40048488	1.117485	1.117485167	protein_coding	NM_013255
ASHGV40037713	1.117468	1.117467829	protein_coding	NM_001037582
ASHGV40024321	1.117361	1.117361446	protein_coding	NM_152279
ASHGV40057051	-1.11712	1.117121591	protein_coding	uc003ihk.1
ASHGV40040252	1.116788	1.116787959	protein_coding	NM_019030
ASHGV40033238	-1.11673	1.116733661	protein_coding	uc003bds.3
ASHGV40006936	1.116535	1.116534707	protein_coding	NM_031909
ASHGV40024440	-1.11624	1.116236048	protein_coding	NM_002573
ASHGV40028941	-1.11619	1.116190177	protein_coding	NM_024676
ASHGV40024384	1.115519	1.115519382	protein_coding	NM_006483
ASHGV40009906	-1.11511	1.115106994	protein_coding	NM_002289
ASHGV40017410	1.114779	1.114778539	protein_coding	NM_201412
ASHGV40030282	-1.11446	1.114462178	protein_coding	NM_019077
ASHGV40046300	-1.11432	1.114322665	protein_coding	NM_024014
ASHGV40002491	-1.11417	1.11417462	protein_coding	ENST00000585033
ASHGV40048657	-1.11388	1.113877863	protein_coding	NM_023942
ASHGV40054315	1.113805	1.113804875	protein_coding	NM_022977
ASHGV40052755	-1.11361	1.113610642	protein_coding	NM_014471
ASHGV40046816	1.113583	1.113583398	protein_coding	NM_001673
ASHGV40041009	1.113392	1.113392293	protein_coding	NM_016221
ASHGV40034455	-1.11319	1.113191563	protein_coding	NM_003865
ASHGV40014078	1.113114	1.113114299	protein_coding	NM_001160148
ASHGV40037004	-1.1131	1.113098216	protein_coding	NM_005524
ASHGV40032902	-1.11271	1.112714882	protein_coding	NM_015978
ASHGV40001286	-1.11255	1.112552175	protein_coding	ENST00000463937
ASHGV40051683	1.112498	1.112497565	protein_coding	NM_007234
ASHGV40024290	-1.11247	1.112466228	protein_coding	NM_004646
ASHGV40008141	1.112463	1.112463346	protein_coding	NM_014267
ASHGV40007125	-1.11242	1.112424522	protein_coding	NM_138689
ASHGV40042456	-1.1124	1.112396407	protein_coding	NM_018918
ASHGV40027672	1.11227	1.112269558	protein_coding	NM_013233
ASHGV40032734	1.111971	1.111970824	protein_coding	NM_020132
ASHGV40022233	1.11159	1.11159037	protein_coding	NM_000213
ASHGV40039282	1.111532	1.111531857	protein_coding	NM_015454
ASHGV40057657	-1.11114	1.11113956	protein_coding	NM_005363
ASHGV40041472	-1.11095	1.110953116	protein_coding	NM_001164440
ASHGV40032271	1.110859	1.110858595	protein_coding	NM_003720
ASHGV40005625	-1.11031	1.110307423	protein_coding	NM_001720

ASHGV40013813	-1.1102	1.11019741	protein_coding	NM_001005465
ASHGV40023660	-1.1098	1.109801562	protein_coding	NM_001270366
ASHGV40032759	-1.10965	1.109649627	protein_coding	NM_198699
ASHGV40011713	-1.10964	1.109643442	protein_coding	NM_001304964
ASHGV40053225	-1.1096	1.109597211	protein_coding	NM_003692
ASHGV40029828	1.109526	1.109526082	protein_coding	NM_032523
ASHGV40053085	-1.10897	1.108968085	protein_coding	NM_001001938
ASHGV40025268	-1.10889	1.108893292	protein_coding	NM_001010881
ASHGV40011497	-1.10874	1.108737521	protein_coding	NM_001005519
ASHGV40045858	1.108705	1.108705124	protein_coding	NM_001010927
ASHGV40035206	1.108134	1.108133929	protein_coding	NM_024687
ASHGV40023856	-1.10794	1.107942538	protein_coding	NM_152476
ASHGV40010164	-1.10779	1.107791084	protein_coding	NM_001170633
ASHGV40005139	1.107455	1.107455044	protein_coding	NM_173575
ASHGV40055723	1.107321	1.107320978	protein_coding	NM_001278612
ASHGV40054724	-1.10686	1.106857378	protein_coding	NM_005089
ASHGV40022169	1.106648	1.106647924	protein_coding	NM_018714
ASHGV40027799	-1.10662	1.106619412	protein_coding	NM_001463
ASHGV40040093	-1.1061	1.106098676	protein_coding	NM_030955
ASHGV40016081	1.106074	1.10607371	protein_coding	NM_020147
ASHGV40025489	1.106028	1.106028255	protein_coding	NM_014717
ASHGV40019837	-1.106	1.106003445	protein_coding	NM_001128076
ASHGV40054095	-1.10572	1.105719341	protein_coding	NM_021783
ASHGV40022030	1.105709	1.105709033	protein_coding	NM_058216
ASHGV40052864	1.10528	1.105280093	protein_coding	ENST00000377391
ASHGV40040980	1.105166	1.105165518	protein_coding	NM_000870
ASHGV40046119	-1.10512	1.105119666	protein_coding	uc011jwh.2
ASHGV40042474	1.105029	1.105029019	protein_coding	NM_173828
ASHGV40009941	-1.10489	1.10489438	protein_coding	NM_147190
ASHGV40018517	-1.10484	1.10484273	protein_coding	NM_001053
ASHGV40010935	1.104483	1.104482765	protein_coding	NM_001130862
ASHGV40037472	1.104143	1.104142556	protein_coding	NM_017845
ASHGV40027009	1.104072	1.104072121	protein_coding	NM_003849
ASHGV40015700	-1.10403	1.104026086	protein_coding	NM_080878
ASHGV40043554	1.103957	1.103957153	protein_coding	NM_005084
ASHGV40056208	-1.10382	1.103823571	protein_coding	NM_001267536
ASHGV40042217	-1.1036	1.103599869	protein_coding	NM_182761
ASHGV40053819	-1.10346	1.103459216	protein_coding	NM_000330
ASHGV40030402	1.103087	1.103087412	protein_coding	NM_000374
ASHGV40018867	1.102845	1.102845111	protein_coding	NM_015375
ASHGV40026835	-1.10275	1.102746744	protein_coding	NM_020651
ASHGV40035173	1.102681	1.102681148	protein_coding	NM_003781
ASHGV40019944	-1.10253	1.102529734	protein_coding	NM_012452
ASHGV40012468	-1.10246	1.102459732	protein_coding	NM_016179
ASHGV40056626	-1.10246	1.102455321	protein_coding	NM_006872
ASHGV40020330	1.102221	1.102221446	protein_coding	NM_000526
ASHGV40011562	1.102031	1.102031273	protein_coding	NM_015906

ASHGV40042844	1.101436	1.101435731	protein_coding	NM_001746
ASHGV40006139	1.101412	1.101411774	protein_coding	NM_001143909
ASHGV40029200	1.101389	1.101389169	protein_coding	NM_212481
ASHGV40057209	-1.10117	1.10117008	protein_coding	NM_004640
ASHGV40019776	-1.10116	1.101157668	protein_coding	NM_080913
ASHGV40005038	-1.10108	1.10108234	protein_coding	NM_198148
ASHGV40027841	1.100986	1.100985899	protein_coding	NM_016311
ASHGV40054461	1.100731	1.100731483	protein_coding	NM_173470
ASHGV40050111	1.100663	1.100663432	protein_coding	NM_078480
ASHGV40033421	1.100419	1.100418522	protein_coding	NM_000754
ASHGV40024792	-1.10028	1.100277736	protein_coding	NM_198988
ASHGV40023886	1.100152	1.100152341	protein_coding	NM_007065
ASHGV40021541	-1.09976	1.099759465	protein_coding	NM_024857
ASHGV40056600	1.09962	1.099620176	protein_coding	NM_138466
ASHGV40006602	1.099458	1.099457891	protein_coding	NM_006458
ASHGV40015503	-1.09943	1.099426959	protein_coding	NM_030943
ASHGV40028712	1.099316	1.099316398	protein_coding	NM_170672
ASHGV40014906	-1.09913	1.099128509	protein_coding	NM_006194
ASHGV40029662	-1.09908	1.099076852	protein_coding	NM_007102
ASHGV40006607	-1.09882	1.098815174	protein_coding	uc021qdb.1
ASHGV40040423	-1.09875	1.098749859	protein_coding	NM_005598
ASHGV40039369	1.098422	1.098422305	protein_coding	NM_014278
ASHGV40032164	-1.09806	1.098063569	protein_coding	NM_181602
ASHGV40023717	-1.0979	1.097897517	protein_coding	NM_021217
ASHGV40008165	-1.0976	1.0976038	protein_coding	NM_144972
ASHGV40048433	-1.09742	1.097420837	protein_coding	NM_013332
ASHGV40035684	1.097337	1.097337039	protein_coding	NM_004162
ASHGV40010594	-1.09721	1.097207315	protein_coding	NM_144671
ASHGV40030883	1.096967	1.096966702	protein_coding	NM_145652
ASHGV40022645	-1.09694	1.096938532	protein_coding	NM_001034172
ASHGV40057660	-1.09663	1.096626536	protein_coding	NM_005362
ASHGV40031558	-1.09613	1.096125765	protein_coding	NM_080628
ASHGV40026121	-1.0959	1.095898829	protein_coding	NM_021216
ASHGV40019662	1.09547	1.095469914	protein_coding	NM_006224
ASHGV40024796	-1.09528	1.095280781	protein_coding	ENST00000456337
ASHGV40025144	-1.09524	1.095243287	protein_coding	NM_001079935
ASHGV40045339	1.095182	1.095182271	protein_coding	NM_138409
ASHGV40002351	-1.09517	1.095172335	protein_coding	ENST00000569317
ASHGV40055938	1.095108	1.095107978	protein_coding	NM_002779
ASHGV40015052	1.094878	1.094878165	protein_coding	NM_207377
ASHGV40037784	1.094873	1.094872547	protein_coding	NM_005723
ASHGV40035732	1.094316	1.094316284	protein_coding	NM_182523
ASHGV40024745	-1.09416	1.09415665	protein_coding	NM_001031665
ASHGV40047164	1.093798	1.093798349	protein_coding	NM_020911
ASHGV40008344	-1.09378	1.09377587	protein_coding	NM_130783
ASHGV40029214	-1.09364	1.093639269	protein_coding	NM_144992
ASHGV40035891	-1.09329	1.093294577	protein_coding	NM_014240

ASHGV40006827	1.093266	1.093265982	protein_coding	NM_001142315
ASHGV40040956	-1.09285	1.092848786	protein_coding	NM_001080516
ASHGV40041879	-1.09282	1.092820923	protein_coding	NM_000639
ASHGV40029211	1.091935	1.091934731	protein_coding	NM_001862
ASHGV40029376	-1.09189	1.091891181	protein_coding	NM_001008740
ASHGV40060866	-1.0917	1.091699271	protein_coding	uc010jrm.1
ASHGV40023890	-1.09134	1.091336805	protein_coding	NM_030760
ASHGV40046772	-1.09134	1.091335085	protein_coding	NM_001161528
ASHGV40028469	-1.09077	1.090770344	protein_coding	NM_001198972
ASHGV40023998	-1.09057	1.090565832	protein_coding	ENST00000595472
ASHGV40008004	-1.09036	1.090364281	protein_coding	NM_152430
ASHGV40007002	-1.08996	1.089964542	protein_coding	NM_001004459
ASHGV40048559	-1.08992	1.089922886	protein_coding	NM_016943
ASHGV40040321	1.089862	1.089862327	protein_coding	NM_000702
ASHGV40044875	-1.08984	1.089837021	protein_coding	uc003nwy.1
ASHGV40054437	-1.08982	1.089819075	protein_coding	NM_031907
ASHGV40014661	1.089634	1.089633933	protein_coding	NM_017582
ASHGV40047290	-1.08963	1.089627235	protein_coding	NM_207336
ASHGV40026402	1.089618	1.089617796	protein_coding	NM_030797
ASHGV40034976	1.089103	1.089103374	protein_coding	NM_006219
ASHGV40042231	-1.08884	1.088842218	protein_coding	NM_005460
ASHGV40036849	1.088841	1.088840589	protein_coding	NM_006218
ASHGV40029876	1.088805	1.088805026	protein_coding	NM_018471
ASHGV40006111	1.088534	1.088534102	protein_coding	NM_033637
ASHGV40053806	-1.08852	1.088523927	protein_coding	NM_019857
ASHGV40043925	1.088408	1.088408097	protein_coding	NM_014028
ASHGV40033383	-1.08832	1.088315685	protein_coding	ENST00000441544
ASHGV40054587	1.088281	1.088281282	protein_coding	NM_006014
ASHGV40005131	-1.08788	1.087876752	protein_coding	ENST00000341866
ASHGV40051002	1.08769	1.087690392	protein_coding	NM_005034
ASHGV40009633	-1.08764	1.087636622	protein_coding	NM_031289
ASHGV40010653	-1.08739	1.087387654	protein_coding	NM_173598
ASHGV40010085	-1.08732	1.087315418	protein_coding	NM_000946
ASHGV40006965	-1.08729	1.087285743	protein_coding	ENST00000533152
ASHGV40040354	-1.0868	1.086803112	protein_coding	NM_144699
ASHGV40027893	1.086426	1.086426276	protein_coding	NM_012433
ASHGV40049770	-1.08636	1.086359236	protein_coding	NM_153015
ASHGV40048313	1.086257	1.086256615	protein_coding	NM_012328
ASHGV40012153	-1.08606	1.086056616	protein_coding	NM_024809
ASHGV40011331	-1.086	1.086003765	protein_coding	NM_001005203
ASHGV40022429	1.085747	1.085746714	protein_coding	NM_004746
ASHGV40050958	-1.08549	1.085486315	protein_coding	NM_017697
ASHGV40028956	-1.08516	1.085158692	protein_coding	ENST00000373137
ASHGV40019303	1.08509	1.085089679	protein_coding	NM_019023
ASHGV40055209	-1.08501	1.085014644	protein_coding	NM_021029
ASHGV40036734	1.084777	1.08477676	protein_coding	NM_016625
ASHGV40025873	-1.08472	1.084717339	protein_coding	NM_004070

ASHGV40006476	-1.08437	1.084370842	protein_coding	NM_021920
ASHGV40039027	1.084362	1.084361957	protein_coding	NM_001098484
ASHGV40047556	-1.08412	1.084120235	protein_coding	NM_014038
ASHGV40040013	-1.08394	1.083935252	protein_coding	uc001frp.2
ASHGV40056030	1.083672	1.083672009	protein_coding	NM_203451
ASHGV40025652	1.083586	1.083586289	protein_coding	NM_002812
ASHGV40044044	-1.08343	1.083425186	protein_coding	NM_006073
ASHGV40022106	-1.0832	1.083200537	protein_coding	ENST00000375684
ASHGV40055555	-1.08316	1.083162556	protein_coding	NM_000033
ASHGV40008469	-1.083	1.082998158	protein_coding	NM_015177
ASHGV40008153	-1.08284	1.082843272	protein_coding	NM_001277269
ASHGV40010415	1.082345	1.082344542	protein_coding	NM_018838
ASHGV40002558	1.082133	1.082132713	protein_coding	NM_175614
ASHGV40044385	1.081772	1.081771997	protein_coding	NM_145169
ASHGV40012927	1.081566	1.081565963	protein_coding	NM_018011
ASHGV40010595	1.081329	1.08132921	protein_coding	NM_002973
ASHGV40046834	1.081166	1.081165646	protein_coding	NM_003676
ASHGV40025389	1.081121	1.08112072	protein_coding	NM_198537
ASHGV40020341	1.080804	1.080803547	protein_coding	NM_003315
ASHGV40041790	1.080582	1.080581639	protein_coding	NM_138453
ASHGV40031707	-1.08048	1.080482485	protein_coding	NM_000562
ASHGV40052769	1.080409	1.080408672	protein_coding	NM_002771
ASHGV40015525	1.079929	1.079929425	protein_coding	NM_024071
ASHGV40027727	-1.07987	1.079868088	protein_coding	NM_152529
ASHGV40039434	1.079841	1.079840571	protein_coding	NM_001153484
ASHGV40017937	-1.07979	1.079785836	protein_coding	NM_024745
ASHGV40010590	-1.07966	1.079663863	protein_coding	NM_000432
ASHGV40031266	-1.07957	1.079566745	protein_coding	uc021waf.1
ASHGV40009967	-1.07938	1.079375688	protein_coding	NM_182507
ASHGV40034780	1.07925	1.079250038	protein_coding	NM_002338
ASHGV40047789	1.079163	1.079163449	protein_coding	NM_031443
ASHGV40008847	-1.07885	1.078854581	protein_coding	NM_153614
ASHGV40043919	-1.0787	1.078699671	protein_coding	NM_020381
ASHGV40021503	-1.07869	1.078687624	protein_coding	NM_004295
ASHGV40027937	1.078571	1.078571204	protein_coding	NM_003352
ASHGV40048555	1.07842	1.078420138	protein_coding	NM_001195278
ASHGV40023751	1.0781	1.078099971	protein_coding	NM_025241
ASHGV40051693	1.077493	1.07749327	protein_coding	NM_007126
ASHGV40015114	1.077476	1.077476028	protein_coding	NM_024540
ASHGV40007399	1.077235	1.077235356	protein_coding	NM_080491
ASHGV40007050	1.077209	1.077208897	protein_coding	NM_007051
ASHGV40000111	-1.07709	1.077085465	protein_coding	NM_021196
ASHGV40022243	-1.077	1.077001141	protein_coding	NM_003857
ASHGV40003079	-1.07663	1.076625972	protein_coding	NM_001014442
ASHGV40054755	-1.07626	1.076264722	protein_coding	NM_206923
ASHGV40023400	-1.07615	1.076151659	protein_coding	NM_173529
ASHGV40017116	-1.07604	1.07603707	protein_coding	NM_004378

ASHGV40043611	-1.07596	1.075964805	protein_coding	NM_003643
ASHGV40014684	-1.07593	1.075927885	protein_coding	NM_005698
ASHGV40042422	-1.07591	1.075909603	protein_coding	NM_018911
ASHGV40026647	1.075904	1.075903953	protein_coding	NM_021097
ASHGV40008269	1.075622	1.075621923	protein_coding	NM_002227
ASHGV40035892	1.075567	1.075567156	protein_coding	NM_014016
ASHGV40039527	-1.07549	1.075485284	protein_coding	NM_032117
ASHGV40024410	-1.07548	1.07547683	protein_coding	NM_005427
ASHGV40022348	1.075439	1.075439458	protein_coding	NM_001167740
ASHGV40026551	-1.07539	1.07539027	protein_coding	NM_004304
ASHGV40057712	-1.07525	1.075253686	protein_coding	ENST00000278064
ASHGV40038826	1.075017	1.075017138	protein_coding	NM_006345
ASHGV40054439	-1.075	1.074997461	protein_coding	NM_016521
ASHGV40028086	1.074852	1.074851784	protein_coding	NM_001007089
ASHGV40041254	1.074488	1.07448818	protein_coding	NM_173465
ASHGV40027616	-1.07422	1.074216724	protein_coding	NM_017940
ASHGV40025415	-1.07361	1.073613122	protein_coding	NM_025189
ASHGV40007418	1.073484	1.073483621	protein_coding	NM_175885
ASHGV40006970	-1.07327	1.073267432	protein_coding	NM_001004742
ASHGV40005928	-1.07316	1.073162832	protein_coding	NM_001956
ASHGV40010502	1.073088	1.073088077	protein_coding	NM_001031701
ASHGV40052548	1.073057	1.073057157	protein_coding	NM_001130969
ASHGV40025230	1.072614	1.072613644	protein_coding	NM_005053
ASHGV40014713	-1.07258	1.072577697	protein_coding	NM_001004715
ASHGV40008496	-1.07203	1.072027825	protein_coding	NM_001012417
ASHGV40013870	-1.07193	1.071931495	protein_coding	NM_000359
ASHGV40030786	-1.07173	1.071730329	protein_coding	uc002xci.1
ASHGV40038045	1.071631	1.071631018	protein_coding	NM_014331
ASHGV40044241	-1.07163	1.071625834	protein_coding	NM_139165
ASHGV40035331	1.071528	1.071527947	protein_coding	NM_020166
ASHGV40036135	1.071508	1.071508189	protein_coding	NM_174907
ASHGV40032901	1.071382	1.071381888	protein_coding	NM_002745
ASHGV40021376	1.071376	1.071376445	protein_coding	NM_201274
ASHGV40040188	1.071157	1.071157206	protein_coding	NM_006451
ASHGV40030884	-1.07102	1.071017709	protein_coding	NM_080869
ASHGV40005268	1.071001	1.0710007	protein_coding	NM_032905
ASHGV40007728	1.070908	1.070907886	protein_coding	NM_002855
ASHGV40007170	1.070889	1.070889035	protein_coding	NM_002419
ASHGV40054986	1.070391	1.07039056	protein_coding	NM_006986
ASHGV40050552	-1.07038	1.070383468	protein_coding	NM_194294
ASHGV40022052	-1.07031	1.07030594	protein_coding	NM_181707
ASHGV40007137	1.070183	1.070182961	protein_coding	NM_005609
ASHGV40010475	1.070163	1.070163362	protein_coding	NM_001177
ASHGV40003344	-1.07008	1.070075318	protein_coding	NM_174981
ASHGV40014599	1.069252	1.069251934	protein_coding	NM_006694
ASHGV40055302	1.069087	1.069086525	protein_coding	NM_005032
ASHGV40028644	-1.06908	1.069084802	protein_coding	uc002riw.3

ASHGV40021263	-1.06896	1.068957683	protein_coding	NM_153007
ASHGV40054947	-1.06876	1.068760647	protein_coding	NM_000377
ASHGV40028357	-1.06851	1.068512163	protein_coding	NM_001099434
ASHGV40000454	1.068136	1.06813608	protein_coding	NM_001271641
ASHGV40033793	1.068024	1.06802383	protein_coding	NM_033318
ASHGV40025806	1.067799	1.067799374	protein_coding	NM_001199867
ASHGV40011357	1.067702	1.067701802	protein_coding	NM_003217
ASHGV40026602	-1.06755	1.067552433	protein_coding	ENST00000406220
ASHGV40032157	-1.06752	1.067522127	protein_coding	NM_181608
ASHGV40019262	1.067346	1.067345996	protein_coding	NM_173815
ASHGV40036898	-1.06717	1.067165791	protein_coding	NM_130770
ASHGV40055575	1.066953	1.066953322	protein_coding	NM_001493
ASHGV40005602	-1.06669	1.066691526	protein_coding	NM_001278923
ASHGV40054600	1.06654	1.066540192	protein_coding	NM_171998
ASHGV40033486	1.0663	1.066299991	protein_coding	NM_002073
ASHGV40004977	1.066089	1.066089033	protein_coding	NM_014904
ASHGV40048541	1.066075	1.066075273	protein_coding	NM_197964
ASHGV40032450	1.065675	1.065675444	protein_coding	NM_013396
ASHGV40034402	-1.06515	1.065154511	protein_coding	NM_015426
ASHGV40042772	1.064998	1.064997719	protein_coding	NM_001265615
ASHGV40056100	-1.06497	1.064973405	protein_coding	NM_001013632
ASHGV40016332	1.064888	1.064888485	protein_coding	NM_015415
ASHGV40040819	1.064592	1.064592311	protein_coding	NM_003374
ASHGV40024784	1.064512	1.06451173	protein_coding	NM_016831
ASHGV40014422	1.064359	1.064358612	protein_coding	NM_000153
ASHGV40008357	1.064068	1.06406755	protein_coding	ENST00000378779
ASHGV40050637	1.064057	1.064057167	protein_coding	NM_018967
ASHGV40050411	1.063924	1.063923519	protein_coding	NM_002717
ASHGV40025019	-1.06381	1.063805191	protein_coding	NM_133261
ASHGV40020076	-1.06377	1.063767096	protein_coding	NM_001174103
ASHGV40009693	1.063566	1.063566155	protein_coding	NM_014802
ASHGV40056371	-1.06313	1.063131741	protein_coding	uc002hll.1
ASHGV40032839	-1.06301	1.063013165	protein_coding	NM_007098
ASHGV40009825	1.062809	1.062809101	protein_coding	NM_153634
ASHGV40023521	1.062765	1.062764943	protein_coding	NM_152721
ASHGV40022644	1.062561	1.062561015	protein_coding	NM_001004692
ASHGV40035959	1.062558	1.062557734	protein_coding	NM_152900
ASHGV40033175	1.062508	1.062507842	protein_coding	NM_007034
ASHGV40013923	1.062438	1.062438141	protein_coding	uc001wrb.1
ASHGV40015790	1.062242	1.062242362	protein_coding	NM_005657
ASHGV40053080	1.062083	1.062083211	protein_coding	NM_006717
ASHGV40045056	-1.06202	1.062015922	protein_coding	NM_015349
ASHGV40035076	1.061997	1.061996816	protein_coding	NM_053024
ASHGV40024224	1.061929	1.061929477	protein_coding	NM_032139
ASHGV40029742	-1.06179	1.061793696	protein_coding	NM_021176
ASHGV40053068	1.061715	1.061714988	protein_coding	NM_001912
ASHGV40030698	1.061584	1.061584408	protein_coding	NM_032501

ASHGV40052195	1.061395	1.061394604	protein_coding	NM_015258
ASHGV40053031	1.061229	1.061229355	protein_coding	NM_001001551
ASHGV40020394	-1.06113	1.061134034	protein_coding	NM_001932
ASHGV40033206	1.061049	1.061048751	protein_coding	NM_002676
ASHGV40033927	1.060786	1.060786498	protein_coding	NM_012324
ASHGV40036434	1.060689	1.060689019	protein_coding	NM_006889
ASHGV40040567	1.060674	1.06067416	protein_coding	NM_152366
ASHGV40057827	-1.06053	1.060531457	protein_coding	NM_001002905
ASHGV40052621	-1.06034	1.06034243	protein_coding	uc001btr.1
ASHGV40022722	1.060341	1.060341099	protein_coding	NM_020783
ASHGV40055974	-1.06021	1.060210372	protein_coding	NM_007350
ASHGV40039819	1.059927	1.059926516	protein_coding	NM_024786
ASHGV40018895	-1.05988	1.059882621	protein_coding	NM_152338
ASHGV40002383	-1.05921	1.059207078	protein_coding	NM_000080
ASHGV40056544	-1.0592	1.059201836	protein_coding	uc021utt.1
ASHGV40006987	-1.05902	1.059024583	protein_coding	NM_006093
ASHGV40048271	-1.05899	1.058992741	protein_coding	ENST00000436228
ASHGV40034533	1.058832	1.058832432	protein_coding	NM_003968
ASHGV40028603	1.058471	1.058470955	protein_coding	NM_004116
ASHGV40056430	1.058405	1.058404842	protein_coding	NM_001270945
ASHGV40054921	-1.05826	1.058257028	protein_coding	NM_003446
ASHGV40018506	1.057864	1.057863597	protein_coding	NM_023933
ASHGV40013322	1.057772	1.057771731	protein_coding	NM_001040443
ASHGV40056765	-1.05777	1.057770721	protein_coding	NM_013248
ASHGV40034141	1.057478	1.057477582	protein_coding	NM_017784
ASHGV40011525	-1.05735	1.057354223	protein_coding	NM_173596
ASHGV40010965	-1.05725	1.057254075	protein_coding	NM_001242
ASHGV40024806	-1.05712	1.057120203	protein_coding	NM_000363
ASHGV40049693	1.056872	1.056872399	protein_coding	NM_183419
ASHGV40031508	-1.05662	1.056615982	protein_coding	NM_178466
ASHGV40025712	-1.05649	1.056488347	protein_coding	NM_053046
ASHGV40009223	-1.05646	1.056458068	protein_coding	NM_181721
ASHGV40004928	1.056126	1.056125812	protein_coding	NM_024874
ASHGV40039254	-1.05607	1.056072637	protein_coding	uc021pao.2
ASHGV40010089	-1.05592	1.055916042	protein_coding	NM_003708
ASHGV40026422	1.055834	1.055834155	protein_coding	NM_001008237
ASHGV40014437	-1.05564	1.055636647	protein_coding	ENST00000550332
ASHGV40055925	1.055373	1.055373388	protein_coding	NM_022039
ASHGV40013146	1.055252	1.055251639	protein_coding	NM_015932
ASHGV40052468	1.055237	1.055236514	protein_coding	ENST00000593613
ASHGV40054117	-1.05517	1.05517414	protein_coding	NM_032803
ASHGV40036104	1.054654	1.054654028	protein_coding	NM_000248
ASHGV40054793	1.054506	1.054506188	protein_coding	NM_021129
ASHGV40017703	-1.0545	1.054499547	protein_coding	NM_001002911
ASHGV40015169	1.054373	1.054372867	protein_coding	NM_000021
ASHGV40013128	-1.05436	1.054362441	protein_coding	NM_206827
ASHGV40047430	-1.05432	1.054324819	protein_coding	NM_001098623

ASHGV40033067	1.054309	1.054308991	protein_coding	NM_014306
ASHGV40021627	-1.05425	1.054254375	protein_coding	NM_001161533
ASHGV40024696	-1.05403	1.054034597	protein_coding	NM_152353
ASHGV40019802	-1.05394	1.053937532	protein_coding	NM_000546
ASHGV40027059	1.053849	1.053848647	protein_coding	NM_020448
ASHGV40037372	1.053825	1.053825143	protein_coding	NM_004284
ASHGV40043186	-1.05327	1.053274825	protein_coding	NM_031935
ASHGV40046744	1.052882	1.052881655	protein_coding	NM_001142749
ASHGV40010584	1.052726	1.052726437	protein_coding	NM_016226
ASHGV40054991	-1.05267	1.052668855	protein_coding	NM_018969
ASHGV40048862	1.052048	1.052047853	protein_coding	ENST00000330777
ASHGV40024227	-1.05189	1.051885278	protein_coding	NM_014270
ASHGV40045063	1.051775	1.051774942	protein_coding	NM_057161
ASHGV40022193	-1.05169	1.051693913	protein_coding	NM_152460
ASHGV40002700	-1.05105	1.051052202	protein_coding	NM_005919
ASHGV40018784	-1.05102	1.051019602	protein_coding	NM_005622
ASHGV40001269	-1.05091	1.050905083	protein_coding	NM_000982
ASHGV40042315	-1.05068	1.05068107	protein_coding	NM_052971
ASHGV40020531	1.050305	1.050304715	protein_coding	NM_001278784
ASHGV40030338	-1.04993	1.049931033	protein_coding	NM_024101
ASHGV40027389	1.049879	1.049878881	protein_coding	NM_017969
ASHGV40007698	-1.04972	1.049723767	protein_coding	NM_001098526
ASHGV40043383	1.049145	1.049145066	protein_coding	NM_024294
ASHGV40034835	1.049099	1.049098569	protein_coding	NM_020978
ASHGV40031447	-1.04904	1.049037033	protein_coding	NM_021067
ASHGV40009413	-1.04903	1.049031145	protein_coding	ENST00000597621
ASHGV40042287	1.048935	1.048934965	protein_coding	NM_181705
ASHGV40028074	1.04891	1.048910296	protein_coding	NM_194302
ASHGV40026565	-1.04885	1.048846622	protein_coding	NM_000379
ASHGV40041033	-1.04882	1.048823912	protein_coding	NM_020167
ASHGV40007135	1.048767	1.048766503	protein_coding	NM_015080
ASHGV40030435	-1.04864	1.048640748	protein_coding	NM_033409
ASHGV40011775	1.048555	1.04855509	protein_coding	NM_001009894
ASHGV40024743	-1.04835	1.048349349	protein_coding	NM_207333
ASHGV40002530	-1.04818	1.048176325	protein_coding	ENST00000588212
ASHGV40045843	-1.04807	1.0480716	protein_coding	NM_000125
ASHGV40035232	1.047952	1.047951829	protein_coding	NM_006513
ASHGV40013593	1.047708	1.047707644	protein_coding	NM_021033
ASHGV40037945	1.047499	1.047498586	protein_coding	NM_152399
ASHGV40034717	-1.04737	1.047369318	protein_coding	NM_018010
ASHGV40035775	1.047358	1.047358272	protein_coding	NM_002884
ASHGV40034553	1.047332	1.047332152	protein_coding	NM_012234
ASHGV40021486	1.047266	1.047265903	protein_coding	NM_021137
ASHGV40054534	1.047033	1.04703276	protein_coding	NM_001013845
ASHGV40042502	1.046908	1.046907735	protein_coding	NM_020768
ASHGV40036451	-1.04691	1.04690723	protein_coding	NM_006810
ASHGV40035236	1.04684	1.046840285	protein_coding	NM_020390

ASHGV40006524	-1.04664	1.046642712	protein_coding	NM_003311
ASHGV40041972	1.046423	1.046422919	protein_coding	NM_001284237
ASHGV40029183	1.046374	1.046373606	protein_coding	NM_013434
ASHGV40057200	1.046363	1.046362961	protein_coding	NM_001005216
ASHGV40048991	1.046284	1.046283692	protein_coding	NM_139278
ASHGV40035734	-1.04611	1.04610696	protein_coding	NM_181643
ASHGV40055946	1.046104	1.0461039	protein_coding	NM_004311
ASHGV40024977	-1.0461	1.046100016	protein_coding	NM_079834
ASHGV40020888	-1.04608	1.046075746	protein_coding	NM_001454
ASHGV40040768	-1.04591	1.045911177	protein_coding	NM_001164479
ASHGV40035090	-1.04574	1.04573768	protein_coding	ENST00000397891
ASHGV40007666	1.045692	1.045691822	protein_coding	NM_014333
ASHGV40010705	1.045572	1.045572397	protein_coding	NM_016237
ASHGV40045840	1.045277	1.045276514	protein_coding	NM_005100
ASHGV40014554	-1.04514	1.045142954	protein_coding	NM_138576
ASHGV40044970	-1.04513	1.045132127	protein_coding	NM_015695
ASHGV40047453	-1.04505	1.045048306	protein_coding	NM_175055
ASHGV40002539	1.045002	1.04500246	protein_coding	ENST00000589057
ASHGV40056075	-1.04486	1.044858433	protein_coding	NM_001197287
ASHGV40040918	1.0448	1.04479986	protein_coding	NM_005642
ASHGV40046384	1.0448	1.044799691	protein_coding	NM_001635
ASHGV40021322	1.044796	1.044796122	protein_coding	NM_020808
ASHGV40054746	1.04459	1.044590489	protein_coding	NM_000284
ASHGV40008097	1.044347	1.044347331	protein_coding	NM_001025389
ASHGV40039079	1.044016	1.044015842	protein_coding	NM_003715
ASHGV40027832	1.043852	1.043851807	protein_coding	NM_018053
ASHGV40016955	-1.04374	1.043743662	protein_coding	NM_017975
ASHGV40022878	-1.04367	1.043673006	protein_coding	NM_002974
ASHGV40036566	-1.04345	1.043448539	protein_coding	NM_144717
ASHGV40054085	-1.04327	1.043270331	protein_coding	NM_017677
ASHGV40050580	1.04315	1.04315035	protein_coding	NM_006803
ASHGV40043462	1.043065	1.043065106	protein_coding	NM_173561
ASHGV40031338	1.042917	1.042916888	protein_coding	NM_024120
ASHGV40024028	1.042389	1.042388552	protein_coding	NM_021187
ASHGV40047953	1.041778	1.041777854	protein_coding	NM_173517
ASHGV40029505	1.041599	1.041598794	protein_coding	NM_033416
ASHGV40025755	-1.04099	1.040986214	protein_coding	NM_001130011
ASHGV40027764	1.04094	1.0409396	protein_coding	NM_003690
ASHGV40006523	1.040927	1.040926696	protein_coding	NM_007105
ASHGV40024509	-1.04089	1.040887046	protein_coding	ENST00000591646
ASHGV40001383	1.040813	1.040812729	protein_coding	NM_016079
ASHGV40033534	-1.04074	1.0407416	protein_coding	NM_004076
ASHGV40047901	1.040709	1.040708967	protein_coding	NM_001483
ASHGV40053603	-1.04071	1.040708437	protein_coding	NM_001164484
ASHGV40034156	1.0407	1.040699885	protein_coding	NM_015097
ASHGV40003194	-1.04066	1.04065727	protein_coding	NM_001256141
ASHGV40050781	1.040644	1.040644456	protein_coding	NM_024870

ASHGV40014042	1.040575	1.040575454	protein_coding	NM_024558
ASHGV40022000	1.04008	1.040079506	protein_coding	NM_021626
ASHGV40042417	-1.03989	1.039886377	protein_coding	NM_018906
ASHGV40033912	-1.03987	1.039872909	protein_coding	NM_025204
ASHGV40053817	-1.03983	1.039833983	protein_coding	NM_006089
ASHGV40005154	1.039639	1.0396385	protein_coding	NM_031280
ASHGV40051558	1.039561	1.039560687	protein_coding	NM_001010
ASHGV40037485	1.039429	1.039429241	protein_coding	NM_001014446
ASHGV40008006	-1.03908	1.039083906	protein_coding	NM_001004759
ASHGV40000184	-1.03903	1.039030022	protein_coding	ENST00000381090
ASHGV40055663	-1.03838	1.038380082	protein_coding	NM_170723
ASHGV40034448	1.038357	1.038357469	protein_coding	NM_015576
ASHGV40049009	-1.03833	1.038333597	protein_coding	NM_003842
ASHGV40029305	-1.03832	1.038318569	protein_coding	NM_006588
ASHGV40038985	-1.03804	1.038040153	protein_coding	NM_080429
ASHGV40029476	-1.03797	1.037966534	protein_coding	NM_001080527
ASHGV40027425	1.03796	1.0379597	protein_coding	NM_032545
ASHGV40006440	-1.03794	1.037935026	protein_coding	NM_014468
ASHGV40008186	1.037742	1.037741632	protein_coding	NM_006157
ASHGV40003068	-1.03756	1.037556465	protein_coding	NM_001005513
ASHGV40026517	-1.03752	1.037518738	protein_coding	NM_177983
ASHGV40009895	-1.03666	1.036658035	protein_coding	NM_000376
ASHGV40047097	-1.03657	1.036566464	protein_coding	NM_031944
ASHGV40027440	-1.03656	1.036564028	protein_coding	NM_001803
ASHGV40015132	-1.03655	1.036549332	protein_coding	NM_133509
ASHGV40010911	1.03634	1.036340111	protein_coding	NM_018463
ASHGV40028202	1.036286	1.036286032	protein_coding	NM_002601
ASHGV40010367	1.036275	1.036274921	protein_coding	NM_001946
ASHGV40016930	1.036205	1.036204776	protein_coding	NM_003099
ASHGV40050522	-1.0361	1.036099382	protein_coding	NM_004095
ASHGV40021764	1.035757	1.035757252	protein_coding	NM_000988
ASHGV40043446	-1.03571	1.035713076	protein_coding	NM_031460
ASHGV40021822	1.035654	1.035654116	protein_coding	NM_006460
ASHGV40038805	-1.03561	1.035606805	protein_coding	NM_018177
ASHGV40026763	1.035436	1.035435711	protein_coding	NM_002453
ASHGV40046240	1.034782	1.034781884	protein_coding	NM_207342
ASHGV40007221	-1.03464	1.034643825	protein_coding	NM_013246
ASHGV40045115	1.034633	1.034633114	protein_coding	NM_001010870
ASHGV40027098	1.034586	1.034586185	protein_coding	NM_020151
ASHGV40008500	-1.03444	1.034437482	protein_coding	NM_001098835
ASHGV40044727	-1.03419	1.034194346	protein_coding	NM_003529
ASHGV40045283	1.034178	1.034178314	protein_coding	NM_015571
ASHGV40054848	1.033862	1.033861654	protein_coding	NM_005765
ASHGV40034017	1.033652	1.033651558	protein_coding	NM_014869
ASHGV40057235	-1.03327	1.033269116	protein_coding	NM_001126063
ASHGV40054170	-1.03302	1.03301587	protein_coding	NM_152694
ASHGV40007968	1.032768	1.032767758	protein_coding	NM_004356

ASHGV40042425	-1.03266	1.032663922	protein_coding	NM_018902
ASHGV40040422	1.032597	1.032596714	protein_coding	NM_003664
ASHGV40027220	1.032591	1.032590745	protein_coding	NM_018202
ASHGV40046514	-1.03258	1.032575743	protein_coding	NM_000790
ASHGV40034673	1.0324	1.032400208	protein_coding	NM_000986
ASHGV40021247	1.032243	1.032242805	protein_coding	NM_175876
ASHGV40020546	1.032124	1.032123715	protein_coding	NM_014698
ASHGV40056574	-1.03209	1.032091483	protein_coding	NM_002030
ASHGV40054366	-1.03187	1.031870968	protein_coding	NM_001099685
ASHGV40050894	-1.03184	1.031836634	protein_coding	NM_001951
ASHGV40008863	1.031809	1.031809264	protein_coding	NM_001195528
ASHGV40050572	1.031761	1.031761453	protein_coding	NM_016099
ASHGV40017290	1.031612	1.031612329	protein_coding	NM_013272
ASHGV40006776	-1.03112	1.031120302	protein_coding	NM_178498
ASHGV40038369	1.030805	1.030804537	protein_coding	NM_002810
ASHGV40021751	-1.03076	1.030764404	protein_coding	NM_002508
ASHGV40050139	-1.03074	1.030741174	protein_coding	ENST00000428558
ASHGV40024301	-1.03057	1.030568788	protein_coding	NM_152477
ASHGV40006825	1.030242	1.030242328	protein_coding	NM_012175
ASHGV40017042	-1.03022	1.030223381	protein_coding	NM_153356
ASHGV40054312	-1.03014	1.030138982	protein_coding	NM_001522
ASHGV40016114	-1.02975	1.029752601	protein_coding	NM_005477
ASHGV40011983	1.029525	1.029524638	protein_coding	NM_170665
ASHGV40018099	-1.02948	1.029483237	protein_coding	NM_133262
ASHGV40026054	-1.02947	1.029469446	protein_coding	ENST00000291860
ASHGV40007890	-1.02938	1.029377411	protein_coding	NM_025092
ASHGV40056141	-1.02918	1.029179134	protein_coding	NM_017955
ASHGV40031855	-1.0288	1.028802686	protein_coding	NM_024663
ASHGV40005288	-1.02858	1.028581341	protein_coding	NM_002216
ASHGV40011381	-1.02843	1.028433278	protein_coding	NM_001109619
ASHGV40005606	-1.02838	1.028378092	protein_coding	NM_014571
ASHGV40010978	1.028155	1.028154695	protein_coding	NM_016319
ASHGV40044876	-1.02804	1.028042129	protein_coding	NM_001039651
ASHGV40008169	-1.02775	1.027747824	protein_coding	NM_153347
ASHGV40030434	-1.02773	1.027726043	protein_coding	NM_033129
ASHGV40010487	1.027621	1.027621343	protein_coding	NM_016053
ASHGV40054708	1.027325	1.02732548	protein_coding	NM_004251
ASHGV40010033	1.027135	1.027134937	protein_coding	NM_002205
ASHGV40025868	-1.02706	1.027060759	protein_coding	NM_031485
ASHGV40055142	-1.02701	1.027007428	protein_coding	ENST00000373200
ASHGV40045250	-1.02682	1.026823322	protein_coding	NM_152365
ASHGV40031152	-1.02678	1.026778148	protein_coding	ENST00000370346
ASHGV40046958	1.026558	1.026558443	protein_coding	NM_015723
ASHGV40025032	1.026486	1.026486214	protein_coding	NM_133475
ASHGV40035803	-1.02544	1.025435941	protein_coding	NM_004256
ASHGV40033533	1.025374	1.025374227	protein_coding	NM_001145206
ASHGV40034967	-1.02521	1.025205052	protein_coding	NM_173543

ASHGV40016745	-1.02518	1.025183545	protein_coding	NM_003104
ASHGV40008980	-1.02516	1.025163878	protein_coding	NM_001195234
ASHGV40028578	-1.02479	1.024786425	protein_coding	NM_182828
ASHGV40056185	-1.02455	1.024548971	protein_coding	NM_001018100
ASHGV40017151	1.024393	1.024392533	protein_coding	NM_022566
ASHGV40007402	-1.02435	1.024348552	protein_coding	NM_001098816
ASHGV40027781	1.024249	1.024248884	protein_coding	NM_177424
ASHGV40026510	1.024148	1.024148362	protein_coding	NM_003459
ASHGV40027778	1.024056	1.02405574	protein_coding	NM_020943
ASHGV40037145	1.024008	1.02400847	protein_coding	NM_006454
ASHGV40030682	-1.02381	1.023806386	protein_coding	NM_178311
ASHGV40018464	-1.02377	1.0237747	protein_coding	NM_002832
ASHGV40006496	-1.02376	1.023762022	protein_coding	NM_001005922
ASHGV40030457	1.023661	1.023660714	protein_coding	NM_006899
ASHGV40038546	1.023546	1.023545698	protein_coding	NM_014743
ASHGV40027987	-1.02327	1.023270676	protein_coding	NM_003468
ASHGV40057349	-1.02304	1.023035585	protein_coding	NM_152706
ASHGV40025065	-1.02271	1.022706649	protein_coding	NM_152784
ASHGV40015365	-1.02263	1.022627889	protein_coding	NM_006215
ASHGV40026117	-1.02253	1.022529852	protein_coding	NM_020813
ASHGV40024846	-1.02231	1.022305476	protein_coding	NM_144690
ASHGV40048038	-1.02226	1.022259217	protein_coding	NM_182683
ASHGV40018479	-1.02217	1.022174908	protein_coding	NM_032039
ASHGV40030081	-1.02216	1.022163334	protein_coding	NM_024532
ASHGV40026490	-1.02209	1.022088959	protein_coding	NM_001145168
ASHGV40014932	1.02208	1.02207967	protein_coding	NM_152447
ASHGV40029106	-1.02206	1.022058191	protein_coding	NM_031283
ASHGV40048179	1.022015	1.02201526	protein_coding	NM_003182
ASHGV40020331	1.021979	1.021979114	protein_coding	NM_005426
ASHGV40033353	-1.02195	1.021951818	protein_coding	NM_001953
ASHGV40000217	1.021503	1.021502837	protein_coding	NM_000971
ASHGV40023799	1.021265	1.021264787	protein_coding	NM_001080452
ASHGV40037947	-1.02115	1.021154724	protein_coding	NM_001237
ASHGV40015291	1.02115	1.021150047	protein_coding	NM_144596
ASHGV40009733	-1.02089	1.020889525	protein_coding	NM_001242672
ASHGV40035819	-1.02089	1.020888486	protein_coding	NM_002295
ASHGV40025807	-1.02064	1.020644421	protein_coding	NM_017556
ASHGV40048475	-1.02057	1.020569953	protein_coding	NM_016352
ASHGV40033166	1.020539	1.020539327	protein_coding	NM_015374
ASHGV40028654	1.019867	1.019866762	protein_coding	NM_014748
ASHGV40006081	1.019863	1.019862537	protein_coding	NM_005063
ASHGV40041856	-1.01983	1.019827156	protein_coding	NM_022909
ASHGV40002686	-1.01973	1.019728825	protein_coding	ENST00000600484
ASHGV40047508	1.019702	1.019701718	protein_coding	NM_020156
ASHGV40007183	1.0197	1.019700096	protein_coding	NM_198897
ASHGV40040478	1.018992	1.018992362	protein_coding	NM_174909
ASHGV40007452	-1.01899	1.018988662	protein_coding	NM_001300995

ASHGV40027067	-1.01895	1.01895413	protein_coding	ENST00000443397
ASHGV40044832	-1.01885	1.018852876	protein_coding	NM_024839
ASHGV40034344	1.018345	1.018345196	protein_coding	NM_003363
ASHGV40005827	1.018088	1.018087805	protein_coding	NM_133467
ASHGV40000900	1.017921	1.017920841	protein_coding	ENST00000440215
ASHGV40048473	-1.0175	1.017496056	protein_coding	NM_145268
ASHGV40033138	-1.01732	1.017323549	protein_coding	NM_006498
ASHGV40043163	1.016935	1.016935187	protein_coding	NM_007149
ASHGV40043012	1.01669	1.016689674	protein_coding	NM_030806
ASHGV40049978	1.01667	1.016670365	protein_coding	NM_006096
ASHGV40045643	1.016565	1.016564831	protein_coding	NM_001031712
ASHGV40006288	1.016556	1.016555831	protein_coding	NM_014937
ASHGV40046840	-1.01639	1.016385265	protein_coding	ENST00000336374
ASHGV40034145	1.016329	1.016329135	protein_coding	NM_016141
ASHGV40048273	-1.01629	1.016288162	protein_coding	NM_001031692
ASHGV40032190	1.015999	1.015998718	protein_coding	NM_001160302
ASHGV40005060	-1.01568	1.015678727	protein_coding	NM_001128202
ASHGV40033107	1.015554	1.015554472	protein_coding	NM_030882
ASHGV40009923	-1.01541	1.015413024	protein_coding	NM_018113
ASHGV40009123	1.014996	1.014995797	protein_coding	NM_015191
ASHGV40027683	1.014896	1.01489561	protein_coding	NM_014168
ASHGV40000215	-1.0148	1.014804502	protein_coding	NM_001042450
ASHGV40057337	-1.01474	1.014735973	protein_coding	ENST00000437796
ASHGV40036940	-1.01455	1.014547209	protein_coding	NM_001102416
ASHGV40032953	-1.01435	1.014348223	protein_coding	NM_001001663
ASHGV40045781	-1.01431	1.014310973	protein_coding	NM_001013623
ASHGV40025035	1.014163	1.01416326	protein_coding	NM_020209
ASHGV40009845	1.013742	1.013742345	protein_coding	NM_033114
ASHGV40015446	-1.01372	1.013719889	protein_coding	NM_003836
ASHGV40025314	-1.01298	1.012977238	protein_coding	NM_003950
ASHGV40053627	-1.01286	1.012855845	protein_coding	NM_001001676
ASHGV40057279	-1.01284	1.012843646	protein_coding	NM_001276687
ASHGV40053653	-1.01268	1.012679274	protein_coding	NM_001039374
ASHGV40016339	-1.0125	1.01249905	protein_coding	NM_153832
ASHGV40029898	1.012315	1.012315079	protein_coding	NM_000534
ASHGV40054463	-1.01228	1.012278663	protein_coding	NM_024597
ASHGV40029407	-1.01202	1.012024859	protein_coding	NM_183240
ASHGV40014742	-1.012	1.012000255	protein_coding	NM_001146683
ASHGV40007573	1.011945	1.01194534	protein_coding	NM_000855
ASHGV40015152	-1.01158	1.011584988	protein_coding	NM_003813
ASHGV40045079	-1.01153	1.011529514	protein_coding	NM_006502
ASHGV40018156	1.011431	1.011431351	protein_coding	NM_004691
ASHGV40055116	-1.01111	1.011112372	protein_coding	NM_005296
ASHGV40009991	-1.01111	1.011110309	protein_coding	NM_000423
ASHGV40015506	-1.01107	1.011067862	protein_coding	NM_001013661
ASHGV40029454	1.010942	1.010941975	protein_coding	NM_130773
ASHGV40020507	-1.01051	1.010511581	protein_coding	NM_024017

ASHGV40005137	-1.01042	1.010420655	protein_coding	ENST00000540159
ASHGV40054460	-1.01036	1.010360255	protein_coding	uc011mvu.2
ASHGV40008402	-1.00995	1.009947224	protein_coding	NM_001206626
ASHGV40012762	1.009893	1.0098934	protein_coding	NM_005842
ASHGV40032172	1.00958	1.009580487	protein_coding	NM_003253
ASHGV40040988	-1.00948	1.009481094	protein_coding	NM_014443
ASHGV40036264	-1.00914	1.009139936	protein_coding	NM_001004737
ASHGV40023131	1.009062	1.009062445	protein_coding	NM_194434
ASHGV40013051	1.008965	1.008965195	protein_coding	NM_003453
ASHGV40008748	-1.00895	1.008947636	protein_coding	NM_002180
ASHGV40017854	-1.00892	1.008921206	protein_coding	NM_033410
ASHGV40001170	-1.0087	1.008704472	protein_coding	ENST00000454512
ASHGV40032256	1.008702	1.008701506	protein_coding	NM_032437
ASHGV40009860	1.008701	1.008701348	protein_coding	NM_001004329
ASHGV40017554	-1.00867	1.008673142	protein_coding	NM_001098814
ASHGV40037801	-1.00856	1.008561625	protein_coding	ENST00000511828
ASHGV40016610	1.008304	1.008303745	protein_coding	NM_016454
ASHGV40010050	1.008286	1.008286274	protein_coding	NM_001780
ASHGV40014719	1.00825	1.008249755	protein_coding	NM_005484
ASHGV40046813	-1.00801	1.008005697	protein_coding	NM_005221
ASHGV40056690	1.007764	1.007764418	protein_coding	NM_004807
ASHGV40020849	-1.00776	1.007762531	protein_coding	NM_016185
ASHGV40028635	-1.00768	1.007681774	protein_coding	NM_001809
ASHGV40014484	1.007303	1.00730332	protein_coding	NM_000295
ASHGV40032000	1.007278	1.00727829	protein_coding	NM_022136
ASHGV40054485	-1.00726	1.007261492	protein_coding	NM_001013403
ASHGV40017091	1.007178	1.007178047	protein_coding	NM_002902
ASHGV40000105	-1.00707	1.007068969	protein_coding	ENST00000354719
ASHGV40008094	-1.00697	1.006970262	protein_coding	NM_001124
ASHGV40036908	1.006943	1.006943324	protein_coding	NM_002808
ASHGV40005003	-1.00682	1.006819528	protein_coding	NM_022111
ASHGV40020188	1.006795	1.006794992	protein_coding	NM_001129820
ASHGV40028609	1.006784	1.006784434	protein_coding	NM_003743
ASHGV40040812	1.006748	1.006747763	protein_coding	NM_014423
ASHGV40055513	-1.00671	1.006712251	protein_coding	NM_000252
ASHGV40015549	1.006662	1.00666218	protein_coding	NM_015005
ASHGV40041959	-1.00633	1.006329022	protein_coding	NM_001713
ASHGV40005472	1.00631	1.006309839	protein_coding	NM_022157
ASHGV40057753	-1.00618	1.006180086	protein_coding	ENST00000428556
ASHGV40008608	1.006055	1.006054793	protein_coding	NM_017768
ASHGV40024281	-1.00602	1.006020909	protein_coding	NM_001166034
ASHGV40057172	1.00596	1.005960095	protein_coding	NM_012135
ASHGV40031006	-1.00591	1.005911937	protein_coding	NM_020436
ASHGV40031532	1.00576	1.005759536	protein_coding	NM_020884
ASHGV40025722	1.005586	1.005586297	protein_coding	NM_007040
ASHGV40043447	-1.00552	1.005522722	protein_coding	NM_032115
ASHGV40050920	1.005414	1.00541435	protein_coding	NM_001359

ASHGV40000192	-1.0054	1.005402512	protein_coding	NM_001143980
ASHGV40044883	1.005379	1.005379334	protein_coding	NM_005076
ASHGV40038240	1.005328	1.005328487	protein_coding	NM_017631
ASHGV40030271	1.005155	1.005154651	protein_coding	NM_206895
ASHGV40007052	-1.0051	1.005098512	protein_coding	NM_153611
ASHGV40028793	1.004876	1.004876032	protein_coding	NM_016008
ASHGV40026664	-1.00452	1.004517823	protein_coding	NM_012205
ASHGV40056480	-1.00438	1.004382734	protein_coding	NM_001100910
ASHGV40024634	-1.00426	1.004263436	protein_coding	NM_014037
ASHGV40048300	1.004141	1.004141407	protein_coding	NM_002736
ASHGV40041960	1.004094	1.004094111	protein_coding	NM_152405
ASHGV40019857	1.003866	1.003866418	protein_coding	NM_201433
ASHGV40057107	1.003785	1.00378464	protein_coding	NM_021967
ASHGV40025927	-1.00375	1.003750193	protein_coding	NM_012068
ASHGV40057028	-1.00346	1.003455776	protein_coding	NM_032693
ASHGV40026617	-1.00341	1.003406732	protein_coding	NM_006449
ASHGV40025299	-1.00325	1.003253074	protein_coding	NM_032855
ASHGV40032128	1.003089	1.003089086	protein_coding	NM_016940
ASHGV40021643	1.003052	1.003052399	protein_coding	NM_016374
ASHGV40000145	-1.0029	1.002904383	protein_coding	ENST00000372492
ASHGV40031024	-1.0026	1.002603018	protein_coding	NM_006526
ASHGV40014041	-1.00243	1.00243334	protein_coding	ENST00000305273
ASHGV40011181	1.002419	1.002418559	protein_coding	NM_015000
ASHGV40041855	-1.00217	1.002174973	protein_coding	NM_031966
ASHGV40017730	1.002021	1.002021299	protein_coding	NM_001802
ASHGV40023550	1.00201	1.002009923	protein_coding	NM_018235
ASHGV40046748	1.00186	1.001860476	protein_coding	NM_018849
ASHGV40046572	-1.00175	1.001749468	protein_coding	uc010kzn.3
ASHGV40018172	1.001685	1.001685107	protein_coding	NM_012482
ASHGV40013253	1.001657	1.00165699	protein_coding	NM_016248
ASHGV40002678	-1.00155	1.001553798	protein_coding	ENST00000599632
ASHGV40018499	-1.0015	1.001504104	protein_coding	NM_145294
ASHGV40055766	-1.0014	1.001398575	protein_coding	NM_001017924
ASHGV40026368	1.000636	1.000636465	protein_coding	NM_005380
ASHGV40018537	1.00058	1.000580435	protein_coding	NM_021633
ASHGV40053227	1.000433	1.000433209	protein_coding	NM_003452
ASHGV40034649	1.000422	1.000422274	protein_coding	NM_032778
ASHGV40025573	1.000006	1.000005969	protein_coding	NM_001771
ASHGV40008337	0.999926	0.999925704	protein_coding	NM_001145033
ASHGV40032706	0.999787	0.99978734	protein_coding	NM_020794
ASHGV40048094	0.999537	0.99953654	protein_coding	NM_138290
ASHGV40007107	-0.99942	0.999421382	protein_coding	NM_173586
ASHGV40027025	0.999408	0.999407773	protein_coding	NM_198843
ASHGV40003223	-0.99938	0.999381908	protein_coding	NM_001290693
ASHGV40020562	0.999045	0.9990451	protein_coding	NM_005749
ASHGV40036433	-0.99871	0.998707534	protein_coding	NM_006784
ASHGV40020634	0.998694	0.99869402	protein_coding	NM_022735

ASHGV40052226	0.998653	0.99865276	protein_coding	NM_014010
ASHGV40003240	0.99863	0.998629974	protein_coding	NM_001487
ASHGV40052261	0.998607	0.998606671	protein_coding	NM_004099
ASHGV40050529	0.998311	0.998310924	protein_coding	NM_015214
ASHGV40037479	-0.9983	0.998303139	protein_coding	NM_003215
ASHGV40025463	-0.99786	0.997864406	protein_coding	ENST00000357726
ASHGV40034854	-0.9977	0.997695611	protein_coding	NM_144639
ASHGV40014492	-0.99769	0.997693938	protein_coding	NM_020699
ASHGV40027666	0.997632	0.997632393	protein_coding	NM_002976
ASHGV40034424	0.997618	0.997617553	protein_coding	NM_001064
ASHGV40050136	-0.99761	0.997613643	protein_coding	NM_013432
ASHGV40045419	0.997496	0.997496368	protein_coding	NM_052904
ASHGV40024947	-0.99716	0.997157488	protein_coding	NM_004368
ASHGV40054561	0.996951	0.996950611	protein_coding	NM_178547
ASHGV40026959	-0.99678	0.996784486	protein_coding	NM_133637
ASHGV40038168	0.99667	0.996670493	protein_coding	NM_001334
ASHGV40012143	-0.99664	0.996642896	protein_coding	NM_152269
ASHGV40009891	0.996546	0.996545888	protein_coding	NM_024604
ASHGV40015899	-0.99642	0.996421165	protein_coding	NM_173814
ASHGV40024358	0.996168	0.996167811	protein_coding	NM_001398
ASHGV40025874	-0.9961	0.99610022	protein_coding	NM_177973
ASHGV40001227	-0.99607	0.996068649	protein_coding	NM_005850
ASHGV40014795	-0.9959	0.995901781	protein_coding	NM_005794
ASHGV40035169	0.99569	0.995690335	protein_coding	NM_013296
ASHGV40044965	-0.99568	0.995675877	protein_coding	NM_207409
ASHGV40007785	-0.99563	0.995631933	protein_coding	NM_014312
ASHGV40040342	0.995586	0.995586039	protein_coding	NM_003187
ASHGV40019650	-0.99551	0.995506515	protein_coding	NM_015721
ASHGV40037807	0.995454	0.995453621	protein_coding	NM_005826
ASHGV40036522	-0.99513	0.995127161	protein_coding	NM_016527
ASHGV40027846	-0.99498	0.994976783	protein_coding	ENST00000421038
ASHGV40046842	0.994967	0.994967346	protein_coding	NM_017460
ASHGV40052155	0.99491	0.994910458	protein_coding	NM_018287
ASHGV40017230	-0.99465	0.994652664	protein_coding	NM_022767
ASHGV40031445	0.994513	0.994513188	protein_coding	NM_001247
ASHGV40033480	0.994459	0.994458929	protein_coding	NM_199127
ASHGV40009318	0.994331	0.994331074	protein_coding	NM_198077
ASHGV40030588	0.994223	0.994222899	protein_coding	NM_014426
ASHGV40055965	-0.99404	0.994042748	protein_coding	NM_001195156
ASHGV40021713	-0.994	0.994003914	protein_coding	NM_145274
ASHGV40030787	-0.99383	0.993825986	protein_coding	uc010gfc.1
ASHGV40041180	-0.99375	0.993753155	protein_coding	NM_001005214
ASHGV40034546	0.993751	0.993750626	protein_coding	NM_032682
ASHGV40003146	0.99351	0.993510439	protein_coding	NM_001694
ASHGV40015465	-0.9931	0.993101361	protein_coding	NM_001004467
ASHGV40007708	0.992972	0.992972004	protein_coding	NM_004397
ASHGV40052307	0.992886	0.992885805	protein_coding	NM_007209

ASHGV40048253	-0.99284	0.992836768	protein_coding	NM_000602
ASHGV40025222	-0.99241	0.992409735	protein_coding	NM_006397
ASHGV40043863	-0.99212	0.992122925	protein_coding	NM_198468
ASHGV40017152	-0.99211	0.992111576	protein_coding	NM_173528
ASHGV40047222	0.992024	0.992024477	protein_coding	NM_016071
ASHGV40008938	-0.99198	0.99198297	protein_coding	NM_145018
ASHGV40008047	-0.99195	0.99195124	protein_coding	NM_001004489
ASHGV40008757	-0.99185	0.991854787	protein_coding	NM_001293291
ASHGV40017056	-0.99181	0.991812128	protein_coding	NM_001307939
ASHGV40014236	0.99168	0.991680354	protein_coding	NM_004450
ASHGV40038570	-0.99162	0.991620986	protein_coding	NM_003652
ASHGV40012543	0.991545	0.991545199	protein_coding	uc010tfw.1
ASHGV40043236	-0.99141	0.991413906	protein_coding	NM_133471
ASHGV40014716	-0.99139	0.991387056	protein_coding	ENST00000553765
ASHGV40054615	-0.99126	0.991256355	protein_coding	NM_002183
ASHGV40043480	-0.99118	0.991177803	protein_coding	NM_002630
ASHGV40056503	-0.99114	0.991143021	protein_coding	uc002mul.1
ASHGV40049696	-0.9911	0.991096452	protein_coding	NM_001270379
ASHGV40045313	0.990748	0.990747908	protein_coding	NM_000056
ASHGV40046356	-0.99048	0.990481899	protein_coding	NM_001077653
ASHGV40052758	-0.99046	0.990462549	protein_coding	NM_002504
ASHGV40034388	-0.99044	0.99044185	protein_coding	NM_145071
ASHGV40020355	0.990173	0.990173376	protein_coding	NM_012232
ASHGV40033535	-0.99014	0.990142836	protein_coding	NM_000496
ASHGV40048444	-0.98994	0.989937951	protein_coding	NM_001458
ASHGV40025381	-0.98969	0.98968977	protein_coding	NM_178526
ASHGV40057692	-0.98969	0.989688717	protein_coding	NM_020999
ASHGV40025451	-0.98956	0.989563764	protein_coding	NM_001013630
ASHGV40000261	-0.98949	0.989490097	protein_coding	ENST00000409518
ASHGV40044200	-0.9892	0.989201876	protein_coding	NM_006718
ASHGV40028040	-0.98875	0.988754608	protein_coding	NM_001282321
ASHGV40026074	-0.98838	0.98837696	protein_coding	ENST00000539076
ASHGV40020744	0.988374	0.988373715	protein_coding	NM_002816
ASHGV40056581	-0.98831	0.988311462	protein_coding	NM_001310127
ASHGV40028643	-0.98825	0.988247943	protein_coding	NM_145238
ASHGV40020336	-0.98802	0.988019712	protein_coding	NM_002230
ASHGV40055323	0.987979	0.987978725	protein_coding	NM_006667
ASHGV40042098	0.987662	0.987662143	protein_coding	NM_198507
ASHGV40045471	-0.98763	0.987627056	protein_coding	NM_018292
ASHGV40002724	-0.98757	0.987569502	protein_coding	ENST00000606025
ASHGV40014428	-0.98736	0.987358388	protein_coding	NM_021161
ASHGV40025055	-0.98713	0.987127247	protein_coding	uc002mbt.1
ASHGV40019424	0.987001	0.987001148	protein_coding	ENST00000563764
ASHGV40018152	0.986673	0.986673047	protein_coding	NM_015964
ASHGV40005066	0.986564	0.986564498	protein_coding	NM_000375
ASHGV40020312	-0.98654	0.986541499	protein_coding	NM_001257309
ASHGV40048975	-0.98646	0.986463187	protein_coding	NM_003053

ASHGV40029405	0.986454	0.986454468	protein_coding	NM_001079862
ASHGV40000776	-0.98645	0.986448844	protein_coding	ENST00000433992
ASHGV40009457	0.986395	0.986394538	protein_coding	NM_004261
ASHGV40025214	0.986229	0.986228573	protein_coding	NM_032332
ASHGV40027078	-0.98604	0.986044566	protein_coding	NM_032788
ASHGV40024895	-0.98596	0.985964173	protein_coding	NM_198458
ASHGV40032193	-0.98596	0.985962542	protein_coding	NM_019596
ASHGV40040662	0.985643	0.985643199	protein_coding	NM_005669
ASHGV40003330	-0.98562	0.985619946	protein_coding	NM_080819
ASHGV40039115	0.985497	0.985497214	protein_coding	NM_016129
ASHGV40012262	-0.98528	0.985283442	protein_coding	uc001ujv.3
ASHGV40036538	-0.98525	0.985252416	protein_coding	NM_001099
ASHGV40001872	-0.98518	0.985179553	protein_coding	ENST00000531323
ASHGV40016968	0.985029	0.98502873	protein_coding	NM_001143936
ASHGV40056146	-0.9848	0.984797247	protein_coding	uc001yta.1
ASHGV40053726	-0.98475	0.984752586	protein_coding	NM_005044
ASHGV40040756	-0.9846	0.984601799	protein_coding	NM_001102566
ASHGV40030263	-0.98448	0.984475089	protein_coding	NM_001195129
ASHGV40023758	0.984288	0.984288271	protein_coding	NM_019107
ASHGV40043072	0.984278	0.984277507	protein_coding	NM_000332
ASHGV40043629	0.984062	0.984062323	protein_coding	NM_019036
ASHGV40006311	0.984031	0.984030881	protein_coding	NM_021622
ASHGV40041265	-0.984	0.984004937	protein_coding	NM_000843
ASHGV40047687	0.983975	0.983975192	protein_coding	NM_022786
ASHGV40011728	0.983797	0.983797416	protein_coding	NM_015336
ASHGV40021857	0.983575	0.98357535	protein_coding	NM_006310
ASHGV40008947	0.983326	0.9833263	protein_coding	NM_032273
ASHGV40045583	-0.98281	0.982810711	protein_coding	NM_001010892
ASHGV40000063	-0.98223	0.982226449	protein_coding	NM_176823
ASHGV40010719	0.98215	0.982150361	protein_coding	NM_001247997
ASHGV40027640	0.982013	0.982013025	protein_coding	NM_022168
ASHGV40037110	0.981554	0.981554084	protein_coding	NM_006651
ASHGV40030807	0.981386	0.981386413	protein_coding	NM_032013
ASHGV40021613	0.981386	0.981386109	protein_coding	NM_001282
ASHGV40049811	0.981368	0.981367818	protein_coding	NM_006265
ASHGV40033792	-0.98134	0.981341949	protein_coding	NM_001002034
ASHGV40002653	0.981193	0.981192761	protein_coding	ENST00000597959
ASHGV40014414	-0.98104	0.981043824	protein_coding	NM_002961
ASHGV40031504	0.981028	0.9810277	protein_coding	NM_182519
ASHGV40024345	0.980863	0.980862928	protein_coding	NM_001039672
ASHGV40042873	-0.9808	0.980802824	protein_coding	NM_178422
ASHGV40021129	0.980624	0.980624125	protein_coding	NM_000049
ASHGV40011244	-0.98057	0.980565924	protein_coding	NM_001013620
ASHGV40003257	0.980456	0.980456224	protein_coding	NM_004074
ASHGV40031351	0.980377	0.980376621	protein_coding	NM_002594
ASHGV40043353	0.980036	0.980036097	protein_coding	NM_004761
ASHGV40032231	-0.97999	0.979985683	protein_coding	uc001bcf.2

ASHGV40060872	0.97994	0.979939516	protein_coding	uc010mmv.3
ASHGV40035211	0.979852	0.979851846	protein_coding	NM_014498
ASHGV40008371	0.979666	0.979666099	protein_coding	NM_001142673
ASHGV40038126	0.97933	0.979329516	protein_coding	NM_001009555
ASHGV40054551	0.979077	0.979077014	protein_coding	NM_021800
ASHGV40041761	-0.97888	0.978881375	protein_coding	NM_024415
ASHGV40056739	-0.97852	0.978521171	protein_coding	NM_016510
ASHGV40026105	-0.97821	0.978211134	protein_coding	NM_018337
ASHGV40014966	-0.97775	0.97775036	protein_coding	NM_152329
ASHGV40042568	-0.97775	0.977745633	protein_coding	NM_133371
ASHGV40003103	-0.97761	0.977605187	protein_coding	NM_001042705
ASHGV40032187	0.977541	0.977541206	protein_coding	NM_021254
ASHGV40034863	-0.97747	0.977471003	protein_coding	NM_001136053
ASHGV40053577	-0.97746	0.977458103	protein_coding	ENST00000371955
ASHGV40052525	-0.97745	0.977452172	protein_coding	NM_004479
ASHGV40035191	0.97745	0.977449942	protein_coding	NM_014926
ASHGV40024954	-0.97744	0.977436732	protein_coding	NM_177401
ASHGV40044971	-0.97725	0.977250439	protein_coding	NM_001145717
ASHGV40025056	-0.97698	0.976979074	protein_coding	NM_015015
ASHGV40052568	0.976817	0.976816896	protein_coding	NM_145011
ASHGV40015546	-0.97637	0.976370341	protein_coding	NM_001137601
ASHGV40002336	-0.97622	0.976217971	protein_coding	ENST00000568114
ASHGV40036207	0.976217	0.976217072	protein_coding	NM_014043
ASHGV40019799	-0.97612	0.976118371	protein_coding	NM_001170754
ASHGV40044949	0.975915	0.975915407	protein_coding	NM_020804
ASHGV40033917	-0.9757	0.97569594	protein_coding	NM_001253845
ASHGV40001849	-0.97568	0.975678655	protein_coding	NM_002728
ASHGV40025418	-0.97559	0.975593095	protein_coding	ENST00000380870
ASHGV40044375	0.975567	0.975566841	protein_coding	ENST00000545867
ASHGV40006844	0.975526	0.975525813	protein_coding	NM_001001991
ASHGV40000400	-0.9755	0.975504818	protein_coding	ENST00000416266
ASHGV40031239	-0.97505	0.975053521	protein_coding	NM_001110514
ASHGV40007212	-0.97505	0.975048101	protein_coding	NM_178545
ASHGV40024848	0.974987	0.97498701	protein_coding	NM_022103
ASHGV40017665	0.97489	0.974889594	protein_coding	NM_144600
ASHGV40028917	0.974701	0.974700712	protein_coding	NM_015252
ASHGV40034255	-0.97469	0.97469372	protein_coding	ENST00000436261
ASHGV40006975	-0.9742	0.974203007	protein_coding	NM_001002925
ASHGV40007488	0.973951	0.973950874	protein_coding	NM_144665
ASHGV40025345	0.97378	0.973779829	protein_coding	NM_002248
ASHGV40027913	0.973427	0.973426567	protein_coding	NM_004071
ASHGV40021011	0.972919	0.9729192	protein_coding	NM_002861
ASHGV40037611	0.972918	0.972918269	protein_coding	NM_001098477
ASHGV40051481	-0.97286	0.972864727	protein_coding	NM_006911
ASHGV40040081	0.972696	0.972695706	protein_coding	NM_016107
ASHGV40005026	-0.97267	0.972672576	protein_coding	NM_022034
ASHGV40030138	0.97267	0.972670405	protein_coding	NM_000784

ASHGV40057804	-0.97263	0.972628892	protein_coding	NM_020179
ASHGV40005744	0.972545	0.972545025	protein_coding	NM_004896
ASHGV40039973	0.972484	0.972484486	protein_coding	NM_001102562
ASHGV40046661	0.972462	0.972462257	protein_coding	NM_004603
ASHGV40005852	-0.97233	0.972333519	protein_coding	NM_144990
ASHGV40002042	-0.9723	0.972302993	protein_coding	ENST00000549576
ASHGV40054949	-0.97164	0.971644284	protein_coding	NM_001080489
ASHGV40017214	-0.9716	0.971595409	protein_coding	NM_001310153
ASHGV40000241	-0.97148	0.971483796	protein_coding	NM_145699
ASHGV40040251	-0.97139	0.971386079	protein_coding	NM_021147
ASHGV40048151	0.971251	0.971251432	protein_coding	NM_015068
ASHGV40025187	-0.97118	0.9711764	protein_coding	NM_018687
ASHGV40042693	-0.97113	0.971130835	protein_coding	NM_012188
ASHGV40031865	-0.97101	0.97101482	protein_coding	NM_178457
ASHGV40026313	0.970629	0.970628789	protein_coding	NM_138799
ASHGV40009326	0.970299	0.97029901	protein_coding	NM_012137
ASHGV40003310	-0.97029	0.970293829	protein_coding	NM_153840
ASHGV40033452	-0.97023	0.970229049	protein_coding	NM_001291006
ASHGV40035297	0.970228	0.97022771	protein_coding	NM_016559
ASHGV40038124	-0.97019	0.970191519	protein_coding	NM_019032
ASHGV40049450	0.970164	0.970163863	protein_coding	NM_018702
ASHGV40055870	0.96988	0.969880058	protein_coding	NM_015179
ASHGV40044399	-0.96987	0.969871563	protein_coding	NM_004610
ASHGV40031529	0.969871	0.969871497	protein_coding	NM_032514
ASHGV40007214	-0.9696	0.969597805	protein_coding	NM_031492
ASHGV40055490	0.969585	0.969584839	protein_coding	NM_002024
ASHGV40028662	0.96957	0.969570199	protein_coding	NM_018158
ASHGV40000157	-0.96946	0.969459203	protein_coding	NM_021246
ASHGV40024937	-0.96942	0.969419351	protein_coding	NM_031991
ASHGV40014767	-0.96933	0.969334527	protein_coding	NM_006912
ASHGV40019513	-0.96922	0.969216281	protein_coding	NM_031476
ASHGV40022593	0.969197	0.96919748	protein_coding	NM_000271
ASHGV40002476	-0.96878	0.968776722	protein_coding	NM_001145127
ASHGV40035503	-0.96873	0.96872579	protein_coding	ENST00000412723
ASHGV40040095	-0.96869	0.968685699	protein_coding	NM_016180
ASHGV40051527	0.96812	0.968120298	protein_coding	NM_020824
ASHGV40032936	0.967937	0.967937439	protein_coding	NM_001080843
ASHGV40034771	-0.96786	0.967863769	protein_coding	NM_001282563
ASHGV40033338	-0.96779	0.967792463	protein_coding	NM_001001694
ASHGV40049601	0.967732	0.967732363	protein_coding	NM_002485
ASHGV40015515	0.967693	0.96769344	protein_coding	NM_002376
ASHGV40055861	-0.96761	0.967608908	protein_coding	NM_152309
ASHGV40034837	0.967453	0.967452566	protein_coding	NM_021964
ASHGV40024596	-0.96735	0.967354981	protein_coding	NM_144577
ASHGV40042214	-0.96729	0.967292679	protein_coding	NM_014350
ASHGV40016680	-0.967	0.966997707	protein_coding	NM_002875
ASHGV40051347	0.966916	0.966915987	protein_coding	NM_001702

ASHGV40033614	-0.96685	0.966845533	protein_coding	NM_006932
ASHGV40013119	0.966673	0.966672609	protein_coding	NM_006646
ASHGV40034009	0.966232	0.966232079	protein_coding	NM_003256
ASHGV40025838	0.966204	0.966203524	protein_coding	NM_004491
ASHGV40020846	0.966147	0.966146867	protein_coding	NM_006356
ASHGV40003314	0.966044	0.966044099	protein_coding	NM_032270
ASHGV40020972	0.96582	0.96582027	protein_coding	NM_002522
ASHGV40050266	-0.96563	0.965630788	protein_coding	NM_001715
ASHGV40048645	-0.9655	0.96550393	protein_coding	NM_198455
ASHGV40006910	0.96533	0.965330372	protein_coding	NM_004813
ASHGV40053747	-0.96533	0.965326327	protein_coding	NM_012080
ASHGV40006525	0.965278	0.965278198	protein_coding	NM_007170
ASHGV40056085	-0.96514	0.965143245	protein_coding	NM_002934
ASHGV40055588	0.96512	0.965119656	protein_coding	NM_001303544
ASHGV40041841	0.964866	0.964865609	protein_coding	NM_015183
ASHGV40028984	0.964844	0.964844263	protein_coding	NM_006857
ASHGV40051643	0.964627	0.964627212	protein_coding	NM_002493
ASHGV40013140	-0.96421	0.964210974	protein_coding	ENST00000381026
ASHGV40032371	-0.96391	0.963911201	protein_coding	ENST00000400368
ASHGV40043316	-0.96376	0.963758077	protein_coding	NM_001304561
ASHGV40049011	-0.96363	0.963629949	protein_coding	NM_003840
ASHGV40029716	0.963301	0.963301171	protein_coding	NM_012198
ASHGV40017553	0.962917	0.962916878	protein_coding	NM_030934
ASHGV40017863	-0.96289	0.962892284	protein_coding	ENST00000602217
ASHGV40019822	-0.96275	0.962746342	protein_coding	NM_004217
ASHGV40038514	-0.9623	0.962299217	protein_coding	NM_002167
ASHGV40044327	0.962268	0.962268031	protein_coding	NM_000636
ASHGV40026972	-0.96223	0.962225165	protein_coding	NM_032181
ASHGV40052198	-0.96221	0.962209907	protein_coding	NM_145051
ASHGV40028673	-0.96215	0.962153922	protein_coding	NM_182756
ASHGV40047713	-0.96181	0.961809152	protein_coding	NM_001004342
ASHGV40023887	0.961792	0.96179219	protein_coding	NM_012289
ASHGV40024741	-0.96172	0.961721284	protein_coding	NM_006969
ASHGV40014454	-0.96166	0.961663052	protein_coding	NM_001080414
ASHGV40003269	-0.96151	0.961505188	protein_coding	NM_005322
ASHGV40029367	0.961482	0.961481637	protein_coding	NM_005721
ASHGV40030373	-0.96145	0.961452605	protein_coding	NM_001136537
ASHGV40021858	0.961081	0.961081177	protein_coding	NM_002265
ASHGV40054692	-0.96107	0.961070101	protein_coding	NM_016562
ASHGV40001339	-0.96102	0.961022734	protein_coding	NM_022551
ASHGV40015629	-0.96084	0.960837325	protein_coding	NM_000275
ASHGV40016692	0.960749	0.960748663	protein_coding	NM_007236
ASHGV40020081	-0.96059	0.960591615	protein_coding	NM_152465
ASHGV40023044	0.960451	0.960450881	protein_coding	NM_001117
ASHGV40019318	0.960265	0.960264968	protein_coding	NM_000228
ASHGV40042072	-0.96007	0.960069095	protein_coding	ENST00000436592
ASHGV40055242	-0.96002	0.960023467	protein_coding	NM_001024593

ASHGV40053845	0.960009	0.96000904	protein_coding	NM_024122
ASHGV40046122	0.959924	0.959924473	protein_coding	NM_001101
ASHGV40005004	-0.95986	0.95986214	protein_coding	NM_024834
ASHGV40049408	-0.95982	0.959815093	protein_coding	NM_001242318
ASHGV40008610	-0.95977	0.959773991	protein_coding	NM_144585
ASHGV40013043	0.959749	0.959748682	protein_coding	NM_017520
ASHGV40031871	0.959721	0.959721116	protein_coding	NM_022106
ASHGV40018595	-0.95961	0.959614453	protein_coding	NM_001276
ASHGV40020265	-0.95951	0.959508388	protein_coding	NM_018530
ASHGV40028209	-0.95942	0.959417277	protein_coding	NM_004826
ASHGV40012133	-0.95939	0.959391633	protein_coding	NM_014708
ASHGV40021005	0.959341	0.959340918	protein_coding	NM_001007533
ASHGV40054950	-0.95931	0.959305825	protein_coding	NM_002049
ASHGV40024561	-0.95909	0.959085481	protein_coding	NM_000960
ASHGV40028571	0.95886	0.958860445	protein_coding	NM_004040
ASHGV40002691	-0.95857	0.958570033	protein_coding	ENST00000601460
ASHGV40009984	-0.95854	0.958540305	protein_coding	NM_005554
ASHGV40001980	0.958486	0.958486441	protein_coding	NM_001004060
ASHGV40009949	0.958287	0.958286575	protein_coding	NM_030809
ASHGV40047316	0.95827	0.958270119	protein_coding	NM_001003801
ASHGV40012870	0.958228	0.958228343	protein_coding	NM_015296
ASHGV40028339	-0.95805	0.958045446	protein_coding	uc002wcb.2
ASHGV40045284	0.95804	0.958039819	protein_coding	NM_004999
ASHGV40054420	0.958026	0.958026	protein_coding	NM_004208
ASHGV40029012	0.957992	0.957991592	protein_coding	NM_003494
ASHGV40023893	-0.95795	0.957949791	protein_coding	NM_005498
ASHGV40020308	-0.95793	0.957931425	protein_coding	NM_031854
ASHGV40020927	-0.95787	0.957873911	protein_coding	ENST00000374983
ASHGV40048278	0.957754	0.957754203	protein_coding	NM_004279
ASHGV40044246	-0.95709	0.957092791	protein_coding	NM_024518
ASHGV40055475	0.957039	0.957039134	protein_coding	NM_032539
ASHGV40026378	0.956936	0.956935508	protein_coding	NM_015909
ASHGV40052684	-0.95676	0.956760009	protein_coding	NM_001010887
ASHGV40007871	0.956754	0.956753788	protein_coding	NM_145243
ASHGV40050826	0.956536	0.956535694	protein_coding	NM_018972
ASHGV40035621	0.956532	0.956532144	protein_coding	NM_003178
ASHGV40053639	-0.95651	0.956513824	protein_coding	NM_001145639
ASHGV40030675	-0.95634	0.956343142	protein_coding	NM_001899
ASHGV40040025	0.955697	0.955696615	protein_coding	NM_006727
ASHGV40028816	-0.95565	0.955645249	protein_coding	NM_178548
ASHGV40048560	-0.9554	0.955396581	protein_coding	NM_016944
ASHGV40033411	0.955328	0.95532758	protein_coding	NM_003776
ASHGV40029659	0.954994	0.954994134	protein_coding	NM_052917
ASHGV40031102	0.954845	0.954844848	protein_coding	NM_014258
ASHGV40021964	-0.95482	0.954821378	protein_coding	NM_001243552
ASHGV40027840	0.954506	0.954505686	protein_coding	NM_016467
ASHGV40029216	0.954414	0.954413868	protein_coding	NM_001134224

ASHGV40017995	-0.95408	0.954083781	protein_coding	NM_001080430
ASHGV40039619	0.954077	0.954077255	protein_coding	NM_001829
ASHGV40002278	-0.95392	0.953918293	protein_coding	ENST00000564080
ASHGV40052180	0.95378	0.95377994	protein_coding	NM_012212
ASHGV40027168	-0.95357	0.953566534	protein_coding	NM_001127691
ASHGV40048500	-0.95318	0.953180941	protein_coding	NM_144648
ASHGV40034840	0.952969	0.952969201	protein_coding	NM_003794
ASHGV40021868	0.952793	0.952793363	protein_coding	NM_018129
ASHGV40012933	0.95247	0.952469898	protein_coding	NM_001080396
ASHGV40041160	0.952448	0.952447577	protein_coding	NM_012300
ASHGV40024119	-0.95226	0.952263908	protein_coding	uc002nnl.2
ASHGV40023736	0.951972	0.951972104	protein_coding	NM_001961
ASHGV40035757	0.951958	0.951957758	protein_coding	NM_178868
ASHGV40013843	0.951915	0.951914952	protein_coding	NM_018379
ASHGV40044884	0.951836	0.951836064	protein_coding	NM_006929
ASHGV40042131	-0.95141	0.951413814	protein_coding	NM_005246
ASHGV40029184	-0.95119	0.951191408	protein_coding	NM_016044
ASHGV40056975	-0.95119	0.951188819	protein_coding	NM_138461
ASHGV40040751	0.95098	0.950979661	protein_coding	NM_032174
ASHGV40053132	-0.95082	0.950821675	protein_coding	NM_033086
ASHGV40015835	-0.9508	0.950801961	protein_coding	NM_001194998
ASHGV40000252	0.950484	0.950483838	protein_coding	NM_002688
ASHGV40038744	0.950359	0.950359269	protein_coding	NM_002589
ASHGV40023899	0.950297	0.950296572	protein_coding	ENST00000253031
ASHGV40011523	0.950292	0.950292107	protein_coding	NM_021019
ASHGV40018916	0.950193	0.950192837	protein_coding	NM_007074
ASHGV40035093	-0.95017	0.950170538	protein_coding	NM_014879
ASHGV40017725	-0.95015	0.950153413	protein_coding	NM_005849
ASHGV40056533	-0.95006	0.950056716	protein_coding	ENST00000454971
ASHGV40023417	-0.94969	0.949691996	protein_coding	NM_001257964
ASHGV40018542	-0.94967	0.949672306	protein_coding	NM_144570
ASHGV40013301	-0.94938	0.949383457	protein_coding	NM_015116
ASHGV40047481	-0.94895	0.948945377	protein_coding	NM_153247
ASHGV40036585	0.948659	0.948659466	protein_coding	NM_020191
ASHGV40056627	0.948581	0.948581474	protein_coding	NM_006794
ASHGV40040184	-0.94851	0.948509883	protein_coding	NM_002130
ASHGV40056171	0.948475	0.948474632	protein_coding	NM_001301268
ASHGV40034969	-0.94841	0.948411293	protein_coding	NM_016161
ASHGV40047447	-0.94838	0.948380233	protein_coding	ENST00000318959
ASHGV40054820	0.948167	0.948166708	protein_coding	NM_000950
ASHGV40029223	0.948066	0.948066107	protein_coding	NM_015904
ASHGV40035885	-0.94769	0.947693927	protein_coding	NM_003241
ASHGV40028340	-0.94744	0.947436266	protein_coding	NM_012145
ASHGV40018968	-0.9472	0.94720185	protein_coding	NM_016633
ASHGV40011924	-0.94717	0.94717404	protein_coding	NM_206996
ASHGV40011066	0.947115	0.947114789	protein_coding	NM_001987
ASHGV40002751	-0.9471	0.947103506	protein_coding	NM_001195541

ASHGV40023415	0.946919	0.946919109	protein_coding	NM_015285
ASHGV40026434	0.946322	0.946322432	protein_coding	NM_015317
ASHGV40025049	-0.94625	0.946252853	protein_coding	NM_152362
ASHGV40054689	0.946034	0.946034071	protein_coding	NM_014728
ASHGV40010344	-0.946	0.945997033	protein_coding	NM_005447
ASHGV40035290	0.945965	0.945965332	protein_coding	NM_002885
ASHGV40010807	-0.94576	0.945762384	protein_coding	NM_001143989
ASHGV40003093	-0.94571	0.945713789	protein_coding	NM_001037675
ASHGV40054832	0.945697	0.94569667	protein_coding	NM_021242
ASHGV40020502	-0.94565	0.945652836	protein_coding	NM_024015
ASHGV40027091	0.945633	0.945633167	protein_coding	NM_207328
ASHGV40002120	-0.94559	0.945593073	protein_coding	ENST00000555591
ASHGV40042420	-0.94555	0.945548069	protein_coding	NM_018909
ASHGV40034445	-0.9455	0.945498863	protein_coding	NM_003392
ASHGV40049260	-0.94538	0.945381017	protein_coding	NM_024593
ASHGV40008419	-0.94526	0.945255324	protein_coding	NM_001004738
ASHGV40008698	-0.94525	0.945250582	protein_coding	NM_005133
ASHGV40012952	0.945151	0.945151379	protein_coding	NM_003749
ASHGV40038513	-0.94502	0.945015614	protein_coding	NM_145291
ASHGV40007078	-0.94483	0.944829496	protein_coding	NM_004739
ASHGV40008268	0.944756	0.944755996	protein_coding	NM_012194
ASHGV40016191	0.944661	0.944660646	protein_coding	NM_025234
ASHGV40053559	-0.9446	0.944603979	protein_coding	NM_020064
ASHGV40017515	-0.94443	0.944427997	protein_coding	NM_022119
ASHGV40007644	0.944348	0.944348083	protein_coding	NM_030770
ASHGV40040866	-0.94424	0.94423668	protein_coding	NM_001496
ASHGV40043876	0.94401	0.944009678	protein_coding	NM_005190
ASHGV40034905	0.943762	0.943762426	protein_coding	NM_014602
ASHGV40044878	0.94345	0.943449639	protein_coding	NM_001040437
ASHGV40032156	-0.94332	0.943318623	protein_coding	NM_181607
ASHGV40052510	-0.94314	0.943142141	protein_coding	NM_198946
ASHGV40047895	0.943068	0.943068056	protein_coding	NM_018697
ASHGV40044740	-0.94293	0.94292781	protein_coding	NM_021052
ASHGV40011120	-0.94288	0.942882593	protein_coding	NM_004570
ASHGV40040565	0.942755	0.942754969	protein_coding	NM_014639
ASHGV40045332	0.942699	0.942698943	protein_coding	NM_015018
ASHGV40026044	-0.94269	0.942686963	protein_coding	NM_004431
ASHGV40006514	0.942518	0.942518223	protein_coding	NM_005170
ASHGV40002681	-0.94244	0.942435488	protein_coding	ENST00000599764
ASHGV40024398	0.942113	0.942112857	protein_coding	NM_182752
ASHGV40009557	0.942055	0.942054616	protein_coding	NM_002355
ASHGV40021335	-0.94185	0.941849637	protein_coding	NM_006041
ASHGV40042868	-0.94184	0.94184359	protein_coding	NM_152547
ASHGV40015602	-0.94181	0.941806011	protein_coding	NM_001304388
ASHGV40016358	-0.94172	0.941717926	protein_coding	NM_003981
ASHGV40035372	-0.94155	0.941548665	protein_coding	NM_018138
ASHGV40042880	-0.9413	0.941299815	protein_coding	NM_002293

ASHGV40046806	0.941295	0.9412948	protein_coding	NM_006304
ASHGV40010536	0.941268	0.941268121	protein_coding	NM_001288821
ASHGV40019590	0.941213	0.941212509	protein_coding	NM_016209
ASHGV40053496	0.941044	0.941043923	protein_coding	NM_153433
ASHGV40018137	0.940958	0.940958022	protein_coding	NM_178818
ASHGV40031872	-0.9409	0.940903232	protein_coding	NM_021810
ASHGV40005079	-0.94063	0.940627158	protein_coding	uc009yar.1
ASHGV40056436	-0.94002	0.940022593	protein_coding	NM_001012716
ASHGV40026076	-0.94	0.94000494	protein_coding	NM_139172
ASHGV40047288	0.939935	0.939934789	protein_coding	NM_152557
ASHGV40053505	-0.93983	0.9398324	protein_coding	NM_004435
ASHGV40039444	-0.93965	0.939648244	protein_coding	NM_000585
ASHGV40010892	0.939474	0.939473519	protein_coding	NM_018979
ASHGV40031199	0.939297	0.939297309	protein_coding	NM_024958
ASHGV40012093	0.939191	0.939190532	protein_coding	NM_014868
ASHGV40026721	0.938976	0.938976083	protein_coding	NM_001190274
ASHGV40046444	-0.93894	0.938940514	protein_coding	NM_021223
ASHGV40032998	0.938315	0.938314776	protein_coding	NM_012399
ASHGV40054896	0.938231	0.938231407	protein_coding	NM_003680
ASHGV40017468	0.938165	0.93816511	protein_coding	NM_015101
ASHGV40024377	0.938131	0.938131288	protein_coding	NM_019088
ASHGV40036752	0.9381	0.938099598	protein_coding	NM_139245
ASHGV40032393	-0.93796	0.937959502	protein_coding	NM_133635
ASHGV40016809	0.937469	0.937468975	protein_coding	NM_181789
ASHGV40046870	-0.93741	0.93741064	protein_coding	NM_004444
ASHGV40021772	-0.93738	0.937381329	protein_coding	NM_145041
ASHGV40039097	0.937069	0.93706932	protein_coding	NM_020236
ASHGV40040904	0.936799	0.936798868	protein_coding	NM_133173
ASHGV40056776	-0.93667	0.936673905	protein_coding	NM_018244
ASHGV40042850	-0.93649	0.936490282	protein_coding	ENST00000499601
ASHGV40036219	0.936366	0.936365908	protein_coding	NM_018420
ASHGV40033351	0.936339	0.936339328	protein_coding	NM_002972
ASHGV40025585	-0.93631	0.936305567	protein_coding	NM_001300974
ASHGV40016248	-0.93623	0.936234863	protein_coding	ENST00000557886
ASHGV40023343	-0.9362	0.936199304	protein_coding	NM_213602
ASHGV40029324	-0.93601	0.936011797	protein_coding	NM_138621
ASHGV40023969	0.936	0.935999962	protein_coding	NM_001127221
ASHGV40045808	-0.93575	0.935753404	protein_coding	NM_001002255
ASHGV40002235	-0.93573	0.935733462	protein_coding	ENST00000562250
ASHGV40042546	-0.93518	0.935179503	protein_coding	NM_024028
ASHGV40022822	0.934955	0.934955022	protein_coding	NM_004786
ASHGV40039262	-0.93495	0.93494595	protein_coding	NM_017918
ASHGV40015314	-0.93487	0.934868885	protein_coding	ENST00000553725
ASHGV40042407	-0.93485	0.934852747	protein_coding	NM_080670
ASHGV40054440	-0.93485	0.934851067	protein_coding	NM_001448
ASHGV40031863	-0.93482	0.934819047	protein_coding	NM_030773
ASHGV40021116	0.934807	0.934806539	protein_coding	NM_000430

ASHGV40000151	-0.93476	0.934764512	protein_coding	NM_000389
ASHGV40040512	0.934752	0.934752403	protein_coding	NM_001239
ASHGV40003260	-0.9345	0.934496194	protein_coding	NM_004367
ASHGV40001885	-0.93449	0.934492903	protein_coding	ENST00000532254
ASHGV40052350	0.934186	0.934185881	protein_coding	NM_004486
ASHGV40021144	-0.93401	0.934013181	protein_coding	NM_182538
ASHGV40037518	-0.93395	0.933945642	protein_coding	NM_001292045
ASHGV40013803	-0.93389	0.933889069	protein_coding	NM_016423
ASHGV40029501	0.933744	0.93374384	protein_coding	NM_001308114
ASHGV40009447	-0.93373	0.933728469	protein_coding	ENST00000382678
ASHGV40050596	0.933604	0.933603524	protein_coding	NM_002027
ASHGV40053419	0.933378	0.933377957	protein_coding	NM_012197
ASHGV40052323	0.9329	0.932899988	protein_coding	NM_012098
ASHGV40047529	0.932613	0.932612722	protein_coding	NM_018374
ASHGV40028942	-0.93253	0.932529323	protein_coding	NM_002398
ASHGV40014619	0.932515	0.932515062	protein_coding	NM_004873
ASHGV40034914	0.9325	0.932500359	protein_coding	NM_007208
ASHGV40045649	0.9323	0.93229986	protein_coding	NM_001242849
ASHGV40055497	-0.93205	0.932049983	protein_coding	NM_001003892
ASHGV40008071	0.931979	0.931979295	protein_coding	NM_000990
ASHGV40021388	-0.93193	0.931929966	protein_coding	NM_006341
ASHGV40019951	0.931472	0.93147233	protein_coding	NM_144997
ASHGV40005986	0.930882	0.930882387	protein_coding	NM_032373
ASHGV40039017	-0.93054	0.930535483	protein_coding	NM_021225
ASHGV40048154	0.930151	0.930151386	protein_coding	NM_017650
ASHGV40033523	-0.92992	0.929916757	protein_coding	NM_016327
ASHGV40050385	0.929778	0.929777902	protein_coding	uc003xds.3
ASHGV40033024	-0.92976	0.92975539	protein_coding	NM_001105572
ASHGV40024230	-0.92972	0.929719949	protein_coding	NM_032816
ASHGV40036506	0.929604	0.929604141	protein_coding	NM_004637
ASHGV40041319	-0.92954	0.929544126	protein_coding	uc011cly.3
ASHGV40005068	-0.9295	0.92949505	protein_coding	NM_003474
ASHGV40030610	0.929382	0.929381874	protein_coding	ENST00000598007
ASHGV40029285	0.929095	0.929095157	protein_coding	NM_032411
ASHGV40055268	0.928821	0.928820807	protein_coding	NM_002764
ASHGV40045805	0.928809	0.928809433	protein_coding	NM_015278
ASHGV40022183	0.928633	0.928633153	protein_coding	NM_000999
ASHGV40026918	0.928622	0.928622467	protein_coding	NM_020459
ASHGV40032370	-0.92858	0.928578819	protein_coding	uc002zfl.1
ASHGV40014248	-0.92851	0.928505423	protein_coding	NM_003814
ASHGV40027125	0.928367	0.928366522	protein_coding	NM_001008215
ASHGV40022051	0.928259	0.928259164	protein_coding	NM_000717
ASHGV40002376	-0.9282	0.928204316	protein_coding	NM_001907
ASHGV40008382	0.928197	0.928197457	protein_coding	NM_003682
ASHGV40030795	-0.9282	0.928195144	protein_coding	NM_001010969
ASHGV40024856	0.928065	0.928064504	protein_coding	ENST00000289877
ASHGV40015570	-0.92799	0.927987117	protein_coding	uc001ysr.3

ASHGV40041832	0.927968	0.927968353	protein_coding	NM_015342
ASHGV40030350	-0.92791	0.927912253	protein_coding	NM_198582
ASHGV40039030	0.927627	0.9276266	protein_coding	NM_004885
ASHGV40006646	-0.92758	0.927581691	protein_coding	ENST00000596206
ASHGV40057859	-0.92746	0.927456844	protein_coding	NM_001145720
ASHGV40053657	-0.92734	0.927342174	protein_coding	NM_206920
ASHGV40052256	0.927332	0.927332068	protein_coding	NM_016322
ASHGV40021239	-0.92733	0.927328948	protein_coding	NM_203411
ASHGV40020290	0.927226	0.92722561	protein_coding	NM_000421
ASHGV40021073	-0.92711	0.927114404	protein_coding	NM_024792
ASHGV40026549	-0.92711	0.927109653	protein_coding	NM_001029883
ASHGV40005650	-0.92705	0.927051391	protein_coding	NM_012242
ASHGV40048958	-0.92675	0.926751452	protein_coding	NM_004467
ASHGV40014054	-0.92666	0.926660169	protein_coding	NM_002863
ASHGV40043328	-0.9264	0.926401827	protein_coding	NM_002120
ASHGV40056019	0.926171	0.926171042	protein_coding	NM_024026
ASHGV40057006	0.925935	0.925935174	protein_coding	NM_000661
ASHGV40021045	0.925896	0.925895827	protein_coding	NM_006822
ASHGV40025331	-0.92561	0.925609696	protein_coding	NM_138401
ASHGV40041004	0.925253	0.925253285	protein_coding	NM_005617
ASHGV40051192	-0.92505	0.925051085	protein_coding	NM_152412
ASHGV40048988	0.924984	0.924983828	protein_coding	NM_024815
ASHGV40040764	0.924952	0.924952248	protein_coding	NM_001182
ASHGV40036800	-0.92489	0.92488562	protein_coding	NM_014373
ASHGV40050357	-0.92487	0.924870626	protein_coding	NM_018068
ASHGV40009209	-0.9248	0.924799397	protein_coding	NM_001080441
ASHGV40009924	-0.92477	0.924766706	protein_coding	NM_005263
ASHGV40018667	-0.92458	0.924584822	protein_coding	uc002czb.3
ASHGV40000009	0.924565	0.924564676	protein_coding	NM_001301726
ASHGV40033508	0.924331	0.924330581	protein_coding	NM_001024938
ASHGV40010572	-0.92432	0.924320736	protein_coding	NM_021625
ASHGV40031362	0.924029	0.924029068	protein_coding	NM_020536
ASHGV40041046	-0.92401	0.924006664	protein_coding	NM_015465
ASHGV40041977	0.923924	0.923924263	protein_coding	NM_001825
ASHGV40001384	-0.92386	0.923859925	protein_coding	NM_001216
ASHGV40002575	0.923844	0.923843724	protein_coding	ENST00000592740
ASHGV40043643	0.92375	0.923749998	protein_coding	NM_016277
ASHGV40036209	0.923648	0.923647795	protein_coding	NM_000866
ASHGV40008263	0.923463	0.923463354	protein_coding	NM_018393
ASHGV40010018	0.923335	0.92333545	protein_coding	NM_014597
ASHGV40012155	-0.92333	0.923328424	protein_coding	NM_012463
ASHGV40027101	0.923321	0.92332093	protein_coding	NM_014014
ASHGV40005051	-0.92328	0.923284738	protein_coding	NM_001329
ASHGV40041259	0.923198	0.923198115	protein_coding	NM_020666
ASHGV40007914	0.923159	0.92315915	protein_coding	NM_006755
ASHGV40015680	0.922997	0.922996938	protein_coding	NM_181077
ASHGV40021178	0.922658	0.922657904	protein_coding	NM_004703

ASHGV40021092	-0.92261	0.922610643	protein_coding	NM_001163809
ASHGV40007020	0.922391	0.922390923	protein_coding	NM_002074
ASHGV40036562	0.922165	0.92216534	protein_coding	NM_000532
ASHGV40017572	0.922082	0.922081752	protein_coding	ENST00000345988
ASHGV40049716	0.922041	0.922041362	protein_coding	NM_032041
ASHGV40036938	-0.92201	0.922006909	protein_coding	NM_014375
ASHGV40027654	0.921709	0.921708964	protein_coding	NM_001199148
ASHGV40025244	-0.92137	0.92137201	protein_coding	NM_020350
ASHGV40057196	-0.9212	0.921199467	protein_coding	NM_003520
ASHGV40047560	0.920998	0.920998416	protein_coding	NM_001621
ASHGV40009118	0.920595	0.920594732	protein_coding	ENST00000528953
ASHGV40021902	-0.92012	0.92012478	protein_coding	ENST00000576461
ASHGV40009846	0.920082	0.920081789	protein_coding	NM_153026
ASHGV40055940	0.920071	0.920070933	protein_coding	NM_024040
ASHGV40009229	-0.91996	0.919962392	protein_coding	NM_001010926
ASHGV40057239	0.919928	0.919928356	protein_coding	NM_000865
ASHGV40038626	-0.91985	0.919847821	protein_coding	NM_001010857
ASHGV40021194	0.919785	0.919785171	protein_coding	NM_017523
ASHGV40053334	-0.91932	0.919319125	protein_coding	NM_133374
ASHGV40055238	0.919258	0.919257782	protein_coding	NM_004780
ASHGV40003248	0.919152	0.919151517	protein_coding	NM_002371
ASHGV40035590	-0.91914	0.91914465	protein_coding	NM_001025930
ASHGV40013338	0.918997	0.918996721	protein_coding	NM_024570
ASHGV40042314	0.918963	0.918962725	protein_coding	NM_014402
ASHGV40056572	0.918947	0.918946509	protein_coding	NM_001985
ASHGV40030770	-0.91889	0.918889082	protein_coding	NM_000779
ASHGV40007119	-0.91881	0.918812307	protein_coding	NM_014067
ASHGV40051705	0.91865	0.91864994	protein_coding	NM_139312
ASHGV40037451	-0.91863	0.918634727	protein_coding	NM_182592
ASHGV40008456	-0.91835	0.918349622	protein_coding	NM_001085458
ASHGV40000485	-0.91829	0.918290109	protein_coding	ENST00000419755
ASHGV40012746	-0.91816	0.918157021	protein_coding	NM_006237
ASHGV40031139	-0.91804	0.918043757	protein_coding	uc021wfy.2
ASHGV40038308	0.917722	0.917722283	protein_coding	NM_000027
ASHGV40045741	-0.91747	0.917472797	protein_coding	ENST00000573100
ASHGV40038163	0.917465	0.917464658	protein_coding	NM_001039580
ASHGV40030521	0.917358	0.917357761	protein_coding	NM_021156
ASHGV40014213	-0.91728	0.917276519	protein_coding	NM_001009931
ASHGV40031191	-0.91726	0.917263219	protein_coding	NM_080831
ASHGV40016025	0.916971	0.916970548	protein_coding	NM_006049
ASHGV40035361	0.916822	0.916822021	protein_coding	NM_004593
ASHGV40054925	-0.91677	0.916768634	protein_coding	ENST00000357412
ASHGV40008050	-0.91667	0.916669193	protein_coding	NM_207186
ASHGV40037245	0.916652	0.91665172	protein_coding	NM_148894
ASHGV40053548	0.91657	0.916570115	protein_coding	NM_031426
ASHGV40034799	0.916557	0.916557252	protein_coding	NM_001146156
ASHGV40057381	0.916551	0.916550503	protein_coding	NM_001130929

ASHGV40043359	0.916166	0.916166429	protein_coding	NM_001014840
ASHGV40025365	-0.91608	0.916075895	protein_coding	NM_001243
ASHGV40036681	-0.91578	0.915778877	protein_coding	NM_207365
ASHGV40044855	-0.91568	0.915678664	protein_coding	NM_007109
ASHGV40009631	-0.91555	0.915549612	protein_coding	NM_018654
ASHGV40005317	-0.91554	0.915542858	protein_coding	NM_024693
ASHGV40047069	-0.91521	0.915213814	protein_coding	NM_178827
ASHGV40007797	-0.91512	0.915123981	protein_coding	NM_001612
ASHGV40005701	0.915096	0.915096002	protein_coding	NM_032199
ASHGV40033988	0.915083	0.915082943	protein_coding	NM_173567
ASHGV40007924	-0.91501	0.915006996	protein_coding	NM_001286606
ASHGV40050374	-0.91483	0.914829976	protein_coding	NM_003841
ASHGV40023770	0.91468	0.914679801	protein_coding	NM_205767
ASHGV40005792	-0.91468	0.914678934	protein_coding	NM_152635
ASHGV40027652	-0.91467	0.914671165	protein_coding	NM_001013642
ASHGV40043795	0.914627	0.914626517	protein_coding	NM_018064
ASHGV40052677	0.914396	0.914396232	protein_coding	NM_003026
ASHGV40022423	0.914246	0.914246184	protein_coding	NM_003803
ASHGV40009792	0.914196	0.914196181	protein_coding	NM_001113402
ASHGV40050183	0.914075	0.914074542	protein_coding	NM_004745
ASHGV40035330	0.913699	0.913699416	protein_coding	NM_020640
ASHGV40009264	-0.9136	0.913601413	protein_coding	NM_005422
ASHGV40035699	0.913583	0.91358265	protein_coding	NM_152653
ASHGV40021730	-0.91342	0.913423092	protein_coding	NM_021939
ASHGV40021212	-0.91339	0.913394827	protein_coding	NM_001251902
ASHGV40019577	-0.91329	0.913287041	protein_coding	ENST00000437464
ASHGV40018520	-0.91311	0.913114416	protein_coding	NM_021098
ASHGV40022350	-0.91274	0.912739756	protein_coding	NM_031945
ASHGV40005809	0.912665	0.912665128	protein_coding	NM_203298
ASHGV40027628	-0.91264	0.912641048	protein_coding	NM_006142
ASHGV40026673	0.912517	0.912517061	protein_coding	NM_133259
ASHGV40016246	-0.91229	0.912293768	protein_coding	uc010unx.1
ASHGV40024256	0.912108	0.912108254	protein_coding	NM_001007248
ASHGV40036542	0.912016	0.912016206	protein_coding	NM_024818
ASHGV40010583	0.911866	0.911865876	protein_coding	NM_016301
ASHGV40054707	-0.91155	0.911552566	protein_coding	NM_001297563
ASHGV40053801	0.911247	0.91124681	protein_coding	NM_003916
ASHGV40026018	0.911125	0.911125263	protein_coding	NM_018555
ASHGV40051022	-0.91068	0.910680469	protein_coding	NM_024915
ASHGV40000883	-0.91036	0.910364732	protein_coding	NM_145861
ASHGV40031957	0.91035	0.910350244	protein_coding	NM_032527
ASHGV40029781	0.910261	0.910260743	protein_coding	NM_024583
ASHGV40024265	-0.91022	0.910219628	protein_coding	NM_175872
ASHGV40016941	-0.91006	0.910059021	protein_coding	NM_182703
ASHGV40025587	-0.91004	0.910043121	protein_coding	NM_007000
ASHGV40015698	-0.90988	0.909877605	protein_coding	NM_170675
ASHGV40000868	-0.90957	0.909571184	protein_coding	ENST00000438485

ASHGV40035355	0.909463	0.909462903	protein_coding	NM_001025266
ASHGV40043428	-0.90944	0.909437863	protein_coding	NM_153487
ASHGV40037424	0.909213	0.90921317	protein_coding	NM_174921
ASHGV40054975	-0.90901	0.90901017	protein_coding	NM_033031
ASHGV40030670	-0.90889	0.90889471	protein_coding	NM_001008693
ASHGV40053903	0.908604	0.908604254	protein_coding	NM_000328
ASHGV40019929	0.908477	0.908476705	protein_coding	NM_001042697
ASHGV40048325	0.908399	0.908399319	protein_coding	NM_021994
ASHGV40046682	-0.90809	0.908089601	protein_coding	NM_006072
ASHGV40033444	-0.90806	0.908057835	protein_coding	NM_000185
ASHGV40021133	-0.90792	0.907918764	protein_coding	NM_031298
ASHGV40028918	-0.90791	0.907909828	protein_coding	NM_014562
ASHGV40026073	0.907705	0.907704638	protein_coding	NM_032430
ASHGV40027571	-0.90761	0.907610162	protein_coding	NM_017892
ASHGV40010074	0.907596	0.907596077	protein_coding	NM_004815
ASHGV40041288	-0.90753	0.907526531	protein_coding	NM_001001657
ASHGV40033842	0.90727	0.907269668	protein_coding	NM_018103
ASHGV40028537	-0.90722	0.90721761	protein_coding	NM_005378
ASHGV40055548	-0.9072	0.907198123	protein_coding	ENST00000428676
ASHGV40009769	0.907196	0.907195926	protein_coding	NM_016570
ASHGV40033127	-0.90711	0.907109463	protein_coding	NM_002872
ASHGV40037314	0.906899	0.90689884	protein_coding	NM_013261
ASHGV40037738	-0.90688	0.906881025	protein_coding	NM_144645
ASHGV40042471	-0.90683	0.906830269	protein_coding	NM_018928
ASHGV40002543	-0.90659	0.906586279	protein_coding	NM_001193552
ASHGV40009735	0.906515	0.906515289	protein_coding	NM_018164
ASHGV40002812	0.906464	0.906463763	protein_coding	NM_001204299
ASHGV40009255	-0.9064	0.906399592	protein_coding	ENST00000319763
ASHGV40047434	-0.9064	0.906397924	protein_coding	NM_001080461
ASHGV40016186	-0.90639	0.90638847	protein_coding	NM_144572
ASHGV40005726	0.906375	0.90637498	protein_coding	NM_178011
ASHGV40035965	-0.90634	0.906335846	protein_coding	NM_153215
ASHGV40011555	0.906296	0.906296073	protein_coding	NM_004984
ASHGV40031158	-0.90629	0.906285997	protein_coding	NM_000744
ASHGV40055550	0.906273	0.906272602	protein_coding	NM_001001344
ASHGV40033456	-0.90627	0.906270687	protein_coding	NM_001128633
ASHGV40040818	0.906259	0.906258725	protein_coding	NM_020199
ASHGV40051832	-0.90616	0.90616384	protein_coding	NM_206948
ASHGV40055153	0.90607	0.906069683	protein_coding	NM_019117
ASHGV40023873	-0.90607	0.906067493	protein_coding	NM_004230
ASHGV40047999	-0.90606	0.906062724	protein_coding	NM_182504
ASHGV40036925	0.906054	0.90605435	protein_coding	NM_021627
ASHGV40052912	-0.90596	0.905963546	protein_coding	ENST00000354995
ASHGV40011028	-0.90584	0.905840755	protein_coding	NM_004426
ASHGV40030238	0.905772	0.905772393	protein_coding	NM_016289
ASHGV40016642	-0.90546	0.90546238	protein_coding	NM_207444
ASHGV40055085	-0.90531	0.905308917	protein_coding	NM_006223

ASHGV40036911	0.905086	0.905086243	protein_coding	NM_144635
ASHGV40030008	-0.90504	0.905039494	protein_coding	NM_001302769
ASHGV40050096	0.904449	0.904449001	protein_coding	NM_001100878
ASHGV40032131	0.904403	0.904403293	protein_coding	NM_006585
ASHGV40044841	-0.90433	0.90433492	protein_coding	NM_178014
ASHGV40031565	0.90423	0.904229882	protein_coding	NM_005386
ASHGV40034262	-0.90415	0.904148546	protein_coding	NM_022842
ASHGV40016290	-0.9041	0.904098323	protein_coding	NM_022480
ASHGV40043544	0.904038	0.904038169	protein_coding	NM_004600
ASHGV40041164	-0.90398	0.903981434	protein_coding	NM_001017995
ASHGV40032407	-0.90385	0.903854028	protein_coding	ENST00000593412
ASHGV40012018	0.903809	0.903809209	protein_coding	NM_032848
ASHGV40053670	-0.90373	0.903733138	protein_coding	NM_207309
ASHGV40007074	0.903699	0.903699084	protein_coding	NM_001620
ASHGV40042339	0.903347	0.903346549	protein_coding	NM_001745
ASHGV40028137	0.903176	0.903176475	protein_coding	NM_003590
ASHGV40010551	-0.90302	0.90301501	protein_coding	NM_181724
ASHGV40014666	0.902925	0.902925331	protein_coding	NM_006556
ASHGV40009022	-0.9028	0.902797804	protein_coding	NM_001161630
ASHGV40017562	0.902693	0.902693057	protein_coding	NM_145253
ASHGV40043145	-0.90265	0.902654369	protein_coding	NM_003543
ASHGV40017845	-0.90248	0.902475588	protein_coding	NM_052838
ASHGV40049049	0.902144	0.902143796	protein_coding	NM_001283054
ASHGV40005141	0.902074	0.902073873	protein_coding	NM_145047
ASHGV40025031	-0.90205	0.902046809	protein_coding	NM_032607
ASHGV40034842	0.901982	0.901981851	protein_coding	NM_022776
ASHGV40023786	0.901911	0.901910523	protein_coding	NM_002096
ASHGV40045240	-0.90188	0.901882371	protein_coding	NM_021969
ASHGV40047503	-0.90183	0.901826027	protein_coding	NM_001278559
ASHGV40023169	-0.90178	0.901781184	protein_coding	uc002ksd.2
ASHGV40049416	-0.90176	0.90176339	protein_coding	ENST00000381466
ASHGV40053998	0.901726	0.901725739	protein_coding	ENST00000376441
ASHGV40021565	0.901686	0.9016856	protein_coding	NM_002815
ASHGV40026567	-0.90159	0.90159141	protein_coding	NM_000348
ASHGV40030676	-0.90149	0.90149232	protein_coding	NM_001898
ASHGV40048222	0.901463	0.901462798	protein_coding	NM_001103
ASHGV40015639	0.901195	0.901194578	protein_coding	NM_003257
ASHGV40008891	-0.90089	0.900887309	protein_coding	NM_138706
ASHGV40041777	0.900886	0.900885777	protein_coding	NM_022913
ASHGV40043406	0.900819	0.900819297	protein_coding	NM_152990
ASHGV40029744	-0.90054	0.900544299	protein_coding	NM_199204
ASHGV40060892	-0.9004	0.900395361	protein_coding	uc011lun.1
ASHGV40057021	-0.89992	0.89992068	protein_coding	NM_214711
ASHGV40019407	0.899858	0.89985816	protein_coding	uc010vmt.1
ASHGV40039257	-0.89968	0.899682108	protein_coding	NM_021227
ASHGV40043330	0.899665	0.899664915	protein_coding	NM_148919
ASHGV40027137	0.899644	0.89964376	protein_coding	NM_005783

ASHGV40037201	0.899522	0.899521785	protein_coding	NM_153376
ASHGV40033436	-0.89952	0.899517126	protein_coding	ENST00000434783
ASHGV40024108	0.899366	0.899366368	protein_coding	NM_023002
ASHGV40056022	-0.89931	0.899311513	protein_coding	NM_001007537
ASHGV40032207	0.899217	0.89921671	protein_coding	NM_017833
ASHGV40009908	0.899123	0.899122577	protein_coding	NM_053274
ASHGV40038862	-0.89876	0.898764366	protein_coding	NM_020846
ASHGV40006236	-0.8982	0.898202949	protein_coding	NM_001272046
ASHGV40046774	0.898051	0.898051052	protein_coding	NM_004912
ASHGV40046096	-0.89794	0.897939964	protein_coding	NM_032415
ASHGV40029008	0.89778	0.897779546	protein_coding	NM_014497
ASHGV40036737	0.897284	0.897283649	protein_coding	NM_024996
ASHGV40013825	-0.8969	0.896898307	protein_coding	NM_001126105
ASHGV40008572	-0.89685	0.896847455	protein_coding	NM_024771
ASHGV40039423	-0.89664	0.896641497	protein_coding	NM_031296
ASHGV40022363	0.896639	0.896638895	protein_coding	NM_024083
ASHGV40021825	0.896594	0.896594065	protein_coding	NM_005892
ASHGV40010070	0.896467	0.896467067	protein_coding	NM_005419
ASHGV40019183	0.896387	0.896387339	protein_coding	NM_032940
ASHGV40048032	0.896182	0.896182226	protein_coding	NM_005918
ASHGV40044729	-0.89602	0.896020399	protein_coding	NM_003531
ASHGV40049219	0.896017	0.896017086	protein_coding	NM_152568
ASHGV40027838	-0.89595	0.895954133	protein_coding	NM_022353
ASHGV40013591	-0.89593	0.895925657	protein_coding	ENST00000369157
ASHGV40029626	-0.89582	0.89581582	protein_coding	NM_177964
ASHGV40031033	-0.89514	0.89514396	protein_coding	NM_000782
ASHGV40028758	0.894812	0.894812139	protein_coding	NM_001145451
ASHGV40037319	0.894737	0.894736501	protein_coding	NM_018176
ASHGV40006553	-0.89472	0.894718899	protein_coding	NM_001004758
ASHGV40023419	-0.89461	0.894609286	protein_coding	NM_004852
ASHGV40055841	0.894342	0.894341561	protein_coding	NM_000770
ASHGV40057757	-0.89425	0.894247746	protein_coding	NM_000864
ASHGV40021024	-0.89397	0.893969538	protein_coding	NM_198082
ASHGV40048212	-0.89342	0.89341742	protein_coding	NM_145914
ASHGV40026107	-0.89341	0.893408599	protein_coding	NM_033106
ASHGV40029746	0.893352	0.89335225	protein_coding	NM_004792
ASHGV40041812	0.893192	0.893192445	protein_coding	NM_004520
ASHGV40035602	0.892901	0.892901309	protein_coding	NM_018462
ASHGV40017494	0.892881	0.89288122	protein_coding	NM_001919
ASHGV40031190	-0.89287	0.892865287	protein_coding	NM_139074
ASHGV40050238	-0.89281	0.892812327	protein_coding	NM_153332
ASHGV40034531	0.892804	0.892803516	protein_coding	NM_001278689
ASHGV40056498	-0.8925	0.892500042	protein_coding	ENST00000586380
ASHGV40026232	0.892367	0.89236745	protein_coding	NM_032880
ASHGV40011330	-0.89227	0.892269786	protein_coding	NM_152319
ASHGV40038040	-0.89201	0.892005553	protein_coding	NM_019035
ASHGV40019296	-0.892	0.891998758	protein_coding	NM_017803

ASHGV40024416	-0.89179	0.891790454	protein_coding	NM_030578
ASHGV40052280	-0.89164	0.891644575	protein_coding	NM_001004450
ASHGV40008733	0.891395	0.891395466	protein_coding	NM_001889
ASHGV40000442	-0.89091	0.890914871	protein_coding	NM_024782
ASHGV40045029	-0.89089	0.890890384	protein_coding	NM_198153
ASHGV40050464	-0.89083	0.890826029	protein_coding	NM_006867
ASHGV40047450	-0.89077	0.890768714	protein_coding	NM_002452
ASHGV40000701	-0.89075	0.890746781	protein_coding	ENST00000430695
ASHGV40057164	-0.89074	0.890743101	protein_coding	NM_020997
ASHGV40020890	0.890742	0.890742309	protein_coding	NM_052916
ASHGV40057605	-0.89057	0.890569813	protein_coding	NM_001098407
ASHGV40039766	0.890538	0.890538327	protein_coding	NM_015398
ASHGV40025935	-0.8903	0.89030122	protein_coding	NM_002691
ASHGV40048595	-0.89028	0.890278646	protein_coding	NM_176883
ASHGV40006613	-0.89025	0.89025448	protein_coding	NM_003700
ASHGV40001447	0.890223	0.890223255	protein_coding	NM_005175
ASHGV40038884	-0.89017	0.89016664	protein_coding	NM_023940
ASHGV40039302	-0.8901	0.890102134	protein_coding	ENST00000604093
ASHGV40021284	-0.89008	0.890078221	protein_coding	ENST00000299764
ASHGV40020121	-0.88942	0.889416462	protein_coding	NM_001045
ASHGV40025710	-0.88941	0.889408896	protein_coding	NM_004596
ASHGV40050027	0.889358	0.889358253	protein_coding	ENST00000303015
ASHGV40017364	-0.88933	0.88932876	protein_coding	NM_144598
ASHGV40016214	-0.88919	0.889188074	protein_coding	NM_006441
ASHGV40034896	-0.88918	0.889179865	protein_coding	NM_207307
ASHGV40049417	0.889066	0.889065748	protein_coding	NM_001080416
ASHGV40000182	-0.88891	0.888905464	protein_coding	ENST00000381051
ASHGV40048454	0.88889	0.888889909	protein_coding	NM_015328
ASHGV40056906	-0.88882	0.888816489	protein_coding	NM_001143833
ASHGV40043267	0.888709	0.888709403	protein_coding	NM_004639
ASHGV40041350	0.88855	0.888549827	protein_coding	NM_004553
ASHGV40022947	0.888484	0.888483645	protein_coding	NM_001044369
ASHGV40045645	-0.88841	0.888412731	protein_coding	NM_001012507
ASHGV40017617	-0.88816	0.88815949	protein_coding	NM_021247
ASHGV40000912	0.888078	0.888078261	protein_coding	NM_001198954
ASHGV40020051	-0.88808	0.888077036	protein_coding	NM_000625
ASHGV40013504	-0.88793	0.887934514	protein_coding	ENST00000369175
ASHGV40044456	-0.88764	0.887638306	protein_coding	NM_005618
ASHGV40036284	-0.88757	0.887569234	protein_coding	NM_018004
ASHGV40032116	-0.88745	0.88744654	protein_coding	NM_007038
ASHGV40038927	-0.88727	0.887267261	protein_coding	ENST00000598320
ASHGV40007624	0.887052	0.887052422	protein_coding	NM_014762
ASHGV40014730	-0.88694	0.886943789	protein_coding	NM_002937
ASHGV40024685	-0.8869	0.886897278	protein_coding	NM_002776
ASHGV40046985	0.886696	0.886696472	protein_coding	ENST00000413744
ASHGV40023752	-0.88662	0.886620683	protein_coding	NM_198317
ASHGV40005826	0.88659	0.886589915	protein_coding	NM_001174156

ASHGV40052541	0.886572	0.886571691	protein_coding	ENST00000344774
ASHGV40031790	-0.88637	0.886365104	protein_coding	NM_018431
ASHGV40016198	-0.88592	0.885922898	protein_coding	NM_000750
ASHGV40011596	0.885742	0.885741746	protein_coding	NM_015026
ASHGV40025172	-0.8856	0.885598216	protein_coding	NM_020428
ASHGV40026467	-0.8856	0.885595728	protein_coding	NM_004881
ASHGV40027678	0.885545	0.885545087	protein_coding	NM_004525
ASHGV40012601	-0.88551	0.885514749	protein_coding	NM_007015
ASHGV40009522	-0.88541	0.885406062	protein_coding	NM_005768
ASHGV40051679	0.885221	0.885221322	protein_coding	NM_001256053
ASHGV40012967	-0.88515	0.885145281	protein_coding	ENST00000538077
ASHGV40011008	-0.88513	0.885130701	protein_coding	NM_024865
ASHGV40018061	-0.88508	0.885084521	protein_coding	NM_033212
ASHGV40030001	-0.88499	0.884988926	protein_coding	NM_005214
ASHGV40057617	0.884746	0.884745716	protein_coding	NM_001007
ASHGV40037410	-0.88474	0.884743123	protein_coding	NM_004326
ASHGV40053830	0.884704	0.884704241	protein_coding	NM_001412
ASHGV40015630	0.884695	0.884694742	protein_coding	NM_004667
ASHGV40021909	-0.88417	0.884166084	protein_coding	NM_001242791
ASHGV40052606	0.883981	0.883980856	protein_coding	NM_004972
ASHGV40002682	-0.88391	0.883907613	protein_coding	ENST00000599994
ASHGV40007127	0.883782	0.883782392	protein_coding	NM_016404
ASHGV40031924	-0.88374	0.883738169	protein_coding	NM_022082
ASHGV40019283	0.883646	0.883645634	protein_coding	NM_016948
ASHGV40053678	-0.88358	0.883583586	protein_coding	NM_006088
ASHGV40011509	-0.88352	0.883523394	protein_coding	uc001shz.1
ASHGV40024987	-0.88346	0.88346163	protein_coding	NM_000479
ASHGV40036485	-0.88333	0.883330893	protein_coding	NM_032242
ASHGV40024417	-0.88326	0.883262143	protein_coding	NM_020158
ASHGV40005796	-0.8831	0.883102627	protein_coding	NM_015901
ASHGV40023976	-0.88307	0.883071381	protein_coding	uc002mxi.4
ASHGV40022215	0.882697	0.882696664	protein_coding	NM_015971
ASHGV40010278	0.882625	0.882625473	protein_coding	NM_007043
ASHGV40040835	-0.8826	0.882603328	protein_coding	ENST00000503143
ASHGV40031201	-0.88224	0.882239046	protein_coding	NM_021158
ASHGV40051080	0.882226	0.88222637	protein_coding	NM_004215
ASHGV40003352	-0.88218	0.882183484	protein_coding	NM_181684
ASHGV40029144	-0.88214	0.882142572	protein_coding	NM_198274
ASHGV40025390	-0.88203	0.882030529	protein_coding	NM_153221
ASHGV40045642	0.881955	0.88195479	protein_coding	NM_138571
ASHGV40055362	-0.88194	0.88194357	protein_coding	NM_002351
ASHGV40032820	0.881884	0.881883575	protein_coding	NM_001288707
ASHGV40022673	0.881815	0.881814621	protein_coding	NM_194281
ASHGV40019205	0.881808	0.881808007	protein_coding	NM_014157
ASHGV40015774	0.881616	0.881616452	protein_coding	NM_015497
ASHGV40039754	-0.88148	0.88148149	protein_coding	NM_001114357
ASHGV40024375	0.881461	0.881461183	protein_coding	NM_004877

ASHGV40035823	0.881383	0.881383265	protein_coding	NM_015460
ASHGV40043017	-0.88117	0.881174944	protein_coding	NM_006403
ASHGV40017540	0.880991	0.880990711	protein_coding	NM_001284527
ASHGV40021940	0.880969	0.880969376	protein_coding	NM_006107
ASHGV40048992	0.880951	0.880950701	protein_coding	NM_014759
ASHGV40021757	0.880905	0.880904735	protein_coding	NM_005789
ASHGV40018841	-0.88066	0.880662549	protein_coding	NM_001169
ASHGV40012300	0.880438	0.880437919	protein_coding	NM_001042414
ASHGV40006187	0.880407	0.8804066	protein_coding	NM_005962
ASHGV40047684	-0.88026	0.880255914	protein_coding	NM_194300
ASHGV40045218	0.87988	0.879879571	protein_coding	NM_001190706
ASHGV40012278	-0.87986	0.879857312	protein_coding	NM_001170543
ASHGV40021991	0.879756	0.879756005	protein_coding	NM_003647
ASHGV40056552	-0.87958	0.879584884	protein_coding	NM_005267
ASHGV40057681	0.879581	0.879580742	protein_coding	NM_020364
ASHGV40035896	-0.87954	0.879542841	protein_coding	NM_001123041
ASHGV40025064	0.879491	0.879490535	protein_coding	NM_015414
ASHGV40010874	0.879248	0.879247581	protein_coding	NM_001142551
ASHGV40000412	-0.87908	0.879082669	protein_coding	ENST00000416839
ASHGV40023795	-0.87898	0.878982918	protein_coding	NM_001252
ASHGV40055236	0.87878	0.878779908	protein_coding	NM_024863
ASHGV40037737	-0.87855	0.878545512	protein_coding	NM_197965
ASHGV40049227	-0.87841	0.87840567	protein_coding	NM_000930
ASHGV40013182	-0.87798	0.877977873	protein_coding	NM_000059
ASHGV40047480	0.877941	0.877941398	protein_coding	NM_015610
ASHGV40048624	0.877668	0.877667669	protein_coding	NM_003592
ASHGV40028675	-0.87764	0.877643456	protein_coding	NM_199280
ASHGV40033841	-0.87737	0.877372443	protein_coding	NM_001996
ASHGV40048582	-0.87726	0.877261227	protein_coding	NM_002652
ASHGV40010010	0.877163	0.877162892	protein_coding	NM_006301
ASHGV40024565	-0.87712	0.877116454	protein_coding	NM_016457
ASHGV40057647	0.876936	0.876936116	protein_coding	NM_001017436
ASHGV40028051	0.87686	0.876859739	protein_coding	NM_022648
ASHGV40003168	0.876822	0.876822275	protein_coding	NM_016282
ASHGV40017229	0.876764	0.876763633	protein_coding	NM_022839
ASHGV40013347	-0.87667	0.87667054	protein_coding	NM_052950
ASHGV40056298	-0.8766	0.876598724	protein_coding	ENST00000544589
ASHGV40037492	0.87651	0.876510058	protein_coding	NM_022832
ASHGV40007751	-0.8765	0.876502969	protein_coding	NM_001098169
ASHGV40008735	-0.87648	0.8764756	protein_coding	NM_006019
ASHGV40044047	-0.87632	0.876320478	protein_coding	NM_005298
ASHGV40035985	-0.87616	0.876163617	protein_coding	NM_203424
ASHGV40020241	-0.87615	0.876149404	protein_coding	ENST00000595377
ASHGV40029019	0.876071	0.876070957	protein_coding	NM_006429
ASHGV40040166	-0.8759	0.875896508	protein_coding	ENST00000504970
ASHGV40054116	-0.87575	0.875754524	protein_coding	NM_001003811
ASHGV40039596	0.87555	0.87554953	protein_coding	NM_007246

ASHGV40017119	-0.87552	0.875518003	protein_coding	ENST00000599596
ASHGV40011938	-0.87546	0.875456805	protein_coding	ENST00000547081
ASHGV40006555	-0.87528	0.875279218	protein_coding	NM_001005238
ASHGV40028972	0.875235	0.875235315	protein_coding	NM_014882
ASHGV40025369	0.8751	0.875100426	protein_coding	NM_012109
ASHGV40032150	-0.87496	0.874963339	protein_coding	NM_203405
ASHGV40018459	-0.87496	0.874960531	protein_coding	NM_145039
ASHGV40022634	-0.87484	0.874835611	protein_coding	NM_024421
ASHGV40028050	-0.87469	0.874693304	protein_coding	ENST00000423123
ASHGV40022671	-0.87462	0.874619299	protein_coding	NM_001004694
ASHGV40054789	0.874335	0.874334761	protein_coding	NM_002365
ASHGV40023279	-0.87425	0.874249193	protein_coding	NM_016271
ASHGV40041161	-0.87424	0.87424155	protein_coding	NM_005990
ASHGV40044588	-0.87406	0.874060054	protein_coding	NM_001040274
ASHGV40039281	-0.87395	0.873950808	protein_coding	NM_025144
ASHGV40038487	0.873932	0.873932299	protein_coding	NM_001119
ASHGV40041468	0.873882	0.873882196	protein_coding	NM_005885
ASHGV40043923	0.873833	0.873832815	protein_coding	NM_007214
ASHGV40024418	-0.87383	0.873828143	protein_coding	NM_198540
ASHGV40045740	0.873791	0.873791462	protein_coding	NM_020340
ASHGV40030887	-0.87378	0.873775163	protein_coding	NM_003064
ASHGV40041391	-0.87374	0.873736975	protein_coding	NM_024337
ASHGV40032167	-0.87371	0.873706428	protein_coding	NM_181619
ASHGV40003285	-0.8737	0.873700481	protein_coding	NM_015585
ASHGV40029519	0.87369	0.873690262	protein_coding	NM_015320
ASHGV40018142	-0.87368	0.873675104	protein_coding	NM_004062
ASHGV40040746	-0.87363	0.873633207	protein_coding	NM_020747
ASHGV40056154	0.873561	0.873560648	protein_coding	NM_002487
ASHGV40038372	0.873431	0.873430891	protein_coding	NM_021069
ASHGV40026851	0.873389	0.873389286	protein_coding	NM_004161
ASHGV40031197	-0.87333	0.873334323	protein_coding	NM_006943
ASHGV40010920	0.872913	0.872913427	protein_coding	NM_002790
ASHGV40007256	-0.87279	0.872785395	protein_coding	NM_198923
ASHGV40032435	0.872665	0.87266451	protein_coding	NM_144770
ASHGV40040873	0.87266	0.872659889	protein_coding	NM_004134
ASHGV40044848	-0.87233	0.872330136	protein_coding	NM_001010909
ASHGV40044439	0.872214	0.872213838	protein_coding	NM_002481
ASHGV40014317	-0.87221	0.872212417	protein_coding	ENST00000553510
ASHGV40045664	-0.87205	0.872049893	protein_coding	NM_005021
ASHGV40045271	-0.87193	0.871926892	protein_coding	NM_133493
ASHGV40042514	0.871654	0.871653545	protein_coding	NM_006706
ASHGV40051675	-0.87158	0.871578552	protein_coding	NM_194313
ASHGV40003084	0.871323	0.871323444	protein_coding	NM_001017998
ASHGV40037316	0.871298	0.871298308	protein_coding	NM_001358
ASHGV40006185	0.87124	0.87123962	protein_coding	NM_016824
ASHGV40039138	-0.87123	0.871231719	protein_coding	NM_004407
ASHGV40034452	-0.87113	0.871132989	protein_coding	NM_017563

ASHGV40003234	0.870708	0.870707691	protein_coding	NM_018283
ASHGV40040767	-0.87068	0.870681647	protein_coding	NM_178450
ASHGV40026819	0.870453	0.870452627	protein_coding	NM_006430
ASHGV40036974	-0.87023	0.870227343	protein_coding	NM_006580
ASHGV40050028	-0.87011	0.870113484	protein_coding	NM_001160372
ASHGV40017867	-0.87002	0.870018942	protein_coding	NM_002773
ASHGV40005356	0.869808	0.869807585	protein_coding	NM_003380
ASHGV40030824	0.869803	0.869802992	protein_coding	NM_014657
ASHGV40024664	-0.86933	0.869334784	protein_coding	NM_004851
ASHGV40028510	-0.86907	0.869071127	protein_coding	NM_014668
ASHGV40018586	-0.86898	0.86898263	protein_coding	uc002cqw.1
ASHGV40045012	-0.86897	0.868972608	protein_coding	ENST00000598338
ASHGV40043116	0.868841	0.868840615	protein_coding	NM_030939
ASHGV40006313	-0.86878	0.868784922	protein_coding	NM_001099667
ASHGV40013867	0.86824	0.868240091	protein_coding	ENST00000609024
ASHGV40052982	-0.8682	0.868200442	protein_coding	NM_001490
ASHGV40022188	-0.86779	0.867788446	protein_coding	NM_181790
ASHGV40001691	0.867773	0.867773364	protein_coding	NM_014175
ASHGV40009883	-0.86772	0.867720999	protein_coding	NM_181847
ASHGV40041888	0.867676	0.867676393	protein_coding	NM_005909
ASHGV40018428	-0.86766	0.86765501	protein_coding	NM_178841
ASHGV40055377	-0.86755	0.867545428	protein_coding	NM_003399
ASHGV40034044	0.86753	0.867530348	protein_coding	NM_004844
ASHGV40020766	0.866984	0.866983776	protein_coding	NM_007168
ASHGV40049150	-0.86698	0.866979924	protein_coding	NM_001002814
ASHGV40007612	-0.86694	0.866938097	protein_coding	NM_207429
ASHGV40027418	0.866909	0.866908551	protein_coding	NM_032357
ASHGV40040417	0.866615	0.866614794	protein_coding	NM_018268
ASHGV40021732	-0.86661	0.866605093	protein_coding	NM_031421
ASHGV40037664	-0.8666	0.866601985	protein_coding	NM_001042784
ASHGV40016775	0.866528	0.866527543	protein_coding	NM_001145668
ASHGV40024652	0.866508	0.866508297	protein_coding	NM_001098632
ASHGV40018482	0.866183	0.866183173	protein_coding	NM_001176
ASHGV40028982	0.866053	0.866052757	protein_coding	NM_001153
ASHGV40056207	-0.86595	0.865950086	protein_coding	ENST00000561062
ASHGV40031376	-0.86594	0.865940287	protein_coding	NM_020689
ASHGV40032369	-0.86585	0.865845799	protein_coding	NM_198696
ASHGV40024960	0.865714	0.865713622	protein_coding	NM_032853
ASHGV40023711	0.865676	0.865675899	protein_coding	NM_145173
ASHGV40032571	-0.86551	0.865508263	protein_coding	NM_181620
ASHGV40024925	0.865436	0.86543635	protein_coding	NM_001194
ASHGV40057591	-0.86538	0.86537673	protein_coding	NM_001277307
ASHGV40000051	-0.86522	0.865221147	protein_coding	ENST00000314040
ASHGV40022661	-0.86482	0.864822226	protein_coding	NM_001310134
ASHGV40020706	-0.86459	0.864588942	protein_coding	NM_001433
ASHGV40060867	0.864383	0.864382998	protein_coding	uc010kak.3
ASHGV40021215	-0.86422	0.864221461	protein_coding	NM_175734

ASHGV40052787	-0.86395	0.863945926	protein_coding	NM_012266
ASHGV40041018	0.863858	0.863857994	protein_coding	NM_015621
ASHGV40016491	-0.86371	0.863708543	protein_coding	NM_001001413
ASHGV40026607	0.863628	0.863627686	protein_coding	NM_019024
ASHGV40009136	0.863324	0.863323617	protein_coding	NM_000317
ASHGV40045266	0.863297	0.86329663	protein_coding	NM_133645
ASHGV40018871	-0.86329	0.863291449	protein_coding	NM_001024401
ASHGV40036227	-0.86323	0.863227741	protein_coding	NM_022072
ASHGV40053827	0.863105	0.863105251	protein_coding	NM_031892
ASHGV40052070	0.863103	0.863102938	protein_coding	NM_016481
ASHGV40036992	0.862812	0.862812305	protein_coding	NM_020386
ASHGV40022875	0.862741	0.862741261	protein_coding	NM_002035
ASHGV40017601	-0.86258	0.862578248	protein_coding	ENST00000561538
ASHGV40053874	-0.86211	0.862109887	protein_coding	NM_000475
ASHGV40048042	0.861707	0.861707057	protein_coding	NM_001037277
ASHGV40001345	-0.86159	0.861589754	protein_coding	NM_005090
ASHGV40044193	0.861512	0.861511539	protein_coding	NM_006734
ASHGV40009566	-0.86145	0.861452572	protein_coding	NM_002258
ASHGV40032929	-0.86133	0.861328908	protein_coding	NM_001002862
ASHGV40006444	-0.86126	0.861258948	protein_coding	NM_138384
ASHGV40032356	0.860942	0.86094176	protein_coding	NM_015259
ASHGV40018461	0.860821	0.860821219	protein_coding	NM_001042610
ASHGV40007341	0.860818	0.860818475	protein_coding	NM_001286577
ASHGV40023094	-0.86068	0.860677222	protein_coding	ENST00000584361
ASHGV40042706	0.860581	0.860581419	protein_coding	NM_001034837
ASHGV40018487	-0.86021	0.860214627	protein_coding	NM_005009
ASHGV40021303	-0.86019	0.860185479	protein_coding	NM_001372
ASHGV40002643	-0.86012	0.860121184	protein_coding	ENST00000597410
ASHGV40009512	-0.85988	0.859881902	protein_coding	NM_032489
ASHGV40017265	-0.85984	0.85983968	protein_coding	NM_022769
ASHGV40041430	0.859769	0.859768744	protein_coding	NM_001145161
ASHGV40031357	-0.85976	0.859763694	protein_coding	NM_052865
ASHGV40003283	-0.85945	0.859454652	protein_coding	NM_015421
ASHGV40014568	0.859427	0.859427488	protein_coding	NM_004184
ASHGV40006421	-0.8594	0.859396208	protein_coding	NM_153274
ASHGV40050589	0.859352	0.859351647	protein_coding	NM_138436
ASHGV40042070	0.859251	0.859251003	protein_coding	NM_014899
ASHGV40007690	-0.85891	0.858912084	protein_coding	NM_001206789
ASHGV40038796	0.858904	0.858904043	protein_coding	NM_175737
ASHGV40016399	-0.8587	0.858695456	protein_coding	ENST00000378936
ASHGV40050520	-0.85866	0.858658706	protein_coding	NM_032777
ASHGV40034049	-0.85862	0.858623717	protein_coding	NM_005677
ASHGV40020329	-0.85855	0.858546471	protein_coding	NM_000226
ASHGV40023468	0.858527	0.858527062	protein_coding	NM_020854
ASHGV40035429	0.858489	0.858488894	protein_coding	NM_021032
ASHGV40002039	-0.85839	0.85838959	protein_coding	ENST00000549424
ASHGV40029985	-0.85835	0.858346547	protein_coding	NM_139158

ASHGV40002191	0.858283	0.858282614	protein_coding	NM_013309
ASHGV40023544	0.858204	0.85820448	protein_coding	NM_014177
ASHGV40028610	-0.85808	0.858078598	protein_coding	NM_152493
ASHGV40010029	-0.85796	0.857959879	protein_coding	NM_020370
ASHGV40024075	-0.85783	0.857829564	protein_coding	NM_005543
ASHGV40038279	-0.85762	0.857620916	protein_coding	NM_021973
ASHGV40019991	-0.85757	0.857571829	protein_coding	NM_002404
ASHGV40009148	0.857464	0.857463711	protein_coding	NM_178510
ASHGV40016797	0.857442	0.857442113	protein_coding	NM_005154
ASHGV40035334	0.857441	0.857440572	protein_coding	NM_006496
ASHGV40013163	-0.85744	0.857439743	protein_coding	NM_032849
ASHGV40027852	0.857415	0.857415133	protein_coding	NM_007315
ASHGV40044390	0.857315	0.857314974	protein_coding	NM_003730
ASHGV40022184	0.857245	0.857245431	protein_coding	NM_032646
ASHGV40045446	0.857223	0.85722329	protein_coding	NM_021956
ASHGV40024687	-0.85711	0.857110211	protein_coding	NM_145894
ASHGV40006133	-0.85694	0.856936966	protein_coding	NM_178858
ASHGV40054319	-0.85687	0.856871792	protein_coding	NM_015365
ASHGV40011402	0.856675	0.856674848	protein_coding	NM_021934
ASHGV40041727	-0.85661	0.856613571	protein_coding	NM_181501
ASHGV40053297	0.856607	0.85660739	protein_coding	NM_007203
ASHGV40025681	-0.85658	0.856575005	protein_coding	ENST00000396843
ASHGV40034342	-0.8565	0.856501348	protein_coding	NM_001080528
ASHGV40018192	-0.85635	0.856352777	protein_coding	NM_022341
ASHGV40042298	-0.8561	0.856101199	protein_coding	NM_003687
ASHGV40021094	0.85608	0.85607984	protein_coding	NM_002615
ASHGV40055232	0.856022	0.856021673	protein_coding	NM_016303
ASHGV40039013	-0.85586	0.855856924	protein_coding	NM_152997
ASHGV40041215	-0.85559	0.855591248	protein_coding	NM_014901
ASHGV40034026	0.855572	0.855571565	protein_coding	NM_001098502
ASHGV40017561	0.855434	0.855434496	protein_coding	NM_013399
ASHGV40034893	-0.85538	0.855377358	protein_coding	NM_006026
ASHGV40034762	-0.85534	0.855335095	protein_coding	NM_018338
ASHGV40045891	0.855299	0.855299337	protein_coding	NM_020245
ASHGV40050438	0.855256	0.855256204	protein_coding	NM_017412
ASHGV40028329	-0.85489	0.854891953	protein_coding	NM_001085437
ASHGV40025671	-0.85484	0.854844734	protein_coding	NM_001004318
ASHGV40034257	0.854804	0.854804011	protein_coding	NM_020696
ASHGV40008815	-0.85462	0.854622324	protein_coding	ENST00000442948
ASHGV40010113	0.854606	0.854605841	protein_coding	NM_000785
ASHGV40017544	-0.85411	0.854114061	protein_coding	NM_178844
ASHGV40009372	-0.85404	0.854038361	protein_coding	NM_021978
ASHGV40028744	0.853967	0.853966641	protein_coding	NM_001170791
ASHGV40049683	0.853791	0.853790543	protein_coding	NM_024759
ASHGV40025586	0.853577	0.853576736	protein_coding	NM_001863
ASHGV40008130	-0.85357	0.853574762	protein_coding	NM_000728
ASHGV40043083	0.853522	0.853522097	protein_coding	NM_000367

ASHGV40057599	-0.85342	0.853418108	protein_coding	NM_001099921
ASHGV40003333	-0.85338	0.853377396	protein_coding	NM_133497
ASHGV40054618	-0.85336	0.853358011	protein_coding	NM_004043
ASHGV40054247	-0.85296	0.852963632	protein_coding	NM_019007
ASHGV40032693	-0.8529	0.852900773	protein_coding	NM_138991
ASHGV40026443	-0.85287	0.852868084	protein_coding	NM_021925
ASHGV40031745	-0.85284	0.852843583	protein_coding	NM_015266
ASHGV40011042	-0.85262	0.852617138	protein_coding	NM_013269
ASHGV40043434	-0.85243	0.852427753	protein_coding	NM_130782
ASHGV40046196	0.852269	0.852269338	protein_coding	NM_001101426
ASHGV40025828	-0.85208	0.852076089	protein_coding	NM_198541
ASHGV40021207	-0.85206	0.852063009	protein_coding	NM_001042
ASHGV40020598	0.851591	0.851590759	protein_coding	NM_005082
ASHGV40057081	-0.85143	0.851426636	protein_coding	NM_178569
ASHGV40050352	0.851262	0.85126192	protein_coding	NM_001978
ASHGV40030797	0.850857	0.850857431	protein_coding	NM_184234
ASHGV40047501	0.850607	0.850606548	protein_coding	NM_024067
ASHGV40010880	0.850446	0.850446168	protein_coding	uc010scx.1
ASHGV40027765	-0.8503	0.850295434	protein_coding	NM_181342
ASHGV40014782	-0.85022	0.850221421	protein_coding	NM_001282322
ASHGV40047936	0.850029	0.850029378	protein_coding	NM_001300845
ASHGV40025865	-0.84994	0.849941747	protein_coding	NM_001425
ASHGV40016132	0.849869	0.849869316	protein_coding	NM_032907
ASHGV40029548	0.849772	0.849772491	protein_coding	NM_014607
ASHGV40002667	0.849722	0.849721649	protein_coding	ENST00000598920
ASHGV40026272	-0.84969	0.849690308	protein_coding	NM_002584
ASHGV40022034	-0.84951	0.849512755	protein_coding	NM_018304
ASHGV40031712	-0.84949	0.849492726	protein_coding	NM_005244
ASHGV40030401	-0.84936	0.849363493	protein_coding	uc002wbj.3
ASHGV40057224	-0.84924	0.849243304	protein_coding	NM_003192
ASHGV40014468	0.849146	0.84914648	protein_coding	NM_004515
ASHGV40007435	0.84912	0.849119827	protein_coding	NM_007166
ASHGV40029475	-0.84905	0.849047621	protein_coding	NM_000312
ASHGV40053293	0.849022	0.849021685	protein_coding	NM_017832
ASHGV40002388	-0.84879	0.848788712	protein_coding	NM_004112
ASHGV40011985	0.848771	0.848770822	protein_coding	NM_013300
ASHGV40051728	-0.84873	0.848733543	protein_coding	NM_014872
ASHGV40042268	0.848541	0.848541276	protein_coding	NM_032177
ASHGV40020967	-0.84854	0.848537437	protein_coding	NM_001287262
ASHGV40043234	0.848417	0.848416507	protein_coding	NM_003587
ASHGV40039481	0.848401	0.848401339	protein_coding	NM_018241
ASHGV40042345	-0.84831	0.848311368	protein_coding	NM_001277348
ASHGV40051824	0.847819	0.847819299	protein_coding	NM_001099666
ASHGV40018541	-0.84778	0.847783876	protein_coding	NM_020825
ASHGV40042924	0.847451	0.847450679	protein_coding	NM_004155
ASHGV40003221	-0.84726	0.847263446	protein_coding	NM_001290053
ASHGV40044748	-0.84708	0.847082117	protein_coding	NM_007048

ASHGV40009844	0.846979	0.846978708	protein_coding	NM_005748
ASHGV40021234	-0.84698	0.846975496	protein_coding	NM_018081
ASHGV40036211	0.846953	0.846953217	protein_coding	NM_018293
ASHGV40024617	-0.84669	0.846685944	protein_coding	NM_020904
ASHGV40028840	0.84666	0.846660315	protein_coding	NM_001135629
ASHGV40016245	-0.84665	0.846646649	protein_coding	ENST00000559949
ASHGV40036367	0.846557	0.846556701	protein_coding	NM_017945
ASHGV40011135	-0.84613	0.846126327	protein_coding	NM_000921
ASHGV40048620	0.845937	0.845937242	protein_coding	NM_014141
ASHGV40025733	-0.84585	0.845847878	protein_coding	NM_033543
ASHGV40010337	0.84577	0.845770187	protein_coding	NM_182767
ASHGV40021913	-0.8457	0.845701198	protein_coding	NM_002204
ASHGV40051930	-0.84552	0.845524685	protein_coding	NM_002048
ASHGV40007560	-0.84551	0.845507553	protein_coding	NM_001017534
ASHGV40002322	-0.84547	0.845465522	protein_coding	ENST00000567078
ASHGV40031234	-0.84532	0.845324829	protein_coding	NM_080751
ASHGV40029301	-0.84531	0.845312894	protein_coding	NM_021815
ASHGV40051702	-0.84501	0.845005082	protein_coding	NM_014450
ASHGV40012315	-0.845	0.844996341	protein_coding	NM_022459
ASHGV40034408	0.844946	0.844946262	protein_coding	NM_004656
ASHGV40016879	-0.84489	0.844887936	protein_coding	NM_033195
ASHGV40032586	0.844884	0.844883655	protein_coding	ENST00000534991
ASHGV40026128	-0.84464	0.84463865	protein_coding	NM_003417
ASHGV40016163	0.844619	0.844619264	protein_coding	NM_138573
ASHGV40023289	-0.84456	0.844561641	protein_coding	NM_030632
ASHGV40049697	-0.84448	0.844478961	protein_coding	NM_152628
ASHGV40015656	-0.84443	0.844433144	protein_coding	NM_001184714
ASHGV40025869	-0.84423	0.844227909	protein_coding	NM_013348
ASHGV40055049	-0.84415	0.844145619	protein_coding	NM_004429
ASHGV40035598	0.844078	0.844077941	protein_coding	NM_001031717
ASHGV40026987	-0.84406	0.844060926	protein_coding	NM_006507
ASHGV40014663	0.844022	0.844021764	protein_coding	NM_001025107
ASHGV40014151	-0.84401	0.844013575	protein_coding	NM_005982
ASHGV40042427	-0.84396	0.843955427	protein_coding	NM_018904
ASHGV40037207	-0.84381	0.84381215	protein_coding	NM_198595
ASHGV40007339	-0.84362	0.84361983	protein_coding	NM_003356
ASHGV40003199	-0.84343	0.843429834	protein_coding	NM_004906
ASHGV40055890	0.843369	0.843369213	protein_coding	NM_021828
ASHGV40014501	0.843203	0.843203289	protein_coding	NM_024734
ASHGV40022394	-0.84319	0.843192368	protein_coding	ENST00000308911
ASHGV40042782	-0.84316	0.84315598	protein_coding	NM_017675
ASHGV40039170	-0.84311	0.843112867	protein_coding	NM_005172
ASHGV40022829	0.843097	0.843096846	protein_coding	NM_000140
ASHGV40016003	-0.8429	0.842902155	protein_coding	NM_016563
ASHGV40020288	-0.84289	0.84289207	protein_coding	NM_181537
ASHGV40042331	0.842783	0.842782935	protein_coding	NM_003337
ASHGV40031389	-0.84278	0.842782038	protein_coding	NM_000792

ASHGV40015841	-0.84273	0.842730907	protein_coding	NM_152647
ASHGV40025858	-0.84268	0.842683863	protein_coding	NM_022142
ASHGV40051524	0.842387	0.842387253	protein_coding	NM_003829
ASHGV40036923	0.842351	0.842351373	protein_coding	NM_004721
ASHGV40054426	0.842227	0.842226575	protein_coding	NM_001555
ASHGV40023797	-0.84211	0.842109307	protein_coding	NM_003807
ASHGV40025978	-0.84204	0.84204227	protein_coding	NM_001462
ASHGV40009899	0.841973	0.841972842	protein_coding	NM_024095
ASHGV40046080	0.84191	0.841910125	protein_coding	NM_032302
ASHGV40023852	0.841818	0.841817502	protein_coding	NM_080605
ASHGV40045622	-0.84168	0.841679392	protein_coding	NM_032471
ASHGV40044839	0.841266	0.841265634	protein_coding	NM_001109938
ASHGV40027436	0.840922	0.840922283	protein_coding	NM_031286
ASHGV40016950	0.84084	0.840840401	protein_coding	NM_004319
ASHGV40016991	-0.84081	0.840806509	protein_coding	NM_017705
ASHGV40008452	0.840594	0.840593706	protein_coding	NM_015959
ASHGV40028682	0.84044	0.840440333	protein_coding	NM_016061
ASHGV40011088	-0.84028	0.840278409	protein_coding	NM_001423
ASHGV40046747	0.840161	0.840161293	protein_coding	NM_024315
ASHGV40008820	-0.84012	0.840116406	protein_coding	NM_000803
ASHGV40028082	-0.84001	0.840006703	protein_coding	NM_024506
ASHGV40055334	-0.8399	0.839899816	protein_coding	NM_178813
ASHGV40007872	0.839866	0.839865933	protein_coding	NM_014174
ASHGV40026888	-0.8397	0.839696867	protein_coding	NM_182536
ASHGV40031380	-0.83945	0.839453891	protein_coding	NM_002196
ASHGV40057604	-0.83914	0.839140201	protein_coding	NM_181532
ASHGV40008734	0.83912	0.839120453	protein_coding	NM_002496
ASHGV40017036	-0.83898	0.838979958	protein_coding	NM_002499
ASHGV40046864	0.838278	0.838277606	protein_coding	NM_002319
ASHGV40027618	-0.83822	0.838220272	protein_coding	NM_014880
ASHGV40036225	-0.83801	0.838008346	protein_coding	NM_182896
ASHGV40039267	-0.838	0.837995584	protein_coding	NM_001963
ASHGV40032788	-0.83794	0.83793984	protein_coding	NM_001848
ASHGV40027729	-0.83792	0.837921681	protein_coding	NM_001039523
ASHGV40033446	0.837898	0.837897935	protein_coding	NM_005207
ASHGV40000466	-0.83785	0.837848595	protein_coding	NM_001004323
ASHGV40031178	0.837782	0.837781571	protein_coding	NM_018419
ASHGV40007701	-0.83778	0.83777777	protein_coding	NM_000732
ASHGV40016732	0.837774	0.83777392	protein_coding	NM_001018108
ASHGV40017741	-0.8376	0.837595863	protein_coding	NM_153603
ASHGV40046029	0.837307	0.837306707	protein_coding	uc010kky.1
ASHGV40030736	-0.83721	0.837206965	protein_coding	NM_001145474
ASHGV40027127	0.837171	0.8371711	protein_coding	NM_012214
ASHGV40015897	-0.83699	0.836987385	protein_coding	NM_130810
ASHGV40023107	0.836891	0.836891358	protein_coding	NM_002845
ASHGV40024716	-0.83676	0.836756067	protein_coding	NM_023074
ASHGV40023830	0.836617	0.836616618	protein_coding	NM_005001

ASHGV40019742	-0.83653	0.836534689	protein_coding	NM_017986
ASHGV40046062	-0.83566	0.835657544	protein_coding	NM_001031617
ASHGV40030590	-0.83559	0.83558856	protein_coding	uc021way.1
ASHGV40057562	-0.83528	0.8352774	protein_coding	NM_001099270
ASHGV40008518	-0.835	0.835000205	protein_coding	NM_001173990
ASHGV40007152	-0.8347	0.834703313	protein_coding	NM_001282448
ASHGV40046057	0.834697	0.83469701	protein_coding	NM_002735
ASHGV40024878	-0.83449	0.834485164	protein_coding	NM_024762
ASHGV40002591	-0.83405	0.834045638	protein_coding	NM_003121
ASHGV40042272	-0.834	0.833995964	protein_coding	NM_032126
ASHGV40049168	-0.83393	0.833934909	protein_coding	NM_207412
ASHGV40037450	0.833802	0.833802399	protein_coding	NM_198353
ASHGV40036356	-0.83373	0.833729957	protein_coding	NM_001185106
ASHGV40053832	0.833606	0.833606265	protein_coding	NM_004586
ASHGV40031525	-0.83354	0.833543559	protein_coding	NM_001672
ASHGV40025737	-0.83354	0.833541333	protein_coding	NM_001040283
ASHGV40035238	0.833533	0.833533014	protein_coding	NM_015028
ASHGV40031964	0.833521	0.833520762	protein_coding	NM_025219
ASHGV40015433	0.833502	0.833501657	protein_coding	NM_003403
ASHGV40042084	-0.83341	0.833407305	protein_coding	NM_022093
ASHGV40056967	-0.83335	0.833352573	protein_coding	NM_018192
ASHGV40033476	-0.83317	0.8331735	protein_coding	NM_021233
ASHGV40025199	-0.83316	0.833157832	protein_coding	NM_152356
ASHGV40036039	0.833154	0.833153601	protein_coding	NM_020676
ASHGV40042484	0.833132	0.833132406	protein_coding	NM_004290
ASHGV40032782	-0.83312	0.833118151	protein_coding	NM_020528
ASHGV40044270	-0.83309	0.833092804	protein_coding	NM_012177
ASHGV40054297	-0.83295	0.832947353	protein_coding	NM_024657
ASHGV40018367	-0.83288	0.832882072	protein_coding	NM_016095
ASHGV40048509	0.832857	0.832856955	protein_coding	NM_033138
ASHGV40053723	-0.83281	0.832812264	protein_coding	NM_015419
ASHGV40041108	0.832779	0.832778579	protein_coding	NM_145266
ASHGV40027641	0.832519	0.832519107	protein_coding	NM_033272
ASHGV40003365	0.832385	0.832385071	protein_coding	NM_207007
ASHGV40009778	0.832151	0.83215063	protein_coding	NM_006390
ASHGV40051673	-0.83183	0.831829543	protein_coding	NM_015397
ASHGV40008623	0.831819	0.831818829	protein_coding	NM_030816
ASHGV40006747	0.831808	0.831808212	protein_coding	NM_014774
ASHGV40031650	-0.83178	0.831784968	protein_coding	NM_178491
ASHGV40014049	0.831528	0.831528148	protein_coding	NM_006575
ASHGV40057255	0.831422	0.831421531	protein_coding	NM_000165
ASHGV40042616	-0.8313	0.831303546	protein_coding	NM_173491
ASHGV40007089	-0.8313	0.831301354	protein_coding	NM_024099
ASHGV40055906	-0.83102	0.831021081	protein_coding	NM_015490
ASHGV40019825	-0.83099	0.830992115	protein_coding	NM_201520
ASHGV40024607	0.830789	0.830788691	protein_coding	NM_001352
ASHGV40002510	-0.83069	0.830689912	protein_coding	ENST00000586572

ASHGV40049965	-0.83037	0.830374744	protein_coding	NM_001354
ASHGV40007191	-0.83032	0.830315605	protein_coding	NM_033036
ASHGV40003087	-0.83008	0.830076463	protein_coding	NM_001019
ASHGV40024374	0.829982	0.829982288	protein_coding	NM_020862
ASHGV40019360	0.829714	0.829714423	protein_coding	NM_014761
ASHGV40010214	-0.8296	0.82959905	protein_coding	NM_020525
ASHGV40008801	-0.82956	0.829556929	protein_coding	NM_001012503
ASHGV40038439	0.829365	0.829365414	protein_coding	NM_001127178
ASHGV40033816	0.829364	0.829363876	protein_coding	NM_015380
ASHGV40007375	0.829336	0.829336155	protein_coding	NM_004705
ASHGV40022210	0.829271	0.829270842	protein_coding	NM_015353
ASHGV40003272	-0.82922	0.829223754	protein_coding	NM_005672
ASHGV40024341	-0.8292	0.829203209	protein_coding	NM_001291088
ASHGV40054862	0.829191	0.829191174	protein_coding	NM_005300
ASHGV40057849	0.829088	0.829088044	protein_coding	NM_001294335
ASHGV40024522	-0.82905	0.829049187	protein_coding	NM_006663
ASHGV40053655	0.82902	0.829020383	protein_coding	NM_001080482
ASHGV40006548	0.829016	0.82901553	protein_coding	NM_144663
ASHGV40025853	-0.82883	0.828829062	protein_coding	NM_015711
ASHGV40054449	-0.82876	0.828759719	protein_coding	NM_019556
ASHGV40054038	0.828663	0.828663264	protein_coding	NM_031407
ASHGV40037601	0.828509	0.828509166	protein_coding	NM_014465
ASHGV40044990	0.828098	0.828098332	protein_coding	NM_003958
ASHGV40006639	0.827948	0.827948134	protein_coding	NM_020644
ASHGV40051537	0.827771	0.827771192	protein_coding	NM_033222
ASHGV40029997	-0.82759	0.827593338	protein_coding	NM_177538
ASHGV40046329	0.827533	0.827533311	protein_coding	NM_024051
ASHGV40041055	0.827523	0.82752315	protein_coding	NM_004270
ASHGV40042790	0.827501	0.827501186	protein_coding	NM_133369
ASHGV40055956	0.827361	0.827360906	protein_coding	NM_014631
ASHGV40041246	-0.8272	0.827196585	protein_coding	ENST00000308304
ASHGV40006592	-0.82708	0.82707966	protein_coding	NM_001004052
ASHGV40031464	-0.82707	0.827069739	protein_coding	NM_153324
ASHGV40052315	0.82668	0.826680132	protein_coding	NM_001006617
ASHGV40002055	0.826673	0.82667314	protein_coding	NM_000476
ASHGV40048590	-0.8265	0.826504688	protein_coding	NM_032982
ASHGV40027551	-0.82635	0.826348424	protein_coding	NM_005168
ASHGV40013647	-0.82633	0.826327862	protein_coding	NM_172370
ASHGV40045496	-0.82627	0.826267824	protein_coding	ENST00000417143
ASHGV40047048	-0.82627	0.826265039	protein_coding	NM_001990
ASHGV40052676	-0.82614	0.826143687	protein_coding	NM_017738
ASHGV40033688	-0.82609	0.826089727	protein_coding	NM_000395
ASHGV40042378	0.826065	0.82606527	protein_coding	NM_001903
ASHGV40028693	0.825822	0.825821608	protein_coding	NM_014600
ASHGV40036279	0.82553	0.825529509	protein_coding	NM_001199198
ASHGV40029704	0.825521	0.825521153	protein_coding	NM_004180
ASHGV40033862	-0.82542	0.825415909	protein_coding	NM_015124

ASHGV40041208	-0.82537	0.825373507	protein_coding	NM_032361
ASHGV40030288	-0.82536	0.825363068	protein_coding	NM_001287395
ASHGV40045990	-0.82523	0.825226304	protein_coding	NM_001040000
ASHGV40008680	-0.82515	0.825150317	protein_coding	NM_153266
ASHGV40037787	0.825016	0.825016357	protein_coding	uc003huc.2
ASHGV40029028	0.824808	0.824807932	protein_coding	NM_006463
ASHGV40035948	0.824703	0.824703331	protein_coding	NM_003458
ASHGV40007700	-0.82452	0.824515517	protein_coding	NM_005797
ASHGV40016598	0.824509	0.824508788	protein_coding	NM_001282494
ASHGV40013708	0.824456	0.824456236	protein_coding	NM_001303133
ASHGV40021682	-0.82445	0.824449627	protein_coding	NM_003673
ASHGV40009231	0.824428	0.824427726	protein_coding	NM_001290474
ASHGV40022497	0.824183	0.824182982	protein_coding	NM_022068
ASHGV40020275	-0.82409	0.824088538	protein_coding	NM_152219
ASHGV40033754	0.823869	0.823869181	protein_coding	NM_004810
ASHGV40014125	-0.82381	0.823811643	protein_coding	NM_005060
ASHGV40036802	0.823542	0.823541964	protein_coding	NM_002740
ASHGV40039849	-0.82308	0.823077467	protein_coding	NM_002529
ASHGV40034188	0.822833	0.822833481	protein_coding	NM_031899
ASHGV40019244	-0.82268	0.822679789	protein_coding	NM_001795
ASHGV40041937	-0.82243	0.822431815	protein_coding	NM_001992
ASHGV40010983	0.822402	0.822401571	protein_coding	NM_019858
ASHGV40018550	0.822303	0.822303185	protein_coding	NM_004548
ASHGV40023729	0.822262	0.822262022	protein_coding	NM_012398
ASHGV40056569	-0.82218	0.82218183	protein_coding	uc010pbf.2
ASHGV40034303	-0.82215	0.822150571	protein_coding	NM_001080525
ASHGV40043813	0.821991	0.821991164	protein_coding	NM_021244
ASHGV40016167	0.82186	0.821860114	protein_coding	NM_020843
ASHGV40000815	-0.82183	0.821831464	protein_coding	ENST00000435837
ASHGV40041938	-0.8218	0.821803037	protein_coding	NM_005242
ASHGV40031808	0.821676	0.821675874	protein_coding	NM_016407
ASHGV40002765	0.821672	0.821672484	protein_coding	NM_002038
ASHGV40047420	-0.82133	0.821329113	protein_coding	NM_017802
ASHGV40054414	0.821232	0.821232309	protein_coding	NM_017413
ASHGV40021685	-0.82085	0.820854831	protein_coding	NM_005310
ASHGV40035827	0.820813	0.820812659	protein_coding	NM_001248
ASHGV40015041	0.820763	0.820762508	protein_coding	NM_001011713
ASHGV40005623	0.820756	0.82075562	protein_coding	NM_022120
ASHGV40010976	0.820625	0.820625407	protein_coding	NM_000849
ASHGV40034970	-0.82062	0.820620402	protein_coding	NM_016216
ASHGV40026936	-0.82036	0.820360159	protein_coding	NM_032319
ASHGV40017939	0.820095	0.820094952	protein_coding	NM_018206
ASHGV40057215	-0.81987	0.819872888	protein_coding	NM_000500
ASHGV40018234	-0.81968	0.819678918	protein_coding	NM_031293
ASHGV40056567	0.819275	0.819275155	protein_coding	NM_017916
ASHGV40024609	-0.81922	0.819220167	protein_coding	ENST00000270235
ASHGV40042141	0.81922	0.819219618	protein_coding	NM_002372

ASHGV40022904	0.819116	0.819116064	protein_coding	NM_019022
ASHGV40006831	-0.81908	0.819081069	protein_coding	NM_198381
ASHGV40030333	0.819028	0.819027689	protein_coding	NM_006710
ASHGV40050271	-0.81886	0.818863529	protein_coding	NM_002052
ASHGV40039448	0.81885	0.81885015	protein_coding	NM_002039
ASHGV40024612	0.818711	0.818711318	protein_coding	NM_017805
ASHGV40032563	-0.81863	0.818633102	protein_coding	ENST00000333765
ASHGV40044739	0.818369	0.818369157	protein_coding	NM_003545
ASHGV40042455	-0.81829	0.818292137	protein_coding	NM_018923
ASHGV40013604	0.817928	0.817927794	protein_coding	NM_014849
ASHGV40020311	-0.81783	0.817828816	protein_coding	NM_033060
ASHGV40006102	-0.81781	0.817810157	protein_coding	NM_030929
ASHGV40032557	-0.81771	0.817713753	protein_coding	NM_020152
ASHGV40031365	0.817583	0.817582529	protein_coding	NM_006466
ASHGV40019774	-0.81718	0.817184686	protein_coding	NM_153357
ASHGV40015791	0.817162	0.817162246	protein_coding	NM_001130858
ASHGV40031535	-0.81652	0.816516132	protein_coding	NM_006690
ASHGV40042465	-0.81619	0.816193543	protein_coding	NM_018913
ASHGV40009434	-0.81619	0.816193296	protein_coding	NM_172364
ASHGV40018510	-0.81604	0.816040894	protein_coding	NM_022092
ASHGV40026881	-0.81588	0.815879208	protein_coding	ENST00000335648
ASHGV40024591	-0.81582	0.815821861	protein_coding	NM_019855
ASHGV40049583	-0.81577	0.81577155	protein_coding	NM_019098
ASHGV40044209	0.815746	0.8157464	protein_coding	NM_005670
ASHGV40023864	-0.81573	0.815731216	protein_coding	NM_017703
ASHGV40051359	-0.81565	0.815646279	protein_coding	NM_002066
ASHGV40008670	0.815514	0.815513668	protein_coding	NM_003860
ASHGV40042330	-0.81533	0.815329448	protein_coding	NM_152663
ASHGV40011324	0.815277	0.815277325	protein_coding	NM_001013635
ASHGV40015792	-0.81527	0.815274497	protein_coding	NM_153700
ASHGV40054813	-0.81515	0.815147822	protein_coding	ENST00000313548
ASHGV40026481	0.814976	0.814976491	protein_coding	NM_021907
ASHGV40049571	-0.81496	0.81496421	protein_coding	ENST00000540724
ASHGV40052812	-0.81461	0.814611692	protein_coding	NM_021111
ASHGV40038802	0.814522	0.814522055	protein_coding	NM_005339
ASHGV40051402	0.81452	0.814520177	protein_coding	ENST00000318911
ASHGV40031005	0.814478	0.814477999	protein_coding	NM_006045
ASHGV40048914	0.814392	0.814391567	protein_coding	ENST00000527396
ASHGV40021628	0.814307	0.814306681	protein_coding	NM_024835
ASHGV40053096	-0.81429	0.814292033	protein_coding	NM_001002266
ASHGV40018173	-0.81396	0.813963187	protein_coding	NM_002801
ASHGV40021482	0.813799	0.81379905	protein_coding	NM_016231
ASHGV40042318	0.813782	0.813782094	protein_coding	NM_002154
ASHGV40057453	-0.81371	0.813713599	protein_coding	NM_017489
ASHGV40011631	0.813703	0.813702711	protein_coding	NM_001007553
ASHGV40024277	-0.81365	0.813646739	protein_coding	NM_207392
ASHGV40029623	0.813542	0.813541803	protein_coding	NM_018328

ASHGV40005889	-0.8135	0.813495525	protein_coding	NM_030927
ASHGV40050919	0.813491	0.813490686	protein_coding	NM_004337
ASHGV40039976	0.8134	0.813400275	protein_coding	NM_033414
ASHGV40028037	-0.81335	0.813351736	protein_coding	NM_020814
ASHGV40026628	-0.81333	0.813329068	protein_coding	NM_000478
ASHGV40018179	0.813144	0.813143759	protein_coding	NM_018380
ASHGV40040680	0.813022	0.81302236	protein_coding	NM_181836
ASHGV40046436	0.812899	0.812899255	protein_coding	NM_018060
ASHGV40054254	0.812741	0.812741141	protein_coding	NM_001011657
ASHGV40018797	0.812654	0.812654396	protein_coding	NM_003366
ASHGV40044271	0.812368	0.812367653	protein_coding	NM_019041
ASHGV40023342	0.812295	0.812295211	protein_coding	NM_015865
ASHGV40042919	-0.81217	0.81217447	protein_coding	NM_001012418
ASHGV40020822	-0.8121	0.812099167	protein_coding	ENST00000599136
ASHGV40018424	-0.81202	0.812017871	protein_coding	uc002fle.1
ASHGV40000174	0.811838	0.811838101	protein_coding	NM_014145
ASHGV40009882	0.811798	0.811798266	protein_coding	NM_003243
ASHGV40057497	0.811753	0.811752883	protein_coding	NM_001010915
ASHGV40048564	-0.81164	0.811641952	protein_coding	NM_004668
ASHGV40013688	0.811627	0.811626581	protein_coding	NM_030920
ASHGV40034180	0.811166	0.81116552	protein_coding	NM_001607
ASHGV40044890	-0.81116	0.811160031	protein_coding	uc011dpe.2
ASHGV40034449	0.811083	0.811082998	protein_coding	NM_015224
ASHGV40038481	0.811001	0.811001057	protein_coding	ENST00000506607
ASHGV40006690	-0.81065	0.810650578	protein_coding	ENST00000331587
ASHGV40018157	-0.81062	0.810618242	protein_coding	NM_001138
ASHGV40021734	-0.81056	0.810555457	protein_coding	NM_017595
ASHGV40038252	0.810454	0.810453509	protein_coding	NM_017867
ASHGV40023696	0.810444	0.810443728	protein_coding	NM_003938
ASHGV40037401	-0.81017	0.810174184	protein_coding	uc003gsx.4
ASHGV40057311	0.81009	0.810090221	protein_coding	NM_001788
ASHGV40020320	-0.81	0.809997261	protein_coding	NM_003770
ASHGV40015235	0.809983	0.809982964	protein_coding	NM_031210
ASHGV40005733	0.809969	0.809968827	protein_coding	NM_030625
ASHGV40050526	0.809961	0.809960828	protein_coding	NM_004674
ASHGV40028611	-0.80968	0.809681209	protein_coding	NM_024322
ASHGV40052102	0.809599	0.809598575	protein_coding	NM_032342
ASHGV40023195	-0.80955	0.809554882	protein_coding	NM_006938
ASHGV40033740	-0.80929	0.809291516	protein_coding	NM_014508
ASHGV40038129	-0.80924	0.809239499	protein_coding	ENST00000508611
ASHGV40037165	0.809107	0.809107428	protein_coding	NM_006099
ASHGV40033516	0.808982	0.808981513	protein_coding	NM_019601
ASHGV40008206	0.808973	0.80897257	protein_coding	NM_031418
ASHGV40039107	-0.80882	0.808818266	protein_coding	NM_152770
ASHGV40019332	-0.80873	0.808725434	protein_coding	NM_018332
ASHGV40032926	-0.80869	0.808691268	protein_coding	NM_021916
ASHGV40037108	-0.80857	0.808566847	protein_coding	NM_153713

ASHGV40038248	0.808482	0.808482376	protein_coding	NM_020870
ASHGV40041933	0.808391	0.808390514	protein_coding	NM_014979
ASHGV40005516	0.808372	0.808371514	protein_coding	NM_145012
ASHGV40032735	0.808164	0.808164213	protein_coding	NM_004768
ASHGV40055962	-0.80803	0.808028483	protein_coding	NM_021104
ASHGV40024584	0.807945	0.807945072	protein_coding	NM_003827
ASHGV40008563	0.807941	0.807941101	protein_coding	NM_004585
ASHGV40041602	-0.8079	0.807895162	protein_coding	NM_015577
ASHGV40042669	-0.80779	0.807792611	protein_coding	NM_012484
ASHGV40018214	-0.80777	0.807773493	protein_coding	NM_001270974
ASHGV40022046	0.807667	0.80766707	protein_coding	NM_030938
ASHGV40042460	-0.80766	0.807660998	protein_coding	NM_003736
ASHGV40022932	0.807648	0.807648239	protein_coding	NM_182511
ASHGV40054024	-0.80752	0.807521147	protein_coding	NM_173358
ASHGV40042423	-0.80751	0.807511929	protein_coding	NM_031857
ASHGV40028177	0.807497	0.80749711	protein_coding	NM_004238
ASHGV40042342	0.807218	0.807217799	protein_coding	NM_024715
ASHGV40005411	0.8072	0.807200201	protein_coding	NM_019590
ASHGV40021535	0.806933	0.806933141	protein_coding	NM_001304
ASHGV40055845	-0.80682	0.806821177	protein_coding	NM_020992
ASHGV40028274	0.806756	0.806755578	protein_coding	NM_030768
ASHGV40044084	-0.80643	0.80642968	protein_coding	NM_030908
ASHGV40031163	-0.80607	0.806072652	protein_coding	NM_005975
ASHGV40041583	-0.80605	0.806048586	protein_coding	ENST00000326958
ASHGV40011513	-0.80589	0.805889903	protein_coding	NM_001798
ASHGV40003137	-0.8057	0.805701381	protein_coding	NM_001168682
ASHGV40053837	0.805571	0.805571054	protein_coding	NM_153270
ASHGV40052166	0.805021	0.80502137	protein_coding	NM_002829
ASHGV40047698	0.804942	0.804942464	protein_coding	NM_133468
ASHGV40009062	-0.80491	0.804905646	protein_coding	NM_001130145
ASHGV40031235	0.804659	0.804659469	protein_coding	NM_024646
ASHGV40040159	0.80458	0.804580427	protein_coding	NM_006251
ASHGV40050588	0.80456	0.804560076	protein_coding	NM_005662
ASHGV40014325	-0.80445	0.804454361	protein_coding	NM_001014450
ASHGV40032840	-0.80435	0.804346144	protein_coding	NM_003325
ASHGV40019188	-0.80429	0.80429333	protein_coding	NM_005682
ASHGV40012843	0.804238	0.804237681	protein_coding	NM_020121
ASHGV40031192	-0.80418	0.804180123	protein_coding	NM_207469
ASHGV40028485	-0.804	0.803998034	protein_coding	NM_182626
ASHGV40048111	0.803785	0.803785081	protein_coding	NM_152999
ASHGV40047676	-0.80375	0.803753941	protein_coding	NM_032222
ASHGV40046770	0.803749	0.803749376	protein_coding	NM_001146068
ASHGV40057752	-0.80374	0.803738202	protein_coding	NM_147199
ASHGV40009428	-0.80358	0.803583511	protein_coding	NM_134424
ASHGV40055358	0.803579	0.803578544	protein_coding	NM_006603
ASHGV40030445	0.803175	0.803174766	protein_coding	NM_016143
ASHGV40025648	-0.80316	0.803160038	protein_coding	NM_201628

ASHGV40007497	0.802912	0.802912451	protein_coding	NM_016156
ASHGV40006967	-0.80282	0.802820796	protein_coding	NM_001005199
ASHGV40009130	0.802796	0.802796043	protein_coding	NM_001037954
ASHGV40040420	0.802795	0.802794785	protein_coding	NM_004607
ASHGV40011522	0.802739	0.802739261	protein_coding	NM_002475
ASHGV40030030	-0.8027	0.802704726	protein_coding	NM_001102659
ASHGV40012569	0.802651	0.80265136	protein_coding	NM_001079670
ASHGV40023637	-0.80263	0.802629315	protein_coding	NM_024805
ASHGV40033798	-0.8024	0.802401677	protein_coding	NM_198460
ASHGV40011304	-0.8023	0.802297114	protein_coding	NM_001281429
ASHGV40022587	-0.80225	0.802252319	protein_coding	NM_001010889
ASHGV40033857	-0.80217	0.802166017	protein_coding	NM_016426
ASHGV40013802	0.801949	0.801949438	protein_coding	NM_001668
ASHGV40027219	0.801799	0.801798881	protein_coding	NM_025076
ASHGV40050911	0.801744	0.801743504	protein_coding	NM_024948
ASHGV40024142	-0.80162	0.80162232	protein_coding	ENST00000597060
ASHGV40042555	-0.80148	0.801478679	protein_coding	NM_000112
ASHGV40007648	0.80095	0.800950457	protein_coding	NM_015306
ASHGV40011642	-0.80079	0.800790886	protein_coding	NM_006482
ASHGV40008609	-0.80072	0.800722762	protein_coding	NM_018484
ASHGV40020287	-0.80057	0.800566956	protein_coding	NM_181539
ASHGV40010587	0.800562	0.800561562	protein_coding	NM_032369
ASHGV40017333	-0.80051	0.800514924	protein_coding	NM_021005
ASHGV40047398	-0.80018	0.800177936	protein_coding	NM_001010867
ASHGV40024300	0.800136	0.800135859	protein_coding	NM_001864
ASHGV40056948	-0.80012	0.800118969	protein_coding	NM_013308
ASHGV40052442	0.800116	0.800115735	protein_coding	NM_001279349
ASHGV40021266	0.799868	0.799868449	protein_coding	NM_030808
ASHGV40014309	-0.79975	0.799749753	protein_coding	NM_006945
ASHGV40020684	-0.7997	0.799698768	protein_coding	NM_001915
ASHGV40050666	-0.79955	0.799549693	protein_coding	NM_022454
ASHGV40014597	0.79954	0.799540282	protein_coding	NM_005348
ASHGV40007952	-0.79953	0.799529706	protein_coding	NM_003282
ASHGV40023701	-0.79948	0.799482779	protein_coding	NM_144616
ASHGV40000247	-0.79925	0.799253725	protein_coding	ENST00000404912
ASHGV40010096	-0.79898	0.798979545	protein_coding	NM_001130963
ASHGV40037197	0.798904	0.79890417	protein_coding	NM_203462
ASHGV40008031	-0.79888	0.798878591	protein_coding	NM_001005178
ASHGV40015876	-0.79847	0.798471524	protein_coding	NM_020396
ASHGV40029579	-0.79831	0.798307558	protein_coding	NM_001291281
ASHGV40057507	-0.79806	0.798063674	protein_coding	ENST00000451672
ASHGV40055736	-0.79803	0.798031456	protein_coding	ENST00000538322
ASHGV40023871	0.797901	0.797901121	protein_coding	NM_001379
ASHGV40009146	0.797876	0.79787556	protein_coding	NM_181351
ASHGV40008721	0.797698	0.797697726	protein_coding	NM_000852
ASHGV40039833	0.797592	0.797591995	protein_coding	NM_030782
ASHGV40053946	0.797501	0.797501345	protein_coding	NM_000266

ASHGV40003163	-0.79747	0.797474536	protein_coding	NM_001199661
ASHGV40051412	0.797401	0.797401203	protein_coding	NM_005526
ASHGV40034476	-0.79715	0.797145598	protein_coding	NM_198463
ASHGV40017736	0.796997	0.796996663	protein_coding	NM_020718
ASHGV40033135	-0.79693	0.796932794	protein_coding	NM_002405
ASHGV40046263	0.796915	0.796914725	protein_coding	NM_015550
ASHGV40009100	0.796915	0.796914529	protein_coding	NM_000019
ASHGV40054055	-0.79687	0.796866843	protein_coding	NM_002625
ASHGV40022609	0.796843	0.796843067	protein_coding	NM_198991
ASHGV40049755	0.796764	0.796764477	protein_coding	NM_139166
ASHGV40052476	-0.79672	0.79672327	protein_coding	NM_182974
ASHGV40018528	0.796578	0.796578264	protein_coding	NM_003933
ASHGV40015779	-0.79652	0.796519929	protein_coding	NM_138477
ASHGV40013830	-0.79651	0.796508563	protein_coding	NM_017815
ASHGV40055585	-0.79631	0.796305789	protein_coding	NM_080720
ASHGV40052395	-0.79629	0.796294987	protein_coding	NM_004878
ASHGV40031195	-0.79618	0.796175866	protein_coding	NM_033089
ASHGV40020242	-0.79605	0.796047803	protein_coding	NM_007144
ASHGV40018253	0.795608	0.795608115	protein_coding	NM_024306
ASHGV40012046	-0.7954	0.795402664	protein_coding	ENST00000547948
ASHGV40005391	-0.79521	0.795208886	protein_coding	NM_004468
ASHGV40028659	-0.79517	0.795170486	protein_coding	NM_032266
ASHGV40008282	0.795152	0.795152311	protein_coding	NM_003477
ASHGV40050893	0.795058	0.79505842	protein_coding	NM_033402
ASHGV40034314	0.794858	0.794858405	protein_coding	NM_003365
ASHGV40010733	0.794854	0.79485374	protein_coding	NM_001167856
ASHGV40046141	0.794738	0.794737851	protein_coding	NM_016265
ASHGV40053423	-0.79457	0.79456874	protein_coding	NM_173689
ASHGV40015562	0.794565	0.794565043	protein_coding	NM_001312
ASHGV40028325	-0.79435	0.794350184	protein_coding	ENST00000599492
ASHGV40008664	0.794282	0.794282364	protein_coding	NM_006442
ASHGV40040896	-0.79428	0.794278743	protein_coding	NM_002622
ASHGV40034986	0.794156	0.794156424	protein_coding	NM_004766
ASHGV40026926	-0.79412	0.794122155	protein_coding	NM_019885
ASHGV40033778	0.794059	0.794059377	protein_coding	NM_001098
ASHGV40043113	0.794023	0.794022859	protein_coding	NM_014809
ASHGV40003125	0.794009	0.79400871	protein_coding	NM_001145073
ASHGV40015586	-0.79388	0.793876425	protein_coding	NM_001145004
ASHGV40030124	-0.7937	0.793695744	protein_coding	NM_170699
ASHGV40008845	0.793696	0.793695597	protein_coding	NM_025155
ASHGV40022781	-0.79349	0.793485188	protein_coding	NM_001080467
ASHGV40040973	-0.79339	0.793394239	protein_coding	NM_206966
ASHGV40016726	-0.79325	0.793252463	protein_coding	NM_033319
ASHGV40034362	-0.79318	0.793183994	protein_coding	NM_002447
ASHGV40040097	0.793159	0.793159272	protein_coding	NM_030945
ASHGV40005970	-0.79304	0.793043296	protein_coding	NM_001284259
ASHGV40045172	0.793013	0.79301343	protein_coding	NM_012347

ASHGV40001667	-0.79269	0.792687494	protein_coding	NM_003301
ASHGV40009854	0.792608	0.792608355	protein_coding	NM_002822
ASHGV40045745	-0.79232	0.792321096	protein_coding	NM_001077706
ASHGV40051718	0.792232	0.792231646	protein_coding	NM_145698
ASHGV40035937	0.792147	0.792147028	protein_coding	NM_199069
ASHGV40024951	0.792116	0.792115664	protein_coding	NM_002085
ASHGV40042642	0.792021	0.792020882	protein_coding	NM_001445
ASHGV40042055	0.79192	0.79192044	protein_coding	NM_014857
ASHGV40023906	0.791854	0.791854402	protein_coding	NM_004283
ASHGV40037628	-0.79182	0.791820181	protein_coding	NM_201431
ASHGV40020979	0.791459	0.791459464	protein_coding	NM_001080395
ASHGV40046750	0.791331	0.791331486	protein_coding	NM_018843
ASHGV40056938	-0.79124	0.791238005	protein_coding	uc003eox.3
ASHGV40018248	-0.79113	0.791125461	protein_coding	NM_001011880
ASHGV40023348	-0.79107	0.791068686	protein_coding	NM_145055
ASHGV40039279	0.791011	0.791010698	protein_coding	NM_152400
ASHGV40016440	0.790903	0.790902659	protein_coding	ENST00000378904
ASHGV40018169	0.790883	0.790882585	protein_coding	NM_020850
ASHGV40031971	-0.79065	0.790645804	protein_coding	NM_004535
ASHGV40025906	-0.79059	0.790590999	protein_coding	NM_153329
ASHGV40046934	0.790559	0.790559109	protein_coding	NM_006754
ASHGV40025565	-0.79038	0.790382826	protein_coding	NM_014164
ASHGV40019953	0.79029	0.790290408	protein_coding	NM_003653
ASHGV40055742	-0.79022	0.790218303	protein_coding	NM_003019
ASHGV40013352	-0.79021	0.790205372	protein_coding	NM_001004127
ASHGV40057651	-0.79017	0.790165934	protein_coding	NM_016249
ASHGV40006324	0.790152	0.790151879	protein_coding	NM_001609
ASHGV40007353	0.789991	0.789991202	protein_coding	NM_017887
ASHGV40017236	0.789706	0.789705533	protein_coding	NM_007011
ASHGV40042586	0.789672	0.789671592	protein_coding	NM_000827
ASHGV40054268	0.789667	0.789666805	protein_coding	NM_153333
ASHGV40002434	-0.78964	0.789636816	protein_coding	ENST00000578340
ASHGV40024545	-0.7896	0.789599476	protein_coding	NM_005091
ASHGV40022077	0.789524	0.789523899	protein_coding	NM_007372
ASHGV40043756	0.789312	0.789311796	protein_coding	NM_015525
ASHGV40026151	-0.78908	0.789078357	protein_coding	NM_001204818
ASHGV40051919	-0.78907	0.789068668	protein_coding	NM_001276366
ASHGV40043615	0.789064	0.789064036	protein_coding	NM_001498
ASHGV40020726	-0.78905	0.789050504	protein_coding	NM_004655
ASHGV40022759	0.78898	0.788979658	protein_coding	NM_005901
ASHGV40039575	-0.78885	0.788850132	protein_coding	NM_170707
ASHGV40000420	0.788604	0.788603988	protein_coding	NM_014166
ASHGV40055213	0.78845	0.788449982	protein_coding	NM_016608
ASHGV40052283	0.788319	0.788319498	protein_coding	NM_001100588
ASHGV40000677	-0.78815	0.788154412	protein_coding	ENST00000429234
ASHGV40043910	0.787955	0.787955106	protein_coding	NM_002838
ASHGV40017702	0.787876	0.787876081	protein_coding	NM_016235

ASHGV40041001	-0.78745	0.787450738	protein_coding	NM_001012301
ASHGV40024990	-0.78743	0.787429281	protein_coding	uc002lvo.1
ASHGV40012598	0.787145	0.787145211	protein_coding	NM_016075
ASHGV40024967	-0.78707	0.787074028	protein_coding	NM_138393
ASHGV40008591	-0.78702	0.787022102	protein_coding	NM_000932
ASHGV40014316	0.787007	0.787006652	protein_coding	NM_006827
ASHGV40027647	-0.78683	0.786832136	protein_coding	NM_018086
ASHGV40021431	0.786806	0.786806489	protein_coding	NM_001033553
ASHGV40025371	0.786697	0.786696752	protein_coding	NM_015321
ASHGV40015846	-0.7866	0.786603087	protein_coding	NM_005254
ASHGV40005748	0.786526	0.786526188	protein_coding	NM_000188
ASHGV40011097	-0.78642	0.786415094	protein_coding	NM_175874
ASHGV40047767	0.786414	0.786414481	protein_coding	NM_015052
ASHGV40015532	-0.78611	0.786105889	protein_coding	NM_001080464
ASHGV40033520	-0.78599	0.785991994	protein_coding	NM_000675
ASHGV40014798	0.785991	0.785990681	protein_coding	NM_001162383
ASHGV40040679	0.785966	0.785965988	protein_coding	NM_020177
ASHGV40012021	0.78581	0.785810365	protein_coding	NM_138432
ASHGV40014306	-0.78578	0.78577838	protein_coding	NM_031464
ASHGV40048763	0.785498	0.785497946	protein_coding	NM_014671
ASHGV40041759	0.785359	0.785359101	protein_coding	NM_015360
ASHGV40040824	0.785341	0.785340693	protein_coding	NM_080656
ASHGV40024400	-0.78514	0.785136842	protein_coding	NM_013368
ASHGV40014567	-0.78507	0.785066831	protein_coding	NM_001039355
ASHGV40040967	-0.78507	0.785066758	protein_coding	NM_001387
ASHGV40003077	-0.78473	0.784726797	protein_coding	NM_001012276
ASHGV40010710	-0.7847	0.784704194	protein_coding	uc001ubf.2
ASHGV40044989	-0.78463	0.784625868	protein_coding	uc003ono.1
ASHGV40027103	0.784384	0.784384018	protein_coding	NM_013943
ASHGV40007049	-0.78416	0.784164282	protein_coding	NM_152718
ASHGV40024955	0.784097	0.784097149	protein_coding	NM_001280
ASHGV40021973	-0.78405	0.784050286	protein_coding	NM_005486
ASHGV40026572	0.783988	0.783988381	protein_coding	NM_032574
ASHGV40028402	0.783796	0.783796434	protein_coding	NM_016030
ASHGV40033183	0.783692	0.783691861	protein_coding	NM_194326
ASHGV40031177	-0.7836	0.783599742	protein_coding	NM_080621
ASHGV40015192	-0.78338	0.783376092	protein_coding	NM_015962
ASHGV40014439	0.783295	0.783294601	protein_coding	NM_017970
ASHGV40057314	-0.78328	0.783275506	protein_coding	NM_032014
ASHGV40018443	-0.78293	0.782926701	protein_coding	NM_001242757
ASHGV40009015	0.782871	0.78287077	protein_coding	NM_001190462
ASHGV40018860	-0.78287	0.782870638	protein_coding	ENST00000566854
ASHGV40048448	-0.78268	0.782683639	protein_coding	NM_032643
ASHGV40018952	0.782594	0.782594258	protein_coding	NM_032188
ASHGV40006258	-0.78259	0.782590841	protein_coding	NM_003054
ASHGV40009369	-0.78246	0.782459176	protein_coding	NM_138788
ASHGV40016951	0.782419	0.78241866	protein_coding	NM_004663

ASHGV40000692	-0.78233	0.782331027	protein_coding	ENST00000429984
ASHGV40009215	0.781961	0.781960791	protein_coding	NM_015157
ASHGV40049426	0.781834	0.781834012	protein_coding	NM_006421
ASHGV40046868	0.781829	0.781829401	protein_coding	NM_022574
ASHGV40021752	0.78181	0.781809801	protein_coding	NM_003632
ASHGV40024071	0.781753	0.781753114	protein_coding	NM_001080421
ASHGV40049002	-0.78173	0.781729095	protein_coding	NM_018688
ASHGV40004981	0.781667	0.781667481	protein_coding	NM_022063
ASHGV40013486	0.781318	0.781317713	protein_coding	NM_144595
ASHGV40034711	-0.78123	0.781233139	protein_coding	ENST00000593496
ASHGV40009311	-0.78113	0.781126008	protein_coding	NM_032184
ASHGV40018494	0.78106	0.781060394	protein_coding	NM_021168
ASHGV40034672	0.781046	0.781045998	protein_coding	NM_014415
ASHGV40026038	-0.78099	0.780990745	protein_coding	NM_024075
ASHGV40021293	-0.78079	0.780787866	protein_coding	NM_020233
ASHGV40040578	-0.78063	0.780633846	protein_coding	NM_153234
ASHGV40042429	0.780566	0.780565705	protein_coding	NM_018899
ASHGV40052372	0.78055	0.780550025	protein_coding	NM_020145
ASHGV40042145	0.78053	0.780529558	protein_coding	NM_138773
ASHGV40007926	-0.78032	0.78032155	protein_coding	NM_004357
ASHGV40013890	0.780203	0.780203489	protein_coding	NM_002515
ASHGV40023485	-0.78018	0.780183636	protein_coding	NM_012397
ASHGV40007935	-0.78011	0.780111068	protein_coding	NM_001304359
ASHGV40054454	-0.78004	0.780038071	protein_coding	NM_001031705
ASHGV40006379	0.779658	0.779657553	protein_coding	NM_006504
ASHGV40033664	0.779637	0.77963665	protein_coding	NM_006769
ASHGV40046289	0.779559	0.779558683	protein_coding	NM_031243
ASHGV40053434	-0.77923	0.779227277	protein_coding	uc010mwo.1
ASHGV40020332	-0.77914	0.779143763	protein_coding	NM_005557
ASHGV40057260	-0.77901	0.779009615	protein_coding	NM_014320
ASHGV40032255	-0.77873	0.778726327	protein_coding	NM_005867
ASHGV40033048	0.778374	0.778373761	protein_coding	NM_080430
ASHGV40045930	0.778372	0.778372315	protein_coding	NM_006243
ASHGV40015563	-0.77828	0.778281657	protein_coding	NM_001311
ASHGV40038463	0.77816	0.778159601	protein_coding	NM_001017405
ASHGV40046935	0.777757	0.77775663	protein_coding	NM_005746
ASHGV40017957	0.777674	0.777674295	protein_coding	NM_198503
ASHGV40050681	0.777617	0.777616648	protein_coding	NM_001011671
ASHGV40029590	-0.77721	0.777213833	protein_coding	NM_003937
ASHGV40044164	0.77713	0.777130324	protein_coding	NM_001286611
ASHGV40008689	-0.77675	0.776751842	protein_coding	NM_145065
ASHGV40034857	-0.77672	0.776720469	protein_coding	NM_052883
ASHGV40014678	-0.77666	0.776663104	protein_coding	NM_002456
ASHGV40030933	0.776621	0.776620822	protein_coding	NM_012408
ASHGV40018653	0.77662	0.776619935	protein_coding	NM_001079514
ASHGV40030452	0.77658	0.776580059	protein_coding	NM_024411
ASHGV40020640	-0.7764	0.776398667	protein_coding	NM_182620

ASHGV40011829	-0.77605	0.776045473	protein_coding	NM_001007237
ASHGV40048279	0.775759	0.775759164	protein_coding	NM_002803
ASHGV40007122	0.775706	0.775706413	protein_coding	NM_001033678
ASHGV40049537	0.775525	0.775524576	protein_coding	NM_005536
ASHGV40051759	-0.77551	0.775514861	protein_coding	NM_001113541
ASHGV40006018	-0.77549	0.775490601	protein_coding	ENST00000394005
ASHGV40044406	-0.77542	0.77541869	protein_coding	uc021zii.2
ASHGV40030354	0.775115	0.775114608	protein_coding	NM_015650
ASHGV40005014	0.775104	0.775103801	protein_coding	NM_001001976
ASHGV40044945	-0.775	0.774998939	protein_coding	NM_145899
ASHGV40054612	-0.7748	0.774799773	protein_coding	NM_000451
ASHGV40035935	0.774752	0.77475175	protein_coding	NM_177939
ASHGV40022041	0.774751	0.77475128	protein_coding	NM_152666
ASHGV40047930	-0.77474	0.774738687	protein_coding	NM_001159522
ASHGV40005474	-0.77447	0.774471301	protein_coding	NM_005204
ASHGV40009037	-0.7744	0.77439942	protein_coding	NM_003772
ASHGV40014867	0.774306	0.774306205	protein_coding	NM_001128126
ASHGV40018593	-0.77425	0.77425098	protein_coding	NM_018992
ASHGV40020253	0.774103	0.774103048	protein_coding	NM_020405
ASHGV40013568	-0.77409	0.77409273	protein_coding	NM_005708
ASHGV40033706	0.77409	0.774089644	protein_coding	NM_002305
ASHGV40048580	-0.77392	0.773924276	protein_coding	NM_178829
ASHGV40027472	0.773686	0.773685934	protein_coding	NM_001349
ASHGV40028685	-0.77366	0.773655206	protein_coding	NM_030915
ASHGV40040660	0.773554	0.773554375	protein_coding	NM_004772
ASHGV40042428	-0.77352	0.773524729	protein_coding	NM_018898
ASHGV40008117	0.773417	0.773416959	protein_coding	ENST00000378545
ASHGV40011426	0.773385	0.773385043	protein_coding	NM_002178
ASHGV40027728	0.773342	0.773341918	protein_coding	NM_003387
ASHGV40025908	0.773252	0.773252204	protein_coding	NM_001015
ASHGV40043384	0.773216	0.77321644	protein_coding	NM_005643
ASHGV40002613	-0.77317	0.773174229	protein_coding	NM_182498
ASHGV40010824	0.773137	0.773136953	protein_coding	NM_015347
ASHGV40041315	0.773013	0.773012962	protein_coding	NM_013232
ASHGV40052807	0.772761	0.772760572	protein_coding	NM_001039792
ASHGV40017211	-0.77273	0.772731184	protein_coding	NM_004213
ASHGV40046323	-0.77267	0.77267306	protein_coding	NM_017946
ASHGV40015350	-0.7726	0.772603219	protein_coding	NM_138344
ASHGV40052188	-0.77257	0.772574937	protein_coding	ENST00000457681
ASHGV40000046	-0.77253	0.772530886	protein_coding	ENST00000308346
ASHGV40051328	-0.77215	0.772148065	protein_coding	NM_207371
ASHGV40012579	0.772146	0.772146082	protein_coding	NM_020456
ASHGV40040674	0.772137	0.772136734	protein_coding	NM_018700
ASHGV40012866	0.772078	0.772078493	protein_coding	NM_003576
ASHGV40043198	0.772063	0.772063149	protein_coding	uc009wym.3
ASHGV40031271	0.771926	0.771926477	protein_coding	NM_003818
ASHGV40045164	0.771909	0.771908854	protein_coding	NM_133367

ASHGV40015203	-0.77187	0.771869093	protein_coding	NM_024643
ASHGV40007498	-0.77164	0.771644704	protein_coding	NM_201546
ASHGV40011714	0.771599	0.771598812	protein_coding	NM_001270396
ASHGV40003303	-0.77154	0.7715415	protein_coding	NM_023016
ASHGV40016454	0.771488	0.77148799	protein_coding	NM_000130
ASHGV40035585	0.771437	0.771436901	protein_coding	NM_153635
ASHGV40025537	-0.77142	0.771418902	protein_coding	NM_001129994
ASHGV40036436	-0.77139	0.771387516	protein_coding	NM_000388
ASHGV40007126	0.77133	0.77133035	protein_coding	NM_004322
ASHGV40044683	-0.7712	0.771201965	protein_coding	NM_017774
ASHGV40017707	-0.77093	0.770926029	protein_coding	NM_182617
ASHGV40031226	0.770672	0.770672269	protein_coding	NM_080792
ASHGV40030110	-0.77048	0.770483707	protein_coding	NM_000597
ASHGV40036097	0.770394	0.770394348	protein_coding	NM_213609
ASHGV40021255	-0.77038	0.77037904	protein_coding	NM_012393
ASHGV40048952	0.770175	0.770175154	protein_coding	NM_013354
ASHGV40021953	0.77001	0.770010386	protein_coding	NM_016001
ASHGV40010410	-0.76995	0.769947875	protein_coding	ENST00000397807
ASHGV40051985	-0.76993	0.769929615	protein_coding	NM_005384
ASHGV40009996	-0.76988	0.769878062	protein_coding	NM_057088
ASHGV40019703	0.769858	0.769857748	protein_coding	NM_002561
ASHGV40042426	-0.76985	0.769853783	protein_coding	NM_018903
ASHGV40030760	0.769842	0.769842251	protein_coding	NM_031232
ASHGV40021836	-0.76934	0.769337891	protein_coding	NM_001256299
ASHGV40023129	0.769171	0.769170885	protein_coding	NM_006868
ASHGV40044112	0.768837	0.768836658	protein_coding	NM_017871
ASHGV40015821	-0.76881	0.768812811	protein_coding	NM_001077480
ASHGV40056203	-0.76874	0.768736482	protein_coding	uc002bhl.2
ASHGV40056335	0.768269	0.768269291	protein_coding	NM_016492
ASHGV40015442	-0.76806	0.768063174	protein_coding	NM_004833
ASHGV40026191	-0.76801	0.768013096	protein_coding	NM_018125
ASHGV40006634	-0.76793	0.76793461	protein_coding	NM_014818
ASHGV40046850	-0.76788	0.767883466	protein_coding	NM_014184
ASHGV40008059	0.767753	0.767752764	protein_coding	NM_003621
ASHGV40044464	-0.76771	0.76771466	protein_coding	NM_002598
ASHGV40055954	-0.76766	0.767661648	protein_coding	NM_001129742
ASHGV40006588	-0.76764	0.767636438	protein_coding	ENST00000332249
ASHGV40045627	0.767627	0.767627404	protein_coding	NM_001010852
ASHGV40005793	0.767502	0.767501851	protein_coding	NM_014747
ASHGV40009640	0.767487	0.767486892	protein_coding	NM_000834
ASHGV40020375	0.76703	0.767030165	protein_coding	NM_003766
ASHGV40051542	-0.76702	0.767021283	protein_coding	NM_017637
ASHGV40019495	0.766991	0.766990617	protein_coding	NM_019065
ASHGV40052398	0.766959	0.766958745	protein_coding	NM_015033
ASHGV40037265	0.766918	0.766917728	protein_coding	NM_153365
ASHGV40007592	-0.76676	0.766761113	protein_coding	NM_153705
ASHGV40056187	-0.76673	0.766729505	protein_coding	NM_012182

ASHGV40025723	-0.76667	0.766666585	protein_coding	NM_052848
ASHGV40026108	-0.76653	0.766530896	protein_coding	ENST00000376267
ASHGV40017542	-0.76629	0.766291882	protein_coding	NM_001080524
ASHGV40017791	-0.76626	0.766262488	protein_coding	ENST00000452313
ASHGV40040168	0.766147	0.766146721	protein_coding	NM_001005473
ASHGV40048140	0.766085	0.766085063	protein_coding	ENST00000317751
ASHGV40042082	0.765974	0.765974135	protein_coding	NM_005575
ASHGV40038357	0.765898	0.76589812	protein_coding	NM_001995
ASHGV40034766	0.765791	0.765790917	protein_coding	NM_025146
ASHGV40006921	0.765652	0.765652259	protein_coding	NM_032389
ASHGV40017465	-0.76563	0.765626959	protein_coding	NM_014714
ASHGV40050947	-0.76557	0.765570947	protein_coding	NM_153704
ASHGV40039129	0.765543	0.765543102	protein_coding	NM_001025616
ASHGV40035072	-0.76545	0.765452953	protein_coding	NM_015472
ASHGV40005732	0.76537	0.765369662	protein_coding	NM_012207
ASHGV40030287	-0.76503	0.765032826	protein_coding	NM_001145636
ASHGV40036250	0.764811	0.76481147	protein_coding	NM_032146
ASHGV40042410	0.76475	0.764749874	protein_coding	NM_006083
ASHGV40006199	-0.76472	0.764721753	protein_coding	NM_000681
ASHGV40035546	0.764711	0.764710713	protein_coding	NM_182916
ASHGV40016681	-0.76436	0.764357371	protein_coding	NM_005258
ASHGV40034271	0.764099	0.7640985	protein_coding	NM_002858
ASHGV40019070	-0.76402	0.764020623	protein_coding	NM_001114
ASHGV40007556	-0.76393	0.763933125	protein_coding	NM_001225
ASHGV40013586	-0.76381	0.763814317	protein_coding	NM_003516
ASHGV40034236	0.763764	0.763764076	protein_coding	uc003cmq.2
ASHGV40051711	-0.76356	0.763555001	protein_coding	NM_172312
ASHGV40021085	-0.76349	0.763488042	protein_coding	NM_001164405
ASHGV40040793	-0.76349	0.763486956	protein_coding	NM_001303622
ASHGV40019658	0.763292	0.763291821	protein_coding	NM_006761
ASHGV40015488	-0.76304	0.763040706	protein_coding	NM_000567
ASHGV40014773	-0.76302	0.763018374	protein_coding	NM_173527
ASHGV40031232	-0.763	0.762997357	protein_coding	NM_198994
ASHGV40015987	0.762969	0.762969269	protein_coding	NM_032231
ASHGV40007116	0.762892	0.762891972	protein_coding	NM_173587
ASHGV40022552	-0.76289	0.762887007	protein_coding	NM_145287
ASHGV40009014	-0.76286	0.762862161	protein_coding	NM_017704
ASHGV40018423	-0.76284	0.762835982	protein_coding	NM_000101
ASHGV40020155	0.762793	0.762793058	protein_coding	NM_001094
ASHGV40023955	-0.76272	0.762717117	protein_coding	NM_001080997
ASHGV40050140	-0.76269	0.762692711	protein_coding	NM_001001795
ASHGV40036420	-0.76267	0.762672741	protein_coding	NM_003889
ASHGV40051860	-0.76265	0.762646544	protein_coding	ENST00000376830
ASHGV40034758	0.76259	0.762590213	protein_coding	NM_015412
ASHGV40047378	0.762553	0.762553079	protein_coding	NM_022458
ASHGV40031241	-0.76239	0.762392224	protein_coding	NM_001167670
ASHGV40016007	0.762355	0.762355497	protein_coding	NM_006660

ASHGV40054967	-0.76218	0.76217509	protein_coding	NM_001098412
ASHGV40031679	0.762072	0.762072069	protein_coding	NM_015937
ASHGV40034802	-0.76206	0.762061856	protein_coding	NM_007085
ASHGV40018617	-0.76192	0.761920883	protein_coding	NM_003456
ASHGV40032718	-0.76182	0.761823576	protein_coding	NM_002606
ASHGV40024383	-0.76175	0.761754087	protein_coding	NM_001828
ASHGV40038886	0.76174	0.761739867	protein_coding	NM_030917
ASHGV40014607	-0.76154	0.761542632	protein_coding	NM_002870
ASHGV40033838	-0.76143	0.761428291	protein_coding	NM_017911
ASHGV40018045	0.761316	0.761316348	protein_coding	NM_001301267
ASHGV40034589	0.76121	0.76121048	protein_coding	NM_000158
ASHGV40017258	-0.76119	0.761188394	protein_coding	ENST00000438251
ASHGV40016460	-0.76111	0.761112086	protein_coding	NM_018445
ASHGV40018261	0.760776	0.760775591	protein_coding	NM_145254
ASHGV40021252	0.76074	0.760739635	protein_coding	NM_022051
ASHGV40010346	0.760378	0.760377515	protein_coding	NM_013244
ASHGV40028983	0.760083	0.76008309	protein_coding	NM_178439
ASHGV40033000	-0.7597	0.75970429	protein_coding	NM_001145418
ASHGV40002224	-0.75945	0.759453826	protein_coding	ENST00000561474
ASHGV40018546	0.759446	0.759445632	protein_coding	NM_015999
ASHGV40033645	0.759352	0.759351785	protein_coding	NM_012179
ASHGV40036431	-0.75922	0.759221392	protein_coding	NM_018456
ASHGV40017246	-0.75922	0.759221132	protein_coding	NM_152259
ASHGV40055096	-0.75901	0.759009466	protein_coding	NM_006517
ASHGV40049759	-0.75894	0.758940844	protein_coding	NM_001146
ASHGV40018476	-0.7588	0.758798106	protein_coding	NM_001003938
ASHGV40038478	0.75867	0.758669632	protein_coding	NM_178557
ASHGV40057788	0.758542	0.758542384	protein_coding	NM_014752
ASHGV40049829	-0.75838	0.758384599	protein_coding	NM_002546
ASHGV40048748	-0.75832	0.758320003	protein_coding	NM_016076
ASHGV40005945	0.758117	0.758117194	protein_coding	NM_000314
ASHGV40013820	0.758054	0.758053921	protein_coding	NM_001344
ASHGV40041076	-0.75795	0.757952187	protein_coding	NM_024007
ASHGV40010861	-0.75782	0.757821042	protein_coding	uc001ukq.1
ASHGV40043570	0.757608	0.757608316	protein_coding	NM_000255
ASHGV40003172	-0.7576	0.75760415	protein_coding	NM_001202509
ASHGV40003202	-0.75757	0.757568981	protein_coding	NM_001277090
ASHGV40009771	0.757313	0.757312759	protein_coding	NM_001193451
ASHGV40035223	0.757292	0.757292454	protein_coding	NM_001172779
ASHGV40009095	0.75726	0.757260283	protein_coding	NM_018712
ASHGV40027566	0.757197	0.757196584	protein_coding	NM_000726
ASHGV40008833	0.757167	0.757166921	protein_coding	NM_033388
ASHGV40026623	0.757067	0.757067072	protein_coding	NM_000104
ASHGV40019831	-0.75695	0.756952532	protein_coding	NM_001304947
ASHGV40054996	-0.75687	0.756869793	protein_coding	NM_001031745
ASHGV40029206	-0.75684	0.756836715	protein_coding	NM_015038
ASHGV40042614	-0.75663	0.756632887	protein_coding	NM_017872

ASHGV40047495	-0.75645	0.756452492	protein_coding	NM_001271700
ASHGV40033473	-0.75645	0.756452157	protein_coding	NM_007128
ASHGV40016746	-0.75642	0.756424964	protein_coding	NM_207581
ASHGV40008558	-0.75626	0.756258679	protein_coding	NM_001039752
ASHGV40000230	-0.75623	0.75622997	protein_coding	NM_001164457
ASHGV40044502	0.756178	0.756178428	protein_coding	NM_000904
ASHGV40010368	0.755902	0.755901653	protein_coding	NM_172240
ASHGV40039060	-0.75572	0.755715186	protein_coding	NM_001432
ASHGV40014472	0.755598	0.755597931	protein_coding	NM_032490
ASHGV40046858	0.755348	0.755348195	protein_coding	NM_017984
ASHGV40047119	-0.75534	0.755336877	protein_coding	NM_001708
ASHGV40053337	-0.75527	0.755271817	protein_coding	NM_032888
ASHGV40017537	-0.75524	0.755236231	protein_coding	NM_052966
ASHGV40015827	0.755023	0.755022755	protein_coding	ENST00000324324
ASHGV40044655	0.754985	0.754985103	protein_coding	NM_006366
ASHGV40052252	0.754927	0.754926911	protein_coding	NM_015651
ASHGV40043369	-0.75487	0.754869075	protein_coding	NM_002418
ASHGV40035933	0.754578	0.754578209	protein_coding	NM_006321
ASHGV40050098	-0.7545	0.754502877	protein_coding	NM_145201
ASHGV40009262	-0.7543	0.754295645	protein_coding	NM_012152
ASHGV40008030	-0.75422	0.754215119	protein_coding	ENST00000316517
ASHGV40026949	-0.7542	0.754201726	protein_coding	NM_003198
ASHGV40055430	0.754197	0.754197021	protein_coding	NM_014500
ASHGV40037805	0.75419	0.754190483	protein_coding	NM_021970
ASHGV40047581	0.754079	0.7540787	protein_coding	NM_002214
ASHGV40020294	-0.75399	0.753991731	protein_coding	NM_213656
ASHGV40019431	-0.75386	0.753862016	protein_coding	NM_001129979
ASHGV40040481	-0.7536	0.753604401	protein_coding	NM_001884
ASHGV40034670	-0.75357	0.753567572	protein_coding	NM_022049
ASHGV40019402	0.753545	0.753545486	protein_coding	NM_002811
ASHGV40042240	0.753457	0.753456557	protein_coding	NM_004841
ASHGV40018614	0.753357	0.753357064	protein_coding	NM_022468
ASHGV40008194	-0.75324	0.753236078	protein_coding	NM_014288
ASHGV40047732	-0.75314	0.753141187	protein_coding	NM_014236
ASHGV40030524	0.752916	0.752915705	protein_coding	NM_020341
ASHGV40034220	-0.75268	0.752680507	protein_coding	NM_017886
ASHGV40006127	0.752641	0.752641218	protein_coding	NM_024326
ASHGV40032828	-0.75254	0.752535815	protein_coding	uc002zns.3
ASHGV40017597	0.752412	0.752412257	protein_coding	NM_003470
ASHGV40047391	0.752376	0.752376475	protein_coding	NM_020728
ASHGV40054231	0.752362	0.752362318	protein_coding	NM_080737
ASHGV40044868	0.752333	0.752332908	protein_coding	NM_015090
ASHGV40024446	-0.75233	0.752326596	protein_coding	NM_001712
ASHGV40037426	0.752014	0.752014045	protein_coding	NM_001100399
ASHGV40034275	0.752009	0.752009483	protein_coding	NM_024512
ASHGV40034687	-0.75164	0.751642222	protein_coding	NM_001078
ASHGV40040893	0.751637	0.751637312	protein_coding	NM_004883

ASHGV40034169	-0.75162	0.751623832	protein_coding	NM_033403
ASHGV40006096	0.751535	0.751534818	protein_coding	uc001kru.1
ASHGV40060896	-0.75145	0.751446618	protein_coding	uc021tyq.2
ASHGV40008667	0.75136	0.751360468	protein_coding	NM_173808
ASHGV40007324	-0.75125	0.751250491	protein_coding	NM_001304360
ASHGV40027136	-0.7511	0.751097567	protein_coding	NM_174898
ASHGV40057669	-0.75107	0.751071599	protein_coding	NM_023934
ASHGV40003164	0.751065	0.751065189	protein_coding	NM_152487
ASHGV40044583	0.750352	0.750351688	protein_coding	NM_017906
ASHGV40029306	0.750322	0.750321532	protein_coding	NM_181453
ASHGV40006565	0.750073	0.750072673	protein_coding	NM_000518
ASHGV40050070	-0.74994	0.749938291	protein_coding	NM_020427
ASHGV40006992	-0.74953	0.749525095	protein_coding	NM_004223
ASHGV40046327	-0.74947	0.749466796	protein_coding	NM_006092
ASHGV40026170	-0.74907	0.749071194	protein_coding	ENST00000601382
ASHGV40003305	-0.74882	0.748819371	protein_coding	NM_024070
ASHGV40040912	-0.74856	0.748559259	protein_coding	NM_001085375
ASHGV40010574	0.748472	0.748471932	protein_coding	NM_016433
ASHGV40052249	0.748448	0.748447658	protein_coding	NM_012164
ASHGV40021605	-0.74837	0.748366977	protein_coding	NM_173167
ASHGV40039077	-0.74833	0.748334111	protein_coding	NM_144721
ASHGV40026091	-0.74829	0.748292187	protein_coding	NM_007279
ASHGV40048204	0.748264	0.748264116	protein_coding	NM_201543
ASHGV40037511	-0.74796	0.74796113	protein_coding	NM_002253
ASHGV40055289	0.747744	0.747744016	protein_coding	NM_001128173
ASHGV40001430	0.747674	0.747674148	protein_coding	NM_178566
ASHGV40026045	-0.74718	0.747184261	protein_coding	NM_052925
ASHGV40023986	-0.74718	0.747182662	protein_coding	NM_018154
ASHGV40034136	-0.74718	0.747182626	protein_coding	NM_207359
ASHGV40029035	-0.74711	0.747106448	protein_coding	ENST00000597714
ASHGV40051035	-0.74694	0.746936261	protein_coding	ENST00000522939
ASHGV40003332	-0.74693	0.746928897	protein_coding	NM_130848
ASHGV40030162	-0.74687	0.746867123	protein_coding	NM_182847
ASHGV40029221	0.746812	0.746811763	protein_coding	NM_145212
ASHGV40020523	0.746757	0.746757143	protein_coding	NM_002634
ASHGV40034548	0.746568	0.746568482	protein_coding	NM_001134651
ASHGV40018883	-0.74641	0.746408175	protein_coding	NM_001770
ASHGV40029541	-0.7464	0.746396328	protein_coding	NM_138326
ASHGV40041923	0.74615	0.746150068	protein_coding	NM_000521
ASHGV40029113	0.745985	0.745985163	protein_coding	NM_006634
ASHGV40033992	-0.74595	0.745952177	protein_coding	NM_173472
ASHGV40028810	0.745699	0.745698922	protein_coding	NM_001430
ASHGV40056731	-0.74537	0.745372709	protein_coding	NM_005544
ASHGV40020839	0.745353	0.745352658	protein_coding	NM_181441
ASHGV40028628	0.745247	0.745247123	protein_coding	NM_000183
ASHGV40029111	0.745243	0.745242616	protein_coding	NM_005911
ASHGV40042031	0.745097	0.74509714	protein_coding	NM_006467

ASHGV40008189	0.744967	0.74496746	protein_coding	NM_213599
ASHGV40055829	0.744927	0.744926959	protein_coding	NM_014912
ASHGV40053403	-0.74465	0.744653532	protein_coding	NM_000962
ASHGV40014229	0.744571	0.744570721	protein_coding	NM_004926
ASHGV40014644	0.744395	0.7443947	protein_coding	NM_138420
ASHGV40055206	0.744221	0.744221323	protein_coding	NM_001939
ASHGV40050227	-0.74419	0.744186456	protein_coding	NM_004942
ASHGV40055237	0.744045	0.74404501	protein_coding	NM_032926
ASHGV40023025	0.743995	0.743994558	protein_coding	NM_005151
ASHGV40052601	0.743942	0.743942074	protein_coding	NM_004170
ASHGV40011927	-0.7438	0.743803387	protein_coding	NM_001145199
ASHGV40054180	0.743777	0.743776709	protein_coding	NM_030763
ASHGV40009486	-0.74376	0.743761222	protein_coding	NM_033467
ASHGV40047783	0.743642	0.743641833	protein_coding	NM_002541
ASHGV40049183	-0.74357	0.743569126	protein_coding	NM_001004696
ASHGV40040716	0.743372	0.743371934	protein_coding	NM_004550
ASHGV40051491	-0.74331	0.743307354	protein_coding	ENST00000355513
ASHGV40033515	0.743299	0.743298894	protein_coding	NM_012295
ASHGV40007678	-0.74328	0.743284482	protein_coding	NM_000039
ASHGV40045050	-0.74308	0.743075317	protein_coding	NM_000409
ASHGV40010885	-0.743	0.742999573	protein_coding	NM_032358
ASHGV40016459	-0.74298	0.742976253	protein_coding	NM_014918
ASHGV40001646	0.742706	0.742705645	protein_coding	NM_006294
ASHGV40004951	-0.7427	0.742700946	protein_coding	NM_005264
ASHGV40047494	0.742656	0.742655703	protein_coding	NM_006303
ASHGV40006892	0.742614	0.742613517	protein_coding	NM_006034
ASHGV40027166	0.742594	0.742594317	protein_coding	NM_153836
ASHGV40015104	0.742536	0.74253578	protein_coding	NM_005956
ASHGV40006990	0.742386	0.742385886	protein_coding	NM_024603
ASHGV40003317	-0.74221	0.742208346	protein_coding	NM_032524
ASHGV40055177	-0.7421	0.742104449	protein_coding	ENST00000372950
ASHGV40020284	0.741996	0.741995745	protein_coding	NM_152349
ASHGV40007831	-0.74198	0.741979803	protein_coding	NM_021080
ASHGV40010655	0.741973	0.741973381	protein_coding	NM_018639
ASHGV40019959	-0.74187	0.74187399	protein_coding	NM_007169
ASHGV40025163	-0.74181	0.741812554	protein_coding	NM_018381
ASHGV40024405	0.741797	0.741796556	protein_coding	NM_198476
ASHGV40036379	0.741749	0.741749415	protein_coding	NM_017577
ASHGV40044719	-0.74158	0.741579408	protein_coding	NM_006998
ASHGV40025479	-0.74155	0.741546054	protein_coding	NM_023014
ASHGV40032939	-0.74153	0.741528525	protein_coding	NM_000853
ASHGV40019652	-0.74147	0.741469523	protein_coding	NM_022463
ASHGV40029834	0.74099	0.740990108	protein_coding	NM_019091
ASHGV40011447	-0.74095	0.740945042	protein_coding	NM_134323
ASHGV40020984	-0.74067	0.740674169	protein_coding	NM_178520
ASHGV40015122	0.740588	0.740587877	protein_coding	NM_001024218
ASHGV40057822	-0.74052	0.740518149	protein_coding	NM_032015

ASHGV40000193	-0.74035	0.740348878	protein_coding	NM_000106
ASHGV40037488	0.740222	0.740221783	protein_coding	NM_000232
ASHGV40041176	-0.7401	0.74009767	protein_coding	NM_004387
ASHGV40016507	-0.74003	0.740030061	protein_coding	NM_005664
ASHGV40030461	0.739943	0.739943282	protein_coding	NM_022760
ASHGV40005897	0.739687	0.73968708	protein_coding	NM_001010848
ASHGV40039916	-0.73966	0.739662941	protein_coding	NM_001089584
ASHGV40054958	-0.73966	0.739655613	protein_coding	NM_002668
ASHGV40011065	0.739612	0.73961178	protein_coding	NM_006402
ASHGV40047091	0.739495	0.739495258	protein_coding	NM_000845
ASHGV40009222	-0.73949	0.73948844	protein_coding	NM_006760
ASHGV40047943	0.739076	0.739075624	protein_coding	NM_001012985
ASHGV40010163	-0.73907	0.739065156	protein_coding	NM_152440
ASHGV40050393	0.739037	0.73903669	protein_coding	NM_014265
ASHGV40028702	-0.73902	0.739020735	protein_coding	NM_017964
ASHGV40026956	-0.73866	0.738658763	protein_coding	NM_032779
ASHGV40043424	-0.73842	0.738417441	protein_coding	NM_138493
ASHGV40034415	0.73835	0.738349858	protein_coding	NM_003157
ASHGV40035772	0.73821	0.738210263	protein_coding	NM_012157
ASHGV40046664	-0.73819	0.738194208	protein_coding	NM_152559
ASHGV40035573	-0.7381	0.738104202	protein_coding	NM_014583
ASHGV40052304	-0.73779	0.737786562	protein_coding	NM_033334
ASHGV40042414	-0.73761	0.737607783	protein_coding	NM_031410
ASHGV40045773	0.737242	0.737242091	protein_coding	NM_016108
ASHGV40003326	-0.73705	0.737049697	protein_coding	NM_052841
ASHGV40022206	-0.73698	0.736984964	protein_coding	NM_178233
ASHGV40024920	0.736851	0.736850785	protein_coding	NM_004359
ASHGV40006543	-0.73664	0.736635682	protein_coding	NM_001114938
ASHGV40053992	-0.73657	0.736565843	protein_coding	NM_005834
ASHGV40008113	0.736318	0.736318066	protein_coding	NM_017944
ASHGV40021475	0.736294	0.736293519	protein_coding	NM_021170
ASHGV40010545	-0.73624	0.73624352	protein_coding	NM_001142343
ASHGV40055639	0.736184	0.73618386	protein_coding	ENST00000304790
ASHGV40057824	-0.73609	0.736092314	protein_coding	NM_152304
ASHGV40001357	-0.73608	0.736084853	protein_coding	NM_173160
ASHGV40009983	-0.73606	0.73605691	protein_coding	NM_173086
ASHGV40052723	0.736002	0.736002356	protein_coding	NM_025103
ASHGV40021821	0.735978	0.735978302	protein_coding	NM_001135707
ASHGV40027245	-0.73596	0.735961176	protein_coding	NM_144710
ASHGV40040113	-0.7358	0.73579657	protein_coding	NM_152404
ASHGV40031655	0.735516	0.735515911	protein_coding	NM_007066
ASHGV40008552	-0.73547	0.735465181	protein_coding	NM_000329
ASHGV40041656	-0.73537	0.73537333	protein_coding	NM_000587
ASHGV40046363	0.735315	0.73531493	protein_coding	NM_022373
ASHGV40026150	0.735181	0.735181209	protein_coding	NM_017652
ASHGV40035639	-0.73505	0.735052731	protein_coding	NM_014463
ASHGV40036384	-0.73494	0.734937381	protein_coding	NM_173799

ASHGV40025237	0.734777	0.73477658	protein_coding	NM_004907
ASHGV40015175	0.73465	0.734650043	protein_coding	NM_152331
ASHGV40032574	-0.7345	0.734501092	protein_coding	NM_181616
ASHGV40024320	0.734337	0.734337354	protein_coding	NM_001288800
ASHGV40030878	-0.73427	0.734268032	protein_coding	NM_182970
ASHGV40018148	-0.73425	0.734249289	protein_coding	ENST00000314586
ASHGV40025857	-0.73417	0.734172783	protein_coding	ENST00000535362
ASHGV40012361	-0.73417	0.734167239	protein_coding	NM_207418
ASHGV40052832	0.734049	0.734049204	protein_coding	NM_012203
ASHGV40051624	0.733831	0.733831117	protein_coding	NM_152570
ASHGV40030116	0.733812	0.733811812	protein_coding	NM_014652
ASHGV40014221	0.733714	0.733713512	protein_coding	NM_016026
ASHGV40041020	0.733689	0.733688506	protein_coding	NM_181776
ASHGV40017176	-0.73363	0.733628583	protein_coding	ENST00000568285
ASHGV40002010	0.733534	0.733533834	protein_coding	ENST00000547302
ASHGV40029079	-0.73348	0.733480722	protein_coding	NM_198448
ASHGV40020196	-0.73345	0.733451001	protein_coding	uc010wcm.1
ASHGV40056978	-0.73336	0.733359815	protein_coding	NM_198565
ASHGV40046881	-0.73335	0.733346413	protein_coding	NM_178176
ASHGV40020303	-0.73334	0.733339468	protein_coding	NM_033032
ASHGV40015677	0.733287	0.733286897	protein_coding	NM_153613
ASHGV40020582	-0.7332	0.733195703	protein_coding	NM_018286
ASHGV40044882	-0.73313	0.733131517	protein_coding	NM_001710
ASHGV40013834	-0.73296	0.732956337	protein_coding	NM_022478
ASHGV40046897	0.732952	0.732951612	protein_coding	NM_006234
ASHGV40013061	0.73288	0.732879928	protein_coding	NM_005870
ASHGV40040875	-0.73287	0.732866068	protein_coding	NM_015564
ASHGV40028014	0.732769	0.732768676	protein_coding	NM_005235
ASHGV40053209	-0.73263	0.732629209	protein_coding	NM_024642
ASHGV40010230	0.732361	0.732360799	protein_coding	NM_000110
ASHGV40044769	-0.73231	0.732308464	protein_coding	uc003njg.1
ASHGV40012532	0.732215	0.732215478	protein_coding	NM_012345
ASHGV40028367	0.732097	0.732097272	protein_coding	NM_004300
ASHGV40021793	-0.73207	0.732069128	protein_coding	NM_024032
ASHGV40023945	-0.73196	0.731957879	protein_coding	NM_001930
ASHGV40055581	-0.73195	0.731951093	protein_coding	NM_003639
ASHGV40010721	0.731846	0.731846226	protein_coding	NM_023012
ASHGV40042969	0.731823	0.731822605	protein_coding	NM_001297671
ASHGV40023997	-0.73177	0.731772926	protein_coding	NM_032571
ASHGV40034237	-0.73176	0.731761324	protein_coding	NM_032806
ASHGV40000050	-0.73169	0.731693389	protein_coding	ENST00000312902
ASHGV40018611	-0.73167	0.731671723	protein_coding	NM_024339
ASHGV40009423	-0.73123	0.731232443	protein_coding	NM_001042603
ASHGV40057126	0.731206	0.731205946	protein_coding	NM_033211
ASHGV40023995	0.731119	0.731119128	protein_coding	NM_006145
ASHGV40045911	0.731045	0.731044783	protein_coding	NM_014161
ASHGV40027269	-0.73103	0.731029009	protein_coding	NM_020451

ASHGV40052604	0.730976	0.730976031	protein_coding	NM_017913
ASHGV40056743	-0.73097	0.730969228	protein_coding	NM_182501
ASHGV40020565	0.730643	0.730642837	protein_coding	NM_001130528
ASHGV40057109	-0.73059	0.730587534	protein_coding	ENST00000503079
ASHGV40055744	-0.73048	0.730479136	protein_coding	NM_000429
ASHGV40043504	0.730404	0.730404352	protein_coding	NM_014623
ASHGV40036606	-0.73037	0.730373185	protein_coding	NM_139209
ASHGV40038965	0.730167	0.730166739	protein_coding	NM_006118
ASHGV40028359	-0.73003	0.730030024	protein_coding	NM_032648
ASHGV40012112	-0.72962	0.729616247	protein_coding	NM_032790
ASHGV40024000	-0.72955	0.729547085	protein_coding	uc031rjt.1
ASHGV40046885	0.729458	0.729457632	protein_coding	NM_016068
ASHGV40049810	0.729104	0.72910426	protein_coding	NM_003756
ASHGV40032887	-0.72908	0.729076862	protein_coding	NM_004173
ASHGV40038467	-0.72907	0.729067429	protein_coding	NM_020894
ASHGV40030684	0.729066	0.729066345	protein_coding	NM_001441
ASHGV40012311	0.728842	0.728842254	protein_coding	NM_015974
ASHGV40040813	0.728779	0.72877945	protein_coding	NM_001300816
ASHGV40039006	-0.72876	0.728762925	protein_coding	NM_000200
ASHGV40043916	0.728661	0.728661336	protein_coding	NM_032730
ASHGV40049299	0.72848	0.728480427	protein_coding	NM_015941
ASHGV40021794	-0.72838	0.728380059	protein_coding	NM_080863
ASHGV40019139	0.728178	0.728177519	protein_coding	NM_017839
ASHGV40025889	-0.72807	0.728074744	protein_coding	NM_033378
ASHGV40026417	-0.72796	0.727964853	protein_coding	NM_020905
ASHGV40036604	-0.72776	0.727760621	protein_coding	NM_014245
ASHGV40003249	0.727729	0.727728746	protein_coding	NM_002699
ASHGV40049491	0.727662	0.727661575	protein_coding	NM_000318
ASHGV40020474	-0.72762	0.727620486	protein_coding	NM_002296
ASHGV40017595	-0.72753	0.727527323	protein_coding	NM_014316
ASHGV40047818	-0.72748	0.727475284	protein_coding	NM_001123065
ASHGV40033180	0.727418	0.727418351	protein_coding	NM_000967
ASHGV40009459	-0.72739	0.727388576	protein_coding	ENST00000444507
ASHGV40021754	-0.72735	0.72734502	protein_coding	ENST00000253794
ASHGV40007775	-0.7273	0.727303902	protein_coding	NM_001005467
ASHGV40018319	-0.72726	0.727258236	protein_coding	NM_145168
ASHGV40026768	0.727185	0.727185422	protein_coding	NM_020463
ASHGV40017428	-0.72707	0.727069369	protein_coding	ENST00000442466
ASHGV40025146	0.726984	0.726983736	protein_coding	NM_032497
ASHGV40029801	-0.72695	0.726953503	protein_coding	NM_014621
ASHGV40043503	-0.72669	0.726685121	protein_coding	NM_000287
ASHGV40055564	-0.72645	0.726445362	protein_coding	NM_003492
ASHGV40000191	-0.72638	0.726383363	protein_coding	ENST00000383331
ASHGV40025364	0.726382	0.726381992	protein_coding	NM_024069
ASHGV40027341	0.726315	0.726315195	protein_coding	NM_004455
ASHGV40024669	0.726274	0.72627432	protein_coding	NM_001114598
ASHGV40048233	0.726193	0.726193481	protein_coding	NM_002593

ASHGV40031279	0.725819	0.725818553	protein_coding	NM_001819
ASHGV40051408	-0.72572	0.725718507	protein_coding	ENST00000340210
ASHGV40008246	0.725631	0.725630597	protein_coding	NM_181706
ASHGV40047181	-0.72545	0.725454079	protein_coding	NM_205855
ASHGV40032357	-0.72541	0.725407193	protein_coding	NM_175867
ASHGV40030268	0.725327	0.725326948	protein_coding	NM_025202
ASHGV40010129	-0.7253	0.725302093	protein_coding	NM_153377
ASHGV40023386	-0.72529	0.725290704	protein_coding	NM_018696
ASHGV40034665	-0.72505	0.725054943	protein_coding	NM_015429
ASHGV40035857	-0.72501	0.725006771	protein_coding	NM_001205272
ASHGV40042569	-0.72472	0.724724159	protein_coding	NM_032947
ASHGV40022244	-0.72464	0.724639919	protein_coding	NM_180990
ASHGV40048986	-0.72461	0.72460615	protein_coding	NM_001495
ASHGV40009926	0.724425	0.724424939	protein_coding	NM_005665
ASHGV40040181	-0.72407	0.724072553	protein_coding	NM_001639
ASHGV40042337	-0.72394	0.723942232	protein_coding	NM_021982
ASHGV40047607	0.723819	0.723819453	protein_coding	NM_138446
ASHGV40012591	0.723748	0.723747792	protein_coding	NM_001270424
ASHGV40029081	0.723744	0.723744	protein_coding	NM_001282597
ASHGV40056648	-0.72367	0.723668519	protein_coding	NM_003125
ASHGV40052801	0.723597	0.723596563	protein_coding	NM_006368
ASHGV40015597	-0.72343	0.723429891	protein_coding	NM_030922
ASHGV40006606	0.723415	0.723414948	protein_coding	NM_000391
ASHGV40013287	-0.72339	0.723394933	protein_coding	NM_004128
ASHGV40026612	0.723337	0.723337303	protein_coding	NM_005760
ASHGV40040117	0.723083	0.723082708	protein_coding	uc010iux.3
ASHGV40038809	-0.72301	0.723014412	protein_coding	NM_017581
ASHGV40052786	0.722996	0.72299564	protein_coding	NM_015297
ASHGV40060870	-0.72296	0.722956706	protein_coding	uc010lxs.3
ASHGV40042573	-0.72294	0.722936119	protein_coding	NM_078483
ASHGV40023338	-0.72291	0.722908796	protein_coding	NM_015559
ASHGV40012538	-0.72285	0.722849538	protein_coding	NM_182542
ASHGV40021905	-0.72283	0.722826782	protein_coding	NM_002507
ASHGV40007583	-0.72262	0.722615825	protein_coding	NM_003063
ASHGV40010919	-0.7226	0.722596368	protein_coding	NM_006675
ASHGV40050434	0.722513	0.722512806	protein_coding	NM_006228
ASHGV40056423	0.722178	0.722178407	protein_coding	ENST00000598378
ASHGV40012574	-0.72208	0.722075308	protein_coding	NM_032565
ASHGV40013950	-0.7219	0.721897334	protein_coding	NM_013448
ASHGV40054361	0.721875	0.721875142	protein_coding	NM_001000
ASHGV40050314	0.721822	0.721821882	protein_coding	NM_006197
ASHGV40001314	0.721791	0.721790751	protein_coding	ENST00000469317
ASHGV40028780	-0.72151	0.721513243	protein_coding	NM_020744
ASHGV40033118	0.721433	0.721432628	protein_coding	NM_002854
ASHGV40011734	0.721423	0.721423083	protein_coding	NM_001024383
ASHGV40005266	0.721394	0.721393589	protein_coding	NM_178150
ASHGV40035949	0.721327	0.721326837	protein_coding	NM_001640

ASHGV40047231	-0.72131	0.721305717	protein_coding	NM_013252
ASHGV40023706	-0.72131	0.721305488	protein_coding	NM_032737
ASHGV40021622	0.721241	0.721241484	protein_coding	NM_014765
ASHGV40029752	-0.72107	0.721066754	protein_coding	NM_138995
ASHGV40046455	0.720953	0.720952836	protein_coding	NM_033224
ASHGV40012179	0.720925	0.720924966	protein_coding	NM_023928
ASHGV40034071	-0.7207	0.720701275	protein_coding	NM_001252657
ASHGV40054357	0.720678	0.720677777	protein_coding	NM_032776
ASHGV40010053	-0.72067	0.720667317	protein_coding	NM_032364
ASHGV40006025	-0.72067	0.720665581	protein_coding	NM_001001732
ASHGV40005252	-0.72065	0.720649838	protein_coding	NM_001353
ASHGV40018893	-0.72058	0.720584476	protein_coding	NM_014298
ASHGV40032744	-0.72053	0.720528741	protein_coding	NM_014589
ASHGV40007453	0.72037	0.720369735	protein_coding	NM_016126
ASHGV40043595	-0.72018	0.720184664	protein_coding	NM_002388
ASHGV40006752	-0.72017	0.720170128	protein_coding	NM_022725
ASHGV40025685	-0.7201	0.720102671	protein_coding	NM_182704
ASHGV40006738	-0.72003	0.720032799	protein_coding	NM_001029865
ASHGV40055404	-0.71992	0.71992259	protein_coding	NM_032458
ASHGV40054945	0.719921	0.719921136	protein_coding	NM_006743
ASHGV40007688	-0.71989	0.719894706	protein_coding	uc001pri.1
ASHGV40029365	0.719876	0.719876347	protein_coding	NM_012184
ASHGV40044617	0.719794	0.719793724	protein_coding	NM_012241
ASHGV40006498	-0.71973	0.71972896	protein_coding	NM_001012708
ASHGV40044681	-0.71973	0.719726848	protein_coding	NM_001949
ASHGV40054911	0.719639	0.719639066	protein_coding	NM_004683
ASHGV40054435	-0.71959	0.719587229	protein_coding	NM_018388
ASHGV40006601	-0.71955	0.719552354	protein_coding	NM_000613
ASHGV40016714	0.719548	0.719548146	protein_coding	NM_003825
ASHGV40007282	-0.7195	0.719495535	protein_coding	NM_005247
ASHGV40014359	-0.71945	0.71944927	protein_coding	NM_005621
ASHGV40037098	0.719435	0.719434653	protein_coding	NM_133474
ASHGV40004945	0.718999	0.71899902	protein_coding	NM_018017
ASHGV40020530	0.718636	0.718636388	protein_coding	NM_003563
ASHGV40010068	0.71853	0.71852999	protein_coding	NM_014255
ASHGV40052250	0.718514	0.718513861	protein_coding	NM_002211
ASHGV40049564	-0.71828	0.718275097	protein_coding	NM_001738
ASHGV40009060	-0.71823	0.718227836	protein_coding	NM_032930
ASHGV40031664	-0.71806	0.718063885	protein_coding	NM_001124756
ASHGV40053549	-0.71804	0.718043784	protein_coding	NM_005085
ASHGV40015006	0.718	0.718000233	protein_coding	NM_006568
ASHGV40023781	0.717948	0.717947866	protein_coding	NM_007322
ASHGV40017545	-0.71792	0.717921472	protein_coding	NM_032444
ASHGV40049649	0.717575	0.717575372	protein_coding	NM_015496
ASHGV40035305	0.717546	0.717546011	protein_coding	NM_145261
ASHGV40024992	-0.71754	0.717537503	protein_coding	NM_152988
ASHGV40040294	0.717526	0.71752584	protein_coding	uc003jss.4

ASHGV40051836	0.717478	0.717477975	protein_coding	NM_001025780
ASHGV40021355	-0.71728	0.717276145	protein_coding	NM_000676
ASHGV40030346	0.717175	0.71717463	protein_coding	NM_005855
ASHGV40024433	0.717049	0.71704863	protein_coding	NM_006423
ASHGV40045090	-0.71677	0.716768398	protein_coding	NM_018426
ASHGV40020875	0.716735	0.716735165	protein_coding	NM_012478
ASHGV40047397	-0.71672	0.716719647	protein_coding	NM_003382
ASHGV40039435	-0.71623	0.716225518	protein_coding	NM_153702
ASHGV40050985	0.716225	0.716224509	protein_coding	NM_002380
ASHGV40002675	-0.71616	0.716159303	protein_coding	ENST00000599428
ASHGV40010859	-0.71587	0.715871962	protein_coding	NM_001195520
ASHGV40045127	-0.71571	0.715705012	protein_coding	uc003oyz.1
ASHGV40034066	0.715622	0.715621705	protein_coding	NM_002971
ASHGV40022774	0.7154	0.715399738	protein_coding	NM_001199355
ASHGV40021014	-0.7154	0.715398243	protein_coding	NM_001145113
ASHGV40031514	-0.71538	0.715379201	protein_coding	NM_005093
ASHGV40008818	0.715083	0.715083264	protein_coding	NM_005482
ASHGV40042408	-0.71506	0.715063106	protein_coding	NM_018502
ASHGV40036460	0.715055	0.715055497	protein_coding	NM_003947
ASHGV40022315	-0.71498	0.714983056	protein_coding	NM_005189
ASHGV40007798	-0.71497	0.71496794	protein_coding	NM_212555
ASHGV40009228	0.714967	0.714966757	protein_coding	NM_021729
ASHGV40024749	0.714952	0.714951613	protein_coding	NM_018355
ASHGV40010133	-0.71494	0.714944177	protein_coding	ENST00000548613
ASHGV40035064	0.714903	0.714902551	protein_coding	NM_003071
ASHGV40031970	-0.71489	0.714894579	protein_coding	NM_000913
ASHGV40024514	-0.71481	0.714808522	protein_coding	NM_198478
ASHGV40027916	0.714746	0.714745654	protein_coding	NM_173822
ASHGV40001006	-0.71472	0.714721894	protein_coding	ENST00000445861
ASHGV40029395	0.714709	0.714708933	protein_coding	NM_005857
ASHGV40018197	-0.71463	0.714633621	protein_coding	NM_014062
ASHGV40056076	-0.71439	0.714388046	protein_coding	NM_001004723
ASHGV40001841	-0.71437	0.714366028	protein_coding	ENST00000529411
ASHGV40052914	0.714325	0.714324838	protein_coding	NM_001098805
ASHGV40020089	-0.7143	0.714300222	protein_coding	NM_018182
ASHGV40037920	-0.71421	0.714214269	protein_coding	NM_001001701
ASHGV40000555	-0.71412	0.714121748	protein_coding	ENST00000423213
ASHGV40013212	-0.71406	0.714057226	protein_coding	ENST00000379848
ASHGV40050716	0.713946	0.713945561	protein_coding	NM_002865
ASHGV40019423	0.713802	0.713801653	protein_coding	NM_033401
ASHGV40013030	-0.71365	0.713650262	protein_coding	NM_005399
ASHGV40037942	0.713624	0.713624141	protein_coding	NM_001154
ASHGV40024353	-0.71333	0.713331772	protein_coding	NM_007181
ASHGV40008501	-0.71322	0.713224154	protein_coding	NM_206893
ASHGV40020932	0.713224	0.713223915	protein_coding	NM_007267
ASHGV40010459	0.713048	0.713047865	protein_coding	NM_152788
ASHGV40017341	-0.71302	0.713023324	protein_coding	NM_173499

ASHGV40048025	-0.71291	0.712910063	protein_coding	NM_198853
ASHGV40021500	-0.7129	0.712902413	protein_coding	NM_000984
ASHGV40053508	-0.71263	0.712634124	protein_coding	NM_015354
ASHGV40033221	-0.71262	0.712624691	protein_coding	uc003bcg.3
ASHGV40048037	-0.71254	0.712543002	protein_coding	NM_020892
ASHGV40031479	-0.71241	0.712408932	protein_coding	NM_001008409
ASHGV40037988	0.712341	0.712340682	protein_coding	NM_006320
ASHGV40019526	-0.71231	0.712306014	protein_coding	NM_014615
ASHGV40027023	-0.71229	0.712290527	protein_coding	NM_001031738
ASHGV40014089	-0.71222	0.712223795	protein_coding	NM_005776
ASHGV40036672	0.712187	0.712186626	protein_coding	NM_016275
ASHGV40041204	0.712114	0.712114089	protein_coding	NM_000794
ASHGV40017630	0.711881	0.71188069	protein_coding	NM_014153
ASHGV40030793	-0.71169	0.711692688	protein_coding	NM_152925
ASHGV40028909	0.711685	0.711684895	protein_coding	NM_152516
ASHGV40017627	-0.71168	0.71168105	protein_coding	NM_004862
ASHGV40052526	0.711552	0.711551811	protein_coding	NM_015392
ASHGV40006684	-0.71124	0.711241759	protein_coding	NM_012250
ASHGV40054607	-0.7111	0.711095088	protein_coding	NM_001080449
ASHGV40010567	0.710996	0.710995802	protein_coding	NM_052845
ASHGV40013851	0.71076	0.71076025	protein_coding	NM_032452
ASHGV40035349	0.710567	0.710567419	protein_coding	NM_005787
ASHGV40042452	-0.71048	0.710484974	protein_coding	NM_018916
ASHGV40025175	-0.71034	0.710339329	protein_coding	NM_013319
ASHGV40021918	-0.71032	0.710318921	protein_coding	NM_000023
ASHGV40018196	0.710177	0.710176641	protein_coding	NM_000903
ASHGV40003300	-0.71003	0.710025158	protein_coding	NM_021649
ASHGV40032917	-0.70987	0.709869255	protein_coding	NM_014433
ASHGV40043703	-0.70975	0.709750172	protein_coding	NM_001402
ASHGV40033011	0.70971	0.709709512	protein_coding	NM_001127
ASHGV40033282	-0.70965	0.709652698	protein_coding	NM_006071
ASHGV40014126	0.709591	0.709590887	protein_coding	NM_001001872
ASHGV40018285	-0.70951	0.709507009	protein_coding	NM_199355
ASHGV40026114	-0.70939	0.709392104	protein_coding	NM_152478
ASHGV40027619	0.709262	0.709262133	protein_coding	NM_002349
ASHGV40021279	-0.70923	0.709227418	protein_coding	NM_004822
ASHGV40052094	0.709227	0.709227081	protein_coding	NM_015051
ASHGV40040572	0.709213	0.709212967	protein_coding	NM_000439
ASHGV40012840	0.709202	0.709201772	protein_coding	NM_198968
ASHGV40028824	0.709094	0.709094353	protein_coding	NM_020458
ASHGV40015785	-0.70894	0.70894193	protein_coding	NM_001114134
ASHGV40034056	0.708836	0.708835773	protein_coding	NM_015150
ASHGV40056485	0.708667	0.708666843	protein_coding	NM_018083
ASHGV40052003	0.708567	0.708566614	protein_coding	NM_015250
ASHGV40031355	0.708562	0.708562043	protein_coding	NM_178477
ASHGV40053878	-0.70851	0.70850657	protein_coding	NM_025159
ASHGV40025759	-0.70812	0.708122928	protein_coding	NM_032341

ASHGV40035625	0.708116	0.708116385	protein_coding	NM_000560
ASHGV40037847	0.708029	0.708028692	protein_coding	NM_001290768
ASHGV40042822	-0.70757	0.707570397	protein_coding	NM_004499
ASHGV40033124	-0.70757	0.707567456	protein_coding	NM_153609
ASHGV40038793	0.70752	0.707519508	protein_coding	NM_015990
ASHGV40005908	-0.70728	0.707275545	protein_coding	NM_018999
ASHGV40036452	-0.70727	0.707274548	protein_coding	NM_012430
ASHGV40051662	-0.70727	0.707272657	protein_coding	NM_001170
ASHGV40009657	0.707214	0.707213549	protein_coding	NM_052941
ASHGV40033424	-0.70718	0.707177046	protein_coding	NM_002882
ASHGV40014354	0.707174	0.707174106	protein_coding	NM_012245
ASHGV40018312	0.707056	0.707056011	protein_coding	NM_020188
ASHGV40017431	-0.70697	0.706970176	protein_coding	NM_058192
ASHGV40021666	0.706952	0.706952344	protein_coding	NM_002795
ASHGV40019660	-0.70689	0.706893058	protein_coding	NM_001080779
ASHGV40011856	-0.70684	0.706842026	protein_coding	uc001teq.2
ASHGV40019736	0.706486	0.706485968	protein_coding	NM_005022
ASHGV40046313	0.70648	0.706479915	protein_coding	NM_152740
ASHGV40022345	-0.70646	0.706460218	protein_coding	NM_001291324
ASHGV40037639	-0.70635	0.706349014	protein_coding	NM_001729
ASHGV40038188	-0.70628	0.706283694	protein_coding	NM_001008393
ASHGV40055494	-0.70625	0.706246389	protein_coding	NM_002025
ASHGV40045256	0.706226	0.706226257	protein_coding	NM_006212
ASHGV40013969	0.70613	0.70613035	protein_coding	NM_016586
ASHGV40011534	-0.70607	0.706068018	protein_coding	NM_002898
ASHGV40020674	-0.70603	0.706030359	protein_coding	NM_152598
ASHGV40048676	-0.70571	0.705705673	protein_coding	NM_000603
ASHGV40036671	0.705619	0.705618605	protein_coding	NM_032025
ASHGV40029655	-0.70541	0.705406119	protein_coding	NM_152522
ASHGV40037650	0.7054	0.705399642	protein_coding	NM_003948
ASHGV40015143	-0.70537	0.705374679	protein_coding	NM_001161498
ASHGV40053582	0.705302	0.705302348	protein_coding	NM_014694
ASHGV40050787	-0.70516	0.705162899	protein_coding	NM_015170
ASHGV40043043	0.705099	0.705099171	protein_coding	NM_005493
ASHGV40050067	-0.70474	0.704736478	protein_coding	NM_003724
ASHGV40003259	0.704561	0.70456118	protein_coding	NM_004158
ASHGV40052899	0.704394	0.704393855	protein_coding	ENST00000417488
ASHGV40023735	0.704295	0.704294843	protein_coding	NM_001348
ASHGV40043736	0.704237	0.704236982	protein_coding	NM_004242
ASHGV40006256	-0.70422	0.704216113	protein_coding	NM_001242699
ASHGV40044349	-0.70421	0.704206055	protein_coding	ENST00000419182
ASHGV40057847	-0.70415	0.704149961	protein_coding	ENST00000538173
ASHGV40024459	-0.70404	0.704039025	protein_coding	NM_002780
ASHGV40050016	-0.70387	0.703874987	protein_coding	uc011ljo.2
ASHGV40026484	0.703751	0.703751379	protein_coding	NM_002254
ASHGV40043700	-0.70366	0.703659432	protein_coding	NM_001080507
ASHGV40041417	0.703513	0.703512593	protein_coding	NM_015325

ASHGV40011137	0.703261	0.703260868	protein_coding	NM_018372
ASHGV40015625	0.703223	0.703223119	protein_coding	NM_004371
ASHGV40012362	0.703136	0.703136163	protein_coding	NM_004685
ASHGV40030997	0.70287	0.702870337	protein_coding	NM_002237
ASHGV40050365	-0.70276	0.702757013	protein_coding	NM_021174
ASHGV40025520	-0.70262	0.702623342	protein_coding	NM_001105570
ASHGV40053863	-0.70255	0.70254695	protein_coding	NM_138932
ASHGV40052990	-0.70236	0.7023589	protein_coding	NM_001013735
ASHGV40023938	-0.70235	0.702351357	protein_coding	NM_152601
ASHGV40056042	0.702194	0.702193982	protein_coding	NM_021645
ASHGV40003292	0.70215	0.702150167	protein_coding	NM_018997
ASHGV40016953	0.701947	0.701947192	protein_coding	NM_001143688
ASHGV40030024	0.701911	0.701910657	protein_coding	NM_020923
ASHGV40042572	0.70171	0.701709753	protein_coding	NM_000405
ASHGV40017524	-0.70161	0.701608339	protein_coding	NM_001103175
ASHGV40025484	-0.7016	0.701603796	protein_coding	ENST00000330881
ASHGV40015301	-0.70155	0.701550648	protein_coding	NM_018319
ASHGV40014429	-0.70147	0.701465901	protein_coding	NM_007039
ASHGV40025647	0.701439	0.701439368	protein_coding	NM_021102
ASHGV40032348	0.701436	0.701436243	protein_coding	NM_000100
ASHGV40043597	-0.70141	0.70141352	protein_coding	NM_012288
ASHGV40013323	-0.70139	0.701394299	protein_coding	NM_138450
ASHGV40029242	0.70119	0.701189574	protein_coding	NM_002518
ASHGV40036595	0.700915	0.70091525	protein_coding	NM_001104647
ASHGV40030320	-0.70089	0.700889432	protein_coding	NM_020311
ASHGV40041230	-0.7007	0.700698795	protein_coding	NM_004395
ASHGV40021950	0.70069	0.700690221	protein_coding	NM_000269
ASHGV40007238	-0.70059	0.700594158	protein_coding	NM_000695
ASHGV40030264	-0.70037	0.700368494	protein_coding	NM_000751
ASHGV40029743	-0.70032	0.70031992	protein_coding	NM_006347
ASHGV40000170	0.700309	0.700309254	protein_coding	NM_016154
ASHGV40037719	-0.7003	0.700303823	protein_coding	NM_015697
ASHGV40027995	-0.70024	0.700237667	protein_coding	NM_014617
ASHGV40044135	0.700194	0.700194372	protein_coding	NM_001278724
ASHGV40036514	0.700114	0.700113776	protein_coding	NM_020187
ASHGV40051680	0.699849	0.699848609	protein_coding	NM_198573
ASHGV40025089	-0.69968	0.699677563	protein_coding	NM_001136507
ASHGV40044867	0.699625	0.699625323	protein_coding	NM_001623
ASHGV40006019	-0.69959	0.699586903	protein_coding	NM_207321
ASHGV40018489	0.699399	0.699399211	protein_coding	NM_014700
ASHGV40043936	-0.69933	0.699330867	protein_coding	NM_173672
ASHGV40012847	-0.69916	0.699163932	protein_coding	NM_080818
ASHGV40048115	0.699111	0.699111246	protein_coding	NM_033107
ASHGV40002459	0.69872	0.698719568	protein_coding	NM_001025300
ASHGV40005393	-0.69872	0.698716486	protein_coding	NM_012443
ASHGV40053466	0.698648	0.698648248	protein_coding	NM_032293
ASHGV40056077	-0.69836	0.698361655	protein_coding	NM_001004063

ASHGV40018948	0.698048	0.698048168	protein_coding	NM_004604
ASHGV40010381	-0.69793	0.697934022	protein_coding	NM_152638
ASHGV40007787	-0.69786	0.697859695	protein_coding	NM_138961
ASHGV40029969	0.697828	0.697828114	protein_coding	NM_015535
ASHGV40001315	-0.69778	0.697777848	protein_coding	NM_178571
ASHGV40001924	0.697776	0.697775816	protein_coding	NM_005687
ASHGV40037808	0.697715	0.697714506	protein_coding	NM_002106
ASHGV40038194	-0.69763	0.697629006	protein_coding	NM_001145415
ASHGV40039280	0.697444	0.697443613	protein_coding	NM_018569
ASHGV40035970	-0.69739	0.697393879	protein_coding	NM_016173
ASHGV40005933	-0.69727	0.697270953	protein_coding	NM_133447
ASHGV40047205	0.697209	0.697209245	protein_coding	NM_022740
ASHGV40004999	0.697102	0.697102191	protein_coding	NM_002925
ASHGV40057174	-0.69697	0.696968054	protein_coding	NM_207582
ASHGV40018988	-0.69648	0.69648357	protein_coding	ENST00000398666
ASHGV40031521	0.696324	0.696324111	protein_coding	NM_176812
ASHGV40019168	-0.69629	0.696294805	protein_coding	NM_000078
ASHGV40031589	-0.69627	0.696271572	protein_coding	NM_001164431
ASHGV40005327	0.69626	0.696260432	protein_coding	NM_024640
ASHGV40015982	-0.69623	0.696233209	protein_coding	NM_178550
ASHGV40046930	-0.69597	0.69597003	protein_coding	NM_020725
ASHGV40056908	-0.69596	0.695961108	protein_coding	NM_000992
ASHGV40005637	0.695863	0.695862824	protein_coding	ENST00000311663
ASHGV40025854	-0.69565	0.695653181	protein_coding	NM_014601
ASHGV40056184	0.69555	0.695549676	protein_coding	NM_004748
ASHGV40033976	-0.69535	0.695345719	protein_coding	NM_000916
ASHGV40020656	0.695313	0.695313008	protein_coding	NM_001282476
ASHGV40056431	-0.69523	0.695233058	protein_coding	uc021ufe.1
ASHGV40016146	0.694994	0.694994173	protein_coding	NM_000696
ASHGV40008674	0.69494	0.694939537	protein_coding	NM_006842
ASHGV40043351	0.694917	0.694917283	protein_coding	NM_005452
ASHGV40057860	-0.6949	0.694902721	protein_coding	ENST00000398092
ASHGV40029346	0.69489	0.69488995	protein_coding	NM_005415
ASHGV40019166	-0.69485	0.694848787	protein_coding	NM_001126107
ASHGV40041606	0.694844	0.694844498	protein_coding	NM_001012339
ASHGV40026814	0.694756	0.694756313	protein_coding	NM_003400
ASHGV40054133	0.69472	0.694719587	protein_coding	NM_005436
ASHGV40045059	-0.69456	0.69455593	protein_coding	NM_138296
ASHGV40005074	0.694547	0.694547356	protein_coding	NM_014408
ASHGV40030246	0.694438	0.694438147	protein_coding	NM_002807
ASHGV40022200	-0.69439	0.694390182	protein_coding	NM_001006638
ASHGV40027721	0.694303	0.694302653	protein_coding	NM_003111
ASHGV40007273	-0.69428	0.694279012	protein_coding	uc021qmp.1
ASHGV40032149	-0.69428	0.694275414	protein_coding	NM_001128598
ASHGV40024939	-0.69421	0.694205364	protein_coding	NM_002777
ASHGV40049899	0.694199	0.694199207	protein_coding	NM_032026
ASHGV40030049	0.694143	0.69414257	protein_coding	NM_015040

ASHGV40038430	-0.69411	0.694107369	protein_coding	NM_020770
ASHGV40030779	0.694082	0.694081716	protein_coding	NM_000178
ASHGV40005226	0.694069	0.694068727	protein_coding	NM_001145210
ASHGV40009404	0.694004	0.694004289	protein_coding	NM_001007022
ASHGV40048284	0.693863	0.693862755	protein_coding	NM_199000
ASHGV40018967	0.693344	0.693343673	protein_coding	NM_003041
ASHGV40033487	-0.69333	0.693329811	protein_coding	NM_004914
ASHGV40018913	-0.69325	0.693253928	protein_coding	NM_002720
ASHGV40023020	-0.69318	0.693180513	protein_coding	NM_032510
ASHGV40053154	-0.69315	0.693146981	protein_coding	NM_133446
ASHGV40035964	0.692989	0.692988807	protein_coding	NM_004636
ASHGV40006686	-0.69294	0.692937346	protein_coding	NM_001097611
ASHGV40028194	0.692812	0.692811689	protein_coding	NM_005381
ASHGV40021786	-0.69277	0.692768983	protein_coding	ENST00000588043
ASHGV40055356	-0.69274	0.692743827	protein_coding	ENST00000595757
ASHGV40047321	0.692716	0.692716006	protein_coding	NM_014280
ASHGV40056339	0.692576	0.692576302	protein_coding	NM_001101387
ASHGV40026187	0.692546	0.692546463	protein_coding	NM_015677
ASHGV40026129	-0.69251	0.692509423	protein_coding	NM_001015878
ASHGV40014784	0.692499	0.692498673	protein_coding	NM_001199839
ASHGV40013942	0.692163	0.692162706	protein_coding	NM_138288
ASHGV40017248	0.692043	0.692042733	protein_coding	NM_020212
ASHGV40031693	-0.69201	0.692006144	protein_coding	NM_080603
ASHGV40024774	-0.6918	0.691803868	protein_coding	ENST00000376591
ASHGV40050048	-0.69178	0.69178194	protein_coding	NM_207414
ASHGV40016697	0.691759	0.691758532	protein_coding	NM_002220
ASHGV40032211	0.691192	0.691192473	protein_coding	NM_145858
ASHGV40046660	-0.69099	0.690994035	protein_coding	NM_032951
ASHGV40035750	0.690843	0.690843248	protein_coding	NM_001688
ASHGV40015873	-0.6908	0.69079841	protein_coding	NM_138792
ASHGV40025160	-0.69071	0.690705328	protein_coding	ENST00000171214
ASHGV40006574	-0.69059	0.690589754	protein_coding	NM_033179
ASHGV40032373	-0.69053	0.690528605	protein_coding	NM_181686
ASHGV40036408	0.690517	0.690516884	protein_coding	NM_006699
ASHGV40014873	-0.6905	0.690496824	protein_coding	ENST00000553330
ASHGV40009894	-0.69039	0.690394707	protein_coding	NM_015401
ASHGV40050614	0.690383	0.690383112	protein_coding	NM_003350
ASHGV40015007	0.690207	0.690207315	protein_coding	NM_005920
ASHGV40048286	-0.69017	0.690174212	protein_coding	NM_018682
ASHGV40054623	-0.69008	0.690076104	protein_coding	NM_002414
ASHGV40028642	0.689897	0.689897335	protein_coding	NM_000221
ASHGV40006451	0.689881	0.689881342	protein_coding	NM_012239
ASHGV40013062	0.689836	0.689835955	protein_coding	uc021rhd.1
ASHGV40024439	-0.68976	0.68976057	protein_coding	NM_006494
ASHGV40037857	-0.6895	0.689498593	protein_coding	NM_016269
ASHGV40030506	-0.68935	0.689346703	protein_coding	NM_152611
ASHGV40008267	0.689323	0.689323493	protein_coding	NM_005734

ASHGV40042273	0.689297	0.689296518	protein_coding	NM_032446
ASHGV40055953	0.688793	0.688793415	protein_coding	NM_001001412
ASHGV40021140	0.688611	0.688610618	protein_coding	NM_144611
ASHGV40015788	-0.68859	0.688589209	protein_coding	NM_014793
ASHGV40033668	0.688548	0.688548003	protein_coding	NM_014310
ASHGV40021813	0.688526	0.688525721	protein_coding	NM_002390
ASHGV40038099	0.688461	0.688460942	protein_coding	NM_004425
ASHGV40015733	-0.68842	0.688424169	protein_coding	NM_001039905
ASHGV40009595	-0.68818	0.68818157	protein_coding	NM_003651
ASHGV40018497	0.688118	0.688117581	protein_coding	NM_138418
ASHGV40047135	0.687961	0.687961486	protein_coding	NM_003344
ASHGV40019544	-0.68778	0.687782835	protein_coding	NM_001451
ASHGV40044230	0.6876	0.687600141	protein_coding	NM_139126
ASHGV40032838	-0.6875	0.687495065	protein_coding	NM_005984
ASHGV40033905	0.687447	0.687446756	protein_coding	NM_024324
ASHGV40025596	-0.68734	0.687339564	protein_coding	NM_052948
ASHGV40009325	0.687323	0.687323458	protein_coding	NM_004879
ASHGV40047695	-0.68732	0.687319743	protein_coding	ENST00000311067
ASHGV40009516	0.687312	0.687312305	protein_coding	NM_005439
ASHGV40024531	0.687242	0.687241931	protein_coding	NM_004597
ASHGV40024654	-0.68717	0.68717093	protein_coding	NM_152899
ASHGV40053396	-0.68688	0.686882497	protein_coding	ENST00000249598
ASHGV40025370	-0.68687	0.686865303	protein_coding	NM_018316
ASHGV40004949	0.6867	0.686699653	protein_coding	NM_002313
ASHGV40007715	-0.68669	0.686690142	protein_coding	NM_001467
ASHGV40044870	-0.68668	0.68667538	protein_coding	NM_019101
ASHGV40016136	-0.68667	0.686674018	protein_coding	NM_000499
ASHGV40006198	0.686512	0.686512167	protein_coding	NM_007373
ASHGV40035487	0.686447	0.686446727	protein_coding	NM_003234
ASHGV40010618	0.686389	0.686389147	protein_coding	ENST00000425410
ASHGV40054774	0.686284	0.686284008	protein_coding	NM_020150
ASHGV40015435	-0.68628	0.686280316	protein_coding	NM_207117
ASHGV40048095	-0.68625	0.686248217	protein_coding	NM_006716
ASHGV40052440	0.686015	0.686014848	protein_coding	NM_003172
ASHGV40014559	0.68597	0.685970405	protein_coding	NM_032233
ASHGV40046435	0.685875	0.685875413	protein_coding	NM_001077663
ASHGV40028091	-0.68568	0.685683953	protein_coding	NM_024536
ASHGV40010488	-0.68555	0.685545253	protein_coding	NM_024057
ASHGV40006703	0.685482	0.685481996	protein_coding	NM_002645
ASHGV40025324	0.685426	0.685426107	protein_coding	NM_023937
ASHGV40040733	-0.68536	0.685364924	protein_coding	NM_000943
ASHGV40024534	-0.68523	0.685228966	protein_coding	NM_004409
ASHGV40014345	-0.68513	0.685125538	protein_coding	NM_013382
ASHGV40039637	0.685106	0.685106378	protein_coding	NM_017423
ASHGV40018747	-0.68483	0.684826781	protein_coding	NM_198447
ASHGV40011893	-0.68473	0.684731787	protein_coding	NM_014503
ASHGV40056059	0.684581	0.684581209	protein_coding	NM_004951

ASHGV40020022	-0.68458	0.684578486	protein_coding	NM_003876
ASHGV40055472	0.68433	0.684329951	protein_coding	NM_001012989
ASHGV40040568	0.684073	0.684072639	protein_coding	NM_002064
ASHGV40051324	0.683795	0.683794595	protein_coding	NM_001010911
ASHGV40040192	-0.6837	0.683700772	protein_coding	NM_004465
ASHGV40030389	0.683695	0.683695054	protein_coding	NM_001033575
ASHGV40042681	0.683584	0.683584406	protein_coding	NM_001122679
ASHGV40034855	-0.68354	0.683539776	protein_coding	NM_152533
ASHGV40013200	-0.6835	0.68349751	protein_coding	NM_002915
ASHGV40012698	-0.68347	0.683465831	protein_coding	ENST00000359684
ASHGV40008857	-0.68346	0.683457031	protein_coding	NM_006591
ASHGV40023807	0.683344	0.683343989	protein_coding	NM_080662
ASHGV40020886	0.683128	0.683128313	protein_coding	NM_014230
ASHGV40012125	0.682942	0.682942364	protein_coding	NM_021794
ASHGV40034383	0.682936	0.682935857	protein_coding	NM_001174051
ASHGV40022073	0.682867	0.682867013	protein_coding	uc021ubm.2
ASHGV40018012	0.682719	0.682719422	protein_coding	NM_024336
ASHGV40014769	-0.68264	0.682636128	protein_coding	NM_005015
ASHGV40053890	0.682522	0.682522325	protein_coding	NM_015235
ASHGV40013618	0.682462	0.682461645	protein_coding	NM_004800
ASHGV40049099	-0.68236	0.682356548	protein_coding	NM_031271
ASHGV40036268	0.682211	0.682210548	protein_coding	NM_006100
ASHGV40007921	-0.68206	0.682061864	protein_coding	ENST00000450448
ASHGV40010049	-0.6819	0.681900019	protein_coding	NM_000350
ASHGV40013972	-0.6818	0.681797242	protein_coding	NM_014360
ASHGV40023692	-0.68176	0.681760786	protein_coding	NM_001130111
ASHGV40013469	0.681722	0.681721506	protein_coding	NM_005358
ASHGV40034955	-0.68168	0.68168319	protein_coding	NM_005862
ASHGV40029250	-0.68167	0.681672696	protein_coding	NM_001288706
ASHGV40028173	0.68162	0.681619571	protein_coding	NM_017933
ASHGV40022058	0.681589	0.681589129	protein_coding	NM_018488
ASHGV40019323	0.681583	0.681582647	protein_coding	NM_006599
ASHGV40026184	0.681424	0.681423625	protein_coding	NM_001077710
ASHGV40048519	-0.6812	0.681196487	protein_coding	ENST00000416501
ASHGV40012434	-0.68108	0.681076812	protein_coding	NM_001136571
ASHGV40014824	-0.68101	0.681011526	protein_coding	NM_004554
ASHGV40010356	0.680955	0.680955295	protein_coding	NM_025114
ASHGV40029364	0.680896	0.680896196	protein_coding	NM_172003
ASHGV40036611	0.680837	0.680837401	protein_coding	NM_001080415
ASHGV40049747	0.680765	0.680764617	protein_coding	NM_013437
ASHGV40019665	0.680719	0.680718766	protein_coding	NM_031430
ASHGV40022134	-0.68064	0.680635146	protein_coding	NM_002758
ASHGV40015171	-0.68063	0.680625079	protein_coding	NM_173462
ASHGV40007051	0.680515	0.680514884	protein_coding	NM_001923
ASHGV40015025	-0.68049	0.680488562	protein_coding	NM_021255
ASHGV40014039	-0.6804	0.680404978	protein_coding	uc010tqj.3
ASHGV40025711	-0.68038	0.680383334	protein_coding	NM_033440

ASHGV40015604	0.680373	0.680372628	protein_coding	NM_002857
ASHGV40003268	-0.68029	0.680290208	protein_coding	NM_005227
ASHGV40002114	-0.68018	0.680175325	protein_coding	ENST00000555109
ASHGV40051259	-0.68016	0.680155591	protein_coding	NM_016018
ASHGV40047661	0.679981	0.679981199	protein_coding	NM_004067
ASHGV40054559	0.679971	0.67997083	protein_coding	NM_022079
ASHGV40038659	-0.67993	0.67993133	protein_coding	NM_025205
ASHGV40053975	-0.67976	0.679755166	protein_coding	uc004dip.3
ASHGV40055068	0.679454	0.679453898	protein_coding	NM_017626
ASHGV40039050	-0.67939	0.679386651	protein_coding	NM_002993
ASHGV40050130	0.67911	0.679110264	protein_coding	NM_001494
ASHGV40025760	-0.67904	0.679044288	protein_coding	NM_001145641
ASHGV40037113	0.679023	0.679022569	protein_coding	NM_005255
ASHGV40047364	-0.67889	0.67888921	protein_coding	NM_000193
ASHGV40033804	-0.67889	0.67888816	protein_coding	NM_000714
ASHGV40012810	-0.6788	0.678795784	protein_coding	NM_007053
ASHGV40030715	-0.67878	0.678777776	protein_coding	ENST00000376403
ASHGV40050917	0.678746	0.678745802	protein_coding	NM_003821
ASHGV40016976	0.678614	0.678613642	protein_coding	NM_015322
ASHGV40003341	-0.67853	0.678525875	protein_coding	NM_172089
ASHGV40053883	0.678472	0.678472421	protein_coding	NM_004015
ASHGV40023194	-0.67843	0.678429442	protein_coding	NM_001142966
ASHGV40048276	0.678124	0.678124128	protein_coding	NM_031905
ASHGV40032852	-0.67809	0.67809492	protein_coding	NM_006440
ASHGV40045209	0.678092	0.678091615	protein_coding	NM_001031623
ASHGV40011326	-0.67798	0.677975966	protein_coding	NM_181788
ASHGV40057656	-0.67796	0.677959729	protein_coding	NM_021048
ASHGV40019674	-0.67788	0.67788012	protein_coding	NM_017575
ASHGV40029279	0.677768	0.677768398	protein_coding	NM_024093
ASHGV40035717	0.677768	0.677767655	protein_coding	NM_017897
ASHGV40005886	-0.67747	0.677471814	protein_coding	NM_032372
ASHGV40033603	0.677433	0.677433407	protein_coding	NM_012429
ASHGV40020702	0.677173	0.677172611	protein_coding	NM_003607
ASHGV40010716	-0.67708	0.677081253	protein_coding	NM_019887
ASHGV40002603	-0.67695	0.676948237	protein_coding	ENST00000594684
ASHGV40043935	0.676767	0.676767463	protein_coding	NM_006016
ASHGV40019274	-0.67672	0.676719307	protein_coding	NM_001129727
ASHGV40042979	0.676589	0.67658922	protein_coding	NM_052965
ASHGV40042881	-0.67657	0.676569513	protein_coding	uc003mns.2
ASHGV40008150	0.67655	0.676549579	protein_coding	NM_001202439
ASHGV40003178	-0.67651	0.676512759	protein_coding	NM_005118
ASHGV40033531	0.676465	0.676465475	protein_coding	NM_001039948
ASHGV40037242	0.676284	0.676283936	protein_coding	NM_001017979
ASHGV40028831	0.676211	0.67621066	protein_coding	NM_000251
ASHGV40050717	-0.6762	0.676198481	protein_coding	NM_017780
ASHGV40048456	0.676161	0.676160649	protein_coding	NM_001195243
ASHGV40041758	-0.67608	0.67608183	protein_coding	NM_002021

ASHGV40019653	0.676073	0.676073494	protein_coding	NM_021962
ASHGV40043381	0.676072	0.676072092	protein_coding	NM_012391
ASHGV40011916	-0.67604	0.676041125	protein_coding	NM_003211
ASHGV40022802	0.676033	0.676033261	protein_coding	NM_003927
ASHGV40019845	0.676004	0.676004227	protein_coding	NM_004853
ASHGV40002496	-0.67595	0.675946571	protein_coding	ENST00000585661
ASHGV40010613	-0.67594	0.67594194	protein_coding	NM_022363
ASHGV40028324	0.675923	0.675923217	protein_coding	NM_006559
ASHGV40050412	0.675804	0.675803994	protein_coding	NM_004331
ASHGV40004961	0.675492	0.675491753	protein_coding	NM_173791
ASHGV40018483	-0.67538	0.675378117	protein_coding	NM_014176
ASHGV40003301	-0.67532	0.675316917	protein_coding	NM_022661
ASHGV40011063	0.675298	0.675297893	protein_coding	NM_001271592
ASHGV40047195	-0.67495	0.674953555	protein_coding	NM_020632
ASHGV40020346	0.674916	0.674915962	protein_coding	NM_004583
ASHGV40037648	0.674744	0.674743714	protein_coding	NM_015436
ASHGV40055020	0.674582	0.674581624	protein_coding	NM_013444
ASHGV40053677	-0.67453	0.674529659	protein_coding	NM_001177316
ASHGV40030094	-0.67452	0.674515717	protein_coding	NM_057091
ASHGV40051991	0.67403	0.674030052	protein_coding	NM_006415
ASHGV40005029	0.673927	0.673926947	protein_coding	NM_001271840
ASHGV40008453	0.673895	0.673895007	protein_coding	NM_170746
ASHGV40037250	-0.67376	0.673764061	protein_coding	uc003gnh.1
ASHGV40028882	0.673593	0.673592516	protein_coding	NM_006296
ASHGV40042057	-0.67351	0.673505541	protein_coding	NM_005654
ASHGV40054723	-0.67344	0.673440429	protein_coding	NM_007220
ASHGV40011024	-0.67329	0.673293201	protein_coding	NM_020734
ASHGV40033222	-0.67321	0.673208862	protein_coding	NM_005650
ASHGV40051423	-0.67316	0.673157185	protein_coding	NM_014665
ASHGV40015882	0.67311	0.673109906	protein_coding	NM_001286495
ASHGV40006486	0.672956	0.672956299	protein_coding	NM_021128
ASHGV40024943	-0.67293	0.672931223	protein_coding	NM_005224
ASHGV40000518	-0.67278	0.672782759	protein_coding	NM_001037501
ASHGV40021124	-0.67273	0.67272829	protein_coding	NM_012352
ASHGV40023157	0.672224	0.672224239	protein_coding	NM_001279
ASHGV40015376	-0.67222	0.672223199	protein_coding	ENST00000554161
ASHGV40026823	0.672136	0.672135964	protein_coding	NM_198276
ASHGV40008802	-0.67211	0.672114237	protein_coding	NM_021046
ASHGV40055900	0.672111	0.672110623	protein_coding	NM_001278
ASHGV40026141	-0.67187	0.671865659	protein_coding	NM_024691
ASHGV40026138	-0.67176	0.671759014	protein_coding	NM_006959
ASHGV40035209	0.671672	0.671672269	protein_coding	NM_007217
ASHGV40005634	-0.67155	0.671548206	protein_coding	NM_003055
ASHGV40036732	-0.67154	0.671543759	protein_coding	NM_001130002
ASHGV40014370	-0.67138	0.671380193	protein_coding	NM_152446
ASHGV40018272	0.671361	0.671361479	protein_coding	NM_005548
ASHGV40009462	-0.67122	0.671224181	protein_coding	NM_020367

ASHGV40057503	0.671062	0.671061985	protein_coding	NM_001004125
ASHGV40035828	-0.67065	0.670653895	protein_coding	NM_024494
ASHGV40033691	0.670565	0.670564786	protein_coding	NM_001282685
ASHGV40028901	0.670564	0.67056402	protein_coding	NM_152392
ASHGV40029336	0.67056	0.670560155	protein_coding	NM_006343
ASHGV40054373	0.670503	0.670503001	protein_coding	NM_152692
ASHGV40045057	-0.67048	0.670475501	protein_coding	NM_001123168
ASHGV40052329	-0.67042	0.670419875	protein_coding	NM_022833
ASHGV40045360	0.670274	0.670274312	protein_coding	NM_012381
ASHGV40003081	0.670209	0.670208837	protein_coding	NM_016283
ASHGV40015388	-0.67004	0.670044641	protein_coding	NM_001282463
ASHGV40030339	-0.66988	0.669884594	protein_coding	NM_015893
ASHGV40054968	-0.66985	0.669847159	protein_coding	NM_001127212
ASHGV40005013	0.669717	0.66971701	protein_coding	NM_000141
ASHGV40034265	0.669548	0.669547697	protein_coding	NM_020208
ASHGV40048600	-0.66937	0.669370761	protein_coding	NM_001004685
ASHGV40057207	-0.66922	0.669223735	protein_coding	NM_002701
ASHGV40016135	-0.6692	0.669202298	protein_coding	NM_025083
ASHGV40013454	0.669161	0.669161336	protein_coding	NM_006346
ASHGV40013072	0.669091	0.669090947	protein_coding	NM_002010
ASHGV40042469	-0.66906	0.669056058	protein_coding	NM_003735
ASHGV40011002	0.668852	0.668851995	protein_coding	NM_014718
ASHGV40023762	0.668796	0.668796454	protein_coding	NM_005817
ASHGV40021502	-0.66827	0.668265653	protein_coding	NM_178170
ASHGV40057558	0.668243	0.66824323	protein_coding	NM_001037293
ASHGV40051994	0.668233	0.668232845	protein_coding	NM_002161
ASHGV40040886	-0.66817	0.668165477	protein_coding	NM_198282
ASHGV40046022	0.668098	0.668098134	protein_coding	NM_001042552
ASHGV40011491	0.668059	0.668059272	protein_coding	NM_020710
ASHGV40039124	-0.66776	0.667758406	protein_coding	NM_152494
ASHGV40035492	0.667689	0.667689261	protein_coding	NM_005017
ASHGV40029030	-0.66768	0.667681396	protein_coding	NM_080916
ASHGV40010732	0.667572	0.667571847	protein_coding	NM_004642
ASHGV40045968	-0.66749	0.667490308	protein_coding	ENST00000598601
ASHGV40029990	-0.66727	0.667266142	protein_coding	ENST00000594829
ASHGV40042438	-0.66717	0.667170599	protein_coding	NM_173509
ASHGV40001238	0.667006	0.667005583	protein_coding	NM_018443
ASHGV40034052	0.666935	0.666935064	protein_coding	NM_015199
ASHGV40031668	-0.66681	0.666814872	protein_coding	NM_002638
ASHGV40037750	0.666808	0.666808034	protein_coding	NM_152542
ASHGV40029103	0.666697	0.666696939	protein_coding	NM_020122
ASHGV40027327	0.666652	0.666651992	protein_coding	NM_001017927
ASHGV40005822	0.666532	0.666532447	protein_coding	NM_006721
ASHGV40034471	0.666513	0.666512938	protein_coding	NM_000925
ASHGV40033595	-0.66647	0.666473424	protein_coding	NM_182527
ASHGV40009221	-0.66642	0.666419179	protein_coding	NM_001716
ASHGV40021871	0.666384	0.666384264	protein_coding	NM_003204

ASHGV40012318	0.666375	0.666375232	protein_coding	NM_153251
ASHGV40051464	0.666119	0.666118518	protein_coding	NM_134428
ASHGV40021230	-0.66592	0.665919458	protein_coding	ENST00000593717
ASHGV40029403	-0.66588	0.665884303	protein_coding	NM_018234
ASHGV40003315	-0.66578	0.665782707	protein_coding	NM_032414
ASHGV40052126	-0.66573	0.665733324	protein_coding	NM_001001956
ASHGV40023634	-0.66565	0.665652228	protein_coding	NM_012283
ASHGV40014576	0.665585	0.665585264	protein_coding	NM_001134888
ASHGV40035915	-0.66558	0.665581703	protein_coding	NM_015466
ASHGV40055711	0.665408	0.665408006	protein_coding	NM_014893
ASHGV40018140	0.665159	0.665159165	protein_coding	NM_003905
ASHGV40028358	-0.66511	0.66510888	protein_coding	NM_003757
ASHGV40030777	0.664876	0.664875808	protein_coding	NM_178026
ASHGV40032721	-0.66465	0.664654992	protein_coding	NM_004571
ASHGV40011161	-0.66445	0.664453129	protein_coding	ENST00000599478
ASHGV40054257	0.664396	0.664396073	protein_coding	NM_001006938
ASHGV40000935	-0.66434	0.664344244	protein_coding	ENST00000441853
ASHGV40024407	-0.66427	0.664269601	protein_coding	uc002opk.1
ASHGV40044907	0.664158	0.664157604	protein_coding	NM_002800
ASHGV40027195	0.664107	0.664107021	protein_coding	NM_014313
ASHGV40010114	-0.66395	0.663952505	protein_coding	NM_005371
ASHGV40042984	-0.66377	0.66376794	protein_coding	NM_030810
ASHGV40000787	-0.66361	0.663606555	protein_coding	NM_030805
ASHGV40046603	0.663506	0.663506163	protein_coding	NM_178558
ASHGV40018503	0.663343	0.663343444	protein_coding	NM_005861
ASHGV40044977	0.663294	0.663293982	protein_coding	NM_003017
ASHGV40033711	0.663261	0.663261091	protein_coding	NM_014291
ASHGV40054364	0.662974	0.662974397	protein_coding	NM_006978
ASHGV40024869	-0.66292	0.662921127	protein_coding	NM_020633
ASHGV40031676	-0.66285	0.662851763	protein_coding	NM_033542
ASHGV40014778	-0.66283	0.662827056	protein_coding	NM_001099780
ASHGV40034398	-0.66282	0.662816842	protein_coding	NM_032750
ASHGV40055873	0.662699	0.662699075	protein_coding	NM_022362
ASHGV40014091	0.66247	0.662469544	protein_coding	NM_004124
ASHGV40041212	0.662462	0.662461608	protein_coding	NM_016391
ASHGV40013049	0.66246	0.662460304	protein_coding	NM_016361
ASHGV40030163	-0.66244	0.662438556	protein_coding	NM_001303098
ASHGV40026989	-0.66242	0.662423127	protein_coding	NM_002580
ASHGV40042449	-0.66242	0.662416872	protein_coding	NM_018935
ASHGV40013877	-0.66239	0.662385371	protein_coding	NM_001039771
ASHGV40053195	0.662341	0.662340935	protein_coding	NM_003275
ASHGV40054726	-0.66232	0.66232432	protein_coding	NM_004057
ASHGV40040148	-0.66218	0.662177717	protein_coding	NM_001343
ASHGV40010091	-0.66212	0.662122534	protein_coding	NM_014830
ASHGV40025094	-0.6619	0.661900952	protein_coding	ENST00000576789
ASHGV40043898	0.661814	0.661813954	protein_coding	NM_020771
ASHGV40046776	0.661789	0.661789272	protein_coding	NM_000466

ASHGV40043608	-0.66178	0.66178141	protein_coding	uc003pbg.3
ASHGV40035115	0.661532	0.661531933	protein_coding	NM_020865
ASHGV40045584	0.661508	0.661507941	protein_coding	NM_002269
ASHGV40040959	0.661503	0.661502607	protein_coding	NM_020117
ASHGV40039091	0.661477	0.661477051	protein_coding	NM_018243
ASHGV40023175	-0.6614	0.66140335	protein_coding	NM_005913
ASHGV40010576	0.661285	0.66128516	protein_coding	NM_057169
ASHGV40052172	-0.66128	0.661282167	protein_coding	NM_001003936
ASHGV40017541	0.661236	0.661236245	protein_coding	NM_152457
ASHGV40057176	-0.66121	0.661210429	protein_coding	NM_138574
ASHGV40023870	0.661046	0.66104587	protein_coding	NM_003755
ASHGV40028764	-0.66101	0.661007696	protein_coding	NM_024772
ASHGV40049303	0.66098	0.660979544	protein_coding	NM_006756
ASHGV40048203	0.660726	0.660726224	protein_coding	NM_138494
ASHGV40032798	0.660596	0.660595823	protein_coding	NM_006031
ASHGV40007175	0.660584	0.660584471	protein_coding	NM_138417
ASHGV40044919	0.660518	0.660517652	protein_coding	NM_003782
ASHGV40020304	-0.6605	0.660500464	protein_coding	NM_001165252
ASHGV40033230	-0.66042	0.660416059	protein_coding	NM_017436
ASHGV40054773	0.660371	0.660371167	protein_coding	NM_005391
ASHGV40030225	-0.66028	0.660282661	protein_coding	NM_178821
ASHGV40011548	-0.6598	0.659798936	protein_coding	NM_031479
ASHGV40007087	0.659785	0.65978472	protein_coding	NM_001297665
ASHGV40043918	-0.65969	0.659692114	protein_coding	NM_001080450
ASHGV40023933	-0.65965	0.659648454	protein_coding	NM_030824
ASHGV40016336	0.659265	0.659264836	protein_coding	NM_018670
ASHGV40054359	0.659	0.659000105	protein_coding	NM_017544
ASHGV40012317	-0.65884	0.658839845	protein_coding	NM_145061
ASHGV40005153	0.658795	0.65879454	protein_coding	NM_017971
ASHGV40021519	0.658724	0.658724208	protein_coding	NM_020791
ASHGV40036749	-0.65847	0.658467754	protein_coding	NM_005496
ASHGV40045661	0.658117	0.658116534	protein_coding	NM_016377
ASHGV40021544	-0.65803	0.658030035	protein_coding	NM_032322
ASHGV40052289	0.657995	0.657994652	protein_coding	NM_018387
ASHGV40015686	-0.65762	0.657621841	protein_coding	NM_020660
ASHGV40024983	0.657551	0.657550662	protein_coding	NM_001039846
ASHGV40002620	-0.65735	0.657352498	protein_coding	ENST00000595956
ASHGV40040142	0.657344	0.657344113	protein_coding	NM_152756
ASHGV40010952	-0.65725	0.657249475	protein_coding	NM_002527
ASHGV40036006	-0.65723	0.657232155	protein_coding	NM_002215
ASHGV40035291	-0.65709	0.657093715	protein_coding	NM_021629
ASHGV40049826	0.657033	0.657032586	protein_coding	NM_207506
ASHGV40050597	-0.65701	0.65700641	protein_coding	NM_032237
ASHGV40041525	-0.65699	0.656992978	protein_coding	ENST00000296682
ASHGV40020615	0.656937	0.656936669	protein_coding	NM_152608
ASHGV40043989	0.656867	0.656866998	protein_coding	NM_153612
ASHGV40007917	0.65685	0.656850201	protein_coding	NM_001293167

ASHGV40055650	-0.65671	0.656707291	protein_coding	NM_001282471
ASHGV40020077	0.656679	0.656678687	protein_coding	NM_014680
ASHGV40040971	-0.65665	0.656649764	protein_coding	NM_006182
ASHGV40047165	0.656638	0.656638364	protein_coding	NM_017812
ASHGV40039265	-0.65632	0.656322913	protein_coding	NM_006583
ASHGV40032715	-0.65623	0.65622574	protein_coding	NM_018964
ASHGV40025233	0.656104	0.65610435	protein_coding	NM_002501
ASHGV40050959	0.656022	0.656021587	protein_coding	NM_181787
ASHGV40000026	-0.6559	0.655896112	protein_coding	ENST00000277540
ASHGV40046447	0.655801	0.655801216	protein_coding	NM_015332
ASHGV40026515	0.655793	0.655792857	protein_coding	NM_001034116
ASHGV40010864	-0.65527	0.655268196	protein_coding	NM_005895
ASHGV40035471	0.655163	0.655162868	protein_coding	NM_006241
ASHGV40032186	-0.65508	0.655075347	protein_coding	NM_144659
ASHGV40019170	-0.65482	0.654821665	protein_coding	NM_032206
ASHGV40034828	0.654818	0.654818114	protein_coding	NM_017578
ASHGV40047613	-0.65476	0.654759587	protein_coding	NM_031414
ASHGV40053634	0.654663	0.654663301	protein_coding	NM_001276451
ASHGV40024976	-0.65464	0.654642267	protein_coding	NM_138422
ASHGV40016311	0.654385	0.654385215	protein_coding	NM_022163
ASHGV40018598	-0.65412	0.654120416	protein_coding	NM_145252
ASHGV40031303	-0.65399	0.653986671	protein_coding	NM_022096
ASHGV40025682	0.653761	0.653761451	protein_coding	NM_003169
ASHGV40056048	-0.65373	0.653729633	protein_coding	NM_199342
ASHGV40029754	0.653688	0.653687728	protein_coding	NM_001289947
ASHGV40016232	0.653627	0.653626949	protein_coding	NM_181900
ASHGV40023783	-0.65353	0.653529602	protein_coding	uc002med.1
ASHGV40031256	-0.65341	0.653408447	protein_coding	NM_052970
ASHGV40008392	-0.65324	0.653235976	protein_coding	ENST00000545087
ASHGV40026902	0.65313	0.653129704	protein_coding	NM_017880
ASHGV40006152	0.653003	0.653003065	protein_coding	NM_014720
ASHGV40006206	0.652915	0.652915144	protein_coding	NM_058222
ASHGV40027717	-0.65283	0.652831122	protein_coding	NM_032125
ASHGV40001268	-0.65276	0.652761269	protein_coding	ENST00000481050
ASHGV40017848	-0.65261	0.652612282	protein_coding	NM_012248
ASHGV40005632	-0.65244	0.652444855	protein_coding	NM_001135196
ASHGV40038434	0.652423	0.652423361	protein_coding	NM_030918
ASHGV40020414	0.652363	0.652363249	protein_coding	NM_000419
ASHGV40047990	-0.65223	0.652226115	protein_coding	NM_003602
ASHGV40032188	0.652187	0.652187107	protein_coding	ENST00000431216
ASHGV40030783	0.652179	0.652178547	protein_coding	NM_178033
ASHGV40042583	-0.65218	0.65217678	protein_coding	NM_004736
ASHGV40014453	-0.65216	0.65215844	protein_coding	NM_003485
ASHGV40035901	0.652007	0.652007259	protein_coding	ENST00000599511
ASHGV40024273	-0.65197	0.6519686	protein_coding	NM_152481
ASHGV40046237	-0.65195	0.651947724	protein_coding	NM_018719
ASHGV40052308	0.65187	0.651869767	protein_coding	NM_002077

ASHGV40019751	-0.65166	0.651664337	protein_coding	NM_016041
ASHGV40021891	0.651591	0.65159063	protein_coding	NM_005831
ASHGV40017835	0.651303	0.651303029	protein_coding	NM_002746
ASHGV40031898	0.651272	0.651271565	protein_coding	NM_144703
ASHGV40057683	0.651243	0.651242524	protein_coding	ENST00000395721
ASHGV40009940	0.651092	0.651092042	protein_coding	NM_001006605
ASHGV40022314	-0.651	0.650997535	protein_coding	ENST00000328313
ASHGV40033802	-0.6509	0.650896491	protein_coding	NM_001197
ASHGV40037910	0.650896	0.65089602	protein_coding	NM_152402
ASHGV40014342	0.650876	0.650876018	protein_coding	NM_174976
ASHGV40024085	0.650871	0.650871024	protein_coding	NM_012321
ASHGV40049037	-0.65085	0.650854749	protein_coding	NM_001083111
ASHGV40035862	0.650827	0.650827071	protein_coding	NM_017719
ASHGV40012219	0.65078	0.650780333	protein_coding	NM_001136103
ASHGV40020707	0.650612	0.650611627	protein_coding	NM_001288732
ASHGV40052505	-0.65045	0.650447066	protein_coding	NM_006412
ASHGV40003261	0.650432	0.650432242	protein_coding	NM_004417
ASHGV40034274	-0.65043	0.650427431	protein_coding	NM_002343
ASHGV40002856	-0.65034	0.650340384	protein_coding	NM_001251
ASHGV40010863	0.650196	0.650195931	protein_coding	NM_015114
ASHGV40020036	-0.65018	0.650177236	protein_coding	NM_006610
ASHGV40031675	-0.65004	0.650040625	protein_coding	NM_014276
ASHGV40028152	-0.64987	0.649872024	protein_coding	NM_000092
ASHGV40047665	0.649759	0.649759113	protein_coding	NM_001080529
ASHGV40035825	0.649517	0.649517073	protein_coding	NM_005875
ASHGV40016083	-0.64943	0.649428805	protein_coding	NM_001102658
ASHGV40036820	-0.64937	0.649369911	protein_coding	ENST00000598405
ASHGV40010311	0.649312	0.649312031	protein_coding	NM_001037317
ASHGV40015061	0.648969	0.648968892	protein_coding	NM_016475
ASHGV40003162	-0.64885	0.648853199	protein_coding	NM_001199577
ASHGV40025516	0.648845	0.648845346	protein_coding	NM_207325
ASHGV40037061	0.648802	0.648801758	protein_coding	NM_001105573
ASHGV40049577	-0.64856	0.648558568	protein_coding	NM_138817
ASHGV40024122	-0.64851	0.648509126	protein_coding	NM_033196
ASHGV40054037	-0.6485	0.648504224	protein_coding	NM_004493
ASHGV40030902	-0.6479	0.647899391	protein_coding	NM_080614
ASHGV40054566	0.647832	0.647832046	protein_coding	NM_005745
ASHGV40024231	-0.64768	0.647679001	protein_coding	NM_033103
ASHGV40032697	-0.64756	0.647558689	protein_coding	NM_002463
ASHGV40008982	-0.64753	0.647528617	protein_coding	NM_005467
ASHGV40046817	-0.64735	0.647351604	protein_coding	NM_006188
ASHGV40009624	0.647245	0.647245267	protein_coding	ENST00000381800
ASHGV40045098	0.647177	0.647176978	protein_coding	NM_001253
ASHGV40044923	0.647121	0.647121178	protein_coding	NM_002636
ASHGV40011855	-0.64709	0.64708522	protein_coding	NM_005230
ASHGV40036579	0.64707	0.647069845	protein_coding	NM_012219
ASHGV40057616	-0.64705	0.647046373	protein_coding	NM_001024455

ASHGV40012500	0.646861	0.646861379	protein_coding	NM_032138
ASHGV40050672	-0.64669	0.646689274	protein_coding	ENST00000520251
ASHGV40024050	-0.64664	0.646640448	protein_coding	NM_033417
ASHGV40022566	0.646554	0.646553523	protein_coding	NM_138340
ASHGV40010429	0.646377	0.646377402	protein_coding	NM_000895
ASHGV40041320	-0.64632	0.646321888	protein_coding	NM_002697
ASHGV40037777	0.646186	0.646185685	protein_coding	NM_003728
ASHGV40043713	-0.64615	0.646150072	protein_coding	NM_001865
ASHGV40019806	-0.64599	0.645985249	protein_coding	NM_032356
ASHGV40043423	-0.64588	0.645878341	protein_coding	NM_145316
ASHGV40029386	0.645747	0.645746907	protein_coding	NM_006773
ASHGV40008819	-0.64559	0.645591483	protein_coding	NM_000802
ASHGV40056563	0.645561	0.645561103	protein_coding	NM_004069
ASHGV40008514	-0.64552	0.645517836	protein_coding	ENST00000524968
ASHGV40055708	-0.64503	0.645031308	protein_coding	NM_004202
ASHGV40029133	0.644962	0.644962272	protein_coding	NM_018433
ASHGV40012738	-0.64489	0.644891069	protein_coding	NM_001310140
ASHGV40006013	0.644704	0.644704475	protein_coding	NM_015188
ASHGV40054761	-0.64466	0.644660416	protein_coding	NM_173495
ASHGV40026474	0.644594	0.644593948	protein_coding	NM_016544
ASHGV40047172	-0.64434	0.64433831	protein_coding	ENST00000452718
ASHGV40036012	0.6441	0.644100328	protein_coding	NM_006254
ASHGV40030582	0.64402	0.644020398	protein_coding	NM_004587
ASHGV40049318	0.644001	0.644000753	protein_coding	NM_001023
ASHGV40028664	0.643802	0.643801844	protein_coding	NM_199191
ASHGV40043871	-0.64377	0.643773397	protein_coding	NM_017421
ASHGV40005937	-0.64371	0.643711753	protein_coding	NM_024503
ASHGV40034321	-0.64364	0.643639182	protein_coding	NM_004157
ASHGV40002164	-0.64338	0.643378752	protein_coding	ENST00000558378
ASHGV40008844	0.643368	0.643367807	protein_coding	NM_016055
ASHGV40046369	0.643239	0.643238658	protein_coding	NM_138794
ASHGV40027680	0.643153	0.643152713	protein_coding	NM_024622
ASHGV40037139	0.64312	0.643119531	protein_coding	NM_012318
ASHGV40024104	0.643095	0.643095148	protein_coding	NM_001145784
ASHGV40047174	0.643048	0.643048155	protein_coding	NM_014149
ASHGV40008596	-0.64302	0.643017416	protein_coding	NM_033310
ASHGV40034517	0.642972	0.642972241	protein_coding	NM_004742
ASHGV40022383	0.64296	0.642959788	protein_coding	NM_024619
ASHGV40056370	-0.64292	0.642915841	protein_coding	NM_001001417
ASHGV40018840	0.642849	0.642849108	protein_coding	NM_016309
ASHGV40046692	-0.6428	0.64279536	protein_coding	NM_032890
ASHGV40016943	-0.64277	0.642768257	protein_coding	NM_001101362
ASHGV40029148	-0.64271	0.642706679	protein_coding	NM_152670
ASHGV40009350	0.642674	0.642674212	protein_coding	NM_152890
ASHGV40027997	-0.64251	0.642509947	protein_coding	NM_005896
ASHGV40001377	-0.64206	0.642064129	protein_coding	NM_001016
ASHGV40056273	-0.64186	0.64185937	protein_coding	NM_024044

ASHGV40040860	0.641852	0.641851703	protein_coding	NM_016603
ASHGV40007357	0.641836	0.641835982	protein_coding	NM_004041
ASHGV40024058	-0.64175	0.641747405	protein_coding	NM_020959
ASHGV40011236	-0.64167	0.641666394	protein_coding	NM_032834
ASHGV40018426	-0.64148	0.641478713	protein_coding	NM_002461
ASHGV40006618	0.641435	0.64143535	protein_coding	NM_003629
ASHGV40025076	0.641419	0.641418908	protein_coding	NM_032306
ASHGV40020426	0.641124	0.641124225	protein_coding	NM_006688
ASHGV40041470	-0.64084	0.64083867	protein_coding	NM_031916
ASHGV40000740	0.64077	0.640770407	protein_coding	NM_003748
ASHGV40000032	-0.64066	0.640655267	protein_coding	uc009vue.3
ASHGV40039726	0.640621	0.640620772	protein_coding	NM_020225
ASHGV40002013	-0.64045	0.640453374	protein_coding	ENST00000547423
ASHGV40041137	0.640295	0.640295337	protein_coding	NM_003062
ASHGV40056610	-0.64029	0.640293129	protein_coding	NM_024575
ASHGV40055984	0.640203	0.640202706	protein_coding	NM_016122
ASHGV40024953	0.640113	0.640113297	protein_coding	NM_001687
ASHGV40029112	-0.64009	0.640092701	protein_coding	NM_003761
ASHGV40052980	-0.64006	0.640055232	protein_coding	NM_001190482
ASHGV40050331	-0.64005	0.640046177	protein_coding	NM_000237
ASHGV40000611	-0.64002	0.640020524	protein_coding	ENST00000425492
ASHGV40043373	0.639831	0.639831399	protein_coding	NM_000841
ASHGV40041964	-0.6398	0.639797966	protein_coding	NM_153610
ASHGV40055532	-0.63977	0.639773051	protein_coding	NM_144589
ASHGV40039235	-0.63972	0.639722508	protein_coding	NM_001031720
ASHGV40038565	-0.63972	0.639717438	protein_coding	NM_005547
ASHGV40009185	0.639583	0.639583298	protein_coding	NM_002572
ASHGV40041789	-0.63952	0.639521712	protein_coding	NM_152687
ASHGV40035248	-0.63946	0.639458616	protein_coding	NM_003810
ASHGV40052971	0.639273	0.639272832	protein_coding	NM_006914
ASHGV40057059	-0.63918	0.639184448	protein_coding	NM_145720
ASHGV40023670	0.639114	0.639114381	protein_coding	NM_002695
ASHGV40048132	-0.63906	0.639058966	protein_coding	uc001hxp.1
ASHGV40048382	0.638976	0.638975841	protein_coding	NM_057168
ASHGV40047693	-0.63894	0.63893548	protein_coding	NM_198428
ASHGV40057522	0.63885	0.63884975	protein_coding	uc010mnd.3
ASHGV40037837	0.638819	0.638819099	protein_coding	NM_176869
ASHGV40023352	0.638767	0.638766765	protein_coding	NM_031303
ASHGV40004983	0.638765	0.638765093	protein_coding	NM_153810
ASHGV40020147	0.638636	0.638636231	protein_coding	NM_022344
ASHGV40021546	0.638483	0.638483298	protein_coding	NM_001042492
ASHGV40009489	-0.63824	0.638239074	protein_coding	NM_001278596
ASHGV40047191	0.63823	0.638230078	protein_coding	NM_002825
ASHGV40039021	0.638226	0.638226377	protein_coding	NM_020368
ASHGV40027881	0.637993	0.637992971	protein_coding	NM_018897
ASHGV40025621	0.637951	0.637950918	protein_coding	NM_001300979
ASHGV40031694	0.637676	0.637676244	protein_coding	NM_001127695

ASHGV40028814	0.637611	0.637610791	protein_coding	NM_014171
ASHGV40030056	-0.6372	0.637195538	protein_coding	NM_199229
ASHGV40011374	-0.63714	0.637144058	protein_coding	ENST00000598429
ASHGV40048380	-0.63685	0.636849359	protein_coding	NM_024913
ASHGV40044281	0.636798	0.636797576	protein_coding	NM_006335
ASHGV40049430	-0.63676	0.636755919	protein_coding	ENST00000512294
ASHGV40019163	-0.63674	0.636736267	protein_coding	NM_014669
ASHGV40030433	-0.63663	0.636632475	protein_coding	NM_080725
ASHGV40051047	-0.63661	0.636605139	protein_coding	NM_015420
ASHGV40028709	-0.63656	0.636559925	protein_coding	NM_005268
ASHGV40020360	-0.63654	0.63653611	protein_coding	NM_016556
ASHGV40037800	0.636391	0.63639065	protein_coding	NM_152292
ASHGV40014771	0.636318	0.636317945	protein_coding	NM_004995
ASHGV40048060	0.636224	0.636223514	protein_coding	NM_002069
ASHGV40006814	-0.63615	0.636154494	protein_coding	NM_001198551
ASHGV40014259	-0.63569	0.635691702	protein_coding	ENST00000594636
ASHGV40034170	0.635603	0.635602587	protein_coding	NM_014831
ASHGV40043272	-0.63559	0.635593731	protein_coding	NM_021160
ASHGV40052103	0.635564	0.635563684	protein_coding	NM_133445
ASHGV40034988	-0.63544	0.635439964	protein_coding	NM_001130992
ASHGV40041239	0.635386	0.635386018	protein_coding	NM_173663
ASHGV40043084	0.635327	0.635327	protein_coding	NM_003472
ASHGV40041903	-0.63528	0.635280584	protein_coding	NM_153217
ASHGV40002621	-0.63519	0.635185632	protein_coding	ENST00000595994
ASHGV40032658	0.635172	0.635172147	protein_coding	NM_001396
ASHGV40052833	0.635095	0.635094675	protein_coding	NM_001282862
ASHGV40055539	0.634988	0.634987748	protein_coding	uc022chp.1
ASHGV40014075	0.634969	0.634969076	protein_coding	NM_014584
ASHGV40003156	-0.63485	0.634854817	protein_coding	NM_001198974
ASHGV40013666	0.634642	0.634641733	protein_coding	NM_006573
ASHGV40047284	0.63463	0.634630223	protein_coding	NM_152411
ASHGV40012476	-0.63453	0.634530336	protein_coding	NM_145286
ASHGV40021589	-0.63445	0.634446118	protein_coding	NM_002986
ASHGV40036964	-0.63418	0.634176072	protein_coding	NM_005578
ASHGV40047185	0.634164	0.634164183	protein_coding	NM_145808
ASHGV40029326	-0.63416	0.634158866	protein_coding	NM_001136493
ASHGV40028078	-0.63414	0.634141174	protein_coding	NM_015680
ASHGV40040116	0.63406	0.634059972	protein_coding	NM_001007527
ASHGV40025622	-0.634	0.633996774	protein_coding	NM_001294306
ASHGV40010359	0.633983	0.633983284	protein_coding	NM_000899
ASHGV40030241	0.633941	0.633940706	protein_coding	NM_019100
ASHGV40055380	-0.63394	0.633939209	protein_coding	NM_001184772
ASHGV40035258	0.633849	0.633848913	protein_coding	NM_001408
ASHGV40053173	0.633823	0.633823115	protein_coding	NM_020207
ASHGV40054167	0.633714	0.633714427	protein_coding	NM_015975
ASHGV40020163	-0.63369	0.6336942	protein_coding	NM_002981
ASHGV40051963	0.633604	0.633603763	protein_coding	NM_006378

ASHGV40032769	-0.63339	0.633389361	protein_coding	NM_058190
ASHGV40046345	0.633345	0.633345474	protein_coding	NM_015483
ASHGV40045695	0.633274	0.633273772	protein_coding	NM_004865
ASHGV40024783	-0.63327	0.633268392	protein_coding	NM_021250
ASHGV40008526	-0.63321	0.633205493	protein_coding	NM_006133
ASHGV40052477	0.632999	0.632999286	protein_coding	NM_001012415
ASHGV40028925	0.632904	0.632903944	protein_coding	NM_017657
ASHGV40041942	0.632773	0.63277251	protein_coding	NM_018046
ASHGV40052898	0.632759	0.632758546	protein_coding	NM_032250
ASHGV40036038	-0.63275	0.632746945	protein_coding	NM_001457
ASHGV40034131	0.632677	0.632676856	protein_coding	NM_001938
ASHGV40011388	0.632636	0.632636261	protein_coding	NM_001039960
ASHGV40030055	0.632539	0.63253936	protein_coding	NM_032504
ASHGV40016501	-0.63246	0.632458731	protein_coding	ENST00000562295
ASHGV40048684	-0.63214	0.632141917	protein_coding	NM_031946
ASHGV40055574	0.632076	0.632076229	protein_coding	ENST00000369762
ASHGV40019941	0.631797	0.631796637	protein_coding	NM_018713
ASHGV40054332	0.631723	0.631722746	protein_coding	NM_001113490
ASHGV40001941	0.631704	0.631703842	protein_coding	ENST00000538264
ASHGV40008443	-0.63166	0.631664112	protein_coding	NM_002559
ASHGV40052509	-0.63141	0.631414181	protein_coding	NM_001001712
ASHGV40009121	0.631369	0.631369234	protein_coding	NM_001258390
ASHGV40009066	0.631369	0.631368614	protein_coding	NM_001166
ASHGV40022359	0.631313	0.631312715	protein_coding	NM_148896
ASHGV40010610	-0.63116	0.631160904	protein_coding	NM_138451
ASHGV40054946	0.63116	0.631159732	protein_coding	NM_017883
ASHGV40050927	0.630942	0.630941966	protein_coding	NM_001286745
ASHGV40036607	0.630875	0.630874832	protein_coding	NM_001679
ASHGV40035807	-0.63078	0.63078054	protein_coding	NM_001106
ASHGV40017471	0.630692	0.630692199	protein_coding	NM_023936
ASHGV40003170	0.6306	0.630599698	protein_coding	NM_001202470
ASHGV40028551	-0.63042	0.630423863	protein_coding	NM_002252
ASHGV40034356	-0.63041	0.630411218	protein_coding	ENST00000308388
ASHGV40029005	0.630373	0.630372641	protein_coding	NM_005791
ASHGV40003346	-0.6302	0.630199279	protein_coding	NM_175858
ASHGV40022367	0.63016	0.630160192	protein_coding	NM_004127
ASHGV40038276	0.62998	0.629980118	protein_coding	NM_006818
ASHGV40000045	-0.62982	0.629821988	protein_coding	NM_001012426
ASHGV40031852	-0.62979	0.62979395	protein_coding	ENST00000598340
ASHGV40050073	-0.62973	0.629734773	protein_coding	NM_003695
ASHGV40048248	-0.62971	0.629706758	protein_coding	NM_001040105
ASHGV40003080	-0.62968	0.629675555	protein_coding	NM_001014985
ASHGV40004984	0.629639	0.629638845	protein_coding	NM_002794
ASHGV40030925	-0.62951	0.62950545	protein_coding	NM_033550
ASHGV40027054	0.62945	0.629450085	protein_coding	NM_004836
ASHGV40014077	0.629351	0.629351422	protein_coding	NM_006832
ASHGV40036337	0.6292	0.629199736	protein_coding	NM_014648

ASHGV40014120	0.629172	0.62917231	protein_coding	NM_006544
ASHGV40017629	0.629066	0.629065984	protein_coding	NM_003292
ASHGV40018146	0.628974	0.628973516	protein_coding	NM_003789
ASHGV40043367	-0.62895	0.628954219	protein_coding	NM_181336
ASHGV40026676	-0.62893	0.62892959	protein_coding	NM_005747
ASHGV40051861	0.628676	0.628676142	protein_coding	NM_017881
ASHGV40040579	0.628373	0.628372606	protein_coding	NM_018343
ASHGV40022327	-0.62818	0.628184584	protein_coding	NM_024110
ASHGV40034404	-0.62818	0.628183035	protein_coding	NM_017442
ASHGV40022390	-0.62813	0.628131247	protein_coding	NM_001004431
ASHGV40045026	-0.62796	0.627960016	protein_coding	NM_001010873
ASHGV40006701	-0.62766	0.627663213	protein_coding	uc009ygt.3
ASHGV40006940	-0.62765	0.62764765	protein_coding	NM_015231
ASHGV40009959	-0.62764	0.62763813	protein_coding	NM_001013690
ASHGV40054156	-0.62752	0.627515429	protein_coding	NM_144969
ASHGV40032277	0.627503	0.627502769	protein_coding	NM_001206540
ASHGV40024661	-0.62733	0.627330156	protein_coding	NM_138697
ASHGV40008019	-0.62714	0.627135145	protein_coding	NM_058166
ASHGV40016264	-0.62703	0.627029098	protein_coding	NM_001717
ASHGV40002659	-0.62699	0.626992907	protein_coding	NM_001459
ASHGV40000053	0.626913	0.62691296	protein_coding	NM_001025077
ASHGV40036622	0.626746	0.626746335	protein_coding	NM_173552
ASHGV40034346	0.626672	0.626672101	protein_coding	NM_000581
ASHGV40055465	-0.62662	0.626619791	protein_coding	NM_005462
ASHGV40032642	0.626527	0.626527161	protein_coding	NM_015358
ASHGV40034648	0.626326	0.626325514	protein_coding	NM_003729
ASHGV40025195	-0.62627	0.6262745	protein_coding	NM_001299
ASHGV40035014	0.626234	0.62623371	protein_coding	NM_198504
ASHGV40021835	-0.62617	0.626165597	protein_coding	NM_001145146
ASHGV40032749	-0.62606	0.626058535	protein_coding	NM_001902
ASHGV40007897	-0.62589	0.625885881	protein_coding	NM_001303029
ASHGV40035493	0.625831	0.625830906	protein_coding	NM_152773
ASHGV40055684	-0.62583	0.625829842	protein_coding	NM_033284
ASHGV40034627	-0.62579	0.625793902	protein_coding	NM_000313
ASHGV40028637	-0.62579	0.625787055	protein_coding	NM_020134
ASHGV40002437	-0.62573	0.625733763	protein_coding	ENST00000578794
ASHGV40033771	0.625675	0.625674829	protein_coding	NM_031488
ASHGV40027905	-0.6255	0.625500778	protein_coding	uc002uvb.1
ASHGV40001912	0.62546	0.625459911	protein_coding	ENST00000534420
ASHGV40045007	-0.62546	0.625457562	protein_coding	NM_002062
ASHGV40021665	-0.62545	0.625449259	protein_coding	ENST00000539023
ASHGV40033690	-0.62538	0.625380217	protein_coding	NM_001013436
ASHGV40014099	-0.62535	0.625350944	protein_coding	NM_007086
ASHGV40047390	-0.62535	0.625345173	protein_coding	NM_017760
ASHGV40035157	-0.62523	0.625226422	protein_coding	NM_206963
ASHGV40056514	-0.62507	0.625072238	protein_coding	NM_006332
ASHGV40007317	0.624804	0.624803737	protein_coding	NM_017907

ASHGV40026897	0.62477	0.624770013	protein_coding	NM_152792
ASHGV40026176	-0.62457	0.624569286	protein_coding	NM_017908
ASHGV40015176	-0.62454	0.624544869	protein_coding	NM_001037162
ASHGV40056861	0.624473	0.62447338	protein_coding	NM_004981
ASHGV40050066	0.624471	0.624471221	protein_coding	NM_015193
ASHGV40016355	0.624389	0.624388558	protein_coding	NM_001286451
ASHGV40013467	0.624254	0.624254118	protein_coding	NM_006002
ASHGV40029130	0.624014	0.624013927	protein_coding	NM_016622
ASHGV40044116	0.623764	0.62376417	protein_coding	NM_022568
ASHGV40041957	0.623658	0.623658295	protein_coding	NM_004866
ASHGV40033382	0.623388	0.623388326	protein_coding	NM_014339
ASHGV40040858	0.623356	0.623356495	protein_coding	NM_017415
ASHGV40043008	-0.6233	0.623302506	protein_coding	NM_001145020
ASHGV40006991	0.623301	0.62330096	protein_coding	NM_012456
ASHGV40034440	-0.62326	0.623263543	protein_coding	NM_020678
ASHGV40007332	0.62309	0.623089507	protein_coding	NM_001286050
ASHGV40056337	-0.62305	0.623053909	protein_coding	NM_000987
ASHGV40028799	0.623	0.623000305	protein_coding	NM_024766
ASHGV40039714	-0.62299	0.622992032	protein_coding	NM_017632
ASHGV40042867	-0.6228	0.62280172	protein_coding	NM_197975
ASHGV40037132	0.622684	0.622684005	protein_coding	NM_001013622
ASHGV40030768	-0.62266	0.62266321	protein_coding	NM_003908
ASHGV40020853	0.622588	0.622588023	protein_coding	NM_006937
ASHGV40015147	-0.62249	0.622486563	protein_coding	NM_022137
ASHGV40005292	-0.62237	0.622365007	protein_coding	NM_002051
ASHGV40030458	0.622248	0.622247914	protein_coding	NM_006066
ASHGV40008379	-0.62222	0.622221154	protein_coding	NM_000107
ASHGV40056267	-0.62219	0.622192847	protein_coding	uc002dpe.2
ASHGV40026829	0.622135	0.622135066	protein_coding	NM_015910
ASHGV40046854	-0.62212	0.622118108	protein_coding	NM_018275
ASHGV40056035	0.62205	0.62204984	protein_coding	NM_018559
ASHGV40016278	0.621843	0.621843119	protein_coding	NM_014300
ASHGV40014817	0.621828	0.621828368	protein_coding	NM_001002000
ASHGV40047051	0.621627	0.621626535	protein_coding	NM_014888
ASHGV40030155	0.621625	0.621625214	protein_coding	NM_006736
ASHGV40037693	0.621599	0.621599408	protein_coding	NM_001040202
ASHGV40025325	0.621348	0.621348097	protein_coding	NM_024050
ASHGV40040958	-0.62117	0.621173591	protein_coding	NM_001029869
ASHGV40046461	0.621142	0.621141781	protein_coding	NM_001146334
ASHGV40007469	0.62112	0.621119564	protein_coding	NM_152313
ASHGV40021681	-0.621	0.621001063	protein_coding	NM_006804
ASHGV40038932	-0.62099	0.620985694	protein_coding	NM_001256475
ASHGV40028323	-0.62091	0.620912776	protein_coding	NM_001102467
ASHGV40003278	-0.62073	0.62073187	protein_coding	NM_012230
ASHGV40016172	-0.62063	0.620630911	protein_coding	uc021sqz.1
ASHGV40054775	-0.62043	0.620431229	protein_coding	NM_016937
ASHGV40012097	0.620376	0.620376356	protein_coding	NM_014730

ASHGV40023017	-0.62032	0.620319778	protein_coding	NM_025078
ASHGV40032372	-0.62019	0.620194102	protein_coding	NM_198698
ASHGV40034107	-0.62017	0.620171129	protein_coding	NM_003615
ASHGV40056174	-0.62012	0.620124517	protein_coding	uc021oei.1
ASHGV40011843	0.619958	0.619958263	protein_coding	NM_006838
ASHGV40025408	0.619938	0.619938329	protein_coding	ENST00000344519
ASHGV40005746	0.619642	0.61964164	protein_coding	NM_003171
ASHGV40020742	0.619509	0.619508735	protein_coding	NM_014877
ASHGV40009914	-0.61929	0.619290404	protein_coding	NM_016594
ASHGV40049261	-0.61914	0.619143314	protein_coding	NM_177987
ASHGV40047227	0.618962	0.618962068	protein_coding	NM_001080392
ASHGV40004926	0.618715	0.618715266	protein_coding	NM_001303134
ASHGV40041092	0.61852	0.618520291	protein_coding	NM_006425
ASHGV40054392	-0.61839	0.618393203	protein_coding	NM_014253
ASHGV40037870	-0.61828	0.618279238	protein_coding	NM_000204
ASHGV40044967	-0.61824	0.618240687	protein_coding	NM_182548
ASHGV40046142	0.618181	0.618181037	protein_coding	NM_198097
ASHGV40051219	-0.61812	0.618121409	protein_coding	NM_002467
ASHGV40005075	-0.61802	0.618015216	protein_coding	NM_001039762
ASHGV40048563	-0.61801	0.618011261	protein_coding	NM_001001656
ASHGV40025109	-0.61794	0.617939648	protein_coding	NM_014257
ASHGV40027843	0.617818	0.617818056	protein_coding	NM_014362
ASHGV40029272	-0.61781	0.61780816	protein_coding	NM_006236
ASHGV40052617	-0.61776	0.617764309	protein_coding	NM_152896
ASHGV40039247	0.617732	0.617731902	protein_coding	NM_183075
ASHGV40032913	-0.61772	0.617716557	protein_coding	uc021wmn.1
ASHGV40000030	0.617629	0.617628934	protein_coding	NM_002737
ASHGV40048954	0.617618	0.617617876	protein_coding	NM_004686
ASHGV40054554	-0.61761	0.617605271	protein_coding	NM_052926
ASHGV40029220	0.617499	0.61749934	protein_coding	NM_015929
ASHGV40033160	-0.61735	0.617351333	protein_coding	NM_007068
ASHGV40052642	-0.61719	0.617190616	protein_coding	NM_203403
ASHGV40002968	-0.61709	0.617092198	protein_coding	NM_000594
ASHGV40008441	-0.61698	0.61697857	protein_coding	NM_001005210
ASHGV40057543	0.61692	0.616920419	protein_coding	NM_024838
ASHGV40001325	-0.61691	0.616910159	protein_coding	ENST00000546504
ASHGV40000141	0.61679	0.616790472	protein_coding	NM_003859
ASHGV40044197	0.616615	0.616614993	protein_coding	NM_032020
ASHGV40033784	0.616532	0.616531937	protein_coding	NM_152513
ASHGV40019158	-0.61653	0.616531397	protein_coding	NM_023938
ASHGV40055572	-0.61648	0.616476596	protein_coding	NM_000117
ASHGV40046303	-0.6163	0.616301252	protein_coding	NM_152739
ASHGV40043028	-0.61628	0.616278484	protein_coding	NM_007212
ASHGV40008752	-0.61621	0.61621455	protein_coding	NM_139075
ASHGV40020670	0.615866	0.615866197	protein_coding	NM_020748
ASHGV40024644	-0.61566	0.615655735	protein_coding	NM_006270
ASHGV40023912	0.615489	0.615488869	protein_coding	NM_001420

ASHGV40023777	0.61545	0.615449763	protein_coding	NM_000150
ASHGV40009539	-0.61536	0.615363105	protein_coding	NM_153449
ASHGV40048182	0.615166	0.615166408	protein_coding	NM_014916
ASHGV40037268	0.615108	0.615108196	protein_coding	NM_001290
ASHGV40029182	-0.61484	0.614844058	protein_coding	NM_144707
ASHGV40022762	-0.61484	0.614842379	protein_coding	NM_001039360
ASHGV40007591	-0.61483	0.614829935	protein_coding	NM_152587
ASHGV40023769	0.614805	0.614805335	protein_coding	NM_014649
ASHGV40041859	-0.61473	0.614731402	protein_coding	NM_133339
ASHGV40014739	-0.61468	0.614678993	protein_coding	uc021rou.1
ASHGV40013760	0.614676	0.6146763	protein_coding	NM_003903
ASHGV40022395	0.614393	0.614392516	protein_coding	NM_005131
ASHGV40013573	0.614244	0.614243762	protein_coding	NM_180989
ASHGV40051579	-0.61418	0.614184133	protein_coding	NM_006900
ASHGV40008467	-0.61389	0.613889631	protein_coding	NM_022074
ASHGV40054367	-0.61381	0.613811388	protein_coding	NM_139282
ASHGV40012123	0.61378	0.613779537	protein_coding	NM_001024808
ASHGV40023101	-0.61375	0.613750653	protein_coding	NM_001105581
ASHGV40021694	-0.61368	0.613678756	protein_coding	NM_000759
ASHGV40045045	0.613629	0.613628937	protein_coding	NM_018066
ASHGV40026906	-0.61354	0.613541263	protein_coding	NM_032822
ASHGV40055942	0.613338	0.61333812	protein_coding	NM_005736
ASHGV40044903	0.613264	0.613263942	protein_coding	NM_002122
ASHGV40018786	-0.61325	0.613246632	protein_coding	NM_030941
ASHGV40055576	0.613236	0.6132356	protein_coding	NM_004699
ASHGV40049041	-0.61318	0.61318219	protein_coding	NM_022659
ASHGV40022044	0.613025	0.613024504	protein_coding	NM_004859
ASHGV40019817	0.612982	0.612982232	protein_coding	NM_018040
ASHGV40028856	0.612949	0.612948823	protein_coding	NM_015701
ASHGV40039668	0.612714	0.6127142	protein_coding	NM_170710
ASHGV40037704	0.612583	0.612583191	protein_coding	NM_152545
ASHGV40051474	-0.61246	0.612461796	protein_coding	ENST00000599351
ASHGV40011508	-0.61241	0.612414846	protein_coding	NM_014182
ASHGV40005351	-0.6123	0.612300777	protein_coding	NM_006414
ASHGV40047426	-0.61226	0.612259768	protein_coding	NM_138445
ASHGV40038725	0.612227	0.612226533	protein_coding	NM_020860
ASHGV40036596	-0.61209	0.612085048	protein_coding	NM_080862
ASHGV40045588	-0.61208	0.612082013	protein_coding	NM_173560
ASHGV40032730	0.611967	0.611967437	protein_coding	NM_003681
ASHGV40021203	-0.61191	0.611914222	protein_coding	NM_181844
ASHGV40043638	-0.61178	0.611779429	protein_coding	NM_001723
ASHGV40021213	0.611723	0.611723185	protein_coding	ENST00000302926
ASHGV40012964	-0.61168	0.611684503	protein_coding	uc001vre.3
ASHGV40006012	-0.61152	0.611524223	protein_coding	NM_016341
ASHGV40017568	-0.61126	0.611263792	protein_coding	NM_021646
ASHGV40006689	-0.61124	0.611242056	protein_coding	NM_024514
ASHGV40014029	0.611234	0.611234172	protein_coding	NM_001032

ASHGV40000399	0.61119	0.611190457	protein_coding	NM_024065
ASHGV40023385	0.611153	0.611152862	protein_coding	NM_002396
ASHGV40028023	-0.61092	0.610924445	protein_coding	NM_000465
ASHGV40000180	0.610922	0.610922463	protein_coding	NM_001018090
ASHGV40009236	-0.61077	0.610771023	protein_coding	NM_005188
ASHGV40027048	0.610459	0.610458776	protein_coding	NM_001304526
ASHGV40017570	0.610254	0.610253739	protein_coding	NM_024589
ASHGV40053339	-0.61022	0.610219661	protein_coding	NM_000607
ASHGV40025485	0.610059	0.610059148	protein_coding	NM_003796
ASHGV40036532	0.609985	0.60998507	protein_coding	NM_014382
ASHGV40033009	-0.60996	0.609962301	protein_coding	NM_012265
ASHGV40019267	-0.60995	0.609953419	protein_coding	NM_001040667
ASHGV40025651	-0.60959	0.60958555	protein_coding	NM_021185
ASHGV40018384	0.609523	0.609522967	protein_coding	NM_001159377
ASHGV40054909	-0.60948	0.609484573	protein_coding	NM_014735
ASHGV40038257	-0.60928	0.609279639	protein_coding	NM_138278
ASHGV40020373	0.609257	0.609256981	protein_coding	NM_001040431
ASHGV40033666	-0.60918	0.60918081	protein_coding	NM_006739
ASHGV40001260	0.60895	0.608949894	protein_coding	ENST00000460532
ASHGV40055262	-0.60895	0.608947722	protein_coding	NM_017752
ASHGV40036578	0.608932	0.608931864	protein_coding	NM_014154
ASHGV40017179	0.608858	0.608858312	protein_coding	NM_022347
ASHGV40018368	-0.60866	0.608660842	protein_coding	NM_206967
ASHGV40044432	0.608447	0.608447274	protein_coding	NM_003247
ASHGV40013783	-0.60841	0.608409323	protein_coding	NM_001001673
ASHGV40030351	-0.60834	0.608340053	protein_coding	NM_001291832
ASHGV40004991	0.608226	0.608225948	protein_coding	NM_213649
ASHGV40012014	-0.60808	0.608082322	protein_coding	NM_006187
ASHGV40034898	0.608033	0.608032966	protein_coding	NM_003925
ASHGV40019189	-0.60802	0.608023436	protein_coding	NM_170776
ASHGV40033704	-0.60797	0.6079656	protein_coding	NM_018957
ASHGV40050823	0.607833	0.607832546	protein_coding	NM_001195797
ASHGV40042401	0.607793	0.607793381	protein_coding	NM_032412
ASHGV40001300	-0.60776	0.60775903	protein_coding	ENST00000467211
ASHGV40006637	0.607654	0.607653863	protein_coding	NM_017739
ASHGV40027692	-0.60757	0.607568724	protein_coding	NM_001276252
ASHGV40015358	0.607387	0.607386861	protein_coding	NM_005532
ASHGV40049100	0.607387	0.607386595	protein_coding	NM_013357
ASHGV40007229	0.607364	0.607364405	protein_coding	NM_004910
ASHGV40024593	-0.60732	0.607319792	protein_coding	NM_000234
ASHGV40045752	-0.60717	0.607173792	protein_coding	NM_018194
ASHGV40018547	-0.60693	0.606928949	protein_coding	NM_012225
ASHGV40048848	-0.60676	0.606760378	protein_coding	NM_001205266
ASHGV40003348	0.606738	0.606738121	protein_coding	NM_178352
ASHGV40007939	0.606705	0.606705304	protein_coding	ENST00000598274
ASHGV40024380	-0.60664	0.606637609	protein_coding	NM_014448
ASHGV40034997	-0.60617	0.606174742	protein_coding	NM_001113226

ASHGV40009210	0.606071	0.606071337	protein_coding	NM_004388
ASHGV40020944	-0.60607	0.606069838	protein_coding	NM_025090
ASHGV40037435	0.606069	0.606068692	protein_coding	NM_001166050
ASHGV40043870	0.605982	0.605981881	protein_coding	NM_032511
ASHGV40054079	-0.60589	0.605892198	protein_coding	NM_001012968
ASHGV40005325	0.605865	0.60586484	protein_coding	NM_006023
ASHGV40034304	0.605751	0.605750577	protein_coding	NM_002673
ASHGV40048510	-0.60574	0.605739218	protein_coding	NM_178563
ASHGV40007033	-0.60562	0.605618741	protein_coding	NM_001062
ASHGV40041769	-0.60556	0.605559994	protein_coding	NM_005921
ASHGV40020647	-0.60546	0.605460293	protein_coding	NM_016261
ASHGV40008480	-0.60542	0.605417032	protein_coding	uc001nok.2
ASHGV40016449	-0.60538	0.605380577	protein_coding	NM_178842
ASHGV40046676	-0.60526	0.605264804	protein_coding	NM_207468
ASHGV40031986	-0.60525	0.605251245	protein_coding	NM_001290224
ASHGV40033767	-0.60519	0.605193091	protein_coding	NM_022098
ASHGV40052436	0.605082	0.60508185	protein_coding	NM_020469
ASHGV40005027	-0.60501	0.605010489	protein_coding	uc010qty.2
ASHGV40056622	0.604727	0.604727387	protein_coding	NM_080653
ASHGV40035356	0.604575	0.604574858	protein_coding	NM_001966
ASHGV40012242	0.604281	0.604281401	protein_coding	NM_198827
ASHGV40016327	-0.60426	0.60426387	protein_coding	NM_016321
ASHGV40026465	-0.60422	0.604224498	protein_coding	uc002rei.4
ASHGV40025529	-0.6042	0.604199738	protein_coding	NM_002333
ASHGV40039907	0.604158	0.60415819	protein_coding	NM_017755
ASHGV40048630	0.604103	0.604102508	protein_coding	NM_006642
ASHGV40016686	-0.60401	0.604012735	protein_coding	NM_181642
ASHGV40015063	-0.60401	0.604007558	protein_coding	ENST00000445360
ASHGV40007198	0.604002	0.604002152	protein_coding	NM_020470
ASHGV40036059	0.60369	0.603689543	protein_coding	NM_020685
ASHGV40041974	-0.60363	0.603630407	protein_coding	NM_205548
ASHGV40003106	-0.60358	0.603576584	protein_coding	NM_001080514
ASHGV40053215	0.603484	0.603483973	protein_coding	NM_006808
ASHGV40003369	-0.60346	0.603455931	protein_coding	NM_207511
ASHGV40007471	0.603422	0.603422222	protein_coding	NM_024116
ASHGV40007479	0.603303	0.603302716	protein_coding	NM_016540
ASHGV40023346	-0.60327	0.603265915	protein_coding	NM_138443
ASHGV40027042	0.603249	0.603248503	protein_coding	ENST00000409310
ASHGV40032682	0.603168	0.603168076	protein_coding	NM_004627
ASHGV40016858	-0.60312	0.603115403	protein_coding	NM_014656
ASHGV40031555	-0.60306	0.603057295	protein_coding	NM_006097
ASHGV40044525	0.603004	0.603004487	protein_coding	NM_003913
ASHGV40010581	0.602943	0.602942832	protein_coding	NM_016238
ASHGV40036581	0.602919	0.602919244	protein_coding	NM_031913
ASHGV40002032	-0.60267	0.602667264	protein_coding	ENST00000548915
ASHGV40044561	-0.60267	0.602665602	protein_coding	NM_001718
ASHGV40007958	-0.60252	0.602522424	protein_coding	uc001luy.2

ASHGV40031686	0.602492	0.602492215	protein_coding	NM_052951
ASHGV40052489	-0.60192	0.601920407	protein_coding	NM_181701
ASHGV40039831	-0.60185	0.60185249	protein_coding	NM_198253
ASHGV40052021	-0.6018	0.601803064	protein_coding	NM_003837
ASHGV40026333	-0.60178	0.601781182	protein_coding	NM_001037160
ASHGV40049540	0.601734	0.601734065	protein_coding	NM_024699
ASHGV40037632	0.601601	0.601600674	protein_coding	NM_002994
ASHGV40014050	0.601284	0.601284244	protein_coding	NM_021818
ASHGV40027816	0.601281	0.601281499	protein_coding	NM_001105556
ASHGV40054942	-0.60116	0.601164016	protein_coding	NM_203475
ASHGV40038053	-0.60111	0.601112876	protein_coding	NM_032623
ASHGV40049281	0.601086	0.601085881	protein_coding	NM_052937
ASHGV40017866	0.601015	0.601014984	protein_coding	NM_024706
ASHGV40017375	-0.60101	0.601011356	protein_coding	NM_198243
ASHGV40032367	-0.60081	0.600808308	protein_coding	NM_198691
ASHGV40030344	0.600716	0.60071564	protein_coding	NM_001137552
ASHGV40023325	0.600564	0.600563995	protein_coding	NM_002647
ASHGV40014815	-0.60047	0.600473871	protein_coding	NM_005132
ASHGV40021974	0.600412	0.600412469	protein_coding	NM_000143
ASHGV40053535	0.600358	0.600357771	protein_coding	NM_014286
ASHGV40007957	0.600347	0.600346551	protein_coding	NM_021134
ASHGV40054678	0.600305	0.600305411	protein_coding	NM_001830
ASHGV40005942	0.600244	0.600243893	protein_coding	NM_004670
ASHGV40007200	0.600224	0.600224363	protein_coding	NM_004292
ASHGV40051658	0.60021	0.600209855	protein_coding	NM_005470
ASHGV40036374	-0.60007	0.600072464	protein_coding	NM_017699
ASHGV40051487	0.599864	0.599864087	protein_coding	NM_001017969
ASHGV40052921	-0.59985	0.599853039	protein_coding	NM_021965
ASHGV40041526	0.599493	0.599493125	protein_coding	NM_003666
ASHGV40056272	-0.59948	0.599475716	protein_coding	uc021tfy.1
ASHGV40057765	0.599413	0.599412795	protein_coding	NM_053023
ASHGV40021313	-0.59941	0.599405189	protein_coding	ENST00000609971
ASHGV40043111	-0.59939	0.599394602	protein_coding	NM_016356
ASHGV40020269	0.599386	0.599385522	protein_coding	NM_021724
ASHGV40052045	0.599285	0.599285152	protein_coding	NM_007001
ASHGV40041332	-0.59918	0.59917965	protein_coding	NM_033120
ASHGV40038679	0.599117	0.59911714	protein_coding	NM_001258345
ASHGV40045480	0.599081	0.59908135	protein_coding	NM_018013
ASHGV40026241	0.599028	0.599028259	protein_coding	NM_003310
ASHGV40060845	-0.59902	0.599020035	protein_coding	uc003wag.2
ASHGV40038864	-0.59898	0.59897847	protein_coding	NM_152679
ASHGV40003366	-0.59894	0.598942868	protein_coding	NM_207118
ASHGV40042454	-0.59888	0.598879548	protein_coding	NM_032053
ASHGV40044633	-0.59858	0.59857943	protein_coding	NM_004973
ASHGV40013869	0.598576	0.598576265	protein_coding	NM_001099274
ASHGV40048740	0.598546	0.598545805	protein_coding	NM_001130957
ASHGV40002273	-0.59853	0.598532682	protein_coding	ENST00000563887

ASHGV40006582	0.598466	0.598466117	protein_coding	NM_021639
ASHGV40048201	-0.59842	0.59841563	protein_coding	NM_213603
ASHGV40040633	-0.59841	0.598410486	protein_coding	NM_001962
ASHGV40044836	0.598397	0.598397268	protein_coding	NM_001025091
ASHGV40000712	0.598328	0.59832798	protein_coding	NM_017747
ASHGV40041281	-0.59809	0.598091525	protein_coding	NM_175062
ASHGV40018571	-0.59809	0.598086238	protein_coding	NM_001374
ASHGV40013268	0.59783	0.597829659	protein_coding	NM_153218
ASHGV40010982	-0.59773	0.597725429	protein_coding	NM_000616
ASHGV40019538	-0.59771	0.597712787	protein_coding	NM_015434
ASHGV40001553	-0.59765	0.597645592	protein_coding	ENST00000509111
ASHGV40053882	-0.59763	0.597627728	protein_coding	NM_031894
ASHGV40046041	-0.59746	0.597457123	protein_coding	NM_003194
ASHGV40024013	0.597313	0.597313305	protein_coding	NM_005858
ASHGV40056782	-0.59715	0.597147356	protein_coding	NM_005461
ASHGV40056695	0.596976	0.596975819	protein_coding	uc010zae.2
ASHGV40007951	0.596963	0.596962725	protein_coding	NM_001290332
ASHGV40040703	0.596925	0.596924981	protein_coding	NM_001199012
ASHGV40006785	0.596824	0.596824073	protein_coding	NM_001709
ASHGV40022419	0.59675	0.596749702	protein_coding	NM_015446
ASHGV40005188	-0.59671	0.596714234	protein_coding	uc001igd.4
ASHGV40012110	0.596506	0.596505947	protein_coding	NM_025126
ASHGV40057544	-0.59638	0.596376727	protein_coding	NM_005226
ASHGV40036256	-0.59628	0.596281678	protein_coding	NM_001005514
ASHGV40008515	0.596207	0.59620667	protein_coding	NM_024911
ASHGV40019735	0.596139	0.596139483	protein_coding	NM_003562
ASHGV40010483	-0.59601	0.596007686	protein_coding	NM_015958
ASHGV40018100	0.595867	0.595866766	protein_coding	NM_001796
ASHGV40035848	0.595861	0.595860961	protein_coding	NM_145166
ASHGV40028910	0.595718	0.595718366	protein_coding	NM_006577
ASHGV40042340	0.595496	0.595496168	protein_coding	NM_014829
ASHGV40016607	0.595491	0.595491298	protein_coding	NM_001243996
ASHGV40036959	-0.59548	0.595483251	protein_coding	ENST00000392468
ASHGV40045478	0.595448	0.595447527	protein_coding	NM_016487
ASHGV40045784	0.595439	0.595438582	protein_coding	NM_007124
ASHGV40056547	0.595431	0.59543119	protein_coding	NM_153232
ASHGV40031340	0.595375	0.595374528	protein_coding	NM_080676
ASHGV40012747	0.595358	0.595358382	protein_coding	NM_024546
ASHGV40044329	0.595166	0.595166171	protein_coding	NM_030752
ASHGV40019749	0.59504	0.595039868	protein_coding	NM_001212
ASHGV40015177	0.594951	0.594951004	protein_coding	NM_031427
ASHGV40021617	-0.5949	0.594896967	protein_coding	NM_139215
ASHGV40000178	0.59487	0.594870259	protein_coding	NM_004568
ASHGV40016717	-0.59476	0.59475991	protein_coding	NM_020759
ASHGV40056403	-0.59464	0.594636934	protein_coding	NM_001018136
ASHGV40056160	0.59461	0.594609663	protein_coding	uc031qrc.1
ASHGV40023285	-0.59453	0.594534506	protein_coding	ENST00000426194

ASHGV40045371	0.594415	0.594414795	protein_coding	NM_006813
ASHGV40036907	-0.59437	0.594371569	protein_coding	NM_014693
ASHGV40007710	-0.5943	0.594297476	protein_coding	NM_182557
ASHGV40037702	-0.59429	0.594289894	protein_coding	NM_006259
ASHGV40027314	-0.59428	0.594276599	protein_coding	NM_019044
ASHGV40029349	0.594159	0.594158764	protein_coding	NM_014439
ASHGV40044469	0.594132	0.594131655	protein_coding	NM_138391
ASHGV40046724	0.59413	0.594130443	protein_coding	NM_000722
ASHGV40024536	0.594052	0.594052445	protein_coding	NM_004943
ASHGV40014917	-0.59401	0.594005228	protein_coding	NM_003616
ASHGV40001282	0.593827	0.593827266	protein_coding	NM_001204062
ASHGV40042598	0.593733	0.593733276	protein_coding	NM_014180
ASHGV40038501	-0.59372	0.593718128	protein_coding	NM_173660
ASHGV40028303	-0.59359	0.593592628	protein_coding	NM_001005853
ASHGV40011439	0.593541	0.593540822	protein_coding	NM_021640
ASHGV40008561	-0.59348	0.593476385	protein_coding	NM_033101
ASHGV40021210	-0.59346	0.593457722	protein_coding	NM_001002914
ASHGV40018251	-0.59342	0.593420935	protein_coding	NM_018124
ASHGV40042891	-0.59322	0.593216881	protein_coding	NM_018303
ASHGV40026751	0.593004	0.593004345	protein_coding	NM_001003937
ASHGV40005258	-0.59296	0.592956213	protein_coding	NM_053049
ASHGV40045183	-0.5929	0.592899407	protein_coding	NM_032960
ASHGV40009312	-0.59286	0.592857295	protein_coding	NM_022370
ASHGV40007907	0.592853	0.592853255	protein_coding	NM_000797
ASHGV40011129	0.592787	0.592786957	protein_coding	NM_001114176
ASHGV40010689	0.592598	0.592597722	protein_coding	NM_003769
ASHGV40010251	0.592281	0.592281197	protein_coding	NM_144982
ASHGV40056815	-0.59228	0.592279451	protein_coding	NM_000628
ASHGV40054854	0.592254	0.59225439	protein_coding	NM_001039590
ASHGV40015600	0.592179	0.59217939	protein_coding	ENST00000558241
ASHGV40006908	-0.59195	0.591950648	protein_coding	NM_001080446
ASHGV40033705	0.591871	0.591870594	protein_coding	NM_020315
ASHGV40038580	-0.59185	0.591851028	protein_coding	NM_001040071
ASHGV40021331	0.591748	0.591748265	protein_coding	NM_001303
ASHGV40008387	-0.5917	0.59169967	protein_coding	NM_175732
ASHGV40046069	0.591689	0.591689125	protein_coding	NM_182924
ASHGV40047318	-0.59139	0.591394647	protein_coding	NM_198285
ASHGV40025794	0.591298	0.59129794	protein_coding	NM_001645
ASHGV40042595	0.591243	0.591243047	protein_coding	NM_015315
ASHGV40019273	-0.59121	0.591207952	protein_coding	NM_004594
ASHGV40019261	-0.59096	0.590959509	protein_coding	NM_024922
ASHGV40013344	-0.59087	0.590867028	protein_coding	NM_001101320
ASHGV40017414	0.590346	0.590345529	protein_coding	NM_006428
ASHGV40025079	-0.59034	0.590337524	protein_coding	NM_139161
ASHGV40041580	0.590263	0.590262845	protein_coding	NM_006713
ASHGV40016990	0.590244	0.590243935	protein_coding	NM_015554
ASHGV40057609	-0.59004	0.59003928	protein_coding	NM_014061

ASHGV40033605	-0.58973	0.589730027	protein_coding	NM_016498
ASHGV40049895	0.589715	0.589714835	protein_coding	NM_194291
ASHGV40040128	-0.58963	0.589630143	protein_coding	NM_153485
ASHGV40051362	-0.58951	0.589505554	protein_coding	NM_022365
ASHGV40035849	-0.58944	0.589435364	protein_coding	NM_152393
ASHGV40038206	0.589091	0.589090998	protein_coding	NM_020116
ASHGV40039810	-0.58897	0.58897161	protein_coding	NM_145265
ASHGV40040129	0.588916	0.588915682	protein_coding	NM_005531
ASHGV40044886	-0.58861	0.588613966	protein_coding	NM_032454
ASHGV40015635	0.588489	0.588489272	protein_coding	NM_015307
ASHGV40036275	0.588483	0.588482664	protein_coding	NM_032359
ASHGV40003324	0.588192	0.588191865	protein_coding	NM_033428
ASHGV40046438	-0.58809	0.588085708	protein_coding	NM_013284
ASHGV40001757	-0.58802	0.588019966	protein_coding	ENST00000523572
ASHGV40016880	0.58792	0.587919617	protein_coding	NM_003285
ASHGV40000022	-0.58786	0.587855544	protein_coding	NM_015871
ASHGV40037276	-0.58779	0.58778761	protein_coding	NM_015688
ASHGV40019484	0.587728	0.587727833	protein_coding	NM_021194
ASHGV40009943	-0.58768	0.587684715	protein_coding	NM_001145475
ASHGV40044732	0.58765	0.587650119	protein_coding	NM_003542
ASHGV40037066	0.587474	0.587473681	protein_coding	NM_017861
ASHGV40056881	-0.58747	0.587467806	protein_coding	NM_020873
ASHGV40050355	-0.58719	0.587189351	protein_coding	NM_006129
ASHGV40037422	0.587046	0.587046166	protein_coding	NM_001204747
ASHGV40048434	0.5869	0.586899844	protein_coding	NM_020066
ASHGV40018931	-0.58689	0.58689179	protein_coding	NM_006662
ASHGV40039611	-0.58662	0.586616681	protein_coding	NM_016081
ASHGV40056868	-0.58648	0.586481877	protein_coding	NM_001284334
ASHGV40003098	-0.58629	0.586287037	protein_coding	NM_001040061
ASHGV40018933	0.586279	0.586279135	protein_coding	ENST00000414729
ASHGV40055840	0.586201	0.586200571	protein_coding	NM_022451
ASHGV40008447	-0.58619	0.586192059	protein_coding	NM_001105565
ASHGV40026684	0.586158	0.586157676	protein_coding	NM_016932
ASHGV40033503	0.586141	0.586141396	protein_coding	NM_005940
ASHGV40013319	0.586081	0.586081041	protein_coding	NM_030911
ASHGV40034819	-0.58597	0.58597049	protein_coding	NM_031458
ASHGV40008954	0.585956	0.585955711	protein_coding	NM_003797
ASHGV40033643	0.585763	0.585763266	protein_coding	NM_001098535
ASHGV40010725	-0.58561	0.585607673	protein_coding	NM_024667
ASHGV40034526	0.585438	0.585438092	protein_coding	NM_003848
ASHGV40041992	-0.58543	0.585427411	protein_coding	NM_004385
ASHGV40029762	0.585278	0.585278366	protein_coding	NM_003642
ASHGV40017099	0.585278	0.585278286	protein_coding	NM_018200
ASHGV40025877	0.585223	0.585223083	protein_coding	NM_020126
ASHGV40045522	-0.58522	0.585215889	protein_coding	NM_153369
ASHGV40043715	0.585023	0.585022669	protein_coding	NM_015687
ASHGV40018963	-0.58501	0.585014515	protein_coding	NM_005353

ASHGV40042306	0.584968	0.584967659	protein_coding	NM_005732
ASHGV40055273	-0.58455	0.58455436	protein_coding	NM_182607
ASHGV40053464	0.584277	0.584277074	protein_coding	NM_014636
ASHGV40025792	0.584256	0.58425551	protein_coding	NM_006114
ASHGV40009519	0.584214	0.58421447	protein_coding	NM_032641
ASHGV40032162	-0.58415	0.58414987	protein_coding	NM_001164434
ASHGV40000650	-0.5841	0.584100601	protein_coding	ENST00000427721
ASHGV40021815	-0.58399	0.583986124	protein_coding	NM_018072
ASHGV40030901	-0.58376	0.583763045	protein_coding	NM_172006
ASHGV40028833	-0.58368	0.583677254	protein_coding	NM_017629
ASHGV40007173	0.58339	0.58339023	protein_coding	NM_032193
ASHGV40045775	0.583351	0.583351149	protein_coding	NM_003630
ASHGV40036744	-0.58334	0.583335127	protein_coding	NM_014575
ASHGV40018454	-0.58333	0.58333254	protein_coding	NM_000135
ASHGV40041802	0.583308	0.583308288	protein_coding	NM_174889
ASHGV40050287	-0.58309	0.583091043	protein_coding	NM_020844
ASHGV40054545	0.582938	0.582937542	protein_coding	NM_004961
ASHGV40054764	-0.58287	0.582870433	protein_coding	NM_173555
ASHGV40011515	-0.5825	0.582499377	protein_coding	NM_006594
ASHGV40042165	0.582467	0.582466851	protein_coding	uc021ycm.1
ASHGV40016618	-0.58244	0.582443604	protein_coding	ENST00000391457
ASHGV40013810	-0.58208	0.582078322	protein_coding	NM_032846
ASHGV40007241	-0.58175	0.581753609	protein_coding	NM_015269
ASHGV40038707	0.581682	0.581681765	protein_coding	NM_013367
ASHGV40052170	-0.58165	0.581651019	protein_coding	NM_001012993
ASHGV40030220	-0.58116	0.581156021	protein_coding	NM_173484
ASHGV40057542	-0.58113	0.581128188	protein_coding	NM_001161625
ASHGV40021356	0.580863	0.580862964	protein_coding	NM_017775
ASHGV40020208	0.580583	0.580583367	protein_coding	NM_022831
ASHGV40041815	0.58047	0.580470114	protein_coding	NM_016338
ASHGV40025075	-0.58042	0.580421954	protein_coding	NM_006012
ASHGV40017682	-0.58036	0.580363289	protein_coding	uc010bvw.2
ASHGV40006630	0.58024	0.580240313	protein_coding	NM_001206671
ASHGV40046488	0.580223	0.580223132	protein_coding	NM_022748
ASHGV40022229	-0.58021	0.580210956	protein_coding	NM_001162995
ASHGV40016668	0.580192	0.580192287	protein_coding	NM_001145643
ASHGV40034501	-0.58017	0.580172191	protein_coding	NM_015976
ASHGV40037789	0.580129	0.580129219	protein_coding	NM_000671
ASHGV40015895	-0.58009	0.58009372	protein_coding	NM_001308421
ASHGV40040158	0.579906	0.579906325	protein_coding	NM_012382
ASHGV40013774	-0.5799	0.57990474	protein_coding	NM_021178
ASHGV40024477	-0.57979	0.579792196	protein_coding	NM_002250
ASHGV40006432	0.579781	0.579781233	protein_coding	NM_005539
ASHGV40032413	0.579676	0.579676476	protein_coding	uc002zio.1
ASHGV40018144	0.579674	0.579674287	protein_coding	NM_016062
ASHGV40049182	0.579605	0.579604518	protein_coding	NM_078473
ASHGV40025793	0.579467	0.579467061	protein_coding	NM_000041

ASHGV40052201	0.579368	0.579368307	protein_coding	NM_000031
ASHGV40045892	0.579343	0.579343292	protein_coding	NM_020823
ASHGV40047179	0.579308	0.579307708	protein_coding	NM_012450
ASHGV40035898	-0.57918	0.579183291	protein_coding	NM_003965
ASHGV40050751	-0.57904	0.579037215	protein_coding	NM_004814
ASHGV40056728	-0.57902	0.579023805	protein_coding	NM_153038
ASHGV40007336	-0.57898	0.578983095	protein_coding	NM_016565
ASHGV40025336	-0.5786	0.578602535	protein_coding	NM_014874
ASHGV40050904	0.578592	0.57859214	protein_coding	NM_007013
ASHGV40030708	-0.57845	0.578450441	protein_coding	NM_025176
ASHGV40000054	0.578416	0.578416007	protein_coding	uc011dig.1
ASHGV40035549	-0.57835	0.578348752	protein_coding	NM_006515
ASHGV40014893	0.578275	0.578275431	protein_coding	NM_001079519
ASHGV40023764	0.578212	0.578212137	protein_coding	NM_001080523
ASHGV40051440	0.578211	0.57821075	protein_coding	NM_207305
ASHGV40019943	0.578138	0.57813818	protein_coding	NM_004446
ASHGV40047194	-0.57811	0.5781061	protein_coding	NM_001139456
ASHGV40003143	-0.57802	0.578024732	protein_coding	NM_025201
ASHGV40044539	0.577962	0.577962262	protein_coding	NM_002725
ASHGV40031308	-0.57794	0.577940509	protein_coding	NM_001009608
ASHGV40019079	0.577922	0.577921574	protein_coding	NM_015247
ASHGV40031454	0.57786	0.577859919	protein_coding	NM_016491
ASHGV40026648	-0.57778	0.577783915	protein_coding	NM_018994
ASHGV40044310	-0.5776	0.577603782	protein_coding	NM_020216
ASHGV40035611	-0.57751	0.577507156	protein_coding	NM_014229
ASHGV40019638	-0.57736	0.577360295	protein_coding	uc002fqs.2
ASHGV40032959	-0.5773	0.577304566	protein_coding	NM_182492
ASHGV40037134	0.577291	0.577290995	protein_coding	NM_006527
ASHGV40031480	-0.57677	0.576767918	protein_coding	NM_001011718
ASHGV40053487	0.576588	0.57658844	protein_coding	NM_004408
ASHGV40016852	-0.57651	0.576505944	protein_coding	NM_022100
ASHGV40043263	0.576465	0.576465314	protein_coding	NM_024420
ASHGV40047268	-0.57646	0.576456577	protein_coding	NM_001080413
ASHGV40025050	0.576451	0.576450699	protein_coding	NM_001242901
ASHGV40013186	0.576339	0.576338723	protein_coding	NM_015032
ASHGV40017539	-0.57633	0.57633374	protein_coding	NM_001190476
ASHGV40021615	-0.57632	0.576315231	protein_coding	NM_145272
ASHGV40046749	0.576231	0.57623142	protein_coding	NM_000927
ASHGV40008638	-0.57619	0.576193088	protein_coding	NM_145719
ASHGV40030444	0.575854	0.575853627	protein_coding	NM_000801
ASHGV40015075	0.575834	0.575833601	protein_coding	NM_002431
ASHGV40050595	0.57573	0.575730175	protein_coding	NM_032410
ASHGV40017664	0.575712	0.575712447	protein_coding	NM_002474
ASHGV40025867	0.575639	0.575638806	protein_coding	NM_000836
ASHGV40015843	-0.57556	0.57555655	protein_coding	NM_000530
ASHGV40045641	-0.57551	0.575508606	protein_coding	NM_015714
ASHGV40038297	-0.57543	0.575432727	protein_coding	NM_144644

ASHGV40009578	-0.57541	0.575411288	protein_coding	NM_016511
ASHGV40035344	0.575138	0.575138285	protein_coding	NM_018622
ASHGV40047057	0.575136	0.575136447	protein_coding	NM_005763
ASHGV40038521	-0.57505	0.575045568	protein_coding	NM_002448
ASHGV40029137	0.57498	0.574980136	protein_coding	NM_022780
ASHGV40002088	-0.57494	0.57494422	protein_coding	ENST00000553728
ASHGV40045506	0.574715	0.574714583	protein_coding	NM_014845
ASHGV40034818	0.574629	0.574629052	protein_coding	NM_017619
ASHGV40026130	-0.57438	0.574380282	protein_coding	NM_001023563
ASHGV40032206	0.574142	0.574141501	protein_coding	NM_006134
ASHGV40012730	0.573835	0.573834627	protein_coding	NM_012158
ASHGV40009115	-0.57378	0.573781953	protein_coding	NM_001136105
ASHGV40024965	-0.5737	0.573695364	protein_coding	NM_005883
ASHGV40016259	0.57363	0.573630404	protein_coding	NM_025238
ASHGV40035805	-0.57342	0.573420859	protein_coding	NM_005108
ASHGV40049066	0.573415	0.573415039	protein_coding	NM_004895
ASHGV40003304	-0.57341	0.573414789	protein_coding	NM_024054
ASHGV40049446	0.573312	0.573312191	protein_coding	ENST00000523987
ASHGV40043518	0.573239	0.573238551	protein_coding	NM_002923
ASHGV40025619	0.573207	0.573207172	protein_coding	NM_032825
ASHGV40001083	-0.5732	0.573196354	protein_coding	ENST00000450043
ASHGV40029674	0.573129	0.573128923	protein_coding	NM_000408
ASHGV40048680	0.573054	0.57305379	protein_coding	NM_004769
ASHGV40002372	-0.57292	0.572923498	protein_coding	NM_001103154
ASHGV40014113	-0.57291	0.572905127	protein_coding	NM_006862
ASHGV40053230	0.57274	0.572739948	protein_coding	NM_019592
ASHGV40002588	-0.57262	0.572620858	protein_coding	ENST00000593873
ASHGV40039086	-0.5725	0.572504511	protein_coding	NM_003943
ASHGV40029048	-0.57238	0.572375591	protein_coding	NM_001381
ASHGV40055239	-0.57232	0.572322333	protein_coding	NM_182541
ASHGV40016012	-0.57224	0.572236128	protein_coding	NM_020962
ASHGV40043266	-0.57197	0.57196624	protein_coding	NM_147130
ASHGV40028625	0.57195	0.571950118	protein_coding	NM_016131
ASHGV40038556	-0.57179	0.571792428	protein_coding	NM_178348
ASHGV40013731	-0.57174	0.571743863	protein_coding	NM_024979
ASHGV40028338	0.571722	0.571721915	protein_coding	NM_015963
ASHGV40036004	0.571606	0.571605519	protein_coding	NM_014041
ASHGV40024818	-0.57157	0.571569746	protein_coding	NM_000641
ASHGV40026613	-0.57132	0.57132088	protein_coding	NM_005813
ASHGV40037094	-0.57128	0.571283381	protein_coding	NM_001137608
ASHGV40018824	0.57122	0.57121961	protein_coding	NM_032486
ASHGV40024662	0.571197	0.571197361	protein_coding	NM_004977
ASHGV40031853	0.57105	0.571050242	protein_coding	NM_001001433
ASHGV40030899	-0.57094	0.570944081	protein_coding	NM_147198
ASHGV40019490	0.570838	0.570838396	protein_coding	NM_001537
ASHGV40023444	-0.57077	0.570766021	protein_coding	NM_002091
ASHGV40034173	0.570727	0.57072744	protein_coding	NM_014805

ASHGV40014048	0.570679	0.57067857	protein_coding	NM_007185
ASHGV40017384	-0.57054	0.570540954	protein_coding	NM_001102450
ASHGV40025515	0.570535	0.570534903	protein_coding	NM_014910
ASHGV40020514	0.570424	0.570423889	protein_coding	NM_007241
ASHGV40002358	0.570287	0.570287111	protein_coding	ENST00000569622
ASHGV40054955	-0.57028	0.570280307	protein_coding	NM_033626
ASHGV40036499	-0.57023	0.570232618	protein_coding	NM_013336
ASHGV40038153	0.569837	0.569836638	protein_coding	NM_017639
ASHGV40031782	-0.56982	0.569815637	protein_coding	ENST00000439873
ASHGV40035647	-0.5698	0.569795888	protein_coding	NM_016474
ASHGV40019779	0.569686	0.569686298	protein_coding	NM_001365
ASHGV40039917	0.569663	0.5696634	protein_coding	NM_024091
ASHGV40012545	-0.5696	0.569597447	protein_coding	NM_002298
ASHGV40023790	0.56937	0.56937043	protein_coding	NM_024103
ASHGV40042754	0.569337	0.569337388	protein_coding	NM_030627
ASHGV40048628	-0.56932	0.569318148	protein_coding	NM_170686
ASHGV40054273	-0.5693	0.569302474	protein_coding	NM_080879
ASHGV40053205	0.569049	0.569049259	protein_coding	NM_006401
ASHGV40040876	0.568914	0.568913982	protein_coding	NM_022464
ASHGV40007436	0.568748	0.568748042	protein_coding	NM_018982
ASHGV40006512	-0.56857	0.568567398	protein_coding	NM_199292
ASHGV40054416	0.568262	0.568262213	protein_coding	NM_016032
ASHGV40016859	-0.56791	0.567905729	protein_coding	NM_032866
ASHGV40030923	-0.56777	0.567770383	protein_coding	NM_080721
ASHGV40034530	-0.56759	0.567586528	protein_coding	NM_182522
ASHGV40032410	-0.56749	0.567491973	protein_coding	NM_006657
ASHGV40048202	-0.5674	0.567396797	protein_coding	NM_014569
ASHGV40002589	0.567351	0.567350856	protein_coding	ENST00000594059
ASHGV40009572	-0.56732	0.567323061	protein_coding	NM_001130711
ASHGV40034354	-0.5671	0.567097444	protein_coding	NM_020998
ASHGV40043973	-0.56699	0.566993577	protein_coding	NM_001105208
ASHGV40019801	0.566945	0.566945115	protein_coding	NM_133491
ASHGV40027658	-0.56693	0.56692642	protein_coding	NM_004482
ASHGV40057668	-0.56669	0.566689175	protein_coding	NM_001017991
ASHGV40020994	-0.56646	0.566458434	protein_coding	NM_017921
ASHGV40047439	0.5664	0.566399742	protein_coding	NM_002360
ASHGV40017069	-0.5663	0.566296578	protein_coding	NM_001164404
ASHGV40053994	-0.56621	0.566210125	protein_coding	NM_006875
ASHGV40028976	-0.5662	0.566197065	protein_coding	NM_032208
ASHGV40025560	-0.56617	0.5661728	protein_coding	ENST00000313865
ASHGV40018628	-0.56578	0.565778599	protein_coding	NM_024845
ASHGV40048215	-0.56569	0.565686657	protein_coding	NM_152755
ASHGV40028084	0.565669	0.565668596	protein_coding	NM_002846
ASHGV40026222	0.565662	0.565661561	protein_coding	NM_001303052
ASHGV40006581	-0.56561	0.565610678	protein_coding	NM_033034
ASHGV40047248	-0.56558	0.565583558	protein_coding	NM_000420
ASHGV40029362	0.565583	0.565582516	protein_coding	NM_006367

ASHGV40047101	0.565482	0.565482102	protein_coding	NM_022143
ASHGV40003153	-0.56545	0.565446067	protein_coding	NM_001198845
ASHGV40054266	0.565202	0.565202155	protein_coding	NM_022052
ASHGV40052862	0.565197	0.56519739	protein_coding	NM_001012421
ASHGV40014812	0.565151	0.565150573	protein_coding	NM_006263
ASHGV40047533	0.565061	0.565061254	protein_coding	NM_001112706
ASHGV40016675	-0.56502	0.565016935	protein_coding	NM_130468
ASHGV40019737	0.564966	0.564966117	protein_coding	NM_004890
ASHGV40037953	-0.56493	0.564932135	protein_coding	NM_012113
ASHGV40054553	0.564917	0.564916717	protein_coding	NM_004344
ASHGV40044196	-0.56469	0.564694816	protein_coding	NM_182503
ASHGV40015131	-0.56469	0.564690814	protein_coding	NM_152443
ASHGV40011956	0.564647	0.564646942	protein_coding	NM_007076
ASHGV40038921	0.564573	0.564572574	protein_coding	NM_018261
ASHGV40031906	0.564484	0.564483735	protein_coding	NM_001024
ASHGV40050063	-0.56443	0.564434961	protein_coding	NM_145003
ASHGV40024683	-0.56424	0.564239389	protein_coding	NM_007196
ASHGV40025184	-0.56414	0.564137256	protein_coding	NM_000527
ASHGV40003075	-0.56405	0.5640473	protein_coding	NM_001010978
ASHGV40034723	-0.56401	0.56401352	protein_coding	NM_032579
ASHGV40032503	-0.564	0.564004785	protein_coding	NM_181719
ASHGV40054479	0.563842	0.563842187	protein_coding	NM_004114
ASHGV40015935	0.563793	0.563792771	protein_coding	NM_004492
ASHGV40007123	-0.56378	0.563782165	protein_coding	uc001nyr.1
ASHGV40008492	-0.56371	0.563707656	protein_coding	NM_023945
ASHGV40053504	-0.56358	0.56357862	protein_coding	NM_018201
ASHGV40039149	-0.56344	0.563439936	protein_coding	NM_014606
ASHGV40009161	0.56337	0.563369875	protein_coding	NM_006169
ASHGV40051677	0.563266	0.563266168	protein_coding	NM_032596
ASHGV40024091	-0.56314	0.56313747	protein_coding	NM_006532
ASHGV40017480	-0.563	0.563001321	protein_coding	NM_005061
ASHGV40046139	-0.563	0.562999541	protein_coding	NM_001145118
ASHGV40043013	-0.56291	0.56290621	protein_coding	NM_005906
ASHGV40047593	-0.56285	0.562850905	protein_coding	NM_001277115
ASHGV40051903	-0.56279	0.562789525	protein_coding	NM_174938
ASHGV40048199	0.562633	0.562633074	protein_coding	NM_003910
ASHGV40056231	-0.56255	0.562551919	protein_coding	NM_004675
ASHGV40009104	0.562525	0.562525393	protein_coding	NM_004398
ASHGV40021013	-0.56234	0.562343177	protein_coding	NM_006907
ASHGV40008433	-0.56228	0.562277545	protein_coding	NM_001005204
ASHGV40038928	-0.56212	0.562119474	protein_coding	NM_006452
ASHGV40018631	-0.56199	0.561987204	protein_coding	NM_005223
ASHGV40016241	0.561585	0.56158532	protein_coding	NM_001164465
ASHGV40035542	0.561577	0.561577275	protein_coding	NM_014461
ASHGV40016222	0.561482	0.561482314	protein_coding	NM_199351
ASHGV40047502	0.561462	0.561461813	protein_coding	NM_017560
ASHGV40034745	0.561457	0.561457349	protein_coding	NM_001400

ASHGV40047903	0.561412	0.56141202	protein_coding	NM_001146333
ASHGV40057332	-0.5614	0.561396737	protein_coding	NM_032317
ASHGV40043269	-0.56139	0.561389264	protein_coding	NM_033177
ASHGV40033237	-0.56126	0.561256775	protein_coding	NM_015140
ASHGV40025604	0.561238	0.561238096	protein_coding	ENST00000378887
ASHGV40049665	-0.56113	0.561131365	protein_coding	NM_001001557
ASHGV40014072	0.560853	0.560852877	protein_coding	NM_031420
ASHGV40025771	-0.56084	0.560844134	protein_coding	NM_003445
ASHGV40028504	-0.56082	0.56081964	protein_coding	NM_022753
ASHGV40025886	-0.56077	0.560767156	protein_coding	NM_138761
ASHGV40016251	-0.56073	0.560732832	protein_coding	ENST00000561157
ASHGV40043209	0.560732	0.560732157	protein_coding	NM_001470
ASHGV40009570	-0.56065	0.56065421	protein_coding	NM_001781
ASHGV40051172	-0.56065	0.560647067	protein_coding	NM_018024
ASHGV40024945	-0.56063	0.560628493	protein_coding	NM_138690
ASHGV40047193	0.560469	0.560468686	protein_coding	NM_194071
ASHGV40007209	0.560376	0.560376296	protein_coding	NM_207340
ASHGV40023685	0.560285	0.560285384	protein_coding	NM_006830
ASHGV40047169	0.560247	0.560246893	protein_coding	NM_032826
ASHGV40019359	-0.5602	0.56019851	protein_coding	NM_001137675
ASHGV40036432	0.560068	0.560067597	protein_coding	NM_021082
ASHGV40026135	-0.56003	0.560030109	protein_coding	NM_012387
ASHGV40001306	-0.56	0.559995933	protein_coding	NM_025165
ASHGV40034774	0.559958	0.559957899	protein_coding	NM_001164342
ASHGV40013725	0.559955	0.559954902	protein_coding	NM_015205
ASHGV40046214	-0.55987	0.55986906	protein_coding	NM_175886
ASHGV40049710	0.559777	0.559777214	protein_coding	NM_002379
ASHGV40029993	0.559681	0.559680742	protein_coding	NM_001204
ASHGV40051751	-0.55948	0.559483428	protein_coding	uc004abo.4
ASHGV40053506	0.559415	0.559414811	protein_coding	NM_019594
ASHGV40052545	0.559404	0.559403827	protein_coding	NM_001004354
ASHGV40025866	-0.55918	0.559177448	protein_coding	NM_012451
ASHGV40001258	0.55911	0.559109833	protein_coding	NM_020357
ASHGV40035882	-0.55897	0.558968894	protein_coding	NM_144638
ASHGV40056235	0.558884	0.558884236	protein_coding	uc002cra.1
ASHGV40016375	-0.5587	0.558703226	protein_coding	uc002bte.1
ASHGV40042439	-0.55864	0.558642926	protein_coding	NM_018940
ASHGV40042464	-0.55842	0.558424681	protein_coding	NM_018926
ASHGV40025727	-0.55842	0.558416378	protein_coding	ENST00000540732
ASHGV40025309	-0.55841	0.558407016	protein_coding	NM_024074
ASHGV40025227	0.558363	0.558363351	protein_coding	NM_000159
ASHGV40011759	0.558154	0.558154064	protein_coding	NM_152588
ASHGV40018539	-0.558	0.558003074	protein_coding	NM_024600
ASHGV40053821	-0.55795	0.557949418	protein_coding	NM_000292
ASHGV40024444	0.557707	0.55770716	protein_coding	NM_005357
ASHGV40011141	0.55768	0.557679792	protein_coding	NM_024854
ASHGV40002346	-0.55766	0.557663877	protein_coding	NM_182854

ASHGV40033790	0.557662	0.55766225	protein_coding	NM_145733
ASHGV40019990	0.557486	0.557486394	protein_coding	NM_015681
ASHGV40023679	-0.55735	0.557349606	protein_coding	NM_213604
ASHGV40060868	-0.55734	0.557342003	protein_coding	uc010kyk.3
ASHGV40013288	0.557299	0.557299298	protein_coding	uc021oww.1
ASHGV40011022	0.557249	0.557249229	protein_coding	NM_017818
ASHGV40020613	0.557076	0.557076307	protein_coding	NM_007146
ASHGV40014660	0.556957	0.55695719	protein_coding	NM_001010846
ASHGV40025918	0.556699	0.556698738	protein_coding	NM_152359
ASHGV40034322	0.556694	0.556693906	protein_coding	NM_000387
ASHGV40028700	0.556536	0.556536028	protein_coding	NM_001134734
ASHGV40043969	0.556501	0.556501233	protein_coding	NM_016262
ASHGV40051682	-0.55637	0.556372185	protein_coding	NM_148178
ASHGV40020672	0.556213	0.556213201	protein_coding	NM_005121
ASHGV40041850	0.556172	0.556172269	protein_coding	NM_015569
ASHGV40005559	-0.55598	0.555977602	protein_coding	uc010qfa.1
ASHGV40049874	0.555656	0.555655937	protein_coding	NM_032847
ASHGV40041640	-0.55553	0.555532615	protein_coding	NM_003999
ASHGV40018800	-0.55542	0.555424185	protein_coding	ENST00000330898
ASHGV40025813	-0.55533	0.555325958	protein_coding	NM_003370
ASHGV40025279	0.555303	0.555302838	protein_coding	NM_173483
ASHGV40024972	-0.55529	0.5552924	protein_coding	NM_001080488
ASHGV40007215	0.555201	0.555200549	protein_coding	NM_006946
ASHGV40054422	-0.55517	0.555174747	protein_coding	NM_178471
ASHGV40008635	-0.5551	0.555097502	protein_coding	NM_002689
ASHGV40031489	-0.55501	0.555005344	protein_coding	NM_015338
ASHGV40006654	0.554967	0.554966698	protein_coding	NM_016422
ASHGV40000240	-0.55484	0.554842842	protein_coding	NM_001193289
ASHGV40021759	-0.55443	0.554431409	protein_coding	NM_003734
ASHGV40034070	0.554094	0.554093506	protein_coding	NM_144715
ASHGV40034888	0.554079	0.554078982	protein_coding	NM_003418
ASHGV40023957	-0.55402	0.554020907	protein_coding	NM_001375
ASHGV40042459	-0.55395	0.553947281	protein_coding	NM_018920
ASHGV40056124	-0.55383	0.55383491	protein_coding	NM_024644
ASHGV40036913	0.553725	0.55372466	protein_coding	NM_001304472
ASHGV40054459	-0.55359	0.553590008	protein_coding	NM_001291527
ASHGV40019547	-0.55354	0.553537319	protein_coding	NM_005250
ASHGV40004992	0.553433	0.553432907	protein_coding	NM_006793
ASHGV40047769	0.553329	0.553328629	protein_coding	NM_000712
ASHGV40029418	0.553318	0.553318422	protein_coding	NM_002881
ASHGV40047428	-0.55296	0.552955644	protein_coding	NM_001505
ASHGV40057748	-0.5529	0.552895823	protein_coding	NM_001017
ASHGV40008163	0.552801	0.552801468	protein_coding	NM_005316
ASHGV40060869	-0.55274	0.552740731	protein_coding	uc010lac.3
ASHGV40028080	0.552714	0.552714021	protein_coding	NM_024085
ASHGV40006995	0.552403	0.552403466	protein_coding	NM_145008
ASHGV40025384	0.552399	0.552399264	protein_coding	NM_004386

ASHGV40050264	-0.5524	0.552395363	protein_coding	ENST00000284481
ASHGV40036035	0.552377	0.552376895	protein_coding	NM_001304420
ASHGV40030362	0.552318	0.552318144	protein_coding	ENST00000409526
ASHGV40027062	-0.55226	0.552259268	protein_coding	NM_013441
ASHGV40021588	0.552153	0.552153338	protein_coding	NM_006273
ASHGV40033687	-0.55196	0.551956252	protein_coding	NM_000631
ASHGV40043309	-0.55194	0.551944337	protein_coding	NM_002586
ASHGV40015065	0.551924	0.551923606	protein_coding	NM_021003
ASHGV40010193	0.551839	0.551838854	protein_coding	NM_032338
ASHGV40034461	-0.55171	0.551714876	protein_coding	NM_152678
ASHGV40021244	-0.55169	0.551691104	protein_coding	NM_001005273
ASHGV40052063	-0.55158	0.551581916	protein_coding	NM_139246
ASHGV40020215	-0.55155	0.551553937	protein_coding	NM_001163735
ASHGV40051533	-0.55155	0.551551783	protein_coding	NM_144966
ASHGV40011873	0.551488	0.551487615	protein_coding	NM_005888
ASHGV40053488	0.551416	0.551415759	protein_coding	NM_001040011
ASHGV40044891	0.551365	0.551365496	protein_coding	NM_014858
ASHGV40048216	0.551253	0.551253357	protein_coding	NM_001008395
ASHGV40053497	-0.55098	0.550976222	protein_coding	NM_001003722
ASHGV40009626	0.550738	0.550737873	protein_coding	uc021qvj.1
ASHGV40006721	0.55056	0.550560497	protein_coding	NM_006292
ASHGV40019869	-0.55049	0.550493793	protein_coding	NM_002470
ASHGV40056496	-0.55041	0.550410052	protein_coding	NM_020230
ASHGV40057364	-0.55025	0.550254758	protein_coding	uc003uze.2
ASHGV40056033	-0.55019	0.550186422	protein_coding	NM_016617
ASHGV40051078	0.550044	0.550044459	protein_coding	NM_020189
ASHGV40023904	-0.55004	0.550041672	protein_coding	NM_020812
ASHGV40018376	0.549838	0.549837795	protein_coding	NM_012396
ASHGV40006824	0.549822	0.549821998	protein_coding	NM_000611
ASHGV40044587	0.549748	0.54974776	protein_coding	NM_001684
ASHGV40027024	-0.54971	0.549712782	protein_coding	NM_001013649
ASHGV40014122	-0.54966	0.549657238	protein_coding	NM_001004432
ASHGV40051388	-0.54961	0.549612253	protein_coding	NM_139021
ASHGV40056879	-0.54956	0.54955921	protein_coding	NM_007081
ASHGV40034101	0.54949	0.549489755	protein_coding	NM_018297
ASHGV40008625	-0.54927	0.549266349	protein_coding	NM_003273
ASHGV40050667	-0.54911	0.54910594	protein_coding	NM_006269
ASHGV40057793	-0.54908	0.549077087	protein_coding	NM_001282456
ASHGV40032210	-0.54889	0.548885675	protein_coding	NM_017613
ASHGV40011878	-0.54872	0.548719453	protein_coding	NM_025188
ASHGV40003128	-0.54865	0.548649883	protein_coding	NM_001145249
ASHGV40036915	-0.54861	0.548608392	protein_coding	NM_004443
ASHGV40053649	0.548315	0.548314612	protein_coding	NM_016215
ASHGV40007383	-0.54831	0.548305382	protein_coding	NM_182833
ASHGV40013812	0.548259	0.548259379	protein_coding	NM_001291446
ASHGV40047493	0.548148	0.548147969	protein_coding	NM_015622
ASHGV40043281	0.547873	0.547872986	protein_coding	NM_021177

ASHGV40052368	0.547499	0.547498823	protein_coding	NM_004059
ASHGV40029860	-0.54739	0.547394574	protein_coding	NM_001080545
ASHGV40008499	-0.54721	0.54721221	protein_coding	NM_001310141
ASHGV40043790	-0.54721	0.547208548	protein_coding	NM_198568
ASHGV40034288	-0.54711	0.547112767	protein_coding	NM_182902
ASHGV40035382	-0.54696	0.546955885	protein_coding	NM_002916
ASHGV40001805	0.546841	0.54684123	protein_coding	NM_018222
ASHGV40050042	-0.54683	0.546834808	protein_coding	NM_001080431
ASHGV40034059	-0.54673	0.54673211	protein_coding	NM_001351
ASHGV40019011	-0.54654	0.546538942	protein_coding	NM_014321
ASHGV40007684	-0.54644	0.546438418	protein_coding	NM_004716
ASHGV40053675	-0.54625	0.54625453	protein_coding	uc022bqd.1
ASHGV40043082	0.546226	0.546226496	protein_coding	NM_198586
ASHGV40055228	-0.54618	0.54618075	protein_coding	NM_001031834
ASHGV40036289	0.546095	0.546095169	protein_coding	NM_017819
ASHGV40029043	-0.54601	0.546010059	protein_coding	NM_022492
ASHGV40007731	-0.546	0.545996821	protein_coding	NM_012101
ASHGV40047469	-0.54588	0.545880768	protein_coding	NM_152744
ASHGV40035417	-0.54585	0.545853224	protein_coding	NM_021101
ASHGV40024885	-0.54583	0.545825537	protein_coding	NM_133460
ASHGV40034603	0.5458	0.54580035	protein_coding	NM_033055
ASHGV40002627	-0.54577	0.54576978	protein_coding	ENST00000596400
ASHGV40006242	0.545749	0.54574912	protein_coding	NM_139169
ASHGV40053976	0.545626	0.545626109	protein_coding	NM_006962
ASHGV40027638	-0.54562	0.54562284	protein_coding	NM_001291807
ASHGV40057670	0.545486	0.545485581	protein_coding	ENST00000369505
ASHGV40051346	0.545463	0.545463262	protein_coding	NM_012392
ASHGV40056532	-0.54546	0.545459772	protein_coding	NM_005304
ASHGV40048956	0.545428	0.545428247	protein_coding	NM_001001924
ASHGV40045722	-0.54536	0.545363688	protein_coding	NM_001008783
ASHGV40025338	0.545361	0.545361345	protein_coding	NM_018174
ASHGV40045145	-0.54532	0.545324451	protein_coding	NM_001010904
ASHGV40020486	-0.54532	0.54531642	protein_coding	NM_024320
ASHGV40016700	-0.54518	0.545179281	protein_coding	NM_001080541
ASHGV40009088	0.545005	0.545004599	protein_coding	NM_015423
ASHGV40008473	-0.54498	0.544978106	protein_coding	NM_001004708
ASHGV40006511	-0.54498	0.544976697	protein_coding	NM_000207
ASHGV40002051	0.544948	0.544947981	protein_coding	uc001tmh.1
ASHGV40022678	0.544868	0.5448683	protein_coding	NM_018170
ASHGV40033982	0.544755	0.544755254	protein_coding	NM_003656
ASHGV40035175	0.544681	0.54468092	protein_coding	NM_001040100
ASHGV40049351	0.544627	0.544627246	protein_coding	NM_014729
ASHGV40042017	0.544537	0.544537407	protein_coding	NM_002890
ASHGV40040822	0.544521	0.544520904	protein_coding	NM_001113575
ASHGV40017860	0.544516	0.544516208	protein_coding	NM_001080417
ASHGV40035008	0.544487	0.544486571	protein_coding	NM_001039547
ASHGV40048733	-0.54436	0.544361968	protein_coding	NM_001427

ASHGV40044920	0.544276	0.544275535	protein_coding	NM_014260
ASHGV40051988	-0.54409	0.544090163	protein_coding	NM_004560
ASHGV40011442	0.544082	0.544082193	protein_coding	NM_006608
ASHGV40007884	0.544029	0.544029113	protein_coding	NM_001085487
ASHGV40039016	0.543873	0.543872587	protein_coding	NM_006685
ASHGV40027251	-0.54381	0.543810878	protein_coding	NM_005434
ASHGV40023486	-0.54366	0.54366387	protein_coding	NM_080475
ASHGV40031847	0.543637	0.543637329	protein_coding	NM_020673
ASHGV40000256	-0.5436	0.543598548	protein_coding	NM_002896
ASHGV40031979	0.543596	0.543596116	protein_coding	NM_012067
ASHGV40013623	-0.54353	0.543534211	protein_coding	NM_001145862
ASHGV40028463	-0.54345	0.543446399	protein_coding	NM_002403
ASHGV40029760	-0.54334	0.543343049	protein_coding	NM_001242750
ASHGV40047954	0.543327	0.543326677	protein_coding	NM_000048
ASHGV40022446	0.543073	0.543073269	protein_coding	uc009vno.2
ASHGV40040864	0.543049	0.543048677	protein_coding	NM_006696
ASHGV40020830	-0.54302	0.543021126	protein_coding	NM_006678
ASHGV40053676	0.542916	0.542916015	protein_coding	NM_001190228
ASHGV40039154	-0.5427	0.542697389	protein_coding	NM_145715
ASHGV40034291	0.542655	0.542655492	protein_coding	NM_001031703
ASHGV40020268	-0.54259	0.542586388	protein_coding	NM_014815
ASHGV40021722	-0.54239	0.542390475	protein_coding	NM_001277332
ASHGV40045156	-0.54235	0.542346248	protein_coding	NM_003221
ASHGV40029055	-0.54229	0.542290838	protein_coding	NM_003462
ASHGV40044212	0.542181	0.542180935	protein_coding	NM_032145
ASHGV40011103	-0.54213	0.542129347	protein_coding	NM_030667
ASHGV40007346	-0.5421	0.542097982	protein_coding	NM_001144869
ASHGV40024474	0.542083	0.542082741	protein_coding	NM_002659
ASHGV40041915	0.54202	0.5420201	protein_coding	NM_001177693
ASHGV40031144	0.541965	0.541964949	protein_coding	NM_024586
ASHGV40053765	-0.54193	0.541927692	protein_coding	NM_000381
ASHGV40050641	0.541757	0.541756828	protein_coding	ENST00000415643
ASHGV40022074	0.541657	0.541657136	protein_coding	NM_016360
ASHGV40051432	0.541636	0.541636453	protein_coding	NM_003416
ASHGV40024802	-0.54138	0.54137537	protein_coding	NM_001145971
ASHGV40015735	-0.54117	0.541169408	protein_coding	NM_004573
ASHGV40000703	-0.54117	0.541168572	protein_coding	ENST00000430850
ASHGV40006074	0.541035	0.541034808	protein_coding	NM_015960
ASHGV40055909	0.540911	0.540910655	protein_coding	NM_138428
ASHGV40024919	-0.54086	0.540855921	protein_coding	NM_130760
ASHGV40000745	0.540814	0.540814217	protein_coding	ENST00000432920
ASHGV40029663	0.540686	0.540686353	protein_coding	NM_002239
ASHGV40035013	-0.54057	0.540569402	protein_coding	NM_013363
ASHGV40025169	0.54041	0.540409804	protein_coding	NM_001111307
ASHGV40030559	0.540361	0.540360553	protein_coding	NM_016649
ASHGV40048128	0.540351	0.54035067	protein_coding	NM_005751
ASHGV40029647	0.54015	0.540149849	protein_coding	NM_018151

ASHGV40041929	0.540025	0.540025198	protein_coding	NM_016218
ASHGV40005290	-0.53987	0.539869046	protein_coding	NM_031923
ASHGV40028991	0.539769	0.53976924	protein_coding	NM_016297
ASHGV40045111	-0.53958	0.53958452	protein_coding	NM_015326
ASHGV40043299	-0.53957	0.539566938	protein_coding	NM_004381
ASHGV40028065	0.539511	0.539511	protein_coding	NM_022453
ASHGV40014499	0.539497	0.539497462	protein_coding	NM_030621
ASHGV40011919	0.539495	0.539495153	protein_coding	NM_001008394
ASHGV40046731	-0.53937	0.539365621	protein_coding	NM_012431
ASHGV40025127	0.539335	0.539334612	protein_coding	NM_005968
ASHGV40013101	-0.5392	0.539201466	protein_coding	uc001eqa.3
ASHGV40007458	0.539195	0.539195094	protein_coding	NM_012124
ASHGV40011438	0.539175	0.539174655	protein_coding	NM_002624
ASHGV40053580	-0.53913	0.539131229	protein_coding	NM_017586
ASHGV40006100	0.5391	0.539100261	protein_coding	NM_032429
ASHGV40041689	0.539097	0.539097353	protein_coding	NM_012343
ASHGV40056376	0.538948	0.538947663	protein_coding	ENST00000370079
ASHGV40019792	-0.53894	0.538937599	protein_coding	ENST00000535512
ASHGV40049068	-0.53883	0.538832015	protein_coding	NM_001004698
ASHGV40035797	-0.53872	0.538715213	protein_coding	NM_002207
ASHGV40011223	0.538659	0.538659127	protein_coding	NM_001714
ASHGV40012341	0.538455	0.538454897	protein_coding	NM_014363
ASHGV40050071	-0.53845	0.538449323	protein_coding	NM_205545
ASHGV40013905	-0.53824	0.538241618	protein_coding	ENST00000447795
ASHGV40048989	0.538198	0.538197642	protein_coding	NM_005144
ASHGV40017432	-0.53802	0.538023533	protein_coding	NM_016541
ASHGV40042356	-0.53777	0.537772726	protein_coding	NM_001001419
ASHGV40024961	0.537735	0.537734978	protein_coding	NM_024407
ASHGV40035980	-0.53764	0.537639941	protein_coding	NM_000839
ASHGV40014623	-0.53746	0.537458564	protein_coding	NM_005432
ASHGV40017123	-0.53715	0.537148952	protein_coding	NM_007314
ASHGV40048517	-0.53697	0.536967119	protein_coding	NM_015135
ASHGV40052355	0.536745	0.536744858	protein_coding	NM_015679
ASHGV40008011	-0.53671	0.536711739	protein_coding	ENST00000445557
ASHGV40033985	-0.53664	0.53664454	protein_coding	NM_173659
ASHGV40038284	0.536597	0.536596659	protein_coding	NM_012180
ASHGV40044264	0.536536	0.536535963	protein_coding	NM_198149
ASHGV40042802	-0.53653	0.536534021	protein_coding	NM_030567
ASHGV40000106	0.53635	0.536349937	protein_coding	NM_001170905
ASHGV40035988	0.536327	0.536327135	protein_coding	NM_015407
ASHGV40041220	-0.53587	0.535872551	protein_coding	NM_002115
ASHGV40056524	0.535823	0.535822925	protein_coding	NM_001039888
ASHGV40048505	0.535738	0.535738215	protein_coding	NM_001724
ASHGV40018719	-0.5357	0.535699417	protein_coding	NM_005236
ASHGV40057180	-0.53556	0.535560496	protein_coding	NM_003530
ASHGV40054005	0.535508	0.535507956	protein_coding	NM_005183
ASHGV40001274	-0.53544	0.535440359	protein_coding	uc001jtv.4

ASHGV40030854	0.535259	0.535258949	protein_coding	NM_032221
ASHGV40021543	-0.53521	0.53521398	protein_coding	NM_018404
ASHGV40020327	-0.53514	0.535143734	protein_coding	NM_002276
ASHGV40018794	0.53501	0.535010235	protein_coding	NM_016025
ASHGV40041325	-0.53487	0.534872412	protein_coding	ENST00000594226
ASHGV40015158	0.53486	0.534859894	protein_coding	NM_014784
ASHGV40011545	-0.53468	0.534676114	protein_coding	NM_005412
ASHGV40014949	0.534489	0.534489046	protein_coding	NM_017922
ASHGV40033437	-0.53439	0.534389339	protein_coding	NM_003426
ASHGV40057500	-0.53424	0.534240525	protein_coding	NM_002170
ASHGV40050034	0.534185	0.534184861	protein_coding	NM_153831
ASHGV40017713	0.534003	0.534002762	protein_coding	NM_001142725
ASHGV40026246	0.533883	0.533882586	protein_coding	NM_002936
ASHGV40045521	-0.53386	0.533863246	protein_coding	NM_018593
ASHGV40002583	-0.5338	0.533803784	protein_coding	ENST00000593391
ASHGV40017757	-0.53374	0.53373954	protein_coding	NM_001012981
ASHGV40025204	-0.5337	0.533704192	protein_coding	NM_144566
ASHGV40000087	0.533666	0.533665615	protein_coding	NM_001010880
ASHGV40017150	-0.53362	0.533618208	protein_coding	NM_018689
ASHGV40056646	-0.53361	0.533614663	protein_coding	NM_005987
ASHGV40009131	0.533489	0.533489084	protein_coding	NM_001931
ASHGV40016713	-0.53342	0.533422885	protein_coding	NM_000070
ASHGV40007643	-0.53321	0.533205816	protein_coding	NM_000795
ASHGV40043340	-0.5332	0.533202978	protein_coding	NM_002119
ASHGV40043821	0.533149	0.533149257	protein_coding	NM_145331
ASHGV40010497	0.532781	0.53278062	protein_coding	NM_198521
ASHGV40051906	-0.53266	0.532664919	protein_coding	NM_017576
ASHGV40041699	0.532623	0.532623481	protein_coding	NM_016640
ASHGV40056381	0.532559	0.532558974	protein_coding	NM_003079
ASHGV40011198	0.532365	0.532364867	protein_coding	NM_018099
ASHGV40046899	-0.53236	0.532357244	protein_coding	uc003uzz.4
ASHGV40056846	-0.53224	0.532236894	protein_coding	uc021wmb.1
ASHGV40018450	0.532158	0.532158198	protein_coding	NM_002768
ASHGV40055215	0.532111	0.532110749	protein_coding	NM_016607
ASHGV40055078	-0.5318	0.531795477	protein_coding	NM_004606
ASHGV40040228	0.531778	0.531777679	protein_coding	NM_004531
ASHGV40047557	0.531507	0.531507317	protein_coding	NM_014399
ASHGV40060891	0.531441	0.531441236	protein_coding	uc011lqu.1
ASHGV40039768	-0.53133	0.531329094	protein_coding	NM_207352
ASHGV40053550	-0.53124	0.531235937	protein_coding	NM_032728
ASHGV40000609	0.531055	0.531054841	protein_coding	NM_025029
ASHGV40057338	-0.53099	0.530985929	protein_coding	NM_001306141
ASHGV40025855	0.530956	0.530955736	protein_coding	NM_015710
ASHGV40041642	0.530944	0.530944028	protein_coding	NM_022716
ASHGV40010152	0.53091	0.530909559	protein_coding	NM_020700
ASHGV40014967	0.530877	0.530876715	protein_coding	NM_172193
ASHGV40034817	0.530763	0.530763356	protein_coding	NM_002264

ASHGV40057762	0.53075	0.530749974	protein_coding	NM_003654
ASHGV40015047	-0.5307	0.530696274	protein_coding	NM_015590
ASHGV40034106	0.530692	0.53069215	protein_coding	NM_152534
ASHGV40043751	-0.53068	0.53068208	protein_coding	NM_017633
ASHGV40032588	-0.53065	0.530647416	protein_coding	NM_058187
ASHGV40051143	-0.53054	0.530542637	protein_coding	NM_021110
ASHGV40011892	0.53053	0.530529934	protein_coding	NM_001286615
ASHGV40025720	0.530523	0.530522962	protein_coding	NM_001278599
ASHGV40025667	-0.53018	0.530178486	protein_coding	NM_021107
ASHGV40055553	-0.53011	0.530108824	protein_coding	NM_005629
ASHGV40036670	0.530002	0.530002411	protein_coding	NM_014779
ASHGV40014266	-0.52969	0.529693728	protein_coding	NM_001280542
ASHGV40053138	0.529694	0.529693524	protein_coding	NM_014612
ASHGV40026631	0.52953	0.52953029	protein_coding	uc002rqv.3
ASHGV40031377	0.529484	0.529484228	protein_coding	NM_018993
ASHGV40024631	-0.52945	0.52945275	protein_coding	NM_002152
ASHGV40009395	0.529299	0.529298513	protein_coding	NM_032801
ASHGV40030403	0.529168	0.529167733	protein_coding	NM_014808
ASHGV40025653	-0.52904	0.529036444	protein_coding	NM_001042522
ASHGV40006699	-0.52902	0.529024661	protein_coding	NM_017508
ASHGV40042819	0.528913	0.528913321	protein_coding	uc003mil.1
ASHGV40011348	-0.52889	0.528894625	protein_coding	NM_024902
ASHGV40016319	-0.52886	0.528857107	protein_coding	NM_005928
ASHGV40022755	0.528848	0.528847781	protein_coding	NM_032124
ASHGV40017486	-0.52876	0.528760622	protein_coding	ENST00000262304
ASHGV40020629	0.528729	0.528728639	protein_coding	NM_004958
ASHGV40035804	-0.52869	0.528690042	protein_coding	NM_004803
ASHGV40044709	-0.52854	0.528538531	protein_coding	NM_015895
ASHGV40056919	0.528517	0.528516993	protein_coding	NM_176815
ASHGV40026670	-0.52841	0.528408861	protein_coding	NM_007352
ASHGV40022635	-0.52833	0.528332005	protein_coding	NM_001004695
ASHGV40011401	0.528311	0.528310549	protein_coding	NM_002135
ASHGV40034318	0.528252	0.528252452	protein_coding	NM_016453
ASHGV40006804	-0.52822	0.528216502	protein_coding	NM_020869
ASHGV40033097	0.528111	0.528110741	protein_coding	NM_030965
ASHGV40033348	0.528024	0.528023717	protein_coding	NM_012401
ASHGV40040605	0.528007	0.528006718	protein_coding	NM_016406
ASHGV40034900	0.527995	0.527995308	protein_coding	NM_001017395
ASHGV40011751	0.527947	0.527946686	protein_coding	NM_024560
ASHGV40025118	-0.52791	0.527905351	protein_coding	NM_004565
ASHGV40046121	-0.52765	0.527651233	protein_coding	NM_024963
ASHGV40044656	0.527574	0.527573886	protein_coding	NM_016255
ASHGV40049007	-0.52755	0.527554196	protein_coding	NM_144962
ASHGV40020210	-0.52752	0.527521899	protein_coding	ENST00000308078
ASHGV40003230	-0.52737	0.527371518	protein_coding	NM_001302551
ASHGV40011636	0.527328	0.52732779	protein_coding	NM_018448
ASHGV40006490	0.527233	0.527233354	protein_coding	NM_005961

ASHGV40024125	0.527097	0.527096965	protein_coding	NM_001076675
ASHGV40033239	-0.52701	0.527010025	protein_coding	NM_173050
ASHGV40008300	0.526994	0.526993665	protein_coding	NM_138787
ASHGV40048575	-0.52698	0.526984129	protein_coding	uc022anu.1
ASHGV40000166	0.526978	0.526978297	protein_coding	NM_001085457
ASHGV40018402	-0.52681	0.526808038	protein_coding	uc021tmk.2
ASHGV40027471	-0.52673	0.526725212	protein_coding	NM_005915
ASHGV40024718	0.526678	0.526677911	protein_coding	NM_021632
ASHGV40034375	-0.5266	0.526602792	protein_coding	NM_033159
ASHGV40043633	0.526553	0.526553126	protein_coding	NM_030820
ASHGV40023165	0.526516	0.526516209	protein_coding	NM_031216
ASHGV40039096	-0.52642	0.526422238	protein_coding	NM_001826
ASHGV40050275	0.526402	0.526401995	protein_coding	NM_004462
ASHGV40039098	-0.52638	0.526377869	protein_coding	NM_025074
ASHGV40002520	-0.52625	0.526249919	protein_coding	ENST00000587459
ASHGV40037116	0.526196	0.526195738	protein_coding	NM_005105
ASHGV40007590	-0.52618	0.526184803	protein_coding	NM_176782
ASHGV40034219	-0.52618	0.526176854	protein_coding	uc010hib.2
ASHGV40024310	-0.52617	0.526172059	protein_coding	NM_020951
ASHGV40017262	-0.52616	0.526160496	protein_coding	NM_001004309
ASHGV40055018	-0.52613	0.52613419	protein_coding	NM_007250
ASHGV40027879	-0.52612	0.526123116	protein_coding	NM_001048183
ASHGV40000779	-0.52594	0.525944982	protein_coding	ENST00000434291
ASHGV40025022	-0.52585	0.525854692	protein_coding	NM_172251
ASHGV40040661	-0.52584	0.525835675	protein_coding	NM_022140
ASHGV40003808	-0.52582	0.525821998	protein_coding	uc010gac.2
ASHGV40025875	-0.52575	0.525747416	protein_coding	NM_133498
ASHGV40038873	0.525688	0.525688001	protein_coding	NM_001040402
ASHGV40050050	-0.5256	0.525600428	protein_coding	NM_017422
ASHGV40025319	0.525549	0.525548589	protein_coding	NM_004145
ASHGV40028304	0.525545	0.5255454	protein_coding	NM_001080835
ASHGV40056788	-0.52551	0.525514171	protein_coding	NM_001032288
ASHGV40050219	0.525441	0.525440743	protein_coding	NM_018361
ASHGV40002404	-0.52535	0.525348962	protein_coding	NM_001201479
ASHGV40037809	0.525265	0.525265256	protein_coding	NM_145244
ASHGV40025196	-0.52524	0.52524182	protein_coding	NM_145295
ASHGV40025239	0.525169	0.525168724	protein_coding	NM_030818
ASHGV40017852	-0.52506	0.525058143	protein_coding	NM_024671
ASHGV40024817	-0.52497	0.52497057	protein_coding	NM_001145402
ASHGV40027740	-0.52481	0.5248082	protein_coding	NM_001193308
ASHGV40002097	-0.52476	0.5247647	protein_coding	uc001ter.1
ASHGV40034355	-0.52475	0.524749658	protein_coding	NM_198722
ASHGV40021600	0.524502	0.524502301	protein_coding	NM_052857
ASHGV40031067	-0.52445	0.524453727	protein_coding	NM_030776
ASHGV40053978	-0.52416	0.52416327	protein_coding	NM_175723
ASHGV40048231	0.524111	0.524110945	protein_coding	uc001brt.1
ASHGV40045331	0.524036	0.524036276	protein_coding	NM_000574

ASHGV40014463	0.523928	0.523927677	protein_coding	NM_004239
ASHGV40003297	-0.52385	0.523847709	protein_coding	NM_021089
ASHGV40005171	0.523781	0.52378149	protein_coding	NM_006624
ASHGV40029215	-0.52371	0.523709955	protein_coding	NM_001298
ASHGV40021217	-0.52363	0.523630791	protein_coding	NM_198552
ASHGV40041301	0.52353	0.523530328	protein_coding	NM_006098
ASHGV40035540	-0.52348	0.523482141	protein_coding	NM_022768
ASHGV40011051	-0.52347	0.52347326	protein_coding	ENST00000355819
ASHGV40032822	0.523344	0.523343843	protein_coding	NM_001196
ASHGV40025966	-0.52313	0.523128113	protein_coding	NM_001101372
ASHGV40003273	0.523076	0.523075629	protein_coding	NM_005999
ASHGV40036655	0.523042	0.52304186	protein_coding	NM_032383
ASHGV40026079	-0.52295	0.52294736	protein_coding	NM_033113
ASHGV40025995	-0.52293	0.522934635	protein_coding	NM_032423
ASHGV40030827	0.522866	0.522865596	protein_coding	NM_001029864
ASHGV40007440	0.522813	0.522813309	protein_coding	NM_012193
ASHGV40055983	0.522678	0.522677613	protein_coding	NM_003348
ASHGV40052199	0.522532	0.522532227	protein_coding	NM_001012361
ASHGV40055382	0.522447	0.522446886	protein_coding	NM_001282195
ASHGV40030914	-0.52231	0.522311705	protein_coding	NM_020967
ASHGV40029646	0.522182	0.522181704	protein_coding	NM_007115
ASHGV40015544	0.522167	0.522167357	protein_coding	NM_152328
ASHGV40048045	0.522128	0.522127912	protein_coding	NM_002835
ASHGV40044320	0.521959	0.521958649	protein_coding	NM_031924
ASHGV40035127	0.521956	0.521956122	protein_coding	NM_001130960
ASHGV40044062	0.521955	0.521954681	protein_coding	NM_001142569
ASHGV40008718	-0.52189	0.521889678	protein_coding	ENST00000294635
ASHGV40014914	-0.5218	0.521801702	protein_coding	NM_001049
ASHGV40016166	0.521787	0.521786631	protein_coding	NM_000126
ASHGV40053662	-0.52172	0.521720989	protein_coding	NM_001277058
ASHGV40031699	0.521685	0.521684816	protein_coding	NM_001134771
ASHGV40032182	-0.52168	0.52168298	protein_coding	NM_018944
ASHGV40034980	-0.52168	0.521681125	protein_coding	NM_001134659
ASHGV40003327	-0.52161	0.521610029	protein_coding	NM_080489
ASHGV40054073	0.52158	0.521580157	protein_coding	NM_001010862
ASHGV40036363	-0.52149	0.52148948	protein_coding	NM_001171747
ASHGV40008125	0.521478	0.521477628	protein_coding	NM_032228
ASHGV40050989	-0.52128	0.521282304	protein_coding	NM_015029
ASHGV40011492	-0.52123	0.521231826	protein_coding	NM_001005490
ASHGV40027041	0.521192	0.521192443	protein_coding	NM_004931
ASHGV40015360	0.521039	0.521038996	protein_coding	NM_058237
ASHGV40013318	-0.52102	0.521024764	protein_coding	NM_001507
ASHGV40051612	0.521014	0.521014047	protein_coding	NM_001031689
ASHGV40050838	-0.52099	0.520993346	protein_coding	NM_024721
ASHGV40029424	-0.52094	0.520939366	protein_coding	NM_198494
ASHGV40047460	-0.5209	0.520900347	protein_coding	NM_025250
ASHGV40052064	0.520893	0.520893181	protein_coding	NM_000380

ASHGV40011828	-0.52083	0.520827281	protein_coding	NM_003877
ASHGV40006046	-0.52073	0.520732046	protein_coding	NM_032327
ASHGV40007080	0.520672	0.520671693	protein_coding	NM_153265
ASHGV40057345	-0.52061	0.520607909	protein_coding	NM_001005522
ASHGV40029145	-0.52056	0.520556857	protein_coding	NM_018271
ASHGV40025569	0.520361	0.520361387	protein_coding	NM_003367
ASHGV40006538	-0.52033	0.520331863	protein_coding	NM_020402
ASHGV40026992	0.520295	0.52029498	protein_coding	NM_017761
ASHGV40051663	-0.52023	0.520227969	protein_coding	NM_004925
ASHGV40027743	-0.51998	0.519984841	protein_coding	uc002ukm.1
ASHGV40008182	0.519807	0.519806799	protein_coding	NM_005788
ASHGV40035998	0.519743	0.519742808	protein_coding	NM_007184
ASHGV40019618	-0.51974	0.519741534	protein_coding	NM_001024601
ASHGV40027455	-0.51973	0.519729308	protein_coding	NM_207363
ASHGV40038105	-0.51949	0.519490347	protein_coding	NM_031956
ASHGV40040069	0.519395	0.5193951	protein_coding	NM_013235
ASHGV40027854	-0.51935	0.519346376	protein_coding	NM_031459
ASHGV40042451	-0.51933	0.519325896	protein_coding	NM_018915
ASHGV40033548	0.519279	0.519279006	protein_coding	NM_021115
ASHGV40028632	-0.51926	0.519260351	protein_coding	NM_002246
ASHGV40033114	0.519233	0.519232507	protein_coding	NM_003753
ASHGV40025708	-0.51922	0.519221201	protein_coding	NM_001042544
ASHGV40051697	-0.51912	0.5191201	protein_coding	NM_025182
ASHGV40041002	0.51908	0.519079808	protein_coding	NM_001025159
ASHGV40056280	-0.51904	0.519040896	protein_coding	NM_016643
ASHGV40018907	0.518916	0.518916042	protein_coding	NM_194280
ASHGV40007954	-0.51888	0.518876739	protein_coding	NM_002339
ASHGV40035166	0.518797	0.518796915	protein_coding	NM_020800
ASHGV40003099	0.518796	0.518796147	protein_coding	NM_004233
ASHGV40060880	0.518734	0.518734442	protein_coding	uc010uaj.1
ASHGV40028276	-0.51872	0.518718612	protein_coding	NM_018645
ASHGV40054981	-0.51872	0.518718609	protein_coding	NM_203407
ASHGV40007376	-0.51864	0.518636835	protein_coding	NM_005512
ASHGV40049990	-0.51849	0.518494233	protein_coding	NM_020863
ASHGV40014741	-0.51845	0.518452116	protein_coding	NM_018071
ASHGV40056964	0.518386	0.51838584	protein_coding	NM_052969
ASHGV40017712	-0.51836	0.518358439	protein_coding	NM_017736
ASHGV40000453	-0.51819	0.518192914	protein_coding	ENST00000418535
ASHGV40027109	0.518187	0.518186519	protein_coding	NM_016466
ASHGV40033545	-0.51815	0.518149626	protein_coding	uc021opf.1
ASHGV40021362	-0.51812	0.518118818	protein_coding	NM_016113
ASHGV40049645	-0.51809	0.518090088	protein_coding	NM_005261
ASHGV40001308	0.517998	0.517998396	protein_coding	NM_138770
ASHGV40015810	-0.51794	0.517939641	protein_coding	NM_014080
ASHGV40025385	-0.51784	0.517835791	protein_coding	NM_015329
ASHGV40010739	-0.51781	0.517805968	protein_coding	NM_001414
ASHGV40024458	-0.51778	0.517781784	protein_coding	NM_002781

ASHGV40014829	-0.51773	0.517725338	protein_coding	NM_003790
ASHGV40033629	-0.51769	0.517694484	protein_coding	NM_001007467
ASHGV40016773	-0.51767	0.517673017	protein_coding	NM_205850
ASHGV40030978	0.517645	0.51764486	protein_coding	NM_006038
ASHGV40046516	-0.51743	0.517428517	protein_coding	NM_021958
ASHGV40054238	0.517426	0.517426351	protein_coding	NM_004085
ASHGV40018174	-0.51732	0.51732237	protein_coding	NM_000229
ASHGV40029309	-0.51723	0.517231747	protein_coding	NM_144978
ASHGV40035995	0.517224	0.517223598	protein_coding	NM_015512
ASHGV40048299	-0.51707	0.517072035	protein_coding	NM_002649
ASHGV40014082	-0.51698	0.516979136	protein_coding	NM_001202
ASHGV40056643	-0.51665	0.51664726	protein_coding	NM_178349
ASHGV40041574	0.516594	0.516593669	protein_coding	NM_178140
ASHGV40025706	-0.51659	0.516586129	protein_coding	NM_138392
ASHGV40050127	0.516155	0.516154905	protein_coding	ENST00000332135
ASHGV40044581	-0.51596	0.515959983	protein_coding	NM_001491
ASHGV40015854	0.515907	0.515906974	protein_coding	NM_032802
ASHGV40015313	0.515902	0.515901846	protein_coding	NM_024952
ASHGV40009134	-0.51588	0.515880755	protein_coding	ENST00000532612
ASHGV40013947	0.515863	0.515862847	protein_coding	NM_021249
ASHGV40009306	0.515767	0.515766713	protein_coding	NM_032811
ASHGV40024711	-0.51577	0.515766102	protein_coding	NM_002029
ASHGV40019107	0.515722	0.515722434	protein_coding	NM_001308319
ASHGV40025992	-0.51556	0.515564084	protein_coding	NM_144684
ASHGV40047028	0.515465	0.51546533	protein_coding	NM_000120
ASHGV40048834	-0.51542	0.515422571	protein_coding	NM_001147
ASHGV40039174	-0.51542	0.515415614	protein_coding	NM_001011515
ASHGV40054570	0.515365	0.515364553	protein_coding	NM_004135
ASHGV40018928	0.515297	0.515296547	protein_coding	NM_022731
ASHGV40055694	-0.51513	0.515134094	protein_coding	NM_001077697
ASHGV40033054	-0.51509	0.515086367	protein_coding	NM_014323
ASHGV40007144	-0.51489	0.514888686	protein_coding	NM_006795
ASHGV40035953	0.514855	0.514855213	protein_coding	NM_203370
ASHGV40024682	0.514781	0.514781182	protein_coding	NM_153812
ASHGV40006930	0.514778	0.514778164	protein_coding	NM_002804
ASHGV40054606	0.514706	0.514706067	protein_coding	NM_017987
ASHGV40017779	0.514612	0.514612233	protein_coding	NM_145080
ASHGV40023898	-0.51453	0.514531653	protein_coding	NM_006858
ASHGV40020266	-0.51447	0.514473159	protein_coding	NM_139280
ASHGV40025340	-0.51443	0.514434601	protein_coding	NM_014256
ASHGV40003130	0.514365	0.514364942	protein_coding	NM_001145268
ASHGV40046863	0.514353	0.514353398	protein_coding	NM_001289933
ASHGV40046405	0.514125	0.514124973	protein_coding	NM_138701
ASHGV40050770	0.514014	0.51401374	protein_coding	NM_001029954
ASHGV40015008	0.513918	0.513917771	protein_coding	NM_015589
ASHGV40003237	-0.51385	0.51384622	protein_coding	NM_001327
ASHGV40024110	0.513817	0.51381722	protein_coding	NM_172231

ASHGV40056603	-0.5138	0.513803028	protein_coding	NM_001011
ASHGV40048379	0.51371	0.513709728	protein_coding	NM_019071
ASHGV40048254	0.51319	0.513189662	protein_coding	NM_001283
ASHGV40026891	0.513042	0.513042251	protein_coding	NM_001002755
ASHGV40055988	-0.51292	0.512922654	protein_coding	NM_004316
ASHGV40014753	-0.51285	0.512847434	protein_coding	uc001wcc.3
ASHGV40000685	0.512707	0.51270745	protein_coding	NM_018447
ASHGV40048148	-0.5126	0.512596006	protein_coding	NM_000089
ASHGV40031806	-0.51259	0.512590183	protein_coding	NM_001324
ASHGV40008502	-0.51257	0.512565269	protein_coding	NM_018841
ASHGV40031527	0.512562	0.512562002	protein_coding	NM_001257137
ASHGV40006469	-0.51251	0.512508226	protein_coding	NM_020883
ASHGV40021008	0.512445	0.512445065	protein_coding	NM_001185077
ASHGV40026521	0.51238	0.512380259	protein_coding	NM_022823
ASHGV40055896	-0.51216	0.512160978	protein_coding	NM_015221
ASHGV40030400	0.512028	0.512027661	protein_coding	NM_004404
ASHGV40025839	-0.512	0.511998705	protein_coding	NM_002517
ASHGV40038341	-0.51172	0.511722295	protein_coding	NM_152682
ASHGV40029343	-0.51169	0.511685559	protein_coding	NM_032309
ASHGV40030356	-0.51167	0.511672404	protein_coding	NM_004073
ASHGV40012099	-0.51166	0.51165708	protein_coding	NM_001080533
ASHGV40017423	0.511651	0.511650792	protein_coding	NM_153350
ASHGV40013283	0.511628	0.511628104	protein_coding	uc001uzr.1
ASHGV40018434	-0.51161	0.511609167	protein_coding	NM_001080487
ASHGV40005345	0.511598	0.511597872	protein_coding	NM_016299
ASHGV40052393	0.511535	0.511534802	protein_coding	NM_017873
ASHGV40028706	-0.5115	0.511503554	protein_coding	NM_016252
ASHGV40032609	0.51144	0.511439773	protein_coding	NM_001291412
ASHGV40026106	-0.51141	0.511406192	protein_coding	NM_013358
ASHGV40034323	-0.51129	0.511289294	protein_coding	NM_001123040
ASHGV40050832	-0.51125	0.511248158	protein_coding	NM_031461
ASHGV40002020	-0.51124	0.51123879	protein_coding	NM_198181
ASHGV40021590	-0.51124	0.511235747	protein_coding	NM_005623
ASHGV40037183	-0.51119	0.511185464	protein_coding	NM_018659
ASHGV40011561	-0.51106	0.511064994	protein_coding	NM_133489
ASHGV40026440	0.510943	0.51094346	protein_coding	NM_022460
ASHGV40049906	0.51087	0.510870178	protein_coding	NM_006762
ASHGV40001264	-0.51082	0.510822037	protein_coding	NM_004377
ASHGV40050153	-0.5108	0.510800163	protein_coding	NM_021061
ASHGV40041613	-0.51073	0.510731084	protein_coding	NM_005983
ASHGV40033788	0.510683	0.510682726	protein_coding	uc003bba.1
ASHGV40057763	-0.51063	0.510633028	protein_coding	NM_014096
ASHGV40006515	-0.51052	0.510520636	protein_coding	NM_001142946
ASHGV40042458	-0.51037	0.510368504	protein_coding	NM_018919
ASHGV40021820	0.510316	0.510315797	protein_coding	NM_021079
ASHGV40026013	-0.51031	0.510307176	protein_coding	ENST00000355326
ASHGV40039270	-0.51014	0.510135978	protein_coding	NM_001977

ASHGV40003265	-0.51009	0.51009335	protein_coding	NM_005088
ASHGV40009053	0.509901	0.509901125	protein_coding	NM_032021
ASHGV40055417	-0.50987	0.509865773	protein_coding	NM_001017417
ASHGV40016736	0.509863	0.509863378	protein_coding	NM_138423
ASHGV40018605	-0.50981	0.509805185	protein_coding	NM_152341
ASHGV40007064	-0.50978	0.509782668	protein_coding	NM_013401
ASHGV40018942	-0.50974	0.509744915	protein_coding	NM_152288
ASHGV40056083	-0.50974	0.509741556	protein_coding	NM_005615
ASHGV40024370	-0.50949	0.509486806	protein_coding	NM_001080468
ASHGV40049437	-0.50941	0.509409879	protein_coding	NM_024504
ASHGV40044235	0.509204	0.509203947	protein_coding	NM_004690
ASHGV40007389	0.509155	0.509154887	protein_coding	NM_016578
ASHGV40012926	0.50905	0.509049523	protein_coding	NM_004093
ASHGV40046116	0.509037	0.509036762	protein_coding	NM_001080495
ASHGV40053709	0.508966	0.50896586	protein_coding	NM_001636
ASHGV40045413	0.508891	0.508890639	protein_coding	NM_020482
ASHGV40033355	-0.50872	0.508720803	protein_coding	NM_001014440
ASHGV40048916	-0.50858	0.508576527	protein_coding	NM_001256869
ASHGV40015805	0.50847	0.508470088	protein_coding	NM_025137
ASHGV40026028	-0.50843	0.508425223	protein_coding	NM_138373
ASHGV40031612	-0.5084	0.508398064	protein_coding	NM_003286
ASHGV40030396	-0.50831	0.508307972	protein_coding	NM_001080437
ASHGV40021048	-0.50825	0.508252076	protein_coding	NM_001009905
ASHGV40017210	-0.50815	0.50814963	protein_coding	NM_020778
ASHGV40000127	0.508101	0.508100662	protein_coding	NM_012259
ASHGV40013734	-0.50809	0.50808814	protein_coding	NM_000504
ASHGV40037897	0.508024	0.508023826	protein_coding	NM_001221
ASHGV40020399	-0.50794	0.507941897	protein_coding	NM_002722
ASHGV40044981	-0.50792	0.507917832	protein_coding	NM_001257357
ASHGV40014313	0.507875	0.507875013	protein_coding	NM_001040108
ASHGV40033744	-0.50774	0.507744381	protein_coding	NM_021822
ASHGV40033590	-0.50771	0.507710394	protein_coding	NM_021026
ASHGV40051676	-0.50763	0.507632608	protein_coding	NM_020702
ASHGV40028601	-0.50753	0.507531302	protein_coding	NM_181713
ASHGV40023705	0.507501	0.507501192	protein_coding	NM_012458
ASHGV40000678	0.507373	0.507372771	protein_coding	ENST00000429238
ASHGV40046689	-0.50711	0.507111654	protein_coding	NM_080744
ASHGV40021721	-0.50709	0.507088357	protein_coding	NM_001277331
ASHGV40029861	0.506928	0.506927756	protein_coding	NM_018981
ASHGV40016739	0.506902	0.506901539	protein_coding	NM_003758
ASHGV40034174	0.506849	0.506848509	protein_coding	NM_006309
ASHGV40006103	-0.50682	0.506822989	protein_coding	ENST00000598040
ASHGV40029120	0.506558	0.506558282	protein_coding	NM_016037
ASHGV40055856	0.506458	0.506457597	protein_coding	NM_013314
ASHGV40022435	0.506451	0.506450853	protein_coding	NM_001145194
ASHGV40043396	0.506418	0.506417671	protein_coding	NM_004117
ASHGV40048845	-0.50619	0.506185829	protein_coding	NM_021010

ASHGV40012903	-0.50605	0.506046895	protein_coding	NM_024089
ASHGV40022772	0.506032	0.506031963	protein_coding	NM_017653
ASHGV40008599	0.50586	0.505859779	protein_coding	NM_032251
ASHGV40007077	0.505845	0.50584518	protein_coding	NM_022830
ASHGV40010390	-0.50576	0.505756553	protein_coding	NM_194292
ASHGV40003267	-0.5057	0.505697036	protein_coding	NM_005138
ASHGV40010602	-0.50545	0.505449912	protein_coding	NM_001109662
ASHGV40005319	-0.50544	0.50544202	protein_coding	NM_153256
ASHGV40019931	-0.50538	0.505381874	protein_coding	NM_181716
ASHGV40015011	0.505371	0.505370502	protein_coding	NM_080867
ASHGV40003293	-0.50524	0.505237867	protein_coding	NM_019845
ASHGV40048001	-0.50516	0.505162415	protein_coding	NM_000501
ASHGV40018219	-0.50505	0.505053529	protein_coding	NM_000353
ASHGV40036480	-0.50504	0.505041128	protein_coding	NM_152889
ASHGV40015931	0.504878	0.504878497	protein_coding	NM_024755
ASHGV40054345	-0.50466	0.504663217	protein_coding	NM_033495
ASHGV40014007	-0.50464	0.504636911	protein_coding	NM_015100
ASHGV40001405	0.504621	0.50462097	protein_coding	NM_003278
ASHGV40046845	-0.5046	0.504597038	protein_coding	NM_181538
ASHGV40033582	-0.50449	0.504491661	protein_coding	NM_001206998
ASHGV40034473	0.504477	0.504476621	protein_coding	NM_003500
ASHGV40005186	0.504411	0.50441077	protein_coding	NM_012341
ASHGV40034348	0.504352	0.504351693	protein_coding	NM_001664
ASHGV40042472	0.50429	0.504289857	protein_coding	NM_018929
ASHGV40008594	-0.50413	0.504132092	protein_coding	NM_020155
ASHGV40057568	-0.50392	0.503923954	protein_coding	NM_030914
ASHGV40024564	-0.50392	0.50391794	protein_coding	NM_145056
ASHGV40005264	0.503873	0.503873129	protein_coding	uc001iik.3
ASHGV40038705	-0.5038	0.503799493	protein_coding	NM_024936
ASHGV40019266	-0.50377	0.5037708	protein_coding	NM_018378
ASHGV40029427	-0.50366	0.503657838	protein_coding	NM_005270
ASHGV40047256	-0.5036	0.50360358	protein_coding	ENST00000439431
ASHGV40015210	0.503261	0.503261477	protein_coding	NM_005252
ASHGV40014376	0.503233	0.503233259	protein_coding	NM_005065
ASHGV40019930	0.503203	0.503202945	protein_coding	NM_006311
ASHGV40010424	-0.50311	0.503113763	protein_coding	NM_032147
ASHGV40031592	-0.50307	0.503067396	protein_coding	NM_024855
ASHGV40038645	-0.50296	0.502957565	protein_coding	uc003gox.1
ASHGV40021702	-0.50288	0.502876657	protein_coding	NM_001254
ASHGV40020555	-0.50267	0.502667851	protein_coding	NM_001267
ASHGV40003370	-0.50259	0.502591569	protein_coding	NM_214461
ASHGV40007905	-0.50259	0.502585718	protein_coding	NM_003475
ASHGV40046070	0.502507	0.50250709	protein_coding	NM_012424
ASHGV40031553	-0.50249	0.502487288	protein_coding	NM_152607
ASHGV40056013	-0.5018	0.501801678	protein_coding	uc001uiv.1
ASHGV40044905	0.501702	0.501702139	protein_coding	NM_020056
ASHGV40006447	-0.50169	0.501688646	protein_coding	NM_000773

ASHGV40042413	0.501653	0.501653218	protein_coding	NM_144723
ASHGV40011652	-0.50155	0.501547485	protein_coding	NM_020401
ASHGV40013379	-0.50144	0.501442648	protein_coding	NM_198441
ASHGV40057742	-0.50141	0.501412113	protein_coding	NM_001005288
ASHGV40020770	0.501211	0.501210569	protein_coding	NM_080282
ASHGV40030906	-0.50115	0.501153137	protein_coding	NM_080749
ASHGV40020415	0.501113	0.501112982	protein_coding	NM_001002909
ASHGV40008178	-0.5009	0.500903725	protein_coding	NM_001244963
ASHGV40016179	0.500741	0.500740838	protein_coding	NM_032808
ASHGV40026144	-0.50065	0.500647123	protein_coding	NM_020880
ASHGV40054716	-0.50064	0.500644445	protein_coding	NM_002063
ASHGV40052415	0.500579	0.500578916	protein_coding	NM_031432
ASHGV40033589	0.500556	0.500555708	protein_coding	NM_001278730
ASHGV40054436	0.500417	0.500416634	protein_coding	NM_147175
ASHGV40019699	-0.5004	0.500399202	protein_coding	NM_018727
ASHGV40021744	-0.50038	0.500380148	protein_coding	NM_000263
ASHGV40010910	0.500322	0.50032184	protein_coding	NM_002014
ASHGV40008112	-0.50029	0.500286597	protein_coding	NM_033407
ASHGV40014431	0.500277	0.500276869	protein_coding	NM_183387
ASHGV40036697	0.500166	0.500165584	protein_coding	NM_002886
ASHGV40050707	0.499973	0.49997261	protein_coding	NM_005625
ASHGV40014066	-0.49996	0.499963964	protein_coding	ENST00000597846
ASHGV40056811	-0.49981	0.499807566	protein_coding	NM_181623
ASHGV40034532	0.49974	0.499740093	protein_coding	NM_007114
ASHGV40017280	0.499643	0.499642509	protein_coding	NM_014848
ASHGV40051053	-0.49955	0.499552443	protein_coding	ENST00000521923
ASHGV40034330	-0.49952	0.499516104	protein_coding	NM_001009996
ASHGV40018452	-0.49942	0.499422695	protein_coding	NM_152339
ASHGV40011988	-0.49938	0.499383225	protein_coding	NM_001082537
ASHGV40018007	0.499365	0.499365279	protein_coding	NM_015272
ASHGV40025594	-0.49933	0.499334403	protein_coding	NM_019104
ASHGV40050619	0.499301	0.499300777	protein_coding	NM_012247
ASHGV40037581	0.499119	0.499118726	protein_coding	NM_001812
ASHGV40048953	0.499066	0.499066396	protein_coding	NM_016002
ASHGV40033247	0.499032	0.49903226	protein_coding	NM_006417
ASHGV40050766	0.498946	0.498946032	protein_coding	NM_152765
ASHGV40034281	-0.49873	0.498730364	protein_coding	NM_182702
ASHGV40045780	0.498722	0.498721881	protein_coding	NM_032860
ASHGV40050906	-0.49864	0.498635594	protein_coding	NM_003909
ASHGV40029991	0.498565	0.498565102	protein_coding	NM_015934
ASHGV40030397	0.498553	0.498553381	protein_coding	NM_002712
ASHGV40040307	-0.49847	0.498471891	protein_coding	NM_173829
ASHGV40039140	0.498456	0.498455952	protein_coding	NM_020203
ASHGV40006122	-0.49827	0.498268964	protein_coding	NM_015062
ASHGV40050150	-0.49795	0.497952415	protein_coding	NM_000973
ASHGV40020541	0.497934	0.497934242	protein_coding	NM_032595
ASHGV40020768	-0.49781	0.497812794	protein_coding	NM_080284

ASHGV40018596	-0.49775	0.497746622	protein_coding	NM_001135086
ASHGV40048307	-0.49771	0.497711209	protein_coding	NM_024814
ASHGV40033783	-0.49771	0.497707121	protein_coding	NM_001142964
ASHGV40045931	0.497651	0.497651118	protein_coding	NM_001291958
ASHGV40023916	-0.49764	0.497640607	protein_coding	NM_032377
ASHGV40056086	-0.49756	0.497557948	protein_coding	NM_001004731
ASHGV40000236	-0.49756	0.497555089	protein_coding	ENST00000401034
ASHGV40032605	0.497187	0.497187401	protein_coding	NM_000629
ASHGV40039246	-0.49711	0.497110347	protein_coding	NM_152621
ASHGV40021404	-0.49687	0.496865437	protein_coding	NM_016078
ASHGV40038382	-0.4968	0.49679801	protein_coding	ENST00000596414
ASHGV40006615	0.496507	0.496506861	protein_coding	NM_013249
ASHGV40014427	-0.49633	0.496332208	protein_coding	uc001xwp.1
ASHGV40048328	-0.49631	0.496314342	protein_coding	NM_182597
ASHGV40026343	0.496273	0.496272911	protein_coding	NM_024894
ASHGV40016139	-0.49617	0.496173882	protein_coding	NM_005697
ASHGV40019024	0.496161	0.496161477	protein_coding	NM_006893
ASHGV40052661	0.49603	0.496029501	protein_coding	NM_144569
ASHGV40018816	-0.49601	0.496011102	protein_coding	NM_001039
ASHGV40019176	0.495974	0.495973583	protein_coding	NM_012106
ASHGV40020511	-0.49597	0.49596946	protein_coding	NM_006361
ASHGV40035944	0.495921	0.495921259	protein_coding	NM_022171
ASHGV40028192	-0.49591	0.495914342	protein_coding	NM_178865
ASHGV40030275	-0.49566	0.495663913	protein_coding	NM_001017915
ASHGV40031763	-0.49562	0.495624403	protein_coding	NM_032521
ASHGV40046673	0.49559	0.495589786	protein_coding	NM_144695
ASHGV40051332	0.495448	0.495447516	protein_coding	uc010met.1
ASHGV40050849	0.495345	0.495344509	protein_coding	NM_016010
ASHGV40020323	-0.49534	0.495336722	protein_coding	NM_002280
ASHGV40046821	0.495279	0.495279356	protein_coding	NM_015176
ASHGV40037827	0.495239	0.495239462	protein_coding	NM_020139
ASHGV40022669	-0.49522	0.495216024	protein_coding	NM_001112734
ASHGV40029806	-0.4952	0.495199153	protein_coding	NM_006554
ASHGV40044722	-0.49511	0.495107688	protein_coding	NM_005495
ASHGV40057600	-0.49495	0.494952333	protein_coding	NM_001271682
ASHGV40042511	0.494708	0.494707968	protein_coding	NM_018989
ASHGV40013168	0.494663	0.494662601	protein_coding	NM_194318
ASHGV40011285	-0.49466	0.494661664	protein_coding	NM_001025356
ASHGV40040531	-0.49442	0.494416523	protein_coding	NM_002348
ASHGV40051329	-0.49437	0.494373881	protein_coding	ENST00000518520
ASHGV40011883	-0.49428	0.494284426	protein_coding	NM_022496
ASHGV40053136	0.494229	0.494229355	protein_coding	NM_001282394
ASHGV40051685	-0.49416	0.494162317	protein_coding	NM_147157
ASHGV40026914	-0.49391	0.493909138	protein_coding	NM_144582
ASHGV40048220	-0.49385	0.49384643	protein_coding	NM_001004351
ASHGV40025606	-0.49376	0.493757585	protein_coding	NM_173636
ASHGV40008838	0.493756	0.49375575	protein_coding	NM_015534

ASHGV40012590	0.493721	0.493721312	protein_coding	NM_012141
ASHGV40044240	0.493701	0.493700976	protein_coding	NM_032832
ASHGV40028333	0.493658	0.493658199	protein_coding	NM_005336
ASHGV40038449	-0.49349	0.493487426	protein_coding	NM_000203
ASHGV40023677	-0.49341	0.493405181	protein_coding	NM_152482
ASHGV40015688	0.493402	0.49340176	protein_coding	NM_014691
ASHGV40041737	0.49339	0.493389793	protein_coding	NM_002495
ASHGV40014665	0.493353	0.493353332	protein_coding	NM_170782
ASHGV40032831	0.49319	0.493189804	protein_coding	NM_001195226
ASHGV40047998	0.492832	0.492832076	protein_coding	NM_005626
ASHGV40023301	-0.49283	0.492831838	protein_coding	NM_031446
ASHGV40039636	-0.49282	0.492819639	protein_coding	NM_199173
ASHGV40028036	-0.49281	0.492807	protein_coding	NM_018441
ASHGV40030579	0.492661	0.492660591	protein_coding	NM_001195
ASHGV40027106	0.49262	0.49261998	protein_coding	NM_001115016
ASHGV40029820	0.492617	0.492617462	protein_coding	NM_003659
ASHGV40016907	0.492559	0.492559298	protein_coding	NM_022457
ASHGV40038448	0.492537	0.492537482	protein_coding	NM_016178
ASHGV40018774	0.492273	0.492272923	protein_coding	NM_020314
ASHGV40022417	0.491855	0.491854828	protein_coding	NM_022840
ASHGV40055936	-0.49185	0.491853387	protein_coding	NM_001113407
ASHGV40010312	-0.49183	0.491834791	protein_coding	NM_002583
ASHGV40021687	-0.49177	0.491768915	protein_coding	NM_199321
ASHGV40028663	0.491602	0.491601528	protein_coding	NM_004891
ASHGV40051418	-0.49158	0.491577891	protein_coding	NM_032902
ASHGV40000133	0.491331	0.491331298	protein_coding	NM_203458
ASHGV40055046	0.49072	0.490719605	protein_coding	NM_173834
ASHGV40009990	-0.49071	0.490708216	protein_coding	NM_175068
ASHGV40042524	0.490691	0.490690674	protein_coding	NM_014810
ASHGV40000205	-0.49067	0.490670334	protein_coding	ENST00000393007
ASHGV40022549	0.490656	0.490655618	protein_coding	NM_152352
ASHGV40041085	0.490655	0.490654679	protein_coding	NM_001130864
ASHGV40026491	-0.49062	0.49062289	protein_coding	NM_194248
ASHGV40009579	-0.49055	0.490545882	protein_coding	NM_197947
ASHGV40039571	0.49042	0.490419614	protein_coding	NM_004453
ASHGV40014473	-0.49015	0.490146707	protein_coding	NM_001002860
ASHGV40029411	0.490128	0.490127563	protein_coding	NM_001271049
ASHGV40030185	-0.49006	0.490058093	protein_coding	NM_058165
ASHGV40023934	0.489956	0.48995615	protein_coding	NM_001080821
ASHGV40018556	-0.48992	0.48992347	protein_coding	NM_004209
ASHGV40020381	-0.48991	0.48990638	protein_coding	NM_006373
ASHGV40021649	0.489891	0.489890627	protein_coding	NM_007026
ASHGV40053674	-0.48989	0.489886182	protein_coding	NM_014434
ASHGV40019256	-0.48971	0.48970744	protein_coding	NM_005182
ASHGV40009362	-0.48969	0.489692838	protein_coding	NM_002017
ASHGV40000069	-0.4896	0.489602838	protein_coding	ENST00000333156
ASHGV40054944	-0.48949	0.48949226	protein_coding	NM_002536

ASHGV40033721	0.489475	0.489475483	protein_coding	NM_012323
ASHGV40027602	-0.48946	0.489459437	protein_coding	NM_004288
ASHGV40060824	-0.4894	0.489403295	protein_coding	uc001yrt.3
ASHGV40057302	-0.48932	0.489319652	protein_coding	NM_014817
ASHGV40053270	-0.48919	0.489191223	protein_coding	ENST00000451160
ASHGV40015366	-0.48916	0.48916291	protein_coding	NM_000624
ASHGV40011682	0.489146	0.489146358	protein_coding	NM_003667
ASHGV40042874	0.489012	0.489012218	protein_coding	NM_033549
ASHGV40041371	-0.48892	0.488923921	protein_coding	NM_003953
ASHGV40012551	0.488874	0.488874478	protein_coding	NM_001984
ASHGV40039893	0.488794	0.488793696	protein_coding	NM_001080471
ASHGV40043377	0.488753	0.488752779	protein_coding	NM_178508
ASHGV40022397	0.488586	0.488586038	protein_coding	NM_130386
ASHGV40035152	-0.48845	0.488450371	protein_coding	NM_006884
ASHGV40017068	-0.48828	0.488281935	protein_coding	NM_015492
ASHGV40023166	0.488259	0.488259246	protein_coding	NM_032142
ASHGV40014141	0.488256	0.488256211	protein_coding	NM_182578
ASHGV40051696	0.488039	0.488039005	protein_coding	NM_013442
ASHGV40046319	-0.48755	0.487547423	protein_coding	NM_019029
ASHGV40031648	-0.48752	0.487517641	protein_coding	NM_024034
ASHGV40031134	-0.48751	0.487511229	protein_coding	NM_031215
ASHGV40048742	-0.4875	0.487498406	protein_coding	NM_001291913
ASHGV40040419	-0.48734	0.487335698	protein_coding	NM_032109
ASHGV40048888	-0.48732	0.487316379	protein_coding	NM_017884
ASHGV40025695	0.487229	0.487229171	protein_coding	NM_006503
ASHGV40047680	0.487177	0.487176819	protein_coding	NM_001118
ASHGV40011934	0.487032	0.487031718	protein_coding	NM_152772
ASHGV40024948	0.486985	0.486985354	protein_coding	NM_019112
ASHGV40044132	-0.48684	0.486844332	protein_coding	NM_001164586
ASHGV40053248	0.486761	0.486760535	protein_coding	ENST00000374762
ASHGV40025911	-0.48676	0.486758044	protein_coding	NM_020650
ASHGV40028408	-0.48675	0.486747638	protein_coding	NM_018436
ASHGV40014031	0.48669	0.486690384	protein_coding	NM_018139
ASHGV40031900	0.486449	0.486448903	protein_coding	NM_018291
ASHGV40036703	-0.48644	0.486440549	protein_coding	NM_001101337
ASHGV40036508	-0.48621	0.486211028	protein_coding	NM_024768
ASHGV40043515	0.486038	0.486037703	protein_coding	NM_023932
ASHGV40023299	0.486026	0.486026222	protein_coding	NM_020474
ASHGV40007222	0.48587	0.48586974	protein_coding	NM_002708
ASHGV40040994	-0.48581	0.485810442	protein_coding	NM_030953
ASHGV40034431	0.485548	0.485547678	protein_coding	NM_022899
ASHGV40049717	0.485459	0.485459225	protein_coding	NM_015713
ASHGV40007169	-0.48538	0.485376409	protein_coding	NM_033347
ASHGV40048915	-0.48527	0.485266834	protein_coding	NM_001037804
ASHGV40025271	-0.48526	0.485264047	protein_coding	NM_173482
ASHGV40037572	0.485252	0.485251975	protein_coding	NM_004439
ASHGV40029862	-0.48518	0.485182346	protein_coding	NM_080876

ASHGV40034708	-0.48508	0.485077265	protein_coding	NM_001397
ASHGV40051193	-0.48486	0.484860827	protein_coding	NM_003129
ASHGV40016793	-0.4848	0.484796122	protein_coding	NM_000488
ASHGV40038563	-0.48479	0.484793606	protein_coding	NM_053044
ASHGV40025351	-0.48473	0.484728967	protein_coding	NM_021933
ASHGV40043277	-0.48468	0.484678533	protein_coding	NM_001288
ASHGV40042405	0.484672	0.48467161	protein_coding	NM_020690
ASHGV40005676	0.484435	0.484435137	protein_coding	NM_001242659
ASHGV40017605	-0.48441	0.48440747	protein_coding	NM_001424
ASHGV40008679	-0.48439	0.484392044	protein_coding	NM_182553
ASHGV40049240	0.484219	0.484218662	protein_coding	NM_018105
ASHGV40056568	-0.48407	0.484070088	protein_coding	NM_012423
ASHGV40019694	0.484049	0.484049272	protein_coding	NM_005401
ASHGV40010466	-0.48405	0.484047683	protein_coding	NM_152317
ASHGV40020842	-0.48404	0.484037572	protein_coding	NM_178128
ASHGV40046546	0.483844	0.483843903	protein_coding	NM_005248
ASHGV40031187	-0.48381	0.483808	protein_coding	NM_032864
ASHGV40011270	0.483772	0.483771761	protein_coding	NM_016488
ASHGV40006727	-0.4837	0.483704536	protein_coding	NM_173588
ASHGV40051566	0.483597	0.483596587	protein_coding	NM_020200
ASHGV40030500	0.483585	0.48358514	protein_coding	NM_019593
ASHGV40027353	-0.48343	0.483428898	protein_coding	NM_015282
ASHGV40044756	-0.48324	0.483242176	protein_coding	NM_006353
ASHGV40014188	-0.48322	0.483216042	protein_coding	NM_000347
ASHGV40050708	0.483054	0.483054418	protein_coding	uc003xtv.1
ASHGV40022190	-0.48298	0.482984326	protein_coding	NM_018653
ASHGV40044287	0.482737	0.482737293	protein_coding	NM_016020
ASHGV40017084	0.48271	0.482710319	protein_coding	NM_173469
ASHGV40014770	-0.48269	0.482687864	protein_coding	NM_178336
ASHGV40008090	0.482649	0.482648976	protein_coding	NM_015055
ASHGV40054141	0.482632	0.482632317	protein_coding	NM_001012977
ASHGV40056898	0.482324	0.48232368	protein_coding	NM_015444
ASHGV40019630	-0.48223	0.482229464	protein_coding	NM_006086
ASHGV40038782	-0.48219	0.482194921	protein_coding	NM_018290
ASHGV40019967	0.482173	0.482172854	protein_coding	NM_002018
ASHGV40006935	-0.4821	0.482102699	protein_coding	NM_016506
ASHGV40020400	-0.48193	0.48193036	protein_coding	NM_004160
ASHGV40012284	-0.48187	0.481867612	protein_coding	NM_003440
ASHGV40034119	0.481814	0.481813704	protein_coding	NM_022461
ASHGV40011030	-0.48177	0.481769817	protein_coding	NM_005810
ASHGV40053001	-0.48174	0.481740338	protein_coding	NM_032171
ASHGV40000057	-0.48162	0.481621123	protein_coding	ENST00000323223
ASHGV40034519	0.481546	0.481546493	protein_coding	NM_015541
ASHGV40016433	0.481374	0.481374248	protein_coding	NM_001300969
ASHGV40048977	-0.48127	0.481266104	protein_coding	NM_021020
ASHGV40019708	0.481187	0.481187379	protein_coding	NM_032294
ASHGV40026892	-0.48118	0.481180609	protein_coding	NM_014911

ASHGV40049900	-0.48118	0.481177902	protein_coding	NM_014751
ASHGV40019732	-0.48108	0.481078678	protein_coding	NM_182566
ASHGV40029114	0.480917	0.480916777	protein_coding	NM_016494
ASHGV40022037	0.4809	0.480899563	protein_coding	NM_001005404
ASHGV40030247	-0.48088	0.480877343	protein_coding	NM_001271466
ASHGV40053387	-0.48073	0.480727652	protein_coding	NM_007018
ASHGV40024963	-0.48068	0.48068218	protein_coding	NM_018959
ASHGV40040607	-0.48047	0.48046518	protein_coding	NM_180991
ASHGV40002593	-0.48035	0.480350716	protein_coding	ENST00000594256
ASHGV40025761	0.480291	0.480291212	protein_coding	NM_006511
ASHGV40017654	0.480278	0.480278045	protein_coding	NM_002582
ASHGV40012223	0.480237	0.480236801	protein_coding	NM_144669
ASHGV40003356	0.480187	0.480187087	protein_coding	NM_182470
ASHGV40016678	-0.48017	0.480171634	protein_coding	NM_152260
ASHGV40055291	-0.48013	0.480125599	protein_coding	NM_001099922
ASHGV40011365	-0.48006	0.480060615	protein_coding	NM_001651
ASHGV40025535	-0.48005	0.480045815	protein_coding	NM_022467
ASHGV40033347	-0.48002	0.480015044	protein_coding	NM_002751
ASHGV40027767	-0.47982	0.479818791	protein_coding	NM_001256850
ASHGV40014104	0.479662	0.479662001	protein_coding	NM_014924
ASHGV40017356	-0.47966	0.479655569	protein_coding	NM_000875
ASHGV40025269	-0.47936	0.479362544	protein_coding	NM_012377
ASHGV40035296	0.479206	0.479205746	protein_coding	NM_020409
ASHGV40043379	0.479026	0.479026362	protein_coding	NM_006703
ASHGV40007154	0.479015	0.479015248	protein_coding	NM_014205
ASHGV40055184	-0.47894	0.478935563	protein_coding	NM_013347
ASHGV40040443	0.478623	0.478622831	protein_coding	NM_001010891
ASHGV40027909	-0.4786	0.478597557	protein_coding	NM_001039693
ASHGV40012835	-0.47847	0.478474135	protein_coding	NM_005845
ASHGV40053814	0.478365	0.478364589	protein_coding	NM_147156
ASHGV40051153	-0.47832	0.478315635	protein_coding	NM_182543
ASHGV40027941	0.478314	0.478313553	protein_coding	NM_138468
ASHGV40037933	-0.47825	0.478250417	protein_coding	NM_018699
ASHGV40001585	-0.47822	0.478217133	protein_coding	ENST00000510604
ASHGV40020553	-0.47819	0.478186798	protein_coding	NM_018509
ASHGV40023439	-0.47811	0.47810833	protein_coding	NM_018181
ASHGV40009907	-0.47802	0.47801905	protein_coding	NM_017822
ASHGV40035570	-0.47795	0.47795079	protein_coding	NM_000844
ASHGV40032032	-0.47792	0.477921425	protein_coding	NM_006806
ASHGV40056098	0.477919	0.477918721	protein_coding	NM_001012
ASHGV40043301	-0.47788	0.47787878	protein_coding	NM_022110
ASHGV40018345	0.477867	0.477867261	protein_coding	NM_020947
ASHGV40034051	-0.47774	0.477742281	protein_coding	NM_012260
ASHGV40016935	0.477712	0.477712067	protein_coding	NM_015042
ASHGV40021869	0.477689	0.477689397	protein_coding	NM_176096
ASHGV40000101	-0.47763	0.477629466	protein_coding	NM_001080770
ASHGV40041252	-0.47748	0.477476203	protein_coding	NM_153373

ASHGV40028476	-0.47742	0.477417173	protein_coding	NM_005680
ASHGV40009784	-0.47738	0.477378863	protein_coding	NM_020063
ASHGV40002103	0.477312	0.477311579	protein_coding	NM_033426
ASHGV40026111	0.477098	0.477098166	protein_coding	ENST00000586573
ASHGV40009094	-0.47703	0.477030312	protein_coding	ENST00000600612
ASHGV40003212	-0.47701	0.477013328	protein_coding	NM_014895
ASHGV40014806	-0.47698	0.476978061	protein_coding	NM_001291556
ASHGV40052955	0.47673	0.476729744	protein_coding	NM_004293
ASHGV40010681	0.476605	0.476605485	protein_coding	NM_006836
ASHGV40055593	-0.47639	0.476393734	protein_coding	NM_003140
ASHGV40008034	0.476362	0.476362252	protein_coding	NM_176875
ASHGV40014677	0.476349	0.47634852	protein_coding	NM_153741
ASHGV40015893	0.476246	0.476245873	protein_coding	NM_016304
ASHGV40036362	-0.4761	0.476104254	protein_coding	NM_001042575
ASHGV40042446	-0.47596	0.475962086	protein_coding	NM_018933
ASHGV40024699	-0.47594	0.475938606	protein_coding	NM_001193623
ASHGV40026643	0.475852	0.475851553	protein_coding	NM_003618
ASHGV40008051	-0.47581	0.475809023	protein_coding	NM_001004684
ASHGV40024538	-0.47581	0.475807078	protein_coding	NM_030785
ASHGV40017380	-0.47577	0.475765857	protein_coding	NM_000693
ASHGV40047304	-0.47575	0.475749531	protein_coding	NM_000238
ASHGV40007106	-0.4755	0.475496761	protein_coding	NM_004254
ASHGV40008691	0.475442	0.475441779	protein_coding	NM_001104
ASHGV40037474	-0.47532	0.475318004	protein_coding	NM_006587
ASHGV40039572	0.475185	0.475184918	protein_coding	NM_020840
ASHGV40013925	0.475152	0.475152487	protein_coding	NM_015473
ASHGV40009947	0.475152	0.475152465	protein_coding	NM_016040
ASHGV40033170	0.47511	0.47510956	protein_coding	NM_014292
ASHGV40003263	0.474921	0.474921123	protein_coding	NM_004711
ASHGV40023123	0.474885	0.474885399	protein_coding	NM_006788
ASHGV40025027	-0.47485	0.474846151	protein_coding	NM_015897
ASHGV40012071	-0.47473	0.474731331	protein_coding	NM_007370
ASHGV40046487	0.474638	0.474637584	protein_coding	NM_017898
ASHGV40022057	-0.47461	0.474612049	protein_coding	NM_203425
ASHGV40033885	0.474558	0.474558183	protein_coding	NM_001082967
ASHGV40049463	0.474481	0.474481074	protein_coding	NM_005648
ASHGV40033053	0.474046	0.474045553	protein_coding	NM_052880
ASHGV40005028	0.474016	0.474015688	protein_coding	NM_024942
ASHGV40015775	0.473883	0.473883132	protein_coding	NM_001284307
ASHGV40057359	-0.47369	0.473689078	protein_coding	NM_203397
ASHGV40019466	0.473687	0.473686814	protein_coding	NM_015251
ASHGV40001106	0.473676	0.473676058	protein_coding	ENST00000451472
ASHGV40041236	-0.47355	0.473546398	protein_coding	NM_001190946
ASHGV40018693	-0.47349	0.473487426	protein_coding	NM_002484
ASHGV40041814	0.473194	0.47319408	protein_coding	NM_015172
ASHGV40008902	0.472945	0.472945119	protein_coding	NM_003902
ASHGV40050482	-0.47284	0.472843479	protein_coding	NM_013964

ASHGV40050112	-0.47281	0.47281142	protein_coding	NM_178564
ASHGV40015197	0.472511	0.47251079	protein_coding	NM_001933
ASHGV40011488	-0.47243	0.472432787	protein_coding	NM_001005243
ASHGV40011320	-0.47234	0.472343269	protein_coding	NM_024056
ASHGV40053846	0.472183	0.472183335	protein_coding	NM_030624
ASHGV40025473	-0.4721	0.472096826	protein_coding	NM_024310
ASHGV40041604	-0.47207	0.47206954	protein_coding	NM_144725
ASHGV40052572	0.471916	0.471915948	protein_coding	NM_015158
ASHGV40050228	-0.47173	0.47172972	protein_coding	NM_001193630
ASHGV40020101	-0.47156	0.471555489	protein_coding	NM_004740
ASHGV40040899	-0.47149	0.471490387	protein_coding	ENST00000431696
ASHGV40005941	-0.47147	0.471473222	protein_coding	NM_004897
ASHGV40034186	-0.47143	0.471430555	protein_coding	NM_014139
ASHGV40052528	0.47137	0.471370368	protein_coding	NM_178448
ASHGV40010611	-0.47127	0.471265367	protein_coding	uc001tvb.3
ASHGV40050963	0.471252	0.471252477	protein_coding	NM_152416
ASHGV40054114	-0.47124	0.471240862	protein_coding	NM_016484
ASHGV40023944	-0.4712	0.471202618	protein_coding	NM_022834
ASHGV40046853	0.471079	0.471078625	protein_coding	NM_002946
ASHGV40036829	-0.47107	0.471071477	protein_coding	NM_207015
ASHGV40003092	-0.47105	0.471045405	protein_coding	NM_001171629
ASHGV40002872	-0.47095	0.470947648	protein_coding	NM_001256795
ASHGV40033672	-0.47082	0.470824242	protein_coding	NM_030642
ASHGV40016768	0.470641	0.470640602	protein_coding	NM_153618
ASHGV40054004	0.470632	0.470631768	protein_coding	NM_003179
ASHGV40023213	0.470425	0.470424601	protein_coding	NM_013326
ASHGV40031844	0.470418	0.470418483	protein_coding	NM_178456
ASHGV40037122	0.470392	0.47039248	protein_coding	NM_003846
ASHGV40046867	0.470375	0.470374996	protein_coding	NM_016188
ASHGV40029696	0.470331	0.470331219	protein_coding	NM_022826
ASHGV40016628	-0.47026	0.470258309	protein_coding	NM_001130010
ASHGV40026179	-0.47025	0.470251157	protein_coding	NM_005762
ASHGV40023278	0.470201	0.470201298	protein_coding	NM_017831
ASHGV40023636	0.470172	0.470171839	protein_coding	NM_001136180
ASHGV40044438	-0.47013	0.470134971	protein_coding	NM_182552
ASHGV40018488	-0.47013	0.470126447	protein_coding	NM_020664
ASHGV40005858	-0.4701	0.470104449	protein_coding	NM_001026
ASHGV40010966	-0.4701	0.470097554	protein_coding	NM_018009
ASHGV40000091	-0.47005	0.47005013	protein_coding	ENST00000342608
ASHGV40008262	0.46992	0.469920199	protein_coding	NM_001077242
ASHGV40031438	-0.46965	0.469645889	protein_coding	ENST00000234827
ASHGV40053240	0.469392	0.469391568	protein_coding	NM_006444
ASHGV40022343	0.469161	0.469161495	protein_coding	ENST00000574717
ASHGV40035929	0.468707	0.468707314	protein_coding	NM_016381
ASHGV40009187	0.46852	0.468520405	protein_coding	NM_003186
ASHGV40034152	-0.46845	0.468446651	protein_coding	NM_001039770
ASHGV40048150	0.46838	0.468380291	protein_coding	NM_003272

ASHGV40005236	0.468297	0.468297034	protein_coding	NM_013285
ASHGV40039004	-0.46821	0.468212673	protein_coding	NM_001890
ASHGV40032950	-0.46819	0.46818615	protein_coding	uc021wni.1
ASHGV40034280	-0.4679	0.467895581	protein_coding	NM_199183
ASHGV40020182	-0.46789	0.467892838	protein_coding	NM_005681
ASHGV40003196	-0.46787	0.467867845	protein_coding	NM_001256932
ASHGV40044983	-0.46782	0.467822448	protein_coding	NM_153370
ASHGV40052438	0.467756	0.467755523	protein_coding	NM_006753
ASHGV40009599	-0.46726	0.467255845	protein_coding	NM_023921
ASHGV40052197	0.467167	0.467167033	protein_coding	NM_139286
ASHGV40028500	-0.46716	0.467155039	protein_coding	ENST00000418835
ASHGV40020611	0.46696	0.46696026	protein_coding	NM_016070
ASHGV40024800	-0.46695	0.466954239	protein_coding	NM_016363
ASHGV40029259	-0.4669	0.466903232	protein_coding	NM_144632
ASHGV40028384	-0.46662	0.466617845	protein_coding	NM_018968
ASHGV40015015	0.466538	0.466537795	protein_coding	NM_017943
ASHGV40044961	0.466433	0.466433181	protein_coding	NM_007104
ASHGV40009379	-0.46635	0.46634682	protein_coding	uc021oev.1
ASHGV40003219	0.466303	0.466303377	protein_coding	NM_001289967
ASHGV40056645	-0.46629	0.466286903	protein_coding	NM_173080
ASHGV40011053	-0.46628	0.466281069	protein_coding	NM_001079815
ASHGV40032848	-0.46613	0.466132321	protein_coding	NM_053004
ASHGV40052254	0.466037	0.466036524	protein_coding	NM_001735
ASHGV40012276	-0.46601	0.466011765	protein_coding	NM_018663
ASHGV40025900	-0.46595	0.465948843	protein_coding	NM_001774
ASHGV40015287	0.46592	0.465920314	protein_coding	NM_018418
ASHGV40042088	-0.46591	0.46590931	protein_coding	NM_001012761
ASHGV40055793	0.465628	0.465628363	protein_coding	NM_032810
ASHGV40052941	-0.46556	0.465560397	protein_coding	NM_153267
ASHGV40056987	0.465415	0.465415421	protein_coding	NM_033296
ASHGV40017638	0.465342	0.465342148	protein_coding	NM_018340
ASHGV40016381	-0.46527	0.465271497	protein_coding	NM_001937
ASHGV40040636	-0.46525	0.465250087	protein_coding	NM_012475
ASHGV40050116	0.465224	0.465224156	protein_coding	NM_017570
ASHGV40051345	-0.46518	0.465184821	protein_coding	ENST00000517894
ASHGV40052161	0.465024	0.465023817	protein_coding	NM_003798
ASHGV40015179	0.464975	0.464974756	protein_coding	NM_152444
ASHGV40025776	0.464928	0.464928211	protein_coding	NM_015164
ASHGV40044746	-0.46485	0.464854769	protein_coding	NM_007047
ASHGV40005991	-0.46484	0.464836927	protein_coding	NM_025235
ASHGV40031926	0.464802	0.464801914	protein_coding	NM_015888
ASHGV40002386	-0.46479	0.464793378	protein_coding	NM_001006607
ASHGV40019970	0.464589	0.464589116	protein_coding	NM_004169
ASHGV40007319	-0.46429	0.464294765	protein_coding	NM_005169
ASHGV40016333	-0.46424	0.464241109	protein_coding	NM_198525
ASHGV40044979	0.464131	0.464130939	protein_coding	NM_002596
ASHGV40035664	0.464048	0.464048148	protein_coding	NM_138381

ASHGV40040239	-0.46403	0.464029092	protein_coding	NM_019087
ASHGV40006911	-0.46401	0.464014477	protein_coding	NM_001101802
ASHGV40029128	0.463817	0.463816958	protein_coding	NM_017952
ASHGV40016129	-0.46368	0.463675295	protein_coding	NM_022369
ASHGV40007626	-0.4636	0.463597208	protein_coding	NM_012459
ASHGV40030785	-0.46355	0.463549841	protein_coding	NM_178468
ASHGV40017715	-0.46352	0.463517436	protein_coding	NM_017539
ASHGV40013849	-0.46324	0.46323891	protein_coding	NM_033400
ASHGV40025754	0.463237	0.463236853	protein_coding	NM_020406
ASHGV40013668	-0.46321	0.463210639	protein_coding	NM_015011
ASHGV40045631	0.463143	0.463142742	protein_coding	NM_001040214
ASHGV40015081	-0.46313	0.463133906	protein_coding	ENST00000556717
ASHGV40052660	0.462973	0.462973046	protein_coding	NM_001039697
ASHGV40022150	0.462948	0.462947714	protein_coding	ENST00000602013
ASHGV40029988	-0.46292	0.462916561	protein_coding	NM_003507
ASHGV40007149	0.462863	0.462863035	protein_coding	NM_138456
ASHGV40024087	-0.4626	0.462598264	protein_coding	NM_145256
ASHGV40016156	0.462577	0.462576774	protein_coding	NM_005701
ASHGV40041667	-0.46254	0.462536068	protein_coding	NM_000163
ASHGV40027099	0.462489	0.462489259	protein_coding	NM_017849
ASHGV40055587	-0.46239	0.462392404	protein_coding	NM_024332
ASHGV40025673	0.462316	0.462315836	protein_coding	NM_001001414
ASHGV40026960	0.462262	0.462262468	protein_coding	NM_181575
ASHGV40030438	-0.46225	0.462246377	protein_coding	NM_001029871
ASHGV40014245	0.462169	0.462168506	protein_coding	NM_018373
ASHGV40049465	0.462155	0.462155071	protein_coding	NM_020647
ASHGV40055246	0.461974	0.46197395	protein_coding	NM_207318
ASHGV40034376	0.461739	0.461739326	protein_coding	NM_003773
ASHGV40054058	-0.46161	0.461609972	protein_coding	NM_138362
ASHGV40051554	-0.46151	0.461506958	protein_coding	NM_001122
ASHGV40019745	-0.4615	0.461502822	protein_coding	NM_207103
ASHGV40034879	0.461228	0.461227877	protein_coding	NM_002950
ASHGV40027689	-0.4611	0.46110362	protein_coding	ENST00000409786
ASHGV40019586	0.461067	0.461066508	protein_coding	NM_001012759
ASHGV40035997	0.460961	0.460961477	protein_coding	NM_198268
ASHGV40036378	-0.46082	0.460819761	protein_coding	NM_003594
ASHGV40010988	0.460767	0.460766677	protein_coding	NM_001159287
ASHGV40007721	-0.46073	0.460733108	protein_coding	NM_001382
ASHGV40026963	-0.46069	0.460690768	protein_coding	NM_138804
ASHGV40025203	-0.46065	0.460654087	protein_coding	NM_020780
ASHGV40029771	-0.46047	0.460466951	protein_coding	ENST00000409176
ASHGV40051184	0.460376	0.460375621	protein_coding	NM_017956
ASHGV40035822	0.460319	0.460318661	protein_coding	NM_001278323
ASHGV40012063	0.460309	0.460309486	protein_coding	NM_001109903
ASHGV40048240	0.460152	0.460152019	protein_coding	NM_003302
ASHGV40052310	-0.46015	0.460148995	protein_coding	uc004bpf.2
ASHGV40010008	-0.46014	0.460136793	protein_coding	NM_015665

ASHGV40030234	0.45995	0.45995017	protein_coding	NM_007237
ASHGV40042415	-0.45956	0.459558541	protein_coding	NM_173533
ASHGV40035365	0.4595	0.459499823	protein_coding	NM_004454
ASHGV40041730	-0.45945	0.459447266	protein_coding	NM_002203
ASHGV40008512	-0.45939	0.459386435	protein_coding	NM_001079808
ASHGV40031133	0.45929	0.45929004	protein_coding	uc021wfw.1
ASHGV40054157	0.459117	0.459117363	protein_coding	NM_138703
ASHGV40045055	0.458999	0.45899865	protein_coding	NM_015255
ASHGV40035996	-0.45899	0.458990682	protein_coding	NM_016483
ASHGV40056558	-0.45897	0.458974254	protein_coding	NM_024707
ASHGV40050015	-0.45895	0.458949998	protein_coding	NM_015912
ASHGV40018349	-0.45888	0.458876904	protein_coding	NM_003281
ASHGV40048133	0.458682	0.458682478	protein_coding	NM_021167
ASHGV40018953	0.45864	0.45863976	protein_coding	NM_152491
ASHGV40012477	-0.45857	0.458569762	protein_coding	NM_025138
ASHGV40055080	-0.45842	0.458422661	protein_coding	NM_052957
ASHGV40037864	0.458409	0.458409106	protein_coding	NM_198721
ASHGV40018257	-0.45841	0.458406299	protein_coding	NM_001025200
ASHGV40025749	-0.45816	0.458156123	protein_coding	NM_173633
ASHGV40024364	-0.4581	0.458099656	protein_coding	NM_017827
ASHGV40025916	-0.45798	0.457976064	protein_coding	NM_138639
ASHGV40020212	-0.45791	0.457905069	protein_coding	NM_001123392
ASHGV40057807	-0.45783	0.457832498	protein_coding	NM_018039
ASHGV40026423	0.457474	0.45747419	protein_coding	NM_000929
ASHGV40043286	-0.45728	0.457277431	protein_coding	NM_025257
ASHGV40033482	-0.45723	0.457231922	protein_coding	NM_001178126
ASHGV40021062	-0.45723	0.457226199	protein_coding	NM_014409
ASHGV40043364	0.457183	0.457182787	protein_coding	NM_032340
ASHGV40003105	0.457086	0.457086018	protein_coding	NM_001079858
ASHGV40020692	0.456861	0.456861131	protein_coding	NM_017647
ASHGV40010898	-0.45685	0.456854154	protein_coding	NM_001039029
ASHGV40012396	0.45683	0.45683015	protein_coding	NM_181785
ASHGV40007108	-0.45676	0.456756173	protein_coding	NM_199352
ASHGV40026321	-0.45668	0.456682387	protein_coding	NM_017765
ASHGV40056283	-0.45667	0.456674791	protein_coding	NM_024006
ASHGV40012247	-0.45666	0.45666339	protein_coding	ENST00000598023
ASHGV40044856	-0.45636	0.456361447	protein_coding	NM_000247
ASHGV40013705	-0.45635	0.456346064	protein_coding	NM_001077628
ASHGV40005001	0.45623	0.456229777	protein_coding	NM_003252
ASHGV40048266	-0.45617	0.456173879	protein_coding	NM_032831
ASHGV40034377	0.456078	0.45607764	protein_coding	NM_007275
ASHGV40052268	0.455989	0.455988737	protein_coding	NM_194252
ASHGV40010897	-0.45592	0.455923608	protein_coding	NM_024551
ASHGV40040878	0.455838	0.45583783	protein_coding	NM_007348
ASHGV40035721	-0.45568	0.455684362	protein_coding	NM_052953
ASHGV40039820	-0.45564	0.455643583	protein_coding	NM_015997
ASHGV40036853	0.455586	0.455586133	protein_coding	NM_033540

ASHGV40051369	-0.45556	0.455562731	protein_coding	uc022bcg.1
ASHGV40055426	-0.45554	0.455535909	protein_coding	NM_001159702
ASHGV40049067	-0.45553	0.455532149	protein_coding	NM_018250
ASHGV40042779	-0.45527	0.455273482	protein_coding	NM_173664
ASHGV40043712	-0.45521	0.45521433	protein_coding	NM_004370
ASHGV40021145	-0.45521	0.455213703	protein_coding	NM_001124758
ASHGV40023674	0.455177	0.455176858	protein_coding	NM_000156
ASHGV40002003	0.45517	0.455169517	protein_coding	ENST00000546840
ASHGV40042525	-0.45512	0.455123864	protein_coding	NM_006846
ASHGV40021756	-0.45503	0.45503167	protein_coding	NM_173478
ASHGV40023216	-0.4548	0.454795596	protein_coding	NM_001135993
ASHGV40042284	-0.45475	0.454753071	protein_coding	NM_133638
ASHGV40033864	-0.45461	0.454611848	protein_coding	NM_014346
ASHGV40006928	-0.45439	0.454386044	protein_coding	NM_001194986
ASHGV40031717	-0.45422	0.454221881	protein_coding	NM_181659
ASHGV40029971	-0.45422	0.454221366	protein_coding	NM_152524
ASHGV40030411	-0.45413	0.45413244	protein_coding	NM_152783
ASHGV40051715	-0.4541	0.454099841	protein_coding	NM_019897
ASHGV40005906	0.454079	0.454079467	protein_coding	NM_001012720
ASHGV40034628	-0.45407	0.454068535	protein_coding	NM_001001850
ASHGV40047237	0.454001	0.454001247	protein_coding	uc011krx.2
ASHGV40008880	-0.45387	0.453868059	protein_coding	NM_020193
ASHGV40025320	0.453842	0.453842493	protein_coding	NM_018467
ASHGV40040863	-0.45368	0.4536757	protein_coding	NM_001002901
ASHGV40026122	0.453636	0.453635758	protein_coding	NM_001193628
ASHGV40021485	-0.45363	0.453625902	protein_coding	NM_014573
ASHGV40042453	-0.45354	0.453541074	protein_coding	NM_018922
ASHGV40019724	-0.4535	0.453502956	protein_coding	NM_014520
ASHGV40038447	-0.45349	0.453494455	protein_coding	NM_032326
ASHGV40026503	0.453446	0.453445942	protein_coding	NM_006569
ASHGV40049427	0.453186	0.453186326	protein_coding	NM_004508
ASHGV40024009	-0.45313	0.453131173	protein_coding	NM_024794
ASHGV40033168	0.453095	0.453094871	protein_coding	NM_005740
ASHGV40010069	-0.45302	0.453019091	protein_coding	NM_014871
ASHGV40057739	-0.45292	0.452923618	protein_coding	NM_001004753
ASHGV40049158	0.452792	0.452791746	protein_coding	NM_000349
ASHGV40029338	0.452784	0.452784209	protein_coding	NM_153214
ASHGV40052441	0.452717	0.45271656	protein_coding	NM_033161
ASHGV40055850	-0.4527	0.452698055	protein_coding	NM_015631
ASHGV40047214	-0.45266	0.452657897	protein_coding	NM_207113
ASHGV40016696	0.452655	0.452654606	protein_coding	NM_015138
ASHGV40049630	0.45253	0.452529706	protein_coding	NM_001171795
ASHGV40056348	-0.45251	0.452505126	protein_coding	NM_006613
ASHGV40015145	0.452408	0.452407748	protein_coding	NM_014734
ASHGV40018501	-0.45232	0.45231917	protein_coding	NM_006618
ASHGV40021921	-0.45219	0.452192138	protein_coding	NM_022167
ASHGV40052830	-0.45219	0.452187797	protein_coding	NM_032226

ASHGV40043354	-0.45215	0.452147741	protein_coding	NM_003190
ASHGV40044916	-0.45211	0.452112366	protein_coding	NM_014234
ASHGV40047569	-0.45198	0.451982409	protein_coding	NM_014777
ASHGV40024100	0.451908	0.45190813	protein_coding	NM_007263
ASHGV40050348	-0.45184	0.451842464	protein_coding	NM_015024
ASHGV40005390	0.451824	0.45182403	protein_coding	NM_005180
ASHGV40033660	-0.45182	0.451818055	protein_coding	NM_001303508
ASHGV40015789	-0.45174	0.451741404	protein_coding	NM_152455
ASHGV40044413	0.45172	0.451720209	protein_coding	NM_001129895
ASHGV40047294	0.451711	0.451710829	protein_coding	NM_002889
ASHGV40052523	0.45155	0.451550436	protein_coding	ENST00000440465
ASHGV40013135	-0.45145	0.451452248	protein_coding	NM_015972
ASHGV40023067	0.451319	0.451319347	protein_coding	NM_033546
ASHGV40050133	0.451196	0.451196213	protein_coding	NM_014676
ASHGV40052574	-0.45112	0.451124895	protein_coding	NM_021951
ASHGV40022186	-0.4511	0.451100833	protein_coding	NM_023036
ASHGV40011917	0.451005	0.451004878	protein_coding	NM_013320
ASHGV40053359	0.450995	0.450995353	protein_coding	NM_138554
ASHGV40047247	-0.45099	0.450986236	protein_coding	ENST00000265310
ASHGV40020848	-0.45096	0.450958449	protein_coding	NM_014595
ASHGV40018252	-0.45086	0.450860843	protein_coding	NM_152649
ASHGV40051163	0.450737	0.450736912	protein_coding	NM_014943
ASHGV40047765	0.450706	0.45070589	protein_coding	NM_031903
ASHGV40052696	-0.45058	0.450579366	protein_coding	NM_002451
ASHGV40024793	-0.4505	0.450500309	protein_coding	NM_145057
ASHGV40053023	-0.4504	0.450395001	protein_coding	NM_001001670
ASHGV40018139	-0.45039	0.450385443	protein_coding	NM_001136505
ASHGV40039492	-0.45024	0.450243532	protein_coding	NM_006439
ASHGV40056062	0.450114	0.450113774	protein_coding	NM_017693
ASHGV40028479	-0.4497	0.449699902	protein_coding	NM_198182
ASHGV40019336	0.449433	0.449433181	protein_coding	NM_012426
ASHGV40015786	-0.44937	0.449368199	protein_coding	NM_201631
ASHGV40038930	0.44933	0.449330385	protein_coding	NM_006947
ASHGV40007201	-0.44931	0.44930804	protein_coding	NM_015399
ASHGV40052870	-0.44929	0.44929156	protein_coding	ENST00000377548
ASHGV40052530	-0.44927	0.449266967	protein_coding	ENST00000596585
ASHGV40037626	-0.4492	0.449202274	protein_coding	NM_173827
ASHGV40054589	-0.44919	0.449190654	protein_coding	NM_019848
ASHGV40025543	-0.44902	0.449018385	protein_coding	NM_032346
ASHGV40025090	-0.44894	0.4489424	protein_coding	NM_144614
ASHGV40021251	0.448889	0.448888599	protein_coding	ENST00000399413
ASHGV40006099	-0.44879	0.448791207	protein_coding	NM_021830
ASHGV40039269	-0.44878	0.448778624	protein_coding	NM_000191
ASHGV40008504	-0.44859	0.448592723	protein_coding	NM_207341
ASHGV40032842	0.448512	0.448511622	protein_coding	NM_005659
ASHGV40050643	0.448296	0.448295861	protein_coding	uc003xqy.1
ASHGV40005450	-0.44827	0.448273275	protein_coding	NM_012342

ASHGV40053469	-0.44809	0.448085199	protein_coding	NM_007135
ASHGV40044585	0.448014	0.448014255	protein_coding	NM_030969
ASHGV40007098	0.448011	0.44801089	protein_coding	NM_006362
ASHGV40009590	0.44801	0.448010449	protein_coding	NM_002260
ASHGV40014658	0.447994	0.447993665	protein_coding	NM_001098616
ASHGV40030918	-0.44786	0.447863686	protein_coding	NM_015945
ASHGV40035006	-0.44776	0.447762741	protein_coding	NM_001178139
ASHGV40043252	-0.44775	0.44774858	protein_coding	NM_019052
ASHGV40043282	0.447594	0.447593746	protein_coding	NM_005527
ASHGV40053983	-0.44755	0.447551527	protein_coding	NM_033518
ASHGV40046035	-0.44755	0.447545677	protein_coding	NM_014053
ASHGV40031261	0.447482	0.447481769	protein_coding	NM_153638
ASHGV40017272	0.447477	0.447476749	protein_coding	NM_006122
ASHGV40009691	0.447445	0.447444701	protein_coding	NM_003034
ASHGV40035776	0.447363	0.44736336	protein_coding	NM_013374
ASHGV40036747	-0.44717	0.447167737	protein_coding	ENST00000473137
ASHGV40018346	-0.44712	0.447119888	protein_coding	NM_021149
ASHGV40024315	-0.44711	0.447109679	protein_coding	NM_206894
ASHGV40014308	-0.44669	0.44668984	protein_coding	NM_002632
ASHGV40021860	-0.4465	0.446504501	protein_coding	NM_013351
ASHGV40048788	-0.44622	0.446219722	protein_coding	NM_001005504
ASHGV40042174	0.44614	0.446140474	protein_coding	NM_021614
ASHGV40018308	0.445845	0.445845436	protein_coding	NM_152342
ASHGV40055888	0.445826	0.445826046	protein_coding	NM_032709
ASHGV40034045	-0.44581	0.445806814	protein_coding	NM_152396
ASHGV40029735	-0.4458	0.445796018	protein_coding	NM_152381
ASHGV40029833	0.445764	0.445763861	protein_coding	NM_001042702
ASHGV40047253	0.445712	0.445711661	protein_coding	NM_014690
ASHGV40009398	0.445689	0.445688657	protein_coding	NM_014384
ASHGV40056398	0.445521	0.445521338	protein_coding	NM_004287
ASHGV40055280	0.44544	0.44544042	protein_coding	NM_001242617
ASHGV40024040	-0.44543	0.445425748	protein_coding	NM_021235
ASHGV40016157	0.445343	0.445343434	protein_coding	NM_019026
ASHGV40033016	0.445299	0.445299176	protein_coding	NM_003634
ASHGV40029507	-0.44516	0.445162619	protein_coding	NM_014369
ASHGV40016101	0.444995	0.444994904	protein_coding	NM_000520
ASHGV40011003	-0.44494	0.444942865	protein_coding	NM_001131025
ASHGV40008547	-0.44486	0.444862113	protein_coding	NM_173810
ASHGV40042363	-0.44485	0.444848811	protein_coding	NM_001300921
ASHGV40051694	-0.44478	0.444778055	protein_coding	NM_004629
ASHGV40034735	-0.4447	0.444695025	protein_coding	ENST00000383686
ASHGV40008707	0.444623	0.444623211	protein_coding	NM_012308
ASHGV40003072	-0.44451	0.444513396	protein_coding	NM_001008226
ASHGV40029513	-0.44424	0.444236992	protein_coding	NM_001277083
ASHGV40057325	-0.44421	0.444214584	protein_coding	NM_014478
ASHGV40008677	0.444099	0.444099176	protein_coding	NM_030981
ASHGV40029273	0.444038	0.44403764	protein_coding	NM_182640

ASHGV40042213	0.443776	0.443775828	protein_coding	NM_005509
ASHGV40031482	-0.44343	0.443426035	protein_coding	NM_002110
ASHGV40024005	0.443373	0.443372809	protein_coding	NM_005071
ASHGV40047603	0.443292	0.443292128	protein_coding	NM_007342
ASHGV40017134	-0.44328	0.443283963	protein_coding	NM_007364
ASHGV40042710	-0.44326	0.443263645	protein_coding	NM_022897
ASHGV40053651	0.443208	0.44320781	protein_coding	NM_000124
ASHGV40009205	0.443113	0.443112604	protein_coding	NM_001204077
ASHGV40000765	-0.44311	0.44311024	protein_coding	ENST00000433584
ASHGV40008360	0.44311	0.443110055	protein_coding	NM_021117
ASHGV40013011	0.443083	0.443083232	protein_coding	NM_001014283
ASHGV40030919	0.442993	0.442993118	protein_coding	NM_133171
ASHGV40046903	-0.44293	0.442933163	protein_coding	NM_145032
ASHGV40002367	0.442906	0.442906161	protein_coding	NM_176870
ASHGV40028865	0.442865	0.442865235	protein_coding	NM_002954
ASHGV40020422	0.442863	0.44286337	protein_coding	NM_025160
ASHGV40043347	0.442821	0.442820976	protein_coding	NM_021976
ASHGV40038098	0.442513	0.442512944	protein_coding	NM_001306215
ASHGV40031827	-0.4424	0.442398811	protein_coding	NM_003610
ASHGV40022846	-0.4422	0.442203287	protein_coding	NM_181654
ASHGV40015168	0.442005	0.442004601	protein_coding	NM_021239
ASHGV40031185	0.442004	0.442003963	protein_coding	NM_004799
ASHGV40027747	-0.44194	0.441935462	protein_coding	NM_005281
ASHGV40011560	0.441805	0.441805483	protein_coding	NM_182947
ASHGV40028354	-0.44178	0.441775128	protein_coding	NM_024296
ASHGV40055584	-0.44177	0.441765909	protein_coding	NM_001288747
ASHGV40009186	-0.44166	0.441658337	protein_coding	NM_001040455
ASHGV40053188	-0.44154	0.441540431	protein_coding	NM_001170741
ASHGV40011325	-0.44143	0.441433391	protein_coding	ENST00000595310
ASHGV40054413	-0.44126	0.441257649	protein_coding	NM_003069
ASHGV40045623	-0.44125	0.44125189	protein_coding	NM_001446
ASHGV40011733	-0.44117	0.441165584	protein_coding	NM_005599
ASHGV40043484	-0.44109	0.441085506	protein_coding	NM_001286554
ASHGV40001393	0.441008	0.441007972	protein_coding	NM_001003
ASHGV40016325	0.440949	0.440948824	protein_coding	NM_002693
ASHGV40010012	0.44093	0.440930017	protein_coding	NM_005176
ASHGV40034007	-0.44084	0.440839808	protein_coding	NM_001128220
ASHGV40001497	-0.44082	0.440822382	protein_coding	ENST00000506417
ASHGV40009497	-0.44071	0.440710423	protein_coding	NM_001065
ASHGV40004919	-0.44057	0.440568279	protein_coding	NM_020918
ASHGV40002619	-0.44055	0.44054934	protein_coding	ENST00000595946
ASHGV40054827	-0.44034	0.440341469	protein_coding	NM_138780
ASHGV40007224	-0.44033	0.440334372	protein_coding	NM_020441
ASHGV40040903	0.440236	0.440235917	protein_coding	NM_001035235
ASHGV40007974	-0.43998	0.439982528	protein_coding	NM_002555
ASHGV40036804	0.439876	0.439876134	protein_coding	NM_005414
ASHGV40015119	0.43983	0.439830215	protein_coding	NM_178155

ASHGV40022165	0.439747	0.439746522	protein_coding	NM_001050
ASHGV40009200	-0.43969	0.43969218	protein_coding	NM_001558
ASHGV40051551	-0.43959	0.43958914	protein_coding	NM_017645
ASHGV40034825	0.439537	0.439536723	protein_coding	NM_198402
ASHGV40013320	0.439465	0.439464876	protein_coding	NM_031915
ASHGV40008395	-0.43946	0.43945736	protein_coding	NM_005478
ASHGV40007320	0.439391	0.439390841	protein_coding	NM_030813
ASHGV40053923	-0.43936	0.439364157	protein_coding	NM_001123385
ASHGV40054118	-0.43936	0.439355381	protein_coding	NM_194298
ASHGV40030270	0.439343	0.439342679	protein_coding	NM_015575
ASHGV40041983	-0.43924	0.43924049	protein_coding	NM_031482
ASHGV40033791	-0.43905	0.439046371	protein_coding	NM_152613
ASHGV40001656	-0.43903	0.439033632	protein_coding	NM_005649
ASHGV40018832	0.439015	0.439014639	protein_coding	NM_006910
ASHGV40025934	0.43901	0.439010373	protein_coding	ENST00000253727
ASHGV40006350	0.438999	0.438998985	protein_coding	NM_017580
ASHGV40018919	0.438938	0.438937608	protein_coding	NM_001973
ASHGV40036335	-0.43893	0.438928848	protein_coding	NM_007072
ASHGV40024984	-0.43886	0.43886263	protein_coding	NM_032482
ASHGV40011976	0.438825	0.438825305	protein_coding	NM_032300
ASHGV40025241	0.438809	0.438808914	protein_coding	NM_014047
ASHGV40032770	0.438804	0.438804476	protein_coding	NM_015833
ASHGV40036614	0.438748	0.438747511	protein_coding	NM_004267
ASHGV40027469	0.438699	0.438698967	protein_coding	NM_032143
ASHGV40005810	-0.43861	0.438613376	protein_coding	uc009xrk.1
ASHGV40054874	0.438385	0.438385025	protein_coding	NM_000240
ASHGV40052047	0.438356	0.438356418	protein_coding	NM_033331
ASHGV40032119	0.437955	0.437955491	protein_coding	NM_003368
ASHGV40018151	-0.43792	0.437917734	protein_coding	NM_001100915
ASHGV40011516	-0.43772	0.437722729	protein_coding	NM_000456
ASHGV40030751	0.437667	0.437666888	protein_coding	NM_080616
ASHGV40031919	0.437642	0.437642247	protein_coding	NM_007346
ASHGV40026507	-0.43743	0.437426033	protein_coding	NM_178553
ASHGV40009021	-0.43741	0.437407367	protein_coding	NM_130847
ASHGV40009515	-0.43736	0.437361822	protein_coding	NM_153685
ASHGV40029738	0.43736	0.437360344	protein_coding	NM_020981
ASHGV40005987	0.437339	0.437338918	protein_coding	NM_001284274
ASHGV40013844	-0.4371	0.437095583	protein_coding	NM_001276318
ASHGV40028757	0.437039	0.437038828	protein_coding	NM_001145450
ASHGV40011086	0.437006	0.437006037	protein_coding	NM_020853
ASHGV40035234	-0.43675	0.436752978	protein_coding	NM_020949
ASHGV40023942	-0.43665	0.436647598	protein_coding	NM_000528
ASHGV40042738	-0.43665	0.436645265	protein_coding	NM_001205
ASHGV40045165	-0.43663	0.436627202	protein_coding	NM_018100
ASHGV40020533	-0.43653	0.436525311	protein_coding	NM_030802
ASHGV40045412	-0.4365	0.436498204	protein_coding	NM_015323
ASHGV40054994	0.436429	0.436428565	protein_coding	NM_001198799

ASHGV40009023	0.436266	0.436266418	protein_coding	NM_032102
ASHGV40020434	-0.43623	0.43623448	protein_coding	uc002iiv.1
ASHGV40007151	-0.43617	0.436168406	protein_coding	NM_080668
ASHGV40030136	0.436124	0.436123763	protein_coding	NM_014640
ASHGV40054194	-0.43611	0.436112112	protein_coding	NM_001012980
ASHGV40054163	0.436088	0.436087729	protein_coding	NM_032121
ASHGV40044347	0.435889	0.435889281	protein_coding	NM_004562
ASHGV40014464	-0.43586	0.435860746	protein_coding	NM_004993
ASHGV40008676	0.43567	0.435669524	protein_coding	NM_022822
ASHGV40038778	-0.43558	0.435579334	protein_coding	NM_001170700
ASHGV40022048	-0.43548	0.435480184	protein_coding	NM_003161
ASHGV40028672	0.435472	0.435472165	protein_coding	NM_002709
ASHGV40060855	-0.4353	0.435303295	protein_coding	uc009ysn.1
ASHGV40030876	-0.43528	0.435282721	protein_coding	NM_000022
ASHGV40011189	-0.43525	0.435251105	protein_coding	NM_021821
ASHGV40041168	0.435215	0.435215254	protein_coding	uc021yhu.1
ASHGV40046028	0.435165	0.435164808	protein_coding	NM_018341
ASHGV40037627	0.435042	0.43504187	protein_coding	NM_032217
ASHGV40050735	0.434803	0.43480259	protein_coding	NM_001282695
ASHGV40037188	-0.43454	0.434543336	protein_coding	ENST00000324058
ASHGV40019617	0.434328	0.434328052	protein_coding	NM_015471
ASHGV40044584	0.434225	0.434225356	protein_coding	NM_016462
ASHGV40036973	0.434193	0.434192546	protein_coding	NM_004892
ASHGV40033034	-0.43418	0.434184194	protein_coding	NM_001017981
ASHGV40055322	-0.43409	0.434087912	protein_coding	NM_001144000
ASHGV40027989	0.434087	0.434087275	protein_coding	NM_001080475
ASHGV40002654	-0.43404	0.43403771	protein_coding	ENST00000597961
ASHGV40020382	-0.43395	0.433948989	protein_coding	NM_007294
ASHGV40009266	0.433741	0.433740764	protein_coding	NM_006918
ASHGV40028293	-0.43361	0.433605179	protein_coding	NM_006037
ASHGV40053258	-0.43352	0.43352384	protein_coding	NM_018112
ASHGV40038065	-0.43352	0.433523136	protein_coding	NM_004362
ASHGV40050544	0.433445	0.433445252	protein_coding	NM_003816
ASHGV40045399	-0.43341	0.433411751	protein_coding	NM_003047
ASHGV40019361	-0.43338	0.433383396	protein_coding	NM_001361
ASHGV40022084	-0.43332	0.433318566	protein_coding	NM_001085423
ASHGV40024941	-0.43328	0.433284207	protein_coding	NM_001928
ASHGV40005015	0.433241	0.433241127	protein_coding	NM_017615
ASHGV40026547	0.433225	0.433225427	protein_coding	NM_017910
ASHGV40035208	0.433197	0.433196663	protein_coding	NM_178824
ASHGV40022591	-0.43315	0.433147993	protein_coding	NM_032933
ASHGV40030996	-0.43313	0.433128725	protein_coding	NM_015339
ASHGV40035749	0.433004	0.433003977	protein_coding	NM_178862
ASHGV40051856	0.433003	0.433003175	protein_coding	NM_017998
ASHGV40010968	-0.43292	0.432918644	protein_coding	NM_014865
ASHGV40018716	-0.43284	0.432837848	protein_coding	NM_001145204
ASHGV40023699	-0.43281	0.432808369	protein_coding	NM_018049

ASHGV40018940	-0.43278	0.432783603	protein_coding	NM_001330
ASHGV40036360	-0.43274	0.432743847	protein_coding	NM_004258
ASHGV40019439	0.432607	0.432606797	protein_coding	NM_016373
ASHGV40051021	-0.4326	0.432602682	protein_coding	NM_004412
ASHGV40036329	0.432586	0.432586015	protein_coding	NM_001142568
ASHGV40056026	-0.43258	0.432577024	protein_coding	NM_005288
ASHGV40046515	-0.43254	0.432539497	protein_coding	NM_005311
ASHGV40031583	-0.43237	0.432366455	protein_coding	ENST00000371268
ASHGV40054728	0.432052	0.432052201	protein_coding	NM_018360
ASHGV40053317	-0.43194	0.431936929	protein_coding	NM_133465
ASHGV40006279	-0.4318	0.431797666	protein_coding	NM_199461
ASHGV40043463	0.431633	0.431632604	protein_coding	NM_145063
ASHGV40011386	0.431605	0.431604559	protein_coding	NM_014764
ASHGV40024423	-0.43153	0.431531394	protein_coding	NM_001817
ASHGV40036417	-0.43136	0.431355276	protein_coding	NM_015900
ASHGV40014863	-0.43134	0.431335955	protein_coding	NM_017769
ASHGV40016981	0.431326	0.431325892	protein_coding	NM_006091
ASHGV40031761	-0.43129	0.431293822	protein_coding	NM_002827
ASHGV40011363	0.431233	0.431233216	protein_coding	NM_000486
ASHGV40012827	0.431193	0.431192696	protein_coding	NM_001097612
ASHGV40031087	0.43116	0.431160035	protein_coding	NM_001336
ASHGV40033631	0.431129	0.431128839	protein_coding	NM_012262
ASHGV40038474	-0.43106	0.431058456	protein_coding	NM_000142
ASHGV40003182	-0.43106	0.431057292	protein_coding	NM_001204848
ASHGV40046066	-0.43097	0.430968735	protein_coding	NM_003665
ASHGV40005274	-0.43094	0.430939787	protein_coding	ENST00000391437
ASHGV40018233	-0.43093	0.430930267	protein_coding	NM_017853
ASHGV40021642	0.430753	0.430752622	protein_coding	NM_012138
ASHGV40017422	0.430512	0.430511844	protein_coding	NM_015039
ASHGV40052783	-0.43051	0.430509589	protein_coding	NM_001142784
ASHGV40020307	-0.4305	0.430498035	protein_coding	ENST00000440582
ASHGV40028779	-0.43035	0.430354755	protein_coding	uc010faq.3
ASHGV40009708	0.430134	0.430133759	protein_coding	NM_005504
ASHGV40027928	-0.43005	0.430049963	protein_coding	NM_033066
ASHGV40018908	-0.42997	0.42997043	protein_coding	NM_016151
ASHGV40053007	0.429968	0.429967691	protein_coding	NM_007005
ASHGV40055934	-0.42983	0.429826193	protein_coding	NM_173191
ASHGV40018039	0.429565	0.429565198	protein_coding	NM_194314
ASHGV40036904	0.42956	0.42956044	protein_coding	NM_018358
ASHGV40017024	0.429533	0.429532978	protein_coding	NM_005744
ASHGV40050167	0.42934	0.429339502	protein_coding	NM_183421
ASHGV40050147	-0.42931	0.429310279	protein_coding	NM_138367
ASHGV40021928	-0.42925	0.429249197	protein_coding	NM_018346
ASHGV40025330	-0.42923	0.429233091	protein_coding	NM_000302
ASHGV40031202	0.429162	0.429161892	protein_coding	NM_031229
ASHGV40018780	-0.42915	0.42915014	protein_coding	NM_017888
ASHGV40035235	-0.42908	0.429084938	protein_coding	NM_001099645

ASHGV40050328	0.429053	0.429052961	protein_coding	NM_018142
ASHGV40008994	0.428825	0.42882483	protein_coding	NM_001008781
ASHGV40010079	0.428762	0.428761933	protein_coding	NM_013449
ASHGV40027914	0.428608	0.428608077	protein_coding	NM_130906
ASHGV40039200	-0.42858	0.428584849	protein_coding	NM_004428
ASHGV40023066	0.428542	0.428541566	protein_coding	NM_001303047
ASHGV40025018	-0.42844	0.428440582	protein_coding	NM_006339
ASHGV40010067	-0.42838	0.428383285	protein_coding	NM_004077
ASHGV40012005	-0.42836	0.428359512	protein_coding	NM_006817
ASHGV40017474	-0.42836	0.428357158	protein_coding	NM_152764
ASHGV40010409	-0.42827	0.428273037	protein_coding	ENST00000551941
ASHGV40010417	0.427996	0.427996152	protein_coding	NM_003297
ASHGV40003063	-0.42799	0.427985492	protein_coding	NM_001005189
ASHGV40021188	-0.42794	0.427943657	protein_coding	NM_001195228
ASHGV40046782	-0.42792	0.427921055	protein_coding	NM_152703
ASHGV40057582	-0.42792	0.427919936	protein_coding	NM_022148
ASHGV40021908	0.427886	0.42788608	protein_coding	NM_007067
ASHGV40042712	-0.42787	0.427871279	protein_coding	NM_002520
ASHGV40060858	-0.42776	0.427758265	protein_coding	uc010aiv.1
ASHGV40045467	-0.42775	0.427747803	protein_coding	NM_001198
ASHGV40020003	0.427717	0.42771744	protein_coding	NM_007202
ASHGV40047611	0.427691	0.427690636	protein_coding	NM_138771
ASHGV40032613	0.427684	0.427684172	protein_coding	NM_001288961
ASHGV40056031	0.427426	0.427426383	protein_coding	NM_145203
ASHGV40010944	0.427358	0.427357965	protein_coding	NM_002234
ASHGV40039787	0.427199	0.427199358	protein_coding	NM_021817
ASHGV40038368	0.42711	0.4271099	protein_coding	NM_018359
ASHGV40032140	0.427103	0.427103355	protein_coding	NM_000830
ASHGV40026157	-0.42696	0.426959526	protein_coding	ENST00000546949
ASHGV40050811	-0.42696	0.426955247	protein_coding	NM_004770
ASHGV40020359	0.426812	0.42681221	protein_coding	uc002hzy.3
ASHGV40000644	-0.42664	0.426636134	protein_coding	uc003ukg.2
ASHGV40030771	-0.42656	0.426558078	protein_coding	NM_000687
ASHGV40023650	-0.42646	0.426458516	protein_coding	NM_182577
ASHGV40011654	-0.42634	0.426343803	protein_coding	ENST00000399333
ASHGV40030853	-0.42619	0.426187311	protein_coding	NM_052846
ASHGV40007171	-0.4261	0.426103709	protein_coding	NM_021975
ASHGV40044810	-0.42609	0.426087694	protein_coding	NM_013941
ASHGV40031259	-0.42602	0.426021255	protein_coding	NM_018347
ASHGV40025192	0.425944	0.425944254	protein_coding	NM_001080503
ASHGV40021498	0.425894	0.425894403	protein_coding	NM_003170
ASHGV40007647	-0.42581	0.425806115	protein_coding	NM_020886
ASHGV40007093	-0.4257	0.425702372	protein_coding	NM_024784
ASHGV40045913	0.425601	0.425600979	protein_coding	NM_173516
ASHGV40056238	-0.42557	0.425569863	protein_coding	NM_021195
ASHGV40026169	0.425344	0.425344205	protein_coding	NM_001009
ASHGV40020391	-0.42532	0.425320242	protein_coding	NM_025237

ASHGV40023841	-0.4253	0.425304764	protein_coding	NM_032152
ASHGV40044475	0.4253	0.425299845	protein_coding	NM_001304331
ASHGV40030741	0.425225	0.425224827	protein_coding	NM_030815
ASHGV40043342	0.425158	0.425157982	protein_coding	NM_033554
ASHGV40018789	-0.42501	0.425005908	protein_coding	NM_020422
ASHGV40054595	-0.42497	0.424973706	protein_coding	NM_080612
ASHGV40027738	0.424901	0.424900562	protein_coding	NM_030650
ASHGV40043506	-0.42486	0.424861776	protein_coding	NM_014780
ASHGV40049065	0.424861	0.424860826	protein_coding	NM_172366
ASHGV40049608	0.424827	0.424827374	protein_coding	NM_001008495
ASHGV40006609	0.424826	0.424825516	protein_coding	NM_022061
ASHGV40050356	-0.42477	0.424770522	protein_coding	NM_001722
ASHGV40029517	-0.42477	0.424767399	protein_coding	NM_152698
ASHGV40057138	-0.4247	0.424698895	protein_coding	NM_001099221
ASHGV40025386	-0.4246	0.424604915	protein_coding	NM_017660
ASHGV40021951	0.424474	0.424474105	protein_coding	NM_002512
ASHGV40035791	0.424454	0.424453546	protein_coding	NM_000249
ASHGV40007427	0.424288	0.4242877	protein_coding	NM_021825
ASHGV40010532	-0.42429	0.424285231	protein_coding	NM_004075
ASHGV40052479	0.424206	0.424205863	protein_coding	NM_015447
ASHGV40019338	0.424155	0.424154542	protein_coding	NM_152456
ASHGV40034349	-0.42382	0.423823198	protein_coding	NM_000481
ASHGV40003169	0.423697	0.423696914	protein_coding	NM_001199973
ASHGV40010993	-0.42367	0.423673786	protein_coding	NM_138425
ASHGV40013058	0.423667	0.423667377	protein_coding	NM_175605
ASHGV40020425	0.423644	0.423643864	protein_coding	NM_001264573
ASHGV40056839	-0.4235	0.423495654	protein_coding	NM_000407
ASHGV40011282	0.423469	0.423469342	protein_coding	NM_032256
ASHGV40003095	-0.42346	0.423458695	protein_coding	NM_001544
ASHGV40005786	0.423261	0.423260726	protein_coding	NM_173473
ASHGV40053342	0.423258	0.423258112	protein_coding	NM_153045
ASHGV40027015	0.423239	0.423239005	protein_coding	NM_006464
ASHGV40006140	0.423234	0.42323356	protein_coding	NM_032727
ASHGV40051003	0.423137	0.423137379	protein_coding	NM_003114
ASHGV40025724	-0.42312	0.423124446	protein_coding	NM_001098821
ASHGV40055537	-0.42294	0.422944617	protein_coding	NM_007150
ASHGV40044496	0.422935	0.42293492	protein_coding	NM_020135
ASHGV40019697	0.422677	0.422677198	protein_coding	NM_032598
ASHGV40019982	0.422608	0.422608224	protein_coding	NM_012414
ASHGV40007035	-0.4224	0.422395044	protein_coding	NM_152852
ASHGV40056387	-0.42238	0.422383154	protein_coding	NM_006455
ASHGV40021191	0.422317	0.422317347	protein_coding	NM_032731
ASHGV40026352	-0.42229	0.42229181	protein_coding	NM_153213
ASHGV40009988	-0.42229	0.422287961	protein_coding	NM_175053
ASHGV40009259	-0.42228	0.422284545	protein_coding	NM_001198671
ASHGV40043949	0.422278	0.422277894	protein_coding	NM_015076
ASHGV40055952	0.422266	0.42226565	protein_coding	NM_015916

ASHGV40041303	0.422253	0.422253118	protein_coding	NM_032858
ASHGV40056671	-0.42223	0.422230926	protein_coding	NM_144706
ASHGV40052019	-0.42222	0.422221177	protein_coding	NM_017561
ASHGV40038552	0.422004	0.422003756	protein_coding	NM_020777
ASHGV40046087	-0.42193	0.421931862	protein_coding	ENST00000222990
ASHGV40025896	0.421875	0.421875349	protein_coding	NM_022165
ASHGV40034285	0.42182	0.421820343	protein_coding	NM_144716
ASHGV40030939	0.421603	0.421602835	protein_coding	NM_018837
ASHGV40008714	0.421207	0.421206796	protein_coding	NM_020811
ASHGV40000095	0.421147	0.421146564	protein_coding	NM_003375
ASHGV40008675	-0.42113	0.421131614	protein_coding	NM_018026
ASHGV40055815	0.421077	0.421076778	protein_coding	NM_019859
ASHGV40025346	0.421031	0.421030876	protein_coding	NM_015683
ASHGV40003206	-0.42079	0.420788046	protein_coding	NM_001278655
ASHGV40021536	0.420722	0.420721586	protein_coding	NM_004871
ASHGV40006360	-0.42058	0.420577969	protein_coding	uc001lis.1
ASHGV40037660	-0.42043	0.420425515	protein_coding	NM_017426
ASHGV40033014	0.42038	0.420379704	protein_coding	NM_003678
ASHGV40045142	0.420374	0.420374275	protein_coding	NM_022078
ASHGV40045335	-0.42011	0.420113865	protein_coding	NM_153362
ASHGV40003343	-0.42009	0.420085124	protein_coding	NM_173821
ASHGV40042571	0.419927	0.419926899	protein_coding	NM_002084
ASHGV40003100	-0.41983	0.419832085	protein_coding	NM_001040448
ASHGV40049698	-0.41976	0.419759361	protein_coding	NM_002568
ASHGV40036133	0.419698	0.419697958	protein_coding	NM_001080393
ASHGV40005759	0.419609	0.41960891	protein_coding	NM_004096
ASHGV40027762	-0.41954	0.419539469	protein_coding	NM_016953
ASHGV40037850	-0.41942	0.419419656	protein_coding	NM_014421
ASHGV40045646	0.419417	0.419416997	protein_coding	NM_005525
ASHGV40038061	-0.41938	0.419379332	protein_coding	NM_018717
ASHGV40011014	0.419306	0.419305643	protein_coding	NM_015509
ASHGV40007421	0.419297	0.419296807	protein_coding	NM_014488
ASHGV40057380	0.419182	0.419181664	protein_coding	NM_018295
ASHGV40060833	-0.41917	0.419169931	protein_coding	uc003eoz.1
ASHGV40056227	0.419013	0.419013026	protein_coding	NM_000517
ASHGV40007927	-0.41879	0.418792832	protein_coding	NM_003271
ASHGV40026511	-0.41878	0.418782816	protein_coding	NM_003353
ASHGV40053492	0.418768	0.418768453	protein_coding	NM_005094
ASHGV40006186	-0.41864	0.418643392	protein_coding	NM_201542
ASHGV40030463	-0.41862	0.418616436	protein_coding	NM_000490
ASHGV40031299	0.418456	0.418455793	protein_coding	NM_182797
ASHGV40024938	0.418192	0.418191606	protein_coding	NM_001700
ASHGV40034847	0.418013	0.41801337	protein_coding	NM_001008487
ASHGV40015392	0.417943	0.417943317	protein_coding	NM_016472
ASHGV40037886	-0.41792	0.41792133	protein_coding	NM_052864
ASHGV40037717	0.417707	0.417706867	protein_coding	NM_001115007
ASHGV40037926	-0.41767	0.417667631	protein_coding	NM_001083

ASHGV40039450	0.417614	0.417613766	protein_coding	NM_004632
ASHGV40009654	0.417588	0.41758826	protein_coding	NM_001175
ASHGV40000254	0.41743	0.417429635	protein_coding	ENST00000406829
ASHGV40044592	-0.41733	0.417328252	protein_coding	NM_001135575
ASHGV40031542	0.417179	0.417179025	protein_coding	NM_080748
ASHGV40008577	0.417067	0.417066818	protein_coding	NM_006819
ASHGV40007099	-0.41703	0.417027655	protein_coding	NM_003164
ASHGV40023743	-0.41698	0.416980532	protein_coding	NM_016539
ASHGV40000640	-0.41692	0.416922563	protein_coding	NM_001205019
ASHGV40054933	-0.41686	0.416861553	protein_coding	NM_007137
ASHGV40009163	0.416849	0.416849332	protein_coding	NM_015523
ASHGV40021010	-0.41673	0.416729935	protein_coding	NM_005782
ASHGV40033478	0.416576	0.416575521	protein_coding	NM_025065
ASHGV40034414	0.416549	0.416548792	protein_coding	NM_018446
ASHGV40054179	-0.41652	0.416524985	protein_coding	NM_153252
ASHGV40014324	0.416514	0.416513924	protein_coding	NM_007176
ASHGV40052160	0.416443	0.416442849	protein_coding	NM_003640
ASHGV40025657	0.416383	0.416382543	protein_coding	NM_000540
ASHGV40051747	-0.41618	0.416182241	protein_coding	NM_033655
ASHGV40044854	-0.41613	0.416134502	protein_coding	NM_014068
ASHGV40046795	-0.4161	0.416101909	protein_coding	NM_000446
ASHGV40018787	0.416027	0.416027075	protein_coding	NM_020424
ASHGV40053397	-0.416	0.416001554	protein_coding	NM_001286828
ASHGV40005840	0.415953	0.415952875	protein_coding	NM_032024
ASHGV40037743	0.41581	0.415809925	protein_coding	ENST00000358290
ASHGV40030100	-0.41568	0.415676648	protein_coding	NM_138390
ASHGV40049043	0.415587	0.415587223	protein_coding	NM_007257
ASHGV40002343	0.415401	0.415400519	protein_coding	ENST00000568766
ASHGV40012463	0.415144	0.415143789	protein_coding	NM_013338
ASHGV40053306	-0.41509	0.415091274	protein_coding	NM_001015882
ASHGV40030504	-0.41502	0.415016064	protein_coding	NM_015939
ASHGV40056655	-0.41497	0.41497013	protein_coding	NM_000427
ASHGV40012348	-0.41496	0.414957991	protein_coding	ENST00000422229
ASHGV40000435	0.414934	0.414933617	protein_coding	uc009wkt.1
ASHGV40038561	-0.41492	0.414924839	protein_coding	NM_018986
ASHGV40013357	-0.41448	0.414483483	protein_coding	NM_018204
ASHGV40027568	-0.41443	0.414425963	protein_coding	NM_005843
ASHGV40020000	0.414365	0.414364745	protein_coding	NM_014683
ASHGV40007228	-0.41429	0.414287887	protein_coding	NM_025124
ASHGV40030210	0.414161	0.414161017	protein_coding	NM_020864
ASHGV40004996	0.414091	0.414091101	protein_coding	NM_152374
ASHGV40045994	0.413677	0.413677404	protein_coding	NM_030615
ASHGV40008821	-0.41349	0.413485609	protein_coding	NM_001567
ASHGV40034225	0.413441	0.413441279	protein_coding	NM_144634
ASHGV40035615	0.413441	0.413441275	protein_coding	NM_006395
ASHGV40029201	-0.41327	0.413274269	protein_coding	NM_001293083
ASHGV40018548	0.413217	0.4132168	protein_coding	NM_031208

ASHGV40005932	0.413109	0.4131091	protein_coding	NM_003087
ASHGV40006221	-0.41301	0.413014579	protein_coding	NM_001227
ASHGV40001985	0.412966	0.412966368	protein_coding	NM_004282
ASHGV40046887	-0.41295	0.412947698	protein_coding	uc011kkm.2
ASHGV40012990	0.412938	0.412938357	protein_coding	NM_006322
ASHGV40007555	-0.41293	0.412926497	protein_coding	NM_001191016
ASHGV40038461	-0.41292	0.412920866	protein_coding	NM_004091
ASHGV40036003	0.412805	0.412805324	protein_coding	NM_014366
ASHGV40052162	0.412787	0.412787272	protein_coding	NM_032012
ASHGV40050768	0.412684	0.412684028	protein_coding	NM_019607
ASHGV40027535	0.412678	0.41267821	protein_coding	NM_002552
ASHGV40041975	0.412631	0.412631359	protein_coding	NM_002439
ASHGV40037365	-0.41253	0.412529502	protein_coding	NM_001242521
ASHGV40046906	0.412432	0.412431902	protein_coding	NM_014377
ASHGV40024525	0.412418	0.412418341	protein_coding	NM_005619
ASHGV40008705	0.412335	0.412334721	protein_coding	NM_177963
ASHGV40005639	0.412119	0.412118793	protein_coding	NM_005437
ASHGV40045859	-0.41208	0.41207632	protein_coding	NM_001001346
ASHGV40003152	0.412044	0.412043617	protein_coding	NM_001198812
ASHGV40037782	-0.41202	0.412018483	protein_coding	NM_174952
ASHGV40021749	-0.41189	0.411891524	protein_coding	NM_001070
ASHGV40009612	0.411806	0.411805504	protein_coding	NM_006249
ASHGV40015323	0.411767	0.411767065	protein_coding	NM_017437
ASHGV40007327	-0.41173	0.411733869	protein_coding	NM_014824
ASHGV40034813	-0.41167	0.411674706	protein_coding	NM_001023570
ASHGV40013832	-0.41167	0.411666546	protein_coding	NM_001130706
ASHGV40023748	-0.41165	0.411652943	protein_coding	NM_003025
ASHGV40041882	0.411479	0.411478933	protein_coding	NM_004291
ASHGV40039327	0.411472	0.411471784	protein_coding	NM_001128933
ASHGV40035497	0.411369	0.411368998	protein_coding	NM_182627
ASHGV40050817	-0.4112	0.411199178	protein_coding	NM_172037
ASHGV40019581	0.411194	0.411194474	protein_coding	NM_144604
ASHGV40047050	0.411193	0.411192808	protein_coding	NM_002107
ASHGV40001852	0.41101	0.411009898	protein_coding	NM_001206631
ASHGV40025236	0.410809	0.410808721	protein_coding	NM_052876
ASHGV40034311	0.410713	0.410712603	protein_coding	NM_000094
ASHGV40016949	-0.41069	0.410691219	protein_coding	NM_004727
ASHGV40013313	0.410683	0.410682774	protein_coding	NM_000321
ASHGV40052361	-0.41068	0.410678728	protein_coding	NM_052844
ASHGV40057687	-0.41064	0.410636902	protein_coding	NM_170753
ASHGV40023943	-0.4104	0.410401783	protein_coding	NM_016145
ASHGV40035906	-0.41038	0.410384827	protein_coding	NM_000316
ASHGV40035821	-0.41036	0.410364319	protein_coding	NM_018704
ASHGV40055010	-0.41032	0.410317303	protein_coding	NM_130467
ASHGV40023209	0.410158	0.410158089	protein_coding	NM_001100619
ASHGV40057525	-0.41012	0.41011685	protein_coding	ENST00000417276
ASHGV40048112	0.410042	0.4100422	protein_coding	NM_001039706

ASHGV40021823	-0.41003	0.410026531	protein_coding	NM_144608
ASHGV40054676	0.409954	0.40995401	protein_coding	NM_015691
ASHGV40045743	-0.40986	0.409855414	protein_coding	uc003qia.1
ASHGV40009289	0.409816	0.409815679	protein_coding	NM_020716
ASHGV40034316	-0.4098	0.409804224	protein_coding	NM_022911
ASHGV40018536	-0.4098	0.409803875	protein_coding	NM_016111
ASHGV40050273	-0.40979	0.409788765	protein_coding	NM_145043
ASHGV40053793	0.409785	0.409785274	protein_coding	NM_002641
ASHGV40006837	0.409734	0.409733994	protein_coding	NM_015957
ASHGV40054673	-0.40961	0.409613043	protein_coding	NM_005647
ASHGV40013620	-0.40954	0.409540882	protein_coding	NM_007129
ASHGV40044311	0.409512	0.409512455	protein_coding	NM_032861
ASHGV40000724	-0.40944	0.40943917	protein_coding	NM_030652
ASHGV40030151	0.409377	0.409377444	protein_coding	NM_138802
ASHGV40025359	-0.40925	0.409252565	protein_coding	NM_004864
ASHGV40056044	-0.40922	0.409218378	protein_coding	NM_004559
ASHGV40047208	0.409045	0.40904524	protein_coding	NM_022750
ASHGV40054546	0.409034	0.409033507	protein_coding	NM_000808
ASHGV40057588	-0.40902	0.409022814	protein_coding	NM_001195081
ASHGV40026012	-0.409	0.409001087	protein_coding	NM_138374
ASHGV40020064	0.408972	0.40897195	protein_coding	NM_015584
ASHGV40017962	-0.40888	0.40888331	protein_coding	NM_004352
ASHGV40008425	-0.40887	0.408874975	protein_coding	NM_015139
ASHGV40055222	0.40881	0.408809939	protein_coding	NM_022838
ASHGV40005229	-0.40846	0.408464766	protein_coding	NM_024700
ASHGV40010101	-0.40822	0.408217634	protein_coding	NM_020142
ASHGV40050674	-0.40816	0.408162829	protein_coding	NM_024831
ASHGV40020136	-0.40816	0.408158523	protein_coding	NM_006495
ASHGV40033588	0.408068	0.408068298	protein_coding	NM_005243
ASHGV40044663	0.408064	0.40806397	protein_coding	NM_182757
ASHGV40010065	-0.40804	0.40804397	protein_coding	NM_173595
ASHGV40025782	-0.40795	0.40795291	protein_coding	NM_001205280
ASHGV40057519	-0.40793	0.407929799	protein_coding	ENST00000377590
ASHGV40000359	0.407819	0.407818798	protein_coding	NM_001002913
ASHGV40029903	0.407727	0.407727323	protein_coding	NM_017694
ASHGV40006472	-0.4075	0.407499178	protein_coding	NM_001572
ASHGV40030828	-0.40728	0.40727876	protein_coding	NM_012186
ASHGV40005640	-0.40726	0.407264699	protein_coding	NM_017646
ASHGV40031470	-0.40721	0.407209632	protein_coding	NM_030789
ASHGV40008049	-0.40719	0.40718959	protein_coding	NM_001004460
ASHGV40045144	0.407006	0.407006139	protein_coding	NM_018132
ASHGV40023492	0.40699	0.406989704	protein_coding	NM_001123366
ASHGV40022191	-0.40687	0.406873237	protein_coding	NM_007261
ASHGV40051365	-0.40687	0.406865438	protein_coding	ENST00000395172
ASHGV40035581	0.406809	0.40680868	protein_coding	NM_015453
ASHGV40019771	-0.40674	0.406742118	protein_coding	ENST00000593646
ASHGV40031635	0.406648	0.406647961	protein_coding	NM_016276

ASHGV40052077	-0.40661	0.406613152	protein_coding	NM_001267571
ASHGV40015543	0.406491	0.406491438	protein_coding	NM_022489
ASHGV40042560	0.406321	0.406320917	protein_coding	NM_001135243
ASHGV40032267	0.406302	0.406302179	protein_coding	NM_002633
ASHGV40022116	0.406213	0.406212832	protein_coding	NM_002266
ASHGV40056834	-0.40606	0.406057624	protein_coding	NM_018943
ASHGV40010875	0.406013	0.406012742	protein_coding	NM_005645
ASHGV40054757	-0.40592	0.405915946	protein_coding	NM_032797
ASHGV40026047	-0.40561	0.405614736	protein_coding	uc010erm.2
ASHGV40035903	0.405499	0.405499007	protein_coding	NM_003212
ASHGV40057866	-0.40538	0.405382754	protein_coding	uc010snb.1
ASHGV40048250	-0.40536	0.405363236	protein_coding	NM_030961
ASHGV40013777	-0.40529	0.4052916	protein_coding	NM_007110
ASHGV40050085	-0.4052	0.405202255	protein_coding	NM_014654
ASHGV40022956	0.405153	0.405153303	protein_coding	NM_175907
ASHGV40031379	0.404975	0.404974605	protein_coding	NM_016100
ASHGV40018567	-0.40495	0.404947082	protein_coding	NM_032271
ASHGV40027432	-0.40487	0.4048725	protein_coding	NM_001281517
ASHGV40008517	-0.40438	0.404380682	protein_coding	NM_016464
ASHGV40055741	0.404171	0.404171487	protein_coding	NM_001080438
ASHGV40022102	-0.40409	0.404085282	protein_coding	NM_145811
ASHGV40011213	-0.40406	0.404055966	protein_coding	NM_152438
ASHGV40031645	-0.40397	0.403966626	protein_coding	NM_001098798
ASHGV40005432	-0.40393	0.403931417	protein_coding	NM_014317
ASHGV40025148	0.403773	0.403772894	protein_coding	NM_007375
ASHGV40015551	0.403454	0.4034543	protein_coding	NM_138790
ASHGV40031810	0.403402	0.403401546	protein_coding	NM_001013646
ASHGV40006604	0.403371	0.403370887	protein_coding	NM_015324
ASHGV40027979	-0.40327	0.40326547	protein_coding	NM_003709
ASHGV40026158	-0.40321	0.403209773	protein_coding	NM_207421
ASHGV40054402	-0.40319	0.403194141	protein_coding	NM_178470
ASHGV40009646	-0.40319	0.403188505	protein_coding	NM_175054
ASHGV40036894	0.403088	0.403088415	protein_coding	NM_017644
ASHGV40050652	-0.40299	0.402988895	protein_coding	NM_005285
ASHGV40034942	0.402987	0.402987246	protein_coding	NM_015391
ASHGV40055141	0.402916	0.4029155	protein_coding	NM_007265
ASHGV40048656	-0.40252	0.402522293	protein_coding	NM_138434
ASHGV40019025	0.402482	0.40248187	protein_coding	NM_000293
ASHGV40025558	-0.40239	0.402393721	protein_coding	NM_002151
ASHGV40037869	-0.40239	0.402389563	protein_coding	NM_030821
ASHGV40024665	-0.40234	0.402338508	protein_coding	NM_001308429
ASHGV40015072	-0.40215	0.402145831	protein_coding	NM_001042663
ASHGV40033070	0.402142	0.402142373	protein_coding	NM_003490
ASHGV40047319	-0.40187	0.401873085	protein_coding	NM_144727
ASHGV40046648	0.401734	0.401734293	protein_coding	NM_001017440
ASHGV40036013	0.401611	0.401611045	protein_coding	NM_001128840
ASHGV40035434	-0.40161	0.401610067	protein_coding	NM_198505

ASHGV40040164	-0.4016	0.401601698	protein_coding	NM_002001
ASHGV40001321	-0.40139	0.401387339	protein_coding	ENST00000605933
ASHGV40016608	-0.40136	0.401358102	protein_coding	NM_012125
ASHGV40012003	0.401213	0.401212755	protein_coding	NM_139078
ASHGV40025229	-0.40119	0.401189205	protein_coding	NM_004343
ASHGV40056257	-0.40118	0.401181137	protein_coding	NM_001034841
ASHGV40022364	0.40095	0.400950389	protein_coding	NM_144999
ASHGV40027149	0.40086	0.400859785	protein_coding	NM_004854
ASHGV40016462	-0.40076	0.400758162	protein_coding	uc021sxw.2
ASHGV40016609	-0.40076	0.400757371	protein_coding	NM_152595
ASHGV40043794	-0.40074	0.400740233	protein_coding	NM_020320
ASHGV40055997	0.40074	0.400740061	protein_coding	NM_001085481
ASHGV40047825	0.400479	0.400479138	protein_coding	NM_003364
ASHGV40047716	0.400162	0.400162128	protein_coding	NM_030636
ASHGV40008536	-0.40016	0.400159085	protein_coding	NM_002407
ASHGV40024643	0.400118	0.400117735	protein_coding	NM_015953
ASHGV40014791	0.400038	0.40003776	protein_coding	NM_001042635
ASHGV40041966	0.399773	0.399772936	protein_coding	NM_014458
ASHGV40055201	-0.39974	0.39973808	protein_coding	NM_001325
ASHGV40031696	0.399637	0.399636646	protein_coding	NM_006252
ASHGV40010637	-0.3996	0.399597967	protein_coding	NM_015335
ASHGV40006040	-0.39932	0.399321157	protein_coding	ENST00000372522
ASHGV40054001	0.399276	0.399275742	protein_coding	NM_001029896
ASHGV40031473	-0.3992	0.399197064	protein_coding	NM_032609
ASHGV40044508	-0.39919	0.399189189	protein_coding	NM_004332
ASHGV40009737	-0.39915	0.39914945	protein_coding	NM_016551
ASHGV40025784	-0.39909	0.39909362	protein_coding	NM_207348
ASHGV40020408	-0.39903	0.399034375	protein_coding	NM_000342
ASHGV40048089	0.399027	0.399027404	protein_coding	NM_021145
ASHGV40012072	0.39898	0.398980053	protein_coding	NM_022491
ASHGV40031420	0.39898	0.39897952	protein_coding	NM_022482
ASHGV40024877	-0.39892	0.398921065	protein_coding	NM_024833
ASHGV40010537	0.398678	0.398678272	protein_coding	NM_012406
ASHGV40009999	-0.3986	0.398602171	protein_coding	NM_001300814
ASHGV40025275	-0.39859	0.398591364	protein_coding	NM_033025
ASHGV40045094	-0.3985	0.398496828	protein_coding	NM_001078175
ASHGV40023197	-0.3984	0.398397474	protein_coding	NM_020774
ASHGV40019841	-0.39839	0.398392023	protein_coding	NM_014308
ASHGV40057495	0.39799	0.397989846	protein_coding	uc022bdi.1
ASHGV40009401	0.397923	0.397923328	protein_coding	uc021qso.2
ASHGV40036370	0.397892	0.39789153	protein_coding	NM_014170
ASHGV40033928	0.397866	0.397866254	protein_coding	NM_033517
ASHGV40044313	-0.39775	0.397747571	protein_coding	NM_006519
ASHGV40034921	0.397742	0.397741508	protein_coding	NM_153240
ASHGV40008889	0.397714	0.397714279	protein_coding	NM_018367
ASHGV40035715	-0.39766	0.397657372	protein_coding	NM_001290216
ASHGV40048659	-0.39765	0.39764955	protein_coding	NM_006352

ASHGV40009920	0.397462	0.397462205	protein_coding	NM_144593
ASHGV40010936	0.3972	0.397200358	protein_coding	NM_003845
ASHGV40016372	-0.39705	0.397047476	protein_coding	NM_020211
ASHGV40036414	-0.39705	0.397047467	protein_coding	NM_152305
ASHGV40040397	0.397015	0.397014864	protein_coding	NM_015566
ASHGV40008653	0.396778	0.396778077	protein_coding	NM_032223
ASHGV40053296	-0.3966	0.396597827	protein_coding	ENST00000452145
ASHGV40031442	-0.39658	0.396576848	protein_coding	ENST00000391366
ASHGV40029981	-0.39656	0.396558013	protein_coding	NM_032974
ASHGV40042371	-0.39652	0.39652315	protein_coding	ENST00000314358
ASHGV40048354	0.396265	0.396264761	protein_coding	NM_000245
ASHGV40000017	-0.3961	0.396097743	protein_coding	ENST00000259631
ASHGV40018943	0.395612	0.395612234	protein_coding	NM_173854
ASHGV40024092	0.395564	0.395563519	protein_coding	NM_001308373
ASHGV40021439	0.395373	0.39537313	protein_coding	NM_005646
ASHGV40054628	-0.39531	0.395310369	protein_coding	NM_001011719
ASHGV40005271	-0.39519	0.39518517	protein_coding	NM_004566
ASHGV40003225	-0.39514	0.395139798	protein_coding	NM_001291468
ASHGV40026502	-0.39494	0.394942336	protein_coding	NM_001134693
ASHGV40030410	-0.39489	0.394886095	protein_coding	NM_032329
ASHGV40025539	0.394669	0.394668847	protein_coding	NM_001114093
ASHGV40021701	-0.39466	0.39466372	protein_coding	NM_133264
ASHGV40041621	-0.3945	0.3944982	protein_coding	NM_152281
ASHGV40002522	-0.39449	0.394485816	protein_coding	ENST00000587519
ASHGV40030740	-0.39448	0.394483972	protein_coding	NM_080611
ASHGV40030051	-0.39434	0.394343316	protein_coding	NM_005048
ASHGV40049304	-0.39429	0.394290035	protein_coding	NM_001279357
ASHGV40000176	0.39426	0.394259824	protein_coding	NM_004271
ASHGV40038029	-0.39422	0.394218055	protein_coding	NM_004698
ASHGV40035671	0.394162	0.394161839	protein_coding	NM_001144382
ASHGV40001816	-0.39416	0.394157074	protein_coding	NM_004517
ASHGV40054824	-0.39389	0.393894998	protein_coding	NM_000397
ASHGV40017574	0.393765	0.393764872	protein_coding	NM_152459
ASHGV40046662	-0.39367	0.393670362	protein_coding	NM_148912
ASHGV40017522	0.393602	0.393601949	protein_coding	NM_004203
ASHGV40006021	0.393554	0.39355394	protein_coding	NM_001776
ASHGV40039805	-0.39352	0.393521052	protein_coding	uc021xvr.2
ASHGV40057197	0.393514	0.393513706	protein_coding	NM_003527
ASHGV40017405	0.393383	0.393382658	protein_coding	NM_016310
ASHGV40055090	0.393301	0.393300631	protein_coding	NM_001042506
ASHGV40005172	-0.39327	0.393265873	protein_coding	NM_000831
ASHGV40018199	0.393198	0.393198463	protein_coding	NM_001031725
ASHGV40022328	-0.3931	0.393095283	protein_coding	NM_173626
ASHGV40003294	-0.39307	0.393072488	protein_coding	NM_021006
ASHGV40041605	-0.39305	0.393051824	protein_coding	NM_018321
ASHGV40035499	0.393027	0.393027025	protein_coding	NM_032898
ASHGV40052424	-0.39286	0.392857526	protein_coding	NM_022779

ASHGV40028468	0.392834	0.392834134	protein_coding	NM_016207
ASHGV40030705	-0.39264	0.392636925	protein_coding	ENST00000593352
ASHGV40008235	0.392636	0.392635858	protein_coding	NM_152316
ASHGV40006196	0.392572	0.392571991	protein_coding	NM_014456
ASHGV40000856	-0.39235	0.392354199	protein_coding	ENST00000437723
ASHGV40044085	-0.39231	0.392307714	protein_coding	NM_001145659
ASHGV40001629	-0.39229	0.392292066	protein_coding	ENST00000514871
ASHGV40011520	0.392021	0.392020714	protein_coding	NM_032786
ASHGV40040905	-0.39198	0.39197872	protein_coding	NM_014697
ASHGV40014908	-0.39195	0.391954866	protein_coding	ENST00000556667
ASHGV40001937	-0.39192	0.3919183	protein_coding	ENST00000537495
ASHGV40031243	0.39181	0.391809655	protein_coding	NM_022575
ASHGV40043872	0.39172	0.391720467	protein_coding	NM_015491
ASHGV40014475	0.391623	0.391623248	protein_coding	NM_016150
ASHGV40006147	-0.39144	0.391444636	protein_coding	NM_004210
ASHGV40042682	0.391293	0.391293038	protein_coding	NM_015238
ASHGV40008038	0.391243	0.391243158	protein_coding	NM_000543
ASHGV40051946	-0.39118	0.391181756	protein_coding	NM_001039803
ASHGV40045006	-0.39106	0.391058224	protein_coding	NM_001206927
ASHGV40035886	0.390992	0.390991971	protein_coding	NM_015004
ASHGV40053125	-0.39087	0.390868625	protein_coding	NM_001012267
ASHGV40034144	-0.39078	0.39078307	protein_coding	NM_017801
ASHGV40009871	0.390774	0.390773728	protein_coding	NM_004719
ASHGV40028871	0.390747	0.39074741	protein_coding	NM_001080433
ASHGV40042033	-0.39073	0.390726899	protein_coding	uc003kjx.3
ASHGV40024401	0.390683	0.390682812	protein_coding	NM_000713
ASHGV40054575	0.390639	0.390638804	protein_coding	NM_003491
ASHGV40047535	0.39056	0.39056043	protein_coding	NM_004578
ASHGV40033610	-0.39053	0.390526288	protein_coding	NM_000355
ASHGV40009010	-0.3905	0.3904966	protein_coding	NM_001098672
ASHGV40017217	0.390467	0.390466768	protein_coding	NM_007200
ASHGV40055099	0.39034	0.390340181	protein_coding	NM_145052
ASHGV40057492	0.390219	0.390218509	protein_coding	NM_182905
ASHGV40032191	0.390171	0.390171248	protein_coding	NM_016631
ASHGV40054142	0.390137	0.390136588	protein_coding	ENST00000373518
ASHGV40052246	-0.39007	0.390067176	protein_coding	NM_001080497
ASHGV40031070	0.390048	0.390047636	protein_coding	NM_020182
ASHGV40019113	0.389944	0.389943705	protein_coding	NM_005611
ASHGV40035167	0.38982	0.38982008	protein_coding	NM_002268
ASHGV40008336	0.38979	0.389789614	protein_coding	NM_139178
ASHGV40011441	-0.38958	0.389583194	protein_coding	NM_138473
ASHGV40048632	-0.38934	0.389338353	protein_coding	NM_001195220
ASHGV40029251	-0.38932	0.389323162	protein_coding	NM_003854
ASHGV40032310	0.389221	0.389220502	protein_coding	ENST00000329015
ASHGV40042062	-0.38917	0.389168877	protein_coding	ENST00000504099
ASHGV40055664	0.389126	0.389126464	protein_coding	ENST00000602680
ASHGV40050362	-0.38908	0.389083494	protein_coding	NM_005775

ASHGV40006611	-0.38894	0.38893849	protein_coding	NM_001004490
ASHGV40007358	0.388811	0.388810762	protein_coding	NM_001039548
ASHGV40006023	0.388759	0.388758783	protein_coding	ENST00000472454
ASHGV40030273	-0.38871	0.388707463	protein_coding	ENST00000409905
ASHGV40028728	0.388392	0.388391904	protein_coding	NM_001278505
ASHGV40007791	-0.38836	0.388360639	protein_coding	NM_001080546
ASHGV40048198	0.38833	0.38832999	protein_coding	NM_005720
ASHGV40019603	-0.38829	0.388285733	protein_coding	NM_182531
ASHGV40035883	-0.38827	0.388267843	protein_coding	NM_020963
ASHGV40034484	0.388259	0.388259425	protein_coding	NM_002012
ASHGV40032849	-0.38823	0.388227541	protein_coding	NM_024627
ASHGV40054562	-0.38816	0.388155646	protein_coding	NM_080701
ASHGV40032569	-0.38812	0.388119896	protein_coding	NM_181600
ASHGV40049253	-0.38791	0.387909364	protein_coding	NM_006904
ASHGV40020073	-0.38789	0.387891449	protein_coding	NM_006461
ASHGV40039148	0.387714	0.387714138	protein_coding	NM_016323
ASHGV40024348	-0.38765	0.387645971	protein_coding	NM_152657
ASHGV40051820	-0.38765	0.387645894	protein_coding	ENST00000600472
ASHGV40053666	0.387619	0.387618543	protein_coding	NM_183241
ASHGV40023726	0.387612	0.387611617	protein_coding	NM_001287529
ASHGV40023184	0.387544	0.387544291	protein_coding	NM_001145029
ASHGV40031563	-0.38751	0.387513902	protein_coding	NM_005417
ASHGV40029547	0.3875	0.387499841	protein_coding	NM_015361
ASHGV40038152	0.387475	0.387474619	protein_coding	NM_003013
ASHGV40001593	-0.38747	0.387472623	protein_coding	NM_178019
ASHGV40052775	0.387457	0.387456681	protein_coding	NM_016525
ASHGV40009903	0.387299	0.387299408	protein_coding	NM_152320
ASHGV40041465	0.387229	0.38722944	protein_coding	NM_012073
ASHGV40049457	-0.38722	0.387220508	protein_coding	NM_153225
ASHGV40014373	0.387176	0.387176356	protein_coding	NM_015859
ASHGV40023987	0.387055	0.387054825	protein_coding	NM_001008701
ASHGV40017271	-0.38701	0.387008035	protein_coding	NM_002005
ASHGV40007076	-0.38685	0.386854746	protein_coding	NM_001404
ASHGV40012138	0.386778	0.386777679	protein_coding	NM_003959
ASHGV40018069	0.386772	0.386771844	protein_coding	NM_020807
ASHGV40031578	0.386751	0.386750803	protein_coding	NM_015047
ASHGV40043520	-0.38672	0.386723	protein_coding	NM_020750
ASHGV40032241	0.3867	0.386699633	protein_coding	NM_000411
ASHGV40009653	0.386571	0.386570624	protein_coding	NM_152321
ASHGV40029312	-0.38654	0.38654019	protein_coding	NM_001099289
ASHGV40009508	0.386499	0.386499218	protein_coding	NM_001258308
ASHGV40027095	0.38647	0.386470359	protein_coding	NM_004418
ASHGV40015767	-0.38643	0.386429037	protein_coding	NM_001206670
ASHGV40022071	-0.38639	0.386389986	protein_coding	NM_030779
ASHGV40014950	0.38638	0.386379713	protein_coding	NM_020937
ASHGV40036251	0.386292	0.386292062	protein_coding	NM_153605
ASHGV40019892	0.386235	0.386234742	protein_coding	NM_018127

ASHGV40013850	-0.38623	0.386226319	protein_coding	NM_003917
ASHGV40033419	-0.38621	0.38620672	protein_coding	NM_080646
ASHGV40042103	-0.38615	0.386151578	protein_coding	ENST00000597120
ASHGV40036281	-0.38595	0.38595145	protein_coding	NM_020202
ASHGV40016672	-0.38576	0.3857578	protein_coding	NM_005092
ASHGV40011669	0.385679	0.385679291	protein_coding	NM_175623
ASHGV40042736	-0.38561	0.385608612	protein_coding	NM_003945
ASHGV40026102	0.385492	0.38549186	protein_coding	NM_176811
ASHGV40009016	0.3854	0.385399519	protein_coding	NM_002033
ASHGV40049031	0.385378	0.38537824	protein_coding	uc010luc.2
ASHGV40017121	-0.38537	0.385370307	protein_coding	NM_000745
ASHGV40008807	-0.38522	0.385217364	protein_coding	NM_018172
ASHGV40043298	-0.38514	0.385141367	protein_coding	NM_019105
ASHGV40053652	-0.385	0.384997642	protein_coding	NM_032928
ASHGV40011926	0.384962	0.384962077	protein_coding	NM_015275
ASHGV40018321	-0.38495	0.384952329	protein_coding	NM_005792
ASHGV40011554	-0.38494	0.384942003	protein_coding	NM_052897
ASHGV40038797	-0.38464	0.384643174	protein_coding	NM_006859
ASHGV40049737	0.384504	0.38450406	protein_coding	NM_030780
ASHGV40039237	-0.38443	0.384429227	protein_coding	NM_025058
ASHGV40024940	-0.38442	0.384419295	protein_coding	NM_001972
ASHGV40045509	0.384306	0.384305829	protein_coding	NM_015891
ASHGV40024557	0.384242	0.384241756	protein_coding	NM_018215
ASHGV40041433	0.384168	0.384168353	protein_coding	NM_152902
ASHGV40019313	-0.384	0.383996538	protein_coding	NM_024562
ASHGV40041766	-0.38398	0.383983191	protein_coding	NM_002022
ASHGV40056319	-0.38396	0.383958252	protein_coding	NM_001242780
ASHGV40056383	0.383917	0.383917493	protein_coding	NM_033059
ASHGV40026120	0.38384	0.383840275	protein_coding	NM_001001668
ASHGV40014733	0.383792	0.383792108	protein_coding	NM_022734
ASHGV40056905	-0.38352	0.383518166	protein_coding	NM_013286
ASHGV40002435	-0.38313	0.38313172	protein_coding	uc002hrf.1
ASHGV40005360	0.38312	0.383119922	protein_coding	NM_003473
ASHGV40024361	0.38289	0.38288982	protein_coding	NM_001195833
ASHGV40009591	0.382867	0.382867186	protein_coding	NM_002259
ASHGV40034351	-0.38284	0.38283653	protein_coding	NM_032316
ASHGV40009110	0.382832	0.382831832	protein_coding	NM_004109
ASHGV40016014	0.382816	0.382815947	protein_coding	NM_001136043
ASHGV40022832	0.382741	0.382741061	protein_coding	NM_005603
ASHGV40033916	0.382613	0.382612747	protein_coding	NM_014678
ASHGV40032801	-0.3823	0.382296782	protein_coding	NM_015151
ASHGV40010325	0.382174	0.382173778	protein_coding	NM_014167
ASHGV40001936	-0.38217	0.382170803	protein_coding	NM_001047
ASHGV40042372	-0.38217	0.382167358	protein_coding	NM_003101
ASHGV40053271	-0.38215	0.382148892	protein_coding	NM_021224
ASHGV40017062	0.382105	0.382104994	protein_coding	NM_001030005
ASHGV40026905	-0.38201	0.382014593	protein_coding	NM_003096

ASHGV40047348	-0.38198	0.381981537	protein_coding	NM_007349
ASHGV40051701	-0.38196	0.381961245	protein_coding	uc022bgl.1
ASHGV40008600	-0.38177	0.381772899	protein_coding	NM_003942
ASHGV40001479	0.381686	0.381686091	protein_coding	NM_001012337
ASHGV40025790	-0.38166	0.381658466	protein_coding	NM_005581
ASHGV40037949	0.381624	0.381623715	protein_coding	NM_003305
ASHGV40026142	0.381602	0.381601559	protein_coding	NM_153263
ASHGV40042448	0.381598	0.381598356	protein_coding	NM_018934
ASHGV40002690	-0.3815	0.381497056	protein_coding	ENST00000601243
ASHGV40014301	0.381448	0.381447994	protein_coding	NM_000428
ASHGV40044625	-0.38138	0.38137907	protein_coding	NM_152737
ASHGV40033205	0.381047	0.381047441	protein_coding	NM_138338
ASHGV40006293	-0.38102	0.38101997	protein_coding	NM_001030059
ASHGV40047476	-0.381	0.38100401	protein_coding	NM_014855
ASHGV40036210	0.380999	0.3809985	protein_coding	ENST00000473136
ASHGV40025098	-0.38081	0.380807722	protein_coding	NM_198534
ASHGV40007165	0.380755	0.380754786	protein_coding	NM_001130144
ASHGV40027929	0.380748	0.380748295	protein_coding	NM_020919
ASHGV40024014	-0.3807	0.380704838	protein_coding	ENST00000397410
ASHGV40001281	0.380655	0.380654736	protein_coding	NM_005638
ASHGV40031486	-0.38051	0.380513646	protein_coding	NM_004798
ASHGV40032819	-0.38046	0.380464757	protein_coding	NM_001282225
ASHGV40017263	0.380437	0.380436739	protein_coding	NM_003870
ASHGV40020727	-0.38009	0.380091583	protein_coding	NM_001199165
ASHGV40027904	0.380017	0.380016937	protein_coding	NM_015265
ASHGV40034332	0.379822	0.379821974	protein_coding	uc010hkp.1
ASHGV40017439	-0.37972	0.379721174	protein_coding	NM_207419
ASHGV40033030	0.379682	0.379681808	protein_coding	NM_001037666
ASHGV40057841	-0.37954	0.379535661	protein_coding	NM_006331
ASHGV40015922	-0.37948	0.379476921	protein_coding	NM_003888
ASHGV40053589	-0.37944	0.379440419	protein_coding	NM_017588
ASHGV40019187	-0.37925	0.379249918	protein_coding	uc002elz.1
ASHGV40003253	0.379221	0.379220556	protein_coding	NM_003406
ASHGV40041662	0.379173	0.379173155	protein_coding	NM_175921
ASHGV40044083	-0.37893	0.37892712	protein_coding	NM_004830
ASHGV40019645	-0.37884	0.378841367	protein_coding	NM_006987
ASHGV40002563	-0.37855	0.378550098	protein_coding	ENST00000591723
ASHGV40033346	0.378457	0.378457189	protein_coding	NM_002969
ASHGV40041221	-0.37846	0.378456869	protein_coding	NM_016290
ASHGV40024054	-0.3784	0.378396642	protein_coding	NM_005234
ASHGV40047775	0.378342	0.378341565	protein_coding	NM_001014436
ASHGV40037935	-0.37831	0.378306233	protein_coding	NM_024574
ASHGV40055075	-0.37823	0.378227353	protein_coding	NM_007363
ASHGV40034386	0.37821	0.378209561	protein_coding	NM_016210
ASHGV40027604	-0.37818	0.378178511	protein_coding	NM_017837
ASHGV40022861	0.378177	0.378177345	protein_coding	NM_012327
ASHGV40046009	-0.37777	0.37776928	protein_coding	NM_001166412

ASHGV40031157	-0.37766	0.377657604	protein_coding	NM_152864
ASHGV40023710	0.37752	0.377520051	protein_coding	uc002lwe.3
ASHGV40030426	-0.37735	0.377350149	protein_coding	NM_025077
ASHGV40023889	-0.37709	0.37708957	protein_coding	ENST00000488011
ASHGV40017370	-0.37694	0.376943434	protein_coding	NM_021133
ASHGV40015561	-0.37677	0.376766036	protein_coding	NM_004689
ASHGV40020881	-0.37674	0.376735755	protein_coding	NM_001080542
ASHGV40002287	-0.37668	0.376683627	protein_coding	ENST00000564543
ASHGV40009261	-0.37636	0.376356035	protein_coding	NM_014619
ASHGV40051370	-0.37634	0.376338077	protein_coding	NM_138465
ASHGV40003210	0.376212	0.376211554	protein_coding	NM_001282484
ASHGV40020964	0.376163	0.376162912	protein_coding	NM_019020
ASHGV40018624	0.376061	0.376060677	protein_coding	NM_153028
ASHGV40021153	-0.37592	0.375916359	protein_coding	NM_001001683
ASHGV40009652	0.375577	0.375576908	protein_coding	NM_000900
ASHGV40024539	0.375458	0.375458149	protein_coding	NM_004819
ASHGV40035042	-0.37544	0.375444687	protein_coding	NM_020353
ASHGV40016861	0.375377	0.375376803	protein_coding	NM_015532
ASHGV40046548	-0.37531	0.375313204	protein_coding	NM_014302
ASHGV40036912	-0.37519	0.375194641	protein_coding	NM_001278698
ASHGV40034770	0.374943	0.374942987	protein_coding	NM_020817
ASHGV40045596	0.374854	0.3748537	protein_coding	NM_138459
ASHGV40032152	-0.37459	0.374591262	protein_coding	NM_181624
ASHGV40035502	0.374586	0.374586491	protein_coding	NM_007362
ASHGV40041911	0.374507	0.374506982	protein_coding	NM_001037637
ASHGV40043190	-0.37449	0.374488638	protein_coding	NM_052923
ASHGV40036693	-0.37441	0.374409492	protein_coding	NM_002563
ASHGV40000791	-0.37439	0.374388636	protein_coding	NM_001172651
ASHGV40039007	-0.37438	0.374377573	protein_coding	NM_020452
ASHGV40006215	-0.37435	0.374349007	protein_coding	NM_030756
ASHGV40020990	-0.3743	0.374302246	protein_coding	NM_001614
ASHGV40005583	0.3742	0.374200151	protein_coding	NM_001123376
ASHGV40016759	0.374189	0.374188656	protein_coding	NM_012388
ASHGV40036663	-0.37418	0.374175186	protein_coding	ENST00000593416
ASHGV40037071	0.37417	0.374169934	protein_coding	NM_152699
ASHGV40053151	0.374151	0.374151431	protein_coding	NM_032558
ASHGV40007223	-0.37414	0.374135479	protein_coding	NM_005608
ASHGV40030493	-0.374	0.374000074	protein_coding	NM_002592
ASHGV40019258	0.373982	0.373982417	protein_coding	NM_020786
ASHGV40035830	-0.37377	0.373773644	protein_coding	NM_001145082
ASHGV40036226	-0.37374	0.373741167	protein_coding	NM_152367
ASHGV40051823	0.373692	0.373692283	protein_coding	NM_001163
ASHGV40025533	0.373591	0.37359107	protein_coding	NM_001806
ASHGV40030413	-0.37355	0.37355497	protein_coding	NM_001167600
ASHGV40015083	0.373536	0.373535866	protein_coding	NM_001530
ASHGV40056887	-0.37345	0.373448275	protein_coding	NM_000994
ASHGV40009638	-0.37336	0.373361821	protein_coding	NM_004120

ASHGV40035469	0.373275	0.37327531	protein_coding	NM_012287
ASHGV40056449	0.373171	0.373171443	protein_coding	NM_006965
ASHGV40050440	-0.37304	0.373044606	protein_coding	NM_001440
ASHGV40023463	0.372953	0.372953362	protein_coding	NM_031891
ASHGV40039459	-0.37292	0.37291633	protein_coding	NM_172250
ASHGV40035832	0.372874	0.372873681	protein_coding	NM_198484
ASHGV40038534	0.37276	0.37276015	protein_coding	NM_006005
ASHGV40017504	0.372731	0.372731184	protein_coding	uc010bsp.1
ASHGV40000047	-0.37272	0.372715372	protein_coding	ENST00000309775
ASHGV40014682	0.37264	0.37264015	protein_coding	NM_000157
ASHGV40003050	0.372619	0.372619403	protein_coding	NM_000290
ASHGV40035960	0.372393	0.372393385	protein_coding	NM_006841
ASHGV40031364	0.372365	0.372364892	protein_coding	NM_001083330
ASHGV40028128	-0.37232	0.372319125	protein_coding	NM_001039569
ASHGV40046419	-0.3723	0.372303613	protein_coding	NM_000168
ASHGV40030732	-0.3722	0.372195115	protein_coding	NM_001037500
ASHGV40019209	-0.3721	0.372100973	protein_coding	NM_025179
ASHGV40046223	0.372092	0.372092348	protein_coding	NM_001002926
ASHGV40036535	-0.37207	0.372065997	protein_coding	NM_152395
ASHGV40038550	-0.37206	0.372062275	protein_coding	NM_152293
ASHGV40052552	-0.37198	0.371975138	protein_coding	NM_138462
ASHGV40009873	0.371877	0.371877283	protein_coding	NM_030674
ASHGV40011688	-0.37169	0.371691387	protein_coding	NM_002506
ASHGV40045124	-0.37159	0.371594722	protein_coding	NM_012120
ASHGV40013971	0.371521	0.371520619	protein_coding	NM_003317
ASHGV40046466	-0.37147	0.371471277	protein_coding	ENST00000596600
ASHGV40041273	-0.37147	0.371469341	protein_coding	NM_001142306
ASHGV40055245	-0.37141	0.37141169	protein_coding	NM_001143978
ASHGV40028710	-0.37126	0.371261776	protein_coding	NM_000627
ASHGV40032307	0.37114	0.371139792	protein_coding	NM_020727
ASHGV40056326	0.371131	0.371130999	protein_coding	NM_153018
ASHGV40049153	-0.37113	0.371126678	protein_coding	NM_000025
ASHGV40020869	0.371119	0.371119296	protein_coding	ENST00000254810
ASHGV40044013	0.37085	0.370850105	protein_coding	NM_020399
ASHGV40014809	-0.37084	0.370839503	protein_coding	ENST00000558325
ASHGV40010481	-0.37071	0.370709755	protein_coding	NM_153694
ASHGV40018193	-0.37069	0.370693869	protein_coding	NM_032382
ASHGV40002644	0.37064	0.370640358	protein_coding	ENST00000597445
ASHGV40014723	-0.37062	0.370622905	protein_coding	NM_000270
ASHGV40015832	0.370343	0.370342545	protein_coding	NM_000138
ASHGV40024007	0.370289	0.370288615	protein_coding	NM_000435
ASHGV40015605	-0.37025	0.370253245	protein_coding	NM_019066
ASHGV40052960	0.370148	0.370148312	protein_coding	NM_000700
ASHGV40042338	0.370101	0.370100686	protein_coding	NM_014864
ASHGV40025335	0.370038	0.370038104	protein_coding	NM_024656
ASHGV40018313	-0.37	0.369999316	protein_coding	NM_152337
ASHGV40006024	-0.3699	0.369900742	protein_coding	NM_022356

ASHGV40031588	-0.36987	0.369869529	protein_coding	NM_001018082
ASHGV40008690	-0.36987	0.369869499	protein_coding	NM_005700
ASHGV40011549	-0.36985	0.369852078	protein_coding	NM_205848
ASHGV40050976	-0.36984	0.36984326	protein_coding	NM_002998
ASHGV40015502	-0.36982	0.369815469	protein_coding	NM_003300
ASHGV40056707	-0.36971	0.36971282	protein_coding	NM_001008489
ASHGV40044707	0.369692	0.369691609	protein_coding	NM_018473
ASHGV40015140	-0.36949	0.369489512	protein_coding	NM_001193360
ASHGV40039490	-0.36944	0.369438716	protein_coding	NM_181885
ASHGV40039299	0.369304	0.369303623	protein_coding	NM_003360
ASHGV40043882	0.369167	0.369166566	protein_coding	NM_006828
ASHGV40006644	-0.36913	0.36912608	protein_coding	NM_015012
ASHGV40035984	-0.36906	0.36905586	protein_coding	NM_001085479
ASHGV40025944	-0.36898	0.368983703	protein_coding	NM_033068
ASHGV40042229	0.368799	0.368799202	protein_coding	NM_152546
ASHGV40015340	0.368579	0.368579364	protein_coding	NM_182971
ASHGV40022480	0.36857	0.368570078	protein_coding	NM_001042388
ASHGV40015227	0.368557	0.368557247	protein_coding	NM_024654
ASHGV40015591	0.368502	0.368501746	protein_coding	NM_015726
ASHGV40018803	0.368498	0.368497577	protein_coding	NM_018119
ASHGV40035041	0.368488	0.36848843	protein_coding	NM_000935
ASHGV40014827	-0.36833	0.368328814	protein_coding	NM_015299
ASHGV40034293	-0.36826	0.368255821	protein_coding	NM_003074
ASHGV40045582	0.368192	0.368191981	protein_coding	NM_015952
ASHGV40000203	0.36794	0.367940333	protein_coding	NM_207310
ASHGV40013936	0.367893	0.367893124	protein_coding	NM_000449
ASHGV40012015	0.367789	0.367789411	protein_coding	NM_016817
ASHGV40018491	-0.36756	0.36756194	protein_coding	NM_145270
ASHGV40005675	-0.36753	0.367532679	protein_coding	NM_003201
ASHGV40029396	0.367414	0.367413734	protein_coding	NM_016133
ASHGV40003205	0.367413	0.367413011	protein_coding	NM_001278267
ASHGV40042551	-0.36737	0.36737155	protein_coding	NM_001001669
ASHGV40023982	-0.36718	0.36718136	protein_coding	NM_001277378
ASHGV40043937	-0.36713	0.367132384	protein_coding	NM_022765
ASHGV40016493	0.367088	0.367087994	protein_coding	NM_052903
ASHGV40000172	0.366979	0.366978503	protein_coding	ENST00000378590
ASHGV40010064	0.366975	0.366974649	protein_coding	NM_005785
ASHGV40032738	0.366906	0.366906298	protein_coding	NM_004649
ASHGV40052690	0.366861	0.366861249	protein_coding	ENST00000338382
ASHGV40045970	-0.36684	0.366837963	protein_coding	NM_007045
ASHGV40025382	0.366799	0.366799367	protein_coding	NM_003721
ASHGV40028342	0.366778	0.366777766	protein_coding	NM_012316
ASHGV40037996	0.36654	0.366540484	protein_coding	NM_144643
ASHGV40033113	-0.36648	0.366482081	protein_coding	NM_024955
ASHGV40044959	0.366408	0.366407651	protein_coding	NM_006238
ASHGV40030137	0.366374	0.366373564	protein_coding	NM_004047
ASHGV40029654	0.366372	0.36637236	protein_coding	NM_052905

ASHGV40039372	-0.36616	0.366160499	protein_coding	NM_001039717
ASHGV40044824	-0.36596	0.365964541	protein_coding	NM_170783
ASHGV40025033	-0.36588	0.365878809	protein_coding	NM_005755
ASHGV40007967	0.365818	0.365818132	protein_coding	NM_139022
ASHGV40015689	0.365686	0.365685736	protein_coding	NM_014106
ASHGV40049963	-0.36567	0.365674453	protein_coding	NM_001115
ASHGV40021679	-0.36535	0.365345803	protein_coding	NM_152490
ASHGV40033227	-0.36523	0.365234372	protein_coding	NM_032311
ASHGV40052837	-0.36523	0.365230808	protein_coding	NM_144964
ASHGV40032713	0.365182	0.365182366	protein_coding	NM_018961
ASHGV40015124	-0.36514	0.36513514	protein_coding	NM_004494
ASHGV40021425	0.36505	0.36504979	protein_coding	NM_000382
ASHGV40037172	0.364892	0.364891709	protein_coding	NM_014455
ASHGV40036027	-0.3647	0.364699289	protein_coding	NM_181727
ASHGV40052189	0.364634	0.364633857	protein_coding	NM_021218
ASHGV40030033	-0.3646	0.364597153	protein_coding	NM_173077
ASHGV40033929	-0.36457	0.364572336	protein_coding	NM_001097
ASHGV40022514	0.364296	0.364296121	protein_coding	NM_023075
ASHGV40020769	0.364293	0.364293411	protein_coding	NM_023007
ASHGV40030252	-0.36409	0.36409382	protein_coding	NM_152614
ASHGV40026937	-0.36409	0.364089245	protein_coding	NM_001080410
ASHGV40027332	0.36402	0.364020477	protein_coding	NM_024121
ASHGV40028932	0.363978	0.363978094	protein_coding	NM_005722
ASHGV40047107	0.363967	0.363967228	protein_coding	NM_018077
ASHGV40023153	-0.36369	0.363690215	protein_coding	NM_014214
ASHGV40020552	0.363582	0.363581882	protein_coding	NM_016504
ASHGV40051168	-0.36358	0.363575258	protein_coding	NM_032899
ASHGV40017127	-0.36346	0.363456923	protein_coding	NM_206839
ASHGV40040929	0.363426	0.363425617	protein_coding	NM_005471
ASHGV40024618	-0.36321	0.363212098	protein_coding	NM_003323
ASHGV40010933	0.363186	0.363185983	protein_coding	NM_020375
ASHGV40003142	0.363171	0.363171181	protein_coding	NM_004987
ASHGV40003090	0.363166	0.363165915	protein_coding	NM_001031827
ASHGV40023367	0.363107	0.363106864	protein_coding	NM_014772
ASHGV40031813	0.363013	0.3630134	protein_coding	NM_003222
ASHGV40046136	0.362952	0.362951627	protein_coding	NM_139179
ASHGV40052277	-0.36292	0.362921615	protein_coding	NM_012363
ASHGV40054926	-0.36289	0.362888083	protein_coding	NM_001654
ASHGV40025034	-0.36279	0.362794021	protein_coding	NM_018074
ASHGV40044236	-0.36274	0.362741452	protein_coding	NM_198887
ASHGV40041235	-0.36258	0.362575488	protein_coding	NM_012474
ASHGV40015084	0.362418	0.362418249	protein_coding	NM_003082
ASHGV40037950	-0.36238	0.362378396	protein_coding	NM_000586
ASHGV40009602	-0.36226	0.36226141	protein_coding	NM_023922
ASHGV40033602	-0.36221	0.362211056	protein_coding	NM_001017437
ASHGV40031388	-0.36218	0.362184197	protein_coding	NM_012255
ASHGV40036901	0.362125	0.362125026	protein_coding	NM_003907

ASHGV40039248	-0.36212	0.362117433	protein_coding	NM_005327
ASHGV40007942	0.361802	0.361801742	protein_coding	NM_001256627
ASHGV40021537	-0.36177	0.36177302	protein_coding	NM_015594
ASHGV40039449	0.361567	0.361566532	protein_coding	NM_003601
ASHGV40006633	-0.36153	0.361530144	protein_coding	NM_001289058
ASHGV40030791	-0.36153	0.361528554	protein_coding	NM_178134
ASHGV40033750	-0.36144	0.361441779	protein_coding	NM_002409
ASHGV40044234	-0.36129	0.361291695	protein_coding	NM_007044
ASHGV40012740	0.361277	0.361276911	protein_coding	NM_000115
ASHGV40020472	0.361176	0.36117567	protein_coding	uc002ilo.2
ASHGV40028674	-0.36109	0.361087687	protein_coding	NM_015131
ASHGV40040610	0.361032	0.361032405	protein_coding	NM_017676
ASHGV40020767	0.360617	0.36061694	protein_coding	NM_080283
ASHGV40024337	0.360461	0.360461437	protein_coding	NM_001172690
ASHGV40055517	-0.36044	0.360440934	protein_coding	NM_004224
ASHGV40052046	-0.36031	0.36030539	protein_coding	NM_153695
ASHGV40033044	0.360273	0.360273169	protein_coding	NM_001303256
ASHGV40014507	-0.36022	0.360223806	protein_coding	NM_021966
ASHGV40005342	-0.35994	0.359941663	protein_coding	NM_005540
ASHGV40036533	0.359864	0.359863517	protein_coding	NM_024800
ASHGV40053538	0.359799	0.359799495	protein_coding	NM_000050
ASHGV40032585	-0.35978	0.359780863	protein_coding	NM_178817
ASHGV40013624	0.359669	0.359668902	protein_coding	NM_004791
ASHGV40044602	0.359445	0.359445486	protein_coding	NM_001100829
ASHGV40021906	0.359411	0.359411043	protein_coding	NM_007225
ASHGV40047841	0.359362	0.359361892	protein_coding	NM_198570
ASHGV40035207	-0.35935	0.359353219	protein_coding	NM_006217
ASHGV40043913	0.35911	0.359109579	protein_coding	NM_004849
ASHGV40040919	-0.35909	0.359093409	protein_coding	NM_005219
ASHGV40029542	-0.35909	0.359091144	protein_coding	NM_058241
ASHGV40021159	0.359072	0.359072087	protein_coding	NM_153827
ASHGV40020354	0.359012	0.359011647	protein_coding	NM_139276
ASHGV40046192	0.358885	0.358885232	protein_coding	NM_005924
ASHGV40048533	-0.35868	0.358676238	protein_coding	NM_015905
ASHGV40044160	-0.3586	0.358596938	protein_coding	NM_020464
ASHGV40020480	-0.35859	0.3585922	protein_coding	NM_138355
ASHGV40014010	0.358528	0.35852781	protein_coding	NM_017658
ASHGV40018954	0.358519	0.358519126	protein_coding	NM_004960
ASHGV40050198	-0.35838	0.358384686	protein_coding	uc003wpz.1
ASHGV40014823	-0.35836	0.358360455	protein_coding	NM_003145
ASHGV40031127	-0.35803	0.358028471	protein_coding	NM_003185
ASHGV40014811	-0.35799	0.357992324	protein_coding	uc031qod.1
ASHGV40020872	-0.35793	0.35792839	protein_coding	NM_199242
ASHGV40006006	-0.35772	0.357721822	protein_coding	NM_181745
ASHGV40051000	0.357559	0.35755939	protein_coding	NM_017890
ASHGV40021803	-0.35735	0.35735317	protein_coding	uc002igq.1
ASHGV40032125	-0.35731	0.357307792	protein_coding	NM_013240

ASHGV40037589	-0.35723	0.357225151	protein_coding	NM_182502
ASHGV40035839	0.357215	0.357215015	protein_coding	NM_006135
ASHGV40034881	-0.3572	0.357202702	protein_coding	uc003elf.1
ASHGV40001831	-0.35719	0.35719034	protein_coding	ENST00000528405
ASHGV40042769	0.357164	0.357163789	protein_coding	NM_022754
ASHGV40010421	-0.35713	0.357126789	protein_coding	ENST00000549961
ASHGV40018177	0.357067	0.357066657	protein_coding	NM_022355
ASHGV40028035	0.356996	0.356996488	protein_coding	NM_018000
ASHGV40034421	-0.35699	0.356989947	protein_coding	NM_016329
ASHGV40041091	-0.35671	0.356706564	protein_coding	NM_022090
ASHGV40050539	0.356704	0.356703688	protein_coding	NM_015542
ASHGV40056589	0.356687	0.356686998	protein_coding	NM_016074
ASHGV40038351	0.356662	0.356661997	protein_coding	NM_002199
ASHGV40023162	0.356583	0.356583087	protein_coding	NM_020232
ASHGV40015416	-0.35656	0.356557255	protein_coding	NM_182560
ASHGV40021080	-0.35632	0.356322891	protein_coding	NM_013337
ASHGV40025788	0.356187	0.356186992	protein_coding	NM_005178
ASHGV40035947	0.356152	0.356152484	protein_coding	NM_004393
ASHGV40050033	0.356145	0.356145062	protein_coding	NM_012154
ASHGV40013143	0.3561	0.356100097	protein_coding	NM_175854
ASHGV40056177	-0.35609	0.356094773	protein_coding	NM_004623
ASHGV40048556	0.356008	0.356008276	protein_coding	NM_018238
ASHGV40023435	-0.35573	0.355727683	protein_coding	NM_006785
ASHGV40024399	0.355675	0.35567503	protein_coding	NM_013376
ASHGV40024367	0.355435	0.355434539	protein_coding	NM_024907
ASHGV40043764	-0.35538	0.355383436	protein_coding	NM_198920
ASHGV40020237	0.355306	0.355305891	protein_coding	NM_025248
ASHGV40055885	-0.3553	0.355297318	protein_coding	NM_032211
ASHGV40014456	0.355178	0.355178128	protein_coding	ENST00000554943
ASHGV40000071	-0.35507	0.355065967	protein_coding	ENST00000334036
ASHGV40036945	-0.35494	0.354936535	protein_coding	NM_003032
ASHGV40026815	0.354914	0.35491448	protein_coding	NM_172369
ASHGV40023513	0.354775	0.354775255	protein_coding	NM_001093729
ASHGV40035631	0.354681	0.354680501	protein_coding	NM_001998
ASHGV40007589	-0.35464	0.354644706	protein_coding	NM_002519
ASHGV40013761	0.354497	0.354496561	protein_coding	NM_023011
ASHGV40024476	0.35443	0.354430139	protein_coding	NM_019108
ASHGV40029863	-0.3543	0.3543021	protein_coding	NM_001287584
ASHGV40044237	-0.35426	0.3542649	protein_coding	NM_020443
ASHGV40045686	-0.35425	0.354251231	protein_coding	NM_014388
ASHGV40055285	-0.3542	0.354204482	protein_coding	NM_032227
ASHGV40052190	-0.35407	0.354069546	protein_coding	NM_033051
ASHGV40050462	-0.35388	0.353875872	protein_coding	NM_015344
ASHGV40042537	0.353843	0.353842533	protein_coding	NM_000024
ASHGV40012084	-0.35382	0.353818122	protein_coding	NM_012240
ASHGV40040112	-0.35371	0.353712591	protein_coding	NM_144647
ASHGV40018225	0.353688	0.353687989	protein_coding	NM_001128

ASHGV40036024	0.353669	0.353668747	protein_coding	NM_001141947
ASHGV40030747	-0.35356	0.353555534	protein_coding	NM_002657
ASHGV40020395	-0.35336	0.353363348	protein_coding	NM_002533
ASHGV40042518	0.353346	0.353345844	protein_coding	NM_001112724
ASHGV40031746	-0.35331	0.353313136	protein_coding	NM_018683
ASHGV40025262	0.35326	0.353259698	protein_coding	NM_138501
ASHGV40030782	-0.35324	0.353241727	protein_coding	NM_018217
ASHGV40008062	0.3529	0.352900159	protein_coding	NM_032027
ASHGV40056305	-0.35287	0.35286842	protein_coding	NM_024533
ASHGV40019612	-0.35286	0.35285754	protein_coding	NM_018664
ASHGV40021020	-0.3528	0.35280014	protein_coding	NM_002917
ASHGV40006961	-0.35266	0.352660683	protein_coding	NM_003697
ASHGV40009950	0.352482	0.352482138	protein_coding	NM_005653
ASHGV40026131	0.352441	0.352441472	protein_coding	NM_006635
ASHGV40046055	0.352415	0.352415496	protein_coding	NM_002607
ASHGV40011724	-0.35239	0.352387769	protein_coding	NM_001232
ASHGV40009190	-0.35238	0.352377689	protein_coding	NM_014956
ASHGV40045355	-0.35232	0.352320223	protein_coding	NM_052943
ASHGV40011239	0.352163	0.352163464	protein_coding	NM_024102
ASHGV40002717	-0.3521	0.35210175	protein_coding	ENST00000603238
ASHGV40021804	0.352071	0.352071397	protein_coding	NM_002087
ASHGV40029463	-0.35189	0.351889663	protein_coding	NM_002101
ASHGV40024599	-0.35181	0.351813782	protein_coding	NM_006801
ASHGV40017823	0.351744	0.351744418	protein_coding	NM_006319
ASHGV40019791	-0.35157	0.351571206	protein_coding	NM_020360
ASHGV40042075	0.35156	0.351559643	protein_coding	NM_173060
ASHGV40018648	-0.35154	0.351543413	protein_coding	NM_001142290
ASHGV40037979	0.351507	0.351506639	protein_coding	NM_024579
ASHGV40001491	-0.35146	0.351462402	protein_coding	NM_001098728
ASHGV40018553	-0.35127	0.35126714	protein_coding	NM_006453
ASHGV40028930	-0.35119	0.351187195	protein_coding	NM_003038
ASHGV40007369	-0.35115	0.351149056	protein_coding	NM_004626
ASHGV40009713	0.351102	0.351102096	protein_coding	NM_001082972
ASHGV40047688	0.350909	0.350909433	protein_coding	NM_015060
ASHGV40051131	0.350893	0.350892774	protein_coding	ENST00000366457
ASHGV40030531	0.350854	0.350853681	protein_coding	NM_018848
ASHGV40047606	0.35072	0.350719639	protein_coding	NM_001005340
ASHGV40036419	-0.35065	0.350654053	protein_coding	ENST00000273390
ASHGV40006823	-0.35054	0.350539845	protein_coding	NM_001166692
ASHGV40039238	0.350512	0.350512331	protein_coding	NM_004757
ASHGV40008516	-0.35041	0.350412558	protein_coding	NM_015533
ASHGV40033781	0.350093	0.350093177	protein_coding	NM_001469
ASHGV40020068	-0.35	0.35000468	protein_coding	NM_080669
ASHGV40014810	0.349704	0.349703836	protein_coding	NM_203402
ASHGV40016920	-0.34969	0.349689317	protein_coding	NM_031301
ASHGV40050216	-0.34962	0.349622759	protein_coding	NM_001172575
ASHGV40020106	0.34956	0.349559816	protein_coding	NM_020772

ASHGV40044864	-0.34953	0.349530165	protein_coding	NM_005007
ASHGV40002650	-0.34946	0.349459004	protein_coding	ENST00000597657
ASHGV40031968	0.349425	0.349424551	protein_coding	NM_012469
ASHGV40012122	-0.34933	0.349331478	protein_coding	NM_144668
ASHGV40057554	0.349299	0.349299473	protein_coding	NM_020893
ASHGV40055226	0.349297	0.349297053	protein_coding	NM_030639
ASHGV40015219	0.349291	0.349290761	protein_coding	NM_001102564
ASHGV40054927	-0.34921	0.349206239	protein_coding	NM_003254
ASHGV40034982	-0.3492	0.349199924	protein_coding	NM_001134657
ASHGV40052562	0.349126	0.349126467	protein_coding	NM_021045
ASHGV40027622	-0.34912	0.349118024	protein_coding	NM_032283
ASHGV40007547	-0.34902	0.349016444	protein_coding	NM_032299
ASHGV40018275	0.348972	0.348972059	protein_coding	NM_001276345
ASHGV40057321	-0.34896	0.348962965	protein_coding	NM_182633
ASHGV40001350	-0.34893	0.348932177	protein_coding	NM_032476
ASHGV40009263	0.348773	0.348772792	protein_coding	NM_152715
ASHGV40008708	0.348676	0.348676248	protein_coding	NM_001619
ASHGV40049038	0.348649	0.348649056	protein_coding	NM_017634
ASHGV40053641	0.348479	0.348478582	protein_coding	NM_015160
ASHGV40042956	0.348391	0.348390672	protein_coding	NM_020408
ASHGV40039122	0.3483	0.348300171	protein_coding	NM_016067
ASHGV40017547	0.348255	0.348255411	protein_coding	NM_004380
ASHGV40049724	0.348203	0.348202626	protein_coding	NM_005655
ASHGV40034429	-0.34817	0.348167447	protein_coding	NM_018397
ASHGV40041145	-0.34808	0.348082821	protein_coding	NM_001129891
ASHGV40031739	0.34804	0.348039941	protein_coding	NM_001316
ASHGV40042444	-0.34791	0.347908193	protein_coding	NM_018931
ASHGV40001280	-0.34784	0.347837451	protein_coding	NM_001010875
ASHGV40051315	-0.34783	0.347829221	protein_coding	ENST00000507535
ASHGV40057778	-0.34777	0.347768194	protein_coding	NM_001997
ASHGV40012261	-0.34741	0.347406847	protein_coding	NM_015409
ASHGV40026783	0.347404	0.347403793	protein_coding	NM_018062
ASHGV40056094	-0.34728	0.347282566	protein_coding	NM_024658
ASHGV40023474	0.347081	0.347081112	protein_coding	NM_194449
ASHGV40025099	0.347037	0.347037103	protein_coding	NM_020533
ASHGV40018965	-0.34688	0.346878164	protein_coding	NM_001105247
ASHGV40046871	-0.34674	0.346737968	protein_coding	NM_001015072
ASHGV40004937	-0.34657	0.346569626	protein_coding	NM_198060
ASHGV40024623	0.346492	0.346492465	protein_coding	NM_000894
ASHGV40008544	0.346284	0.34628403	protein_coding	ENST00000278833
ASHGV40030774	-0.34625	0.346249952	protein_coding	NM_080476
ASHGV40017798	0.346237	0.346237301	protein_coding	NM_001055
ASHGV40043332	0.346072	0.346071956	protein_coding	NM_000593
ASHGV40008641	-0.34586	0.34586298	protein_coding	NM_031904
ASHGV40051905	-0.3458	0.345801461	protein_coding	NM_025211
ASHGV40036980	-0.34579	0.345785023	protein_coding	NM_174908
ASHGV40034707	-0.34577	0.345773344	protein_coding	NM_133496

ASHGV40057805	-0.34568	0.345676735	protein_coding	NM_001199206
ASHGV40009105	0.345652	0.345651845	protein_coding	NM_207645
ASHGV40019481	0.345608	0.345608436	protein_coding	NM_002661
ASHGV40055110	-0.34557	0.345570531	protein_coding	NM_001017962
ASHGV40018559	0.345331	0.345330612	protein_coding	NM_016243
ASHGV40013014	0.34531	0.345309651	protein_coding	NM_000705
ASHGV40050468	-0.34527	0.345265263	protein_coding	NM_000553
ASHGV40025246	-0.34512	0.345122167	protein_coding	NM_017721
ASHGV40024629	-0.34507	0.345071135	protein_coding	NM_031886
ASHGV40011664	0.344957	0.344956604	protein_coding	NM_006654
ASHGV40043778	-0.34482	0.344818329	protein_coding	NM_001080508
ASHGV40055448	0.344812	0.344811712	protein_coding	NM_001271560
ASHGV40015489	0.344778	0.344777518	protein_coding	NM_002719
ASHGV40036544	0.344671	0.344671004	protein_coding	NM_023943
ASHGV40018554	-0.34465	0.344649713	protein_coding	ENST00000598236
ASHGV40015498	0.344608	0.344607884	protein_coding	NM_018198
ASHGV40016966	0.344542	0.344541712	protein_coding	NM_005902
ASHGV40045222	0.344521	0.344521036	protein_coding	ENST00000356170
ASHGV40011517	-0.34452	0.344518242	protein_coding	NM_022465
ASHGV40020878	-0.34447	0.344471064	protein_coding	NM_173547
ASHGV40040539	0.344231	0.344231215	protein_coding	NM_198273
ASHGV40048435	-0.34386	0.343861714	protein_coding	NM_018396
ASHGV40019268	0.343829	0.343829256	protein_coding	NM_001185057
ASHGV40043862	0.343688	0.343687937	protein_coding	NM_014165
ASHGV40021055	0.34326	0.343260091	protein_coding	NM_018230
ASHGV40010108	0.343205	0.34320539	protein_coding	NM_001478
ASHGV40031613	-0.34318	0.343183006	protein_coding	NM_182811
ASHGV40024891	-0.34318	0.343176165	protein_coding	NM_024620
ASHGV40041569	0.343155	0.343155357	protein_coding	NM_018356
ASHGV40038273	-0.34296	0.342963216	protein_coding	NM_002129
ASHGV40013853	0.342887	0.342886574	protein_coding	NM_020239
ASHGV40020718	0.342876	0.34287626	protein_coding	NM_006572
ASHGV40025684	-0.34278	0.342784019	protein_coding	NM_016941
ASHGV40049675	0.342613	0.342613257	protein_coding	NM_033512
ASHGV40045243	0.342604	0.34260434	protein_coding	NM_001162529
ASHGV40040078	0.342459	0.342459156	protein_coding	NM_022130
ASHGV40038560	-0.34246	0.342457716	protein_coding	NM_030663
ASHGV40021630	0.34242	0.34241973	protein_coding	NM_024864
ASHGV40015669	-0.34231	0.342312624	protein_coding	NM_020371
ASHGV40007103	0.34226	0.342260484	protein_coding	NM_000738
ASHGV40007431	-0.34214	0.342135176	protein_coding	NM_152723
ASHGV40049056	0.342012	0.342011583	protein_coding	NM_018246
ASHGV40016724	0.341998	0.341997795	protein_coding	NM_001286414
ASHGV40008378	0.341871	0.341870633	protein_coding	NM_024113
ASHGV40010418	-0.34185	0.341852782	protein_coding	NM_018351
ASHGV40008065	-0.34179	0.341794682	protein_coding	NM_003754
ASHGV40009343	-0.34179	0.341793938	protein_coding	ENST00000263579

ASHGV40055126	0.341744	0.341743626	protein_coding	NM_003022
ASHGV40043206	0.341742	0.341741859	protein_coding	NM_030876
ASHGV40026116	-0.34173	0.341726777	protein_coding	NM_016233
ASHGV40048049	0.341432	0.34143219	protein_coding	NM_198467
ASHGV40023823	-0.34138	0.341380404	protein_coding	NM_001419
ASHGV40051378	0.341358	0.341358493	protein_coding	NM_024736
ASHGV40003203	0.341356	0.341355833	protein_coding	NM_001277308
ASHGV40044825	0.341344	0.341344297	protein_coding	NM_021959
ASHGV40020966	0.341188	0.341187823	protein_coding	NM_014740
ASHGV40040107	-0.34115	0.341150898	protein_coding	NM_000949
ASHGV40046611	0.340974	0.340973817	protein_coding	ENST00000593865
ASHGV40002252	-0.34096	0.340960481	protein_coding	ENST00000562949
ASHGV40029101	-0.34084	0.340835154	protein_coding	NM_001370
ASHGV40057105	0.340768	0.340768359	protein_coding	NM_033281
ASHGV40015766	-0.34073	0.340725405	protein_coding	NM_139265
ASHGV40011544	-0.3406	0.340597377	protein_coding	NM_007224
ASHGV40000164	0.340551	0.340550988	protein_coding	NM_138346
ASHGV40021420	-0.34051	0.340505817	protein_coding	NM_018242
ASHGV40008656	0.340505	0.34050504	protein_coding	NM_006388
ASHGV40019412	0.340364	0.340363521	protein_coding	NM_153688
ASHGV40049586	-0.3403	0.34030327	protein_coding	NM_152418
ASHGV40046836	0.340271	0.340271121	protein_coding	NM_032164
ASHGV40056653	0.340219	0.340218578	protein_coding	NM_031288
ASHGV40025555	0.340205	0.340204527	protein_coding	NM_020895
ASHGV40023160	-0.34012	0.340122817	protein_coding	NM_006553
ASHGV40009927	0.340105	0.340105404	protein_coding	NM_006082
ASHGV40015092	0.340085	0.340085355	protein_coding	NM_020663
ASHGV40008659	0.339931	0.33993116	protein_coding	NM_025128
ASHGV40041088	-0.33964	0.339635386	protein_coding	NM_024565
ASHGV40008520	-0.33955	0.339551373	protein_coding	NM_145017
ASHGV40002200	-0.33941	0.33940851	protein_coding	ENST00000560347
ASHGV40025388	-0.33928	0.339282262	protein_coding	ENST00000555938
ASHGV40045922	-0.33918	0.339183605	protein_coding	NM_003057
ASHGV40009006	0.339174	0.339173702	protein_coding	NM_001286067
ASHGV40047422	-0.33915	0.33915329	protein_coding	NM_015949
ASHGV40028004	-0.33909	0.339087909	protein_coding	NM_152519
ASHGV40038212	0.338967	0.338966601	protein_coding	NM_000909
ASHGV40029040	-0.33896	0.338962513	protein_coding	NM_032118
ASHGV40011322	-0.33888	0.338880182	protein_coding	NM_017744
ASHGV40026133	-0.33861	0.338610494	protein_coding	NM_213598
ASHGV40054629	-0.33847	0.338470616	protein_coding	NM_004042
ASHGV40025785	0.338434	0.338433698	protein_coding	NM_020219
ASHGV40015676	0.338341	0.338341227	protein_coding	NM_018648
ASHGV40000089	-0.33834	0.338337783	protein_coding	ENST00000342005
ASHGV40016005	-0.33831	0.338310724	protein_coding	NM_001163692
ASHGV40042421	-0.33827	0.338271606	protein_coding	NM_018910
ASHGV40055554	-0.33826	0.338261156	protein_coding	NM_032772

ASHGV40052029	-0.33816	0.338164122	protein_coding	NM_000264
ASHGV40016894	0.338076	0.338076422	protein_coding	NM_018948
ASHGV40029316	-0.33804	0.338043975	protein_coding	NM_018715
ASHGV40056247	-0.33787	0.337870124	protein_coding	NM_001112808
ASHGV40050782	-0.33782	0.33782133	protein_coding	NM_052958
ASHGV40050371	0.337644	0.337644439	protein_coding	NM_015178
ASHGV40041940	0.337532	0.337531551	protein_coding	NM_001882
ASHGV40056330	0.337513	0.337512783	protein_coding	NM_015670
ASHGV40051122	0.337505	0.337504746	protein_coding	NM_004421
ASHGV40006056	0.337465	0.337464748	protein_coding	NM_001010917
ASHGV40044771	0.337316	0.337316362	protein_coding	NM_001076781
ASHGV40034292	-0.33732	0.337316162	protein_coding	NM_001206943
ASHGV40025980	-0.33728	0.337283139	protein_coding	uc001ayv.2
ASHGV40011190	0.337259	0.337259221	protein_coding	NM_020782
ASHGV40032900	-0.33725	0.337248752	protein_coding	NM_013313
ASHGV40001262	-0.33725	0.337248697	protein_coding	NM_001017928
ASHGV40026137	-0.33722	0.337220018	protein_coding	NM_152909
ASHGV40000124	-0.33715	0.337151448	protein_coding	NM_006615
ASHGV40050753	-0.33703	0.337031803	protein_coding	NM_014637
ASHGV40043939	-0.33685	0.336845595	protein_coding	NM_001145128
ASHGV40024655	-0.33678	0.336782811	protein_coding	NM_012346
ASHGV40039838	-0.33673	0.336734818	protein_coding	NM_024830
ASHGV40008393	0.336682	0.3366816	protein_coding	NM_002843
ASHGV40043659	0.336565	0.336565288	protein_coding	NM_152688
ASHGV40024343	0.33656	0.336559511	protein_coding	NM_004647
ASHGV40041934	0.336429	0.336429186	protein_coding	NM_004905
ASHGV40041862	-0.33637	0.336367008	protein_coding	NM_001042490
ASHGV40009614	-0.33625	0.336250681	protein_coding	NM_005039
ASHGV40048479	-0.3362	0.336197372	protein_coding	NM_002402
ASHGV40021397	0.336192	0.336192459	protein_coding	NM_144775
ASHGV40027926	0.33617	0.336170184	protein_coding	NM_001044385
ASHGV40012256	0.336169	0.336169029	protein_coding	NM_004592
ASHGV40017567	0.336084	0.336084143	protein_coding	NM_006469
ASHGV40013189	0.335621	0.335620982	protein_coding	NM_004795
ASHGV40045520	0.335288	0.335287746	protein_coding	NM_032194
ASHGV40027119	0.335223	0.335223445	protein_coding	NM_005735
ASHGV40036507	0.335158	0.335158253	protein_coding	NM_014049
ASHGV40024751	0.335131	0.335131491	protein_coding	NM_032584
ASHGV40032238	-0.33504	0.335044299	protein_coding	NM_012130
ASHGV40007904	0.334921	0.334920522	protein_coding	NM_198075
ASHGV40043180	0.334639	0.334639101	protein_coding	NM_019110
ASHGV40053974	0.334619	0.33461919	protein_coding	NM_033056
ASHGV40038366	0.334582	0.334581747	protein_coding	ENST00000296775
ASHGV40014793	-0.33458	0.334580901	protein_coding	NM_024328
ASHGV40008330	-0.33456	0.33455816	protein_coding	NM_018259
ASHGV40011568	0.334544	0.334543978	protein_coding	NM_005726
ASHGV40038794	0.334525	0.334524506	protein_coding	NM_023015

ASHGV40018249	-0.33447	0.334473406	protein_coding	NM_001145667
ASHGV40012494	0.334464	0.334463506	protein_coding	NM_005830
ASHGV40010852	0.334377	0.334376918	protein_coding	NM_175066
ASHGV40006937	0.334285	0.334285351	protein_coding	NM_014342
ASHGV40008000	-0.33412	0.334124752	protein_coding	NM_001004137
ASHGV40046848	-0.33403	0.334034336	protein_coding	NM_032924
ASHGV40011128	0.334005	0.334004501	protein_coding	NM_019012
ASHGV40031834	-0.33398	0.333977567	protein_coding	NM_002591
ASHGV40018228	0.333947	0.333947146	protein_coding	NM_001201552
ASHGV40033349	-0.33389	0.333892875	protein_coding	NM_001001794
ASHGV40041680	0.333803	0.333803364	protein_coding	NM_001297548
ASHGV40033263	-0.33377	0.333768403	protein_coding	NM_001009880
ASHGV40020082	-0.33357	0.333569502	protein_coding	NM_031934
ASHGV40048629	-0.33335	0.333354873	protein_coding	NM_001303481
ASHGV40053329	0.333201	0.333201199	protein_coding	NM_144488
ASHGV40057186	0.332954	0.332953613	protein_coding	NM_024639
ASHGV40026472	0.332916	0.3329158	protein_coding	NM_001013663
ASHGV40018966	-0.33285	0.332847836	protein_coding	NM_001042454
ASHGV40000775	-0.33276	0.332763982	protein_coding	NM_001014979
ASHGV40044735	-0.3326	0.332595004	protein_coding	NM_005321
ASHGV40020293	-0.33256	0.332555029	protein_coding	NM_015515
ASHGV40057623	-0.33253	0.332531785	protein_coding	NM_020932
ASHGV40012482	0.332527	0.332526939	protein_coding	NM_005780
ASHGV40047513	0.332372	0.332372473	protein_coding	NM_019005
ASHGV40014991	0.332284	0.332283749	protein_coding	NM_016039
ASHGV40052309	0.331916	0.33191601	protein_coding	NM_001144877
ASHGV40050984	-0.33182	0.3318248	protein_coding	NM_018407
ASHGV40006101	-0.33174	0.331743294	protein_coding	NM_030971
ASHGV40013866	-0.33163	0.331634371	protein_coding	NM_006405
ASHGV40034329	0.331594	0.331594093	protein_coding	NM_001199691
ASHGV40032778	-0.33157	0.331570229	protein_coding	NM_030582
ASHGV40060823	0.331451	0.331451294	protein_coding	uc001xtc.1
ASHGV40001630	0.331377	0.331377371	protein_coding	ENST00000514917
ASHGV40052781	0.331339	0.331339147	protein_coding	NM_000155
ASHGV40045746	-0.33132	0.331316836	protein_coding	NM_021243
ASHGV40007969	0.331228	0.33122842	protein_coding	NM_005706
ASHGV40025432	0.331097	0.3310968	protein_coding	NM_015378
ASHGV40025830	-0.33109	0.33109078	protein_coding	NM_006247
ASHGV40040586	0.331024	0.331024422	protein_coding	NM_001270
ASHGV40034277	-0.33095	0.330952129	protein_coding	NM_147129
ASHGV40015332	0.330929	0.330928886	protein_coding	ENST00000163416
ASHGV40035876	-0.33087	0.330867288	protein_coding	NM_006991
ASHGV40042690	-0.33085	0.330851826	protein_coding	NM_017785
ASHGV40037176	0.330765	0.33076519	protein_coding	NM_016930
ASHGV40055519	-0.33073	0.330733833	protein_coding	NM_001017980
ASHGV40027975	-0.33061	0.330613015	protein_coding	NM_001093730
ASHGV40030405	-0.33058	0.330577629	protein_coding	NM_032515

ASHGV40021205	0.330552	0.330552226	protein_coding	NM_000018
ASHGV40055932	0.330549	0.330548813	protein_coding	NM_012215
ASHGV40015438	0.330513	0.330512999	protein_coding	ENST00000377658
ASHGV40028895	-0.33047	0.330465976	protein_coding	NM_002908
ASHGV40040881	-0.33041	0.330413582	protein_coding	NM_194296
ASHGV40027761	0.330331	0.330330741	protein_coding	NM_152275
ASHGV40042533	0.330241	0.330240628	protein_coding	NM_030793
ASHGV40022141	-0.33015	0.330152722	protein_coding	NM_001270422
ASHGV40030618	-0.32989	0.329886083	protein_coding	NM_003579
ASHGV40001955	-0.32986	0.329857208	protein_coding	ENST00000540061
ASHGV40044802	-0.32985	0.329847863	protein_coding	ENST00000377171
ASHGV40037230	-0.32983	0.329826683	protein_coding	NM_005114
ASHGV40041068	0.329826	0.329825647	protein_coding	NM_014666
ASHGV40020649	-0.32974	0.329738829	protein_coding	NM_022070
ASHGV40052426	0.329599	0.329599347	protein_coding	NM_003591
ASHGV40014735	-0.32956	0.329557819	protein_coding	NM_019089
ASHGV40027373	0.329542	0.329542318	protein_coding	uc002tnn.1
ASHGV40020694	-0.32932	0.32931941	protein_coding	NM_001098426
ASHGV40050424	0.329119	0.329118985	protein_coding	NM_001979
ASHGV40015397	0.328831	0.328831155	protein_coding	NM_003384
ASHGV40042800	-0.3288	0.328800587	protein_coding	NM_003052
ASHGV40039541	-0.32874	0.328741604	protein_coding	uc021xtm.1
ASHGV40025417	0.32864	0.3286396	protein_coding	NM_133473
ASHGV40055314	0.328568	0.328568011	protein_coding	NM_019045
ASHGV40033835	0.328529	0.328528849	protein_coding	NM_007172
ASHGV40056191	-0.32844	0.328443117	protein_coding	ENST00000560278
ASHGV40038636	-0.32841	0.328412196	protein_coding	NM_001080522
ASHGV40033233	0.328371	0.32837141	protein_coding	uc010orq.2
ASHGV40025872	0.328356	0.328355589	protein_coding	NM_017457
ASHGV40023981	-0.32822	0.328222145	protein_coding	NM_001145028
ASHGV40003340	-0.32821	0.328213203	protein_coding	NM_153708
ASHGV40022020	-0.32819	0.328191026	protein_coding	NM_001004707
ASHGV40021095	0.328165	0.32816459	protein_coding	NM_002945
ASHGV40038966	0.328128	0.32812764	protein_coding	NM_015236
ASHGV40031369	-0.3281	0.328098472	protein_coding	NM_033067
ASHGV40024228	0.328074	0.328073983	protein_coding	NM_007033
ASHGV40032261	-0.32794	0.327940463	protein_coding	NM_001136154
ASHGV40013993	0.327924	0.327923863	protein_coding	NM_001079537
ASHGV40052793	0.327876	0.327876065	protein_coding	ENST00000361226
ASHGV40022268	0.327731	0.327730561	protein_coding	NM_031844
ASHGV40044811	-0.3276	0.32760203	protein_coding	NM_030883
ASHGV40053319	0.327407	0.327406744	protein_coding	NM_001012994
ASHGV40024594	-0.32732	0.327318084	protein_coding	NM_014959
ASHGV40042808	0.327263	0.327263114	protein_coding	NM_030769
ASHGV40003256	0.327166	0.32716643	protein_coding	NM_003717
ASHGV40035357	-0.32706	0.327063162	protein_coding	NM_080652
ASHGV40017747	-0.32693	0.326933887	protein_coding	NM_033266

ASHGV40021390	-0.32676	0.326757981	protein_coding	NM_024052
ASHGV40034549	-0.32667	0.32666624	protein_coding	NM_001126128
ASHGV40050961	0.326658	0.326658468	protein_coding	NM_017864
ASHGV40038824	0.326544	0.326544009	protein_coding	NM_018126
ASHGV40019968	-0.32647	0.326473896	protein_coding	NM_004618
ASHGV40018688	0.32643	0.326430451	protein_coding	NM_024997
ASHGV40044337	0.326428	0.326427688	protein_coding	NM_004767
ASHGV40051368	-0.32638	0.326376375	protein_coding	NM_001301772
ASHGV40020902	0.326114	0.326113712	protein_coding	NM_006456
ASHGV40040414	-0.32585	0.325854797	protein_coding	NM_032367
ASHGV40048033	-0.32573	0.325729698	protein_coding	NM_001291831
ASHGV40003129	-0.32568	0.325681979	protein_coding	NM_001145250
ASHGV40011689	0.32567	0.325670246	protein_coding	NM_022771
ASHGV40006077	0.325451	0.32545055	protein_coding	NM_006516
ASHGV40005334	-0.32528	0.32527971	protein_coding	NM_003675
ASHGV40007057	0.325249	0.325249101	protein_coding	NM_004200
ASHGV40026496	-0.32524	0.32523756	protein_coding	NM_207334
ASHGV40001177	-0.32522	0.325220063	protein_coding	ENST00000454671
ASHGV40028634	-0.32516	0.325157956	protein_coding	NM_017877
ASHGV40008023	-0.32513	0.325134835	protein_coding	NM_001005180
ASHGV40014892	0.324655	0.32465491	protein_coding	NM_003136
ASHGV40056234	0.324631	0.324630578	protein_coding	NM_006711
ASHGV40045602	-0.32436	0.32436162	protein_coding	NM_014034
ASHGV40054089	-0.32435	0.324350767	protein_coding	NM_031206
ASHGV40015894	-0.32432	0.324318955	protein_coding	NM_004580
ASHGV40002363	0.324245	0.324245213	protein_coding	ENST00000570054
ASHGV40055249	-0.32412	0.324121344	protein_coding	NM_017416
ASHGV40016006	-0.32402	0.324015705	protein_coding	NM_005707
ASHGV40031189	-0.324	0.323999624	protein_coding	NM_030931
ASHGV40053316	0.323878	0.323877824	protein_coding	NM_032303
ASHGV40028458	0.323854	0.323854192	protein_coding	NM_002166
ASHGV40027208	-0.32382	0.323815799	protein_coding	ENST00000595531
ASHGV40020018	0.323812	0.323811534	protein_coding	NM_015276
ASHGV40043363	0.323762	0.323761877	protein_coding	ENST00000594414
ASHGV40051381	-0.32366	0.323658307	protein_coding	NM_032862
ASHGV40000076	-0.32335	0.323346776	protein_coding	ENST00000334973
ASHGV40005384	0.323201	0.323201061	protein_coding	NM_001195626
ASHGV40050736	0.323085	0.323085181	protein_coding	NM_152758
ASHGV40056488	-0.32306	0.323055559	protein_coding	NM_206833
ASHGV40048646	-0.32284	0.32284218	protein_coding	NM_001099220
ASHGV40051890	0.322775	0.322774903	protein_coding	NM_005077
ASHGV40027725	-0.3226	0.322604762	protein_coding	ENST00000595354
ASHGV40021229	-0.32251	0.322514063	protein_coding	NM_004870
ASHGV40000741	0.322471	0.322470922	protein_coding	NM_033083
ASHGV40043492	-0.32233	0.322325019	protein_coding	NM_018141
ASHGV40030258	-0.32229	0.32229476	protein_coding	NM_152383
ASHGV40047266	-0.32229	0.322293526	protein_coding	NM_001278507

ASHGV40049142	-0.32226	0.322262873	protein_coding	NM_001004687
ASHGV40011446	0.322147	0.32214663	protein_coding	NM_001128911
ASHGV40055355	0.322138	0.322138031	protein_coding	NM_001167
ASHGV40003085	0.321987	0.321986809	protein_coding	NM_001018024
ASHGV40020401	-0.32193	0.321928975	protein_coding	NM_032376
ASHGV40021438	-0.32187	0.321865143	protein_coding	NM_001190790
ASHGV40054741	0.321832	0.321831856	protein_coding	NM_006240
ASHGV40023985	-0.32174	0.321743752	protein_coding	NM_002730
ASHGV40011684	-0.32168	0.321675923	protein_coding	NM_031435
ASHGV40040706	0.32163	0.321629602	protein_coding	NM_173666
ASHGV40011017	-0.32162	0.321623776	protein_coding	NM_006492
ASHGV40010708	-0.32159	0.321588338	protein_coding	NM_019034
ASHGV40021716	-0.3214	0.321403391	protein_coding	NM_001146041
ASHGV40035874	-0.32137	0.321371857	protein_coding	NM_018651
ASHGV40005561	-0.32133	0.321331117	protein_coding	ENST00000431662
ASHGV40007187	-0.32128	0.321284389	protein_coding	NM_032325
ASHGV40014872	-0.32127	0.321267457	protein_coding	NM_025152
ASHGV40010034	-0.32105	0.321049356	protein_coding	NM_144594
ASHGV40007204	0.320972	0.320972072	protein_coding	NM_016050
ASHGV40015907	-0.32096	0.320963772	protein_coding	NM_018365
ASHGV40026669	-0.32091	0.32091121	protein_coding	NM_022065
ASHGV40016638	0.320698	0.320698092	protein_coding	NM_173611
ASHGV40054162	0.320677	0.320677445	protein_coding	uc004ecr.2
ASHGV40015564	0.320425	0.320425324	protein_coding	NM_145167
ASHGV40048551	0.320405	0.320404585	protein_coding	NM_052853
ASHGV40011746	-0.32038	0.320383441	protein_coding	NM_173591
ASHGV40000173	-0.32033	0.320328744	protein_coding	NM_001076680
ASHGV40050135	0.320273	0.320272954	protein_coding	ENST00000292510
ASHGV40038706	-0.32014	0.320139138	protein_coding	NM_015607
ASHGV40049773	-0.32013	0.320134565	protein_coding	NM_032869
ASHGV40053977	-0.32009	0.320092257	protein_coding	NM_001037735
ASHGV40042424	0.319887	0.319886749	protein_coding	NM_031859
ASHGV40053671	-0.31977	0.319771269	protein_coding	NM_016219
ASHGV40017481	-0.31946	0.319458189	protein_coding	NM_172168
ASHGV40007886	-0.31924	0.319242942	protein_coding	NM_053280
ASHGV40020128	0.31924	0.319239574	protein_coding	NM_015986
ASHGV40014683	0.319139	0.319139161	protein_coding	NM_006589
ASHGV40031682	-0.31911	0.319107431	protein_coding	NM_006103
ASHGV40039276	-0.31899	0.318985774	protein_coding	ENST00000368376
ASHGV40032817	-0.31896	0.318962508	protein_coding	NM_031890
ASHGV40044199	-0.31893	0.318934139	protein_coding	ENST00000593583
ASHGV40051999	0.318892	0.318891511	protein_coding	NM_005014
ASHGV40025066	-0.31873	0.318727237	protein_coding	NM_004558
ASHGV40008466	-0.31828	0.318275764	protein_coding	NM_198947
ASHGV40014722	0.3182	0.318199615	protein_coding	NM_001641
ASHGV40011943	0.318032	0.318031829	protein_coding	NM_007062
ASHGV40018775	0.317959	0.317958686	protein_coding	NM_153208

ASHGV40040832	0.317859	0.317858638	protein_coding	NM_138610
ASHGV40008967	0.317841	0.317840994	protein_coding	NM_022159
ASHGV40016553	0.317768	0.317768179	protein_coding	NM_033223
ASHGV40003112	0.317758	0.317758336	protein_coding	NM_001099285
ASHGV40043128	-0.31756	0.31755712	protein_coding	NM_005074
ASHGV40026903	-0.31756	0.317556258	protein_coding	NM_022173
ASHGV40003211	0.317511	0.317510908	protein_coding	NM_001282493
ASHGV40038371	-0.31736	0.317360829	protein_coding	NM_014476
ASHGV40019800	0.31721	0.317209927	protein_coding	NM_004860
ASHGV40053680	-0.31714	0.317136905	protein_coding	NM_001004353
ASHGV40024904	0.31712	0.317119793	protein_coding	NM_032792
ASHGV40025285	-0.31709	0.317093871	protein_coding	NM_013938
ASHGV40008726	-0.31708	0.317077109	protein_coding	ENST00000420661
ASHGV40029673	0.31694	0.316940074	protein_coding	NM_173642
ASHGV40007429	-0.31685	0.316852414	protein_coding	NM_018087
ASHGV40027845	-0.31655	0.316551799	protein_coding	NM_001142645
ASHGV40018255	0.31655	0.316550082	protein_coding	NM_016456
ASHGV40051198	-0.31649	0.316491109	protein_coding	NM_025195
ASHGV40041162	-0.31647	0.3164723	protein_coding	NM_152277
ASHGV40032698	0.3164	0.316399553	protein_coding	NM_002462
ASHGV40057534	0.316353	0.316353077	protein_coding	NM_201453
ASHGV40017970	0.316245	0.316245191	protein_coding	NM_013263
ASHGV40010997	0.316243	0.316243432	protein_coding	NM_001734
ASHGV40033624	0.316116	0.31611566	protein_coding	NM_004147
ASHGV40012232	-0.31606	0.316063253	protein_coding	NM_004764
ASHGV40016788	0.315716	0.315715899	protein_coding	NM_020234
ASHGV40045427	0.315651	0.315650725	protein_coding	NM_005604
ASHGV40042442	-0.31562	0.315618484	protein_coding	NM_019119
ASHGV40040808	-0.3156	0.315596187	protein_coding	NM_002155
ASHGV40024210	-0.31557	0.315573292	protein_coding	NM_020856
ASHGV40005751	0.315554	0.315553633	protein_coding	NM_012339
ASHGV40048229	0.315404	0.315403887	protein_coding	NM_173564
ASHGV40010554	0.315391	0.315390887	protein_coding	NM_014325
ASHGV40040118	0.315331	0.315330689	protein_coding	NM_001085411
ASHGV40017672	-0.3153	0.315304238	protein_coding	NM_022166
ASHGV40007788	-0.31521	0.315208259	protein_coding	NM_001308027
ASHGV40017753	-0.31514	0.315143218	protein_coding	NM_001006634
ASHGV40011573	-0.31509	0.315088661	protein_coding	NM_033276
ASHGV40046781	-0.31505	0.315052058	protein_coding	NM_017654
ASHGV40016461	0.314853	0.314852532	protein_coding	NM_003090
ASHGV40036857	-0.31485	0.314845728	protein_coding	NM_004301
ASHGV40031257	0.314842	0.314841873	protein_coding	NM_021873
ASHGV40006808	0.314783	0.314782955	protein_coding	NM_000280
ASHGV40038367	0.314626	0.31462565	protein_coding	NM_018409
ASHGV40026802	-0.31457	0.3145704	protein_coding	NM_022893
ASHGV40024770	0.314556	0.314555901	protein_coding	NM_013342
ASHGV40025270	0.314484	0.314484477	protein_coding	NM_024758

ASHGV40043958	0.314461	0.314461078	protein_coding	NM_002912
ASHGV40023948	0.314443	0.314442613	protein_coding	NM_001136196
ASHGV40035304	-0.31436	0.314355311	protein_coding	NM_181426
ASHGV40020197	0.314276	0.314276157	protein_coding	NM_002985
ASHGV40049611	0.314195	0.314195019	protein_coding	NM_018710
ASHGV40009621	0.314185	0.314184818	protein_coding	NM_002336
ASHGV40018435	-0.31415	0.31415078	protein_coding	NM_005187
ASHGV40010173	-0.31411	0.314110301	protein_coding	NM_002076
ASHGV40038084	0.314074	0.314074102	protein_coding	NM_001168235
ASHGV40003313	-0.31407	0.314071907	protein_coding	NM_030975
ASHGV40003174	-0.31406	0.314055931	protein_coding	NM_001203261
ASHGV40005761	0.314019	0.314018995	protein_coding	NM_014431
ASHGV40014624	0.313973	0.313973043	protein_coding	NM_015316
ASHGV40014995	-0.31397	0.313972949	protein_coding	NM_001099652
ASHGV40039196	0.313943	0.313943413	protein_coding	NM_015143
ASHGV40010215	0.3139	0.313900333	protein_coding	NM_017440
ASHGV40010512	-0.31387	0.313871871	protein_coding	NM_001034173
ASHGV40043797	-0.31369	0.313693862	protein_coding	NM_016083
ASHGV40049871	-0.31369	0.313690939	protein_coding	NM_024295
ASHGV40050943	0.31365	0.313649866	protein_coding	NM_145269
ASHGV40024672	0.313581	0.313581223	protein_coding	NM_032298
ASHGV40055419	0.313553	0.313552559	protein_coding	NM_001017435
ASHGV40005040	0.313511	0.31351125	protein_coding	NM_015892
ASHGV40027433	0.313434	0.313434148	protein_coding	NM_001077637
ASHGV40019497	-0.31341	0.31340645	protein_coding	NM_178452
ASHGV40047567	0.313313	0.313312958	protein_coding	NM_058176
ASHGV40009147	0.313091	0.31309135	protein_coding	NM_017868
ASHGV40042470	-0.31295	0.312947835	protein_coding	NM_002588
ASHGV40034503	0.312825	0.312825127	protein_coding	NM_198859
ASHGV40022208	-0.31278	0.312781791	protein_coding	NM_014603
ASHGV40047315	0.31266	0.312659584	protein_coding	NM_001658
ASHGV40055344	0.312583	0.312583408	protein_coding	NM_014060
ASHGV40043350	0.312576	0.312576277	protein_coding	NM_022553
ASHGV40043536	-0.31254	0.312536355	protein_coding	NM_182539
ASHGV40035358	-0.31251	0.312507763	protein_coding	NM_139248
ASHGV40017470	0.312479	0.312479288	protein_coding	NM_002513
ASHGV40044968	0.312398	0.312397684	protein_coding	NM_139012
ASHGV40047249	-0.31236	0.312361458	protein_coding	NM_001001658
ASHGV40011975	-0.31233	0.312333489	protein_coding	NM_032829
ASHGV40033398	-0.31231	0.312309396	protein_coding	NM_017414
ASHGV40047230	-0.31231	0.312307794	protein_coding	NM_001008270
ASHGV40007388	0.312297	0.312296644	protein_coding	NM_001293
ASHGV40019766	0.312146	0.312145928	protein_coding	NM_177550
ASHGV40038330	0.312055	0.312054763	protein_coding	NM_001921
ASHGV40037929	-0.31192	0.311920753	protein_coding	NM_002358
ASHGV40054822	0.311832	0.311832235	protein_coding	NM_001170331
ASHGV40053618	-0.31165	0.311647933	protein_coding	NM_019118

ASHGV40056729	-0.31162	0.311622964	protein_coding	NM_144580
ASHGV40055654	-0.31152	0.311521837	protein_coding	NM_004676
ASHGV40022604	0.311504	0.311503574	protein_coding	NM_015461
ASHGV40033199	0.311388	0.311387924	protein_coding	NM_138481
ASHGV40054763	-0.31132	0.311324152	protein_coding	NM_006406
ASHGV40051714	-0.31119	0.311194275	protein_coding	NM_001004487
ASHGV40007114	-0.31117	0.311169695	protein_coding	NM_001144936
ASHGV40008636	0.31107	0.311069612	protein_coding	NM_006779
ASHGV40037150	-0.3108	0.310796399	protein_coding	NM_001161527
ASHGV40007558	0.310783	0.310783402	protein_coding	NM_145716
ASHGV40023172	0.31072	0.310719703	protein_coding	NM_181481
ASHGV40022736	-0.31059	0.310593896	protein_coding	NM_001085474
ASHGV40041824	0.310526	0.310526311	protein_coding	NM_001113561
ASHGV40013626	0.310517	0.310517481	protein_coding	NM_003291
ASHGV40017424	-0.31029	0.310290687	protein_coding	NM_001031737
ASHGV40021934	-0.31022	0.31022086	protein_coding	NM_022469
ASHGV40019964	0.310195	0.310194774	protein_coding	NM_145691
ASHGV40039737	0.310178	0.310177783	protein_coding	NM_001300767
ASHGV40045081	0.310037	0.310036634	protein_coding	NM_014628
ASHGV40043998	0.310027	0.310027474	protein_coding	NM_145062
ASHGV40008941	0.310007	0.31000732	protein_coding	NM_015885
ASHGV40022384	0.310005	0.310004972	protein_coding	NM_022158
ASHGV40053462	0.309923	0.309922877	protein_coding	NM_014007
ASHGV40028645	-0.30983	0.309829869	protein_coding	NM_032604
ASHGV40022324	-0.30983	0.309826196	protein_coding	NM_017950
ASHGV40019763	-0.30943	0.309432234	protein_coding	NM_016060
ASHGV40053453	-0.30936	0.309363494	protein_coding	NM_033446
ASHGV40053486	0.309263	0.309262627	protein_coding	NM_024112
ASHGV40010560	-0.30925	0.309252023	protein_coding	NM_001001655
ASHGV40052743	-0.30922	0.309218089	protein_coding	NM_002197
ASHGV40057069	-0.30904	0.309040119	protein_coding	NM_001185149
ASHGV40055464	-0.30903	0.309031306	protein_coding	NM_138702
ASHGV40007226	-0.30898	0.308982805	protein_coding	NM_206997
ASHGV40005930	-0.30881	0.308814059	protein_coding	uc021pvr.1
ASHGV40032903	-0.30866	0.308658586	protein_coding	NM_014634
ASHGV40037757	-0.30861	0.308614549	protein_coding	uc003hsl.2
ASHGV40047776	-0.30849	0.308488186	protein_coding	NM_001129
ASHGV40019178	-0.30834	0.308338684	protein_coding	NM_002990
ASHGV40035626	0.30833	0.308330333	protein_coding	NM_001162499
ASHGV40018869	-0.30833	0.308327238	protein_coding	NM_015202
ASHGV40018205	0.308303	0.308302569	protein_coding	NM_006927
ASHGV40000160	-0.30828	0.308280957	protein_coding	NM_001517
ASHGV40021160	-0.30819	0.308188543	protein_coding	NM_001145536
ASHGV40029747	-0.30813	0.308127926	protein_coding	NM_144711
ASHGV40044747	0.308077	0.308077229	protein_coding	NM_006995
ASHGV40017632	0.308028	0.308027909	protein_coding	NM_001130007
ASHGV40008716	-0.30793	0.307929434	protein_coding	NM_003952

ASHGV40044549	0.307863	0.307862884	protein_coding	NM_014359
ASHGV40052001	0.307785	0.307785051	protein_coding	NM_001393
ASHGV40056476	-0.30777	0.307774874	protein_coding	ENST00000599630
ASHGV40047985	0.307757	0.307756522	protein_coding	NM_022479
ASHGV40039606	-0.30762	0.307619345	protein_coding	NM_022367
ASHGV40015781	0.307407	0.307407057	protein_coding	NM_173500
ASHGV40036001	0.307364	0.307363696	protein_coding	NM_001124767
ASHGV40009919	-0.30723	0.30722534	protein_coding	NM_003482
ASHGV40034827	-0.30722	0.307221348	protein_coding	NM_022757
ASHGV40050896	-0.30695	0.306946494	protein_coding	NM_198584
ASHGV40007230	0.306932	0.306932452	protein_coding	NM_005851
ASHGV40032897	0.306922	0.306922429	protein_coding	ENST00000609362
ASHGV40028638	0.306784	0.306783998	protein_coding	NM_012326
ASHGV40057057	-0.30678	0.306776778	protein_coding	NM_004564
ASHGV40025829	-0.30676	0.306756314	protein_coding	NM_152795
ASHGV40048200	0.306674	0.306674105	protein_coding	NM_006693
ASHGV40028792	0.306614	0.306614435	protein_coding	NM_172069
ASHGV40049064	0.306607	0.30660662	protein_coding	NM_018660
ASHGV40040946	0.306533	0.306532719	protein_coding	NM_030799
ASHGV40033241	0.306504	0.306504094	protein_coding	NM_022785
ASHGV40026890	-0.30634	0.306339704	protein_coding	NM_001244710
ASHGV40053485	0.306286	0.306286234	protein_coding	NM_005564
ASHGV40036516	0.306165	0.306164836	protein_coding	NM_052989
ASHGV40005739	-0.30613	0.306132316	protein_coding	NM_004728
ASHGV40039354	-0.30609	0.30609399	protein_coding	NM_001105203
ASHGV40028725	-0.30572	0.305716231	protein_coding	NM_002060
ASHGV40033817	0.305634	0.305634174	protein_coding	NM_013327
ASHGV40019013	0.305605	0.305605199	protein_coding	NM_133443
ASHGV40044129	0.305449	0.305448617	protein_coding	NM_014739
ASHGV40007109	0.305336	0.305335629	protein_coding	NM_054108
ASHGV40028813	0.305327	0.305327267	protein_coding	NM_012249
ASHGV40012464	-0.30482	0.304821101	protein_coding	NM_001014286
ASHGV40034934	-0.30463	0.304632607	protein_coding	NM_025041
ASHGV40014670	-0.30462	0.304621984	protein_coding	NM_183001
ASHGV40012349	0.304523	0.304523442	protein_coding	NM_006437
ASHGV40009893	0.304511	0.304511371	protein_coding	NM_006105
ASHGV40012007	-0.30447	0.30447122	protein_coding	NM_006700
ASHGV40003354	-0.30446	0.304462899	protein_coding	NM_181809
ASHGV40027161	0.30428	0.304279914	protein_coding	NM_001102426
ASHGV40009575	-0.30428	0.30427702	protein_coding	NM_016509
ASHGV40019301	-0.30424	0.304236863	protein_coding	NM_003983
ASHGV40005434	-0.30421	0.304212412	protein_coding	NM_001172303
ASHGV40013992	0.304199	0.304199083	protein_coding	NM_006364
ASHGV40056474	-0.30417	0.30416798	protein_coding	uc010xhf.1
ASHGV40027497	0.304081	0.304081405	protein_coding	NM_005517
ASHGV40032731	0.303987	0.303987132	protein_coding	NM_003683
ASHGV40008503	0.303962	0.3039624	protein_coding	NM_024098

ASHGV40023754	-0.30392	0.303924777	protein_coding	NM_001013706
ASHGV40002605	-0.30386	0.303864035	protein_coding	ENST00000594769
ASHGV40046789	0.303849	0.303849192	protein_coding	NM_005868
ASHGV40017792	-0.30382	0.303823705	protein_coding	NM_000086
ASHGV40031678	0.303821	0.303821157	protein_coding	NM_018478
ASHGV40034331	-0.30372	0.303719792	protein_coding	NM_000884
ASHGV40020246	0.303662	0.30366206	protein_coding	NM_000978
ASHGV40035811	0.303658	0.303658232	protein_coding	NM_020839
ASHGV40017502	-0.30361	0.303609754	protein_coding	NM_001048212
ASHGV40012412	-0.30358	0.303577544	protein_coding	NM_002128
ASHGV40052048	-0.30354	0.303540001	protein_coding	NM_153698
ASHGV40017367	0.303516	0.303516322	protein_coding	NM_005587
ASHGV40013321	-0.30351	0.30350727	protein_coding	NM_015383
ASHGV40021231	-0.30342	0.303420025	protein_coding	NM_001040
ASHGV40025666	-0.3034	0.303399374	protein_coding	NM_002503
ASHGV40020096	-0.30317	0.303167318	protein_coding	NM_178860
ASHGV40025014	-0.30312	0.303124383	protein_coding	NM_001245002
ASHGV40050364	-0.30297	0.302966608	protein_coding	NM_001013842
ASHGV40027607	0.302943	0.302943033	protein_coding	NM_138803
ASHGV40046677	-0.30291	0.302906759	protein_coding	NM_001145064
ASHGV40013005	-0.3029	0.302903636	protein_coding	NM_018386
ASHGV40041854	0.302799	0.302799446	protein_coding	NM_022902
ASHGV40045971	-0.30278	0.302776214	protein_coding	NM_001145121
ASHGV40001301	-0.30275	0.302749998	protein_coding	NM_001197113
ASHGV40037500	-0.30274	0.302741719	protein_coding	NM_012110
ASHGV40033774	0.302688	0.30268826	protein_coding	NM_003216
ASHGV40024294	-0.30259	0.302592888	protein_coding	NM_001039876
ASHGV40022309	-0.30246	0.302462497	protein_coding	NM_030968
ASHGV40013252	0.302391	0.302391015	protein_coding	uc001uyq.2
ASHGV40040186	0.302326	0.302326252	protein_coding	NM_022483
ASHGV40031509	-0.30213	0.302127486	protein_coding	ENST00000601172
ASHGV40047993	0.302	0.301999913	protein_coding	NM_017528
ASHGV40057658	-0.30196	0.301959852	protein_coding	NM_005361
ASHGV40037487	0.301911	0.301910569	protein_coding	NM_001024611
ASHGV40003115	-0.30189	0.301887156	protein_coding	NM_001101391
ASHGV40033810	0.301816	0.301815884	protein_coding	NM_001044370
ASHGV40054940	0.301667	0.301666593	protein_coding	NM_012280
ASHGV40005781	-0.30163	0.301629204	protein_coding	NM_004273
ASHGV40038468	-0.30163	0.301626242	protein_coding	NM_175918
ASHGV40017832	0.301601	0.301600787	protein_coding	NM_001145524
ASHGV40016142	-0.30159	0.301591327	protein_coding	NM_020447
ASHGV40021563	-0.30146	0.301455438	protein_coding	NM_005957
ASHGV40050988	0.301399	0.301398919	protein_coding	NM_173549
ASHGV40021601	-0.30132	0.301320539	protein_coding	NM_013975
ASHGV40003190	0.301249	0.301249122	protein_coding	NM_001242939
ASHGV40016158	0.301215	0.301215002	protein_coding	NM_018285
ASHGV40008573	0.301098	0.301097701	protein_coding	NM_017670

ASHGV40039350	0.301091	0.301090664	protein_coding	NM_152618
ASHGV40046859	-0.30108	0.301084665	protein_coding	NM_145030
ASHGV40027693	0.301026	0.301025614	protein_coding	NM_024770
ASHGV40033350	0.300994	0.300993644	protein_coding	uc001diw.3
ASHGV40026172	-0.30095	0.300947631	protein_coding	NM_173548
ASHGV40032742	0.300929	0.300929163	protein_coding	NM_002626
ASHGV40011981	-0.30084	0.300836585	protein_coding	NM_031473
ASHGV40006651	-0.30074	0.300735816	protein_coding	NM_030962
ASHGV40047891	0.300727	0.300726601	protein_coding	NM_032324
ASHGV40024466	-0.30061	0.300611862	protein_coding	NM_198850
ASHGV40005107	-0.30044	0.300444698	protein_coding	ENST00000601031
ASHGV40000146	-0.30038	0.300384106	protein_coding	ENST00000372526
ASHGV40035221	0.300309	0.300309289	protein_coding	NM_032487
ASHGV40045067	-0.30029	0.300288669	protein_coding	NM_003131
ASHGV40029974	-0.30027	0.300270722	protein_coding	NM_001207067
ASHGV40034930	0.300243	0.300243077	protein_coding	NM_007027
ASHGV40015793	0.300239	0.300238576	protein_coding	NM_003779
ASHGV40019743	-0.30021	0.300210013	protein_coding	NM_014519
ASHGV40050363	-0.30018	0.300175432	protein_coding	NM_021630
ASHGV40048751	-0.30016	0.300162345	protein_coding	NM_030936
ASHGV40027233	-0.30011	0.300108866	protein_coding	NM_015627
ASHGV40007799	-0.29999	0.299991607	protein_coding	NM_031307
ASHGV40056122	-0.29998	0.299977916	protein_coding	NM_001271507
ASHGV40031918	-0.29996	0.299963794	protein_coding	NM_018270
ASHGV40041031	-0.29967	0.299673197	protein_coding	NM_001146040
ASHGV40042187	0.29965	0.299649545	protein_coding	NM_001284
ASHGV40031161	-0.29933	0.29932912	protein_coding	NM_172107
ASHGV40008804	-0.29927	0.299270666	protein_coding	NM_001012710
ASHGV40054759	-0.29922	0.299219749	protein_coding	NM_000444
ASHGV40049243	-0.29905	0.299054588	protein_coding	NM_024836
ASHGV40053973	-0.299	0.299002909	protein_coding	NM_004182
ASHGV40010626	-0.29899	0.298994067	protein_coding	NM_016569
ASHGV40015430	-0.29897	0.298969972	protein_coding	NM_004434
ASHGV40057116	-0.29888	0.298884352	protein_coding	NM_000791
ASHGV40039090	-0.29885	0.29884534	protein_coding	NM_020859
ASHGV40021867	-0.29875	0.298751433	protein_coding	NM_003110
ASHGV40055074	0.298418	0.298418256	protein_coding	NM_000166
ASHGV40043407	-0.2984	0.298395915	protein_coding	NM_001305102
ASHGV40024760	0.298219	0.298218911	protein_coding	NM_004781
ASHGV40000126	0.298192	0.298192395	protein_coding	NM_018452
ASHGV40039485	-0.29814	0.298140388	protein_coding	NM_024605
ASHGV40025800	-0.29801	0.298007994	protein_coding	NM_006509
ASHGV40020406	-0.29785	0.297854687	protein_coding	NM_001098833
ASHGV40043571	-0.29781	0.297814818	protein_coding	NM_000324
ASHGV40038936	-0.29776	0.297761405	protein_coding	NM_014847
ASHGV40047309	0.297722	0.297722341	protein_coding	NM_006712
ASHGV40044954	0.297718	0.297717758	protein_coding	NM_015245

ASHGV40050556	0.29769	0.297689683	protein_coding	NM_020130
ASHGV40006424	0.297682	0.297681798	protein_coding	NM_001105521
ASHGV40007246	-0.29762	0.297622471	protein_coding	NM_016028
ASHGV40014182	-0.29762	0.297618733	protein_coding	NM_005620
ASHGV40057061	0.297554	0.29755383	protein_coding	NM_006174
ASHGV40031628	0.297504	0.297503583	protein_coding	NM_006275
ASHGV40006323	0.297489	0.297489467	protein_coding	NM_153336
ASHGV40009976	-0.29737	0.297368737	protein_coding	NM_002282
ASHGV40019259	0.297335	0.297335381	protein_coding	NM_003869
ASHGV40041896	-0.29723	0.297232439	protein_coding	NM_002270
ASHGV40039532	0.297231	0.297231061	protein_coding	NM_014017
ASHGV40053398	-0.29723	0.297230828	protein_coding	NM_138777
ASHGV40000569	0.297199	0.297198781	protein_coding	NM_020787
ASHGV40054710	0.297185	0.297185027	protein_coding	NM_003611
ASHGV40050611	0.297069	0.297069391	protein_coding	NM_001080394
ASHGV40041183	-0.29699	0.296985264	protein_coding	NM_138369
ASHGV40028777	-0.29698	0.296979155	protein_coding	NM_138370
ASHGV40016703	0.296962	0.296961631	protein_coding	NM_178527
ASHGV40007971	0.296935	0.296935309	protein_coding	NM_000218
ASHGV40019681	0.29692	0.296920061	protein_coding	NM_020310
ASHGV40008198	0.296852	0.296852137	protein_coding	NM_001009909
ASHGV40009445	-0.29672	0.296719536	protein_coding	NM_031474
ASHGV40011422	-0.29655	0.296549049	protein_coding	NM_001417
ASHGV40024293	-0.29655	0.29654698	protein_coding	NM_003332
ASHGV40023161	-0.29652	0.296522415	protein_coding	ENST00000400512
ASHGV40042168	-0.29652	0.296518066	protein_coding	NM_152624
ASHGV40022353	-0.29651	0.296509787	protein_coding	NM_012140
ASHGV40008259	-0.29636	0.296359288	protein_coding	NM_006360
ASHGV40015545	-0.29633	0.296334463	protein_coding	NM_006427
ASHGV40057023	0.296265	0.296265418	protein_coding	NM_144646
ASHGV40044973	0.296066	0.296065508	protein_coding	NM_173562
ASHGV40039843	-0.29596	0.295961171	protein_coding	NM_032479
ASHGV40006349	-0.29587	0.295868547	protein_coding	NM_032182
ASHGV40045246	0.295698	0.295698153	protein_coding	NM_001044305
ASHGV40048682	-0.2956	0.295601217	protein_coding	NM_003040
ASHGV40039100	0.295379	0.295379434	protein_coding	NM_198892
ASHGV40035350	0.29501	0.295010428	protein_coding	NM_033259
ASHGV40011526	-0.29499	0.294991112	protein_coding	NM_144576
ASHGV40011474	-0.29487	0.294873449	protein_coding	NM_018364
ASHGV40021235	-0.29486	0.294858693	protein_coding	NM_001406
ASHGV40040360	-0.29469	0.294692609	protein_coding	NM_015084
ASHGV40045591	-0.29468	0.294680982	protein_coding	NM_182645
ASHGV40055556	0.294671	0.294670649	protein_coding	NM_005393
ASHGV40020322	-0.29459	0.294591325	protein_coding	NM_002278
ASHGV40053036	-0.29457	0.294569059	protein_coding	NM_024945
ASHGV40011075	0.294434	0.294433916	protein_coding	NM_004064
ASHGV40011376	0.294195	0.294194638	protein_coding	NM_173602

ASHGV40053510	-0.29414	0.29414452	protein_coding	NM_032809
ASHGV40007807	0.294076	0.294075706	protein_coding	NM_003139
ASHGV40049347	0.29402	0.294019799	protein_coding	NM_003580
ASHGV40031413	-0.29401	0.294014427	protein_coding	NM_052940
ASHGV40018230	-0.2938	0.29380241	protein_coding	NM_181536
ASHGV40002780	-0.29372	0.293719388	protein_coding	NM_017787
ASHGV40055156	-0.29365	0.293654912	protein_coding	NM_033048
ASHGV40027561	0.293455	0.293454859	protein_coding	NM_004543
ASHGV40051801	-0.29336	0.293355569	protein_coding	NM_001085476
ASHGV40048722	0.293353	0.293353365	protein_coding	NM_024012
ASHGV40021587	-0.29325	0.293249395	protein_coding	NM_002982
ASHGV40024312	0.293158	0.293158463	protein_coding	NM_153257
ASHGV40021521	0.292947	0.292947276	protein_coding	NM_138349
ASHGV40037153	-0.29291	0.292908504	protein_coding	NM_003703
ASHGV40026346	0.292852	0.292851587	protein_coding	NM_005742
ASHGV40021994	-0.29267	0.292673008	protein_coding	uc010dck.1
ASHGV40025750	0.292645	0.292645476	protein_coding	NM_001271938
ASHGV40020715	-0.29264	0.292636509	protein_coding	NM_199340
ASHGV40026063	0.292558	0.292557998	protein_coding	NM_017852
ASHGV40049462	-0.2923	0.29230339	protein_coding	NM_018299
ASHGV40054229	0.292178	0.292177709	protein_coding	NM_001184880
ASHGV40024495	-0.29212	0.292122176	protein_coding	NM_152354
ASHGV40028838	0.291994	0.291993833	protein_coding	NM_002158
ASHGV40020693	0.291927	0.291926518	protein_coding	NM_002221
ASHGV40037084	0.291904	0.291904249	protein_coding	NM_032288
ASHGV40019068	0.291894	0.291893762	protein_coding	NM_001040284
ASHGV40054985	0.291874	0.291873953	protein_coding	NM_018094
ASHGV40024479	-0.29185	0.291853442	protein_coding	NM_001031749
ASHGV40020595	-0.29157	0.291572248	protein_coding	NM_001085430
ASHGV40017374	-0.29149	0.291489911	protein_coding	NM_002928
ASHGV40037141	-0.29137	0.291368796	protein_coding	NM_003637
ASHGV40037453	0.291357	0.291357484	protein_coding	NM_138335
ASHGV40005866	-0.2913	0.291300013	protein_coding	NM_020338
ASHGV40024929	-0.29115	0.291154616	protein_coding	NM_005860
ASHGV40014314	0.291048	0.291047964	protein_coding	NM_001107
ASHGV40010656	-0.29062	0.2906229	protein_coding	NM_019086
ASHGV40050093	-0.29028	0.290276352	protein_coding	NM_201589
ASHGV40044659	-0.29026	0.290263483	protein_coding	NM_003094
ASHGV40041628	-0.29023	0.290231898	protein_coding	NM_018034
ASHGV40010556	-0.2901	0.290095299	protein_coding	NM_018984
ASHGV40050600	-0.29	0.290002577	protein_coding	NM_145314
ASHGV40020279	-0.28998	0.289975821	protein_coding	NM_032865
ASHGV40028651	0.289906	0.289906361	protein_coding	NM_187841
ASHGV40048135	0.289895	0.289895218	protein_coding	NM_032120
ASHGV40011281	0.289776	0.289775651	protein_coding	NM_016123
ASHGV40007026	0.289693	0.289693491	protein_coding	NM_002556
ASHGV40028602	-0.28953	0.289528493	protein_coding	NM_001080473

ASHGV40003054	0.289393	0.289393273	protein_coding	NM_001001
ASHGV40033095	-0.28933	0.289334052	protein_coding	NM_005368
ASHGV40019781	0.289183	0.289182884	protein_coding	NM_024297
ASHGV40028660	0.289177	0.28917709	protein_coding	NM_032434
ASHGV40021927	0.289155	0.289154807	protein_coding	NM_025149
ASHGV40054469	0.289069	0.289069116	protein_coding	NM_002139
ASHGV40056995	0.289037	0.289036968	protein_coding	NM_001145191
ASHGV40017085	-0.28893	0.288933023	protein_coding	NM_147188
ASHGV40043874	0.288882	0.28888178	protein_coding	NM_001080481
ASHGV40026134	-0.28883	0.288834221	protein_coding	NM_020657
ASHGV40041827	0.288653	0.28865254	protein_coding	NM_005869
ASHGV40027354	-0.28844	0.288442104	protein_coding	NM_152835
ASHGV40056391	-0.28834	0.288335063	protein_coding	NM_001661
ASHGV40006138	0.288333	0.28833277	protein_coding	NM_017649
ASHGV40009005	0.288215	0.288214824	protein_coding	NM_033395
ASHGV40041253	-0.28798	0.28798012	protein_coding	NM_004350
ASHGV40009099	0.287909	0.287908651	protein_coding	NM_003478
ASHGV40027223	-0.2878	0.287796219	protein_coding	NM_032528
ASHGV40055408	-0.28779	0.287794302	protein_coding	NM_001170779
ASHGV40036101	0.287725	0.28772534	protein_coding	NM_006407
ASHGV40043701	-0.28745	0.287448928	protein_coding	NM_138441
ASHGV40021639	-0.28738	0.287380324	protein_coding	NM_005568
ASHGV40017864	0.287335	0.287334705	protein_coding	NM_052874
ASHGV40008022	-0.28732	0.287320355	protein_coding	NM_006074
ASHGV40021391	-0.28729	0.287293662	protein_coding	NM_001388
ASHGV40013980	-0.28723	0.287228463	protein_coding	NM_004496
ASHGV40033235	-0.28723	0.287228294	protein_coding	NM_012263
ASHGV40040948	0.287093	0.287092526	protein_coding	NM_003115
ASHGV40026152	-0.28707	0.287071479	protein_coding	NM_032828
ASHGV40031596	-0.28703	0.287033479	protein_coding	NM_021931
ASHGV40000107	0.286712	0.286711827	protein_coding	uc010oyb.2
ASHGV40043574	-0.28655	0.286549837	protein_coding	NM_006061
ASHGV40023501	0.286538	0.286537829	protein_coding	NM_004361
ASHGV40022876	0.286533	0.286532778	protein_coding	NM_004869
ASHGV40018642	-0.28644	0.286444792	protein_coding	NM_032575
ASHGV40046874	-0.28641	0.286413521	protein_coding	NM_152495
ASHGV40040187	0.286398	0.286397895	protein_coding	NM_198566
ASHGV40020424	-0.28637	0.286365744	protein_coding	uc021tyg.1
ASHGV40028666	0.286332	0.286331619	protein_coding	NM_005253
ASHGV40013232	0.286289	0.286288749	protein_coding	NM_020751
ASHGV40035722	0.286107	0.28610672	protein_coding	NM_201653
ASHGV40020149	0.286057	0.286056821	protein_coding	NM_015194
ASHGV40049361	0.285995	0.285995383	protein_coding	NM_004056
ASHGV40023584	-0.28587	0.285868222	protein_coding	NM_007345
ASHGV40009107	-0.28582	0.285818463	protein_coding	NM_033390
ASHGV40006590	-0.28578	0.28578342	protein_coding	NM_001001917
ASHGV40023782	-0.28577	0.285772748	protein_coding	NM_000635

ASHGV40056447	0.285701	0.285700813	protein_coding	NM_001137671
ASHGV40041045	0.285691	0.285690686	protein_coding	NM_032385
ASHGV40021717	-0.28563	0.285628218	protein_coding	NM_001190460
ASHGV40049767	-0.28557	0.285572773	protein_coding	NM_001300
ASHGV40036446	0.285501	0.285500648	protein_coding	NM_032839
ASHGV40011416	-0.28538	0.285375645	protein_coding	ENST00000601218
ASHGV40041234	0.285358	0.285357867	protein_coding	NM_016222
ASHGV40040291	-0.28526	0.28526469	protein_coding	NM_001048249
ASHGV40045974	0.285098	0.285097656	protein_coding	NM_001674
ASHGV40028844	-0.28456	0.28455616	protein_coding	NM_012199
ASHGV40036677	-0.28446	0.284457699	protein_coding	NM_053002
ASHGV40055549	-0.28437	0.284368224	protein_coding	NM_001711
ASHGV40001854	0.284292	0.284291911	protein_coding	NM_001206627
ASHGV40022230	-0.28424	0.284238044	protein_coding	NM_001162997
ASHGV40051713	-0.28408	0.284075145	protein_coding	NM_001012446
ASHGV40022223	-0.28387	0.283865808	protein_coding	NM_207346
ASHGV40057724	0.283638	0.283638233	protein_coding	NM_145202
ASHGV40046555	-0.28359	0.283591701	protein_coding	NM_030796
ASHGV40044504	0.283589	0.283588623	protein_coding	NM_006763
ASHGV40030621	-0.28351	0.283510864	protein_coding	NM_020343
ASHGV40000042	0.283482	0.283481883	protein_coding	NM_001144013
ASHGV40054337	-0.28348	0.283475481	protein_coding	NM_020871
ASHGV40055170	-0.28342	0.28341509	protein_coding	NM_173698
ASHGV40011433	0.28338	0.2833797	protein_coding	NM_032889
ASHGV40020347	-0.28328	0.283278996	protein_coding	NM_012285
ASHGV40050501	-0.28327	0.283266482	protein_coding	NM_080872
ASHGV40037914	0.283263	0.283263069	protein_coding	NM_014822
ASHGV40010103	0.283191	0.283191099	protein_coding	NM_014925
ASHGV40019063	-0.2831	0.283095745	protein_coding	NM_001281789
ASHGV40054365	-0.28303	0.283032383	protein_coding	NM_024528
ASHGV40036812	-0.283	0.282995336	protein_coding	NM_022763
ASHGV40048131	0.282717	0.282717117	protein_coding	NM_019004
ASHGV40008042	-0.28271	0.28270923	protein_coding	NM_144666
ASHGV40006143	-0.28262	0.282619341	protein_coding	NM_014976
ASHGV40018583	-0.28255	0.28254842	protein_coding	NM_015944
ASHGV40035795	0.28253	0.282530107	protein_coding	NM_002078
ASHGV40023857	0.282453	0.282453379	protein_coding	NM_024106
ASHGV40033154	0.282405	0.282404553	protein_coding	NM_012264
ASHGV40016340	-0.28235	0.282349166	protein_coding	NM_005829
ASHGV40018773	-0.28233	0.282334717	protein_coding	NM_002646
ASHGV40043265	-0.28225	0.282245829	protein_coding	NM_002341
ASHGV40020254	0.282075	0.282074559	protein_coding	NM_017982
ASHGV40057162	-0.28179	0.281787095	protein_coding	NM_052899
ASHGV40015675	0.281709	0.281708667	protein_coding	NM_133647
ASHGV40001976	0.281706	0.281705726	protein_coding	NM_014042
ASHGV40002467	-0.28166	0.281656866	protein_coding	ENST00000581929
ASHGV40053843	-0.28165	0.281650631	protein_coding	NM_001033583

ASHGV40020192	-0.28165	0.281645562	protein_coding	NM_024302
ASHGV40025894	0.281603	0.281602908	protein_coding	NM_003089
ASHGV40033484	-0.28143	0.281428616	protein_coding	NM_001310156
ASHGV40035594	0.281408	0.281408366	protein_coding	NM_032492
ASHGV40037774	0.281282	0.281282477	protein_coding	NM_014485
ASHGV40009127	0.281277	0.281276564	protein_coding	NM_080659
ASHGV40030809	-0.28124	0.281243246	protein_coding	NM_024918
ASHGV40030221	0.28122	0.281219668	protein_coding	NM_004504
ASHGV40025787	-0.28115	0.281150381	protein_coding	NM_001013641
ASHGV40049848	0.281005	0.281004766	protein_coding	NM_021021
ASHGV40050443	0.281	0.281000361	protein_coding	NM_024567
ASHGV40032405	0.280982	0.280981995	protein_coding	ENST00000601068
ASHGV40027092	-0.28097	0.280969172	protein_coding	NM_000682
ASHGV40041978	-0.28089	0.280887336	protein_coding	NM_018122
ASHGV40033390	-0.28066	0.280657061	protein_coding	NM_015367
ASHGV40029769	-0.28061	0.280611064	protein_coding	NM_002610
ASHGV40040924	-0.28049	0.280488266	protein_coding	NM_002587
ASHGV40027653	0.280444	0.280443796	protein_coding	NM_014900
ASHGV40040587	0.28025	0.280249811	protein_coding	NM_005600
ASHGV40048352	0.280167	0.280167077	protein_coding	NM_001233
ASHGV40019780	-0.28017	0.280165479	protein_coding	NM_004422
ASHGV40017139	0.280057	0.280056747	protein_coding	ENST00000560255
ASHGV40005261	-0.27999	0.279991785	protein_coding	NM_005185
ASHGV40007136	0.279971	0.27997122	protein_coding	NM_153819
ASHGV40054301	0.27997	0.279969668	protein_coding	NM_004089
ASHGV40050502	-0.27993	0.279932808	protein_coding	ENST00000437887
ASHGV40007722	-0.27987	0.279867589	protein_coding	NM_001145018
ASHGV40057643	-0.27985	0.279847916	protein_coding	NM_012084
ASHGV40006655	-0.27968	0.279684062	protein_coding	NM_006691
ASHGV40017365	-0.2796	0.279602495	protein_coding	NM_172000
ASHGV40047492	-0.27957	0.279568242	protein_coding	NM_001097622
ASHGV40025921	-0.27951	0.279510352	protein_coding	NM_014203
ASHGV40021766	0.279494	0.279493818	protein_coding	NM_005440
ASHGV40008876	-0.27893	0.278932852	protein_coding	ENST00000530460
ASHGV40054608	-0.27871	0.278708249	protein_coding	NM_018390
ASHGV40028599	-0.27867	0.278666982	protein_coding	NM_052920
ASHGV40054400	0.278654	0.278654385	protein_coding	NM_001013628
ASHGV40022296	0.278637	0.278637081	protein_coding	NM_001204210
ASHGV40050117	0.278589	0.278588527	protein_coding	NM_024701
ASHGV40006443	0.278366	0.278366343	protein_coding	NM_152911
ASHGV40057355	0.278285	0.278285044	protein_coding	NM_020186
ASHGV40041984	-0.27826	0.27825599	protein_coding	NM_001017971
ASHGV40057574	0.278254	0.278253571	protein_coding	NM_000972
ASHGV40050251	0.278127	0.278127002	protein_coding	NM_012331
ASHGV40000612	-0.27811	0.27811247	protein_coding	ENST00000425577
ASHGV40056453	-0.27777	0.277768739	protein_coding	NM_000985
ASHGV40023655	0.277725	0.277724681	protein_coding	NM_194460

ASHGV40021683	-0.2777	0.277699494	protein_coding	NM_002686
ASHGV40031947	0.277611	0.277610658	protein_coding	NM_024299
ASHGV40032868	0.277516	0.27751603	protein_coding	NM_023004
ASHGV40020823	-0.2775	0.277498938	protein_coding	NM_001080466
ASHGV40000584	-0.27747	0.277469453	protein_coding	ENST00000424293
ASHGV40055937	-0.27742	0.277420414	protein_coding	NM_005029
ASHGV40045624	-0.27737	0.277371627	protein_coding	NM_006714
ASHGV40047082	0.277358	0.277358468	protein_coding	NM_015450
ASHGV40000019	-0.27735	0.277350102	protein_coding	NM_015544
ASHGV40038354	-0.27726	0.277260918	protein_coding	NM_004346
ASHGV40040105	0.27723	0.277230208	protein_coding	NM_002853
ASHGV40019181	0.277054	0.277054029	protein_coding	NM_020312
ASHGV40008513	-0.27701	0.277007785	protein_coding	NM_014224
ASHGV40019271	-0.277	0.277004018	protein_coding	NM_014187
ASHGV40057514	-0.27696	0.276964078	protein_coding	NM_033412
ASHGV40034935	-0.27684	0.276841375	protein_coding	NM_005630
ASHGV40039765	-0.27679	0.27679059	protein_coding	NM_003265
ASHGV40013785	0.276777	0.27677715	protein_coding	NM_004079
ASHGV40008400	0.276767	0.27676741	protein_coding	NM_024763
ASHGV40034846	-0.27666	0.276661144	protein_coding	NM_001015050
ASHGV40034954	0.276639	0.276639075	protein_coding	NM_018133
ASHGV40014276	0.276582	0.276582342	protein_coding	NM_203309
ASHGV40051670	-0.27654	0.276535387	protein_coding	NM_001282530
ASHGV40028729	-0.27653	0.276531579	protein_coding	ENST00000392061
ASHGV40052291	-0.2758	0.275796625	protein_coding	NM_020946
ASHGV40054582	-0.27562	0.275619817	protein_coding	NM_001110556
ASHGV40054886	0.275613	0.275613077	protein_coding	NM_021140
ASHGV40051719	-0.27532	0.275317105	protein_coding	NM_022781
ASHGV40033518	0.275199	0.275198894	protein_coding	NM_001254733
ASHGV40026635	0.275075	0.275074861	protein_coding	NM_001031684
ASHGV40048258	-0.27504	0.275037857	protein_coding	NM_001278563
ASHGV40015955	-0.27503	0.275026809	protein_coding	NM_020821
ASHGV40018074	0.275006	0.275006357	protein_coding	NM_016284
ASHGV40020345	0.274985	0.274984719	protein_coding	NM_021078
ASHGV40006153	-0.27498	0.274979457	protein_coding	NM_001002759
ASHGV40043735	0.274926	0.27492638	protein_coding	NM_017934
ASHGV40018431	-0.27465	0.27464577	protein_coding	NM_000485
ASHGV40015891	-0.27441	0.274408099	protein_coding	NM_000570
ASHGV40034419	0.2744	0.274399544	protein_coding	ENST00000446157
ASHGV40045915	0.274042	0.274041894	protein_coding	NM_002377
ASHGV40045084	-0.27393	0.273928033	protein_coding	NM_001171623
ASHGV40014307	0.2739	0.273899659	protein_coding	NM_015557
ASHGV40048782	0.27377	0.273770419	protein_coding	NM_018051
ASHGV40014298	-0.27356	0.273562769	protein_coding	NM_001105579
ASHGV40044992	0.273547	0.273546619	protein_coding	NM_015050
ASHGV40011370	0.273516	0.273515884	protein_coding	NM_032901
ASHGV40015901	-0.27335	0.27334858	protein_coding	NM_001284338

ASHGV40009101	0.273305	0.273305047	protein_coding	NM_000051
ASHGV40022747	0.273284	0.273284371	protein_coding	NM_017865
ASHGV40055190	-0.27325	0.27325017	protein_coding	NM_016065
ASHGV40041833	0.273226	0.273226386	protein_coding	NM_015935
ASHGV40011062	0.273205	0.27320514	protein_coding	ENST00000601123
ASHGV40056858	-0.27289	0.272889127	protein_coding	NM_001051
ASHGV40022294	0.272883	0.272882872	protein_coding	NM_004710
ASHGV40015783	-0.27285	0.272846398	protein_coding	NM_032998
ASHGV40034763	-0.2726	0.272597709	protein_coding	NM_144718
ASHGV40012888	0.272548	0.272547738	protein_coding	NM_033110
ASHGV40018557	0.272533	0.272532913	protein_coding	NM_001099456
ASHGV40041868	-0.27249	0.272494088	protein_coding	NM_015484
ASHGV40013773	-0.27248	0.272478746	protein_coding	NM_138376
ASHGV40011913	-0.27234	0.272335206	protein_coding	NM_017686
ASHGV40001685	0.272154	0.27215402	protein_coding	NM_016096
ASHGV40031003	0.27215	0.272149816	protein_coding	NM_173091
ASHGV40008651	-0.27195	0.271954128	protein_coding	NM_001098785
ASHGV40006917	0.271936	0.271936323	protein_coding	NM_001008938
ASHGV40055329	0.271883	0.271882679	protein_coding	NM_003336
ASHGV40035240	0.271866	0.271865878	protein_coding	NM_002662
ASHGV40041447	0.271534	0.271533898	protein_coding	NM_024010
ASHGV40010503	0.27152	0.271519821	protein_coding	NM_001135570
ASHGV40000040	-0.27125	0.271249751	protein_coding	ENST00000302096
ASHGV40005745	0.271238	0.271237607	protein_coding	NM_001852
ASHGV40026036	-0.27121	0.271210771	protein_coding	NM_015629
ASHGV40030419	-0.27112	0.271122308	protein_coding	uc002wct.1
ASHGV40047784	0.271105	0.27110462	protein_coding	NM_031449
ASHGV40020599	-0.27101	0.271011756	protein_coding	NM_004645
ASHGV40031366	0.270879	0.270878636	protein_coding	NM_006363
ASHGV40037147	-0.27086	0.270860929	protein_coding	NM_144698
ASHGV40025321	0.270468	0.270468314	protein_coding	NM_024578
ASHGV40053467	0.270351	0.270350609	protein_coding	NM_014580
ASHGV40030150	0.270187	0.270186956	protein_coding	NM_024293
ASHGV40039264	-0.27004	0.270038344	protein_coding	NM_018983
ASHGV40030131	0.269888	0.269887619	protein_coding	NM_005444
ASHGV40046946	-0.26987	0.269872407	protein_coding	NM_181733
ASHGV40016913	0.269802	0.269802298	protein_coding	NM_001018005
ASHGV40019473	-0.26974	0.269742042	protein_coding	NM_017429
ASHGV40053853	-0.26973	0.269728693	protein_coding	NM_004845
ASHGV40018036	0.269715	0.269714845	protein_coding	NM_001144
ASHGV40029202	-0.2696	0.269598235	protein_coding	NM_020184
ASHGV40014807	0.269563	0.269562962	protein_coding	NM_025230
ASHGV40032611	0.26935	0.269349754	protein_coding	NM_003024
ASHGV40060828	0.26917	0.269170286	protein_coding	uc002ryz.3
ASHGV40005980	0.26915	0.269149838	protein_coding	NM_032257
ASHGV40043560	0.269041	0.269041446	protein_coding	NM_024529
ASHGV40032221	-0.26904	0.269041108	protein_coding	NM_000219

ASHGV40006925	-0.26901	0.269008029	protein_coding	NM_001302489
ASHGV40046168	0.26889	0.268889561	protein_coding	NM_015204
ASHGV40024493	0.268815	0.268814628	protein_coding	NM_001083335
ASHGV40009327	-0.26878	0.268784004	protein_coding	NM_152713
ASHGV40045936	0.268733	0.268733492	protein_coding	NM_152410
ASHGV40020536	-0.26859	0.268593431	protein_coding	NM_005220
ASHGV40056119	0.26859	0.268590255	protein_coding	NM_004857
ASHGV40015182	-0.26852	0.268521169	protein_coding	NM_182476
ASHGV40019698	-0.26851	0.268508978	protein_coding	NM_145068
ASHGV40031654	0.268478	0.268478046	protein_coding	NM_024331
ASHGV40007892	-0.26835	0.268349515	protein_coding	NM_003641
ASHGV40011861	-0.26835	0.268345967	protein_coding	NM_207356
ASHGV40041771	0.268298	0.268298492	protein_coding	NM_153706
ASHGV40036493	-0.26828	0.268284528	protein_coding	NM_015720
ASHGV40021963	-0.26825	0.268254539	protein_coding	ENST00000412360
ASHGV40017631	0.268114	0.268114362	protein_coding	NM_015659
ASHGV40052311	0.268043	0.268043085	protein_coding	NM_002721
ASHGV40018704	-0.26802	0.26802485	protein_coding	NM_003498
ASHGV40006714	-0.2679	0.267898679	protein_coding	ENST00000527671
ASHGV40046328	0.267841	0.267841356	protein_coding	NM_003238
ASHGV40044767	-0.26782	0.267824305	protein_coding	NM_005865
ASHGV40000262	0.267812	0.267812158	protein_coding	NM_007276
ASHGV40026954	0.267764	0.267764027	protein_coding	NM_006302
ASHGV40022382	-0.26771	0.26771314	protein_coding	NM_022366
ASHGV40000258	-0.26766	0.26765619	protein_coding	NM_007082
ASHGV40047920	-0.26744	0.267435752	protein_coding	ENST00000456890
ASHGV40034821	0.267417	0.267416931	protein_coding	NM_024610
ASHGV40005601	-0.26739	0.267387352	protein_coding	NM_001278688
ASHGV40051765	0.26737	0.267370116	protein_coding	NM_173576
ASHGV40031633	-0.26736	0.267362577	protein_coding	NM_015478
ASHGV40022746	-0.26728	0.267275438	protein_coding	NM_001145472
ASHGV40017748	0.267219	0.267219106	protein_coding	NM_199051
ASHGV40006779	0.267003	0.267002796	protein_coding	NM_030771
ASHGV40024029	0.266586	0.266586005	protein_coding	NM_001170686
ASHGV40044511	-0.26624	0.266240411	protein_coding	NM_001128591
ASHGV40036573	0.266231	0.266230792	protein_coding	NM_006623
ASHGV40013158	0.266203	0.266202654	protein_coding	NM_005800
ASHGV40046321	-0.26619	0.266190115	protein_coding	NM_016052
ASHGV40038640	0.266004	0.266003804	protein_coding	NM_001775
ASHGV40050242	-0.26598	0.265982164	protein_coding	ENST00000518496
ASHGV40050612	0.265819	0.265818765	protein_coding	NM_005914
ASHGV40012136	-0.26567	0.265671017	protein_coding	NM_003677
ASHGV40050405	0.265506	0.265505569	protein_coding	NM_024940
ASHGV40036565	0.265504	0.265503565	protein_coding	NM_001291999
ASHGV40011152	-0.26548	0.265480802	protein_coding	NM_018638
ASHGV40036609	0.265467	0.265466712	protein_coding	NM_001251845
ASHGV40053740	0.265414	0.265414478	protein_coding	NM_020742

ASHGV40009807	-0.26507	0.265065517	protein_coding	NM_004572
ASHGV40018029	0.265033	0.265032906	protein_coding	NM_001143685
ASHGV40009227	0.264948	0.264948497	protein_coding	NM_016146
ASHGV40041963	0.264912	0.26491168	protein_coding	NM_173797
ASHGV40010524	0.264803	0.264802583	protein_coding	NM_006825
ASHGV40052200	-0.26475	0.264748841	protein_coding	NM_031219
ASHGV40027355	0.264739	0.264739247	protein_coding	NM_032390
ASHGV40031586	-0.26466	0.264662185	protein_coding	NM_020336
ASHGV40040361	0.264581	0.264581489	protein_coding	NM_001231
ASHGV40034773	0.264545	0.264545121	protein_coding	NM_007136
ASHGV40041317	0.264465	0.264464725	protein_coding	NM_007277
ASHGV40032359	-0.26443	0.264433659	protein_coding	NM_004928
ASHGV40057785	-0.26441	0.264411402	protein_coding	NM_024869
ASHGV40035129	-0.26435	0.264349493	protein_coding	NM_001308229
ASHGV40024195	0.264246	0.264245544	protein_coding	NM_001031726
ASHGV40005157	-0.26421	0.264209233	protein_coding	NM_006659
ASHGV40045910	-0.2642	0.2642008	protein_coding	NM_005891
ASHGV40010688	0.264172	0.264172326	protein_coding	NM_016399
ASHGV40021169	0.264146	0.264146026	protein_coding	NM_006612
ASHGV40015523	-0.26408	0.264076168	protein_coding	ENST00000429169
ASHGV40039521	-0.26395	0.263947069	protein_coding	NM_001025595
ASHGV40049970	0.263803	0.263803499	protein_coding	NM_012472
ASHGV40001690	-0.2638	0.263797693	protein_coding	NM_006571
ASHGV40022112	0.263684	0.263683649	protein_coding	NM_012417
ASHGV40035651	-0.26355	0.263550149	protein_coding	NM_152536
ASHGV40041302	0.263543	0.263543205	protein_coding	NM_032765
ASHGV40046768	0.263457	0.263456915	protein_coding	NM_006980
ASHGV40011954	0.263456	0.263456002	protein_coding	NM_014653
ASHGV40041889	0.263332	0.263331614	protein_coding	NM_024754
ASHGV40034944	0.26331	0.263309805	protein_coding	NM_178554
ASHGV40035074	-0.26323	0.263227606	protein_coding	NM_016094
ASHGV40034335	0.263048	0.263047812	protein_coding	NM_005051
ASHGV40049159	0.26295	0.262950252	protein_coding	NM_014462
ASHGV40048540	-0.26275	0.262751826	protein_coding	NM_173569
ASHGV40040462	0.262663	0.262663074	protein_coding	NM_012446
ASHGV40030031	0.262522	0.262521534	protein_coding	NM_014929
ASHGV40019840	-0.26243	0.262431791	protein_coding	NM_001010855
ASHGV40046796	-0.26242	0.262415508	protein_coding	NM_000940
ASHGV40041442	-0.26238	0.262376699	protein_coding	NM_199344
ASHGV40025250	-0.26232	0.262315521	protein_coding	NM_004843
ASHGV40014946	0.262228	0.262228211	protein_coding	NM_001017923
ASHGV40040429	0.262158	0.262158162	protein_coding	NM_013391
ASHGV40052202	-0.26216	0.262155186	protein_coding	NM_017443
ASHGV40006986	-0.26205	0.262053473	protein_coding	NM_003146
ASHGV40012437	0.262046	0.262045678	protein_coding	NM_014887
ASHGV40055005	0.262037	0.262037274	protein_coding	NM_014599
ASHGV40010796	-0.26197	0.261966896	protein_coding	NM_013386

ASHGV40002002	-0.26197	0.261966092	protein_coding	ENST00000546837
ASHGV40052445	0.261954	0.261954374	protein_coding	NM_001080515
ASHGV40029624	0.261867	0.261866851	protein_coding	NM_015630
ASHGV40031959	-0.26181	0.26180808	protein_coding	NM_020062
ASHGV40043241	0.261775	0.261775271	protein_coding	NM_003897
ASHGV40051145	-0.26173	0.261727074	protein_coding	NM_022045
ASHGV40033039	-0.26169	0.261694012	protein_coding	NM_001193336
ASHGV40026702	-0.26163	0.261628331	protein_coding	NM_002643
ASHGV40049110	0.26152	0.261519811	protein_coding	NM_032664
ASHGV40043401	0.261425	0.261425397	protein_coding	NM_138718
ASHGV40041225	-0.26141	0.261413932	protein_coding	NM_006816
ASHGV40014772	-0.26139	0.261390482	protein_coding	NM_014045
ASHGV40033549	0.261357	0.261357133	protein_coding	NM_020437
ASHGV40024060	0.261337	0.261337346	protein_coding	NM_004335
ASHGV40008545	-0.26128	0.261279543	protein_coding	NM_001085372
ASHGV40048631	0.261257	0.261256726	protein_coding	NM_012256
ASHGV40012297	-0.26118	0.261179655	protein_coding	NM_199254
ASHGV40043284	-0.26115	0.261146902	protein_coding	NM_000434
ASHGV40024295	0.26107	0.261069645	protein_coding	NM_003820
ASHGV40023792	-0.26097	0.26097281	protein_coding	NM_198576
ASHGV40007889	-0.2609	0.260897839	protein_coding	NM_138329
ASHGV40018271	0.260895	0.260894754	protein_coding	NM_012091
ASHGV40043250	-0.26083	0.260829374	protein_coding	NM_001264
ASHGV40040792	0.260809	0.260809256	protein_coding	NM_133372
ASHGV40055895	-0.26068	0.260679389	protein_coding	NM_078470
ASHGV40031280	-0.26066	0.260656221	protein_coding	NM_032485
ASHGV40053656	0.260461	0.260461496	protein_coding	NM_014172
ASHGV40054580	-0.26044	0.260435234	protein_coding	NM_001586
ASHGV40022608	0.260362	0.260361588	protein_coding	NM_001007559
ASHGV40028704	0.260303	0.260302527	protein_coding	NM_032312
ASHGV40011096	0.260195	0.260195345	protein_coding	NM_177925
ASHGV40060890	-0.26015	0.260153179	protein_coding	uc011kro.1
ASHGV40004905	-0.26012	0.260119846	protein_coding	NM_005871
ASHGV40015601	0.259889	0.259888685	protein_coding	uc010uab.1
ASHGV40057673	0.259763	0.259762979	protein_coding	NM_032973
ASHGV40026408	0.259635	0.259635246	protein_coding	NM_024624
ASHGV40002560	-0.2596	0.259596913	protein_coding	ENST00000591656
ASHGV40055391	-0.25957	0.259574306	protein_coding	NM_016542
ASHGV40005791	-0.25938	0.25937715	protein_coding	NM_138357
ASHGV40007086	0.259303	0.259302746	protein_coding	NM_001286086
ASHGV40040796	-0.25923	0.259230858	protein_coding	NM_004199
ASHGV40011858	-0.25923	0.259225076	protein_coding	NM_001306084
ASHGV40033640	0.259072	0.259071512	protein_coding	NM_020816
ASHGV40000044	-0.25897	0.258968229	protein_coding	ENST00000307745
ASHGV40015086	0.258959	0.258959054	protein_coding	NM_031914
ASHGV40025108	-0.25894	0.25894413	protein_coding	NM_174894
ASHGV40011347	-0.25886	0.258862987	protein_coding	NM_005480

ASHGV40034334	0.258666	0.258666148	protein_coding	NM_001128142
ASHGV40020108	0.258465	0.258465499	protein_coding	NM_014030
ASHGV40031361	-0.25839	0.25838548	protein_coding	NM_001164811
ASHGV40029483	-0.25835	0.258350284	protein_coding	NM_020120
ASHGV40050307	0.258318	0.258318443	protein_coding	NM_152415
ASHGV40036413	-0.25822	0.258216926	protein_coding	NM_020754
ASHGV40055381	0.25818	0.258179962	protein_coding	NM_004794
ASHGV40057719	-0.25804	0.258042175	protein_coding	NM_152644
ASHGV40023925	-0.25798	0.257978149	protein_coding	NM_001080411
ASHGV40042466	-0.25785	0.257852871	protein_coding	NM_018927
ASHGV40008684	0.257811	0.257811443	protein_coding	ENST00000531602
ASHGV40023703	-0.25781	0.257810105	protein_coding	NM_016199
ASHGV40016754	-0.25778	0.257775861	protein_coding	NM_024063
ASHGV40028862	-0.25776	0.257761495	protein_coding	NM_001039753
ASHGV40016037	-0.25773	0.257732892	protein_coding	NM_017882
ASHGV40011610	0.257704	0.257703967	protein_coding	NM_178169
ASHGV40033041	-0.25769	0.257689157	protein_coding	NM_014303
ASHGV40014322	-0.2576	0.257600901	protein_coding	uc021ozs.1
ASHGV40027094	-0.25758	0.257581248	protein_coding	NM_001002036
ASHGV40003328	-0.25748	0.257476258	protein_coding	NM_080593
ASHGV40031920	-0.2574	0.257396815	protein_coding	NM_001853
ASHGV40040111	-0.25736	0.257360892	protein_coding	NM_152501
ASHGV40011625	-0.25734	0.257336189	protein_coding	NM_002524
ASHGV40008729	-0.25725	0.257251975	protein_coding	NM_001002912
ASHGV40035460	0.257149	0.257149357	protein_coding	NM_000851
ASHGV40028954	0.25702	0.257019568	protein_coding	NM_019002
ASHGV40026672	-0.25686	0.256857047	protein_coding	NM_022436
ASHGV40039099	0.256834	0.25683403	protein_coding	NM_005139
ASHGV40027773	0.256785	0.256784757	protein_coding	NM_178123
ASHGV40018654	-0.25675	0.256746017	protein_coding	NM_014692
ASHGV40037528	0.25674	0.256739926	protein_coding	NM_032313
ASHGV40032720	0.256703	0.256703257	protein_coding	NM_001001503
ASHGV40024676	-0.25668	0.256678303	protein_coding	NM_017509
ASHGV40037423	-0.2564	0.256397003	protein_coding	NM_003359
ASHGV40052128	0.256377	0.256376691	protein_coding	NM_005502
ASHGV40009910	-0.25626	0.256256172	protein_coding	NM_015270
ASHGV40023791	-0.25618	0.25617862	protein_coding	NM_024898
ASHGV40057528	-0.25611	0.256108439	protein_coding	uc004aee.1
ASHGV40044752	-0.25603	0.25602986	protein_coding	NM_007049
ASHGV40039491	0.255936	0.255935712	protein_coding	NM_001040260
ASHGV40043612	-0.2559	0.255899193	protein_coding	NM_021814
ASHGV40020392	-0.25582	0.255816367	protein_coding	NM_004090
ASHGV40050083	0.255742	0.255741926	protein_coding	NM_002347
ASHGV40014800	-0.25562	0.255620999	protein_coding	ENST00000354854
ASHGV40023860	-0.25556	0.25556139	protein_coding	NM_001130031
ASHGV40026933	0.255459	0.255458655	protein_coding	NM_144579
ASHGV40053134	0.255285	0.255284967	protein_coding	NM_032310

ASHGV40024082	-0.25527	0.2552655	protein_coding	NM_000923
ASHGV40005427	-0.25513	0.255127698	protein_coding	NM_019043
ASHGV40024017	0.255127	0.255127072	protein_coding	NM_052890
ASHGV40034886	0.255106	0.255106138	protein_coding	NM_020701
ASHGV40050145	0.255026	0.255025708	protein_coding	NM_019046
ASHGV40047210	0.254879	0.254878759	protein_coding	NM_030647
ASHGV40009437	-0.25484	0.254835127	protein_coding	NM_152640
ASHGV40053276	0.254797	0.254796946	protein_coding	NM_002874
ASHGV40035286	0.254766	0.254765882	protein_coding	NM_022470
ASHGV40039827	0.254752	0.254751744	protein_coding	NM_006598
ASHGV40046833	0.254723	0.254723326	protein_coding	NM_014891
ASHGV40007827	0.254687	0.254686733	protein_coding	NM_145013
ASHGV40023035	-0.25463	0.254625797	protein_coding	NM_001071
ASHGV40057308	-0.25447	0.254467586	protein_coding	NM_022728
ASHGV40056553	0.254453	0.254452967	protein_coding	uc009wjm.3
ASHGV40023725	-0.25445	0.254452748	protein_coding	NM_031304
ASHGV40048242	0.254431	0.254430794	protein_coding	NM_015908
ASHGV40050107	-0.25429	0.254294895	protein_coding	NM_001162914
ASHGV40015125	0.254067	0.254067066	protein_coding	NM_022474
ASHGV40019465	-0.25393	0.253928057	protein_coding	NM_001100624
ASHGV40016792	-0.25392	0.253915049	protein_coding	NM_003645
ASHGV40050806	0.253871	0.253871477	protein_coding	ENST00000521467
ASHGV40052994	0.253843	0.253842595	protein_coding	NM_007021
ASHGV40015032	0.25366	0.253659866	protein_coding	NM_017799
ASHGV40022203	-0.25348	0.253480621	protein_coding	NM_017728
ASHGV40028079	-0.25348	0.253480466	protein_coding	NM_005689
ASHGV40043485	0.253361	0.253360846	protein_coding	NM_004275
ASHGV40008649	0.253343	0.253342621	protein_coding	NM_020680
ASHGV40042434	-0.25333	0.253326069	protein_coding	NM_018938
ASHGV40031698	-0.25321	0.253210924	protein_coding	NM_004994
ASHGV40005635	-0.25315	0.253153134	protein_coding	ENST00000337653
ASHGV40053799	0.253052	0.253051622	protein_coding	ENST00000380342
ASHGV40034974	0.253045	0.25304522	protein_coding	NM_024491
ASHGV40009589	-0.25303	0.253025419	protein_coding	NM_002261
ASHGV40009399	0.252947	0.252947464	protein_coding	NM_001080407
ASHGV40021415	-0.25288	0.25287705	protein_coding	NM_014964
ASHGV40021763	0.252832	0.252832088	protein_coding	ENST00000361677
ASHGV40023207	0.252629	0.252628798	protein_coding	NM_002894
ASHGV40056621	-0.25244	0.252440324	protein_coding	NM_001101330
ASHGV40032412	-0.25221	0.252207208	protein_coding	NM_002340
ASHGV40057247	-0.2521	0.252096285	protein_coding	NM_001013734
ASHGV40029152	-0.25206	0.252063592	protein_coding	NM_144563
ASHGV40042148	-0.25195	0.25194706	protein_coding	NM_139281
ASHGV40013599	0.251928	0.251927766	protein_coding	NM_002271
ASHGV40032610	0.25191	0.251909857	protein_coding	NM_001924
ASHGV40014975	-0.25179	0.251793066	protein_coding	NM_006365
ASHGV40056406	-0.25177	0.251767641	protein_coding	NM_199290

ASHGV40047777	0.251713	0.251712655	protein_coding	NM_006555
ASHGV40020029	0.251692	0.251691662	protein_coding	NM_001113434
ASHGV40035495	-0.25164	0.25163769	protein_coding	NM_152617
ASHGV40023695	-0.25161	0.251610948	protein_coding	NM_130807
ASHGV40045337	0.251557	0.251556895	protein_coding	NM_016230
ASHGV40021747	0.251259	0.251259192	protein_coding	NM_025233
ASHGV40013921	0.251177	0.251176558	protein_coding	NM_001083893
ASHGV40015659	0.251038	0.251037859	protein_coding	NM_130901
ASHGV40038537	0.250989	0.250989237	protein_coding	NM_015274
ASHGV40003167	0.250777	0.250777387	protein_coding	NM_001199823
ASHGV40024994	0.250323	0.250323176	protein_coding	NM_015675
ASHGV40010862	0.250183	0.250182879	protein_coding	NM_001048210
ASHGV40025526	-0.25014	0.250144257	protein_coding	NM_018025
ASHGV40054588	0.250114	0.250114041	protein_coding	NM_014235
ASHGV40030428	-0.24997	0.249972118	protein_coding	NM_144628
ASHGV40057610	0.249919	0.249919236	protein_coding	NM_007157
ASHGV40044861	-0.24987	0.249865593	protein_coding	NM_005931
ASHGV40009125	0.249796	0.249795737	protein_coding	NM_022761
ASHGV40009875	0.249755	0.249755217	protein_coding	NM_018976
ASHGV40017117	0.249748	0.249748175	protein_coding	NM_004136
ASHGV40041987	0.249694	0.249693787	protein_coding	NM_022406
ASHGV40027613	0.249499	0.249498788	protein_coding	NM_152528
ASHGV40026962	-0.24942	0.249419857	protein_coding	NM_007260
ASHGV40014990	-0.24942	0.249419112	protein_coding	NM_053064
ASHGV40023882	0.249214	0.249214304	protein_coding	NM_133452
ASHGV40037417	0.249184	0.24918435	protein_coding	NM_006068
ASHGV40018432	-0.24913	0.249126416	protein_coding	NM_000512
ASHGV40023810	-0.24912	0.249124596	protein_coding	NM_174895
ASHGV40041027	0.248856	0.248855548	protein_coding	NM_004045
ASHGV40047660	0.248786	0.248786313	protein_coding	NM_007357
ASHGV40024630	0.24878	0.248780029	protein_coding	NM_018111
ASHGV40019648	0.248679	0.24867864	protein_coding	NM_182705
ASHGV40047832	-0.24868	0.248676988	protein_coding	ENST00000596947
ASHGV40047217	0.248636	0.248635594	protein_coding	NM_013446
ASHGV40053206	0.248619	0.248619224	protein_coding	NM_018946
ASHGV40044640	0.248458	0.248458378	protein_coding	NM_014827
ASHGV40035979	0.248414	0.248414058	protein_coding	NM_015926
ASHGV40009704	-0.24825	0.248248683	protein_coding	NM_006940
ASHGV40009757	0.248225	0.24822519	protein_coding	NM_198965
ASHGV40021043	0.24817	0.248170168	protein_coding	NM_019613
ASHGV40019627	0.248165	0.248165137	protein_coding	NM_014972
ASHGV40016723	-0.24815	0.248149316	protein_coding	NM_001012969
ASHGV40046430	0.248103	0.248103422	protein_coding	NM_002787
ASHGV40036285	-0.24803	0.248034661	protein_coding	NM_032787
ASHGV40043187	0.248012	0.248012492	protein_coding	NM_182701
ASHGV40033918	-0.24789	0.247889912	protein_coding	NM_017584
ASHGV40027919	-0.24782	0.247818533	protein_coding	NM_017846

ASHGV40020906	-0.24778	0.247781354	protein_coding	NM_015167
ASHGV40056255	-0.24756	0.247561024	protein_coding	uc002dey.2
ASHGV40019816	-0.24752	0.247522124	protein_coding	NM_032580
ASHGV40043543	-0.2475	0.247499694	protein_coding	NM_003599
ASHGV40055424	-0.24746	0.247464453	protein_coding	NM_006359
ASHGV40047489	-0.24742	0.247415034	protein_coding	NM_003088
ASHGV40047363	0.247394	0.247394192	protein_coding	ENST00000401694
ASHGV40009942	0.247316	0.247315859	protein_coding	NM_016357
ASHGV40051735	0.247289	0.247288728	protein_coding	NM_016042
ASHGV40039716	0.247278	0.247278052	protein_coding	NM_001564
ASHGV40038937	-0.24726	0.247264029	protein_coding	NM_005612
ASHGV40030152	0.247201	0.247200735	protein_coding	NM_018089
ASHGV40055762	0.247074	0.247073917	protein_coding	NM_198040
ASHGV40032736	-0.24702	0.24701671	protein_coding	NM_003274
ASHGV40056716	0.247016	0.247015956	protein_coding	NM_000983
ASHGV40035194	0.246864	0.246863948	protein_coding	NM_000055
ASHGV40010896	-0.24686	0.246860992	protein_coding	NM_001010985
ASHGV40049206	-0.2468	0.246803622	protein_coding	NM_003012
ASHGV40041861	-0.24669	0.246690771	protein_coding	NM_002538
ASHGV40035452	0.24669	0.246689678	protein_coding	NM_024524
ASHGV40044484	-0.24667	0.246666255	protein_coding	NM_033260
ASHGV40048446	-0.24662	0.246615504	protein_coding	NM_001195150
ASHGV40039477	-0.24646	0.24646233	protein_coding	NM_001957
ASHGV40015048	0.246454	0.246454429	protein_coding	NM_002788
ASHGV40046649	-0.24632	0.246318407	protein_coding	uc011kej.2
ASHGV40028505	-0.24632	0.246316054	protein_coding	NM_182500
ASHGV40038500	-0.24632	0.246315314	protein_coding	NM_001528
ASHGV40034381	0.246178	0.246178423	protein_coding	NM_006545
ASHGV40042351	-0.24601	0.246011326	protein_coding	NM_022371
ASHGV40031899	0.245962	0.245962139	protein_coding	NM_198935
ASHGV40051493	0.245943	0.2459431	protein_coding	NM_000170
ASHGV40017070	0.245929	0.245929029	protein_coding	NM_001145224
ASHGV40026770	0.245818	0.24581777	protein_coding	NM_033109
ASHGV40055625	-0.24573	0.245733347	protein_coding	NM_004679
ASHGV40017847	0.245725	0.245724679	protein_coding	NM_024096
ASHGV40032100	0.245492	0.245491733	protein_coding	NM_017446
ASHGV40030167	0.245444	0.245443963	protein_coding	NM_005070
ASHGV40000560	-0.24525	0.245250474	protein_coding	ENST00000423372
ASHGV40015504	0.245165	0.245165412	protein_coding	NM_001134233
ASHGV40057363	0.245008	0.245008429	protein_coding	NM_003378
ASHGV40051384	0.244912	0.244912043	protein_coding	NM_001082480
ASHGV40011481	0.244669	0.244669166	protein_coding	NM_000924
ASHGV40043739	0.24445	0.244450074	protein_coding	NM_181714
ASHGV40021528	-0.24437	0.244368922	protein_coding	NM_032141
ASHGV40056380	-0.2442	0.244200044	protein_coding	uc031rad.1
ASHGV40054450	-0.24409	0.244088961	protein_coding	NM_001078173
ASHGV40057159	0.244069	0.244068629	protein_coding	ENST00000519717

ASHGV40006142	-0.24404	0.244042355	protein_coding	NM_006951
ASHGV40046170	0.243972	0.243972285	protein_coding	NM_001135924
ASHGV40015184	-0.24391	0.24390714	protein_coding	NM_025057
ASHGV40029523	-0.24388	0.243879971	protein_coding	NM_080386
ASHGV40053341	0.243849	0.243848534	protein_coding	NM_004888
ASHGV40043514	-0.24378	0.243778323	protein_coding	NM_014345
ASHGV40014846	-0.24374	0.243739707	protein_coding	NM_005249
ASHGV40035430	0.243733	0.243732655	protein_coding	NM_178496
ASHGV40040694	0.243673	0.243672608	protein_coding	ENST00000343348
ASHGV40006450	-0.24366	0.243659852	protein_coding	NM_001098787
ASHGV40027776	-0.2436	0.243600553	protein_coding	NM_152520
ASHGV40011860	-0.2436	0.243598377	protein_coding	NM_152905
ASHGV40017668	-0.24358	0.243583017	protein_coding	NM_001079528
ASHGV40030964	-0.24334	0.243340621	protein_coding	NM_017453
ASHGV40024434	0.243282	0.24328194	protein_coding	NM_152296
ASHGV40013227	-0.24327	0.243268469	protein_coding	NM_001012754
ASHGV40019477	0.243222	0.243221862	protein_coding	NM_198390
ASHGV40021288	0.243086	0.243085873	protein_coding	NM_004246
ASHGV40054727	0.242955	0.242955023	protein_coding	NM_032796
ASHGV40013226	-0.24274	0.2427353	protein_coding	NM_207361
ASHGV40056323	-0.24266	0.242662896	protein_coding	NM_031965
ASHGV40019704	0.242637	0.242636775	protein_coding	NM_002208
ASHGV40010026	0.242561	0.242561222	protein_coding	NM_014311
ASHGV40022085	-0.24236	0.242359866	protein_coding	NM_138363
ASHGV40052434	-0.24222	0.242223039	protein_coding	NM_014581
ASHGV40047146	-0.24222	0.24221506	protein_coding	NM_018718
ASHGV40006706	0.242206	0.242206254	protein_coding	NM_000525
ASHGV40034075	0.242152	0.242152391	protein_coding	NM_007358
ASHGV40029998	-0.24209	0.242094313	protein_coding	NM_005759
ASHGV40008569	0.241929	0.24192921	protein_coding	NM_138471
ASHGV40024622	0.241823	0.241823442	protein_coding	NM_002103
ASHGV40034656	-0.24161	0.241605583	protein_coding	NM_080927
ASHGV40055516	-0.2415	0.241495789	protein_coding	NM_005342
ASHGV40025008	0.241292	0.241292024	protein_coding	NM_002067
ASHGV40048587	-0.24123	0.241226566	protein_coding	NM_015917
ASHGV40045076	0.241186	0.241186332	protein_coding	NM_001146016
ASHGV40025571	0.24118	0.241180495	protein_coding	NM_021175
ASHGV40028130	0.241115	0.241114828	protein_coding	NM_020830
ASHGV40008368	-0.24105	0.241046656	protein_coding	NM_052854
ASHGV40035932	-0.241	0.241004673	protein_coding	NM_005529
ASHGV40015142	-0.24091	0.240911712	protein_coding	NM_018375
ASHGV40019115	0.240848	0.240847588	protein_coding	NM_001080432
ASHGV40021566	-0.2406	0.240603006	protein_coding	NM_003885
ASHGV40045486	-0.24059	0.240591041	protein_coding	NM_003269
ASHGV40014074	0.240572	0.24057171	protein_coding	NM_020784
ASHGV40011757	-0.24053	0.240528742	protein_coding	NM_032230
ASHGV40048110	0.240522	0.240522384	protein_coding	NM_012449

ASHGV40051082	-0.24024	0.24024316	protein_coding	ENST00000499579
ASHGV40040144	-0.24012	0.240120218	protein_coding	NM_001127173
ASHGV40050505	-0.24006	0.240064027	protein_coding	NM_001031836
ASHGV40052164	0.239814	0.239813588	protein_coding	uc004bdy.2
ASHGV40002509	-0.23966	0.239660104	protein_coding	ENST00000586517
ASHGV40021936	-0.23963	0.239633322	protein_coding	NM_018896
ASHGV40025655	0.239565	0.239564781	protein_coding	NM_174905
ASHGV40043182	0.23954	0.239539817	protein_coding	NM_030899
ASHGV40003229	0.23944	0.239439857	protein_coding	NM_001302350
ASHGV40039618	0.23941	0.239410405	protein_coding	NM_014655
ASHGV40050122	0.239287	0.239286634	protein_coding	ENST00000377412
ASHGV40054240	-0.23925	0.239251823	protein_coding	NM_000169
ASHGV40017143	-0.23917	0.239171253	protein_coding	NM_000137
ASHGV40060854	-0.23904	0.239040021	protein_coding	uc009vmq.3
ASHGV40021223	-0.23894	0.238939506	protein_coding	NM_000937
ASHGV40035863	0.238929	0.23892897	protein_coding	NM_016006
ASHGV40037992	-0.23893	0.2389287	protein_coding	NM_144697
ASHGV40034374	0.23891	0.238910251	protein_coding	NM_003549
ASHGV40038527	-0.23891	0.238906641	protein_coding	NM_153717
ASHGV40001336	0.238726	0.238725936	protein_coding	NM_003288
ASHGV40008361	0.238697	0.238696728	protein_coding	NM_005456
ASHGV40024799	-0.23866	0.238656885	protein_coding	NM_206828
ASHGV40014013	0.238656	0.238656372	protein_coding	NM_018353
ASHGV40023222	0.238501	0.238501402	protein_coding	NM_018439
ASHGV40017118	-0.23847	0.238473313	protein_coding	NM_001013619
ASHGV40018170	-0.23838	0.238384308	protein_coding	NM_025082
ASHGV40025926	0.238366	0.238366361	protein_coding	NM_024682
ASHGV40035738	-0.23837	0.238365345	protein_coding	NM_001003793
ASHGV40023866	0.238352	0.238351536	protein_coding	NM_058164
ASHGV40052616	0.23826	0.238260164	protein_coding	NM_033516
ASHGV40037633	0.238241	0.238240609	protein_coding	NM_002090
ASHGV40018725	-0.23823	0.238225914	protein_coding	NM_016561
ASHGV40007236	0.238204	0.238204411	protein_coding	NM_005995
ASHGV40056565	-0.23818	0.238175088	protein_coding	NM_000511
ASHGV40026489	-0.23806	0.238060313	protein_coding	NM_000182
ASHGV40023678	-0.23801	0.238011391	protein_coding	uc002lsz.2
ASHGV40050519	0.237932	0.237932021	protein_coding	NM_007198
ASHGV40010883	0.237859	0.237858567	protein_coding	NM_001170738
ASHGV40019783	-0.23784	0.237843173	protein_coding	NM_015343
ASHGV40027252	0.23778	0.237779997	protein_coding	NM_207181
ASHGV40006488	-0.23763	0.237626022	protein_coding	NM_023947
ASHGV40038720	0.237358	0.237358434	protein_coding	NM_005349
ASHGV40000725	-0.23721	0.237209465	protein_coding	NM_001207052
ASHGV40052159	-0.23719	0.237194176	protein_coding	NM_006686
ASHGV40016817	0.237106	0.237105575	protein_coding	NM_002748
ASHGV40039102	-0.23708	0.237081725	protein_coding	NM_025207
ASHGV40011109	0.237025	0.237024844	protein_coding	NM_015954

ASHGV40018333	-0.23697	0.236968852	protein_coding	ENST00000299709
ASHGV40043405	-0.23683	0.236827096	protein_coding	NM_016135
ASHGV40051138	0.236823	0.236822988	protein_coding	NM_002514
ASHGV40048366	0.236803	0.236802679	protein_coding	NM_019644
ASHGV40007231	0.236761	0.236760876	protein_coding	NM_016366
ASHGV40023471	0.236725	0.236724749	protein_coding	NM_017742
ASHGV40052379	-0.23671	0.236713868	protein_coding	NM_019619
ASHGV40045794	-0.23659	0.23659334	protein_coding	NM_024694
ASHGV40020244	-0.23635	0.236350271	protein_coding	NM_017748
ASHGV40035755	0.236342	0.23634219	protein_coding	NM_015141
ASHGV40043064	0.236299	0.236299109	protein_coding	NM_032122
ASHGV40000155	-0.23625	0.236245267	protein_coding	ENST00000375223
ASHGV40011777	0.236114	0.236113695	protein_coding	NM_181783
ASHGV40024736	0.235999	0.235999482	protein_coding	NM_018300
ASHGV40024011	0.235992	0.235992359	protein_coding	NM_058243
ASHGV40045432	0.235932	0.235932299	protein_coding	NM_001195131
ASHGV40020804	-0.23591	0.235909425	protein_coding	NM_012121
ASHGV40018973	0.235896	0.235896401	protein_coding	NM_003414
ASHGV40036641	0.235678	0.235678207	protein_coding	NM_003412
ASHGV40009302	0.235629	0.235628731	protein_coding	NM_001005198
ASHGV40018250	-0.23561	0.235610472	protein_coding	NM_001270601
ASHGV40009233	-0.23555	0.235548746	protein_coding	NM_022169
ASHGV40052009	-0.23545	0.235445135	protein_coding	NM_004148
ASHGV40034046	0.235407	0.235406524	protein_coding	NM_000969
ASHGV40030267	-0.23531	0.235312751	protein_coding	NM_004846
ASHGV40016257	0.235238	0.235238379	protein_coding	NM_199330
ASHGV40055514	0.235213	0.235212547	protein_coding	NM_003828
ASHGV40025677	0.235071	0.23507145	protein_coding	NM_018028
ASHGV40055379	-0.23498	0.234980899	protein_coding	NM_006649
ASHGV40031218	0.234889	0.234889271	protein_coding	NM_014723
ASHGV40040683	-0.23483	0.234827971	protein_coding	NM_001801
ASHGV40037938	-0.23471	0.234706964	protein_coding	NM_198179
ASHGV40056982	-0.2347	0.234703448	protein_coding	NM_001193282
ASHGV40021302	-0.23465	0.23464808	protein_coding	NM_001173461
ASHGV40023662	-0.23437	0.234370351	protein_coding	NM_138774
ASHGV40029031	-0.23436	0.234361099	protein_coding	NM_001287491
ASHGV40031501	-0.23427	0.234274693	protein_coding	NM_025227
ASHGV40015341	0.233959	0.233959477	protein_coding	NM_020818
ASHGV40000957	0.233953	0.233952957	protein_coding	ENST00000442729
ASHGV40003102	-0.23393	0.233930647	protein_coding	NM_001042376
ASHGV40055524	0.2338	0.233799551	protein_coding	NM_033085
ASHGV40051178	0.23371	0.233710475	protein_coding	NM_144963
ASHGV40023883	-0.23361	0.233608069	protein_coding	NM_002162
ASHGV40052867	0.233442	0.233441987	protein_coding	ENST00000377437
ASHGV40026571	0.233324	0.233323537	protein_coding	NM_015955
ASHGV40014203	0.233091	0.23309088	protein_coding	NM_007113
ASHGV40012571	0.233022	0.233022385	protein_coding	NM_018191

ASHGV40034027	0.233018	0.233017698	protein_coding	NM_004628
ASHGV40022329	-0.233	0.233003999	protein_coding	NM_020954
ASHGV40032683	0.232928	0.232927751	protein_coding	NM_001001713
ASHGV40023911	-0.23277	0.232768993	protein_coding	NM_145045
ASHGV40022943	0.232711	0.232710948	protein_coding	NM_001142958
ASHGV40054363	-0.2327	0.23269627	protein_coding	NM_080632
ASHGV40036071	-0.23229	0.232287454	protein_coding	NM_000333
ASHGV40024628	-0.23223	0.232227298	protein_coding	NM_006179
ASHGV40054635	-0.23221	0.232208164	protein_coding	ENST00000457435
ASHGV40015553	0.232205	0.232204744	protein_coding	NM_174891
ASHGV40025885	0.232045	0.232044655	protein_coding	NM_014475
ASHGV40011486	-0.23203	0.232029551	protein_coding	NM_058173
ASHGV40052340	-0.23202	0.232024317	protein_coding	NM_001135219
ASHGV40035603	-0.23189	0.231892221	protein_coding	NM_000551
ASHGV40054000	-0.23176	0.231756367	protein_coding	NM_007213
ASHGV40012282	-0.23173	0.231726471	protein_coding	NM_019591
ASHGV40024605	-0.2317	0.231701927	protein_coding	NM_000979
ASHGV40041227	0.231689	0.231689144	protein_coding	NM_000505
ASHGV40049093	0.231509	0.231508829	protein_coding	NM_002095
ASHGV40017233	-0.2315	0.231501097	protein_coding	NM_001135
ASHGV40033768	0.231485	0.231484878	protein_coding	NM_014248
ASHGV40010426	0.231206	0.23120589	protein_coding	NM_021229
ASHGV40023631	-0.23109	0.231087959	protein_coding	NM_004715
ASHGV40020378	0.231001	0.231000724	protein_coding	NM_001261434
ASHGV40025178	0.230709	0.230708703	protein_coding	NM_001136482
ASHGV40032163	-0.23069	0.230690988	protein_coding	NM_181604
ASHGV40047096	0.230536	0.230536217	protein_coding	NM_024523
ASHGV40026504	0.230369	0.230368973	protein_coding	NM_013388
ASHGV40047458	0.230309	0.230308518	protein_coding	NM_001287501
ASHGV40012075	-0.23007	0.23006815	protein_coding	NM_194286
ASHGV40019596	0.230056	0.230056474	protein_coding	NM_001290330
ASHGV40008587	0.229815	0.229814931	protein_coding	NM_004470
ASHGV40038223	0.229731	0.229730679	protein_coding	NM_001100389
ASHGV40006898	0.229561	0.229560809	protein_coding	NM_020826
ASHGV40024969	0.229545	0.229545002	protein_coding	NM_017891
ASHGV40052404	0.22936	0.229359703	protein_coding	NM_032843
ASHGV40025798	0.229335	0.229334856	protein_coding	NM_000483
ASHGV40013564	0.229274	0.229273749	protein_coding	NM_004466
ASHGV40056219	-0.22925	0.229254734	protein_coding	uc001dcb.1
ASHGV40021780	-0.2292	0.22919658	protein_coding	NM_004941
ASHGV40012575	-0.22914	0.229138276	protein_coding	NM_002267
ASHGV40010600	0.229097	0.22909741	protein_coding	NM_138341
ASHGV40047500	0.229068	0.229068495	protein_coding	NM_018106
ASHGV40056583	0.22903	0.229029833	protein_coding	uc001esq.1
ASHGV40028657	-0.22891	0.228907904	protein_coding	NM_001486
ASHGV40041681	-0.22862	0.228617527	protein_coding	NM_153361
ASHGV40018134	0.228591	0.228591217	protein_coding	NM_004614

ASHGV40030497	0.22856	0.228560285	protein_coding	NM_002482
ASHGV40055931	-0.2285	0.22850212	protein_coding	NM_006993
ASHGV40006394	0.228451	0.2284513	protein_coding	NM_002412
ASHGV40018743	-0.22842	0.228415563	protein_coding	NM_004996
ASHGV40034869	0.228366	0.228366016	protein_coding	NM_003707
ASHGV40017052	0.228293	0.228292803	protein_coding	NM_025055
ASHGV40011375	0.228257	0.228257371	protein_coding	NM_052879
ASHGV40047734	0.228187	0.22818726	protein_coding	NM_032016
ASHGV40048235	-0.22812	0.228116466	protein_coding	NM_005273
ASHGV40050295	0.228076	0.228075722	protein_coding	NM_006765
ASHGV40035490	0.22805	0.228050008	protein_coding	NM_001039617
ASHGV40045064	0.228018	0.228018249	protein_coding	NM_033112
ASHGV40045792	-0.22796	0.227959813	protein_coding	NM_006834
ASHGV40006531	0.227834	0.227834015	protein_coding	NM_001130520
ASHGV40021389	-0.2278	0.227800787	protein_coding	NM_031294
ASHGV40020367	-0.22742	0.227416892	protein_coding	NM_001991
ASHGV40050191	0.22721	0.227209867	protein_coding	NM_014629
ASHGV40025358	-0.22702	0.227016721	protein_coding	NM_017712
ASHGV40052427	-0.22697	0.226967094	protein_coding	uc004cbw.1
ASHGV40038722	-0.22688	0.226883978	protein_coding	NM_018317
ASHGV40026573	-0.22675	0.226746407	protein_coding	NM_021209
ASHGV40052209	0.226657	0.226657095	protein_coding	NM_030767
ASHGV40055185	0.226622	0.226622342	protein_coding	NM_006729
ASHGV40004901	-0.22661	0.226613196	protein_coding	NM_001167604
ASHGV40032533	-0.22651	0.226507628	protein_coding	NM_001077701
ASHGV40057291	0.226165	0.226164533	protein_coding	NM_005738
ASHGV40015067	-0.2261	0.226098313	protein_coding	NM_007374
ASHGV40016673	0.225967	0.225967001	protein_coding	NM_001159508
ASHGV40015576	0.225914	0.225914481	protein_coding	NM_052868
ASHGV40025862	-0.22581	0.225807626	protein_coding	NM_199341
ASHGV40021384	-0.22578	0.225776996	protein_coding	NM_018019
ASHGV40048014	0.225734	0.225733981	protein_coding	ENST00000361825
ASHGV40014295	-0.22572	0.22572046	protein_coding	NM_005050
ASHGV40013106	0.225655	0.225655338	protein_coding	NM_001676
ASHGV40024656	-0.22558	0.225577479	protein_coding	NM_052884
ASHGV40038112	0.225444	0.225444122	protein_coding	NM_000901
ASHGV40019155	-0.2254	0.225400845	protein_coding	NM_005946
ASHGV40054954	0.225381	0.225380891	protein_coding	NM_005710
ASHGV40040872	0.22536	0.225360344	protein_coding	NM_004730
ASHGV40026929	-0.22528	0.225283197	protein_coding	NM_000975
ASHGV40041354	0.225276	0.225275976	protein_coding	NM_052862
ASHGV40020335	0.225242	0.225241699	protein_coding	NM_177977
ASHGV40024519	-0.22514	0.225142572	protein_coding	NM_000400
ASHGV40049005	-0.22489	0.224888383	protein_coding	NM_004430
ASHGV40006688	0.224708	0.224708419	protein_coding	NM_003684
ASHGV40032247	-0.22448	0.224480534	protein_coding	NM_006052
ASHGV40051468	-0.22445	0.224445835	protein_coding	NM_152629

ASHGV40030020	-0.22427	0.224274024	protein_coding	NM_001959
ASHGV40033649	0.224009	0.224008793	protein_coding	NM_000362
ASHGV40022042	-0.22368	0.22367615	protein_coding	NM_024612
ASHGV40017086	-0.22362	0.223622855	protein_coding	NM_152335
ASHGV40022055	0.22346	0.223460234	protein_coding	NM_001099432
ASHGV40033467	-0.22313	0.223134347	protein_coding	NM_001010971
ASHGV40035143	0.222948	0.222947915	protein_coding	NM_020307
ASHGV40000143	0.222872	0.222871985	protein_coding	NM_001190880
ASHGV40052929	-0.22282	0.222816172	protein_coding	NM_000144
ASHGV40055949	0.22275	0.22274954	protein_coding	NM_012229
ASHGV40052547	-0.22267	0.222672964	protein_coding	NM_001033113
ASHGV40035628	0.222664	0.222663828	protein_coding	NM_024827
ASHGV40003184	-0.22266	0.222662612	protein_coding	NM_001204890
ASHGV40025910	-0.22254	0.222538265	protein_coding	NM_004107
ASHGV40009340	0.222531	0.222530998	protein_coding	NM_024556
ASHGV40002696	-0.22252	0.222523625	protein_coding	ENST00000601918
ASHGV40017460	-0.22239	0.222394595	protein_coding	NM_001272051
ASHGV40015748	-0.22233	0.222327122	protein_coding	NM_018163
ASHGV40041804	-0.22227	0.22227166	protein_coding	NM_020928
ASHGV40020397	0.222217	0.222217415	protein_coding	NM_001278374
ASHGV40008550	0.222185	0.222184794	protein_coding	NM_199337
ASHGV40036096	-0.22216	0.22215584	protein_coding	uc003dnf.2
ASHGV40017608	-0.22215	0.222148084	protein_coding	NM_001079512
ASHGV40032824	0.221957	0.221956682	protein_coding	NM_015241
ASHGV40050134	0.221729	0.221728608	protein_coding	NM_130849
ASHGV40034154	-0.22155	0.221551477	protein_coding	NM_015551
ASHGV40038856	-0.22148	0.221483309	protein_coding	NM_020453
ASHGV40044724	-0.2214	0.221400542	protein_coding	NM_006355
ASHGV40050891	-0.22133	0.221331314	protein_coding	NM_003638
ASHGV40024553	-0.22128	0.221277983	protein_coding	NM_032040
ASHGV40008900	-0.22128	0.221276989	protein_coding	NM_024684
ASHGV40026092	-0.2212	0.221199136	protein_coding	NM_001130072
ASHGV40015393	-0.22117	0.221174464	protein_coding	NM_152327
ASHGV40015936	-0.22113	0.221133371	protein_coding	NM_004330
ASHGV40052332	0.221071	0.221071171	protein_coding	NM_001134430
ASHGV40048306	0.22093	0.220929934	protein_coding	NM_000441
ASHGV40020825	-0.22091	0.220905958	protein_coding	NM_024319
ASHGV40048584	-0.22081	0.220814375	protein_coding	NM_176882
ASHGV40007579	0.220619	0.220618844	protein_coding	NM_152434
ASHGV40021664	-0.22053	0.220534943	protein_coding	NM_001136498
ASHGV40015189	0.22028	0.220280427	protein_coding	NM_194279
ASHGV40010055	-0.22025	0.220247996	protein_coding	NM_032345
ASHGV40031930	-0.22021	0.220208425	protein_coding	NM_139317
ASHGV40025040	-0.22007	0.220070023	protein_coding	NM_005483
ASHGV40038089	0.219989	0.219989488	protein_coding	NM_017493
ASHGV40023966	-0.21996	0.219959757	protein_coding	NM_031921
ASHGV40027139	0.219938	0.219938427	protein_coding	NM_016316

ASHGV40042843	0.21991	0.219909519	protein_coding	NM_001357
ASHGV40040935	-0.2199	0.219896327	protein_coding	NM_001127496
ASHGV40043609	-0.21964	0.219638557	protein_coding	NM_014920
ASHGV40008511	-0.21958	0.219577984	protein_coding	NM_001079807
ASHGV40009911	0.219539	0.219538848	protein_coding	NM_004818
ASHGV40044831	0.219502	0.219502474	protein_coding	NM_021253
ASHGV40052549	0.219381	0.219381111	protein_coding	uc004cnd.1
ASHGV40040448	0.21931	0.219310296	protein_coding	NM_178276
ASHGV40038704	-0.21927	0.219270518	protein_coding	NM_018323
ASHGV40035445	0.219161	0.219161267	protein_coding	NM_000561
ASHGV40024740	-0.21901	0.219009892	protein_coding	NM_198457
ASHGV40025030	-0.21898	0.218978893	protein_coding	NM_001105562
ASHGV40024492	0.218661	0.218661023	protein_coding	NM_004234
ASHGV40008700	-0.21864	0.218637045	protein_coding	NM_024036
ASHGV40019798	0.218455	0.218455463	protein_coding	NM_006942
ASHGV40014451	0.21844	0.218440093	protein_coding	NM_004755
ASHGV40029995	-0.21836	0.218358228	protein_coding	NM_024744
ASHGV40033993	-0.21824	0.21823672	protein_coding	NM_016362
ASHGV40017744	-0.21817	0.218173234	protein_coding	NM_001083614
ASHGV40019748	-0.21799	0.217990419	protein_coding	NM_002532
ASHGV40043816	0.217977	0.217976896	protein_coding	NM_014611
ASHGV40037824	-0.21759	0.217591372	protein_coding	NM_005908
ASHGV40054320	-0.21757	0.217568687	protein_coding	ENST00000372054
ASHGV40038494	-0.21749	0.217487007	protein_coding	NM_001042690
ASHGV40018772	0.217267	0.217266978	protein_coding	ENST00000367188
ASHGV40030101	0.217027	0.217027499	protein_coding	NM_021141
ASHGV40017699	-0.21694	0.216936707	protein_coding	NM_001012991
ASHGV40000764	0.216817	0.216817144	protein_coding	NM_138785
ASHGV40056373	0.216791	0.216791474	protein_coding	NM_000699
ASHGV40010104	0.216779	0.216778711	protein_coding	NM_032496
ASHGV40044415	0.216683	0.216683448	protein_coding	NM_024919
ASHGV40033117	0.216592	0.216591567	protein_coding	NM_001177701
ASHGV40042796	-0.21655	0.216552578	protein_coding	NM_022455
ASHGV40050586	-0.21652	0.216519676	protein_coding	NM_002690
ASHGV40006209	0.216487	0.216486524	protein_coding	NM_145206
ASHGV40008810	0.21639	0.216389695	protein_coding	NM_018320
ASHGV40025937	0.216387	0.216386979	protein_coding	NM_206538
ASHGV40055808	0.216359	0.216358545	protein_coding	NM_148977
ASHGV40049070	-0.21631	0.216310296	protein_coding	NM_015254
ASHGV40042833	0.216269	0.216269151	protein_coding	NM_058230
ASHGV40013874	0.216241	0.216240768	protein_coding	NM_139247
ASHGV40060881	0.216193	0.216192874	protein_coding	uc010usj.2
ASHGV40005166	-0.21607	0.216066664	protein_coding	NM_001143764
ASHGV40034938	-0.21605	0.216046529	protein_coding	NM_002958
ASHGV40043238	-0.216	0.216004411	protein_coding	NM_014641
ASHGV40006048	0.215975	0.21597452	protein_coding	NM_020349
ASHGV40006539	0.215765	0.215764805	protein_coding	NM_005387

ASHGV40019854	-0.21576	0.215756486	protein_coding	NM_001310219
ASHGV40046093	0.215592	0.2155921	protein_coding	NM_007353
ASHGV40055199	0.215499	0.215498839	protein_coding	NM_145170
ASHGV40045654	0.215399	0.215398584	protein_coding	NM_000426
ASHGV40057315	0.215051	0.215051431	protein_coding	NM_021130
ASHGV40034661	0.21505	0.21504988	protein_coding	NM_182909
ASHGV40048594	-0.21502	0.215020438	protein_coding	NM_177437
ASHGV40021696	0.214945	0.214945209	protein_coding	NM_001012241
ASHGV40030455	0.214804	0.214803667	protein_coding	NM_024325
ASHGV40039617	0.214797	0.214796533	protein_coding	NM_054016
ASHGV40052499	0.214622	0.214621879	protein_coding	NM_017617
ASHGV40031637	-0.21455	0.21455186	protein_coding	NM_016004
ASHGV40019719	0.214507	0.214507183	protein_coding	NM_003342
ASHGV40028270	-0.21444	0.214436162	protein_coding	NM_022449
ASHGV40023939	-0.21439	0.214392683	protein_coding	NM_001146685
ASHGV40014434	0.214388	0.214388126	protein_coding	NM_001085471
ASHGV40027943	0.214372	0.214371536	protein_coding	NM_018256
ASHGV40006984	-0.21417	0.214165745	protein_coding	NM_033396
ASHGV40017489	0.214127	0.21412667	protein_coding	NM_020764
ASHGV40009474	0.213768	0.213768337	protein_coding	NM_020374
ASHGV40014242	-0.21374	0.213737831	protein_coding	NM_183002
ASHGV40029980	0.213705	0.213704777	protein_coding	NM_003879
ASHGV40036902	-0.21368	0.213677253	protein_coding	NM_004423
ASHGV40023992	-0.21358	0.213583863	protein_coding	NM_005804
ASHGV40008584	-0.21358	0.213581171	protein_coding	NM_005528
ASHGV40052497	0.213548	0.213548381	protein_coding	NM_014866
ASHGV40041726	0.213415	0.213415363	protein_coding	NM_015946
ASHGV40051617	0.21329	0.213290098	protein_coding	NM_024761
ASHGV40052374	-0.21325	0.213250398	protein_coding	NM_203434
ASHGV40036868	-0.21306	0.213059041	protein_coding	NM_005087
ASHGV40048194	-0.21306	0.213055502	protein_coding	NM_001244580
ASHGV40015165	-0.21294	0.212938025	protein_coding	NM_004296
ASHGV40027614	-0.21293	0.212930911	protein_coding	NM_013450
ASHGV40008874	-0.21291	0.212912109	protein_coding	NM_032564
ASHGV40051486	0.212888	0.212887694	protein_coding	NM_024896
ASHGV40019174	0.212872	0.212872069	protein_coding	NM_133368
ASHGV40012263	0.212815	0.212814546	protein_coding	ENST00000330579
ASHGV40034008	0.212783	0.212783424	protein_coding	NM_138807
ASHGV40027142	-0.21273	0.212725735	protein_coding	NM_002285
ASHGV40043524	-0.21272	0.212722364	protein_coding	NM_018135
ASHGV40040658	0.212614	0.212613887	protein_coding	NM_139164
ASHGV40027306	0.212611	0.212610857	protein_coding	NM_019557
ASHGV40025899	-0.21248	0.212483312	protein_coding	NM_017636
ASHGV40033358	-0.2124	0.212398089	protein_coding	NM_001123225
ASHGV40026611	0.212079	0.21207912	protein_coding	NM_001032377
ASHGV40015972	-0.21184	0.211839933	protein_coding	NM_015920
ASHGV40007789	-0.21169	0.211692661	protein_coding	NM_019055

ASHGV40039446	0.211628	0.21162805	protein_coding	NM_032557
ASHGV40056929	-0.21142	0.21141825	protein_coding	NM_019069
ASHGV40016044	-0.21125	0.211253407	protein_coding	NM_006305
ASHGV40019133	0.211231	0.211230907	protein_coding	NM_032029
ASHGV40033717	-0.21122	0.211217005	protein_coding	uc003aup.3
ASHGV40015357	0.211144	0.211143566	protein_coding	NM_145249
ASHGV40039319	0.211132	0.211132254	protein_coding	NM_004784
ASHGV40001263	-0.21106	0.211057389	protein_coding	NM_001145312
ASHGV40025932	-0.21095	0.210953393	protein_coding	NM_001114600
ASHGV40053929	-0.21083	0.210828637	protein_coding	NM_004229
ASHGV40045796	-0.2108	0.210797045	protein_coding	NM_004672
ASHGV40036015	0.21079	0.210790021	protein_coding	NM_018725
ASHGV40009332	0.210762	0.210761681	protein_coding	NM_145014
ASHGV40042352	0.210734	0.210734158	protein_coding	NM_145282
ASHGV40025344	-0.21072	0.210724307	protein_coding	NM_138442
ASHGV40014196	0.210653	0.2106533	protein_coding	NM_002382
ASHGV40048234	-0.21061	0.210608461	protein_coding	NM_023948
ASHGV40053673	0.210545	0.210544925	protein_coding	NM_003731
ASHGV40048321	0.21039	0.210389518	protein_coding	NM_018334
ASHGV40019782	-0.21035	0.210347985	protein_coding	NM_007278
ASHGV40046150	0.210322	0.210321585	protein_coding	NM_002947
ASHGV40015039	0.210292	0.210291873	protein_coding	NM_018229
ASHGV40003258	-0.21026	0.210261171	protein_coding	NM_004125
ASHGV40024089	0.210184	0.210183835	protein_coding	NM_001205252
ASHGV40008519	-0.21001	0.210013645	protein_coding	NM_017841
ASHGV40039455	-0.20999	0.209993671	protein_coding	NM_002940
ASHGV40047737	-0.20995	0.209951316	protein_coding	NM_020192
ASHGV40028093	0.209717	0.209717205	protein_coding	NM_015311
ASHGV40028791	0.209659	0.209659097	protein_coding	NM_005095
ASHGV40007172	0.209601	0.209601147	protein_coding	NM_015913
ASHGV40050164	-0.20959	0.20958555	protein_coding	NM_173539
ASHGV40028868	0.209471	0.20947128	protein_coding	ENST00000403007
ASHGV40026917	-0.20945	0.209453675	protein_coding	NM_032601
ASHGV40005422	-0.20945	0.209449828	protein_coding	NM_017433
ASHGV40049440	0.209384	0.209384075	protein_coding	NM_014294
ASHGV40016868	0.209294	0.209294447	protein_coding	NM_000236
ASHGV40047598	0.209251	0.209251471	protein_coding	NM_000600
ASHGV40035084	0.20917	0.209169717	protein_coding	NM_014445
ASHGV40014680	0.209089	0.209088889	protein_coding	NM_007112
ASHGV40015798	0.208997	0.208997281	protein_coding	NM_032892
ASHGV40030276	0.208888	0.208888289	protein_coding	NM_030803
ASHGV40050359	-0.20888	0.208880837	protein_coding	NM_001128431
ASHGV40045687	-0.20871	0.208708967	protein_coding	NM_004100
ASHGV40028176	-0.20869	0.208693721	protein_coding	NM_139072
ASHGV40024785	0.208608	0.208608267	protein_coding	uc002qfi.3
ASHGV40018267	-0.20842	0.208421626	protein_coding	NM_001077418
ASHGV40051619	0.208412	0.208412215	protein_coding	NM_018325

ASHGV40046038	0.208404	0.208404165	protein_coding	NM_032448
ASHGV40008899	0.208393	0.208393212	protein_coding	NM_173039
ASHGV40012103	-0.20835	0.208345629	protein_coding	NM_000545
ASHGV40029895	0.20822	0.20822048	protein_coding	NM_005424
ASHGV40021595	-0.20814	0.208138111	protein_coding	NM_001304438
ASHGV40029917	-0.20814	0.208136622	protein_coding	NM_001031716
ASHGV40030044	-0.20809	0.208087748	protein_coding	NM_152523
ASHGV40023784	0.208057	0.208057225	protein_coding	NM_005934
ASHGV40025698	-0.20794	0.207940198	protein_coding	NM_002446
ASHGV40025678	0.207926	0.207926393	protein_coding	NM_017592
ASHGV40056421	0.207749	0.207749327	protein_coding	NM_003955
ASHGV40005568	0.207668	0.207668354	protein_coding	NM_003819
ASHGV40055501	-0.20758	0.207582642	protein_coding	NM_001164415
ASHGV40008553	0.207544	0.207543786	protein_coding	NM_002394
ASHGV40047475	0.207501	0.207501165	protein_coding	NM_021205
ASHGV40010914	0.207426	0.207425554	protein_coding	NM_001252499
ASHGV40052569	-0.20734	0.207343383	protein_coding	NM_203447
ASHGV40036975	0.207223	0.207223472	protein_coding	NM_002182
ASHGV40004914	-0.20708	0.207079986	protein_coding	NM_001195305
ASHGV40049766	-0.20658	0.206582967	protein_coding	NM_001568
ASHGV40035652	0.206569	0.206568677	protein_coding	NM_001291694
ASHGV40021248	-0.20651	0.2065096	protein_coding	NM_000180
ASHGV40047252	0.206464	0.206463527	protein_coding	ENST00000595842
ASHGV40041567	-0.20604	0.206040032	protein_coding	NM_004932
ASHGV40020493	-0.20589	0.205893555	protein_coding	NM_006807
ASHGV40045579	0.205806	0.20580615	protein_coding	NM_153711
ASHGV40018651	-0.20566	0.205661429	protein_coding	NM_001253790
ASHGV40036590	-0.20559	0.205588098	protein_coding	NM_022131
ASHGV40051771	-0.20555	0.205547429	protein_coding	NM_001290020
ASHGV40010698	0.205467	0.205466513	protein_coding	NM_001286191
ASHGV40053753	0.205351	0.205350723	protein_coding	NM_004650
ASHGV40024638	-0.20531	0.205312265	protein_coding	NM_178449
ASHGV40056459	0.205242	0.205242087	protein_coding	NM_032160
ASHGV40005046	-0.20502	0.205021498	protein_coding	NM_014661
ASHGV40043104	0.204865	0.204864834	protein_coding	NM_000948
ASHGV40019750	-0.20476	0.204757344	protein_coding	NM_020162
ASHGV40048010	-0.20434	0.204343076	protein_coding	NM_032999
ASHGV40010990	0.204306	0.20430627	protein_coding	NM_201650
ASHGV40032035	0.204302	0.204302494	protein_coding	NM_001100420
ASHGV40017472	-0.20426	0.204261121	protein_coding	NM_004970
ASHGV40042764	-0.20425	0.204251787	protein_coding	NM_002449
ASHGV40035872	0.204205	0.204204809	protein_coding	NM_173826
ASHGV40039604	-0.20371	0.203713678	protein_coding	NM_012464
ASHGV40035340	-0.20353	0.203530396	protein_coding	uc003fma.1
ASHGV40048866	-0.2035	0.203499349	protein_coding	NM_004225
ASHGV40017249	-0.20344	0.203439403	protein_coding	NM_001039958
ASHGV40050797	-0.20343	0.203425578	protein_coding	NM_001011720

ASHGV40050542	-0.20335	0.203349275	protein_coding	NM_021623
ASHGV40029868	0.203347	0.203346501	protein_coding	NM_194250
ASHGV40043077	-0.20317	0.203171253	protein_coding	NM_005124
ASHGV40034836	-0.20316	0.203160087	protein_coding	NM_024628
ASHGV40015989	0.203128	0.203128312	protein_coding	NM_000942
ASHGV40018430	0.203092	0.203091536	protein_coding	NM_001142864
ASHGV40049384	0.203081	0.203081064	protein_coding	NM_003878
ASHGV40024737	0.202986	0.202985579	protein_coding	NM_030972
ASHGV40035993	-0.20294	0.20294333	protein_coding	NM_001122870
ASHGV40005377	0.202837	0.202836717	protein_coding	NM_032812
ASHGV40052272	-0.20278	0.202782914	protein_coding	NM_033117
ASHGV40026993	0.202683	0.202683125	protein_coding	NM_178839
ASHGV40005067	-0.20265	0.202645698	protein_coding	NM_018180
ASHGV40027593	0.202564	0.202563643	protein_coding	NM_006186
ASHGV40020167	0.20226	0.202260385	protein_coding	NM_003132
ASHGV40024615	-0.20204	0.202044177	protein_coding	NM_001190
ASHGV40057252	-0.202	0.201998381	protein_coding	NM_002667
ASHGV40019435	0.201935	0.201934565	protein_coding	NM_001105663
ASHGV40049291	-0.20192	0.201923126	protein_coding	NM_000912
ASHGV40033514	0.201903	0.201903185	protein_coding	NM_005216
ASHGV40027077	0.201799	0.201799072	protein_coding	NM_031902
ASHGV40038450	0.201796	0.201796153	protein_coding	NM_021923
ASHGV40033103	-0.20177	0.201766059	protein_coding	NM_145640
ASHGV40044187	-0.2017	0.201702339	protein_coding	uc003qit.1
ASHGV40024403	-0.20169	0.201688575	protein_coding	NM_024876
ASHGV40023399	0.201677	0.201676858	protein_coding	NM_007195
ASHGV40030009	0.201623	0.201622871	protein_coding	NM_002840
ASHGV40018643	-0.20152	0.201518155	protein_coding	NM_138440
ASHGV40011106	0.2015	0.201499897	protein_coding	NM_002232
ASHGV40016130	-0.20118	0.201182189	protein_coding	NM_000781
ASHGV40056486	-0.20117	0.201174793	protein_coding	NM_001171155
ASHGV40055476	-0.20112	0.201122085	protein_coding	NM_004709
ASHGV40027544	-0.20105	0.201054708	protein_coding	NM_015702
ASHGV40020324	-0.20083	0.200834477	protein_coding	NM_003771
ASHGV40026862	-0.20056	0.200561526	protein_coding	NM_015013
ASHGV40026661	0.20026	0.200259873	protein_coding	NM_004718
ASHGV40016176	0.20016	0.20016045	protein_coding	ENST00000595430
ASHGV40025584	0.200138	0.200138406	protein_coding	NM_012231
ASHGV40053219	0.200093	0.200093179	protein_coding	NM_006981
ASHGV40014600	-0.19994	0.199940895	protein_coding	NM_032630
ASHGV40015727	0.199934	0.19993374	protein_coding	NM_007223
ASHGV40036134	-0.19986	0.199860587	protein_coding	NM_018029
ASHGV40011937	0.199796	0.199796041	protein_coding	NM_018157
ASHGV40029885	0.199695	0.199694961	protein_coding	NM_016315
ASHGV40048339	0.199643	0.199642999	protein_coding	NM_001166345
ASHGV40025043	0.199594	0.199594451	protein_coding	NM_001001520
ASHGV40015133	0.199437	0.199436987	protein_coding	NM_003975

ASHGV40056311	-0.19931	0.199309794	protein_coding	NM_022818
ASHGV40037792	0.199176	0.199176303	protein_coding	NM_000672
ASHGV40019210	-0.1991	0.199100045	protein_coding	NM_001160305
ASHGV40052513	-0.19908	0.199080625	protein_coding	NM_203347
ASHGV40038493	0.198997	0.198997259	protein_coding	ENST00000355072
ASHGV40055320	-0.19891	0.198911812	protein_coding	NM_001031855
ASHGV40025973	0.198835	0.198835327	protein_coding	NM_007147
ASHGV40053418	0.198692	0.198691679	protein_coding	NM_005294
ASHGV40055468	0.19853	0.198530455	protein_coding	NM_001009613
ASHGV40010383	-0.19851	0.198511254	protein_coding	NM_007035
ASHGV40043045	0.198374	0.198374306	protein_coding	NM_001031713
ASHGV40022895	0.198112	0.198112047	protein_coding	NM_021153
ASHGV40045359	-0.19807	0.198074232	protein_coding	NM_001031743
ASHGV40006916	0.198053	0.198052952	protein_coding	NM_004308
ASHGV40030315	-0.19799	0.197991995	protein_coding	NM_001037131
ASHGV40001291	0.197923	0.197923353	protein_coding	NM_020704
ASHGV40036540	0.197918	0.197918411	protein_coding	NM_015268
ASHGV40052495	-0.19789	0.197894476	protein_coding	NM_001039707
ASHGV40013393	-0.19787	0.197869153	protein_coding	NM_030794
ASHGV40054960	-0.19759	0.197585643	protein_coding	NM_014008
ASHGV40036736	0.197565	0.197564859	protein_coding	NM_022443
ASHGV40025633	0.197557	0.197557237	protein_coding	NM_001300993
ASHGV40012511	-0.19756	0.197556196	protein_coding	NM_033255
ASHGV40033108	0.197491	0.197490851	protein_coding	NM_002473
ASHGV40052002	0.197462	0.197461638	protein_coding	NM_022755
ASHGV40008445	-0.19744	0.197440501	protein_coding	NM_178570
ASHGV40055523	-0.19742	0.197421283	protein_coding	NM_024082
ASHGV40056694	-0.19734	0.197335923	protein_coding	NM_001099771
ASHGV40048918	0.197238	0.197238183	protein_coding	NM_001083537
ASHGV40048664	-0.19718	0.197181092	protein_coding	NM_175571
ASHGV40020102	0.197143	0.197142959	protein_coding	NM_078471
ASHGV40003171	0.197071	0.197070579	protein_coding	NM_001202485
ASHGV40054212	-0.19706	0.197060458	protein_coding	NM_014836
ASHGV40039457	-0.19704	0.197044943	protein_coding	NM_005900
ASHGV40051461	-0.19702	0.197023835	protein_coding	NM_014878
ASHGV40057789	-0.19698	0.196982416	protein_coding	NM_001005
ASHGV40056401	0.196816	0.196815665	protein_coding	NM_020703
ASHGV40055721	-0.1968	0.196795925	protein_coding	NM_018207
ASHGV40022946	0.196749	0.196748945	protein_coding	NM_148923
ASHGV40045168	-0.1966	0.196597407	protein_coding	NM_003582
ASHGV40034420	0.196554	0.196554195	protein_coding	NM_198563
ASHGV40039123	0.196534	0.196533883	protein_coding	NM_032717
ASHGV40014303	-0.19645	0.196450979	protein_coding	NM_001039479
ASHGV40022564	-0.19645	0.196450746	protein_coding	NM_052911
ASHGV40015674	0.196448	0.196448481	protein_coding	NM_024713
ASHGV40028512	0.196381	0.196380571	protein_coding	NM_145693
ASHGV40046686	-0.19631	0.196314717	protein_coding	NM_016086

ASHGV40025436	0.196254	0.196253908	protein_coding	NM_020855
ASHGV40035764	-0.19615	0.196146553	protein_coding	NM_015442
ASHGV40020349	0.196141	0.196140877	protein_coding	NM_032484
ASHGV40011327	-0.19602	0.196017523	protein_coding	ENST00000598930
ASHGV40035095	-0.196	0.19600291	protein_coding	NM_176894
ASHGV40027915	-0.19592	0.195915535	protein_coding	NM_006190
ASHGV40045824	0.195879	0.195878518	protein_coding	NM_030949
ASHGV40035327	-0.19579	0.195785635	protein_coding	NM_031936
ASHGV40008510	0.195695	0.195694564	protein_coding	NM_014207
ASHGV40015933	0.195619	0.195618543	protein_coding	NM_004998
ASHGV40052876	0.195519	0.195519083	protein_coding	NM_006973
ASHGV40019422	-0.19551	0.195508082	protein_coding	NM_015658
ASHGV40051055	0.195485	0.195485065	protein_coding	NM_012082
ASHGV40006913	-0.19543	0.195434835	protein_coding	NM_000741
ASHGV40043292	-0.19515	0.195149171	protein_coding	NM_005510
ASHGV40014972	0.195036	0.195035742	protein_coding	NM_001003803
ASHGV40042775	0.194929	0.194929242	protein_coding	uc003mdu.1
ASHGV40034179	-0.19492	0.194920466	protein_coding	NM_006225
ASHGV40045748	-0.19486	0.19485728	protein_coding	NM_016217
ASHGV40043183	-0.1948	0.194799768	protein_coding	NM_000437
ASHGV40043901	-0.19475	0.194746919	protein_coding	NM_007073
ASHGV40033354	0.194676	0.194676379	protein_coding	NM_012302
ASHGV40051835	-0.19466	0.194661759	protein_coding	NM_013390
ASHGV40056427	-0.19434	0.194336613	protein_coding	NM_001081976
ASHGV40011186	0.194227	0.194226604	protein_coding	NM_001198915
ASHGV40031246	0.194208	0.194208147	protein_coding	NM_030811
ASHGV40023552	-0.19406	0.194064023	protein_coding	NM_017757
ASHGV40020140	0.193939	0.193938972	protein_coding	NM_018428
ASHGV40045620	-0.19373	0.193733021	protein_coding	NM_004506
ASHGV40041589	0.193728	0.193728276	protein_coding	NM_152295
ASHGV40021360	-0.19362	0.193617523	protein_coding	NM_018955
ASHGV40045435	-0.19357	0.193567673	protein_coding	NM_021620
ASHGV40035613	0.1935	0.193499722	protein_coding	NM_003042
ASHGV40051907	0.193487	0.193487276	protein_coding	NM_032307
ASHGV40032376	0.193465	0.193464933	protein_coding	NM_003343
ASHGV40003176	0.19324	0.193240244	protein_coding	NM_001204173
ASHGV40027399	0.193084	0.193083778	protein_coding	NM_024545
ASHGV40042564	0.192991	0.192991053	protein_coding	NM_001543
ASHGV40029827	0.192794	0.192794067	protein_coding	NM_152945
ASHGV40007695	0.192751	0.192751172	protein_coding	NM_174934
ASHGV40056101	0.192705	0.192704873	protein_coding	NM_002791
ASHGV40027276	-0.19268	0.192683374	protein_coding	NM_000575
ASHGV40042164	-0.19253	0.192534631	protein_coding	NM_003135
ASHGV40000098	0.192444	0.192444353	protein_coding	NM_024660
ASHGV40016688	0.192417	0.192417048	protein_coding	NM_020857
ASHGV40018735	-0.19234	0.192336024	protein_coding	NM_001128423
ASHGV40003053	-0.19233	0.192331206	protein_coding	NM_000915

ASHGV40013846	-0.19226	0.192261895	protein_coding	NM_005864
ASHGV40006346	-0.19217	0.192169818	protein_coding	NM_022126
ASHGV40015495	0.192044	0.192044201	protein_coding	NM_001267827
ASHGV40014347	0.191982	0.191982462	protein_coding	NM_213601
ASHGV40015109	-0.19192	0.1919177	protein_coding	ENST00000298705
ASHGV40031660	-0.19182	0.191817875	protein_coding	NM_022358
ASHGV40022400	0.191688	0.191687958	protein_coding	NM_017512
ASHGV40008099	0.191569	0.191568599	protein_coding	NM_014633
ASHGV40053566	0.191561	0.191561149	protein_coding	NM_012087
ASHGV40057347	0.191301	0.191301343	protein_coding	NM_001004689
ASHGV40004954	-0.19129	0.191287844	protein_coding	uc001lcv.2
ASHGV40033234	0.191123	0.191122702	protein_coding	NM_007229
ASHGV40053482	0.191002	0.19100211	protein_coding	NM_052901
ASHGV40016669	-0.19098	0.190979234	protein_coding	NM_033510
ASHGV40018961	-0.1909	0.190904332	protein_coding	NM_000632
ASHGV40022222	0.190783	0.190783227	protein_coding	NM_014738
ASHGV40018813	-0.19058	0.190579761	protein_coding	NM_006043
ASHGV40025628	0.190514	0.190513737	protein_coding	NM_144689
ASHGV40018238	-0.18988	0.189875213	protein_coding	NM_006885
ASHGV40014448	0.189852	0.189852234	protein_coding	NM_001010854
ASHGV40014562	-0.18984	0.189838421	protein_coding	NM_181715
ASHGV40050148	-0.18944	0.189442714	protein_coding	NM_030580
ASHGV40032524	0.189402	0.189402269	protein_coding	NM_002040
ASHGV40036088	0.189396	0.189395842	protein_coding	NM_032505
ASHGV40049279	-0.1894	0.189395601	protein_coding	NM_144651
ASHGV40026973	0.189332	0.189332418	protein_coding	NM_003203
ASHGV40057429	-0.18915	0.189153343	protein_coding	NM_001206847
ASHGV40027294	-0.18907	0.189067428	protein_coding	NM_025181
ASHGV40046064	0.189009	0.189009282	protein_coding	NM_032350
ASHGV40022365	-0.18899	0.188992338	protein_coding	NM_005052
ASHGV40055964	0.188941	0.188941447	protein_coding	NM_001029885
ASHGV40018207	-0.18889	0.188886893	protein_coding	NM_015386
ASHGV40053642	0.188835	0.188835478	protein_coding	NM_152571
ASHGV40018962	0.188696	0.188696268	protein_coding	NM_000887
ASHGV40036372	-0.18867	0.188668862	protein_coding	NM_033254
ASHGV40042729	0.188621	0.188620666	protein_coding	NM_001142651
ASHGV40006662	0.188563	0.188562904	protein_coding	NM_198516
ASHGV40056106	-0.18852	0.188523372	protein_coding	NM_002408
ASHGV40019167	0.188517	0.188516801	protein_coding	NM_014685
ASHGV40019661	0.188489	0.188489333	protein_coding	NM_016532
ASHGV40015903	-0.18844	0.188444274	protein_coding	NM_022841
ASHGV40031741	0.188309	0.188309322	protein_coding	NM_017895
ASHGV40052776	-0.1883	0.188296001	protein_coding	NM_001161
ASHGV40037634	-0.18829	0.188291143	protein_coding	NM_002089
ASHGV40011171	0.188238	0.188237992	protein_coding	NM_001164746
ASHGV40025181	-0.18802	0.188015819	protein_coding	NM_138358
ASHGV40042282	-0.18798	0.187976191	protein_coding	NM_014031

ASHGV40026641	-0.18792	0.187924946	protein_coding	NM_001009565
ASHGV40055057	-0.18782	0.187823603	protein_coding	NM_015686
ASHGV40036654	0.18766	0.187660361	protein_coding	NM_004130
ASHGV40051664	0.187484	0.187483678	protein_coding	NM_022917
ASHGV40044791	0.187465	0.187464578	protein_coding	NM_001007531
ASHGV40028041	-0.18736	0.187362339	protein_coding	NM_000599
ASHGV40029726	0.187336	0.187335926	protein_coding	NM_001080850
ASHGV40055071	0.187305	0.187305287	protein_coding	NM_005938
ASHGV40057352	-0.18721	0.187211638	protein_coding	NM_017667
ASHGV40007891	-0.18716	0.187161282	protein_coding	NM_006435
ASHGV40010056	-0.18714	0.187140206	protein_coding	NM_006928
ASHGV40011848	-0.18713	0.187125881	protein_coding	NM_152435
ASHGV40013214	0.186844	0.186844305	protein_coding	NM_000538
ASHGV40000062	-0.18684	0.18684242	protein_coding	NM_006856
ASHGV40055227	0.186808	0.186808166	protein_coding	NM_004034
ASHGV40036358	0.186651	0.186651425	protein_coding	NM_001134438
ASHGV40035067	0.186555	0.186555424	protein_coding	NM_138786
ASHGV40025125	0.186449	0.186449251	protein_coding	NM_004218
ASHGV40038632	0.186368	0.186368487	protein_coding	NM_001177381
ASHGV40007397	0.186255	0.186254785	protein_coding	NM_024079
ASHGV40055901	-0.18625	0.186250451	protein_coding	NM_001303405
ASHGV40054681	0.186146	0.18614614	protein_coding	NM_005333
ASHGV40027237	-0.18608	0.186082588	protein_coding	NM_022336
ASHGV40024923	0.18605	0.186050176	protein_coding	NM_001728
ASHGV40024886	-0.186	0.186003539	protein_coding	NM_005773
ASHGV40052790	0.185925	0.185924838	protein_coding	NM_006377
ASHGV40051126	-0.18567	0.185673893	protein_coding	NM_080651
ASHGV40012512	-0.18567	0.18566534	protein_coding	NM_017993
ASHGV40048499	-0.1856	0.18560403	protein_coding	NM_021807
ASHGV40035809	0.185575	0.185575331	protein_coding	NM_005107
ASHGV40046857	0.185493	0.185492737	protein_coding	NM_178831
ASHGV40021065	0.185395	0.185395356	protein_coding	NM_001013672
ASHGV40012277	0.185383	0.185383024	protein_coding	ENST00000537262
ASHGV40053223	0.185359	0.185359156	protein_coding	NM_014425
ASHGV40020379	0.185331	0.18533121	protein_coding	NM_001142653
ASHGV40025143	-0.1853	0.185300908	protein_coding	NM_020933
ASHGV40047219	-0.18518	0.185178646	protein_coding	NM_015689
ASHGV40031955	-0.18517	0.185171518	protein_coding	uc002yfy.3
ASHGV40001445	0.185148	0.185148142	protein_coding	uc003hcs.1
ASHGV40005362	0.185078	0.185077795	protein_coding	NM_001098844
ASHGV40050465	0.18507	0.185070361	protein_coding	NM_005671
ASHGV40028707	-0.18507	0.18506753	protein_coding	NM_017735
ASHGV40035456	-0.18505	0.185050455	protein_coding	NM_018385
ASHGV40022807	0.185046	0.185046287	protein_coding	NM_025214
ASHGV40010720	0.184995	0.184994817	protein_coding	NM_017612
ASHGV40005626	0.184771	0.184770829	protein_coding	NM_020945
ASHGV40048901	-0.1847	0.184696855	protein_coding	NM_053279

ASHGV40018516	-0.18464	0.184641442	protein_coding	NM_014587
ASHGV40002704	0.18451	0.184509925	protein_coding	NM_001270517
ASHGV40028332	0.184244	0.184243881	protein_coding	NM_015148
ASHGV40051588	0.184195	0.184195465	protein_coding	NM_000077
ASHGV40024714	0.184123	0.184122919	protein_coding	NM_032679
ASHGV40047739	0.184047	0.184047193	protein_coding	NM_005402
ASHGV40015559	0.184009	0.184008877	protein_coding	NM_033271
ASHGV40032870	0.183973	0.183973278	protein_coding	NM_033257
ASHGV40009537	-0.18395	0.183951847	protein_coding	NM_130441
ASHGV40042481	0.183946	0.183946376	protein_coding	NM_014773
ASHGV40023217	0.183903	0.183903015	protein_coding	NM_012189
ASHGV40007401	0.183774	0.18377352	protein_coding	NM_024678
ASHGV40024368	-0.18367	0.183668542	protein_coding	NM_178820
ASHGV40033616	0.183644	0.183643575	protein_coding	NM_152267
ASHGV40007620	-0.18353	0.183534278	protein_coding	NM_001077690
ASHGV40017944	0.183438	0.183437505	protein_coding	NM_018092
ASHGV40018114	0.183408	0.183407772	protein_coding	NM_003039
ASHGV40018226	0.183393	0.183392668	protein_coding	NM_000069
ASHGV40056537	0.18334	0.183340413	protein_coding	NM_001297701
ASHGV40029768	-0.18332	0.183316288	protein_coding	NM_000210
ASHGV40042590	0.183206	0.183205984	protein_coding	NM_024632
ASHGV40047285	-0.18309	0.183094816	protein_coding	NM_001001661
ASHGV40049050	0.183069	0.18306903	protein_coding	NM_171982
ASHGV40026957	-0.18301	0.183009695	protein_coding	NM_001282430
ASHGV40030762	-0.18294	0.182938103	protein_coding	NM_007238
ASHGV40031738	0.182874	0.18287365	protein_coding	NM_006420
ASHGV40020894	0.182703	0.18270266	protein_coding	NM_002766
ASHGV40014346	0.182616	0.182616381	protein_coding	NM_052891
ASHGV40054592	-0.18256	0.182555231	protein_coding	NM_001042351
ASHGV40054601	0.182501	0.182501409	protein_coding	NM_001289
ASHGV40013619	0.182389	0.182388898	protein_coding	NM_206808
ASHGV40025101	-0.18219	0.182187103	protein_coding	NM_001302
ASHGV40046779	-0.18207	0.182067542	protein_coding	NM_001259
ASHGV40048441	-0.18198	0.181984437	protein_coding	NM_001219
ASHGV40020844	-0.18197	0.18197098	protein_coding	NM_030630
ASHGV40021606	0.181935	0.181934526	protein_coding	NM_144975
ASHGV40013110	-0.18185	0.181847907	protein_coding	NM_014089
ASHGV40014193	0.181799	0.181799011	protein_coding	NM_001308154
ASHGV40018941	-0.18169	0.181688508	protein_coding	NM_001099784
ASHGV40010515	0.181629	0.181629382	protein_coding	NM_018171
ASHGV40017516	0.181507	0.181506734	protein_coding	NM_025191
ASHGV40008085	-0.18128	0.181284314	protein_coding	NM_003442
ASHGV40055830	-0.18123	0.181230953	protein_coding	NM_004969
ASHGV40019128	-0.18123	0.181226708	protein_coding	NM_005853
ASHGV40055559	-0.18122	0.1812245	protein_coding	NM_006280
ASHGV40046740	-0.18122	0.181222826	protein_coding	NM_152754
ASHGV40030087	0.181212	0.181211567	protein_coding	NM_004044

ASHGV40043867	0.181151	0.181151248	protein_coding	NM_001278716
ASHGV40019753	-0.18112	0.181117023	protein_coding	NM_033004
ASHGV40050306	0.181115	0.181114553	protein_coding	NM_016353
ASHGV40000096	-0.18104	0.181037794	protein_coding	NM_194356
ASHGV40056654	0.181023	0.181023076	protein_coding	NM_053050
ASHGV40045065	-0.18102	0.181017351	protein_coding	NM_201521
ASHGV40005753	-0.18093	0.180928979	protein_coding	NM_145306
ASHGV40024775	0.18067	0.180669599	protein_coding	NM_024298
ASHGV40051417	0.180596	0.180596336	protein_coding	NM_145754
ASHGV40029109	0.180567	0.180566861	protein_coding	NM_198482
ASHGV40033633	-0.18056	0.18055963	protein_coding	NM_001242896
ASHGV40024722	-0.1805	0.18049948	protein_coding	NM_014650
ASHGV40033422	0.180446	0.180446041	protein_coding	NM_152906
ASHGV40036746	-0.18035	0.18035064	protein_coding	NM_000882
ASHGV40019150	0.180286	0.180285804	protein_coding	NM_018233
ASHGV40006687	0.180228	0.180227594	protein_coding	NM_002786
ASHGV40016664	-0.18013	0.180131178	protein_coding	NM_020168
ASHGV40018674	0.180129	0.180129038	protein_coding	NM_014117
ASHGV40016931	0.180001	0.180000601	protein_coding	NM_024798
ASHGV40025115	0.179968	0.179967605	protein_coding	NM_003083
ASHGV40026747	-0.17976	0.179758469	protein_coding	NM_016115
ASHGV40025631	-0.17974	0.179736475	protein_coding	NM_181786
ASHGV40020867	-0.1796	0.179603776	protein_coding	NM_000154
ASHGV40007581	0.179475	0.179474534	protein_coding	NM_138775
ASHGV40028382	0.179436	0.179436496	protein_coding	NM_004964
ASHGV40042411	-0.17935	0.179354851	protein_coding	NM_017706
ASHGV40032904	0.179323	0.179322703	protein_coding	NM_001282112
ASHGV40021181	0.179283	0.179282679	protein_coding	NM_024039
ASHGV40003287	0.179268	0.179268098	protein_coding	NM_016047
ASHGV40018544	0.179175	0.179175263	protein_coding	NM_015133
ASHGV40050940	-0.17905	0.179047666	protein_coding	ENST00000391681
ASHGV40053557	-0.17904	0.179041229	protein_coding	NM_032536
ASHGV40039933	0.178865	0.178865005	protein_coding	NM_199133
ASHGV40049015	-0.1788	0.178796364	protein_coding	NM_004901
ASHGV40055260	-0.1788	0.178795134	protein_coding	NM_018015
ASHGV40055939	0.178778	0.178778216	protein_coding	NM_007167
ASHGV40019897	-0.17869	0.178687121	protein_coding	ENST00000284110
ASHGV40053787	-0.17854	0.178539018	protein_coding	NM_152633
ASHGV40020112	-0.17838	0.178383682	protein_coding	NM_033389
ASHGV40053593	-0.17829	0.178291914	protein_coding	NM_002957
ASHGV40033713	-0.17824	0.178244225	protein_coding	NM_016091
ASHGV40052073	-0.1781	0.178096585	protein_coding	NM_182755
ASHGV40044139	0.178085	0.178084866	protein_coding	NM_000416
ASHGV40018582	0.178048	0.178047783	protein_coding	NM_002479
ASHGV40051372	0.177962	0.17796238	protein_coding	NM_052924
ASHGV40022232	0.177939	0.177938516	protein_coding	NM_004753
ASHGV40025567	-0.1779	0.177902914	protein_coding	NM_205834

ASHGV40047121	0.177793	0.177792629	protein_coding	NM_012470
ASHGV40027446	-0.17778	0.177776391	protein_coding	ENST00000409867
ASHGV40054939	-0.17765	0.177646423	protein_coding	NM_001164375
ASHGV40043199	-0.17759	0.177589252	protein_coding	NM_006510
ASHGV40036805	0.177556	0.177555566	protein_coding	NM_005602
ASHGV40011665	0.17751	0.177509655	protein_coding	NM_006431
ASHGV40000197	-0.17745	0.177445716	protein_coding	NM_001013
ASHGV40011069	0.177437	0.177437449	protein_coding	NM_058169
ASHGV40047692	0.177432	0.177432447	protein_coding	NM_007270
ASHGV40026403	-0.17743	0.177431359	protein_coding	NM_015207
ASHGV40056201	-0.17742	0.177420797	protein_coding	ENST00000560560
ASHGV40046984	-0.17735	0.177346306	protein_coding	NM_018970
ASHGV40008784	0.17722	0.177219564	protein_coding	NM_005231
ASHGV40018597	0.177167	0.177167435	protein_coding	NM_006799
ASHGV40053654	0.177165	0.177164537	protein_coding	NM_024718
ASHGV40057726	-0.17708	0.177084838	protein_coding	NM_001012508
ASHGV40044409	0.177066	0.177066372	protein_coding	NM_001017403
ASHGV40054565	-0.17683	0.176829494	protein_coding	NM_001039582
ASHGV40016755	-0.17675	0.176753267	protein_coding	NM_032413
ASHGV40048184	0.176697	0.176697302	protein_coding	NM_015379
ASHGV40003132	0.176541	0.176540789	protein_coding	NM_001146210
ASHGV40001962	-0.17653	0.1765295	protein_coding	ENST00000541232
ASHGV40020123	-0.17647	0.176473047	protein_coding	NM_000386
ASHGV40021748	-0.17634	0.176344359	protein_coding	NM_170607
ASHGV40011565	0.17633	0.176329926	protein_coding	NM_005981
ASHGV40007213	0.176251	0.176251126	protein_coding	NM_018219
ASHGV40053090	0.176235	0.176234617	protein_coding	NM_024077
ASHGV40032208	-0.17615	0.176146205	protein_coding	NM_000819
ASHGV40003266	0.176138	0.17613809	protein_coding	NM_005101
ASHGV40019786	-0.17592	0.175923824	protein_coding	NM_015982
ASHGV40021219	0.175842	0.175841934	protein_coding	NM_000747
ASHGV40000210	0.175807	0.175806922	protein_coding	ENST00000394484
ASHGV40010964	-0.17574	0.175744918	protein_coding	NM_002342
ASHGV40031999	0.175726	0.175725645	protein_coding	NM_006948
ASHGV40050587	0.175656	0.175655895	protein_coding	NM_031455
ASHGV40034267	0.175635	0.175634792	protein_coding	NM_024513
ASHGV40038780	0.175601	0.175601348	protein_coding	NM_001144990
ASHGV40038154	0.175585	0.175585193	protein_coding	NM_002669
ASHGV40055512	0.175545	0.175544573	protein_coding	NM_005491
ASHGV40012562	-0.17551	0.175508246	protein_coding	NM_005767
ASHGV40029107	-0.17542	0.175419614	protein_coding	NM_001135021
ASHGV40010416	0.175348	0.175347904	protein_coding	NM_001918
ASHGV40019978	0.175329	0.175329084	protein_coding	ENST00000392176
ASHGV40054850	-0.17523	0.175231993	protein_coding	NM_001289773
ASHGV40022615	0.175143	0.175142731	protein_coding	NM_001792
ASHGV40024767	-0.17512	0.175117191	protein_coding	NM_198481
ASHGV40025933	0.175109	0.175108651	protein_coding	NM_024729

ASHGV40031531	0.174984	0.174983519	protein_coding	NM_018677
ASHGV40053375	-0.17497	0.174970176	protein_coding	ENST00000565320
ASHGV40033678	0.174842	0.174842277	protein_coding	NM_003661
ASHGV40000104	0.174774	0.174773641	protein_coding	NM_014387
ASHGV40002662	0.174687	0.174686593	protein_coding	ENST00000598661
ASHGV40020412	-0.17453	0.174530643	protein_coding	NM_001143780
ASHGV40046911	0.174496	0.174495677	protein_coding	NM_002553
ASHGV40033662	-0.17444	0.17443732	protein_coding	NM_001003681
ASHGV40030811	-0.17437	0.174370272	protein_coding	NM_080627
ASHGV40048007	0.174319	0.174318914	protein_coding	NM_032421
ASHGV40025774	-0.17422	0.174218808	protein_coding	NM_013361
ASHGV40013910	0.174149	0.174148661	protein_coding	NM_002742
ASHGV40049111	-0.17415	0.174145462	protein_coding	NM_025115
ASHGV40002770	0.174128	0.174128428	protein_coding	NM_002241
ASHGV40047108	-0.17397	0.173974129	protein_coding	NM_001174164
ASHGV40020258	0.173865	0.173865439	protein_coding	NM_032875
ASHGV40038715	0.173841	0.173840995	protein_coding	NM_001145432
ASHGV40024651	0.173805	0.173805192	protein_coding	NM_007254
ASHGV40028005	0.173788	0.173788086	protein_coding	NM_001608
ASHGV40029413	0.173681	0.173680786	protein_coding	NM_030577
ASHGV40034340	-0.17351	0.17351126	protein_coding	NM_002292
ASHGV40054455	0.173404	0.173404209	protein_coding	NM_007131
ASHGV40053952	-0.17331	0.173312822	protein_coding	NM_176819
ASHGV40010075	0.173297	0.173297147	protein_coding	NM_012064
ASHGV40018526	0.173221	0.173220853	protein_coding	NM_003345
ASHGV40000083	-0.17311	0.173111669	protein_coding	NM_198216
ASHGV40044783	0.172982	0.17298177	protein_coding	NM_003447
ASHGV40008894	0.172846	0.17284588	protein_coding	NM_004055
ASHGV40012109	-0.17284	0.172842192	protein_coding	NM_002560
ASHGV40014909	-0.17264	0.172644158	protein_coding	NM_138731
ASHGV40049980	-0.17258	0.172581611	protein_coding	NM_003033
ASHGV40031285	-0.17249	0.172494204	protein_coding	NM_001200
ASHGV40006522	0.172474	0.172474211	protein_coding	NM_000076
ASHGV40014046	0.17241	0.17241025	protein_coding	NM_024884
ASHGV40049655	0.172396	0.172396271	protein_coding	NM_033285
ASHGV40016733	-0.17231	0.172306162	protein_coding	NM_016400
ASHGV40021122	0.172262	0.172262366	protein_coding	NM_015085
ASHGV40031344	-0.17221	0.172207715	protein_coding	NM_003092
ASHGV40000656	-0.17218	0.172176656	protein_coding	NM_001291314
ASHGV40022786	0.172072	0.172072028	protein_coding	NM_015846
ASHGV40042806	-0.17189	0.171891791	protein_coding	NM_017510
ASHGV40007707	-0.17185	0.171847501	protein_coding	NM_001301065
ASHGV40000231	-0.17182	0.17182023	protein_coding	NM_013421
ASHGV40029963	0.171819	0.171819007	protein_coding	NM_024520
ASHGV40026199	-0.17173	0.171733792	protein_coding	NM_152834
ASHGV40000200	0.171684	0.171684026	protein_coding	NM_018657
ASHGV40021030	-0.17161	0.171613289	protein_coding	NM_003004

ASHGV40035596	0.171559	0.171558715	protein_coding	NM_032732
ASHGV40031320	-0.17151	0.171505615	protein_coding	NM_000098
ASHGV40004955	-0.1715	0.171500722	protein_coding	NM_001127211
ASHGV40051695	0.17149	0.171490383	protein_coding	NM_032634
ASHGV40049877	0.171478	0.171477929	protein_coding	NM_014109
ASHGV40053292	0.171458	0.171457803	protein_coding	NM_006687
ASHGV40009550	0.171401	0.171401355	protein_coding	NM_014358
ASHGV40020995	-0.17134	0.17134332	protein_coding	NM_002602
ASHGV40019430	-0.17111	0.171114974	protein_coding	NM_014940
ASHGV40007674	0.171089	0.171088712	protein_coding	NM_003904
ASHGV40025062	0.171071	0.171070819	protein_coding	NM_002967
ASHGV40048002	-0.17098	0.170981132	protein_coding	NM_002314
ASHGV40030387	0.170929	0.170929052	protein_coding	NM_002081
ASHGV40056429	0.170861	0.170860983	protein_coding	NM_002949
ASHGV40023151	0.170716	0.170716401	protein_coding	NM_001142339
ASHGV40046353	0.170703	0.170702969	protein_coding	NM_015283
ASHGV40046253	-0.17047	0.170473111	protein_coding	NM_013293
ASHGV40031574	-0.17046	0.170459272	protein_coding	NM_021215
ASHGV40006547	-0.17039	0.170390743	protein_coding	NM_003141
ASHGV40053507	-0.17032	0.170320638	protein_coding	NM_001100876
ASHGV40013622	0.170037	0.170037013	protein_coding	NM_000282
ASHGV40043806	-0.17003	0.170028713	protein_coding	NM_003800
ASHGV40013691	-0.16983	0.169832332	protein_coding	NM_001242881
ASHGV40010038	-0.16974	0.16973556	protein_coding	NM_033277
ASHGV40010413	-0.16967	0.169673628	protein_coding	NM_001301036
ASHGV40008637	0.169673	0.169673344	protein_coding	NM_006268
ASHGV40015879	0.169585	0.169585248	protein_coding	NM_001142495
ASHGV40024582	-0.16942	0.169423692	protein_coding	NM_015063
ASHGV40036291	0.169321	0.169320767	protein_coding	NM_024548
ASHGV40014918	0.169204	0.169203622	protein_coding	NM_002687
ASHGV40042406	-0.16903	0.169025898	protein_coding	NM_003732
ASHGV40003309	0.168869	0.168869451	protein_coding	NM_024688
ASHGV40054732	-0.16868	0.16867649	protein_coding	NM_001037540
ASHGV40031921	0.168671	0.168670974	protein_coding	NM_080750
ASHGV40037656	0.168658	0.168657761	protein_coding	NM_018115
ASHGV40047255	0.168504	0.168503709	protein_coding	NM_005232
ASHGV40013147	0.168376	0.168375947	protein_coding	NM_015233
ASHGV40040259	0.168306	0.168306255	protein_coding	NM_024669
ASHGV40018739	-0.16809	0.168089166	protein_coding	NM_017668
ASHGV40030475	0.16807	0.168070475	protein_coding	NM_001810
ASHGV40046463	0.168007	0.168006596	protein_coding	NM_018650
ASHGV40050123	-0.16788	0.167878225	protein_coding	NM_015201
ASHGV40021790	-0.16779	0.167791899	protein_coding	NM_138387
ASHGV40033232	-0.16776	0.167762077	protein_coding	NM_014570
ASHGV40040859	0.167688	0.167687966	protein_coding	NM_006805
ASHGV40006377	0.167655	0.167655184	protein_coding	NM_024587
ASHGV40015782	0.167637	0.167637135	protein_coding	NM_174916

ASHGV40022115	0.167489	0.167489476	protein_coding	NM_004459
ASHGV40023110	-0.16743	0.167431072	protein_coding	ENST00000306329
ASHGV40024677	-0.16727	0.167274176	protein_coding	NM_005341
ASHGV40024657	0.167266	0.16726571	protein_coding	NM_016440
ASHGV40037521	0.167124	0.16712356	protein_coding	NM_181806
ASHGV40014500	-0.16707	0.167074798	protein_coding	NM_014856
ASHGV40041623	0.167022	0.167021697	protein_coding	NM_133433
ASHGV40018403	-0.16696	0.166963208	protein_coding	NM_017566
ASHGV40013111	-0.16693	0.166927545	protein_coding	NM_016529
ASHGV40021648	0.166889	0.166889218	protein_coding	NM_001488
ASHGV40013614	-0.16689	0.166885134	protein_coding	NM_001144072
ASHGV40047902	0.166879	0.166879425	protein_coding	NM_001762
ASHGV40050703	-0.16671	0.166712671	protein_coding	NM_147189
ASHGV40050353	-0.16667	0.166669147	protein_coding	NM_022749
ASHGV40009510	0.166643	0.166643144	protein_coding	NM_001273
ASHGV40050960	-0.1666	0.166602683	protein_coding	NM_012425
ASHGV40041874	-0.16623	0.166226816	protein_coding	NM_014283
ASHGV40053458	-0.16619	0.166189449	protein_coding	NM_001174147
ASHGV40011369	0.166056	0.166055804	protein_coding	NM_005276
ASHGV40017941	0.165925	0.165924536	protein_coding	NM_001001436
ASHGV40007723	0.165806	0.165806484	protein_coding	NM_006500
ASHGV40050518	0.165777	0.1657768	protein_coding	NM_007175
ASHGV40021417	-0.16572	0.165723925	protein_coding	NM_007148
ASHGV40049223	-0.16572	0.165721097	protein_coding	ENST00000265713
ASHGV40018629	0.165687	0.165687496	protein_coding	NM_015041
ASHGV40026749	-0.16563	0.165630546	protein_coding	NM_014614
ASHGV40029337	-0.1656	0.165602345	protein_coding	NM_032824
ASHGV40054590	0.165598	0.165598239	protein_coding	NM_021806
ASHGV40040466	0.165562	0.165562399	protein_coding	NM_001025
ASHGV40019264	0.165258	0.165258199	protein_coding	NM_025187
ASHGV40047959	0.165256	0.165255879	protein_coding	NM_153033
ASHGV40035956	-0.16519	0.165192315	protein_coding	NM_005778
ASHGV40005134	0.165171	0.165171162	protein_coding	uc021olj.1
ASHGV40054943	-0.16517	0.165170774	protein_coding	NM_006579
ASHGV40025983	0.165061	0.165060777	protein_coding	NM_001031721
ASHGV40045779	-0.16504	0.165035472	protein_coding	NM_014721
ASHGV40021093	0.164968	0.164967702	protein_coding	NM_000934
ASHGV40014335	0.164891	0.164890795	protein_coding	NM_015305
ASHGV40036859	0.164851	0.164851485	protein_coding	NM_003940
ASHGV40037958	0.164488	0.164488314	protein_coding	NM_007083
ASHGV40006780	0.164443	0.164442917	protein_coding	NM_018490
ASHGV40026910	-0.16442	0.164415706	protein_coding	NM_001617
ASHGV40054360	-0.1644	0.164397452	protein_coding	NM_015129
ASHGV40005808	-0.16438	0.164382987	protein_coding	NM_173540
ASHGV40044653	-0.16412	0.164115881	protein_coding	NM_001143942
ASHGV40040167	-0.16397	0.163971313	protein_coding	NM_012351
ASHGV40047138	0.163922	0.163922387	protein_coding	NM_016478

ASHGV40000433	-0.1639	0.16390037	protein_coding	NM_145911
ASHGV40024822	0.163886	0.163885556	protein_coding	NM_014501
ASHGV40030464	-0.16389	0.16388546	protein_coding	NM_014948
ASHGV40029297	-0.16384	0.163842988	protein_coding	NM_006112
ASHGV40049147	-0.16381	0.163814649	protein_coding	NM_175911
ASHGV40034972	0.163739	0.163738556	protein_coding	NM_178130
ASHGV40014130	-0.16364	0.163638704	protein_coding	NM_012460
ASHGV40054572	-0.16362	0.163623562	protein_coding	NM_032512
ASHGV40033075	-0.16356	0.163561357	protein_coding	NM_152996
ASHGV40054583	0.163535	0.163534978	protein_coding	NM_145178
ASHGV40030469	0.163352	0.163352195	protein_coding	NM_001009984
ASHGV40030130	0.163343	0.163342691	protein_coding	NM_007127
ASHGV40043534	-0.16331	0.163305739	protein_coding	NM_178148
ASHGV40025756	-0.16319	0.163189581	protein_coding	NM_174945
ASHGV40043689	-0.16318	0.163176967	protein_coding	NM_080742
ASHGV40014304	0.163149	0.163148573	protein_coding	ENST00000338772
ASHGV40000131	-0.16293	0.162925984	protein_coding	NM_001017986
ASHGV40052483	0.162875	0.162874658	protein_coding	NM_144653
ASHGV40014802	0.16285	0.162850018	protein_coding	NM_198083
ASHGV40033274	0.162844	0.162843782	protein_coding	NM_058238
ASHGV40045096	-0.1628	0.16279838	protein_coding	NM_001137560
ASHGV40045369	-0.16266	0.162657413	protein_coding	ENST00000369474
ASHGV40030254	-0.16254	0.162544682	protein_coding	NM_022730
ASHGV40015424	0.162477	0.162477482	protein_coding	NM_001099402
ASHGV40054903	-0.16247	0.162467402	protein_coding	uc004dgq.2
ASHGV40008341	-0.16225	0.162252089	protein_coding	NM_207122
ASHGV40051400	-0.1622	0.162201049	protein_coding	NM_019037
ASHGV40038791	-0.16216	0.162157056	protein_coding	NM_138389
ASHGV40024958	0.162128	0.162127727	protein_coding	NM_001405
ASHGV40047667	-0.16207	0.162069263	protein_coding	NM_001197026
ASHGV40010553	0.161938	0.161938095	protein_coding	NM_003006
ASHGV40048703	0.161928	0.161928101	protein_coding	NM_020445
ASHGV40032737	0.161916	0.161916116	protein_coding	NM_005049
ASHGV40015223	-0.16165	0.161647398	protein_coding	NM_014909
ASHGV40005620	-0.16164	0.1616363	protein_coding	NM_139049
ASHGV40046125	-0.16158	0.161575645	protein_coding	NM_207116
ASHGV40007472	-0.16157	0.16156886	protein_coding	NM_001144871
ASHGV40032972	-0.16148	0.161476234	protein_coding	NM_022081
ASHGV40031690	-0.16146	0.161463326	protein_coding	NM_033421
ASHGV40038210	-0.16142	0.161421599	protein_coding	NM_138386
ASHGV40024980	0.161378	0.161378184	protein_coding	NM_001319
ASHGV40016215	0.161293	0.161292676	protein_coding	NM_001100879
ASHGV40027557	0.161275	0.161274785	protein_coding	NM_198557
ASHGV40027414	0.161201	0.161200716	protein_coding	NM_001171083
ASHGV40052682	-0.16117	0.16117111	protein_coding	NM_017925
ASHGV40015986	-0.16108	0.16108222	protein_coding	NM_014326
ASHGV40050282	-0.16081	0.160805246	protein_coding	NM_012311

ASHGV40033122	-0.16064	0.160641395	protein_coding	NM_003312
ASHGV40035065	-0.16052	0.160523703	protein_coding	NM_000096
ASHGV40051045	-0.1604	0.160396222	protein_coding	NM_003506
ASHGV40016464	0.160368	0.160367827	protein_coding	NM_002570
ASHGV40039702	-0.16029	0.160290626	protein_coding	NM_001256395
ASHGV40028648	0.160231	0.160231357	protein_coding	NM_016085
ASHGV40019474	0.160127	0.160127283	protein_coding	NM_022041
ASHGV40020648	0.159976	0.159976256	protein_coding	NM_016125
ASHGV40016182	0.159845	0.159844931	protein_coding	NM_001017961
ASHGV40011378	-0.15981	0.159809138	protein_coding	NM_005171
ASHGV40000249	-0.15977	0.159768941	protein_coding	NM_001002010
ASHGV40022248	0.159749	0.159748919	protein_coding	NM_001142601
ASHGV40057432	-0.15973	0.159730568	protein_coding	uc003xki.3
ASHGV40034460	-0.15972	0.159719142	protein_coding	NM_001660
ASHGV40040244	-0.15963	0.159630326	protein_coding	NM_020125
ASHGV40016933	-0.15955	0.159552542	protein_coding	NM_016213
ASHGV40006718	0.159541	0.159541049	protein_coding	NM_181507
ASHGV40007994	-0.1595	0.159497486	protein_coding	NM_001033
ASHGV40011827	0.159486	0.159486285	protein_coding	NM_014050
ASHGV40012702	0.159467	0.159466591	protein_coding	NM_014953
ASHGV40047094	0.159444	0.159443618	protein_coding	NM_176814
ASHGV40011593	-0.15942	0.159417545	protein_coding	NM_005872
ASHGV40026050	-0.15937	0.159368063	protein_coding	NM_006863
ASHGV40030868	-0.15933	0.159334577	protein_coding	NM_001080472
ASHGV40011847	0.159219	0.159219021	protein_coding	NM_003095
ASHGV40051611	-0.15918	0.159181788	protein_coding	NM_024828
ASHGV40033989	-0.15918	0.159177153	protein_coding	NM_207351
ASHGV40006054	-0.15905	0.159047173	protein_coding	NM_144588
ASHGV40023269	-0.15899	0.15899481	protein_coding	NM_000371
ASHGV40049920	-0.15897	0.158965335	protein_coding	NM_174911
ASHGV40026646	-0.1589	0.158901639	protein_coding	NM_025264
ASHGV40034033	0.158878	0.15887783	protein_coding	NM_001080423
ASHGV40017057	0.158435	0.158435101	protein_coding	NM_001130028
ASHGV40009156	-0.15839	0.158392131	protein_coding	NM_006028
ASHGV40042492	-0.15831	0.158306608	protein_coding	NM_015071
ASHGV40036721	0.158128	0.158127955	protein_coding	NM_003875
ASHGV40046703	-0.15807	0.158065828	protein_coding	NM_032936
ASHGV40016217	-0.15804	0.158036325	protein_coding	NM_004049
ASHGV40018150	-0.15795	0.157954666	protein_coding	NM_013241
ASHGV40051054	-0.15791	0.157912776	protein_coding	NM_001004470
ASHGV40039978	-0.15782	0.157816543	protein_coding	NM_012334
ASHGV40056165	0.157711	0.157710618	protein_coding	NM_024011
ASHGV40009955	-0.15767	0.157669247	protein_coding	NM_007210
ASHGV40036294	-0.15752	0.157516451	protein_coding	NM_031419
ASHGV40017868	0.157262	0.157262331	protein_coding	NM_173502
ASHGV40057346	-0.15712	0.157121945	protein_coding	NM_001004686
ASHGV40008298	-0.15697	0.156967203	protein_coding	NM_024841

ASHGV40050154	-0.15684	0.156842531	protein_coding	NM_006958
ASHGV40007081	0.156765	0.156764566	protein_coding	NM_012200
ASHGV40048842	0.156565	0.156565296	protein_coding	NM_004084
ASHGV40032790	-0.15655	0.156546414	protein_coding	NM_001849
ASHGV40021157	-0.15649	0.156493786	protein_coding	NM_002663
ASHGV40037476	-0.15649	0.156489932	protein_coding	NM_152995
ASHGV40021771	0.156447	0.156446817	protein_coding	NM_005899
ASHGV40056836	0.156386	0.156386316	protein_coding	NM_005675
ASHGV40010385	0.156291	0.156290643	protein_coding	NM_001920
ASHGV40002133	-0.1562	0.156201376	protein_coding	ENST00000556347
ASHGV40024468	0.156153	0.156153254	protein_coding	NM_006297
ASHGV40027891	0.156146	0.156145683	protein_coding	NM_001195144
ASHGV40007717	-0.15614	0.156139969	protein_coding	NM_006389
ASHGV40008498	-0.15603	0.156026959	protein_coding	NM_031457
ASHGV40038630	-0.156	0.155996582	protein_coding	NM_001195571
ASHGV40014369	0.155951	0.155950681	protein_coding	NM_000793
ASHGV40051589	-0.15592	0.155922853	protein_coding	NM_004936
ASHGV40001978	-0.15569	0.155689202	protein_coding	ENST00000543226
ASHGV40019679	0.155677	0.155677035	protein_coding	NM_018128
ASHGV40046953	0.155622	0.155622179	protein_coding	NM_002291
ASHGV40035518	0.155313	0.155313384	protein_coding	NM_014687
ASHGV40055551	-0.15526	0.155263246	protein_coding	NM_001395
ASHGV40016842	0.155219	0.155219153	protein_coding	NM_004855
ASHGV40036030	0.155172	0.155171919	protein_coding	NM_177966
ASHGV40014249	0.15514	0.155140082	protein_coding	NM_005466
ASHGV40055957	-0.1548	0.154797168	protein_coding	NM_024928
ASHGV40044984	0.154704	0.154703878	protein_coding	NM_173558
ASHGV40028922	0.154579	0.154579339	protein_coding	NM_005119
ASHGV40039588	0.15447	0.154469576	protein_coding	NM_018352
ASHGV40037721	0.154393	0.154392806	protein_coding	NM_001297755
ASHGV40009230	0.154289	0.154289383	protein_coding	NM_000190
ASHGV40050898	-0.15421	0.154209998	protein_coding	ENST00000425429
ASHGV40011923	0.154066	0.154065748	protein_coding	NM_018413
ASHGV40036266	0.154053	0.154052689	protein_coding	NM_005290
ASHGV40007992	0.154043	0.15404317	protein_coding	NM_003156
ASHGV40014894	0.153795	0.15379545	protein_coding	NM_014672
ASHGV40006501	0.15373	0.153729822	protein_coding	NM_001170820
ASHGV40013024	0.153687	0.153687253	protein_coding	NM_007368
ASHGV40028968	0.153621	0.153621221	protein_coding	NM_173545
ASHGV40006287	0.153603	0.1536027	protein_coding	NM_004281
ASHGV40045575	-0.15354	0.153539059	protein_coding	NM_013352
ASHGV40020907	0.153513	0.15351308	protein_coding	NM_003016
ASHGV40006298	0.153456	0.153455673	protein_coding	NM_018117
ASHGV40016753	-0.15342	0.153424792	protein_coding	uc021sko.2
ASHGV40029593	-0.15324	0.153235591	protein_coding	NM_018460
ASHGV40011557	0.153185	0.153185226	protein_coding	NM_024779
ASHGV40036248	-0.15308	0.153082717	protein_coding	NM_173655

ASHGV40013159	0.15306	0.153060257	protein_coding	NM_001629
ASHGV40016128	0.153045	0.153045342	protein_coding	NM_006917
ASHGV40052605	-0.15304	0.15304203	protein_coding	NM_005772
ASHGV40010563	-0.15287	0.152871934	protein_coding	NM_001854
ASHGV40039720	0.152858	0.152857627	protein_coding	NM_021942
ASHGV40019659	0.152844	0.152844168	protein_coding	NM_016823
ASHGV40015996	0.152674	0.152673686	protein_coding	NM_194272
ASHGV40018175	-0.15266	0.152662114	protein_coding	NM_005072
ASHGV40023926	-0.15262	0.152617968	protein_coding	NM_001080404
ASHGV40014030	-0.15257	0.152565347	protein_coding	ENST00000539688
ASHGV40015153	0.152543	0.152542574	protein_coding	NM_015351
ASHGV40046653	0.152469	0.1524686	protein_coding	NM_018044
ASHGV40024109	0.152461	0.152461003	protein_coding	NM_001001524
ASHGV40036874	0.152453	0.152453307	protein_coding	NM_003106
ASHGV40029115	-0.1524	0.152397252	protein_coding	NM_006590
ASHGV40056869	-0.15237	0.152369533	protein_coding	NM_032287
ASHGV40039105	-0.15234	0.152338475	protein_coding	NM_020226
ASHGV40049718	0.152141	0.152141318	protein_coding	NM_015902
ASHGV40053757	0.152139	0.152139396	protein_coding	NM_000216
ASHGV40052954	-0.15209	0.152094006	protein_coding	NM_182505
ASHGV40028262	-0.1519	0.151895835	protein_coding	NM_001271733
ASHGV40012595	0.151805	0.151804664	protein_coding	NM_002498
ASHGV40031537	-0.15175	0.151754355	protein_coding	NM_007186
ASHGV40037718	0.151703	0.151703457	protein_coding	NM_016619
ASHGV40026071	0.151701	0.151700961	protein_coding	ENST00000598855
ASHGV40033450	-0.1517	0.151700773	protein_coding	NM_005446
ASHGV40023173	0.151597	0.151597208	protein_coding	NM_003799
ASHGV40017415	-0.15158	0.15158394	protein_coding	NM_021259
ASHGV40025096	0.151431	0.151430984	protein_coding	ENST00000593531
ASHGV40041924	0.151294	0.151294377	protein_coding	NM_014886
ASHGV40039344	0.151208	0.151207814	protein_coding	NM_005033
ASHGV40003138	-0.1512	0.151195985	protein_coding	NM_022737
ASHGV40037041	-0.15112	0.151122659	protein_coding	NM_001282506
ASHGV40044758	-0.15109	0.15109219	protein_coding	NM_013375
ASHGV40001923	0.15096	0.150960019	protein_coding	NM_001039496
ASHGV40011044	0.150694	0.150693728	protein_coding	NM_001291823
ASHGV40010707	0.15056	0.150560348	protein_coding	NM_173855
ASHGV40030492	-0.15052	0.150520221	protein_coding	NM_005116
ASHGV40018427	-0.15051	0.150506341	protein_coding	NM_178310
ASHGV40005813	-0.15035	0.150347065	protein_coding	NM_001242487
ASHGV40029460	0.150339	0.150338598	protein_coding	NM_152373
ASHGV40046493	-0.15033	0.150328294	protein_coding	NM_022746
ASHGV40031614	-0.15033	0.150325961	protein_coding	NM_001301860
ASHGV40038052	-0.15017	0.150168405	protein_coding	NM_006874
ASHGV40031281	0.150135	0.15013505	protein_coding	NM_019095
ASHGV40039464	0.150004	0.150004047	protein_coding	NM_007080
ASHGV40020577	0.149949	0.149949348	protein_coding	NM_012329

ASHGV40022069	-0.14994	0.14993758	protein_coding	NM_025185
ASHGV40011013	-0.14993	0.149929505	protein_coding	NM_018416
ASHGV40040561	0.149812	0.149812325	protein_coding	NM_001145678
ASHGV40010942	0.1498	0.149799515	protein_coding	NM_000217
ASHGV40027662	-0.14977	0.149772413	protein_coding	NM_024753
ASHGV40008875	0.1496	0.149600044	protein_coding	NM_003369
ASHGV40035088	0.149481	0.149481241	protein_coding	NM_005067
ASHGV40040810	0.149363	0.149362656	protein_coding	NM_001172700
ASHGV40008278	0.149213	0.149213381	protein_coding	NM_001752
ASHGV40028087	0.14912	0.149119748	protein_coding	NM_012100
ASHGV40047677	0.149028	0.149027619	protein_coding	NM_198098
ASHGV40018149	-0.14903	0.149027257	protein_coding	NM_012163
ASHGV40035604	-0.14898	0.148976981	protein_coding	NM_001570
ASHGV40055569	-0.14891	0.148908346	protein_coding	ENST00000369951
ASHGV40033096	-0.14887	0.148870407	protein_coding	ENST00000594060
ASHGV40010691	-0.14875	0.148750622	protein_coding	NM_032314
ASHGV40006685	0.148704	0.148703933	protein_coding	NM_016451
ASHGV40053561	0.148624	0.148623846	protein_coding	NM_012204
ASHGV40005497	0.148611	0.148610552	protein_coding	NM_030772
ASHGV40023149	-0.14857	0.148569966	protein_coding	NM_001282300
ASHGV40022032	-0.14849	0.14848535	protein_coding	NM_014906
ASHGV40035905	0.148449	0.148449332	protein_coding	NM_147196
ASHGV40039936	0.148403	0.148402981	protein_coding	NM_138809
ASHGV40048043	0.148133	0.148133007	protein_coding	NM_020879
ASHGV40016228	0.1481	0.148099509	protein_coding	NM_015154
ASHGV40001609	0.148084	0.148083858	protein_coding	ENST00000513209
ASHGV40024385	0.148068	0.148067853	protein_coding	NM_001436
ASHGV40026473	-0.14787	0.147874456	protein_coding	NM_004036
ASHGV40057707	-0.14784	0.147844172	protein_coding	NM_002629
ASHGV40030082	0.147833	0.147833237	protein_coding	NM_001080500
ASHGV40018068	-0.14766	0.147661725	protein_coding	NM_001297
ASHGV40035367	-0.14763	0.147631828	protein_coding	NM_001346
ASHGV40050091	-0.14751	0.14750879	protein_coding	NM_052963
ASHGV40017406	0.147408	0.14740753	protein_coding	NM_022450
ASHGV40042440	-0.14726	0.147257559	protein_coding	ENST00000239444
ASHGV40018880	-0.14725	0.147251823	protein_coding	NM_001145795
ASHGV40034312	0.147241	0.14724074	protein_coding	NM_001114106
ASHGV40009514	-0.14723	0.147227864	protein_coding	NM_001135734
ASHGV40015111	0.147185	0.14718463	protein_coding	NM_001204063
ASHGV40034361	0.147147	0.14714687	protein_coding	NM_024046
ASHGV40000014	0.147054	0.147053757	protein_coding	uc010lde.1
ASHGV40037135	0.147044	0.147043613	protein_coding	NM_001127266
ASHGV40029889	0.14701	0.14701029	protein_coding	NM_032168
ASHGV40034425	0.14697	0.146970489	protein_coding	NM_018403
ASHGV40047197	0.146958	0.146958475	protein_coding	NM_001164665
ASHGV40003334	0.146926	0.146925749	protein_coding	NM_144591
ASHGV40000676	0.146723	0.146722842	protein_coding	ENST00000429220

ASHGV40003359	-0.14668	0.146676711	protein_coding	NM_198998
ASHGV40003241	0.146654	0.146654085	protein_coding	NM_001763
ASHGV40052493	0.146629	0.146628612	protein_coding	NM_052813
ASHGV40055002	-0.14657	0.146570892	protein_coding	NM_019067
ASHGV40026940	0.146569	0.14656942	protein_coding	NM_016058
ASHGV40009211	0.146518	0.146517953	protein_coding	NM_032780
ASHGV40053196	-0.14651	0.14651022	protein_coding	NM_002486
ASHGV40052282	-0.1465	0.146500967	protein_coding	NM_005388
ASHGV40017940	-0.14648	0.146480049	protein_coding	NM_182493
ASHGV40008032	-0.1464	0.146398427	protein_coding	NM_173525
ASHGV40057427	-0.14636	0.146358691	protein_coding	NM_016127
ASHGV40057225	0.146199	0.14619865	protein_coding	NM_198486
ASHGV40052511	-0.14616	0.146156847	protein_coding	NM_178469
ASHGV40035958	0.146121	0.146121307	protein_coding	NM_000172
ASHGV40040898	-0.14611	0.146109799	protein_coding	NM_001945
ASHGV40039131	0.146007	0.146007246	protein_coding	NM_080683
ASHGV40057806	0.145953	0.145953285	protein_coding	ENST00000440961
ASHGV40019853	-0.1459	0.145904954	protein_coding	NM_001220493
ASHGV40024787	-0.1458	0.145797266	protein_coding	NM_001289025
ASHGV40048601	-0.14575	0.145750792	protein_coding	NM_012369
ASHGV40030614	-0.14572	0.145722492	protein_coding	NM_001278628
ASHGV40048801	0.14561	0.145610154	protein_coding	NM_207332
ASHGV40047802	-0.14553	0.145526479	protein_coding	NM_005644
ASHGV40049648	-0.14534	0.145336543	protein_coding	NM_012415
ASHGV40047657	-0.14531	0.145309464	protein_coding	NM_182898
ASHGV40007681	0.145261	0.145260892	protein_coding	uc001pqc.4
ASHGV40016240	0.145136	0.145135507	protein_coding	NM_024580
ASHGV40043280	0.145112	0.145111663	protein_coding	NM_006295
ASHGV40042241	0.145047	0.145047169	protein_coding	NM_004384
ASHGV40019208	0.145022	0.145022018	protein_coding	NM_001242835
ASHGV40008276	0.144634	0.144634257	protein_coding	NM_024662
ASHGV40034832	-0.14459	0.144593576	protein_coding	NM_002213
ASHGV40007950	-0.14453	0.144531176	protein_coding	NM_152377
ASHGV40009347	-0.14453	0.144525864	protein_coding	NM_017953
ASHGV40023910	0.144503	0.144503396	protein_coding	NM_001035223
ASHGV40056535	0.144353	0.144352587	protein_coding	ENST00000536950
ASHGV40009540	-0.14432	0.14432394	protein_coding	NM_006931
ASHGV40032225	-0.14431	0.144306466	protein_coding	NM_013339
ASHGV40009135	0.144301	0.144300639	protein_coding	NM_031938
ASHGV40012394	-0.1443	0.144295445	protein_coding	NM_002019
ASHGV40057464	-0.14429	0.144287516	protein_coding	ENST00000522822
ASHGV40009521	0.144252	0.144251919	protein_coding	NM_001144831
ASHGV40033858	0.14422	0.14422003	protein_coding	NM_018006
ASHGV40019642	-0.14412	0.144115395	protein_coding	NM_003585
ASHGV40050585	-0.1441	0.144101212	protein_coding	NM_001556
ASHGV40025300	-0.14404	0.144042829	protein_coding	NM_014077
ASHGV40015750	-0.14397	0.143973498	protein_coding	NM_133639

ASHGV40026188	0.143922	0.143921527	protein_coding	NM_001002919
ASHGV40041039	0.143684	0.14368374	protein_coding	NM_018691
ASHGV40014470	0.143507	0.143507226	protein_coding	NM_005606
ASHGV40030259	-0.14344	0.14344251	protein_coding	NM_001632
ASHGV40042758	-0.14341	0.143405569	protein_coding	ENST00000536896
ASHGV40042991	0.14339	0.143390366	protein_coding	NM_015948
ASHGV40011399	0.14333	0.143330209	protein_coding	NM_181711
ASHGV40032170	0.143251	0.143251238	protein_coding	NM_001099219
ASHGV40047405	0.142961	0.142960614	protein_coding	NM_020223
ASHGV40021105	-0.14293	0.142930828	protein_coding	NM_001098202
ASHGV40022780	0.142614	0.142614303	protein_coding	NM_006111
ASHGV40013360	-0.14258	0.142581458	protein_coding	NM_001130912
ASHGV40046451	0.142557	0.142556637	protein_coding	NM_182547
ASHGV40021933	0.142552	0.142552327	protein_coding	NM_001258372
ASHGV40055874	-0.14255	0.14254627	protein_coding	NM_030937
ASHGV40048923	0.142543	0.142542635	protein_coding	uc003wwc.5
ASHGV40041293	-0.14248	0.142482167	protein_coding	NM_017542
ASHGV40054153	0.142354	0.142354414	protein_coding	NM_001008537
ASHGV40000041	-0.14233	0.142331498	protein_coding	ENST00000304311
ASHGV40024169	-0.14232	0.142315592	protein_coding	NM_003036
ASHGV40018626	-0.1423	0.142300133	protein_coding	NM_003450
ASHGV40005324	0.14226	0.142260317	protein_coding	NM_018144
ASHGV40008688	-0.14225	0.142254043	protein_coding	NM_178864
ASHGV40032494	0.142234	0.142233614	protein_coding	NM_152665
ASHGV40006846	-0.14219	0.142185244	protein_coding	NM_005764
ASHGV40033760	0.142068	0.142068226	protein_coding	NM_000026
ASHGV40033340	0.141985	0.141984702	protein_coding	NM_015166
ASHGV40012635	0.14197	0.14197005	protein_coding	NM_001042517
ASHGV40011996	-0.14195	0.141954286	protein_coding	NM_015836
ASHGV40033752	0.141948	0.141947679	protein_coding	NM_021096
ASHGV40001494	0.141895	0.141895339	protein_coding	NM_002102
ASHGV40032921	0.141872	0.141872332	protein_coding	NM_020070
ASHGV40037712	-0.14175	0.141750761	protein_coding	NM_001080506
ASHGV40040664	0.141741	0.141741311	protein_coding	NM_002387
ASHGV40056433	-0.1417	0.141698769	protein_coding	NM_018949
ASHGV40036914	-0.14167	0.141672895	protein_coding	ENST00000419960
ASHGV40005161	-0.14163	0.141627804	protein_coding	NM_000760
ASHGV40018847	-0.14158	0.14158071	protein_coding	NM_203376
ASHGV40028985	0.141418	0.141418101	protein_coding	NM_002357
ASHGV40012381	-0.14134	0.14134453	protein_coding	NM_152912
ASHGV40041278	-0.14133	0.141325952	protein_coding	NM_016175
ASHGV40014135	0.141264	0.141264161	protein_coding	NM_144581
ASHGV40035842	-0.14126	0.141257829	protein_coding	NM_001042646
ASHGV40011350	-0.14121	0.141206308	protein_coding	NM_023071
ASHGV40011151	0.141096	0.141096471	protein_coding	NM_178454
ASHGV40051997	0.141063	0.141063468	protein_coding	NM_017948
ASHGV40044917	0.140967	0.140966609	protein_coding	NM_002931

ASHGV40006087	0.140908	0.140907775	protein_coding	NM_017902
ASHGV40009432	0.140876	0.140876288	protein_coding	NM_152441
ASHGV40038658	0.140653	0.140652685	protein_coding	NM_015907
ASHGV40006702	-0.14063	0.1406279	protein_coding	NM_175058
ASHGV40025996	0.14054	0.140539612	protein_coding	NM_001143939
ASHGV40060812	-0.14042	0.140418005	protein_coding	uc001psy.3
ASHGV40043473	-0.14034	0.140344651	protein_coding	NM_002922
ASHGV40032401	-0.14024	0.140242565	protein_coding	NM_017526
ASHGV40021394	0.140128	0.140128436	protein_coding	NM_004140
ASHGV40030233	-0.14012	0.140116351	protein_coding	NM_174899
ASHGV40036847	-0.1401	0.140100203	protein_coding	NM_005832
ASHGV40014779	0.140049	0.140048509	protein_coding	NM_017924
ASHGV40006208	-0.13985	0.139846703	protein_coding	NM_203379
ASHGV40029896	0.139836	0.139836061	protein_coding	NM_144708
ASHGV40015004	-0.13974	0.139744757	protein_coding	NM_005192
ASHGV40056096	-0.13973	0.139731359	protein_coding	NM_019839
ASHGV40054046	0.139724	0.139723854	protein_coding	NM_152230
ASHGV40045489	-0.13969	0.139689234	protein_coding	NM_145315
ASHGV40020627	0.139637	0.139636763	protein_coding	NM_003168
ASHGV40009576	0.139525	0.139524981	protein_coding	NM_019610
ASHGV40011257	0.13934	0.139340016	protein_coding	NM_000677
ASHGV40019270	0.139339	0.139339131	protein_coding	NM_001950
ASHGV40037986	0.139284	0.139283766	protein_coding	NM_152778
ASHGV40011662	0.139236	0.139236439	protein_coding	NM_006530
ASHGV40013926	0.139165	0.139164697	protein_coding	NM_080664
ASHGV40043435	0.13915	0.139150353	protein_coding	NM_052893
ASHGV40046928	-0.1391	0.139097495	protein_coding	NM_019042
ASHGV40018215	-0.13903	0.139027529	protein_coding	NM_018348
ASHGV40008764	-0.139	0.139002477	protein_coding	NM_002617
ASHGV40050318	-0.13897	0.138968275	protein_coding	NM_000662
ASHGV40017871	-0.13885	0.138846636	protein_coding	NM_152901
ASHGV40051573	0.138822	0.1388216	protein_coding	NM_145010
ASHGV40023863	-0.13876	0.138760037	protein_coding	NM_001077624
ASHGV40039175	-0.13872	0.138722168	protein_coding	NM_001203
ASHGV40044157	-0.13869	0.138685042	protein_coding	NM_022121
ASHGV40010566	-0.13868	0.138683138	protein_coding	NM_031954
ASHGV40022063	-0.13864	0.138641266	protein_coding	NM_181725
ASHGV40006482	-0.13834	0.138340868	protein_coding	NM_012222
ASHGV40022560	0.1383	0.138300018	protein_coding	NM_005406
ASHGV40035812	-0.1383	0.138298776	protein_coding	NM_145755
ASHGV40023793	-0.1383	0.138297907	protein_coding	NM_006087
ASHGV40007027	-0.13826	0.138262748	protein_coding	NM_152716
ASHGV40050779	0.138246	0.138246014	protein_coding	uc003xxt.3
ASHGV40039767	-0.13817	0.138166914	protein_coding	uc003iyx.1
ASHGV40047942	0.138016	0.138016383	protein_coding	NM_021148
ASHGV40021629	0.137862	0.137862168	protein_coding	NM_024308
ASHGV40039216	0.137797	0.137797174	protein_coding	NM_018845

ASHGV40021286	0.137764	0.13776403	protein_coding	NM_153210
ASHGV40002595	-0.13775	0.1377513	protein_coding	NM_014297
ASHGV40052095	-0.13768	0.137678737	protein_coding	NM_017746
ASHGV40036513	0.137531	0.13753102	protein_coding	NM_016128
ASHGV40038525	0.137402	0.137402286	protein_coding	NM_018401
ASHGV40016154	-0.13737	0.137368866	protein_coding	NM_015477
ASHGV40055848	0.1373	0.137300076	protein_coding	NM_002860
ASHGV40012308	-0.13722	0.137217872	protein_coding	NM_021954
ASHGV40017746	0.137159	0.137159105	protein_coding	NM_024675
ASHGV40011397	0.137137	0.137136803	protein_coding	NM_000020
ASHGV40028661	0.137124	0.137124469	protein_coding	NM_007266
ASHGV40002573	0.137022	0.137021999	protein_coding	ENST00000592574
ASHGV40027947	0.136982	0.136981911	protein_coding	NM_016258
ASHGV40052878	-0.13676	0.136761147	protein_coding	ENST00000377531
ASHGV40000201	0.136457	0.136457137	protein_coding	NM_020425
ASHGV40005585	-0.13644	0.136439437	protein_coding	NM_006963
ASHGV40041286	-0.13641	0.13641206	protein_coding	NM_052863
ASHGV40020407	-0.13624	0.136240834	protein_coding	NM_014233
ASHGV40054769	-0.13619	0.136187439	protein_coding	NM_003410
ASHGV40049132	-0.13617	0.136167337	protein_coding	NM_001258284
ASHGV40019629	0.1361	0.136100217	protein_coding	NM_001300757
ASHGV40027626	-0.13603	0.136027766	protein_coding	NM_016836
ASHGV40047747	0.135893	0.135893164	protein_coding	NM_032018
ASHGV40019280	-0.13586	0.135862801	protein_coding	NM_006565
ASHGV40034373	-0.13586	0.135856786	protein_coding	NM_006764
ASHGV40031156	0.135842	0.135841587	protein_coding	NM_017798
ASHGV40006951	0.135637	0.13563716	protein_coding	NM_019073
ASHGV40009933	0.135449	0.135449409	protein_coding	NM_006337
ASHGV40010646	-0.13536	0.135361269	protein_coding	NM_024738
ASHGV40029757	0.135212	0.135211549	protein_coding	NM_024097
ASHGV40020540	0.135201	0.135200997	protein_coding	NM_001257359
ASHGV40055415	0.134993	0.134992962	protein_coding	NM_152695
ASHGV40028017	0.13496	0.134959751	protein_coding	NM_016260
ASHGV40005738	0.13482	0.134820039	protein_coding	NM_024045
ASHGV40012785	0.134774	0.134773758	protein_coding	NM_006468
ASHGV40055531	-0.13473	0.13472643	protein_coding	NM_018558
ASHGV40024515	-0.1347	0.134697304	protein_coding	NM_018836
ASHGV40015126	0.134676	0.134675604	protein_coding	NM_004094
ASHGV40020838	0.134644	0.134644422	protein_coding	NM_001305078
ASHGV40007062	0.134586	0.134586001	protein_coding	NM_013402
ASHGV40055867	-0.13454	0.13454061	protein_coding	NM_003061
ASHGV40007836	-0.13442	0.134418025	protein_coding	NM_020228
ASHGV40014217	-0.13439	0.134393703	protein_coding	NM_182526
ASHGV40025100	0.134235	0.134234982	protein_coding	NM_199294
ASHGV40052622	0.134185	0.134185283	protein_coding	NM_015061
ASHGV40047269	0.134108	0.13410797	protein_coding	NM_022445
ASHGV40044256	-0.13402	0.134023357	protein_coding	NM_020861

ASHGV40015064	0.133963	0.133962721	protein_coding	NM_022495
ASHGV40053226	-0.13389	0.133885961	protein_coding	NM_017753
ASHGV40031539	-0.13381	0.13380872	protein_coding	NM_015966
ASHGV40012597	0.133749	0.1337491	protein_coding	NM_018676
ASHGV40057457	0.133683	0.13368279	protein_coding	ENST00000379010
ASHGV40033981	-0.13365	0.133649171	protein_coding	NM_198560
ASHGV40046450	0.133517	0.13351677	protein_coding	NM_019082
ASHGV40056200	0.133208	0.133207545	protein_coding	NM_019006
ASHGV40024956	-0.133	0.133004695	protein_coding	NM_001130924
ASHGV40017559	-0.13299	0.132993709	protein_coding	NM_024535
ASHGV40033542	0.132954	0.132953598	protein_coding	NM_005160
ASHGV40030574	0.132792	0.132792478	protein_coding	NM_024704
ASHGV40046442	0.132773	0.132772874	protein_coding	NM_001127218
ASHGV40010596	0.132764	0.132763819	protein_coding	ENST00000419234
ASHGV40044953	-0.13276	0.132762584	protein_coding	NM_017754
ASHGV40057358	-0.13275	0.132753559	protein_coding	NM_145111
ASHGV40025114	-0.13272	0.132718563	protein_coding	NM_145185
ASHGV40007203	-0.1326	0.132595586	protein_coding	NM_001532
ASHGV40010116	-0.13255	0.132546189	protein_coding	NM_006576
ASHGV40015691	-0.13246	0.132464465	protein_coding	NM_080650
ASHGV40023930	-0.13239	0.132390045	protein_coding	NM_001164276
ASHGV40006341	-0.13222	0.132221636	protein_coding	NM_153442
ASHGV40026637	-0.13172	0.131717896	protein_coding	NM_198963
ASHGV40054322	-0.13172	0.131716657	protein_coding	NM_014289
ASHGV40052794	0.131711	0.131711039	protein_coding	NM_006285
ASHGV40025969	-0.1317	0.131695841	protein_coding	NM_001278392
ASHGV40032942	0.131691	0.131690847	protein_coding	NM_004121
ASHGV40055889	0.131686	0.1316857	protein_coding	NM_000195
ASHGV40054895	-0.13166	0.131663037	protein_coding	NM_017900
ASHGV40012129	-0.13163	0.131629687	protein_coding	NM_030765
ASHGV40008803	-0.13151	0.131505055	protein_coding	NM_005553
ASHGV40030903	0.131505	0.131504957	protein_coding	NM_003279
ASHGV40008508	-0.1315	0.131504485	protein_coding	NM_006725
ASHGV40046908	0.131457	0.131457439	protein_coding	NM_005045
ASHGV40015531	0.131438	0.131438091	protein_coding	NM_153046
ASHGV40034796	0.131435	0.131434869	protein_coding	NM_005694
ASHGV40001837	0.131361	0.131360536	protein_coding	ENST00000529296
ASHGV40013215	-0.13132	0.131322965	protein_coding	NM_181503
ASHGV40038185	-0.13131	0.131306963	protein_coding	NM_017707
ASHGV40048036	-0.1312	0.131204964	protein_coding	NM_001110354
ASHGV40024379	-0.13103	0.131034584	protein_coding	NM_001020
ASHGV40048544	0.130993	0.130993485	protein_coding	NM_001166254
ASHGV40009946	-0.13097	0.130972049	protein_coding	NM_001174126
ASHGV40003135	0.130942	0.130942063	protein_coding	NM_001163438
ASHGV40043016	-0.13073	0.130732924	protein_coding	NM_017770
ASHGV40016447	0.130662	0.130662488	protein_coding	NM_006996
ASHGV40050249	-0.13051	0.130510648	protein_coding	NM_003747

ASHGV40030479	0.130506	0.130506427	protein_coding	NM_007219
ASHGV40017334	-0.13047	0.130465707	protein_coding	ENST00000600790
ASHGV40040677	0.130314	0.130314333	protein_coding	NM_005023
ASHGV40053526	0.130301	0.130300983	protein_coding	NM_014064
ASHGV40023058	-0.13029	0.130285855	protein_coding	NM_032048
ASHGV40009052	0.130247	0.130246882	protein_coding	NM_152432
ASHGV40029015	0.130096	0.130095516	protein_coding	NM_004097
ASHGV40054299	0.130004	0.130003723	protein_coding	NM_018301
ASHGV40032948	-0.12998	0.129976643	protein_coding	NM_138467
ASHGV40026052	-0.1299	0.129902253	protein_coding	NM_001278428
ASHGV40054576	-0.12983	0.129827261	protein_coding	NM_002910
ASHGV40055007	-0.12975	0.129751642	protein_coding	NM_001039705
ASHGV40044556	-0.12974	0.12974181	protein_coding	NM_031480
ASHGV40037742	-0.12972	0.129723343	protein_coding	NM_178135
ASHGV40024999	-0.12972	0.129721896	protein_coding	NM_001102651
ASHGV40044958	0.129718	0.129718124	protein_coding	NM_022047
ASHGV40031141	-0.12971	0.129708861	protein_coding	ENST00000370520
ASHGV40050593	0.129567	0.129566718	protein_coding	NM_000749
ASHGV40034022	0.129516	0.129516394	protein_coding	NM_024813
ASHGV40015758	-0.12951	0.129506915	protein_coding	NM_002344
ASHGV40053996	-0.12943	0.129429777	protein_coding	NM_001167676
ASHGV40035800	0.129383	0.12938271	protein_coding	NM_007335
ASHGV40037655	-0.1291	0.129102732	protein_coding	NM_014435
ASHGV40009388	0.129074	0.12907444	protein_coding	NM_016522
ASHGV40006939	0.128964	0.128964025	protein_coding	NM_015308
ASHGV40025350	0.128895	0.128895016	protein_coding	NM_032683
ASHGV40015661	-0.12876	0.128755259	protein_coding	NM_001184879
ASHGV40039210	0.12837	0.128370123	protein_coding	NM_017935
ASHGV40042781	0.128323	0.128322523	protein_coding	NM_014613
ASHGV40032584	-0.12809	0.128091862	protein_coding	NM_001559
ASHGV40048063	0.12805	0.128049594	protein_coding	NM_003193
ASHGV40043223	0.127664	0.127664251	protein_coding	NM_025236
ASHGV40024748	0.12763	0.12762987	protein_coding	NM_033288
ASHGV40035556	-0.12739	0.127389066	protein_coding	NM_014674
ASHGV40024419	0.127361	0.127360731	protein_coding	NM_018035
ASHGV40055573	-0.12728	0.127277525	protein_coding	NM_000116
ASHGV40050795	0.127265	0.127264997	protein_coding	ENST00000391684
ASHGV40041857	-0.12726	0.12725938	protein_coding	NM_001799
ASHGV40039545	-0.12721	0.127208737	protein_coding	NM_000856
ASHGV40048797	-0.12716	0.127160673	protein_coding	NM_175075
ASHGV40006060	-0.12714	0.127142582	protein_coding	NM_014472
ASHGV40035890	0.126965	0.126964638	protein_coding	NM_015340
ASHGV40056256	0.126912	0.126911927	protein_coding	ENST00000525596
ASHGV40053430	-0.12688	0.126880484	protein_coding	NM_014397
ASHGV40054674	-0.12685	0.126851204	protein_coding	NM_001649
ASHGV40007296	0.126541	0.126540818	protein_coding	NM_012309
ASHGV40038444	-0.12644	0.126442279	protein_coding	NM_006315

ASHGV40023952	-0.12644	0.126442	protein_coding	NM_013312
ASHGV40028348	0.126322	0.126322134	protein_coding	ENST00000401641
ASHGV40013330	0.126295	0.126295457	protein_coding	NM_005798
ASHGV40017492	0.126292	0.126292149	protein_coding	NM_001042371
ASHGV40047899	0.126284	0.126283507	protein_coding	NM_015969
ASHGV40049684	-0.12627	0.12627078	protein_coding	NM_006281
ASHGV40008658	-0.12625	0.126253004	protein_coding	NM_152760
ASHGV40037128	-0.1262	0.126200164	protein_coding	ENST00000422806
ASHGV40006485	0.126159	0.126158978	protein_coding	NM_145886
ASHGV40047892	-0.12605	0.126052614	protein_coding	NM_005228
ASHGV40052534	0.12605	0.126049683	protein_coding	NM_013366
ASHGV40013256	0.126008	0.126007753	protein_coding	NM_182508
ASHGV40043290	-0.12584	0.125842095	protein_coding	NM_002904
ASHGV40011653	0.125765	0.125764774	protein_coding	NM_018656
ASHGV40021700	0.125467	0.12546698	protein_coding	NM_001303533
ASHGV40036921	0.125444	0.125443992	protein_coding	NM_001009921
ASHGV40048327	0.125396	0.125396417	protein_coding	NM_001550
ASHGV40010005	-0.12532	0.125315961	protein_coding	NM_000966
ASHGV40023653	0.125154	0.125154415	protein_coding	NM_005035
ASHGV40054466	0.125088	0.1250876	protein_coding	NM_004840
ASHGV40056577	0.125081	0.125080623	protein_coding	NM_001039886
ASHGV40041927	0.125075	0.125075344	protein_coding	NM_000859
ASHGV40056560	-0.12507	0.125066715	protein_coding	NM_001080469
ASHGV40035080	0.124998	0.124997818	protein_coding	NM_001010883
ASHGV40007237	0.124978	0.124977514	protein_coding	NM_080658
ASHGV40036866	-0.12494	0.12494052	protein_coding	NM_133462
ASHGV40016804	-0.12493	0.124926301	protein_coding	NM_007347
ASHGV40017351	0.124833	0.124833147	protein_coding	NM_183376
ASHGV40056640	-0.12471	0.124705889	protein_coding	NM_138458
ASHGV40042069	0.124564	0.124563559	protein_coding	NM_173362
ASHGV40022352	0.12455	0.124550272	protein_coding	NM_004712
ASHGV40010248	-0.12451	0.124508748	protein_coding	NM_004616
ASHGV40022555	0.124501	0.124500672	protein_coding	NM_001004492
ASHGV40003114	0.124418	0.124417823	protein_coding	NM_001101340
ASHGV40037833	0.124358	0.124357636	protein_coding	NM_025212
ASHGV40002087	-0.12426	0.124263246	protein_coding	ENST00000553678
ASHGV40010609	-0.12421	0.124210491	protein_coding	NM_024072
ASHGV40033597	-0.12398	0.123980547	protein_coding	NM_013387
ASHGV40028834	0.123927	0.123927005	protein_coding	NM_000179
ASHGV40036546	0.123924	0.123923588	protein_coding	NM_017548
ASHGV40055673	-0.12375	0.123745801	protein_coding	NM_001008
ASHGV40036741	0.123714	0.123713511	protein_coding	NM_022736
ASHGV40021843	-0.12367	0.12367415	protein_coding	NM_014834
ASHGV40042311	-0.12334	0.12334187	protein_coding	NM_001287252
ASHGV40015106	0.123216	0.123215971	protein_coding	NM_001303095
ASHGV40048050	0.122789	0.122788596	protein_coding	NM_020432
ASHGV40019326	-0.12274	0.122742037	protein_coding	NM_007014

ASHGV40052187	-0.12273	0.122728685	protein_coding	NM_005156
ASHGV40052373	0.122728	0.122727915	protein_coding	NM_000755
ASHGV40023589	-0.12268	0.122677785	protein_coding	NM_001480
ASHGV40012389	0.12257	0.122570303	protein_coding	NM_001105577
ASHGV40024044	-0.12252	0.122518558	protein_coding	NM_024881
ASHGV40030815	0.12248	0.122480303	protein_coding	NM_016308
ASHGV40056244	-0.12242	0.122418208	protein_coding	NM_201400
ASHGV40031935	-0.1224	0.122402798	protein_coding	NM_020882
ASHGV40018785	-0.12225	0.122250735	protein_coding	NM_020248
ASHGV40002602	-0.12225	0.122250197	protein_coding	ENST00000594664
ASHGV40025600	-0.12202	0.122016878	protein_coding	NM_014266
ASHGV40020172	0.121987	0.121986662	protein_coding	NM_006584
ASHGV40006636	0.121934	0.121933642	protein_coding	NM_005418
ASHGV40033218	-0.12191	0.121911207	protein_coding	NM_000262
ASHGV40055263	0.121903	0.121902998	protein_coding	NM_020384
ASHGV40008799	0.121856	0.121856157	protein_coding	NM_018161
ASHGV40030819	0.121851	0.12185127	protein_coding	NM_006698
ASHGV40034333	0.121838	0.121838456	protein_coding	NM_017730
ASHGV40025812	0.121804	0.121804185	protein_coding	NM_006732
ASHGV40026529	-0.12178	0.121775478	protein_coding	NM_022128
ASHGV40018445	0.121747	0.121747112	protein_coding	NM_013275
ASHGV40000074	-0.12172	0.121715316	protein_coding	NM_181756
ASHGV40017071	-0.12168	0.121678387	protein_coding	NM_017828
ASHGV40021753	0.121553	0.121553288	protein_coding	NM_005854
ASHGV40017825	0.121511	0.121511432	protein_coding	NM_178863
ASHGV40052702	-0.12149	0.12148581	protein_coding	NM_022160
ASHGV40057717	-0.12146	0.12146144	protein_coding	NM_207009
ASHGV40027950	-0.12145	0.121445204	protein_coding	NM_213589
ASHGV40029913	0.121419	0.121419372	protein_coding	NM_001130158
ASHGV40038542	-0.12138	0.121377784	protein_coding	NM_018366
ASHGV40034561	-0.12135	0.121346681	protein_coding	NM_015009
ASHGV40040300	0.121322	0.121322469	protein_coding	NM_014473
ASHGV40045346	0.121254	0.12125364	protein_coding	NM_002526
ASHGV40007499	-0.12114	0.121143145	protein_coding	NM_032427
ASHGV40021743	0.121072	0.121071645	protein_coding	NM_001130021
ASHGV40037103	0.120958	0.120958137	protein_coding	NM_001294341
ASHGV40030656	-0.12094	0.120943994	protein_coding	NM_012072
ASHGV40042176	0.120779	0.120779343	protein_coding	NM_020318
ASHGV40025847	0.120767	0.120766739	protein_coding	NM_001736
ASHGV40048144	0.12076	0.120759896	protein_coding	NM_004126
ASHGV40014985	-0.12063	0.120632524	protein_coding	NM_030755
ASHGV40055868	-0.12048	0.120480096	protein_coding	NM_032900
ASHGV40000159	-0.12043	0.120427468	protein_coding	NM_138779
ASHGV40005797	0.12024	0.120239563	protein_coding	NM_173348
ASHGV40018877	-0.12018	0.120184603	protein_coding	NM_001287251
ASHGV40017180	0.120154	0.120154106	protein_coding	NM_001080435
ASHGV40032918	-0.12014	0.120143407	protein_coding	uc010gtv.1

ASHGV40036058	0.120125	0.120124821	protein_coding	NM_002841
ASHGV40027118	-0.12011	0.120107181	protein_coding	NM_025190
ASHGV40048762	0.120095	0.120095399	protein_coding	NM_198076
ASHGV40028726	0.120008	0.120007733	protein_coding	NM_016441
ASHGV40006530	0.119979	0.119978971	protein_coding	NM_001164377
ASHGV40020759	-0.11977	0.119765428	protein_coding	NM_004694
ASHGV40031570	-0.11966	0.119655365	protein_coding	NM_030877
ASHGV40021553	0.119464	0.119463965	protein_coding	NM_015355
ASHGV40038484	0.11942	0.119420433	protein_coding	NM_001256666
ASHGV40026908	0.119414	0.119413894	protein_coding	NM_003236
ASHGV40019715	0.119394	0.119393723	protein_coding	NM_016376
ASHGV40013865	-0.11937	0.119366604	protein_coding	NM_030913
ASHGV40030398	0.119301	0.119300534	protein_coding	NM_001001891
ASHGV40042735	0.119258	0.119257601	protein_coding	NM_016093
ASHGV40020612	0.119207	0.119206958	protein_coding	NM_001271875
ASHGV40056633	-0.11912	0.11911535	protein_coding	NM_019060
ASHGV40010490	0.1191	0.11909982	protein_coding	NM_001111285
ASHGV40017425	0.118903	0.118902618	protein_coding	NM_022493
ASHGV40017231	-0.11885	0.11885045	protein_coding	NM_002201
ASHGV40017382	0.118844	0.118844461	protein_coding	NM_024652
ASHGV40013603	-0.11866	0.11865527	protein_coding	NM_005766
ASHGV40023384	-0.11866	0.11865504	protein_coding	NM_002747
ASHGV40011978	-0.1186	0.118600471	protein_coding	NM_033121
ASHGV40052451	0.118414	0.118413506	protein_coding	NM_007371
ASHGV40049542	-0.11841	0.118412762	protein_coding	NM_022133
ASHGV40036748	0.118343	0.118342567	protein_coding	NM_001168214
ASHGV40031515	-0.11824	0.118243743	protein_coding	NM_080825
ASHGV40007839	0.118117	0.118117011	protein_coding	NM_014155
ASHGV40023712	0.118106	0.1181062	protein_coding	NM_144564
ASHGV40053444	0.118012	0.118011974	protein_coding	NM_001282679
ASHGV40034232	-0.11796	0.117955365	protein_coding	NM_144719
ASHGV40029154	0.11793	0.11793042	protein_coding	NM_024595
ASHGV40036689	-0.11792	0.117920055	protein_coding	NM_207293
ASHGV40021914	-0.11768	0.117679215	protein_coding	NM_002611
ASHGV40037813	-0.11764	0.11763878	protein_coding	NM_016242
ASHGV40032618	0.117605	0.117604659	protein_coding	NM_058182
ASHGV40054331	0.117557	0.117557076	protein_coding	NM_178175
ASHGV40050261	-0.11746	0.117455146	protein_coding	NM_015458
ASHGV40051290	-0.11746	0.117455034	protein_coding	NM_001010896
ASHGV40032739	-0.11743	0.117433163	protein_coding	NM_001036645
ASHGV40044344	0.117431	0.117430634	protein_coding	NM_020133
ASHGV40056794	-0.11704	0.117035514	protein_coding	NM_006886
ASHGV40034941	0.117026	0.117025574	protein_coding	NM_016201
ASHGV40056241	0.116874	0.116874375	protein_coding	NM_033208
ASHGV40033178	-0.11687	0.116873358	protein_coding	NM_002608
ASHGV40060864	-0.11687	0.116869011	protein_coding	uc010hmp.1
ASHGV40024645	0.116848	0.116847618	protein_coding	NM_001571

ASHGV40042063	-0.11682	0.116818994	protein_coding	NM_032290
ASHGV40053539	0.116756	0.116756315	protein_coding	NM_003934
ASHGV40025394	-0.11675	0.116751952	protein_coding	NM_033204
ASHGV40002601	0.116693	0.116693267	protein_coding	ENST00000594663
ASHGV40000079	0.11662	0.116619885	protein_coding	NM_001134493
ASHGV40053981	-0.11656	0.116557814	protein_coding	NM_021014
ASHGV40017482	-0.11647	0.116466158	protein_coding	NM_178167
ASHGV40021893	0.116409	0.116409365	protein_coding	NM_023079
ASHGV40026149	-0.11633	0.116327499	protein_coding	NM_173632
ASHGV40048948	0.116316	0.116315993	protein_coding	NM_019851
ASHGV40012150	-0.11628	0.11628254	protein_coding	NM_020936
ASHGV40009561	0.116058	0.116057732	protein_coding	NM_002864
ASHGV40054134	0.115979	0.11597945	protein_coding	NM_018486
ASHGV40035217	-0.11595	0.115952659	protein_coding	NM_005241
ASHGV40051922	0.115942	0.115942334	protein_coding	NM_024617
ASHGV40034041	0.115927	0.115927442	protein_coding	NM_001302378
ASHGV40027882	-0.11589	0.115892019	protein_coding	NM_004226
ASHGV40039215	0.115802	0.115802219	protein_coding	NM_001165412
ASHGV40011356	0.115711	0.11571142	protein_coding	NM_175744
ASHGV40046434	0.115693	0.1156934	protein_coding	NM_018224
ASHGV40025488	-0.1156	0.115600059	protein_coding	NM_001100631
ASHGV40008486	0.115352	0.115351994	protein_coding	NM_024848
ASHGV40010960	-0.11499	0.114990332	protein_coding	NM_001769
ASHGV40055083	-0.11484	0.114842737	protein_coding	NM_001013627
ASHGV40031130	-0.11484	0.114835829	protein_coding	NM_007232
ASHGV40024811	0.114827	0.114827151	protein_coding	NM_014931
ASHGV40041493	-0.11472	0.114719001	protein_coding	NM_001278317
ASHGV40030983	0.114709	0.114708988	protein_coding	NM_199129
ASHGV40042080	0.114637	0.11463689	protein_coding	NM_022350
ASHGV40003096	-0.11452	0.114515403	protein_coding	NM_001039380
ASHGV40029340	0.114282	0.114281597	protein_coding	NM_198581
ASHGV40007174	0.114191	0.114190826	protein_coding	NM_138368
ASHGV40026357	-0.11405	0.114049301	protein_coding	NM_198256
ASHGV40048429	-0.11364	0.113639055	protein_coding	NM_014390
ASHGV40056623	0.113539	0.113538599	protein_coding	NM_014011
ASHGV40057032	0.113364	0.113364491	protein_coding	NM_001968
ASHGV40006368	0.113276	0.113275968	protein_coding	NM_001290223
ASHGV40024773	0.11325	0.113249781	protein_coding	ENST00000222224
ASHGV40034243	-0.11318	0.113183377	protein_coding	NM_018075
ASHGV40046283	-0.11307	0.11306558	protein_coding	NM_006990
ASHGV40057671	0.113047	0.113046764	protein_coding	NM_005840
ASHGV40021006	0.113028	0.113028284	protein_coding	NM_000918
ASHGV40035009	0.113007	0.113006506	protein_coding	NM_019001
ASHGV40053828	0.112855	0.112855174	protein_coding	NM_198279
ASHGV40047602	0.112796	0.112796094	protein_coding	NM_001031710
ASHGV40012887	-0.11278	0.11278394	protein_coding	NM_001101663
ASHGV40024579	0.112643	0.112643462	protein_coding	NM_001301059

ASHGV40021561	-0.11256	0.112556511	protein_coding	NM_003457
ASHGV40035798	0.112539	0.112538618	protein_coding	NM_001008392
ASHGV40015129	-0.11251	0.112513434	protein_coding	NM_001172
ASHGV40006098	0.112483	0.11248292	protein_coding	NM_017893
ASHGV40055276	-0.11245	0.112452714	protein_coding	NM_000495
ASHGV40018930	-0.11228	0.112277049	protein_coding	NM_001105079
ASHGV40011946	0.112216	0.112215661	protein_coding	NM_203436
ASHGV40036283	0.112099	0.112098685	protein_coding	NM_001085451
ASHGV40047461	0.112097	0.112096748	protein_coding	NM_001010858
ASHGV40016657	0.112081	0.112080641	protein_coding	NM_001013703
ASHGV40055674	-0.11207	0.112066625	protein_coding	NM_003411
ASHGV40013665	-0.11188	0.111882046	protein_coding	NM_032859
ASHGV40051166	0.111808	0.111808371	protein_coding	uc003ypu.2
ASHGV40001410	-0.1118	0.1118009	protein_coding	NM_031454
ASHGV40000199	0.111719	0.11171859	protein_coding	NM_152384
ASHGV40029543	0.111501	0.111501321	protein_coding	NM_001301237
ASHGV40025298	-0.11147	0.111474481	protein_coding	NM_001286
ASHGV40034394	0.111436	0.11143628	protein_coding	NM_004704
ASHGV40055724	-0.11142	0.111421043	protein_coding	NM_001039567
ASHGV40043311	-0.11138	0.111378565	protein_coding	NM_004557
ASHGV40008622	0.111222	0.111221974	protein_coding	NM_006782
ASHGV40034258	0.111195	0.11119495	protein_coding	NM_001135179
ASHGV40036724	0.111189	0.111189433	protein_coding	NM_015508
ASHGV40002674	0.111023	0.111022855	protein_coding	ENST00000599312
ASHGV40040289	-0.11068	0.110678741	protein_coding	NM_000082
ASHGV40007559	-0.11063	0.110628392	protein_coding	NM_033292
ASHGV40025103	0.110627	0.110627302	protein_coding	NM_020902
ASHGV40007419	0.110406	0.11040587	protein_coding	NM_147193
ASHGV40008743	0.110377	0.110377035	protein_coding	NM_001164161
ASHGV40036549	0.110366	0.11036586	protein_coding	NM_021203
ASHGV40000202	0.110243	0.110242919	protein_coding	uc021pzk.1
ASHGV40048117	-0.11021	0.110213105	protein_coding	NM_012129
ASHGV40043491	-0.11017	0.110165918	protein_coding	NM_002098
ASHGV40030243	-0.11013	0.110125148	protein_coding	NM_001144994
ASHGV40045889	0.110122	0.11012246	protein_coding	NM_003898
ASHGV40011685	0.1101	0.110100385	protein_coding	NM_018279
ASHGV40024120	0.110018	0.110017887	protein_coding	NM_001099269
ASHGV40047797	0.109901	0.10990074	protein_coding	NM_000596
ASHGV40031560	0.109881	0.109880761	protein_coding	NM_002951
ASHGV40021731	-0.10982	0.109815363	protein_coding	NM_152467
ASHGV40051998	-0.1098	0.109801788	protein_coding	NM_014057
ASHGV40046133	0.109682	0.109682137	protein_coding	NM_004227
ASHGV40039132	0.109667	0.109667454	protein_coding	NM_005935
ASHGV40009132	0.109638	0.109638422	protein_coding	NM_001082970
ASHGV40039058	-0.10954	0.109542191	protein_coding	NM_001144978
ASHGV40039709	0.109397	0.109396599	protein_coding	NM_024949
ASHGV40035531	0.109393	0.109392928	protein_coding	NM_004978

ASHGV40052086	0.109393	0.109392699	protein_coding	NM_033087
ASHGV40014988	-0.10936	0.10936344	protein_coding	NM_001267046
ASHGV40048264	-0.10928	0.109284898	protein_coding	NM_024653
ASHGV40009939	0.10923	0.109230214	protein_coding	NM_013277
ASHGV40048558	0.109227	0.109226519	protein_coding	NM_003143
ASHGV40029976	0.108988	0.108988379	protein_coding	NM_001136039
ASHGV40024978	-0.10893	0.108927983	protein_coding	NM_005026
ASHGV40052546	-0.10892	0.108920134	protein_coding	NM_017820
ASHGV40024668	-0.10888	0.108881687	protein_coding	NM_138334
ASHGV40043938	-0.10886	0.108856758	protein_coding	NM_014797
ASHGV40015186	-0.10879	0.10878755	protein_coding	NM_182894
ASHGV40031493	-0.10858	0.10858116	protein_coding	NM_015547
ASHGV40033770	-0.10844	0.108440709	protein_coding	NM_001429
ASHGV40018195	0.108116	0.108115859	protein_coding	NM_005652
ASHGV40046612	0.108084	0.108084367	protein_coding	NM_000181
ASHGV40042412	-0.10803	0.108026689	protein_coding	NM_012208
ASHGV40003320	0.107956	0.107955709	protein_coding	NM_033127
ASHGV40053310	0.107918	0.107917601	protein_coding	NM_003358
ASHGV40035967	-0.10767	0.107672358	protein_coding	NM_007022
ASHGV40026201	0.107664	0.107664329	protein_coding	NM_030812
ASHGV40015080	0.107619	0.107619291	protein_coding	NM_006255
ASHGV40012275	-0.10748	0.107484473	protein_coding	NM_170682
ASHGV40039143	-0.10747	0.107468976	protein_coding	NM_000297
ASHGV40025832	-0.10747	0.107465205	protein_coding	ENST00000377652
ASHGV40042700	0.107286	0.107285917	protein_coding	NM_001205293
ASHGV40055832	-0.10721	0.10721082	protein_coding	NM_013451
ASHGV40016276	0.107113	0.107112555	protein_coding	NM_032856
ASHGV40040684	0.107062	0.107061525	protein_coding	NM_004707
ASHGV40053355	-0.10692	0.106922628	protein_coding	NM_012210
ASHGV40051794	0.106753	0.106752849	protein_coding	ENST00000543078
ASHGV40038250	0.106696	0.106695689	protein_coding	NM_012224
ASHGV40045060	0.106604	0.106603527	protein_coding	NM_006586
ASHGV40014168	0.106354	0.106354227	protein_coding	NM_139318
ASHGV40033949	-0.10629	0.106288366	protein_coding	NM_182760
ASHGV40018072	-0.10627	0.106267237	protein_coding	NM_001896
ASHGV40034979	-0.10624	0.106239442	protein_coding	NM_023067
ASHGV40033646	-0.10592	0.105922317	protein_coding	uc021opj.1
ASHGV40023559	-0.10557	0.105572767	protein_coding	NM_001308210
ASHGV40006495	0.105527	0.105526585	protein_coding	NM_004420
ASHGV40005958	0.105521	0.105520856	protein_coding	NM_000043
ASHGV40006047	0.105411	0.10541067	protein_coding	NM_024954
ASHGV40048438	-0.10536	0.105361895	protein_coding	NM_001012454
ASHGV40036430	-0.10536	0.10535604	protein_coding	NM_016298
ASHGV40010731	-0.10481	0.104808061	protein_coding	NM_022782
ASHGV40010985	-0.10478	0.10477585	protein_coding	NM_133181
ASHGV40052927	0.104677	0.104676563	protein_coding	NM_138333
ASHGV40046348	0.10462	0.104620222	protein_coding	NM_203288

ASHGV40009035	-0.10434	0.104335656	protein_coding	NM_014679
ASHGV40011939	-0.10431	0.104313955	protein_coding	NM_152261
ASHGV40003302	0.104278	0.104277781	protein_coding	NM_022804
ASHGV40010585	-0.10424	0.10424022	protein_coding	NM_139283
ASHGV40018625	0.104222	0.104221802	protein_coding	NM_012368
ASHGV40013811	0.104134	0.104133558	protein_coding	NM_019852
ASHGV40037607	-0.10402	0.104024391	protein_coding	NM_058167
ASHGV40050725	0.103866	0.103865933	protein_coding	NM_173519
ASHGV40038783	-0.10383	0.103834974	protein_coding	NM_015173
ASHGV40055374	-0.10369	0.103690638	protein_coding	NM_000276
ASHGV40001284	-0.1036	0.103601393	protein_coding	NM_025222
ASHGV40005588	-0.10355	0.103551069	protein_coding	NM_000698
ASHGV40048891	0.103523	0.10352259	protein_coding	NM_173683
ASHGV40036608	0.10332	0.103320498	protein_coding	NM_002670
ASHGV40024528	-0.10326	0.10326493	protein_coding	NM_025136
ASHGV40016002	0.103262	0.103261643	protein_coding	NM_139242
ASHGV40040519	0.103235	0.103235097	protein_coding	NM_001289007
ASHGV40013584	0.103162	0.103161825	protein_coding	NM_006260
ASHGV40047123	0.103027	0.103027242	protein_coding	NM_001003665
ASHGV40015501	0.102785	0.102785433	protein_coding	uc001ymf.1
ASHGV40044257	0.102594	0.102593661	protein_coding	NM_017909
ASHGV40031472	-0.10248	0.102481323	protein_coding	NM_002165
ASHGV40012146	-0.10247	0.102470827	protein_coding	NM_020382
ASHGV40030260	-0.10243	0.102434375	protein_coding	NM_031313
ASHGV40035847	0.102199	0.102198958	protein_coding	NM_005385
ASHGV40041610	0.102181	0.102180926	protein_coding	NM_024867
ASHGV40047515	0.10209	0.102090073	protein_coding	NM_138426
ASHGV40019649	0.102053	0.10205335	protein_coding	NM_018289
ASHGV40008528	0.102018	0.10201764	protein_coding	NM_004111
ASHGV40050481	0.101895	0.10189452	protein_coding	NM_014688
ASHGV40047283	0.101861	0.101861309	protein_coding	NM_004911
ASHGV40017742	0.101658	0.101658108	protein_coding	NM_015044
ASHGV40056910	-0.10163	0.101627674	protein_coding	NM_002218
ASHGV40050378	0.101626	0.101625508	protein_coding	NM_152272
ASHGV40023820	0.101604	0.101603641	protein_coding	NM_006351
ASHGV40035928	-0.10141	0.1014132	protein_coding	NM_130384
ASHGV40038676	-0.10137	0.101372157	protein_coding	NM_004787
ASHGV40015202	0.101339	0.101339012	protein_coding	NM_014239
ASHGV40014175	0.101199	0.101199225	protein_coding	NM_080666
ASHGV40042601	0.101193	0.101193344	protein_coding	NM_001128209
ASHGV40000639	-0.10113	0.101134343	protein_coding	NM_006913
ASHGV40051410	-0.10104	0.101044637	protein_coding	uc003zbp.3
ASHGV40010718	0.101012	0.101011641	protein_coding	NM_022916
ASHGV40030128	-0.10097	0.100967954	protein_coding	NM_021198
ASHGV40021659	0.100892	0.100892067	protein_coding	NM_014598
ASHGV40025741	-0.10086	0.100861195	protein_coding	NM_004706
ASHGV40009731	0.100854	0.100853574	protein_coding	NM_002223

ASHGV40022035	-0.10078	0.100784622	protein_coding	NM_018149
ASHGV40020319	0.100718	0.100718332	protein_coding	NM_002277
ASHGV40018396	-0.10064	0.100642341	protein_coding	NM_015144
ASHGV40047443	0.100598	0.100598243	protein_coding	uc021zys.1
ASHGV40014536	-0.10048	0.100481677	protein_coding	ENST00000499006
ASHGV40028931	0.100446	0.100445877	protein_coding	NM_015147
ASHGV40052443	0.100402	0.100401871	protein_coding	NM_017585
ASHGV40009909	-0.10038	0.100384278	protein_coding	NM_001240
ASHGV40057513	-0.10028	0.100280678	protein_coding	NM_012166
ASHGV40008451	0.100227	0.100227222	protein_coding	NM_015457
ASHGV40002344	0.100052	0.100051529	protein_coding	ENST00000568879
ASHGV40021874	-0.09982	0.099819896	protein_coding	NM_013323
ASHGV40033994	-0.0998	0.09980403	protein_coding	NM_183352
ASHGV40009024	0.099781	0.099780527	protein_coding	NM_015036
ASHGV40018951	-0.09973	0.099730172	protein_coding	NM_005881
ASHGV40002157	-0.09973	0.099727767	protein_coding	NM_001013657
ASHGV40042980	0.099692	0.099692419	protein_coding	NM_003144
ASHGV40028899	0.099591	0.099590937	protein_coding	NM_001129993
ASHGV40024900	0.099489	0.099488932	protein_coding	NM_003433
ASHGV40024884	0.099488	0.099487539	protein_coding	NM_152475
ASHGV40026426	0.099478	0.09947848	protein_coding	NM_014713
ASHGV40038923	-0.09921	0.099212862	protein_coding	NM_025009
ASHGV40045505	0.099086	0.099085583	protein_coding	NM_003080
ASHGV40048302	0.09908	0.099080337	protein_coding	NM_012257
ASHGV40006850	0.099059	0.099058829	protein_coding	NM_003189
ASHGV40029741	0.098892	0.098892428	protein_coding	NM_001039724
ASHGV40033977	-0.09879	0.098786605	protein_coding	NM_020165
ASHGV40024049	-0.09862	0.098615586	protein_coding	NM_015692
ASHGV40051142	0.098578	0.098578111	protein_coding	NM_022783
ASHGV40033519	-0.09825	0.098246993	protein_coding	NM_003327
ASHGV40024286	0.098203	0.098202585	protein_coding	NM_144987
ASHGV40053968	0.098185	0.098184781	protein_coding	NM_007130
ASHGV40010172	0.098163	0.098163029	protein_coding	ENST00000594966
ASHGV40040400	0.098163	0.098162794	protein_coding	NM_001164443
ASHGV40014044	0.098141	0.098140968	protein_coding	NM_006939
ASHGV40031666	0.09806	0.098059636	protein_coding	NM_006282
ASHGV40025842	-0.09788	0.097877836	protein_coding	NM_178840
ASHGV40021469	-0.09771	0.097713165	protein_coding	NM_015626
ASHGV40051165	-0.09769	0.097692619	protein_coding	NM_145647
ASHGV40025168	-0.09765	0.097645745	protein_coding	NM_003259
ASHGV40017556	-0.09746	0.097461491	protein_coding	NM_003223
ASHGV40041053	-0.09736	0.097363432	protein_coding	NM_032782
ASHGV40022751	0.09723	0.097230387	protein_coding	NM_004671
ASHGV40052412	-0.09723	0.097230131	protein_coding	NM_033387
ASHGV40030826	-0.09718	0.097183764	protein_coding	NM_004613
ASHGV40034806	0.097176	0.097176449	protein_coding	NM_173825
ASHGV40033023	-0.09715	0.097154272	protein_coding	NM_032204

ASHGV40045856	0.09684	0.096840455	protein_coding	NM_001008503
ASHGV40033773	0.096826	0.096825673	protein_coding	NM_017590
ASHGV40024986	0.096813	0.096813373	protein_coding	NM_007165
ASHGV40022259	0.096526	0.096525687	protein_coding	NM_001080510
ASHGV40041314	-0.09645	0.096454882	protein_coding	NM_004168
ASHGV40060902	0.096353	0.096352795	protein_coding	uc031pxv.1
ASHGV40011288	-0.09625	0.09624604	protein_coding	NM_152641
ASHGV40025660	-0.09603	0.09602911	protein_coding	NM_004924
ASHGV40033031	-0.09601	0.09600873	protein_coding	NM_031937
ASHGV40011659	0.095979	0.095978816	protein_coding	NM_025073
ASHGV40052448	0.095958	0.095957581	protein_coding	NM_001134398
ASHGV40012016	-0.09556	0.095557205	protein_coding	NM_004416
ASHGV40020087	-0.09518	0.095182626	protein_coding	NM_138463
ASHGV40054603	0.095056	0.09505602	protein_coding	NM_018196
ASHGV40057615	0.095005	0.09500484	protein_coding	NM_002565
ASHGV40048924	0.094994	0.0949937	protein_coding	NM_152609
ASHGV40021179	0.09477	0.094769747	protein_coding	NM_001033002
ASHGV40044982	0.094712	0.094711912	protein_coding	NM_001286635
ASHGV40007031	-0.09461	0.094607238	protein_coding	NM_017840
ASHGV40039813	0.094554	0.094553762	protein_coding	uc010ita.3
ASHGV40009806	-0.09449	0.094485578	protein_coding	NM_001040436
ASHGV40027278	-0.09429	0.094291857	protein_coding	NM_000576
ASHGV40044321	-0.09424	0.094235906	protein_coding	NM_054114
ASHGV40021450	-0.09415	0.094153737	protein_coding	NM_021012
ASHGV40023056	-0.09409	0.094094959	protein_coding	NM_015295
ASHGV40046672	-0.09397	0.093974049	protein_coding	NM_001281447
ASHGV40017267	-0.0939	0.093904013	protein_coding	NM_000057
ASHGV40036555	-0.0937	0.093702457	protein_coding	NM_004441
ASHGV40040297	-0.09337	0.093366925	protein_coding	NM_004983
ASHGV40045457	0.093298	0.093297621	protein_coding	NM_002389
ASHGV40042780	-0.09318	0.0931792	protein_coding	NM_138820
ASHGV40035343	0.093113	0.09311272	protein_coding	NM_024871
ASHGV40012390	0.093109	0.0931094	protein_coding	NM_004119
ASHGV40045492	-0.09306	0.093060587	protein_coding	NM_032131
ASHGV40019604	0.092935	0.092934995	protein_coding	uc010vph.1
ASHGV40013872	0.092903	0.092903401	protein_coding	NM_138452
ASHGV40019298	-0.09269	0.092689411	protein_coding	NM_173165
ASHGV40026938	0.092631	0.092630908	protein_coding	NM_001965
ASHGV40046213	0.092624	0.092623552	protein_coding	NM_016121
ASHGV40044263	-0.09239	0.092388309	protein_coding	NM_018085
ASHGV40023139	-0.09236	0.092355565	protein_coding	NM_153000
ASHGV40007255	-0.09233	0.092328272	protein_coding	NM_181514
ASHGV40026706	0.092284	0.092283524	protein_coding	NM_139279
ASHGV40041272	-0.09216	0.092161654	protein_coding	NM_005520
ASHGV40035352	0.09209	0.092090341	protein_coding	NM_000460
ASHGV40051262	-0.09208	0.092075716	protein_coding	NM_003235
ASHGV40028711	-0.09197	0.091966438	protein_coding	NM_153212

ASHGV40052469	0.091915	0.091915243	protein_coding	NM_001048265
ASHGV40053964	0.091866	0.091865676	protein_coding	NM_001135998
ASHGV40021660	0.091771	0.091771338	protein_coding	NM_001199417
ASHGV40007673	0.091733	0.091732512	protein_coding	NM_032725
ASHGV40009257	0.09171	0.09170974	protein_coding	NM_178507
ASHGV40023788	0.091686	0.091686436	protein_coding	NM_003685
ASHGV40054855	-0.09166	0.091660533	protein_coding	NM_001356
ASHGV40046884	0.091605	0.091605107	protein_coding	NM_014343
ASHGV40057646	0.091537	0.091537253	protein_coding	NM_004966
ASHGV40031254	0.091271	0.091271405	protein_coding	NM_139321
ASHGV40047047	0.091116	0.091116401	protein_coding	NM_012338
ASHGV40034573	0.091113	0.091112881	protein_coding	NM_001128223
ASHGV40039051	0.091111	0.091110792	protein_coding	NM_000748
ASHGV40008955	-0.09105	0.091049228	protein_coding	NM_016401
ASHGV40004877	0.090933	0.090932839	protein_coding	NM_033397
ASHGV40019705	-0.09092	0.090920603	protein_coding	NM_001114118
ASHGV40026032	-0.09092	0.090919422	protein_coding	NM_031895
ASHGV40048856	-0.09082	0.090819329	protein_coding	NM_001256871
ASHGV40018601	-0.09081	0.090813701	protein_coding	NM_138439
ASHGV40000073	-0.09075	0.090751556	protein_coding	NM_033053
ASHGV40002501	-0.0906	0.090603464	protein_coding	ENST00000586012
ASHGV40021626	0.090589	0.090589325	protein_coding	NM_004773
ASHGV40030907	-0.09054	0.090539205	protein_coding	NM_006227
ASHGV40026139	-0.09019	0.09018943	protein_coding	NM_001023561
ASHGV40053440	-0.08993	0.089926599	protein_coding	NM_001045476
ASHGV40003208	0.089921	0.089921015	protein_coding	NM_001282468
ASHGV40000540	-0.08971	0.089712721	protein_coding	ENST00000422294
ASHGV40055696	0.089591	0.089591094	protein_coding	ENST00000429039
ASHGV40024710	-0.08947	0.089474281	protein_coding	NM_001297436
ASHGV40018929	-0.08947	0.089466728	protein_coding	NM_024031
ASHGV40036040	-0.08942	0.089422484	protein_coding	NM_007042
ASHGV40000158	0.089346	0.089346087	protein_coding	NM_001320
ASHGV40034055	-0.08926	0.089261547	protein_coding	NM_206831
ASHGV40038386	-0.08926	0.089256832	protein_coding	NM_005245
ASHGV40006132	-0.08921	0.089214978	protein_coding	NM_030912
ASHGV40045125	-0.08882	0.088815515	protein_coding	NM_153839
ASHGV40047143	0.08881	0.088809884	protein_coding	NM_032842
ASHGV40020428	-0.08878	0.088775149	protein_coding	NM_133373
ASHGV40016026	0.088716	0.088716061	protein_coding	NM_000968
ASHGV40053246	-0.08832	0.088319243	protein_coding	NM_015469
ASHGV40028923	0.088302	0.088301668	protein_coding	NM_014181
ASHGV40026832	0.088247	0.088247284	protein_coding	NM_016516
ASHGV40007157	0.08809	0.088089579	protein_coding	ENST00000526623
ASHGV40037832	-0.08807	0.088066743	protein_coding	NM_001059
ASHGV40039325	0.088019	0.088018605	protein_coding	NM_020961
ASHGV40034559	-0.08794	0.087943765	protein_coding	NM_018130
ASHGV40026951	0.08793	0.087930322	protein_coding	NM_001145054

ASHGV40037089	0.087917	0.087916756	protein_coding	NM_000996
ASHGV40018493	-0.08787	0.087867252	protein_coding	NM_148920
ASHGV40025773	0.087855	0.087854529	protein_coding	NM_013360
ASHGV40040865	-0.08783	0.087833237	protein_coding	NM_004661
ASHGV40038842	-0.08782	0.08782367	protein_coding	NM_021927
ASHGV40008247	-0.08779	0.087792216	protein_coding	NM_019040
ASHGV40054823	-0.08769	0.087694725	protein_coding	NM_021083
ASHGV40056435	-0.08735	0.087349963	protein_coding	NM_004066
ASHGV40027132	0.087305	0.087305441	protein_coding	NM_138798
ASHGV40012883	-0.0871	0.087102453	protein_coding	NM_033132
ASHGV40022971	0.087077	0.087076539	protein_coding	NM_014643
ASHGV40003336	-0.08706	0.087063291	protein_coding	NM_145290
ASHGV40022679	-0.08703	0.087028752	protein_coding	NM_001099406
ASHGV40019793	0.086843	0.086842844	protein_coding	NM_152766
ASHGV40039284	-0.08684	0.086840042	protein_coding	uc021pav.1
ASHGV40033333	0.086677	0.086676545	protein_coding	NM_024105
ASHGV40007888	0.086554	0.08655409	protein_coding	NM_002817
ASHGV40046674	0.086488	0.08648816	protein_coding	ENST00000329959
ASHGV40005556	0.086142	0.08614215	protein_coding	NM_018590
ASHGV40012147	-0.08593	0.085929096	protein_coding	NM_022717
ASHGV40046115	0.085889	0.085889067	protein_coding	NM_001100600
ASHGV40006052	-0.08586	0.085862253	protein_coding	NM_018425
ASHGV40053542	0.085806	0.085806408	protein_coding	NM_014285
ASHGV40025736	-0.08576	0.085758066	protein_coding	NM_001815
ASHGV40017462	-0.08573	0.085727546	protein_coding	NM_001287
ASHGV40032639	-0.08572	0.085724347	protein_coding	NM_005128
ASHGV40037088	0.085642	0.085642488	protein_coding	uc003fyk.2
ASHGV40014252	0.085605	0.085604737	protein_coding	NM_001284230
ASHGV40031692	0.085599	0.085598851	protein_coding	NM_080752
ASHGV40017571	0.085557	0.085557281	protein_coding	NM_032569
ASHGV40025086	-0.08504	0.085040074	protein_coding	NM_001974
ASHGV40003148	-0.08495	0.084945084	protein_coding	NM_001198690
ASHGV40051688	0.084851	0.084851101	protein_coding	NM_002989
ASHGV40002606	-0.08467	0.084674666	protein_coding	ENST00000594872
ASHGV40008692	0.084673	0.084673266	protein_coding	NM_005125
ASHGV40035650	0.084657	0.084657093	protein_coding	NM_006090
ASHGV40044541	0.084602	0.084601597	protein_coding	NM_006567
ASHGV40033002	-0.08434	0.08434061	protein_coding	NM_005080
ASHGV40040982	-0.08433	0.084330743	protein_coding	NM_024577
ASHGV40011715	-0.0843	0.084303961	protein_coding	NM_006851
ASHGV40031135	0.084299	0.084299285	protein_coding	NM_080833
ASHGV40046976	0.084173	0.084173177	protein_coding	NM_014705
ASHGV40022240	-0.08416	0.08416059	protein_coding	NM_001113324
ASHGV40039943	-0.08413	0.084128167	protein_coding	NM_004394
ASHGV40012115	0.084102	0.084101914	protein_coding	NM_001080825
ASHGV40015507	-0.08402	0.08402357	protein_coding	NM_006291
ASHGV40052208	-0.08398	0.083978898	protein_coding	NM_138424

ASHGV40019317	0.08394	0.083939524	protein_coding	NM_006750
ASHGV40039373	0.083804	0.083803991	protein_coding	NM_018078
ASHGV40049122	-0.08379	0.083792485	protein_coding	NM_001001963
ASHGV40036359	-0.08377	0.083772208	protein_coding	NM_018394
ASHGV40012497	-0.08373	0.083734562	protein_coding	NM_172373
ASHGV40035574	0.083644	0.083643845	protein_coding	uc031ryf.1
ASHGV40026760	-0.08363	0.083630435	protein_coding	NM_152385
ASHGV40035484	-0.0836	0.083598759	protein_coding	NM_000757
ASHGV40057287	-0.08348	0.083481235	protein_coding	ENST00000601673
ASHGV40040305	-0.08345	0.083448691	protein_coding	NM_000524
ASHGV40025343	0.083336	0.083335871	protein_coding	NM_000453
ASHGV40039261	-0.08331	0.083310196	protein_coding	NM_006323
ASHGV40005612	-0.08313	0.083134024	protein_coding	NM_153034
ASHGV40037315	-0.08303	0.083029421	protein_coding	NM_001278141
ASHGV40008328	0.083027	0.083026732	protein_coding	NM_001142930
ASHGV40048668	0.082971	0.082971229	protein_coding	NM_018326
ASHGV40037937	-0.08296	0.082959855	protein_coding	NM_024873
ASHGV40024282	-0.0828	0.082800375	protein_coding	NM_000704
ASHGV40038931	-0.08279	0.082791639	protein_coding	NM_206919
ASHGV40005339	-0.08255	0.082551687	protein_coding	NM_005955
ASHGV40023489	0.082449	0.082449201	protein_coding	NM_002575
ASHGV40057234	-0.08234	0.082341051	protein_coding	NM_018662
ASHGV40003224	-0.08226	0.082255011	protein_coding	NM_001291085
ASHGV40052344	-0.08216	0.082155657	protein_coding	NM_197956
ASHGV40007817	0.082035	0.082034644	protein_coding	NM_005238
ASHGV40028734	-0.08202	0.082018106	protein_coding	NM_144736
ASHGV40011315	0.081971	0.081971268	protein_coding	NM_017842
ASHGV40012078	-0.08189	0.081887043	protein_coding	NM_001136534
ASHGV40010617	-0.08184	0.081835233	protein_coding	NM_016196
ASHGV40023302	-0.08173	0.08172811	protein_coding	NM_018255
ASHGV40015426	0.081723	0.081723234	protein_coding	NM_001127258
ASHGV40050114	0.08143	0.081430069	protein_coding	NM_000445
ASHGV40057747	-0.08119	0.081191275	protein_coding	NM_001256686
ASHGV40054952	-0.08118	0.081176612	protein_coding	NM_006044
ASHGV40006240	0.081086	0.081086436	protein_coding	NM_020940
ASHGV40050384	-0.08109	0.081085206	protein_coding	NM_016612
ASHGV40024297	-0.08096	0.080955586	protein_coding	NM_152658
ASHGV40017958	0.080905	0.080905152	protein_coding	NM_153029
ASHGV40041805	-0.08088	0.080882639	protein_coding	ENST00000313303
ASHGV40018868	-0.08088	0.080876459	protein_coding	NM_032368
ASHGV40054305	0.080642	0.080642235	protein_coding	NM_002814
ASHGV40010916	0.080588	0.080587635	protein_coding	NM_003213
ASHGV40035086	0.080507	0.080506965	protein_coding	ENST00000474463
ASHGV40019294	-0.08029	0.080292843	protein_coding	NM_006742
ASHGV40037843	0.080242	0.080242084	protein_coding	NM_020395
ASHGV40045597	0.079878	0.079878154	protein_coding	NM_001029858
ASHGV40044866	-0.07985	0.079853073	protein_coding	NM_205839

ASHGV40016110	0.079852	0.079852319	protein_coding	NM_031284
ASHGV40005929	-0.07982	0.079815198	protein_coding	NM_007078
ASHGV40035654	0.079785	0.079785426	protein_coding	NM_014296
ASHGV40042784	-0.07961	0.079612064	protein_coding	NM_001099408
ASHGV40037714	0.07959	0.079590089	protein_coding	NM_014933
ASHGV40053881	0.079546	0.079546106	protein_coding	NM_152787
ASHGV40050308	-0.0795	0.079503061	protein_coding	NM_001008539
ASHGV40048353	0.079289	0.079289289	protein_coding	NM_001753
ASHGV40019177	0.07923	0.079229709	protein_coding	NM_004401
ASHGV40050705	-0.07919	0.079190715	protein_coding	NM_001077619
ASHGV40000061	-0.07911	0.079114382	protein_coding	ENST00000327333
ASHGV40039755	-0.07905	0.079053635	protein_coding	ENST00000512874
ASHGV40047173	0.079039	0.079039194	protein_coding	NM_024033
ASHGV40046657	-0.07904	0.079037502	protein_coding	NM_001707
ASHGV40023211	-0.07889	0.078885075	protein_coding	NM_003831
ASHGV40022335	-0.07875	0.07874851	protein_coding	NM_017451
ASHGV40012316	-0.0787	0.078699313	protein_coding	NM_014572
ASHGV40028436	-0.07845	0.078450682	protein_coding	NM_014746
ASHGV40041484	-0.07831	0.078309981	protein_coding	NM_007118
ASHGV40034012	-0.0783	0.078300152	protein_coding	NM_018306
ASHGV40007630	-0.07828	0.078279868	protein_coding	NM_001562
ASHGV40052313	-0.07823	0.078227673	protein_coding	NM_005347
ASHGV40026627	0.078225	0.078224939	protein_coding	NM_001135673
ASHGV40013497	0.078014	0.078013512	protein_coding	ENST00000392948
ASHGV40035250	0.077991	0.077990559	protein_coding	NM_001146277
ASHGV40010696	-0.07795	0.077949035	protein_coding	ENST00000539163
ASHGV40014438	-0.07776	0.077762348	protein_coding	NM_145231
ASHGV40018950	0.077723	0.077723136	protein_coding	NM_014699
ASHGV40044768	-0.07769	0.07768791	protein_coding	NM_002393
ASHGV40019458	0.077651	0.077650914	protein_coding	NM_001305017
ASHGV40022847	-0.07762	0.077616012	protein_coding	NM_005570
ASHGV40029417	0.077462	0.077461556	protein_coding	NM_020909
ASHGV40022523	0.077397	0.077397381	protein_coding	NM_001128626
ASHGV40020079	-0.0774	0.077396014	protein_coding	NM_006923
ASHGV40021762	-0.07739	0.077388838	protein_coding	uc021txw.1
ASHGV40044643	0.077341	0.077341082	protein_coding	NM_006877
ASHGV40009642	0.077295	0.077294547	protein_coding	NM_024829
ASHGV40005992	-0.07711	0.077114987	protein_coding	NM_003972
ASHGV40055800	-0.07709	0.077091512	protein_coding	NM_001613
ASHGV40051366	-0.07708	0.077084469	protein_coding	ENST00000562505
ASHGV40011142	0.076971	0.076971426	protein_coding	NM_016072
ASHGV40016718	0.076971	0.076971019	protein_coding	NM_024956
ASHGV40005649	-0.0767	0.076698459	protein_coding	NM_001098512
ASHGV40050545	0.076659	0.076659029	protein_coding	NM_014142
ASHGV40043507	-0.07661	0.076611646	protein_coding	NM_015950
ASHGV40023829	0.076605	0.076604596	protein_coding	NM_016579
ASHGV40007685	-0.07651	0.076513938	protein_coding	NM_138971

ASHGV40047380	0.076493	0.076493437	protein_coding	NM_005515
ASHGV40031545	0.076479	0.076478863	protein_coding	NM_016436
ASHGV40023671	0.076423	0.076423395	protein_coding	NM_014963
ASHGV40054614	0.076388	0.076388022	protein_coding	NM_006140
ASHGV40024523	0.076362	0.076361793	protein_coding	NM_001983
ASHGV40042876	-0.07617	0.076174098	protein_coding	ENST00000599439
ASHGV40025245	0.076091	0.076091092	protein_coding	NM_001098622
ASHGV40025174	0.075948	0.075948371	protein_coding	NM_012218
ASHGV40019810	-0.07585	0.075854888	protein_coding	NM_004732
ASHGV40050354	0.075827	0.075826915	protein_coding	NM_003018
ASHGV40012314	-0.07579	0.075792766	protein_coding	NM_174928
ASHGV40016583	-0.07575	0.075754073	protein_coding	NM_014967
ASHGV40011264	0.075521	0.075521033	protein_coding	NM_019099
ASHGV40054278	-0.07545	0.07544512	protein_coding	NM_012286
ASHGV40015797	0.075415	0.075414727	protein_coding	NM_005926
ASHGV40032245	0.075317	0.075317297	protein_coding	NM_153682
ASHGV40003157	0.075255	0.075255173	protein_coding	NM_001199104
ASHGV40015556	-0.07524	0.075238001	protein_coding	NM_033438
ASHGV40045935	0.075165	0.075165235	protein_coding	NM_013349
ASHGV40047591	-0.07499	0.074990229	protein_coding	NM_003112
ASHGV40008837	-0.07478	0.074783371	protein_coding	NM_176796
ASHGV40002636	-0.07461	0.074608689	protein_coding	NM_025194
ASHGV40016843	-0.07454	0.074538449	protein_coding	NM_001198784
ASHGV40015076	0.074472	0.074471521	protein_coding	NM_153811
ASHGV40042581	-0.07436	0.074364336	protein_coding	NM_005754
ASHGV40033457	0.07421	0.074209687	protein_coding	NM_001128635
ASHGV40023730	-0.07389	0.073891671	protein_coding	NM_004886
ASHGV40048005	-0.07387	0.073865164	protein_coding	NM_014146
ASHGV40003161	0.073529	0.073528616	protein_coding	NM_001199535
ASHGV40009689	0.073505	0.073505409	protein_coding	NM_004982
ASHGV40045536	0.073504	0.073503891	protein_coding	NM_001033564
ASHGV40040444	0.073404	0.073403611	protein_coding	uc003kgg.1
ASHGV40031923	0.073387	0.073386894	protein_coding	NM_017896
ASHGV40031590	-0.07337	0.073371147	protein_coding	NM_080552
ASHGV40014047	0.073296	0.073295811	protein_coding	NM_004196
ASHGV40035986	-0.07325	0.073254154	protein_coding	ENST00000417220
ASHGV40056294	-0.07305	0.073046181	protein_coding	ENST00000561084
ASHGV40018077	-0.073	0.073003429	protein_coding	NM_018231
ASHGV40021773	0.07292	0.072920332	protein_coding	NM_019891
ASHGV40021704	0.072851	0.07285115	protein_coding	NM_000964
ASHGV40050765	0.072793	0.072792703	protein_coding	NM_144650
ASHGV40035735	0.072762	0.072761613	protein_coding	NM_001040432
ASHGV40021758	-0.07264	0.072644055	protein_coding	NM_009590
ASHGV40056555	0.072586	0.072586395	protein_coding	NM_013398
ASHGV40047314	0.07255	0.07255006	protein_coding	NM_007189
ASHGV40015141	0.072517	0.072516504	protein_coding	NM_020692
ASHGV40030165	0.072459	0.072458681	protein_coding	NM_002191

ASHGV40013359	-0.07246	0.072457104	protein_coding	NM_001011724
ASHGV40048288	0.072439	0.072438714	protein_coding	NM_021930
ASHGV40047768	-0.07214	0.072143144	protein_coding	NM_004760
ASHGV40003051	0.072115	0.072114895	protein_coding	NM_000442
ASHGV40037320	0.072075	0.072074593	protein_coding	NM_016955
ASHGV40054031	-0.07195	0.071949909	protein_coding	NM_004187
ASHGV40036905	-0.07184	0.071835193	protein_coding	NM_138345
ASHGV40034150	0.071786	0.071786471	protein_coding	NM_000404
ASHGV40022285	0.071386	0.071385723	protein_coding	ENST00000607453
ASHGV40033202	-0.07138	0.071384579	protein_coding	NM_016272
ASHGV40034432	0.07132	0.071320207	protein_coding	NM_021237
ASHGV40021559	-0.07132	0.071317595	protein_coding	NM_138328
ASHGV40022378	0.071308	0.071308251	protein_coding	NM_012336
ASHGV40007420	0.071234	0.071233899	protein_coding	NM_005040
ASHGV40038241	0.071138	0.071137598	protein_coding	uc021xuh.1
ASHGV40025540	-0.07112	0.071121477	protein_coding	NM_014686
ASHGV40020861	-0.07104	0.071039297	protein_coding	NM_020753
ASHGV40031587	-0.071	0.07099945	protein_coding	NM_057176
ASHGV40040880	-0.07024	0.070238724	protein_coding	NM_001161546
ASHGV40037174	0.070186	0.070185875	protein_coding	NM_017816
ASHGV40050546	-0.07018	0.070179609	protein_coding	NM_145004
ASHGV40045577	0.070145	0.070144527	protein_coding	NM_001010919
ASHGV40014881	-0.07014	0.070136988	protein_coding	NM_001164749
ASHGV40016915	0.070073	0.070072651	protein_coding	NM_171846
ASHGV40032046	0.070015	0.070014502	protein_coding	NM_003689
ASHGV40035047	-0.06991	0.069913259	protein_coding	NM_021105
ASHGV40030889	0.069866	0.069865797	protein_coding	NM_002999
ASHGV40022911	-0.06978	0.069784742	protein_coding	NM_173630
ASHGV40005760	0.069778	0.069777827	protein_coding	ENST00000420338
ASHGV40011398	-0.06962	0.069622247	protein_coding	NM_004302
ASHGV40028754	-0.0696	0.069599843	protein_coding	NM_024775
ASHGV40044716	0.069583	0.069582825	protein_coding	NM_017640
ASHGV40030816	0.069564	0.069564428	protein_coding	NM_152503
ASHGV40054956	-0.06951	0.069508945	protein_coding	NM_024859
ASHGV40008965	0.069453	0.06945302	protein_coding	NM_022918
ASHGV40048508	-0.06941	0.069408788	protein_coding	NM_003679
ASHGV40017404	0.069358	0.069357538	protein_coding	NM_012102
ASHGV40046656	0.069319	0.069318514	protein_coding	NM_032408
ASHGV40049095	-0.0693	0.069299127	protein_coding	NM_000637
ASHGV40037921	-0.06919	0.069192459	protein_coding	NM_007259
ASHGV40018058	-0.06915	0.069154674	protein_coding	NM_020313
ASHGV40042435	-0.06914	0.069142648	protein_coding	NM_018939
ASHGV40016405	-0.06897	0.068968325	protein_coding	NM_013330
ASHGV40011316	-0.06866	0.068661619	protein_coding	ENST00000599515
ASHGV40051413	-0.06864	0.068638388	protein_coding	NM_024531
ASHGV40011831	-0.06854	0.068538197	protein_coding	NM_003805
ASHGV40032684	-0.06844	0.068437733	protein_coding	NM_006057

ASHGV40048755	-0.06839	0.068387842	protein_coding	NM_138400
ASHGV40052044	0.068289	0.068289083	protein_coding	NM_000197
ASHGV40046802	0.068225	0.068225224	protein_coding	NM_014251
ASHGV40026882	-0.06816	0.068160964	protein_coding	NM_001024680
ASHGV40017175	0.068157	0.068156537	protein_coding	ENST00000553856
ASHGV40015185	-0.06815	0.068148394	protein_coding	NM_001024674
ASHGV40057463	0.068126	0.068125971	protein_coding	NM_024613
ASHGV40001692	0.067962	0.067962336	protein_coding	ENST00000519853
ASHGV40054962	0.067918	0.067918021	protein_coding	NM_033215
ASHGV40053501	-0.06785	0.067852836	protein_coding	NM_013355
ASHGV40024482	0.067837	0.067837119	protein_coding	NM_001033719
ASHGV40048647	-0.06779	0.067789224	protein_coding	ENST00000425642
ASHGV40030169	0.067784	0.067784425	protein_coding	NM_152499
ASHGV40035624	-0.06773	0.067734526	protein_coding	NM_014160
ASHGV40034577	0.067503	0.067502779	protein_coding	NM_002941
ASHGV40037203	0.067501	0.067501115	protein_coding	NM_025196
ASHGV40008650	0.067481	0.067480954	protein_coding	NM_006396
ASHGV40045032	-0.06746	0.067461989	protein_coding	NM_004828
ASHGV40039116	-0.06729	0.067291751	protein_coding	NM_001256455
ASHGV40020362	0.06728	0.067280188	protein_coding	NM_178126
ASHGV40054106	0.06714	0.067139748	protein_coding	NM_145119
ASHGV40051656	0.067117	0.067116604	protein_coding	NM_001195248
ASHGV40052943	0.067071	0.067071402	protein_coding	NM_015110
ASHGV40039366	0.06653	0.066530483	protein_coding	NM_015693
ASHGV40054632	-0.06646	0.066462711	protein_coding	NM_152707
ASHGV40012722	-0.06642	0.066422954	protein_coding	NM_203497
ASHGV40013723	-0.06637	0.066366671	protein_coding	NM_207440
ASHGV40013829	0.066294	0.066293741	protein_coding	NM_006109
ASHGV40052285	0.066286	0.066286287	protein_coding	NM_020924
ASHGV40027985	0.066153	0.066153186	protein_coding	NM_145280
ASHGV40044673	0.066138	0.066137726	protein_coding	NM_001546
ASHGV40001147	-0.06612	0.066119723	protein_coding	ENST00000453547
ASHGV40014461	0.06612	0.066119688	protein_coding	ENST00000340892
ASHGV40029759	-0.0661	0.066095452	protein_coding	NM_024843
ASHGV40043218	-0.06605	0.066045467	protein_coding	NM_017847
ASHGV40053235	0.065915	0.065915427	protein_coding	NM_031912
ASHGV40016715	-0.06589	0.065894342	protein_coding	NM_018097
ASHGV40031973	0.065781	0.065781033	protein_coding	NM_018257
ASHGV40044865	0.065721	0.065721479	protein_coding	NM_000595
ASHGV40043809	0.065523	0.065522512	protein_coding	NM_001024594
ASHGV40005714	-0.06546	0.065463045	protein_coding	NM_001001330
ASHGV40035595	-0.06545	0.065449692	protein_coding	NM_153480
ASHGV40060806	0.065314	0.065314254	protein_coding	uc001iaf.1
ASHGV40057754	0.06529	0.065289984	protein_coding	NM_001113528
ASHGV40016875	0.065233	0.065233205	protein_coding	NM_001270530
ASHGV40053936	-0.06517	0.065173151	protein_coding	NM_003688
ASHGV40006123	0.064991	0.064991023	protein_coding	NM_004741

ASHGV40048887	-0.06484	0.064837067	protein_coding	ENST00000304501
ASHGV40007083	0.064701	0.064701427	protein_coding	NM_198334
ASHGV40047790	0.064575	0.064574846	protein_coding	NM_005856
ASHGV40057179	0.064575	0.064574541	protein_coding	NM_003523
ASHGV40023639	-0.06449	0.064486672	protein_coding	NM_014913
ASHGV40022213	-0.06445	0.06444522	protein_coding	NM_024844
ASHGV40025194	0.064385	0.064384816	protein_coding	NM_002743
ASHGV40052366	-0.06428	0.064276907	protein_coding	NM_016390
ASHGV40010756	-0.06423	0.064227256	protein_coding	NM_006113
ASHGV40048633	-0.06415	0.064150432	protein_coding	uc003wfu.3
ASHGV40006398	0.064102	0.064102094	protein_coding	NM_006541
ASHGV40040256	-0.06405	0.064050648	protein_coding	NM_001258286
ASHGV40026051	-0.06395	0.063951914	protein_coding	NM_001278398
ASHGV40033663	-0.06377	0.063769721	protein_coding	NM_005488
ASHGV40005269	0.063536	0.063535985	protein_coding	NM_001303030
ASHGV40013059	0.063521	0.063521054	protein_coding	NM_138284
ASHGV40051121	-0.06344	0.063437263	protein_coding	NM_001025357
ASHGV40008782	-0.0634	0.063404061	protein_coding	NM_003626
ASHGV40013693	0.063367	0.063366735	protein_coding	NM_198217
ASHGV40019600	-0.06316	0.063155318	protein_coding	NM_001127214
ASHGV40038081	0.063018	0.063018126	protein_coding	NM_025150
ASHGV40057397	-0.06281	0.062811502	protein_coding	NM_001164458
ASHGV40054913	-0.06281	0.062808633	protein_coding	NM_005676
ASHGV40024778	0.062798	0.062797515	protein_coding	NM_024318
ASHGV40024307	0.062783	0.06278291	protein_coding	NM_032838
ASHGV40002225	0.062673	0.062673045	protein_coding	NM_005953
ASHGV40001771	-0.06264	0.062640757	protein_coding	NM_006476
ASHGV40025164	0.062297	0.062296606	protein_coding	NM_002566
ASHGV40038216	0.062043	0.062042858	protein_coding	NM_001166373
ASHGV40057240	-0.06179	0.061789881	protein_coding	NM_006416
ASHGV40047736	0.061771	0.061771104	protein_coding	NM_007252
ASHGV40023120	-0.06172	0.061719608	protein_coding	NM_020648
ASHGV40006484	-0.06172	0.061717076	protein_coding	NM_024698
ASHGV40019761	-0.06171	0.061710594	protein_coding	NM_031220
ASHGV40031485	0.061662	0.061662107	protein_coding	NM_015352
ASHGV40052836	0.061616	0.061615606	protein_coding	NM_014907
ASHGV40025917	0.061533	0.061533416	protein_coding	NM_198318
ASHGV40009345	0.061347	0.061346629	protein_coding	NM_001254757
ASHGV40033703	0.061336	0.061336466	protein_coding	NM_013365
ASHGV40020863	0.061323	0.061323298	protein_coding	NM_004259
ASHGV40015458	0.061268	0.061268126	protein_coding	ENST00000599197
ASHGV40008358	-0.06123	0.061231033	protein_coding	NM_018389
ASHGV40033787	-0.06117	0.06116971	protein_coding	NM_004599
ASHGV40009937	-0.06114	0.061141213	protein_coding	NM_181708
ASHGV40023789	-0.06112	0.061118945	protein_coding	NM_173637
ASHGV40045379	-0.06109	0.061093437	protein_coding	NM_012115
ASHGV40003193	0.060971	0.060970876	protein_coding	NM_001244584

ASHGV40003166	-0.06085	0.060850498	protein_coding	NM_001199782
ASHGV40011406	0.060837	0.060836575	protein_coding	NM_003051
ASHGV40050773	0.060679	0.060678952	protein_coding	NM_024790
ASHGV40036426	0.060591	0.060590686	protein_coding	NM_005513
ASHGV40010940	-0.06059	0.06059053	protein_coding	NM_002235
ASHGV40056712	-0.06055	0.06054804	protein_coding	NM_194247
ASHGV40018898	0.060497	0.060497163	protein_coding	uc002dtz.1
ASHGV40035761	-0.06032	0.060316724	protein_coding	NM_174896
ASHGV40014992	-0.06025	0.06025233	protein_coding	NM_000953
ASHGV40009513	0.060094	0.060094368	protein_coding	NM_001127582
ASHGV40031149	0.060001	0.060001364	protein_coding	NM_033081
ASHGV40051325	-0.05983	0.059833313	protein_coding	NM_014957
ASHGV40029962	-0.05983	0.059829412	protein_coding	NM_153689
ASHGV40055274	-0.05968	0.059680828	protein_coding	NM_152586
ASHGV40053399	-0.05957	0.059574565	protein_coding	NM_004962
ASHGV40013752	0.059503	0.059502942	protein_coding	NM_182614
ASHGV40054447	0.059473	0.059473284	protein_coding	NM_001166599
ASHGV40003111	-0.05941	0.059414164	protein_coding	NM_001098808
ASHGV40013835	0.059384	0.059384181	protein_coding	NM_001164816
ASHGV40006000	0.059168	0.059167985	protein_coding	NM_019053
ASHGV40006295	-0.05913	0.05913123	protein_coding	ENST00000369071
ASHGV40046980	-0.05911	0.059114685	protein_coding	NM_022484
ASHGV40051857	-0.05909	0.059091861	protein_coding	NM_152420
ASHGV40038370	-0.05899	0.05899418	protein_coding	NM_152775
ASHGV40013749	0.058983	0.058982761	protein_coding	NM_007111
ASHGV40001776	-0.05893	0.058934139	protein_coding	NM_002696
ASHGV40052365	0.058863	0.05886285	protein_coding	NM_006336
ASHGV40001379	-0.05881	0.058814954	protein_coding	NM_001242713
ASHGV40034626	0.058813	0.058813472	protein_coding	NM_019083
ASHGV40029951	0.058682	0.058681549	protein_coding	NM_025147
ASHGV40051333	0.058662	0.058661884	protein_coding	ENST00000427937
ASHGV40023130	0.058503	0.058502606	protein_coding	NM_001098529
ASHGV40034378	0.058434	0.058434488	protein_coding	NM_170714
ASHGV40023960	-0.05834	0.058341056	protein_coding	NM_004461
ASHGV40028435	-0.05834	0.058337656	protein_coding	NM_080657
ASHGV40025998	0.058236	0.058236017	protein_coding	NM_182623
ASHGV40018370	0.05818	0.058180356	protein_coding	NM_006067
ASHGV40048857	-0.05809	0.058089951	protein_coding	ENST00000594215
ASHGV40048877	-0.05793	0.057930706	protein_coding	uc022ars.2
ASHGV40043517	0.057642	0.057642426	protein_coding	NM_015388
ASHGV40029528	-0.05761	0.057606794	protein_coding	NM_013310
ASHGV40006328	0.057356	0.057355643	protein_coding	NM_004725
ASHGV40034409	0.057344	0.057344093	protein_coding	NM_020163
ASHGV40008083	-0.05729	0.057285785	protein_coding	NM_006391
ASHGV40044837	0.057147	0.057147373	protein_coding	NM_014046
ASHGV40007521	0.057135	0.057135435	protein_coding	NM_004621
ASHGV40046454	0.057024	0.057023842	protein_coding	NM_012412

ASHGV40042394	0.05701	0.057010454	protein_coding	NM_032289
ASHGV40041663	0.056968	0.056968062	protein_coding	NM_012176
ASHGV40007252	0.056939	0.056938928	protein_coding	NM_001876
ASHGV40011942	-0.05692	0.056923793	protein_coding	NM_001018072
ASHGV40026818	-0.05681	0.056814688	protein_coding	NM_032180
ASHGV40032860	0.056684	0.056683624	protein_coding	NM_022727
ASHGV40017688	-0.05651	0.056514391	protein_coding	NM_015092
ASHGV40050309	-0.05618	0.056184608	protein_coding	NM_006207
ASHGV40015522	0.056123	0.05612323	protein_coding	NM_182923
ASHGV40009298	0.056109	0.056108952	protein_coding	NM_014622
ASHGV40014244	0.056069	0.056068992	protein_coding	NM_016468
ASHGV40019927	0.056004	0.056004415	protein_coding	ENST00000455584
ASHGV40044019	0.055999	0.055999407	protein_coding	NM_001042475
ASHGV40024870	0.055991	0.055990792	protein_coding	ENST00000596831
ASHGV40015290	0.055817	0.05581681	protein_coding	NM_024824
ASHGV40055060	-0.05577	0.055770103	protein_coding	NM_001551
ASHGV40047962	-0.05551	0.055513213	protein_coding	NM_017994
ASHGV40049847	0.055511	0.055511268	protein_coding	NM_014078
ASHGV40007480	-0.0555	0.055495972	protein_coding	NM_005591
ASHGV40041714	0.055441	0.055441221	protein_coding	NM_024615
ASHGV40017784	0.055372	0.055371561	protein_coding	NM_001109763
ASHGV40057786	-0.05532	0.055316801	protein_coding	NM_005472
ASHGV40053786	-0.05527	0.055265315	protein_coding	NM_017856
ASHGV40006982	0.055263	0.055263126	protein_coding	NM_005161
ASHGV40041233	0.055257	0.055256548	protein_coding	NM_024872
ASHGV40015794	0.055235	0.055234791	protein_coding	NM_172095
ASHGV40025083	-0.05505	0.055048591	protein_coding	NM_001288962
ASHGV40008527	0.05502	0.055019797	protein_coding	NM_001127392
ASHGV40053537	-0.05478	0.054776204	protein_coding	NM_001291815
ASHGV40013814	0.054749	0.054748998	protein_coding	uc010tmc.2
ASHGV40031562	-0.05474	0.054744265	protein_coding	NM_022077
ASHGV40026046	0.054629	0.054628908	protein_coding	NM_002288
ASHGV40053783	0.054545	0.054545464	protein_coding	NM_014563
ASHGV40042668	-0.05442	0.054416872	protein_coding	NM_004060
ASHGV40035345	0.054399	0.054399485	protein_coding	NM_005688
ASHGV40007090	-0.0544	0.054399053	protein_coding	ENST00000431002
ASHGV40056862	0.054307	0.054307307	protein_coding	NM_001675
ASHGV40045376	-0.05389	0.053887297	protein_coding	NM_001242809
ASHGV40046147	0.053884	0.053883634	protein_coding	NM_001037763
ASHGV40007326	0.05387	0.053869548	protein_coding	NM_006645
ASHGV40011409	-0.05385	0.053854125	protein_coding	NM_002284
ASHGV40051731	-0.05382	0.053818634	protein_coding	NM_001001790
ASHGV40015923	-0.05379	0.053789455	protein_coding	NM_001297713
ASHGV40048230	-0.05371	0.05370872	protein_coding	NM_006076
ASHGV40037262	-0.05364	0.053640438	protein_coding	NM_031950
ASHGV40040418	-0.05358	0.053582591	protein_coding	NM_015331
ASHGV40054077	-0.05354	0.053543496	protein_coding	NM_007156

ASHGV40017660	-0.05341	0.053410846	protein_coding	NM_173474
ASHGV40050184	-0.05336	0.053364669	protein_coding	NM_018941
ASHGV40039108	0.053166	0.053166113	protein_coding	NM_001201
ASHGV40008149	-0.05302	0.053017375	protein_coding	NM_005013
ASHGV40031038	0.053014	0.053014232	protein_coding	NM_001262
ASHGV40023855	0.052994	0.052994364	protein_coding	NM_006631
ASHGV40011936	0.052962	0.052962083	protein_coding	NM_213594
ASHGV40052816	-0.05281	0.052808739	protein_coding	NM_005893
ASHGV40046564	0.052771	0.05277134	protein_coding	NM_006213
ASHGV40010434	0.052751	0.052750757	protein_coding	NM_002595
ASHGV40018224	-0.05262	0.052617394	protein_coding	NM_015020
ASHGV40024755	-0.05259	0.052589789	protein_coding	NM_173857
ASHGV40037582	-0.0525	0.052496844	protein_coding	NM_018227
ASHGV40040145	-0.05248	0.052479193	protein_coding	ENST00000537147
ASHGV40056612	0.052473	0.052472557	protein_coding	NM_024584
ASHGV40042299	-0.05245	0.052454332	protein_coding	NM_003059
ASHGV40029684	-0.05244	0.05244112	protein_coding	NM_173355
ASHGV40026460	-0.05241	0.052405626	protein_coding	ENST00000430988
ASHGV40016684	0.052193	0.052192752	protein_coding	NM_001077268
ASHGV40006712	-0.05216	0.052162912	protein_coding	NM_138421
ASHGV40044660	-0.05209	0.052088298	protein_coding	NM_153042
ASHGV40049105	-0.05201	0.052005394	protein_coding	NM_001001957
ASHGV40011514	0.051953	0.051953087	protein_coding	NM_002868
ASHGV40011925	-0.0519	0.051898739	protein_coding	NM_152318
ASHGV40017209	0.051643	0.051643214	protein_coding	NM_014630
ASHGV40042303	-0.05162	0.051617052	protein_coding	uc010jds.2
ASHGV40029740	-0.05146	0.051463176	protein_coding	NM_203463
ASHGV40054135	0.051212	0.051211765	protein_coding	NM_002637
ASHGV40016212	-0.05107	0.051070806	protein_coding	NM_001199760
ASHGV40010030	0.050958	0.050957673	protein_coding	NM_001130967
ASHGV40028350	-0.05073	0.050728007	protein_coding	NM_175852
ASHGV40038123	-0.0507	0.05070092	protein_coding	NM_006726
ASHGV40012121	0.050663	0.050662961	protein_coding	NM_002813
ASHGV40030652	0.050662	0.050662316	protein_coding	NM_199044
ASHGV40038905	0.050446	0.050446294	protein_coding	NM_000222
ASHGV40006130	0.05042	0.050419839	protein_coding	NM_024789
ASHGV40022803	-0.05034	0.050342251	protein_coding	NM_139171
ASHGV40021238	-0.05027	0.050272281	protein_coding	NM_001080424
ASHGV40043482	-0.0502	0.050202423	protein_coding	NM_006653
ASHGV40042985	-0.0501	0.050104606	protein_coding	NM_201280
ASHGV40010915	0.050018	0.050017553	protein_coding	NM_003324
ASHGV40010155	-0.04996	0.049962028	protein_coding	NM_173812
ASHGV40023112	-0.04996	0.049958447	protein_coding	NM_015210
ASHGV40013179	-0.04982	0.049818069	protein_coding	NM_023037
ASHGV40033606	0.049791	0.049791202	protein_coding	ENST00000439023
ASHGV40005487	0.049726	0.049726094	protein_coding	NM_012333
ASHGV40035856	-0.04969	0.049688493	protein_coding	NM_207404

ASHGV40011969	-0.04962	0.04962387	protein_coding	NM_001093
ASHGV40030076	0.049587	0.049587432	protein_coding	NM_006279
ASHGV40049561	-0.04945	0.049454267	protein_coding	NM_001099670
ASHGV40049438	0.04945	0.049450488	protein_coding	NM_006540
ASHGV40026948	0.049413	0.049412778	protein_coding	NM_018221
ASHGV40043324	-0.04936	0.049362074	protein_coding	NM_002123
ASHGV40030776	0.049302	0.049302285	protein_coding	NM_014071
ASHGV40051046	0.04916	0.049159658	protein_coding	NM_138455
ASHGV40045013	0.049118	0.049118174	protein_coding	NM_001201427
ASHGV40005995	0.049026	0.049025897	protein_coding	NM_017824
ASHGV40020062	-0.04891	0.048913865	protein_coding	NM_001267775
ASHGV40020893	-0.04886	0.048855634	protein_coding	NM_032134
ASHGV40017566	0.048708	0.048707923	protein_coding	NM_133450
ASHGV40025518	-0.0485	0.048501093	protein_coding	NM_004708
ASHGV40033619	0.048325	0.048325143	protein_coding	NM_005569
ASHGV40005610	0.048208	0.048207832	protein_coding	NM_032526
ASHGV40054771	0.048154	0.048154108	protein_coding	NM_001136234
ASHGV40031862	0.048149	0.048148593	protein_coding	NM_198976
ASHGV40007113	0.048123	0.048122552	protein_coding	NM_015459
ASHGV40018436	0.047936	0.047936032	protein_coding	NM_012134
ASHGV40055389	-0.0479	0.047897763	protein_coding	NM_001004486
ASHGV40014219	-0.04789	0.047885659	protein_coding	NM_004569
ASHGV40050126	-0.04777	0.047769337	protein_coding	NM_012079
ASHGV40048534	0.047753	0.047753151	protein_coding	NM_001085429
ASHGV40034788	0.047708	0.047708023	protein_coding	NM_003778
ASHGV40050876	0.047676	0.047675945	protein_coding	NM_152284
ASHGV40028203	-0.04757	0.047572582	protein_coding	NM_024409
ASHGV40045325	0.047544	0.047544037	protein_coding	NM_006670
ASHGV40020427	-0.0474	0.047401336	protein_coding	NM_024819
ASHGV40023493	0.0474	0.047400178	protein_coding	NM_002640
ASHGV40017417	-0.04737	0.047368576	protein_coding	NM_032366
ASHGV40030591	-0.04714	0.047141429	protein_coding	NM_001013615
ASHGV40055204	-0.04712	0.047122603	protein_coding	NM_021637
ASHGV40024816	0.046954	0.046953783	protein_coding	NM_001282011
ASHGV40041222	0.046932	0.046932427	protein_coding	NM_130781
ASHGV40040285	0.046521	0.04652123	protein_coding	NM_001104631
ASHGV40026086	0.046484	0.046483858	protein_coding	NM_016202
ASHGV40053443	0.046467	0.046466743	protein_coding	NM_005833
ASHGV40026958	-0.04643	0.046429488	protein_coding	NM_032673
ASHGV40021314	0.046129	0.046128637	protein_coding	NM_014859
ASHGV40032272	-0.04603	0.0460319	protein_coding	NM_018963
ASHGV40056870	0.045839	0.045839231	protein_coding	NM_002642
ASHGV40052099	0.045815	0.045815188	protein_coding	NM_019051
ASHGV40016342	0.045794	0.045793781	protein_coding	ENST00000560096
ASHGV40026638	-0.04571	0.045714172	protein_coding	ENST00000601251
ASHGV40008617	0.045656	0.045656172	protein_coding	NM_013306
ASHGV40041059	-0.04565	0.045653198	protein_coding	NM_001001343

ASHGV40005966	0.045652	0.045651785	protein_coding	NM_012420
ASHGV40041140	0.045215	0.04521474	protein_coding	NM_002585
ASHGV40000988	0.045161	0.045161112	protein_coding	NM_012071
ASHGV40033132	0.045022	0.045022419	protein_coding	NM_052906
ASHGV40019619	-0.0448	0.044798665	protein_coding	NM_052988
ASHGV40024016	0.044682	0.044682246	protein_coding	NM_022904
ASHGV40023967	0.044611	0.044611473	protein_coding	NM_003765
ASHGV40030161	-0.04453	0.044525313	protein_coding	NM_013335
ASHGV40033578	0.044475	0.044475433	protein_coding	NM_001284263
ASHGV40048246	0.044407	0.044406821	protein_coding	NM_001164462
ASHGV40036121	0.044354	0.044354425	protein_coding	NM_018971
ASHGV40022539	0.044242	0.044241529	protein_coding	NM_032752
ASHGV40049396	-0.04417	0.044174805	protein_coding	NM_004820
ASHGV40022171	-0.04417	0.044174357	protein_coding	NM_001288770
ASHGV40051686	-0.04412	0.044116577	protein_coding	ENST00000416454
ASHGV40052005	0.04411	0.044110201	protein_coding	NM_031486
ASHGV40001842	0.044068	0.044068146	protein_coding	ENST00000529564
ASHGV40056180	0.044059	0.044059427	protein_coding	NM_152268
ASHGV40005176	-0.04381	0.04381246	protein_coding	ENST00000441152
ASHGV40043042	0.043645	0.043645362	protein_coding	ENST00000600057
ASHGV40054383	0.0436	0.043600213	protein_coding	NM_001081550
ASHGV40044952	0.043549	0.04354877	protein_coding	NM_003093
ASHGV40018254	0.043506	0.043505997	protein_coding	NM_030581
ASHGV40032741	0.043369	0.043369237	protein_coding	NM_000383
ASHGV40025297	0.043264	0.043264228	protein_coding	NM_005370
ASHGV40026610	-0.04326	0.043260685	protein_coding	NM_030907
ASHGV40045838	0.043166	0.043165653	protein_coding	NM_015440
ASHGV40050615	0.043146	0.043145749	protein_coding	ENST00000429930
ASHGV40000279	0.043139	0.043138909	protein_coding	uc001pdx.1
ASHGV40027393	-0.04312	0.043116126	protein_coding	NM_001161403
ASHGV40027284	0.042998	0.042998253	protein_coding	NM_003466
ASHGV40043486	0.04296	0.042959841	protein_coding	NM_001760
ASHGV40049373	-0.04288	0.042879001	protein_coding	NM_004318
ASHGV40012287	-0.04286	0.042858941	protein_coding	NM_015394
ASHGV40002144	-0.04283	0.042830141	protein_coding	NM_001199864
ASHGV40037623	-0.04269	0.042693822	protein_coding	NM_014243
ASHGV40025962	0.042631	0.042630751	protein_coding	NM_001772
ASHGV40008840	0.042448	0.042447983	protein_coding	NM_032871
ASHGV40012830	-0.04226	0.04226462	protein_coding	NM_001304430
ASHGV40035955	0.042251	0.042251005	protein_coding	NM_005777
ASHGV40055992	0.041986	0.041985711	protein_coding	NM_025198
ASHGV40024823	-0.04193	0.041930189	protein_coding	NM_001145176
ASHGV40035106	0.041923	0.041923103	protein_coding	NM_018061
ASHGV40002708	-0.04188	0.041882805	protein_coding	NM_001204425
ASHGV40031523	-0.04175	0.041753706	protein_coding	NM_016732
ASHGV40001684	-0.04173	0.041733962	protein_coding	ENST00000519718
ASHGV40009936	-0.04161	0.041612105	protein_coding	NM_001037806

ASHGV40015053	0.041179	0.041178856	protein_coding	NM_001244190
ASHGV40021911	0.041167	0.041167072	protein_coding	NM_138281
ASHGV40012501	-0.0411	0.041096811	protein_coding	NM_004294
ASHGV40046491	-0.04107	0.041066032	protein_coding	NM_004507
ASHGV40045671	0.041059	0.041058638	protein_coding	NM_025228
ASHGV40032600	0.041009	0.041008551	protein_coding	NM_138983
ASHGV40036885	-0.04099	0.040989793	protein_coding	NM_014616
ASHGV40026445	0.040968	0.040968113	protein_coding	NM_152376
ASHGV40010402	-0.04088	0.040875655	protein_coding	NM_144620
ASHGV40032836	0.040872	0.0408715	protein_coding	NM_022719
ASHGV40030127	0.040559	0.040559443	protein_coding	NM_000578
ASHGV40008190	-0.04052	0.040516759	protein_coding	NM_020346
ASHGV40003251	-0.04048	0.04047506	protein_coding	NM_002984
ASHGV40008648	-0.0404	0.040399794	protein_coding	NM_198714
ASHGV40005736	0.04037	0.040370158	protein_coding	NM_018237
ASHGV40025919	0.040228	0.040227659	protein_coding	NM_014424
ASHGV40010722	0.039915	0.039914588	protein_coding	NM_177551
ASHGV40054434	-0.03989	0.039891027	protein_coding	NM_021183
ASHGV40002349	0.03977	0.039769605	protein_coding	NM_005951
ASHGV40025481	0.039755	0.039754572	protein_coding	NM_001238
ASHGV40007714	0.039747	0.03974689	protein_coding	NM_001028
ASHGV40013251	0.039705	0.039705082	protein_coding	NM_178009
ASHGV40054336	0.039675	0.03967465	protein_coding	NM_000640
ASHGV40038517	0.039635	0.039635073	protein_coding	NM_014392
ASHGV40023891	-0.03954	0.039543016	protein_coding	NM_023008
ASHGV40009419	0.039537	0.039537132	protein_coding	NM_016615
ASHGV40055316	-0.03939	0.039389688	protein_coding	NM_144658
ASHGV40016258	0.039365	0.039364673	protein_coding	NM_144597
ASHGV40023387	-0.03931	0.039308191	protein_coding	NM_005359
ASHGV40020259	-0.03926	0.03926136	protein_coding	NM_004774
ASHGV40038443	-0.03923	0.039233367	protein_coding	NM_002477
ASHGV40027088	0.039154	0.039154014	protein_coding	NM_005839
ASHGV40027837	-0.03893	0.038928382	protein_coding	ENST00000596102
ASHGV40020710	-0.03891	0.03890744	protein_coding	NM_007215
ASHGV40014067	-0.03884	0.038835929	protein_coding	NM_007361
ASHGV40047220	0.03883	0.038829793	protein_coding	NM_004333
ASHGV40003363	-0.03877	0.038765923	protein_coding	NM_203412
ASHGV40025679	0.038707	0.038707276	protein_coding	NM_003407
ASHGV40046982	0.038655	0.038654943	protein_coding	NM_152556
ASHGV40016477	-0.0385	0.038497447	protein_coding	NM_000655
ASHGV40050785	0.038488	0.038488309	protein_coding	NM_001033855
ASHGV40041292	-0.03837	0.038372535	protein_coding	NM_001172638
ASHGV40036754	-0.03837	0.038366078	protein_coding	NM_015938
ASHGV40033594	0.03836	0.038359888	protein_coding	NM_000268
ASHGV40051118	0.038329	0.038328896	protein_coding	NM_032334
ASHGV40028616	-0.0382	0.038203227	protein_coding	NM_014971
ASHGV40016452	-0.03811	0.038114582	protein_coding	NM_001040616

ASHGV40039653	-0.03793	0.037926317	protein_coding	NM_001040157
ASHGV40018210	-0.03785	0.03784757	protein_coding	NM_018052
ASHGV40009952	0.037837	0.037837187	protein_coding	NM_001031628
ASHGV40034850	-0.03783	0.03782895	protein_coding	NM_014079
ASHGV40035562	-0.03781	0.037805632	protein_coding	NM_032236
ASHGV40050764	0.037805	0.037805325	protein_coding	NM_015169
ASHGV40023681	0.037761	0.03776067	protein_coding	NM_203304
ASHGV40024516	0.03771	0.037710424	protein_coding	NM_001270891
ASHGV40035846	0.037647	0.037647215	protein_coding	NM_016305
ASHGV40020695	0.037557	0.037556551	protein_coding	NM_020991
ASHGV40011219	0.037502	0.037501744	protein_coding	NM_173802
ASHGV40030050	-0.03746	0.037463016	protein_coding	NM_014663
ASHGV40009853	0.037455	0.037454678	protein_coding	NM_031292
ASHGV40005680	0.037405	0.037404625	protein_coding	NM_001080512
ASHGV40002533	0.037287	0.037286765	protein_coding	NM_033520
ASHGV40055152	0.037268	0.03726796	protein_coding	NM_053281
ASHGV40040379	-0.03724	0.037237215	protein_coding	NM_023039
ASHGV40012176	0.037235	0.037235363	protein_coding	NM_080626
ASHGV40035494	0.037192	0.037192189	protein_coding	NM_015562
ASHGV40043808	0.036978	0.036977593	protein_coding	NM_080743
ASHGV40053902	-0.03693	0.036932944	protein_coding	NM_006307
ASHGV40002597	0.036915	0.036914728	protein_coding	ENST00000594486
ASHGV40011475	-0.03684	0.036838592	protein_coding	NM_016057
ASHGV40002036	0.036805	0.036804532	protein_coding	NM_032704
ASHGV40052802	0.03678	0.036780133	protein_coding	NM_001080496
ASHGV40053779	-0.03641	0.036411064	protein_coding	NM_001135995
ASHGV40046929	0.036403	0.036403392	protein_coding	ENST00000460135
ASHGV40037416	0.036342	0.036342414	protein_coding	NM_003263
ASHGV40026088	0.036305	0.036304856	protein_coding	NM_013301
ASHGV40031169	0.036299	0.03629938	protein_coding	NM_003224
ASHGV40007207	-0.03628	0.036284083	protein_coding	NM_032449
ASHGV40014462	-0.0362	0.036203271	protein_coding	NM_006329
ASHGV40048091	-0.03602	0.036015211	protein_coding	NM_021151
ASHGV40026514	0.036013	0.036013292	protein_coding	NM_001521
ASHGV40038258	-0.03575	0.035754462	protein_coding	NM_016228
ASHGV40018859	-0.03521	0.035206125	protein_coding	NM_024773
ASHGV40033732	-0.03517	0.035165459	protein_coding	NM_020243
ASHGV40022004	-0.03507	0.035072104	protein_coding	NM_138962
ASHGV40019330	0.034982	0.034981922	protein_coding	NM_007242
ASHGV40010808	-0.03497	0.034974149	protein_coding	NM_145648
ASHGV40019684	0.034956	0.034955666	protein_coding	NM_024086
ASHGV40027976	-0.0349	0.034897506	protein_coding	NM_001039845
ASHGV40026663	-0.03488	0.034880789	protein_coding	NM_148962
ASHGV40048255	0.034786	0.034785789	protein_coding	NM_006349
ASHGV40042943	0.034772	0.034772107	protein_coding	NM_206836
ASHGV40047795	-0.03474	0.034736936	protein_coding	NM_021116
ASHGV40023702	0.034618	0.034618071	protein_coding	NM_198532

ASHGV40015337	-0.03452	0.034524359	protein_coding	NM_015676
ASHGV40044474	-0.03451	0.034507979	protein_coding	NM_020185
ASHGV40012151	-0.03444	0.03443868	protein_coding	NM_001516
ASHGV40035522	0.034436	0.034436179	protein_coding	NM_032263
ASHGV40044090	0.034396	0.034395825	protein_coding	NM_003569
ASHGV40044915	-0.0344	0.03439535	protein_coding	NM_006979
ASHGV40031901	-0.03439	0.034393034	protein_coding	NM_015666
ASHGV40019733	-0.03431	0.034310387	protein_coding	uc002fyz.2
ASHGV40016747	0.034215	0.034215142	protein_coding	NM_017434
ASHGV40049745	0.034202	0.034202101	protein_coding	NM_001385
ASHGV40014774	0.034154	0.034154399	protein_coding	NM_014949
ASHGV40029758	0.034114	0.034114337	protein_coding	NM_025000
ASHGV40030794	-0.03391	0.033914339	protein_coding	NM_006047
ASHGV40020632	-0.03387	0.033873975	protein_coding	NM_004687
ASHGV40054593	-0.03382	0.033815271	protein_coding	NM_022129
ASHGV40053621	0.033803	0.033803208	protein_coding	NM_002571
ASHGV40027771	-0.03374	0.033743867	protein_coding	NM_152660
ASHGV40014564	-0.03374	0.033741937	protein_coding	NM_206918
ASHGV40042067	0.033723	0.033722806	protein_coding	NM_198150
ASHGV40014805	-0.03355	0.033546278	protein_coding	NM_006032
ASHGV40007495	0.033528	0.033528206	protein_coding	NM_144664
ASHGV40011825	-0.03346	0.033461076	protein_coding	NM_001779
ASHGV40008173	-0.03344	0.033437367	protein_coding	NM_019028
ASHGV40040879	-0.03337	0.033369293	protein_coding	NM_016459
ASHGV40006441	0.033339	0.033339373	protein_coding	NM_145806
ASHGV40028218	0.033263	0.033262961	protein_coding	NM_018218
ASHGV40030143	0.033259	0.033259471	protein_coding	NM_003936
ASHGV40044523	-0.03313	0.033127878	protein_coding	ENST00000356722
ASHGV40032975	-0.033	0.032999104	protein_coding	NM_003595
ASHGV40029953	-0.03296	0.03296123	protein_coding	NM_015387
ASHGV40030780	0.032885	0.032884644	protein_coding	NM_015638
ASHGV40044213	0.032873	0.032873047	protein_coding	NM_001042683
ASHGV40025802	0.032863	0.032863294	protein_coding	NM_019121
ASHGV40055544	0.032691	0.032691345	protein_coding	NM_001080485
ASHGV40055079	0.032659	0.032658903	protein_coding	NM_181672
ASHGV40034669	0.032653	0.032653411	protein_coding	NM_020654
ASHGV40006723	0.032471	0.032470633	protein_coding	NM_001040697
ASHGV40019198	-0.03246	0.03246236	protein_coding	NM_199456
ASHGV40005578	0.032402	0.03240226	protein_coding	NM_001258000
ASHGV40014748	-0.03227	0.032271531	protein_coding	NM_014828
ASHGV40054941	-0.03221	0.032213139	protein_coding	NM_022153
ASHGV40010612	-0.0322	0.032201456	protein_coding	NM_006843
ASHGV40019744	-0.0321	0.032098788	protein_coding	NM_032530
ASHGV40011163	-0.03196	0.031958688	protein_coding	NM_006152
ASHGV40006541	-0.03192	0.031921525	protein_coding	NM_001665
ASHGV40013481	-0.03182	0.031817373	protein_coding	NM_006493
ASHGV40010283	0.031801	0.031801247	protein_coding	NM_004537

ASHGV40031435	0.031767	0.031767225	protein_coding	NM_024893
ASHGV40005882	0.031761	0.031761218	protein_coding	NM_001012973
ASHGV40035975	0.031691	0.031691486	protein_coding	ENST00000528157
ASHGV40041979	-0.03155	0.031554247	protein_coding	NM_032280
ASHGV40051185	0.031551	0.031550944	protein_coding	NM_007218
ASHGV40009188	-0.03148	0.031481516	protein_coding	NM_207343
ASHGV40000181	0.031471	0.031471073	protein_coding	ENST00000380604
ASHGV40055882	0.031422	0.031422033	protein_coding	NM_018058
ASHGV40027037	0.031382	0.031381784	protein_coding	NM_022912
ASHGV40046138	0.031339	0.031339252	protein_coding	NM_006854
ASHGV40017795	-0.03134	0.031336415	protein_coding	NM_012385
ASHGV40002282	0.031315	0.031314797	protein_coding	NM_001195259
ASHGV40000137	-0.03124	0.031236517	protein_coding	NM_016354
ASHGV40012079	0.031151	0.031151267	protein_coding	NM_006253
ASHGV40040827	-0.03102	0.031020497	protein_coding	NM_016103
ASHGV40047393	0.030966	0.030965991	protein_coding	NM_020435
ASHGV40039380	0.030808	0.030807851	protein_coding	NM_173487
ASHGV40030784	-0.03079	0.030787572	protein_coding	NM_002212
ASHGV40006612	-0.03054	0.030536953	protein_coding	NM_003696
ASHGV40008339	0.030526	0.030525973	protein_coding	NM_001031854
ASHGV40040303	0.030405	0.030405453	protein_coding	NM_030649
ASHGV40055403	-0.03014	0.030138624	protein_coding	NM_001101357
ASHGV40043765	0.030107	0.030106932	protein_coding	NM_015599
ASHGV40015517	0.030086	0.030085651	protein_coding	NM_032374
ASHGV40046828	-0.03006	0.030055539	protein_coding	NM_020429
ASHGV40044463	0.029984	0.029983518	protein_coding	NM_002793
ASHGV40021658	-0.02998	0.029976178	protein_coding	NM_001278279
ASHGV40024438	-0.02982	0.029823514	protein_coding	NM_019884
ASHGV40034228	-0.02977	0.029770904	protein_coding	NM_032970
ASHGV40028308	0.029765	0.029764924	protein_coding	NM_148961
ASHGV40057365	0.029723	0.029723275	protein_coding	NM_032959
ASHGV40057004	-0.02968	0.029682769	protein_coding	NM_006607
ASHGV40031244	0.029631	0.029630956	protein_coding	NM_002836
ASHGV40027423	0.029522	0.029521701	protein_coding	uc010fmm.1
ASHGV40057868	-0.02942	0.029415425	protein_coding	uc001sbo.1
ASHGV40005770	0.029187	0.029187493	protein_coding	NM_018344
ASHGV40051996	-0.02903	0.029029009	protein_coding	NM_018109
ASHGV40024391	0.028921	0.028921025	protein_coding	NM_001626
ASHGV40044998	-0.02891	0.028910997	protein_coding	NM_021943
ASHGV40050673	-0.02889	0.028888795	protein_coding	NM_052898
ASHGV40006660	0.028743	0.028743435	protein_coding	NM_021211
ASHGV40042870	0.028546	0.028545701	protein_coding	NM_206880
ASHGV40022439	0.028545	0.028544794	protein_coding	NM_003409
ASHGV40011355	0.028428	0.028428239	protein_coding	NM_012272
ASHGV40029049	-0.02842	0.028424721	protein_coding	NM_004263
ASHGV40029414	-0.02833	0.028326514	protein_coding	NM_023070
ASHGV40036286	0.028295	0.028295338	protein_coding	NM_006070

ASHGV40031809	0.028208	0.028208389	protein_coding	NM_001012971
ASHGV40047452	-0.02817	0.028166174	protein_coding	NM_003751
ASHGV40057737	0.027992	0.027992361	protein_coding	NM_001039165
ASHGV40055416	-0.02792	0.027921861	protein_coding	NM_182540
ASHGV40045886	-0.02788	0.027883483	protein_coding	NM_016224
ASHGV40035588	0.0278	0.027799675	protein_coding	NM_002542
ASHGV40029906	-0.02777	0.02777148	protein_coding	NM_005966
ASHGV40031175	-0.0275	0.027497658	protein_coding	NM_017859
ASHGV40019493	0.027384	0.027384337	protein_coding	NM_012213
ASHGV40027272	0.027361	0.027361442	protein_coding	NM_032494
ASHGV40024359	-0.02734	0.027342233	protein_coding	NM_001533
ASHGV40035213	-0.02733	0.027330912	protein_coding	NM_020775
ASHGV40041745	0.027207	0.027207292	protein_coding	NM_052870
ASHGV40050236	0.027193	0.027193235	protein_coding	NM_001018039
ASHGV40050099	0.027159	0.027159165	protein_coding	NM_001960
ASHGV40049666	0.027092	0.027092445	protein_coding	NM_015942
ASHGV40014152	-0.02707	0.027072362	protein_coding	NM_017420
ASHGV40020376	-0.02704	0.027044704	protein_coding	uc002ica.2
ASHGV40024055	-0.02683	0.02683437	protein_coding	NM_031941
ASHGV40022796	-0.02683	0.026830745	protein_coding	NM_016626
ASHGV40032708	0.026711	0.026711237	protein_coding	NM_004915
ASHGV40015496	0.026606	0.026606028	protein_coding	NM_014844
ASHGV40007349	-0.02632	0.026323939	protein_coding	NM_182969
ASHGV40033151	-0.02618	0.026175388	protein_coding	NM_025045
ASHGV40035841	-0.02611	0.026111997	protein_coding	NM_001904
ASHGV40011180	-0.02607	0.026072923	protein_coding	NM_004264
ASHGV40008040	-0.02596	0.025958988	protein_coding	NM_012192
ASHGV40010117	0.025944	0.025944476	protein_coding	NM_005730
ASHGV40007101	-0.02593	0.025933169	protein_coding	NM_018093
ASHGV40027923	0.025881	0.025880917	protein_coding	NM_001127391
ASHGV40043268	0.025764	0.025764451	protein_coding	NM_021184
ASHGV40056057	0.025718	0.025717907	protein_coding	NM_144626
ASHGV40038549	-0.02562	0.025620026	protein_coding	NM_020773
ASHGV40055105	0.025591	0.025591422	protein_coding	NM_016500
ASHGV40043561	-0.02553	0.025534571	protein_coding	NM_001013732
ASHGV40010511	-0.02549	0.025485093	protein_coding	NM_032148
ASHGV40036355	-0.02543	0.025434904	protein_coding	NM_198196
ASHGV40031781	-0.02536	0.025361026	protein_coding	NM_173485
ASHGV40018495	0.025303	0.025303434	protein_coding	NM_053284
ASHGV40045857	0.025264	0.025263604	protein_coding	NM_014892
ASHGV40001343	0.025215	0.025214545	protein_coding	NM_002664
ASHGV40035135	-0.02501	0.025011589	protein_coding	NM_001308197
ASHGV40030355	0.025003	0.025003495	protein_coding	NM_001040445
ASHGV40054873	0.024895	0.02489459	protein_coding	NM_001289797
ASHGV40013912	0.024832	0.024831753	protein_coding	NM_001198773
ASHGV40057399	-0.0246	0.024601162	protein_coding	NM_130759
ASHGV40014601	-0.02448	0.024482926	protein_coding	NM_152326

ASHGV40019290	-0.02439	0.024387246	protein_coding	NM_005796
ASHGV40050425	-0.02437	0.02437015	protein_coding	NM_016240
ASHGV40055032	0.024243	0.024242883	protein_coding	NM_001010888
ASHGV40037090	0.024229	0.02422938	protein_coding	NM_033029
ASHGV40028718	0.02422	0.024220146	protein_coding	NM_024009
ASHGV40035622	0.024154	0.024153652	protein_coding	NM_015869
ASHGV40025091	0.024118	0.024118047	protein_coding	NM_002631
ASHGV40044534	-0.02412	0.02411713	protein_coding	NM_004824
ASHGV40021984	-0.02411	0.024111818	protein_coding	NM_021213
ASHGV40005936	0.024061	0.024061025	protein_coding	NM_019054
ASHGV40014291	-0.02399	0.023991578	protein_coding	NM_152445
ASHGV40000086	-0.02399	0.023987668	protein_coding	NM_014695
ASHGV40008121	0.02381	0.023810268	protein_coding	NM_021961
ASHGV40010961	-0.02376	0.023763967	protein_coding	NM_018173
ASHGV40005769	-0.02371	0.023710526	protein_coding	NM_170744
ASHGV40051641	-0.02341	0.023412369	protein_coding	NM_014314
ASHGV40010032	-0.02337	0.023373156	protein_coding	NM_002061
ASHGV40056056	0.02323	0.023230069	protein_coding	NM_015567
ASHGV40010128	0.023155	0.02315501	protein_coding	NM_144988
ASHGV40007043	0.023064	0.023063607	protein_coding	NM_016582
ASHGV40046086	-0.02302	0.023023596	protein_coding	NM_013393
ASHGV40025676	0.022884	0.022884398	protein_coding	NM_172140
ASHGV40001913	0.022874	0.022874408	protein_coding	ENST00000534438
ASHGV40018900	0.022805	0.022805098	protein_coding	NM_145239
ASHGV40038213	-0.02277	0.022769897	protein_coding	uc021xtv.1
ASHGV40043815	-0.02269	0.022692141	protein_coding	NM_020466
ASHGV40041881	-0.02265	0.022645978	protein_coding	NM_022132
ASHGV40046343	0.022617	0.02261689	protein_coding	NM_012322
ASHGV40025183	-0.02256	0.022557822	protein_coding	NM_003072
ASHGV40026845	0.022521	0.022521476	protein_coding	NM_014755
ASHGV40013878	0.022405	0.022405395	protein_coding	NM_020195
ASHGV40016671	-0.02239	0.02239381	protein_coding	NM_033286
ASHGV40025024	-0.02231	0.02231456	protein_coding	NM_170678
ASHGV40039440	-0.02229	0.02229284	protein_coding	NM_014487
ASHGV40025629	-0.02222	0.022223273	protein_coding	NM_152604
ASHGV40026077	-0.02213	0.022125315	protein_coding	NM_000991
ASHGV40009841	0.022087	0.022086755	protein_coding	NM_173601
ASHGV40021706	0.022057	0.022056862	protein_coding	NM_001552
ASHGV40050361	0.022	0.022000338	protein_coding	NM_005605
ASHGV40013245	-0.022	0.02199793	protein_coding	NM_024561
ASHGV40027268	-0.02185	0.021849736	protein_coding	NM_022662
ASHGV40006614	0.021827	0.021826815	protein_coding	NM_005897
ASHGV40048102	0.021624	0.021623979	protein_coding	NM_181646
ASHGV40032274	0.021571	0.021570576	protein_coding	NM_004965
ASHGV40043719	-0.02157	0.021565152	protein_coding	NM_001563
ASHGV40025843	0.021557	0.02155731	protein_coding	NM_015603
ASHGV40016489	-0.02153	0.021529803	protein_coding	NM_033418

ASHGV40014270	0.021388	0.021388009	protein_coding	NM_016190
ASHGV40051721	-0.02138	0.021377353	protein_coding	NM_016734
ASHGV40040287	-0.02129	0.021290679	protein_coding	NM_024930
ASHGV40035683	-0.02126	0.02125539	protein_coding	NM_144633
ASHGV40007302	0.021248	0.02124844	protein_coding	NM_023077
ASHGV40046849	0.021037	0.021036948	protein_coding	NM_005916
ASHGV40006156	0.021027	0.021026975	protein_coding	NM_183239
ASHGV40035477	0.020904	0.020904064	protein_coding	NM_004532
ASHGV40025683	0.020852	0.020851553	protein_coding	NM_001001563
ASHGV40038795	-0.02083	0.020826962	protein_coding	NM_025132
ASHGV40029341	-0.02082	0.020817069	protein_coding	NM_153712
ASHGV40017302	0.0207	0.020699777	protein_coding	NM_001271
ASHGV40022209	0.020673	0.020673305	protein_coding	NM_001545
ASHGV40026772	-0.02058	0.020579851	protein_coding	NM_014870
ASHGV40009916	-0.02048	0.020484946	protein_coding	NM_003394
ASHGV40045633	-0.02035	0.020351992	protein_coding	NM_001286398
ASHGV40042634	-0.02034	0.020343294	protein_coding	NM_001531
ASHGV40024995	-0.02024	0.020235688	protein_coding	NM_022787
ASHGV40049163	0.020231	0.02023057	protein_coding	NM_023034
ASHGV40052615	0.020206	0.020206406	protein_coding	NM_033439
ASHGV40014455	-0.02015	0.020150741	protein_coding	NM_001284280
ASHGV40031260	0.019976	0.019975902	protein_coding	NM_020746
ASHGV40026690	-0.01988	0.019880742	protein_coding	NM_018079
ASHGV40025366	-0.01984	0.019841931	protein_coding	NM_003333
ASHGV40056372	-0.01982	0.019822649	protein_coding	NM_178517
ASHGV40018934	0.019766	0.019765832	protein_coding	NM_000294
ASHGV40013653	-0.01975	0.019754838	protein_coding	NM_020205
ASHGV40008629	-0.01974	0.019737344	protein_coding	NM_001008778
ASHGV40050598	-0.01934	0.01933946	protein_coding	NM_152419
ASHGV40007603	-0.01931	0.019307333	protein_coding	NM_020809
ASHGV40042807	0.019252	0.019251764	protein_coding	NM_007255
ASHGV40020760	-0.01922	0.019224645	protein_coding	NM_017983
ASHGV40015853	0.019207	0.019207492	protein_coding	NM_017672
ASHGV40023727	0.019032	0.019031763	protein_coding	NM_001060
ASHGV40057390	-0.01902	0.01902141	protein_coding	NM_001008747
ASHGV40048377	0.018961	0.018961414	protein_coding	NM_012281
ASHGV40045047	0.018961	0.018960752	protein_coding	NM_138572
ASHGV40023913	0.018765	0.018764536	protein_coding	NM_138783
ASHGV40046175	0.018703	0.018702639	protein_coding	NM_001017425
ASHGV40000113	0.018643	0.018642641	protein_coding	ENST00000358799
ASHGV40021226	-0.01853	0.018531323	protein_coding	NM_003809
ASHGV40028626	0.018466	0.018465595	protein_coding	NM_001168241
ASHGV40009568	-0.01847	0.018465505	protein_coding	NM_001008661
ASHGV40034042	-0.01847	0.018465425	protein_coding	ENST00000253699
ASHGV40009321	0.018373	0.018373264	protein_coding	NM_003921
ASHGV40025923	-0.01837	0.018369808	protein_coding	NM_030973
ASHGV40036461	0.018212	0.018212378	protein_coding	NM_000373

ASHGV40057663	-0.01819	0.018185301	protein_coding	NM_017518
ASHGV40025111	-0.01815	0.018146046	protein_coding	NM_145245
ASHGV40046131	-0.01809	0.018091004	protein_coding	NM_014413
ASHGV40030596	-0.01803	0.018031899	protein_coding	NM_006606
ASHGV40050847	-0.01787	0.01787258	protein_coding	NM_006823
ASHGV40045333	-0.01787	0.017869916	protein_coding	NM_033411
ASHGV40013115	-0.01777	0.017770116	protein_coding	NM_001260
ASHGV40025994	0.01772	0.017720264	protein_coding	NM_001145434
ASHGV40019320	-0.01772	0.017718005	protein_coding	NM_016101
ASHGV40022292	-0.01768	0.017677533	protein_coding	NM_152468
ASHGV40028689	-0.01767	0.017672133	protein_coding	NM_001002257
ASHGV40011425	0.017629	0.017629123	protein_coding	NM_170754
ASHGV40006112	0.017472	0.017471882	protein_coding	NM_015448
ASHGV40019073	0.017393	0.017393349	protein_coding	NM_033119
ASHGV40056953	0.017248	0.017248232	protein_coding	NM_173084
ASHGV40043987	-0.0172	0.017198058	protein_coding	NM_001527
ASHGV40053720	0.017185	0.017185099	protein_coding	NM_000047
ASHGV40056306	-0.01718	0.017178989	protein_coding	NM_004483
ASHGV40003264	0.017136	0.017135759	protein_coding	NM_004765
ASHGV40019334	-0.01704	0.017036822	protein_coding	NM_152485
ASHGV40031215	0.016986	0.016985927	protein_coding	NM_006814
ASHGV40048214	0.016967	0.016967391	protein_coding	NM_004722
ASHGV40015107	0.016896	0.016896404	protein_coding	NM_001123329
ASHGV40025208	0.016797	0.016796671	protein_coding	NM_001136501
ASHGV40020433	0.016792	0.01679166	protein_coding	NM_152343
ASHGV40007513	-0.01658	0.016582337	protein_coding	ENST00000536061
ASHGV40015956	-0.01657	0.016565747	protein_coding	NM_001007595
ASHGV40024805	0.016531	0.016531291	protein_coding	NM_003283
ASHGV40054100	0.016498	0.016498275	protein_coding	NM_002547
ASHGV40030443	0.016495	0.016495353	protein_coding	NM_015506
ASHGV40008289	-0.01645	0.016447362	protein_coding	NM_014344
ASHGV40034283	-0.0163	0.016299353	protein_coding	NM_000258
ASHGV40002236	0.016282	0.016281541	protein_coding	NM_024516
ASHGV40044606	-0.01614	0.016143296	protein_coding	NM_002114
ASHGV40006797	0.016072	0.016072376	protein_coding	NM_002233
ASHGV40036017	-0.01589	0.015890628	protein_coding	NM_018398
ASHGV40021515	0.015851	0.015851119	protein_coding	NM_016518
ASHGV40008253	-0.0158	0.015801953	protein_coding	NM_002901
ASHGV40049313	0.015732	0.015732463	protein_coding	NM_001286657
ASHGV40026471	0.015701	0.015700852	protein_coding	NM_006277
ASHGV40012968	0.015679	0.015679488	protein_coding	NM_001286721
ASHGV40027130	-0.01558	0.015584649	protein_coding	NM_025244
ASHGV40023859	-0.01553	0.015525571	protein_coding	NM_152289
ASHGV40056037	-0.01546	0.015456365	protein_coding	NM_003295
ASHGV40029356	0.015405	0.015404855	protein_coding	NM_012455
ASHGV40006363	-0.01515	0.015150449	protein_coding	NM_145235
ASHGV40038286	0.015123	0.015122709	protein_coding	NM_144618

ASHGV40040870	-0.01494	0.014944082	protein_coding	NM_007240
ASHGV40019533	-0.01492	0.014922695	protein_coding	NM_002163
ASHGV40049072	0.014873	0.014873404	protein_coding	NM_001394
ASHGV40018581	-0.01474	0.014739946	protein_coding	NM_001199107
ASHGV40024436	-0.01473	0.014726035	protein_coding	NM_001207025
ASHGV40029707	0.014726	0.01472559	protein_coding	NM_006593
ASHGV40033628	-0.01468	0.014675377	protein_coding	NM_016009
ASHGV40032382	-0.01459	0.014586488	protein_coding	uc002zgk.4
ASHGV40037522	0.014268	0.014268147	protein_coding	NM_002703
ASHGV40045790	0.014221	0.014221264	protein_coding	NM_001278064
ASHGV40015112	-0.01387	0.01387095	protein_coding	NM_002028
ASHGV40016075	0.013853	0.013853298	protein_coding	NM_018003
ASHGV40021233	0.013773	0.013773229	protein_coding	NM_152379
ASHGV40006362	-0.0137	0.013702185	protein_coding	NM_078468
ASHGV40026424	0.01365	0.013650205	protein_coding	NM_001006657
ASHGV40038054	-0.01355	0.013548069	protein_coding	NM_002494
ASHGV40028864	0.013544	0.013544269	protein_coding	NM_024852
ASHGV40009580	-0.0135	0.01349897	protein_coding	NM_002543
ASHGV40006001	-0.01338	0.013379044	protein_coding	NM_183374
ASHGV40025985	-0.01319	0.013194421	protein_coding	NM_001310155
ASHGV40025333	-0.01314	0.013138406	protein_coding	NM_012088
ASHGV40005251	-0.01313	0.013133833	protein_coding	NM_001040177
ASHGV40043033	0.013106	0.013106418	protein_coding	NM_016495
ASHGV40021018	-0.01293	0.012928372	protein_coding	NM_001271006
ASHGV40003140	0.012842	0.012841772	protein_coding	NM_001191
ASHGV40022113	0.012801	0.012801318	protein_coding	NM_015462
ASHGV40045921	-0.0128	0.0127951	protein_coding	ENST00000356956
ASHGV40022872	-0.01279	0.012786266	protein_coding	NM_000633
ASHGV40013848	-0.01265	0.012646177	protein_coding	NM_000257
ASHGV40044847	-0.01264	0.012639025	protein_coding	NM_020442
ASHGV40039093	-0.01256	0.012561952	protein_coding	NM_004354
ASHGV40006290	0.012437	0.012437012	protein_coding	NM_007190
ASHGV40005768	0.012389	0.012389242	protein_coding	NM_033487
ASHGV40033755	-0.01233	0.012332537	protein_coding	NM_006256
ASHGV40020276	0.012275	0.012275119	protein_coding	NM_001001998
ASHGV40003110	0.01216	0.012160247	protein_coding	NM_001098527
ASHGV40036043	0.011904	0.01190361	protein_coding	NM_017771
ASHGV40055914	-0.01187	0.011868849	protein_coding	NM_176792
ASHGV40024647	0.011857	0.011857268	protein_coding	NM_021733
ASHGV40011382	-0.01152	0.011522278	protein_coding	NM_015416
ASHGV40057264	-0.01135	0.011347121	protein_coding	NM_003764
ASHGV40024443	-0.01122	0.011220132	protein_coding	NM_032488
ASHGV40007374	0.010852	0.010851888	protein_coding	NM_002370
ASHGV40038915	-0.01084	0.010836172	protein_coding	NM_024592
ASHGV40011015	0.010566	0.010566398	protein_coding	NM_016184
ASHGV40024888	-0.0105	0.010500308	protein_coding	NM_152474
ASHGV40057583	0.010495	0.010494654	protein_coding	NM_004192

ASHGV40023154	0.01048	0.01047956	protein_coding	NM_001277333
ASHGV40038290	-0.01047	0.010471909	protein_coding	NM_006529
ASHGV40008618	-0.01045	0.010448934	protein_coding	NM_013299
ASHGV40008380	-0.0104	0.010400145	protein_coding	NM_005693
ASHGV40026058	0.010339	0.010339228	protein_coding	NM_013289
ASHGV40018932	-0.01027	0.010266317	protein_coding	NM_003929
ASHGV40035130	0.010259	0.010259268	protein_coding	NM_004733
ASHGV40044559	-0.01021	0.010210942	protein_coding	NM_152551
ASHGV40054191	-0.01019	0.0101927	protein_coding	NM_144657
ASHGV40016786	0.010092	0.010091759	protein_coding	NM_002044
ASHGV40043020	0.01	0.009999882	protein_coding	NM_032744
ASHGV40019823	-0.00999	0.009990082	protein_coding	NM_025099
ASHGV40015776	0.009977	0.009977129	protein_coding	NM_153260
ASHGV40007245	-0.00986	0.00985548	protein_coding	NM_001277
ASHGV40049510	0.009852	0.009852092	protein_coding	NM_014018
ASHGV40023688	-0.00977	0.009766142	protein_coding	NM_020695
ASHGV40055317	-0.00976	0.009756922	protein_coding	NM_001560
ASHGV40027463	-0.00965	0.009654761	protein_coding	NM_030923
ASHGV40013801	-0.00965	0.009645994	protein_coding	NM_000396
ASHGV40011891	-0.00946	0.009455849	protein_coding	NM_001253849
ASHGV40035858	-0.00932	0.009315797	protein_coding	NM_001129908
ASHGV40020129	0.009237	0.009236733	protein_coding	NM_024683
ASHGV40016334	-0.00907	0.009066758	protein_coding	NM_002666
ASHGV40033162	0.008963	0.008963378	protein_coding	NM_144573
ASHGV40048452	-0.00896	0.008957936	protein_coding	NM_178562
ASHGV40013764	0.008893	0.008893067	protein_coding	NM_018178
ASHGV40021859	-0.00886	0.008864553	protein_coding	NM_014726
ASHGV40043275	-0.00884	0.008844072	protein_coding	NM_025261
ASHGV40011076	0.008843	0.008842621	protein_coding	NM_016355
ASHGV40034565	0.008826	0.008826105	protein_coding	NM_000028
ASHGV40009307	0.008817	0.008816523	protein_coding	NM_017425
ASHGV40031571	-0.00881	0.008814893	protein_coding	NM_080607
ASHGV40015231	-0.0087	0.008695821	protein_coding	NM_145870
ASHGV40012013	0.008562	0.008562357	protein_coding	NM_016816
ASHGV40056587	-0.00853	0.008528863	protein_coding	ENST00000369159
ASHGV40041008	0.008459	0.00845936	protein_coding	NM_018047
ASHGV40055147	0.008279	0.008278855	protein_coding	NM_198450
ASHGV40020949	0.008243	0.008242895	protein_coding	ENST00000586713
ASHGV40010886	-0.00824	0.008236013	protein_coding	NM_032636
ASHGV40036895	0.008155	0.008154519	protein_coding	NM_018023
ASHGV40028528	-0.00805	0.008054431	protein_coding	NM_145175
ASHGV40015981	-0.00795	0.007952579	protein_coding	NM_003922
ASHGV40040537	-0.00793	0.00792984	protein_coding	NM_004365
ASHGV40027887	0.007924	0.007923713	protein_coding	NM_012086
ASHGV40032415	0.007874	0.007873935	protein_coding	NM_058180
ASHGV40039428	0.007819	0.007819289	protein_coding	NM_002413
ASHGV40051195	-0.00772	0.007721327	protein_coding	NM_173685

ASHGV40035845	-0.00751	0.007513507	protein_coding	NM_004624
ASHGV40041914	-0.00749	0.007491406	protein_coding	NM_032175
ASHGV40009341	-0.00743	0.007429832	protein_coding	NM_017547
ASHGV40005515	0.00734	0.0073397	protein_coding	NM_001881
ASHGV40003286	-0.00731	0.007308399	protein_coding	NM_016019
ASHGV40008814	0.007185	0.007184535	protein_coding	NM_145309
ASHGV40039324	0.007162	0.007161889	protein_coding	NM_002004
ASHGV40037115	-0.00711	0.007113757	protein_coding	NM_001347
ASHGV40030716	-0.00711	0.007105584	protein_coding	NM_147192
ASHGV40036509	-0.007	0.007004812	protein_coding	NM_000174
ASHGV40033525	0.006798	0.006797987	protein_coding	NM_004175
ASHGV40005463	0.00674	0.006740124	protein_coding	uc001iur.2
ASHGV40037117	-0.00673	0.006725339	protein_coding	NM_022042
ASHGV40042683	0.006412	0.006411526	protein_coding	NM_002887
ASHGV40025011	0.006349	0.00634873	protein_coding	NM_020170
ASHGV40049582	0.006348	0.006348326	protein_coding	NM_016033
ASHGV40029258	0.00634	0.006340351	protein_coding	NM_003048
ASHGV40047178	0.006331	0.006330731	protein_coding	NM_001190848
ASHGV40003307	-0.00624	0.006239263	protein_coding	NM_024496
ASHGV40031789	-0.00624	0.006235951	protein_coding	NM_002623
ASHGV40018822	0.006196	0.006196106	protein_coding	NM_019116
ASHGV40053142	-0.00598	0.005978243	protein_coding	ENST00000411791
ASHGV40038088	-0.0059	0.005902223	protein_coding	NM_014885
ASHGV40013861	-0.00572	0.005724257	protein_coding	NM_006177
ASHGV40055073	-0.00562	0.005615805	protein_coding	NM_181303
ASHGV40018740	0.005554	0.005554432	protein_coding	NM_000537
ASHGV40030408	-0.00554	0.005537266	protein_coding	NM_013325
ASHGV40054754	-0.00549	0.005486407	protein_coding	NM_015884
ASHGV40039635	-0.00542	0.005420376	protein_coding	NM_001034845
ASHGV40053692	-0.00533	0.005334485	protein_coding	NM_003631
ASHGV40015687	0.005245	0.005244701	protein_coding	NM_005159
ASHGV40045408	-0.00519	0.005188521	protein_coding	NM_024641
ASHGV40010588	0.005002	0.005001702	protein_coding	NM_002710
ASHGV40037186	-0.00493	0.004932453	protein_coding	NM_001313
ASHGV40053157	0.004923	0.004923442	protein_coding	NM_001193329
ASHGV40021021	0.00478	0.004779744	protein_coding	NM_022156
ASHGV40045877	0.004776	0.004775754	protein_coding	NM_017519
ASHGV40056001	0.004668	0.004668137	protein_coding	uc001txn.1
ASHGV40043531	0.004548	0.004548297	protein_coding	NM_032111
ASHGV40003220	-0.00445	0.004451477	protein_coding	NM_001289984
ASHGV40054791	-0.00444	0.004439874	protein_coding	NM_002363
ASHGV40042588	-0.00439	0.004386907	protein_coding	NM_005927
ASHGV40048304	-0.00438	0.004375568	protein_coding	NM_181581
ASHGV40048917	-0.00437	0.004368198	protein_coding	NM_201402
ASHGV40053995	0.004367	0.004366677	protein_coding	NM_017602
ASHGV40012492	0.004118	0.004118452	protein_coding	NM_002015
ASHGV40045721	0.004109	0.004108617	protein_coding	NM_000288

ASHGV40056418	-0.00397	0.003967448	protein_coding	NM_032478
ASHGV40058273	0.003775	0.003775472	protein_coding	uc001yte.1
ASHGV40054577	-0.00362	0.003622957	protein_coding	NM_005334
ASHGV40029753	-0.00361	0.003608458	protein_coding	NM_001003845
ASHGV40042236	0.003578	0.003578224	protein_coding	NM_014035
ASHGV40050194	0.003501	0.003501345	protein_coding	NM_014867
ASHGV40055033	-0.00334	0.003341294	protein_coding	NM_002444
ASHGV40003136	0.00334	0.003339739	protein_coding	NM_001164165
ASHGV40045354	-0.00315	0.003146421	protein_coding	NM_015021
ASHGV40014679	-0.0031	0.003103993	protein_coding	NM_207370
ASHGV40037097	0.003091	0.003091014	protein_coding	NM_006472
ASHGV40055266	0.003083	0.003082769	protein_coding	NM_032428
ASHGV40019671	-0.00291	0.002914965	protein_coding	NM_052928
ASHGV40013780	-0.00287	0.002865561	protein_coding	NM_017807
ASHGV40054654	-0.00285	0.002851001	protein_coding	NM_000351
ASHGV40053172	0.002807	0.002806634	protein_coding	NM_001042357
ASHGV40033911	0.00278	0.002779541	protein_coding	NM_052839
ASHGV40033076	0.002778	0.002777617	protein_coding	NM_004737
ASHGV40052338	-0.00273	0.00272605	protein_coding	NM_013443
ASHGV40031764	-0.00262	0.002620994	protein_coding	NM_017843
ASHGV40025311	-0.00258	0.002584063	protein_coding	NM_001007525
ASHGV40003362	0.002456	0.002455998	protein_coding	NM_201550
ASHGV40025092	-0.00233	0.002327445	protein_coding	NM_001044388
ASHGV40040146	0.002313	0.002313021	protein_coding	NM_002036
ASHGV40054731	-0.00228	0.002283635	protein_coding	NM_001291867
ASHGV40031483	0.002233	0.002233489	protein_coding	NM_014742
ASHGV40022330	-0.00223	0.00222644	protein_coding	NM_173627
ASHGV40018066	0.002168	0.002167654	protein_coding	NM_005550
ASHGV40055069	-0.00214	0.002144735	protein_coding	NM_021120
ASHGV40000139	0.002065	0.002065473	protein_coding	NM_001291332
ASHGV40008093	0.001985	0.001984866	protein_coding	NM_181712
ASHGV40033584	0.001736	0.001736105	protein_coding	NM_001267895
ASHGV40010692	-0.00168	0.001677974	protein_coding	NM_198202
ASHGV40020413	0.001609	0.001609367	protein_coding	NM_198475
ASHGV40008655	-0.00142	0.001418229	protein_coding	NM_203350
ASHGV40021025	0.001384	0.001384214	protein_coding	NM_001893
ASHGV40038719	-0.00119	0.0011923	protein_coding	NM_012437
ASHGV40018165	0.001161	0.001161151	protein_coding	NM_032140
ASHGV40029539	0.00115	0.001149708	protein_coding	NM_002410
ASHGV40033176	-0.00106	0.001059942	protein_coding	NM_175709
ASHGV40033702	-0.00105	0.001054107	protein_coding	NM_152243
ASHGV40011619	0.00098	0.000979633	protein_coding	NM_198080
ASHGV40017276	-0.00095	0.000947322	protein_coding	NM_033544
ASHGV40013945	0.00094	0.000939585	protein_coding	NM_018453
ASHGV40042603	0.000858	0.000857909	protein_coding	NM_020950
ASHGV40007781	-0.0008	0.000804464	protein_coding	NM_170601
ASHGV40030216	0.000595	0.000595202	protein_coding	NM_032276

ASHGV40035794	-0.00059	0.000590368	protein_coding	NM_007204
ASHGV40047209	-0.00054	0.000540059	protein_coding	NM_020247
ASHGV40010004	0.000538	0.000538018	protein_coding	NM_000889
ASHGV40052244	-0.0005	0.000498379	protein_coding	NM_018249
ASHGV40018765	-0.00049	0.000486394	protein_coding	NM_016138
ASHGV40005098	-0.00031	0.000310885	protein_coding	NM_001304762
ASHGV40007645	0.000309	0.000309371	protein_coding	NM_004724
ASHGV40042374	0.000304	0.000304499	protein_coding	NM_001964
ASHGV40025725	-0.00028	0.000282877	protein_coding	NM_000709
ASHGV40040998	-0.00018	0.000175234	protein_coding	NM_002609

transcript_type	GeneSymbol
protein_coding	OPALIN
protein_coding	TMSB15A
protein_coding	SPARCL1
protein_coding	ERMN
protein_coding	S100B
protein_coding	CNDP1
protein_coding	ETNPPL
protein_coding	SNAP25
protein_coding	PLP1
protein_coding	KIAA1211L
protein_coding	TF
protein_coding	CHN1
protein_coding	CCNB2
protein_coding	GABRA1
protein_coding	SLC1A2
protein_coding	TPPP
protein_coding	SPINK1
protein_coding	CRYGC
protein_coding	CABP1
protein_coding	APOA2
protein_coding	FAM107A
protein_coding	CRYBA4
protein_coding	MTRNR2L2
protein_coding	NUSAP1
protein_coding	PAQR6
protein_coding	APOH
protein_coding	PTGDS
protein_coding	SPOCK2
protein_coding	VSNL1
protein_coding	RRM2
protein_coding	SOX11
protein_coding	MELK
protein_coding	HHATL
protein_coding	PITX2
protein_coding	AFP
protein_coding	ENPP2
protein_coding	CRYBA1
protein_coding	CX3CL1
protein_coding	HIST1H4L
protein_coding	AK5
protein_coding	LIM2
protein_coding	KLK6
protein_coding	BCAS1
protein_coding	FGB
protein_coding	COL3A1

protein_coding	NTSR2
protein_coding	ST8SIA2
protein_coding	E2F7
protein_coding	HBG1
protein_coding	RGS4
protein_coding	AMBP
protein_coding	CRYAB
protein_coding	SOX10
protein_coding	MBP
protein_coding	TOP2A
protein_coding	RASD1
protein_coding	KCNC2
protein_coding	ARAP2
protein_coding	COL1A1
protein_coding	VASH2
protein_coding	ALB
protein_coding	GABRG1
protein_coding	KLF9
protein_coding	EOMES
protein_coding	RAPGEF4
protein_coding	CLU
protein_coding	LRRC3DN
protein_coding	PMP2
protein_coding	AQP4
protein_coding	BUB1
protein_coding	GPM6B
protein_coding	BC033961
protein_coding	SYT1
protein_coding	OGDHL
protein_coding	HOTS
protein_coding	SERPINI1
protein_coding	IGF2BP3
protein_coding	LAD1
protein_coding	ADRB1
protein_coding	HIST1H2AJ
protein_coding	NAT8
protein_coding	CAMK2N1
protein_coding	PLA2G16
protein_coding	MTRNR2L8
protein_coding	PLG
protein_coding	GABARAPL3
protein_coding	AGR2
protein_coding	EDIL3
protein_coding	RASGRF1
protein_coding	NKX6-2
protein_coding	IGF2BP2
protein_coding	HOXB6

protein_coding	IGIP
protein_coding	AHSG
protein_coding	LCE3E
protein_coding	CENPU
protein_coding	GFAP
protein_coding	ASPM
protein_coding	AK125437
protein_coding	RPH3A
protein_coding	STMN3
protein_coding	EVI2A
protein_coding	MT-ND3
protein_coding	AGR3
protein_coding	MBNL2
protein_coding	SCN2B
protein_coding	KIAA0845
protein_coding	LECT2
protein_coding	MT-ND2
protein_coding	FAM102A
protein_coding	DGKB
protein_coding	FOLH1
protein_coding	HOPX
protein_coding	RNASE1
protein_coding	KIF14
protein_coding	CAMK2A
protein_coding	MT-ATP8
protein_coding	SULT1E1
protein_coding	NAP1L2
protein_coding	SEPT4
protein_coding	NDRG2
protein_coding	HEPN1
protein_coding	TM4SF5
protein_coding	NEFL
protein_coding	SCN1A
protein_coding	LUM
protein_coding	OLIG3
protein_coding	YWHAH
protein_coding	HSPA12A
protein_coding	PTPRN2
protein_coding	SOWAHA
protein_coding	NEFM
protein_coding	SSBP3-AS1
protein_coding	MOG
protein_coding	NSF
protein_coding	WEE1
protein_coding	SLC22A17
protein_coding	TKTL1
protein_coding	NECAB1

protein_coding	KIF4A
protein_coding	NAP1L3
protein_coding	MDH1
protein_coding	CD99L2
protein_coding	KCNV1
protein_coding	CCDC85B
protein_coding	CDK1
protein_coding	FBN2
protein_coding	ATP2B2
protein_coding	FBN3
protein_coding	DOCK10
protein_coding	NAP1L5
protein_coding	COL15A1
protein_coding	UGT2A3
protein_coding	HIST1H4I
protein_coding	CRYM
protein_coding	SLC5A11
protein_coding	SORL1
protein_coding	GPR158
protein_coding	AMER2
protein_coding	CCDC92
protein_coding	MYH8
protein_coding	LEMD1
protein_coding	PNMA6A
protein_coding	ESRP2
protein_coding	MT-CO2
protein_coding	DCX
protein_coding	SLC51A
protein_coding	NGFRAP1
protein_coding	GATA5
protein_coding	SPOCK3
protein_coding	EZH2
protein_coding	BUB1B
protein_coding	GSTA1
protein_coding	CELSR1
protein_coding	PSAP
protein_coding	SVOP
protein_coding	MT-CO1
protein_coding	MTRNR2L1
protein_coding	AKAP6
protein_coding	POTEH
protein_coding	LIPG
protein_coding	HOXB3
protein_coding	HOXA5
protein_coding	KIF18A
protein_coding	LPAR2
protein_coding	AIFM3

protein_coding	GPX8
protein_coding	GIMAP7
protein_coding	KCND3
protein_coding	C17orf96
protein_coding	CRNDE
protein_coding	ABCC2
protein_coding	POTEG
protein_coding	CEP55
protein_coding	GRIA2
protein_coding	YWHAG
protein_coding	MEP1A
protein_coding	CPXM1
protein_coding	ABLIM2
protein_coding	WIF1
protein_coding	NPTN
protein_coding	CDC25C
protein_coding	RP11-1026M7.2
protein_coding	LYNX1
protein_coding	HOXA2
protein_coding	SYCE2
protein_coding	MYBPC1
protein_coding	MKI67
protein_coding	CA10
protein_coding	CDHR5
protein_coding	MAG
protein_coding	MT-ATP6
protein_coding	BEX4
protein_coding	BHLHE22
protein_coding	MAGEA2B
protein_coding	HOXA1
protein_coding	FGA
protein_coding	STAT4
protein_coding	ENO1
protein_coding	BEX1
protein_coding	POLQ
protein_coding	POTEI
protein_coding	SULT2A1
protein_coding	RBFOX1
protein_coding	PBK
protein_coding	MTURN
protein_coding	GSTA5
protein_coding	PTPN5
protein_coding	ARHGAP11B
protein_coding	GPR37
protein_coding	LCT
protein_coding	GSTA3
protein_coding	LOX

protein_coding	CNP
protein_coding	FEZ1
protein_coding	CA11
protein_coding	DIO3
protein_coding	MMP1
protein_coding	COX6A1P2
protein_coding	TNK2
protein_coding	NGEF
protein_coding	FGG
protein_coding	ITPK1
protein_coding	RAB6C
protein_coding	NRCAM
protein_coding	OXR1
protein_coding	MAGEA12
protein_coding	RTKN
protein_coding	BEND6
protein_coding	RERG
protein_coding	ATP1B1
protein_coding	PRNP
protein_coding	HCN1
protein_coding	QDPR
protein_coding	CCK
protein_coding	CPA2
protein_coding	CTSF
protein_coding	LOC283685
protein_coding	ANLN
protein_coding	NALCN
protein_coding	TSPYL1
protein_coding	RAB38
protein_coding	NDUFA4
protein_coding	SLC24A2
protein_coding	GPRIN2
protein_coding	MT-CO3
protein_coding	HSD17B2
protein_coding	KIAA0101
protein_coding	CPNE5
protein_coding	IHH
protein_coding	HBT8
protein_coding	KIF20A
protein_coding	NEUROG2
protein_coding	RBP2
protein_coding	LYSMD2
protein_coding	CHCHD2
protein_coding	PLEKHH1
protein_coding	TFF1
protein_coding	STC1
protein_coding	SNRPN

protein_coding	KDM5D
protein_coding	MT-ND5
protein_coding	HIST1H3B
protein_coding	NEUROG1
protein_coding	MYLK
protein_coding	PRR18
protein_coding	PLCB1
protein_coding	FAM171B
protein_coding	APOD
protein_coding	PEG3
protein_coding	APELA
protein_coding	OR14I1
protein_coding	YWHAB
protein_coding	ANXA13
protein_coding	ZBTB16
protein_coding	CENPK
protein_coding	F8
protein_coding	DEPDC1B
protein_coding	OMG
protein_coding	PRPH2
protein_coding	RPL10
protein_coding	CBLN4
protein_coding	PTPRT
protein_coding	AX748369
protein_coding	SERINC3
protein_coding	ENPP6
protein_coding	RCAN2
protein_coding	SLCO6A1
protein_coding	SETD1A
protein_coding	MAGEA1
protein_coding	MYBL2
protein_coding	MDK
protein_coding	NHP2L1
protein_coding	NR5A2
protein_coding	HOXC9
protein_coding	IQGAP2
protein_coding	DEFB1
protein_coding	RDM1
protein_coding	MT-ND4L
protein_coding	CHCHD10
protein_coding	C4BPB
protein_coding	GATM
protein_coding	ACYP2
protein_coding	HSPB3
protein_coding	PYCR2
protein_coding	KRT15
protein_coding	EPS8L1

protein_coding	HIGD1A
protein_coding	CSRP1
protein_coding	UGP2
protein_coding	NEFH
protein_coding	TCEAL7
protein_coding	PMP22
protein_coding	MT-ND1
protein_coding	HSPA8
protein_coding	GRIA4
protein_coding	TESC
protein_coding	HIST1H4G
protein_coding	HIST1H2AD
protein_coding	AJUBA
protein_coding	EPB41L3
protein_coding	COL6A3
protein_coding	ATXN7L3B
protein_coding	TTLL7
protein_coding	COL4A6
protein_coding	PIP4K2A
protein_coding	HIST1H2AC
protein_coding	ACSL6
protein_coding	SOX3
protein_coding	USP2
protein_coding	AGT
protein_coding	FHL2
protein_coding	CYP27C1
protein_coding	MUC13
protein_coding	RP11-366L20.2
protein_coding	ME1
protein_coding	CST3
protein_coding	TMEM144
protein_coding	PPIAL4G
protein_coding	APOA4
protein_coding	PPP1R14A
protein_coding	CALM1
protein_coding	CPA6
protein_coding	VEPH1
protein_coding	CABP4
protein_coding	GSX2
protein_coding	DPPA4
protein_coding	LCE2C
protein_coding	AC068987.1
protein_coding	ORC1
protein_coding	DCLK1
protein_coding	ANK3
protein_coding	PRKCB
protein_coding	ZMAT4

protein_coding	VIP
protein_coding	CKS2
protein_coding	CACNB1
protein_coding	FAIM2
protein_coding	HSD17B6
protein_coding	E2F8
protein_coding	MFSD4
protein_coding	MIA2
protein_coding	ALDH1A1
protein_coding	EN1
protein_coding	TCEAL5
protein_coding	NRXN1
protein_coding	HIST1H3J
protein_coding	OSBPL1A
protein_coding	MPP1
protein_coding	CDRT4
protein_coding	GLUL
protein_coding	GAPDH
protein_coding	RASL10A
protein_coding	RIMS2
protein_coding	AC137932.1
protein_coding	PNMA1
protein_coding	STYK1
protein_coding	RGS5
protein_coding	HOXD9
protein_coding	FSIP2
protein_coding	S100A6
protein_coding	KCNAB2
protein_coding	RXFP1
protein_coding	GNGT1
protein_coding	GBA3
protein_coding	NANOS2
protein_coding	GALNTL5
protein_coding	DSC3
protein_coding	LDHB
protein_coding	DEFB135
protein_coding	APP
protein_coding	GAS2L3
protein_coding	RPL34
protein_coding	KCNK1
protein_coding	SH2D5
protein_coding	VWA8
protein_coding	HIST1H2AI
protein_coding	MAP1A
protein_coding	ITPR1
protein_coding	MYL10
protein_coding	TTC9B

protein_coding	XAGE3
protein_coding	SLC20A2
protein_coding	C2orf74
protein_coding	NRN1
protein_coding	CDH17
protein_coding	LPAR1
protein_coding	XG
protein_coding	SST
protein_coding	KIF1A
protein_coding	HIST1H3G
protein_coding	TCF21
protein_coding	CAMK2B
protein_coding	ZFYVE28
protein_coding	HOXA11
protein_coding	LRRC4B
protein_coding	C15orf59
protein_coding	GHITM
protein_coding	STAC2
protein_coding	CHEK1
protein_coding	SERINC1
protein_coding	RGCC
protein_coding	PNMAL2
protein_coding	MAP6
protein_coding	SNCB
protein_coding	ERCC6L
protein_coding	FBXO2
protein_coding	PPIAL4A
protein_coding	GPR62
protein_coding	PIP4K2B
protein_coding	SLC2A13
protein_coding	PF4V1
protein_coding	HOXA3
protein_coding	BAALC
protein_coding	ATP2B1
protein_coding	SLC4A10
protein_coding	HIST1H2AH
protein_coding	S100A1
protein_coding	ADIRF
protein_coding	AL135998.1
protein_coding	PLEKHG3
protein_coding	GNAS
protein_coding	MLF1IP
protein_coding	FTL
protein_coding	HIST1H4D
protein_coding	LGALS4
protein_coding	DIRAS2
protein_coding	DMRTA2

protein_coding	SLC26A3
protein_coding	ADAMTS2
protein_coding	CPE
protein_coding	HIST1H2BH
protein_coding	MXD3
protein_coding	EFEMP1
protein_coding	HIST1H1A
protein_coding	NEK2
protein_coding	CTNNA3
protein_coding	F2
protein_coding	ADAMDEC1
protein_coding	PPP2R2C
protein_coding	STARD13
protein_coding	IP6K3
protein_coding	OR52N5
protein_coding	SGTB
protein_coding	FAM134B
protein_coding	NCAM2
protein_coding	E2F1
protein_coding	JUNB
protein_coding	NDC80
protein_coding	GRIA3
protein_coding	AK123878
protein_coding	MAP3K5
protein_coding	IQSEC2
protein_coding	SEPP1
protein_coding	ACVR1C
protein_coding	CDH18
protein_coding	ABCA3
protein_coding	APOB
protein_coding	ANXA11
protein_coding	SLC17A7
protein_coding	COX7C
protein_coding	KCNB1
protein_coding	HELLS
protein_coding	TULP1
protein_coding	PREPL
protein_coding	SNX10
protein_coding	PPP2R2B
protein_coding	UNC13C
protein_coding	HABP4
protein_coding	AC092811.1
protein_coding	CHL1
protein_coding	MATK
protein_coding	SLC39A12
protein_coding	AL590714.1
protein_coding	CPEB1

protein_coding	CTAG2
protein_coding	LCE3D
protein_coding	HJURP
protein_coding	LMTK3
protein_coding	GPC2
protein_coding	MYEOV2
protein_coding	PRKACB
protein_coding	GDF3
protein_coding	FBXW7
protein_coding	ARPP19
protein_coding	NGB
protein_coding	PEA15
protein_coding	RNH1
protein_coding	NUPR1L
protein_coding	NINJ2
protein_coding	KIF3A
protein_coding	VAMP1
protein_coding	BTG1
protein_coding	PDP1
protein_coding	GTF2H2
protein_coding	PPAP2B
protein_coding	LANCL1
protein_coding	HOMER1
protein_coding	CPM
protein_coding	BDH1
protein_coding	IRX2
protein_coding	SLC1A3
protein_coding	SERPINA6
protein_coding	CHRNA3
protein_coding	ALDH6A1
protein_coding	PAGE2B
protein_coding	BTD
protein_coding	DEXI
protein_coding	PLA2G12B
protein_coding	SI
protein_coding	DKFZP761K2322
protein_coding	CADM2
protein_coding	F8A3
protein_coding	PITX1
protein_coding	C1QL3
protein_coding	GALNT8
protein_coding	PMCH
protein_coding	HSPB1
protein_coding	UBC
protein_coding	CBR1
protein_coding	HAVCR1
protein_coding	CH25H

protein_coding	SLC12A2
protein_coding	GPC3
protein_coding	AP2M1
protein_coding	CDR1
protein_coding	TACC1
protein_coding	AHCYL1
protein_coding	SLC17A2
protein_coding	EFR3A
protein_coding	APMAP
protein_coding	KCNH1
protein_coding	MAGEA4
protein_coding	HSPH1
protein_coding	PCDH9
protein_coding	WNT8B
protein_coding	GRIN2C
protein_coding	FABP1
protein_coding	ADAMTS7
protein_coding	ATP6V1C1
protein_coding	DPPA2
protein_coding	AC006372.1
protein_coding	CACNG2
protein_coding	CAMK2G
protein_coding	SIRT2
protein_coding	MEGT1
protein_coding	RTN4
protein_coding	HAS2
protein_coding	MCOLN3
protein_coding	TRIM71
protein_coding	CRYGD
protein_coding	EPCAM
protein_coding	SCG5
protein_coding	KRT5
protein_coding	GRID1
protein_coding	CYP3A5
protein_coding	CDKN2D
protein_coding	THY1
protein_coding	NDUFB8
protein_coding	OSR2
protein_coding	SKOR2
protein_coding	SPATC1L
protein_coding	AP001055.1
protein_coding	SEZ6L2
protein_coding	LRRK2
protein_coding	ATP6V1E1
protein_coding	ADAMTS6
protein_coding	TK1
protein_coding	SGSM3

protein_coding	TUBB2A
protein_coding	LIFR
protein_coding	RBFOX3
protein_coding	FAM83B
protein_coding	WDR72
protein_coding	APC
protein_coding	PDZD3
protein_coding	PRRT1
protein_coding	ABCD2
protein_coding	LRRC4C
protein_coding	NTRK2
protein_coding	AC008498.1
protein_coding	C17orf49
protein_coding	STXBP5L
protein_coding	STOML1
protein_coding	HIST1H2AG
protein_coding	ISL1
protein_coding	SOX1
protein_coding	GPR87
protein_coding	ARL6IP4
protein_coding	CPB2
protein_coding	HSPB8
protein_coding	HIST1H4B
protein_coding	ACOT7
protein_coding	KIF21A
protein_coding	CILP
protein_coding	PCP4
protein_coding	FGFR4
protein_coding	HPS6
protein_coding	IGF2
protein_coding	HTR2A
protein_coding	UQCRH
protein_coding	NEBL
protein_coding	AC005609.1
protein_coding	MTRNR2L7
protein_coding	HOXC8
protein_coding	DYNC1LI2
protein_coding	TBX2
protein_coding	HOXA10
protein_coding	NAPB
protein_coding	KLK1
protein_coding	NDUFS3
protein_coding	SLC23A1
protein_coding	BFSP2
protein_coding	KIF5C
protein_coding	AREG
protein_coding	EIF4A2

protein_coding	KIF23
protein_coding	SLCO1A2
protein_coding	ARMCX5-GPRASP2
protein_coding	FIGF
protein_coding	SHC3
protein_coding	FST
protein_coding	BCL2L14
protein_coding	PCDH8
protein_coding	AADAC
protein_coding	PATE3
protein_coding	MFSD6L
protein_coding	AP000867.1
protein_coding	OLFM1
protein_coding	OR7G1
protein_coding	TMEM229A
protein_coding	SLC10A1
protein_coding	NAGPA
protein_coding	TRIM43B
protein_coding	CKB
protein_coding	MGST3
protein_coding	COL16A1
protein_coding	SEPT8
protein_coding	HIST1H3H
protein_coding	AGXT2
protein_coding	PLA2G2A
protein_coding	HBG2
protein_coding	UGT2B4
protein_coding	TAGLN3
protein_coding	NRGN
protein_coding	HIST1H3F
protein_coding	MCTP1
protein_coding	HPGD
protein_coding	RUNDC3A
protein_coding	PINK1
protein_coding	KCNIP4
protein_coding	AL590867.1
protein_coding	ADH4
protein_coding	PLEKHG4B
protein_coding	GUCY1B3
protein_coding	NDUFB2
protein_coding	CNKSR2
protein_coding	AX747227
protein_coding	NCKAP1
protein_coding	ADAMTS4
protein_coding	EEA1
protein_coding	CXCL13
protein_coding	AP000783.1

protein_coding	C8orf89
protein_coding	IL1RAPL1
protein_coding	DSG2
protein_coding	ATL1
protein_coding	DLG1
protein_coding	CKAP2L
protein_coding	CYGB
protein_coding	CERS1
protein_coding	TPD52
protein_coding	GLRB
protein_coding	ANO9
protein_coding	STXBP1
protein_coding	RP11-429E11.3
protein_coding	KRTAP10-10
protein_coding	ARGFX
protein_coding	SPC25
protein_coding	CEACAM5
protein_coding	MICAL2
protein_coding	LRRIQ1
protein_coding	MEOX1
protein_coding	GSN
protein_coding	AKR1B10
protein_coding	MAP2K4
protein_coding	KRTAP9-4
protein_coding	DNAH17
protein_coding	ANXA10
protein_coding	CCDC37
protein_coding	AK055785
protein_coding	SDPR
protein_coding	HSP90AB1
protein_coding	PRKCZ
protein_coding	ZNF365
protein_coding	HMGA2
protein_coding	SPEF1
protein_coding	NPRL3
protein_coding	P2RY12
protein_coding	KCTD4
protein_coding	CDC20B
protein_coding	TSPYL2
protein_coding	CALM3
protein_coding	SLC39A2
protein_coding	PIK3R1
protein_coding	GOT2
protein_coding	FAM83G
protein_coding	CLEC14A
protein_coding	GALNT9
protein_coding	SYN1

protein_coding	PGAM4
protein_coding	PXDN
protein_coding	FTCDNL1
protein_coding	NPC1L1
protein_coding	B4GAT1
protein_coding	CLDN4
protein_coding	DRAXIN
protein_coding	TFPI2
protein_coding	A1BG
protein_coding	SCG3
protein_coding	ELF3
protein_coding	CHGA
protein_coding	SKP1
protein_coding	HHIP
protein_coding	PRPH
protein_coding	NPIP
protein_coding	SECISBP2L
protein_coding	IQCF1
protein_coding	KIFAP3
protein_coding	LAMP3
protein_coding	MAGEA8
protein_coding	UQCRHL
protein_coding	PRUNE2
protein_coding	RPL15
protein_coding	DLG2
protein_coding	LCE2D
protein_coding	OAT
protein_coding	ARMC3
protein_coding	GAB4
protein_coding	CCL25
protein_coding	MATR3
protein_coding	TPX2
protein_coding	SREBF1
protein_coding	RANBP3L
protein_coding	C9orf69
protein_coding	AVPR2
protein_coding	SYNM
protein_coding	PLK1
protein_coding	NR3C1
protein_coding	PHACTR1
protein_coding	MUC15
protein_coding	LOC642846
protein_coding	POPDC3
protein_coding	RAB11FIP5
protein_coding	ENPP1
protein_coding	RNF122
protein_coding	CYFIP2

protein_coding	MYCBP2
protein_coding	MGLL
protein_coding	BTBD18
protein_coding	SVIL
protein_coding	AK127378
protein_coding	HOXC5
protein_coding	OR2T27
protein_coding	G3BP2
protein_coding	MTTP
protein_coding	FAM183A
protein_coding	HMGCS2
protein_coding	UBBP4
protein_coding	ATP5J2
protein_coding	C6orf223
protein_coding	ZYG11A
protein_coding	IGF2BP1
protein_coding	ENC1
protein_coding	DPEP1
protein_coding	C6orf118
protein_coding	LOXL1
protein_coding	TRIM9
protein_coding	GABRB3
protein_coding	ATP6V1D
protein_coding	SLCO1B1
protein_coding	LILRA3
protein_coding	STON1-GTF2A1L
protein_coding	OR6K2
protein_coding	NES
protein_coding	RAB25
protein_coding	WASL
protein_coding	ATP7B
protein_coding	ARR3
protein_coding	B3GALT2
protein_coding	KCNJ2
protein_coding	KIAA1107
protein_coding	EEF1A2
protein_coding	HIST1H2BI
protein_coding	EFCAB13
protein_coding	BACH2
protein_coding	BEGAIN
protein_coding	PRODH2
protein_coding	OR4D5
protein_coding	ATG3
protein_coding	GABRD
protein_coding	BRINP1
protein_coding	HIST2H3D
protein_coding	REG4

protein_coding	GRM3
protein_coding	AC091150.1
protein_coding	ACE2
protein_coding	NR2E3
protein_coding	AK127555
protein_coding	GCG
protein_coding	CAMKK2
protein_coding	RAPGEF5
protein_coding	KRTAP27-1
protein_coding	ATP5B
protein_coding	ZFAND5
protein_coding	FTH1
protein_coding	GPR18
protein_coding	AX747565
protein_coding	AVPI1
protein_coding	FAM198B
protein_coding	P2RX7
protein_coding	TGFB1
protein_coding	DPP9
protein_coding	SPG20
protein_coding	CSAG3
protein_coding	UBE2Q2L
protein_coding	ALDH2
protein_coding	PLA2G2D
protein_coding	SCARB2
protein_coding	UBD
protein_coding	PHYHIPL
protein_coding	SUCLA2
protein_coding	ZBTB38
protein_coding	IL15RA
protein_coding	RGS7
protein_coding	UGT1A9
protein_coding	SDR16C5
protein_coding	AL353698.1
protein_coding	CTCFL
protein_coding	TSPAN2
protein_coding	SPATA31A1
protein_coding	AP000974.1
protein_coding	PPP3CA
protein_coding	OR51D1
protein_coding	NKX6-1
protein_coding	RFTN2
protein_coding	LMBRD1
protein_coding	CDCA7
protein_coding	RPL18A
protein_coding	RMDN3
protein_coding	HTRA1

protein_coding	PSAPL1
protein_coding	RNF130
protein_coding	PTPN11
protein_coding	OGFRL1
protein_coding	AP3B2
protein_coding	ARHGEF5
protein_coding	MAP7D2
protein_coding	KIRREL
protein_coding	KLHDC8A
protein_coding	SERF1B
protein_coding	BC037321
protein_coding	ITM2B
protein_coding	CDHR4
protein_coding	OR14C36
protein_coding	APLP1
protein_coding	SNAI2
protein_coding	IDS
protein_coding	TEKT2
protein_coding	PAIP2
protein_coding	PGK1
protein_coding	DKFZp586G0322
protein_coding	ALDOA
protein_coding	PRDX1
protein_coding	MYB
protein_coding	HLA-C
protein_coding	CTSV
protein_coding	RYR2
protein_coding	SMYD2
protein_coding	AKR1D1
protein_coding	CALM2
protein_coding	KCNK5
protein_coding	MCM3AP-AS1
protein_coding	ACTL9
protein_coding	EPHA4
protein_coding	ATP5O
protein_coding	CDKL5
protein_coding	TAL2
protein_coding	GNL1
protein_coding	GJB6
protein_coding	MCHR2
protein_coding	DPP8
protein_coding	MAOB
protein_coding	DYNLL1
protein_coding	TES
protein_coding	B4GALT6
protein_coding	SIX5
protein_coding	NRXN3

protein_coding	A2M
protein_coding	ADAMTS8
protein_coding	TFF3
protein_coding	SYTL2
protein_coding	TRHDE
protein_coding	PINLYP
protein_coding	SPP1
protein_coding	CDCA3
protein_coding	METTL21C
protein_coding	CNTD2
protein_coding	ACSM4
protein_coding	C11orf44
protein_coding	GP1BA
protein_coding	FZD2
protein_coding	KRTAP4-6
protein_coding	STXBP3
protein_coding	CCNI
protein_coding	RAB6A
protein_coding	FAM127B
protein_coding	HPRT1
protein_coding	HMP19
protein_coding	ZHX1
protein_coding	IAPP
protein_coding	OR1M1
protein_coding	XAGE2
protein_coding	NDUFB4
protein_coding	FOXN1
protein_coding	AC132216.1
protein_coding	KRTAP3-3
protein_coding	UBL3
protein_coding	MGAT4D
protein_coding	KRTAP9-8
protein_coding	ZSCAN10
protein_coding	GUK1
protein_coding	IGFBP7
protein_coding	C3
protein_coding	TNFAIP8L3
protein_coding	CDK5
protein_coding	PCMT1
protein_coding	UBE2C
protein_coding	POTEE
protein_coding	CHRM3
protein_coding	HOGA1
protein_coding	PPP1R3C
protein_coding	HRSP12
protein_coding	CMAS
protein_coding	COBL

protein_coding	RIPPLY1
protein_coding	ALDOC
protein_coding	GDE1
protein_coding	ST8SIA3
protein_coding	PRAMEF9
protein_coding	CADPS2
protein_coding	KIF2C
protein_coding	SOD1
protein_coding	PLCH2
protein_coding	AL354808.2
protein_coding	LOC619207
protein_coding	CCDC91
protein_coding	PSG6
protein_coding	IL36B
protein_coding	FAM90A1
protein_coding	IFIT1
protein_coding	RBM7
protein_coding	C16orf3
protein_coding	MMP2
protein_coding	KCNS2
protein_coding	MKL2
protein_coding	SMIM7
protein_coding	FAM24A
protein_coding	TFF2
protein_coding	MFAP3L
protein_coding	LAMP2
protein_coding	PDX1
protein_coding	PSPH
protein_coding	TMEM59
protein_coding	AC026407.1
protein_coding	S100A13
protein_coding	TFE3
protein_coding	ADAM7
protein_coding	ANGPTL3
protein_coding	PEBP1
protein_coding	TNNC1
protein_coding	AFG3L2
protein_coding	MRO
protein_coding	KHDRBS3
protein_coding	NMBR
protein_coding	OR10H2
protein_coding	HMOX2
protein_coding	BCL2L1
protein_coding	EPB41L2
protein_coding	NAPEPLD
protein_coding	LPPR4
protein_coding	SLC25A18

protein_coding	MAP1LC3C
protein_coding	KLK2
protein_coding	GSC
protein_coding	KIAA1644
protein_coding	PPP1R16B
protein_coding	RGS13
protein_coding	ZNF483
protein_coding	GBP7
protein_coding	PHACTR3
protein_coding	NRIP3
protein_coding	DUSP13
protein_coding	HACD3
protein_coding	FAM98A
protein_coding	CALML4
protein_coding	NETO1
protein_coding	CCDC122
protein_coding	SH2B3
protein_coding	CLDN3
protein_coding	SPINK13
protein_coding	FAM13C
protein_coding	TPH1
protein_coding	BC016978
protein_coding	GJC1
protein_coding	NTRK3
protein_coding	TFAP2A
protein_coding	CLUH
protein_coding	DEAF1
protein_coding	ACTG2
protein_coding	SLAMF7
protein_coding	ZNF526
protein_coding	RBL1
protein_coding	AX746903
protein_coding	PTP4A2
protein_coding	MMP10
protein_coding	EMC2
protein_coding	ARHGEF39
protein_coding	LOC100130301
protein_coding	CLCN1
protein_coding	KAT2B
protein_coding	UGT2B15
protein_coding	ACSL3
protein_coding	HSPA2
protein_coding	AC115618.1
protein_coding	HDDC2
protein_coding	ISLR2
protein_coding	SCRG1
protein_coding	SOAT2

protein_coding	TLK1
protein_coding	KCNH3
protein_coding	SH3GL3
protein_coding	C10orf90
protein_coding	RERGL
protein_coding	ZIC3
protein_coding	RPL31
protein_coding	NR1D2
protein_coding	KCNK12
protein_coding	KIFC1
protein_coding	GPM6A
protein_coding	NTS
protein_coding	ELMSAN1
protein_coding	HOXA13
protein_coding	CXCR4
protein_coding	HOXC6
protein_coding	AC016885.1
protein_coding	TXN
protein_coding	RP11-180C1.1
protein_coding	GNAO1
protein_coding	GCNT7
protein_coding	DPPA5
protein_coding	RASSF5
protein_coding	PBOV1
protein_coding	VWF
protein_coding	PSG2
protein_coding	CEL
protein_coding	STC2
protein_coding	SCN2A
protein_coding	SESN1
protein_coding	TMEM176B
protein_coding	LIF
protein_coding	KRT34
protein_coding	RTN3
protein_coding	MUM1L1
protein_coding	ABAT
protein_coding	AC138517.1
protein_coding	STAC
protein_coding	KIF22
protein_coding	ALDH1L1
protein_coding	CTSL2
protein_coding	L1TD1
protein_coding	F8A1
protein_coding	SMCO3
protein_coding	P4HA3
protein_coding	ROCK2
protein_coding	TEX14

protein_coding	ART5
protein_coding	CGB8
protein_coding	LCA5L
protein_coding	CACNG3
protein_coding	AICDA
protein_coding	GSTA2
protein_coding	GPR82
protein_coding	TMEM114
protein_coding	SDHC
protein_coding	MAL2
protein_coding	RUFY3
protein_coding	PROZ
protein_coding	GABARAPL1
protein_coding	AP000688.1
protein_coding	MBL2
protein_coding	CTB-78H18.1
protein_coding	ATRNL1
protein_coding	ZSCAN2
protein_coding	TTBK1
protein_coding	PTMS
protein_coding	HOXD11
protein_coding	AK125157
protein_coding	FAM179B
protein_coding	CUBN
protein_coding	TRIM2
protein_coding	C11orf88
protein_coding	SLC51B
protein_coding	SYT11
protein_coding	PDZK1
protein_coding	CHRNG
protein_coding	SPANXB1
protein_coding	UBQLN3
protein_coding	NDUFS5
protein_coding	KLHL14
protein_coding	IRS4
protein_coding	NUDT12
protein_coding	GOT1
protein_coding	RP11-94B19.4
protein_coding	PCNXL2
protein_coding	IL2RA
protein_coding	AL359091.2
protein_coding	S100A14
protein_coding	SMPDL3B
protein_coding	EMR1
protein_coding	TTK
protein_coding	NPPB
protein_coding	SFTA3

protein_coding	APOBEC3D
protein_coding	OR2B6
protein_coding	CXorf65
protein_coding	C6orf132
protein_coding	C1orf195
protein_coding	LCTL
protein_coding	ZBED4
protein_coding	HOXB5
protein_coding	OSCAR
protein_coding	SLC3A1
protein_coding	SOX4
protein_coding	PTK2B
protein_coding	SRMS
protein_coding	KRT13
protein_coding	SEC62
protein_coding	ADH1B
protein_coding	SLC2A2
protein_coding	APOBEC2
protein_coding	CAPG
protein_coding	IGSF9
protein_coding	MAGI2
protein_coding	UGT2B10
protein_coding	PNLIPRP3
protein_coding	CELF4
protein_coding	AX746996
protein_coding	MACC1
protein_coding	CREG1
protein_coding	MASP1
protein_coding	TMEM207
protein_coding	RNF6
protein_coding	LGI1
protein_coding	PGM2L1
protein_coding	INSRR
protein_coding	ATP1B2
protein_coding	LHX9
protein_coding	TM4SF20
protein_coding	OR5H8P
protein_coding	DAPL1
protein_coding	CD1E
protein_coding	MAB21L1
protein_coding	PITPNM2
protein_coding	ATP2A1
protein_coding	ZNF677
protein_coding	CCNF
protein_coding	CYP2J2
protein_coding	IPCEF1
protein_coding	RASA4B

protein_coding	EPHB6
protein_coding	PPP1R14D
protein_coding	ZSCAN18
protein_coding	KCNMB4
protein_coding	C16orf52
protein_coding	ONECUT1
protein_coding	LRRC19
protein_coding	GNG7
protein_coding	VCPIP1
protein_coding	ATP5I
protein_coding	OR4P4
protein_coding	PLEKHB1
protein_coding	NUAK1
protein_coding	CCDC73
protein_coding	ITPA
protein_coding	AF131215.5
protein_coding	RP11-352D3.2
protein_coding	C17orf78
protein_coding	TCRBV6S6A2T
protein_coding	TCRGV
protein_coding	ENO2
protein_coding	PAX3
protein_coding	MEX3A
protein_coding	SHCBP1L
protein_coding	SLC47A2
protein_coding	AC131971.1
protein_coding	NCOA7
protein_coding	GRIN2A
protein_coding	PDE2A
protein_coding	C9orf131
protein_coding	HBS1L
protein_coding	KCNF1
protein_coding	GPR125
protein_coding	HLA-DRA
protein_coding	OAZ1
protein_coding	INSC
protein_coding	CLDN10
protein_coding	ATP6V1A
protein_coding	TADA3
protein_coding	HIST1H2BG
protein_coding	MZT1
protein_coding	FAM81A
protein_coding	XIRP1
protein_coding	SVIP
protein_coding	IDH3A
protein_coding	KCNAB1
protein_coding	OR11A1

protein_coding	RP11-347C12.1
protein_coding	KLK14
protein_coding	ANKRD29
protein_coding	MAGEB6
protein_coding	AES
protein_coding	GOLGB1
protein_coding	OR1L8
protein_coding	ALOXE3
protein_coding	GPR156
protein_coding	AC026202.1
protein_coding	ACSM2A
protein_coding	HPCA
protein_coding	EU250752
protein_coding	AC024940.1
protein_coding	SP8
protein_coding	MAP7D1
protein_coding	PDE8B
protein_coding	TSPAN3
protein_coding	TEX13B
protein_coding	TMSB15B
protein_coding	RIBC2
protein_coding	OR4C15
protein_coding	NAPRT1
protein_coding	PDE6A
protein_coding	COX4I1
protein_coding	LIPA
protein_coding	RSPH1
protein_coding	HV535487
protein_coding	FOLH1B
protein_coding	SAG
protein_coding	CAPS2
protein_coding	GAD1
protein_coding	OIP5
protein_coding	DMXL2
protein_coding	ATP5J
protein_coding	RNF208
protein_coding	HIST2H4A
protein_coding	HOXB8
protein_coding	NDUFB3
protein_coding	SFTA2
protein_coding	RAB41
protein_coding	ATP5A1
protein_coding	IL24
protein_coding	NOXA1
protein_coding	TMOD2
protein_coding	EME1
protein_coding	CRH

protein_coding	TAGLN2
protein_coding	MTRNR2L3
protein_coding	HSP90AB4P
protein_coding	EBNA1BP2
protein_coding	SAGE1
protein_coding	NELL2
protein_coding	GLYCTK
protein_coding	AURKA
protein_coding	KRTAP7-1
protein_coding	CST5
protein_coding	AUH
protein_coding	DEPDC1
protein_coding	RPL37
protein_coding	AX747630
protein_coding	PDE7B
protein_coding	ELF4
protein_coding	OXCT1
protein_coding	GPKOW
protein_coding	PDK4
protein_coding	BAGE5
protein_coding	CD200R1L
protein_coding	PIR
protein_coding	C3orf65
protein_coding	SKA1
protein_coding	MC3R
protein_coding	ZBTB4
protein_coding	MMP13
protein_coding	RB1CC1
protein_coding	LOC541467
protein_coding	GMNC
protein_coding	RNF175
protein_coding	GCNT4
protein_coding	CRLF1
protein_coding	DYNLT3
protein_coding	PPM1J
protein_coding	AC104472.1
protein_coding	CLIP4
protein_coding	OR1Q1
protein_coding	ADAMTS15
protein_coding	PRKAR1A
protein_coding	ITM2C
protein_coding	VWA5B1
protein_coding	NPY
protein_coding	CENPM
protein_coding	NUP62CL
protein_coding	MARCKSL1
protein_coding	MTMR10

protein_coding	PPP2CB
protein_coding	DCAF6
protein_coding	FAM71A
protein_coding	DQ580766
protein_coding	DKFZp434J194
protein_coding	OPCML
protein_coding	ANK2
protein_coding	C2orf80
protein_coding	TNFRSF12A
protein_coding	HIST1H1D
protein_coding	ATXN10
protein_coding	LAG3
protein_coding	CT47A1
protein_coding	CLOCK
protein_coding	DTNA
protein_coding	USP11
protein_coding	MEX3B
protein_coding	MOCOS
protein_coding	ADAMTS1
protein_coding	CD244
protein_coding	C1orf189
protein_coding	BAG1
protein_coding	POTEA
protein_coding	DLGAP5
protein_coding	EBF3
protein_coding	NDFIP1
protein_coding	NACA
protein_coding	MYL1
protein_coding	IRF6
protein_coding	GRAMD3
protein_coding	OPTN
protein_coding	SLCO1B7
protein_coding	DEFB110
protein_coding	BEST2
protein_coding	MOAP1
protein_coding	SLC7A8
protein_coding	RPP25
protein_coding	PPP4R1L
protein_coding	ALOX15B
protein_coding	CXXC5
protein_coding	AK124121
protein_coding	C10orf91
protein_coding	MINOS1
protein_coding	RP11-595B24.2
protein_coding	TMEM247
protein_coding	PPAP2C
protein_coding	C14orf178

protein_coding	KIAA1109
protein_coding	COL6A5
protein_coding	BIN2
protein_coding	ME3
protein_coding	TSC22D4
protein_coding	ADORA1
protein_coding	ANK1
protein_coding	SPTBN1
protein_coding	TGIF2LX
protein_coding	PDE1C
protein_coding	MFSD9
protein_coding	TUBA1A
protein_coding	PIN1
protein_coding	NEDD8
protein_coding	SLC23A3
protein_coding	TNFRSF19
protein_coding	CEACAM8
protein_coding	ACSBG1
protein_coding	KIF15
protein_coding	HIST1H2BF
protein_coding	C17orf70
protein_coding	ZC3H12A
protein_coding	RP11-219B4.5
protein_coding	CACNB2
protein_coding	ATP5G3
protein_coding	KRTCAP3
protein_coding	AGTR1
protein_coding	BHLHE40
protein_coding	ULBP1
protein_coding	LAMC2
protein_coding	PTK7
protein_coding	ANKRD42
protein_coding	NME5
protein_coding	CBR3
protein_coding	ESPN
protein_coding	BPY2
protein_coding	PLEKHH3
protein_coding	HNRNPCL2
protein_coding	METTL7A
protein_coding	CD1C
protein_coding	UHRF1BP1L
protein_coding	ADAM23
protein_coding	TRPM6
protein_coding	CLDN9
protein_coding	TPM3
protein_coding	CDON
protein_coding	KIAA0247

protein_coding	FAAP100
protein_coding	ATP6V1C2
protein_coding	PRAMEF19
protein_coding	SYT2
protein_coding	PRED57
protein_coding	CYP46A1
protein_coding	CRYAA
protein_coding	CFL2
protein_coding	PPP3R1
protein_coding	MRPL51
protein_coding	DDAH2
protein_coding	BPIFC
protein_coding	PAPOLB
protein_coding	FAM19A2
protein_coding	MAPT
protein_coding	CYP19A1
protein_coding	TM2D3
protein_coding	HIST1H2BA
protein_coding	AC226150.4
protein_coding	SCEL
protein_coding	STON1
protein_coding	HTRA4
protein_coding	OR2H2
protein_coding	FGF7
protein_coding	CCDC85C
protein_coding	C9orf57
protein_coding	FUT3
protein_coding	KTN1
protein_coding	MME
protein_coding	C1QTNF6
protein_coding	GIPR
protein_coding	BCAP29
protein_coding	RP11-368I7.4
protein_coding	SGK1
protein_coding	CAMTA1
protein_coding	MFAP5
protein_coding	NDUFB5
protein_coding	NRSN1
protein_coding	HMX2
protein_coding	WI2-3308P17.2
protein_coding	PAK1
protein_coding	CAPZA2
protein_coding	PLSCR5
protein_coding	LMAN1L
protein_coding	TLL2
protein_coding	DNAJA4
protein_coding	PROM1

protein_coding	GRTP1
protein_coding	PRKCQ
protein_coding	BMP5
protein_coding	RP11-108K14.8
protein_coding	CTAGE4
protein_coding	OAZ2
protein_coding	CTD-2054N24.2
protein_coding	HOXD10
protein_coding	SLC9A9
protein_coding	PARPBP
protein_coding	MAP2K1
protein_coding	MYH6
protein_coding	SHB
protein_coding	CD24
protein_coding	C1QL2
protein_coding	IMPAD1
protein_coding	DEFB106A
protein_coding	AC019294.1
protein_coding	PKHD1L1
protein_coding	RP11-169F17.1
protein_coding	FBP1
protein_coding	C1orf210
protein_coding	IL22RA2
protein_coding	PLVAP
protein_coding	PSTPIP2
protein_coding	TOMM70A
protein_coding	FRRS1L
protein_coding	LIN28B
protein_coding	SCN4A
protein_coding	DNMT3B
protein_coding	FAM90A26
protein_coding	RPL12
protein_coding	RPLP0
protein_coding	HIST1H4J
protein_coding	GRHL3
protein_coding	CIR1
protein_coding	hsa-mir-150
protein_coding	SELENBP1
protein_coding	UTS2B
protein_coding	TESPA1
protein_coding	STOX1
protein_coding	PSMB6
protein_coding	KRTAP4-8
protein_coding	SLC31A2
protein_coding	HCRTR1
protein_coding	RORA
protein_coding	LRRC36

protein_coding	IFNK
protein_coding	OR5A1
protein_coding	TRAK2
protein_coding	RP11-204N11.1
protein_coding	PPP2CA
protein_coding	NCAPG
protein_coding	AC069547.1
protein_coding	MFSD3
protein_coding	SPACA3
protein_coding	C20orf96
protein_coding	NDUFA5
protein_coding	ACBD7
protein_coding	EPDR1
protein_coding	WDR63
protein_coding	GABRA4
protein_coding	BTLA
protein_coding	OLAH
protein_coding	COL4A1
protein_coding	NODAL
protein_coding	CRABP2
protein_coding	TWIST1
protein_coding	TAOK3
protein_coding	TPRN
protein_coding	RSPO2
protein_coding	VNN2
protein_coding	FUT1
protein_coding	PLCL1
protein_coding	ACTBL2
protein_coding	SLCO1C1
protein_coding	PSMA7
protein_coding	COPS5
protein_coding	RTN1
protein_coding	THEM4
protein_coding	RP11-9B6.1
protein_coding	BC118554
protein_coding	GABRB2
protein_coding	BIRC8
protein_coding	GDF1
protein_coding	FAM83D
protein_coding	INHBC
protein_coding	GPR64
protein_coding	NEK7
protein_coding	UQCRFS1
protein_coding	IL18RAP
protein_coding	RET
protein_coding	FXYD6
protein_coding	VPS13A

protein_coding	UBE2D3
protein_coding	DCT
protein_coding	RP11-404P21.8
protein_coding	TBX19
protein_coding	FXYD3
protein_coding	RIPPLY2
protein_coding	HMHB1
protein_coding	MBTD1
protein_coding	MEF2B
protein_coding	NCDN
protein_coding	OSR1
protein_coding	OR2B3
protein_coding	TMA7
protein_coding	TFPI
protein_coding	KIR2DS4
protein_coding	MAP7
protein_coding	AFM
protein_coding	LOC100129940
protein_coding	H2BFM
protein_coding	OR9Q1
protein_coding	TSPY2
protein_coding	NDUFA10
protein_coding	CCDC173
protein_coding	FSTL4
protein_coding	PRR13
protein_coding	MYH4
protein_coding	GAS2
protein_coding	DFNB31
protein_coding	MTFR2
protein_coding	DPP6
protein_coding	PLA2G4C
protein_coding	REXO1L1
protein_coding	IL5RA
protein_coding	HS6ST3
protein_coding	NARS
protein_coding	HLA-B
protein_coding	AX747171
protein_coding	CPLX2
protein_coding	GPRASP1
protein_coding	NT5DC1
protein_coding	DYNC1H1
protein_coding	CD72
protein_coding	PFKP
protein_coding	C2orf83
protein_coding	FNDC3A
protein_coding	SRPK3
protein_coding	LRRC8B

protein_coding	SNU13
protein_coding	NDUFS1
protein_coding	ASB14
protein_coding	QPCT
protein_coding	SLC25A4
protein_coding	FCRL1
protein_coding	BTBD3
protein_coding	B2M
protein_coding	C17orf102
protein_coding	EIF3A
protein_coding	B3GNT5
protein_coding	SLC25A31
protein_coding	SLC44A5
protein_coding	AC114783.1
protein_coding	SPX
protein_coding	CPNE4
protein_coding	ATP8A1
protein_coding	CNTN1
protein_coding	SAMD3
protein_coding	CXCL17
protein_coding	RP11-105C20.2
protein_coding	AX747659
protein_coding	OR2T11
protein_coding	GAGE12I
protein_coding	STAT5A
protein_coding	JAKMIP1
protein_coding	RBM12B-AS1
protein_coding	ST18
protein_coding	HNRNPA1
protein_coding	TRIM43
protein_coding	AC074091.13
protein_coding	RP11-216L13.17
protein_coding	HIST1H4K
protein_coding	PSMC6
protein_coding	EXO1
protein_coding	RNF11
protein_coding	NFE2L2
protein_coding	CCDC28A
protein_coding	BOLL
protein_coding	AMHR2
protein_coding	TMEM126B
protein_coding	AX746699
protein_coding	ZNF729
protein_coding	C1QL4
protein_coding	PENK
protein_coding	ST3GAL5
protein_coding	CENPF

protein_coding	PNLIPRP2
protein_coding	CTSB
protein_coding	SCN1B
protein_coding	STMND1
protein_coding	MPP7
protein_coding	PCDHGA8
protein_coding	GBX2
protein_coding	SLC35G6
protein_coding	RBMX2
protein_coding	EPHA3
protein_coding	OR8J1
protein_coding	PSMA4
protein_coding	TNFRSF1B
protein_coding	ATPAF1
protein_coding	RP11-187E13.2
protein_coding	CFAP43
protein_coding	IFNA6
protein_coding	TRPC7
protein_coding	LPIN2
protein_coding	SERPINA7
protein_coding	DLL4
protein_coding	ZMYM5
protein_coding	PRDM12
protein_coding	RPL6
protein_coding	TYRO3
protein_coding	SLC25A12
protein_coding	PAH
protein_coding	FAM83F
protein_coding	ENSA
protein_coding	AL009178.1
protein_coding	CLRN1
protein_coding	KLF2
protein_coding	CDC25A
protein_coding	GPHB5
protein_coding	SERPINE2
protein_coding	AP000758.1
protein_coding	AKR1B1
protein_coding	DACT1
protein_coding	MROH2B
protein_coding	FLG
protein_coding	HPCAL1
protein_coding	PCLO
protein_coding	SPAG11B
protein_coding	NDUFAF1
protein_coding	WASH4P
protein_coding	PRMT8
protein_coding	ABHD12B

protein_coding	AXDND1
protein_coding	SMIM2
protein_coding	SPRYD3
protein_coding	NEMF
protein_coding	PTGIS
protein_coding	C10orf62
protein_coding	RASGRP1
protein_coding	ART3
protein_coding	SRPK2
protein_coding	ANKMY1
protein_coding	TSPAN11
protein_coding	CDC45
protein_coding	GRIN1
protein_coding	ADRA1D
protein_coding	FBXL17
protein_coding	RP11-131H24.4
protein_coding	ATP6V1B2
protein_coding	LOC100134391
protein_coding	HIST1H2BC
protein_coding	ABCC8
protein_coding	TRIM40
protein_coding	CCR5
protein_coding	RNF13
protein_coding	SLITRK6
protein_coding	NFE2L3
protein_coding	NBPF7
protein_coding	CTC-432M15.3
protein_coding	TKTL2
protein_coding	ACP5
protein_coding	MCF2
protein_coding	TEX22
protein_coding	TMPRSS4
protein_coding	TSPYL4
protein_coding	CLEC4F
protein_coding	ITM2A
protein_coding	DKKL1
protein_coding	CSNK2B-LY6G5B-1181
protein_coding	CKMT1A
protein_coding	PRAMEF10
protein_coding	UCHL1
protein_coding	MAST3
protein_coding	FSCN3
protein_coding	RCAN1
protein_coding	LRRC73
protein_coding	MEF2C
protein_coding	C9orf84
protein_coding	XRCC2

protein_coding	BBOX1
protein_coding	IFNA10
protein_coding	FNDC8
protein_coding	ITGB1BP1
protein_coding	OR10AD1
protein_coding	LOC391322
protein_coding	TLN1
protein_coding	C5orf58
protein_coding	THOC7
protein_coding	MIEN1
protein_coding	TUBB6
protein_coding	ZAK
protein_coding	LOC653513
protein_coding	BAG4
protein_coding	NT5C1B
protein_coding	PRSS38
protein_coding	IL6ST
protein_coding	FAM124A
protein_coding	TMC7
protein_coding	ICMT
protein_coding	CCNJ
protein_coding	UMOD
protein_coding	METTL11B
protein_coding	DNAH12
protein_coding	DTL
protein_coding	NAT8B
protein_coding	FAM47A
protein_coding	SPANXB2
protein_coding	FOXN4
protein_coding	LIG4
protein_coding	ANKRD1
protein_coding	GHSR
protein_coding	LCP2
protein_coding	DOC2A
protein_coding	SUN5
protein_coding	C9orf117
protein_coding	ARF3
protein_coding	OR6T1
protein_coding	KCNJ6
protein_coding	SPTLC3
protein_coding	AB429224
protein_coding	PSMC5
protein_coding	RNF212
protein_coding	CAPNS1
protein_coding	CSMD2
protein_coding	RHEB
protein_coding	REPS2

protein_coding	ATG4C
protein_coding	SLITRK1
protein_coding	C7orf57
protein_coding	STMN1
protein_coding	LRRC63
protein_coding	SLA
protein_coding	GABRG2
protein_coding	IGDCC3
protein_coding	ARPC5L
protein_coding	SERGEF
protein_coding	CYP2A7
protein_coding	GLUD1
protein_coding	RPSAP58
protein_coding	EIF1
protein_coding	ZNF578
protein_coding	ABCA5
protein_coding	OR8I2
protein_coding	LINC00908
protein_coding	WDR76
protein_coding	SSX2IP
protein_coding	MDFI
protein_coding	CDS1
protein_coding	DAZ4
protein_coding	DGKK
protein_coding	LMNB1
protein_coding	ARNT2
protein_coding	PPP1R1B
protein_coding	ZNF283
protein_coding	PIM1
protein_coding	COPS2
protein_coding	EPB41
protein_coding	SLC5A9
protein_coding	AK097878
protein_coding	GIMAP5
protein_coding	NUF2
protein_coding	HIST1H3I
protein_coding	AFAP1L2
protein_coding	MTRNR2L11
protein_coding	CCT3
protein_coding	CCDC144NL
protein_coding	SLC6A9
protein_coding	CTAGE1
protein_coding	HSD17B14
protein_coding	IQCA1
protein_coding	ALDH5A1
protein_coding	PRAMEF14
protein_coding	CERS4

protein_coding	NFKB2
protein_coding	PCDH20
protein_coding	TCRVA15
protein_coding	CCDC163P
protein_coding	AC010536.1
protein_coding	COX7B
protein_coding	TMEFF2
protein_coding	MS4A3
protein_coding	KLRD1
protein_coding	CISD1
protein_coding	HPCAL4
protein_coding	OTUD6A
protein_coding	GNG3
protein_coding	TREM2
protein_coding	AMER1
protein_coding	VAPB
protein_coding	SERPINA10
protein_coding	KIAA1024L
protein_coding	PDCD1
protein_coding	PRSS12
protein_coding	DNAJA2
protein_coding	SPTLC2
protein_coding	AARS
protein_coding	SUGP2
protein_coding	MVP
protein_coding	TCEAL2
protein_coding	RBMY1E
protein_coding	C1orf112
protein_coding	DSG4
protein_coding	UBE3A
protein_coding	DLC1
protein_coding	CXCL14
protein_coding	PITHD1
protein_coding	PSD3
protein_coding	POSTN
protein_coding	SERINC4
protein_coding	C16orf98
protein_coding	AC074389.6
protein_coding	FAM162B
protein_coding	SPC24
protein_coding	SRSF1
protein_coding	AC004017.1
protein_coding	RRAGA
protein_coding	SGIP1
protein_coding	C20orf203
protein_coding	LONRF2
protein_coding	RIPK4

protein_coding	SUV420H2
protein_coding	C16orf95
protein_coding	RHBDL2
protein_coding	OLIG2
protein_coding	PRPS2
protein_coding	SRGN
protein_coding	HSD11B1L
protein_coding	OR2G3
protein_coding	HPR
protein_coding	USP33
protein_coding	NXPE4
protein_coding	SLC1A5
protein_coding	C12orf71
protein_coding	CTSC
protein_coding	SCN8A
protein_coding	HAGH
protein_coding	KCNG4
protein_coding	UBE2L3
protein_coding	PRDX5
protein_coding	AMD1
protein_coding	TEKT5
protein_coding	CSAG1
protein_coding	HS3ST6
protein_coding	SETD7
protein_coding	MYH10
protein_coding	ZBTB12
protein_coding	RGS7BP
protein_coding	CTXN3
protein_coding	LARP6
protein_coding	CENPE
protein_coding	C20orf27
protein_coding	CCDC136
protein_coding	OLFML1
protein_coding	AANAT
protein_coding	CLEC2L
protein_coding	OSBPL8
protein_coding	BPIFB6
protein_coding	WNT5B
protein_coding	IFNL2
protein_coding	CCBE1
protein_coding	MARVELD3
protein_coding	CLDN19
protein_coding	PCBP1
protein_coding	PCDHA4
protein_coding	OR2Z1
protein_coding	OR2T6
protein_coding	RAP1GDS1

protein_coding	RNF170
protein_coding	DYNC1I1
protein_coding	OLFR959
protein_coding	ZHX3
protein_coding	PLLP
protein_coding	RP11-298I3.5
protein_coding	HLA-DMB
protein_coding	CNNM1
protein_coding	CAMK1G
protein_coding	NKIRAS1
protein_coding	SORT1
protein_coding	GTDC1
protein_coding	LIN28A
protein_coding	TBC1D5
protein_coding	GYPB
protein_coding	CADPS
protein_coding	BIRC3
protein_coding	RBP5
protein_coding	MARVELD2
protein_coding	CFH
protein_coding	TRABD2A
protein_coding	CMTM5
protein_coding	COL2A1
protein_coding	SHISA2
protein_coding	TMEM185A
protein_coding	ENPP4
protein_coding	RUFY4
protein_coding	CACHD1
protein_coding	TMEM109
protein_coding	DCHS1
protein_coding	FLJ44635
protein_coding	SLC28A2
protein_coding	TRIM49
protein_coding	GRK1
protein_coding	SPDYE1
protein_coding	RPS27
protein_coding	C20orf78
protein_coding	NLRP9
protein_coding	CX3CR1
protein_coding	TEKT1
protein_coding	FGF1
protein_coding	PBX4
protein_coding	CCP110
protein_coding	CCDC47
protein_coding	OR2C3
protein_coding	CACYBP
protein_coding	MTHFD2

protein_coding	RP11-661C8.3
protein_coding	RP11-386G21.1
protein_coding	GADD45GIP1
protein_coding	BTBD10
protein_coding	AC008271.1
protein_coding	TNFRSF18
protein_coding	OR51I2
protein_coding	C10orf112
protein_coding	STAT5B
protein_coding	VSTM2A
protein_coding	TEKT4
protein_coding	FAM83E
protein_coding	TRIM23
protein_coding	ARL5A
protein_coding	ATP6V1F
protein_coding	KRTAP21-2
protein_coding	KCNG3
protein_coding	PSMB4
protein_coding	SCN9A
protein_coding	KCNS1
protein_coding	LRG1
protein_coding	ACSM1
protein_coding	SPATA17
protein_coding	FGF14
protein_coding	RPP30
protein_coding	TMEM47
protein_coding	SCNN1A
protein_coding	SOX13
protein_coding	SLC26A5
protein_coding	NDUFA8
protein_coding	C9orf135
protein_coding	HSPD1
protein_coding	PFN3
protein_coding	STXBP6
protein_coding	PTPN4
protein_coding	SLC5A1
protein_coding	AC008132.1
protein_coding	INPP1
protein_coding	ATP6V1B1
protein_coding	OR6Q1
protein_coding	TGFBR1
protein_coding	LRP4
protein_coding	MSI1
protein_coding	AC005493.1
protein_coding	PRR4
protein_coding	CYB5D1
protein_coding	FAM153C

protein_coding	FAM81B
protein_coding	LHX4
protein_coding	SPO11
protein_coding	RBM20
protein_coding	SGCG
protein_coding	KREMEN2
protein_coding	HIST1H4A
protein_coding	OR51F1
protein_coding	HEATR9
protein_coding	ATP10A
protein_coding	HDGFRP3
protein_coding	C20orf197
protein_coding	CRYBA2
protein_coding	ADH1C
protein_coding	ADCY9
protein_coding	DCTN1
protein_coding	SCN3B
protein_coding	AAGAB
protein_coding	ICOS
protein_coding	GPR22
protein_coding	SMARCA2
protein_coding	KRT75
protein_coding	MYOT
protein_coding	GNRHR
protein_coding	HABP2
protein_coding	ZWINT
protein_coding	IGFL3
protein_coding	MYCL
protein_coding	SGCE
protein_coding	TMSB4X
protein_coding	FBXO32
protein_coding	NKAIN1
protein_coding	TGIF1
protein_coding	COL11A2
protein_coding	SPOCK1
protein_coding	RASSF2
protein_coding	TOMM34
protein_coding	TSPAN7
protein_coding	CCNDBP1
protein_coding	TRAIP
protein_coding	WFDC10A
protein_coding	EYS
protein_coding	SNAP91
protein_coding	ZNF300
protein_coding	CGB7
protein_coding	SPHAR
protein_coding	RSPO1

protein_coding	RSPO3
protein_coding	GAGE10
protein_coding	H2BFWT
protein_coding	TTLL2
protein_coding	BEX5
protein_coding	DCAF8L1
protein_coding	S100P
protein_coding	ESRRG
protein_coding	AC135178.1
protein_coding	ATP6V0D2
protein_coding	METRN
protein_coding	PPP1R12A
protein_coding	CATSPER1
protein_coding	GNG8
protein_coding	HIST1H3E
protein_coding	OR14K1
protein_coding	NLRP12
protein_coding	ARHGEF9
protein_coding	SLC25A27
protein_coding	PRR5-ARHGAP8
protein_coding	SDHB
protein_coding	ISYNA1
protein_coding	EDF1
protein_coding	SBDS
protein_coding	NGRN
protein_coding	OR10P1
protein_coding	SLAMF1
protein_coding	FAM92B
protein_coding	FLOT1
protein_coding	IL17C
protein_coding	CGB1
protein_coding	PSMB7
protein_coding	SMARCD1
protein_coding	TIMELESS
protein_coding	TLR8
protein_coding	CTNND2
protein_coding	DDX3Y
protein_coding	GTF2IRD1
protein_coding	ANG
protein_coding	TGFBI
protein_coding	PI4KA
protein_coding	TEKT3
protein_coding	PLAU
protein_coding	BAI2
protein_coding	FGFR1OP2
protein_coding	NDUFC2
protein_coding	ESR2

protein_coding	TXNRD3NB
protein_coding	GIMAP6
protein_coding	USP3
protein_coding	ZNF280D
protein_coding	RP11-73M18.2
protein_coding	RHOD
protein_coding	THAP5
protein_coding	LOC339862
protein_coding	HIST1H2BJ
protein_coding	RAB18
protein_coding	SYT5
protein_coding	OCIAD1
protein_coding	DPP10
protein_coding	SLC2A10
protein_coding	SERP2
protein_coding	RANBP6
protein_coding	GABRR2
protein_coding	MRGPRX2
protein_coding	C15orf49
protein_coding	TMEM205
protein_coding	STAC3
protein_coding	MYL4
protein_coding	MGC20647
protein_coding	FUNDC1
protein_coding	AHI1
protein_coding	C16orf45
protein_coding	LRRC38
protein_coding	CIB2
protein_coding	PTTG1
protein_coding	MOSPD2
protein_coding	ATG2B
protein_coding	FAM154A
protein_coding	OR5AS1
protein_coding	DQ580909
protein_coding	HCLS1
protein_coding	SETX
protein_coding	ITFG1
protein_coding	ERVH48-1
protein_coding	ST8SIA5
protein_coding	TAS1R3
protein_coding	CGB
protein_coding	GAGE12F
protein_coding	ARPP21
protein_coding	COPRS
protein_coding	OR8B4
protein_coding	OR2A14
protein_coding	GSTO1

protein_coding	IL18BP
protein_coding	RPL19
protein_coding	DDO
protein_coding	C9orf171
protein_coding	XCL2
protein_coding	ZSCAN9
protein_coding	CKMT1B
protein_coding	HIST2H3C
protein_coding	CFAP45
protein_coding	APBB1
protein_coding	TMEM178A
protein_coding	OR11H12
protein_coding	IDH2
protein_coding	ANGPTL6
protein_coding	MAPRE2
protein_coding	TMEM165
protein_coding	SLC16A5
protein_coding	C11orf68
protein_coding	SMN2
protein_coding	SLC22A2
protein_coding	MYLPF
protein_coding	RCC1
protein_coding	AC105020.1
protein_coding	ABTB1
protein_coding	RP11-297N6.4
protein_coding	CR1
protein_coding	IFFO1
protein_coding	FAM178B
protein_coding	ABCG2
protein_coding	ERVMER34-1
protein_coding	MYF5
protein_coding	SAA2-SAA4
protein_coding	PRR19
protein_coding	ZNF154
protein_coding	PROKR2
protein_coding	ZNF730
protein_coding	ADRA1B
protein_coding	SLC44A1
protein_coding	MNDA
protein_coding	SYNPR
protein_coding	TLCD2
protein_coding	PRSS46
protein_coding	GTPBP6
protein_coding	UBL5
protein_coding	SLCO5A1
protein_coding	COX6C
protein_coding	MAT2B

protein_coding	C12orf74
protein_coding	CCT8L2
protein_coding	GMIP
protein_coding	HNRNPUL2
protein_coding	YTHDC1
protein_coding	SLITRK4
protein_coding	ZAN
protein_coding	FDXR
protein_coding	AC064874.1
protein_coding	HES3
protein_coding	MKS1
protein_coding	APOA1BP
protein_coding	KRAS
protein_coding	CIB1
protein_coding	MBOAT4
protein_coding	PEAK1
protein_coding	HEPACAM
protein_coding	GAL
protein_coding	PSG8
protein_coding	TERF2IP
protein_coding	ALCAM
protein_coding	IFNL3
protein_coding	PTCHD3
protein_coding	CTTNBP2
protein_coding	LY6K
protein_coding	CSMD1
protein_coding	KRTAP20-1
protein_coding	HNF4A
protein_coding	FAT4
protein_coding	NPM2
protein_coding	DNAJB14
protein_coding	PLIN4
protein_coding	TMEM238
protein_coding	BIN1
protein_coding	ITIH3
protein_coding	UBAC1
protein_coding	ASB4
protein_coding	PARP16
protein_coding	CCDC169
protein_coding	CD36
protein_coding	CYP2C19
protein_coding	SEPW1
protein_coding	ALPK2
protein_coding	ATP1A1
protein_coding	PTGS2
protein_coding	NUCB1
protein_coding	LZTFL1

protein_coding	CDH1
protein_coding	GTF3A
protein_coding	RAB11FIP4
protein_coding	GPR20
protein_coding	AC037199.1
protein_coding	PITRM1
protein_coding	MPC1
protein_coding	NT5C3B
protein_coding	ASGR1
protein_coding	CLMP
protein_coding	RAG1
protein_coding	SLC7A1
protein_coding	TSKU
protein_coding	GARS
protein_coding	OR51B6
protein_coding	LAMA1
protein_coding	FAM25A
protein_coding	LINC00696
protein_coding	CHEK2
protein_coding	CDH13
protein_coding	LAMP1
protein_coding	TMEM69
protein_coding	HPD
protein_coding	SPHKAP
protein_coding	OR8B2
protein_coding	C1D
protein_coding	MRGPRX4
protein_coding	TMEM66
protein_coding	ZBED2
protein_coding	SNCA
protein_coding	KCNT1
protein_coding	COX5A
protein_coding	LYZL2
protein_coding	CLIC6
protein_coding	KCNQ3
protein_coding	CPOX
protein_coding	STAG3
protein_coding	PTPRR
protein_coding	FAM171A1
protein_coding	IL1RN
protein_coding	DNMT3A
protein_coding	PTF1A
protein_coding	ADH1A
protein_coding	SSFA2
protein_coding	SQSTM1
protein_coding	RPLP2
protein_coding	TMEM14E

protein_coding	YWHAQ
protein_coding	IL1RL1
protein_coding	LIMCH1
protein_coding	GRB2
protein_coding	BMF
protein_coding	SERPINH1
protein_coding	AC127496.1
protein_coding	NUAK2
protein_coding	RAX
protein_coding	CCDC116
protein_coding	HENMT1
protein_coding	ASPN
protein_coding	SEMA7A
protein_coding	MARCH2
protein_coding	CHMP2A
protein_coding	WBP11
protein_coding	KIF2B
protein_coding	DSC2
protein_coding	ART1
protein_coding	SPINK14
protein_coding	C6orf58
protein_coding	HIST1H2BD
protein_coding	DSCAM
protein_coding	AL354898.1
protein_coding	DDN
protein_coding	SGPP2
protein_coding	SNX14
protein_coding	FAT2
protein_coding	RGS14
protein_coding	ENO3
protein_coding	GPR39
protein_coding	C11orf24
protein_coding	CTD-3193O13.9
protein_coding	OTX2
protein_coding	ELOVL4
protein_coding	CREBL2
protein_coding	KCNQ5
protein_coding	LMO3
protein_coding	TCEB3CL2
protein_coding	MUL1
protein_coding	AK096230
protein_coding	LRP1B
protein_coding	S100A10
protein_coding	LIN9
protein_coding	REEP4
protein_coding	MCM2
protein_coding	TOLLIP

protein_coding	PRPF8
protein_coding	FZD10
protein_coding	SCG2
protein_coding	PHF21B
protein_coding	SH2D3A
protein_coding	IFI27L2
protein_coding	ETV4
protein_coding	TJP2
protein_coding	ARHGAP22
protein_coding	ELL2
protein_coding	NIPA1
protein_coding	GORASP2
protein_coding	CELA2B
protein_coding	HSP90B1
protein_coding	RNF128
protein_coding	BCAN
protein_coding	OR5L2
protein_coding	PLA2G4B
protein_coding	DBH
protein_coding	DENND5B
protein_coding	SRGAP3
protein_coding	LMX1A
protein_coding	TP53TG5
protein_coding	KRT24
protein_coding	SLC45A3
protein_coding	DPP7
protein_coding	RAPGEF1
protein_coding	CDC20
protein_coding	TEX9
protein_coding	CD276
protein_coding	NDUFB9
protein_coding	HTATIP2
protein_coding	LYST
protein_coding	CACNA1C
protein_coding	DDRGK1
protein_coding	BRINP2
protein_coding	NDUFA3
protein_coding	ATG2A
protein_coding	TMED7-TICAM2
protein_coding	RPL37A
protein_coding	SMLR1
protein_coding	TTC39B
protein_coding	PC
protein_coding	AKT3
protein_coding	OR11H1
protein_coding	TJP3
protein_coding	OVCH1

protein_coding	RGS11
protein_coding	FAM172A
protein_coding	CLDND1
protein_coding	MTDH
protein_coding	OR2T1
protein_coding	TBK1
protein_coding	INPP4B
protein_coding	AC107021.1
protein_coding	GAGE2B
protein_coding	SERPINA11
protein_coding	PRDX2
protein_coding	TLE3
protein_coding	TFEB
protein_coding	RASA4
protein_coding	GAGE4
protein_coding	BEND4
protein_coding	TMEM132D
protein_coding	TBATA
protein_coding	RP11-315D16.2
protein_coding	RIMBP3
protein_coding	SLC25A28
protein_coding	CBX8
protein_coding	RP11-162A12.2
protein_coding	HNRNPDL
protein_coding	UGT2B11
protein_coding	MARS
protein_coding	TUFM
protein_coding	SCUBE3
protein_coding	ALOX12B
protein_coding	NPR1
protein_coding	TCF3
protein_coding	MALRD1
protein_coding	SPRED1
protein_coding	TBCB
protein_coding	MTRNR2L10
protein_coding	TUBG2
protein_coding	AC145676.2
protein_coding	EPOR
protein_coding	SMR3A
protein_coding	HIST3H2A
protein_coding	SSU72
protein_coding	FAM157A
protein_coding	CDK14
protein_coding	ARL8B
protein_coding	MYOM2
protein_coding	CLSTN1
protein_coding	C1orf186

protein_coding	BCL6
protein_coding	NOMO3
protein_coding	C18orf32
protein_coding	DHH
protein_coding	SLC16A7
protein_coding	DGKD
protein_coding	MTERFD2
protein_coding	ACTR10
protein_coding	CACNG4
protein_coding	HSD17B4
protein_coding	ARHGAP5
protein_coding	HERC6
protein_coding	SIRPB1
protein_coding	UBE2O
protein_coding	LRRTM4
protein_coding	SFTPA1
protein_coding	C22orf39
protein_coding	CCDC19
protein_coding	ADAMTS9
protein_coding	SLC19A3
protein_coding	IL21R
protein_coding	AL353354.1
protein_coding	SLC5A8
protein_coding	C11orf82
protein_coding	WHSC1
protein_coding	UTY
protein_coding	KLK13
protein_coding	MOB2
protein_coding	DDX24
protein_coding	GLS
protein_coding	BBS2
protein_coding	HOXD8
protein_coding	GP2
protein_coding	FNDC1
protein_coding	TTC1
protein_coding	HFM1
protein_coding	SIX3
protein_coding	CNPY1
protein_coding	ARHGAP11A
protein_coding	FEZ2
protein_coding	IGSF11
protein_coding	LTB4R
protein_coding	LTN1
protein_coding	TAX1BP1
protein_coding	FAM175A
protein_coding	OSTF1
protein_coding	UHRF1

protein_coding	KRTAP4-2
protein_coding	PLA2G10
protein_coding	ALX1
protein_coding	PTGES2
protein_coding	PRM2
protein_coding	ZNF445
protein_coding	TSR2
protein_coding	TM9SF3
protein_coding	FGFBP3
protein_coding	CRX
protein_coding	BMX
protein_coding	FXYD7
protein_coding	CLUL1
protein_coding	GAP43
protein_coding	ZNF311
protein_coding	RPS10
protein_coding	MGST1
protein_coding	NOMO1
protein_coding	UBE2F
protein_coding	CCDC18
protein_coding	FABP3
protein_coding	GPS2
protein_coding	OR7G2
protein_coding	PAPOLA
protein_coding	PRR29
protein_coding	ZNF345
protein_coding	HLA-A
protein_coding	KRT76
protein_coding	PLAC1
protein_coding	AC139100.2
protein_coding	HNF4G
protein_coding	FOXO3
protein_coding	LOC146880
protein_coding	HSD17B1
protein_coding	LNX1
protein_coding	ADPRH
protein_coding	ORM2
protein_coding	AL445989.1
protein_coding	CCDC182
protein_coding	IQGAP3
protein_coding	IMMP1L
protein_coding	FGF16
protein_coding	BLOC1S3
protein_coding	RFPL4AL1
protein_coding	LFNG
protein_coding	CDH9
protein_coding	FAM3B

protein_coding	ASPHD1
protein_coding	LOC100130357
protein_coding	HNRNPC
protein_coding	PERM1
protein_coding	OTUB2
protein_coding	GABRA5
protein_coding	DKK3
protein_coding	FAM73A
protein_coding	GABRB1
protein_coding	BEX2
protein_coding	ARFIP2
protein_coding	FAM227A
protein_coding	SNRNP25
protein_coding	TMEM206
protein_coding	C11orf16
protein_coding	SCUBE2
protein_coding	ADCY2
protein_coding	OR1F1
protein_coding	BEST1
protein_coding	RAC1
protein_coding	EGLN3
protein_coding	RNF103
protein_coding	PPFIA2
protein_coding	ELK1
protein_coding	SNTA1
protein_coding	RSPH9
protein_coding	GINS4
protein_coding	GDPD3
protein_coding	S1PR4
protein_coding	NBEAL2
protein_coding	U2AF1
protein_coding	C7orf33
protein_coding	ATP11C
protein_coding	TNFSF8
protein_coding	THRB
protein_coding	KRBOX4
protein_coding	KCNMA1
protein_coding	ATP5L2
protein_coding	ARPC3
protein_coding	FAM60A
protein_coding	FMN1
protein_coding	LRRC20
protein_coding	UBQLN1
protein_coding	SLC6A17
protein_coding	CSNK2A1
protein_coding	DHX58
protein_coding	C22orf42

protein_coding	AZGP1
protein_coding	KIZ
protein_coding	THEM6
protein_coding	ENOPH1
protein_coding	MAGEA5
protein_coding	DNAAF3
protein_coding	GABBR2
protein_coding	KIR3DL2
protein_coding	MRPL41
protein_coding	PREX1
protein_coding	TBX22
protein_coding	ART4
protein_coding	RAB26
protein_coding	DYNLRB1
protein_coding	OC90
protein_coding	AGAP2
protein_coding	TRIP13
protein_coding	ALAS2
protein_coding	SLC7A10
protein_coding	ZNF581
protein_coding	PLEKHS1
protein_coding	SHROOM4
protein_coding	C6orf201
protein_coding	LOC100506422
protein_coding	CBR4
protein_coding	OR2B2
protein_coding	PTPRB
protein_coding	KLHDC8B
protein_coding	MPL
protein_coding	RPS3A
protein_coding	OR12D3
protein_coding	GSX1
protein_coding	ZNF788
protein_coding	STRN
protein_coding	SEC14L4
protein_coding	FRA10AC1
protein_coding	DSTN
protein_coding	MICU3
protein_coding	RANBP2
protein_coding	FYB
protein_coding	KHDC1
protein_coding	AX748291
protein_coding	ZMYND10
protein_coding	KRT81
protein_coding	PTPN20B
protein_coding	COL4A3BP
protein_coding	WTIP

protein_coding	NDUFA6
protein_coding	KCNA2
protein_coding	FCAR
protein_coding	ZNF69
protein_coding	LRPAP1
protein_coding	PFDN2
protein_coding	EID1
protein_coding	CH17-360D5.1
protein_coding	GYLTL1B
protein_coding	MAGEF1
protein_coding	HIST2H2AC
protein_coding	AL136531.1
protein_coding	LOC149373
protein_coding	ESPL1
protein_coding	TMEM92
protein_coding	AAR2
protein_coding	TBC1D9B
protein_coding	H3F3C
protein_coding	SH3BGRL2
protein_coding	DCST2
protein_coding	CAPN12
protein_coding	MKL1
protein_coding	MSANTD4
protein_coding	LMNTD2
protein_coding	EIF4G2
protein_coding	TMEM132B
protein_coding	TSPEAR
protein_coding	NEIL3
protein_coding	LCE1E
protein_coding	OPN3
protein_coding	ADI1
protein_coding	GAS6
protein_coding	PRICKLE3
protein_coding	VTI1B
protein_coding	DRICH1
protein_coding	HIST1H2BL
protein_coding	CIZ1
protein_coding	MRVI1
protein_coding	AMY1A
protein_coding	ISOC2
protein_coding	F11R
protein_coding	AC106873.4
protein_coding	SIM1
protein_coding	KBTBD3
protein_coding	CTB-96E2.2
protein_coding	KIF1B
protein_coding	CTC-429P9.4

protein_coding	CHST12
protein_coding	SULT1C2
protein_coding	GPR6
protein_coding	SSB
protein_coding	AC079341.1
protein_coding	ZBTB7A
protein_coding	AKTIP
protein_coding	ALDOB
protein_coding	PHOSPHO2-KLHL23
protein_coding	ATP6V1G2
protein_coding	IMMT
protein_coding	MSH5
protein_coding	SART1
protein_coding	SYVN1
protein_coding	INCA1
protein_coding	ZNF559-ZNF177
protein_coding	PNLIPRP1
protein_coding	CCR4
protein_coding	MMP15
protein_coding	LOC728637
protein_coding	OR52I1
protein_coding	FBXL5
protein_coding	CCDC88A
protein_coding	ZNF114
protein_coding	SKAP2
protein_coding	MBLAC2
protein_coding	FLVCR2
protein_coding	FAM47E
protein_coding	SRP14
protein_coding	KRTAP2-4
protein_coding	ADO
protein_coding	RP11-89K11.1
protein_coding	RPS28
protein_coding	C14orf2
protein_coding	TTPA
protein_coding	MRPS25
protein_coding	DKFZP434O1614
protein_coding	DEFB105A
protein_coding	CLDN5
protein_coding	EML2
protein_coding	GFRA4
protein_coding	NOVA2
protein_coding	LYZL1
protein_coding	DDX5
protein_coding	GLI1
protein_coding	FAM213A
protein_coding	CWC15

protein_coding	STAU2
protein_coding	AX747517
protein_coding	HGD
protein_coding	TVP23C-CDRT4
protein_coding	NUDT4
protein_coding	KIAA0368
protein_coding	ERBB2IP
protein_coding	ZEB2
protein_coding	PRRC1
protein_coding	AC011551.3
protein_coding	C19orf31
protein_coding	SAMD11
protein_coding	DDX25
protein_coding	BAIAP2L1
protein_coding	RASAL1
protein_coding	NDUFA1
protein_coding	NPR3
protein_coding	CLTB
protein_coding	BLOC1S2
protein_coding	VAMP4
protein_coding	FBLL1
protein_coding	ECHDC1
protein_coding	KCNK6
protein_coding	MSH4
protein_coding	THBS4
protein_coding	ATP13A4
protein_coding	BV13S1J2.7
protein_coding	PRR25
protein_coding	DDX1
protein_coding	ETS2
protein_coding	SOWAHB
protein_coding	HLF
protein_coding	CERS2
protein_coding	AK297683
protein_coding	FRYL
protein_coding	FANCC
protein_coding	CD248
protein_coding	PER2
protein_coding	CA2
protein_coding	CTC-786C10.1
protein_coding	FOXJ3
protein_coding	PCDH10
protein_coding	EPB41L1
protein_coding	MN1
protein_coding	OLA1
protein_coding	NCMAP
protein_coding	CBX4

protein_coding	FGL2
protein_coding	LYRM5
protein_coding	MEGF6
protein_coding	RP11-599B13.6
protein_coding	ZNF652
protein_coding	AL117190.2
protein_coding	CCDC63
protein_coding	MORN4
protein_coding	VCX3B
protein_coding	MS4A6E
protein_coding	SGPP1
protein_coding	PRR23B
protein_coding	SEC11C
protein_coding	ARHGAP18
protein_coding	LRRC71
protein_coding	DLX1
protein_coding	SLC19A1
protein_coding	HSD17B12
protein_coding	GALNT15
protein_coding	FBXO39
protein_coding	CPQ
protein_coding	CACNA1B
protein_coding	DLD
protein_coding	LMO1
protein_coding	CCDC62
protein_coding	CMTM3
protein_coding	METAP1D
protein_coding	COL5A1
protein_coding	PPP1R12C
protein_coding	POLE2
protein_coding	UHMK1
protein_coding	GPR157
protein_coding	LRFN2
protein_coding	DNAJC6
protein_coding	PADI2
protein_coding	FAM21C
protein_coding	CXorf30
protein_coding	ADAM8
protein_coding	GTF3C6
protein_coding	KIRREL2
protein_coding	PHF10
protein_coding	RNF113B
protein_coding	MOS
protein_coding	AQPEP
protein_coding	NOL7
protein_coding	IER5
protein_coding	NAPG

protein_coding	COPS6
protein_coding	SIK3
protein_coding	C10orf111
protein_coding	GNPTG
protein_coding	PLK4
protein_coding	IZUMO2
protein_coding	EPPIN
protein_coding	PSMC1
protein_coding	PCDHA2
protein_coding	SSPN
protein_coding	SNX2
protein_coding	NCF1
protein_coding	SYBU
protein_coding	EPPK1
protein_coding	TMEM14A
protein_coding	TRIM61
protein_coding	SERPINB10
protein_coding	CA6
protein_coding	AGAP8
protein_coding	DLGAP3
protein_coding	CLEC18A
protein_coding	HECW2
protein_coding	KLHL22
protein_coding	TOMM7
protein_coding	RNF186
protein_coding	NPTXR
protein_coding	ARMCX2
protein_coding	SERPINB12
protein_coding	RAPGEF2
protein_coding	C7orf31
protein_coding	SLC22A7
protein_coding	APAF1
protein_coding	SMG7
protein_coding	TMED6
protein_coding	PRB2
protein_coding	TMEM30A
protein_coding	NDNL2
protein_coding	ZEB1
protein_coding	ENPP5
protein_coding	ASRGL1
protein_coding	HCFC1R1
protein_coding	KLF5
protein_coding	HARS
protein_coding	GAGE8
protein_coding	ANKRD40
protein_coding	THRA
protein_coding	CTD-2330K9.3

protein_coding	NKG2-E
protein_coding	RAPSN
protein_coding	CRYBB1
protein_coding	KDELR3
protein_coding	HINT1
protein_coding	CCSAP
protein_coding	KCNJ13
protein_coding	ZNF606
protein_coding	FAM63B
protein_coding	OASL
protein_coding	AHSA1
protein_coding	PHF15
protein_coding	CHML
protein_coding	TPSG1
protein_coding	RBM26
protein_coding	ARHGEF3
protein_coding	CGB5
protein_coding	KIF5B
protein_coding	TMTC4
protein_coding	EEF1E1
protein_coding	FBXW5
protein_coding	PJA2
protein_coding	VN1R2
protein_coding	MT3
protein_coding	TLR5
protein_coding	HIGD1B
protein_coding	TSPAN6
protein_coding	NTSR1
protein_coding	FCHSD1
protein_coding	C3orf35
protein_coding	SLC18B1
protein_coding	FANCD2
protein_coding	KRTAP3-2
protein_coding	BC131755
protein_coding	BTBD16
protein_coding	SLC35G2
protein_coding	HOXC13
protein_coding	ETV1
protein_coding	CDH12
protein_coding	EXOC6B
protein_coding	ARL2
protein_coding	JPH3
protein_coding	AC015989.2
protein_coding	SLC25A2
protein_coding	XPO6
protein_coding	MYOCD
protein_coding	MVK

protein_coding	DDIT3
protein_coding	FAM115C
protein_coding	LPGAT1
protein_coding	SFPQ
protein_coding	NUDT9
protein_coding	CALCR
protein_coding	TNFSF13
protein_coding	CYP17A1
protein_coding	PDE4B
protein_coding	NDST4
protein_coding	TTC26
protein_coding	OR10A6
protein_coding	GADD45G
protein_coding	CAD
protein_coding	AS3MT
protein_coding	CCDC150
protein_coding	HTR5A-AS1
protein_coding	FBXO31
protein_coding	RPL13
protein_coding	C12orf36
protein_coding	DLG5
protein_coding	CDX2
protein_coding	SDF4
protein_coding	ANKRD18B
protein_coding	RPL14
protein_coding	WFDC1
protein_coding	IL36G
protein_coding	MRPL19
protein_coding	ACADM
protein_coding	PIF1
protein_coding	PLA2G4F
protein_coding	ASAH1
protein_coding	INSM2
protein_coding	SLC38A4
protein_coding	KRT33A
protein_coding	XKRX
protein_coding	CCDC51
protein_coding	MMP26
protein_coding	CLEC19A
protein_coding	GNRH2
protein_coding	RASSF4
protein_coding	FAAH2
protein_coding	ZFP42
protein_coding	FAM71D
protein_coding	C20orf195
protein_coding	SERBP1
protein_coding	GNB5

protein_coding	NAT2
protein_coding	ARAP3
protein_coding	KIAA0196
protein_coding	STRAP
protein_coding	LUZP1
protein_coding	NFYB
protein_coding	MYO1A
protein_coding	MEGF11
protein_coding	PSMD6
protein_coding	NDUFA13
protein_coding	GLYATL2
protein_coding	SCP2
protein_coding	ZNF229
protein_coding	DPP4
protein_coding	ODC1
protein_coding	KRT84
protein_coding	TTC23
protein_coding	ATP10B
protein_coding	CYP2A13
protein_coding	DUX4L7
protein_coding	ARHGAP32
protein_coding	CFAP126
protein_coding	RIMS1
protein_coding	KIAA0430
protein_coding	C19orf48
protein_coding	DNM1L
protein_coding	DUSP7
protein_coding	TMEM130
protein_coding	CALY
protein_coding	FAM231C
protein_coding	RNF220
protein_coding	CCDC43
protein_coding	DENND4A
protein_coding	ARNTL
protein_coding	SLC24A4
protein_coding	ARID3C
protein_coding	CYSLTR2
protein_coding	PRAMEF20
protein_coding	FAM162A
protein_coding	HAND1
protein_coding	THBS1
protein_coding	PHYH
protein_coding	KIAA0513
protein_coding	TMEM214
protein_coding	PLK2
protein_coding	ANKRD63
protein_coding	MLIP

protein_coding	KIAA1377
protein_coding	UBLCP1
protein_coding	RPRML
protein_coding	PPP1R3B
protein_coding	NBPF3
protein_coding	HIST1H2BM
protein_coding	NFS1
protein_coding	RPS19
protein_coding	MXRA8
protein_coding	AC091801.1
protein_coding	OR9G1
protein_coding	LOC100130880
protein_coding	OPN4
protein_coding	NDFIP2
protein_coding	ANXA2
protein_coding	TUBA4A
protein_coding	USMG5
protein_coding	RD3
protein_coding	TRIM69
protein_coding	TMEM221
protein_coding	PDE8A
protein_coding	TNC
protein_coding	CMPK2
protein_coding	ZNF260
protein_coding	PFKM
protein_coding	MRC2
protein_coding	KRT17
protein_coding	FXYD6-FXYD2
protein_coding	SLC34A2
protein_coding	ALX4
protein_coding	IL6R
protein_coding	OR2M5
protein_coding	ZKSCAN3
protein_coding	NPTX2
protein_coding	CYB5RL
protein_coding	SLC35F2
protein_coding	CHIC1
protein_coding	NUDT21
protein_coding	UCHL5
protein_coding	EPYC
protein_coding	CST7
protein_coding	EXPH5
protein_coding	RNF43
protein_coding	UNC50
protein_coding	PLA2G3
protein_coding	GFRAL
protein_coding	APOC3

protein_coding	PRR20E
protein_coding	KRTCAP2
protein_coding	BBS7
protein_coding	POU5F1B
protein_coding	PGBD5
protein_coding	CD47
protein_coding	HELB
protein_coding	ACAP1
protein_coding	CCDC65
protein_coding	RNPEPL1
protein_coding	ISM1
protein_coding	DHRSX
protein_coding	FLJ00104
protein_coding	WNK4
protein_coding	BABAM1
protein_coding	SYNPO
protein_coding	SYT17
protein_coding	CHM
protein_coding	AX747757
protein_coding	TM4SF2
protein_coding	TAS2R5
protein_coding	AAMP
protein_coding	GPAA1
protein_coding	IL19
protein_coding	FLJ00388
protein_coding	NMS
protein_coding	SYNE1
protein_coding	ANPEP
protein_coding	NUTM1
protein_coding	NPIPB15
protein_coding	AX747161
protein_coding	CCDC81
protein_coding	TBC1D9
protein_coding	TDO2
protein_coding	PTPRD
protein_coding	AL591684.1
protein_coding	C14orf39
protein_coding	CAMK1D
protein_coding	PRAMEF4
protein_coding	ANKRD18A
protein_coding	ANXA6
protein_coding	ATR
protein_coding	AC007952.6
protein_coding	SLC39A10
protein_coding	DYNLL2
protein_coding	PRKAG2
protein_coding	BOLA3

protein_coding	CRBN
protein_coding	TM6SF1
protein_coding	ARL17A
protein_coding	C19orf26
protein_coding	ETV3L
protein_coding	C10orf107
protein_coding	PPM1B
protein_coding	SMAP2
protein_coding	TNNT3
protein_coding	DGKZ
protein_coding	ZNF688
protein_coding	TNRC6C
protein_coding	HCRT
protein_coding	KCNRG
protein_coding	KRT18
protein_coding	CXADR
protein_coding	FANCI
protein_coding	LYPD3
protein_coding	HSPE1
protein_coding	OR1A1
protein_coding	CALB1
protein_coding	LOC391003
protein_coding	ANKH
protein_coding	NDUFB1
protein_coding	WASF1
protein_coding	SIAH3
protein_coding	CLTA
protein_coding	FXYD1
protein_coding	NRM
protein_coding	NDUFB7
protein_coding	ECHS1
protein_coding	VTN
protein_coding	CUX2
protein_coding	CDT1
protein_coding	GRM5
protein_coding	SPTBN4
protein_coding	AK123872
protein_coding	TACC3
protein_coding	TMPRSS9
protein_coding	PLOD3
protein_coding	DUT
protein_coding	ST13
protein_coding	MED18
protein_coding	CSRP2
protein_coding	AC126614.1
protein_coding	EIF5
protein_coding	TMEM143

protein_coding	COX11
protein_coding	OXLD1
protein_coding	FKBP3
protein_coding	HEBP1
protein_coding	C1orf115
protein_coding	PPP3CB
protein_coding	HS3ST4
protein_coding	ZC3H3
protein_coding	CORO6
protein_coding	ZNF787
protein_coding	DHRS7
protein_coding	GLRX5
protein_coding	BZRAP1
protein_coding	UBE2D4
protein_coding	SIPA1L1
protein_coding	TDP2
protein_coding	SCRN1
protein_coding	GGT6
protein_coding	HBA1
protein_coding	SRGAP1
protein_coding	GABRP
protein_coding	PKP4
protein_coding	LRRC58
protein_coding	FAAP24
protein_coding	STAMBPL1
protein_coding	ECT2
protein_coding	MTL5
protein_coding	HBE1
protein_coding	MZT2A
protein_coding	AP001362.1
protein_coding	FOXD1
protein_coding	NXPH1
protein_coding	C2CD4A
protein_coding	CAPN8
protein_coding	CDC42BPB
protein_coding	RIT2
protein_coding	TCAF1
protein_coding	DSP
protein_coding	WBP4
protein_coding	NPIPB8
protein_coding	SCFD1
protein_coding	TAF7L
protein_coding	QKI
protein_coding	ZPLD1
protein_coding	PIH1
protein_coding	HRASLS2
protein_coding	ZNF664

protein_coding	PDE1A
protein_coding	GPR143
protein_coding	ZNRF4
protein_coding	SULT1C3
protein_coding	LOC101929983
protein_coding	CSH1
protein_coding	PCDHGB3
protein_coding	ERBB3
protein_coding	GCK
protein_coding	TMEM232
protein_coding	RP11-166B2.1
protein_coding	L3MBTL4
protein_coding	TRIM44
protein_coding	C6orf15
protein_coding	JAZF1
protein_coding	ADGRF5
protein_coding	C19orf82
protein_coding	ZNF843
protein_coding	ICAM1
protein_coding	SEMA4C
protein_coding	DSCC1
protein_coding	CARS
protein_coding	RAB3B
protein_coding	SPP2
protein_coding	PTRH2
protein_coding	CYP3A7-CYP3AP1
protein_coding	MT-ND6
protein_coding	ARG1
protein_coding	PLA2G2F
protein_coding	HLA-F
protein_coding	HINT2
protein_coding	C2orf88
protein_coding	MT-CYB
protein_coding	DQ580039
protein_coding	SLC39A8
protein_coding	LXN
protein_coding	ATP13A2
protein_coding	CHPT1
protein_coding	UBR3
protein_coding	PIGY
protein_coding	GAL3ST4
protein_coding	SDK2
protein_coding	DPRX
protein_coding	ADAM22
protein_coding	SRI
protein_coding	ABCC12
protein_coding	CERCAM

protein_coding	OPA1
protein_coding	CNN3
protein_coding	UBXN1
protein_coding	ZNF679
protein_coding	IDI2
protein_coding	C1orf168
protein_coding	HECTD3
protein_coding	OR14A16
protein_coding	KLHL1
protein_coding	SLC43A1
protein_coding	TPO
protein_coding	C16orf92
protein_coding	TMEM39A
protein_coding	DPH1
protein_coding	PTCD1
protein_coding	MYBPH
protein_coding	SGCZ
protein_coding	CCDC134
protein_coding	MOGAT2
protein_coding	RP11-371E8.4
protein_coding	SAT1
protein_coding	USP12
protein_coding	PDCL2
protein_coding	BRMS1L
protein_coding	RLBP1
protein_coding	SCNM1
protein_coding	CNTNAP3B
protein_coding	C10orf99
protein_coding	KIAA1279
protein_coding	OTC
protein_coding	DOCK3
protein_coding	AGAP9
protein_coding	LMO7DN
protein_coding	MTMR12
protein_coding	EPHB2
protein_coding	AL138815.1
protein_coding	RND1
protein_coding	KRT8
protein_coding	FAM49B
protein_coding	IFNL4
protein_coding	FEM1A
protein_coding	HCN3
protein_coding	TMEM70
protein_coding	SH3BP4
protein_coding	SNX3
protein_coding	APLP2
protein_coding	SLC6A18

protein_coding	IFIT3
protein_coding	GNG5
protein_coding	UGT2B7
protein_coding	TMEM44
protein_coding	RPS2
protein_coding	C1orf63
protein_coding	FCER1G
protein_coding	TPD52L1
protein_coding	CD207
protein_coding	RTN4RL1
protein_coding	CDK4
protein_coding	PBX3
protein_coding	HP
protein_coding	CHMP5
protein_coding	TBC1D4
protein_coding	GATC
protein_coding	ZNF540
protein_coding	FANCE
protein_coding	PDPK1
protein_coding	MED17
protein_coding	APBA2
protein_coding	LOC388849
protein_coding	FOXD3
protein_coding	OR1E2
protein_coding	GLRX2
protein_coding	MICU2
protein_coding	CAPRIN2
protein_coding	APOA5
protein_coding	COL19A1
protein_coding	KIF21B
protein_coding	SORBS1
protein_coding	AK026502
protein_coding	C16orf82
protein_coding	PRKCE
protein_coding	CASD1
protein_coding	CRYGS
protein_coding	MXRA7
protein_coding	WAC
protein_coding	CDRT15
protein_coding	XCL1
protein_coding	RALYL
protein_coding	PIP5K1B
protein_coding	AV4S1
protein_coding	SRP9
protein_coding	PAG1
protein_coding	ZNF785
protein_coding	AC121757.1

protein_coding	CREB3L4
protein_coding	OOSP1
protein_coding	MAP2
protein_coding	CDC42SE2
protein_coding	RILPL1
protein_coding	BSCL2
protein_coding	METTL21B
protein_coding	PP13004
protein_coding	MAPK10
protein_coding	PPT1
protein_coding	MAP3K19
protein_coding	CSGALNACT1
protein_coding	TRIM34
protein_coding	THRSP
protein_coding	KRTAP17-1
protein_coding	DRD5
protein_coding	PSMD14
protein_coding	ZNF814
protein_coding	PDE4DIP
protein_coding	CA12
protein_coding	AK096898
protein_coding	CYP2F1
protein_coding	MAN1C1
protein_coding	CTSD
protein_coding	MISP
protein_coding	POTEM
protein_coding	TCEB2
protein_coding	PTPDC1
protein_coding	TOM1L2
protein_coding	ALOX15
protein_coding	TBL1XR1
protein_coding	FLJ20373
protein_coding	FAM132A
protein_coding	TEAD3
protein_coding	CFAP53
protein_coding	IER3IP1
protein_coding	MARCO
protein_coding	CCDC82
protein_coding	CDC42BPG
protein_coding	FAIM
protein_coding	LOXL3
protein_coding	GSTT2
protein_coding	SCAMP5
protein_coding	TFEC
protein_coding	PPAP2A
protein_coding	C15ORF31
protein_coding	CNTN3

protein_coding	C6orf222
protein_coding	PPBP
protein_coding	BC007896
protein_coding	C12orf76
protein_coding	PRIM2
protein_coding	AGER
protein_coding	HIST1H2AK
protein_coding	MED10
protein_coding	NMRAL1
protein_coding	PRKCG
protein_coding	MKNK2
protein_coding	KLHDC2
protein_coding	HHIPL2
protein_coding	SP6
protein_coding	SYTL3
protein_coding	NXF5
protein_coding	IGFBPL1
protein_coding	CYB5B
protein_coding	FGF2
protein_coding	MAPK9
protein_coding	PHF5A
protein_coding	BORA
protein_coding	NUDC
protein_coding	TRIM37
protein_coding	CNRIP1
protein_coding	MYPOP
protein_coding	ASB10
protein_coding	TOR1B
protein_coding	STXBP2
protein_coding	CCNE2
protein_coding	DNAJA1
protein_coding	USP32
protein_coding	DAZ2
protein_coding	THSD7B
protein_coding	CDRT1
protein_coding	C19orf57
protein_coding	AC007382.1
protein_coding	SMIM1
protein_coding	ATP5C1
protein_coding	LBP
protein_coding	LRRN4CL
protein_coding	GALM
protein_coding	ADGRF4
protein_coding	PPP1R1A
protein_coding	C8orf49
protein_coding	TIMMDC1
protein_coding	EIF2AK2

protein_coding	PCDHGA11
protein_coding	PCSK9
protein_coding	GRINA
protein_coding	SPACA5B
protein_coding	C6orf163
protein_coding	PTGDR2
protein_coding	TAP2
protein_coding	MDGA2
protein_coding	DESI1
protein_coding	AL591025.1
protein_coding	RASL10B
protein_coding	ISM2
protein_coding	GNA14
protein_coding	SLC13A1
protein_coding	VANGL1
protein_coding	PNMA3
protein_coding	LRRC72
protein_coding	SHF
protein_coding	LOC100144595
protein_coding	DEFA4
protein_coding	CLDN25
protein_coding	ELOVL3
protein_coding	LGALS9B
protein_coding	CISD2
protein_coding	TIMM23
protein_coding	LY75-CD302
protein_coding	NDUFAB1
protein_coding	SPARC
protein_coding	ELOVL1
protein_coding	STXBP5
protein_coding	HCAR3
protein_coding	CASP6
protein_coding	EPS15
protein_coding	RNF152
protein_coding	WDFY3
protein_coding	AVPR1B
protein_coding	LOC101927572
protein_coding	HYAL4
protein_coding	URGCP-MRPS24
protein_coding	GFOD1
protein_coding	PPAPDC1B
protein_coding	KRTAP10-8
protein_coding	AMZ2
protein_coding	ARPC2
protein_coding	DND1
protein_coding	BC038455
protein_coding	PDPN

protein_coding	ITLN1
protein_coding	SETSIP
protein_coding	LONP2
protein_coding	RAB27B
protein_coding	MAP4K2
protein_coding	GPR132
protein_coding	WISP2
protein_coding	EGFL6
protein_coding	ENG
protein_coding	CCDC149
protein_coding	CYB5R2
protein_coding	SH3BP2
protein_coding	EIF3K
protein_coding	EPB41L4A-AS2
protein_coding	SMPX
protein_coding	POU6F1
protein_coding	ZIC4
protein_coding	SHANK1
protein_coding	KMT2B
protein_coding	KLK7
protein_coding	HK2
protein_coding	PON2
protein_coding	LCE2B
protein_coding	PMM2
protein_coding	RAB39A
protein_coding	TRAPPC8
protein_coding	C10orf126
protein_coding	SSUH2
protein_coding	H2AFY2
protein_coding	TARSL2
protein_coding	ZNF674
protein_coding	FUT5
protein_coding	SH2D4B
protein_coding	NAALADL1
protein_coding	DCAF7
protein_coding	RABGGTB
protein_coding	RPL30
protein_coding	AX746654
protein_coding	RBP7
protein_coding	IL23A
protein_coding	MTSS1L
protein_coding	ITPR3
protein_coding	RGPD5
protein_coding	IBSP
protein_coding	DLEU7
protein_coding	ZDHHC11B
protein_coding	NT5DC2

protein_coding	BSDC1
protein_coding	DENND5A
protein_coding	GLB1L2
protein_coding	TMOD4
protein_coding	C5orf67
protein_coding	RBM47
protein_coding	KRTAP1-5
protein_coding	KRT6B
protein_coding	RILPL2
protein_coding	EPS8L2
protein_coding	AL355490.1
protein_coding	TCEA2
protein_coding	LUZP6
protein_coding	C10orf40
protein_coding	ASH1L
protein_coding	PABPC4L
protein_coding	PTGES3
protein_coding	ACTRT1
protein_coding	ISCU
protein_coding	USP53
protein_coding	SLC16A3
protein_coding	GLYATL1
protein_coding	ABCA2
protein_coding	C2orf73
protein_coding	NBEA
protein_coding	PSME2
protein_coding	SLC25A5
protein_coding	RBBP7
protein_coding	SLC16A8
protein_coding	VPS41
protein_coding	GNAQ
protein_coding	CREBRF
protein_coding	PRPF19
protein_coding	OR2T34
protein_coding	SF3B5
protein_coding	CTAGE6
protein_coding	CASC5
protein_coding	NXPE3
protein_coding	DPYSL2
protein_coding	ABHD12
protein_coding	ITGAV
protein_coding	TRIM58
protein_coding	BBS10
protein_coding	TRAF1
protein_coding	RP1L1
protein_coding	OVCH2
protein_coding	IAH1

protein_coding	ICA1
protein_coding	CYP51A1
protein_coding	ZNF665
protein_coding	TWF2
protein_coding	TLN2
protein_coding	ADSS
protein_coding	GPR150
protein_coding	AL356356.1
protein_coding	AC002365.1
protein_coding	RFK
protein_coding	SIRPG
protein_coding	APOBEC3H
protein_coding	CMTM2
protein_coding	C20orf112
protein_coding	PARK7
protein_coding	WBP1
protein_coding	RDX
protein_coding	SPRED2
protein_coding	MARCKS
protein_coding	KRTAP6-3
protein_coding	NOTUM
protein_coding	EMC7
protein_coding	CAMK4
protein_coding	CSNK1G1
protein_coding	APOBEC3B
protein_coding	UBE2M
protein_coding	SRSF5
protein_coding	RIC1
protein_coding	CNBD2
protein_coding	SIGLEC8
protein_coding	STK40
protein_coding	TMEM139
protein_coding	EVX2
protein_coding	LCE1C
protein_coding	EXOSC1
protein_coding	CFDP1
protein_coding	AK125701
protein_coding	PGAP2
protein_coding	SDHD
protein_coding	EFCAB2
protein_coding	PCDH17
protein_coding	BC068088
protein_coding	CAMSAP2
protein_coding	TOR1AIP1
protein_coding	N4BP2L1
protein_coding	GGT2
protein_coding	GPI

protein_coding	VWA7
protein_coding	SNX25
protein_coding	WDR85
protein_coding	C3orf27
protein_coding	SHC4
protein_coding	ANKLE1
protein_coding	RIPPLY3
protein_coding	AL445665.1
protein_coding	LZTS3
protein_coding	DNAH14
protein_coding	GABRA2
protein_coding	VIT
protein_coding	AGTPBP1
protein_coding	DDX17
protein_coding	SUPT7L
protein_coding	SOX30
protein_coding	MSRB2
protein_coding	RAN
protein_coding	ASAP1
protein_coding	KIF11
protein_coding	RP5-850E9.3
protein_coding	WNT6
protein_coding	ISCA1
protein_coding	EIF4G3
protein_coding	MSMP
protein_coding	CEP131
protein_coding	TMEM160
protein_coding	DRC1
protein_coding	EIF2S3
protein_coding	KRTAP10-2
protein_coding	OPN1MW
protein_coding	KNDC1
protein_coding	DEFA6
protein_coding	YOD1
protein_coding	PAM
protein_coding	C15orf52
protein_coding	ZNFX1
protein_coding	PPP2R3C
protein_coding	SERPING1
protein_coding	FAM221A
protein_coding	GAREM
protein_coding	HLA-DPB1
protein_coding	HOXD1
protein_coding	ADAP1
protein_coding	FAM120AOS
protein_coding	CBLB
protein_coding	PELP1

protein_coding	AX746604
protein_coding	GSAP
protein_coding	GAST
protein_coding	GRIP1
protein_coding	ZFR2
protein_coding	ARL6IP1
protein_coding	USP16
protein_coding	ZHX1-C8orf76
protein_coding	ZNF544
protein_coding	MKLN1
protein_coding	SCD5
protein_coding	ZNF585B
protein_coding	CCRN4L
protein_coding	DHX29
protein_coding	AK056490
protein_coding	C1QTNF4
protein_coding	PAFAH1B3
protein_coding	SH3D21
protein_coding	DYRK1B
protein_coding	LALBA
protein_coding	LUC7L
protein_coding	UGT1A7
protein_coding	HOXA6
protein_coding	C18orf56
protein_coding	LRRC61
protein_coding	ACSL4
protein_coding	SPINK4
protein_coding	ASNS
protein_coding	DCTN4
protein_coding	HESX1
protein_coding	DDHD1
protein_coding	HES1
protein_coding	TNNI3K
protein_coding	ABHD14A-ACY1
protein_coding	DCTN3
protein_coding	NPHS1
protein_coding	C11orf58
protein_coding	PPP1R14B
protein_coding	PCDHGA5
protein_coding	STK39
protein_coding	AGPAT3
protein_coding	ITGB4
protein_coding	LARP7
protein_coding	MAGEA6
protein_coding	ANKRD33B
protein_coding	PSMG1
protein_coding	BMP8B

protein_coding	OR10G3
protein_coding	LPPR3
protein_coding	KRTAP10-12
protein_coding	GLIPR1L1
protein_coding	TMEFF1
protein_coding	OSBPL6
protein_coding	C9orf47
protein_coding	C1orf167
protein_coding	OR6C68
protein_coding	TIAM2
protein_coding	ZBBX
protein_coding	ZNF560
protein_coding	C12orf56
protein_coding	STK32C
protein_coding	EIF1AY
protein_coding	ZRSR2
protein_coding	COG1
protein_coding	FRZB
protein_coding	ADAMTS12
protein_coding	THAP10
protein_coding	ZNF536
protein_coding	SPDYE4
protein_coding	EDA2R
protein_coding	RAD51C
protein_coding	CBWD7
protein_coding	HTR4
protein_coding	BC140723
protein_coding	RELL2
protein_coding	CERS5
protein_coding	SSTR5
protein_coding	RAD51AP1
protein_coding	COMMD8
protein_coding	SUCLG1
protein_coding	ITLN2
protein_coding	PLA2G7
protein_coding	GOLGA6L4
protein_coding	FAM170A
protein_coding	RS1
protein_coding	UROD
protein_coding	DSTYK
protein_coding	PELI1
protein_coding	B3GALNT1
protein_coding	TNFRSF13B
protein_coding	TRPC4
protein_coding	GTF2A1L
protein_coding	KRT14
protein_coding	TRIM33

protein_coding	CANX
protein_coding	RPEL1
protein_coding	ARID5A
protein_coding	DDX39B
protein_coding	ASGR2
protein_coding	CPXM2
protein_coding	ATPIF1
protein_coding	MMGT1
protein_coding	PUF60
protein_coding	COMT
protein_coding	LENG9
protein_coding	CDC37
protein_coding	ATAD5
protein_coding	ZNF837
protein_coding	TRIM3
protein_coding	AMN
protein_coding	RASGRP3
protein_coding	PAX9
protein_coding	GUCA2B
protein_coding	BC038382
protein_coding	NHLH1
protein_coding	HSPA4L
protein_coding	KRTAP6-1
protein_coding	ZNF77
protein_coding	LDHAL6A
protein_coding	HILPDA
protein_coding	RAB5A
protein_coding	FAM109A
protein_coding	WFDC5
protein_coding	SLC25A52
protein_coding	MAGEA3
protein_coding	TLDC2
protein_coding	ZNF71
protein_coding	PITPNA
protein_coding	AC009892.10
protein_coding	OR7E24
protein_coding	MRAP2
protein_coding	ATP6C
protein_coding	PSD
protein_coding	TOMM20L
protein_coding	TSPAN5
protein_coding	CMC1
protein_coding	ZNF816
protein_coding	PLXNA4
protein_coding	TSPAN18
protein_coding	VWA3B
protein_coding	LIMD1

protein_coding	LMO2
protein_coding	GRXCR2
protein_coding	FASLG
protein_coding	COX5B
protein_coding	TMCO2
protein_coding	LOC554223
protein_coding	S1PR5
protein_coding	LRRD1
protein_coding	KIAA1522
protein_coding	EMR3
protein_coding	OR51E1
protein_coding	OR1S2
protein_coding	TAS2R3
protein_coding	ATP1A2
protein_coding	MSH5-SAPCD1
protein_coding	USP26
protein_coding	UBE2Q1
protein_coding	ZNF467
protein_coding	FAM49A
protein_coding	PIK3CB
protein_coding	SNCAIP
protein_coding	PIK3CA
protein_coding	ZC3H15
protein_coding	BTRC
protein_coding	CTPS2
protein_coding	OSTM1
protein_coding	AC006946.15
protein_coding	LAGE3
protein_coding	AL450307.1
protein_coding	POLR2K
protein_coding	GSG1
protein_coding	KSR2
protein_coding	PRIM1
protein_coding	OR8J2
protein_coding	ATP1A4
protein_coding	SF3B1
protein_coding	TMEM74
protein_coding	DNAJB9
protein_coding	TCTN2
protein_coding	OR8S1
protein_coding	DLGAP1
protein_coding	ESRP1
protein_coding	RP11-268J15.5
protein_coding	PRMT7
protein_coding	RPL36A
protein_coding	RSRC1
protein_coding	CLCNKA

protein_coding	SCT
protein_coding	SLC4A4
protein_coding	BZW2
protein_coding	hCG_1995134
protein_coding	SERTM1
protein_coding	PSMD8
protein_coding	TRDN
protein_coding	AC005544.1
protein_coding	ABCD1
protein_coding	DTX4
protein_coding	OTOG
protein_coding	NDUFA12
protein_coding	NDUFA11
protein_coding	SFT2D1
protein_coding	ARGLU1
protein_coding	ATXN2
protein_coding	DEGS1
protein_coding	YJEFN3
protein_coding	DNAJC7
protein_coding	RAB3C
protein_coding	C8A
protein_coding	PRSS3
protein_coding	ZFYVE21
protein_coding	GPR155
protein_coding	SCOC
protein_coding	SHCBP1
protein_coding	MYL2
protein_coding	PRND
protein_coding	KRT80
protein_coding	LSAMP
protein_coding	CCM2
protein_coding	DNAJB13
protein_coding	PDSS2
protein_coding	TRAF4
protein_coding	SUMO1
protein_coding	TMEM178B
protein_coding	UBXN6
protein_coding	VCP
protein_coding	MRPL24
protein_coding	GAB2
protein_coding	FAF1
protein_coding	SLC4A5
protein_coding	GALR2
protein_coding	C1QTNF9B-AS1
protein_coding	YY2
protein_coding	C18orf54
protein_coding	CRABP1

protein_coding	GCM1
protein_coding	SCAMP3
protein_coding	PCDHA8
protein_coding	SLC8A1
protein_coding	JAK1
protein_coding	SACM1L
protein_coding	MND1
protein_coding	TP73
protein_coding	SMYD3
protein_coding	ALK
protein_coding	WDR96
protein_coding	SLC30A9
protein_coding	TFDP3
protein_coding	RESP18
protein_coding	COL23A1
protein_coding	NBPF1
protein_coding	ZNF430
protein_coding	FAM181B
protein_coding	OR5M3
protein_coding	EDN2
protein_coding	NT5DC3
protein_coding	NSMF
protein_coding	RAD23A
protein_coding	OR4K17
protein_coding	MS4A13
protein_coding	TGM1
protein_coding	UQCC
protein_coding	SLC7A11
protein_coding	RAET1E
protein_coding	MCCC1
protein_coding	PPP4R2
protein_coding	MAPK1
protein_coding	MPRIP
protein_coding	PAIP1
protein_coding	WFDC12
protein_coding	RBM17
protein_coding	PVRL1
protein_coding	MAP3K11
protein_coding	MAGED1
protein_coding	IDO2
protein_coding	C17orf64
protein_coding	PYGM
protein_coding	ARL1
protein_coding	POTED
protein_coding	JTB
protein_coding	PLS3
protein_coding	AK074615

protein_coding	ODF4
protein_coding	WAS
protein_coding	DCDC2B
protein_coding	MTCH1
protein_coding	SMDT1
protein_coding	MARK4
protein_coding	TMBIM6
protein_coding	AC007401.2
protein_coding	KRTAP19-2
protein_coding	CES4A
protein_coding	HTR3C
protein_coding	GDI1
protein_coding	ANXA8L1
protein_coding	RAB39B
protein_coding	GNAZ
protein_coding	RAB11FIP2
protein_coding	C7orf55
protein_coding	USP25
protein_coding	POC1A
protein_coding	FAM153B
protein_coding	TCTEX1D4
protein_coding	MPC2
protein_coding	VDAC1
protein_coding	PER3
protein_coding	GALC
protein_coding	CTD-2210P24.4
protein_coding	SNTG1
protein_coding	PPP2R2A
protein_coding	GIPC3
protein_coding	SGK494
protein_coding	C2CD5
protein_coding	TBC1D3G
protein_coding	CLTCL1
protein_coding	CPNE8
protein_coding	DOK6
protein_coding	OR2T12
protein_coding	MAGI3
protein_coding	DNAJB4
protein_coding	HECTD1
protein_coding	TP53BP1
protein_coding	SPIN1
protein_coding	GLTSCR1L
protein_coding	PFN2
protein_coding	ANKRD27
protein_coding	G6PC2
protein_coding	CTSL
protein_coding	ACSS1

protein_coding	FKBP15
protein_coding	IDNK
protein_coding	MPP3
protein_coding	PMM1
protein_coding	MAPK8IP2
protein_coding	CD86
protein_coding	KLHDC9
protein_coding	OR8G1
protein_coding	BC069257
protein_coding	SYT4
protein_coding	PHLDA1
protein_coding	ZDHHC11
protein_coding	ZG16
protein_coding	CHRNE
protein_coding	AX747376
protein_coding	PRG3
protein_coding	SPDYE2B
protein_coding	UBA3
protein_coding	FKBP1B
protein_coding	LOC643355
protein_coding	ZNF157
protein_coding	FAM173A
protein_coding	PHF11
protein_coding	NXT1
protein_coding	OSBPL10
protein_coding	SLC39A5
protein_coding	CD27
protein_coding	TNNI3
protein_coding	RNF19A
protein_coding	BPIFA3
protein_coding	EGLN2
protein_coding	FOXR1
protein_coding	KIAA0319L
protein_coding	AX746485
protein_coding	RDH16
protein_coding	TTC32
protein_coding	RP11-944C7.1
protein_coding	FBXW4
protein_coding	POMP
protein_coding	AL390778.1
protein_coding	SLC7A3
protein_coding	MITF
protein_coding	PPA1
protein_coding	GPR139
protein_coding	PSEN1
protein_coding	RASL11A
protein_coding	OBSCN

protein_coding	RTCB
protein_coding	RBM34
protein_coding	CLDND2
protein_coding	TP53
protein_coding	NIPAL3
protein_coding	CHD1L
protein_coding	HMCN1
protein_coding	KIAA1324L
protein_coding	VPS29
protein_coding	GPR173
protein_coding	SGK223
protein_coding	SLC7A9
protein_coding	KLHDC3
protein_coding	C17orf77
protein_coding	MEF2BNB-MEF2B
protein_coding	ACSM3
protein_coding	RPL21
protein_coding	LEAP2
protein_coding	SLC35B1
protein_coding	MLPH
protein_coding	IWS1
protein_coding	AMICA1
protein_coding	C6orf106
protein_coding	AMY2B
protein_coding	GINS1
protein_coding	AP003062.1
protein_coding	LYRM7
protein_coding	CCDC108
protein_coding	XDH
protein_coding	NMUR2
protein_coding	NRXN2
protein_coding	SLC52A3
protein_coding	C12orf29
protein_coding	ZNF320
protein_coding	CTC-512J12.6
protein_coding	ESR1
protein_coding	SARS
protein_coding	RAP2A
protein_coding	TMEM155
protein_coding	IFT57
protein_coding	RAP1A
protein_coding	RYBP
protein_coding	TNFAIP1
protein_coding	CXorf40B
protein_coding	KCTD16
protein_coding	PDIA5
protein_coding	EIF5A2

protein_coding	PHLDA2
protein_coding	ZFYVE16
protein_coding	KCNIP3
protein_coding	OR2J3
protein_coding	LGI3
protein_coding	PIFO
protein_coding	ARL3
protein_coding	SCAMP4
protein_coding	FOXJ1
protein_coding	C5orf63
protein_coding	FAM188B2
protein_coding	CADM1
protein_coding	ANAPC5
protein_coding	AKAP12
protein_coding	BCL11B
protein_coding	BRPF3
protein_coding	HIST3H2BB
protein_coding	APOC4-APOC2
protein_coding	OR11H2
protein_coding	TAF7
protein_coding	AMPH
protein_coding	SIPA1L2
protein_coding	PDHA1
protein_coding	AMPD3
protein_coding	USO1
protein_coding	XKR8
protein_coding	ZWILCH
protein_coding	SERPINB4
protein_coding	IL20RB
protein_coding	MTMR8
protein_coding	AP3M2
protein_coding	UNC5CL
protein_coding	NDUFAF5
protein_coding	CYP4F11
protein_coding	VKORC1L1
protein_coding	IMP4
protein_coding	TEX101
protein_coding	PRKRA
protein_coding	SLC22A18AS
protein_coding	CTB-129P6.11
protein_coding	CHMP3
protein_coding	CRYBB3
protein_coding	GBAS
protein_coding	FAM170B
protein_coding	CLASP2
protein_coding	FSBP
protein_coding	PREX2

protein_coding	VCPKMT
protein_coding	SCPEP1
protein_coding	PCDHA3
protein_coding	TRABD
protein_coding	SCML2
protein_coding	MRPS15
protein_coding	RPS6
protein_coding	OCIAD2
protein_coding	OR51T1
protein_coding	C2orf43
protein_coding	CDY1
protein_coding	ERC2
protein_coding	TNFRSF10B
protein_coding	SULT1C4
protein_coding	AQP10
protein_coding	MYO7B
protein_coding	CFC1
protein_coding	VENTX
protein_coding	NELL1
protein_coding	OR4C45
protein_coding	PPM1G
protein_coding	VDR
protein_coding	MIXL1
protein_coding	CD52
protein_coding	RAD51B
protein_coding	ITFG2
protein_coding	PDE6D
protein_coding	DUSP6
protein_coding	SNX1
protein_coding	EIF4EBP1
protein_coding	RPL27
protein_coding	KCNK17
protein_coding	HEXIM1
protein_coding	N4BP2
protein_coding	MTIF2
protein_coding	STEAP1B
protein_coding	CLCF1
protein_coding	TDRD6
protein_coding	STARD7
protein_coding	MS4A15
protein_coding	HIST1H3A
protein_coding	SENP6
protein_coding	ATP6AP2
protein_coding	IQSEC1
protein_coding	KHDC1L
protein_coding	ZCCHC5
protein_coding	CD81

protein_coding	PCDHA11
protein_coding	AP3B1
protein_coding	TMEM57
protein_coding	DDC
protein_coding	RPL24
protein_coding	EXOC8
protein_coding	TMEM63A
protein_coding	FPR3
protein_coding	RHOXF2B
protein_coding	E2F5
protein_coding	TPBGL
protein_coding	GOLGA7
protein_coding	SLCO3A1
protein_coding	SLC5A12
protein_coding	PSMD4
protein_coding	NID1
protein_coding	RECQL4
protein_coding	ZNF565
protein_coding	FBXO3
protein_coding	TBC1D21
protein_coding	GUCY2F
protein_coding	HCN4
protein_coding	ATP2A2
protein_coding	ATP6V1G3
protein_coding	KIR3DL3
protein_coding	ATHL1
protein_coding	CDCA4
protein_coding	NPEPL1
protein_coding	ITIH2
protein_coding	HIGD1C
protein_coding	HEYL
protein_coding	COPS7A
protein_coding	SAPCD1
protein_coding	TMEM86A
protein_coding	SCRT2
protein_coding	CCDC53
protein_coding	RAB9A
protein_coding	ITGA5
protein_coding	GRWD1
protein_coding	POU3F4
protein_coding	KDF1
protein_coding	BHLHE23
protein_coding	PNPLA8
protein_coding	ANKRD24
protein_coding	SLC22A13
protein_coding	KIAA1671
protein_coding	DZIP1L

protein_coding	SORD
protein_coding	TRIM49C
protein_coding	GDF7
protein_coding	MYZAP
protein_coding	MESDC1
protein_coding	TENM4
protein_coding	STX12
protein_coding	SLC30A3
protein_coding	CWC22
protein_coding	MXD4
protein_coding	GGTLC1
protein_coding	PTPN7
protein_coding	KRTAP5-1
protein_coding	IDH3B
protein_coding	KIAA0232
protein_coding	FZD5
protein_coding	C7orf62
protein_coding	CATSPERD
protein_coding	SERPINA4
protein_coding	ZNF471
protein_coding	ZNF582
protein_coding	UPK3B
protein_coding	ITFG3
protein_coding	SPAG16
protein_coding	ADGRF3
protein_coding	LRFN5
protein_coding	TCF7L1
protein_coding	TAC1
protein_coding	TP53BP2
protein_coding	TYMP
protein_coding	RPL7
protein_coding	GPR108
protein_coding	CCNA2
protein_coding	TTC8
protein_coding	TTC34
protein_coding	RPSA
protein_coding	FBLIM1
protein_coding	CPA4
protein_coding	SUN2
protein_coding	SNX17
protein_coding	SCD
protein_coding	CENPH
protein_coding	AC087645.1
protein_coding	C1GALT1
protein_coding	FIBP
protein_coding	TMEM167A
protein_coding	NOX4

protein_coding	RP11-685N3.1
protein_coding	RPP21
protein_coding	USP4
protein_coding	CITED4
protein_coding	TSPY6P
protein_coding	SSMEM1
protein_coding	LGALS2
protein_coding	ZNF184
protein_coding	C1orf21
protein_coding	NDRG1
protein_coding	TRMT11
protein_coding	INPP5F
protein_coding	CYP3A7
protein_coding	DYNC1LI1
protein_coding	LRRC17
protein_coding	SYNJ1
protein_coding	TEX36
protein_coding	APOL2
protein_coding	LMBR1L
protein_coding	SIK2
protein_coding	METTL5
protein_coding	SLC5A10
protein_coding	TRIM73
protein_coding	KNG1
protein_coding	TMEM211
protein_coding	ZC2HC1B
protein_coding	SHD
protein_coding	ZCRB1
protein_coding	DLK1
protein_coding	F2RL3
protein_coding	LCN9
protein_coding	MT1HL1
protein_coding	CCDC183
protein_coding	GPR161
protein_coding	PMS1
protein_coding	MAP7D3
protein_coding	TMEM37
protein_coding	TMEM253
protein_coding	GUCY1A2
protein_coding	ADAM21
protein_coding	POLH
protein_coding	ATP6V0D1
protein_coding	LPAR4
protein_coding	KRT2
protein_coding	VSIG8
protein_coding	CNTNAP5
protein_coding	HOXB9

protein_coding	BNIP3
protein_coding	CT45A5
protein_coding	TRIM49B
protein_coding	SPRY2
protein_coding	TIAM1
protein_coding	IL17B
protein_coding	OR5K2
protein_coding	VAPA
protein_coding	ZMYM2
protein_coding	IGHMBP2
protein_coding	ZNF764
protein_coding	AP001652.1
protein_coding	EFCAB7
protein_coding	DBX2
protein_coding	SRL
protein_coding	RP11-766F14.2
protein_coding	EMC4
protein_coding	CD63
protein_coding	PARP2
protein_coding	DLX5
protein_coding	HS6ST1
protein_coding	HN1
protein_coding	CENPA
protein_coding	SERPINA1
protein_coding	SAMSN1
protein_coding	CXorf66
protein_coding	RCN2
protein_coding	MST4
protein_coding	ADM
protein_coding	PSMD2
protein_coding	CLSPN
protein_coding	SLFN14
protein_coding	NCOA1
protein_coding	AFF4
protein_coding	MTM1
protein_coding	CEP170B
protein_coding	BHMT
protein_coding	RRAGC
protein_coding	AC103801.2
protein_coding	LRRC40
protein_coding	SBSN
protein_coding	FAM50B
protein_coding	SALL4
protein_coding	MYH7B
protein_coding	HNRNPUL1
protein_coding	KCNK16
protein_coding	DECR1

protein_coding	CCDC154
protein_coding	CNTN2
protein_coding	DDX60
protein_coding	C2orf82
protein_coding	CYB561A3
protein_coding	DYNC2LI1
protein_coding	HAAO
protein_coding	FAM72B
protein_coding	SLC6A16
protein_coding	PRKAR2B
protein_coding	JMY
protein_coding	GAS7
protein_coding	SERF1A
protein_coding	ATF5
protein_coding	NAA11
protein_coding	CDC42EP3
protein_coding	HSH2D
protein_coding	RWDD2B
protein_coding	ARID4B
protein_coding	WDR65
protein_coding	ZNF217
protein_coding	C14orf183
protein_coding	STK38L
protein_coding	CCNB1
protein_coding	CDR2
protein_coding	CNDP2
protein_coding	ABCB4
protein_coding	BC215
protein_coding	ZNF281
protein_coding	AKAP11
protein_coding	CTD-2545M3.6
protein_coding	WDR90
protein_coding	LRIT2
protein_coding	NBL1
protein_coding	KLHL12
protein_coding	ZNF189
protein_coding	MINA
protein_coding	CD22
protein_coding	C11orf96
protein_coding	LRRC7
protein_coding	RUNDC3B
protein_coding	SLC22A24
protein_coding	SFTPB
protein_coding	BECN2
protein_coding	TOB1
protein_coding	WDR3
protein_coding	ACBD3

protein_coding	ASTN2
protein_coding	BLOC1S1
protein_coding	STOM
protein_coding	DDHD2
protein_coding	TEC
protein_coding	PRAMEF12
protein_coding	UROC1
protein_coding	GATAD2B
protein_coding	SCN7A
protein_coding	TKT
protein_coding	TONSL
protein_coding	KLHL32
protein_coding	CNN2
protein_coding	ZBTB8OS
protein_coding	DQX1
protein_coding	CTSO
protein_coding	C12orf65
protein_coding	RPAP3
protein_coding	PRTG
protein_coding	ECH1
protein_coding	SULT2B1
protein_coding	SF3B4
protein_coding	DHRS2
protein_coding	GPSM2
protein_coding	CLPSL2
protein_coding	VSIG2
protein_coding	TAF9
protein_coding	GEMIN4
protein_coding	HNRNPR
protein_coding	HAO2
protein_coding	TMEM194B
protein_coding	CYP3A4
protein_coding	ARHGAP12
protein_coding	AEN
protein_coding	ENTPD6
protein_coding	GGTLC2
protein_coding	C1orf52
protein_coding	SNX5
protein_coding	ZMYM6NB
protein_coding	TMEM99
protein_coding	GDF5
protein_coding	LRRC52
protein_coding	FOXP1
protein_coding	ATP6V0C
protein_coding	OR10J3
protein_coding	DDX6
protein_coding	RPL35

protein_coding	SERPINE1
protein_coding	RNASEH2A
protein_coding	MMS22L
protein_coding	C15orf26
protein_coding	MRPS33
protein_coding	DDIAS
protein_coding	OR2AG1
protein_coding	MYEOV
protein_coding	ARID3B
protein_coding	ERH
protein_coding	CPZ
protein_coding	ZC3H13
protein_coding	PPP1R18
protein_coding	OR11H7
protein_coding	IL3RA
protein_coding	PGC
protein_coding	AX747991
protein_coding	ANKRD46
protein_coding	BCKDHB
protein_coding	TBX20
protein_coding	NFX1
protein_coding	CISH
protein_coding	PTRF
protein_coding	CRYBB2
protein_coding	FLNC
protein_coding	SLC25A42
protein_coding	NEUROG3
protein_coding	AADACL4
protein_coding	LINC01118
protein_coding	PLAGL1
protein_coding	FAM231A
protein_coding	AC020922.1
protein_coding	PSMD12
protein_coding	ZNF888
protein_coding	ZSCAN20
protein_coding	JUP
protein_coding	PGRMC1
protein_coding	FAM174A
protein_coding	QRSL1
protein_coding	AC007040.11
protein_coding	KCNK10
protein_coding	BC032415
protein_coding	RP11-58C22.1
protein_coding	TPPP3
protein_coding	UROS
protein_coding	KRTAP29-1
protein_coding	SLC18A1

protein_coding	DBI
protein_coding	CYP2D7P
protein_coding	SEP15
protein_coding	WDR83
protein_coding	ZNF514
protein_coding	ZNF497
protein_coding	C21orf62
protein_coding	REEP5
protein_coding	GPR78
protein_coding	COPS4
protein_coding	EP400NL
protein_coding	ACPP
protein_coding	C11orf83
protein_coding	C15orf61
protein_coding	LOC90925
protein_coding	PRKX
protein_coding	PCP4L1
protein_coding	PRSS56
protein_coding	MYDGF
protein_coding	ATXN1
protein_coding	HMGCLL1
protein_coding	PLEKHA1
protein_coding	GRM6
protein_coding	ARV1
protein_coding	ZDHHC17
protein_coding	NPEPPS
protein_coding	TMEM126A
protein_coding	RSPH4A
protein_coding	S100A7A
protein_coding	CLIP1
protein_coding	IFIH1
protein_coding	CPLX1
protein_coding	NDRG3
protein_coding	AP2B1
protein_coding	RAD21
protein_coding	FAM109B
protein_coding	CTD-3214H19.16
protein_coding	S100A4
protein_coding	BPIFB4
protein_coding	YIF1B
protein_coding	PAQR7
protein_coding	ASPA
protein_coding	ALG10B
protein_coding	COX8A
protein_coding	PCSK2
protein_coding	RGL2
protein_coding	AX748283

protein_coding	ANKRD20A3
protein_coding	GOLIM4
protein_coding	ATG13
protein_coding	SH3D19
protein_coding	DNAJC12
protein_coding	DDX4
protein_coding	SCLY
protein_coding	ZNF444
protein_coding	LRR1
protein_coding	MYOZ3
protein_coding	IQCJ
protein_coding	C21orf59
protein_coding	TPRA1
protein_coding	C9orf96
protein_coding	FUT7
protein_coding	SLITRK3
protein_coding	MIDN
protein_coding	PNPLA1
protein_coding	KDM4B
protein_coding	ZNF25
protein_coding	ZBTB42
protein_coding	AC002310.13
protein_coding	CHMP2B
protein_coding	C1orf127
protein_coding	PACSIN1
protein_coding	ADM2
protein_coding	PRG2
protein_coding	ZNF738
protein_coding	SDIM1
protein_coding	PAMR1
protein_coding	CYP4F31P
protein_coding	EBF4
protein_coding	TMEM52
protein_coding	ZNF667
protein_coding	FOPNL
protein_coding	EHBP1
protein_coding	ZNF852
protein_coding	OR5AP2
protein_coding	SESN3
protein_coding	KCNN1
protein_coding	CLK1
protein_coding	PCYT2
protein_coding	GRSF1
protein_coding	RLN1
protein_coding	ZFR
protein_coding	CUZD1
protein_coding	CYP27A1

protein_coding	SMCO4
protein_coding	VPS26A
protein_coding	MARCH11
protein_coding	STX1A
protein_coding	SLFNL1
protein_coding	RP11-480I12.4
protein_coding	GLOD5
protein_coding	GOLGA6L3
protein_coding	APOBEC3A
protein_coding	CCNO
protein_coding	PEG10
protein_coding	C19orf80
protein_coding	FOXI1
protein_coding	ZNF831
protein_coding	MBOAT2
protein_coding	DDAH1
protein_coding	ADGRF1
protein_coding	LRRC74B
protein_coding	PEX5L
protein_coding	ADAMTSL4
protein_coding	ADARB2
protein_coding	RRP12
protein_coding	TCP10
protein_coding	MAP1LC3A
protein_coding	RBM4B
protein_coding	FMR1
protein_coding	SLC4A1AP
protein_coding	LY6G6D
protein_coding	PTBP1
protein_coding	RIT1
protein_coding	CRISPLD2
protein_coding	NPC1
protein_coding	EVPLL
protein_coding	PIGZ
protein_coding	SLC45A2
protein_coding	ARHGAP21
protein_coding	GSTT2B
protein_coding	DRD3
protein_coding	IL17REL
protein_coding	NBN
protein_coding	MARK3
protein_coding	PIK3AP1
protein_coding	ZNF148
protein_coding	CCDC114
protein_coding	TNFAIP8
protein_coding	RAD51
protein_coding	ADGRB1

protein_coding	SMTN
protein_coding	WASF3
protein_coding	TIMP4
protein_coding	ARHGAP35
protein_coding	ATP5H
protein_coding	LRRC8C
protein_coding	NPTX1
protein_coding	BLK
protein_coding	SSPO
protein_coding	PEX16
protein_coding	HDHD1
protein_coding	TESK2
protein_coding	RNASE2
protein_coding	VBP1
protein_coding	MAST4
protein_coding	SNRNP27
protein_coding	NDUFB6
protein_coding	ATP5EP2
protein_coding	KRTAP10-6
protein_coding	BTNL2
protein_coding	TNFRSF10D
protein_coding	GCA
protein_coding	TRMT1L
protein_coding	AC135048.1
protein_coding	AURKB
protein_coding	ID3
protein_coding	SOD2
protein_coding	EVA1A
protein_coding	RNF183
protein_coding	SPDYA
protein_coding	TRIM67
protein_coding	KEAP1
protein_coding	ZNF28
protein_coding	CCDC88C
protein_coding	HIST1H1B
protein_coding	ACTR3
protein_coding	BTBD19
protein_coding	KPNB1
protein_coding	TLR7
protein_coding	RPS18
protein_coding	OCA2
protein_coding	CHP1
protein_coding	PROCA1
protein_coding	ADCYAP1
protein_coding	LAMB3
protein_coding	C5orf27
protein_coding	MSS51

protein_coding	APOO
protein_coding	ACTB
protein_coding	MCMBP
protein_coding	PDE7A
protein_coding	SLC22A12
protein_coding	MPHOSPH8
protein_coding	FAM217B
protein_coding	CHI3L1
protein_coding	GSDMB
protein_coding	ECEL1
protein_coding	KNTC1
protein_coding	PPP1R27
protein_coding	GATA1
protein_coding	PTGIR
protein_coding	RHOB
protein_coding	RP11-295P9.3
protein_coding	KRT6A
protein_coding	NOMO2
protein_coding	CSRNP2
protein_coding	SMARCD3
protein_coding	DOCK9
protein_coding	AK126180
protein_coding	MYO6
protein_coding	AIFM1
protein_coding	DYSF
protein_coding	AP1M2
protein_coding	KRTAP4-12
protein_coding	FLJ45079
protein_coding	PMPCB
protein_coding	ULBP3
protein_coding	SLITRK2
protein_coding	NBAS
protein_coding	ACER2
protein_coding	OMA1
protein_coding	GDAP1
protein_coding	SYN2
protein_coding	GPSM1
protein_coding	CST4
protein_coding	CDH10
protein_coding	TFAP2E
protein_coding	TAS2R4
protein_coding	MRPL40
protein_coding	GALNT13
protein_coding	SYCP2
protein_coding	C17orf112
protein_coding	ORMDL1
protein_coding	INPP4A

protein_coding	TOX3
protein_coding	CLCN3
protein_coding	RP11-96O20.4
protein_coding	PTGR1
protein_coding	RHD
protein_coding	LRGUK
protein_coding	SNX4
protein_coding	PNPO
protein_coding	FAM155A
protein_coding	FBXW11
protein_coding	DQ574239
protein_coding	EEF2
protein_coding	CMTM8
protein_coding	FAM63A
protein_coding	SKIV2L
protein_coding	FER
protein_coding	FAHD2A
protein_coding	TM4SF19
protein_coding	TOMM40L
protein_coding	FGD3
protein_coding	CEP152
protein_coding	SEPT5
protein_coding	PCDH7
protein_coding	YIPF2
protein_coding	MYL6
protein_coding	CORO1A
protein_coding	P2RY14
protein_coding	IGSF6
protein_coding	GPR42
protein_coding	BOD1L2
protein_coding	HN1L
protein_coding	LRCH1
protein_coding	SLC29A4
protein_coding	MRPS22
protein_coding	GPR75
protein_coding	HMGCS1
protein_coding	INAFM2
protein_coding	A4GNT
protein_coding	AC110781.3
protein_coding	PRRG1
protein_coding	EIF5B
protein_coding	TGM4
protein_coding	DTYMK
protein_coding	AHSP
protein_coding	SPAG17
protein_coding	ETV6
protein_coding	GS1-259H13.2

protein_coding	WDR7
protein_coding	PUM2
protein_coding	TNFAIP8L1
protein_coding	FRMPD4
protein_coding	RASSF9
protein_coding	RAP1GAP
protein_coding	NBPF4
protein_coding	NBPF9
protein_coding	MID1IP1
protein_coding	HOXB4
protein_coding	GPAT2
protein_coding	RP11-934B9.3
protein_coding	PCDHA6
protein_coding	WNT5A
protein_coding	EFCAB1
protein_coding	OR5L1
protein_coding	RCE1
protein_coding	IRS2
protein_coding	ZBTB49
protein_coding	MTA2
protein_coding	KIAA1549L
protein_coding	WDR61
protein_coding	BARHL1
protein_coding	PRSS22
protein_coding	TMPRSS5
protein_coding	GFRA3
protein_coding	CCNC
protein_coding	PIK3R4
protein_coding	C6orf48
protein_coding	KRTAP19-1
protein_coding	LCN6
protein_coding	LANCL2
protein_coding	HIST1H2AE
protein_coding	PIK3C2G
protein_coding	TTC37
protein_coding	DOPEY1
protein_coding	EPHA2
protein_coding	ASCL2
protein_coding	AC110615.1
protein_coding	TPRG1L
protein_coding	M6PR
protein_coding	HS3ST3B1
protein_coding	BTNL9
protein_coding	GOLGA6L2
protein_coding	PRC1
protein_coding	TBCCD1
protein_coding	LAMC1

protein_coding	SHFM1
protein_coding	OLFM3
protein_coding	TRAPPC2L
protein_coding	ODF2
protein_coding	CMTM4
protein_coding	CDH26
protein_coding	BC132944
protein_coding	TYMSOS
protein_coding	TMEM190
protein_coding	ZNF746
protein_coding	ENDOG
protein_coding	IL15
protein_coding	WNK1
protein_coding	NRSN2
protein_coding	RNF10
protein_coding	FBXO11
protein_coding	MYL7
protein_coding	PITPNB
protein_coding	YARS
protein_coding	COLGALT2
protein_coding	PAF1
protein_coding	PPM1L
protein_coding	POFUT2
protein_coding	GLDN
protein_coding	EPHB4
protein_coding	TMEM106A
protein_coding	MRPL1
protein_coding	APBB3
protein_coding	UQCC1
protein_coding	CTC-241N9.1
protein_coding	SLC22A15
protein_coding	SBF1
protein_coding	ETV2
protein_coding	GOLGA6L19
protein_coding	SIGLEC15
protein_coding	BCL2L11
protein_coding	CACNA1A
protein_coding	SUMO4
protein_coding	CCDC135
protein_coding	PCYOX1L
protein_coding	TXNL1
protein_coding	CCDC109B
protein_coding	CTD-2547L24.3
protein_coding	SLC35A4
protein_coding	GPC4
protein_coding	TUBB1
protein_coding	PAFAH1B1

protein_coding	CDKN1A
protein_coding	CCNH
protein_coding	CCR6
protein_coding	NPIPB6
protein_coding	GOLGA2
protein_coding	SPNS3
protein_coding	NMU
protein_coding	ZNF219
protein_coding	NFYC
protein_coding	AC005841.1
protein_coding	FNTA
protein_coding	RABGAP1
protein_coding	ANGPTL2
protein_coding	TMEM106B
protein_coding	MEIS1
protein_coding	BAG5
protein_coding	MRPL3
protein_coding	RNF146
protein_coding	DUPD1
protein_coding	RPL27A
protein_coding	MAD2L2
protein_coding	FLCN
protein_coding	PCGF5
protein_coding	PROL1
protein_coding	PPP1R9A
protein_coding	UPB1
protein_coding	FP15737
protein_coding	PLA2G2C
protein_coding	CEP89
protein_coding	RAB7A
protein_coding	BC013821
protein_coding	ADAM12
protein_coding	AL121761.2
protein_coding	C2orf40
protein_coding	PRPS1
protein_coding	SASH1
protein_coding	RPL38
protein_coding	PAIP2B
protein_coding	KRTAP10-5
protein_coding	ADAM20
protein_coding	COA5
protein_coding	CA4
protein_coding	CTRL
protein_coding	MADD
protein_coding	CYP4A22
protein_coding	SLC45A1
protein_coding	KIAA0125

protein_coding	PPWD1
protein_coding	KLHL30
protein_coding	NPFFR2
protein_coding	AC132192.1
protein_coding	ZBTB8B
protein_coding	MAMDC4
protein_coding	RAB14
protein_coding	TMEM88
protein_coding	KRT10
protein_coding	FAM57A
protein_coding	C2orf71
protein_coding	DKK1
protein_coding	FGL1
protein_coding	PYGL
protein_coding	HLA-DOB
protein_coding	MRPL57
protein_coding	RPL9
protein_coding	RAB40B
protein_coding	MVB12A
protein_coding	RPS14
protein_coding	ZNF572
protein_coding	NUDT18
protein_coding	ALDH7A1
protein_coding	GPR160
protein_coding	PIWIL2
protein_coding	TTC36
protein_coding	GFI1
protein_coding	BX537921
protein_coding	TCFL5
protein_coding	SLC2A11
protein_coding	TRPV4
protein_coding	CSRP2BP
protein_coding	GEMIN5
protein_coding	CKMT2
protein_coding	CA9
protein_coding	RP11-618P17.4
protein_coding	RAB23
protein_coding	HTR1F
protein_coding	TCP11L1
protein_coding	DNTTIP2
protein_coding	ATP6V0A2
protein_coding	SNRNP200
protein_coding	CTBP2
protein_coding	CLK4
protein_coding	TALDO1
protein_coding	GOLGA8A
protein_coding	RABEP1

protein_coding	WDR81
protein_coding	GNB1
protein_coding	PCCB
protein_coding	PPL
protein_coding	NCALD
protein_coding	FETUB
protein_coding	SLC38A11
protein_coding	AGTRAP
protein_coding	HIST1H2BN
protein_coding	AHR
protein_coding	RP11-794P6.2
protein_coding	RP11-81K2.1
protein_coding	PRICKLE1
protein_coding	CUEDC2
protein_coding	HES5
protein_coding	HTR1E
protein_coding	LELP1
protein_coding	XAF1
protein_coding	ZNF618
protein_coding	TCEAL1
protein_coding	MAL
protein_coding	TTLL3
protein_coding	RNASEH2B
protein_coding	UQCRQ
protein_coding	ETFB
protein_coding	CYP4B1
protein_coding	MACROD1
protein_coding	YME1L1
protein_coding	YIPF7
protein_coding	CTNND1
protein_coding	CTD-3074O7.11
protein_coding	POU4F1
protein_coding	C20orf166-AS1
protein_coding	AGA
protein_coding	RP3-422G23.4
protein_coding	MAP9
protein_coding	TMX4
protein_coding	HRNR
protein_coding	DEFB129
protein_coding	SNAPC5
protein_coding	TRA2B
protein_coding	CXorf24
protein_coding	OR10A4
protein_coding	BOD1L1
protein_coding	AIF1L
protein_coding	GSK3B
protein_coding	C7orf73

protein_coding	CUTA
protein_coding	TNFRSF8
protein_coding	AADACL2
protein_coding	TCF19
protein_coding	GPRC5D
protein_coding	ECHDC3
protein_coding	IQUB
protein_coding	ACRV1
protein_coding	ARID5B
protein_coding	EPHX4
protein_coding	CRACR2B
protein_coding	TNFRSF10C
protein_coding	C19orf70
protein_coding	OIT3
protein_coding	TRNP1
protein_coding	AKIRIN2
protein_coding	SH3GL2
protein_coding	MYOM1
protein_coding	AMN1
protein_coding	DLGAP2
protein_coding	DCUN1D1
protein_coding	TECTA
protein_coding	UBE2E2
protein_coding	FKBP10
protein_coding	TNK1
protein_coding	ZNF469
protein_coding	CACNA1H
protein_coding	TSPAN10
protein_coding	CHCHD1
protein_coding	SFN
protein_coding	LRPPRC
protein_coding	AK302092
protein_coding	ZNF599
protein_coding	UBA5
protein_coding	GPN3
protein_coding	TCEANC
protein_coding	AP1S2
protein_coding	ZNF331
protein_coding	GRHL2
protein_coding	EDARADD
protein_coding	ZGPAT
protein_coding	SCRN3
protein_coding	ZNF792
protein_coding	ANKDD1A
protein_coding	UPK1A
protein_coding	MEIS2
protein_coding	RP13-512J5.1

protein_coding	C3orf70
protein_coding	MDGA1
protein_coding	SMIM14
protein_coding	CCNB3
protein_coding	CST9
protein_coding	RPGR
protein_coding	ZSWIM7
protein_coding	ZNF277
protein_coding	CCL26
protein_coding	SERPIND1
protein_coding	EMC6
protein_coding	OTX1
protein_coding	BRSK1
protein_coding	PRPF40A
protein_coding	ARHGAP29
protein_coding	OR2Y1
protein_coding	LRRC8D
protein_coding	MYCN
protein_coding	U82695.9
protein_coding	ERGIC2
protein_coding	RAC2
protein_coding	PPARGC1A
protein_coding	C4orf36
protein_coding	PCDHGC4
protein_coding	ZNF850
protein_coding	ASUN
protein_coding	ZNF664-FAM101A
protein_coding	AP000679.2
protein_coding	UNCX
protein_coding	TBC1D2B
protein_coding	LRRTM3
protein_coding	LSMEM2
protein_coding	KIF5A
protein_coding	CHRNA4
protein_coding	ATP2B3
protein_coding	RIMBP3C
protein_coding	C5orf15
protein_coding	TRPM3
protein_coding	KLHL4
protein_coding	S1PR2
protein_coding	WBSCR28
protein_coding	SENP2
protein_coding	BX255923.1
protein_coding	PHC1
protein_coding	CAB39
protein_coding	C15orf53
protein_coding	PIN4

protein_coding	FAM131A
protein_coding	PARD3B
protein_coding	MROH6
protein_coding	CCT8
protein_coding	TUBB
protein_coding	NNAT
protein_coding	CDCP1
protein_coding	KLHL25
protein_coding	TROVE2
protein_coding	SH3PXD2B
protein_coding	PRED62
protein_coding	RITA1
protein_coding	UAP1L1
protein_coding	AHNAK
protein_coding	CAMLG
protein_coding	CUL3
protein_coding	TMEM119
protein_coding	PMVK
protein_coding	KDM4E
protein_coding	UBALD1
protein_coding	HIST1H4H
protein_coding	SEPT1
protein_coding	STMN4
protein_coding	OSCP1
protein_coding	CREB3L3
protein_coding	OSBPL11
protein_coding	GTF2F1
protein_coding	NR0B2
protein_coding	ZNF316
protein_coding	AX748268
protein_coding	AL359878.1
protein_coding	GRIPAP1
protein_coding	PSMD11
protein_coding	SRD5A2
protein_coding	CST1
protein_coding	ACTN2
protein_coding	TJP1
protein_coding	B3GNT6
protein_coding	GPBP1
protein_coding	PXT1
protein_coding	DHRS9
protein_coding	C9orf102
protein_coding	PRR27
protein_coding	NPIPL2
protein_coding	OSTC
protein_coding	PSMB8
protein_coding	TXNDC9

protein_coding	CCDC96
protein_coding	FAM230A
protein_coding	HAPLN4
protein_coding	C1QTNF9B
protein_coding	DNAJC28
protein_coding	GLMN
protein_coding	SLAIN2
protein_coding	VWA2
protein_coding	KRIT1
protein_coding	CARD11
protein_coding	ZNF638
protein_coding	GFM1
protein_coding	SLC7A7
protein_coding	NAA40
protein_coding	RAB33B
protein_coding	ASPSCR1
protein_coding	FMNL1
protein_coding	STAT2
protein_coding	POLR2C
protein_coding	MDH2
protein_coding	HIST1H3C
protein_coding	NKX6-3
protein_coding	OSGEPL1
protein_coding	HIST2H4B
protein_coding	LYPD6B
protein_coding	CYP24A1
protein_coding	ARHGEF33
protein_coding	LGI2
protein_coding	OR51S1
protein_coding	ONECUT2
protein_coding	CYP2C8
protein_coding	HTR1D
protein_coding	CCDC57
protein_coding	ZSCAN21
protein_coding	GALP
protein_coding	PPIG
protein_coding	KIF2A
protein_coding	BRK1
protein_coding	ECI1
protein_coding	DEFB127
protein_coding	ERI1
protein_coding	EOGT
protein_coding	DKFZP761J1410
protein_coding	IGSF21
protein_coding	C12orf54
protein_coding	PCDH18
protein_coding	DUS2

protein_coding	B9D2
protein_coding	OR1B1
protein_coding	CRYZ
protein_coding	NHEJ1
protein_coding	TREML4
protein_coding	RBPMS
protein_coding	NUDT1
protein_coding	AL121963.1
protein_coding	LEFTY1
protein_coding	RNF157
protein_coding	GAGE2D
protein_coding	FAM149A
protein_coding	POLD1
protein_coding	TAS2R41
protein_coding	OR2D2
protein_coding	ATP5G1
protein_coding	RASL11B
protein_coding	MTRNR2L13
protein_coding	WDR16
protein_coding	SLC6A4
protein_coding	SNRPA
protein_coding	KCNK9
protein_coding	LRRC28
protein_coding	MTHFS
protein_coding	EFCAB12
protein_coding	MYBL1
protein_coding	FLJ27365
protein_coding	AHCYL2
protein_coding	IQCF6
protein_coding	BAG6
protein_coding	NDUFS6
protein_coding	FAM69C
protein_coding	CENPW
protein_coding	PRM3
protein_coding	RNF103-CHMP3
protein_coding	NOS2
protein_coding	FAM72C
protein_coding	DLL1
protein_coding	TMEM45A
protein_coding	ADAMTS5
protein_coding	AC068620.1
protein_coding	DHCR24
protein_coding	RNASE4
protein_coding	KLK10
protein_coding	LINC00998
protein_coding	KLHL17
protein_coding	SAMD8

protein_coding	FAM166A
protein_coding	DOK5
protein_coding	CHRNB4
protein_coding	MON2
protein_coding	SLC44A2
protein_coding	TP53I3
protein_coding	LRP2
protein_coding	LECT1
protein_coding	LPCAT3
protein_coding	ANKRD26
protein_coding	LINC00346
protein_coding	NANOG
protein_coding	CCDC102A
protein_coding	CTLA4
protein_coding	RPS4X
protein_coding	BCL9
protein_coding	EIF1AX
protein_coding	HERC2
protein_coding	FLJ45513
protein_coding	JAK2
protein_coding	AC187652.1
protein_coding	TRMT112
protein_coding	SLC17A9
protein_coding	PARD6A
protein_coding	TUBB4B
protein_coding	AX747140
protein_coding	AMH
protein_coding	PLXNA1
protein_coding	EXOSC5
protein_coding	NUDT13
protein_coding	LOC284454
protein_coding	MRPS7
protein_coding	KRR1
protein_coding	C5orf20
protein_coding	TRIB3
protein_coding	EBAG9
protein_coding	KRTAP12-2
protein_coding	SMYD1
protein_coding	CILP2
protein_coding	HINT3
protein_coding	SH2D1A
protein_coding	LOC101929372
protein_coding	INO80C
protein_coding	CCDC113
protein_coding	TMEM87A
protein_coding	C4orf47
protein_coding	GMFG

protein_coding	MYRIP
protein_coding	NEDD9
protein_coding	ZSCAN32
protein_coding	LUC7L3
protein_coding	PHYHIP
protein_coding	PSME3
protein_coding	AQP8
protein_coding	PSPC1
protein_coding	MXI1
protein_coding	CCDC129
protein_coding	MTRNR2L9
protein_coding	PGAM5
protein_coding	DGKE
protein_coding	GJA8
protein_coding	DAZ3
protein_coding	CCR2
protein_coding	RPL36
protein_coding	WDR47
protein_coding	AC096644.1
protein_coding	CD70
protein_coding	TCEAL4
protein_coding	SLC10A6
protein_coding	PLAT
protein_coding	BRCA2
protein_coding	WIPI2
protein_coding	CUL1
protein_coding	FAM179A
protein_coding	FBLN1
protein_coding	PIP
protein_coding	MAP3K12
protein_coding	PRKD2
protein_coding	CT45A4
protein_coding	TNS1
protein_coding	AK3
protein_coding	MRPS11
protein_coding	WDFY2
protein_coding	AC132186.1
protein_coding	USP46
protein_coding	BSX
protein_coding	TCIRG1
protein_coding	GPR25
protein_coding	IQCF2
protein_coding	AC006449.1
protein_coding	CCT7
protein_coding	OR10J4
protein_coding	TEX11
protein_coding	KLHL2

protein_coding	AC027228.1
protein_coding	C12orf23
protein_coding	OR51G2
protein_coding	ARHGAP25
protein_coding	TMEM59L
protein_coding	KRTAP26-1
protein_coding	CENPBD1
protein_coding	DSC1
protein_coding	DIRC3
protein_coding	OR2T29
protein_coding	MAGEB3
protein_coding	RNF138
protein_coding	STK10
protein_coding	SYCP2L
protein_coding	ALPK1
protein_coding	ADD1
protein_coding	MARCH6
protein_coding	SEC63
protein_coding	B3GNT8
protein_coding	ARFGEF3
protein_coding	SLPI
protein_coding	IRX1
protein_coding	KRTAP21-1
protein_coding	CFAP61
protein_coding	ARHGEF4
protein_coding	CDH16
protein_coding	ZNF608
protein_coding	NDN
protein_coding	SORBS2
protein_coding	RAB1A
protein_coding	SOX12
protein_coding	PSMA5
protein_coding	MRGPRD
protein_coding	RBM11
protein_coding	HSPA9
protein_coding	MUC21
protein_coding	PPP1R12B
protein_coding	RP11-293M10.1
protein_coding	ENPP3
protein_coding	CD109
protein_coding	TCERG1
protein_coding	KIF24
protein_coding	GNG10
protein_coding	DHX15
protein_coding	ADD3
protein_coding	DMP1
protein_coding	IL17RD

protein_coding	NUDT15
protein_coding	MARCH3
protein_coding	CCT4
protein_coding	CLDN16
protein_coding	TRAPPC9
protein_coding	PRSS8
protein_coding	VIM
protein_coding	TTI1
protein_coding	NAPSA
protein_coding	GREB1
protein_coding	AX748261
protein_coding	AC119673.1
protein_coding	C6orf62
protein_coding	ARMS2
protein_coding	CHMP4A
protein_coding	GCNT1
protein_coding	GPR142
protein_coding	MRPL15
protein_coding	AMIGO2
protein_coding	MAP1B
protein_coding	RNF166
protein_coding	XPNPEP2
protein_coding	SH3BP5
protein_coding	ABCA8
protein_coding	RAB11FIP1
protein_coding	COLCA1
protein_coding	CCDC115
protein_coding	WDR41
protein_coding	TTC25
protein_coding	CCDC158
protein_coding	CTXN2
protein_coding	AKT1S1
protein_coding	ARHGDIG
protein_coding	ANXA4
protein_coding	GOLGA6L20
protein_coding	SLC24A3
protein_coding	KRTAP10-3
protein_coding	MUM1
protein_coding	DIRAS1
protein_coding	KRTAP22-1
protein_coding	HCN2
protein_coding	MAGEB17
protein_coding	AL033381.1
protein_coding	PRAMEF25
protein_coding	ERN1
protein_coding	BAI3
protein_coding	C17orf74

protein_coding	DNAJB5
protein_coding	CCDC69
protein_coding	GOLGA6L1
protein_coding	HEATR5B
protein_coding	PTS
protein_coding	MTO1
protein_coding	SBK1
protein_coding	NSUN3
protein_coding	SH3KBP1
protein_coding	C9orf156
protein_coding	HRASLS
protein_coding	KDSR
protein_coding	RP11-297M9.1
protein_coding	NR0B1
protein_coding	GGPS1
protein_coding	JMJD7-PLA2G4B
protein_coding	HIVEP2
protein_coding	KLRB1
protein_coding	DERL3
protein_coding	MTG1
protein_coding	ICOSLG
protein_coding	DBNDD1
protein_coding	C2CD3
protein_coding	C18orf64
protein_coding	KCNIP1
protein_coding	NME4
protein_coding	DNAH9
protein_coding	AC004076.7
protein_coding	ACRBP
protein_coding	CRTC3
protein_coding	UBE2QL1
protein_coding	MGME1
protein_coding	TMEM186
protein_coding	WARS
protein_coding	BEST4
protein_coding	SMIM19
protein_coding	RHOBTB3
protein_coding	TMPRSS13
protein_coding	KLB
protein_coding	AC016251.1
protein_coding	ADGRA2
protein_coding	COLQ
protein_coding	KRT9
protein_coding	KIAA1468
protein_coding	FGF12
protein_coding	RP11-644F5.10
protein_coding	CDK15

protein_coding	SLC30A4
protein_coding	TIMM21
protein_coding	ZNF362
protein_coding	GPR84
protein_coding	INSL3
protein_coding	HAND2
protein_coding	MFAP4
protein_coding	ANKK1
protein_coding	USP8
protein_coding	GNAI3
protein_coding	MEDAG
protein_coding	STAT1
protein_coding	RNASET2
protein_coding	TTYH2
protein_coding	GRIK2
protein_coding	KLK12
protein_coding	SFXN2
protein_coding	AMMECR1
protein_coding	ATG101
protein_coding	ITGA1
protein_coding	PALM2-AKAP2
protein_coding	AC011500.1
protein_coding	C3orf84
protein_coding	PDF
protein_coding	PDLIM4
protein_coding	SERPINF1
protein_coding	WBP5
protein_coding	FDCSP
protein_coding	RNF44
protein_coding	CHCHD4
protein_coding	CDIP1
protein_coding	H1FX
protein_coding	CFAP44
protein_coding	TULP4
protein_coding	FZD3
protein_coding	C2orf54
protein_coding	PAPL
protein_coding	KIAA1143
protein_coding	FOLR3
protein_coding	CYP27B1
protein_coding	NLRC3
protein_coding	ST14
protein_coding	RMDN2
protein_coding	NIPAL2
protein_coding	COX6B1
protein_coding	CALCB
protein_coding	TPMT

protein_coding	MAGEB16
protein_coding	KCNV2
protein_coding	ASMT
protein_coding	ARMCX6
protein_coding	BACE2
protein_coding	LDAH
protein_coding	SLC9A8
protein_coding	CLEC2D
protein_coding	RGS18
protein_coding	ISPD
protein_coding	IGFL1
protein_coding	SLC2A4
protein_coding	TRIM25
protein_coding	C5orf38
protein_coding	DMTN
protein_coding	RBM39
protein_coding	C7orf26
protein_coding	DKFZp434K1323
protein_coding	FKBP7
protein_coding	RNF212B
protein_coding	SLC35F3
protein_coding	EMP3
protein_coding	UBL7
protein_coding	UBXN4
protein_coding	AC012485.1
protein_coding	PAX7
protein_coding	PRR11
protein_coding	EYA2
protein_coding	AK055601
protein_coding	TBCC
protein_coding	ILF2
protein_coding	PICALM
protein_coding	PROC
protein_coding	FAM206A
protein_coding	FGF11
protein_coding	FAM216A
protein_coding	ZBTB5
protein_coding	PHAX
protein_coding	BTNL10
protein_coding	DHX16
protein_coding	TMEM184C
protein_coding	C5orf66
protein_coding	PTAR1
protein_coding	CRAMP1L
protein_coding	SERPINB9
protein_coding	LOC101927844
protein_coding	BTN3A1

protein_coding	YAF2
protein_coding	WRAP53
protein_coding	ZNF654
protein_coding	PLEKHA4
protein_coding	PPP1R21
protein_coding	GOLGA6L18
protein_coding	SLC35A5
protein_coding	PDE3A
protein_coding	CNTNAP2
protein_coding	CEACAM21
protein_coding	SLC6A15
protein_coding	ITGA3
protein_coding	GAS1
protein_coding	CARD16
protein_coding	RP11-1035H13.3
protein_coding	TMC2
protein_coding	SLC5A7
protein_coding	SIT1
protein_coding	XPO4
protein_coding	BAP1
protein_coding	LDHAL6B
protein_coding	C21orf119
protein_coding	ZNF264
protein_coding	NRG4
protein_coding	ASXL3
protein_coding	SNX31
protein_coding	SLAMF6
protein_coding	KCNJ14
protein_coding	EFNB1
protein_coding	CRELD1
protein_coding	REG1B
protein_coding	ADAR
protein_coding	SIX1
protein_coding	PCDHA13
protein_coding	AFAP1
protein_coding	UCP3
protein_coding	WTAP
protein_coding	HPSE2
protein_coding	CLMN
protein_coding	RP11-683L23.1
protein_coding	CDHR2
protein_coding	ATOH1
protein_coding	FECH
protein_coding	RASL12
protein_coding	KRT27
protein_coding	UBE2B
protein_coding	DIO1

protein_coding	FAM227B
protein_coding	ELSPBP1
protein_coding	MPDZ
protein_coding	MAP3K13
protein_coding	IGSF1
protein_coding	TNFSF14
protein_coding	FPR2
protein_coding	ASB8
protein_coding	PSMG3
protein_coding	B3GALT6
protein_coding	PKIB
protein_coding	C6orf136
protein_coding	SH3BGRL3
protein_coding	ASTN1
protein_coding	PAQR5
protein_coding	TMX2
protein_coding	YPEL5
protein_coding	EMP1
protein_coding	TMEM243
protein_coding	FOLR2
protein_coding	GLB1L
protein_coding	AKAP14
protein_coding	THYN1
protein_coding	GKN2
protein_coding	INSM1
protein_coding	ERAS
protein_coding	NDUFS8
protein_coding	NEO1
protein_coding	LRCH4
protein_coding	CD302
protein_coding	ARL13B
protein_coding	EGF
protein_coding	COL6A1
protein_coding	CHRNA1
protein_coding	CRKL
protein_coding	C7orf61
protein_coding	SOX18
protein_coding	CD3D
protein_coding	SERF2
protein_coding	COG7
protein_coding	C6orf70
protein_coding	TEX38
protein_coding	MGAT4A
protein_coding	DYX1C1
protein_coding	PTPRM
protein_coding	ZNF649
protein_coding	NDUFA7

protein_coding	SLC52A1
protein_coding	COX19
protein_coding	AK296947
protein_coding	ZBTB34
protein_coding	TMEM216
protein_coding	TMEM262
protein_coding	PRKAR1B
protein_coding	ZNF552
protein_coding	SPIB
protein_coding	TEX35
protein_coding	C8orf86
protein_coding	KCTD8
protein_coding	PLCXD2
protein_coding	RPS6KA3
protein_coding	ASIP
protein_coding	DMRTC2
protein_coding	TNIK
protein_coding	DNAJC5
protein_coding	YY1
protein_coding	TNN
protein_coding	P3H2
protein_coding	DNASE2B
protein_coding	ZNF491
protein_coding	ABHD6
protein_coding	RNF14
protein_coding	PCBP3
protein_coding	FBXO5
protein_coding	MORC4
protein_coding	GINS2
protein_coding	CALD1
protein_coding	MXRA5
protein_coding	NUDCD2
protein_coding	KCNH7
protein_coding	CCL4L1
protein_coding	IPO8
protein_coding	DCAF12
protein_coding	ANKRD13C
protein_coding	EFCAB14
protein_coding	R3HDML
protein_coding	MAP4K5
protein_coding	GJA1
protein_coding	LSM11
protein_coding	LBHD1
protein_coding	SEC31B
protein_coding	SLC25A35
protein_coding	DBP
protein_coding	AC006538.4

protein_coding	AKR1C2
protein_coding	GAL3ST3
protein_coding	RPS15A
protein_coding	LRFN1
protein_coding	IST1
protein_coding	IL22
protein_coding	KRTAP5-7
protein_coding	PIGG
protein_coding	SAMM50
protein_coding	PRKRIR
protein_coding	KCTD2
protein_coding	PSCA
protein_coding	WDR87
protein_coding	GPR34
protein_coding	ADGRB2
protein_coding	PPP1R13L
protein_coding	C9orf172
protein_coding	C11orf40
protein_coding	GLTSCR1
protein_coding	MOSPD1
protein_coding	HUWE1
protein_coding	SULT1B1
protein_coding	RNF8
protein_coding	TMEM9B
protein_coding	PSIP1
protein_coding	CYP20A1
protein_coding	GGCT
protein_coding	MED7
protein_coding	UNC5A
protein_coding	SH3PXD2A
protein_coding	PROP1
protein_coding	OR52B2
protein_coding	DEFB123
protein_coding	MAPKAP1
protein_coding	AK1
protein_coding	CASP2
protein_coding	RND3
protein_coding	DAOA
protein_coding	C6orf183
protein_coding	EYA3
protein_coding	CNTLN
protein_coding	CSF2RB
protein_coding	CTNNA1
protein_coding	EHD3
protein_coding	TBC1D23
protein_coding	TANK
protein_coding	GRAMD4

protein_coding	THOC3
protein_coding	MROH2A
protein_coding	MLLT4
protein_coding	TMEM151A
protein_coding	AK098333
protein_coding	STAMBP
protein_coding	BSN
protein_coding	MPZL2
protein_coding	GOLGA8N
protein_coding	TEX29
protein_coding	TCAP
protein_coding	C2CD2L
protein_coding	PIEZO2
protein_coding	GJD3
protein_coding	GRAP2
protein_coding	RORC
protein_coding	PRKCI
protein_coding	NTRK1
protein_coding	GORASP1
protein_coding	CDH5
protein_coding	F2R
protein_coding	GPR162
protein_coding	NDUFB10
protein_coding	PIP5K1C
protein_coding	LOC388692
protein_coding	SPINK8
protein_coding	RRAGD
protein_coding	SCAPER
protein_coding	CEA
protein_coding	F2RL1
protein_coding	RTFDC1
protein_coding	IFI6
protein_coding	DNAAF5
protein_coding	APLN
protein_coding	GRB7
protein_coding	ENTPD3
protein_coding	NAA30
protein_coding	OXCT2
protein_coding	GSTM3
protein_coding	DBR1
protein_coding	PRADC1
protein_coding	VPS35
protein_coding	CYP21A2
protein_coding	PMFBP1
protein_coding	PIH1D1
protein_coding	NTN5
protein_coding	MAN2A1

protein_coding	TMX3
protein_coding	ELF5
protein_coding	COPS8
protein_coding	GATA4
protein_coding	GAB1
protein_coding	RASIP1
protein_coding	GRIK1-AS2
protein_coding	HIST1H4E
protein_coding	PCDHGB2
protein_coding	SV2A
protein_coding	KRTAP4-1
protein_coding	KAZALD1
protein_coding	MAP3K7CL
protein_coding	POLR3F
protein_coding	SLC16A11
protein_coding	PPIP5K1
protein_coding	MMP24
protein_coding	PCDHGA10
protein_coding	CACNA2D4
protein_coding	CHTF18
protein_coding	C1orf213
protein_coding	CABP5
protein_coding	CNGB3
protein_coding	EPM2A
protein_coding	FBXL12
protein_coding	GML
protein_coding	BANF1
protein_coding	RALGPS2
protein_coding	CCDC184
protein_coding	STRC
protein_coding	CHDC2
protein_coding	DTNB
protein_coding	REXO1L10P
protein_coding	RECK
protein_coding	UBE2K
protein_coding	CYC1
protein_coding	ATP9A
protein_coding	RP11-481A20.11
protein_coding	GGNBP2
protein_coding	MARCH8
protein_coding	PSMB10
protein_coding	NLK
protein_coding	HSPA4
protein_coding	TERF1
protein_coding	CSDE1
protein_coding	KRTDAP
protein_coding	MBD5

protein_coding	TSPAN14
protein_coding	OSGIN2
protein_coding	ZNF622
protein_coding	MARCH4
protein_coding	ALPL
protein_coding	DDX28
protein_coding	TMED7
protein_coding	IARS2
protein_coding	ZMAT1
protein_coding	UQCRC2
protein_coding	MTRF1L
protein_coding	SLC14A1
protein_coding	MYLK4
protein_coding	AC103809.2
protein_coding	BC033739
protein_coding	TMEM230
protein_coding	TGFBR3
protein_coding	HACD4
protein_coding	MGAM
protein_coding	ANP32E
protein_coding	ACAA1
protein_coding	C4B_2
protein_coding	FAM208A
protein_coding	RP11-503N18.3
protein_coding	CALCA
protein_coding	AGRP
protein_coding	NKIRAS2
protein_coding	C4orf27
protein_coding	AP3D1
protein_coding	AX748249
protein_coding	SEPT7
protein_coding	KRT37
protein_coding	SLIRP
protein_coding	TET1
protein_coding	ASH2L
protein_coding	CENPO
protein_coding	TMEM246
protein_coding	SNRPD1
protein_coding	APOBEC3C
protein_coding	PET112
protein_coding	PIAS3
protein_coding	SUSD2
protein_coding	ANO3
protein_coding	C4orf22
protein_coding	DDX19A
protein_coding	ZNF70
protein_coding	LIX1L

protein_coding	SH3RF1
protein_coding	SV2C
protein_coding	CCNY
protein_coding	SRSF11
protein_coding	RPL41
protein_coding	NAPA
protein_coding	RARRES3
protein_coding	RAI14
protein_coding	HMMR
protein_coding	HYDIN
protein_coding	VMP1
protein_coding	PCDHGB4
protein_coding	CBLN2
protein_coding	SSX7
protein_coding	PCDHA9
protein_coding	TRIP12
protein_coding	TXNDC15
protein_coding	KIAA1217
protein_coding	CPD
protein_coding	PDLIM1
protein_coding	ILKAP
protein_coding	OR2A4
protein_coding	PTK6
protein_coding	AC026703.1
protein_coding	CDK2
protein_coding	SAP25
protein_coding	KLHL34
protein_coding	PTPN3
protein_coding	BMPER
protein_coding	YAP1
protein_coding	ZYG11B
protein_coding	PRKAA1
protein_coding	VDAC3
protein_coding	SPRR2F
protein_coding	HIRA
protein_coding	ADGRG1
protein_coding	UGGT2
protein_coding	DEFB132
protein_coding	C2orf48
protein_coding	STEAP2
protein_coding	FAM188B
protein_coding	CAPN2
protein_coding	MRGPRX1
protein_coding	RAD52
protein_coding	STAG2
protein_coding	NSFL1C
protein_coding	KAZN

protein_coding	MTMR2
protein_coding	OR8H1
protein_coding	DIXDC1
protein_coding	TBCA
protein_coding	MYL6B
protein_coding	LOC200726
protein_coding	CAB39L
protein_coding	RBFA
protein_coding	GBP6
protein_coding	PCED1B
protein_coding	PRAMEF6
protein_coding	GTSE1
protein_coding	ARNT
protein_coding	UXS1
protein_coding	FAM188A
protein_coding	AL590822.2
protein_coding	SLC26A2
protein_coding	USP24
protein_coding	DYRK2
protein_coding	SLC22A11
protein_coding	KRT26
protein_coding	HVCN1
protein_coding	NR2F2
protein_coding	IBA57
protein_coding	COX7A1
protein_coding	GPR171
protein_coding	REXO4
protein_coding	NDEL1
protein_coding	SPRR2D
protein_coding	CYB561
protein_coding	SOX17
protein_coding	HSP90AA1
protein_coding	TNNI2
protein_coding	JSRP1
protein_coding	GGTLC3
protein_coding	NEMP1
protein_coding	MRFAP1L1
protein_coding	OR52W1
protein_coding	BCL2L10
protein_coding	FOXO6
protein_coding	GVQW1
protein_coding	AL133481.1
protein_coding	DNMT1
protein_coding	NCAM1
protein_coding	GSTP1
protein_coding	CLPTM1L
protein_coding	NDP

protein_coding	PMF1-BGLAP
protein_coding	HSF1
protein_coding	C3orf67
protein_coding	USP31
protein_coding	MFNG
protein_coding	OSBPL3
protein_coding	ACAT1
protein_coding	PFKFB1
protein_coding	KCTD1
protein_coding	ABRA
protein_coding	GLT6D1
protein_coding	BAIAP3
protein_coding	CDAN1
protein_coding	HAUS4
protein_coding	H2AFB3
protein_coding	PTGES
protein_coding	ZCCHC3
protein_coding	PCGF2
protein_coding	FA2H
protein_coding	RP11-116D17.1
protein_coding	FHL3
protein_coding	C2orf16
protein_coding	PDHX
protein_coding	LRRCC1
protein_coding	UQCRC1
protein_coding	SBNO1
protein_coding	ZNF12
protein_coding	CRB2
protein_coding	CRIP2
protein_coding	AC011298.1
protein_coding	DRAP1
protein_coding	PFDN1
protein_coding	COPB2
protein_coding	CYP26B1
protein_coding	ACO2
protein_coding	KIAA0319
protein_coding	USP27X
protein_coding	GOLGA6L6
protein_coding	GPBAR1
protein_coding	PAAF1
protein_coding	MYO5B
protein_coding	C5orf46
protein_coding	CENPL
protein_coding	MST1R
protein_coding	C1QTNF3
protein_coding	KIF20B
protein_coding	FBXO9

protein_coding	TRHR
protein_coding	TWF1
protein_coding	ECT2L
protein_coding	ACBD5
protein_coding	NDUFAF3
protein_coding	GPX4
protein_coding	FABP6
protein_coding	RABGAP1L
protein_coding	RAB3D
protein_coding	RASSF6
protein_coding	AATK
protein_coding	SLC25A40
protein_coding	CCRL1
protein_coding	CLEC18B
protein_coding	C18orf25
protein_coding	C4orf32
protein_coding	DKFZP779J2370
protein_coding	RANBP10
protein_coding	MYT1
protein_coding	ALDH16A1
protein_coding	SYPL1
protein_coding	FXYD5
protein_coding	COPS3
protein_coding	SFTPD
protein_coding	ALG11
protein_coding	MAGEC2
protein_coding	ACADSB
protein_coding	C1orf123
protein_coding	ABHD2
protein_coding	GRIA1
protein_coding	TCEAL8
protein_coding	RP11-321M21.3
protein_coding	PGLYRP1
protein_coding	DDX42
protein_coding	IBTK
protein_coding	ZNF587B
protein_coding	C9orf153
protein_coding	GCLC
protein_coding	AXIN2
protein_coding	SMAD2
protein_coding	LMNA
protein_coding	MED4
protein_coding	ARMCX1
protein_coding	RC3H2
protein_coding	XXbac-BPG181M17.5
protein_coding	PTPRC
protein_coding	GPRC5B

protein_coding	ARSI
protein_coding	AX747191
protein_coding	VPS36
protein_coding	REEP6
protein_coding	PLCB3
protein_coding	TMED10
protein_coding	FIGN
protein_coding	SPECC1
protein_coding	CRTC1
protein_coding	GABPB1
protein_coding	HK1
protein_coding	C12orf60
protein_coding	HECW1
protein_coding	ASPG
protein_coding	ADORA2A
protein_coding	ARHGEF2
protein_coding	FEM1C
protein_coding	SDSL
protein_coding	RPS6KL1
protein_coding	UBE3C
protein_coding	SKIV2L2
protein_coding	CDKN2AIPNL
protein_coding	SERTAD3
protein_coding	SLC25A29
protein_coding	DPYSL3
protein_coding	PRAMEF8
protein_coding	AK095700
protein_coding	TBC1D22B
protein_coding	CLIC4
protein_coding	VWCE
protein_coding	CIRBP
protein_coding	TOM1L1
protein_coding	DPY30
protein_coding	TRAPPC12
protein_coding	RPS19BP1
protein_coding	SAMD10
protein_coding	FCF1
protein_coding	NRDE2
protein_coding	MRPS24
protein_coding	SLC22A31
protein_coding	C11orf97
protein_coding	CTD-3203P2.2
protein_coding	IRF5
protein_coding	KAT8
protein_coding	SLC18A2
protein_coding	TMEM45B
protein_coding	RAB11A

protein_coding	NUTM2E
protein_coding	PHLDB1
protein_coding	ARFGEF1
protein_coding	GIGYF1
protein_coding	CNTNAP1
protein_coding	UNC13A
protein_coding	BIN3
protein_coding	FAM204A
protein_coding	SLAIN1
protein_coding	AC093157.1
protein_coding	SYDE2
protein_coding	RAB40C
protein_coding	ZBTB11
protein_coding	TSEN34
protein_coding	ADPRM
protein_coding	LIX1
protein_coding	PCDHAC2
protein_coding	SH3GLB2
protein_coding	SLC25A46
protein_coding	CD151
protein_coding	NOVA1
protein_coding	SERPINB13
protein_coding	MUC5AC
protein_coding	CT55
protein_coding	PTPRE
protein_coding	LMO4
protein_coding	HNRNPA2B1
protein_coding	GPR144
protein_coding	KRT16
protein_coding	HEBP2
protein_coding	DSCR4
protein_coding	SELM
protein_coding	PPP2R5A
protein_coding	CRIP1
protein_coding	MAEA
protein_coding	NAMPT
protein_coding	KCNT2
protein_coding	CHCHD7
protein_coding	KYNU
protein_coding	REPS1
protein_coding	PELI3
protein_coding	TXNRD3
protein_coding	MUC1
protein_coding	ZMYND8
protein_coding	UBN1
protein_coding	PDYN
protein_coding	SKA2

protein_coding	IGSF3
protein_coding	PSMC2
protein_coding	TRPT1
protein_coding	IMPA1
protein_coding	SPATA31A5
protein_coding	C10orf129
protein_coding	LOC441178
protein_coding	TRAF3IP1
protein_coding	ATE1
protein_coding	HMGA1
protein_coding	SHOX
protein_coding	P4HTM
protein_coding	PLD5
protein_coding	ZNF727
protein_coding	MAP3K8
protein_coding	JRKL
protein_coding	AP4S1
protein_coding	KCTD5
protein_coding	PLXDC1
protein_coding	GPC6
protein_coding	LGALS1
protein_coding	C7orf34
protein_coding	DARS
protein_coding	LBH
protein_coding	NREP
protein_coding	PCDHAC1
protein_coding	C1orf86
protein_coding	IGFBP6
protein_coding	WIPF1
protein_coding	RPS11
protein_coding	TAF11
protein_coding	ZNF428
protein_coding	RIMBP2
protein_coding	PDCD6
protein_coding	HRCT1
protein_coding	SLC28A1
protein_coding	FKBP14
protein_coding	FAM181A
protein_coding	C9orf147
protein_coding	WDR52
protein_coding	SKIDA1
protein_coding	SPRYD7
protein_coding	TRIM36
protein_coding	STK24
protein_coding	PRG4
protein_coding	CDS2
protein_coding	PAQR8

protein_coding	ZC2HC1C
protein_coding	CDCP2
protein_coding	GLIPR1L2
protein_coding	SOWAHC
protein_coding	F5
protein_coding	CPNE9
protein_coding	KCTD15
protein_coding	CASR
protein_coding	BAD
protein_coding	CDKAL1
protein_coding	ACSM2B
protein_coding	SIRPA
protein_coding	IGFBP2
protein_coding	FAM19A1
protein_coding	PFAS
protein_coding	CNOT7
protein_coding	UTP18
protein_coding	CCDC41
protein_coding	NFIL3
protein_coding	KRT3
protein_coding	P2RX5
protein_coding	PCDHA12
protein_coding	NECAB3
protein_coding	MGC57346-CRHR1
protein_coding	RAB31
protein_coding	CPSF3L
protein_coding	NR1I3
protein_coding	LOC440295
protein_coding	RANGRF
protein_coding	AIM2
protein_coding	ARHGEF10L
protein_coding	TRIM66
protein_coding	CNIH4
protein_coding	PPFIBP2
protein_coding	PDCD2
protein_coding	CALHM3
protein_coding	OR52L1
protein_coding	CLVS2
protein_coding	RIMS3
protein_coding	GRIN2B
protein_coding	BECN1
protein_coding	BNC2
protein_coding	NECAB2
protein_coding	FNBP1
protein_coding	TAPT1
protein_coding	KDELC2
protein_coding	FOXB1

protein_coding	CCDC97
protein_coding	ZSCAN5C
protein_coding	C16orf90
protein_coding	NPIPB7
protein_coding	PLCXD3
protein_coding	CCDC132
protein_coding	LNPEP
protein_coding	ACSL1
protein_coding	NAA50
protein_coding	ARFGAP2
protein_coding	IFT140
protein_coding	TMEM67
protein_coding	ARHGAP24
protein_coding	WWTR1
protein_coding	HNRNPH3
protein_coding	C1orf228
protein_coding	ARL6
protein_coding	IK
protein_coding	ADRA2A
protein_coding	TRNT1
protein_coding	GCHFR
protein_coding	ABCD3
protein_coding	ADCY7
protein_coding	CASP4
protein_coding	HIST2H2AA3
protein_coding	GTDC2
protein_coding	SPAG8
protein_coding	BHLHA9
protein_coding	MEIKIN
protein_coding	YWHAE
protein_coding	CRP
protein_coding	REM2
protein_coding	TGM6
protein_coding	FAM96A
protein_coding	RCOR2
protein_coding	ZNF519
protein_coding	ANKRD49
protein_coding	CYBA
protein_coding	ASIC2
protein_coding	RTBDN
protein_coding	C8orf82
protein_coding	NR1I2
protein_coding	C9orf41
protein_coding	C3orf17
protein_coding	LMBR1
protein_coding	TMEM239
protein_coding	CLPX

protein_coding	GAGE13
protein_coding	PIGT
protein_coding	FSTL1
protein_coding	ZNF205
protein_coding	PDE9A
protein_coding	CLC
protein_coding	FIP1L1
protein_coding	RAB13
protein_coding	FAM118A
protein_coding	MT1G
protein_coding	GBE1
protein_coding	TTLL13
protein_coding	VIMP
protein_coding	TMEM170A
protein_coding	EGLN1
protein_coding	MGAT4C
protein_coding	GMCL1
protein_coding	TTC28
protein_coding	AC004381.6
protein_coding	ADIPOR1
protein_coding	FBXO7
protein_coding	EAF2
protein_coding	TICRR
protein_coding	SLC16A2
protein_coding	ANGPT1
protein_coding	HBM
protein_coding	NAT8L
protein_coding	SPCS2
protein_coding	TNFRSF11B
protein_coding	DESI2
protein_coding	PTEN
protein_coding	DAD1
protein_coding	EBF1
protein_coding	POLE
protein_coding	MUT
protein_coding	CKLF-CMTM1
protein_coding	ZNF550
protein_coding	TMTC1
protein_coding	LRRC34
protein_coding	ELMOD1
protein_coding	CACNB4
protein_coding	ATG16L2
protein_coding	CYP1B1
protein_coding	KRBA2
protein_coding	RIBC1
protein_coding	KIAA0754
protein_coding	THG1L

protein_coding	ANKRD61
protein_coding	VPREB1
protein_coding	DUOXA2
protein_coding	SLC22A10
protein_coding	ZNF705G
protein_coding	NQO2
protein_coding	POC1B
protein_coding	EREG
protein_coding	C14orf142
protein_coding	ZCWPW1
protein_coding	OPN1SW
protein_coding	COL27A1
protein_coding	FAM129A
protein_coding	MYEF2
protein_coding	CAP2
protein_coding	PHF19
protein_coding	MLN
protein_coding	ARIH2
protein_coding	NAPRT
protein_coding	LPAR3
protein_coding	OR56B3P
protein_coding	TCEB3
protein_coding	HTATSF1
protein_coding	LAMTOR3
protein_coding	ITGB8
protein_coding	KRT39
protein_coding	SYCE1L
protein_coding	HAPLN1
protein_coding	GPR88
protein_coding	PSMD7
protein_coding	RASAL2
protein_coding	MMP25
protein_coding	ITGB3BP
protein_coding	GNPAT
protein_coding	PAK7
protein_coding	ULK4
protein_coding	FBXL15
protein_coding	Em:AC008101.5
protein_coding	USP7
protein_coding	ESYT2
protein_coding	SYTL4
protein_coding	NFASC
protein_coding	CEACAM1
protein_coding	PDS5A
protein_coding	LRRC2
protein_coding	VCAM1
protein_coding	NRG2

protein_coding	DCLK3
protein_coding	FAM178A
protein_coding	LRRC37
protein_coding	NEGR1
protein_coding	CFAP74
protein_coding	LYG1
protein_coding	FUNDC2
protein_coding	TMEM56
protein_coding	PAK1IP1
protein_coding	GCC2
protein_coding	HBB
protein_coding	SLURP1
protein_coding	UBE2L6
protein_coding	NOD1
protein_coding	AC012313.1
protein_coding	PVRIG
protein_coding	C1orf226
protein_coding	GLTP
protein_coding	FBXW2
protein_coding	UNC45B
protein_coding	THAP6
protein_coding	U2AF2
protein_coding	LGALS8
protein_coding	KDR
protein_coding	PAK3
protein_coding	ZDHHC21
protein_coding	LENG8
protein_coding	ASF1B
protein_coding	GADL1
protein_coding	CATX-2
protein_coding	KB-1507C5.2
protein_coding	DCANP1
protein_coding	ASIC4
protein_coding	MRPL30
protein_coding	PHB
protein_coding	EIF4E3
protein_coding	CD19
protein_coding	ACMSD
protein_coding	HEXB
protein_coding	VAMP5
protein_coding	FANCD2OS
protein_coding	EPAS1
protein_coding	IRS1
protein_coding	MRPL55
protein_coding	HADHB
protein_coding	MAT2A
protein_coding	POLR3G

protein_coding	ANO5
protein_coding	CPEB3
protein_coding	PTGS1
protein_coding	ZFP36L1
protein_coding	AHNAK2
protein_coding	DRP2
protein_coding	DEFB4A
protein_coding	TCEAL3
protein_coding	USP14
protein_coding	SLC1A1
protein_coding	C12orf75
protein_coding	HMGN5
protein_coding	MMEL1
protein_coding	OGDH
protein_coding	OR2T4
protein_coding	NDUFS2
protein_coding	C9orf38
protein_coding	CABIN1
protein_coding	APOA1
protein_coding	GUCA1A
protein_coding	CCDC77
protein_coding	CHSY1
protein_coding	UQCRB
protein_coding	GFRA1
protein_coding	AIMP2
protein_coding	TP53I11
protein_coding	CREG2
protein_coding	MTHFD1
protein_coding	BEND5
protein_coding	KRTAP4-4
protein_coding	DNAJC9
protein_coding	KRT222
protein_coding	DAB1
protein_coding	WSB2
protein_coding	PEMT
protein_coding	C19orf66
protein_coding	C19orf54
protein_coding	GRAMD1C
protein_coding	SCGN
protein_coding	PRAMEF2
protein_coding	GSTT1
protein_coding	NXN
protein_coding	PLEKHA3
protein_coding	TARBP2
protein_coding	TMEM105
protein_coding	GPHN
protein_coding	RNF26

protein_coding	CYP2D6
protein_coding	SGCB
protein_coding	NKX2-5
protein_coding	MKRN3
protein_coding	PCED1A
protein_coding	NRG3
protein_coding	C5orf49
protein_coding	PLP2
protein_coding	LAMTOR5
protein_coding	GRM8
protein_coding	UPK2
protein_coding	COA6
protein_coding	C12orf66
protein_coding	ADAM28
protein_coding	SLC30A6
protein_coding	CCDC142
protein_coding	CCDC167
protein_coding	NEK4
protein_coding	FBXL2
protein_coding	WBSCR27
protein_coding	LMCD1
protein_coding	NR6A1
protein_coding	PCDHA1
protein_coding	AIG1
protein_coding	TSSK3
protein_coding	OTOP3
protein_coding	CDC34
protein_coding	CCDC17
protein_coding	TIMM17B
protein_coding	USP47
protein_coding	HES4
protein_coding	CMKLR1
protein_coding	HSFY2
protein_coding	RAB42
protein_coding	FXYD4
protein_coding	KRT6C
protein_coding	IFT74
protein_coding	ACBD4
protein_coding	SEPT10
protein_coding	UGT3A1
protein_coding	PKIG
protein_coding	RPE65
protein_coding	C7
protein_coding	HERPUD2
protein_coding	ZNF586
protein_coding	LSM3
protein_coding	TIGIT

protein_coding	IER2
protein_coding	ACOT4
protein_coding	KRTAP20-2
protein_coding	ZNF585A
protein_coding	RIMS4
protein_coding	EXOC3L1
protein_coding	TPRX2P
protein_coding	FAM72D
protein_coding	GRHPR
protein_coding	LINGO2
protein_coding	IPO13
protein_coding	RDH11
protein_coding	SLC36A2
protein_coding	RP11-379H8.1
protein_coding	RNASEK-C17orf49
protein_coding	REG3G
protein_coding	C17orf66
protein_coding	NRROS
protein_coding	MOGAT3
protein_coding	KRTAP2-2
protein_coding	LPCAT4
protein_coding	TMEM100
protein_coding	CFB
protein_coding	CDH24
protein_coding	POLR2J
protein_coding	SAP18
protein_coding	LRRTM2
protein_coding	ERBB4
protein_coding	GALNT12
protein_coding	DPYD
protein_coding	BC132797
protein_coding	NUFIP1
protein_coding	ACP1
protein_coding	C17orf53
protein_coding	DHPS
protein_coding	IKBKG
protein_coding	RSRC2
protein_coding	RGL1
protein_coding	ADGRE3
protein_coding	POMGNT2
protein_coding	CCDC147
protein_coding	THOC6
protein_coding	KDM5A
protein_coding	C5orf30
protein_coding	DNAJB1
protein_coding	MRPL18
protein_coding	SEPN1

protein_coding	CDC37L1
protein_coding	MTERF4
protein_coding	SPAG9
protein_coding	SMN1
protein_coding	MAT1A
protein_coding	MEA1
protein_coding	GRK7
protein_coding	HAX1
protein_coding	FAM167B
protein_coding	ORAI1
protein_coding	EMR2
protein_coding	FIS1
protein_coding	EIF3H
protein_coding	SLC7A4
protein_coding	UVSSA
protein_coding	FAAH
protein_coding	CRYL1
protein_coding	ZCCHC10
protein_coding	HTN3
protein_coding	RTN4IP1
protein_coding	ATP6V1H
protein_coding	ASB16
protein_coding	LPCAT2
protein_coding	CGB2
protein_coding	RDH14
protein_coding	RNF7
protein_coding	POU3F1
protein_coding	PEX2
protein_coding	LBR
protein_coding	CARHSP1
protein_coding	C7orf65
protein_coding	RPL3
protein_coding	EFCAB4B
protein_coding	VPS25
protein_coding	OR8B3
protein_coding	SDR42E1
protein_coding	PPP4R3B
protein_coding	MSLNL
protein_coding	ZNF559
protein_coding	HOXD4
protein_coding	PEX6
protein_coding	TMEM187
protein_coding	HCG27
protein_coding	KXD1
protein_coding	EXTL1
protein_coding	ASPDH
protein_coding	PCOLCE

protein_coding	CHGB
protein_coding	SCXB
protein_coding	DNAJC24
protein_coding	FAM180A
protein_coding	DNMT3L
protein_coding	EFHD1
protein_coding	LRIG3
protein_coding	ELAC1
protein_coding	ABI3BP
protein_coding	KRBOX1
protein_coding	SMIM3
protein_coding	ZACN
protein_coding	GFRA2
protein_coding	EVI5
protein_coding	APCS
protein_coding	SEC24A
protein_coding	MALSU1
protein_coding	DHRS12
protein_coding	CTNNA2
protein_coding	SPRR1B
protein_coding	CREB3
protein_coding	NIPA2
protein_coding	TPP1
protein_coding	GTF2F2
protein_coding	CEBPZ
protein_coding	NADKD1
protein_coding	CHRNA9
protein_coding	PHF24
protein_coding	KIAA0146
protein_coding	SLC36A1
protein_coding	SETBP1
protein_coding	ERICH6B
protein_coding	NGFR
protein_coding	SLN
protein_coding	TSPAN9
protein_coding	PNOC
protein_coding	DNAH17-AS1
protein_coding	EBPL
protein_coding	BAZ1A
protein_coding	RPL39
protein_coding	PCM1
protein_coding	PTPLB
protein_coding	MTA3
protein_coding	PVALB
protein_coding	NAV3
protein_coding	FBXO18
protein_coding	APEH

protein_coding	CLEC5A
protein_coding	LMNB2
protein_coding	TOMM20
protein_coding	MYO3B
protein_coding	PURB
protein_coding	AACS
protein_coding	PP2D1
protein_coding	JMJD1C
protein_coding	DNAJC14
protein_coding	CC2D2B
protein_coding	AKR1C1
protein_coding	QPRT
protein_coding	PLA2G2E
protein_coding	HSPB11
protein_coding	MCM3
protein_coding	FANCF
protein_coding	SELV
protein_coding	DBX1
protein_coding	PHF6
protein_coding	RBM3
protein_coding	DSCAML1
protein_coding	FOXD4L1
protein_coding	SIRT5
protein_coding	KRTAP5-3
protein_coding	E2F3
protein_coding	RGN
protein_coding	MBNL3
protein_coding	HPX
protein_coding	SNAP23
protein_coding	FGF3
protein_coding	S100A12
protein_coding	ZNF721
protein_coding	CCDC186
protein_coding	SPOP
protein_coding	CNPY2
protein_coding	ITGB1
protein_coding	CA1
protein_coding	C11orf70
protein_coding	PABPC1L
protein_coding	NUP214
protein_coding	CGRRF1
protein_coding	RANBP3
protein_coding	SLX4
protein_coding	KIAA1429
protein_coding	DNAJC19
protein_coding	SPPL2B
protein_coding	BC032910

protein_coding	ABHD17B
protein_coding	ADORA2B
protein_coding	RAMP1
protein_coding	RABAC1
protein_coding	TMEM63B
protein_coding	WBP2
protein_coding	VIPR2
protein_coding	ELMOD2
protein_coding	MATN2
protein_coding	AL161645.2
protein_coding	LRCOL1
protein_coding	GPR115
protein_coding	SATB1
protein_coding	RPL17-C18orf32
protein_coding	MYADML2
protein_coding	CBFA2T2
protein_coding	PIGK
protein_coding	TMCO6
protein_coding	KALRN
protein_coding	CBX2
protein_coding	PATE2
protein_coding	VPS11
protein_coding	ZNF415
protein_coding	RP11-272B17.2
protein_coding	HLTF
protein_coding	OPRL1
protein_coding	NKPD1
protein_coding	FAM126B
protein_coding	PAPPA-AS1
protein_coding	ZMPSTE24
protein_coding	NOB1
protein_coding	OR4N2
protein_coding	RP11-872D17.8
protein_coding	ANKRD20A4
protein_coding	FAM222B
protein_coding	C4orf3
protein_coding	AC006156.1
protein_coding	SPG20OS
protein_coding	RAB2A
protein_coding	CNTNAP4
protein_coding	PRKAB2
protein_coding	ANXA5
protein_coding	MAP4K1
protein_coding	MS4A10
protein_coding	TMC6
protein_coding	ANKS1B
protein_coding	SPATA8

protein_coding	TRIM74
protein_coding	RPL23A
protein_coding	NUP188
protein_coding	CYP2D7P1
protein_coding	DTX2
protein_coding	TTLL9
protein_coding	PGRMC2
protein_coding	GSE1
protein_coding	TMEM150A
protein_coding	CNIH1
protein_coding	SELT
protein_coding	DRD1
protein_coding	ZC3H7A
protein_coding	CPNE1
protein_coding	COMMD1
protein_coding	LITAF
protein_coding	NPDC1
protein_coding	RRAS2
protein_coding	DNA2
protein_coding	MMAB
protein_coding	JPH4
protein_coding	ALG3
protein_coding	PCDHGA3
protein_coding	UBIAD1
protein_coding	SGCA
protein_coding	NQO1
protein_coding	TICAM2
protein_coding	RSPH14
protein_coding	EEF1A1
protein_coding	AP1B1
protein_coding	PKDREJ
protein_coding	C14orf37
protein_coding	ADAMTS18
protein_coding	ZNF583
protein_coding	LY75
protein_coding	NTN1
protein_coding	ERP44
protein_coding	PCSK1
protein_coding	DZIP1
protein_coding	TTC7A
protein_coding	EPB42
protein_coding	RFTN1
protein_coding	ZNF358
protein_coding	BICD2
protein_coding	BANF2
protein_coding	CXorf21
protein_coding	DDI2

protein_coding	CD53
protein_coding	TBCK
protein_coding	HNRNPAB
protein_coding	TMPRSS6
protein_coding	KLHL5
protein_coding	CCSER2
protein_coding	SEC22A
protein_coding	AQP7
protein_coding	GBP4
protein_coding	RANBP1
protein_coding	SNW1
protein_coding	CMC2
protein_coding	RPUSD1
protein_coding	PSMB3
protein_coding	MYO1C
protein_coding	C12orf55
protein_coding	PFN1
protein_coding	HIBADH
protein_coding	BAHCC1
protein_coding	BTC
protein_coding	C4orf46
protein_coding	AFF2
protein_coding	PFKFB2
protein_coding	MBIP
protein_coding	RBMS2
protein_coding	MARCH10
protein_coding	NOS3
protein_coding	EIF2A
protein_coding	ARL6IP6
protein_coding	CDKL2
protein_coding	PLEKHD1
protein_coding	ADAMTSL2
protein_coding	SULF1
protein_coding	RANBP9
protein_coding	JRK
protein_coding	PSPN
protein_coding	RP11-195B21.3
protein_coding	DAPK3
protein_coding	HMGN3
protein_coding	ENO4
protein_coding	AL078585.1
protein_coding	EIF2S3L
protein_coding	PSG4
protein_coding	COL22A1
protein_coding	KIF3C
protein_coding	OOEP
protein_coding	ICE1

protein_coding	LRIF1
protein_coding	COPA
protein_coding	MTMR6
protein_coding	KCNG1
protein_coding	CCAR2
protein_coding	NUDT19
protein_coding	A1CF
protein_coding	FOXB2
protein_coding	ZNF709
protein_coding	UTP14C
protein_coding	MRPS21
protein_coding	DIS3L
protein_coding	ZDBF2
protein_coding	GM2A
protein_coding	CCDC64B
protein_coding	PRAMEF7
protein_coding	TDP1
protein_coding	PTPN21
protein_coding	SPINT2
protein_coding	CSTB
protein_coding	TRAM2
protein_coding	ARL11
protein_coding	NPAS2
protein_coding	SLC25A36
protein_coding	ACKR3
protein_coding	DBN1
protein_coding	NME1
protein_coding	ALDH3B2
protein_coding	CHRND
protein_coding	PPIH
protein_coding	RAB4B
protein_coding	COQ2
protein_coding	CRYGA
protein_coding	IL20RA
protein_coding	HMCES
protein_coding	ENHO
protein_coding	MBD3L5
protein_coding	AIF1
protein_coding	ACSM6
protein_coding	RAB11FIP3
protein_coding	PPIL6
protein_coding	OXGR1
protein_coding	GTPBP10
protein_coding	RAB12
protein_coding	SPAG6
protein_coding	GARNL3
protein_coding	OR4K1

protein_coding	STX4
protein_coding	CCER1
protein_coding	ESAM
protein_coding	SPATS2L
protein_coding	TBC1D26
protein_coding	FARSB
protein_coding	H2AFZ
protein_coding	SETDB1
protein_coding	AP1AR
protein_coding	HEMK1
protein_coding	AGAP11
protein_coding	HIPK2
protein_coding	RGS10
protein_coding	ERVFRD-1
protein_coding	TP53TG3C
protein_coding	CHMP4B
protein_coding	CETP
protein_coding	ARHGAP40
protein_coding	YRDC
protein_coding	C1orf110
protein_coding	ATXN7L1
protein_coding	RPL29
protein_coding	FAM21D
protein_coding	EHD2
protein_coding	CCPG1
protein_coding	OXTR
protein_coding	APPBP2
protein_coding	AK293147
protein_coding	ALDH9A1
protein_coding	SF3B2
protein_coding	WDR46
protein_coding	RP11-302B13.5
protein_coding	SLC20A1
protein_coding	SLC12A3
protein_coding	DNAJC21
protein_coding	XPO1
protein_coding	CCDC6
protein_coding	PTCRA
protein_coding	TRAPPC3
protein_coding	PSMD1
protein_coding	RAB37
protein_coding	SP3
protein_coding	BC133018
protein_coding	KRTAP25-1
protein_coding	PRTN3
protein_coding	TATDN1
protein_coding	PIKFYVE

protein_coding	CGN
protein_coding	GSS
protein_coding	ANKRD65
protein_coding	ODF2L
protein_coding	LHFPL3
protein_coding	SLC5A2
protein_coding	RAB36
protein_coding	PPP4C
protein_coding	PARD6G
protein_coding	AGAP4
protein_coding	SEMA3B
protein_coding	KNCN
protein_coding	NCL
protein_coding	FAM215A
protein_coding	AC022400.2
protein_coding	DNAJC8
protein_coding	PIRT
protein_coding	SH3YL1
protein_coding	AURKC
protein_coding	BCL2L2
protein_coding	SPTSSA
protein_coding	WDR93
protein_coding	ZSWIM1
protein_coding	TMC4
protein_coding	MROH5
protein_coding	ITPKA
protein_coding	CRYZL1
protein_coding	MLXIPL
protein_coding	ATP5F1
protein_coding	LEO1
protein_coding	RDH8
protein_coding	OR51B4
protein_coding	KRTAP12-1
protein_coding	MAN1A2
protein_coding	RP11-187E13.1
protein_coding	HDAC7
protein_coding	UBE2V2
protein_coding	MEF2D
protein_coding	KMT2E
protein_coding	CD99
protein_coding	KHK
protein_coding	SIRT3
protein_coding	MRP63
protein_coding	ERF
protein_coding	LEF1
protein_coding	LRRN4
protein_coding	HIPK3

protein_coding	MEGF10
protein_coding	CALHM1
protein_coding	CYB5D2
protein_coding	LCMT2
protein_coding	RASD2
protein_coding	ADAM11
protein_coding	ECM1
protein_coding	C15orf56
protein_coding	YBX3
protein_coding	FAM195A
protein_coding	UBE2H
protein_coding	FOXF1
protein_coding	PPIL4
protein_coding	SLC25A1
protein_coding	CRELD2
protein_coding	ARHGAP33
protein_coding	EI24
protein_coding	RP11-89N17.1
protein_coding	MLF2
protein_coding	SNRPD2
protein_coding	IL4I1
protein_coding	GDF2
protein_coding	KLHL26
protein_coding	ABLIM1
protein_coding	SLC37A4
protein_coding	APOM
protein_coding	CYP1A1
protein_coding	SHOC2
protein_coding	TFRC
protein_coding	AMY1B
protein_coding	SAR1A
protein_coding	SLC25A47
protein_coding	DBF4
protein_coding	SURF1
protein_coding	SETD3
protein_coding	URGCP
protein_coding	CHPF
protein_coding	NUP37
protein_coding	PIK3C2A
protein_coding	MRPL34
protein_coding	PPIC
protein_coding	DMPK
protein_coding	POMT2
protein_coding	GALNT7
protein_coding	GOLT1A
protein_coding	UTP20
protein_coding	GPR183

protein_coding	TMEM11
protein_coding	UBE2NL
protein_coding	GLRX
protein_coding	CASC10
protein_coding	FGF10
protein_coding	DUSP28
protein_coding	TENM2
protein_coding	C3orf22
protein_coding	RFC3
protein_coding	DACH1
protein_coding	POLD3
protein_coding	PEX11G
protein_coding	SRP68
protein_coding	ADAM30
protein_coding	CACNA2D2
protein_coding	BC024682
protein_coding	IRX3
protein_coding	OXA1L
protein_coding	CSTF2T
protein_coding	TM9SF2
protein_coding	TEX15
protein_coding	ST3GAL6
protein_coding	EFCAB4A
protein_coding	ABCA4
protein_coding	NKX2-8
protein_coding	ABHD17A
protein_coding	LMO7
protein_coding	STAG1
protein_coding	IL1R1
protein_coding	PID1
protein_coding	TBX4
protein_coding	NFAT5
protein_coding	FAM110C
protein_coding	AC015987.2
protein_coding	ZAR1L
protein_coding	NFATC4
protein_coding	CEP290
protein_coding	CBWD2
protein_coding	U2SURP
protein_coding	LRP12
protein_coding	RILP
protein_coding	MAP2K6
protein_coding	PAPLN
protein_coding	DDB1
protein_coding	PELI2
protein_coding	BC068095
protein_coding	CELA2A

protein_coding	PEX19
protein_coding	EFNA4
protein_coding	RP11-80A15.1
protein_coding	PHF20L1
protein_coding	CHN2
protein_coding	HERC4
protein_coding	MED28
protein_coding	LOC441493
protein_coding	DNAJB12
protein_coding	CXCL6
protein_coding	GDI2
protein_coding	SRRM5
protein_coding	GAK
protein_coding	SHH
protein_coding	TSPO
protein_coding	CD160
protein_coding	FAM182B
protein_coding	RIPK2
protein_coding	FEM1B
protein_coding	TNFSF12-TNFSF13
protein_coding	DMD
protein_coding	GREB1L
protein_coding	ARMC10
protein_coding	TXNRD2
protein_coding	ZNF451
protein_coding	H1FNT
protein_coding	MAGEA10
protein_coding	SMG6
protein_coding	C2orf49
protein_coding	OXSM
protein_coding	DYDC2
protein_coding	SEC14L2
protein_coding	CDC42BPA
protein_coding	DIABLO
protein_coding	AC003006.7
protein_coding	CD164
protein_coding	PLEKHG4
protein_coding	TSEN15
protein_coding	LOC100133331
protein_coding	NCR3LG1
protein_coding	TNFSF15
protein_coding	SGSM1
protein_coding	RAB28
protein_coding	MSH2
protein_coding	CHD7
protein_coding	SMKR1
protein_coding	FMO1

protein_coding	ABR
protein_coding	SPDEF
protein_coding	TDG
protein_coding	MBD2
protein_coding	STX8
protein_coding	AC024592.12
protein_coding	LHX5
protein_coding	KHDRBS1
protein_coding	BNIP3L
protein_coding	PDZD8
protein_coding	UBE2T
protein_coding	SPANXC
protein_coding	SMIM10L1
protein_coding	ATP6V0A4
protein_coding	RAB5C
protein_coding	RCHY1
protein_coding	UBQLN2
protein_coding	SLC34A3
protein_coding	ARTN
protein_coding	SPTLC1
protein_coding	IKZF5
protein_coding	C11orf31
protein_coding	BC113726
protein_coding	VRK2
protein_coding	NR2F1
protein_coding	CA5B
protein_coding	RIMKLB
protein_coding	TCF20
protein_coding	LRRC14
protein_coding	FAM214A
protein_coding	POLR2L
protein_coding	ARID3A
protein_coding	NBPF8
protein_coding	OR1A2
protein_coding	CIDEA
protein_coding	RP11-1070N10.3
protein_coding	TMEM17
protein_coding	KRTAP5-8
protein_coding	CHUK
protein_coding	ZNF419
protein_coding	ZNF17
protein_coding	PDCD10
protein_coding	SLC18A3
protein_coding	PQLC2L
protein_coding	CEP128
protein_coding	KARS
protein_coding	PARP11

protein_coding	TUSC1
protein_coding	WNT2B
protein_coding	KCTD17
protein_coding	AHSA2
protein_coding	MERTK
protein_coding	C1GALT1C1
protein_coding	FAM72A
protein_coding	FAM129B
protein_coding	ORC3
protein_coding	AK6
protein_coding	C14orf132
protein_coding	PRLH
protein_coding	GAGE2A
protein_coding	FGFR2
protein_coding	SLC6A20
protein_coding	OR2F2
protein_coding	POU5F1
protein_coding	EDC3
protein_coding	PIBF1
protein_coding	FGF9
protein_coding	PCDHGA12
protein_coding	CLSTN3
protein_coding	PLIN3
protein_coding	NEK8
protein_coding	PALM2
protein_coding	IARS
protein_coding	TMEM173
protein_coding	TATDN3
protein_coding	LRRC47
protein_coding	DCST1
protein_coding	PCYT1A
protein_coding	DGUOK
protein_coding	CDK2AP1
protein_coding	Z98049.1
protein_coding	AC079354.2
protein_coding	FAM163A
protein_coding	ZNF302
protein_coding	ANKRD28
protein_coding	PI3
protein_coding	PPM1K
protein_coding	KCMF1
protein_coding	C2orf76
protein_coding	ADK
protein_coding	PDHB
protein_coding	CABP7
protein_coding	CXCR5
protein_coding	NFE2L1

protein_coding	ZDHHC20
protein_coding	RFX3
protein_coding	AC007421.1
protein_coding	STEAP3
protein_coding	PROK1
protein_coding	OR13C9
protein_coding	KCNG2
protein_coding	RTL1
protein_coding	PTPN23
protein_coding	NLGN4Y
protein_coding	NAE1
protein_coding	EIF3I
protein_coding	GGT7
protein_coding	PKNOX1
protein_coding	AC026310.1
protein_coding	TCEAL6
protein_coding	C11orf35
protein_coding	AK097370
protein_coding	PSMB9
protein_coding	TMEM50A
protein_coding	METTL1
protein_coding	TXNDC5
protein_coding	LMAN2L
protein_coding	ZNF680
protein_coding	STUB1
protein_coding	SRSF3
protein_coding	GCAT
protein_coding	RNF113A
protein_coding	VN1R1
protein_coding	SYS1
protein_coding	PSMB11
protein_coding	ABHD14B
protein_coding	MMS19
protein_coding	GMFB
protein_coding	NOP16
protein_coding	ACP6
protein_coding	TMEM198
protein_coding	REG3A
protein_coding	PCDHB15
protein_coding	CBLN3
protein_coding	TMOD1
protein_coding	S100G
protein_coding	DAB2
protein_coding	ZBTB39
protein_coding	CTB-133G6.1
protein_coding	HACE1
protein_coding	PEX1

protein_coding	AK125212
protein_coding	DHX36
protein_coding	KPNA5
protein_coding	LARS
protein_coding	SEPT11
protein_coding	MC5R
protein_coding	GIT2
protein_coding	TXNDC8
protein_coding	ZNF597
protein_coding	HDGFL1
protein_coding	EIF3G
protein_coding	ZMYM1
protein_coding	TCEA1
protein_coding	ZNF655
protein_coding	PCNT
protein_coding	KTI12
protein_coding	B3GALT4
protein_coding	KRTAP2-3
protein_coding	A4GALT
protein_coding	PDK3
protein_coding	DAW1
protein_coding	INHBE
protein_coding	TTC39A
protein_coding	BEND3
protein_coding	ZNF442
protein_coding	MESP1
protein_coding	NKRF
protein_coding	SKA3
protein_coding	MRPL20
protein_coding	TAOK1
protein_coding	SMC4
protein_coding	AKAP7
protein_coding	RNF135
protein_coding	STRBP
protein_coding	GJD2
protein_coding	IZUMO4
protein_coding	AC018867.2
protein_coding	RICTOR
protein_coding	NTF3
protein_coding	ITIH1
protein_coding	GNB4
protein_coding	SAMD12
protein_coding	POMK
protein_coding	PRDM9
protein_coding	SDE2
protein_coding	HS3ST5
protein_coding	PANO1

protein_coding	PRORY
protein_coding	KIAA0100
protein_coding	DDR2
protein_coding	CHCHD3
protein_coding	RRH
protein_coding	SLC37A1
protein_coding	NFIX
protein_coding	DPY19L4
protein_coding	DPH7
protein_coding	NUDCD3
protein_coding	EIF2B4
protein_coding	GOLGA3
protein_coding	PPP1R2
protein_coding	TCP10L
protein_coding	NLRC5
protein_coding	ROPN1
protein_coding	STK31
protein_coding	DRGX
protein_coding	ADAT3
protein_coding	MRPL46
protein_coding	ZG16B
protein_coding	ANKEF1
protein_coding	SUPT5H
protein_coding	SVBP
protein_coding	ERICH2
protein_coding	STARD5
protein_coding	AX748210
protein_coding	HSPA12B
protein_coding	AL590822.1
protein_coding	C2orf42
protein_coding	SLK
protein_coding	TECTB
protein_coding	TMEM222
protein_coding	C1orf85
protein_coding	SEPHS2
protein_coding	C10orf71
protein_coding	SNX27
protein_coding	ITGA2B
protein_coding	FKBP6
protein_coding	AP000275.65
protein_coding	CYP4X1
protein_coding	XPR1
protein_coding	GPR68
protein_coding	LUZPP1
protein_coding	FAM187B
protein_coding	CDCA7L
protein_coding	GOLGA1

protein_coding	DERL2
protein_coding	CALCOCO2
protein_coding	MAPK3
protein_coding	LSM14B
protein_coding	PTPN20A
protein_coding	FAM69A
protein_coding	ENPP7
protein_coding	BIK
protein_coding	TRAM1L1
protein_coding	ZDHHC22
protein_coding	LSM4
protein_coding	GNRH1
protein_coding	SNRK
protein_coding	TMEM132C
protein_coding	TEX2
protein_coding	AGPAT2
protein_coding	DUSP1
protein_coding	LTF
protein_coding	CD68
protein_coding	ANKLE2
protein_coding	MASP2
protein_coding	RBPJL
protein_coding	COL4A4
protein_coding	WIPF3
protein_coding	EIF1B
protein_coding	CT62
protein_coding	AC007919.2
protein_coding	LPPR5
protein_coding	JKAMP
protein_coding	GIMAP1-GIMAP5
protein_coding	DPY19L3
protein_coding	FBXO45
protein_coding	SLC7A13
protein_coding	ZNF682
protein_coding	HSD17B10
protein_coding	WFDC3
protein_coding	BCAP31
protein_coding	RHPN2
protein_coding	MX2
protein_coding	NAALAD2
protein_coding	OCM2
protein_coding	LOH12CR2
protein_coding	CDC5L
protein_coding	PHF1
protein_coding	ELK3
protein_coding	MRAS
protein_coding	RGAG4

protein_coding	KBTBD7
protein_coding	RP11-386G21.2
protein_coding	HAUS8
protein_coding	ABHD3
protein_coding	LTA4H
protein_coding	POU2F1
protein_coding	UNC5C
protein_coding	COX7A2
protein_coding	NAA38
protein_coding	TMEM217
protein_coding	DDX18
protein_coding	FOLR1
protein_coding	AP2S1
protein_coding	DAK
protein_coding	TMSB4Y
protein_coding	KDM3A
protein_coding	LOC100129307
protein_coding	TBC1D12
protein_coding	PTCHD1
protein_coding	DNAJC27
protein_coding	AC083862.1
protein_coding	PRKCD
protein_coding	RRBP1
protein_coding	RPS20
protein_coding	BRE
protein_coding	COQ3
protein_coding	HIVEP3
protein_coding	PRKAR2A
protein_coding	AL583828.1
protein_coding	MRPL48
protein_coding	LYPLAL1
protein_coding	FASTKD1
protein_coding	LETM1
protein_coding	MEF2BNB
protein_coding	WDR91
protein_coding	KCNK4
protein_coding	MAGI1
protein_coding	FN3KRP
protein_coding	TBC1D3B
protein_coding	LCMT1
protein_coding	DISP1
protein_coding	KBTBD13
protein_coding	TEX37
protein_coding	COL24A1
protein_coding	IDH1
protein_coding	RPS12
protein_coding	SLX1B

protein_coding	FAM13B
protein_coding	ARRB1
protein_coding	ANO8
protein_coding	ALG10
protein_coding	MVD
protein_coding	PIK3R3
protein_coding	ALKBH7
protein_coding	C1QL1
protein_coding	ROPN1L
protein_coding	ALDH4A1
protein_coding	ADC
protein_coding	STOX2
protein_coding	RP11-977G19.10
protein_coding	SLIT3
protein_coding	TNFAIP8L2
protein_coding	CEP83
protein_coding	ATP5D
protein_coding	VAMP8
protein_coding	PCSK5
protein_coding	LPL
protein_coding	RP11-706O15.1
protein_coding	GRM4
protein_coding	CMYA5
protein_coding	COMTD1
protein_coding	GSTCD
protein_coding	IVL
protein_coding	PAFAH1B2
protein_coding	GAPT
protein_coding	TNFSF10
protein_coding	RORB
protein_coding	TIGD4
protein_coding	POLR2E
protein_coding	AX747246
protein_coding	WNT16
protein_coding	BBS9
protein_coding	LOC100132167
protein_coding	PPA2
protein_coding	KATNAL2
protein_coding	CACUL1
protein_coding	C17orf75
protein_coding	NF1
protein_coding	ANO2
protein_coding	PTN
protein_coding	UTP3
protein_coding	DNAH7
protein_coding	ZNF567
protein_coding	CTSA

protein_coding	CRIPT
protein_coding	RPE
protein_coding	AC140061.12
protein_coding	CPED1
protein_coding	TIMM17A
protein_coding	RP11-664D7.4
protein_coding	NUP93
protein_coding	SRXN1
protein_coding	DCAF13
protein_coding	GJB5
protein_coding	PSMC3IP
protein_coding	TRMT10A
protein_coding	MMP14
protein_coding	GNAI1
protein_coding	WT1
protein_coding	AC004817.1
protein_coding	TRANK1
protein_coding	ABHD16A
protein_coding	GRIN3A
protein_coding	RBP1
protein_coding	FAM153A
protein_coding	DEK
protein_coding	TMEM174
protein_coding	MUC8
protein_coding	DYRK1A
protein_coding	RASGEF1A
protein_coding	PNMA6C
protein_coding	ERO1A
protein_coding	TMEM110-MUSTN1
protein_coding	TNFSF13B
protein_coding	ZNF786
protein_coding	STOML3
protein_coding	CCL11
protein_coding	LPP
protein_coding	MTPN
protein_coding	MFSD2A
protein_coding	CNPPD1
protein_coding	LMBRD2
protein_coding	LOC728485
protein_coding	KITLG
protein_coding	DMAP1
protein_coding	BCORL1
protein_coding	CELSR2
protein_coding	ERCC6L2
protein_coding	TAF9B
protein_coding	CCL1
protein_coding	SEMA4D

protein_coding	FAM207A
protein_coding	KBTBD2
protein_coding	TBPL1
protein_coding	LILRA5
protein_coding	DAGLA
protein_coding	SOHLH1
protein_coding	AFTPH
protein_coding	AGGF1
protein_coding	ANKRD20A1
protein_coding	FLNB
protein_coding	DR1
protein_coding	SLC4A8
protein_coding	UNC80
protein_coding	GOLGA8S
protein_coding	AGAP3
protein_coding	ATP6AP1
protein_coding	SLC30A10
protein_coding	AMOT
protein_coding	AL592284.1
protein_coding	P2RX3
protein_coding	LCN10
protein_coding	LAYN
protein_coding	BIRC2
protein_coding	NPB
protein_coding	IQCD
protein_coding	WDR13
protein_coding	OTUD6B
protein_coding	ATP1B3
protein_coding	ACVR2B
protein_coding	MRPS34
protein_coding	RPS10-NUDT3
protein_coding	KCNS3
protein_coding	GMPPB
protein_coding	MPHOSPH10
protein_coding	KRTAP11-1
protein_coding	GPS1
protein_coding	MLLT11
protein_coding	FOXP4
protein_coding	MGC4294
protein_coding	LY6D
protein_coding	MUC17
protein_coding	GLTPD2
protein_coding	PSMB2
protein_coding	TP53RK
protein_coding	EIF2AK3
protein_coding	FERMT2
protein_coding	DZIP3

protein_coding	EXOC5
protein_coding	TPR
protein_coding	TRADD
protein_coding	LEMD2
protein_coding	CELA3A
protein_coding	NMRK1
protein_coding	RIOK2
protein_coding	CARD14
protein_coding	TLR9
protein_coding	METRNL
protein_coding	TSPO2
protein_coding	AL833346
protein_coding	NUP160
protein_coding	FIGNL2
protein_coding	ZDHHC15
protein_coding	CAPZB
protein_coding	TAS1R1
protein_coding	TRIM6
protein_coding	BNC1
protein_coding	FLT3LG
protein_coding	CELF2
protein_coding	C3orf58
protein_coding	GPX1
protein_coding	MAGEC1
protein_coding	MORC3
protein_coding	RTCA
protein_coding	CNN1
protein_coding	PAQR9
protein_coding	CRHR1
protein_coding	CTH
protein_coding	PKP3
protein_coding	TCTEX1D2
protein_coding	TBL1Y
protein_coding	PROS1
protein_coding	DPYSL5
protein_coding	RP11-159D12.5
protein_coding	L3MBTL2
protein_coding	AK025127
protein_coding	RP11-598P20.5
protein_coding	GLP1R
protein_coding	CTB-58E17.5
protein_coding	MPST
protein_coding	WDHD1
protein_coding	NCAPG2
protein_coding	RARRES1
protein_coding	IFI30
protein_coding	LAMTOR1

protein_coding	ASPRV1
protein_coding	ZNF446
protein_coding	ACOT6
protein_coding	KCNJ4
protein_coding	ARC
protein_coding	HDDC3
protein_coding	UCHL3
protein_coding	MRPL35
protein_coding	ALDH8A1
protein_coding	SCAMP1
protein_coding	IL17RA
protein_coding	KLHL3
protein_coding	C6orf52
protein_coding	TIMM10
protein_coding	LRTM1
protein_coding	FAM168A
protein_coding	RPL26
protein_coding	CAMKMT
protein_coding	CDKN2AIP
protein_coding	BTNL3
protein_coding	FAM53A
protein_coding	EIF2S2
protein_coding	SUMO2
protein_coding	SMOC1
protein_coding	GATA3
protein_coding	AKR1A1
protein_coding	DDB2
protein_coding	NPIPL1
protein_coding	WDPCP
protein_coding	C7orf43
protein_coding	GPALPP1
protein_coding	SEC11A
protein_coding	GMPR2
protein_coding	FAM3C
protein_coding	DNAJB2
protein_coding	PAQR3
protein_coding	DDA1
protein_coding	PLAC8L1
protein_coding	NACAD
protein_coding	SLC36A4
protein_coding	STARD3
protein_coding	THEGL
protein_coding	AQP12B
protein_coding	POMZP3
protein_coding	AX747193
protein_coding	POLA1
protein_coding	MLEC

protein_coding	PQLC1
protein_coding	KRTAP12-4
protein_coding	SLC4A7
protein_coding	LOC388312
protein_coding	METAP2
protein_coding	ZNF66
protein_coding	SUPV3L1
protein_coding	HELZ
protein_coding	FKBP11
protein_coding	TUBB8
protein_coding	KIAA1147
protein_coding	ZDHHC6
protein_coding	SLU7
protein_coding	TENM1
protein_coding	CFI
protein_coding	LHFPL5
protein_coding	CCZ1B
protein_coding	MYC
protein_coding	FAM196A
protein_coding	OR9A4
protein_coding	CLEC4M
protein_coding	HIBCH
protein_coding	POU3F3
protein_coding	UHRF2
protein_coding	CYP2U1
protein_coding	ZNF280B
protein_coding	PRKCA
protein_coding	MTMR7
protein_coding	PNMA5
protein_coding	LIPT1
protein_coding	DMC1
protein_coding	LURAP1L
protein_coding	TNF
protein_coding	LRRC55
protein_coding	THNSL1
protein_coding	RP11-571M6.15
protein_coding	DPM1
protein_coding	FUCA2
protein_coding	MEI1
protein_coding	C1orf116
protein_coding	EMD
protein_coding	HOXA9
protein_coding	RNF2
protein_coding	TPCN2
protein_coding	INTS2
protein_coding	RRAS
protein_coding	ELAVL3

protein_coding	FUT6
protein_coding	SLC2A14
protein_coding	LMTK2
protein_coding	LDB2
protein_coding	PROM2
protein_coding	ZBTB7C
protein_coding	C11orf65
protein_coding	SAFB2
protein_coding	RAD17
protein_coding	RNASE7
protein_coding	CDC16
protein_coding	THOC1
protein_coding	GPR180
protein_coding	IFNA13
protein_coding	FAM111A
protein_coding	RHOXF1
protein_coding	BCL7A
protein_coding	LRRC30
protein_coding	CSF3
protein_coding	GPN2
protein_coding	FAM136A
protein_coding	ACTR1A
protein_coding	HLA-DQA1
protein_coding	LOC81691
protein_coding	FAM50A
protein_coding	EBF2
protein_coding	CLTC
protein_coding	GPATCH2
protein_coding	ERLEC1
protein_coding	WDR17
protein_coding	RASGEF1B
protein_coding	AL158147.2
protein_coding	ORMDL2
protein_coding	RPP38
protein_coding	GPR146
protein_coding	STIM2
protein_coding	SPSB4
protein_coding	RFX6
protein_coding	PDXK
protein_coding	BCL6B
protein_coding	DST
protein_coding	NLGN2
protein_coding	DJ031140
protein_coding	PLCE1
protein_coding	ZNF500
protein_coding	CYP2R1
protein_coding	RPS29

protein_coding	PDCL3
protein_coding	ME2
protein_coding	BARD1
protein_coding	GCOM1
protein_coding	CBL
protein_coding	KRCC1
protein_coding	ROGDI
protein_coding	ORM1
protein_coding	URI1
protein_coding	ATP2C1
protein_coding	RHBDD3
protein_coding	HSF4
protein_coding	CATSPERG
protein_coding	MTHFSD
protein_coding	JADE3
protein_coding	BNIPL
protein_coding	COA3
protein_coding	MCM5
protein_coding	FAM115A
protein_coding	TBC1D8B
protein_coding	ARMC8
protein_coding	TOR1AIP2
protein_coding	C16orf74
protein_coding	THBS2
protein_coding	RNASE9
protein_coding	FAM132B
protein_coding	SFXN4
protein_coding	OAS3
protein_coding	MBD4
protein_coding	ADGRG3
protein_coding	SH3BP1
protein_coding	LY96
protein_coding	CYSTM1
protein_coding	RP4-583P15.14
protein_coding	POMGNT1
protein_coding	WDTC1
protein_coding	IFI27
protein_coding	PURG
protein_coding	PITPNM1
protein_coding	LIG1
protein_coding	HHAT
protein_coding	NUBP2
protein_coding	DEFB4B
protein_coding	LCE1D
protein_coding	AC136297.1
protein_coding	ARHGEF16
protein_coding	NTNG1

protein_coding	CTBS
protein_coding	USP36
protein_coding	APBB2
protein_coding	FAXC
protein_coding	SPIN4
protein_coding	CDC123
protein_coding	PLXNB1
protein_coding	AGBL3
protein_coding	TCN1
protein_coding	MAP3K1
protein_coding	TUBD1
protein_coding	AB231702
protein_coding	CERS3
protein_coding	FAM177B
protein_coding	TPTE
protein_coding	XPNPEP3
protein_coding	ABO
protein_coding	FAM24B-CUZD1
protein_coding	ATP6V1E2
protein_coding	EHHADH
protein_coding	ADGRD1
protein_coding	RHCG
protein_coding	ATAD2B
protein_coding	LRP3
protein_coding	NSUN2
protein_coding	SDCCAG8
protein_coding	SPINT1
protein_coding	LRRC9
protein_coding	YIF1A
protein_coding	C3orf14
protein_coding	FAM151B
protein_coding	SCX
protein_coding	SEC61B
protein_coding	C9orf139
protein_coding	TAF1D
protein_coding	GPR83
protein_coding	HAUS1
protein_coding	PLGLB1
protein_coding	WRB
protein_coding	KIAA0040
protein_coding	MYL9
protein_coding	PRPF4B
protein_coding	ANAPC7
protein_coding	ESYT3
protein_coding	C12orf44
protein_coding	BMP6
protein_coding	AK126380

protein_coding	DNTTIP1
protein_coding	QSOX2
protein_coding	TERT
protein_coding	FBP2
protein_coding	CYS1
protein_coding	ZFAND1
protein_coding	CXCL5
protein_coding	SAV1
protein_coding	THEMIS2
protein_coding	PORCN
protein_coding	MGARP
protein_coding	PCMTD1
protein_coding	ZNF668
protein_coding	ASB7
protein_coding	KRTAP10-1
protein_coding	LRRFIP1
protein_coding	PIK3C3
protein_coding	REC8
protein_coding	FH
protein_coding	NCS1
protein_coding	MRPL23
protein_coding	CLCN4
protein_coding	PAPSS2
protein_coding	RIN1
protein_coding	ABI1
protein_coding	SIDT1
protein_coding	KIAA2026
protein_coding	PGM5
protein_coding	BLZF1
protein_coding	AK096982
protein_coding	ZFP91
protein_coding	AC005358.1
protein_coding	DCDC2
protein_coding	NR1D1
protein_coding	SLC35D2
protein_coding	NKD2
protein_coding	PACRGL
protein_coding	SOBP
protein_coding	TSSC1
protein_coding	BV03S1J2.2
protein_coding	SLC10A4
protein_coding	GTF2H5
protein_coding	PCDHGA4
protein_coding	JARID2
protein_coding	TINF2
protein_coding	C1orf101
protein_coding	RP6-24A23.6

protein_coding	GPBP1L1
protein_coding	ZNF789
protein_coding	EFNA5
protein_coding	ABCF1
protein_coding	ANKHD1
protein_coding	RASGEF1C
protein_coding	DNASE1L2
protein_coding	LACC1
protein_coding	CD4
protein_coding	INTS7
protein_coding	RP11-215A19.2
protein_coding	FTHL17
protein_coding	TBP
protein_coding	AKAP8
protein_coding	MAFB
protein_coding	FLJ14346
protein_coding	SYT8
protein_coding	STPG1
protein_coding	BDNF
protein_coding	AHCTF1
protein_coding	BC046483
protein_coding	RNF34
protein_coding	S1PR3
protein_coding	OR5H14
protein_coding	WLS
protein_coding	SLC25A11
protein_coding	DPH5
protein_coding	CDH8
protein_coding	ZBTB47
protein_coding	B3GNT2
protein_coding	DDX46
protein_coding	RYR3
protein_coding	AC022498.1
protein_coding	C6orf203
protein_coding	UTRN
protein_coding	EID2
protein_coding	MACROD2
protein_coding	RNF219
protein_coding	TCP1
protein_coding	C1QBP
protein_coding	DNAL1
protein_coding	TAF15
protein_coding	SERPINB6
protein_coding	STARD9
protein_coding	NME1-NME2
protein_coding	GOLGA8G
protein_coding	AC012123.1

protein_coding	PNRC1
protein_coding	ECE2
protein_coding	BCL9L
protein_coding	PRKG2
protein_coding	CCDC93
protein_coding	IL37
protein_coding	TMEM183A
protein_coding	CACNA2D1
protein_coding	DMWD
protein_coding	GEMIN2
protein_coding	COMMD3-BMI1
protein_coding	MRPL22
protein_coding	DOK7
protein_coding	OR6B2
protein_coding	C12orf10
protein_coding	LGALS12
protein_coding	KCTD11
protein_coding	RFWD3
protein_coding	EXOC2
protein_coding	TSPYL6
protein_coding	UCN3
protein_coding	MAPKAPK2
protein_coding	ROBO3
protein_coding	DRD4
protein_coding	AEBP2
protein_coding	SRSF9
protein_coding	ZFC3H1
protein_coding	IL10RB
protein_coding	USP9X
protein_coding	RP11-467N20.5
protein_coding	C11orf94
protein_coding	PDXP
protein_coding	LOC650293
protein_coding	COX10
protein_coding	PTPMT1
protein_coding	MICALL2
protein_coding	WDR86
protein_coding	APOC1
protein_coding	LARP1
protein_coding	SLC9A5
protein_coding	CES3
protein_coding	SERPINE3
protein_coding	MRPL28
protein_coding	CRB3
protein_coding	SUB1
protein_coding	GLCE
protein_coding	MAGEH1

protein_coding	MTFP1
protein_coding	TMEM65
protein_coding	NUP155
protein_coding	DNAJC1
protein_coding	KLHL40
protein_coding	FSTL5
protein_coding	CCDC127
protein_coding	IFI16
protein_coding	STK19
protein_coding	FAM189A1
protein_coding	CMSS1
protein_coding	TMEM261
protein_coding	POLM
protein_coding	KB-1980E6.3
protein_coding	TNR
protein_coding	ZNF593
protein_coding	FAM184B
protein_coding	SLC30A1
protein_coding	FAM186A
protein_coding	HIST1H4C
protein_coding	PIGX
protein_coding	LRRN1
protein_coding	BMP1
protein_coding	RFC1
protein_coding	FMN2
protein_coding	SRCAP
protein_coding	PALLD
protein_coding	SERHL2
protein_coding	FOXL2NB
protein_coding	RAB7L1
protein_coding	NOC3L
protein_coding	SMTNL1
protein_coding	SIX2
protein_coding	MMP11
protein_coding	CDADC1
protein_coding	PARP9
protein_coding	EED
protein_coding	RFPL3
protein_coding	VPS37B
protein_coding	SUCLG2
protein_coding	VCAN
protein_coding	HAT1
protein_coding	HMG20A
protein_coding	SPHK2
protein_coding	KIAA1919
protein_coding	FILIP1
protein_coding	ITGAD

protein_coding	RAD50
protein_coding	VSIG1
protein_coding	RALGPS1
protein_coding	TOMM40
protein_coding	SPSB2
protein_coding	KRTAP22-2
protein_coding	RP11-295K3.1
protein_coding	HEATR1
protein_coding	WFDC10B
protein_coding	AGO4
protein_coding	RNASEH2C
protein_coding	PEX3
protein_coding	SCHIP1
protein_coding	FANCA
protein_coding	NDUFAF2
protein_coding	KIAA1456
protein_coding	GABRE
protein_coding	TYSND1
protein_coding	AP4B1
protein_coding	ZRSR1
protein_coding	AC114546.1
protein_coding	RAB2B
protein_coding	ZCCHC11
protein_coding	ANAPC4
protein_coding	C9orf152
protein_coding	KLF17
protein_coding	NXNL2
protein_coding	TTC19
protein_coding	AIDA
protein_coding	IPO11
protein_coding	CLPP
protein_coding	AK304826
protein_coding	RIC3
protein_coding	TNS3
protein_coding	SMIM5
protein_coding	PHGR1
protein_coding	SNX7
protein_coding	ADH5
protein_coding	PIGBOS1
protein_coding	TTC33
protein_coding	CCNB1IP1
protein_coding	KCNN4
protein_coding	INPP5A
protein_coding	MCM3AP
protein_coding	FAM96B
protein_coding	TM2D2
protein_coding	APOE

protein_coding	ALAD
protein_coding	TMEM181
protein_coding	SLC13A4
protein_coding	CCRL2
protein_coding	SNRNP40
protein_coding	CCDC140
protein_coding	COA4
protein_coding	MFN2
protein_coding	WWP1
protein_coding	NINL
protein_coding	OFCC1
protein_coding	SETMAR
protein_coding	FAM177A1
protein_coding	ARRDC5
protein_coding	FOXD4
protein_coding	EPRS
protein_coding	SVOPL
protein_coding	PLEKHO2
protein_coding	PRELP
protein_coding	SLX4IP
protein_coding	CYLD
protein_coding	MRPL37
protein_coding	FBXO42
protein_coding	RNPEP
protein_coding	SLC6A11
protein_coding	AK302511
protein_coding	LRP5L
protein_coding	SLBP
protein_coding	XKR7
protein_coding	DNM1
protein_coding	MRPS14
protein_coding	PLA2G4A
protein_coding	NOBOX
protein_coding	DPP9-AS1
protein_coding	PDS5B
protein_coding	MTRNR2L4
protein_coding	C17orf50
protein_coding	ABCB1
protein_coding	TIGD3
protein_coding	FKBP1A
protein_coding	MNAT1
protein_coding	HOOK3
protein_coding	MYH11
protein_coding	GRIN2D
protein_coding	MPZ
protein_coding	G0S2
protein_coding	SPATA4

protein_coding	CLEC1A
protein_coding	PARL
protein_coding	AASS
protein_coding	MSX1
protein_coding	RMND5A
protein_coding	RP11-407N17.3
protein_coding	FIG4
protein_coding	RNPC3
protein_coding	ZNF805
protein_coding	TMEM50B
protein_coding	FBXL3
protein_coding	COLCA2
protein_coding	APC2
protein_coding	BTBD1
protein_coding	XYLB
protein_coding	NLRP3
protein_coding	C7orf25
protein_coding	RP11-1102P16.1
protein_coding	RGS2
protein_coding	ZNF382
protein_coding	AC068533.7
protein_coding	GPD2
protein_coding	ASIC3
protein_coding	ARL17B
protein_coding	TDRKH
protein_coding	RNF20
protein_coding	CTD-2583A14.10
protein_coding	STBD1
protein_coding	DOK1
protein_coding	TMEM31
protein_coding	IGDCC4
protein_coding	NCR3
protein_coding	RAB10
protein_coding	LCE1A
protein_coding	MCF2L
protein_coding	THAP4
protein_coding	SPCS1
protein_coding	IL11
protein_coding	PRKD3
protein_coding	ZNF732
protein_coding	DCTN5
protein_coding	KCNC3
protein_coding	STX16
protein_coding	WFDC9
protein_coding	HSBP1
protein_coding	GRP
protein_coding	EPM2AIP1

protein_coding	CELF3
protein_coding	RGS8
protein_coding	ZNF507
protein_coding	SNF8
protein_coding	RP11-345J4.5
protein_coding	CCDC120
protein_coding	SEC61A1
protein_coding	DCHS2
protein_coding	AL354993.1
protein_coding	CCDC174
protein_coding	DLG4
protein_coding	FASTKD3
protein_coding	LCP1
protein_coding	SLC25A23
protein_coding	CPEB4
protein_coding	ZNF398
protein_coding	RAB40A
protein_coding	ANP32B
protein_coding	SIL1
protein_coding	YIPF1
protein_coding	TH
protein_coding	ZDHHC9
protein_coding	CGNL1
protein_coding	OCSTAMP
protein_coding	FAM19A4
protein_coding	FTCD
protein_coding	ZKSCAN5
protein_coding	AC010646.3
protein_coding	CLEC2A
protein_coding	MST1
protein_coding	LAMA4
protein_coding	SAT2
protein_coding	GALNT3
protein_coding	H2AFB2
protein_coding	NPLOC4
protein_coding	MAFK
protein_coding	GOLGA6C
protein_coding	PIM2
protein_coding	ANTXR1
protein_coding	AC020907.1
protein_coding	NAA60
protein_coding	CNPY4
protein_coding	PTPRN
protein_coding	MYT1L
protein_coding	TRIM5
protein_coding	KEL
protein_coding	CAP1

protein_coding	LRRC4
protein_coding	RBM14-RBM4
protein_coding	NXF3
protein_coding	ANKRD20A2
protein_coding	PSME1
protein_coding	SCIN
protein_coding	CHST14
protein_coding	SPAG7
protein_coding	CA14
protein_coding	CETN2
protein_coding	ADAT2
protein_coding	RDH12
protein_coding	FICD
protein_coding	EXOC1
protein_coding	RPS21
protein_coding	TSNARE1
protein_coding	KLK8
protein_coding	LDLR
protein_coding	LDLRAD1
protein_coding	RETNLB
protein_coding	TMCO4
protein_coding	FGF13
protein_coding	GTF2A2
protein_coding	AX747192
protein_coding	MS4A5
protein_coding	TBC1D13
protein_coding	HERC3
protein_coding	NNMT
protein_coding	C9orf24
protein_coding	ELL
protein_coding	RPL3L
protein_coding	GRID2IP
protein_coding	MAK
protein_coding	DNAH11
protein_coding	FRMD3
protein_coding	BUD31
protein_coding	DIRAS3
protein_coding	DDX10
protein_coding	PYCR1
protein_coding	OR8U1
protein_coding	PAICS
protein_coding	DNASE1
protein_coding	GOLGA6L10
protein_coding	CNTN6
protein_coding	ILDR2
protein_coding	ZNF853
protein_coding	S1PR1

protein_coding	SUMF2
protein_coding	DNAJC30
protein_coding	GPANK1
protein_coding	TTLL12
protein_coding	SDHAF1
protein_coding	GDF6
protein_coding	MRPL9
protein_coding	ZNF155
protein_coding	S100PBP
protein_coding	BAX
protein_coding	RPS17L
protein_coding	GABBR1
protein_coding	CD69
protein_coding	WDYHV1
protein_coding	GRIN3B
protein_coding	CREB3L2
protein_coding	ZDHHC24
protein_coding	UQCR11
protein_coding	SLC35B4
protein_coding	ATXN1L
protein_coding	SLC15A2
protein_coding	PADI4
protein_coding	ELL3
protein_coding	ZBTB20
protein_coding	ATP11A
protein_coding	PRPS1L1
protein_coding	MATN1
protein_coding	BMPR2
protein_coding	LOC653501
protein_coding	LRRC8A
protein_coding	NRARP
protein_coding	SYNGR4
protein_coding	PCNP
protein_coding	TMEM42
protein_coding	AK056253
protein_coding	BC037497
protein_coding	PCDHB7
protein_coding	PCDHGB6
protein_coding	CTC-435M10.3
protein_coding	TMEM38A
protein_coding	GCDH
protein_coding	TMTC2
protein_coding	TMEM204
protein_coding	PHKA2
protein_coding	LIPE
protein_coding	PYROXD1
protein_coding	SNX20

protein_coding	SEPT3
protein_coding	B9D1
protein_coding	ADAMTSL5
protein_coding	BC008049
protein_coding	DQ786323
protein_coding	WRAP73
protein_coding	VEZF1
protein_coding	SHE
protein_coding	CPT1C
protein_coding	SLC25A20
protein_coding	C1orf94
protein_coding	TUBE1
protein_coding	RPP25L
protein_coding	MED13
protein_coding	DNM3
protein_coding	HNRPF
protein_coding	C8orf76
protein_coding	OSMR
protein_coding	SDR42E2
protein_coding	VASP
protein_coding	CYP4F22
protein_coding	ONECUT3
protein_coding	SPTBN2
protein_coding	GPR119
protein_coding	POLA2
protein_coding	ASXL1
protein_coding	RNF141
protein_coding	APOBEC3A_B
protein_coding	AOC3
protein_coding	EFHB
protein_coding	CNBP
protein_coding	DNASE2
protein_coding	PCDHGA7
protein_coding	C14orf169
protein_coding	CHRD
protein_coding	CT45A10
protein_coding	FOXL1
protein_coding	PRDX3
protein_coding	BLVRA
protein_coding	RALB
protein_coding	GPER1
protein_coding	RPS13
protein_coding	GTF2H1
protein_coding	LOC493754
protein_coding	ATG9A
protein_coding	YPEL4
protein_coding	NCAN

protein_coding	C8orf12
protein_coding	SLMAP
protein_coding	AC062017.1
protein_coding	RCAN3
protein_coding	CCL7
protein_coding	NCF4
protein_coding	PBX2
protein_coding	PPM1A
protein_coding	LLPH
protein_coding	DENND6A
protein_coding	CHD3
protein_coding	TSTD2
protein_coding	MYO19
protein_coding	FREM1
protein_coding	SLC25A3
protein_coding	SWI5
protein_coding	TMCC2
protein_coding	LAMTOR4
protein_coding	GLE1
protein_coding	GPR19
protein_coding	TSG101
protein_coding	MYH3
protein_coding	PPAN
protein_coding	LOC100630923
protein_coding	UFM1
protein_coding	ENY2
protein_coding	DOCK6
protein_coding	PHLDA3
protein_coding	CD59
protein_coding	ATP2B4
protein_coding	C2orf68
protein_coding	LINGO4
protein_coding	MAPK15
protein_coding	RABL2B
protein_coding	NGLY1
protein_coding	TM7SF2
protein_coding	RP1
protein_coding	LOC100506127
protein_coding	DONSON
protein_coding	TRIM45
protein_coding	FAM157B
protein_coding	EPHB3
protein_coding	EGFL7
protein_coding	GDPD4
protein_coding	SALL2
protein_coding	CCZ1
protein_coding	LSM2

protein_coding	CCBL1
protein_coding	PPP1R1C
protein_coding	MS4A18
protein_coding	GJB7
protein_coding	KIF9
protein_coding	RFC4
protein_coding	PARVA
protein_coding	SLC45A4
protein_coding	DAZL
protein_coding	ORC6
protein_coding	PCSK7
protein_coding	C9orf169
protein_coding	NHLRC1
protein_coding	RAB40AL
protein_coding	TRMT10C
protein_coding	TTC31
protein_coding	TRIM29
protein_coding	SDK1
protein_coding	CLDN1
protein_coding	ZNF418
protein_coding	HIAT1
protein_coding	AC003002.6
protein_coding	TRUB1
protein_coding	ZNF182
protein_coding	FAP
protein_coding	F8A2
protein_coding	PEF1
protein_coding	FFAR3
protein_coding	MTUS1
protein_coding	SLC35D3
protein_coding	MAP1S
protein_coding	GLYATL3
protein_coding	PRR15L
protein_coding	MGA
protein_coding	AASDHPPT
protein_coding	OR4D6
protein_coding	INS
protein_coding	MTERFD3
protein_coding	RPRD1A
protein_coding	CAMK1
protein_coding	SPTSSB
protein_coding	TOX
protein_coding	RASA1
protein_coding	CDKL3
protein_coding	ZNF629
protein_coding	GK5
protein_coding	EN2

protein_coding	PFDN6
protein_coding	ROR2
protein_coding	PHTF1
protein_coding	MYSM1
protein_coding	SMR3B
protein_coding	MALL
protein_coding	SERPINB11
protein_coding	RAB22A
protein_coding	RBM4
protein_coding	AKR7A3
protein_coding	MTMR11
protein_coding	MFAP2
protein_coding	LOC100129924
protein_coding	ASL
protein_coding	LOC649330
protein_coding	BRD8
protein_coding	CD300C
protein_coding	RNF224
protein_coding	TIGD2
protein_coding	ELP6
protein_coding	MED24
protein_coding	KRTAP9-7
protein_coding	TFAP2B
protein_coding	DNALI1
protein_coding	FBXO30
protein_coding	PTPRO
protein_coding	LIPT2
protein_coding	PLAUR
protein_coding	ARHGEF28
protein_coding	OSBPL9
protein_coding	MID1
protein_coding	AC090186.1
protein_coding	TACO1
protein_coding	ZNF7
protein_coding	RDH13
protein_coding	PLCB2
protein_coding	AC037459.4
protein_coding	CUTC
protein_coding	SMIM12
protein_coding	MADCAM1
protein_coding	RP4-559A3.7
protein_coding	KCNJ3
protein_coding	PCOLCE2
protein_coding	PDE4A
protein_coding	ESF1
protein_coding	AKAP9
protein_coding	RIF1

protein_coding	POLK
protein_coding	TAF3
protein_coding	PCYOX1
protein_coding	SRGAP2
protein_coding	ATF6B
protein_coding	RNF25
protein_coding	DICER1
protein_coding	EID3
protein_coding	SEMA3E
protein_coding	HNRNPM
protein_coding	NBPF24
protein_coding	CHORDC1
protein_coding	PFDN5
protein_coding	CACFD1
protein_coding	LZTS2
protein_coding	NNT
protein_coding	AMY1C
protein_coding	TMEM256-PLSCR3
protein_coding	OR2W5
protein_coding	ITGA9
protein_coding	BICD1
protein_coding	SACS
protein_coding	LYPD2
protein_coding	RP11-126K1.2
protein_coding	HR
protein_coding	GNG13
protein_coding	SMAD5
protein_coding	NDUFS7
protein_coding	GRM2
protein_coding	XRCC3
protein_coding	ABL2
protein_coding	NUP205
protein_coding	TRUB2
protein_coding	OR52E1
protein_coding	RPUSD3
protein_coding	FBXO8
protein_coding	SHISA4
protein_coding	PRR7
protein_coding	ZNF736
protein_coding	ABHD14A
protein_coding	HK3
protein_coding	ANKRD34A
protein_coding	BPGM
protein_coding	ERCC4
protein_coding	HIST1H3D
protein_coding	CACNA1F
protein_coding	TTC18

protein_coding	CHD6
protein_coding	ADAP2
protein_coding	KRT19
protein_coding	METTL9
protein_coding	AC026740.1
protein_coding	ARHGEF11
protein_coding	SHMT2
protein_coding	PRPF39
protein_coding	ZNF74
protein_coding	IFNA8
protein_coding	PTK2
protein_coding	ERI2
protein_coding	RNASEH1
protein_coding	SLC16A10
protein_coding	AC109583.1
protein_coding	ZKSCAN2
protein_coding	ZNF700
protein_coding	ZNF780A
protein_coding	CEMIP
protein_coding	SPRR1A
protein_coding	DLAT
protein_coding	CAPN3
protein_coding	DRD2
protein_coding	HLA-DOA
protein_coding	MAP3K7
protein_coding	C12orf42
protein_coding	KIF27
protein_coding	MRPS30
protein_coding	SMARCE1
protein_coding	FAR2
protein_coding	BC041025
protein_coding	POM121L7
protein_coding	CHMP1A
protein_coding	ARMCX3
protein_coding	TAF1
protein_coding	MOCS2
protein_coding	TSPAN13
protein_coding	KGFLP2
protein_coding	CYP4V2
protein_coding	PPAPDC3
protein_coding	MZT2B
protein_coding	SPDYE5
protein_coding	GLTSCR2
protein_coding	PRRX1
protein_coding	PPM1H
protein_coding	KLHDC1
protein_coding	KPNA1

protein_coding	CHST1
protein_coding	GPATCH4
protein_coding	NEK10
protein_coding	FAM46A
protein_coding	EVA1C
protein_coding	COL14A1
protein_coding	ANO4
protein_coding	AXL
protein_coding	MRPS12
protein_coding	SLC6A8
protein_coding	TSC22D2
protein_coding	DPF3
protein_coding	FAM120A
protein_coding	HNRPLL
protein_coding	RIN2
protein_coding	HRC
protein_coding	JAM3
protein_coding	FARP2
protein_coding	SPRED3
protein_coding	SOX6
protein_coding	AK127224
protein_coding	DNAJC22
protein_coding	MFGE8
protein_coding	HDHD2
protein_coding	PKD1
protein_coding	MTOR
protein_coding	SLC22A14
protein_coding	GMNN
protein_coding	DHFRL1
protein_coding	CELA3B
protein_coding	OR2T33
protein_coding	NR4A1
protein_coding	NCKIPSD
protein_coding	DCDC5
protein_coding	ST6GALNAC5
protein_coding	PLXNB2
protein_coding	UFC1
protein_coding	TMCC1
protein_coding	ACSS3
protein_coding	PEX14
protein_coding	FBXL18
protein_coding	FAM8A1
protein_coding	PEBP4
protein_coding	TBC1D3C
protein_coding	HNRNPCL4
protein_coding	CAND1
protein_coding	MUC6

protein_coding	ZNF626
protein_coding	SCUBE1
protein_coding	C11orf74
protein_coding	TCRBV4S1A1T
protein_coding	CBWD6
protein_coding	AX747795
protein_coding	MCM6
protein_coding	ZNF350
protein_coding	HYAL1
protein_coding	COL21A1
protein_coding	SEH1L
protein_coding	CKS1B
protein_coding	FDFT1
protein_coding	FRAS1
protein_coding	RP11-318A15.7
protein_coding	RBM8A
protein_coding	FAM151A
protein_coding	AK311005
protein_coding	ZNF529
protein_coding	ZNF774
protein_coding	KLF8
protein_coding	PHACTR4
protein_coding	RP1-130H16.18
protein_coding	MRPL54
protein_coding	EPB41L4A
protein_coding	FKBP1A-SDCBP2
protein_coding	SPACA4
protein_coding	DCUN1D4
protein_coding	CALML5
protein_coding	MYO9B
protein_coding	PRR21
protein_coding	UBE2V1
protein_coding	AGPAT5
protein_coding	CORO7-PAM16
protein_coding	DDIT4L
protein_coding	ZNF627
protein_coding	CCDC130
protein_coding	ZNF768
protein_coding	FAM71E2
protein_coding	SYTL1
protein_coding	AX747187
protein_coding	AMIGO3
protein_coding	ZNF830
protein_coding	ZBP1
protein_coding	SSX5
protein_coding	MECR
protein_coding	CD55

protein_coding	TRIP11
protein_coding	ZNF8
protein_coding	ZMYND11
protein_coding	CNGA3
protein_coding	FAM89A
protein_coding	GNB2L1
protein_coding	RBM15
protein_coding	CLEC9A
protein_coding	BID
protein_coding	IGLON5
protein_coding	TSNAX
protein_coding	HPS3
protein_coding	ZNF628
protein_coding	ZNF528
protein_coding	KIAA1755
protein_coding	FZD4
protein_coding	UBE2N
protein_coding	WDR31
protein_coding	SLC25A14
protein_coding	NCOA5
protein_coding	TNFAIP6
protein_coding	ADSSL1
protein_coding	PTPN12
protein_coding	RSPH3
protein_coding	PLCH1
protein_coding	C1orf106
protein_coding	LRRC53
protein_coding	SSTR1
protein_coding	ETFA
protein_coding	ERCC6-PGBD3
protein_coding	SLC12A5
protein_coding	MIS18A
protein_coding	PRR23A
protein_coding	SDCBP2
protein_coding	SPIN3
protein_coding	C3orf52
protein_coding	FAR1
protein_coding	POP1
protein_coding	OR6C74
protein_coding	CD8B
protein_coding	PPP4R4
protein_coding	MLNR
protein_coding	PLAA
protein_coding	ZFHX4
protein_coding	ZFP69
protein_coding	TTYH3
protein_coding	XPA

protein_coding	SOCS2
protein_coding	ZDHHC16
protein_coding	EML3
protein_coding	OR2T8
protein_coding	THNSL2
protein_coding	USF2
protein_coding	CHRNA10
protein_coding	PNRC2
protein_coding	AQP3
protein_coding	AX747372
protein_coding	PRMT3
protein_coding	NISCH
protein_coding	SPATA45
protein_coding	NCKAP5
protein_coding	TTC29
protein_coding	DROSHA
protein_coding	SESN2
protein_coding	PCDHGA2
protein_coding	SEZ6L
protein_coding	KCNK3
protein_coding	EIF3D
protein_coding	LTBP4
protein_coding	FAM214B
protein_coding	CD74
protein_coding	ZNF771
protein_coding	TMEM219
protein_coding	LSP1
protein_coding	IFT80
protein_coding	CD83
protein_coding	LOC283767
protein_coding	HES6
protein_coding	CXorf67
protein_coding	LRRC32
protein_coding	ZFAT
protein_coding	ARHGEF40
protein_coding	RPL39L
protein_coding	THUMPD1
protein_coding	AC136604.1
protein_coding	ANKRD39
protein_coding	AL832891
protein_coding	TRPV2
protein_coding	GEM
protein_coding	CCDC74A
protein_coding	DUOX2
protein_coding	MAU2
protein_coding	EIF2B1
protein_coding	PSG5

protein_coding	TNFRSF25
protein_coding	SFI1
protein_coding	SLC24A5
protein_coding	SPATA2
protein_coding	HLX
protein_coding	TIMM8A
protein_coding	LCAT
protein_coding	CCDC138
protein_coding	DNAH1
protein_coding	PIK3CG
protein_coding	BMP4
protein_coding	LCE1B
protein_coding	PDZD2
protein_coding	SHKBP1
protein_coding	SCRT1
protein_coding	GCNT2
protein_coding	SPPL2A
protein_coding	C14orf159
protein_coding	AP002884.3
protein_coding	SNX6
protein_coding	TBRG1
protein_coding	FPR1
protein_coding	CHD9
protein_coding	ZNF480
protein_coding	EPHX1
protein_coding	ANGPT2
protein_coding	PDLIM5
protein_coding	IDH3G
protein_coding	NUCKS1
protein_coding	TSPY3
protein_coding	PATZ1
protein_coding	EHD1
protein_coding	FAM212A
protein_coding	PHF13
protein_coding	PSMC3
protein_coding	RUFY2
protein_coding	NSMCE1
protein_coding	TMED1
protein_coding	ORMDL3
protein_coding	B3GNT3
protein_coding	FAM185A
protein_coding	ZASP
protein_coding	MPLKIP
protein_coding	CDNF
protein_coding	SAMD4A
protein_coding	CTAG1B
protein_coding	SUGP1

protein_coding	RPS7
protein_coding	ING3
protein_coding	AP1S1
protein_coding	NFU1
protein_coding	ASCL1
protein_coding	AV1S4A1N1T
protein_coding	EMC3
protein_coding	COL1A2
protein_coding	CSTF1
protein_coding	GNG12
protein_coding	ITCH
protein_coding	ZSWIM5
protein_coding	ARHGDIA
protein_coding	FNDC4
protein_coding	DNMBP
protein_coding	SEPT2
protein_coding	NPAS1
protein_coding	RWDD4
protein_coding	CHCHD5
protein_coding	PLK3
protein_coding	UNC119B
protein_coding	FBXL16
protein_coding	KIAA1704
protein_coding	PABPN1L
protein_coding	HSPA14
protein_coding	ASB6
protein_coding	BIRC6
protein_coding	SON
protein_coding	PADI1
protein_coding	ARIH2OS
protein_coding	CRISPLD1
protein_coding	GOLGA6L9
protein_coding	CCL8
protein_coding	CYTL1
protein_coding	SLC26A10
protein_coding	HS1BP3
protein_coding	LAPTM5
protein_coding	CPT1B
protein_coding	ZNF250
protein_coding	SKP2
protein_coding	bK250D10.C22.8
protein_coding	SLC43A3
protein_coding	C11orf21
protein_coding	PCDHGA6
protein_coding	NMT1
protein_coding	ZNF525
protein_coding	ENPEP

protein_coding	AKAP17A
protein_coding	TMEM133
protein_coding	CT45A1
protein_coding	CASC4
protein_coding	PAQR4
protein_coding	RAB3IL1
protein_coding	ORAI3
protein_coding	RNASE6
protein_coding	SYCN
protein_coding	PRDM14
protein_coding	LATS1
protein_coding	RSF1
protein_coding	EFNB2
protein_coding	TNRC18
protein_coding	SLC25A6
protein_coding	FHL5
protein_coding	ODF3B
protein_coding	USP17L7
protein_coding	SPG11
protein_coding	MYADM
protein_coding	TOP1
protein_coding	SNED1
protein_coding	B3GNTL1
protein_coding	ALPK3
protein_coding	HEY2
protein_coding	F10
protein_coding	CAMK2D
protein_coding	PPY
protein_coding	RAB44
protein_coding	MLH3
protein_coding	APOBEC3G
protein_coding	RFPL1
protein_coding	KIAA1161
protein_coding	UBXN2A
protein_coding	TIMM13
protein_coding	AP000304.12
protein_coding	SSC4D
protein_coding	KRTAP9-6
protein_coding	DNAJC10
protein_coding	EIF3J
protein_coding	LRRFIP2
protein_coding	HUG1
protein_coding	UTP11L
protein_coding	BLNK
protein_coding	C18orf42
protein_coding	FKBP5
protein_coding	DEFA5

protein_coding	KDELC1
protein_coding	DYM
protein_coding	CCDC88B
protein_coding	TUT1
protein_coding	SASS6
protein_coding	SCO2
protein_coding	HECTD4
protein_coding	PROSER2
protein_coding	CENPV
protein_coding	SOCS4
protein_coding	RPRM
protein_coding	ELN
protein_coding	TAT
protein_coding	CHST13
protein_coding	SLTM
protein_coding	KLHL13
protein_coding	POGZ
protein_coding	CLEC3B
protein_coding	GJC3
protein_coding	ZNRF3
protein_coding	ACOX2
protein_coding	GTPBP4
protein_coding	RHOA
protein_coding	PCDHGC5
protein_coding	GPR137
protein_coding	URM1
protein_coding	DACT3
protein_coding	FAM208B
protein_coding	ZCCHC4
protein_coding	FBXL8
protein_coding	GLI2
protein_coding	OR10AC1P
protein_coding	FOS
protein_coding	SEL1L
protein_coding	NCOR1
protein_coding	USP44
protein_coding	ACTR5
protein_coding	BC010030
protein_coding	CDC6
protein_coding	CHAD
protein_coding	C2orf27B
protein_coding	RASSF7
protein_coding	RPS6KC1
protein_coding	C1orf177
protein_coding	GPR133
protein_coding	HLA-DQA2
protein_coding	CYP2E1

protein_coding	ZMAT2
protein_coding	NUP107
protein_coding	PRR20A
protein_coding	OR51I1
protein_coding	ABCA10
protein_coding	NEURL2
protein_coding	GPATCH8
protein_coding	NAV2
protein_coding	LINGO1
protein_coding	ZNF530
protein_coding	GLRA2
protein_coding	UCK1
protein_coding	GAS2L1
protein_coding	HS6ST2
protein_coding	TRPV1
protein_coding	NAGLU
protein_coding	FKBP4
protein_coding	DOCK7
protein_coding	EML5
protein_coding	RAP2B
protein_coding	SDCBP
protein_coding	AL358333.1
protein_coding	KRTAP15-1
protein_coding	TMF1
protein_coding	SV2B
protein_coding	RP11-127H5.1
protein_coding	DALRD3
protein_coding	SPATA2L
protein_coding	TCTN1
protein_coding	RPGRIP1L
protein_coding	LIN37
protein_coding	SEPHS1
protein_coding	CENPC
protein_coding	SCCPDH
protein_coding	IFI44
protein_coding	C8orf46
protein_coding	PRSS42
protein_coding	LTV1
protein_coding	CPNE3
protein_coding	NOP58
protein_coding	PPP1R7
protein_coding	SREK1IP1
protein_coding	MEPE
protein_coding	PPRC1
protein_coding	RPL8
protein_coding	PPP1R9B
protein_coding	ABCA6

protein_coding	PRSS41
protein_coding	CBLL1
protein_coding	C22orf46
protein_coding	MAP3K4
protein_coding	ELOF1
protein_coding	OR5AU1
protein_coding	ELTD1
protein_coding	IFNAR1
protein_coding	SGMS2
protein_coding	TVP23B
protein_coding	AC110771.1
protein_coding	ZNF214
protein_coding	AX747031
protein_coding	LSMEM1
protein_coding	NOL10
protein_coding	SCAMP2
protein_coding	EIF2D
protein_coding	SPOCD1
protein_coding	SCNN1G
protein_coding	ARL2BP
protein_coding	HOXB13
protein_coding	TCTA
protein_coding	SERINC2
protein_coding	INPP5D
protein_coding	PARD6B
protein_coding	BROX
protein_coding	PTP4A3
protein_coding	ZC2HC1A
protein_coding	KRT35
protein_coding	FBXO28
protein_coding	BDH2
protein_coding	ZSCAN30
protein_coding	MTX2
protein_coding	SLC17A4
protein_coding	FTH1P18
protein_coding	RBM27
protein_coding	B3GLCT
protein_coding	ANO6
protein_coding	LY9
protein_coding	RP11-10J21.3
protein_coding	ACTR6
protein_coding	WNK2
protein_coding	SIGMAR1
protein_coding	TEX261
protein_coding	SPDYE3
protein_coding	WDR62
protein_coding	ZZZ3

protein_coding	INTS6
protein_coding	LRP11
protein_coding	HDLBP
protein_coding	IDUA
protein_coding	C19orf25
protein_coding	AQR
protein_coding	NDUFS4
protein_coding	KCNN3
protein_coding	PRODH
protein_coding	SRSF4
protein_coding	C18orf21
protein_coding	BGLAP
protein_coding	PECR
protein_coding	BFSP1
protein_coding	KANSL3
protein_coding	AGPS
protein_coding	RFWD2
protein_coding	OAZ3
protein_coding	C16orf62
protein_coding	METTL4
protein_coding	LDB1
protein_coding	PAWR
protein_coding	ZPBP2
protein_coding	MRPL33
protein_coding	PPP1R16A
protein_coding	NOTCH2NL
protein_coding	YIPF6
protein_coding	KRT73
protein_coding	CEP350
protein_coding	ACPL2
protein_coding	FAM210A
protein_coding	PWWP2A
protein_coding	OTOF
protein_coding	CLEC7A
protein_coding	ETFDH
protein_coding	BTBD7
protein_coding	CFAP221
protein_coding	MOGAT1
protein_coding	ZNF799
protein_coding	SYNGR3
protein_coding	VAT1
protein_coding	DUSP14
protein_coding	NDOR1
protein_coding	CA7
protein_coding	FLI1
protein_coding	GOLGA8Q
protein_coding	TBC1D25

protein_coding	MAFF
protein_coding	CYTIP
protein_coding	DKFZp686O16217
protein_coding	TRIL
protein_coding	RP11-508N12.4
protein_coding	SERPINA5
protein_coding	LGR5
protein_coding	TRIM41
protein_coding	MPZL1
protein_coding	ESD
protein_coding	PEAR1
protein_coding	C6orf1
protein_coding	COLEC12
protein_coding	SHOX2
protein_coding	C15orf39
protein_coding	CEP192
protein_coding	THEM5
protein_coding	STOML2
protein_coding	CPVL
protein_coding	GDAP1L1
protein_coding	CABLES2
protein_coding	LOC389602
protein_coding	OTP
protein_coding	PINX1
protein_coding	PSMC4
protein_coding	ADCYAP1R1
protein_coding	TCP11L2
protein_coding	ABCA7
protein_coding	IGFN1
protein_coding	NIPSNAP3B
protein_coding	RCN3
protein_coding	ALLC
protein_coding	DNAAF2
protein_coding	FGGY
protein_coding	C3orf79
protein_coding	EFCC1
protein_coding	DLK2
protein_coding	GALNT1
protein_coding	PPP1CA
protein_coding	TIGD6
protein_coding	ACTR8
protein_coding	RRM2B
protein_coding	KCNK7
protein_coding	DEFB130
protein_coding	CCDC105
protein_coding	EPHA5
protein_coding	DUSP19

protein_coding	ECE1
protein_coding	SQLE
protein_coding	SERPINC1
protein_coding	HTRA3
protein_coding	MIIP
protein_coding	CLIC1
protein_coding	ANKHD1-EIF4EBP3
protein_coding	C1orf233
protein_coding	EMP2
protein_coding	CNIH2
protein_coding	THAP1
protein_coding	RPL13A
protein_coding	PTPN14
protein_coding	DEPDC4
protein_coding	FADS6
protein_coding	FGR
protein_coding	PRPF38A
protein_coding	PPHLN1
protein_coding	IGSF22
protein_coding	PRTFDC1
protein_coding	GPCPD1
protein_coding	CLASP1
protein_coding	HMGN4
protein_coding	SPTB
protein_coding	AX747379
protein_coding	GPRC5C
protein_coding	TFB1M
protein_coding	UBE2Q2
protein_coding	MRPL52
protein_coding	SWAP70
protein_coding	PABPC1L2A
protein_coding	TMEM158
protein_coding	TUBB3
protein_coding	PGM2
protein_coding	FLII
protein_coding	KBTBD4
protein_coding	PYY
protein_coding	ZNF140
protein_coding	AZI2
protein_coding	KLRG1
protein_coding	CEP78
protein_coding	TMEM78
protein_coding	LRIG1
protein_coding	CCDC181
protein_coding	LZTS1
protein_coding	CAMKK1
protein_coding	AAK1

protein_coding	MTSS1
protein_coding	VMO1
protein_coding	RNF181
protein_coding	YPEL2
protein_coding	ARMC9
protein_coding	CNTRL
protein_coding	DAZAP1
protein_coding	SLCO4C1
protein_coding	AL645922.1
protein_coding	RSC1A1
protein_coding	PARN
protein_coding	GLT1D1
protein_coding	PKM
protein_coding	RPUSD2
protein_coding	ALG13
protein_coding	AQP5
protein_coding	CHST8
protein_coding	MAPK11
protein_coding	TTN
protein_coding	ATG14
protein_coding	IGF1R
protein_coding	OR7C2
protein_coding	MRPL47
protein_coding	NUDT3
protein_coding	ZNHIT2
protein_coding	RPA4
protein_coding	MTX3
protein_coding	TYW5
protein_coding	ABCC4
protein_coding	SGMS1
protein_coding	NSUN6
protein_coding	ICA1L
protein_coding	PRDM5
protein_coding	CTD-2228K2.5
protein_coding	LRRC59
protein_coding	ZNF532
protein_coding	KANSL2
protein_coding	GRM7
protein_coding	BTG3
protein_coding	RPS8
protein_coding	FKBPL
protein_coding	TLDC1
protein_coding	HACL1
protein_coding	ZNF609
protein_coding	CDK5RAP3
protein_coding	KIR2DL4
protein_coding	PHYKPL

protein_coding	TAF1B
protein_coding	BARHL2
protein_coding	CIPC
protein_coding	AC006116.20
protein_coding	AP000889.3
protein_coding	CEP162
protein_coding	PCK2
protein_coding	GDA
protein_coding	GCN1
protein_coding	SRY
protein_coding	CCKBR
protein_coding	DPM3
protein_coding	RSL24D1
protein_coding	TMPRSS7
protein_coding	PCDHB13
protein_coding	C19orf84
protein_coding	MAP4K3
protein_coding	OR2D3
protein_coding	RSPH6A
protein_coding	ALDH1A3
protein_coding	KCNH2
protein_coding	SLC22A8
protein_coding	ACTN3
protein_coding	CORIN
protein_coding	FNIP2
protein_coding	HEATR5A
protein_coding	TMED5
protein_coding	CBX6
protein_coding	SYNGR1
protein_coding	RALBP1
protein_coding	PIAS4
protein_coding	RFC5
protein_coding	MARC2
protein_coding	C17orf82
protein_coding	FAM19A5
protein_coding	TCEB1
protein_coding	PIK3IP1
protein_coding	C10orf88
protein_coding	ZNF106
protein_coding	MBLAC1
protein_coding	ATMIN
protein_coding	AL162407.1
protein_coding	FAM193B
protein_coding	NUBP1
protein_coding	PRRC2C
protein_coding	FUBP1
protein_coding	NRG1

protein_coding	NRBP2
protein_coding	DLST
protein_coding	OR9K2
protein_coding	TMEM106C
protein_coding	KLHL15
protein_coding	PLEKHF1
protein_coding	TTC23L
protein_coding	KANK1
protein_coding	ZNF705B
protein_coding	TIAF1
protein_coding	RP11-565P22.6
protein_coding	MINPP1
protein_coding	SCN11A
protein_coding	SAPCD2
protein_coding	SLC24A6
protein_coding	NDUFAF6
protein_coding	PDZD11
protein_coding	VWA1
protein_coding	RPA2
protein_coding	NAALADL2
protein_coding	VEGFA
protein_coding	LOC100129083
protein_coding	APOL5
protein_coding	SEMA6D
protein_coding	SYP
protein_coding	C18orf8
protein_coding	C20orf85
protein_coding	PEX11B
protein_coding	ACTL6B
protein_coding	MARCH7
protein_coding	C15orf41
protein_coding	TRIM28
protein_coding	RNF125
protein_coding	HSBP1L1
protein_coding	WDR27
protein_coding	DECR2
protein_coding	RPS24
protein_coding	TAPBPL
protein_coding	AC002472.13
protein_coding	DEPDC7
protein_coding	TCEANC2
protein_coding	SMC2
protein_coding	RP11-1055B8.6
protein_coding	TREX1
protein_coding	TAGLN
protein_coding	TMPPE
protein_coding	GPR137B

protein_coding	GNL2
protein_coding	CSN1S1
protein_coding	DKFZp434K191
protein_coding	PRSS45
protein_coding	TAF1A
protein_coding	LOC730183
protein_coding	PI16
protein_coding	SURF6
protein_coding	TAS2R10
protein_coding	CDC26
protein_coding	AC092687.4
protein_coding	MRPS23
protein_coding	GP6
protein_coding	TMEM182
protein_coding	SNTG2
protein_coding	FBXO34
protein_coding	RPL10A
protein_coding	LOC100133445
protein_coding	LOC101927322
protein_coding	SPRR4
protein_coding	TMEM52B
protein_coding	GNB1L
protein_coding	C5
protein_coding	PXMP2
protein_coding	CD37
protein_coding	SPATA7
protein_coding	RGMB
protein_coding	ATAD1
protein_coding	MAMDC2
protein_coding	MRFAP1
protein_coding	CPPED1
protein_coding	DPT
protein_coding	USP21
protein_coding	OPLAH
protein_coding	BAI1
protein_coding	CTNNAL1
protein_coding	PTGR2
protein_coding	PLEKHM2
protein_coding	BTN3A2
protein_coding	TNKS2
protein_coding	HOOK1
protein_coding	LRRC37A2
protein_coding	SHMT1
protein_coding	PHOX2A
protein_coding	KIF7
protein_coding	CDK18
protein_coding	OXNAD1

protein_coding	ARL15
protein_coding	PHF21A
protein_coding	PTCD3
protein_coding	STRA6
protein_coding	TIMM8B
protein_coding	FAM83C
protein_coding	DNAH3
protein_coding	ZFHX2
protein_coding	CD177
protein_coding	MYO16
protein_coding	NKAIN2
protein_coding	RP11-47I22.3
protein_coding	SNAPC3
protein_coding	AC007461.1
protein_coding	FZD7
protein_coding	BATF2
protein_coding	LRRC25
protein_coding	SNUPN
protein_coding	GHR
protein_coding	TMEM127
protein_coding	BRCC3
protein_coding	NCCRP1
protein_coding	AUP1
protein_coding	RSPO4
protein_coding	SYNJ2BP
protein_coding	JPH1
protein_coding	FAM199X
protein_coding	HYAL2
protein_coding	FAM104B
protein_coding	PLIN2
protein_coding	SCIMP
protein_coding	RPN1
protein_coding	LINC01124
protein_coding	CTU2
protein_coding	HIPK1
protein_coding	TTF2
protein_coding	TPI1
protein_coding	DPAGT1
protein_coding	M1AP
protein_coding	PTCHD2
protein_coding	MLTK
protein_coding	TRMT12
protein_coding	MOBP
protein_coding	RNFT2
protein_coding	TRIP6
protein_coding	AX748313
protein_coding	AAAS

protein_coding	SP140
protein_coding	TDRD5
protein_coding	ETV5
protein_coding	ITGA2
protein_coding	PGA4
protein_coding	LAMA5
protein_coding	MAGEE2
protein_coding	UBR2
protein_coding	PHF7
protein_coding	GEMIN7
protein_coding	FAM135B
protein_coding	TNNI1
protein_coding	GATAD1
protein_coding	PM20D1
protein_coding	PROSER1
protein_coding	ACRC
protein_coding	COL25A1
protein_coding	CTRB2
protein_coding	TMEM145
protein_coding	SARS2
protein_coding	BCL2L12
protein_coding	TBC1D3H
protein_coding	KDM4D
protein_coding	PLA2G5
protein_coding	SLC44A4
protein_coding	IGLL5
protein_coding	TAF5L
protein_coding	UQCC2
protein_coding	ADGRG2
protein_coding	FTSJ3
protein_coding	LRTM2
protein_coding	SLC46A3
protein_coding	SLC22A25
protein_coding	PQLC2
protein_coding	VKORC1
protein_coding	AC092850.1
protein_coding	MICA
protein_coding	APH1A
protein_coding	TIAL1
protein_coding	ORAI2
protein_coding	TUSC2
protein_coding	TTLL11
protein_coding	ADIPOR2
protein_coding	ATF6
protein_coding	LRRC3B
protein_coding	RRNAD1
protein_coding	MFN1

protein_coding	ZFP41
protein_coding	FHL1
protein_coding	INTS9
protein_coding	ARL10
protein_coding	COL12A1
protein_coding	SPNS2
protein_coding	GAMT
protein_coding	RP11-162P23.2
protein_coding	SPINK5
protein_coding	CNTD1
protein_coding	TTC39C
protein_coding	ADAMTS19
protein_coding	TBC1D22A
protein_coding	TRABD2B
protein_coding	NCOA3
protein_coding	SGOL2
protein_coding	D2HGDH
protein_coding	OR2S2
protein_coding	RGR
protein_coding	STX19
protein_coding	BV13S6J2.1
protein_coding	C11orf30
protein_coding	USE1
protein_coding	FCRLB
protein_coding	SMIM17
protein_coding	TMEM97
protein_coding	PCDHGB1
protein_coding	MYBBP1A
protein_coding	TMEM175
protein_coding	CGREF1
protein_coding	IDI1
protein_coding	EPHX3
protein_coding	DNAL4
protein_coding	PAN2
protein_coding	OR51F2
protein_coding	STAR
protein_coding	FBLN7
protein_coding	SURF4
protein_coding	TCTN3
protein_coding	SLC37A3
protein_coding	RTF1
protein_coding	TRIQK
protein_coding	GRAP
protein_coding	SUSD6
protein_coding	KDM5B
protein_coding	XYLT2
protein_coding	ZCCHC7

protein_coding	TAPBP
protein_coding	HSD17B8
protein_coding	URB2
protein_coding	COPE
protein_coding	XPO7
protein_coding	BMI1
protein_coding	ISX
protein_coding	ZSCAN29
protein_coding	HGC6.3
protein_coding	RARRES2
protein_coding	NAMPTL
protein_coding	POLR1D
protein_coding	MYL12B
protein_coding	PUM1
protein_coding	DMRT1
protein_coding	DNAI2
protein_coding	HCFC2
protein_coding	TLR4
protein_coding	TRPV5
protein_coding	NT5C
protein_coding	MLKL
protein_coding	ZHX2
protein_coding	MRPL32
protein_coding	MTAP
protein_coding	CDC42EP5
protein_coding	SPATA31D1
protein_coding	CCDC79
protein_coding	MAB21L2
protein_coding	BIVM
protein_coding	GRHL1
protein_coding	SF3B3
protein_coding	TGM5
protein_coding	SRP72
protein_coding	BRMS1
protein_coding	RP11-160N1.10
protein_coding	AL807752.1
protein_coding	COX18
protein_coding	SLC10A3
protein_coding	PDCD2L
protein_coding	MBD3L2
protein_coding	AC129492.6
protein_coding	C10orf2
protein_coding	HMGCL
protein_coding	ZP1
protein_coding	UFD1L
protein_coding	AX747167
protein_coding	BAMBI

protein_coding	ZNF79
protein_coding	TMEM14B
protein_coding	NXF1
protein_coding	KLRC2
protein_coding	C1orf43
protein_coding	SLC35C2
protein_coding	TFDP2
protein_coding	CCHCR1
protein_coding	HSPA1L
protein_coding	SLC38A5
protein_coding	FLVCR1
protein_coding	PANK2
protein_coding	MAN2A2
protein_coding	ST8SIA1
protein_coding	PDCD6IP
protein_coding	LINC01100
protein_coding	COTL1
protein_coding	ZNF790
protein_coding	PGF
protein_coding	TBX21
protein_coding	OR4F21
protein_coding	KCNN2
protein_coding	CDYL2
protein_coding	PYROXD2
protein_coding	METTL6
protein_coding	XIRP2
protein_coding	DFNB59
protein_coding	FAM131B
protein_coding	ACAD8
protein_coding	GOSR2
protein_coding	NXT2
protein_coding	EPS15L1
protein_coding	TMCO1
protein_coding	NIPSNAP1
protein_coding	PTPN18
protein_coding	HEXA
protein_coding	PEX5
protein_coding	TTC9C
protein_coding	PKD2L2
protein_coding	FANCG
protein_coding	RP11-553A10.1
protein_coding	KDM2A
protein_coding	SAXO2
protein_coding	POTEJ
protein_coding	CRCP
protein_coding	RAB1B
protein_coding	MRPS9

protein_coding	DMXL1
protein_coding	HCK
protein_coding	SLC1A6
protein_coding	NUPL2
protein_coding	TMED3
protein_coding	RANBP17
protein_coding	ERCC6
protein_coding	UBE4A
protein_coding	GPR113
protein_coding	CRY2
protein_coding	DCUN1D2
protein_coding	ELMO2
protein_coding	FBXL13
protein_coding	MT1M
protein_coding	RPS27A
protein_coding	WDR26
protein_coding	RXRB
protein_coding	ZNF827
protein_coding	RAE1
protein_coding	CPLX4
protein_coding	RBM25
protein_coding	ZFYVE9
protein_coding	GPR3
protein_coding	ARHGEF25
protein_coding	CCDC28B
protein_coding	DKC1
protein_coding	SIDT2
protein_coding	NUTM2G
protein_coding	DKFZP779L1853
protein_coding	SMARCA1
protein_coding	FABP7
protein_coding	NHLH2
protein_coding	USP49
protein_coding	RPLP1
protein_coding	POLG
protein_coding	ATP5G2
protein_coding	VGLL4
protein_coding	FAM105B
protein_coding	TNFRSF1A
protein_coding	GPAM
protein_coding	AC011530.4
protein_coding	SYTL5
protein_coding	CORO1B
protein_coding	SRA1
protein_coding	SLC22A18
protein_coding	SKIL
protein_coding	FUT8

protein_coding	SSTR2
protein_coding	IL10RA
protein_coding	HAUS6
protein_coding	HACD2
protein_coding	SETDB2
protein_coding	INSL5
protein_coding	CLPB
protein_coding	BCOR
protein_coding	SLC16A9
protein_coding	GIGYF2
protein_coding	ATG10
protein_coding	WBP2NL
protein_coding	ZNF354A
protein_coding	RBBP6
protein_coding	NR1H2
protein_coding	ZRANB1
protein_coding	ELK4
protein_coding	HHLA2
protein_coding	DOT1L
protein_coding	TCHP
protein_coding	C19orf53
protein_coding	ADARB1
protein_coding	CHST2
protein_coding	ZRANB3
protein_coding	KIAA0913
protein_coding	MAOA
protein_coding	CDC14B
protein_coding	USP1
protein_coding	KCTD19
protein_coding	SUOX
protein_coding	NOL4L
protein_coding	OGFR
protein_coding	PRR30
protein_coding	AMOTL1
protein_coding	PIANP
protein_coding	B3GALT1
protein_coding	HECTD2
protein_coding	PPP1R3E
protein_coding	MORN2
protein_coding	KIAA1467
protein_coding	SLC7A14
protein_coding	MAN2B1
protein_coding	BNIP1
protein_coding	EFHC1
protein_coding	FAM117A
protein_coding	UFL1
protein_coding	ASCC1

protein_coding	SRSF8
protein_coding	MAP3K14
protein_coding	CDCA5
protein_coding	TTLL4
protein_coding	SATL1
protein_coding	MAGT1
protein_coding	PARK2
protein_coding	ATXN3
protein_coding	KLC2
protein_coding	DTHD1
protein_coding	RPS6KB1
protein_coding	PPP1CB
protein_coding	BC127192
protein_coding	ADA
protein_coding	MRPS35
protein_coding	AK316321
protein_coding	ERMARD
protein_coding	ANKRD17
protein_coding	FAM107B
protein_coding	C4orf50
protein_coding	NSL1
protein_coding	TMEM14C
protein_coding	SEC22B
protein_coding	RNF215
protein_coding	AGAP5
protein_coding	PLEKHM3
protein_coding	CTD-2192J16.24
protein_coding	BRCA1
protein_coding	SC5D
protein_coding	HDAC4
protein_coding	TMEM38B
protein_coding	CLGN
protein_coding	ADAM9
protein_coding	SLC9A1
protein_coding	DHODH
protein_coding	MILR1
protein_coding	CFD
protein_coding	NSMCE4A
protein_coding	TRMT61B
protein_coding	WDR49
protein_coding	TMEM241
protein_coding	ADNP
protein_coding	STT3B
protein_coding	C9orf40
protein_coding	NCAPD2
protein_coding	SHISA9
protein_coding	PLEKHJ1

protein_coding	CTF1
protein_coding	CD101
protein_coding	WWOX
protein_coding	TRDMT1
protein_coding	BBX
protein_coding	GPR12
protein_coding	GRB10
protein_coding	TMEM61
protein_coding	TXLNG
protein_coding	KIAA1958
protein_coding	NANOS1
protein_coding	OARD1
protein_coding	DAZAP2
protein_coding	CEACAM4
protein_coding	PLA1A
protein_coding	G2E3
protein_coding	CORO2B
protein_coding	PTPN1
protein_coding	AQP2
protein_coding	GPR89A
protein_coding	CTSZ
protein_coding	HS2ST1
protein_coding	FGFR3
protein_coding	TNFAIP8L2-SCNM1
protein_coding	FCN3
protein_coding	DKFZP667F0711
protein_coding	TXNL4B
protein_coding	AATF
protein_coding	NMNAT2
protein_coding	IL11RA
protein_coding	KRTAP4-16P
protein_coding	EML4
protein_coding	BCAT1
protein_coding	MPP4
protein_coding	TAOK2
protein_coding	TLE4
protein_coding	KCNIP2
protein_coding	ZBTB41
protein_coding	ABCF3
protein_coding	ARIH1
protein_coding	FBXO25
protein_coding	ZNF251
protein_coding	RSAD1
protein_coding	PLOD1
protein_coding	RBCK1
protein_coding	ACSM5
protein_coding	RPL22L1

protein_coding	INTS10
protein_coding	FAT3
protein_coding	BAZ2A
protein_coding	PPIL3
protein_coding	EFNA1
protein_coding	MYL12A
protein_coding	HMG20B
protein_coding	CS
protein_coding	ERP29
protein_coding	MEIOB
protein_coding	RP11-1105G2.3
protein_coding	NR2C1
protein_coding	OR6Y1
protein_coding	FAM64A
protein_coding	SAMD9L
protein_coding	CRLF2
protein_coding	KAT7
protein_coding	NPM1
protein_coding	AV2S1A1
protein_coding	PRDM1
protein_coding	AKAP10
protein_coding	CCDC126
protein_coding	LINC00649
protein_coding	CSNK1A1L
protein_coding	KCNA5
protein_coding	HAPLN2
protein_coding	UFSP2
protein_coding	GRIK1
protein_coding	CTD-2368P22.1
protein_coding	KCNB2
protein_coding	BC043620
protein_coding	C7orf63
protein_coding	AHCY
protein_coding	ODF3L2
protein_coding	AC124890.1
protein_coding	EMILIN3
protein_coding	RELA
protein_coding	OR10C1
protein_coding	AP5S1
protein_coding	CCDC159
protein_coding	SUPT6H
protein_coding	USP28
protein_coding	ZBTB3
protein_coding	PNLDC1
protein_coding	CLDN6
protein_coding	RPS5
protein_coding	SOST

protein_coding	PRAM1
protein_coding	PPFIA4
protein_coding	PDRG1
protein_coding	HLA-DPA1
protein_coding	TMEM159
protein_coding	GAB3
protein_coding	KIAA1715
protein_coding	CUL7
protein_coding	FBXO16
protein_coding	TMEM64
protein_coding	MRPL17
protein_coding	POLR3D
protein_coding	AMER3
protein_coding	TIFAB
protein_coding	GATAD2A
protein_coding	NME2
protein_coding	MLH1
protein_coding	CCDC90B
protein_coding	CRY1
protein_coding	CAMSAP1
protein_coding	IL34
protein_coding	AMT
protein_coding	RPL36A-HNRNPH2
protein_coding	C12orf57
protein_coding	IFT88
protein_coding	KIF18B
protein_coding	GP1BB
protein_coding	TMEM117
protein_coding	ICAM4
protein_coding	ANAPC16
protein_coding	C9orf91
protein_coding	TGOLN2
protein_coding	INA
protein_coding	SPAG1
protein_coding	TMEM91
protein_coding	ZNF185
protein_coding	WRNIP1
protein_coding	SPATA22
protein_coding	RAB3GAP2
protein_coding	MS4A6A
protein_coding	P3H4
protein_coding	TXNDC17
protein_coding	ARHGEF19
protein_coding	KRT74
protein_coding	TMEM136
protein_coding	CDK19
protein_coding	CALHM2

protein_coding	MAEL
protein_coding	C2orf15
protein_coding	NUTM2F
protein_coding	SORCS2
protein_coding	SNX8
protein_coding	LIN7B
protein_coding	CCDC12
protein_coding	SULF2
protein_coding	CARNS1
protein_coding	VDAC2
protein_coding	PACS1
protein_coding	HTR7
protein_coding	ARRDC2
protein_coding	LINC01272
protein_coding	GOSR1
protein_coding	C10orf137
protein_coding	NUP54
protein_coding	THOC5
protein_coding	GPATCH3
protein_coding	PRSS35
protein_coding	RTP5
protein_coding	GPX3
protein_coding	DEFB131
protein_coding	PABPC1
protein_coding	GXYLT2
protein_coding	EIF4EBP2
protein_coding	PDE11A
protein_coding	DKK2
protein_coding	HSD11B1
protein_coding	MAML3
protein_coding	NECAP1
protein_coding	RAB30
protein_coding	TMEM140
protein_coding	NPHP3-ACAD11
protein_coding	HBA2
protein_coding	TSPAN4
protein_coding	UCN
protein_coding	SLC27A4
protein_coding	MED8
protein_coding	AVP
protein_coding	PLCB4
protein_coding	AZU1
protein_coding	SLC41A3
protein_coding	GSKIP
protein_coding	TIFA
protein_coding	LIN54
protein_coding	PDE5A

protein_coding	DAP3
protein_coding	ARHGDIB
protein_coding	RPA3-AS1
protein_coding	SMIM13
protein_coding	ROMO1
protein_coding	STIP1
protein_coding	STX5
protein_coding	SIRT6
protein_coding	GK
protein_coding	ZNF81
protein_coding	REXO2
protein_coding	ALYREF
protein_coding	RPF1
protein_coding	GLT8D1
protein_coding	BRWD3
protein_coding	C14orf1
protein_coding	IKBKAP
protein_coding	RYR1
protein_coding	CNTNAP3
protein_coding	PSORS1C1
protein_coding	PON1
protein_coding	LYRM1
protein_coding	MORN5
protein_coding	C10orf11
protein_coding	HSD17B11
protein_coding	TMEM169
protein_coding	PNMA2
protein_coding	RP11-1021N1.1
protein_coding	ALG5
protein_coding	DNAJC25
protein_coding	TRMT6
protein_coding	LOR
protein_coding	AL359736.1
protein_coding	NBPF16
protein_coding	SH3TC1
protein_coding	CKAP2
protein_coding	STAM2
protein_coding	ULK2
protein_coding	TMEM134
protein_coding	NYAP2
protein_coding	C1orf216
protein_coding	KIF25
protein_coding	INPPL1
protein_coding	LYZL4
protein_coding	ATG7
protein_coding	FER1L5
protein_coding	FAHD1

protein_coding	SNCG
protein_coding	CASP7
protein_coding	BAG2
protein_coding	RABL5
protein_coding	TUBGCP3
protein_coding	CASP12
protein_coding	E2F2
protein_coding	GNL3
protein_coding	TMEM245
protein_coding	C8orf44
protein_coding	ORC4
protein_coding	MSH3
protein_coding	C1orf234
protein_coding	DNAJC2
protein_coding	RTN2
protein_coding	SYT12
protein_coding	NCOA4
protein_coding	CLDN20
protein_coding	MSANTD3-TMEFF1
protein_coding	STPG2
protein_coding	TUBG1
protein_coding	PRB3
protein_coding	CPSF2
protein_coding	FCHSD2
protein_coding	IQCB1
protein_coding	C14orf93
protein_coding	SH3GL1
protein_coding	CARTPT
protein_coding	SYNPO2
protein_coding	WDR53
protein_coding	RDH10
protein_coding	ZC3H18
protein_coding	H3F3A
protein_coding	TRIM64C
protein_coding	NACC1
protein_coding	COL7A1
protein_coding	SLC24A1
protein_coding	RB1
protein_coding	WDR34
protein_coding	PGBD3
protein_coding	WDR83OS
protein_coding	PTH1R
protein_coding	CTTNBP2NL
protein_coding	PAGE5
protein_coding	CABLES1
protein_coding	FAM27D1
protein_coding	CFAP69

protein_coding	HEXIM2
protein_coding	WWC3
protein_coding	FLJ46906
protein_coding	GRAMD1B
protein_coding	SLC26A6
protein_coding	TELO2
protein_coding	NEIL2
protein_coding	PIGA
protein_coding	APIP
protein_coding	TBL1X
protein_coding	ZIC2
protein_coding	SERAC1
protein_coding	EGFL8
protein_coding	ZFAND2B
protein_coding	GDF15
protein_coding	YBX1
protein_coding	PARP12
protein_coding	GABRA3
protein_coding	CLDN34
protein_coding	ZNF845
protein_coding	POLDIP2
protein_coding	CBLN1
protein_coding	SLC35D1
protein_coding	ARMCX5
protein_coding	SNIP1
protein_coding	NDUFA4L2
protein_coding	TGS1
protein_coding	EVI2B
protein_coding	EWSR1
protein_coding	RNF144B
protein_coding	ANKRD52
protein_coding	IGSF23
protein_coding	FOXD4L2
protein_coding	PTRH1
protein_coding	MFSD6
protein_coding	IRF7
protein_coding	FOXE3
protein_coding	TRIT1
protein_coding	HM13
protein_coding	OR10A2
protein_coding	CENPQ
protein_coding	HMSD
protein_coding	CD300A
protein_coding	C8orf31
protein_coding	THUMPD3
protein_coding	AC040977.1
protein_coding	SGK2

protein_coding	TBC1D2
protein_coding	INF2
protein_coding	TCOF1
protein_coding	PGM1
protein_coding	KPNA2
protein_coding	TUBA8
protein_coding	TAF13
protein_coding	AIFM2
protein_coding	KIR3DX1
protein_coding	TDGF1
protein_coding	LOC494150
protein_coding	TRIM56
protein_coding	TEP1
protein_coding	SDC3
protein_coding	ZADH2
protein_coding	NAA20
protein_coding	TRAF7
protein_coding	CEP85
protein_coding	TMEM138
protein_coding	A3GALT2
protein_coding	CACNG5
protein_coding	DDX11
protein_coding	TOX2
protein_coding	PDSS1
protein_coding	TARDBP
protein_coding	PLD4
protein_coding	FAM209B
protein_coding	RRP8
protein_coding	KLF7
protein_coding	PADI6
protein_coding	DCAF12L1
protein_coding	HIST4H4
protein_coding	KLHL24
protein_coding	NPBWR1
protein_coding	ANAPC13
protein_coding	ECD
protein_coding	ZBED6CL
protein_coding	PHKB
protein_coding	HPN
protein_coding	PLA2G12A
protein_coding	FAM71E1
protein_coding	PLEKHG5
protein_coding	SYN3
protein_coding	CRYGN
protein_coding	CALN1
protein_coding	CACNA1D
protein_coding	ATP13A5

protein_coding	FCER1A
protein_coding	RP11-426L16.10
protein_coding	CHRM5
protein_coding	MAPKAPK5
protein_coding	CALR
protein_coding	ITPRIPL2
protein_coding	LRRC45
protein_coding	CHST10
protein_coding	AK130759
protein_coding	PGBD4
protein_coding	RARS2
protein_coding	MAP1LC3B2
protein_coding	UPP1
protein_coding	EEPD1
protein_coding	SCGB2A1
protein_coding	NOSIP
protein_coding	NGDN
protein_coding	KLHL20
protein_coding	CSTF2
protein_coding	PRKAA2
protein_coding	MED13L
protein_coding	CCDC23
protein_coding	WDR45
protein_coding	COX4I2
protein_coding	BPHL
protein_coding	TM7SF3
protein_coding	SLC25A34
protein_coding	SLC4A1
protein_coding	DMTF1
protein_coding	SUDS3
protein_coding	GZF1
protein_coding	ZNF671
protein_coding	PRDM4
protein_coding	KRT78
protein_coding	SYDE1
protein_coding	SLC29A1
protein_coding	MIB1
protein_coding	PIK3R5
protein_coding	C9orf123
protein_coding	AK095081
protein_coding	GTPBP8
protein_coding	SHANK3
protein_coding	DYNLT1
protein_coding	NPHP3
protein_coding	ACER3
protein_coding	RARB
protein_coding	ZBTB18

protein_coding	RHEBL1
protein_coding	DYRK4
protein_coding	RGMA
protein_coding	POGLUT1
protein_coding	FAM169A
protein_coding	PCNXL3
protein_coding	AGAP10
protein_coding	AL161915.1
protein_coding	CASP10
protein_coding	KDM3B
protein_coding	MET
protein_coding	CCL27
protein_coding	SLC41A1
protein_coding	FKBP8
protein_coding	TARBP1
protein_coding	ARSH
protein_coding	PFKFB3
protein_coding	CCL4L2
protein_coding	OST4
protein_coding	ING5
protein_coding	LSM14A
protein_coding	WIPF2
protein_coding	GORAB
protein_coding	CTB-102L5.4
protein_coding	DUSP15
protein_coding	PTH2R
protein_coding	LYPLA1
protein_coding	LY86
protein_coding	PRPF3
protein_coding	PLCL2
protein_coding	ILK
protein_coding	CYBB
protein_coding	C16orf89
protein_coding	ABHD11
protein_coding	PKMYT1
protein_coding	ENTPD1
protein_coding	DUX4L4
protein_coding	HIST1H2BO
protein_coding	POLR3K
protein_coding	PABPC1L2B
protein_coding	GRIK3
protein_coding	DDX59
protein_coding	SLC26A11
protein_coding	CCL3L1
protein_coding	BRIX1
protein_coding	CEP19
protein_coding	DDX31

protein_coding	CPSF3
protein_coding	AL035252.1
protein_coding	ARL14EP
protein_coding	PDCD4
protein_coding	AC069368.3
protein_coding	CTAGE9
protein_coding	RP11-247C2.2
protein_coding	ZC3H10
protein_coding	NOS1AP
protein_coding	SLC25A21-AS1
protein_coding	AC073610.5
protein_coding	VPS16
protein_coding	PNISR
protein_coding	ASB2
protein_coding	NEURL1
protein_coding	WWC1
protein_coding	SMPD1
protein_coding	CDK20
protein_coding	DNAH8
protein_coding	EXOSC7
protein_coding	CENPP
protein_coding	CMTM6
protein_coding	SCAF11
protein_coding	CCDC85A
protein_coding	GPR98
protein_coding	BLVRB
protein_coding	NAA10
protein_coding	RAB4A
protein_coding	TCN2
protein_coding	HEPHL1
protein_coding	AKAP13
protein_coding	UPRT
protein_coding	WASH1
protein_coding	PAXBP1
protein_coding	NAP1L6
protein_coding	MEGF9
protein_coding	PMEPA1
protein_coding	RBL2
protein_coding	KPNA4
protein_coding	ALKBH3
protein_coding	SP1
protein_coding	ZNF783
protein_coding	IL1RL2
protein_coding	C21orf128
protein_coding	ANKRD32
protein_coding	BPY2C
protein_coding	SORBS3

protein_coding	OR2AG2
protein_coding	KLHL35
protein_coding	RP11-248J23.6
protein_coding	AC106876.2
protein_coding	GPATCH11
protein_coding	TMEM218
protein_coding	ARPC1B
protein_coding	ZNF778
protein_coding	MOV10
protein_coding	FHIT
protein_coding	C22orf29
protein_coding	TREX2
protein_coding	KRTAP13-4
protein_coding	PRKDC
protein_coding	SPAG5
protein_coding	HERC5
protein_coding	GGN
protein_coding	AL358113.1
protein_coding	C9orf142
protein_coding	MFSD12
protein_coding	ANKRD30B
protein_coding	SRC
protein_coding	R3HDM1
protein_coding	SFRP2
protein_coding	CATSPER3
protein_coding	UBAP1
protein_coding	ZNF641
protein_coding	CCT5
protein_coding	SBSPON
protein_coding	GTF2A1
protein_coding	ADGRL1
protein_coding	FES
protein_coding	EEF1G
protein_coding	HIP1R
protein_coding	ZNF319
protein_coding	EMC1
protein_coding	XPO5
protein_coding	HLCS
protein_coding	ERP27
protein_coding	SH3RF3
protein_coding	NOP2
protein_coding	DUSP2
protein_coding	PLA2G4E
protein_coding	KCNH6
protein_coding	FANCM
protein_coding	CRYBG3
protein_coding	ELAC2

protein_coding	AP1G2
protein_coding	TBX1
protein_coding	AC008948.1
protein_coding	NIT2
protein_coding	TNFSF18
protein_coding	RAB3IP
protein_coding	ATP6V0E1
protein_coding	NLRP8
protein_coding	FUT4
protein_coding	AK308605
protein_coding	CHRNA5
protein_coding	FAM86C1
protein_coding	TNXB
protein_coding	TMEM141
protein_coding	KIAA1033
protein_coding	MPHOSPH6
protein_coding	MBD6
protein_coding	LIAS
protein_coding	SLC25A32
protein_coding	TRIM46
protein_coding	ELANE
protein_coding	CDC40
protein_coding	PNMAL1
protein_coding	TIPRL
protein_coding	TANGO6
protein_coding	FMO4
protein_coding	LOC100506388
protein_coding	KRTAP4-11
protein_coding	ZNF470
protein_coding	METTL17
protein_coding	RBM15B
protein_coding	LOC100131347
protein_coding	STAM
protein_coding	RINL
protein_coding	KLRC1
protein_coding	NICN1
protein_coding	FDX1
protein_coding	VWA9
protein_coding	ATP8B1
protein_coding	PPP6R2
protein_coding	DIP2A
protein_coding	CCDC59
protein_coding	SRD5A1
protein_coding	SOAT1
protein_coding	ZNF462
protein_coding	CPLX3
protein_coding	SNRPG

protein_coding	PAXIP1
protein_coding	AX748309
protein_coding	RPS6KA4
protein_coding	ROPN1B
protein_coding	BCAM
protein_coding	TRPC3
protein_coding	ZNF549
protein_coding	PCDHB14
protein_coding	AC138655.1
protein_coding	LTBP2
protein_coding	RNF182
protein_coding	POLR3H
protein_coding	PPAPDC1A
protein_coding	AP5Z1
protein_coding	RP11-159G9.5
protein_coding	C19orf45
protein_coding	LTBP3
protein_coding	ALS2
protein_coding	AKAP8L
protein_coding	VAMP7
protein_coding	KIF3B
protein_coding	CECR1
protein_coding	IQGAP1
protein_coding	CEP112
protein_coding	SATB2
protein_coding	BC009321
protein_coding	C1QTNF8
protein_coding	GATSL3
protein_coding	EMG1
protein_coding	ALDH1A2
protein_coding	WDR5
protein_coding	GPR56
protein_coding	YWHAZ
protein_coding	C5orf51
protein_coding	MED23
protein_coding	RPH3AL
protein_coding	RAD51L3-RFFL
protein_coding	MAPK12
protein_coding	UIMC1
protein_coding	NR2F6
protein_coding	DBNL
protein_coding	NDNF
protein_coding	NONO
protein_coding	C3orf18
protein_coding	PIGV
protein_coding	PIGN
protein_coding	SMOC2

protein_coding	NKAIN4
protein_coding	BC022568
protein_coding	TOE1
protein_coding	GLTPD1
protein_coding	RNASEL
protein_coding	MTA1
protein_coding	FBF1
protein_coding	RP11-20I23.1
protein_coding	GRIK4
protein_coding	GLI4
protein_coding	GOLGA8R
protein_coding	TBC1D16
protein_coding	ZNF75A
protein_coding	MED11
protein_coding	MGP
protein_coding	SYMPK
protein_coding	PLSCR4
protein_coding	POLR2M
protein_coding	SEC61G
protein_coding	POLR2H
protein_coding	KIAA1407
protein_coding	NUS1
protein_coding	KRTAP23-1
protein_coding	NCBP2
protein_coding	BTF3
protein_coding	ZBED9
protein_coding	P2RY1
protein_coding	ZNF177
protein_coding	ATP8B2
protein_coding	TCF7L2
protein_coding	ACTG1
protein_coding	TMEM72
protein_coding	BLOC1S6
protein_coding	AC117395.1
protein_coding	SENP5
protein_coding	HIATL1
protein_coding	PTPRCAP
protein_coding	PCNA
protein_coding	PDP2
protein_coding	ZNF619
protein_coding	MAB21L3
protein_coding	APBA1
protein_coding	CEBPG
protein_coding	NEU4
protein_coding	HIF1A
protein_coding	RPL32
protein_coding	GBP2

protein_coding	ACAP2
protein_coding	ZNF24
protein_coding	EXTL3
protein_coding	CDH20
protein_coding	MMAA
protein_coding	ZNF621
protein_coding	WFS1
protein_coding	DQ577714
protein_coding	AP000769.1
protein_coding	GBA
protein_coding	PGAM2
protein_coding	SLC38A3
protein_coding	ZNF133
protein_coding	AP1S3
protein_coding	GLI3
protein_coding	DEFB124
protein_coding	PLXNA2
protein_coding	TWISTNB
protein_coding	NUDT16
protein_coding	TADA2B
protein_coding	ZMYND19
protein_coding	SLC38A1
protein_coding	NGF
protein_coding	CD2AP
protein_coding	NKX2-1
protein_coding	AC096582.1
protein_coding	C5orf60
protein_coding	ZCCHC18
protein_coding	LTBP1
protein_coding	ZBTB21
protein_coding	ZFP3
protein_coding	ADRB3
protein_coding	H3F3B
protein_coding	GOPC
protein_coding	RP11-468E2.6
protein_coding	SYCP3
protein_coding	COG8
protein_coding	C19orf59
protein_coding	PNP
protein_coding	FBN1
protein_coding	NOTCH3
protein_coding	MAGEL2
protein_coding	ANXA1
protein_coding	FAM20B
protein_coding	COLGALT1
protein_coding	C16orf46
protein_coding	P3H1

protein_coding	ADIG
protein_coding	DPP3
protein_coding	SYT6
protein_coding	SDC2
protein_coding	TRAF3
protein_coding	PHOSPHO2
protein_coding	ACOT13
protein_coding	EXD2
protein_coding	RXFP4
protein_coding	UGT8
protein_coding	ASCC3
protein_coding	TMEM41B
protein_coding	IQCF3
protein_coding	ACPT
protein_coding	SRFBP1
protein_coding	COX8C
protein_coding	PPP4R1
protein_coding	NOL9
protein_coding	DCAF8
protein_coding	POLR3E
protein_coding	PLOD2
protein_coding	KHNYN
protein_coding	SMARCC1
protein_coding	RWDD1
protein_coding	CCDC74B
protein_coding	RFX5
protein_coding	OAS2
protein_coding	PRR35
protein_coding	TFAM
protein_coding	INSIG2
protein_coding	NBPF20
protein_coding	ARHGEF37
protein_coding	C19orf67
protein_coding	MICAL1
protein_coding	TUBGCP5
protein_coding	C1orf222
protein_coding	RNF41
protein_coding	C21orf33
protein_coding	FOCAD
protein_coding	FGFR1OP
protein_coding	RFXANK
protein_coding	KPNA6
protein_coding	SCLT1
protein_coding	FOXRED2
protein_coding	PPARD
protein_coding	ATP6V0B
protein_coding	FMNL2

protein_coding	C4orf29
protein_coding	ZNRD1
protein_coding	EBI3
protein_coding	TSPAN32
protein_coding	ZNF770
protein_coding	ADCY8
protein_coding	B3GALNT2
protein_coding	POLDIP3
protein_coding	TRMT10B
protein_coding	UBASH3A
protein_coding	HDGF
protein_coding	ALDH3A2
protein_coding	RNF115
protein_coding	SPATA12
protein_coding	INIP
protein_coding	CPO
protein_coding	ACR
protein_coding	MPPE1
protein_coding	JMJD4
protein_coding	C2orf57
protein_coding	FBXO41
protein_coding	TMEM185B
protein_coding	ACTR2
protein_coding	RBM28
protein_coding	IMPA2
protein_coding	MRPL27
protein_coding	FAM83A
protein_coding	MORF4L1
protein_coding	GNPDA1
protein_coding	TULP2
protein_coding	TIGAR
protein_coding	LIMS1
protein_coding	BOLA2
protein_coding	CTIF
protein_coding	TFAP2C
protein_coding	DAGLB
protein_coding	OR1N1
protein_coding	ARAF
protein_coding	CCDC94
protein_coding	NUP43
protein_coding	UCK2
protein_coding	SNAPC1
protein_coding	IL2
protein_coding	TAS2R14
protein_coding	CCDC157
protein_coding	XRN2
protein_coding	EIF2B5

protein_coding	HADH
protein_coding	BRSK2
protein_coding	TBC1D29
protein_coding	SMARCA5
protein_coding	STK33
protein_coding	CYP4Z1
protein_coding	MGAT3
protein_coding	KATNA1
protein_coding	EDNRB
protein_coding	AX748120
protein_coding	WDR43
protein_coding	GIN1
protein_coding	ABCA9
protein_coding	ZNF573
protein_coding	GPR50
protein_coding	ZNF367
protein_coding	MORC2
protein_coding	TCL1A
protein_coding	INPP5B
protein_coding	NEK11
protein_coding	ASS1
protein_coding	MRAP
protein_coding	ITGBL1
protein_coding	TMEM170B
protein_coding	NXPH3
protein_coding	VWC2
protein_coding	SERPINI2
protein_coding	ATG5
protein_coding	DIAPH1
protein_coding	CCNT2
protein_coding	MINK1
protein_coding	STAT3
protein_coding	MEOX2
protein_coding	TRIM24
protein_coding	NHSL1
protein_coding	SCRN2
protein_coding	KLHL28
protein_coding	FUS
protein_coding	AX747124
protein_coding	SSR2
protein_coding	TAF4
protein_coding	AK307150
protein_coding	UNC13D
protein_coding	FFAR4
protein_coding	VPS13B
protein_coding	L01117
protein_coding	N6AMT1

protein_coding	TMPRSS11B
protein_coding	CAPZA1
protein_coding	AK125726
protein_coding	RP11-831H9.11
protein_coding	SFXN1
protein_coding	RP11-167N24.6
protein_coding	DPEP2
protein_coding	MREG
protein_coding	SFMBT1
protein_coding	ZBED8
protein_coding	UPF2
protein_coding	BOLA1
protein_coding	IRF2
protein_coding	PSMG2
protein_coding	C14orf177
protein_coding	TIMM22
protein_coding	BCL3
protein_coding	DAG1
protein_coding	AGO2
protein_coding	PAN3
protein_coding	TTC4
protein_coding	AGK
protein_coding	MALT1
protein_coding	SERTAD1
protein_coding	FBXO17
protein_coding	UBE3D
protein_coding	SRCIN1
protein_coding	LOXL4
protein_coding	SMEK1
protein_coding	DMRTC1B
protein_coding	ST6GAL1
protein_coding	C1QC
protein_coding	CCDC102B
protein_coding	FBLN2
protein_coding	NPAT
protein_coding	UPF3A
protein_coding	SMG9
protein_coding	NUP35
protein_coding	NAV1
protein_coding	DIEXF
protein_coding	TMEM164
protein_coding	SLC46A2
protein_coding	LEPROTL1
protein_coding	ADRB2
protein_coding	SIRT4
protein_coding	CAPSL
protein_coding	AP1G1

protein_coding	CCDC66
protein_coding	PLAGL2
protein_coding	NVL
protein_coding	STK32A
protein_coding	RNF114
protein_coding	TECR
protein_coding	EDEM2
protein_coding	TM2D1
protein_coding	CHST5
protein_coding	BATF3
protein_coding	RFNG
protein_coding	OR5F1
protein_coding	TFCP2
protein_coding	ZNF460
protein_coding	PDGFA
protein_coding	CASQ2
protein_coding	CEP164
protein_coding	FAM46B
protein_coding	WDR77
protein_coding	CH17-132F21.1
protein_coding	GRN
protein_coding	GYPC
protein_coding	KDELR1
protein_coding	CDIPT
protein_coding	PLSCR3
protein_coding	CAST
protein_coding	MGRN1
protein_coding	C1orf54
protein_coding	GTF2H2C
protein_coding	TBL3
protein_coding	SLC1A4
protein_coding	WNT11
protein_coding	CASC1
protein_coding	AVL9
protein_coding	AC023590.1
protein_coding	MKKS
protein_coding	GPNMB
protein_coding	MAATS1
protein_coding	C11orf91
protein_coding	AIMP1
protein_coding	TKFC
protein_coding	XRCC6
protein_coding	SLC46A1
protein_coding	FITM1
protein_coding	APH1B
protein_coding	MCPH1
protein_coding	NUFIP2

protein_coding	NFKBIL1
protein_coding	AL360181.1
protein_coding	PRPF6
protein_coding	WDR66
protein_coding	CCDC180
protein_coding	BHLHB9
protein_coding	IFT43
protein_coding	TIMP1
protein_coding	PRR23C
protein_coding	ZNF248
protein_coding	ZDHHC18
protein_coding	DCUN1D5
protein_coding	TNNT2
protein_coding	ZNF713
protein_coding	MRPS6
protein_coding	TBCEL
protein_coding	ADRBK1
protein_coding	KCTD9
protein_coding	PMPCA
protein_coding	LYRM4
protein_coding	MRPS18C
protein_coding	CREBBP
protein_coding	KLF10
protein_coding	CHDH
protein_coding	FAM196B
protein_coding	CSE1L
protein_coding	PCDHB11
protein_coding	SLC25A30
protein_coding	C8orf17
protein_coding	FAU
protein_coding	EP400
protein_coding	FANCL
protein_coding	IPO4
protein_coding	PHLPP1
protein_coding	MCOLN1
protein_coding	ARMC5
protein_coding	UFSP1
protein_coding	NRAP
protein_coding	LHB
protein_coding	ROM1
protein_coding	PIGU
protein_coding	SULT1A1
protein_coding	TAP1
protein_coding	FRMD8
protein_coding	GKAP1
protein_coding	CCDC50
protein_coding	SLC30A7

protein_coding	IZUMO1R
protein_coding	C11orf87
protein_coding	PLCG2
protein_coding	P4HA1
protein_coding	CYB5R1
protein_coding	ATP4B
protein_coding	WRN
protein_coding	CC2D1A
protein_coding	KCNA7
protein_coding	FRS2
protein_coding	TBX18
protein_coding	LOC389895
protein_coding	PPP2R5C
protein_coding	TMEM108
protein_coding	AC005606.1
protein_coding	DNAJC11
protein_coding	SMAD3
protein_coding	FKBP1C
protein_coding	IKZF4
protein_coding	TRIM65
protein_coding	LYSMD3
protein_coding	METTL2B
protein_coding	NOL3
protein_coding	NDUFAF4
protein_coding	NUP133
protein_coding	B4GALNT1
protein_coding	PLCG1
protein_coding	ZNF329
protein_coding	C5orf22
protein_coding	HMGB2
protein_coding	CDC42SE1
protein_coding	GNA13
protein_coding	DLL3
protein_coding	TSPYL5
protein_coding	FAM135A
protein_coding	GOLPH3
protein_coding	SMCP
protein_coding	MRM1
protein_coding	AVEN
protein_coding	CHRM1
protein_coding	CCDC89
protein_coding	CCDC25
protein_coding	TUBGCP4
protein_coding	C11orf49
protein_coding	FGD6
protein_coding	EIF3F
protein_coding	DCPS

protein_coding	SH3BGRL
protein_coding	OR5V1
protein_coding	PADI3
protein_coding	RSBN1L
protein_coding	ELAVL1
protein_coding	GSDMD
protein_coding	GOLGA8O
protein_coding	PPP1R11
protein_coding	EIF4A3
protein_coding	PRLR
protein_coding	AC104057.1
protein_coding	RP11-343C2.12
protein_coding	DNAH6
protein_coding	MRPS36
protein_coding	EHD4
protein_coding	NXPH4
protein_coding	KIAA2013
protein_coding	SLC47A1
protein_coding	KAT5
protein_coding	ZFP1
protein_coding	DCAF4L2
protein_coding	ZNF394
protein_coding	INO80B
protein_coding	GRAMD1A
protein_coding	SLMO1
protein_coding	TUBA1B
protein_coding	RHOJ
protein_coding	MUS81
protein_coding	CCNJL
protein_coding	PPP1R32
protein_coding	RP11-625H11.1
protein_coding	CTC-260F20.3
protein_coding	SLC22A1
protein_coding	C11orf54
protein_coding	GET4
protein_coding	KANSL1L
protein_coding	NPY1R
protein_coding	WDR54
protein_coding	ST7L
protein_coding	ZNF543
protein_coding	ARSF
protein_coding	CEACAM19
protein_coding	NOP10
protein_coding	AC008132.13
protein_coding	UBAP1L
protein_coding	PCDHA7
protein_coding	ZNF503

protein_coding	PTCH1
protein_coding	ERRFI1
protein_coding	RCC2
protein_coding	FPGT-TNNI3K
protein_coding	C8orf34
protein_coding	RHOBTB2
protein_coding	CRHBP
protein_coding	SENP3
protein_coding	DVL1
protein_coding	GOLGA7B
protein_coding	ZNF391
protein_coding	CSPG5
protein_coding	AX747988
protein_coding	KLHL42
protein_coding	YPEL1
protein_coding	CCDC58
protein_coding	ZNF548
protein_coding	CAPN9
protein_coding	MTFR1
protein_coding	AK9
protein_coding	NUP62
protein_coding	LPCAT1
protein_coding	PTPRJ
protein_coding	KHDRBS2
protein_coding	DPF1
protein_coding	PRDX6
protein_coding	GTF2H2C_2
protein_coding	PRB1
protein_coding	MEST
protein_coding	SMCR8
protein_coding	TMEM237
protein_coding	SFSWAP
protein_coding	IVNS1ABP
protein_coding	KL
protein_coding	RPF2
protein_coding	ACTR1B
protein_coding	ACAD9
protein_coding	ZNF347
protein_coding	CLDN14
protein_coding	LRRC56
protein_coding	ZKSCAN4
protein_coding	PCDH15
protein_coding	KIAA1430
protein_coding	THTPA
protein_coding	TTC17
protein_coding	TSFM
protein_coding	INTS3

protein_coding	GLG1
protein_coding	MRPS31
protein_coding	DDX51
protein_coding	MTCH2
protein_coding	OR52M1
protein_coding	ZNF3
protein_coding	PLEKHA5
protein_coding	PCK1
protein_coding	ZNF821
protein_coding	DENND6B
protein_coding	ZNF131
protein_coding	KIAA0930
protein_coding	RAB34
protein_coding	ZNF282
protein_coding	RGS3
protein_coding	ZNF322
protein_coding	PTRHD1
protein_coding	TGFB1I1
protein_coding	C16orf93
protein_coding	HIST1H1E
protein_coding	KRT23
protein_coding	MAGEE1
protein_coding	LHFP
protein_coding	MIOS
protein_coding	C14orf166
protein_coding	SCAI
protein_coding	LAPTM4B
protein_coding	SFXN3
protein_coding	TM9SF1
protein_coding	TMEM56-RWDD3
protein_coding	COL18A1
protein_coding	KIAA1737
protein_coding	RP11-514P8.7
protein_coding	GALT
protein_coding	ABRACL
protein_coding	TSSC4
protein_coding	VPS13D
protein_coding	PPP5C
protein_coding	CHD1
protein_coding	ALS2CL
protein_coding	GOLGA5
protein_coding	ZNF197
protein_coding	SPDL1
protein_coding	STX18
protein_coding	VMA21
protein_coding	DYTN
protein_coding	BOK

protein_coding	ACADVL
protein_coding	MGEA5
protein_coding	KLHL21
protein_coding	REL
protein_coding	SPATA24
protein_coding	TTC30A
protein_coding	FBXO38
protein_coding	KCNJ16
protein_coding	RAD54L
protein_coding	AL050302.1
protein_coding	OR2J1
protein_coding	HS3ST1
protein_coding	CLINT1
protein_coding	HEATR6
protein_coding	CUL2
protein_coding	HES2
protein_coding	AX747402
protein_coding	SMARCD2
protein_coding	EPHX2
protein_coding	VRK1
protein_coding	SLC34A1
protein_coding	NPY2R
protein_coding	ZNF431
protein_coding	WDR44
protein_coding	NUP50
protein_coding	CTD-2116N17.1
protein_coding	CC2D2A
protein_coding	IFI44L
protein_coding	CYTH2
protein_coding	PALM3
protein_coding	RTP1
protein_coding	OR4D2
protein_coding	RPA1
protein_coding	ADGRL3
protein_coding	DMRTB1
protein_coding	RER1
protein_coding	ERG
protein_coding	TRAPPC6B
protein_coding	RUSC2
protein_coding	HNRNPU
protein_coding	OR2H1
protein_coding	SNX30
protein_coding	CARD8
protein_coding	NPL
protein_coding	NPFF
protein_coding	TMEM41A
protein_coding	ERN2

protein_coding	GID4
protein_coding	PROK2
protein_coding	INTS8
protein_coding	TMEM33
protein_coding	TOP3A
protein_coding	ATF7IP2
protein_coding	GPR37L1
protein_coding	GPIHBP1
protein_coding	ST6GALNAC2
protein_coding	ZBED3
protein_coding	SRRM3
protein_coding	SP9
protein_coding	TBC1D15
protein_coding	SLC2A1
protein_coding	PRPF18
protein_coding	SYT7
protein_coding	FAM43B
protein_coding	AC009065.1
protein_coding	SLC35F6
protein_coding	OR56B1
protein_coding	SRP54
protein_coding	RNPS1
protein_coding	ASF1A
protein_coding	LAS1L
protein_coding	RAB27A
protein_coding	RP11-343C2.11
protein_coding	IL1RAPL2
protein_coding	PDCD7
protein_coding	DEFB126
protein_coding	HSDL2
protein_coding	ID2
protein_coding	AC012360.2
protein_coding	USP22
protein_coding	SBP1
protein_coding	TIGD5
protein_coding	AC016757.3
protein_coding	MLLT10
protein_coding	YTHDF3
protein_coding	CTXN1
protein_coding	ZNF862
protein_coding	TLE1
protein_coding	AC018470.1
protein_coding	MPDU1
protein_coding	EAF1
protein_coding	MRPS10
protein_coding	DIS3L2
protein_coding	CTAGE8

protein_coding	OR2L3
protein_coding	PCBP2
protein_coding	XIAP
protein_coding	CMC4
protein_coding	TMEM101
protein_coding	CDRT15L2
protein_coding	PPEF1
protein_coding	PRKACA
protein_coding	THAP2
protein_coding	DTWD2
protein_coding	ALX3
protein_coding	RHOF
protein_coding	KRTAP4-9
protein_coding	ZKSCAN7
protein_coding	ZNF487
protein_coding	EIF1AD
protein_coding	NUBPL
protein_coding	GTSF1
protein_coding	MRPL11
protein_coding	MNS1
protein_coding	THADA
protein_coding	FAM98B
protein_coding	ATRX
protein_coding	PIGM
protein_coding	ADCK2
protein_coding	OTOGL
protein_coding	LYRM9
protein_coding	VPS28
protein_coding	CHTOP
protein_coding	NUDCD1
protein_coding	ZNF630
protein_coding	PCDHA10
protein_coding	MAN1B1
protein_coding	NOXO1
protein_coding	ODF3
protein_coding	CRLF3
protein_coding	FAM189B
protein_coding	WFDC2
protein_coding	MTX1
protein_coding	CECR6
protein_coding	AC096677.1
protein_coding	OMD
protein_coding	NRTN
protein_coding	FAM111B
protein_coding	APEX1
protein_coding	PWP1
protein_coding	IQCK

protein_coding	H2AFY
protein_coding	ADGRL4
protein_coding	GABRG3
protein_coding	PTMA
protein_coding	SLC17A1
protein_coding	TIA1
protein_coding	GOLGA8K
protein_coding	PDLIM3
protein_coding	FXR2
protein_coding	C9orf173
protein_coding	ZBTB45
protein_coding	OR10H3
protein_coding	C1orf173
protein_coding	RIMKLA
protein_coding	NDC1
protein_coding	NEMP2
protein_coding	TMEM9
protein_coding	TRIB1
protein_coding	UBTD2
protein_coding	MX1
protein_coding	CBWD3
protein_coding	BRD7
protein_coding	C1S
protein_coding	DRG1
protein_coding	PIWIL1
protein_coding	DTWD1
protein_coding	POU3F2
protein_coding	PCDHB9
protein_coding	HSPA6
protein_coding	TSHZ3
protein_coding	TSPAN15
protein_coding	NYAP1
protein_coding	CORO1C
protein_coding	NADK2
protein_coding	XYLT1
protein_coding	MSANTD2
protein_coding	ARHGAP17
protein_coding	XRCC6BP1
protein_coding	SAMD9
protein_coding	SNRPA1
protein_coding	ACTL6A
protein_coding	CDC25B
protein_coding	PAX6
protein_coding	LRP2BP
protein_coding	BCL11A
protein_coding	TFPT
protein_coding	AGMAT

protein_coding	REV3L
protein_coding	TNPO2
protein_coding	CCDC39
protein_coding	CCL5
protein_coding	TMEM55A
protein_coding	LRP6
protein_coding	CBFA2T3
protein_coding	GNS
protein_coding	FREM3
protein_coding	KRTAP9-9
protein_coding	NDUFC2-KCTD14
protein_coding	PALD1
protein_coding	PPP1R13B
protein_coding	GPR137C
protein_coding	METAP1
protein_coding	MDM1
protein_coding	ALDH1L2
protein_coding	CNR1
protein_coding	DERL1
protein_coding	FAM92A1
protein_coding	SYT3
protein_coding	CT45A3
protein_coding	CHST15
protein_coding	WTH3DI
protein_coding	DNAAF1
protein_coding	HDAC9
protein_coding	TTC12
protein_coding	PCDHGC3
protein_coding	PRICKLE2
protein_coding	CDR2L
protein_coding	ARF1
protein_coding	MCTS1
protein_coding	VPS52
protein_coding	TCTE1
protein_coding	LIPH
protein_coding	NME3
protein_coding	MAPK14
protein_coding	OR9A2
protein_coding	FAM222A
protein_coding	USP18
protein_coding	PRSS37
protein_coding	CLNS1A
protein_coding	SLC13A5
protein_coding	DCTD
protein_coding	MAD2L1
protein_coding	LANCL3
protein_coding	TMEM234

protein_coding	GLMP
protein_coding	PRY
protein_coding	ZNF521
protein_coding	CHADL
protein_coding	PRDX4
protein_coding	OR13J1
protein_coding	C11orf95
protein_coding	CDC42EP2
protein_coding	TNIP2
protein_coding	SSBP3
protein_coding	LDLRAD4
protein_coding	LYPD8
protein_coding	RNF180
protein_coding	TPP2
protein_coding	CCDC78
protein_coding	GREM2
protein_coding	ATPAF2
protein_coding	PRIMPOL
protein_coding	MAD2L1BP
protein_coding	ZUFSP
protein_coding	PCF11
protein_coding	FN3K
protein_coding	ZBTB43
protein_coding	ABHD1
protein_coding	CCDC40
protein_coding	MED31
protein_coding	MVB12B
protein_coding	C9orf16
protein_coding	ALKBH2
protein_coding	ACO1
protein_coding	CLDN24
protein_coding	MAGEC3
protein_coding	GPR152
protein_coding	AX747977
protein_coding	PPM1F
protein_coding	AK026379
protein_coding	AEBP1
protein_coding	CCL22
protein_coding	CAND2
protein_coding	KIAA0556
protein_coding	ST3GAL2
protein_coding	GTF2H4
protein_coding	C17orf107
protein_coding	KLHL23
protein_coding	BTN2A2
protein_coding	GSPT1
protein_coding	RPS6KB2

protein_coding	OPTC
protein_coding	ECM2
protein_coding	C19orf10
protein_coding	WBSCR17
protein_coding	SEMA4A
protein_coding	TTBK2
protein_coding	SMIM4
protein_coding	KMT2D
protein_coding	CCDC14
protein_coding	CA13
protein_coding	CDK2AP2
protein_coding	FPGT
protein_coding	MAPRE3
protein_coding	GATB
protein_coding	HIF3A
protein_coding	CPSF4
protein_coding	PLEKHH2
protein_coding	ZNF395
protein_coding	YIPF5
protein_coding	EFCAB6
protein_coding	GFPT1
protein_coding	LCN2
protein_coding	IFT122
protein_coding	DDX21
protein_coding	RUSC1
protein_coding	GJA4
protein_coding	PARVB
protein_coding	GPT2
protein_coding	BCLAF1
protein_coding	HRASLS5
protein_coding	RHOQ
protein_coding	SUPT20H
protein_coding	C3orf36
protein_coding	SHC1
protein_coding	PARP4
protein_coding	RAPGEF3
protein_coding	TRAFD1
protein_coding	BMP8A
protein_coding	TBC1D8
protein_coding	CLEC1B
protein_coding	SLC7A6
protein_coding	MASTL
protein_coding	SEC23A
protein_coding	GNA15
protein_coding	HMGN2
protein_coding	RRP1
protein_coding	CCDC86

protein_coding	PLIN5
protein_coding	AC104534.3
protein_coding	BET1
protein_coding	CLN3
protein_coding	DBNDD2
protein_coding	IMPDH2
protein_coding	RPL23
protein_coding	WDR48
protein_coding	CEMP1
protein_coding	HMGB1
protein_coding	AAED1
protein_coding	MEF2A
protein_coding	NBPF14
protein_coding	SHBG
protein_coding	NFKBIB
protein_coding	SEZ6
protein_coding	NFIC
protein_coding	C8orf58
protein_coding	CCDC148
protein_coding	GATSL2
protein_coding	PCID2
protein_coding	SLC30A5
protein_coding	TCP10L2
protein_coding	IQCJ-SCHIP1
protein_coding	CHIC2
protein_coding	TEF
protein_coding	SYNE4
protein_coding	C1QTNF1
protein_coding	AK054970
protein_coding	C5orf28
protein_coding	AL121901.1
protein_coding	WBSCR22
protein_coding	MAGEA2
protein_coding	LRRC66
protein_coding	LINGO3
protein_coding	MPPED1
protein_coding	FTSJ1
protein_coding	CHST3
protein_coding	CRIPAK
protein_coding	YPEL3
protein_coding	FAM219B
protein_coding	MTHFR
protein_coding	ERICH5
protein_coding	LIG3
protein_coding	FAM47E-STBD1
protein_coding	IMP3
protein_coding	OTUB1

protein_coding	BBS12
protein_coding	PPP1R35
protein_coding	METTL8
protein_coding	LPHN2
protein_coding	ZNF584
protein_coding	PFKL
protein_coding	IFT81
protein_coding	SBF2
protein_coding	NTPCR
protein_coding	PHLDB3
protein_coding	AL355531.2
protein_coding	LEPRE1
protein_coding	ACTRT3
protein_coding	SRF
protein_coding	BZW1
protein_coding	TOPBP1
protein_coding	B4GALT3
protein_coding	ZNF232
protein_coding	PDLIM2
protein_coding	RNF32
protein_coding	LDLRAP1
protein_coding	PUS3
protein_coding	CCDC177
protein_coding	MRGBP
protein_coding	GLRA1
protein_coding	AP3S1
protein_coding	KCNQ2
protein_coding	KRTAP5-10
protein_coding	PHEX
protein_coding	ZNF672
protein_coding	UXT
protein_coding	TBX3
protein_coding	EML1
protein_coding	DHFR
protein_coding	SHROOM3
protein_coding	SP2
protein_coding	GJB1
protein_coding	STK38
protein_coding	VAMP3
protein_coding	TMEM242
protein_coding	ARHGAP10
protein_coding	RELB
protein_coding	ATXN7L3
protein_coding	RHAG
protein_coding	UBAP2L
protein_coding	FASTK
protein_coding	ANKS1A

protein_coding	C8orf4
protein_coding	JAKMIP3
protein_coding	SUV420H1
protein_coding	S100A11
protein_coding	NPY5R
protein_coding	SRSF6
protein_coding	PSTK
protein_coding	KRT83
protein_coding	CES2
protein_coding	TNPO1
protein_coding	LAMTOR2
protein_coding	MRRF
protein_coding	ZNF624
protein_coding	OFD1
protein_coding	SPIDR
protein_coding	BOD1
protein_coding	PKDCC
protein_coding	SLC9C2
protein_coding	KCNQ1
protein_coding	MNT
protein_coding	LUZP2
protein_coding	NRIP2
protein_coding	EIF4B
protein_coding	TYROBP
protein_coding	AP005482.1
protein_coding	DCP2
protein_coding	SLC25A10
protein_coding	EIF3M
protein_coding	SIVA1
protein_coding	JCHAIN
protein_coding	KCTD20
protein_coding	MRPL36
protein_coding	FAM175B
protein_coding	SMAP1
protein_coding	SLC4A2
protein_coding	BMP2K
protein_coding	CAMK2N2
protein_coding	COQ10A
protein_coding	RSBN1
protein_coding	EFNB3
protein_coding	MRPS27
protein_coding	VGLL2
protein_coding	PLXNB3
protein_coding	KRT32
protein_coding	RMI1
protein_coding	CDKN1B
protein_coding	DIP2B

protein_coding	FAM73B
protein_coding	SRPR
protein_coding	NSMAF
protein_coding	LRRC42
protein_coding	PKD1L3
protein_coding	WBP1L
protein_coding	CPXCR1
protein_coding	NEB
protein_coding	FOXD4L6
protein_coding	HTR5A
protein_coding	CCL2
protein_coding	ZNF461
protein_coding	TP53I13
protein_coding	NOP14
protein_coding	PDIA6
protein_coding	BC114339
protein_coding	MEGF8
protein_coding	LRRC37A3
protein_coding	NLRP2
protein_coding	UBE2W
protein_coding	PCDH19
protein_coding	ZNF285
protein_coding	FOXN2
protein_coding	ITPKB
protein_coding	FYTTD1
protein_coding	PAPD5
protein_coding	GSPT2
protein_coding	LYPD5
protein_coding	C17orf67
protein_coding	RGS16
protein_coding	ITGA10
protein_coding	GNPDA2
protein_coding	ZMIZ1
protein_coding	FSTL3
protein_coding	ACYP1
protein_coding	VSIG10
protein_coding	MAFA
protein_coding	SNRPE
protein_coding	WDR70
protein_coding	SSH1
protein_coding	UCMA
protein_coding	TNS4
protein_coding	TRIM54
protein_coding	RBM48
protein_coding	IRAK4
protein_coding	OSBP
protein_coding	MFSD2B

protein_coding	RPL36AL
protein_coding	MB
protein_coding	PHF23
protein_coding	ZNF512
protein_coding	ACSF2
protein_coding	RBMX
protein_coding	FAM200B
protein_coding	FBXO22
protein_coding	USP45
protein_coding	ZNF304
protein_coding	CWC27
protein_coding	PDIK1L
protein_coding	ARL4D
protein_coding	CNNM2
protein_coding	CEP295
protein_coding	RUNX3
protein_coding	CUL5
protein_coding	ST6GAL2
protein_coding	FAM122C
protein_coding	ARL6IP5
protein_coding	MB21D1
protein_coding	LHX1
protein_coding	STX1B
protein_coding	TRIM22
protein_coding	DRG2
protein_coding	FOXA1
protein_coding	TTLL1
protein_coding	UAP1
protein_coding	ZNF587
protein_coding	DHX35
protein_coding	LOC100288142
protein_coding	CRISP3
protein_coding	CDH7
protein_coding	VPS4B
protein_coding	GLIS2
protein_coding	CNIH3
protein_coding	C5orf34
protein_coding	AK124465
protein_coding	FOSL2
protein_coding	COG6
protein_coding	CHIA
protein_coding	MYO1D
protein_coding	CA8
protein_coding	ZNF236
protein_coding	ZC3H12C
protein_coding	OR56A1
protein_coding	RFX2

protein_coding	POTEC
protein_coding	FAXDC2
protein_coding	KRTAP9-1
protein_coding	KLF6
protein_coding	DIRC2
protein_coding	AC055736.1
protein_coding	DDX41
protein_coding	SMIM15
protein_coding	ATF3
protein_coding	AGO1
protein_coding	MED12L
protein_coding	BGN
protein_coding	TRIM49D1
protein_coding	SMIM6
protein_coding	FAM221B
protein_coding	TSEN54
protein_coding	PRAP1
protein_coding	VOPP1
protein_coding	BTG2
protein_coding	RALGAPA2
protein_coding	RGPD3
protein_coding	LRCH2
protein_coding	FAM133A
protein_coding	MFSD5
protein_coding	KCNH4
protein_coding	UNC5D
protein_coding	SEC24D
protein_coding	R3HDM2
protein_coding	CNEP1R1
protein_coding	NKAP
protein_coding	FNDC3B
protein_coding	ANKIB1
protein_coding	DNHD1
protein_coding	PDCD11
protein_coding	AMDHD2
protein_coding	GOLGA4
protein_coding	ZNF426
protein_coding	TMEM184B
protein_coding	AP3S2
protein_coding	PIK3C2B
protein_coding	LTB
protein_coding	SUSD4
protein_coding	GPRIN1
protein_coding	SLC12A6
protein_coding	ANAPC15
protein_coding	AC011897.1
protein_coding	ACOT9

protein_coding	MMP28
protein_coding	SNRNP70
protein_coding	SPATA1
protein_coding	JAGN1
protein_coding	HPGDS
protein_coding	C11orf52
protein_coding	DSN1
protein_coding	AGFG1
protein_coding	TMEM82
protein_coding	SNTB1
protein_coding	HMBOX1
protein_coding	PRED60
protein_coding	ADRA2B
protein_coding	DARS2
protein_coding	BCL2L13
protein_coding	PDK1
protein_coding	PCDH1
protein_coding	COBLL1
protein_coding	NIT1
protein_coding	CAV2
protein_coding	DVL2
protein_coding	C15orf37
protein_coding	CALML3
protein_coding	RASGRP2
protein_coding	TSC22D3
protein_coding	AC012215.1
protein_coding	CCDC153
protein_coding	GLUD2
protein_coding	LYVE1
protein_coding	TEDDM1
protein_coding	OCM
protein_coding	AP2A1
protein_coding	RND2
protein_coding	RP11-111M22.2
protein_coding	PLCXD1
protein_coding	KLHL29
protein_coding	DCAF12L2
protein_coding	TMEM235
protein_coding	ASB13
protein_coding	PAOX
protein_coding	SDHAF3
protein_coding	ATP6AP1L
protein_coding	RPL7A
protein_coding	MSRA
protein_coding	BCMO1
protein_coding	RPL17
protein_coding	RNF126

protein_coding	PNMT
protein_coding	PPDPF
protein_coding	RTN4R
protein_coding	BTBD17
protein_coding	CTB-134H23.2
protein_coding	PITX3
protein_coding	SMPDL3A
protein_coding	POT1
protein_coding	TMEM98
protein_coding	CASP3
protein_coding	RAD1
protein_coding	COQ9
protein_coding	PGA5
protein_coding	TMEM208
protein_coding	SLC25A51
protein_coding	SLCO2A1
protein_coding	TLR3
protein_coding	CTSS
protein_coding	WDR78
protein_coding	ALG1L
protein_coding	MSL2
protein_coding	HEATR4
protein_coding	UBAP2
protein_coding	CEBPZ-AS1
protein_coding	DENND1A
protein_coding	FLNA
protein_coding	KDM6A
protein_coding	RNF38
protein_coding	SPECC1L
protein_coding	SRSF7
protein_coding	COL26A1
protein_coding	VPS13C
protein_coding	CNOT1
protein_coding	KAT2A
protein_coding	SFR1
protein_coding	PHIP
protein_coding	APRT
protein_coding	FCGR3B
protein_coding	MUSTN1
protein_coding	MAS1
protein_coding	VEGFA
protein_coding	CHD5
protein_coding	WDR60
protein_coding	SYNDIG1L
protein_coding	CMTR1
protein_coding	COX14
protein_coding	NEDD4

protein_coding	ATM
protein_coding	ZNF692
protein_coding	MRPS16
protein_coding	METTL13
protein_coding	AC018630.1
protein_coding	SSTR3
protein_coding	SYNGR2
protein_coding	DEDD
protein_coding	SPICE1
protein_coding	GGACT
protein_coding	NPW
protein_coding	SYF2
protein_coding	TTC5
protein_coding	GDAP2
protein_coding	ZNF706
protein_coding	NFATC2
protein_coding	FAM89B
protein_coding	CKAP5
protein_coding	UBE2A
protein_coding	PLD1
protein_coding	MTRR
protein_coding	C12orf73
protein_coding	C20orf201
protein_coding	COL9A2
protein_coding	PRPF31
protein_coding	BC101234
protein_coding	ZMIZ2
protein_coding	COIL
protein_coding	SEC23B
protein_coding	ANKRD35
protein_coding	OCEL1
protein_coding	SLC2A8
protein_coding	FAM134A
protein_coding	GAR1
protein_coding	RQCD1
protein_coding	COG5
protein_coding	TPM1
protein_coding	BCO1
protein_coding	PCYT1B
protein_coding	AMFR
protein_coding	CNNM4
protein_coding	DCAF11
protein_coding	ITSN1
protein_coding	SMEK2
protein_coding	ZMYND12
protein_coding	CDC73
protein_coding	KCNE1

protein_coding	ACP2
protein_coding	THSD7A
protein_coding	ZNF112
protein_coding	STT3A
protein_coding	PACRG
protein_coding	DLX3
protein_coding	AKAP5
protein_coding	COQ6
protein_coding	TRPV3
protein_coding	TTPAL
protein_coding	IFITM1
protein_coding	C1orf174
protein_coding	SETD9
protein_coding	PODXL2
protein_coding	AC102948.2
protein_coding	RSL1D1
protein_coding	PPP6C
protein_coding	SNN
protein_coding	RP11-113D6.6
protein_coding	TGFB2
protein_coding	PRSS16
protein_coding	CBX3
protein_coding	MOGS
protein_coding	TFB2M
protein_coding	RABL2A
protein_coding	AC006455.1
protein_coding	HSPBAP1
protein_coding	ANTXRL
protein_coding	MKX
protein_coding	L3MBTL1
protein_coding	LOXHD1
protein_coding	BRINP3
protein_coding	CCDC34
protein_coding	MIB2
protein_coding	PSMG4
protein_coding	PHGDH
protein_coding	USPL1
protein_coding	RRP15
protein_coding	CD38
protein_coding	RP11-10A14.4
protein_coding	MCM4
protein_coding	DENR
protein_coding	DOCK5
protein_coding	NCK1
protein_coding	ETNK1
protein_coding	TRPC1
protein_coding	NLGN4X

protein_coding	PKP2
protein_coding	CES5A
protein_coding	TRAPPC4
protein_coding	PAPD4
protein_coding	CKAP4
protein_coding	HDHD3
protein_coding	NIFK
protein_coding	RALGAPB
protein_coding	CASQ1
protein_coding	ZNF80
protein_coding	EXOC3
protein_coding	C21orf2
protein_coding	FAM110D
protein_coding	C3orf33
protein_coding	C19orf12
protein_coding	TUBGCP2
protein_coding	ACAT2
protein_coding	TRIAP1
protein_coding	KIF1C
protein_coding	AL049840.1
protein_coding	ARFIP1
protein_coding	LRRC6
protein_coding	DCTN6
protein_coding	PITPNC1
protein_coding	FGD5
protein_coding	TRIM52
protein_coding	MTERF1
protein_coding	WSCD2
protein_coding	PTCD2
protein_coding	KY
protein_coding	COMMD2
protein_coding	QARS
protein_coding	LSM1
protein_coding	UBN2
protein_coding	SSBP2
protein_coding	FASTKD2
protein_coding	PIK3R6
protein_coding	PON3
protein_coding	SFT2D2
protein_coding	IL27RA
protein_coding	C14orf28
protein_coding	DMGDH
protein_coding	POLE3
protein_coding	SSRP1
protein_coding	N4BP2L2
protein_coding	MAGED2
protein_coding	SLC25A24

protein_coding	RP11-762I7.5
protein_coding	FAM163B
protein_coding	EPC2
protein_coding	SLC2A4RG
protein_coding	IER3
protein_coding	MTBP
protein_coding	SEC14L6
protein_coding	PIGF
protein_coding	FUT10
protein_coding	SLC26A8
protein_coding	LMAN2
protein_coding	LRP10
protein_coding	ASPHD2
protein_coding	BST2
protein_coding	UQCC3
protein_coding	ZNF212
protein_coding	TPTE2
protein_coding	NEU1
protein_coding	TNFRSF14
protein_coding	AGRN
protein_coding	NLRP6
protein_coding	ADAT1
protein_coding	CDSN
protein_coding	FNIP1
protein_coding	COX15
protein_coding	MCM8
protein_coding	PHPT1
protein_coding	TEX28
protein_coding	SS18
protein_coding	YIPF4
protein_coding	H2AFJ
protein_coding	TCRBV22S1A2N1T
protein_coding	SMNDC1
protein_coding	LOC440243
protein_coding	PCDH11Y
protein_coding	SMC6
protein_coding	CTB-60B18.6
protein_coding	STK26
protein_coding	MCU
protein_coding	C11orf98
protein_coding	P4HA2
protein_coding	CFAP54
protein_coding	KIF17
protein_coding	RP11-1055B8.7
protein_coding	SYT16
protein_coding	TRAPPC5
protein_coding	TROAP

protein_coding	RWDD3
protein_coding	GIT1
protein_coding	PET117
protein_coding	UGGT1
protein_coding	VPS37A
protein_coding	ARHGAP31
protein_coding	RAB33A
protein_coding	FAM24B
protein_coding	ZNF433
protein_coding	PCDHGB7
protein_coding	RP11-867G23.8
protein_coding	LSM7
protein_coding	SPATA5L1
protein_coding	EML6
protein_coding	CLN6
protein_coding	RASSF3
protein_coding	PES1
protein_coding	SPRR2E
protein_coding	ASTL
protein_coding	HIST1H2BK
protein_coding	COL9A3
protein_coding	PYHIN1
protein_coding	NRAS
protein_coding	ERICH3
protein_coding	GSTM5
protein_coding	ETAA1
protein_coding	ABCG5
protein_coding	ANXA3
protein_coding	SESTD1
protein_coding	SEC14L5
protein_coding	NOA1
protein_coding	NDUFV3
protein_coding	KLK15
protein_coding	UGDH
protein_coding	ABCA1
protein_coding	ADCY6
protein_coding	DENND1C
protein_coding	X97876
protein_coding	BTN2A1
protein_coding	DCLK2
protein_coding	ELOVL5
protein_coding	DUSP3
protein_coding	LY6H
protein_coding	DHRS4-AS1
protein_coding	ZNF562
protein_coding	SFXN5
protein_coding	C9orf89

protein_coding	PDE4C
protein_coding	APBB1IP
protein_coding	PGLYRP2
protein_coding	ISY1
protein_coding	ANKRD16
protein_coding	KDM7A
protein_coding	DCP1B
protein_coding	RAD23B
protein_coding	ZMAT3
protein_coding	SLC12A7
protein_coding	PDAP1
protein_coding	C11orf45
protein_coding	TYMS
protein_coding	NEUROD6
protein_coding	GPR89C
protein_coding	DOHH
protein_coding	SRRT
protein_coding	CCDC166
protein_coding	MPP5
protein_coding	CENPN
protein_coding	SLC27A2
protein_coding	RP11-383H13.1
protein_coding	C10orf10
protein_coding	TMEM260
protein_coding	TMEM104
protein_coding	ABCB6
protein_coding	MED20
protein_coding	SCYL1
protein_coding	PCDHB4
protein_coding	MMP9
protein_coding	CHAT
protein_coding	TMEM27
protein_coding	CEP70
protein_coding	KLRC3
protein_coding	GLB1L3
protein_coding	EPN2
protein_coding	RUNDC1
protein_coding	RBBP8
protein_coding	C1GALT1C1L
protein_coding	LSS
protein_coding	RFPL4B
protein_coding	RPIA
protein_coding	WDR36
protein_coding	IPO5
protein_coding	GADD45A
protein_coding	C1orf61
protein_coding	NACA2

protein_coding	YKT6
protein_coding	C17orf51
protein_coding	RNF168
protein_coding	MOB3A
protein_coding	CYB5R4
protein_coding	COASY
protein_coding	STRN3
protein_coding	OTUD7A
protein_coding	MAN2B2
protein_coding	NEDD8-MDP1
protein_coding	GADD45B
protein_coding	CLCC1
protein_coding	GPATCH1
protein_coding	UBL4A
protein_coding	TBC1D20
protein_coding	ZXDB
protein_coding	MICB
protein_coding	C11orf1
protein_coding	SLC38A2
protein_coding	IREB2
protein_coding	XRCC4
protein_coding	WDSUB1
protein_coding	LYPLA2
protein_coding	GNG2
protein_coding	RAVER1
protein_coding	TLR6
protein_coding	GALNS
protein_coding	PCP2
protein_coding	ATOX1
protein_coding	COG2
protein_coding	C19orf73
protein_coding	FAM101B
protein_coding	AC004899.1
protein_coding	MKRN1
protein_coding	NANS
protein_coding	ZC3H11A
protein_coding	TEX264
protein_coding	SOX5
protein_coding	PTHLH
protein_coding	WDR45B
protein_coding	TCF25
protein_coding	ADAL
protein_coding	PSMA2
protein_coding	ADGRG7
protein_coding	GPX6
protein_coding	MIOX
protein_coding	TRNAU1AP

protein_coding	JMJD6
protein_coding	AK310228
protein_coding	HES7
protein_coding	SUPT3H
protein_coding	SLC9A6
protein_coding	FSCN1
protein_coding	AC009403.2
protein_coding	LIMA1
protein_coding	EXOSC3
protein_coding	ING2
protein_coding	REST
protein_coding	ANKZF1
protein_coding	PHC2
protein_coding	TRAPPC10
protein_coding	RPL22
protein_coding	BCHE
protein_coding	MYBPHL
protein_coding	SFRP1
protein_coding	OCLN
protein_coding	ATP13A3
protein_coding	FOXQ1
protein_coding	LOC100130705
protein_coding	EDNRA
protein_coding	PSMA3
protein_coding	TYW1B
protein_coding	C2orf50
protein_coding	HGFAC
protein_coding	NPRL2
protein_coding	TOR3A
protein_coding	SS18L1
protein_coding	GLDC
protein_coding	GOLGA6D
protein_coding	PNPT1
protein_coding	VCY
protein_coding	DCTPP1
protein_coding	MRPL39
protein_coding	SLC4A3
protein_coding	AL627309.1
protein_coding	C1orf204
protein_coding	VGF
protein_coding	ZNF623
protein_coding	PDE1B
protein_coding	LCA5
protein_coding	NSRP1
protein_coding	THRA1
protein_coding	FAM127C
protein_coding	NSG2

protein_coding	TAF5
protein_coding	VWDE
protein_coding	CCDC176
protein_coding	TUBA3D
protein_coding	ATP6V1G1
protein_coding	ZNF318
protein_coding	FOXG1
protein_coding	MB21D2
protein_coding	SEMA6A
protein_coding	BET1L
protein_coding	ZNF385B
protein_coding	NEDD1
protein_coding	ABCC6
protein_coding	STAU1
protein_coding	ATP1A3
protein_coding	NHLRC3
protein_coding	CMIP
protein_coding	GLP2R
protein_coding	SYAP1
protein_coding	FREM2
protein_coding	GSG2
protein_coding	ITGAE
protein_coding	SMUG1
protein_coding	CEP95
protein_coding	OBP2B
protein_coding	CEP41
protein_coding	KCNJ11
protein_coding	MTF2
protein_coding	ABI2
protein_coding	C11orf84
protein_coding	GYS1
protein_coding	DCBLD2
protein_coding	HMGB3
protein_coding	GNA11
protein_coding	GSTK1
protein_coding	TJAP1
protein_coding	HAMP
protein_coding	WDFY1
protein_coding	CREB3L1
protein_coding	HSPG2
protein_coding	SLC39A9
protein_coding	FTO
protein_coding	CDK5R1
protein_coding	NR2E1
protein_coding	TXNDC16
protein_coding	METTL25
protein_coding	STEAP1

protein_coding	RP11-422N16.3
protein_coding	CADM3
protein_coding	KCNU1
protein_coding	AX747119
protein_coding	CD97
protein_coding	CACNA1G
protein_coding	FAM98C
protein_coding	ZSCAN31
protein_coding	UMAD1
protein_coding	SLC25A44
protein_coding	KM-PA-2
protein_coding	GLA
protein_coding	FAH
protein_coding	mir-34
protein_coding	POLR2A
protein_coding	ABHD5
protein_coding	CIART
protein_coding	HYAL3
protein_coding	EVC
protein_coding	TPD52L2
protein_coding	MAPK8IP1
protein_coding	NLRP7
protein_coding	MIS18BP1
protein_coding	IMPACT
protein_coding	HYKK
protein_coding	CENPT
protein_coding	TBC1D17
protein_coding	RBMS3
protein_coding	OLFM2
protein_coding	TPD52L3
protein_coding	CXCL3
protein_coding	BFAR
protein_coding	TBX10
protein_coding	FUT2
protein_coding	HADHA
protein_coding	PCSK4
protein_coding	PROSC
protein_coding	IQSEC3
protein_coding	CTDNEP1
protein_coding	NPHP1
protein_coding	CHID1
protein_coding	RBPJ
protein_coding	TMEM191C
protein_coding	ACTL7B
protein_coding	MAPK6
protein_coding	FLAD1
protein_coding	DERA

protein_coding	SLC38A8
protein_coding	ETV7
protein_coding	NOV
protein_coding	ANKRD7
protein_coding	CABP2
protein_coding	ZCCHC2
protein_coding	PARD3
protein_coding	ADGB
protein_coding	CWC25
protein_coding	GPD1L
protein_coding	DTNBP1
protein_coding	HIATL2
protein_coding	TMTC3
protein_coding	ZNF83
protein_coding	BRD4
protein_coding	TSTD3
protein_coding	CDC42EP4
protein_coding	ZNF267
protein_coding	ZIC1
protein_coding	OR8G5
protein_coding	ASCL5
protein_coding	ABCG4
protein_coding	NINJ1
protein_coding	RPL5
protein_coding	EIF4E2
protein_coding	HOMER2
protein_coding	MTMR1
protein_coding	SAMD4B
protein_coding	UTP14A
protein_coding	SNPH
protein_coding	CDO1
protein_coding	QRFPR
protein_coding	CFAP99
protein_coding	SHISA6
protein_coding	R3HDM4
protein_coding	TET3
protein_coding	BPIFB2
protein_coding	UNC79
protein_coding	PTPLAD1
protein_coding	INS-IGF2
protein_coding	FATE1
protein_coding	FAM91A1
protein_coding	ICAM3
protein_coding	AL513478.1
protein_coding	MEMO1
protein_coding	TCHH
protein_coding	RCBTB1

protein_coding	XPC
protein_coding	RNF213
protein_coding	SH3BGR
protein_coding	CCDC151
protein_coding	FBXO15
protein_coding	UPF3B
protein_coding	ATXN7
protein_coding	NTF4
protein_coding	CXorf28
protein_coding	C14orf79
protein_coding	DHDH
protein_coding	MUCL1
protein_coding	PIP5KL1
protein_coding	VHL
protein_coding	PRAF2
protein_coding	ZNF26
protein_coding	RPL18
protein_coding	F12
protein_coding	GTF2E2
protein_coding	ACAN
protein_coding	RBX1
protein_coding	NTN4
protein_coding	CTDP1
protein_coding	AARSD1
protein_coding	C19orf38
protein_coding	KRTAP6-2
protein_coding	GCC1
protein_coding	PREB
protein_coding	IQCE
protein_coding	SRRM4
protein_coding	LOC100129697
protein_coding	FKBP2
protein_coding	TMEM192
protein_coding	SYT13
protein_coding	C1orf159
protein_coding	FIBCD1
protein_coding	APOC2
protein_coding	GPC5
protein_coding	AK123450
protein_coding	DHX8
protein_coding	KPNA3
protein_coding	TMEM116
protein_coding	ZDHHC4
protein_coding	AX747534
protein_coding	GCKR
protein_coding	NIM1K
protein_coding	TK2

protein_coding	NASP
protein_coding	NPM3
protein_coding	MGMT
protein_coding	ABCC1
protein_coding	RUVBL1
protein_coding	CCDC33
protein_coding	LARP4
protein_coding	STARD3NL
protein_coding	GNB2
protein_coding	TUSC3
protein_coding	ZDHHC19
protein_coding	RRP36
protein_coding	RAB32
protein_coding	ZNF195
protein_coding	LRRC48
protein_coding	EZH1
protein_coding	ARHGEF10
protein_coding	PGPEP1
protein_coding	AX748058
protein_coding	TBC1D19
protein_coding	NLRC4
protein_coding	AKNA
protein_coding	DIAPH2
protein_coding	XPNPEP1
protein_coding	MIER1
protein_coding	ARL4A
protein_coding	SIX6
protein_coding	IVD
protein_coding	IGSF8
protein_coding	C19orf68
protein_coding	MED9
protein_coding	GATSL1
protein_coding	ABCD4
protein_coding	ATP12A
protein_coding	SIGLEC11
protein_coding	NR3C2
protein_coding	MT1A
protein_coding	PQBP1
protein_coding	ETF1
protein_coding	RPL11
protein_coding	RCSD1
protein_coding	HAP1
protein_coding	ERCC2
protein_coding	EGR3
protein_coding	MKNK1
protein_coding	DSCR3
protein_coding	GLIS3

protein_coding	EEF1B2
protein_coding	TIMP3
protein_coding	DHX40
protein_coding	C15orf27
protein_coding	BCAS3
protein_coding	SAMD13
protein_coding	CCNL1
protein_coding	HYI
protein_coding	FXN
protein_coding	NT5C2
protein_coding	ENTPD8
protein_coding	HDAC11
protein_coding	ISY1-RAB43
protein_coding	FCGRT
protein_coding	FAM118B
protein_coding	AC005549.3
protein_coding	C16orf91
protein_coding	DNAJC17
protein_coding	ZSWIM6
protein_coding	MPP2
protein_coding	TMEM179B
protein_coding	AX747367
protein_coding	TVP23A
protein_coding	MICAL3
protein_coding	SLC39A4
protein_coding	SUSD5
protein_coding	ATP10D
protein_coding	TRIM38
protein_coding	ITGA8
protein_coding	CCDC8
protein_coding	AAMDC
protein_coding	EPN1
protein_coding	AK7
protein_coding	BNIP2
protein_coding	TOR2A
protein_coding	SLC26A4
protein_coding	C1orf35
protein_coding	TAS2R40
protein_coding	CWF19L2
protein_coding	CISD3
protein_coding	ISCA2
protein_coding	WIBG
protein_coding	BIRC7
protein_coding	CHAF1A
protein_coding	OTUD4
protein_coding	ATAD3B
protein_coding	REV1

protein_coding	DHX9
protein_coding	SPRY4
protein_coding	ICK
protein_coding	PGA3
protein_coding	DDX23
protein_coding	TRIM39
protein_coding	PNPLA7
protein_coding	SERINC5
protein_coding	PI4K2B
protein_coding	GSTM1
protein_coding	ZNF600
protein_coding	UBE4B
protein_coding	ZNF235
protein_coding	LRFN4
protein_coding	SOX15
protein_coding	RPS6KA5
protein_coding	CARF
protein_coding	GHRL
protein_coding	EARS2
protein_coding	NUP88
protein_coding	MDN1
protein_coding	MANBA
protein_coding	GNG5P2
protein_coding	MSANTD1
protein_coding	PPP1R15B
protein_coding	XRCC5
protein_coding	KNOP1
protein_coding	GINM1
protein_coding	AMY2A
protein_coding	ARHGAP9
protein_coding	FRMD1
protein_coding	IFT27
protein_coding	NSD1
protein_coding	POLB
protein_coding	VTI1A
protein_coding	RNF121
protein_coding	EMC10
protein_coding	PANK1
protein_coding	KIF13B
protein_coding	ZNF354B
protein_coding	ADCY4
protein_coding	BC101079
protein_coding	SYCE1
protein_coding	RYK
protein_coding	MDC1
protein_coding	ANKRD2
protein_coding	NUP98

protein_coding	GSG1L2
protein_coding	GNA12
protein_coding	CFAP70
protein_coding	LAMA2
protein_coding	PPIA
protein_coding	FILIP1L
protein_coding	TAS2R60
protein_coding	MSL1
protein_coding	ZNF343
protein_coding	SRSF10
protein_coding	NOTCH1
protein_coding	IFT52
protein_coding	UBE2G1
protein_coding	RAB17
protein_coding	TMEM88B
protein_coding	FOXN3
protein_coding	WDR12
protein_coding	TNKS1BP1
protein_coding	CASKIN1
protein_coding	C12orf4
protein_coding	SLC8A3
protein_coding	CFLAR
protein_coding	DVL3
protein_coding	DDX39A
protein_coding	DNAJC4
protein_coding	SEC16A
protein_coding	PELO
protein_coding	MOB3B
protein_coding	IER5L
protein_coding	FXR1
protein_coding	TRRAP
protein_coding	RGS6
protein_coding	BAZ2B
protein_coding	DGAT2
protein_coding	ERMP1
protein_coding	RSPRY1
protein_coding	NOC4L
protein_coding	TAMM41
protein_coding	AFF3
protein_coding	MRPS18A
protein_coding	STARD4
protein_coding	MTFR1L
protein_coding	TRPM4
protein_coding	SYCE3
protein_coding	SULT6B1
protein_coding	RPS27L
protein_coding	ROBO4

protein_coding	USP38
protein_coding	WDR5B
protein_coding	ANP32A
protein_coding	FCAMR
protein_coding	AK098727
protein_coding	IFI27L1
protein_coding	NDST3
protein_coding	ETV3
protein_coding	SZRD1
protein_coding	MED14
protein_coding	MAP3K6
protein_coding	IL17RB
protein_coding	HYLS1
protein_coding	SLC25A48
protein_coding	CCDC124
protein_coding	MAX
protein_coding	MOSPD3
protein_coding	SSNA1
protein_coding	LRRN3
protein_coding	GABARAP
protein_coding	RPA3
protein_coding	AP5M1
protein_coding	DNAJC25-GNG10
protein_coding	RNF223
protein_coding	SDHAF2
protein_coding	ABCE1
protein_coding	YAE1D1
protein_coding	OBSL1
protein_coding	ZMYM4
protein_coding	TXNDC12
protein_coding	ZNF596
protein_coding	CCDC104
protein_coding	MCEE
protein_coding	MYO3A
protein_coding	TRAM1
protein_coding	LIPC
protein_coding	IL6
protein_coding	SERP1
protein_coding	THBS3
protein_coding	FRMD5
protein_coding	ATG16L1
protein_coding	SLC39A14
protein_coding	EYA4
protein_coding	DNER
protein_coding	LILRA4
protein_coding	TMEM231
protein_coding	C9orf72

protein_coding	FAM120B
protein_coding	AQP11
protein_coding	HNF1A
protein_coding	TIE1
protein_coding	TMEM132E
protein_coding	NABP1
protein_coding	CCNYL1
protein_coding	MLLT1
protein_coding	MAP3K10
protein_coding	MED29
protein_coding	SOCS3
protein_coding	PABPC4
protein_coding	HSFX2
protein_coding	SLC3A2
protein_coding	RHOU
protein_coding	RHNO1
protein_coding	DOCK8
protein_coding	IL1RAP
protein_coding	BBIP1
protein_coding	EIF3E
protein_coding	NR2C2
protein_coding	GUCY2D
protein_coding	AC073342.1
protein_coding	CDH6
protein_coding	CBX1
protein_coding	FAM26E
protein_coding	SMIM22
protein_coding	CLSTN2
protein_coding	ARMC4
protein_coding	C12orf43
protein_coding	PNPLA4
protein_coding	PTH2
protein_coding	DSEL
protein_coding	FAM53B
protein_coding	PRL
protein_coding	DHX33
protein_coding	GTF2I
protein_coding	LRRC23
protein_coding	C21orf91
protein_coding	IGFALS
protein_coding	MSX2
protein_coding	TCAIM
protein_coding	TLL1
protein_coding	AX746590
protein_coding	MFHAS1
protein_coding	MESP2
protein_coding	XKR9

protein_coding	PLEKHA2
protein_coding	ZNF804A
protein_coding	NUP153
protein_coding	SLC12A8
protein_coding	PPIB
protein_coding	PIEZO1
protein_coding	GGH
protein_coding	ZNF611
protein_coding	PPM1M
protein_coding	PLXDC2
protein_coding	RBM18
protein_coding	LRRTM1
protein_coding	DHX32
protein_coding	NR4A2
protein_coding	SRM
protein_coding	BCAT2
protein_coding	PLN
protein_coding	NUDT7
protein_coding	OPRK1
protein_coding	DDOST
protein_coding	MRPS5
protein_coding	FGFRL1
protein_coding	APOL3
protein_coding	AK097143
protein_coding	ADCK4
protein_coding	POLI
protein_coding	PTPRF
protein_coding	VASN
protein_coding	KCNA3
protein_coding	CYP11A1
protein_coding	PET100
protein_coding	TMEM257
protein_coding	MMADHC
protein_coding	KRT36
protein_coding	KDM1A
protein_coding	COX7A2L
protein_coding	AL626787.1
protein_coding	PRDM2
protein_coding	NR4A3
protein_coding	CINP
protein_coding	GPR176
protein_coding	EBLN2
protein_coding	RIC8B
protein_coding	GULP1
protein_coding	MDFIC
protein_coding	HDGFRP2
protein_coding	SH2D2A

protein_coding	MAP1LC3B
protein_coding	ADH6
protein_coding	SETD6
protein_coding	LCN15
protein_coding	HTT
protein_coding	LONRF3
protein_coding	ZNF175
protein_coding	GPR21
protein_coding	SPANXN4
protein_coding	KERA
protein_coding	MCUR1
protein_coding	CDH19
protein_coding	C6orf165
protein_coding	ARHGAP1
protein_coding	AGAP1
protein_coding	STRIP2
protein_coding	DNAJC13
protein_coding	SDCCAG3
protein_coding	TDRD3
protein_coding	CCDC22
protein_coding	MLF1
protein_coding	ZNF570
protein_coding	EPSTI1
protein_coding	MYH9
protein_coding	IPPK
protein_coding	RTN4RL2
protein_coding	PRRG3
protein_coding	POTEF
protein_coding	FAM86B1
protein_coding	GIMAP8
protein_coding	MYO18A
protein_coding	HSPE1-MOB4
protein_coding	RHOBTB1
protein_coding	SMAD1
protein_coding	KIAA0020
protein_coding	RPS3
protein_coding	AMIGO1
protein_coding	TRIM62
protein_coding	CYB5A
protein_coding	DYRK3
protein_coding	TMEM110
protein_coding	GPAT3
protein_coding	AREL1
protein_coding	ESCO1
protein_coding	KATNBL1
protein_coding	LPIN1
protein_coding	STYXL1

protein_coding	ZNF492
protein_coding	CNOT10
protein_coding	GHDC
protein_coding	AC024257.1
protein_coding	P2RY13
protein_coding	ORC2
protein_coding	PPP1R14C
protein_coding	GPR61
protein_coding	CD5
protein_coding	MYO1E
protein_coding	ZNF32
protein_coding	NOC2L
protein_coding	ZFPM2
protein_coding	CHRM4
protein_coding	DXO
protein_coding	ATP5S
protein_coding	C5orf25
protein_coding	PLCD1
protein_coding	HECA
protein_coding	PAFAH2
protein_coding	BVES
protein_coding	ADGRL2
protein_coding	TMEM2
protein_coding	TMIGD3
protein_coding	PPFIBP1
protein_coding	MRPS26
protein_coding	ZNF407
protein_coding	UTP6
protein_coding	HSF2
protein_coding	TARS
protein_coding	UBB
protein_coding	PRDM13
protein_coding	SLC6A1
protein_coding	C9orf64
protein_coding	UBE2G2
protein_coding	C8orf44-SGK3
protein_coding	SAP130
protein_coding	NDST1
protein_coding	RBM45
protein_coding	SCN4B
protein_coding	PSMA6
protein_coding	IL1A
protein_coding	SRP19
protein_coding	IGFLR1
protein_coding	VPS18
protein_coding	MPV17L
protein_coding	OXT

protein_coding	EFS
protein_coding	LHPP
protein_coding	ZNF839
protein_coding	TMED8
protein_coding	PPP1R36
protein_coding	KCNK15
protein_coding	ENOSF1
protein_coding	CTR9
protein_coding	GTF3C5
protein_coding	OR2M3
protein_coding	DQ596646
protein_coding	PACSIN2
protein_coding	SLC25A25
protein_coding	DISP2
protein_coding	ITGAM
protein_coding	KIAA0195
protein_coding	HS3ST2
protein_coding	ZNF420
protein_coding	ZFHX3
protein_coding	TTC7B
protein_coding	CRTC2
protein_coding	ZNF34
protein_coding	GABPA
protein_coding	KBTBD8
protein_coding	PXDNL
protein_coding	GCFC2
protein_coding	SMIM18
protein_coding	SLC35F5
protein_coding	C7orf50
protein_coding	RAC3
protein_coding	CPTP
protein_coding	COG4
protein_coding	C9orf163
protein_coding	ITGAX
protein_coding	BOC
protein_coding	NEURL1B
protein_coding	GALNT18
protein_coding	MGAT2
protein_coding	HERPUD1
protein_coding	INPP5K
protein_coding	RFX7
protein_coding	DDX27
protein_coding	NUDT2
protein_coding	CXCL2
protein_coding	RASSF8
protein_coding	C19orf52
protein_coding	SLC27A6

protein_coding	CDKL4
protein_coding	FAM155B
protein_coding	GYG1
protein_coding	NOL6
protein_coding	NKAPL
protein_coding	IGFBP5
protein_coding	CCDC30
protein_coding	FOXO4
protein_coding	VPS50
protein_coding	IFITM2
protein_coding	PMEL
protein_coding	AMDHD1
protein_coding	RFXAP
protein_coding	ATF7
protein_coding	ANXA7
protein_coding	PHLDB2
protein_coding	TM4SF18
protein_coding	RAB11B
protein_coding	CPEB2
protein_coding	ALG8
protein_coding	CWF19L1
protein_coding	HCCS
protein_coding	EDAR
protein_coding	BSG
protein_coding	ZNF256
protein_coding	UNC13B
protein_coding	MED30
protein_coding	ENOX1
protein_coding	EXOC4
protein_coding	EXOG
protein_coding	GATS
protein_coding	C17orf97
protein_coding	RP13-672B3.2
protein_coding	INVS
protein_coding	PTGES3L
protein_coding	ZNF317
protein_coding	DENND2A
protein_coding	RTEL1-TNFRSF6B
protein_coding	LPHN3
protein_coding	TMEM236
protein_coding	UBXN8
protein_coding	TTC27
protein_coding	LSG1
protein_coding	CCDC68
protein_coding	ZCCHC8
protein_coding	WDFY4
protein_coding	FAM167A

protein_coding	SOX8
protein_coding	APITD1-CORT
protein_coding	PASK
protein_coding	CDKN2A
protein_coding	ZNF577
protein_coding	RALA
protein_coding	BTBD6
protein_coding	DGCR6L
protein_coding	CLEC4C
protein_coding	KIAA0141
protein_coding	CABYR
protein_coding	NARS2
protein_coding	FBXO27
protein_coding	RNF185
protein_coding	ALG9
protein_coding	NETO2
protein_coding	SLC2A5
protein_coding	CACNA1S
protein_coding	ALKBH6
protein_coding	ITGA6
protein_coding	SAP30L
protein_coding	ZNF425
protein_coding	TRIM35
protein_coding	LBX2
protein_coding	PXMP4
protein_coding	ARFGEF2
protein_coding	PRPSAP1
protein_coding	PGLYRP3
protein_coding	G6PD
protein_coding	CLIC2
protein_coding	CLYBL
protein_coding	CORT
protein_coding	CDK6
protein_coding	CALU
protein_coding	HID1
protein_coding	SLFN5
protein_coding	NUPL1
protein_coding	RAB15
protein_coding	FBXL19
protein_coding	APPL2
protein_coding	EDEM3
protein_coding	ZNF143
protein_coding	IDE
protein_coding	IRX5
protein_coding	SSR4
protein_coding	SEMA3D
protein_coding	ATIC

protein_coding	FBXL4
protein_coding	NLRP1
protein_coding	ZDHHC2
protein_coding	STX2
protein_coding	MRPL53
protein_coding	KLC4
protein_coding	C10orf35
protein_coding	MBOAT7
protein_coding	KIFC2
protein_coding	SH2D6
protein_coding	DEPDC5
protein_coding	ZNF432
protein_coding	TANGO2
protein_coding	IL12A
protein_coding	OGFOD1
protein_coding	PSMA1
protein_coding	PAK6
protein_coding	C16orf72
protein_coding	SNX22
protein_coding	SNAPC2
protein_coding	ASB3
protein_coding	HKR1
protein_coding	GALK1
protein_coding	ALKBH8
protein_coding	HDAC1
protein_coding	WDR55
protein_coding	TOP3B
protein_coding	MIS12
protein_coding	SF3B6
protein_coding	MAPK8IP3
protein_coding	AC117834.1
protein_coding	NTNG2
protein_coding	FAM173B
protein_coding	ENTPD4
protein_coding	CXorf57
protein_coding	ZMYM6
protein_coding	HS3ST3A1
protein_coding	FANCB
protein_coding	SSH2
protein_coding	RXRA
protein_coding	EIF3L
protein_coding	ZNF438
protein_coding	IFNGR1
protein_coding	MYOG
protein_coding	RHPN1
protein_coding	DHRS3
protein_coding	LSR

protein_coding	TNPO3
protein_coding	ANKRD30BL
protein_coding	C10orf105
protein_coding	TRIM27
protein_coding	CLDN11
protein_coding	CCT2
protein_coding	RPS9
protein_coding	LOH12CR1
protein_coding	FKBP9
protein_coding	OTUD3
protein_coding	RP11-351M8.1
protein_coding	GPR85
protein_coding	CTTN
protein_coding	PRSS21
protein_coding	RABL6
protein_coding	SPRN
protein_coding	LGR6
protein_coding	PNCK
protein_coding	C15orf48
protein_coding	BRI3
protein_coding	SPDYE6
protein_coding	PRMT10
protein_coding	BLMH
protein_coding	MLX
protein_coding	TSPAN31
protein_coding	CCDC87
protein_coding	SECISBP2
protein_coding	GART
protein_coding	ISG15
protein_coding	YBX2
protein_coding	CHRNB1
protein_coding	CCL3L3
protein_coding	LTBR
protein_coding	HSPA13
protein_coding	CCDC3
protein_coding	FYCO1
protein_coding	NWD2
protein_coding	PLRG1
protein_coding	MAMLD1
protein_coding	LPAR6
protein_coding	ELMOD3
protein_coding	DBT
protein_coding	FAM106A
protein_coding	MED14OS
protein_coding	CDH2
protein_coding	VSTM1
protein_coding	MYH14

protein_coding	ACSS2
protein_coding	RP11-295D22.1
protein_coding	APOL1
protein_coding	LAT
protein_coding	AL136376.1
protein_coding	SLC25A39
protein_coding	ORC5
protein_coding	HMGXB4
protein_coding	SOGA1
protein_coding	CLIP2
protein_coding	ZNF223
protein_coding	PRKD1
protein_coding	TTI2
protein_coding	KCNJ10
protein_coding	PRRT4
protein_coding	FBXL20
protein_coding	SMIM20
protein_coding	PNKP
protein_coding	ACADL
protein_coding	TMEM177
protein_coding	LAMB2
protein_coding	ZNF75D
protein_coding	CXorf36
protein_coding	MIP
protein_coding	UBE2I
protein_coding	SNRPB
protein_coding	ZNF165
protein_coding	CAPN5
protein_coding	P2RX4
protein_coding	MIPOL1
protein_coding	ST3GAL1
protein_coding	BMP2
protein_coding	CDKN1C
protein_coding	L2HGDH
protein_coding	TP53INP1
protein_coding	HYPK
protein_coding	RAP1GAP2
protein_coding	SNRPB2
protein_coding	PRH1
protein_coding	MBD1
protein_coding	TMED9
protein_coding	TREH
protein_coding	GGT1
protein_coding	C2orf47
protein_coding	TMEM18
protein_coding	MYNN
protein_coding	SECTM1

protein_coding	IL17RC
protein_coding	CPT2
protein_coding	SHTN1
protein_coding	PIGO
protein_coding	ATAD2
protein_coding	ACTL7A
protein_coding	CLEC4E
protein_coding	PDE6G
protein_coding	MON1B
protein_coding	ZPR1
protein_coding	SAFB
protein_coding	LIMK1
protein_coding	GPC1
protein_coding	MRPL12
protein_coding	GNAL
protein_coding	DPY19L1
protein_coding	TRA2A
protein_coding	RPRD1B
protein_coding	TRIM21
protein_coding	PHYHD1
protein_coding	PCCA
protein_coding	RNGTT
protein_coding	CARKD
protein_coding	LACRT
protein_coding	TMCC3
protein_coding	DPF2
protein_coding	MYO5A
protein_coding	SLC8A2
protein_coding	CEP97
protein_coding	PNN
protein_coding	EIF4EBP3
protein_coding	CCDC7
protein_coding	SCML1
protein_coding	DPH3P1
protein_coding	SDAD1
protein_coding	EPHA1
protein_coding	MTUS2
protein_coding	ANKRD55
protein_coding	NDE1
protein_coding	CENPB
protein_coding	MARK1
protein_coding	BOP1
protein_coding	G6PC3
protein_coding	ARFGAP3
protein_coding	HNRNPA0
protein_coding	TMEM53
protein_coding	UBR1

protein_coding	BPTF
protein_coding	SOGA2
protein_coding	ZBTB48
protein_coding	VRK3
protein_coding	AASDH
protein_coding	DENND4B
protein_coding	NIPBL
protein_coding	KLHDC4
protein_coding	ATP8A2
protein_coding	TADA2A
protein_coding	UBAC2
protein_coding	CCT6A
protein_coding	FAM110B
protein_coding	FAM160B2
protein_coding	CHD4
protein_coding	RSU1
protein_coding	SUCO
protein_coding	LMX1B
protein_coding	GPD1
protein_coding	C16orf87
protein_coding	MCAM
protein_coding	ERLIN2
protein_coding	RNF112
protein_coding	KAT6A
protein_coding	CLUAP1
protein_coding	PSME4
protein_coding	TMEM87B
protein_coding	FAM3A
protein_coding	RPS23
protein_coding	C16orf70
protein_coding	KCTD7
protein_coding	RBM5
protein_coding	LSM10
protein_coding	EBP
protein_coding	ZNF613
protein_coding	PHACTR2
protein_coding	SERPINF2
protein_coding	ANGEL1
protein_coding	USP13
protein_coding	NUDT6
protein_coding	LGR4
protein_coding	ADD2
protein_coding	SEPT6
protein_coding	FUT11
protein_coding	RBM24
protein_coding	OR10J1
protein_coding	ZC3HC1

protein_coding	ZNF23
protein_coding	UBE2S
protein_coding	UBOX5
protein_coding	PPIE
protein_coding	OR2L13
protein_coding	NME9
protein_coding	TIMM9
protein_coding	PDZD4
protein_coding	ST6GALNAC3
protein_coding	ATOH7
protein_coding	C20orf194
protein_coding	VIL1
protein_coding	SLC35B2
protein_coding	ZNF575
protein_coding	B3GAT2
protein_coding	AC007956.1
protein_coding	FCGR1B
protein_coding	NACC2
protein_coding	DHRS4L2
protein_coding	WNT7B
protein_coding	TMEM151B
protein_coding	AL079342.1
protein_coding	COPS7B
protein_coding	CCNK
protein_coding	AX747333
protein_coding	EXT2
protein_coding	EXOSC4
protein_coding	FAM114A1
protein_coding	EFNA2
protein_coding	PLEKHA8
protein_coding	SELPLG
protein_coding	ACTR3B
protein_coding	PWP2
protein_coding	VASH1
protein_coding	MAPK8
protein_coding	RNF216
protein_coding	VSTM5
protein_coding	HPS4
protein_coding	SNX21
protein_coding	NAF1
protein_coding	CSNK1G2
protein_coding	ST20
protein_coding	RBM43
protein_coding	SMPD4
protein_coding	DENND4C
protein_coding	DAPK2
protein_coding	KIN

protein_coding	TST
protein_coding	CP
protein_coding	FZD6
protein_coding	PCSK6
protein_coding	RHBG
protein_coding	ATRAID
protein_coding	GAN
protein_coding	RNFT1
protein_coding	FAM78B
protein_coding	ATF1
protein_coding	NT5C3A
protein_coding	SPHK1
protein_coding	GPR124
protein_coding	ARF4
protein_coding	SLAMF8
protein_coding	TRIP4
protein_coding	HPS5
protein_coding	RRM1
protein_coding	MRPL42
protein_coding	DIS3
protein_coding	ZNF800
protein_coding	BCAS2
protein_coding	LILRA1
protein_coding	FITM2
protein_coding	SNRPF
protein_coding	CAAP1
protein_coding	PRRT3
protein_coding	ZFYVE27
protein_coding	TTR
protein_coding	FAM84B
protein_coding	THUMPD2
protein_coding	GRIP2
protein_coding	CLK3
protein_coding	HTR3B
protein_coding	ARHGAP26
protein_coding	GMPS
protein_coding	TMEM60
protein_coding	BCL2A1
protein_coding	FHOD1
protein_coding	ST8SIA6
protein_coding	MYO10
protein_coding	CDK11A
protein_coding	GALNT6
protein_coding	NFKBIZ
protein_coding	PRSS36
protein_coding	OR2L2
protein_coding	PRR5L

protein_coding	ZNF16
protein_coding	B3GAT3
protein_coding	DEFA1
protein_coding	COL6A2
protein_coding	PLD2
protein_coding	NFXL1
protein_coding	NBR1
protein_coding	DGCR6
protein_coding	DCN
protein_coding	RP11-47I22.4
protein_coding	XRCC1
protein_coding	ANKRD44
protein_coding	HYOU1
protein_coding	MS4A8
protein_coding	PRR9
protein_coding	DIO2
protein_coding	CDKN2B
protein_coding	TRAPPC2P1
protein_coding	TSR1
protein_coding	LAMB1
protein_coding	KIAA0226
protein_coding	DUSP9
protein_coding	PIGB
protein_coding	PDE12
protein_coding	MED6
protein_coding	OBFC1
protein_coding	FGD2
protein_coding	THRAP3
protein_coding	TMA16
protein_coding	HELQ
protein_coding	HMBS
protein_coding	REXO1L11P
protein_coding	CHST11
protein_coding	GPR15
protein_coding	STIM1
protein_coding	KIAA0391
protein_coding	IFITM10
protein_coding	RASA3
protein_coding	APLF
protein_coding	BAG3
protein_coding	DSE
protein_coding	SRSF2
protein_coding	WDR11
protein_coding	GATM-AS1
protein_coding	ARHGAP15
protein_coding	PIP4K2C
protein_coding	EPHA6

protein_coding	ALOX5AP
protein_coding	RXRG
protein_coding	RCL1
protein_coding	COL11A1
protein_coding	TRAPPC11
protein_coding	CRK
protein_coding	RBPMS2
protein_coding	SLC12A4
protein_coding	ZNF878
protein_coding	AL139099.1
protein_coding	TTC9
protein_coding	NSUN5
protein_coding	TM6SF2
protein_coding	SOX2
protein_coding	USP39
protein_coding	LDOC1L
protein_coding	PRDM8
protein_coding	UBR5
protein_coding	ANOS1
protein_coding	C9orf85
protein_coding	MST1L
protein_coding	NEK3
protein_coding	CEP250
protein_coding	PLAC8
protein_coding	AC010327.2
protein_coding	P2RX6
protein_coding	RNMT
protein_coding	TMEM8A
protein_coding	CTD-2207O23.3
protein_coding	NSA2
protein_coding	EXOSC9
protein_coding	LPPR2
protein_coding	MUC20
protein_coding	ABT1
protein_coding	TEX40
protein_coding	KLRF1
protein_coding	MORN3
protein_coding	SLC23A2
protein_coding	SNAI3
protein_coding	ZSWIM8
protein_coding	ZNF684
protein_coding	MARC1
protein_coding	LPIN3
protein_coding	ELF2
protein_coding	CRLS1
protein_coding	LSM6
protein_coding	MMD

protein_coding	TANC2
protein_coding	FOXJ2
protein_coding	KIAA0825
protein_coding	KCNA1
protein_coding	TTC21B
protein_coding	UVRAG
protein_coding	SIAH2
protein_coding	SHROOM1
protein_coding	CAT
protein_coding	DNPEP
protein_coding	AQP1
protein_coding	LRRC29
protein_coding	IRAK2
protein_coding	OPN1LW
protein_coding	AL049747.1
protein_coding	COQ5
protein_coding	COPB1
protein_coding	GTF3C4
protein_coding	GJA9
protein_coding	SLC35G4
protein_coding	PPM1E
protein_coding	TMIE
protein_coding	CMBL
protein_coding	CCDC146
protein_coding	MESDC2
protein_coding	RP11-834C11.12
protein_coding	FBL
protein_coding	ADCY3
protein_coding	PGAM1
protein_coding	VWC2L
protein_coding	CNGB1
protein_coding	DGKG
protein_coding	TOP1MT
protein_coding	RHBDF1
protein_coding	PCDHB8
protein_coding	SH2B1
protein_coding	SLC44A3
protein_coding	ZNF384
protein_coding	CHURC1
protein_coding	CAMKV
protein_coding	POM121C
protein_coding	TMEM129
protein_coding	WDR75
protein_coding	DCP1A
protein_coding	KIAA1549
protein_coding	C10orf32
protein_coding	ZNF259

protein_coding	AQP12A
protein_coding	CD1A
protein_coding	CARD9
protein_coding	GNL3L
protein_coding	TPRKB
protein_coding	TMEM25
protein_coding	NCBP1
protein_coding	PDCL
protein_coding	MYLK3
protein_coding	C11orf42
protein_coding	SARAF
protein_coding	RPL7L1
protein_coding	LCN8
protein_coding	GNAT1
protein_coding	HBEGF
protein_coding	PTPN13
protein_coding	FOLR4
protein_coding	DHRS7C
protein_coding	LAIR1
protein_coding	OR2F1
protein_coding	CRNKL1
protein_coding	ERICH1
protein_coding	TAF12
protein_coding	RAD54B
protein_coding	CREB5
protein_coding	BC042385
protein_coding	EFTUD1
protein_coding	VARS
protein_coding	CSNK1G3
protein_coding	NDRG4
protein_coding	NAT10
protein_coding	ITGB5
protein_coding	C1orf87
protein_coding	ZNHIT6
protein_coding	RGL3
protein_coding	C19orf55
protein_coding	SLC2A3
protein_coding	ALG6
protein_coding	BCO2
protein_coding	FLT1
protein_coding	MTERFD1
protein_coding	PHB2
protein_coding	TRMU
protein_coding	DOC2B
protein_coding	IKBKB
protein_coding	FAM32A
protein_coding	RHOV

protein_coding	FAM150B
protein_coding	FAM114A2
protein_coding	LGMN
protein_coding	ALPP
protein_coding	AL020996.1
protein_coding	SLC35B3
protein_coding	GRASP
protein_coding	KRTAP19-8
protein_coding	FAM20C
protein_coding	HIC1
protein_coding	ACAA2
protein_coding	SUGT1
protein_coding	TMED4
protein_coding	SPATA20
protein_coding	CCNL2
protein_coding	LOC729732
protein_coding	POGK
protein_coding	KIAA2022
protein_coding	DPCR1
protein_coding	SKI
protein_coding	ZNF174
protein_coding	SEC61A2
protein_coding	NPAS4
protein_coding	TCTEX1D1
protein_coding	PDZK1IP1
protein_coding	ADSL
protein_coding	MLC1
protein_coding	DIAPH3
protein_coding	WARS2
protein_coding	CACNA1I
protein_coding	GYPE
protein_coding	IGLL1
protein_coding	TMEM150C
protein_coding	MCC
protein_coding	UTS2R
protein_coding	RP11-433C9.2
protein_coding	CSF3R
protein_coding	TMEM81
protein_coding	MXD1
protein_coding	MTIF3
protein_coding	C5orf45
protein_coding	L3HYPDH
protein_coding	TRAK1
protein_coding	SPATS2
protein_coding	DRAM2
protein_coding	NOL8
protein_coding	RING1

protein_coding	HIF1AN
protein_coding	FBXL14
protein_coding	LAP3
protein_coding	PLEKHA7
protein_coding	ZNF534
protein_coding	MGC13053
protein_coding	RGS1
protein_coding	LEPROT
protein_coding	LLGL1
protein_coding	FBXO36
protein_coding	KCNMB2
protein_coding	C14orf119
protein_coding	ACSL5
protein_coding	ANKAR
protein_coding	CDKN3
protein_coding	LTB4R2
protein_coding	IPMK
protein_coding	LACE1
protein_coding	SUPT4H1
protein_coding	RBMXL1
protein_coding	ADORA3
protein_coding	E2F4
protein_coding	MFSD8
protein_coding	YEATS4
protein_coding	DTD2
protein_coding	BTBD9
protein_coding	PUS7
protein_coding	CMTR2
protein_coding	PEX10
protein_coding	NAT1
protein_coding	PYDC1
protein_coding	ENKUR
protein_coding	ZNF846
protein_coding	BMPR1B
protein_coding	PERP
protein_coding	KCTD10
protein_coding	METTL2A
protein_coding	MUTYH
protein_coding	ROCK1
protein_coding	TTC21A
protein_coding	TUBB4A
protein_coding	PATL1
protein_coding	BC036055
protein_coding	UNQ2560
protein_coding	ZNF273
protein_coding	DHRS11
protein_coding	SLC50A1

protein_coding	USP43
protein_coding	ETHE1
protein_coding	TEX10
protein_coding	COPG1
protein_coding	STK32B
protein_coding	SIN3A
protein_coding	ALDH18A1
protein_coding	GJA3
protein_coding	PALB2
protein_coding	ACVRL1
protein_coding	GPN1
protein_coding	CTD-2132N18.3
protein_coding	YTHDF2
protein_coding	FAM27A
protein_coding	SMIM8
protein_coding	ZNF22
protein_coding	SCGB3A1
protein_coding	UBTF
protein_coding	ZFX
protein_coding	OR2L5
protein_coding	ANGEL2
protein_coding	RBMS1
protein_coding	SPRTN
protein_coding	CTCF
protein_coding	IFRD2
protein_coding	YTHDF1
protein_coding	SPATA6
protein_coding	MCRS1
protein_coding	C12orf49
protein_coding	C1orf50
protein_coding	SAMD14
protein_coding	ZNF449
protein_coding	IKZF2
protein_coding	DDX50
protein_coding	POLR3C
protein_coding	GABRQ
protein_coding	AJAP1
protein_coding	EIF2S1
protein_coding	NAT9
protein_coding	FADS1
protein_coding	SLIT1
protein_coding	PRDM10
protein_coding	TMEM229B
protein_coding	APITD1
protein_coding	KDM4C
protein_coding	TPK1
protein_coding	ZBTB2

protein_coding	PCNXL4
protein_coding	LPPR1
protein_coding	ERGIC3
protein_coding	THSD1
protein_coding	REXO1L1P
protein_coding	LHFPL4
protein_coding	DDX56
protein_coding	ZFAND6
protein_coding	TMEM201
protein_coding	CORO7
protein_coding	ADRBK2
protein_coding	KIF16B
protein_coding	POLD2
protein_coding	BRAP
protein_coding	UHRF1BP1
protein_coding	FAM200A
protein_coding	MAP2K7
protein_coding	SLC29A2
protein_coding	AVIL
protein_coding	DPH6
protein_coding	ZNF44
protein_coding	GPR26
protein_coding	DHX57
protein_coding	CAPN6
protein_coding	TESK1
protein_coding	CEACAM18
protein_coding	GGT5
protein_coding	HPS1
protein_coding	AURKAIP1
protein_coding	B3GNT4
protein_coding	KRTAP5-9
protein_coding	TNNC2
protein_coding	CD6
protein_coding	RELN
protein_coding	TDRD9
protein_coding	COX17
protein_coding	AP001816.1
protein_coding	EXOSC8
protein_coding	ASAP3
protein_coding	ZP3
protein_coding	RPS16
protein_coding	TBXAS1
protein_coding	SLC11A2
protein_coding	SMIM10
protein_coding	ELOVL2
protein_coding	SLC19A2
protein_coding	TNKS

protein_coding	RNF24
protein_coding	AC087477.1
protein_coding	PGGT1B
protein_coding	NTMT1
protein_coding	EMILIN2
protein_coding	ARHGAP42
protein_coding	EMX1
protein_coding	RBM41
protein_coding	TYW3
protein_coding	LILRB4
protein_coding	RENBP
protein_coding	TRO
protein_coding	RIOK1
protein_coding	HSD17B13
protein_coding	ZNF554
protein_coding	DEF6
protein_coding	RP11-93B14.6
protein_coding	CHRNB3
protein_coding	RPAP2
protein_coding	LTK
protein_coding	FAM229A
protein_coding	DLEC1
protein_coding	NAAA
protein_coding	NTM
protein_coding	FNBP4
protein_coding	MPV17L2
protein_coding	CD84
protein_coding	BANK1
protein_coding	FAF2
protein_coding	IL12RB2
protein_coding	TBCE
protein_coding	RNF39
protein_coding	ZNF160
protein_coding	EDEM1
protein_coding	ATP5SL
protein_coding	TAZ
protein_coding	AC120194.1
protein_coding	CDK7
protein_coding	GUCY1A3
protein_coding	TDRP
protein_coding	R3HCC1L
protein_coding	LARS2
protein_coding	NPIPA8
protein_coding	NEK6
protein_coding	SHROOM2
protein_coding	SHANK2
protein_coding	PCGF3

protein_coding	HOOK2
protein_coding	AC131097.4
protein_coding	TRIM13
protein_coding	PGP
protein_coding	MRPS17
protein_coding	STK3
protein_coding	SNX32
protein_coding	NKX1-1
protein_coding	PIDD1
protein_coding	EGFR
protein_coding	ANAPC2
protein_coding	FAM216B
protein_coding	NELFE
protein_coding	SLC35E3
protein_coding	RAPGEFL1
protein_coding	VPS8
protein_coding	IFRD1
protein_coding	RARG
protein_coding	POLRMT
protein_coding	ARHGEF6
protein_coding	ZNF808
protein_coding	HMGCR
protein_coding	FBXO46
protein_coding	FAM102B
protein_coding	ACY3
protein_coding	TTC14
protein_coding	AP4E1
protein_coding	ARRDC4
protein_coding	WDR92
protein_coding	RFESD
protein_coding	HGS
protein_coding	TSPAN8
protein_coding	OR2B11
protein_coding	ADM5
protein_coding	CXXC4
protein_coding	RP11-497E19.2
protein_coding	DDX54
protein_coding	UQCR10
protein_coding	MSH6
protein_coding	CDV3
protein_coding	RPS4Y1
protein_coding	MFSD1
protein_coding	LRRC37A
protein_coding	CCNI2
protein_coding	ISG20L2
protein_coding	PHTF2
protein_coding	WWP2

protein_coding	PTBP3
protein_coding	CRAT
protein_coding	GALR1
protein_coding	URAD
protein_coding	SLC35E1
protein_coding	CMPK1
protein_coding	EEF2KMT
protein_coding	COL20A1
protein_coding	CTNNBIP1
protein_coding	AC006486.9
protein_coding	HCST
protein_coding	CCT6B
protein_coding	ST5
protein_coding	NAGA
protein_coding	CLDN2
protein_coding	NADSYN1
protein_coding	BLCAP
protein_coding	QRICH1
protein_coding	FOSB
protein_coding	RBKS
protein_coding	ANKRD11
protein_coding	ZNF233
protein_coding	COMMD4
protein_coding	RAMP2
protein_coding	KCTD13
protein_coding	DMRTA1
protein_coding	FAM45A
protein_coding	RAPH1
protein_coding	MYO1B
protein_coding	BLOC1S4
protein_coding	PDZRN3
protein_coding	DIMT1
protein_coding	NT5E
protein_coding	MAML2
protein_coding	ATP6V0A1
protein_coding	MFSD7
protein_coding	CD93
protein_coding	PAPPA2
protein_coding	C5AR1
protein_coding	GNG11
protein_coding	TMX1
protein_coding	ARHGAP19
protein_coding	TEX30
protein_coding	FAM149B1
protein_coding	NPIPB9
protein_coding	WHAMM
protein_coding	RTDR1

protein_coding	PTPRG
protein_coding	ANKRD36B
protein_coding	COX20
protein_coding	CRIM1
protein_coding	MRGPRG
protein_coding	SLC16A6
protein_coding	CTNNBL1
protein_coding	SUZ12
protein_coding	FAM193A
protein_coding	TGFA
protein_coding	ANKFY1
protein_coding	SEMA6C
protein_coding	ANO7
protein_coding	RPL26L1
protein_coding	CUEDC1
protein_coding	CRCT1
protein_coding	IGF1
protein_coding	NARFL
protein_coding	ISG20
protein_coding	LRRK1
protein_coding	FARP1
protein_coding	MAPK4
protein_coding	ANKRD13A
protein_coding	BRD3
protein_coding	SNX16
protein_coding	C3orf80
protein_coding	C20orf144
protein_coding	ZBTB44
protein_coding	SLC39A3
protein_coding	GAPVD1
protein_coding	CCDC13
protein_coding	AKIRIN1
protein_coding	MBNL1
protein_coding	PDK2
protein_coding	EMCN
protein_coding	SMIM11
protein_coding	LHFPL1
protein_coding	MTMR9
protein_coding	C10orf113
protein_coding	HHLA3
protein_coding	AGPAT4
protein_coding	ATP5E
protein_coding	AMOTL2
protein_coding	TIGD7
protein_coding	PDGFB
protein_coding	DKFZp686G21125
protein_coding	IRF3

protein_coding	SLF1
protein_coding	FUBP3
protein_coding	ZNF101
protein_coding	CTD-2521M24.10
protein_coding	TOMM6
protein_coding	SSX3
protein_coding	ZNF598
protein_coding	UBE2Z
protein_coding	ZNF776
protein_coding	FGF20
protein_coding	DDX55
protein_coding	PZP
protein_coding	HDAC8
protein_coding	MECOM
protein_coding	ZCCHC6
protein_coding	RBSN
protein_coding	STK17B
protein_coding	NFKB1
protein_coding	RHOC
protein_coding	COA1
protein_coding	PRAMEF22
protein_coding	MORN1
protein_coding	CD9
protein_coding	NHSL2
protein_coding	HRH3
protein_coding	PPP6R1
protein_coding	FBXL7
protein_coding	TMEM189
protein_coding	ERAP2
protein_coding	C10orf25
protein_coding	ZC3H6
protein_coding	AP5B1
protein_coding	E2F6
protein_coding	SND1
protein_coding	SOCS5
protein_coding	EIF4E
protein_coding	DOCK1
protein_coding	LENG1
protein_coding	ANO10
protein_coding	WASF2
protein_coding	SPRY3
protein_coding	P4HB
protein_coding	XRN1
protein_coding	CXorf23
protein_coding	KLHL7
protein_coding	NBPF11
protein_coding	MEIS3

protein_coding	ZNF207
protein_coding	CTDSPL
protein_coding	ARG2
protein_coding	SEMA4G
protein_coding	COL4A5
protein_coding	FBRS
protein_coding	ASCL4
protein_coding	LNP1
protein_coding	RNF187
protein_coding	EIF2AK4
protein_coding	ZFY
protein_coding	ABHD13
protein_coding	WDR67
protein_coding	SELO
protein_coding	BBS5
protein_coding	CTPS1
protein_coding	CLCN6
protein_coding	RRP9
protein_coding	RPS4Y2
protein_coding	NOTCH4
protein_coding	ZFPL1
protein_coding	ZDHHC3
protein_coding	TIPARP
protein_coding	CTD-2207O23.12
protein_coding	ERCC8
protein_coding	CASP1
protein_coding	CAMSAP3
protein_coding	GLIS1
protein_coding	PPP6R3
protein_coding	SRPRB
protein_coding	KIAA1598
protein_coding	CLDN12
protein_coding	GUCA1B
protein_coding	C2orf72
protein_coding	SYNJ2
protein_coding	TMEM19
protein_coding	ZNF506
protein_coding	IGFBP1
protein_coding	RPN2
protein_coding	KLHL10
protein_coding	OGN
protein_coding	CYTH3
protein_coding	AFF1
protein_coding	C11orf57
protein_coding	MTHFD2L
protein_coding	WWC2
protein_coding	KCNC4

protein_coding	ALG2
protein_coding	FRMD6
protein_coding	PRKRIP1
protein_coding	RACGAP1
protein_coding	SSBP1
protein_coding	NIF3L1
protein_coding	PIK3CD
protein_coding	EXD3
protein_coding	JOSD2
protein_coding	ZBTB24
protein_coding	VSX2
protein_coding	ACOT11
protein_coding	EP300
protein_coding	TERF2
protein_coding	GUSB
protein_coding	HARS2
protein_coding	SEC16B
protein_coding	UGCG
protein_coding	CYB561D2
protein_coding	ACTL8
protein_coding	PRKCH
protein_coding	P2RX2
protein_coding	PKD2
protein_coding	AC011484.1
protein_coding	CACNA1E
protein_coding	MYOF
protein_coding	WDR73
protein_coding	ATG12
protein_coding	TRIM32
protein_coding	BX649567.1
protein_coding	NEK1
protein_coding	CNPY3
protein_coding	KCNH5
protein_coding	SUMF1
protein_coding	CSNK2A2
protein_coding	FOXL2
protein_coding	LOC339524
protein_coding	TSHZ1
protein_coding	DUSP8
protein_coding	FAS
protein_coding	UBTD1
protein_coding	FAM71F2
protein_coding	FBXO40
protein_coding	MPHOSPH9
protein_coding	EPS8L3
protein_coding	FAM122A
protein_coding	RP9

protein_coding	CEP57
protein_coding	TMEM263
protein_coding	SNURF
protein_coding	PPTC7
protein_coding	OR2C1
protein_coding	METTL3
protein_coding	UBE2J2
protein_coding	CLVS1
protein_coding	TBC1D1
protein_coding	OCRL
protein_coding	WDR82
protein_coding	ALOX5
protein_coding	XKR6
protein_coding	PLS1
protein_coding	OPA3
protein_coding	MTFMT
protein_coding	TMEM161B
protein_coding	DNAJC3
protein_coding	C1orf95
protein_coding	AX746522
protein_coding	RMND1
protein_coding	ID1
protein_coding	SETD8
protein_coding	ALPPL2
protein_coding	NKTR
protein_coding	SPEF2
protein_coding	GLCCI1
protein_coding	VPS53
protein_coding	FEN1
protein_coding	USP6NL
protein_coding	PDIA4
protein_coding	GGA2
protein_coding	ITIH4
protein_coding	CHMP7
protein_coding	TIMM44
protein_coding	ATRIP
protein_coding	SLIT2
protein_coding	EIF2B2
protein_coding	WDR89
protein_coding	SGCD
protein_coding	RNF5
protein_coding	HEATR7A
protein_coding	VPS33A
protein_coding	CTDSP1
protein_coding	SOCS7
protein_coding	ARHGEF1
protein_coding	ITPR2

protein_coding	SMG8
protein_coding	KRT31
protein_coding	ZCCHC14
protein_coding	KIAA1908
protein_coding	C14orf64
protein_coding	CEP68
protein_coding	SLC2A6
protein_coding	CCNT1
protein_coding	FBXO10
protein_coding	ZDHHC5
protein_coding	RP11-178L8.4
protein_coding	SNX11
protein_coding	SEC13
protein_coding	ENDOD1
protein_coding	BCKDK
protein_coding	GDPGP1
protein_coding	SSR1
protein_coding	KIAA1841
protein_coding	ZNF132
protein_coding	ZNF417
protein_coding	LAPTM4A
protein_coding	CEP135
protein_coding	SMPD2
protein_coding	HBP1
protein_coding	TAL1
protein_coding	NOSTRIN
protein_coding	RAD18
protein_coding	CPAMD8
protein_coding	DEPTOR
protein_coding	TNFRSF4
protein_coding	U2AF1L4
protein_coding	ZNF41
protein_coding	AC025262.1
protein_coding	ANKRD31
protein_coding	SOS2
protein_coding	STK4
protein_coding	C1orf64
protein_coding	WSB1
protein_coding	TBC1D31
protein_coding	ICAM5
protein_coding	TFAP4
protein_coding	HAVCR2
protein_coding	PIAS2
protein_coding	FAM78A
protein_coding	TGM2
protein_coding	RABL3
protein_coding	ASCC2

protein_coding	OPRM1
protein_coding	ZC3H7B
protein_coding	SF3A2
protein_coding	METTL23
protein_coding	SDHA
protein_coding	DUX4
protein_coding	ARID2
protein_coding	ACTN4
protein_coding	TBC1D10A
protein_coding	SIKE1
protein_coding	VAV2
protein_coding	DTX1
protein_coding	TLCD1
protein_coding	TMLHE
protein_coding	P2RY4
protein_coding	CNST
protein_coding	RPAIN
protein_coding	C6orf89
protein_coding	MRPL16
protein_coding	C5orf55
protein_coding	YARS2
protein_coding	IL1B
protein_coding	TAGAP
protein_coding	KCNJ12
protein_coding	SMCHD1
protein_coding	GTF2IRD2
protein_coding	BLM
protein_coding	EPHB1
protein_coding	KCNJ9
protein_coding	CD46
protein_coding	HIGD2A
protein_coding	MAP6D1
protein_coding	FLT3
protein_coding	ARMC2
protein_coding	DQ583205
protein_coding	DHRS1
protein_coding	NFATC3
protein_coding	EGR4
protein_coding	KCTD3
protein_coding	IPO9
protein_coding	APCDD1
protein_coding	MRPL21
protein_coding	MCFD2
protein_coding	HNRNPH1
protein_coding	THPO
protein_coding	TG
protein_coding	GJB4

protein_coding	C9orf116
protein_coding	NDUFB11
protein_coding	ARHGAP23
protein_coding	BUD13
protein_coding	OAF
protein_coding	KHSRP
protein_coding	DDX3X
protein_coding	CLDN15
protein_coding	HNRNPF
protein_coding	ATRN
protein_coding	TSPAN12
protein_coding	ZNF717
protein_coding	CHRNB2
protein_coding	C11orf73
protein_coding	ITPRIP
protein_coding	C17orf85
protein_coding	CACNG8
protein_coding	USP17L3
protein_coding	FLYWCH2
protein_coding	DMRTC1
protein_coding	CTB-54O9.9
protein_coding	ZNHIT3
protein_coding	PLTP
protein_coding	ZNF749
protein_coding	WDR38
protein_coding	GOLGA8M
protein_coding	LINC01119
protein_coding	TSPY10
protein_coding	HAS1
protein_coding	PRR14
protein_coding	RPP14
protein_coding	CSNK2B
protein_coding	DPH3
protein_coding	FAT1
protein_coding	TRIM8
protein_coding	ADGRF2
protein_coding	TMEM209
protein_coding	PLCD3
protein_coding	RPL4
protein_coding	NIPSNAP3A
protein_coding	LGALSL
protein_coding	VPS54
protein_coding	AP003068.23
protein_coding	TACR3
protein_coding	METTL14
protein_coding	SHQ1
protein_coding	C2orf81

protein_coding	RPL35A
protein_coding	PIGQ
protein_coding	ZNF222
protein_coding	CDC23
protein_coding	GUF1
protein_coding	ELP4
protein_coding	XK
protein_coding	CETN1
protein_coding	MITD1
protein_coding	ZIC5
protein_coding	ZNF516
protein_coding	ADGRA3
protein_coding	SLC39A6
protein_coding	TMEM256
protein_coding	DQ786325
protein_coding	ALG12
protein_coding	PSMD13
protein_coding	WBSCR16
protein_coding	CSGALNACT2
protein_coding	SNRNP35
protein_coding	MMD2
protein_coding	PI4K2A
protein_coding	EXOSC2
protein_coding	CEACAM3
protein_coding	CLCN7
protein_coding	DOPEY2
protein_coding	LRCH3
protein_coding	MAP3K9
protein_coding	ZSWIM3
protein_coding	GLYR1
protein_coding	ADGRE1
protein_coding	PPAN-P2RY11
protein_coding	CCL21
protein_coding	AL049829.1
protein_coding	CCS
protein_coding	CEPT1
protein_coding	FARS2
protein_coding	XBP1
protein_coding	SH3TC2
protein_coding	GLIPR1
protein_coding	RBBP8NL
protein_coding	DOCK4
protein_coding	TEN1
protein_coding	DAP
protein_coding	TMEM120B
protein_coding	TNFAIP2
protein_coding	KIF12

protein_coding	SNTB2
protein_coding	LARP1B
protein_coding	OR2L8
protein_coding	ABHD10
protein_coding	ELF1
protein_coding	DQ591848
protein_coding	CLHC1
protein_coding	CSF1
protein_coding	FLJ20306
protein_coding	HTR1A
protein_coding	SLC5A5
protein_coding	SEC24B
protein_coding	ZNF488
protein_coding	NBPF12
protein_coding	API5
protein_coding	GIMAP4
protein_coding	TNIP3
protein_coding	ATP4A
protein_coding	ARL9
protein_coding	MTF1
protein_coding	SERPINB2
protein_coding	DISC1
protein_coding	ADGRA1
protein_coding	NAIF1
protein_coding	ETS1
protein_coding	NDUFAF7
protein_coding	SLC48A1
protein_coding	TMEM233
protein_coding	RBM19
protein_coding	ELP2
protein_coding	HHIPL1
protein_coding	PLEC
protein_coding	CSNK2A3
protein_coding	HDAC6
protein_coding	FAM160B1
protein_coding	SLC25A37
protein_coding	THAP8
protein_coding	N4BP1
protein_coding	C5orf64
protein_coding	LZIC
protein_coding	PSMD10
protein_coding	TEAD4
protein_coding	FAM194A
protein_coding	PSKH1
protein_coding	INTS12
protein_coding	SLC35F1
protein_coding	LST1

protein_coding	ADPGK
protein_coding	LDB3
protein_coding	CAPN7
protein_coding	EIF4E1B
protein_coding	SEC31A
protein_coding	TAB3
protein_coding	SLC7A2
protein_coding	CAV1
protein_coding	DFFA
protein_coding	UBXN2B
protein_coding	ERO1LB
protein_coding	RP11-279O9.4
protein_coding	C7orf49
protein_coding	BCL7B
protein_coding	RIOK3
protein_coding	BAIAP2
protein_coding	LATS2
protein_coding	RNF144A
protein_coding	TRIO
protein_coding	TMEM40
protein_coding	IL18
protein_coding	HSPA5
protein_coding	ATL2
protein_coding	HIST2H3PS2
protein_coding	NCEH1
protein_coding	AC079602.1
protein_coding	EFCAB11
protein_coding	ZNF646
protein_coding	MDM4
protein_coding	DYNLRB2
protein_coding	LMAN1
protein_coding	EPB41L5
protein_coding	SPIRE1
protein_coding	SDF2
protein_coding	L77588
protein_coding	GMPR
protein_coding	PLBD1
protein_coding	BTAF1
protein_coding	ACTA2
protein_coding	RP11-520P18.5
protein_coding	GOLT1B
protein_coding	TMEM62
protein_coding	PRKG1
protein_coding	NUDT5
protein_coding	MRPL2
protein_coding	CD320
protein_coding	BACE1

protein_coding	MNX1
protein_coding	PHF20
protein_coding	SBNO2
protein_coding	CSF2RA
protein_coding	ERCC1
protein_coding	AC008443.1
protein_coding	NANOS3
protein_coding	ILF3
protein_coding	KCNAB3
protein_coding	SFTPC
protein_coding	N6AMT2
protein_coding	FAN1
protein_coding	FAM212B
protein_coding	MORF4L2
protein_coding	MFAP1
protein_coding	PIGP
protein_coding	NT5C1B-RDH14
protein_coding	SLAMF9
protein_coding	NENF
protein_coding	SP4
protein_coding	P2RY6
protein_coding	ITPKC
protein_coding	C15orf65
protein_coding	SLC38A6
protein_coding	G3BP1
protein_coding	RIMBP3B
protein_coding	APBA3
protein_coding	LAT2
protein_coding	TGIF2-C20orf24
protein_coding	KCNJ8
protein_coding	FAM229B
protein_coding	AX746944
protein_coding	GID8
protein_coding	SLC32A1
protein_coding	CDKL1
protein_coding	PARP3
protein_coding	RP11-324D17.1
protein_coding	SLC38A7
protein_coding	ERO1B
protein_coding	RARA
protein_coding	ADHFE1
protein_coding	ZCWPW2
protein_coding	AOC2
protein_coding	ZNF224
protein_coding	ABCF2
protein_coding	GALNT16
protein_coding	INHA

protein_coding	HNRNPA1L2
protein_coding	RINT1
protein_coding	STK17A
protein_coding	PECAM1
protein_coding	SEPSECS
protein_coding	KDM5C
protein_coding	VWA5B2
protein_coding	GLB1
protein_coding	RP11-156E8.1
protein_coding	TOB2
protein_coding	SELK
protein_coding	RHBDL3
protein_coding	NARF
protein_coding	PRCP
protein_coding	DDX60L
protein_coding	KIAA0355
protein_coding	CASKIN2
protein_coding	BSND
protein_coding	PROB1
protein_coding	LYAR
protein_coding	ADAM32
protein_coding	FAM26F
protein_coding	NPAS3
protein_coding	LACTB
protein_coding	AKR7A2
protein_coding	PLSCR1
protein_coding	SDC4
protein_coding	RTTN
protein_coding	AC022532.1
protein_coding	ACVR1B
protein_coding	GEMIN6
protein_coding	LRRC16A
protein_coding	MROH8
protein_coding	MAGIX
protein_coding	TMEM135
protein_coding	KMO
protein_coding	RERE
protein_coding	BAZ1B
protein_coding	GSR
protein_coding	VPS45
protein_coding	CIAPIN1
protein_coding	PCDHB6
protein_coding	NME7
protein_coding	AC004466.1
protein_coding	SLC52A2
protein_coding	CRADD
protein_coding	B3GALT5

protein_coding	NOM1
protein_coding	HSD17B3
protein_coding	SLC25A13
protein_coding	FBXO48
protein_coding	IFRG15
protein_coding	LIN52
protein_coding	PLEKHF2
protein_coding	AGPAT6
protein_coding	PPP1R3F
protein_coding	PKN3
protein_coding	ZNF404
protein_coding	ATP6V0E2
protein_coding	CCDC24
protein_coding	MKRN2
protein_coding	ROBO1
protein_coding	GRPEL1
protein_coding	SSSCA1
protein_coding	NCR2
protein_coding	ZBTB7B
protein_coding	FAM134C
protein_coding	PJA1
protein_coding	APTX
protein_coding	SMC5
protein_coding	INTU
protein_coding	SLC25A16
protein_coding	COMMD6
protein_coding	ATP11AUN
protein_coding	PRMT5
protein_coding	ZBTB26
protein_coding	METTL21A
protein_coding	ID4
protein_coding	ARHGAP19-SLIT1
protein_coding	TC2N
protein_coding	CYBRD1
protein_coding	C1orf27
protein_coding	SYT15
protein_coding	HAUS2
protein_coding	PCMTD2
protein_coding	LTA
protein_coding	C1orf53
protein_coding	REEP3
protein_coding	IL17RE
protein_coding	AK310634
protein_coding	METTL15
protein_coding	RNF111
protein_coding	CASK
protein_coding	NOLC1

protein_coding	SOX7
protein_coding	GANAB
protein_coding	RAMP3
protein_coding	HIST1H2BE
protein_coding	ADNP2
protein_coding	NUP85
protein_coding	PRKCSH
protein_coding	C9orf114
protein_coding	VAV3
protein_coding	AF035281
protein_coding	GLRX3
protein_coding	SLC38A9
protein_coding	LILRB1
protein_coding	TOM1
protein_coding	C1orf109
protein_coding	IL17D
protein_coding	AARD
protein_coding	PPFIA1
protein_coding	ING1
protein_coding	ACSF3
protein_coding	TARS2
protein_coding	ACTR3C
protein_coding	RBM10
protein_coding	LILRA6
protein_coding	ZNF566
protein_coding	MT2A
protein_coding	ATP5L
protein_coding	P2RY11
protein_coding	MARCH1
protein_coding	SLC35A1
protein_coding	POU6F2
protein_coding	TWSG1
protein_coding	SLC25A22
protein_coding	PITPNM3
protein_coding	POFUT1
protein_coding	FRMPD1
protein_coding	PRMT1
protein_coding	ST3GAL4
protein_coding	GGA1
protein_coding	RECQL5
protein_coding	AL117190.3
protein_coding	SLC35C1
protein_coding	SREBF2
protein_coding	BCDIN3D
protein_coding	SLC25A41
protein_coding	CASP8AP2
protein_coding	C7orf55-LUC7L2

protein_coding	POC1B-GALNT4
protein_coding	SLC16A1
protein_coding	CSPP1
protein_coding	GTF2E1
protein_coding	KCNA6
protein_coding	HNRNPA3
protein_coding	AB209061
protein_coding	C1orf162
protein_coding	PTGDR
protein_coding	ING4
protein_coding	DIDO1
protein_coding	DENND3
protein_coding	C2orf69
protein_coding	USP54
protein_coding	GDF10
protein_coding	TMEM255B
protein_coding	FAM122B
protein_coding	C9orf129
protein_coding	ACIN1
protein_coding	EXOC6
protein_coding	C10orf85
protein_coding	TMEM168
protein_coding	CARNMT1
protein_coding	CCDC110
protein_coding	TFDP1
protein_coding	POLR2G
protein_coding	ZER1
protein_coding	LOC100289561
protein_coding	TRMT13
protein_coding	COQ10B
protein_coding	AC138647.1
protein_coding	TXNDC2
protein_coding	RASSF1
protein_coding	FARSA
protein_coding	RSAD2
protein_coding	FAM131C
protein_coding	EMC8
protein_coding	LRLE1
protein_coding	AK091593
protein_coding	YIPF3
protein_coding	C2orf27A
protein_coding	BUB3
protein_coding	SEMA3G
protein_coding	IPO7
protein_coding	MRPS18B
protein_coding	TRPC6
protein_coding	H2AFV

protein_coding	PSD2
protein_coding	FBXO4
protein_coding	CPT1A
protein_coding	BTBD11
protein_coding	FAM161A
protein_coding	TRMT2A
protein_coding	SMG1
protein_coding	PDGFRL
protein_coding	KLC1
protein_coding	VWA5A
protein_coding	COX16
protein_coding	RP11-385D13.1
protein_coding	CEP85L
protein_coding	AC004076.9
protein_coding	ZC3H14
protein_coding	IGBP1
protein_coding	TMEM248
protein_coding	MRPL13
protein_coding	MRE11A
protein_coding	PARP8
protein_coding	GSG1L
protein_coding	KCNE3
protein_coding	GEMIN8
protein_coding	APLNR
protein_coding	DOK3
protein_coding	CATSPER2
protein_coding	TRIP10
protein_coding	MYRF
protein_coding	HMCN2
protein_coding	OR10G2
protein_coding	MANBAL
protein_coding	LAIR2
protein_coding	TRAPPC2
protein_coding	CCNG1
protein_coding	ABCC5
protein_coding	C11orf48
protein_coding	ATF4
protein_coding	ANKRD6
protein_coding	COL28A1
protein_coding	STARD10
protein_coding	KRT86
protein_coding	TOMM5
protein_coding	OLFML2B
protein_coding	AGFG2
protein_coding	FGFBP2
protein_coding	NCSTN
protein_coding	ZXDA

protein_coding	NTAN1
protein_coding	CLN8
protein_coding	BMP3
protein_coding	NUCB2
protein_coding	CDKN2C
protein_coding	ZNF266
protein_coding	RFX4
protein_coding	CCIN
protein_coding	PHKG1
protein_coding	CDK17
protein_coding	PHLPP2
protein_coding	VN1R4
protein_coding	UBA6
protein_coding	DARC
protein_coding	CCDC121
protein_coding	SLC22A4
protein_coding	UPP2
protein_coding	AC011239.1
protein_coding	ZFYVE19
protein_coding	SAAL1
protein_coding	KDM1B
protein_coding	OR2W3
protein_coding	RAB5B
protein_coding	C12orf45
protein_coding	ZNF592
protein_coding	C5orf56
protein_coding	CERS6
protein_coding	PHKA1
protein_coding	ST20-MTHFS
protein_coding	ZNF385A
protein_coding	TXLNA
protein_coding	LRBA
protein_coding	PSMD9
protein_coding	NSUN4
protein_coding	KIT
protein_coding	TMEM180
protein_coding	STARD6
protein_coding	KDM6B
protein_coding	FRS3
protein_coding	BLOC1S5
protein_coding	TULP3
protein_coding	DPY19L2
protein_coding	MTCL1
protein_coding	FRY
protein_coding	RP4-539M6.19
protein_coding	MYCBP
protein_coding	ZNF662

protein_coding	ACACB
protein_coding	ST3GAL3
protein_coding	C8orf59
protein_coding	NCOA2
protein_coding	MOB1A
protein_coding	HLA-DQB1
protein_coding	NCOA6
protein_coding	CTHRC1
protein_coding	DAAM2
protein_coding	MARCH5
protein_coding	IFT20
protein_coding	QRICH2
protein_coding	ANKS3
protein_coding	PDCD5
protein_coding	LIMK2
protein_coding	NT5C1A
protein_coding	SUPT20HL1
protein_coding	NELFCD
protein_coding	ATL3
protein_coding	LMOD1
protein_coding	OR13H1
protein_coding	PIGH
protein_coding	DGAT1
protein_coding	TMEM213
protein_coding	B4GALT4
protein_coding	CHMP4C
protein_coding	NPPC
protein_coding	TPBG
protein_coding	DCAKD
protein_coding	SERPINB8
protein_coding	C16orf13
protein_coding	LURAP1
protein_coding	TMEM35
protein_coding	TMEM150B
protein_coding	RAB24
protein_coding	PDE4D
protein_coding	ZNF580
protein_coding	RABEPK
protein_coding	PCGF1
protein_coding	ARHGAP44
protein_coding	BRWD1
protein_coding	PIGC
protein_coding	MRPL50
protein_coding	C15orf38
protein_coding	AC019171.1
protein_coding	SNX15
protein_coding	FNDC9

protein_coding	IFIT5
protein_coding	PBX1
protein_coding	COMMD3
protein_coding	ELFN2
protein_coding	CDK10
protein_coding	RASAL3
protein_coding	STX10
protein_coding	GMPPA
protein_coding	CCDC117
protein_coding	MUC12
protein_coding	GPR27
protein_coding	ZNF496
protein_coding	CYP7B1
protein_coding	C17orf80
protein_coding	RP11-195F19.5
protein_coding	ZNF484
protein_coding	RP11-196G11.1
protein_coding	PARS2
protein_coding	PRR26
protein_coding	AL441883.1
protein_coding	THOC2
protein_coding	SNRPC
protein_coding	WDR59
protein_coding	AIRE
protein_coding	RAB8A
protein_coding	RSG1
protein_coding	MTHFD1L
protein_coding	AL138764.1
protein_coding	KIAA1731
protein_coding	LIMS2
protein_coding	PAX8
protein_coding	CCND3
protein_coding	ASPH
protein_coding	ZNF10
protein_coding	BCL2L2-PABPN1
protein_coding	ADAMTS3
protein_coding	CD33
protein_coding	RELT
protein_coding	TGDS
protein_coding	RBM6
protein_coding	MTERF2
protein_coding	SHISA7
protein_coding	PRPF38B
protein_coding	BIVM-ERCC5
protein_coding	RALY
protein_coding	CTD-2410N18.5
protein_coding	NCKAP5L

protein_coding	KIAA0586
protein_coding	DLX4
protein_coding	MTRF1
protein_coding	HUS1
protein_coding	TRAF3IP3
protein_coding	OLIG1
protein_coding	ATP11B
protein_coding	UBXN10
protein_coding	LRRC39
protein_coding	DGCR14
protein_coding	SLC11A1
protein_coding	SLC17A6
protein_coding	CCL4
protein_coding	PTGER3
protein_coding	CCAR1
protein_coding	HSPB7
protein_coding	HCAR2
protein_coding	RAP2C
protein_coding	MT1H
protein_coding	CCNE1
protein_coding	RPS25
protein_coding	DGKH
protein_coding	IL13RA2
protein_coding	NSG1
protein_coding	KRI1
protein_coding	SLC6A13
protein_coding	DOCK11
protein_coding	C15orf40
protein_coding	SMAD4
protein_coding	MED1
protein_coding	MYL5
protein_coding	SRRM1
protein_coding	AL353354.2
protein_coding	POLG2
protein_coding	NID2
protein_coding	BRAF
protein_coding	UBL4B
protein_coding	ZFP36
protein_coding	C7orf60
protein_coding	SELL
protein_coding	DCLRE1C
protein_coding	ZFP62
protein_coding	NMD3
protein_coding	NF2
protein_coding	UTP23
protein_coding	EFR3B
protein_coding	LINS

protein_coding	CEP44
protein_coding	VAC14
protein_coding	SMAGP
protein_coding	KLF15
protein_coding	USP48
protein_coding	RRS1
protein_coding	MEX3D
protein_coding	TRAPPC6A
protein_coding	SS18L2
protein_coding	CSH2
protein_coding	METTL20
protein_coding	KDM4A
protein_coding	PUS7L
protein_coding	BICC1
protein_coding	C19orf33
protein_coding	DACH2
protein_coding	ANKRA2
protein_coding	BRI3BP
protein_coding	UBXN7
protein_coding	SRSF12
protein_coding	SRPX
protein_coding	AP001468.1
protein_coding	COPZ1
protein_coding	TUBA1C
protein_coding	RGP1
protein_coding	ATXN3L
protein_coding	EFCAB10
protein_coding	TLR1
protein_coding	CCDC106
protein_coding	ARFRP1
protein_coding	CC2D1B
protein_coding	FBLN5
protein_coding	CROT
protein_coding	GTF3C2
protein_coding	AADAT
protein_coding	KDM8
protein_coding	TOMM22
protein_coding	MSI2
protein_coding	DDX19B
protein_coding	SLC15A4
protein_coding	METTL16
protein_coding	MDH1B
protein_coding	OXER1
protein_coding	ZNHIT1
protein_coding	ECI2
protein_coding	ADCY1
protein_coding	C19orf35

protein_coding	TMEM251
protein_coding	DUSP22
protein_coding	GTF2H3
protein_coding	IQCG
protein_coding	STX7
protein_coding	SLC39A7
protein_coding	MTG2
protein_coding	BC150535
protein_coding	DUOX1
protein_coding	DPYS
protein_coding	KIAA0907
protein_coding	DCAF17
protein_coding	RBM12
protein_coding	MTMR4
protein_coding	PBLD
protein_coding	PAEP
protein_coding	FAM76A
protein_coding	DEGS2
protein_coding	ARSK
protein_coding	CPNE6
protein_coding	FAM76B
protein_coding	CD58
protein_coding	ZDHHC13
protein_coding	MZB1
protein_coding	ZNF511
protein_coding	USP40
protein_coding	CDK5R2
protein_coding	C6ORF50
protein_coding	TPST2
protein_coding	MOB4
protein_coding	TRPC4AP
protein_coding	SHPRH
protein_coding	PPP1R37
protein_coding	ZNF275
protein_coding	OGT
protein_coding	SENP7
protein_coding	UEVLD
protein_coding	TEPP
protein_coding	C10orf142
protein_coding	TOX4
protein_coding	C10orf54
protein_coding	SDS
protein_coding	ZNF594
protein_coding	LRMP
protein_coding	RHOG
protein_coding	CLN5
protein_coding	NAP1L1

protein_coding	SYNDIG1
protein_coding	PLAC9
protein_coding	MANF
protein_coding	ZCCHC9
protein_coding	RNF139
protein_coding	RNF214
protein_coding	C21orf88
protein_coding	CRTAC1
protein_coding	REEP1
protein_coding	KDELR2
protein_coding	NUPR1
protein_coding	TGFBR3L
protein_coding	SLCO4A1
protein_coding	PRKAB1
protein_coding	SAR1B
protein_coding	GJC2
protein_coding	C4orf33
protein_coding	EIF6
protein_coding	OR6A2
protein_coding	ACCSL
protein_coding	ACAP3
protein_coding	CCDC160
protein_coding	PGM3
protein_coding	APOPT1
protein_coding	SMURF1
protein_coding	PSMB1
protein_coding	MRPL45
protein_coding	GSK3A
protein_coding	SEC22C
protein_coding	OTOS
protein_coding	POLR2J2
protein_coding	PTTG2
protein_coding	PTPRA
protein_coding	TISP43
protein_coding	TENC1
protein_coding	SLC29A3
protein_coding	MTPAP
protein_coding	AKT2
protein_coding	ZFAND3
protein_coding	XKR4
protein_coding	ZBED5
protein_coding	OR2V2
protein_coding	ZBTB14
protein_coding	PRPF40B
protein_coding	SEMA4F
protein_coding	ZFP69B
protein_coding	TFG

protein_coding	FAM209A
protein_coding	EIF3B
protein_coding	MRGPRE
protein_coding	DDX26B
protein_coding	SNX9
protein_coding	OGG1
protein_coding	NAB1
protein_coding	UCKL1
protein_coding	MLYCD
protein_coding	ZC3H8
protein_coding	HNRNPL
protein_coding	KIAA1324
protein_coding	SNX18
protein_coding	SFMBT2
protein_coding	EEF1D
protein_coding	MTERF3
protein_coding	SIX4
protein_coding	LINC00671
protein_coding	USHBP1
protein_coding	MEX3C
protein_coding	ABCG1
protein_coding	TECPR2
protein_coding	XRRA1
protein_coding	BAIAP2L2
protein_coding	CTNNB1
protein_coding	MED21
protein_coding	TIMM10B
protein_coding	CTDSP2
protein_coding	WDR74
protein_coding	ALS2CR12
protein_coding	C6orf47
protein_coding	TMEM125
protein_coding	TBC1D14
protein_coding	PBDC1
protein_coding	PTCHD4
protein_coding	SLC41A2
protein_coding	CD96
protein_coding	TSHZ2
protein_coding	WFIKKN1
protein_coding	SCAF8
protein_coding	PLEK
protein_coding	SSR3
protein_coding	ASB1
protein_coding	PCBD1
protein_coding	PI4KB
protein_coding	GIMAP1
protein_coding	ANKRD9

protein_coding	NUTF2
protein_coding	SCARA3
protein_coding	ZC3H12B
protein_coding	LMLN
protein_coding	GJB3
protein_coding	PPARG
protein_coding	PGD
protein_coding	CDYL
protein_coding	PCTP
protein_coding	FAM35A
protein_coding	FAM161B
protein_coding	CCDC144A
protein_coding	TEAD1
protein_coding	PLEKHG6
protein_coding	UNC5B
protein_coding	DDX58
protein_coding	GCLM
protein_coding	SLITRK5
protein_coding	ALG14
protein_coding	SLC15A3
protein_coding	FTSJ2
protein_coding	IFNL1
protein_coding	RP11-770J1.5
protein_coding	PRRT2
protein_coding	NPYY1
protein_coding	LYRM2
protein_coding	MCCC2
protein_coding	LSM5
protein_coding	SMARCA4
protein_coding	SERTAD2
protein_coding	SDR39U1
protein_coding	KNSTRN
protein_coding	NMRK2
protein_coding	ZNF330
protein_coding	ZNF383
protein_coding	RPL28
protein_coding	GXYLT1
protein_coding	IGFBP4
protein_coding	PPP3CC
protein_coding	NAA16
protein_coding	ANAPC1
protein_coding	IPP
protein_coding	ZNF804B
protein_coding	HMGN1
protein_coding	IMPG1
protein_coding	CCDC9
protein_coding	METTL18

protein_coding	CRNN
protein_coding	PAX5
protein_coding	ELOVL7
protein_coding	KCNH8
protein_coding	COA7
protein_coding	MCM7
protein_coding	GSTO2
protein_coding	MUC4
protein_coding	TIMM50
protein_coding	WDR19
protein_coding	TTL
protein_coding	CHD2
protein_coding	ICT1
protein_coding	ZBTB40
protein_coding	WNT10B
protein_coding	RNF217
protein_coding	MR1
protein_coding	NMNAT1
protein_coding	WHSC1L1
protein_coding	IL33
protein_coding	PPP4R3A
protein_coding	MAVS
protein_coding	SRBD1
protein_coding	UBA52
protein_coding	PIGW
protein_coding	PHKG2
protein_coding	OTUD7B
protein_coding	SPDYC
protein_coding	HGSNAT
protein_coding	ARHGAP20
protein_coding	B4GALT7
protein_coding	WIPI1
protein_coding	TRPM7
protein_coding	TBXA2R
protein_coding	CTAGE15
protein_coding	KCND2
protein_coding	TAF8
protein_coding	ZNF653
protein_coding	KCNK2
protein_coding	AL627171.1
protein_coding	TNFSF12
protein_coding	GAREML
protein_coding	CCBL2
protein_coding	ZFYVE20
protein_coding	BCL10
protein_coding	MED25
protein_coding	UMPS

protein_coding	HAUS7
protein_coding	EVI5L
protein_coding	EIF2AK1
protein_coding	RBBP9
protein_coding	PKIA
protein_coding	RWDD2A
protein_coding	CDK8
protein_coding	ZNF880
protein_coding	NIP7
protein_coding	TMC8
protein_coding	LCLAT1
protein_coding	TNS2
protein_coding	DPCD
protein_coding	NKD1
protein_coding	TRIM59
protein_coding	HDAC2
protein_coding	ARSE
protein_coding	GCSH
protein_coding	BCL7C
protein_coding	C1orf74
protein_coding	PSMF1
protein_coding	AP4M1
protein_coding	ZBTB1
protein_coding	ZNF844
protein_coding	SPATA32
protein_coding	AL357673.1
protein_coding	C2CD4B
protein_coding	TNNT1
protein_coding	OPHN1
protein_coding	MMACHC
protein_coding	FJX1
protein_coding	MYL3
protein_coding	PAGR1
protein_coding	HIVEP1
protein_coding	KCNA4
protein_coding	CACNA2D3
protein_coding	PIPOX
protein_coding	RCN1
protein_coding	TMEM68
protein_coding	ITSN2
protein_coding	ANKRD10
protein_coding	TSGA10
protein_coding	ZNF561
protein_coding	TPT1
protein_coding	PSD4
protein_coding	FANK1
protein_coding	GABPB2

protein_coding	DUSP12
protein_coding	IRF8
protein_coding	DUSP4
protein_coding	TBC1D24
protein_coding	POU2F2
protein_coding	TBR1
protein_coding	SH3GLB1
protein_coding	C21orf67
protein_coding	PPAT
protein_coding	GRM1
protein_coding	FNTB
protein_coding	UACA
protein_coding	C1orf131
protein_coding	BCCIP
protein_coding	WDR35
protein_coding	NDUFC1
protein_coding	AGO3
protein_coding	OLR1
protein_coding	CYP26C1
protein_coding	FAM231B
protein_coding	PGLS
protein_coding	AKR1E2
protein_coding	TBC1D7
protein_coding	STRA13
protein_coding	BCL2L1
protein_coding	NOL11
protein_coding	IGF2R
protein_coding	BCL2
protein_coding	MYH7
protein_coding	VARS2
protein_coding	CCNG2
protein_coding	SEC23IP
protein_coding	CDK11B
protein_coding	PKN2
protein_coding	EXOSC10
protein_coding	RFPL2
protein_coding	PXK
protein_coding	MRPL43
protein_coding	TSKS
protein_coding	LETMD1
protein_coding	STX11
protein_coding	CNFN
protein_coding	MAGOH
protein_coding	SRD5A3
protein_coding	CLEC4A
protein_coding	C19orf18
protein_coding	ASMTL

protein_coding	ANKRD62
protein_coding	GLRA3
protein_coding	SAC3D1
protein_coding	NR1H3
protein_coding	KIR3DL1
protein_coding	RAB29
protein_coding	SLC33A1
protein_coding	SNRNP48
protein_coding	HDX
protein_coding	GALK2
protein_coding	ADTRP
protein_coding	CTC1
protein_coding	LRRC57
protein_coding	CHKA
protein_coding	MRPS28
protein_coding	REXO1
protein_coding	IL13RA1
protein_coding	TMEM163
protein_coding	CTSK
protein_coding	VTCN1
protein_coding	FAM198A
protein_coding	TEFM
protein_coding	PLIN1
protein_coding	NEXN
protein_coding	TSPAN33
protein_coding	GOLPH3L
protein_coding	TBKBP1
protein_coding	LY6G6C
protein_coding	DDX47
protein_coding	AGL
protein_coding	SPA17
protein_coding	VSTM2L
protein_coding	GSTZ1
protein_coding	OAS1
protein_coding	HIST2H2AA4
protein_coding	RBM22
protein_coding	APOOL
protein_coding	DDC8
protein_coding	PSRC1
protein_coding	YEATS2
protein_coding	FAM84A
protein_coding	HERC1
protein_coding	CETN3
protein_coding	GTF3C3
protein_coding	C21orf58
protein_coding	MGST2
protein_coding	NSMCE2

protein_coding	VIPR1
protein_coding	UTP15
protein_coding	FOXRED1
protein_coding	CREM
protein_coding	LUC7L2
protein_coding	LRTOMT
protein_coding	FDPS
protein_coding	DGKQ
protein_coding	DMBX1
protein_coding	GP9
protein_coding	SNRPD3
protein_coding	AX747507
protein_coding	SLC26A1
protein_coding	RARS
protein_coding	NCLN
protein_coding	RMDN1
protein_coding	SLC9A2
protein_coding	CNOT4
protein_coding	IRF2BPL
protein_coding	PFDN4
protein_coding	UBFD1
protein_coding	FAM25E
protein_coding	ANAPC10
protein_coding	NRL
protein_coding	NLGN3
protein_coding	REN
protein_coding	ATG4B
protein_coding	MBTPS2
protein_coding	GALNTL6
protein_coding	PARG
protein_coding	ACTC1
protein_coding	MANEA
protein_coding	PPP1CC
protein_coding	CRMP1
protein_coding	C9orf3
protein_coding	DUS1L
protein_coding	ARID1B
protein_coding	AK130486
protein_coding	MRPL14
protein_coding	JADE2
protein_coding	MAGEB1
protein_coding	MFAP3
protein_coding	DUS4L
protein_coding	USP17L2
protein_coding	OTUD5
protein_coding	FOXO1
protein_coding	PEX7

protein_coding	MRPL38
protein_coding	BC107108
protein_coding	HCFC1
protein_coding	SP5
protein_coding	SNX24
protein_coding	KBTBD11
protein_coding	MSN
protein_coding	GPR75-ASB3
protein_coding	ZNF292
protein_coding	GPR153
protein_coding	TXNIP
protein_coding	FRMPD3
protein_coding	SMYD4
protein_coding	OSGEP
protein_coding	STS
protein_coding	PTPN20
protein_coding	PANX2
protein_coding	LARGE
protein_coding	ST6GALNAC6
protein_coding	BCAS4
protein_coding	NWD1
protein_coding	LRRC10
protein_coding	ZNF557
protein_coding	ACKR1
protein_coding	NHS
protein_coding	TM9SF4
protein_coding	ENDOV
protein_coding	KIFC3
protein_coding	DLG3
protein_coding	MROH7
protein_coding	KANK4
protein_coding	EMID1
protein_coding	POP5
protein_coding	FAM171A2
protein_coding	ZRANB2
protein_coding	CSNK1D
protein_coding	SNAPIN
protein_coding	ENKD1
protein_coding	MGAT5
protein_coding	CBX7
protein_coding	CDC42EP1
protein_coding	MSRB3
protein_coding	RCCD1
protein_coding	EAPP
protein_coding	KIAA1614
protein_coding	SIAE
protein_coding	RHBDD1

protein_coding	DDX20
protein_coding	ADCK3
protein_coding	ITGB7
protein_coding	CDK5RAP2
protein_coding	COQ7
protein_coding	EVA1B
protein_coding	ZW10
protein_coding	EGR1
protein_coding	BCKDHA
protein_coding	PDGFRB
